# A Signature for Smoking Status of Coronary Heart Disease Patients through Weighted Gene Coexpression Network Analysis

**DOI:** 10.1155/2022/5777946

**Published:** 2022-01-18

**Authors:** Zhenglu Shang, Jiashun Sun, Jingjiao Hui, Yanhua Yu, Xiaoyun Bian, Bowen Yang, Kewu Deng, Li Lin

**Affiliations:** ^1^Department of Cardiology, Wuxi Huishan District People's Hospital, Wuxi, China; ^2^Department of Hospital, Wuxi Huishan District People's Hospital, Wuxi, China; ^3^Department of Cardiology, Beijing Tongren Hospital, Beijing, China; ^4^Department of Cardiology, Shanghai Dongfang Hospital, Shanghai, China

## Abstract

**Background:**

Smoking is one of the risk factors of coronary heart disease (CHD), while its underlying mechanism is less well defined.

**Purpose:**

To identify and testify 6 key genes of CHD related to smoking through weighted gene coexpression network analysis (WGCNA), protein-protein interaction (PPI) network analysis, and pathway analysis.

**Methods:**

CHD patients' samples were first downloaded from Gene Expression Omnibus (GEO). Then, genes of interest were obtained after analysis of variance (ANOVA). Thereafter, 23 coexpressed modules that were determined after genes with similar expression were incorporated via WGCNA. The biological functions of genes in the modules were researched by enrichment analysis. Pearson correlation analysis and PPI network analysis were used to screen core genes related to smoking in CHD.

**Results:**

The violet module was the most significantly associated with smoking (*r* = −0.28, *p* = 0.006). Genes in this module mainly participated in biological functions related to the heart. Altogether, 6 smoking-related core genes were identified through bioinformatics analyses. Their expressions in animal models were detected through the animal experiment.

**Conclusion:**

This study identified 6 core genes to serve as underlying biomarkers for monitoring and predicting smoker's CHD risk.

## 1. Introduction

Coronary artery heart disease (CAHD) is the most predominant type of cardiovascular disease and arises from the interaction of various risk factors. One significant pathophysiological basis of CAHD is stenosis of coronary artery lumen due to coronary artery atherosclerosis (CAAS) or myocardial ischemia, hypoxia, or necrosis because of functional changes of coronary artery. All of the above symptoms are called coronary heart diseases (CHDs) [1]. It is known that smoking is the strongest changeable factor of morbidity and mortality of cardiovascular diseases, and quitting smoking can evidently reduce the risk of CHD [2]. The risk of CHD in ex-smokers is lower than current smokers, and differences in the risk would increase since smoke cessation as time passed [3]. Thus, it is urgent to identify the molecular mechanism of CHD related to smoking to understand its pathogenesis.

Weighted gene coexpression network analysis (WGCNA) is a method of analyzing the expression mode of multiple sample genes. The method clusters genes with similar expression modes and analyzes the correlation between modules and specific traits or phenotypes [4]. WGCNA applies the information of numerous genes that varies the most or all of them instead of some differentially expressed genes solely, to identify gene sets of interest [5]. Liu et al. [6] identified 8 lncRNAs that notably influence the overall survival of patients with larynx cancer (LC) via WGCNA, and these identified lncRNAs are testified in many LC cells. Ding et al. [7] used WGCNA to screen 5 key genes, which sheds light on future prognosis and treatment of hepatocellular carcinoma (HCC). However, the research on the potential molecular mechanism of WGCNA for CHD is still very inadequate.

Besides, the molecular mechanism between smoking and clear cell renal cell cancer (ccRCC) in previous studies has been studied via protein-protein interaction (PPI) network and WGCNA [8]. PPI network is crucial in most biological functions and processes, and most proteins activate functions through interactions with each other. Hence, PPI network helps researchers more comprehensively and systematically understand the molecular mechanism of diseases [9]. For example, Huo et al. [10] explained the effect mechanism of Danshensu on CHD via constructing a PPI network. Jensen et al. [11] determined several genes related to CHD risk using a PPI network. However, little was reported about PPI in researching the effect of smoking on CHD risk.

In conclusion, our study provides preliminary information for deeply researching the molecular mechanism of CHD pathogenesis, thereby defining suitable biomarkers that can monitor and predict the risk for CHD in smokers.

## 2. Materials and Methods

### 2.1. Data Source and Preprocess

Whole blood cell gene alignments of CHD patients and clinical data (Supplementary Table 1) were obtained from Gene Expression Omnibus (GEO) (https://www.ncbi.nlm.nih.gov/geo/query/acc.cgi?acc=GSE20681). Patient's quantitative coronary angiography (QCA) ≥ 50% stenosis in at least 1 large vessel was selected for research, and 99 samples were ultimately obtained. Probes were annotated via GPL4133.

The missing expression data were calculated with the k-nearest neighbors (KNN) algorithm and normalized with LIMMA package [12, 13]. As for several probes that match a gene signature solely, the average expression values of corresponding genes of these probes were calculated and taken as ultimate gene expression values. Analysis of variance (ANOVA) was performed on all genes [14]. Genes with variances greater than the quartile of all variances were selected as the genes of interest. Next, WGCNA was performed combining gender and smoking status.

### 2.2. Construction of Gene Coexpression Network

WGCNA construction utilized R package WGCNA [4]. A sample cluster tree was established to remove outlier samples. Afterward, Pearson correlation analysis was performed on all genes to construct an adjacent matrix. Correlation between genes was further calculated with a suitable soft threshold value parameter *β*. To this end, the connection between genes in the network was subjected to scale-free network distribution (*β* is a soft threshold value parameter that can emphasize the strong correlation among genes and punish weak correlation). Then, the result was transferred to the adjacent matrix and further into the topology overlap matrix (TOM).

To classify genes with similar expression modes to the same gene modules, genes were hierarchically clustered with dissimilarity degree (1-TOM), and the minimum module was set to contain 50 genes. In principle component analysis, the principle component was composed of module eigengene (ME) that representing the gene expression profile of each module. Next, module eigenvalue was defined as the most important part of modules, and all genes in the modules were summarized into a single characteristic expression profile. Finally, highly similar gene modules were merged (threshold value: dissimilarity less than 0.25).

### 2.3. Determination of Relevant Modules of Interest and Module Function Annotation

The correlation between module eigenvalue and smoking was calculated via Pearson correlation analysis, and the module most related to smoking was chosen as the module of interest.

Thereafter, to further explore the biological functions that genes in the module of interest may affect, GO functional annotation and KEGG enrichment analyses were performed by using clusterProfiler package [15] (threshold value: *p* value < 0.05 and *q* value < 0.05).

### 2.4. Key Gene Identification

After determining the module of interest, the Gene Significance (correlation between smoking status and genes in the module) and Module Membership (genes in the module and module eigenvalue) were calculated, with cor.geneModuleMembership > 0.8 and cor.geneTraitSignificance > 0.2 as threshold values to screen hub genes in the module.

To further screen key genes with more significance, all gene lists in the module of interest were uploaded to STRING (http://string-db.org/cgi/input.pl) website to construct a PPI network. The genes with the top 20 highest connectivity were chosen as hub nodes in the PPI network (threshold value: interaction score > 0.4). Then, hub genes in the module were intersected with hub nodes in the PPI network to acquire potential key genes of CHD related to smoking.

### 2.5. Validation of Key Genes

To testify whether screened key genes participated in regulating biological processes related to the heart, coexpression analysis and corresponding pathway analysis were performed on screened potential genes related to smoking in GeneMANIA (http://genemania.org/). Thereafter, boxplots of key gene expression were drawn according to smoking status in different groups to verify the effect of smoking on key gene expression.

### 2.6. Cigarette Smoke Extract (CSE) Preparation

A vacuum pump (pressure: 300 mmHg) was employed to filter the smoke of two lit cigarettes (China Tobacco Hunan Industrial Co., Ltd. Tar: 12 mg, nicotine: 1.1 mg, carbon monoxide: 14 mg, China). Particles in the smoke were removed. Then, the filtered smoke was collected in the PBS buffer. The pH of CSE-PBS solution was controlled between 7.2 and 7.4. Lastly, fresh-prepared CSE was filtered and disinfected for further use.

### 2.7. Animal Model Construction and Hemocyte Isolation

Male BALB/c mice (8-week) were purchased from Vital River Animal Center (Beijing, China) and placed in a 12 h/12 h day-night environment with a free diet at room temperature of 22°C. Both smoking and nonsmoking CHD mouse models were induced by dietary stimulation. In brief, 10 mice were divided into the smoking group and the normal group (5 mice a group). Mice in two groups were fed with a high-fat diet (TP-2003) (Trophic Animal Feed, China) containing the following components: 0.21% propylthiouracil, 0.49% sodium cholate hydrate, 87.3% standard chow, 2% cholesterol, and 10% lard. CSE of two cigarettes was added to the water bottles of the smoking group every day, while no CSE was added to the water bottles of the nonsmoking group. After 8 weeks of culture following the above diet, blood was collected from the eyeballs of mice and then diverted into tubules (Eppendorf, Germany) using heparin sodium capillary tubes. Then, 300 *μ*l of whole blood was obtained from each mouse. The fresh blood was centrifuged at 1800 × g for 10 min, and then, the upper plasma was discarded to obtain blood cells. All mouse-related experiments were approved by the Animal Ethics Committee of Wuxi Huishan District People's Hospital.

### 2.8. Polymerase Chain Reaction (PCR)

Total RNA was isolated from mouse hemocytes using TRIzol reagent (Invitrogen, USA). After RNA concentration was detected by Nanodrop 2000, cDNA was obtained by reverse transcription of total RNA through PrimeScriptTRT Reagent (Takara, Japan). Finally, the obtained cDNA was amplified by PCR using SYBRPremix Ex Taq. The relative expression of DNA was calculated by 2^-*ΔΔ*CT^ method. All experiments were repeated three times, and the corresponding data were averaged. Primers used for PCR are shown in the following table (Table 1).

## 3. Results

### 3.1. Determination of Genes of Interest

After probes were annotated, ANOVA was undertaken on all genes based on the gene expression matrix. A total of 4,937 genes with variances that were greater than the quartile of all variances were selected (Supplementary Table 2).

### 3.2. WGCNA

**Figure 1:**
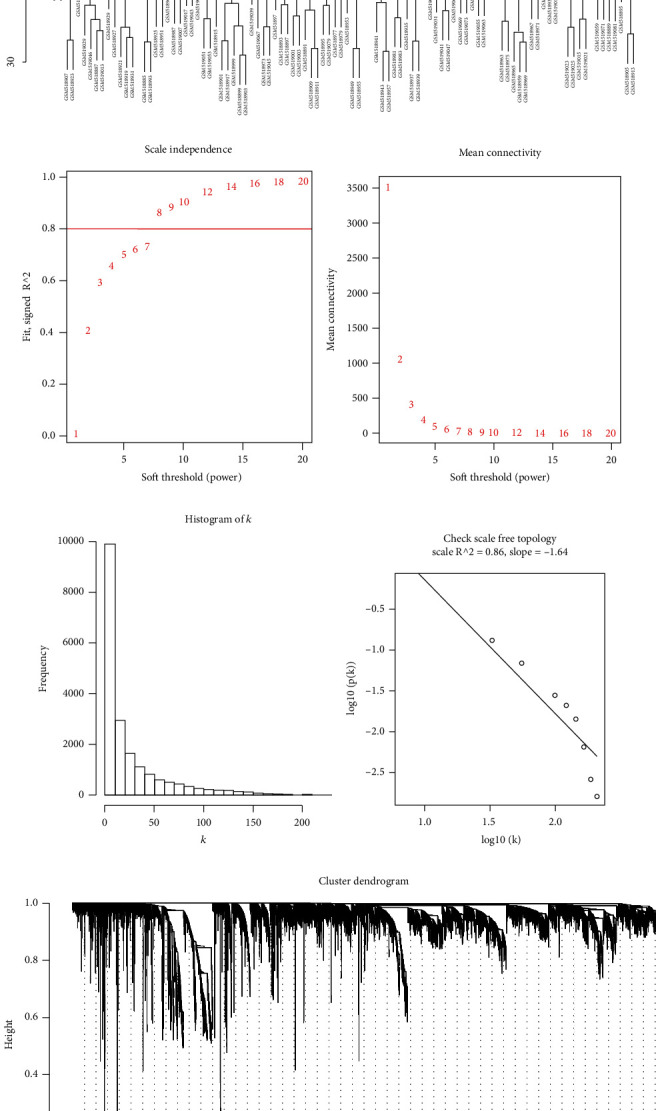
WGCNA: (a) clustering analysis of 99 CHD samples; (b) analysis of scale-free fit index of various threshold values (*β*); (c) analysis of mean connectivity of various threshold values; (d) histogram of connectivity distribution when *β* = 8; (e) check of scale-free topology when *β* = 8; (f) clustering dendrogram of all differentially expressed genes.

Weighted gene coexpression network was built on 4,937 genes in 99 CHD samples with WGCNA package. First of all, 3 outlier samples were removed by sample clustering analysis (Figure 1(a)). Then, coexpression network was constructed with 96 samples and their 4,937 corresponding genes. The connectivity between genes in the gene network satisfied the scale-free network distribution as the threshold value *β* was 8 (scale-free *R*^2^ = 0.86) (Figures 1(b)–1(e)). Thereafter, hierarchical clustering was undertaken on genes with dissimilarity degree (1-TOM). Thirty-seven modules were obtained, and eigenvalue of each module was calculated. Highly similar gene modules were merged with dissimilarity degree < 0.25, and 23 coexpression modules were finally determined (Figure 1(f)).

### 3.3. Functional Annotation of the Modules of Interest

**Figure 2:**
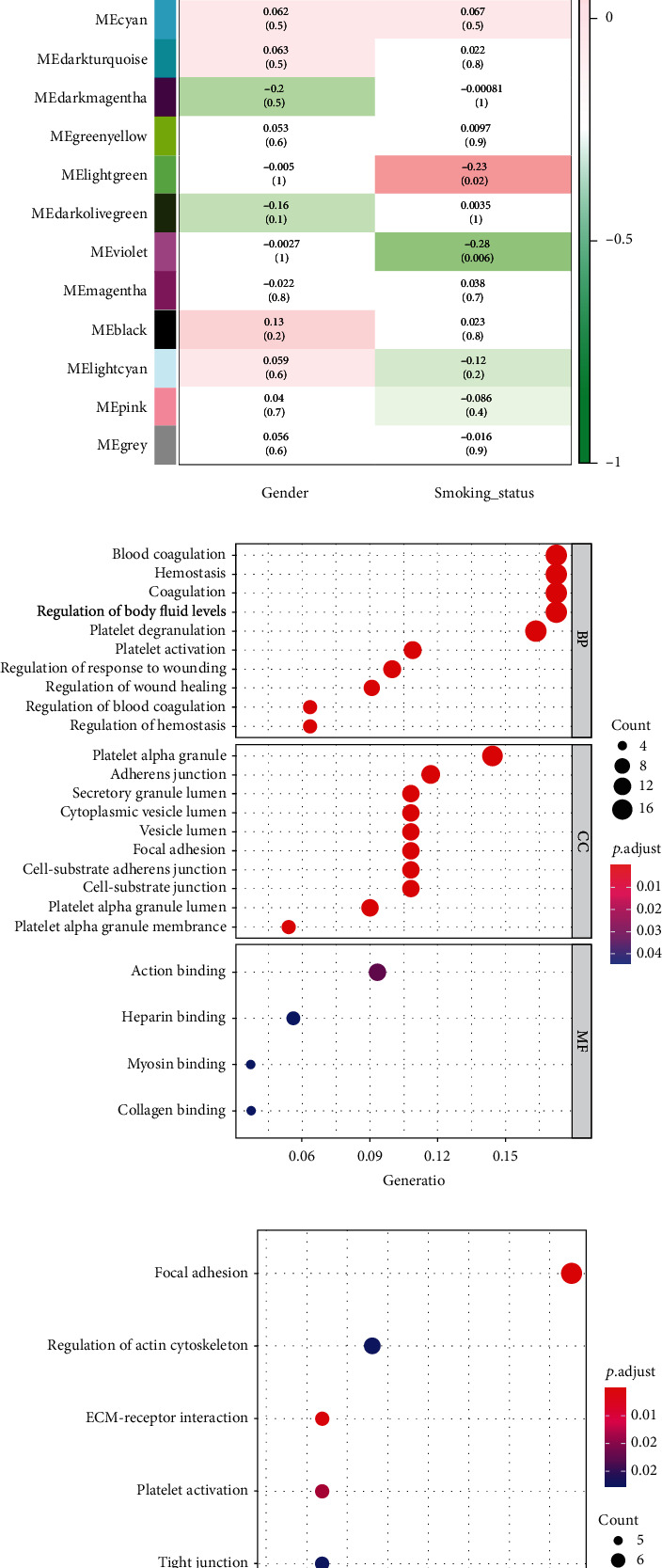
Functional annotation of modules of interest: (a) correlation between gene modules and smoking status; (b) GO enrichment analysis of genes in the violet module; (c) KEGG enrichment analysis of genes in the violet module.

The correlation between module and smoking status was calculated via the module-trait relationship of WGCNA. It was illustrated that the violet module presented the most significant correlation with smoking status (*r* = −0.28, *p* = 0.006) (Figure 2(a)). Next, GO and KEGG enrichment analyses were performed on 143 genes in the violet module. GO analysis indicated that genes were mainly enriched in biological functions related to the heart like blood coagulation, hemostasis, coagulation, adherens junction, focal adhesion, and actin binding (Figure 2(b)). KEGG showed that genes were mainly enriched in biological pathways like focal adhesion, platelet activation, and tight junction (Figure 2(c)). The above results represented that genes significantly related to smoking may be involved in regulating the biological functions of the heart.

### 3.4. Determination of Key Genes

**Figure 3:**
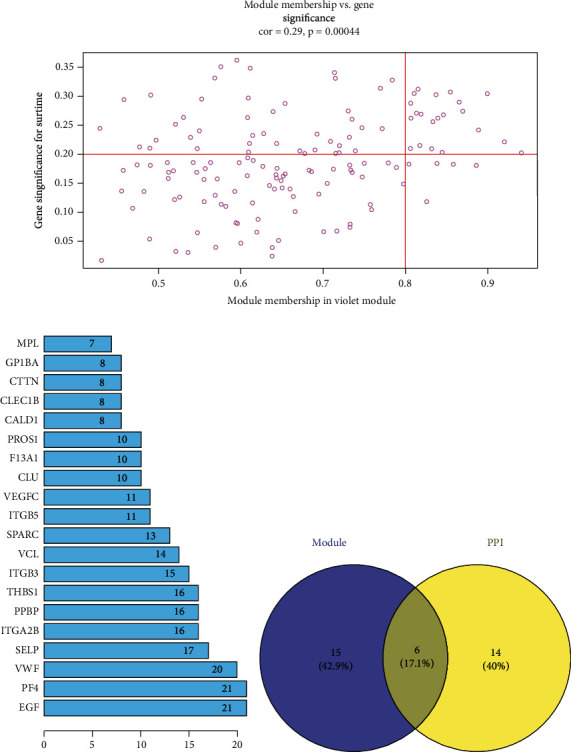
Determination of key genes: (a) scatter diagram of module eigengenes related to smoking status in the violet module; (b) genes with top 20 nudes in PPI network in the violet module. *X*-axis is the degree value of genes; (c) Venn diagram of top 20 genes in PPI and hub genes in the violet module.

In this study, 21 genes with high correlation with smoking in the violet module were screened as hub genes with cor.geneModuleMembership > 0.8 and cor.geneTraitSignificance > 0.2 as threshold values (Figure 3(a)).

PPI network was constructed with all genes in the violet module. Top 20 genes with the highest connectivity were screened as hub nodes of the PPI network with interaction score > 0.4 as a threshold value (Figure 3(b)). The hub nodes of the PPI network were then intersected with 21 hub genes in the violet module, and finally, 6 potential key genes related to smoking were obtained (Figure 3(c)).

### 3.5. Verification of Key Genes

**Figure 4:**
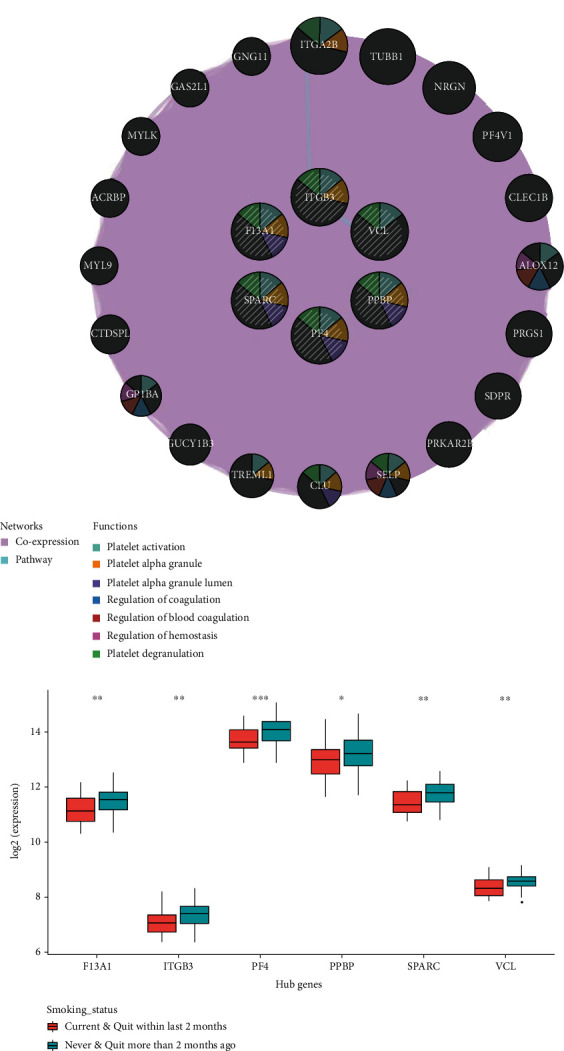
Verification of key genes: (a) coexpression network and pathway analysis of potential genes related to smoking in GeneMANIA; (b) boxplot of expression differences of potential genes related to smoking in different groups of smoking status.

To verify whether the 6 key genes were related to smoking status and CHD risk of patients, coexpression analysis was undertaken in GeneMANIA on these 6 key genes, and 26 genes were obtained (including these 6 key genes). It was found that parts of genes were enriched in biological functions related to the heart like platelet activation, regulation of coagulation, regulation of blood coagulation, regulation of hemostasis, platelet alpha granule lumen, platelet degranulation, and platelet alpha granule (Figure 4(a)).

Then, analysis of grouped smoking status exhibited that 6 genes were significantly lowly expressed in recent or current smoking group, which was consistent with the negative correlation between corresponding WGCNA module and smoking status (Figure 4(b)).

The above results exhibited that the 6 key genes may be involved in regulating biological functions related to the heart and were relevant to smoking status.

### 3.6. The Expression of Key Genes in the Smoking and Nonsmoking CHD Mouse Models

**Figure 5:**
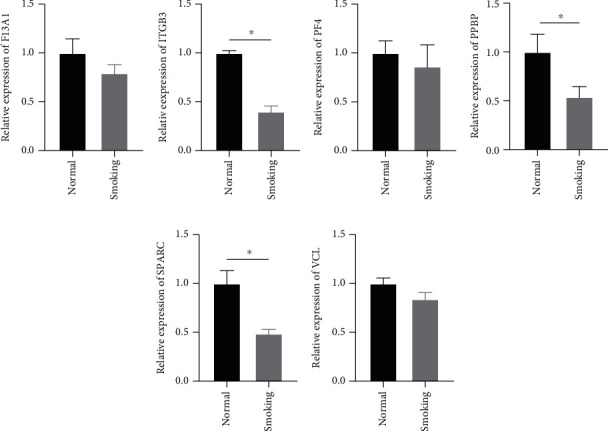
The expression of key genes in smoking and nonsmoking CHD mouse models. (a–f) qRT-PCR detected the expression of key genes (F13A1, ITGB3, PF4, PBP, SPARC, and VCL) in mouse hemocytes. ^∗^*p* < 0.05.

In an effort to measure key gene expression in the smoking and nonsmoking patients with CHD from the animal experiment, we built corresponding CHD mice models. The expression levels of F13A1, ITGB3, PF4, PBP, SPARC, and VCL in mouse hemocytes were detected by qRT-PCR (Figures 5(a)–5(f)). The results showed that the expression of ITGB3, PPBP, and SPARC was significantly low in the smoking group. The expression levels of F13A1, PF4, and VCL in the smoking group showed a trend of low expression, but there was no significant difference.

## 4. Discussion

CHD mainly features atherosclerosis and coronary artery dynamic vasospasm and myocardial infarction. Myocardial ischemia and hypoxia may occur as disease worsen, or more seriously, disability rate and fatality rate will increase [16]. To relieve coronary artery stenosis as soon as possible and reduce the mortality rate, a reasonable diagnostic method is necessary to detect CHD progression. Smoking is known as a main independent risk factor for CHD, and the risk and status of CHD are negatively correlated with the age of smoking initiation, daily smoking amount, and smoking years [17]. Critchley and Capewell [18] found that the mortality of patients who quit smoking declines 36% compared with smoking patients, and the mortality is not relevant to patients' other features (such as age, gender, type of CHD, and years of study) [18]. Hence, it was considered that exploring genes related to smoking may help us to further understand the molecular mechanism of CHD pathogenesis, thereby increasing its diagnostic and cure rates for smoking patients.

In our study, 99 CHD patient samples were accessed from GEO first. The violet module that had the most significant correlation with smoking was identified with WGCNA. WGCNA is an association analysis between modules and external sample traits by searching clusters (modules) of highly correlated genes, which can be used for identifying candidate biomarkers or therapeutic targets [4]. Next, enrichment analysis was performed on genes in the above module. The results showed that genes were mainly enriched in biological pathways like focal adhesion, platelet activation, and tight junction. GO analysis exhibited that genes in the module may affect biological functions of the heart like clotting, myocardial contractions, and signal transduction. A study exhibited that specific components of the cardiomyocyte costamere (focal adhesion) play a role in initiating and maintaining the transduction of aberrant signal which contributes to cardiac remodeling and development [19]. The above references and results testified that the determined genes in the module that significantly related to smoking were involved in regulating biological functions related to the heart.

Next, 21 hub genes were acquired from the violet module. Then, PPI network was established with all genes in the violet module, and 20 hub nodes were found. Later, PPI was intersected with hub genes in WGCNA, and 6 key genes were ultimately obtained (F13A1, ITGB3, PF4, PPBP, SPARC, VCL). The activity of F13A1 and coagulation factor XIII (FXII) plasma can affect the risk for myocardial infarction [20]. Polymorphism of rs5918 (PlA1/A2) in ITGB3 correlates with coronary artery [21]. ITGB3 polymorphism may be implicated in blood platelet inhibiting the coagulation of aspirin [22]. Levine et al. [23] observed a remarkable increase in plasma PF4 in patients with coronary artery. In coronary heart disease, PPBP may work as a feasible synergistic inflammatory biomarker [24]. SPARC presents in the myocardial membrane, and its expression is elevated after myocardial damage and during fibrosis and hypertrophy [25]. VCL is a critical adhesion molecule connecting adhesion compound with extracellular matrix based on integrin [26]. VCL-SSH1-CFL is capable of mediating the maturation of cardiomyocyte myofilament [27]. These genes closely correlate with coronary heart disease and cardiac muscle cells as suggested in earlier investigations, possibly serving as a novel option for treatment target to enhance patient's cure rate. Lastly, coexpression network analysis was undertaken on smoking-related hub genes of coronary heart disease in GeneMANIA, and then, ITGA2B, ALOX12, ESLP, and CLU were acquired. Aspirin-associated gene ITGA2B correlates with smoking status of patients with cardiovascular disease [28]. ALOX12 promoter methylation changes in atherosclerosis might function as a new epigenetic indicator [29]. The polymorphism combination of CLU is implicated with DMDM status in coronary artery [30]. Additionally, these genes are involved in platelet degranulation and platelet activation, which are closely bound up with CHD [31, 32]. Thus, it was posited that the identified hub genes relevant to smoking in CHD might increase the cardiovascular events by polymorphism and platelet activation.

In conclusion, WGCNA, PPI, and pathway analyses were used to identify and testify 6 key genes of CHD related to smoking. The expression of these genes was then testified through animal experiment. They may be potential biomarkers of smoking-related CHD and assist the diagnosis and treatment of CHD for smoking people. However, further experiments need to be done to explore the specific molecular mechanism of the above hub genes that affect CHD risk.

## Figures and Tables

**Table 1:** PCR primer list.

F13A1	F: 5′-CAACAGCCACAACCGTTACACC-3′
	R: 5′-CTTGGATCAGCACCGCCTCTTT-3′
ITGB3	F: 5′-CATGGATTCCAGCAATGTCCTCC-3′
	R: 5′-TTGAGGCAGGTGGCATTGAAGG-3′

PF4	F: 5′-AGTGCCTGTGTGTGAAGACCAC-3′
	R: 5′-TTCCTCCCATTCTTCAGCGTGG-3′

PPBP	F: 5′-TGCTCTGGCTTCCTCCACCAAA-3′
	R: 5′-ACACATGCAGCGGAGTTCAGCA-3′

SPARC	F: 5′-GTGAAGGCAACATGAGGGTGCA-3′
	R: 5′-GTTGGAGGACAAGTCACTGGATC-3′

GAPDH	F: 5′-GTCTCCTCTGACTTCAACAGCG-3′
	R: 5′-ACCACCCTGTTGCTGTAGCCAA-3′

VCL	F: 5′-CCTATCAAGCTGTTGGCAGTAGC-3′
	R: 5′-TGTGGCTCCAAGCCTTCCTGAA-3′

## Data Availability

The data used to support the findings of this study are included within the article. The data and materials in the current study are available from the corresponding author on reasonable request.

## Supplementary Materials

Supplementary 1 Supplementary Table 1: clinical information of coronary artery disease patients in GSE20681 dataset.

Supplementary 2 Supplementary Table 2: 4,937 genes obtained by ANOVA.
