# Supplementary material for: A Signature for Smoking Status of Coronary Heart Disease Patients through Weighted Gene Coexpression Network Analysis
Source: Comput Math Methods Med. 2022 Jan 18;2022:5777946. doi: 10.1155/2022/5777946 (PMC8791244; doi:10.1155/2022/5777946)
Supplement: Supplementary 1 — Supplementary Table 1: clinical information of coronary artery disease patients in GSE20681 dataset. [file 5777946.f1.docx]

| id | gender | Smoking_status |
| --- | --- | --- |
| GSM518885 | 1 | 3 |
| GSM518887 | 1 | 1 |
| GSM518889 | 0 | 0 |
| GSM518891 | 1 | 0 |
| GSM518893 | 0 | 2 |
| GSM518895 | 1 | 0 |
| GSM518897 | 1 | 1 |
| GSM518899 | 1 | 0 |
| GSM518901 | 0 | 1 |
| GSM518903 | 1 | 0 |
| GSM518905 | 1 | 1 |
| GSM518907 | 1 | 0 |
| GSM518909 | 1 | 0 |
| GSM518911 | 1 | 3 |
| GSM518913 | 1 | 0 |
| GSM518915 | 1 | 0 |
| GSM518917 | 0 | 0 |
| GSM518919 | 1 | 0 |
| GSM518921 | 1 | 3 |
| GSM518923 | 1 | 0 |
| GSM518925 | 1 | 3 |
| GSM518927 | 1 | 0 |
| GSM518929 | 1 | 3 |
| GSM518931 | 1 | 1 |
| GSM518933 | 0 | 1 |
| GSM518935 | 1 | 0 |
| GSM518937 | 1 | 1 |
| GSM518939 | 0 | 0 |
| GSM518941 | 1 | 0 |
| GSM518943 | 1 | 1 |
| GSM518945 | 1 | 0 |
| GSM518947 | 0 | 0 |
| GSM518949 | 1 | 0 |
| GSM518951 | 1 | 0 |
| GSM518953 | 1 | 1 |
| GSM518955 | 1 | 3 |
| GSM518957 | 1 | 1 |
| GSM518959 | 1 | 1 |
| GSM518961 | 1 | 0 |
| GSM518963 | 0 | 0 |
| GSM518965 | 1 | 3 |
| GSM518967 | 1 | 3 |
| GSM518969 | 1 | 0 |
| GSM518971 | 1 | 1 |
| GSM518973 | 1 | 0 |
| GSM518975 | 0 | 0 |
| GSM518977 | 1 | 1 |
| GSM518979 | 1 | 0 |
| GSM518981 | 1 | 1 |
| GSM518983 | 1 | 2 |
| GSM518985 | 0 | 0 |
| GSM518987 | 1 | 1 |
| GSM518989 | 1 | 3 |
| GSM518991 | 0 | 0 |
| GSM518993 | 1 | 3 |
| GSM518995 | 1 | 1 |
| GSM518997 | 1 | 0 |
| GSM518999 | 1 | 3 |
| GSM519001 | 1 | 0 |
| GSM519003 | 1 | 0 |
| GSM519005 | 0 | 0 |
| GSM519007 | 1 | 1 |
| GSM519009 | 0 | 0 |
| GSM519011 | 1 | 1 |
| GSM519013 | 1 | 1 |
| GSM519015 | 1 | 3 |
| GSM519017 | 1 | 3 |
| GSM519019 | 0 | 3 |
| GSM519021 | 1 | 0 |
| GSM519023 | 1 | 0 |
| GSM519025 | 1 | 3 |
| GSM519027 | 1 | 3 |
| GSM519029 | 0 | 0 |
| GSM519031 | 1 | 1 |
| GSM519033 | 0 | 1 |
| GSM519035 | 1 | 1 |
| GSM519037 | 1 | 0 |
| GSM519039 | 0 | 0 |
| GSM519041 | 0 | 0 |
| GSM519043 | 0 | 3 |
| GSM519045 | 1 | 0 |
| GSM519047 | 0 | 0 |
| GSM519049 | 1 | 0 |
| GSM519051 | 1 | 0 |
| GSM519053 | 0 | 1 |
| GSM519055 | 1 | 3 |
| GSM519057 | 1 | 3 |
| GSM519059 | 1 | 0 |
| GSM519061 | 0 | 3 |
| GSM519063 | 1 | 1 |
| GSM519065 | 0 | 0 |
| GSM519067 | 0 | 1 |
| GSM519069 | 1 | 3 |
| GSM519071 | 1 | 3 |
| GSM519073 | 1 | 0 |
| GSM519075 | 1 | 0 |
| GSM519077 | 1 | 3 |
| GSM519079 | 1 | 3 |
| GSM519081 | 1 | 3 |
