# Supplementary material for: A Signature for Smoking Status of Coronary Heart Disease Patients through Weighted Gene Coexpression Network Analysis
Source: Comput Math Methods Med. 2022 Jan 18;2022:5777946. doi: 10.1155/2022/5777946 (PMC8791244; doi:10.1155/2022/5777946)
Supplement: Supplementary 2 — Supplementary Table 2: 4,937 genes obtained by ANOVA. [file 5777946.f2.pdf]

id	GSM518885	GSM518887	GSM518889	GSM518891	GSM518893
A2LD1	6.653830545	6.866597293	6.582439566	6.174647253	6.587993828
A2ML1	9.355403384	8.947623838	8.471488636	8.771294657	8.840590677
AAK1	6.92802298	6.37918304	7.300330596	6.878162091	7.013396273
AAMP	8.25927404	8.483763566	7.718747404	8.854603364	8.373157505
AANAT	7.623693616	6.967234434	7.560118414	7.318843495	7.183404394
AARS2	7.447717303	7.532708636	8.051625212	7.887186596	7.901917293
AATK	9.54312803	8.185563303	8.50111796	9.072403576	8.870056939
ABAT	8.247392788	8.163475424	8.160644788	7.423442323	7.645943414
ABCA7	10.69574272	10.35809493	9.139724848	10.83223099	9.718307192
ABCC10	8.24438497	7.734896879	7.977952253	7.733842657	7.799524616
ABCC13	7.709511535	8.289212586	8.474228909	7.541750556	8.736221495
ABCC3	8.354182626	8.991410131	7.891531071	8.800782677	7.911995121
ABCC5	8.086153313	8.145962667	8.144161414	7.71023803	7.792995667
ABCD4	8.182386152	8.419866586	8.423139949	8.094605657	8.109266465
ABCF1	8.090488788	8.345781111	8.713417869	8.867918475	8.593101434
ABCF3	8.466852202	8.318250424	8.163475424	8.759757687	8.445759657
ABCG1	8.676791091	7.537240869	7.143440162	7.550541152	7.360165061
ABCG2	7.162716495	7.603719626	7.599486586	6.880775747	7.944736172
ABHD1	7.535195111	7.21150504	7.353900202	7.250545919	7.616552222
ABHD3	8.904239657	8.266842545	8.879330566	8.390114414	8.119288848
ABHD5	11.00902763	9.881176566	11.11856721	11.06732533	10.6488688
ABHD9	11.26262606	11.25898056	10.68072859	10.77507269	10.86305205
ABI1	9.499247869	9.096947232	9.62024901	9.819688798	9.66831603
ABI3	9.023071899	9.047491869	8.60560303	9.431415586	9.17861001
ABLIM1	10.71613341	11.48840455	11.85301976	11.07013707	11.63401423
ABTB1	12.93430142	12.3613163	10.62764398	12.68484185	12.052647
ACAA2	8.903025495	9.585510535	8.600831152	8.835019636	9.202552434
ACBD4	9.003357768	9.759931222	8.185563303	8.726978374	9.147296667
ACBD7	9.131560131	9.452497313	9.002847848	9.318228697	8.838020616
ACCN4	6.456921838	5.915758535	6.585712788	6.540929384	6.203185697
ACCS	8.47107903	7.482605354	7.90331499	8.864430475	7.370079717
ACER1	8.901331343	9.749004051	8.911109919	8.838020616	10.08599138
ACHE	7.390207394	8.244006505	6.800782586	6.884558242	7.479706475
ACIN1	7.567690545	7.751645273	7.591105343	7.711178283	7.833640626
ACOT1	7.29078701	8.188779434	8.740195303	7.801006182	7.945864808
ACOT12	6.934051768	6.554596747	7.250238485	7.453021566	8.061425949
ACP5	10.22761479	10.63870636	10.12408141	10.61867663	10.13193078
ACPL2	9.436898869	8.095330333	8.346198293	7.932792414	7.795765162
ACRBP	10.56207051	11.1612024	11.08264222	11.53473316	10.62764398
ACSL1	13.03025597	11.10035391	12.37416009	11.97944932	11.91416468
ACSL4	8.612685364	8.108111747	8.102124455	8.226165	8.040196323
ACTB	14.05136004	13.84455043	12.96662998	14.35197184	13.85817003
ACTBL2	9.32739897	9.438799404	7.820605525	8.858692343	8.680072242
ACTC1	8.771294657	8.71185199	7.866306808	8.676791091	8.622644364
ACTL6B	6.992079758	8.009440313	6.10298801	6.715418303	6.918279333
ACTL7A	7.821971636	6.76459802	7.436640343	7.224700556	7.752360667
ACTN1	12.36473556	11.50425191	10.07956055	12.01427079	11.41605585
ACTN3	6.565175343	6.48772704	5.907643202	6.560645212	6.473654737
ACTR1B	8.601386131	8.226500707	8.842109	9.052474394	9.068490586
ACTR5	9.238221677	8.691979949	9.344138121	7.874403566	8.399553939
ACTR6	8.802385697	9.09178804	9.166446616	9.158023	9.054860525
ACVR1B	7.907197636	8.127056495	8.50618604	8.216823323	8.470647758
ACY3	7.665314131	8.020484273	8.350619919	7.990313949	8.395296687
ACYP1	8.351752182	8.821885465	9.551451798	9.036569232	8.943348051
ADA	10.21279484	10.76361324	11.00754401	10.76857843	10.75062885
ADAM10	7.838558162	7.647631172	7.161342051	7.305287707	7.205804788
ADAM17	9.252443646	8.881310737	8.891390919	9.084956182	9.194378495

ADAM33	8.291610121	8.108523939	8.527598192	8.280707687	8.692870869
ADAM8	16.02065153	14.6970922	15.22815255	15.72204116	15.16803204
ADAMTS5	6.776493808	7.006764162	6.124834283	6.955646677	6.460074192
ADAMTS7	9.528804131	9.915234909	8.418118394	9.400327152	9.402230293
ADAMTSL2	6.696224707	6.909921869	7.366646525	6.920433444	7.019424707
ADAP1	7.395300939	7.138031798	6.775746758	7.179153636	6.791252535
ADAP2	9.280885222	9.813557354	9.66831603	9.66613299	9.480384485
ADARB2	5.843842091	6.632101727	7.355706667	6.392522354	6.29488699
ADCK5	7.730154939	8.205457293	7.694191859	8.374810828	8.343220798
ADCY7	9.400327152	8.844732879	9.271123182	9.631972414	9.875361242
ADCYAP1R1	6.97260104	6.145681525	7.239398515	6.60058796	6.344827192
ADD3	10.81142627	11.1502402	10.5104744	10.9889639	10.73264046
ADFP	7.009209616	7.422831253	7.912365798	7.736964596	7.19003896
ADI1	7.918472515	8.210186616	8.533908051	8.104133364	7.805446323
ADIPOR1	12.52461444	13.23341018	12.32659908	13.05428976	13.42153085
ADK	7.590787525	8.063008152	8.59655801	8.222223545	7.892277636
ADM	12.25844332	10.46342714	11.6137143	11.38025327	11.47871073
ADORA1	7.36166898	6.669224869	7.902575141	6.791252535	6.669224869
ADRA1D	7.955993172	7.945111576	8.452374444	8.963499596	9.62845904
ADRB2	9.629101929	9.870576439	9.827596687	9.808672222	9.380344141
ADRB3	8.134790222	7.615524929	8.314398727	7.858964434	7.837840162
ADRM1	9.057828929	8.959130566	8.068200283	8.688838939	8.625486818
AGAP2	9.552159131	9.624391323	9.084956182	9.641787333	9.447242061
AGAP3	10.53842125	11.27153548	9.744663444	10.31597323	10.6820228
AGBL3	6.970769556	7.425203606	6.645437646	6.764835354	7.182269455
AGL	7.243824414	7.745466071	7.922041747	7.659186848	7.535866141
AGPAT9	10.23752804	9.55462702	9.293068828	9.695719162	9.013830768
AGRP	7.706911222	6.366139859	6.561721071	7.850180343	6.399871131
AGTRAP	8.341977727	7.688370495	7.335878859	8.178268222	7.779041818
AHDC1	7.297796364	7.11489098	6.984268657	7.202978657	7.506719576
AHR	7.788923101	8.374810828	8.489580414	8.06745399	8.14677703
AHSA1	8.398231626	8.030181424	9.395854121	8.823889374	8.627796121
AHSA2	10.36032821	10.85654613	10.99046505	10.8778129	10.59418919
AHSG	7.103272273	6.323128667	6.305549929	6.283913465	6.360006374
AIF1	14.66395575	13.99495772	14.31917195	14.7528858	14.01682491
AIFM1	8.832790172	9.139724848	9.477759242	9.157435455	9.300983919
AIFM2	8.455403657	8.65497299	8.123132566	8.200961636	8.468898455
AIM1	8.667554273	8.530767747	9.238865747	8.531247657	8.451947071
AIM2	8.774537848	7.709511535	8.33273403	8.41598997	8.986432162
AK1	9.890949131	10.86305205	8.953653768	9.84311402	10.05970796
AKAP1	8.840590677	9.463745455	9.59387599	8.80133401	9.212349869
AKAP10	8.582147707	8.442251212	7.764199131	8.701109788	8.543234566
AKAP7	7.641344242	8.212685152	8.379056919	8.125376242	7.856753939
AKAP8L	9.586238192	9.229082869	8.197004869	9.30770201	9.205404404
AKNA	7.673376626	7.470612081	6.515423162	7.314926455	7.195783737
AKR1B1	7.970574879	8.401710747	8.955359253	8.443547162	8.396963808
AKR1C3	8.303793737	8.33024502	8.507211606	8.685201879	7.736964596
AKT2	8.491898242	8.86024204	8.076845152	8.453639616	8.267244596
AKTIP	6.957895121	6.406814333	7.017519192	6.531066697	6.453641556
ALAS2	16.36806566	17.12376032	17.67797782	16.73234105	17.67797782
ALDH1A1	7.359873626	7.997183333	7.832217929	8.344441616	7.832217929
ALDH2	10.1250404	10.50914282	11.00183361	10.2166926	10.15585749
ALDH5A1	6.449076475	7.488608758	7.41731598	7.481001515	7.240898899
ALDOA	15.31193978	15.22815255	14.37933382	15.41210544	14.99939481
ALDOAP2	9.874496818	9.679645121	7.604109192	10.20859114	9.887613414
ALG3	8.686126354	8.913588253	8.810446485	8.618800343	8.499836475
ALOX12	8.019769071	7.762120596	7.870669919	7.771540485	6.832875889
ALOX5	14.65397593	13.35919327	13.35044872	13.80033164	13.55640443

ALOX5AP	13.70717091	12.48835313	12.44950322	13.02518615	12.91656586
ALOXE3	7.602282465	7.064817384	6.654064545	6.909691071	6.75222999
ALPK1	11.86401838	11.01209098	11.55928848	11.40306525	11.56988377
ALPL	8.257984293	7.658148485	6.946968899	7.373377929	7.507381747
ALPP	8.262596626	7.982442283	7.788532485	8.122341	8.022369434
ALPPL2	8.555833323	8.671294253	8.396963808	8.352537101	8.771856424
AMD1	11.56988377	11.26815786	11.08936677	11.58814301	11.58814301
AMFR	8.123471404	8.944409727	7.209785606	7.524103909	8.554971
AMICA1	13.95955505	13.14093405	13.88591407	13.80704919	13.50411017
AMIGO2	6.943317879	7.601973414	7.774283859	7.130177747	7.044969556
AMN	9.791460222	11.59021288	8.844262616	9.361074667	10.35178281
AMPD2	10.9889639	10.3896003	9.458377051	10.86447048	10.3810842
AMPD3	9.722667242	9.726443586	8.803935202	8.82789604	8.853585596
ANG	7.938205586	8.272231939	8.140128606	7.599211495	8.060661899
ANK1	7.849463182	8.609952768	8.309247939	8.037984616	8.879851283
ANK2	5.85114799	6.004759152	6.194928222	5.853358202	5.951941121
ANKDD1A	7.340746384	8.509065354	6.403910828	6.629727051	6.886152545
ANKK1	16.28343863	15.59877501	16.8710039	16.68963031	16.4534382
ANKMY2	6.668735424	6.202989697	6.415279879	6.615899091	6.018274323
ANKRD10	8.353342091	8.287081333	9.013314737	8.460488909	8.233006697
ANKRD13C	6.907093253	7.393167162	6.538425424	7.056715131	7.327495141
ANKRD13D	9.913634949	9.064430111	8.127495596	9.393335687	9.178084667
ANKRD18A	7.186679828	7.128773535	6.924186162	7.529315566	7.734170172
ANKRD22	7.531511131	6.873794323	7.665987182	7.353900202	7.413750677
ANKRD26L1	7.315524667	7.49893601	6.387499121	7.067324051	6.809755232
ANKRD33	14.45281427	14.86987024	14.28325373	13.72760196	14.10328164
ANKRD33B	9.025691242	9.493200828	8.675348646	8.716864899	8.573273283
ANKRD35	7.154007354	7.258693172	8.24122704	7.638981707	7.541090081
ANKRD36B	7.600248455	7.97830402	8.338686465	8.207926798	8.370830747
ANKRD37	7.438614949	7.530907465	7.451512687	7.051937434	7.342871717
ANKRD58	11.3290504	11.01807842	11.17041501	10.76737368	10.64623997
ANKRD9	8.081502051	9.997192202	8.735287485	8.220225172	9.291393586
ANKS3	12.03310273	13.27940818	12.38794592	12.43944324	12.97977545
ANO8	6.852390212	7.252038162	5.994768081	7.146637616	7.358688323
ANO9	7.221626808	7.532150707	6.734222485	7.641683626	7.627373273
ANP32B	9.288999586	9.513795939	9.648698737	9.723272263	10.29949835
ANP32D	8.022369434	7.086968939	7.409045616	7.20183702	6.845837626
ANPEP	10.84705809	10.42297323	8.549141545	10.75178718	9.937540263
ANXA1	12.94367069	12.97555423	13.32669092	13.35359128	12.92937388
ANXA2P1	9.031713778	9.321347081	9.571117879	9.405539273	9.263851586
ANXA3	10.34100163	8.849981545	9.713858919	9.595333525	9.733950273
ANXA4	8.461315273	8.82842597	9.314493212	8.609952768	8.887684293
ANXA5	9.58708204	9.675185677	11.05356995	10.18762619	10.16937171
ANXA6	8.007258162	8.079973212	7.741457929	8.326884343	8.009440313
AOAH	9.682548525	8.465000141	9.198999222	9.666877556	8.65914896
AOC3	8.386601707	7.510343899	7.828991111	7.687700545	7.380686859
AP1G2	8.631321253	8.566415323	7.633560879	8.40783399	8.24281297
AP1M1	9.254253192	8.868452172	7.813414727	9.451212687	8.656360697
AP1M2	6.619744263	6.968393455	6.323529697	6.918844495	6.959189505
AP1S2	10.45754412	10.67815746	10.34311059	10.82945836	10.97322727
AP2A1	7.063754707	7.697220202	6.524953909	7.078018687	7.124332475
AP2B1	6.768172747	7.116341071	6.708558414	6.756409455	7.084441778
AP2M1	9.818860343	10.46766096	8.922632727	10.00985275	10.35284213
AP3M2	6.821005303	7.342557798	7.345589182	6.726480707	7.118793697
AP3S1	10.80782516	10.44832821	10.90324033	10.70181173	10.65987528
APAF1	8.706127333	8.385691051	8.063461202	8.475210818	8.19621002
APBB1	8.048299333	7.629989283	7.446082798	7.935081758	8.314398727
APBB1IP	11.70348036	10.78856661	11.36793803	11.43938222	11.23280633

APBB3	9.109120374	8.328154263	7.812327374	8.76626204	8.660608859
APH1B	8.925229707	8.519178475	9.096947232	8.514037303	8.583048444
APOB48R	12.56726296	11.671419	11.50632759	12.32964397	12.10951308
APOBEC3A	13.09045797	11.65052704	12.59323492	11.95261936	11.9982543
APOBEC3B	11.87445957	9.415860727	10.95533553	11.36610866	10.73661507
APOBEC3C	7.45822897	7.89560499	6.84909404	7.791533949	7.844550919
APOBEC3G	8.167383949	8.343220798	8.566415323	8.31049196	8.411696677
APOBEC3H	6.234440616	6.170864828	6.142392444	6.357131071	6.585495889
APOL2	9.256767414	8.917682242	8.91530399	9.285000091	9.004398101
APOL6	6.953914061	7.287828434	7.879296202	7.629344343	8.133993636
AQP1	7.269598859	7.969427899	7.783110646	7.173915838	7.604109192
AQP10	7.925642	7.956615091	7.925310818	7.639990515	7.357173859
AQP2	8.726978374	9.11211799	8.433939838	8.074904687	8.569209263
AQP3	7.790916071	7.565422434	7.93027604	8.014589606	7.796432081
AQP9	14.47046648	13.09977851	13.46005067	13.69473258	13.46712151
ARAF	8.007640657	7.984708455	6.92551101	7.97471396	7.590248525
ARAP1	12.08128174	11.3290504	10.36349344	11.76336308	11.05191968
ARAP2	8.972927475	8.557697889	8.591239465	9.180906778	9.061649657
ARAP3	11.53239215	10.82945836	10.71613341	11.49239347	11.11388317
ARF5	9.90675497	9.441234879	9.031155364	9.513795939	9.196132455
ARFGAP2	7.301216929	7.089652374	6.531281859	7.564741838	7.035145869
ARFIP2	7.872718384	7.933573889	7.076304455	7.821271606	7.861535657
ARG1	11.22294817	8.822318	10.09506496	10.20532801	9.209526556
ARGLU1	9.505859384	9.265583747	8.115023838	9.568406747	9.126663535
ARHGAP12	9.140392293	9.187808616	8.816214848	8.929384727	8.972363838
ARHGAP18	8.08075902	8.130283051	6.996514071	8.096910323	7.689572788
ARHGAP26	9.149684939	9.041236566	9.727935707	9.045755677	9.533596364
ARHGAP27	8.907915899	7.802503596	7.004269242	8.637033606	7.813018717
ARHGAP30	9.961780586	9.01707996	9.044608384	10.13862753	9.143918798
ARHGAP9	14.1282639	12.86955734	13.27940818	13.66784462	13.34730616
ARHGEF1	8.373157505	8.085771838	7.64030596	8.518658667	8.123132566
ARHGEF10L	9.481000434	9.385290182	9.391460545	10.09426568	9.501970121
ARHGEF15	9.120933788	7.936282586	8.017104828	9.143279535	9.080683394
ARHGEF16	7.595878485	7.933221424	7.136875657	7.487351192	8.100639
ARHGEF18	12.01673563	11.72126504	12.2031413	11.40725584	11.38391359
ARHGEF2	8.475612051	8.157901343	8.702574091	8.080939525	7.980934222
ARID1B	9.157435455	9.204345657	8.482406475	8.336257222	8.832790172
ARID3A	9.93187304	9.216574283	8.269818727	9.615324495	8.908475283
ARID5A	10.72246175	10.06071544	9.320176919	10.20964764	10.06356315
ARL11	7.866306808	7.177746566	7.528640475	7.678591667	7.650531152
ARL16	8.681067818	8.669446475	9.769994879	9.057828929	8.924201657
ARL17P1	8.509936919	7.542431869	8.62991701	7.766738848	8.020484273
ARL2BP	9.521972354	10.03983393	9.399129424	9.875361242	10.36589334
ARL4C	9.685649929	9.631182697	9.206631485	10.61371787	10.17122401
ARL6IP1	10.21054893	10.83930015	11.02551486	10.45041513	10.77103399
ARMC1	7.567404636	7.924502232	7.877844556	7.799854778	8.232163879
ARMC5	8.204737434	8.158968566	8.60413102	8.86024204	8.567801737
ARMC6	8.629055081	9.307024131	8.021628354	8.681067818	8.688838939
ARMCX2	6.51170598	7.128516444	7.188612899	6.480794707	6.641155141
ARMCX5	6.962306323	7.381324374	7.672658859	6.936612586	6.950382242
ARPC1B	12.65819872	12.24351077	12.55625498	12.4571909	11.85617239
ARPC2	14.80749806	13.9071606	14.3093669	14.85646221	14.14208591
ARPC3	15.27762024	14.79546777	14.58095582	15.16803204	14.66395575
ARPC5	13.39659876	12.84197228	13.15082863	13.17281418	12.88265743
ARPC5L	8.481411768	8.538919798	8.87289497	8.491049758	8.447526838
ARRB1	7.578291899	7.515816273	7.067324051	7.924172566	7.518430525
ARRB2	13.16151113	12.31419772	11.97944932	13.09463792	12.3843369
ARRDC2	9.632768747	9.379720636	9.31327601	10.19332507	9.776839404

ARRDC4	8.50673198	9.53690398	8.833337414	8.337550606	8.613985131
ARSA	9.634173323	8.547449101	7.967920667	9.185497838	9.149195859
ARSD	7.647631172	7.190644495	7.287828434	6.863923949	7.249375465
ARSG	7.049798838	6.670140313	7.698928879	7.036743576	7.22559698
ARTN	8.460488909	9.688441414	7.627373273	7.784199556	8.289996192
ASAH1	9.163332636	8.460863192	9.228437333	9.452497313	9.19736601
ASB16	7.738299929	8.369612525	7.952386505	7.555661677	7.887186596
ASB2	7.164674212	8.006915303	7.328995606	7.381652111	7.279114576
ASCC2	9.435722162	9.641787333	8.638487899	9.984347768	9.568406747
ASCL5	7.839647131	8.744081071	7.661901192	7.935468505	8.116212253
ASF1B	8.53579602	7.614831859	8.015705778	8.290353162	8.509936919
ASGR1	10.89755671	10.65500785	12.45390404	11.25349741	11.36423723
ASGR2	10.08799683	10.74558579	10.32849396	9.702758343	9.93187304
ASNA1	8.359650919	8.701614283	7.274095485	8.325316889	8.218433131
ASNS	9.021393263	8.423588242	8.862225737	9.000069889	9.148511889
ASPHD1	7.223246162	7.127106434	6.578698909	7.154937303	7.732550404
ASPRV1	12.06098883	11.23116994	11.76336308	11.58005536	11.35475345
ATAD2B	7.989899475	8.038729465	7.152420131	7.620680879	7.60736902
ATAD3C	10.93789932	12.63888703	9.733230051	10.35069541	10.8254891
ATF1	6.544097556	6.704711566	6.956897717	6.895207929	6.757183747
ATG16L2	15.48449671	14.4335752	14.9847254	14.94534759	14.45281427
ATG2A	9.068490586	8.805485081	7.683318778	9.499917182	8.793874616
ATG9A	9.09178804	9.400967465	7.886079293	9.099925818	9.411394242
ATHL1	11.83072395	11.17378617	9.992988556	12.24097391	11.8358519
ATIC	7.848096879	8.478760515	8.692466525	8.495447152	8.503061444
ATM	7.239727717	7.53302901	8.59026096	7.966054535	8.24438497
ATN1	8.750916455	9.647889364	7.827906879	8.306613333	8.549141545
ATOH7	7.51310204	7.216096788	7.526167626	6.865784889	7.094132182
ATOX1	8.341518747	8.30620801	8.539328929	8.211847566	8.381754576
ATP13A1	7.326350616	7.57799899	6.900488939	7.673376626	7.520992313
ATP1A1	9.389466253	9.490338525	9.396536798	10.1597097	9.096395636
ATP1A3	7.462771485	7.304711495	7.20918101	7.848409778	6.993764091
ATP1B3	10.761049	10.96162926	11.6295292	11.29076429	11.65052704
ATP2A3	8.417196576	8.31318196	6.881026485	8.240375404	8.033283465
ATP5A1	10.51400776	10.7532413	11.9577766	11.2278827	11.10460602
ATP5E	13.95167504	14.14208591	14.11151028	14.09521817	14.5513462
ATP5L	10.67815746	10.79956984	11.15183093	11.11388317	11.08107282
ATP5S	7.575132364	8.487847515	7.95565902	7.799196616	8.236633323
ATP6AP2	10.24038579	9.894213939	10.76983035	10.29851595	10.24612484
ATP6V0A2	7.148452788	7.149049263	7.678926152	7.165538758	7.28082202
ATP6V0B	12.48835313	11.76804793	11.48258282	12.27421104	11.69440164
ATP6V0C	13.90036211	14.09521817	13.36582475	13.81266894	14.28325373
ATP6V0D1	10.91903182	10.36589334	10.17904437	11.09885051	10.78705857
ATP6V0E1	9.510536	9.148511889	10.0053333	8.957944414	9.078885242
ATP6V1B2	11.77802923	10.93171462	12.13867659	11.82112126	11.3733012
ATP6V1D	8.81572204	8.589797808	9.833438364	8.899757485	9.125507586
ATP6V1E1	8.971160737	8.598479657	9.712341424	9.11393401	8.866888202
ATP6V1G1	11.17910752	11.14385742	12.09804166	11.63174178	11.49636188
ATP6V1G2	9.719000626	9.724814657	9.619644949	8.395296687	9.293068828
ATP7A	7.340448859	7.079375364	6.969691626	7.08589403	6.811363364
ATP8B4	6.778194636	7.065642788	6.535531162	6.55365803	6.612991111
ATXN10	6.989960667	7.028571808	8.722953263	7.97284099	7.843077242
ATXN1L	8.640789455	7.902229798	7.823083152	8.445759657	9.005975162
ATXN7L2	8.528915121	8.974101818	8.568242293	8.718321818	8.886620687
AUP1	9.55462702	9.408173051	8.565878434	9.859664869	9.211184727
AVIL	8.35796399	7.931708101	8.178694051	8.19621002	7.615524929
AVP	7.815915242	8.558108202	7.178323677	7.357173859	7.431067202
AXIN1	7.966742939	7.768402071	7.147201253	8.327353333	8.000144121

AXL	7.19832496	7.491021444	6.995550687	6.530395293	7.049798838
AXUD1	7.515531818	6.728952394	6.702267949	7.185402798	6.700834859
B2M	16.33688632	15.24813401	16.28343863	16.14978837	16.02065153
B3GALNT1	6.701095535	6.603384646	6.508927202	6.796831899	6.985120646
B3GAT1	6.686049697	6.420787636	6.898276768	6.611655717	7.692217293
B3GNT5	9.575401111	8.63568099	8.769354545	8.647922808	8.994589434
B3GNT6	6.744595263	6.660795162	6.943317879	6.962574283	7.271416848
B3GNT8	12.47130519	11.32721575	11.17910752	11.89876792	11.66934136
B4GALNT1	7.541384646	7.522209596	7.095275111	7.231823384	7.222702545
B4GALT1	9.692043475	9.089024899	8.940148162	9.470840162	9.405539273
B4GALT3	9.469429253	9.574750414	9.302735434	10.22448985	9.821914929
B4GALT5	9.847335909	9.323195364	9.374690616	10.06255975	9.715870253
B9D2	11.18471248	10.73264046	10.6362571	10.5431016	10.41731261
BACE1	7.998587576	8.510894717	8.187968455	8.002717273	7.732206152
BACE2	7.381324374	7.799854778	8.39867397	7.523206263	7.271131505
BACH2	6.930743303	7.355380455	7.82717702	7.320252869	7.529315566
BAG1	15.95256028	15.97330865	16.61084374	16.4534382	16.3077943
BAHD1	9.060618192	9.146732444	9.547339828	9.398445515	8.996148737
BAIAP3	7.159001404	6.810582646	6.219828162	7.378508566	7.121055596
BAK1	8.831201768	8.691458141	8.064713051	9.344138121	9.062782333
BAMBI	7.89560499	7.034867141	7.094132182	7.047846657	7.233950838
BANK1	8.555429434	8.823394192	8.949261586	8.452374444	8.85308204
BAP1	7.980568657	7.831138545	6.975188859	8.073652495	7.87472
BASP1	14.83117585	13.32021166	14.010587	14.4335752	14.07988992
BAT2	7.577325354	7.36076402	6.59799604	7.311037152	7.095275111
BAT2D1	9.873591818	9.818860343	9.735412273	10.1905069	10.20859114
BAT3	12.0750029	12.84627851	11.2278827	12.12939166	12.24933389
BATF	10.04682541	9.921211657	10.95683616	9.902579253	9.917863333
BATF2	8.695776131	8.257595869	7.790916071	7.678926152	8.089360869
BAX	8.445759657	8.387507626	7.587331949	7.149668323	7.781852747
BAZ1A	10.04511943	9.830354566	10.21484784	9.834210131	9.843921515
BAZ2A	8.939019091	8.802954374	8.815152444	8.678658293	8.550414556
BBS10	7.644277273	7.760648222	6.794878162	7.081067566	7.039966242
BCAM	15.76560628	16.68963031	15.02501841	15.56435861	16.25437967
BCKDHA	9.622300838	10.65014074	9.332175414	9.521192909	9.713188212
BCKDK	9.406893465	9.084956182	8.009440313	9.016491202	8.877626323
BCL2	8.390560192	9.500664495	9.489614919	8.907429495	9.487680697
BCL2A1	12.22629088	11.06261544	11.84027237	11.85617239	11.46724679
BCL2L1	8.959130566	9.873591818	8.019769071	9.422377343	9.783680727
BCL3	13.17778072	12.04734694	12.40735915	12.06964699	11.94745099
BCL6	14.14208591	12.50706636	13.63435472	13.39037726	13.16824615
BCR	8.39308204	8.214296636	7.533990333	8.479727162	8.058246657
BEGAIN	6.373570848	6.073352293	7.417598061	7.367866364	7.098999354
BEND3	6.766293242	6.748847646	6.113469414	6.29488699	6.533325687
BEST1	7.695265798	7.280239242	7.444499747	7.313451111	6.92669298
BEST2	7.041898303	6.625311455	7.042119313	6.896711899	6.326506525
BEST4	10.52689599	11.20163629	10.81664077	10.03299879	10.66342196
BEX2	8.560860505	8.972363838	9.872596081	9.272374697	9.072998758
BEX4	7.063477202	7.660499434	8.791657869	8.187593091	7.952029848
BFSP1	6.765793475	6.943550111	6.876146636	6.94985404	7.102685081
BHLHE23	13.04381444	14.38889659	10.35699559	12.09497108	12.62449502
BHLHE40	7.161605929	7.232151747	7.452109758	7.050623333	6.967663404
BID	11.76336308	11.11560115	11.56291631	11.32027501	11.21338774
BIN1	9.139095455	9.344897333	8.984231152	9.402814081	9.307024131
BIRC2	9.059459505	8.931557869	9.838413929	9.154469939	9.518503263
BIRC3	10.35699559	10.87518762	10.33252085	10.38001468	10.48185706
BIRC5	6.585931495	6.830079818	7.013931768	6.582941808	6.372952485
BLK	8.159907626	8.174069929	7.377269152	7.572084505	8.190781646

BLMH	8.524867051	8.652865111	9.483552818	9.148511889	9.131560131
BLVRA	11.18291631	11.542568	12.02240324	11.52448673	11.59427705
BLVRB	13.14093405	13.14611959	13.55640443	12.57486869	12.99565877
BMF	7.870978364	7.962004212	7.696877364	7.989899475	7.540114586
BMP6	7.460903263	8.129067798	8.079973212	8.064713051	7.777661384
BMX	8.255317818	6.948047899	7.880750535	7.158043384	7.055635202
BNIP2	10.87235819	10.70775781	10.68338253	10.55213977	10.2829717
BNIP3L	12.7939548	13.07947925	13.17778072	13.06488572	13.87958112
BOK	7.65457904	7.177177071	6.981335535	7.364797848	7.741148566
BOLA3	7.246997929	7.427086202	8.100222556	7.387654737	7.802503596
BOP1	7.928481586	8.41382397	7.466652071	8.470647758	8.302498081
BPGM	10.42062148	11.28053313	11.72355631	11.07493153	12.55983323
BPI	6.447674828	6.003663404	6.54114802	6.967928273	6.299772566
BPIL1	11.04464523	13.11515098	9.87177298	9.997192202	11.542568
BRD1	10.35178281	10.4994288	10.4283734	10.23160947	9.927686616
BRD3	8.775519707	8.484240687	8.436995596	8.990220404	8.448456354
BRD7P3	6.886441253	6.597017333	8.07053003	7.129307667	7.070666535
BRI3	15.15443825	14.25042984	14.62217632	14.1830221	14.02572128
BRP44L	10.35069541	10.29000276	10.43440935	10.11051676	10.36903954
BRPF1	7.898595626	7.695912303	6.831893455	7.705454838	7.197080263
BRWD2	7.733228788	8.072110051	8.749910828	7.57049196	7.943433747
BSDC1	11.24457024	11.39382595	11.38577951	11.75215821	11.61117883
BSG	9.516398909	10.15875628	9.625623061	9.322592778	10.10841911
BST1	11.18941159	10.27714677	11.19638858	11.04156144	10.43872423
BTBD2	10.94496034	9.175472828	10.85654613	10.08231743	9.895856061
BTF3	11.86125412	12.23505088	12.68097339	12.19727311	12.25844332
BTG1	12.2031413	11.64637724	12.27101	12.25251148	11.93770961
BTN2A1	8.429582192	8.067838768	8.113343889	7.979449616	7.841440677
BTN3A2	10.4283734	10.40387449	10.8254891	10.88302044	10.81280665
BTNL3	5.770458121	8.765233303	5.831211101	10.23660558	5.795333303
BTNL8	11.28565422	9.671912455	11.04030995	5.802397606	10.65246846
BUD13	10.30058205	10.48897537	10.78705857	10.11664425	10.12697515
BUD31	9.010430586	8.600831152	9.629839465	9.419020677	9.214111545
BXDC2	7.569572879	7.967524909	8.926362727	8.145194646	8.227270172
BZRAP1	8.400903717	8.273061586	8.209801687	9.192533242	8.088956818
C10orf114	6.415689222	6.275054576	6.829338141	6.716118697	7.226157253
C10orf118	8.094605657	7.623693616	7.495760485	8.033283465	7.967113929
C10orf27	8.835019636	8.143040162	8.116212253	8.687541707	8.933180404
C10orf35	6.92164503	7.143149899	8.000883182	7.359577545	7.273507545
C10orf54	12.95303817	11.8164662	11.13879804	12.90359001	12.27101
C10orf62	6.392522354	5.794044828	5.752387838	6.244753081	5.768296495
C10orf78	7.349181162	7.678291495	7.816627253	7.933221424	7.82717702
C11orf31	12.33907422	12.83405786	12.89113428	12.74093701	12.81735602
C11orf42	7.768055657	7.122138172	7.489478697	6.565610293	7.031184939
C11orf68	9.909202788	9.591816061	10.19649023	9.857940545	10.28506122
C11orf73	9.434425707	9.738398616	10.39072525	9.798623747	9.855298394
C11orf75	10.45628541	10.1684149	11.542568	10.49140257	10.84302834
C11orf9	7.633913808	8.051625212	6.913266485	7.561051303	7.294284717
C12orf10	10.06904603	10.4283734	9.687048303	9.789981192	9.959124273
C12orf23	8.263464364	8.85518403	8.956909848	8.816703535	8.989652131
C12orf27	6.560645212	6.355908172	6.195589626	6.811363364	7.289658101
C12orf35	12.02240324	11.27306561	11.3403052	11.56725755	11.34576518
C12orf57	10.44934262	11.22600179	11.6157426	10.74819911	10.95683616
C13orf15	9.346872071	10.4655823	10.67305197	9.812775374	10.67815746
C13orf18	8.513135323	7.874063444	8.19458801	8.270601414	7.792587404
C13orf27	7.542783869	7.759260374	7.952029848	7.955335374	7.468171747
C13orf38	8.906910121	8.731576192	8.062539303	7.144029273	8.238272808
C14orf101	8.743617505	8.423139949	7.836828747	8.072924455	8.142577798

C14orf138	9.007167758	8.504327141	8.929972798	8.598980253	8.145617202
C14orf139	14.16629104	15.16803204	13.80704919	13.41514643	14.60195877
C14orf147	8.138908141	8.59026096	8.03601997	7.938531869	8.211847566
C14orf162	6.941709929	6.796566808	7.222444626	6.538664657	6.590229374
C14orf166	11.47682743	11.92396137	12.22629088	11.9091429	11.94234713
C14orf169	8.050484323	8.493653778	8.058592364	8.266388465	8.337114525
C14orf4	9.368224626	9.427546879	9.526870273	8.775001677	9.319510202
C14orf43	9.751885293	10.54800525	8.981479273	9.293710889	9.370832535
C14orf45	6.968156515	8.066736566	8.667049253	7.201015091	8.44129198
C14orf56	6.643064293	5.766159869	5.750733778	6.535299384	6.426632697
C14orf68	8.220225172	8.673363869	7.252580253	6.795847303	8.350619919
C14orf94	7.995268899	7.457078101	8.383846919	8.137860071	8.066048727
C15orf21	14.33555643	13.74093847	14.34357305	14.64328928	14.24162707
C15orf23	9.162016475	8.793874616	8.53537201	8.182386152	8.22479296
C15orf29	8.581684657	7.940254566	8.280295667	8.305359949	8.247731232
C15orf39	11.22119175	11.08435313	10.7982674	10.76609029	10.84568098
C15orf5	7.094450071	6.860390606	6.447481424	6.562503909	6.464011586
C15orf52	7.827906879	7.82475102	8.254919778	8.184050869	7.585519525
C15orf61	8.347717242	9.121511687	9.126663535	8.239938859	8.670447949
C16orf11	9.450568111	11.66934136	8.003903253	8.255317818	8.721054424
C16orf13	13.92172837	14.66395575	13.50997822	13.15645152	13.87263802
C16orf14	8.675789182	8.485174677	8.456191465	8.628622212	8.855784909
C16orf3	12.46466592	14.06476988	11.57178154	11.66189432	12.90783816
C16orf35	11.30301738	12.47761486	10.81796524	12.39058663	11.47322198
C16orf54	7.263859848	7.171589687	6.317874636	6.785769293	6.466022525
C16orf59	6.828622869	6.386460131	6.882244081	6.882010616	6.976233182
C16orf67	7.392218323	7.723009	7.725703576	7.927799515	8.293769586
C16orf7	9.729555121	9.649399939	8.801904485	9.885156737	10.15086094
C16orf73	7.332078838	5.760130717	5.758874768	7.208321485	5.774262081
C16orf74	7.712908172	8.046104414	8.285312424	7.29687102	7.034867141
C16orf81	10.76983035	11.17041501	10.11452638	10.29949835	10.89224764
C16orf88	6.698080333	7.279410566	6.985120646	6.822666394	6.92644801
C17orf54	7.470329323	7.560401535	7.539167263	7.125696242	7.175476838
C17orf60	7.011288768	6.959189505	7.122424869	6.904054566	7.303307
C17orf61	8.897177899	9.505258828	10.1905069	9.312041414	9.598893818
C17orf62	10.18291229	9.582761798	9.049629657	10.18563382	9.565032515
C17orf63	8.731005677	8.956909848	7.933961929	8.635209293	8.733083212
C17orf65	11.52645916	11.68700199	11.7349036	11.22119175	11.93178106
C17orf70	8.347358778	8.716387192	7.637927172	9.081927515	8.714332636
C17orf72	10.14432724	9.271759586	9.312041414	8.608532485	9.155642525
C17orf74	7.892936636	6.610658212	6.977291071	8.848410616	6.568165434
C17orf76	10.04340808	10.09161001	9.804037697	9.491725303	9.925226333
C17orf87	6.941957697	7.418256545	6.115154242	6.427492121	6.17279397
C17orf89	9.318836889	9.922126404	10.49140257	9.786685869	9.817379515
C17orf91	9.952967535	10.93023415	9.761765848	9.789131121	10.47820756
C17orf96	15.70163214	16.36806566	13.09977851	15.34617167	15.63778652
C18orf10	8.883993253	9.905938879	9.671912455	9.215946081	9.634846707
C18orf12	10.71390019	12.16942168	9.424985495	9.692043475	10.87235819
C18orf22	7.441392919	7.767042424	8.549517636	7.736260545	7.865908465
C18orf23	10.9660467	11.45002291	9.899969596	10.59062542	10.95533553
C18orf25	7.727729838	7.446760616	7.961295828	7.276635293	7.039183869
C18orf32	7.754407434	7.320933798	7.222702545	7.424653535	7.411868313
C18orf49	6.371987444	6.230315707	6.383834626	6.494784798	6.321469101
C19orf22	11.17213305	11.68243664	9.863774293	11.9875501	11.42787377
C19orf23	8.750392838	7.988882495	8.318250424	7.424024737	8.582147707
C19orf29	7.880363606	8.883414061	7.364797848	7.74961303	7.979092162
C19orf31	9.614560172	10.66587479	8.743617505	9.573320202	9.681142253
C19orf33	7.521885657	8.305787747	9.331038253	7.205804788	7.84876403

C19orf38	11.09089144	10.56908161	10.98350754	10.89604789	10.4283734
C19orf44	7.822730929	8.437898414	7.767702525	7.548584576	7.728044798
C19orf59	10.12909315	8.904793061	8.83948901	9.642500081	9.574750414
C19orf61	6.821271707	6.735177838	6.20939903	7.071197202	6.968156515
C19orf62	10.10278369	10.84974643	9.843921515	10.16743096	10.01974654
C1D	8.464081152	8.308331848	8.671294253	7.596246919	8.08193004
C1QA	7.432070859	6.151165091	6.612514242	6.741002212	6.77698199
C1QB	6.908606212	6.139758162	6.218400657	6.434478081	6.056728475
C1QBP	10.11555853	10.65634987	11.29597305	10.41330878	10.93494087
C1RL	8.472925747	7.976858394	8.387939343	8.17219503	8.363019899
C1S	8.745043505	7.40526796	8.596127869	7.988582505	8.043387838
C1orf109	6.862895808	7.194952889	7.435155859	7.071486061	7.227000899
C1orf113	8.273483707	9.254253192	7.426469556	7.882850313	8.201745222
C1orf128	8.047188455	8.35018502	9.788274313	8.160266646	9.206093556
C1orf152	11.52824	11.54675633	10.79686873	11.77802923	11.79615897
C1orf156	8.016429949	8.283330131	8.130283051	8.570262	8.344860929
C1orf158	8.968391182	10.92190902	7.477539293	8.405727848	9.036569232
C1orf174	9.817379515	9.848945263	9.661659101	9.892629697	9.716648152
C1orf175	7.33350399	7.21346404	6.887565899	7.022558657	7.412806768
C1orf183	10.32093584	10.09254304	10.50914282	9.94402302	9.452497313
C1orf186	7.483852838	6.828875111	6.835859525	6.521234222	6.447271273
C1orf187	6.546653242	6.850381687	7.259665485	6.858224505	7.203245626
C1orf198	10.07107569	10.05426836	10.06255975	9.815129384	9.791460222
C1orf31	8.532516465	8.939019091	8.907429495	9.003357768	9.445852485
C1orf38	12.73352188	11.60266156	12.18142186	12.69676846	12.11562205
C1orf43	9.244930505	9.203760495	10.03385507	9.247274333	9.042346232
C1orf52	7.719466677	7.422224808	8.746311293	8.039451586	7.828584192
C1orf54	8.621689657	9.168324747	9.438799404	8.716387192	9.019731374
C1orf63	10.3548828	9.445852485	10.38842688	9.924448444	9.757590192
C1orf64	7.005636414	6.554821889	7.624016909	7.14635899	7.314616131
C1orf97	7.235668455	7.28903701	7.506719576	7.595597162	6.968641576
C20orf103	7.688949081	8.250622253	7.511268929	7.361082222	7.44112097
C20orf106	9.011040293	8.830660505	9.242021586	8.436995596	8.480158333
C20orf108	11.92665931	13.05428976	12.83405786	12.33576886	13.01016522
C20orf141	9.806332162	10.67572435	9.431415586	9.362238061	9.873591818
C20orf144	7.883157929	7.619933919	7.304125475	7.853698798	7.519990596
C20orf149	12.44264271	13.58209631	13.22156044	12.73731843	12.62127559
C20orf24	12.797444	11.94979793	12.41634374	12.06692054	11.82616001
C20orf3	12.00354228	10.41957053	11.24816417	11.73710477	10.84176532
C20orf54	6.797074616	6.10953001	7.282635596	7.25402203	7.055367566
C20orf59	7.853698798	7.67441401	8.817191758	8.028563859	8.483326677
C20orf94	8.222643394	7.966742939	8.246470545	8.191909586	8.12156602
C21orf104	8.27903296	9.437491071	7.937437293	7.841729333	8.510894717
C21orf109	7.115448364	6.519889687	5.788254818	6.043315566	6.655494515
C21orf33	9.751080646	10.14631732	10.5431016	10.27714677	10.35809493
C21orf57	7.307847101	8.317411434	7.30728296	7.62575301	7.723009
C21orf58	15.36066179	16.04217659	14.93426708	14.76275492	16.04217659
C21orf7	9.393934131	10.04963797	10.48185706	10.62895885	9.800904051
C21orf89	8.585836293	7.528303323	8.822853162	8.675789182	8.825312061
C21orf96	9.432003333	9.316319051	9.042346232	9.644739414	8.954176293
C22orf13	8.914720172	9.588410576	8.916479111	9.267923343	9.540446515
C22orf25	8.989142232	9.722667242	8.252794354	8.627322343	9.260607
C22orf40	8.045722828	8.468418253	8.924201657	8.322342051	8.386601707
C22orf9	9.121511687	8.829061111	8.475612051	9.225515485	9.11154599
C2orf24	8.725555596	8.77608297	8.279462	8.979298788	8.824381182
C2orf29	8.092691677	8.184050869	8.412956141	8.066374202	8.119679141
C2orf30	7.196550485	7.350388939	8.171788646	7.606370131	7.731871869
C2orf39	7.82475102	6.747571626	6.695720192	6.713056273	6.94985404

C2orf79	8.509502414	9.068490586	9.602761333	8.698751323	8.911109919
C3AR1	7.662925475	8.269818727	8.652374404	8.734416606	8.077645899
C3orf18	8.010919919	8.776574232	8.597027768	8.083135202	8.193428737
C3orf26	8.073652495	8.421973258	8.750392838	8.227704253	8.856343687
C3orf31	8.874692596	9.21352099	9.490338525	9.171801828	9.598265626
C3orf34	9.538964939	8.936128005	9.050797061	9.767642525	9.374121768
C3orf54	8.626355717	9.851320949	8.209054152	8.861258687	9.256085253
C4B	8.401710747	7.387654737	8.090488788	7.692217293	8.149448323
C4BPA	7.040523253	6.33399804	6.547344253	6.586655667	6.567684242
C4orf14	9.811898121	9.991229606	10.01340346	9.787586646	10.08521343
C4orf18	8.540202646	8.953164051	8.010523545	8.906407303	8.587234515
C4orf34	9.167758485	8.903025495	9.550769788	9.238865747	8.986932636
C4orf48	10.03983393	10.10841911	10.93334198	9.76471501	9.878686616
C5AR1	8.848410616	7.991889949	8.192996515	8.402589283	8.689422596
C5orf17	5.773313677	5.794171475	5.85496197	5.781597485	5.779230333
C5orf26	10.28406896	10.94786936	11.15503783	10.72384747	11.23641801
C5orf32	11.31053596	10.12799521	11.13713447	10.70775781	11.308834
C5orf39	9.742174424	10.21484784	10.5199714	10.080472	10.50166798
C5orf4	10.37894268	11.46320141	10.91766747	10.62142174	11.25898056
C5orf41	10.90466263	11.03296099	10.66105788	11.05356995	11.16734497
C5orf44	6.853461566	6.762066263	7.787525596	7.103541778	7.100167172
C5orf46	6.943066869	6.857999616	7.580904889	7.195224778	7.724673141
C6orf105	6.665174586	8.233006697	7.468171747	6.798354081	7.142620404
C6orf115	10.88430477	11.16579624	10.86305205	10.52135963	10.89755671
C6orf124	7.791244131	6.588238949	8.354182626	7.341966626	9.282486081
C6orf129	7.727011939	8.503920141	8.285708747	7.408375899	8.168587596
C6orf130	9.011546121	9.235179424	10.47584162	9.826032111	9.79615097
C6orf192	9.056062667	10.47123164	10.75178718	9.432636424	10.15483313
C6orf208	6.482726162	5.946739657	6.121104576	6.138065879	6.616628394
C6orf25	8.375668162	9.126663535	8.891888576	8.672881101	8.218887434
C6orf27	9.168324747	9.601568657	8.849981545	8.830660505	9.406893465
C6orf48	11.27499716	11.89361272	12.8652792	12.23769992	12.12436236
C6orf62	11.85301976	11.97447062	11.73710477	12.05813886	12.25251148
C7	6.637667434	6.258190556	6.304770596	6.948313626	6.050726626
C7orf11	8.614442374	8.659649677	9.310205828	8.722953263	8.781156657
C7orf16	6.338010192	6.913051818	6.984018879	6.367811737	6.236419566
C7orf23	8.346989566	8.337114525	9.485668061	8.539328929	8.653906556
C7orf25	6.461471737	6.265371141	7.149903152	6.626873192	6.475960141
C7orf29	7.963371182	8.399553939	8.451947071	8.000521869	8.108917222
C7orf40	8.663769939	9.036076414	9.611261414	9.09178804	9.024212859
C7orf42	8.45451298	8.514909313	9.057196687	8.524356212	8.554027364
C7orf43	9.258612929	11.62494396	8.509936919	9.448606737	10.02439619
C7orf47	12.71142619	12.32964397	12.5521346	12.50706636	12.16279806
C7orf53	10.08325108	9.433849505	9.015412101	9.332795131	8.865915939
C8orf15	6.486835303	6.877631798	5.762963323	6.611373919	6.788320081
C8orf4	7.469344313	6.708053838	6.774688626	7.262957343	7.428304182
C8orf44	7.593664717	7.566119051	8.454954939	7.692888131	7.710578727
C8orf55	7.909412687	8.005727263	7.677264869	7.797844434	8.097958939
C8orf60	10.33890802	9.570470929	9.716648152	9.907546465	9.652169778
C8orf73	8.867918475	8.64224801	8.550897414	8.784649081	8.715845505
C9	7.016197848	6.430485283	6.693279889	7.344882899	8.051625212
C9orf139	9.333361616	9.211184727	8.32400903	9.801802283	8.898231131
C9orf142	8.957944414	9.257979848	8.765233303	9.618955576	9.331038253
C9orf163	8.316969364	8.534897646	8.62412801	8.092218303	7.790604293
C9orf167	9.470840162	9.062220818	9.406893465	9.35371997	9.034403333
C9orf25	12.53911948	13.48990673	12.20640951	11.83813795	12.63500423
C9orf4	5.945299364	6.018143222	5.76081802	5.759448747	5.923636364
C9orf40	7.325469747	7.642608182	8.373514202	7.035420475	8.236217485

C9orf50	7.704008545	7.169717707	7.552833101	6.959189505	7.339214566
C9orf62	13.50997822	14.60195877	12.44611991	12.22629088	13.00101781
C9orf71	9.177504333	10.39296715	9.380979222	9.886023253	10.10542249
C9orf72	8.017104828	7.433916687	8.008400859	7.725703576	7.587956192
C9orf78	12.66627862	12.0089388	13.15645152	13.1944969	13.37822002
C9orf91	7.284105152	7.535866141	7.697940283	7.583998525	7.568924192
C9orf95	9.152615889	8.786134101	9.934615404	9.504584061	9.072403576
CA1	11.39772243	11.95261936	11.58621592	10.60161194	12.17216641
CA14	7.152687384	6.942817626	6.988399394	7.355706667	7.275508121
CA2	8.477856384	9.015412101	9.719000626	9.35728196	9.015412101
CA4	9.050797061	7.570765313	7.715405939	8.152443121	8.239938859
CAB39	9.271123182	8.816214848	9.846508495	9.20200202	9.189590182
CABC1	8.711304737	7.598885727	8.777050384	8.677215515	8.082337909
CABP5	11.27306561	12.09225922	10.7532413	10.10841911	11.12935449
CABP7	6.089123677	6.496571788	6.019482414	6.073352293	6.009435414
CACNA1B	7.738613697	7.797125465	7.502729737	7.07857001	7.367866364
CACNA1E	12.34237091	14.56207529	8.871209182	10.91356479	12.03068847
CACNA1H	7.119081899	6.575874879	6.857008404	7.145769434	7.243253313
CACNA1I	11.24816417	11.85033795	10.0178996	10.8947391	11.54675633
CACNA2D3	6.885618131	7.315524667	7.633233848	7.263859848	7.183932212
CACNA2D4	6.768970657	7.241538444	7.606990414	6.766521566	6.812925505
CACNB3	8.876650909	9.438094949	8.621272737	8.268510495	8.823889374
CACNG5	7.102414636	6.640921556	7.02750098	6.978602455	7.196550485
CACNG6	7.013396273	7.656877838	8.551311374	7.135537212	6.641378788
CACNG8	7.201570444	6.506758646	7.45523802	6.579152939	6.997949788
CAD	7.792587404	8.035675313	7.041369354	8.190781646	7.826840444
CADM3	9.066117364	9.769223525	8.907915899	8.843670869	8.81572204
CALCOCO1	8.113343889	7.627030141	6.982654747	7.743466333	7.283275424
CALHM2	8.511758747	8.513135323	8.647073404	8.950355889	8.556357949
CALML3	8.111336758	7.885002121	8.634342798	7.984288364	8.04259702
CALML4	9.089024899	8.951989212	9.969141677	9.412044242	10.02923217
CALR	10.37215733	11.06123261	10.67190595	10.55333109	10.58935103
CALY	6.244753081	6.126877838	6.688196	6.563203242	6.740481444
CAMK1	6.648620667	6.885887889	6.221783758	7.005077596	6.757659566
CAMK1D	9.339764051	9.413942061	9.030619364	9.646274414	9.044046343
CAMK2N2	9.037153444	10.17702971	9.150235333	7.360165061	7.980568657
CAMKK2	9.678297182	9.457741556	8.86024204	9.424273283	9.408808788
CAMP	11.6157426	10.0841781	11.28207759	11.93975968	10.39641485
CAP1	10.72900488	9.742174424	10.90466263	10.70052069	10.37215733
CAPG	12.18492446	12.14191765	12.39058663	11.98196729	11.82346178
CAPN2	11.84027237	12.46797904	12.797444	12.36759935	12.54906863
CAPN6	5.819048768	5.75088698	5.757505747	6.355365798	6.709797242
CAPNS1	10.21583282	10.36134852	9.197870838	10.60743282	10.24727703
CAPZA1	10.68687892	9.953782444	9.653591273	10.23351878	10.48074239
CAPZA2	9.243679384	8.874191697	8.322342051	9.087865687	9.128533354
CARD11	7.756889051	7.910975505	7.504269545	8.558556152	8.158968566
CARD17	10.57885616	9.989520798	10.35917202	10.21583282	10.35385551
CARD6	10.52135963	10.03876748	9.960976253	9.566949	9.763138212
CARD8	9.246644323	8.351752182	8.781156657	8.988033424	8.209054152
CARD9	9.745446081	9.513164303	9.638309758	9.466304111	9.609756293
CARHSP1	10.0178996	10.44094498	9.324388263	10.22267877	9.794631646
CASKIN1	11.35080354	11.86401838	10.54565144	10.9088749	11.109559
CASP4	14.5413574	13.85817003	14.32804886	13.99495772	14.010587
CASP5	11.2608846	10.6362571	12.27421104	10.687913	11.00496707
CASP7	8.310875455	8.778623323	8.650965909	8.468418253	8.668536687
CASP8	10.00985275	11.15966862	10.20060626	10.30810853	10.28190294
CASS4	7.227602475	7.666604434	7.391081455	6.966976535	7.809982667
CAT	10.64502212	10.49009312	11.28565422	10.77387872	11.04886125

CAV1	7.344596535	7.092334828	6.965717616	7.168103949	7.380377788
CBLN3	6.541858778	6.108576444	6.093769455	6.479217141	6.652192667
CBR1	7.06161799	7.308145939	7.821971636	7.223533071	7.518993111
CBS	8.019097616	7.175214949	7.149668323	7.357456071	7.666604434
CBWD2	8.431326525	8.548679899	9.404880263	8.824851909	8.825911939
CBWD5	7.957018646	8.144161414	8.598008343	8.156410909	8.246087697
CBX6	7.665987182	7.372238061	7.295703091	7.74578199	7.325469747
CCAR1	8.600831152	8.814624313	8.668062838	8.733083212	8.595283071
CCBL2	8.308331848	7.554702939	8.222223545	8.434806414	8.181196919
CCDC102A	7.098999354	7.210929778	6.87215099	7.532150707	7.225001677
CCDC104	8.668931657	9.124449354	9.469429253	9.249868985	9.804809051
CCDC107	8.078389566	8.030983081	7.942803596	8.149032212	7.957018646
CCDC109B	8.248566778	8.788663253	9.835081424	8.728449242	9.186096828
CCDC114	6.420140667	6.305549929	6.550314424	6.767752323	7.229904545
CCDC115	8.542859455	8.820759798	9.702758343	8.733877859	8.652374404
CCDC120	7.591410606	8.422643323	7.732919727	8.080354404	7.846688253
CCDC124	8.934248131	9.262561	8.125818909	9.144457596	9.352487919
CCDC125	9.273022949	8.872352707	8.199709495	8.64224801	8.759757687
CCDC13	5.957462212	5.769292242	6.268423737	5.814814606	5.912062667
CCDC137	8.937936253	8.837022465	9.07526898	9.584803364	9.501306556
CCDC14	8.314398727	8.670884566	8.934248131	8.643157737	8.841575667
CCDC144B	6.23359303	6.800553495	7.181699919	6.767482869	6.448364717
CCDC146	7.884680111	7.811684818	8.544134919	8.141770949	7.90331499
CCDC149	9.600888859	6.824112909	6.92669298	10.60507136	7.029601354
CCDC153	8.088528121	7.58864003	8.211847566	8.057421596	7.896955242
CCDC19	7.892604899	8.000521869	7.837132667	7.501110505	7.349181162
CCDC22	8.378142071	8.588971687	7.919797354	9.234418071	8.885588879
CCDC23	9.923012889	9.751885293	11.04886125	9.777520899	11.52052132
CCDC3	6.264838313	6.541370778	5.759634101	6.647920455	6.050896313
CCDC5	8.233421293	8.648914657	9.383480283	8.405355091	8.59942799
CCDC56	9.204855576	9.820433545	10.66342196	9.670530667	9.667708556
CCDC58	6.395573626	6.16228202	6.082806303	6.121897343	6.225547869
CCDC61	8.419470808	9.422377343	7.475616394	7.988882495	8.671783687
CCDC64B	8.578393657	7.817975838	7.038917566	6.420787636	7.384295283
CCDC8	6.629005263	6.320079859	7.120482	6.690165707	6.987890152
CCDC84	9.713188212	9.911926424	10.70299608	9.502644929	9.840616414
CCDC85B	8.539776737	9.112697707	9.139095455	9.004940778	8.881310737
CCDC88A	8.120062485	8.745474636	8.782655051	8.465492313	8.594016788
CCDC88B	11.64244603	11.26990549	10.71503879	11.52052132	11.39594259
CCDC92	7.95873603	8.563941778	8.107705111	8.513601889	7.845601525
CCHCR1	8.160644788	8.050105303	7.885002121	8.078044465	8.379510323
CCIN	7.599486586	6.460516394	6.796831899	7.464044657	7.131863879
CCL21	6.86986903	6.471263556	6.673038182	6.854191545	7.005354859
CCL23	5.875586646	6.295493455	6.258944949	5.83591897	5.825744636
CCL24	10.31095185	11.61117883	10.55842197	10.47479655	10.71992703
CCL3	7.873377879	9.968215758	8.055916879	10.32531651	7.468494556
CCL3L3	6.653596384	8.394903626	6.190240242	8.736738475	6.223332818
CCL4	9.798623747	10.25627793	10.31095185	10.52247938	9.442729677
CCL5	11.0387546	11.7894918	9.988725727	11.85301976	11.45002291
CCM2	11.73710477	11.37670858	10.14044783	12.03912361	11.22119175
CCND3	9.382882596	8.677215515	8.66101001	9.580097263	8.818210192
CCNDBP1	13.60269863	13.22156044	13.57574016	13.00101781	13.39659876
CCNG2	8.131433848	7.865908465	8.513601889	7.968277051	7.608041616
CCNJ	7.065642788	7.379773919	6.939713242	6.959689121	7.126519737
CCNJL	8.668062838	7.822730929	7.823737283	8.021628354	8.004282899
CCNY	7.832217929	7.41959598	7.97284099	7.535866141	7.188870657
CCPG1	9.771430818	9.237664879	9.432636424	9.259301556	9.243679384
CCR1	9.531623293	8.954798808	8.965143333	9.067856838	8.474658303

CCR10	7.865549818	8.696280707	7.243557061	7.783465061	8.098747556
CCR2	8.269395232	8.468898455	8.972363838	8.423139949	8.26142704
CCR3	8.375190152	7.542783869	8.682574626	9.088505869	7.92597801
CCR5	9.058356636	9.372025394	9.678297182	8.773471869	9.698896848
CCR6	6.768428949	6.771643182	6.158655535	6.741723879	7.11962903
CCR7	12.14789109	12.47441306	13.58996905	12.83827393	13.30255942
CCT3	10.11150219	10.43655428	10.48897537	11.109559	10.61480751
CD14	11.3403052	11.2345748	10.66706732	11.22294817	10.79956984
CD151	6.934796758	7.250899535	6.574452879	7.386173242	6.519436343
CD160	6.221954697	6.964119424	7.232737253	7.332963121	6.941467394
CD163	10.22267877	10.25038968	10.66587479	10.27905981	10.30569114
CD177	6.457381818	6.181117949	6.084661768	6.499478283	6.662952677
CD19	10.17030341	10.01257663	10.78987961	10.06450841	10.34100163
CD1C	7.053026414	7.381027636	7.870356606	7.560118414	7.500167182
CD1D	7.412474121	7.198597879	7.995673758	7.437630343	7.049507061
CD2	12.10414281	12.74093701	12.78185519	12.55983323	12.86086109
CD22	6.618269616	6.746600626	6.997949788	6.829579545	6.850381687
CD24	7.613240606	7.479422939	8.08193004	8.174069929	7.666980273
CD244	9.265583747	9.562364657	9.844817354	9.830354566	9.730279475
CD247	9.785178838	9.839111	10.00361533	10.63752253	10.31894523
CD248	7.482291222	7.848096879	7.981345788	7.469344313	7.307022919
CD27	11.2518391	11.55106122	11.41415047	11.17910752	11.46539851
CD274	6.253125879	6.346259394	6.735177838	6.095658889	6.766521566
CD28	8.342368121	8.995584808	9.082448343	8.607126586	8.87289497
CD300A	12.17216641	10.88430477	11.10460602	11.66934136	11.39185194
CD300E	6.841133828	7.183662697	7.13441502	6.723255293	6.739283293
CD300LB	8.744562374	8.021984222	6.994769535	8.342368121	8.242002535
CD302	9.593075545	9.147296667	9.382205394	9.629101929	9.255548899
CD320	8.105629576	8.154705535	7.835124576	8.382194192	8.527598192
CD36	8.587234515	9.484840202	9.439397061	9.45706402	9.528804131
CD37	9.724047525	9.731806091	9.488337717	9.815893677	9.882779162
CD3D	8.808928141	9.727935707	10.32750324	9.946747141	10.25422487
CD3G	9.451851384	10.23752804	11.0035084	9.977292899	10.83930015
CD4	13.05987966	14.83117585	12.06397065	12.43279956	13.2158469
CD47	8.498503737	8.438325768	8.758871	8.756396081	8.600831152
CD5	7.894965162	8.01423197	7.736260545	7.964547263	8.115821848
CD52	13.30795266	13.94443	12.62449502	14.28325373	14.05136004
CD55	8.389653889	7.991026727	8.446184828	8.295436051	8.300103657
CD6	10.40701089	11.07013707	10.18291229	10.39968902	10.85100254
CD68	9.768390455	9.788274313	9.684048687	9.948421707	9.548618051
CD69	7.49864798	7.831503424	8.320817869	8.063898495	7.952386505
CD70	6.863661697	7.031184939	7.310197869	7.013396273	7.048124798
CD72	6.831619364	6.800063838	7.053327354	6.729463253	6.629250202
CD74	9.251353071	9.679062616	8.803482152	9.266794162	9.065009323
CD79A	14.64328928	15.54332231	14.17411554	13.73439002	15.06622261
CD79B	7.534556657	7.922697606	7.394370293	7.944058707	7.835788293
CD81	9.005975162	9.142159707	8.768300525	9.881912677	9.917043434
CD82	11.14385742	10.60863639	9.862946677	10.82664672	10.44832821
CD83	7.191807606	7.634532263	8.097222414	7.704350384	7.88753598
CD86	7.525850576	7.795765162	7.36510997	7.790916071	7.669657737
CD8A	9.528176495	10.02439619	10.97767761	9.66831603	10.39072525
CD8B	8.090886384	8.555429434	8.403471909	8.656360697	8.323598384
CD9	8.135177152	8.811517808	9.005975162	8.783158111	7.977952253
CD93	9.759931222	8.851018515	8.724556485	8.984231152	8.829061111
CD96	7.560401535	7.815532475	8.346989566	7.795765162	8.011643808
CD97	13.33661988	12.66627862	12.48124823	13.26276274	12.51424883
CD99	9.779738	10.38001468	9.696433626	10.35178281	9.888393747
CDA	10.59418919	9.707282667	9.162016475	10.54692936	9.781348374

CDC16	8.499836475	9.198436465	9.884409465	9.315088131	9.435722162
CDC20	8.085049192	6.883278424	8.369612525	8.29291099	8.515864333
CDC25B	9.446464909	9.132651727	9.586238192	9.633493677	9.534194535
CDC26	10.25038968	10.17789322	11.20660223	10.35809493	10.40592026
CDC27	7.674109172	7.651222556	7.414093131	7.666604434	7.87816304
CDC34	12.16942168	13.15645152	11.85033795	12.71520416	13.44126502
CDC37	9.766131535	9.579413505	8.737817051	10.26224218	9.901640636
CDC42EP1	7.679634566	8.390114414	8.195405505	7.861189172	7.721580051
CDC42EP2	10.33696433	8.969483	9.951175646	9.763138212	10.0060683
CDC42EP3	10.56306707	9.919491525	8.962104076	9.513164303	9.631182697
CDC42EP5	17.12376032	17.29704085	15.24813401	16.25437967	16.52993947
CDC42SE1	10.28005063	9.76546198	8.687053253	9.70798304	9.34289298
CDC45L	7.627670859	7.192412434	7.442908828	6.944866586	6.789755162
CDC7	7.805761556	7.358075697	8.122747707	7.261824374	8.493155899
CDC73	7.662615848	7.272914455	7.712540141	7.140269465	7.552094283
CDCA7L	7.716120232	7.892604899	8.459227657	7.825767899	7.872718384
CDH22	8.300888657	9.465659495	7.615901626	7.982442283	8.351407394
CDH24	9.625623061	10.29542336	8.440058758	9.210065	9.526870273
CDIPT	8.994589434	9.25368898	8.419866586	9.212980727	8.984231152
CDK2AP2	8.139691131	8.189895404	7.66930299	8.35372597	8.129067798
CDK5R2	7.933221424	8.741130788	7.617533182	7.548285091	8.204737434
CDKL1	7.02612702	7.237206848	7.503972212	7.199149121	7.56320098
CDKL5	9.691258919	9.168828475	8.542429747	9.410728505	9.130979939
CDKN1A	8.783584889	9.123926859	8.197369283	8.981975535	8.863920758
CDKN1B	10.03492192	9.825199323	8.997224192	10.08799683	9.830354566
CDKN1C	9.974493182	10.53247367	10.79564384	11.20163629	10.48897537
CDKN2D	14.63205333	14.28325373	15.34617167	14.42673578	14.67552064
CDR2L	7.690840778	7.605113071	7.527339687	7.734550869	7.248166182
CDS2	8.668536687	8.025051697	8.63794701	8.124599212	8.249350414
CDSN	8.030983081	7.437630343	7.170232747	7.541384646	7.899333596
CEACAM19	8.589388657	8.864430475	8.385303111	8.373514202	8.533908051
CEACAM3	8.109636222	7.323124384	7.149049263	7.830100949	7.558170465
CEACAM4	12.12692782	10.6820228	11.17571904	10.79686873	10.79564384
CEACAM6	6.286506818	5.948262556	6.178987475	6.522760475	6.10953001
CEACAM8	7.487351192	6.482522545	7.415309101	8.548679899	6.639726192
CEBPA	12.65104366	12.24933389	11.14707085	12.8293171	12.06397065
CEBPB	14.3093669	13.32669092	14.1282639	13.56234785	13.10985871
CEBPD	14.1830221	13.03957854	13.61616406	13.44849295	13.09977851
CEBPE	9.217165182	8.613130333	8.872352707	8.665165081	8.333227545
CEBPZ	8.380828141	8.686126354	8.812013889	8.547449101	8.859179455
CECR2	9.959124273	8.804952828	9.928455091	9.751885293	9.623794152
CECR6	8.550414556	7.54886499	7.724049626	7.11316801	8.402589283
CECR7	9.728754818	8.692466525	9.220765354	9.324388263	8.975838535
CEMP1	12.11562205	11.55524693	11.88649013	11.97447062	11.90384839
CEND1	7.863195747	8.709464434	7.561342071	7.662615848	7.960941889
CENPK	6.184352576	6.948610667	6.174156556	6.773240303	7.337386909
CEP63	9.681893121	8.794361798	9.853021111	9.588958727	9.397864101
CERK	7.496050374	7.48289603	7.401155333	7.251769333	8.073294879
CES1	7.373377929	7.268778051	7.376443525	8.007258162	7.506099556
CFD	13.1944969	12.93862501	13.73439002	15.05177986	12.37416009
CFDP1	8.813595202	8.622644364	9.900778232	9.412634061	9.653591273
CFH	6.489542818	6.532834	6.710494808	6.401394333	6.723002525
CFL1	9.831087364	9.515069697	8.775519707	9.862946677	9.671170444
CFLAR	9.799317404	9.238865747	9.821195081	9.753529758	9.261851101
CFP	14.4185289	13.5964752	13.92172837	13.96779242	13.47832004
CG012	7.579628838	7.09353204	7.02200603	7.335553323	7.317897131
CHAC1	8.798818646	8.539776737	8.648450303	8.732044667	8.651919778
CHADL	7.457359414	7.696259101	8.467667768	7.663954061	7.576363505

CHCHD1	9.762542495	9.629839465	10.45041513	10.18468509	10.09506496
CHCHD10	8.517695303	9.094691273	8.034450929	8.773988747	8.782655051
CHCHD4	7.891912283	8.10683704	8.647922808	8.159434576	8.228066545
CHCHD8	12.31719428	14.4427031	10.60863639	11.62494396	12.52130815
CHERP	8.71185199	8.498949848	8.587234515	8.088117667	8.045324071
CHGA	7.52530399	7.597541737	7.567690545	7.258984768	7.301506111
CHI3L1	8.908475283	9.138451657	7.928832091	8.724556485	8.424058939
CHI3L2	6.130281505	6.400112778	6.850381687	6.821271707	6.537953232
CHIC2	8.532980343	8.059832677	6.94531798	8.542429747	8.522641273
CHKB	10.29752835	9.723272263	9.780583495	10.84176532	9.983540212
CHMP1B	9.629839465	9.120933788	9.93099102	9.408808788	9.037616626
CHMP4B	9.574750414	9.359207242	9.276189121	9.856174394	9.972781707
CHMP6	9.203189636	8.271029889	8.882379495	8.31912796	9.354289121
CHODL	6.500408909	6.185179869	7.031727121	6.635819404	6.732525747
CHORDC1	8.003903253	8.118879606	8.378142071	8.013081051	8.243637242
CHPT1	9.532335293	10.35069541	10.31597323	10.17613521	10.47242114
CHRD	7.204610747	6.875689354	7.169190859	6.905316545	7.119081899
CHRM1	6.559504222	6.733937384	7.011036525	6.787827455	7.157499455
CHRNA4	7.686444293	8.442699879	7.57799899	8.078389566	7.919459465
CHRNA7	7.381950828	7.066192545	6.872611121	7.028039657	6.751169162
CHRNB2	7.425828556	7.309928404	6.548235576	6.679714263	7.007603253
CHST11	8.307893798	7.789573343	8.887684293	7.983913121	7.892604899
CHST12	9.463745455	9.687758929	10.18762619	10.4322159	10.34193651
CHST13	10.61371787	11.01376888	9.232683343	10.33989796	10.14809211
CHST2	8.233006697	7.895961929	7.984708455	8.908935182	8.143776848
CHST5	6.557897323	6.593634414	6.001600899	6.126072101	6.976769172
CHST7	8.891888576	8.446184828	8.689422596	8.552719747	8.594861495
CHSY1	11.6137143	11.14866374	11.09721747	10.6362571	11.11230451
CHUK	7.039183869	6.773917293	7.282065253	6.800063838	6.685804808
CIC	9.212349869	8.913024061	7.792215626	8.840590677	8.581169111
CIP29	9.568406747	9.948421707	10.82153564	9.712341424	10.1905069
CIR	11.74205097	11.28565422	11.95261936	11.49636188	11.65261158
CISD2	8.503061444	9.412044242	9.629101929	8.205133263	9.853774707
CIZ1	8.878201818	9.029402949	8.496365737	8.822853162	9.085558838
CKAP4	9.354902374	8.833963152	8.042974687	8.819236758	9.103811545
CKB	10.38430498	11.17750487	9.415217505	10.34626898	10.51174846
CLC	10.41426339	10.9660467	11.64637724	10.75987116	10.6240143
CLCA3	7.877844556	7.74007802	8.015388182	8.115023838	8.174468182
CLCN1	6.971315	6.620737576	6.809486374	7.307562737	7.410002717
CLCN7	7.842052606	8.037287293	7.051121485	7.774283859	7.239990273
CLCNKA	9.076382242	8.431738505	8.877626323	8.185177828	8.256499515
CLCNKB	6.163811747	6.131379717	6.544328465	6.245450939	6.219828162
CLDN15	7.549206232	7.105225354	6.873999848	7.565085768	7.547650101
CLDN3	7.828260424	8.03601997	7.553068374	7.346859747	7.686775222
CLDN5	8.336257222	9.260607	9.650129091	9.171164091	8.553595747
CLDN9	7.017237545	6.766293242	6.903241343	7.114036293	6.819189677
CLEC10A	7.449616394	7.912365798	7.901917293	7.065949717	7.08018898
CLEC12A	9.569854202	7.020975202	9.592426051	9.07419996	9.06557301
CLEC12B	9.542425758	7.662615848	8.502042192	8.810986707	8.592622071
CLEC1B	8.068564879	8.119679141	9.689129545	9.206631485	7.841729333
CLEC2B	11.0473733	10.1597097	10.78014639	11.00183361	11.01683173
CLEC3B	8.685647889	8.232591606	8.835019636	8.730448515	8.35372597
CLEC4A	10.92608881	10.84302834	11.20840189	11.18471248	10.83790729
CLEC4D	11.5571076	8.936938576	10.18666124	9.393934131	9.390110384
CLEC4E	13.09977851	11.34381562	13.56886427	12.68903729	12.04734694
CLEC4G	6.593634414	7.848409778	6.889250929	7.279114576	6.804729515
CLEC7A	7.943134	7.629344343	6.862647808	7.493863788	7.369773828
CLECL1	6.231781505	7.142620404	6.416313212	6.737098263	6.190072818

CLIC3	7.334066545	7.459405444	6.697417434	7.736619182	6.652395162
CLK1	10.04600105	9.503858424	10.49348589	9.686378667	9.509226798
CLNS1A	10.21484784	10.88809182	11.41605585	10.73264046	10.69574272
CLPS	8.387939343	8.317824071	7.703321374	7.730873535	8.022720303
CLSTN1	7.766357737	7.918848717	8.50673198	8.760676242	8.892970697
CLU	10.44094498	11.18800276	10.03299879	11.18096106	10.09597734
CLUAP1	7.258984768	7.640655485	7.807908545	7.299385535	7.966742939
CMAS	7.061330929	7.516462556	7.580266434	7.560401535	7.508036465
CMBL	6.690572232	5.997671162	6.845301404	6.009435414	6.241029384
CMC1	9.372025394	9.277992232	9.948421707	9.605576293	9.933588354
CMIP	10.03026493	10.98186414	7.796747616	8.894491141	9.52752797
CMPK1	8.642670768	9.526870273	8.76626204	9.50650501	9.332175414
CMTM2	12.34577038	10.87092264	12.34855768	12.00354228	11.47127063
CMTM5	8.944409727	9.37540303	9.430738808	9.177504333	8.344441616
CMTM8	7.334908263	7.920877394	6.630444394	7.042386798	6.842028788
CNFN	9.741357455	10.55474917	10.11246778	9.793942798	9.910074283
CNIH	9.444645091	9.655770101	9.103811545	9.487680697	9.660861778
CNIH2	7.14635899	7.780116303	6.627836202	6.816827283	7.377888556
CNIH4	10.69354222	10.05780386	10.63014735	10.15379546	10.27236099
CNN1	6.637469778	6.649897606	6.674983667	6.403910828	6.090583616
CNN2	10.84302834	10.1250404	9.086722374	10.67190595	9.430738808
CNNM3	8.762742172	8.523456192	7.766357737	8.710842768	8.233421293
CNNM4	10.07388225	9.94767803	10.13580598	9.589694828	9.625623061
CNO	7.41439101	7.553773697	7.900388485	7.169190859	7.803554182
CNOT7	8.900236364	8.877626323	9.321347081	9.03221704	9.053043889
CNPY4	7.143149899	7.224435778	8.028141747	7.295094879	7.271701444
CNTNAP1	8.529417101	8.705145061	7.507667889	8.226840717	8.91530399
CNTNAP3	6.313338182	7.502729737	8.106019798	7.276013455	6.832363455
CNTROB	8.153159131	8.162329636	7.778362485	8.043818737	7.962380919
COBRA1	9.513795939	9.250770152	8.619347838	9.269847242	9.204855576
COL18A1	11.20345844	9.977292899	9.411394242	10.24511785	9.578067192
COL8A2	6.349206475	7.074987333	5.764100606	6.098211364	6.389460141
COL9A3	10.2187154	8.574674323	9.794631646	9.400967465	8.32400903
COMMD10	7.894315778	8.264240727	8.528915121	8.311348051	8.357077121
COPG	7.352546465	7.264798293	6.157664758	7.211210626	6.767482869
COPS3	10.82034332	11.31267384	11.51647662	10.86691694	11.44491584
CORO1A	13.5964752	12.62449502	11.12613667	13.47327752	12.77841715
CORO1B	9.096947232	8.901935111	7.381950828	9.193130899	8.664254273
CORO1C	7.842683778	7.796085586	7.529315566	8.175968162	7.900715717
CORO7	10.4524716	10.36032821	9.645545465	10.48185706	10.32750324
CORT	9.945028121	10.03492192	9.876880455	9.338458455	9.942152202
COX1	12.3543152	12.76207554	13.09045797	12.66167346	12.66167346
COX4NB	9.858738737	9.79615097	10.12909315	9.629839465	9.753529758
COX6A2	15.67809237	16.3077943	13.76108056	15.29549959	15.52328152
COX6B2	7.459710222	6.750902172	7.21064097	7.604109192	7.97830402
COX6C	9.991229606	10.70643744	9.905108616	10.52364746	10.72900488
CPA3	6.793900848	6.698319919	7.460903263	6.794399263	6.299210717
CPAMD8	8.462236515	8.725113212	8.695335444	8.53537201	7.744142424
CPB1	6.531281859	6.486601465	6.073896293	6.568383192	7.189144101
CPD	9.09824599	8.631321253	8.213427111	8.757405333	8.611286687
CPEB4	8.833337414	8.673843717	8.573273283	8.562227051	8.597027768
CPLX2	8.633848111	9.521192909	8.099112939	7.985767212	8.957404525
CPN1	6.51696904	5.830121162	6.397600616	6.45826596	6.585712788
CPNE1	8.213832515	7.784888444	7.555972677	8.343220798	7.685352364
CPNE5	10.83223099	11.05651949	11.83072395	11.91912022	11.08435313
CPNE6	7.25402203	6.647679424	7.106592788	7.03599896	7.164403717
CPSF1	7.874063444	7.972411364	6.971045939	8.023866626	7.641985
CPT1B	8.923576949	8.471488636	8.376704939	8.556357949	8.950355889

CPVL	9.008373061	9.627013505	10.77762269	10.00262223	9.462343535
CPXM1	7.579015667	6.590449828	7.717096556	7.760648222	8.037984616
CR1	7.603719626	6.779180051	6.957636657	6.92164503	6.858444758
CRABP1	8.116928465	8.197004869	7.784888444	7.535530424	7.845981061
CRAMP1L	8.403930677	7.996393263	8.439112919	7.860050667	7.904381697
CRB3	6.867411697	6.593389535	6.703725465	6.442315303	6.817996071
CRCT1	7.85604701	7.102116364	7.755132808	7.245884828	7.653651778
CREB3L1	11.64637724	12.51066587	10.60383217	10.29101087	11.08936677
CREB5	11.02851208	10.21583282	10.27338352	10.07670007	10.19528987
CREBBP	9.479084687	8.961854929	8.820759798	8.617840212	8.474228909
CREBL2	7.229324455	7.61856696	8.486968232	7.670902747	7.970574879
CREG1	10.1885747	10.1838089	11.01209098	9.597513697	10.70052069
CRELD1	8.996667384	8.531247657	8.169382444	9.271123182	8.577417788
CRELD2	10.26332497	11.35657003	10.29851595	9.960976253	10.1250404
CRHR1	9.453123465	9.214694788	9.170513566	9.800904051	9.644739414
CRHR2	7.940978939	7.414093131	7.246435697	6.807473404	7.583321859
CRIP1	9.821914929	11.0473733	10.79956984	10.46108665	11.04030995
CRIPAK	8.510413414	8.040196323	8.560860505	9.25368898	9.150811232
CRK	7.68296396	7.127674667	7.957660747	7.68296396	7.262654505
CRLF3	10.61480751	10.25220538	10.34514895	10.4450719	10.86173253
CROCCL1	10.27035429	10.19332507	10.26541026	9.925226333	10.12909315
CRTAM	6.850608899	7.481960152	7.74646602	7.358963616	7.590558081
CRYBA4	6.638813101	7.018669091	6.69943696	6.677794545	6.857241172
CRYBB2	6.469635131	6.405058101	6.253499364	6.389261576	6.496794495
CRYL1	8.829514889	9.573320202	9.819688798	8.878201818	8.559011545
CSAD	7.987912222	7.36694602	7.389558374	7.918848717	7.565422434
CSDA	11.82346178	12.69676846	12.61761735	12.55625498	12.70390423
CSF1R	13.78027417	13.66158872	13.46712151	13.62784329	13.0147344
CSF2RA	9.502644929	8.20265299	8.910531141	9.316888828	8.387083515
CSF2RB	8.442699879	8.019769071	8.328992859	7.665987182	7.457632404
CSF3R	12.57486869	11.69658017	10.13768673	12.052647	11.49420288
CSGALNACT1	7.809982667	7.723384495	7.844877657	7.882189879	7.79851096
CSGALNACT2	7.859670455	7.399710364	8.464558212	7.53302901	7.453291162
CSK	9.46096901	8.791137414	8.41552702	9.406893465	8.810986707
CSN1S1	7.098240939	6.471031303	6.151923424	6.819189677	6.794399263
CSNK1G2	12.35798465	12.62807191	11.06573849	11.95514832	12.12151457
CSNK2B	9.615324495	9.428190232	8.908935182	9.848227263	9.471411141
CSRP2BP	9.095162899	9.59460096	9.726443586	9.35858599	9.387059283
CST2	6.783830859	6.487932242	7.472500828	7.528640475	7.930919909
CST3	9.012031232	9.904237354	8.659649677	9.727055768	9.281923404
CST5	7.831138545	9.103235919	8.197805768	8.675348646	8.398231626
CST7	9.341818545	7.761378475	7.182546172	8.586731747	8.515350859
CSTA	12.00644476	11.34892675	11.28896545	11.40145091	11.19447199
CTAG1A	6.228606576	5.765796283	5.776721404	5.749786202	5.758240646
CTBS	10.45041513	10.00886723	9.980047091	9.935661677	9.971733293
CTDSP2	11.34202326	10.79145221	11.86652814	11.47127063	11.22947614
CTDSPL	7.025021455	7.690204818	7.382821899	7.393489313	6.747331182
CTF8	6.779669273	6.425110121	6.140397172	6.714232707	6.506758646
CTNNAL1	8.920488949	9.856174394	10.29208493	9.823678374	10.68960982
CTPS	7.580904889	7.783847071	7.643628758	7.729367111	7.695265798
CTRB2	10.88809182	12.82558839	10.40074116	10.42062148	11.20660223
CTSA	10.64005918	10.8947391	10.46766096	10.90192126	10.08799683
CTSB	10.97896732	10.97476091	10.60635433	10.68960982	10.39521192
CTSD	8.374363061	7.949400121	7.039421667	8.369167818	8.18280501
CTSE	6.401214677	6.784609556	6.744072859	6.384764838	6.730943374
CTSF	8.466026899	9.524763869	9.285000091	9.23383296	9.377908
CTSG	8.228946717	8.093077828	8.960218374	8.345781111	8.505797091
CTSH	10.80239406	11.40306525	11.34732253	11.41778198	11.06261544

CTSL1	8.444861909	8.110280409	9.691258919	8.986932636	8.300483646
CTSS	10.68960982	10.23351878	12.16942168	12.00086001	11.38025327
CTSW	7.187484444	7.000584848	6.599831253	7.658441616	7.723687384
CTSZ	7.627030141	7.427701768	6.346864081	6.759203	7.076844636
CTXN1	7.367866364	7.189410667	7.372532788	6.916684768	7.130781323
CUTC	8.616405606	8.198946162	9.478383576	8.519692939	8.291610121
CWC22	7.430512737	7.286993737	7.805446323	7.631600293	7.568310444
CX3CR1	12.32964397	12.25251148	12.43600068	12.78185519	12.60396528
CXCL1	11.52052132	10.85923156	10.92190902	9.880317061	10.31095185
CXCL3	7.261194687	7.324608717	6.988144556	7.01811702	6.779669273
CXCL5	7.791885697	8.301264212	9.46096901	9.423026242	7.922697606
CXCR3	9.605576293	10.27236099	10.04511943	9.704210374	10.2166926
CXCR6	6.35000001	6.004633889	6.839911636	6.402020111	6.583167424
CXCR7	7.223533071	7.589604303	7.459710222	7.369773828	7.231529313
CXXC1	8.491049758	8.395713444	7.404680798	8.871209182	8.379884889
CXXC5	8.478760515	8.462236515	8.47107903	8.82842597	8.410908071
CXorf18	6.615162343	6.264245596	6.743803525	6.850381687	6.315805677
CXorf21	7.988882495	7.627670859	8.798818646	8.354947869	8.323157747
CXorf48	7.163863636	6.603596232	6.203676525	6.440187172	6.772514131
CYB561D1	10.93171462	13.07454735	9.747577162	10.27139209	11.15183093
CYB5A	8.369612525	8.620790505	9.558925838	8.602859283	9.562994313
CYB5B	7.330801667	7.317304515	7.568036061	7.294038515	7.216692263
CYB5R2	6.30015496	6.339864232	7.404051424	6.049725535	6.126232838
CYB5R3	12.18142186	12.6076243	11.54675633	11.79171062	12.13538265
CYBASC3	8.861728919	9.012760899	8.492322828	9.194378495	9.10215096
CYBB	8.918756414	9.484209455	9.34289298	9.381544081	9.441234879
CYBRD1	8.641223283	7.959830364	8.131862263	8.175629323	7.953486636
CYFIP1	6.824112909	7.186028475	7.715785354	6.812413525	7.022261949
CYP11B1	8.796283929	9.277455131	8.458294828	7.912365798	8.492747404
CYP1B1	9.951175646	10.33476777	10.71279176	11.12151875	10.25127201
CYP21A2	7.034613909	6.591366	7.079662576	6.678083162	7.065642788
CYP26B1	5.771822061	5.982672192	5.861477071	6.31869002	6.446834051
CYP27A1	7.608041616	7.673376626	7.055367566	7.591751808	7.032840081
CYP2B6	8.209801687	7.437044505	8.326162172	8.540635232	8.903602263
CYP2D6	7.838908253	7.117115202	7.088518818	6.997949788	7.134684455
CYP2F1	7.235098212	7.078835444	7.320252869	7.504860717	7.741457929
CYP2W1	7.536554758	9.11154599	7.23119303	6.893115293	7.714317071
CYP3A43	7.646275747	7.666324061	7.189144101	6.660321343	7.470886646
CYP4F2	8.55725397	9.681142253	7.672313364	8.24438497	8.524867051
CYP4F3	10.05880999	9.90675497	9.426142444	9.804809051	9.372705202
CYP4F8	7.911629535	7.610260323	7.706911222	7.61856696	7.290204495
CYP51A1	6.559271747	6.995800606	6.59504903	6.566529242	7.127106434
CYTB	11.60266156	11.84529477	10.80389925	11.64244603	11.91912022
CYTH1	13.52959744	13.25686564	14.1830221	13.61616406	13.32021166
D2HGDH	9.746145949	9.209526556	9.221294354	9.083741606	9.687048303
D4S234E	6.417030697	6.440833869	6.556473354	6.246552232	5.920283455
DAAM2	7.871683444	6.653092081	6.004759152	6.648141899	6.440187172
DAB1	8.197369283	7.756175162	7.57049196	7.300330596	7.558798354
DAB2	8.132293172	8.668536687	8.606624646	8.335469222	7.879985051
DAGLB	7.545986354	7.223856	6.409677646	7.704713596	7.541750556
DAPK2	10.64623997	9.144457596	9.273640758	10.01257663	9.066605263
DAPP1	10.43655428	10.32946621	11.07158908	10.53141254	10.52247938
DAXX	8.746311293	8.307093606	8.543234566	9.196132455	8.778623323
DAZAP1	9.485668061	10.03123363	8.195856758	8.961854929	9.245445293
DAZAP2	14.39736901	13.68884286	13.7211242	13.98081649	13.80033164
DBF4B	7.596851556	7.014542111	7.724369343	6.981086404	7.272331859
DBNL	9.026636737	8.335469222	7.643944697	8.923097444	8.277405556
DBP	9.620873889	10.687913	8.864955909	9.170513566	9.966143818

DCLRE1A	7.36076402	7.261194687	7.557888444	7.402701172	7.150169929
DCP1B	8.77608297	9.366312899	9.69725304	8.805907646	9.460382818
DCST2	6.936097616	6.188194515	6.622230818	6.835608667	6.608273162
DCTN1	8.712915394	9.430143091	8.236217485	8.48742196	8.917682242
DDAH2	9.491039162	8.599954677	9.000672535	8.717867636	8.617420111
DDB1	8.682101313	9.225515485	9.180285111	9.065009323	9.503858424
DDEF1IT1	7.909763434	7.792215626	6.646173444	7.238770545	7.366006364
DDIT4	9.953782444	9.069558111	10.29324401	10.29660096	9.504584061
DDX11	7.416641556	7.909031556	7.336756222	7.923423949	8.086953081
DDX21	7.913459869	8.262596626	9.390834152	8.372772768	8.366003535
DDX26B	7.411561343	7.127947263	8.039451586	7.679285535	7.807908545
DDX27	11.06573849	10.49592664	11.45526933	10.32093584	10.61621963
DDX39	10.26445444	10.59809845	10.2692332	10.81540317	10.71390019
DDX3Y	7.995673758	7.818322879	5.776376717	8.035288182	5.851690192
DDX41	8.657372182	8.749910828	8.044980354	9.139095455	8.767368071
DDX43	6.663185626	7.269064929	5.812557061	6.929929141	6.510064202
DDX5	13.35359128	12.82153585	13.95167504	13.64789782	12.96662998
DDX51	7.875808798	8.552719747	7.747885899	8.584830636	8.507211606
DDX58	10.25811169	10.1885747	9.961780586	9.680349586	10.22448985
DDX6	8.125376242	7.500167182	6.442114232	8.087781242	7.203497747
DDX60	7.161889071	7.25118401	7.764931778	7.112898869	7.314025869
DDX60L	8.101780545	7.496977949	7.647631172	7.491357222	7.553068374
DEAF1	8.609952768	9.056533626	8.188779434	8.670884566	8.513135323
DEDD	7.41345197	7.157253707	6.603596232	7.222960121	7.138289616
DEF6	11.07956266	10.52476206	9.365016101	10.78269205	10.55966675
DEFA3	14.56207529	13.13522491	14.79546777	15.86132141	14.13521971
DEFA4	9.965319232	8.934777717	10.0188097	10.88166557	10.02644328
DEFB1	6.818961091	6.357923899	6.948313626	6.398234566	6.945089283
DEFB4	7.424927172	6.984268657	7.995268899	8.021984222	7.808676939
DEGS1	8.661508737	8.023866626	8.947896359	8.532119949	8.346585616
DENND4B	10.47242114	9.198999222	9.216574283	10.14131493	9.493894545
DENND5A	10.94640834	10.27432009	10.98186414	10.8193093	10.63266161
DEPDC5	7.68569196	7.705454838	8.226165	7.632861657	7.681488071
DERA	9.822898081	9.996420616	10.06904603	9.363307667	9.67665103
DET1	7.60736902	7.704008545	8.085771838	7.345267404	7.680240364
DGAT1	8.980444384	8.996148737	7.994228667	9.118629636	8.752460404
DGAT2	10.24139873	8.900236364	8.723452192	9.740584727	9.017695354
DGCR2	12.03068847	11.52238659	12.34577038	12.12436236	12.06098883
DGCR6L	8.30460302	8.720163182	7.996393263	8.772410586	8.705145061
DGKD	11.7894918	11.47322198	12.33576886	11.79615897	11.62059475
DGKZ	8.928157121	8.838951081	8.584413131	8.354570556	8.674281929
DHH	7.988582505	8.27018399	8.29635502	8.155735586	7.879296202
DHRS1	9.895856061	9.387656788	9.762542495	9.639623879	9.36748
DHRS12	9.598265626	9.517013525	10.05970796	8.918756414	9.146178758
DHRS13	8.707057788	7.85604701	8.238629646	8.359263212	8.139691131
DHRS7	11.09250749	10.2166926	11.37847359	10.98350754	10.87907816
DHRS9	8.682574626	8.977558859	8.50473503	9.086722374	8.614861051
DHRSX	9.220211768	8.589388657	7.862492343	8.690381253	8.563501616
DHX15	8.980943495	9.118059475	10.2600862	9.325601172	9.304577515
DHX30	8.643157737	8.968391182	7.914251515	8.838951081	8.79576798
DHX38	8.64752602	8.157542717	8.248566778	8.707515232	8.436545051
DHX58	10.0672481	10.14131493	11.04316622	9.657223606	10.156858
DICER1	10.18000397	9.790672212	9.190767364	9.894213939	10.09682592
DIO3OS	6.567684242	6.095072758	6.836530859	6.24729001	6.599129636
DIP2B	7.735932323	7.509707515	7.983548525	7.458795919	7.50339202
DIRAS1	6.917518081	6.328723111	6.315391253	6.814975071	6.785592192
DIRC1	9.439917707	8.957404525	10.01703805	9.02886299	10.41426339
DIRC2	8.236217485	7.891143768	8.40783399	7.831503424	8.179581869

DIS3L	7.997875909	8.345353354	8.967895455	8.430929525	8.761644081
DISP2	7.167545535	6.294120212	7.642932737	7.077096727	6.942544717
DKFZP434B0335	10.69931975	10.50057506	10.41123643	10.34311059	10.66229262
DKFZP586B0319	6.559042364	6.515215919	6.785592192	6.622442465	6.341475172
DKFZP586I1420	9.423636879	8.781156657	10.12697515	9.911926424	9.773700283
DKFZp313P036	9.108564253	8.550414556	8.253254586	8.509502414	8.731576192
DKFZp434F142	10.67190595	10.1309568	10.02362526	9.745446081	9.987077505
DKFZp566H0824	8.792768747	8.436995596	8.939630657	8.666601818	8.863375313
DKFZp667E0512	9.412044242	8.842639202	9.033394545	8.760291222	8.722452253
DKFZp761E198	8.256833364	7.512168657	6.850882899	7.933573889	7.450590374
DLEC1	7.479706475	7.803554182	7.664565879	6.983407384	7.461509737
DLGAP3	15.06622261	16.02065153	12.84197228	14.27397716	14.85646221
DMWD	9.524763869	10.48185706	9.028300162	9.074692404	9.579413505
DNAJA2	8.35667099	8.660166475	9.476588061	8.703733141	8.956909848
DNAJA3	7.994228667	8.285708747	8.561321323	8.613521394	8.276438626
DNAJA4	7.689572788	8.170129495	8.320357909	7.959830364	8.661956242
DNAJB2	10.53028115	11.07956266	10.63870636	10.58935103	11.32376318
DNAJB5	7.732206152	7.985767212	8.283707475	7.689234404	7.002421121
DNAJC15	8.066736566	8.188379657	8.522641273	8.246087697	7.475616394
DNAJC4	8.517288667	8.807484626	7.967524909	8.353342091	8.804952828
DNAJC5	9.293710889	8.721054424	8.281167657	9.279768394	9.012760899
DNAJC5G	6.94072599	6.307499242	6.091000384	6.742219182	6.843046434
DNAJC7	8.518658667	8.249350414	8.893968333	8.73262002	8.279462
DNAL4	10.4322159	9.876880455	9.895856061	9.219562515	10.50914282
DNASE1L1	8.41552702	8.014979636	8.611710212	8.809442889	8.478263182
DNASE2	7.879985051	7.252580253	7.005354859	7.18421398	7.089371313
DND1	9.054276	9.908305848	9.400327152	9.607593848	9.53690398
DNLZ	8.722953263	8.298004222	8.514431737	8.357077121	8.486543404
DNM1P35	17.36803328	17.58093558	15.74256723	17.07010739	16.90924948
DNM2	10.63391466	10.25519474	9.109760465	10.50914282	10.04160563
DNM3	8.453639616	8.118473111	8.610845737	9.043522909	8.293340606
DOCK3	7.068375576	7.98690202	6.718038374	6.698319919	7.089056525
DOCK9	6.346474485	6.637264	7.007335798	6.437651364	6.888155525
DOHH	7.378186273	7.783110646	7.319616859	7.970926879	8.103690354
DOK2	8.908935182	9.023071899	8.03179299	9.503858424	8.710842768
DOK3	11.2278827	10.58689262	10.6240143	10.48782122	10.57758511
DOK7	6.534413101	6.969423263	7.209485919	6.60386102	6.929631566
DPEP2	11.36610866	10.61621963	10.53247367	10.98749259	10.08978265
DPEP3	10.55966675	10.4429644	10.18468509	9.836777707	9.529469889
DPM2	10.43122177	10.91514001	9.897493586	10.50794857	11.32559397
DPP3	7.391952354	7.518430525	6.606197394	7.528303323	7.10439698
DPP7	10.17224075	10.58469265	9.500664495	10.60863639	10.71145754
DPPA5	6.652654	6.448364717	6.291508596	6.788535525	7.993428586
DPT	7.337670899	7.289331586	7.127106434	7.130479657	7.106302455
DRD3	7.166369152	6.131811687	7.200097273	6.99155401	6.481687929
DRG1	7.522869182	7.541384646	8.745043505	8.040196323	7.777311636
DSCR8	6.759203	6.478787505	7.816902374	6.312971121	7.478150263
DSE	8.708467242	9.169326323	7.993875677	8.831201768	8.773471869
DSN1	7.944406939	7.599486586	8.999492384	7.916025434	8.035288182
DSP	6.35978	6.509135818	6.253672202	5.810202495	6.063067495
DSTN	10.24727703	10.32750324	9.548618051	9.514496606	10.06633871
DTX3L	7.515816273	7.451812333	7.788172758	7.348914263	7.600248455
DUOXA2	7.256041222	7.304125475	7.034867141	7.247290424	7.068375576
DUS1L	8.724556485	9.158023	8.116577384	9.35188598	9.167114
DUS3L	7.347416111	7.736260545	6.727688212	7.568924192	7.562928222
DUSP1	11.70557621	10.69354222	12.28985985	11.32185019	11.14057221
DUSP13	8.271029889	6.618749384	7.036210545	7.03650198	7.433916687
DUSP15	11.02700786	12.71830686	9.62302801	10.36349344	11.07956266

DUSP16	7.086157636	6.999250869	6.953378071	6.681164444	6.903241343
DUSP2	7.924885333	7.575420636	8.153159131	7.987573434	7.936282586
DUSP26	5.972872515	5.749103394	6.156181131	6.16346097	5.752805141
DUSP4	7.868335495	8.475612051	7.738963899	8.193428737	8.477505576
DUSP6	12.10694646	12.57877126	11.92937262	12.29886409	12.09804166
DUX2	7.899682051	6.029734202	6.07129398	7.050890818	5.822777545
DUX3	6.677794545	5.773173455	6.326196631	6.243182121	5.866835152
DUX4	17.29704085	17.43906183	15.92931651	16.57174008	17.07010739
DVL2	6.988974929	6.814975071	6.117409697	7.177458596	6.859701798
DYNLL1	10.59924244	10.82804231	11.91912022	11.23641801	11.17378617
DYRK1B	9.223302313	8.628622212	8.308331848	8.43006898	9.276789111
DYSF	11.43005301	10.00985275	9.643287657	10.68338253	10.5199714
E2F2	9.171801828	9.714566535	9.598893818	9.37540303	9.620873889
E2F3	11.84291489	10.92880859	10.82034332	10.72633412	10.71031814
EAPP	11.39983314	11.43938222	11.16281786	11.02851208	10.84974643
EBAG9	7.258107788	7.30673204	8.415109929	7.490707535	7.775630727
EBF4	9.833438364	12.07810634	8.006495333	8.633848111	9.074692404
EBNA1BP2	9.786685869	10.49241362	10.80644997	10.2752886	10.44094498
EBP	9.261851101	9.319510202	10.09994749	9.89348604	9.815129384
EBPL	8.64224801	8.503455465	9.112697707	8.022369434	8.721926778
ECE1	8.946596606	7.854325061	7.568310444	8.580722586	8.105207222
ECHDC2	9.06333597	9.473962505	9.769223525	9.654354424	9.719000626
ECHDC3	8.272231939	7.079910848	7.648986848	6.999250869	7.207500556
ECOP	9.334658071	9.279768394	9.775265525	9.188424626	9.101086141
ECSIT	9.390110384	10.72127155	9.137887434	9.839882283	10.03611568
EDARADD	9.726443586	9.766921414	9.565660949	10.47370876	9.975431768
EDEM1	7.422224808	7.259665485	7.130177747	7.237838192	7.260583141
EDF1	10.8605042	11.2608846	11.35657003	11.50814936	11.41238356
EDIL3	6.488188354	5.751376051	7.120197273	6.889002121	7.322217273
EEF1A1	12.02811467	12.41033676	14.15092087	12.97555423	13.05987966
EEF1B2	12.08680505	12.50328361	12.96241124	12.57107442	12.68484185
EEF1G	13.23864174	13.44126502	13.69473258	13.70717091	13.62106704
EEF2	11.65918316	11.82616001	9.877792838	12.11562205	10.98492733
EFCAB2	6.934568707	7.402701172	7.194433949	6.871904646	7.118512586
EFCAB3	7.586125333	6.273468646	7.624419899	7.439837192	8.887109626
EFCAB4A	8.892970697	9.777520899	9.649399939	8.746767313	9.264483889
EFNB1	8.556357949	8.685201879	8.287888051	8.804952828	8.494130061
EFR3A	10.48432644	10.44195016	10.23550694	10.82804231	10.69099899
EGFL8	8.559975162	8.120062485	8.448929687	8.147481596	8.551311374
EGLN2	10.58812248	10.28005063	8.470647758	10.46232362	10.58236152
EGR1	6.551008899	7.282365313	6.459423596	6.562721354	6.780688182
EHBP1L1	10.03123363	9.537497323	9.307024131	10.37124354	10.01257663
EID1	10.28899471	10.77637516	11.671419	11.03737422	11.15966862
EID2	7.788172758	8.314398727	8.412578899	8.019769071	8.235388636
EIF1	16.82803465	15.90458538	16.57174008	16.52993947	16.49551516
EIF1AY	10.40592026	10.1905069	7.215458101	9.897493586	7.229587778
EIF1B	10.41530743	10.45375986	10.24511785	10.45996909	11.52238659
EIF2AK1	7.696877364	8.174871818	8.442251212	7.892604899	8.388817192
EIF2AK2	8.321919424	7.730873535	7.765298909	7.872718384	7.920494253
EIF2C2	10.22448985	10.92757694	10.70052069	10.09254304	10.73014715
EIF2C3	8.229339455	8.028563859	7.32157099	7.720541949	7.517077263
EIF2C4	7.889761727	7.409710545	7.490096869	7.398020586	7.41345197
EIF3A	12.58957491	12.75396286	13.44849295	12.88265743	13.05428976
EIF3B	10.71731517	11.4090051	10.8443678	10.93023415	11.35080354
EIF3G	10.96886286	11.76101506	11.29230964	11.4481816	11.42562271
EIF3H	13.76108056	14.00297944	13.91489381	13.86604428	13.95167504
EIF3I	8.459596535	8.695335444	9.266188263	9.000672535	8.759332636
EIF3L	10.2829717	10.52904685	10.57758511	10.73517618	10.46766096

EIF4ENIF1	8.292068253	8.147147636	7.854325061	7.834378071	7.896600606
EIF4G1	11.35278885	11.38178745	10.70902317	10.17440526	10.66945215
EIF5A	7.183932212	7.20183702	6.414650828	6.927779576	7.298462212
ELA2	6.338442758	6.110927697	6.188035909	6.961503899	6.676440081
ELA2A	9.061649657	8.996667384	8.286610323	7.898974697	8.500729384
ELAC1	6.552305929	6.770142061	7.436027273	6.873334899	6.889250929
ELF1	7.903688606	7.464355384	8.500729384	7.741457929	7.556302505
ELF2	8.826393101	8.465492313	8.226840717	7.630594101	8.490531303
ELFN1	14.50017253	15.38120792	12.17513939	14.47046648	14.30038792
ELL	9.556000646	8.914132333	9.423026242	9.068490586	8.677707162
ELOF1	8.451550535	9.055478465	9.484840202	9.078331919	9.512482222
EMG1	8.938439909	9.390834152	10.13862753	9.511202364	9.524156586
EMILIN1	8.268937657	9.087255717	8.289212586	7.890755525	8.704708384
EMILIN2	11.93486375	11.96636849	12.04432	11.84291489	11.95261936
EMILIN3	7.475955232	7.330192545	7.40526796	7.521572545	7.774939586
EML2	6.762834384	6.688924818	7.203497747	6.510975394	6.668735424
EML3	7.494798263	7.438935576	6.583409828	7.519990596	7.426803586
EML4	10.71860391	11.16423752	11.26262606	10.573928	10.94640834
EMP3	10.46108665	10.6768734	10.37894268	10.94353341	10.76737368
EMR1	8.284505182	9.038849273	9.757590192	8.158602879	9.23383296
EMR2	10.42622906	9.969141677	10.76737368	10.89755671	10.03701736
EMR3	10.57028524	9.607593848	9.534194535	10.21769501	9.560346242
EMX1	9.694213162	10.43872423	8.233838889	8.856343687	9.182204323
EN2	14.58095582	15.92931651	11.07013707	13.30255942	14.25895571
ENC1	7.979449616	7.411868313	6.608482505	6.703235576	6.471031303
ENDOD1	8.247731232	8.938439909	9.326739394	9.096395636	8.999492384
ENDOG	8.44047504	8.409548323	7.851994586	8.342773172	8.727989172
ENHO	8.086566798	7.778752535	7.543124535	7.562928222	8.152443121
ENO1	10.687913	10.76239578	10.56421743	11.33848921	10.97896732
ENPP7	6.38083603	6.079177879	5.797574525	6.567684242	6.015023111
ENTPD2	7.744459273	7.303562909	7.735579636	7.734896879	7.633233848
EOMES	7.268488485	7.548584576	8.04259702	7.392563535	7.90609097
EPB41	8.540635232	9.249612495	8.514909313	8.704708384	8.971160737
EPB41L3	7.149343495	7.334639071	7.989587677	7.154007354	7.200097273
EPB42	12.25572441	12.96241124	12.76207554	12.39660011	13.02006671
EPB49	8.395713444	9.750460616	7.644277273	8.868452172	8.679110091
EPC1	8.421350475	8.187136596	8.431326525	8.151247717	8.36556403
EPHX2	7.964547263	8.618800343	8.77608297	8.244805758	8.363862202
EPN2	9.133918929	9.620873889	9.403514808	8.397394545	8.358418384
EPS8L3	6.286870242	6.163811747	6.728207192	6.297066051	6.429848434
EPSTI1	6.529968303	6.855940818	7.206091434	6.678558889	6.819670818
ERAF	11.29399858	11.74205097	11.83330316	10.06547375	11.9091429
ERAP2	7.431067202	7.639990515	7.499556727	7.97128602	8.136641818
ERBB3	6.858224505	6.527907232	6.748587242	6.760385848	6.963370131
ERCC2	8.652374404	8.868979273	7.94734596	8.318668263	8.811517808
ERH	9.550085313	10.02644328	8.588971687	9.352487919	9.406122899
ERICH1	9.852239606	9.149684939	9.528804131	8.958493495	9.277455131
ERN2	7.101832475	8.681579566	6.587110859	6.620950929	7.003981192
ERO1L	7.362377768	8.225777414	8.01013803	8.351407394	8.205457293
ESAM	7.816902374	7.930615646	7.985767212	8.147147636	7.404051424
ETAA1	7.140009717	7.627373273	7.36166898	7.279410566	7.065949717
ETFA	8.521230828	8.486968232	9.577319545	8.625920364	8.77962597
ETV7	6.765091091	6.940977263	6.865784889	6.710946737	7.416977434
EVI2A	10.70902317	10.52689599	10.85782556	11.09565519	10.88166557
EVI2B	11.10460602	10.30151978	10.57885616	10.93171462	10.92608881
EVI5L	7.71023803	7.205228253	7.252299788	7.485478828	7.624016909
EVPL	12.28369472	12.39660011	11.3290504	10.73132595	11.74935785
EVX1	12.97977545	14.7172184	11.36610866	11.57178154	13.17778072

EXOC3L	6.673543687	6.81111	6.966460535	6.768428949	7.016197848
EXOC3L2	15.38120792	15.81050193	14.35197184	15.13975511	15.31193978
EXOSC10	9.191263899	9.514496606	9.158023	8.951513343	9.312041414
EXOSC8	8.478263182	8.855784909	9.533033172	8.85049999	8.945002717
EXOSC9	8.29635502	8.854095293	9.359207242	9.079484556	9.484209455
EZH2	8.529888505	8.84786598	8.781680606	8.347717242	8.492322828
F11R	9.935661677	9.910074283	10.2829717	10.04776272	9.670530667
F12	10.31193963	9.717365758	10.0672481	10.01340346	10.48432644
F13A1	11.15808934	11.54053762	12.15130062	11.48258282	10.90754603
F2R	6.26253897	6.741460808	6.759458	6.982135444	6.841391899
F2RL1	6.873334899	6.780968242	6.94985404	6.401596919	6.447674828
F5	10.05607537	8.893419636	9.224452333	8.664725051	9.132080535
FAAH2	6.604981172	7.06161799	6.575765641	7.051418091	6.734222485
FADD	9.695719162	8.896578162	9.11211799	9.484209455	9.203189636
FADS1	7.509397909	7.753435232	7.640998838	7.873714818	7.942409707
FADS2	7.327495141	7.655968232	7.072036626	8.08193004	7.301802939
FAH	7.412806768	7.534302667	7.143674071	7.486746556	7.250238485
FAHD1	6.985120646	7.533663232	7.695265798	6.877410515	7.496345141
FAIM3	8.265565879	8.700606657	7.962680434	8.747331152	8.446184828
FAM100A	8.434806414	8.99408802	7.519695475	8.323598384	8.695335444
FAM100B	14.62217632	14.05855866	14.25042984	13.78027417	14.17411554
FAM101B	10.82416773	10.48310631	9.553430788	10.10454015	9.782868192
FAM102A	11.53473316	12.05489479	12.86086109	12.21312889	12.3613163
FAM103A1	10.13671639	10.15186663	10.09795649	9.94767803	10.34717129
FAM104A	10.16454544	10.78551178	10.6426412	10.08599138	10.65365596
FAM107B	10.75600216	10.89755671	12.02514953	10.92337564	10.86566339
FAM108A1	10.73132595	10.75178718	9.861430101	11.13085117	10.51886057
FAM109A	7.680542242	7.707585606	7.166060222	7.391081455	7.711891616
FAM109B	10.32418729	9.822898081	9.492465394	8.459227657	9.258612929
FAM111A	10.17122401	10.08325108	10.81142627	10.36134852	10.34418014
FAM113A	7.210358909	6.495272566	6.613716152	6.98921798	7.079103131
FAM113B	8.867411152	9.701165495	9.154469939	9.273640758	9.371514253
FAM117A	10.8947391	11.76588718	10.83363242	11.67849095	12.51066587
FAM118A	7.341966626	6.542971677	7.894315778	7.487940131	6.590932293
FAM122A	8.611710212	9.026117657	8.741655828	8.354182626	9.009915859
FAM124A	7.520306162	8.149032212	7.354199768	7.329290475	7.636262586
FAM126B	9.466909636	8.844262616	8.444861909	9.321890899	9.226128374
FAM129A	11.97196754	11.19638858	11.18471248	11.39772243	11.63629658
FAM129B	13.58996905	14.15092087	13.23864174	13.04869312	13.52959744
FAM129C	8.337114525	8.003147788	8.445297313	8.194244232	8.154321646
FAM131C	16.8710039	17.23317693	16.3077943	16.33688632	16.82803465
FAM138B	7.148192263	6.525168444	6.407035273	6.841827051	7.012571859
FAM13A1OS	8.191909586	7.430781919	7.635907081	7.654890798	7.517772121
FAM148A	10.25906505	11.55928848	8.20265299	10.4429644	10.81664077
FAM148B	7.311327071	7.93888704	6.986766929	7.003455566	7.037511364
FAM153B	7.049242879	7.382214869	7.422534091	7.246997929	7.446082798
FAM153C	6.656891394	7.011036525	6.395573626	6.727438899	6.74432
FAM160A2	11.35475345	10.30358984	10.39521192	10.88945836	10.92337564
FAM160B2	7.912742707	8.209054152	7.226681909	8.300483646	8.039451586
FAM162A	9.845595727	10.39747702	10.25627793	10.22557955	10.67305197
FAM164A	7.619556354	8.503061444	8.295436051	8.432612263	8.302900242
FAM168B	7.691187768	7.665645141	8.731005677	8.093860848	8.053207788
FAM173A	8.108111747	8.389653889	8.302062758	8.511758747	8.368780919
FAM174A	10.10542249	9.610538374	9.621550919	9.30577901	9.522660222
FAM176B	9.245445293	9.580097263	8.814624313	9.468772697	9.30770201
FAM178B	7.422534091	6.912275354	7.536179778	7.717737636	7.819838162
FAM180A	9.517771202	9.291393586	9.311440606	9.184527788	9.647889364
FAM181A	12.27421104	12.72610186	12.29275278	11.32559397	12.36473556

FAM186B	7.639990515	7.223246162	7.274888343	7.017803152	7.214035101
FAM20C	8.841575667	9.407554697	8.620285051	9.305189859	9.070661273
FAM24B	6.64233597	7.259306869	7.77977198	6.636058242	6.874731313
FAM26F	9.541041677	8.39867397	10.26116661	9.346206162	9.784374313
FAM27A	7.74007802	7.461239687	7.217862778	7.558170465	7.047573414
FAM30A	7.918105152	8.016429949	8.546982556	8.069753788	7.889381343
FAM38A	8.391869283	8.454049747	8.304980606	8.705608919	8.230588212
FAM3B	6.306878242	6.482726162	6.725034798	6.374790061	6.431354778
FAM3C	6.720840657	7.007335798	7.782178323	7.028313949	7.05790099
FAM44A	9.444057586	8.941795242	9.11093099	9.150811232	9.026636737
FAM45B	8.276014121	7.434843313	8.391020323	7.91465098	7.991889949
FAM46C	8.130283051	9.088505869	8.181196919	8.39867397	9.188424626
FAM47C	8.614861051	9.271123182	7.935875313	8.138908141	8.758364879
FAM49A	10.22951336	9.503271071	9.820433545	9.814415596	9.612498475
FAM49B	13.29117716	12.52461444	11.71042621	12.85589012	12.65472241
FAM53C	9.928455091	8.923097444	9.450568111	9.008373061	9.071276071
FAM62A	8.949261586	9.195011616	9.022478232	9.784374313	9.35858599
FAM65A	9.537497323	10.43757507	9.169326323	9.236429667	9.924448444
FAM65B	11.39185194	10.58236152	10.99469779	11.28896545	11.21668249
FAM69B	7.601973414	7.771866444	7.056486293	7.311629869	7.210929778
FAM73B	7.66930299	7.344013535	7.133813545	7.939228495	7.932453717
FAM78A	10.45877561	10.5029511	10.65500785	10.61480751	10.71731517
FAM81B	6.414872495	6.615401818	6.828146828	6.627335	6.759969273
FAM90A1	6.72401001	5.827491222	5.99330002	6.514326051	5.906986101
FAM90A7	7.189710394	6.062205586	6.787317758	6.876878333	6.224063596
FANCA	8.020096283	7.896600606	7.310724131	7.290204495	7.73355204
FANCE	9.405539273	9.376667182	9.624391323	9.247858869	9.312727606
FANCL	7.570765313	7.963792818	8.181963182	7.683675283	7.952774121
FAR2	7.476910626	7.384605828	7.813759899	7.229904545	6.827413455
FAS	8.789683394	8.516311424	9.474605101	8.634342798	9.09824599
FASLG	6.950382242	7.003715121	7.11489098	7.700026646	7.214922364
FAU	13.66784462	13.92172837	15.13975511	14.08841453	13.96779242
FBN1	7.901142576	7.732919727	8.112876232	8.526186556	8.925229707
FBN3	8.671294253	8.989652131	8.263041909	7.326350616	7.88825004
FBP1	9.095762	9.625623061	9.240719434	8.885078273	9.09178804
FBXL13	8.35752198	7.132449434	7.236577444	8.168969465	7.690840778
FBXL14	8.672348475	9.901640636	7.232456929	7.95873603	8.392711081
FBXL16	8.702574091	9.167114	8.884521697	8.399975414	8.978723121
FBXL17	10.55842197	11.08756575	9.121511687	10.08700287	10.41530743
FBXL19	9.838413929	11.60680648	9.099925818	9.286110869	10.4450719
FBXL3	9.986114121	9.713188212	9.945994374	10.16454544	9.940265465
FBXL5	12.3613163	11.74935785	12.41337695	12.06397065	12.12939166
FBXL6	7.194433949	7.387061818	6.676225182	7.34349297	7.429567525
FBXO15	7.157774636	6.775501273	7.157499455	6.955441212	7.20666299
FBXO17	9.472030465	9.945994374	8.962901121	9.025691242	9.433258586
FBXO2	6.842504626	7.374297343	6.740481444	6.97809596	6.883542434
FBXO30	8.89604703	8.562227051	8.450646323	8.673363869	8.685201879
FBXO33	9.922126404	9.602112616	9.782868192	9.749004051	9.641007131
FBXO38	8.053619525	7.73355204	8.52575703	8.166172404	8.480536717
FBXO7	14.06476988	14.02572128	14.70594079	14.5513462	14.70594079
FBXO8	8.26142704	8.115023838	8.963499596	8.450171172	8.619347838
FBXO9	9.883591758	10.80389925	10.53842125	10.38208186	10.47479655
FBXW4	8.057864374	8.733447455	7.723009	8.498167798	8.096128192
FBXW5	10.50166798	10.66945215	9.782133848	11.32376318	11.00902763
FCAR	7.550195808	6.784856394	7.195224778	6.84778002	6.801828172
FCER1A	9.024707707	9.352487919	9.932690333	9.417037424	8.762742172
FCER1G	12.32659908	13.55640443	11.25523353	12.0750029	12.66627862
FCGBP	8.238272808	8.009072212	8.566871343	8.447526838	7.85507

FCGR1B	10.23550694	10.24612484	10.0406788	9.542425758	11.09721747
FCGR2B	7.816627253	8.225261616	8.544633697	7.392836818	7.351279444
FCGR3A	16.90924948	15.03840752	16.4534382	17.12376032	16.36806566
FCGR3B	12.86955734	10.72633412	12.26162026	12.26162026	11.43773109
FCHO1	9.080123545	8.799825313	8.303793737	9.023639636	8.336257222
FCHO2	7.924502232	7.516138929	7.035678848	7.646666444	7.900388485
FCHSD1	7.074987333	6.535531162	7.201015091	7.449265303	7.479422939
FCN1	13.26276274	13.30795266	13.04869312	13.45466248	13.12562627
FCRL2	7.421037465	7.100441616	7.669657737	7.126812859	7.099269525
FCRL3	6.194253556	6.592903869	7.052221778	6.817257404	6.574185192
FCRL5	6.870354606	7.227882465	7.352900667	6.66759498	6.941216071
FCRLA	6.723002525	6.578458283	6.774175343	6.420979687	6.201940414
FCRLB	10.96311364	11.51465712	9.580097263	10.7982674	10.97185531
FDX1	7.36694602	7.650858424	8.349393212	7.777311636	7.764199131
FECH	8.209054152	8.366462404	9.094691273	8.463578343	9.509876273
FEM1A	7.288128061	7.862160495	7.020975202	7.229324455	7.809001414
FERMT3	10.29851595	9.913634949	8.954798808	10.48659409	9.711723394
FES	11.29744903	10.83642757	9.235747242	11.34732253	10.61001127
FETUB	6.879719566	6.266464606	7.013654717	6.905589111	6.963895283
FFAR2	10.06255975	8.714332636	8.997807768	10.33476777	9.861430101
FFAR3	9.099925818	8.793339475	8.757405333	8.400464071	9.039454747
FGD1	7.384903253	8.043818737	7.363924808	7.246141232	7.709511535
FGD3	10.71031814	10.10187506	9.159785394	10.31378621	9.904237354
FGD4	9.522660222	8.357077121	9.299916465	8.654464586	8.406205495
FGF3	13.64789782	14.9847254	12.63888703	12.94367069	13.58996905
FGF9	6.74001704	6.521458212	7.00817199	7.458509535	7.21466303
FGFBP2	9.896649636	9.702758343	9.950180667	11.44643795	10.01524205
FGFR1OP	8.629474212	8.580231323	8.023866626	8.239474646	8.068931465
FGFR4	6.884060404	6.751169162	6.71142402	7.370661465	7.210091465
FGL2	12.89113428	12.25572441	13.56234785	13.31405593	12.67378338
FGR	12.22341713	10.93789932	10.55213977	11.89628941	11.03008027
FHIT	9.980047091	10.50677257	9.244930505	9.603529667	10.00186072
FHL2	8.499404646	9.270407879	10.28190294	9.353057535	10.27804811
FHL3	8.378515131	7.119081899	6.661555384	7.32489101	7.613528424
FHOD1	9.515069697	9.035040848	8.551751717	9.355403384	8.912452162
FIBCD1	7.15072903	7.800216455	6.629471747	7.443557333	7.570191354
FIBIN	6.876372364	6.186368	6.727962222	6.370541444	6.462851414
FIS1	10.50543333	11.24323027	11.28053313	10.59292728	11.15342364
FKBP1B	8.178268222	8.580722586	8.784105778	8.418118394	8.548304869
FKBP5	9.621550919	8.158321101	7.713612677	7.889381343	8.608532485
FKBP8	9.377330121	10.61261659	8.646531515	9.354902374	9.720489222
FKSG2	7.803554182	8.32856404	8.358783152	7.560760293	8.335469222
FLI1	11.58005536	11.10170724	11.24457024	11.83072395	11.74670945
FLJ00049	6.933276949	6.879943566	7.369510384	7.058212263	7.084144263
FLJ10357	12.86086109	12.38794592	13.34204398	12.92937388	12.19398134
FLJ10490	11.31267384	11.79870316	10.34717129	11.16281786	11.24457024
FLJ11151	11.26623333	11.25523353	10.97054498	11.34381562	10.85782556
FLJ11710	8.500240172	8.57070398	7.675893758	8.269818727	7.85299496
FLJ13197	6.399020677	6.962306323	6.866055313	6.498091727	6.906817121
FLJ20021	9.530205505	9.632768747	9.710962273	10.03385507	10.17224075
FLJ21272	7.982442283	7.614497747	7.769805091	7.589888475	7.869396606
FLJ21865	9.763952101	9.093001495	8.838951081	9.611261414	9.446464909
FLJ22184	15.65613201	16.4534382	13.25686564	14.66395575	15.19830883
FLJ22662	13.83772389	12.80949032	12.85589012	13.42153085	12.97555423
FLJ23185	10.24227579	9.962571606	9.925226333	10.57282784	9.751885293
FLJ25006	6.80380202	6.853461566	7.751645273	6.478358667	7.510907818
FLJ30064	6.833840465	6.696696758	6.727438899	6.561505162	6.119415222
FLJ30901	7.723384495	7.320252869	7.803944101	7.828260424	8.21550303

FLJ32214	7.011036525	8.209418434	6.300754434	6.808010384	7.087495071
FLJ32252	9.242526283	8.447076788	9.195534384	9.426842081	9.946747141
FLJ34651	8.273061586	8.30460302	8.341518747	8.33024502	8.584413131
FLJ35390	10.75737971	12.68903729	9.915234909	10.26116661	11.03452834
FLJ36070	7.151291707	8.553595747	6.501249222	6.792458242	7.052765889
FLJ37078	7.928832091	8.428176394	7.772843323	7.350388939	7.806845253
FLJ38359	8.129067798	6.559739475	7.314926455	7.543741273	6.674272111
FLJ40113	6.620481919	6.205762343	6.466022525	6.499057505	6.434478081
FLJ40125	7.693828071	7.926719768	8.561789232	8.234636465	8.603745778
FLJ40142	8.984797889	9.240082808	9.412634061	9.099242404	9.271759586
FLJ40330	8.607626293	9.552821717	9.612498475	9.577319545	9.751080646
FLJ40504	7.419275485	7.025866354	6.838696091	7.224144889	6.749084333
FLJ43093	6.413438525	6.250488859	7.378842657	6.274896838	6.47944304
FLJ43276	7.763504859	7.390471152	8.003479939	7.437384141	8.339546939
FLJ44715	6.067709717	6.100921808	6.633233677	6.377717758	5.925955323
FLJ45055	10.95374716	11.24816417	12.01956714	10.55966675	10.30151978
FLJ45422	10.28190294	9.563605545	8.430929525	9.987874111	9.706432808
FLJ45717	11.30690717	13.39659876	9.679645121	10.5041569	11.71659127
FLJ77644	8.663303283	8.757405333	8.64224801	8.577417788	8.945493505
FLNA	9.356741384	9.576740677	7.972012263	9.739033131	9.345614434
FLOT1	9.829822242	8.870602515	8.632349838	9.824540899	9.265583747
FLOT2	9.287277576	8.597027768	8.029005444	8.976374343	8.740195303
FLT3	6.638550414	6.051299838	6.278635202	6.239248505	6.346660364
FLVCR1	7.008962394	7.959080606	7.456429202	8.066048727	7.706567859
FLYWCH1	7.566119051	7.160253414	7.662925475	7.723687384	7.873377879
FMC1	8.001277303	8.273878929	8.157153081	8.228510596	8.391460737
FMN1	6.634850818	6.036027182	6.104869535	6.834332747	5.898805899
FMNL1	10.32309273	9.772980061	7.927799515	9.982665242	9.379180747
FN3KRP	8.082778869	8.348145404	9.415860727	8.835493283	8.650384111
FNBP4	9.696433626	10.05053825	10.38631174	9.58708204	9.785983535
FOLR2	7.312858101	7.070381242	7.746135697	7.079239247	6.904353566
FOLR3	10.44832821	8.998971485	8.614861051	9.039454747	10.92880859
FOS	10.00361533	9.117478424	10.32635032	9.299916465	9.21352099
FOXB1	11.62059475	13.49745762	9.634846707	12.0724233	11.91651973
FOXC1	7.154661646	6.819670818	6.265371141	6.852686475	6.675226152
FOXC2	10.87637444	12.9851923	10.20164867	10.65871503	11.34892675
FOXD3	8.853585596	9.631972414	8.643157737	9.442729677	9.17294002
FOXE1	7.789573343	8.295009141	6.662284414	6.766293242	7.143149899
FOXE3	7.695611414	7.536878778	7.358375414	7.533663232	7.81270001
FOXH1	10.7442872	10.98350754	10.05053825	10.06822268	10.83223099
FOXJ1	6.747331182	6.610905657	7.162716495	6.750367323	7.023397576
FOXL1	8.695335444	8.210994667	8.678658293	8.435650677	8.551751717
FOXN1	6.929372162	6.958444212	7.28903701	6.702513515	7.141717727
FOXN2	9.020821455	9.109760465	8.372433475	8.914132333	9.215946081
FOXO3	9.385837242	9.704210374	9.245445293	10.05236285	9.789131121
FOXS1	7.736619182	8.181574081	7.350114798	7.932453717	8.181963182
FPR1	15.13975511	13.54221797	13.85817003	14.53168901	13.98081649
FPR2	11.14866374	10.2600862	11.09250749	10.6820228	10.56084718
FRAT1	7.451812333	6.526955566	6.262348667	6.801276	6.429246364
FRAT2	13.9071606	12.87421343	13.11515098	13.54221797	12.9851923
FRG1B	8.166602566	8.291166909	7.81061403	8.301264212	8.411285283
FRMD1	7.52646604	7.436336172	6.821271707	7.02555202	7.088019323
FRMD4A	7.215770273	7.544047919	6.255807596	6.904353566	6.976489687
FRMPD3	8.890373556	7.916712838	8.810986707	8.624974828	9.050265121
FRS3	8.012698303	7.668960768	7.638629778	7.867668606	7.242951465
FRY	10.77637516	9.976273051	10.68452674	10.16546821	9.851320949
FSCN2	7.372532788	6.450607838	7.198597879	7.158590636	7.02612702
FSTL1	7.134964384	8.200163707	8.490053091	7.320621818	6.768172747

FTHL17	11.76804793	11.20345844	12.15752365	11.60266156	11.30301738
FTL	14.59173737	14.76275492	14.4185289	14.86987024	14.29173122
FTSJ3	8.45110203	8.884521697	9.802496737	9.647013263	9.889177586
FTSJD2	7.556918475	7.511838192	6.725993303	7.16725497	7.224435778
FUBP1	7.102982434	7.486457414	6.488640525	7.399358222	7.573788949
FUCA1	8.097958939	8.329784131	9.811898121	8.205457293	8.195405505
FURIN	7.545042545	7.694540808	6.073749475	7.33350399	7.212087616
FUT11	7.886820596	7.568622485	7.655304091	7.244714616	7.709201212
FUT2	7.142620404	6.510509061	6.984849141	6.745632475	7.208062909
FUT7	7.84876403	6.925244263	6.447869455	6.976489687	6.634850818
FXYD5	10.00094823	10.51650912	9.186096828	10.37346225	10.15186663
G0S2	9.126663535	7.721580051	8.952561111	8.547862646	7.578291899
G3BP1	8.248137606	8.690918616	8.260520646	8.403471909	8.734416606
G6PC3	7.496684051	6.97940904	7.21289504	7.122697263	7.525550222
G6PD	7.026391172	6.963895283	6.034044859	6.845837626	6.602902657
GAA	13.25686564	12.99088515	12.46055504	13.29684189	12.70002263
GABARAP	11.81018832	11.38391359	11.21816154	12.03602449	11.92665931
GABARAPL1	10.34626898	9.684886576	10.43564423	10.26332497	9.952967535
GABARAPL2	11.87912023	11.84291489	12.23505088	12.09804166	12.71520416
GABBR1	7.328126596	7.144617717	7.266813283	7.315791616	7.491688212
GABRA3	6.848551889	6.395106727	6.699888758	6.669931545	6.690572232
GABRB2	7.81270001	7.471589222	8.134790222	7.363924808	7.75479003
GABRE	6.719810283	6.228764364	5.938785545	7.328995606	6.195258121
GABRP	12.59323492	12.92937388	12.86955734	12.93862501	13.29684189
GADD45A	8.808345061	8.046784596	9.168828475	8.44129198	8.345353354
GAGE7	6.587380232	6.056434111	5.762353717	6.171621859	5.758957838
GAK	9.930141081	9.542425758	8.677707162	10.09506496	9.599465606
GAL	6.386235697	5.829781091	6.537953232	6.190072818	6.136533313
GAL3ST3	6.924186162	6.307051293	7.693516556	7.197763283	7.298769596
GALNAC4S-6ST	12.59668127	11.71443529	12.08680505	11.59021288	11.63837311
GALNS	8.412578899	7.592047838	7.213710414	8.189895404	7.502072596
GALNT14	9.455711606	8.246470545	7.68296396	7.306206455	8.314030071
GALNT3	8.257595869	7.478150263	7.251486172	7.444173596	7.47214603
GALNT6	6.677606	6.891782758	6.237576566	6.763342091	6.652654
GALNT7	8.96575796	8.79576798	8.002342131	8.714332636	8.513601889
GALR3	15.72204116	16.28343863	13.39037726	15.08134613	15.449005
GAPDH	12.38063147	12.04432	11.04464523	12.59668127	12.01673563
GAPT	9.811091061	9.269847242	8.98371896	8.757866131	8.978204141
GAR1	8.992473788	9.329922273	10.14326599	9.479084687	9.694213162
GARNL4	6.544328465	6.470121364	6.564057303	6.112523455	5.962632949
GAS5	9.582110717	10.29432836	10.62647628	10.05053825	10.13375911
GATA1	8.31214998	9.337187111	7.710578727	8.999492384	9.190205293
GATAD2B	6.861656576	6.208721374	6.34126302	6.866597293	7.487078798
GATM	6.341087596	6.619037364	5.914668949	6.37731698	6.570729323
GBA	8.632799758	8.143419303	7.587635919	8.268052495	7.844877657
GBE1	9.837575172	9.288419414	9.57181996	9.16271003	8.955359253
GBGT1	7.619933919	8.165380303	6.975683768	7.707249808	7.617533182
GBP1	8.951989212	9.171801828	9.848227263	8.833337414	9.220211768
GBP2	11.65052704	10.5752557	11.87912023	11.84529477	11.60266156
GBP3	8.123132566	7.857447394	9.088505869	8.899254879	9.165786899
GBP4	8.894491141	9.293068828	9.283736939	9.269224374	9.35188598
GBP5	11.58195332	11.03580591	12.31419772	11.39983314	12.0750029
GBP6	11.67593314	12.03602449	12.19996238	12.29275278	11.82112126
GCA	15.22815255	13.65450155	14.40789433	14.58095582	14.50017253
GCAT	7.444499747	6.632973788	6.152834101	6.628772899	6.890245747
GCC2	8.425403333	8.204380404	8.133179586	7.937437293	8.29291099
GCDH	7.779426687	8.137479222	7.505175253	8.347358778	8.175968162
GCN1L1	11.20660223	11.62739451	11.49239347	11.93770961	11.671419

GCS1	10.0719806	10.93494087	9.184000242	9.554043101	10.4655823
GDE1	9.468134182	9.231349182	9.770750222	9.62845904	9.814415596
GDF15	7.121365859	6.836284939	7.85710597	7.728044798	7.820605525
GDF5	9.835081424	8.668931657	9.134591697	8.93638097	8.895503556
GDF5OS	7.034867141	8.353342091	6.710071909	6.814458515	6.941709929
GDPD3	8.030181424	7.433629313	6.595700424	7.695611414	7.186028475
GDPD5	7.962380919	8.069753788	7.110844919	8.136641818	8.059832677
GFI1	6.320458162	6.475742909	7.377037515	6.695959364	6.759458
GFI1B	7.898205273	7.812003626	7.956615091	8.331046747	8.010919919
GFOD1	10.19332507	10.55333109	10.44602022	10.66229262	9.955574404
GFOD2	9.901640636	10.45628541	8.840590677	10.15483313	9.346872071
GFRA3	8.092218303	8.076845152	7.897352323	7.688949081	8.363468485
GFRA4	10.18468509	11.64848719	9.287277576	9.799317404	9.923012889
GGT1	8.460863192	8.209801687	7.249375465	8.212685152	8.241600556
GGT3P	7.663618646	7.001869131	6.541370778	7.449946354	7.353560545
GGTA1	8.886620687	10.28089537	11.44643795	10.88031852	10.26445444
GGTLC1	8.756886111	8.090886384	7.639662323	8.069395848	7.983548525
GGTLC2	7.961295828	7.284105152	6.971315	7.745466071	7.733228788
GH2	7.279410566	8.309247939	6.740256788	7.111963455	7.009209616
GHITM	10.48897537	10.30470526	10.6768734	10.41426339	10.73384189
GHRHR	8.185563303	8.122747707	8.235388636	8.503455465	8.00874802
GHRL	9.18155302	9.150235333	8.989652131	8.646531515	8.717364515
GHRLOS	8.339115859	8.085049192	7.693828071	7.433629313	7.854005263
GHSR	8.293340606	7.776629576	8.095746162	7.828991111	7.841061444
GIGYF1	9.35067002	10.05144087	8.812517081	8.745474636	9.392652323
GIMAP1	9.925226333	10.04511943	10.69682519	10.60044575	10.2861211
GIMAP2	9.473962505	9.405539273	10.38738452	10.03611568	10.08325108
GIMAP4	12.30167305	12.052647	12.11841559	12.54589185	12.57877126
GIMAP5	9.778131475	9.93099102	10.58935103	10.36465285	10.25627793
GIMAP6	9.421059	9.518503263	10.46232362	9.774477667	9.724814657
GIMAP7	12.05813886	12.27773509	13.47832004	12.91656586	12.82153585
GIMAP8	8.871209182	8.694840596	9.35128199	8.796283929	8.973478152
GIPR	9.090721626	8.578393657	8.905887879	8.197369283	9.177504333
GIYD1	9.503271071	9.224923687	8.657372182	9.141588162	8.930963202
GJC2	7.317304515	7.230210646	7.524403394	7.537240869	7.180020091
GJD3	8.205457293	9.274850788	8.463578343	8.350619919	8.877161475
GJD4	7.883536596	7.141446111	8.052077899	8.010919919	7.385857192
GLIPR1	11.08756575	10.72516317	10.10736711	10.80782516	10.80644997
GLIPR2	14.38889659	13.46005067	14.07988992	13.46712151	13.73439002
GLIS1	7.203497747	6.760820788	6.653830545	6.629005263	6.282201616
GLIS2	8.660608859	11.09565519	8.212243444	8.642670768	8.021628354
GLOD4	8.407442505	8.933684374	8.998971485	8.862785404	9.032817303
GLRX	12.63102018	12.01427079	12.62807191	12.35798465	11.59021288
GLRX3	8.646531515	8.968910758	9.35858599	9.344897333	9.456392222
GLRX5	13.69473258	14.25895571	14.72870348	13.98831971	15.29549959
GLRXL	8.852092202	8.163053768	9.304577515	8.721926778	8.179120646
GLT1D1	12.57107442	11.308834	11.71895032	12.16942168	11.86922315
GLT25D2	6.58891503	6.626176061	6.110613111	6.771643182	5.863786071
GLTP	8.322806515	8.082337909	7.965660091	8.107270071	7.920144808
GLTPD1	8.950874253	9.266794162	7.987573434	8.603745778	8.752912545
GLTPD2	7.233346848	6.927522051	7.523756919	7.752360667	7.163863636
GLTSCR1	9.155079848	9.345614434	9.240082808	9.413321525	9.32739897
GLTSCR2	12.65472241	12.67378338	12.33273925	12.69316533	12.84197228
GLUL	10.8193093	10.61001127	11.96636849	11.12001247	11.73070023
GMEB2	7.79851096	8.025864485	6.644450293	7.384605828	7.451812333
GMFG	15.86132141	14.65397593	15.83165575	15.58150095	15.32551587
GMIP	11.42787377	10.86963124	10.29949835	11.06573849	10.82664672
GMPR	10.11452638	10.69574272	10.20748734	10.37467131	10.51650912

GMPS	8.526649808	8.899254879	8.390560192	8.594016788	8.870602515
GNA12	7.488894061	8.376150616	7.424927172	7.521266808	7.622357354
GNA15	6.754244737	6.894391313	5.857349727	6.741204455	6.633233677
GNAI2	9.698896848	9.000672535	8.248986687	9.243096889	8.82024202
GNAZ	9.326739394	9.644080808	10.41846051	9.548028869	9.005433152
GNB2	10.75453103	9.786685869	7.841729333	10.23550694	9.790672212
GNE	7.387061818	7.56574702	8.274698687	7.684641212	7.973227556
GNG10	12.7452022	11.77053658	12.62127559	11.94234713	12.04990566
GNG11	11.89628941	11.94481318	12.89886333	12.77015535	12.24351077
GNG13	7.066192545	7.106592788	6.951169364	6.680925848	6.829338141
GNG8	8.157542717	9.519820636	7.940254566	8.476485121	8.764084763
GNL3	8.434412929	9.082448343	9.039989364	8.729972586	9.108564253
GNL3L	8.459227657	8.629474212	9.072998758	8.538380869	8.812517081
GNLY	9.281446566	10.042425	8.683087172	11.30506756	10.63870636
GNPAT	8.188779434	8.180785838	9.388912091	8.581169111	8.39402401
GNRH2	7.474059606	7.389558374	7.759260374	7.653990838	7.873714818
GOLGA8A	7.200755828	7.442908828	8.183602061	7.708578687	7.847720859
GOLGA8F	7.096055535	7.07857001	6.858224505	7.238429162	7.459405444
GOLGA9P	8.838951081	8.591239465	8.589797808	8.69335002	8.687541707
GP9	10.60507136	10.84844593	11.11230451	10.8605042	10.52689599
GPAA1	6.950101798	7.02934998	6.579844919	7.172411071	7.096055535
GPATCH4	7.866656354	8.526186556	8.618302869	7.700334879	8.086566798
GPC1	7.618249414	7.214315212	7.197080263	7.805068949	7.299714313
GPC2	7.597541737	7.122424869	7.065103374	7.058764576	6.790568566
GPC4	10.73911267	9.519086535	10.05320582	10.12799521	9.533033172
GPER	6.964119424	6.579152939	7.593349828	6.835859525	7.266003808
GPI	11.02551486	11.63401423	12.0089388	11.70348036	11.85033795
GPIHBP1	7.25490398	6.925960677	7.92237903	7.207500556	7.458795919
GPR108	8.025051697	7.626470505	7.376746737	8.082778869	7.644277273
GPR109A	7.994566778	6.71776502	8.598980253	7.370950798	7.489812667
GPR109B	11.79171062	11.19966733	11.4481816	11.89361272	11.60680648
GPR114	8.014589606	8.004655889	7.911995121	7.928832091	8.023506091
GPR119	10.50677257	10.99763472	9.923658606	9.53973704	10.30058205
GPR12	7.607714535	7.579015667	7.195783737	7.941721242	8.239474646
GPR142	8.295436051	8.124212202	8.437457535	7.417598061	8.534432515
GPR146	7.670275758	8.769354545	8.321919424	8.112876232	8.842639202
GPR15	7.637927172	6.269158071	7.005636414	7.88825004	8.327353333
GPR150	14.77222727	15.70163214	12.59668127	14.223913	14.74028573
GPR153	15.99508933	16.49551516	13.26910509	15.59877501	15.54332231
GPR156	11.86652814	12.0838996	11.68948846	11.25898056	11.88148631
GPR160	9.66831603	7.880750535	9.832711535	8.632799758	8.580722586
GPR162	8.418553323	8.005371424	7.688949081	7.947667091	6.998508111
GPR171	8.367840636	8.819236758	9.434425707	9.01707996	9.11211799
GPR177	7.527697455	6.415875869	7.413750677	7.04545701	8.035675313
GPR18	7.400864778	7.725703576	7.927113394	7.633560879	8.144161414
GPR182	7.396159798	6.566725596	9.016491202	7.951633808	8.338355444
GPR183	8.321152475	9.046362323	9.269224374	9.36439596	9.230770667
GPR20	7.303163338	8.114611232	7.418946535	7.789255535	7.609232899
GPR25	8.893968333	8.971727333	8.546545061	8.344860929	8.509502414
GPR31	8.855784909	7.85710597	8.528049141	7.883536596	8.268510495
GPR32	7.00817199	6.401803212	7.057011424	7.015344242	6.971045939
GPR37	9.11510898	8.340681505	8.799825313	8.109266465	8.494982343
GPR44	6.828875111	7.34349297	6.684542485	6.993463051	6.417620515
GPR56	9.785983535	9.804037697	9.560346242	10.35284213	10.20060626
GPR65	8.653441707	8.352537101	9.692793657	9.205404404	9.310205828
GPR77	8.207099091	7.485478828	7.803193929	7.545335788	7.767362636
GPR78	8.206288727	9.622300838	6.806225091	8.803482152	9.885156737
GPR97	10.08145981	8.645541747	7.52469401	9.300983919	8.677215515

GPRASP1	7.067324051	7.492628778	8.375668162	7.525550222	7.460591212
GPRIN2	7.285574899	6.538933859	6.515779859	6.875689354	6.270128909
GPS1	8.388817192	8.962901121	7.586712596	8.918202434	8.725113212
GPSM3	9.69725304	9.895061889	9.425521616	9.237064687	9.385290182
GPSN2	10.1300726	9.912769303	9.447242061	9.526200354	9.673359929
GPT	8.405088753	8.165787747	9.003357768	7.98285402	9.393934131
GPX1	16.61084374	16.8710039	17.29704085	16.7804214	16.61084374
GPX2	7.034331232	6.821773354	7.040256253	6.762066263	7.339805162
GPX3	7.028313949	6.292920576	5.795144121	7.731201121	6.258382485
GPX4	9.597513697	10.29208493	9.646274414	10.41224321	10.66105788
GPX5	6.635819404	6.594827192	6.106316394	6.633233677	6.597486293
GRAMD1A	7.757833798	6.847553121	6.454301414	7.255477111	7.208855192
GRASP	9.022478232	9.380979222	9.626304798	8.932093424	9.124449354
GRB10	9.294355394	6.972885929	7.18283101	7.556609313	7.877844556
GRB14	7.048124798	7.379463242	7.05481101	7.274379929	7.099574394
GRB7	6.874731313	6.294305606	6.902232848	6.477312747	6.996297444
GRIN1	9.997192202	11.27695531	9.212349869	9.602112616	10.43332542
GRIN2C	6.577277596	6.516258222	6.477312747	6.62116196	6.68968302
GRIN2D	15.08134613	15.99508933	13.42153085	14.31917195	14.83117585
GRINA	8.978204141	9.291911616	8.083485404	9.203760495	9.392082222
GRK5	9.829119768	10.0728998	10.36465285	9.627745303	9.161459798
GRK6	10.57996368	10.68338253	9.290152727	10.60383217	10.41957053
GRN	15.21350605	15.13975511	15.21350605	15.03840752	14.88238784
GSDMB	8.524356212	8.047188455	8.271405859	8.668536687	8.600356081
GSDMD	9.752732475	9.341222778	8.51269104	9.975431768	9.818860343
GSN	11.01376888	10.81034167	11.21187068	11.09089144	10.93023415
GSPT1	7.652952818	8.433468657	8.404822414	7.892936636	8.710391727
GSTM3	6.608949242	6.598705667	7.092334828	6.453863293	6.08292096
GSTP1	13.33155169	13.69473258	14.5413574	13.74093847	13.6823979
GSTZ1	7.485802727	7.470886646	8.127056495	7.239990273	7.618887111
GTF2B	9.960013465	9.747577162	10.75453103	9.93187304	9.631972414
GTF2E1	8.174468182	8.003903253	8.859715222	8.199709495	8.013857071
GTF2E2	7.676615465	7.475955232	8.538380869	7.866656354	7.700670768
GTF2H2D	9.308265131	8.252372808	9.879566364	8.743124222	9.342353505
GTPBP3	7.61520499	8.039790061	8.738735232	7.669657737	7.584965596
GUCY2G	6.444835424	7.626793707	5.772434121	6.353596172	7.083894141
GUK1	15.56435861	15.74256723	15.76560628	16.02065153	16.2036826
GUSB	11.68038318	11.48061491	11.86922315	11.8358519	11.77305263
GUSBL1	8.755916071	8.464081152	9.636929838	9.232683343	8.700142556
GYG1	8.141770949	7.353560545	8.079553929	7.643944697	7.729367111
GYLTL1B	8.41598997	8.657806242	7.861832121	8.286610323	8.751946515
GYPB	7.31991999	7.627997354	7.730484909	7.089371313	8.598008343
GYPC	13.94443	14.33555643	13.29684189	13.97489728	13.56886427
GYS1	7.766738848	7.737346818	7.280525364	8.063008152	7.561342071
GZMA	11.13085117	11.44643795	12.24933389	12.24933389	12.16627503
GZMB	11.1060681	11.02403513	11.27306561	11.88909817	11.6157426
GZMH	10.68338253	11.07337622	12.31719428	11.85033795	12.21991364
GZMK	9.505258828	9.538964939	10.73911267	9.939327313	10.34311059
GZMM	9.283145788	9.458377051	9.722667242	10.31894523	10.20164867
H1F0	6.642822768	6.680176879	6.329524818	6.510509061	7.552833101
H1FX	7.626793707	8.011239071	7.002156515	8.644542606	8.140977576
H2AFX	9.50650501	9.603529667	9.707282667	9.550085313	9.744663444
H2AFY	11.85617239	11.13240418	11.87175588	11.55106122	11.36979611
H2AFY2	6.630903202	7.095534354	6.038814121	6.189422515	6.227503758
H2BFXP	7.019697232	6.757183747	6.897441	6.55251301	6.449932949
H3F3A	16.49551516	15.39581235	15.90458538	16.3077943	16.14978837
H3F3B	14.61202705	13.73439002	14.59173737	14.21449649	13.78737352
HACL1	8.98371896	9.556000646	10.156858	9.433849505	9.458377051

HAGH	12.56315114	13.61616406	12.40343679	12.81735602	13.11515098
HAGHL	7.479422939	8.556357949	7.19681397	7.64232702	7.555333263
HAL	8.627322343	7.423727141	7.781852747	7.801818071	7.581254909
HAPLN4	6.270128909	5.969211687	6.515007141	6.22946	6.194928222
HAR1A	7.978709162	8.495994869	7.652952818	8.00165403	7.549884232
HAT1	10.05144087	10.06547375	10.12041459	10.0719806	10.17440526
HBD	16.12384164	16.09492117	16.22759729	15.65613201	16.7804214
HBE1	7.612909768	7.422534091	8.07651197	7.179445253	7.474397404
HBG1	17.86179439	16.90924948	17.86179439	15.81050193	16.8710039
HBM	15.58150095	16.39933956	16.73234105	15.24813401	16.33688632
HBP1	8.57971698	8.189194404	9.179819687	8.746311293	8.479727162
HBQ1	17.1822602	17.1822602	17.1822602	17.1822602	17.1822602
HBZ	9.877792838	9.886023253	9.424273283	6.982654747	6.709290939
HCCA2	9.703591263	10.18198959	8.913588253	10.05688349	10.09426568
HCFC2	7.082435283	7.452739495	6.977041091	7.060794313	7.222444626
HCG26	7.666604434	7.826840444	7.692552364	7.294583061	7.622739535
HCG27	9.366312899	8.740682	8.733877859	8.919862162	8.676316919
HCG8	7.787223354	6.583409828	7.292561596	7.137147091	6.984574354
HCK	12.03912361	10.89912578	10.56802411	11.90384839	10.94071069
HCLS1	15.88528111	14.36999747	15.78787594	15.02501841	14.76275492
HCN2	17.67797782	17.86179439	16.2036826	17.67797782	17.86179439
HCN4	16.64557528	17.36803328	14.56207529	16.28343863	16.57174008
HCRT	12.31419772	12.3543152	12.01191659	12.19086998	11.92156179
HCRTR1	8.752460404	8.496365737	8.645985424	8.444861909	9.317615606
HDAC10	8.296772869	7.840676768	7.351625758	8.376150616	7.959080606
HDAC3	7.336476646	7.076844636	8.115437899	7.436640343	7.082160636
HDAC5	9.797856576	9.281446566	8.756396081	9.46096901	9.396536798
HDC	10.09795649	9.671170444	10.49241362	9.197870838	8.460053242
HDGF	10.32635032	10.92337564	11.21958266	10.9102587	11.53473316
HEATR2	6.680176879	6.575018	7.538797101	7.928481586	7.934739636
HEBP1	12.61761735	11.34576518	11.49420288	12.04734694	11.33292491
HEBP2	12.28693608	11.26623333	11.93975968	11.14385742	11.69188536
HELB	6.810307121	6.839237455	7.70299303	6.843295	6.967457677
HELZ	8.57597697	8.554528737	7.356542485	8.372086737	8.138908141
HEMGN	9.576740677	10.08893073	10.59418919	10.56655729	11.0035084
HEPACAM2	6.792041707	7.545335788	7.350971899	6.618269616	7.54731098
HERC2P2	7.405823253	7.618249414	8.144908747	7.464704768	7.663954061
HERC5	7.916025434	7.81867298	7.924502232	7.264798293	7.764609818
HES4	10.78705857	12.43944324	10.38531322	10.56548536	11.11714857
HES7	17.43906183	17.67797782	16.82803465	17.01900663	17.12376032
HEXB	9.917863333	9.742895778	10.82416773	9.999132495	10.06450841
HEXDC	8.319901444	8.366948404	7.358963616	8.443175586	8.434412929
HEXIM2	9.859664869	9.965319232	10.35809493	10.02362526	10.20964764
HEY1	8.213079374	6.937104768	6.48058603	6.817048798	6.908859909
HGC6.3	7.889031475	8.722953263	7.005077596	7.426469556	8.324880909
HGD	8.31008898	9.487680697	10.52689599	8.639417101	9.095162899
HGFAC	7.020179424	7.106302455	7.316406697	7.048729333	7.840343939
HIF1A	9.712341424	9.373397364	8.460053242	9.508442768	9.237064687
HIG2	7.060794313	7.509397909	7.761378475	7.122942091	6.965960869
HINT1	11.07797425	11.49239347	12.41033676	11.72355631	11.89130603
HINT3	6.982909788	6.731175838	7.422831253	6.861656576	6.825325
HIP1R	8.380307172	8.62412801	8.438712657	8.507211606	8.591651626
HIPK1	9.09013302	9.283736939	8.244805758	8.726024485	8.876209859
HIRA	8.363468485	7.459115707	8.710842768	7.241538444	7.511581475
HISPPD1	9.381544081	9.492465394	9.101086141	9.070661273	8.57070398
HIST1H1C	12.4571909	11.5737214	11.66441291	11.84736958	11.29744903
HIST1H1D	8.516311424	8.508645727	8.604635949	8.771856424	9.20200202
HIST1H1E	8.480158333	7.741457929	8.387507626	8.277405556	7.939903758

HIST1H2AD	12.50706636	11.68948846	11.89361272	12.10135206	11.43565115
HIST1H2AE	9.499917182	9.588958727	10.27804811	10.04862183	9.356058495
HIST1H2AH	10.36465285	10.71860391	11.33100665	10.82290783	10.67190595
HIST1H2AJ	9.199527535	8.926362727	9.122138505	8.818695101	8.584830636
HIST1H2BI	9.193130899	8.680513909	9.410728505	9.189045596	8.590695768
HIST1H2BK	10.27338352	10.16454544	10.66465811	10.31289083	10.3305428
HIST1H2BM	9.240082808	8.586731747	9.706432808	9.323195364	8.813032152
HIST1H3A	13.70143341	13.0147344	13.50411017	12.60022648	12.52461444
HIST1H3B	9.644739414	9.425521616	10.08521343	9.702015293	9.323195364
HIST1H3C	13.29684189	12.29275278	13.66784462	12.73352188	12.38794592
HIST1H3D	9.180285111	8.480158333	9.077100232	8.955359253	8.578393657
HIST1H3E	13.38447499	13.02518615	13.14093405	13.04381444	12.89886333
HIST1H3G	10.82804231	10.23452156	11.238428	10.43332542	10.08231743
HIST1H3H	8.24122704	8.835019636	8.462236515	8.708030737	8.397853727
HIST1H3I	9.815129384	9.227275152	9.644739414	9.298556212	9.714566535
HIST1H3J	9.338458455	9.362770111	8.841037687	7.858214495	8.985325394
HIST1H4B	9.658772677	10.40178901	10.36239309	9.933588354	10.38738452
HIST1H4C	12.95787308	13.42153085	13.76702458	13.5964752	13.48401144
HIST1H4E	6.33568397	6.718256293	6.862895808	6.802568323	6.734736768
HIST1H4H	8.049019253	7.443192556	8.52305899	8.563085788	7.905419798
HIST1H4L	10.5199714	11.47871073	11.47127063	11.11714857	11.41071085
HIST2H2AA4	12.40735915	11.65455063	10.72246175	11.59427705	10.94208941
HIST2H3A	11.69188536	11.05356995	11.31267384	11.27153548	11.01376888
HIST2H3D	13.42888134	12.33907422	13.93694444	13.14611959	12.83405786
HIST3H2A	11.16281786	10.82290783	11.03296099	10.78705857	10.31480319
HIST3H3	15.18392271	14.52085241	15.88528111	15.31193978	15.15443825
HIVEP2	10.1905069	10.53623255	10.50057506	10.67815746	10.56908161
HK2	9.184527788	8.434806414	8.213079374	8.327767232	8.621272737
HK3	10.3090972	9.461673394	8.177906263	10.10365156	9.329922273
HLA-A	13.92894576	13.09463792	13.40302459	14.40789433	13.64789782
HLA-B	16.3077943	14.84412351	14.5513462	16.22759729	15.13975511
HLA-DMA	9.066605263	9.485668061	9.267923343	9.360502949	9.12042397
HLA-DMB	8.824851909	8.980943495	9.930141081	9.19736601	9.046362323
HLA-DPA1	10.27714677	10.71390019	10.73014715	10.79145221	10.32635032
HLA-DPB1	12.62449502	12.92542832	13.44126502	13.14093405	12.55625498
HLA-DQA1	7.17606597	7.245884828	7.021219939	6.935285707	7.186679828
HLA-DQA2	7.466913717	7.600942677	7.132449434	7.21466303	7.811684818
HLA-DQB1	6.900739596	7.737649848	8.029836697	7.54886499	6.549824202
HLA-DQB2	8.205892515	9.711723394	10.33596201	9.015412101	8.234636465
HLA-DRA	10.53505365	11.43380494	12.12692782	11.8164662	11.71895032
HLA-DRB1	12.33273925	12.7939548	13.12562627	13.16824615	12.41337695
HLA-DRB3	9.719830172	10.21157382	11.05191968	10.66587479	10.16642595
HLA-DRB4	10.30810853	10.10454015	10.13276962	11.12935449	10.94786936
HLA-DRB5	10.85100254	11.9577766	11.9091429	11.70138789	11.50425191
HLA-E	16.06827159	14.88238784	15.03840752	16.36806566	15.18392271
HLA-F	15.10875812	14.30038792	14.68633385	15.61926687	14.6970922
HLA-H	15.78787594	14.42673578	14.19920898	15.90458538	14.80749806
HLA-J	16.68963031	15.32551587	15.63778652	16.8710039	16.12384164
HLX	9.112697707	8.301657889	7.015344242	8.919352101	8.19458801
HM13	8.300483646	7.850890354	7.673376626	8.751946515	8.276867707
HMBOX1	8.658285303	9.053706202	7.008714091	8.391869283	8.570262
HMBS	11.34892675	12.42616911	11.59021288	11.31852116	11.84291489
HMCN2	8.019434586	7.772177253	7.562627657	7.576037556	8.184399818
HMGB1L1	11.28896545	11.07493153	11.81439214	11.87912023	11.83072395
HMGB2	13.67554111	11.82852227	12.57486869	12.41033676	12.33907422
HMGB4	8.881310737	8.851571051	9.054276	8.27861104	9.109760465
HMGN1	10.02362526	9.66613299	10.65987528	10.30569114	9.819688798
HMGN3	7.44112097	7.64232702	8.170954818	7.750590293	7.968691808

HMGN4	9.601568657	9.366909111	10.76361324	9.625041525	9.399129424
HMOX1	11.48061491	11.44491584	12.32964397	10.42512206	10.69222123
HMX2	10.64738653	11.36245739	10.4655823	10.40178901	11.20498985
HN1	11.90674449	10.87907816	11.66189432	11.36245739	11.51260306
HNMT	6.883278424	7.135222657	7.590558081	7.209785606	7.13133802
HNRNPA1	10.31480319	10.78269205	11.48061491	11.21338774	11.28207759
HNRNPA1L2	10.02644328	10.35385551	11.12001247	10.69682519	10.76239578
HNRNPAB	6.458058747	6.473835768	7.689234404	6.790317606	6.806487737
HNRNPF	10.1309568	10.20859114	10.98627861	10.64623997	10.80782516
HNRNPH1	9.236429667	9.507772687	9.306330646	9.545881535	9.682548525
HNRNPL	7.668638899	8.005065495	6.477739737	7.693213707	6.971796172
HNRNPR	9.447242061	9.612498475	10.10278369	9.795448596	9.779738
HNRPDL	9.554043101	9.91449603	10.20452777	9.934615404	10.18762619
HNRPLL	6.933815889	7.08160301	8.227270172	7.337386909	7.300330596
HOMER3	12.26775966	11.84736958	11.39185194	11.86401838	11.49239347
HOPX	7.823083152	8.073652495	8.895503556	8.324880909	8.708995576
HOXB2	7.502729737	7.972012263	7.898205273	7.753435232	7.240600646
HP	11.38025327	10.32418729	11.12151875	10.41123643	9.913634949
HPCAL1	9.693515768	9.173615515	8.404396899	9.663106646	9.218466939
HPR	7.474397404	6.629982465	7.192707293	6.877148152	6.308110818
HPS1	8.912452162	9.852239606	9.158666485	8.950874253	10.28795108
HPSE	7.537817576	6.880775747	7.790242404	7.119081899	7.522869182
HPX-2	7.868007081	6.286707364	6.514109778	7.144617717	6.378965828
HRASLS	7.496977949	7.214922364	8.08737399	7.143440162	6.74585002
HRASLS5	10.01168912	9.596088263	9.904237354	10.20634509	9.872596081
HRC	8.136235949	7.812327374	7.92031953	7.171589687	7.569223354
HRH2	7.131581374	6.861656576	6.856233343	7.023397576	7.107390485
HRH3	7.624735818	7.504556444	7.736964596	7.50339202	7.30556202
HRH4	5.89725504	6.076308061	6.18159802	5.823899061	5.763886222
HRK	9.093526566	10.04421951	8.62460002	8.541972566	8.668062838
HSCB	6.650544455	7.030426444	7.503692152	6.894957566	7.071771354
HSD17B11	12.17829278	11.71244686	12.30167305	12.43600068	12.18492446
HSD17B14	6.254184596	6.445674283	6.57397498	6.696473434	6.592293788
HSD17B4	8.503455465	8.52575703	9.517771202	9.109760465	8.931557869
HSF4	6.984849141	6.741974778	7.066488111	7.075225141	7.373983485
HSH2D	11.75462574	11.07158908	12.06964699	12.03310273	11.56725755
HSP90AA1	10.97185531	11.09721747	11.79870316	11.71244686	11.74439188
HSP90AB3P	6.561305909	6.801007778	7.004539727	7.080799364	7.364797848
HSP90B1	10.96162926	11.79615897	11.36245739	11.3290504	11.70812672
HSPA1A	11.36979611	10.04600105	11.05033578	11.1060681	10.28710845
HSPA5	8.318250424	8.439112919	8.962104076	8.309668384	8.383429707
HSPA6	13.03496614	11.66708917	11.38970236	12.31419772	11.70348036
HSPB1	9.179226364	9.927686616	9.010430586	9.182731869	9.368875727
HSPB6	6.707366768	6.631857323	6.907839071	6.57823699	7.132177788
HSPB9	7.676257364	8.734806657	6.915952788	6.939171505	7.449265303
HSPBAP1	9.639623879	9.50650501	10.41224321	9.283736939	9.724047525
HSPC159	9.445852485	9.491725303	9.826032111	9.923658606	9.604175636
HSPD1P6	7.538797101	7.768055657	8.79576798	8.595283071	8.871209182
HSPE1	10.7982674	11.33848921	11.32185019	11.35475345	11.69658017
HSPH1	9.842388081	10.01071956	9.76546198	9.756840071	10.24511785
HTATSF1	9.361671283	9.670530667	10.07764109	10.03796113	9.890135131
HTR1B	7.94734596	6.909148313	8.012346152	7.90609097	8.104866828
HTRA1	6.669224869	6.982654747	7.514494576	6.853717071	6.560197424
HTT	7.882189879	8.145617202	7.785596848	8.454954939	8.161911788
HUMBINDC	8.887109626	8.016751283	9.771430818	10.08978265	10.73132595
HYAL2	8.302062758	7.770089636	7.912742707	7.989587677	8.613130333
HYPK	10.90192126	11.24993851	11.72592113	11.12454893	11.28565422
IARS	7.475616394	8.031407364	8.417604414	7.619556354	7.974325596

ICA1	7.033114263	6.325549091	7.727011939	6.783595586	7.577325354
ICAM3	15.24813401	14.26676228	13.70717091	15.449005	14.59173737
ICAM4	7.520992313	7.540776061	7.552094283	7.337989111	7.427372788
ICOS	8.005371424	8.141770949	8.581169111	7.740855141	7.837132667
ICT1	9.656571869	10.23160947	10.48659409	10.02161965	10.24414187
IDH2	8.088956818	7.942071303	7.595597162	8.582147707	8.237787788
IDI1	8.915814212	8.469779808	9.783680727	9.296131263	9.152615889
IDO1	6.635568162	7.334908263	8.456593071	7.073657889	7.258693172
IER2	14.94534759	13.97489728	14.91973608	14.62217632	14.5108222
IER3	8.988577162	8.472435657	8.992957212	8.218433131	8.666113404
IER5	13.05428976	13.70143341	11.5571076	12.80602179	12.96241124
IER5L	8.922632727	8.803482152	8.382194192	8.975838535	8.838951081
IFFO2	8.85785699	9.804809051	9.416442616	9.559682182	10.07107569
IFI16	11.40145091	10.8935809	11.24993851	11.08936677	11.565007
IFI27	8.90531298	9.80784303	8.939019091	8.061425949	9.732604394
IFI30	14.223913	14.24162707	14.83117585	14.19920898	14.23242051
IFI35	8.527198758	8.152794889	8.335469222	8.363019899	8.354182626
IFI44	8.947623838	8.905887879	10.22448985	9.132651727	9.344897333
IFI44L	7.902229798	7.604463141	8.526649808	7.728706424	7.933961929
IFI6	9.804037697	9.161011869	8.406205495	9.137887434	9.746868596
IFIH1	8.479185606	8.826846768	9.203760495	8.671783687	8.980943495
IFIT1	11.26446827	10.80239406	11.49839615	10.50166798	11.10170724
IFIT2	10.96460614	10.52247938	10.59809845	10.5152666	10.81142627
IFIT3	9.701165495	9.110297333	10.03205964	9.572453101	9.591816061
IFIT5	8.903602263	8.43615796	8.266842545	8.424471616	8.734806657
IFITM1	13.97489728	13.24400831	13.89331098	13.67554111	13.50997822
IFITM2	14.84412351	13.56886427	13.35919327	14.72870348	13.92894576
IFITM3	14.26676228	13.20536123	12.81379045	13.84455043	13.34204398
IFITM4P	12.69676846	11.5571076	10.86566339	12.39348455	11.68948846
IFITM5	15.83165575	16.73234105	12.75784116	15.09418019	15.70163214
IFNA4	6.97809596	6.304241131	6.545751121	6.824533111	6.372158566
IFNAR1	8.917069505	8.732044667	8.571194222	8.647073404	8.906910121
IFNAR2	6.641155141	6.772514131	7.503050071	7.488608758	7.615901626
IFNGR1	13.18348162	12.34237091	11.84291489	12.51066587	12.46055504
IFRD1	10.51886057	9.62845904	9.86640601	9.843921515	9.741357455
IFRD2	8.721054424	9.234418071	8.848914293	8.91530399	9.290785283
IGF2	8.630379313	8.702074263	8.592622071	8.334981101	8.568242293
IGF2AS	8.476994374	7.421647414	8.169730152	7.628690051	7.858964434
IGF2BP2	7.694916828	8.375190152	7.731542758	7.724369343	8.102484232
IGF2BP3	8.546982556	8.70424704	8.72650097	8.696280707	8.495994869
IGF2R	11.238428	9.698181576	10.25127201	10.85513517	10.24316974
IGFBP2	8.687969051	10.0178996	10.09426568	7.957321232	7.688949081
IGFBP7	9.228437333	9.019287687	9.042944374	9.335272182	9.090721626
IGH@	9.689129545	9.230202616	9.895061889	9.70034896	9.536330707
IGHA1	7.572084505	7.049507061	7.321884212	7.51310204	7.210358909
IGHD	7.749299101	7.606699253	7.476910626	7.514105485	7.591751808
IGHG1	7.170007293	6.369108576	6.508699242	6.938620788	6.470357717
IGHM	10.76609029	10.61371787	8.978204141	10.87637444	10.4994288
IGHV1-69	7.56320098	7.294038515	7.336476646	7.866306808	7.084738172
IGJ	9.324388263	9.18155302	9.417037424	9.972781707	9.407554697
IGK@	7.717096556	7.401460495	7.457359414	7.596851556	7.384903253
IGKC	7.892277636	8.32939098	7.626470505	7.612294131	7.874063444
IGKV1-5	7.553068374	7.222163485	7.427086202	8.024267525	7.264510162
IGKV2-24	7.32338401	7.033625222	7.480656747	7.967524909	8.221010253
IGLL1	8.825911939	8.252794354	8.263464364	8.562664263	8.422204465
IGLL3	9.832711535	9.499917182	9.503858424	9.348136535	9.565660949
IGSF6	10.79686873	9.797856576	9.513795939	10.32946621	10.06173934
IGSF9	7.28082202	6.426421657	6.804242869	7.229046354	7.690510313

IKBKE	6.283913465	6.630696545	5.762362404	6.816280253	6.782198505
IKBKG	10.12041459	9.695719162	8.729485838	9.526870273	9.479084687
IKIP	8.816214848	8.412145273	8.780092131	8.457043091	8.262596626
IL12RB1	8.222994222	7.999327475	7.777311636	7.98690202	8.049019253
IL13RA1	10.86963124	10.19757035	10.8405808	10.21157382	10.33890802
IL15	8.027386222	8.801904485	8.921533879	8.703161646	8.135526051
IL17F	6.939713242	6.444835424	6.949335212	6.557897323	6.997069465
IL17RA	13.01016522	11.99535117	11.57567047	12.60396528	12.44264271
IL17RE	8.230588212	7.953486636	7.296570525	7.60255999	7.985458788
IL18R1	7.534302667	6.564717364	7.325162263	6.93243001	7.008416879
IL18RAP	13.6823979	11.36065784	12.08943903	12.61090773	12.06692054
IL1B	11.36793803	10.59180142	10.70775781	10.98186414	11.05651949
IL1R2	11.58814301	10.16546821	10.58117749	10.64379219	10.64738653
IL1RAP	7.524403394	7.655611	6.875236313	7.344596535	7.189410667
IL21R	7.966054535	8.63611798	8.990220404	8.447076788	8.562664263
IL23A	8.565346707	9.287819051	9.588958727	9.041820404	9.193817232
IL27	10.20859114	8.524867051	10.76857843	8.529417101	11.89361272
IL28A	7.214035101	7.158304313	7.022843394	7.779426687	8.127495596
IL2RB	9.231946182	9.674270303	10.45375986	10.21975253	9.459664354
IL32	9.262561	9.961780586	8.606099333	9.831087364	9.709426838
IL3RA	8.01013803	8.818695101	9.012031232	9.337862091	8.504327141
IL4	6.397996646	6.410292616	6.389892586	6.566085596	5.899000414
IL4I1	7.642608182	8.463578343	8.08075902	7.557268586	7.52469401
IL4R	14.01682491	12.72972704	13.5964752	13.50997822	13.71423947
IL5RA	6.128153374	5.948711596	6.674468929	6.622911394	6.696473434
IL6R	12.36759935	11.54871614	12.01673563	12.40735915	11.97196754
IL7R	11.11230451	11.54470354	10.55602586	11.49029795	12.14507673
IL8	7.179153636	8.002011657	6.784856394	6.580819444	7.274888343
IL8RA	11.94481318	10.70299608	10.49009312	11.46320141	11.00183361
IL8RB	11.63174178	10.09994749	10.2166926	11.10761793	10.53623255
IL8RBP	12.80166365	11.45932673	11.15966862	12.37416009	11.78265022
ILDR1	14.37933382	13.42888134	15.41210544	15.32551587	14.9847254
ILK	9.45706402	9.049629657	8.935320828	9.980047091	9.310775172
ILKAP	7.287539616	7.23423804	8.292068253	7.477539293	7.486746556
ILVBL	8.565878434	8.619347838	8.567801737	8.761177727	8.732044667
IMPA2	12.34855768	11.45526933	11.33848921	11.82852227	11.51008986
IMPG2	7.269324172	6.629005263	6.529968303	6.497644434	7.117401788
INE1	9.062220818	8.843148071	9.657223606	9.080683394	9.365016101
INHBB	7.252299788	6.005792444	6.440187172	6.639045768	6.14215451
INHBC	7.819838162	7.480029465	7.586125333	7.634532263	7.539487212
INHBE	6.782643404	6.486059364	6.705162535	6.664178717	6.735177838
INPPL1	9.310205828	8.740195303	7.629344343	9.128533354	8.358783152
INTS3	7.714642404	7.263859848	6.296876485	7.413750677	7.098240939
IPO11	7.482605354	6.933815889	7.926719768	7.633233848	7.890057202
IPP	6.893606717	7.074173121	7.391952354	7.142620404	7.143440162
IPPK	7.415013667	7.636934919	6.713757434	7.195502081	7.195224778
IQCB1	8.091323071	8.48742196	8.248137606	8.054318061	7.791533949
IQGAP1	11.14211434	10.573928	10.73384189	10.80900786	11.05356995
IQGAP2	10.09682592	9.62024901	9.084401768	10.00186072	9.747577162
IQGAP3	7.660795616	8.167813576	7.832540091	7.972411364	8.213079374
IQWD1	9.04056096	9.746145949	9.606954152	9.276189121	10.02161965
IRAK1	11.3733012	11.3403052	10.59685822	10.3986043	11.18291631
IRAK3	8.636577313	7.701991131	8.628622212	7.84876403	7.933573889
IRF1	11.66934136	10.98037262	10.51886057	11.42178201	10.69793218
IRF2BP1	8.168969465	8.65914896	7.920877394	8.991970091	9.651465606
IRF3	9.488337717	9.497084828	9.821914929	9.870931818	10.26332497
IRF5	8.57070398	8.713417869	8.250194202	8.578393657	8.292068253
IRF7	9.732604394	9.256085253	9.21067496	9.26122399	9.382882596

IRF8	6.472782788	6.728447091	7.205228253	6.688196	6.540677515
IRGC	7.780116303	6.627087081	7.327495141	8.043387838	7.727729838
IRX3	9.596773313	10.3810842	7.856753939	8.497259596	8.90531298
IRX4	7.712213273	8.537956091	6.86313897	7.276277889	7.614831859
IRX5	6.553214606	6.564307798	5.999131566	6.547344253	6.348225889
ISCA1	7.335252788	7.585232141	8.084674061	7.262654505	7.979863071
ISCA2	10.1597097	10.28899471	10.69354222	9.617464455	9.95201103
ISCU	12.39348455	12.02514953	12.53563192	12.63102018	12.797444
ISG15	11.4090051	10.6426412	11.77552743	11.27695531	11.55524693
ISG20	14.91973608	14.19920898	15.15443825	14.30038792	14.15092087
ISLR	7.969793182	7.538797101	7.478449101	7.262118616	8.005065495
ISLR2	6.536206333	5.873988283	7.163287778	6.50824402	6.089123677
ISOC1	9.020337111	8.177906263	9.11154599	8.546545061	8.699656879
ISOC2	7.118512586	7.395598354	6.619037364	7.149343495	6.861163889
ITGA2B	8.752912545	10.07580535	8.593558293	9.270407879	8.651452677
ITGA5	8.618800343	8.131433848	7.71023803	8.32400903	7.925310818
ITGA6	8.149448323	9.051375475	8.524867051	8.108917222	8.657372182
ITGAL	9.800083455	8.965143333	8.73262002	10.10946026	9.690534253
ITGAM	11.37490296	10.68072859	11.40495249	10.85100254	10.72127155
ITGAX	11.22456459	9.861430101	10.23247836	10.05523038	9.957374697
ITGB1	8.287888051	8.895503556	9.417594202	9.136296333	8.988577162
ITGB1BP1	8.704708384	8.710391727	9.379720636	8.47014499	8.966282283
ITGB2	14.25895571	13.52959744	13.5360606	13.83772389	13.64083669
ITGB3	6.821773354	7.928832091	7.691535778	7.627373273	6.869371495
ITGB3BP	7.420173404	7.943747505	7.601304687	7.836138323	7.358375414
ITGB5	7.67441401	8.250194202	8.34368697	8.801904485	7.554702939
ITGB7	11.50632759	12.18492446	11.88375452	12.25572441	12.29275278
ITGBL1	6.671548495	5.834101232	5.890178222	7.479119	6.256334263
ITK	7.780823677	8.450171172	8.655934636	8.140538697	8.320357909
ITLN1	8.240781051	9.190767364	10.18198959	8.497692788	9.535633242
ITM2A	8.31049196	9.116279576	9.349388778	9.063885091	9.449948384
ITM2B	13.35919327	12.77015535	11.75662781	12.47441306	12.42616911
ITM2C	7.087210828	7.364797848	6.844259293	7.299074768	7.403028737
ITPKB	9.828404283	9.06333597	9.208914778	9.690534253	9.867911152
ITPR2	7.228468091	6.951961606	7.541750556	6.917518081	6.921167192
ITPR3	8.263866778	8.529417101	8.477856384	8.431738505	8.417196576
ITPRIP	11.57765358	10.74045328	10.573928	10.8443678	10.55726192
IYD	6.326292525	6.008751121	5.970832818	6.252603323	6.981850818
JAK3	10.69099899	10.43998577	10.04421951	10.17702971	10.56548536
JAKMIP1	8.370422404	8.485631939	9.392652323	8.037287293	8.586230848
JARID1D	7.800624061	8.17219503	6.01168199	7.761029182	5.965621172
JAZF1	8.772410586	9.156840283	8.145962667	8.07651197	9.294355394
JDP2	11.99535117	11.13713447	10.53623255	11.19817469	11.21016953
JMJD2A	7.266813283	7.172125253	7.246141232	7.289911515	7.488273475
JMJD3	7.327857495	6.677368899	5.768664949	6.495011879	6.276608434
JOSD2	8.638487899	9.140392293	8.58807297	8.309247939	8.713417869
JUN	8.957404525	8.033283465	8.487847515	8.393536232	8.125376242
JUNB	11.04886125	9.94402302	9.917043434	10.61992902	10.32093584
JUND	12.37416009	13.03496614	12.77015535	13.22156044	13.38447499
JUP	6.751729354	6.928572354	7.216973434	6.786766677	6.75400198
KANK1	6.936369818	7.325732596	7.834378071	6.458735293	7.063222061
KANK2	6.97994296	7.454874889	7.421037465	6.965960869	7.701686677
KARS	7.207790596	7.373983485	8.523456192	8.032957859	7.825440475
KAT2B	8.571194222	9.129654758	9.193817232	9.023071899	9.894213939
KBTBD11	11.11388317	10.59292728	11.01951417	10.58347296	10.31193963
KBTBD5	6.171325172	6.102491535	6.032452212	6.556698131	6.562194212
KBTBD7	8.498167798	8.242002535	8.636577313	8.314030071	8.061773232
KCNC3	7.276277889	6.585712788	7.047046253	7.013137848	6.501455758

KCNE3	10.73014715	10.02741959	10.65871503	10.25627793	9.767642525
KCNG1	7.371907727	7.476910626	7.150454879	7.084441778	7.886079293
KCNH2	6.883793818	7.229587778	6.722265657	7.026391172	7.756512818
KCNH4	6.377717758	6.295706747	7.219623727	7.007889061	6.962034242
KCNH8	6.302130162	6.662501485	6.007701727	6.185014717	6.396397444
KCNJ15	11.69891546	10.62280049	11.59870577	11.5386618	10.99469779
KCNJ2	11.87175588	10.80782516	10.57028524	10.5199714	10.48782122
KCNK1	5.779733556	6.449508273	5.759283646	5.987385263	6.538425424
KCNK15	8.655437626	9.217871889	8.31008898	8.277763646	8.576430182
KCNK4	6.918844495	6.838435556	7.312252182	6.82246696	6.432015545
KCNK7	12.25251148	13.96779242	12.0838996	12.75396286	13.32669092
KCNMB4	7.116047475	6.698080333	6.981618121	6.675450515	6.500607838
KCNN2	6.748324394	6.205248909	6.866852303	6.521008828	6.994029394
KCNN4	9.291911616	9.538263677	9.079484556	9.096947232	9.215289101
KCNQ1	11.04030995	11.25700815	11.0387546	11.00053539	10.74305691
KCNQ3	12.12939166	12.39973853	11.34576518	10.67572435	11.69891546
KCTD11	8.453185455	7.704350384	7.545986354	8.394903626	8.500240172
KCTD12	8.753927596	8.971160737	8.402148465	8.670447949	8.48742196
KDELR1	8.009440313	8.069395848	7.760294414	8.187968455	7.838173152
KEL	6.434266404	7.237499505	5.906592949	6.521458212	6.594827192
KHDRBS3	6.800063838	7.450888848	6.567922788	6.290280232	6.55006301
KIAA0101	7.089056525	7.328126596	6.913511828	7.072297535	7.100441616
KIAA0125	6.387713778	6.738778758	6.507609283	6.460273586	6.386847838
KIAA0146	7.737346818	7.432999727	8.138156535	7.857845061	7.523206263
KIAA0240	10.74819911	10.6488688	9.634173323	10.2829717	10.29000276
KIAA0247	12.13867659	11.51647662	11.9982543	11.91651973	11.51647662
KIAA0317	8.508645727	7.883157929	8.26142704	7.800624061	8.052077899
KIAA0319L	8.836542848	8.064713051	7.797507939	8.541524636	8.055916879
KIAA0329	11.43380494	10.52585606	11.06261544	10.50677257	10.70775781
KIAA0355	9.451212687	9.713858919	9.636296737	9.224923687	9.53973704
KIAA0406	8.011239071	7.506719576	8.53579602	8.163923192	8.498949848
KIAA0427	8.691979949	8.373514202	9.072403576	8.691979949	9.060618192
KIAA0485	6.829579545	7.067324051	6.221954697	6.702042636	6.900739596
KIAA0652	7.462491444	7.205488778	6.795634141	7.629673333	7.218399283
KIAA0746	9.393335687	9.67665103	9.595333525	9.46304199	9.905108616
KIAA0748	7.273826505	7.914251515	7.448962859	7.601304687	7.648986848
KIAA0892	9.150235333	8.667554273	8.447076788	8.887109626	9.233272283
KIAA1257	8.324880909	7.846992646	8.607126586	7.959080606	7.885378
KIAA1267	10.57649171	10.46232362	9.168324747	10.09795649	10.0079343
KIAA1303	8.044980354	7.954533778	7.850535525	7.826151263	8.274284293
KIAA1324	10.41957053	9.267923343	8.290353162	8.215082485	9.014335657
KIAA1466	8.172571899	7.346859747	7.990313949	8.101780545	7.726392606
KIAA1539	8.166983455	8.788135667	8.061012707	8.01013803	8.333749495
KIAA1545	11.64044206	12.78645268	9.378583414	10.81796524	11.39983314
KIAA1598	6.618022394	6.899234475	6.827661505	7.059356717	6.691055667
KIAA1602	9.868690293	9.848227263	9.730279475	9.588410576	9.556759333
KIAA1618	9.407554697	9.003925646	9.857104919	9.606954152	9.738398616
KIAA1627	7.506434626	7.70299303	8.370422404	7.544047919	7.816627253
KIAA1652	8.918202434	9.206631485	9.853774707	9.373397364	9.39974104
KIAA1683	9.553430788	9.533596364	10.22865659	9.779738	8.43615796
KIAA1737	8.240375404	7.975072535	8.210186616	7.941320778	7.951633808
KIAA1875	8.148308414	8.073294879	8.718321818	8.049757475	8.375190152
KIF17	6.963095333	6.408811828	6.989960667	6.812677121	6.956897717
KIF1A	6.683324051	6.293534101	6.444123263	6.599487732	6.874988444
KIF1C	11.16734497	12.52130815	11.04156144	12.03068847	12.47441306
KIF21B	9.886023253	9.046862848	8.626833051	10.06071544	9.247858869
KIF22	7.009988212	7.209785606	6.940218677	7.72538904	7.559825556
KIF3A	6.742510061	6.693002899	7.582966818	7.304428303	7.330801667

KIF5C	6.697842384	6.869634889	6.34228097	6.851123798	7.18421398
KIFC2	8.253254586	9.275532697	7.70365599	8.431326525	8.716387192
KIFC3	8.373514202	7.493254434	8.050484323	7.859293	8.641737071
KIR2DL2	7.354804152	6.761831111	6.846305687	7.300031424	6.421771838
KIR2DL4	8.592161788	8.25927404	8.013487535	8.433042727	8.503920141
KIR2DS2	7.245014414	6.818245747	6.404620899	7.653305051	6.210980111
KIR3DL3	6.245450939	5.964641646	6.714232707	6.219076051	5.949274
KIRREL3	7.413750677	7.155827141	7.620680879	7.315524667	7.659822475
KISS1R	11.4535969	13.1944969	9.958247111	11.33100665	10.98627861
KLC2	7.026621727	6.764835354	6.569841374	6.858687869	7.164674212
KLF1	7.29687102	8.056301667	7.423727141	7.375117525	7.647332323
KLF10	7.224144889	7.560760293	7.684267263	7.735250384	7.719835253
KLF12	8.460053242	9.071276071	9.151976485	9.263233333	9.369545939
KLF14	11.91416468	12.63102018	11.23641801	11.07647857	11.97944932
KLF16	10.55070525	11.23641801	10.20964764	10.1838089	10.29660096
KLF6	12.79024082	11.9875501	13.2158469	12.93430142	12.42943771
KLF7	7.341339747	6.55365803	7.07857001	6.989443677	6.879229626
KLF9	9.767642525	9.761765848	10.24139873	10.29208493	10.41022215
KLHDC7A	8.689422596	9.689129545	7.854005263	7.508388697	8.673843717
KLHDC8A	6.295918879	7.222444626	6.365312818	6.550562869	6.527644212
KLHDC8B	8.034450929	7.193007566	6.977525061	7.262427071	7.073400869
KLHL17	8.546056747	7.398977253	8.597538556	8.219387636	8.639417101
KLHL2	9.284395657	8.381309202	8.301264212	7.87816304	8.019434586
KLHL25	8.381309202	8.543686687	8.439612798	8.360024717	8.53670504
KLHL33	7.205228253	6.594580697	6.94072599	7.874063444	6.842504626
KLHL6	8.163923192	8.297630758	9.099242404	7.995673758	8.284505182
KLHL8	7.987573434	7.639662323	7.939903758	7.845242687	7.948048111
KLK14	8.328992859	8.398231626	8.221794202	8.154321646	8.59655801
KLK3	9.447991384	9.808672222	8.236633323	9.066605263	9.416442616
KLK9	7.944058707	7.950135394	7.755825222	7.886079293	8.150448232
KLKB1	7.945864808	7.577683121	8.94874	9.280305798	9.684886576
KLRB1	10.26729954	10.11349335	9.625041525	11.04316622	10.89604789
KLRC1	7.525550222	6.946711626	7.203788636	8.269395232	7.317590788
KLRC2	7.363617495	6.850608899	5.939868061	7.976858394	7.25490398
KLRC3	6.513004101	6.31869002	7.28497204	7.483257202	6.786546495
KLRC4	7.844550919	7.658441616	8.685647889	8.769354545	8.129874121
KLRD1	7.282945384	7.327857495	7.793711495	8.086953081	7.748548515
KLRF1	8.581169111	8.672348475	9.241421747	9.368875727	8.345781111
KLRG1	7.394691081	7.248451727	8.295839707	7.584965596	8.348543172
KLRK1	6.739283293	6.677139455	7.03699504	7.780823677	7.180839586
KPNB1	12.67378338	11.81439214	11.71244686	12.41634374	12.49170848
KRCC1	9.546645747	9.238221677	10.00985275	9.650129091	9.30577901
KREMEN1	8.002342131	7.286424495	7.390471152	7.186960111	7.672658859
KRI1	9.839882283	10.60635433	9.957374697	10.49830212	10.52476206
KRT1	10.93023415	13.08418191	14.86987024	12.95303817	14.62217632
KRT10	10.45996909	10.62895885	10.761049	10.45877561	11.24001011
KRT14	7.163006	7.646666444	6.942817626	7.182015707	7.356848556
KRT18P24	7.242650333	6.413679545	7.384903253	7.29540803	7.536878778
KRT19P2	7.208855192	6.017908828	5.928380071	6.836284939	5.761780854
KRT23	13.22156044	12.41931404	13.1944969	12.15752365	11.99535117
KRT3	8.785177889	10.3548828	8.33796001	8.485631939	8.324453253
KRT31	8.696739152	9.004398101	9.323731354	9.21352099	9.802496737
KRT32	7.307562737	6.322925	6.921417374	7.321250939	7.679634566
KRT71	6.966460535	6.216046091	6.999830061	6.61398898	6.76459802
KRT72	8.079973212	9.060017677	9.294355394	7.524403394	8.754441596
KRT77	5.762979192	5.846144172	7.881467646	5.782176343	7.870356606
KRT81	11.671419	12.86086109	11.16423752	11.40495249	12.14789109
KRT85	7.59859104	8.440058758	7.370383778	7.673764162	8.065602475

KRTAP1-3	9.140978061	10.03299879	9.392082222	8.791137414	9.465659495
KRTAP10-10	8.575566677	7.851994586	8.909404788	8.85785699	8.478760515
KRTAP11-1	7.325732596	7.092673939	8.229734556	7.88183703	8.516793455
KRTAP12-1	6.842273677	6.555968283	6.443018677	6.590449828	6.787827455
KRTAP2-1	11.27695531	11.09250749	10.39414857	10.97185531	11.60069924
KRTAP2-4	8.857327323	9.379180747	8.081502051	8.53715797	8.913588253
KRTAP3-1	6.697417434	6.406575848	6.996042242	6.584387121	7.009472515
KRTAP4-1	9.472607081	10.3090972	9.218466939	8.992473788	9.422377343
KRTAP4-11	10.09597734	8.888204949	9.479084687	9.482964828	10.43655428
KRTAP4-12	8.673843717	8.126700889	7.64232702	7.612584303	8.251492909
KRTAP5-8	9.164000111	8.226840717	9.870221061	8.566871343	10.33596201
KRTAP5-9	8.469779808	8.153957263	7.937009	7.716120232	7.770089636
KRTAP6-3	8.907429495	8.208692758	7.990677606	8.331458747	8.701614283
KRTAP8-1	7.001869131	6.380219	6.961028939	7.832540091	6.457858202
KYNU	7.689234404	8.00874802	9.172421828	8.373157505	8.418118394
L3MBTL3	8.020484273	7.980256929	7.628690051	7.791244131	7.959830364
LAG3	7.217862778	7.564402343	8.153957263	7.512765949	8.588971687
LAIR2	8.147481596	7.832217929	7.218710525	7.430156263	6.597742293
LALBA	9.363307667	9.122138505	7.279669313	8.78664797	9.573320202
LAMC3	7.632861657	7.247605232	7.026870727	7.615901626	7.989204111
LAMP1	11.08435313	10.55070525	11.28370047	10.83642757	10.54441943
LAMP2	10.18942995	9.681893121	9.366909111	9.755162434	9.689783586
LAMP3	6.817996071	7.131581374	6.824112909	6.132403121	6.508047626
LAP3	8.232163879	9.350012182	9.560980909	8.901331343	9.334658071
LAPTM5	11.98999321	11.18941159	11.54053762	12.10951308	11.55322016
LASP1	10.90324033	10.40810668	11.49029795	10.89912578	10.21769501
LASS2	9.509876273	8.646531515	10.00450786	9.051375475	8.62460002
LASS4	7.756175162	6.995042596	7.094997242	7.369172101	7.192707293
LAX1	6.706206909	6.67667401	7.453956636	7.377037515	7.683943101
LBH	9.029402949	10.17224075	9.191263899	8.680513909	9.438799404
LBR	14.15092087	12.95787308	12.93430142	13.82538175	13.31405593
LBX2	6.530171141	6.532200808	6.612755929	6.722265657	6.875911141
LCE1A	13.12562627	14.39736901	13.03957854	12.89113428	13.58209631
LCE1C	12.8652792	14.48025691	10.42927797	12.00644476	12.39058663
LCE1D	15.63778652	16.64557528	12.61449239	14.93426708	15.65613201
LCE1F	7.421928768	6.80697597	7.657544313	8.127495596	7.139090576
LCE2A	7.449946354	7.373116051	8.367422212	7.56165098	7.686071172
LCE2D	9.616057222	10.80091358	9.437491071	8.800397414	8.998420707
LCE3D	6.57397498	6.770611535	7.207252646	6.978866758	7.286424495
LCE3E	10.79956984	12.77454263	9.742895778	9.945994374	10.7442872
LCE5A	16.42659037	17.07010739	15.16803204	16.2036826	16.39933956
LCK	10.01071956	10.52799495	9.84311402	10.63391466	10.56655729
LCN2	7.544047919	7.452109758	7.096955818	8.636577313	7.013931768
LCP1	14.6970922	13.95955505	14.24162707	14.7172184	14.1830221
LDB1	7.80435998	7.233950838	7.893273434	8.086566798	8.114611232
LDHB	11.78265022	12.59323492	13.30795266	13.02006671	12.90359001
LDHD	6.982384606	6.943317879	7.110215707	6.973416747	7.531058566
LDOC1L	7.059062808	7.207790596	7.752360667	7.035678848	7.287828434
LEAP2	7.174439091	7.266525586	7.442271202	7.072869879	7.235098212
LEF1	8.534432515	9.331636121	8.921011566	8.796713535	9.151976485
LEMD3	8.593558293	8.665165081	8.469779808	8.607626293	8.508189222
LEPROT	8.730448515	8.712915394	10.05688349	8.807959646	9.998242414
LETMD1	8.100639	8.370422404	9.37540303	8.401317182	8.39446903
LFNG	10.08599138	11.41071085	9.508442768	10.27338352	10.48310631
LGALS1	12.01956714	12.25844332	12.14191765	12.02811467	12.37087917
LGALS12	9.070661273	8.970028374	9.652939283	9.209526556	9.133918929
LGALS2	11.00620373	13.03025597	12.95787308	12.72972704	12.5521346
LGALS3	12.23191382	12.92081703	13.48401144	12.15130062	13.41514643

LGALS3BP	6.997357515	7.045942	6.579391333	6.893369586	7.015941667
LGALS7	8.302498081	6.970472768	7.313208869	9.024212859	7.098484101
LGALS7B	6.952529081	6.701095535	6.76700904	6.889521525	6.894957566
LGALS9	8.719245818	8.284073869	7.487351192	8.21550303	8.01423197
LGALS9C	11.79405389	11.41238356	10.99763472	11.31267384	11.01209098
LGI3	7.335553323	7.36203996	7.089056525	7.305875394	7.522559465
LGR4	7.000811505	7.202978657	6.71730499	7.363288879	7.438305869
LGR6	9.675904202	9.899010949	9.992117576	9.485668061	9.566949
LHB	12.57877126	12.89886333	10.63391466	10.57649171	11.98196729
LHFPL5	7.503692152	7.436640343	9.130293909	7.675893758	7.832540091
LHX1	8.454954939	9.815129384	8.222643394	7.783847071	8.109636222
LIG1	9.185002364	9.633493677	9.869466131	9.818860343	9.839882283
LILRA2	10.49009312	9.370832535	8.888204949	9.93663897	9.513795939
LILRA3	10.36589334	9.318228697	8.595703707	7.79851096	9.138451657
LILRA4	10.6362571	9.963504081	8.942756889	10.1684149	10.06822268
LILRA5	9.165150051	8.50618604	7.303019677	7.722297051	8.108111747
LILRB1	6.869142343	6.94614002	6.445241798	7.138289616	7.172666465
LILRB2	13.77352346	12.44611991	13.13522491	13.64083669	12.72610186
LILRB3	14.07245549	12.80602179	12.94821125	13.42888134	13.21058954
LILRP2	10.70425349	9.769994879	9.125507586	10.48310631	10.04340808
LIMA1	6.904353566	7.490096869	7.184502222	6.862375091	7.266813283
LIME1	10.07479617	9.844817354	9.426842081	9.655088667	10.27139209
LIMK2	10.5152666	9.465071586	9.908305848	10.12224713	9.725631121
LIMS1	9.324974768	9.759214424	10.40592026	10.05607537	9.470840162
LIN7A	9.128533354	8.367422212	8.874191697	8.466026899	8.659649677
LIN9	8.870056939	9.652169778	8.453185455	8.116212253	8.78767504
LIPA	10.25422487	10.5199714	10.48432644	10.57885616	10.68072859
LIPC	6.01048397	6.662284414	5.773570354	6.595504071	6.23392001
LITAF	11.74439188	10.97631922	10.64005918	11.38775416	11.54871614
LLGL2	7.399358222	7.593349828	6.958200131	7.851994586	7.772503354
LMAN1L	7.464044657	6.778194636	7.518430525	7.466006556	7.849137545
LMBRD1	11.565007	11.18636156	11.68700199	11.48061491	11.13713447
LMF2	7.91707401	8.456191465	7.515531818	7.917403434	8.075284384
LMNA	8.344860929	9.113305192	8.329784131	8.928157121	8.684654737
LMNB1	8.632349838	7.386763141	7.271701444	7.165793091	7.371907727
LMNB2	6.602666131	7.028313949	6.463301323	6.607592929	6.807214747
LMO1	6.746340717	6.767263616	6.749084333	6.980785162	7.484854374
LMO2	10.62647628	10.2761618	10.54441943	10.05880999	10.03492192
LMOD1	7.139668182	6.97809596	6.991264788	6.996514071	6.573072253
LOC100127980	8.570262	7.919797354	8.408296424	7.871683444	8.425847798
LOC100128079	6.709290939	6.132403121	6.300351141	6.260841061	6.486163293
LOC100128108	7.842359758	7.965660091	7.895961929	7.831138545	7.794719919
LOC100128140	9.482964828	10.01524205	11.90674449	10.21054893	11.18800276
LOC100128295	9.716648152	9.214111545	8.48093102	9.813557354	9.195534384
LOC100128364	8.713915657	9.212980727	8.268937657	8.188379657	8.204025576
LOC100128439	9.533033172	8.261781515	8.532980343	9.481000434	8.211411717
LOC100128485	10.94071069	11.50051559	8.704708384	11.72807534	11.02403513
LOC100128548	9.117478424	9.829119768	11.01807842	9.456392222	9.593075545
LOC100128626	6.01801997	6.031316343	6.542523919	6.013673768	5.87837302
LOC100128737	9.438799404	9.066605263	9.536330707	9.005975162	8.546982556
LOC100128760	7.721759747	8.240781051	7.745118444	8.168196313	8.223899788
LOC100128822	7.847379061	7.612294131	8.545617303	7.45998899	7.652632424
LOC100128836	8.886096818	9.362238061	10.45996909	9.29496	9.208368677
LOC100128942	10.98627861	12.33576886	10.20634509	10.45628541	10.98186414
LOC100128961	8.786134101	8.942313313	8.090886384	9.002847848	8.803935202
LOC100128977	10.75865445	11.74439188	9.207219232	11.41981305	11.81887725
LOC100129113	7.577683121	8.056742909	7.531770848	7.717096556	8.044608626
LOC100129115	7.417598061	7.133298475	6.955646677	7.007603253	6.716343364

LOC100129122	14.48025691	15.48449671	12.39973853	13.46005067	14.1282639
LOC100129292	9.272374697	10.31997281	10.30810853	9.524763869	9.821195081
LOC100129424	12.77454263	13.22730043	13.99495772	13.34730616	13.26276274
LOC100129434	8.047553323	6.777950687	7.564402343	7.864224778	7.042119313
LOC100129455	7.006187899	6.276206182	6.804033586	6.751979343	6.847553121
LOC100129500	7.817975838	8.456593071	7.70790696	8.341133162	8.013081051
LOC100129553	10.3380268	10.71992703	11.71659127	11.30131952	11.28896545
LOC100129742	12.62807191	13.04381444	13.38447499	13.25686564	13.22730043
LOC100130107	8.692870869	9.180906778	10.64623997	9.085558838	9.564343333
LOC100130127	8.773988747	8.433042727	8.452737606	8.238272808	8.159434576
LOC100130152	16.73234105	17.01900663	16.90924948	16.64557528	16.73234105
LOC100130233	8.698215303	9.392082222	10.41957053	9.388912091	9.745446081
LOC100130274	7.390752071	6.616118788	7.014842626	7.02612702	6.891529131
LOC100130357	7.757234778	7.378842657	6.514775394	6.916181	6.61996199
LOC100130476	6.416786646	6.510269939	5.767680525	6.033623869	6.211355596
LOC100130557	8.578858313	9.459003	7.875433545	8.099112939	8.50618604
LOC100130600	7.610260323	7.008416879	7.445127778	7.033845343	6.706206909
LOC100130713	7.818322879	7.960941889	7.871272152	7.337098929	7.550195808
LOC100130797	7.452109758	6.372542859	7.21466303	7.621361828	8.458294828
LOC100130856	8.110083838	8.766842081	7.787223354	7.895961929	7.971654859
LOC100130886	11.59212007	10.95849458	10.23948175	11.24816417	10.54078574
LOC100130892	13.49745762	13.76702458	14.57091557	14.03424604	14.04335209
LOC100130920	9.271759586	8.388817192	9.122783717	8.456191465	8.025864485
LOC100130938	9.483552818	9.636296737	10.73517618	9.604846424	9.110297333
LOC100130950	10.03611568	8.381754576	10.24612484	9.854472677	10.54190997
LOC100130958	15.39581235	16.42659037	13.09463792	15.06622261	15.24813401
LOC100131053	6.923412444	6.735607747	6.367511859	6.828622869	6.668984303
LOC100131117	6.294120212	6.646655162	6.562194212	6.022865747	6.24214599
LOC100131138	6.551639909	6.293334414	6.94614002	6.865560778	6.980238687
LOC100131170	8.067838768	6.801564111	7.160791343	7.457078101	7.168942192
LOC100131262	12.92542832	14.40789433	11.53239215	12.08128174	12.39660011
LOC100131310	11.07647857	11.77802923	12.19086998	11.52824	11.55928848
LOC100131323	8.985821889	10.08145981	9.675185677	9.731150899	10.22865659
LOC100131360	7.216973434	7.103272273	7.274379929	7.001377828	7.444499747
LOC100131582	12.80949032	14.23242051	10.40387449	11.9577766	12.12692782
LOC100131662	6.483801374	6.141738101	6.851905687	6.435963586	6.931701566
LOC100131699	7.797507939	8.583542788	9.143279535	8.100639	8.194244232
LOC100131707	8.688838939	8.133179586	8.563941778	8.660608859	9.03221704
LOC100131733	8.360024717	8.885588879	9.345614434	8.863375313	9.338458455
LOC100131744	6.632345869	6.252089525	6.73803103	6.698531303	7.159962485
LOC100131785	8.110881556	8.14677703	8.113745737	8.396963808	7.931708101
LOC100131845	7.092334828	6.23392001	6.830332131	6.901187545	6.323307657
LOC100131971	8.952561111	7.798177384	9.026117657	9.350012182	9.488971182
LOC100131993	8.544134919	8.172571899	8.109636222	7.92237903	7.823083152
LOC100132050	10.22668357	10.18762619	9.878686616	9.896649636	9.908305848
LOC100132053	10.45150222	11.43208235	11.90384839	11.02403513	11.22456459
LOC100132247	13.83173935	13.90036211	13.52334642	14.1282639	13.84455043
LOC100132357	6.632973788	6.515007141	5.772560434	6.212018222	5.945080303
LOC100132409	7.048974677	6.121104576	7.930919909	7.915342566	8.464081152
LOC100132640	8.729485838	8.009790253	7.665314131	7.579959303	7.400015081
LOC100132673	14.04335209	14.5413574	14.77222727	14.59173737	14.38889659
LOC100132738	8.579336081	6.608273162	6.266464606	8.18280501	6.161943081
LOC100132816	6.809486374	7.073657889	6.477491586	5.878464747	6.91172998
LOC100132831	8.82842597	8.391460737	8.127895253	8.932594273	8.612168455
LOC100132941	7.356281616	6.655494515	7.602282465	7.455517596	6.883018111
LOC100133075	7.576996152	6.933000202	7.595878485	8.127056495	7.092334828
LOC100133121	7.248766182	6.954672071	6.508494242	6.714736646	6.197355596
LOC100133627	7.10439698	7.109400202	8.542859455	8.810446485	8.496365737

LOC100133781	8.155735586	7.611975737	8.215082485	7.541090081	8.219387636
LOC100133788	9.545191596	9.617464455	9.153232364	9.482360616	9.35371997
LOC100133897	7.838173152	9.466909636	7.874063444	7.815532475	7.842052606
LOC100134167	9.534194535	10.31289083	10.97476091	9.578067192	9.945028121
LOC100134229	7.473129747	7.586712596	7.957018646	7.797507939	8.373514202
LOC100134231	12.06964699	13.77352346	12.74093701	11.78023213	12.53197264
LOC100134282	8.815152444	8.986432162	8.399075535	9.858738737	9.386484303
LOC100134331	7.290498626	6.930222384	6.741723879	6.896238273	6.816537596
LOC100134400	7.453956636	6.04186799	6.871158202	6.980492495	6.133965273
LOC100170939	9.214111545	9.376046232	9.962571606	8.937936253	9.475206131
LOC100190890	7.380085202	7.394027687	6.72626003	6.480359747	7.29228501
LOC100192204	9.496413788	10.14326599	9.919491525	9.950180667	10.19649023
LOC113230	7.387388131	7.982067081	6.690165707	7.152420131	7.083013909
LOC119358	10.13193078	10.55966675	10.60277882	10.54565144	10.74045328
LOC120364	7.681167667	8.086953081	9.243679384	8.522641273	8.529888505
LOC122589	10.97476091	11.65698449	11.00902763	11.64848719	11.48258282
LOC127099	10.5029511	11.51008986	12.30465105	11.26262606	11.31852116
LOC129293	7.638981707	8.52305899	8.396532182	8.289996192	8.272231939
LOC130728	7.140269465	8.102862354	7.943433747	7.496977949	8.056301667
LOC131054	12.11841559	12.23191382	12.37087917	12.66627862	12.6076243
LOC131055	11.51260306	10.5104744	11.46724679	11.04030995	11.09250749
LOC131691	11.54470354	10.70181173	11.30690717	10.65246846	10.72516317
LOC137107	8.113745737	9.170513566	8.809951364	8.508645727	8.843670869
LOC142937	6.962034242	7.041369354	8.104500051	8.530767747	8.422643323
LOC145694	9.979092404	12.12939166	8.052417212	9.06557301	10.0178996
LOC146346	8.495447152	8.869521111	8.650384111	8.448008778	8.430929525
LOC147645	8.953164051	7.995268899	10.27035429	9.176817596	9.788274313
LOC150166	11.53677901	10.08599138	10.26632574	11.10170724	10.50794857
LOC150759	8.177146606	8.706127333	9.576136707	8.267698071	8.322806515
LOC150763	7.016958768	6.762568667	7.099574394	6.81111	6.258571172
LOC151438	10.37573078	8.818210192	9.739033131	9.59460096	9.928455091
LOC152663	11.00754401	11.04886125	12.57107442	11.64637724	11.38577951
LOC152719	6.207117515	6.057027071	6.628772899	6.288951848	6.696696758
LOC157562	8.041792596	7.901561475	8.06745399	7.418614091	7.550541152
LOC157740	8.585303495	9.197870838	7.713270545	8.492747404	8.653441707
LOC158345	11.58621592	11.77305263	12.82153585	12.14191765	11.87175588
LOC158830	9.714566535	9.980879424	10.23160947	9.35128199	9.497084828
LOC161527	7.006478434	7.044506424	7.23423804	7.802180768	7.228468091
LOC201175	9.412634061	10.28506122	9.667708556	9.109120374	9.576136707
LOC202181	8.298896434	7.152420131	6.995294596	9.095762	6.896711899
LOC202227	10.47702836	9.932690333	10.85361094	10.40592026	10.42062148
LOC204010	10.5104744	11.19114259	11.86401838	11.39382595	11.58621592
LOC220433	12.01427079	12.56315114	13.54965001	12.87421343	13.48990673
LOC220594	7.243253313	7.666980273	7.574795778	6.993183869	6.799383374
LOC220906	9.077686808	9.281923404	10.22164118	9.071813909	9.176817596
LOC221442	8.069753788	7.810980737	8.086153313	7.331138333	7.21931098
LOC222159	7.000584848	6.560197424	7.050623333	6.934568707	7.122424869
LOC23117	12.91656586	13.27418186	12.34237091	12.7494647	12.8293171
LOC253039	7.735579636	7.731871869	8.705145061	8.302900242	7.82475102
LOC255783	12.63500423	12.87819387	11.56725755	12.39973853	12.42233701
LOC257039	7.965251131	9.003357768	8.354570556	8.249350414	8.487847515
LOC26010	6.815206394	6.716586707	7.103541778	6.530842141	6.705162535
LOC283174	7.20666299	7.427999919	7.582311101	6.980238687	7.056715131
LOC283177	15.59877501	16.14978837	15.56435861	15.22815255	15.56435861
LOC283412	10.89224764	11.63174178	11.31661678	11.48655657	11.40306525
LOC283454	6.046027313	6.831129859	6.144529202	6.168988222	5.930116374
LOC283663	11.41415047	11.38025327	11.63174178	11.19638858	11.77552743
LOC283767	9.35128199	9.455015404	8.289996192	7.06536003	8.008022949

LOC283788	9.753529758	10.18666124	10.18942995	9.684886576	10.39296715
LOC284230	11.95514832	12.28105924	12.54906863	12.82558839	13.00581854
LOC284297	7.510907818	6.962822313	7.532150707	7.376746737	7.928832091
LOC284402	7.993077394	7.387388131	7.771143222	7.982067081	8.247392788
LOC284422	8.046433182	8.457043091	8.761644081	8.728878424	7.776955808
LOC284441	8.879330566	8.008400859	9.544495798	9.093001495	8.992473788
LOC284454	9.071813909	9.052474394	9.006527424	9.012760899	8.720627081
LOC284542	9.543825273	10.03611568	8.401710747	8.845785121	9.333361616
LOC284570	7.763186	7.048124798	7.248166182	7.664957788	7.185712515
LOC285535	6.711872081	7.050890818	6.511217374	6.871158202	6.497193293
LOC285550	6.905316545	6.762834384	5.766620949	6.409677646	6.326292525
LOC285708	6.499296646	6.207117515	6.173448717	6.010748859	7.154661646
LOC286254	8.833963152	8.66101001	8.629055081	7.88791003	7.923048354
LOC338620	6.595947525	7.136095172	6.11789698	6.662284414	6.580062899
LOC339344	10.84568098	11.28370047	10.43757507	10.71992703	10.57282784
LOC339483	6.203185697	6.632345869	6.238699505	6.506758646	6.524259657
LOC340508	7.729367111	8.539328929	7.935081758	7.739314727	7.502411182
LOC340947	7.641985	8.095746162	8.448008778	8.379884889	8.315715768
LOC342994	14.60195877	14.93426708	15.48449671	15.26374094	15.26374094
LOC343515	9.134591697	9.410728505	9.746145949	9.243679384	8.86638896
LOC344328	8.501579646	9.083741606	9.579413505	9.041236566	9.486281202
LOC346887	8.237787788	7.034331232	7.687700545	8.903602263	7.30673204
LOC349196	6.696473434	5.926764929	5.951607646	6.424911465	5.849599879
LOC374491	6.938411576	6.307933879	6.641155141	6.543902859	6.163980101
LOC376693	13.40302459	13.79381649	13.72760196	13.85817003	14.31917195
LOC388122	11.69658017	12.11216258	13.01016522	12.4956701	12.63888703
LOC388242	7.389558374	7.649291899	8.184050869	7.316995263	7.84419497
LOC388401	12.71520416	13.10484893	14.11997744	13.32021166	13.40990013
LOC388494	9.518503263	9.638947465	9.231349182	9.472607081	9.731806091
LOC388532	11.48459908	12.69316533	11.92156179	11.5571076	13.23341018
LOC388588	7.709201212	11.21958266	10.2761618	10.68452674	10.54800525
LOC388965	8.872352707	9.853774707	10.08231743	9.673359929	9.877792838
LOC389033	7.641683626	7.828584192	7.656238101	7.93888704	7.634226727
LOC389127	12.47441306	13.06967433	13.79381649	13.03957854	13.04381444
LOC389199	9.374121768	11.72355631	8.474658303	8.788663253	9.383480283
LOC389634	8.921011566	8.39402401	8.960773354	8.922632727	8.891390919
LOC389641	6.32793802	6.787317758	7.231529313	6.613208707	6.708558414
LOC390183	9.429506515	10.04862183	10.29000276	8.880853535	10.84705809
LOC390282	11.0940021	10.91903182	11.78725933	11.13240418	11.36065784
LOC390413	10.25627793	11.15183093	11.69658017	10.43757507	10.79420974
LOC390612	9.391460545	10.18000397	10.75987116	9.891739697	10.0188097
LOC390876	10.74690378	11.35080354	11.93178106	11.59870577	11.75462574
LOC391126	13.16824615	13.40990013	14.61202705	13.40302459	13.54221797
LOC391132	8.352944343	8.773471869	9.182204323	9.189590182	9.288419414
LOC391160	11.20498985	10.76471511	11.73070023	11.46724679	11.29399858
LOC391282	10.10736711	10.20452777	11.52052132	9.76546198	9.514496606
LOC391334	11.49839615	11.30131952	9.797856576	11.11560115	10.70534521
LOC391504	12.00086001	12.3774088	14.21449649	12.53197264	12.78185519
LOC391655	12.06397065	12.33273925	13.20018245	13.38447499	13.44849295
LOC391656	12.21312889	12.53197264	12.9851923	12.76628311	12.78645268
LOC391769	16.39933956	15.46469304	16.33688632	16.09492117	15.90458538
LOC391777	10.85513517	11.41778198	11.95514832	11.42787377	12.01956714
LOC392288	7.997183333	7.815211869	8.553595747	7.476255091	7.779426687
LOC392382	7.588938485	8.179120646	7.825440475	8.516311424	7.945111576
LOC392425	12.45390404	13.16151113	13.14611959	13.30795266	13.70717091
LOC392522	10.90606985	11.911688	10.54190997	11.69891546	11.84736958
LOC399851	8.62460002	9.421059	8.858692343	8.144584758	8.305359949
LOC400036	8.008022949	7.435709141	6.829579545	8.007640657	7.492282404

LOC400061	7.159715333	7.604109192	7.754082929	8.025425616	8.316570081
LOC400960	8.06213903	7.724049626	7.228759818	7.815915242	7.524103909
LOC400986	6.751435283	6.903822293	8.40702104	7.317304515	7.152687384
LOC401127	7.523479626	7.587331949	7.98690202	7.719466677	8.743617505
LOC401233	9.458377051	6.834332747	10.28089537	8.883993253	9.787586646
LOC401357	13.2504616	12.19086998	11.94979793	12.42616911	12.26475658
LOC401504	8.227270172	7.938205586	8.472435657	8.39308204	8.321152475
LOC401588	7.527339687	7.363924808	7.171072313	7.108492263	7.222163485
LOC401648	9.915234909	10.4524716	10.92757694	10.43655428	10.56802411
LOC401650	16.14978837	16.52993947	14.6970922	15.52328152	16.22759729
LOC401717	10.38631174	10.88945836	12.73352188	11.35657003	11.5386618
LOC402026	8.84935699	8.630877737	8.804477505	9.127872697	9.242021586
LOC402198	6.148191788	5.756678677	7.120752303	6.430485283	6.657421566
LOC407835	9.692793657	10.16937171	8.458697343	10.13193078	10.09795649
LOC439911	7.236577444	7.326634626	8.497692788	7.106592788	7.133813545
LOC439949	9.133284677	10.42622906	10.4524716	10.14326599	10.64379219
LOC439951	14.9727616	15.83165575	12.82558839	14.10328164	14.52085241
LOC440093	15.09418019	14.1282639	15.36066179	14.74028573	14.42673578
LOC440311	13.57574016	13.34730616	13.33661988	13.60269863	13.81935116
LOC440348	9.648698737	9.89348604	9.328711051	9.744663444	10.40387449
LOC440350	8.225777414	8.130993828	7.485478828	8.320817869	8.336651323
LOC440396	6.538664657	6.837226	7.338887687	7.325732596	7.389558374
LOC440419	7.770465818	7.318843495	7.126018333	7.339805162	7.293748525
LOC440461	13.52334642	14.58095582	11.4535969	12.2293359	12.99088515
LOC440731	8.677215515	7.690840778	8.324453253	8.195405505	8.462637525
LOC440737	11.10035391	11.60888892	12.67700968	11.84027237	12.00644476
LOC440894	7.003715121	8.052783737	7.048124798	6.54114802	6.932196717
LOC440917	7.519695475	7.667309091	8.277405556	7.998587576	8.294235768
LOC440957	8.24281297	8.949261586	8.556855828	8.518144394	9.058356636
LOC440983	10.56655729	10.37686258	9.421700798	10.28506122	10.3779146
LOC441073	15.12351424	15.08134613	16.42659037	15.67809237	15.67809237
LOC441131	10.67939021	10.18468509	11.48655657	10.80091358	10.71613341
LOC441245	7.442271202	6.79370899	7.581657414	7.178323677	7.883917657
LOC441251	7.510027525	7.630934212	8.359650919	7.743818828	7.308713444
LOC441377	12.03602449	10.4687107	12.18792926	12.48124823	12.57486869
LOC441455	10.89094316	11.69440164	11.89876792	11.6295292	11.80104424
LOC441768	9.349388778	9.308918081	10.1752482	8.930433505	8.638487899
LOC441896	12.40343679	12.76628311	13.81266894	12.72219022	12.87421343
LOC442160	10.57758511	11.71659127	11.33477589	11.00496707	11.73710477
LOC442175	6.91094498	6.853963192	6.355365798	6.460516394	6.702734242
LOC442211	10.31795252	10.95234717	9.292435667	10.39747702	10.77890896
LOC442270	12.96241124	14.07245549	14.67552064	13.55640443	14.07245549
LOC442572	7.457078101	7.85268101	8.369167818	7.61520499	7.192412434
LOC442590	7.746135697	7.566773798	7.200755828	7.099269525	6.771393131
LOC494558	8.530333535	8.516793455	7.939609869	8.709941838	9.473222667
LOC497190	9.484840202	8.908475283	8.541125101	7.557551899	7.750279556
LOC497257	10.16937171	9.795448596	10.03492192	10.50543333	10.41330878
LOC51233	8.316081222	7.442271202	7.518690889	8.559975162	8.826846768
LOC541469	7.170797768	8.276014121	7.098999354	6.901740727	7.548285091
LOC595101	7.174161374	7.140009717	7.31258798	6.990487535	7.603101596
LOC641518	5.964275485	6.953109798	7.091328697	6.333400636	6.458494616
LOC642424	7.568924192	7.325162263	7.275765808	7.400314747	7.066776273
LOC642521	8.137479222	8.135850465	8.696739152	6.558125737	7.885731566
LOC642738	13.93694444	14.57091557	15.19830883	14.24162707	14.58095582
LOC643220	8.429130081	9.329342374	8.69919198	8.885588879	9.580783667
LOC643313	7.309928404	6.677606	6.112376939	6.255807596	6.494395354
LOC643733	7.381652111	7.932792414	8.152443121	7.969793182	8.215875455
LOC643779	11.38970236	12.08943903	11.94234713	12.23191382	11.92937262

LOC643905	7.763846354	6.975683768	7.770089636	6.896472707	7.619933919
LOC643932	14.31917195	14.53168901	14.63205333	15.21350605	15.09418019
LOC643960	12.04160066	12.65104366	12.60396528	12.77454263	12.76628311
LOC643997	8.297630758	8.808928141	9.749660394	9.038261515	8.700606657
LOC644029	11.77053658	12.10694646	13.07947925	12.46466592	12.47130519
LOC644063	9.17294002	8.803935202	8.499404646	9.333361616	9.077686808
LOC644215	8.207537162	8.864955909	8.043818737	8.181196919	8.191204879
LOC644236	12.43279956	12.66985986	14.26676228	13.13007894	12.87819387
LOC644246	9.276789111	11.49636188	10.05144087	8.34982299	9.278597747
LOC644297	9.348735909	8.777572838	9.830354566	8.921533879	9.776037091
LOC644384	11.27153548	11.78265022	12.94367069	11.97660652	12.2293359
LOC644387	7.271416848	6.419103354	6.812168303	6.832629131	6.866597293
LOC644450	6.317278899	6.758395253	6.344995131	6.724256232	6.768970657
LOC644456	8.129874121	7.644959949	8.164979273	8.572163455	8.322342051
LOC644563	9.249612495	9.710962273	10.12224713	10.07956055	10.24139873
LOC644613	9.408808788	8.866888202	9.565032515	9.683334444	10.43757507
LOC644936	12.32364634	11.84027237	10.86963124	12.09225922	11.38775416
LOC644937	12.94821125	13.26276274	13.48990673	14.010587	13.9071606
LOC644950	15.01206542	14.32804886	15.32551587	14.77222727	14.5413574
LOC645000	11.10170724	12.61449239	12.68484185	12.56315114	12.41634374
LOC645195	7.014842626	7.967920667	7.249962576	6.79984598	7.051121485
LOC645231	7.507381747	8.479185606	7.450590374	7.881102323	8.482852263
LOC645251	9.101086141	8.515864333	8.653441707	8.76386996	8.914132333
LOC645427	7.432999727	7.111963455	6.456479596	7.047046253	7.323994646
LOC645683	13.72760196	13.93694444	13.92894576	14.4427031	14.89443037
LOC645693	12.84197228	13.10985871	14.90726461	13.62106704	13.75520609
LOC645733	5.766954061	5.803409091	5.887644263	5.784754697	5.794478101
LOC645884	11.31852116	11.59212007	12.7494647	12.16279806	12.36759935
LOC645955	8.983202444	10.33596201	8.411285283	8.484723788	9.022478232
LOC646048	8.165380303	8.173633596	7.230210646	7.82717702	7.954205616
LOC646119	11.75908761	12.32659908	12.80949032	12.28369472	12.48835313
LOC646346	12.8293171	13.01016522	13.83772389	13.27418186	13.35359128
LOC646626	8.880853535	8.58807297	9.388273121	8.962353222	9.269847242
LOC646652	7.199434576	6.624585818	6.960198707	6.385847152	6.397781283
LOC646672	10.81796524	11.33292491	11.90152869	11.74935785	11.67371856
LOC646753	10.29660096	8.943348051	10.67428006	10.98037262	11.34202326
LOC646791	9.207219232	9.276789111	9.778131475	9.613206141	10.40486524
LOC646875	12.88265743	13.35359128	13.97489728	13.35919327	13.45466248
LOC646890	9.176817596	9.523397737	10.46108665	9.329922273	9.810255869
LOC646909	8.691458141	9.434425707	9.033915556	9.40167604	9.806332162
LOC646949	12.84627851	13.38447499	13.98081649	13.29117716	13.62784329
LOC646960	9.223885747	11.35475345	8.434412929	8.476085384	8.816214848
LOC646973	6.295493455	6.787827455	7.821271606	7.265354444	7.57799899
LOC647131	6.835371293	6.838696091	7.043463859	6.887875313	6.714000354
LOC647195	9.146732444	9.035586242	8.609952768	9.094076404	9.322592778
LOC647954	6.974671657	6.262151687	6.028808465	6.591813727	6.605442354
LOC648232	7.743818828	8.000883182	7.587956192	7.838173152	7.755503707
LOC648294	12.82153585	13.02006671	15.10875812	13.5160991	13.83772389
LOC649294	10.81034167	12.73731843	9.081281455	9.515069697	10.31795252
LOC650392	13.10985871	14.50017253	11.34892675	12.47130519	13.5964752
LOC650405	8.144908747	7.657544313	7.928481586	8.020096283	7.258429848
LOC652147	7.98285402	8.295839707	7.311629869	7.425203606	7.978709162
LOC652494	7.091827414	6.354946212	6.74585002	7.517436172	6.698319919
LOC653080	7.225301495	7.135797152	7.949790515	7.421928768	7.606699253
LOC653284	5.881116071	6.046443465	6.792730596	6.35000001	6.804494667
LOC653773	11.89130603	12.68097339	11.96947069	12.36473556	12.80602179
LOC653884	10.14631732	11.73972102	9.037616626	9.178084667	10.80091358
LOC654170	11.79870316	12.54906863	12.76628311	12.35168631	12.61761735

LOC654350	11.45932673	11.99323436	12.53197264	12.18792926	12.21312889
LOC654433	7.213178616	5.792049859	5.91279402	6.273265505	5.786127747
LOC727805	7.03599896	6.344827192	6.166487869	6.615162343	6.367696616
LOC727820	10.75987116	10.41022215	9.831834717	10.69574272	10.53737043
LOC727834	5.802713354	6.518533202	5.759121182	6.068447646	6.625506687
LOC727835	13.74759592	13.98081649	15.72204116	14.60195877	14.90726461
LOC727865	14.20688311	14.5513462	15.86132141	14.82007331	14.94534759
LOC727997	14.25042984	15.18392271	15.59877501	14.63205333	14.93426708
LOC728126	11.48655657	12.05813886	12.57877126	12.17216641	12.33273925
LOC728131	13.32021166	13.5160991	15.42966281	14.11997744	14.49002723
LOC728153	7.019151556	7.352546465	7.427372788	8.583996586	8.313601758
LOC728323	8.331046747	8.892970697	7.754407434	8.030983081	8.227704253
LOC728380	14.07988992	14.34357305	14.78379092	14.4185289	14.26676228
LOC728411	9.211846273	9.048100778	8.899254879	9.873591818	9.497817424
LOC728428	12.69316533	13.34204398	13.9071606	13.71423947	13.5160991
LOC728448	8.062539303	8.282900293	8.486053202	8.181574081	7.756889051
LOC728449	8.700606657	9.630502939	7.951199808	8.186373323	8.555833323
LOC728450	7.950135394	7.72538904	8.155393222	7.89464897	7.915639949
LOC728537	6.981850818	7.307847101	6.873566444	6.750902172	7.018669091
LOC728723	11.57567047	11.22947614	12.03602449	11.92665931	11.98999321
LOC728732	10.65500785	10.89604789	12.03068847	11.565007	11.58421758
LOC728774	6.96124304	7.85507	7.345977091	7.175798869	7.32338401
LOC728820	9.08959502	9.558925838	9.949411172	9.59387599	9.832711535
LOC728971	13.58209631	13.71423947	15.31193978	14.68633385	14.72870348
LOC729029	6.864750879	7.361380131	9.223302313	8.154705535	7.768055657
LOC729046	10.47941256	11.13879804	11.1276132	11.26990549	11.38970236
LOC729082	9.165786899	9.420326576	9.538263677	8.937429899	9.31327601
LOC729090	11.94745099	12.62127559	13.74093847	12.90783816	12.95303817
LOC729173	8.173308798	9.063885091	6.520115535	7.556918475	7.612294131
LOC729259	11.85033795	12.15130062	12.41931404	12.58612865	12.53563192
LOC729279	7.659822475	8.079179394	8.622644364	8.352134333	8.229734556
LOC729298	7.509707515	7.572084505	8.252372808	7.648616949	7.836508475
LOC729313	12.46797904	11.31661678	12.38063147	11.92937262	11.96362869
LOC729314	8.905887879	7.671593667	8.574218515	8.462637525	8.394903626
LOC729449	12.10135206	12.18792926	13.81935116	12.75784116	13.12023156
LOC729484	14.9591327	15.42966281	16.02065153	15.38120792	15.81050193
LOC729497	7.021742939	6.740481444	7.744777657	6.610458242	6.729463253
LOC729556	7.322572717	6.679026717	7.627670859	7.855388333	8.142197384
LOC729603	8.396532182	7.170797768	7.520648323	8.114219303	7.377037515
LOC729608	6.968936475	7.884314808	7.514821949	7.563439404	7.937868929
LOC729742	12.93862501	13.72760196	13.94443	13.44126502	13.77352346
LOC729760	11.02403513	11.9982543	12.20960187	11.36793803	12.06964699
LOC729798	13.06488572	13.63435472	14.33555643	13.56886427	13.7211242
LOC729858	10.4700758	10.4302696	10.64128512	10.6426412	10.61743608
LOC729915	7.983184263	6.757659566	7.228169333	7.000811505	6.856233343
LOC729956	9.918671212	12.10951308	8.257253131	8.397853727	9.41003803
LOC729983	6.925244263	5.801694131	6.411697737	6.744878414	6.658852677
LOC730070	15.81050193	15.52328152	16.96059838	16.39933956	16.28343863
LOC730092	6.734989303	7.024222101	7.070939121	6.833358384	6.946711626
LOC730144	11.38775416	11.42562271	11.68484165	11.68948846	11.81221198
LOC730168	8.000883182	9.342353505	7.344596535	6.986217889	8.178268222
LOC730235	10.32946621	9.862946677	9.348735909	9.204345657	9.531623293
LOC730286	12.55625498	11.81221198	11.25349741	11.48459908	11.58195332
LOC730392	7.742113232	7.051418091	7.479119	7.476561202	7.587635919
LOC730747	10.74558579	11.36610866	12.32364634	11.25700815	11.70557621
LOC731275	11.22947614	10.761049	9.943097475	10.24942024	10.17336172
LOC731688	14.21449649	14.67552064	15.08134613	14.70594079	15.01206542
LOC732199	8.787164939	9.393934131	10.04600105	9.382205394	9.647013263

LOC90499	9.580097263	10.21769501	8.580722586	9.439917707	9.748306121
LOC92249	13.22730043	14.77222727	9.991229606	11.69440164	12.67700968
LOC92659	12.92937388	14.49002723	10.21279484	11.94481318	13.06488572
LONP1	9.198436465	9.835081424	9.325601172	9.776037091	9.648698737
LPAR2	10.35385551	9.033915556	8.012698303	10.16355358	9.207791152
LPGAT1	10.00262223	9.32739897	9.941224515	9.853021111	9.712341424
LPIN1	7.247290424	7.279669313	7.810269677	7.93027604	8.27903296
LPIN3	6.989715687	6.140075394	6.403697939	6.560320591	6.635108111
LPL	5.896564141	5.769748374	6.704451293	6.277331707	5.902862586
LPO	5.891798727	6.371622788	6.313976677	6.620195253	7.02750098
LPPR2	7.991889949	7.460268556	7.563777768	7.624419899	7.707585606
LQK1	7.871272152	7.752031929	8.327767232	6.649897606	7.436336172
LRBA	7.542431869	7.752360667	8.671783687	8.015705778	8.287081333
LRFN1	6.961028939	7.189710394	6.68968302	6.98587896	7.330546788
LRG1	11.35657003	9.994772242	9.562994313	10.59924244	10.00695165
LRIG1	8.370830747	8.663303283	9.651465606	8.667049253	8.586731747
LRP10	10.17613521	9.605576293	8.668536687	9.742174424	9.627013505
LRP3	14.35197184	15.26374094	12.12151457	12.53563192	14.11151028
LRP5L	7.317897131	7.335878859	6.185826515	7.044743657	7.110528152
LRPPRC	6.845568374	6.988716071	7.450888848	6.926221848	7.209485919
LRRC16B	6.911201424	7.159962485	6.909148313	7.110528152	7.303562909
LRRC37A4	6.953109798	6.466022525	7.767042424	7.168718192	7.07468796
LRRC4	8.297224525	7.500795283	7.953486636	7.481322818	7.838558162
LRRC40	7.557551899	8.226165	6.939171505	7.317590788	7.419275485
LRRC45	8.200539131	8.206690121	7.899333596	7.617890202	7.796085586
LRRC56	7.232456929	7.763504859	7.867668606	7.039966242	7.198026636
LRRC6	7.528986283	7.109678444	7.756512818	7.253764222	7.747885899
LRRK2	11.05033578	10.25320711	11.05796986	10.99763472	11.29597305
LRRN2	8.02125603	8.83948901	7.60255999	7.602282465	8.093473121
LRRN3	6.728447091	7.237838192	6.773240303	6.739283293	7.372532788
LRWD1	11.62494396	10.84705809	11.06877356	10.96162926	10.76857843
LSDP5	8.210994667	7.588938485	7.357775939	7.999737535	7.416298071
LSM5	8.641737071	9.223885747	8.852576737	8.908475283	9.123297364
LSM6	9.912769303	9.958247111	9.456392222	9.49850403	9.584100455
LSM7	11.21816154	11.70812672	11.25898056	11.21016953	11.13240418
LSM8	10.29542336	10.36465285	10.10454015	10.02439619	10.37124354
LSP1	13.99495772	13.30255942	13.06488572	13.66158872	13.18348162
LSR	7.620272192	7.868335495	7.381652111	7.474059606	7.365664505
LST1	14.42673578	13.64789782	14.15863941	14.46162431	13.69473258
LTA	9.964343626	9.547339828	10.37026232	10.28795108	10.40178901
LTA4H	12.17513939	11.92937262	13.17281418	12.7939548	12.64708972
LTB	15.46469304	15.10875812	15.54332231	15.78787594	15.39581235
LTB4R	8.784105778	7.749299101	7.549206232	8.236986626	7.910975505
LTB4R2	6.972058485	7.13133802	7.701352778	7.680845586	7.634532263
LTBP1	7.024771818	7.742830394	7.848409778	7.505759091	6.611130667
LTBP4	7.369773828	7.555972677	8.24438497	7.592388485	7.815532475
LTBR	8.816703535	8.218887434	7.710917071	8.972927475	8.583996586
LTF	8.997807768	8.044980354	7.617890202	10.67305197	7.72538904
LTK	7.491357222	6.966460535	6.845568374	7.141717727	7.437044505
LTV1	8.025864485	8.476994374	9.025691242	8.576430182	8.334587636
LXN	8.29927897	8.571194222	8.701109788	7.873377879	8.039068889
LY6E	7.079375364	7.228468091	7.287539616	7.131863879	7.363924808
LY75	9.048620525	7.787223354	8.654464586	8.964085616	7.807154444
LY86	10.39296715	10.74305691	10.70181173	10.31795252	10.44712342
LY96	12.11216258	11.6295292	11.03737422	11.07956266	11.57567047
LYAR	8.288815495	8.507703202	9.505859384	8.799348343	9.050797061
LYL1	11.21338774	12.81735602	10.61001127	11.89130603	12.04160066
LYN	13.04869312	12.11841559	13.07454735	12.61761735	12.30465105

LYNX1	9.793942798	10.08231743	9.227275152	9.105035414	9.388912091
LYPD3	7.907924051	9.169862626	7.104662848	7.40526796	8.137069859
LYRM1	8.321509909	7.851640596	8.885588879	8.126700889	8.152794889
LYSMD2	10.41123643	10.31480319	11.25700815	10.0406788	10.4322159
LYST	12.20960187	11.75662781	11.18291631	11.86125412	11.6295292
LYZ	11.80791686	12.00354228	9.465659495	10.55842197	12.11841559
LYZL1	7.187213162	7.10076196	7.090449141	7.611975737	8.212685152
LZTR1	7.476561202	7.301216929	6.749332859	7.70790696	7.238133434
M6PR	7.55178802	7.828991111	8.219832939	8.050868949	7.999737535
M6PRBP1	9.226128374	8.542859455	8.294235768	8.563941778	8.320817869
MAB21L2	7.546918172	6.337413646	8.142197384	6.950101798	8.961854929
MAD1L1	7.757532545	8.877161475	7.477877475	8.816214848	8.309247939
MADD	9.853774707	9.661659101	9.110297333	10.46766096	10.18468509
MAF1	8.569735879	9.204855576	8.021984222	8.809951364	8.922082808
MAFA	9.31393701	10.83363242	10.25038968	8.983202444	9.999132495
MAFB	9.882779162	10.01703805	9.702015293	10.23452156	9.570470929
MAFF	7.587005707	7.209485919	7.347416111	7.36298202	7.532708636
MAGEA6	6.909691071	6.569841374	6.683324051	6.691558677	7.110844919
MAGEA8	6.253857596	5.974464051	6.071029081	6.492854313	7.016433495
MAGEH1	8.261007747	8.817758687	9.220211768	8.649944576	8.900779434
MAGOH	7.752687697	7.347734202	6.863661697	7.637262101	7.104098253
MAK	7.896955242	7.122697263	7.307562737	7.196057152	7.227304778
MAL	9.917043434	11.04316622	11.1502402	10.2761618	10.78269205
MAML1	9.151976485	8.881793333	9.67665103	8.890936242	8.886096818
MAN1A1	7.033365899	7.420741101	7.102414636	7.086390323	7.366646525
MAN1B1	7.864861071	8.198176101	6.91172998	7.869396606	8.150892606
MAN2B1	11.28731295	11.46539851	10.65127749	11.51008986	11.36793803
MAN2C1	7.495760485	7.503692152	6.84678303	7.542431869	7.756175162
MANSC1	8.703161646	7.571747222	7.534556657	8.015388182	7.683675283
MAOA	7.072869879	6.477312747	7.134092121	6.864476545	6.694222899
MAP1LC3A	9.318228697	8.617420111	7.849821828	8.880364303	9.246644323
MAP2K1	8.710842768	8.629055081	9.511857455	8.783584889	8.23581501
MAP2K2	10.12224713	10.68960982	8.481851586	10.42409385	10.29324401
MAP2K3	12.87421343	13.26910509	13.62784329	13.12023156	13.03496614
MAP2K4	11.28053313	10.73911267	10.95374716	10.47702836	10.55213977
MAP3K10	7.146069808	7.532439838	7.911309091	7.276913788	7.268778051
MAP3K11	10.36134852	10.31795252	8.945002717	10.21279484	10.11051676
MAP3K3	12.21991364	11.41981305	11.42562271	11.75662781	11.4090051
MAP3K5	10.60743282	10.27139209	10.94208941	10.6313464	10.35699559
MAP3K9	7.392563535	6.838224475	6.893606717	6.81164602	6.817526586
MAP4K1	8.044191424	8.624974828	7.244122677	8.648450303	8.537956091
MAP4K2	8.707515232	8.517695303	7.260895414	8.50111796	7.967524909
MAP6D1	8.647073404	8.846850111	8.35018502	8.213832515	8.789683394
MAP7D1	12.49965499	11.82112126	10.78408516	12.51424883	12.03310273
MAPK1	9.939327313	9.460382818	10.27905981	9.643287657	9.413942061
MAPK11	8.761644081	8.475210818	9.011040293	9.256085253	9.47593803
MAPK12	6.033910394	5.967782929	6.57287004	6.573737152	6.705415545
MAPK14	11.47496104	10.47941256	10.50794857	10.61001127	10.67428006
MAPK3	8.033283465	7.42612497	7.704350384	8.04259702	8.063898495
MAPK9	6.372351394	6.245450939	7.043955899	6.571432061	6.234440616
MAPKAPK2	8.665646899	8.018337859	7.348618394	8.426330949	8.094218354
2-Mar	9.123926859	10.73661507	8.132293172	9.434425707	9.669847545
7-Mar	9.21905796	8.794788636	9.070117818	8.792195697	8.791137414
8-Mar	7.480348535	8.241600556	8.552208182	8.013487535	8.518658667
9-Mar	9.493200828	10.37467131	9.587719636	9.645545465	9.988725727
MARCKS	13.96779242	12.90783816	12.25251148	13.07454735	13.25686564
MARCKSL1	8.140128606	7.400314747	7.776293606	7.683318778	7.374563525
MARCO	9.098746404	9.000069889	9.262561	9.134591697	9.674270303

MARK2	9.127251283	8.950874253	7.843077242	8.849981545	8.430534091
MARK3	9.49850403	9.692043475	10.48782122	10.03026493	10.05320582
MAST1	7.548584576	8.143776848	6.758676202	7.107390485	7.877487515
MAST3	10.06173934	9.17861001	8.797743222	9.979092404	9.122138505
MATK	7.426803586	7.635907081	7.41439101	8.580231323	8.250622253
MBD6	10.55213977	9.589694828	10.71390019	9.759214424	10.96886286
MBOAT2	8.998420707	8.363019899	8.508645727	8.476994374	8.926362727
MBOAT7	11.31661678	10.49713487	10.15483313	10.95374716	10.74172297
MCAT	9.541747455	8.930433505	11.37490296	9.763952101	11.17910752
MCCC1	9.260036485	9.694213162	9.62845904	9.291911616	9.605576293
MCL1	11.83813795	10.90057618	12.54268587	11.79870316	11.49029795
MCM3	9.563605545	10.07670007	9.954681949	9.940265465	9.825199323
MCM5	9.67665103	9.476588061	8.485174677	9.793126455	9.230202616
MCM6	7.967113929	8.307483899	9.20200202	8.36556403	8.690918616
MCOLN1	8.639872707	9.235747242	8.03364202	8.876650909	9.250770152
MCOLN2	6.457858202	6.660321343	7.723384495	6.90271597	7.385541293
MCTP1	7.576037556	6.581495737	7.389870293	7.272914455	7.196285434
MCTP2	9.009407333	8.409959899	8.688838939	8.409548323	8.463578343
MDGA1	6.787317758	6.662035182	5.946203788	6.437856202	6.617782121
MDK	8.189895404	9.164545343	7.639990515	7.356848556	7.994228667
MED12	7.440186081	7.025021455	6.539984525	7.248766182	7.08136002
MED15	8.30620801	8.062539303	7.248451727	8.437457535	8.147147636
MED16	7.253486596	8.300888657	7.133007667	8.252794354	7.883536596
MED18	5.888175556	5.757366934	6.635819404	5.862853758	6.591366
MED24	9.042346232	10.0672481	8.077230061	8.844732879	9.279768394
MEF2D	10.46232362	11.08107282	9.865550636	9.507772687	10.38430498
MEFV	8.226840717	7.785596848	7.289911515	8.03601997	7.660795616
MEGF6	9.028300162	8.768863636	8.847386828	8.429130081	8.356302828
MEGF9	11.05959848	10.12314196	10.47820756	10.45150222	10.23660558
MEIS1	7.096955818	7.716120232	7.599870798	7.802836333	7.616235111
MEMO1	8.228510596	7.931306778	8.767368071	7.731871869	7.710917071
MEPCE	8.623178879	8.409136758	7.694916828	9.022478232	8.618302869
MESDC2	8.697732283	8.947127939	9.737663949	9.240082808	9.045168101
MESP1	7.326634626	7.252299788	7.589604303	7.480029465	7.519364889
MESTIT1	6.449508273	6.178002222	6.344827192	6.242810566	6.72626003
METTL10	6.757434848	7.212383152	7.363288879	6.987320737	7.178905394
METTL7A	8.48873997	9.127872697	9.03221704	8.916479111	9.592426051
MEX3D	11.6445291	12.64265677	10.79284767	11.19447199	11.72355631
MFAP2	6.644999202	6.558125737	6.285113455	6.305155434	6.711872081
MFN2	12.60022648	11.92665931	11.07797425	11.27499716	12.33576886
MFRP	7.55945201	7.515531818	7.346280818	7.491977768	7.285287818
MFSD10	8.267698071	7.779426687	7.439506505	8.304980606	7.997498828
MFSD6L	7.66151798	6.723002525	7.573484121	7.421037465	7.697220202
MFSD7	9.238865747	8.706567556	8.391869283	8.256076333	8.329784131
MFSD9	8.344441616	7.559118222	7.758641747	7.816902374	7.962004212
MGAM	10.23452156	8.382609505	9.889177586	8.951989212	8.675348646
MGAT1	12.99565877	12.81379045	12.02811467	12.70390423	12.27773509
MGAT2	8.66101001	8.78767504	8.823889374	9.101613848	9.179819687
MGAT4B	7.648319737	7.681167667	6.786032343	8.216297535	7.346859747
MGC13005	7.484183869	7.766738848	7.024222101	7.568622485	6.924744576
MGC16121	6.981086404	6.637667434	6.678558889	6.716824636	6.519217414
MGC16384	8.945002717	8.294235768	8.499836475	8.960218374	8.521696414
MGC23284	8.078044465	7.856391758	7.365381232	7.904381697	7.225864141
MGC24103	6.946417495	6.929106091	6.60520404	7.293153485	6.542971677
MGC26718	7.254644071	6.498588101	6.793493859	6.947497737	8.718321818
MGC2752	8.38301802	8.150448232	7.884314808	8.50673198	8.29635502
MGC29506	7.875022121	7.750957677	7.558798354	8.362537455	7.904747051
MGC3207	7.683943101	8.279889293	7.630594101	7.42989803	7.675499444

MGC34800	8.164575929	9.737663949	7.39712799	8.756886111	7.592047838
MGC39821	7.977207111	7.267388222	8.802954374	8.264694495	8.716864899
MGC40574	8.487847515	7.989204111	8.261007747	7.927113394	7.454874889
MGC42157	6.736847222	6.555734869	7.128516444	7.101049919	7.135537212
MGC4473	11.81887725	11.33100665	11.10761793	11.33292491	10.6768734
MGC45922	13.54965001	15.21350605	12.0724233	12.21630458	13.94443
MGC52282	6.275248485	6.408579424	6.511476192	6.720058788	6.678083162
MGC5370	7.916391202	7.660150727	7.569223354	7.26025397	7.564402343
MGC70863	10.97054498	11.31449746	12.42233701	10.98627861	10.91176188
MGC87631	5.827257091	6.256172131	7.265354444	6.570961455	6.198219838
MGLL	8.941795242	9.725631121	10.11849919	10.00002479	8.941795242
MGRN1	13.24400831	12.19727311	11.06123261	12.59323492	12.45390404
MGST1	7.359287172	7.853698798	8.332313525	7.622739535	7.481001515
MGST3	9.855298394	10.61992902	10.3986043	9.879566364	10.72786832
MIAT	8.201303758	8.764299566	8.750916455	8.622644364	8.77608297
MICA	6.718779485	6.676905949	7.842052606	7.318539616	7.130177747
MICAL1	11.47871073	10.68687892	9.606298465	11.50261564	10.94353341
MICALCL	8.552719747	8.860747333	7.416977434	8.006495333	8.742149222
MICALL2	8.32856404	8.354570556	7.799524616	7.697220202	7.834787162
MID1IP1	8.686679798	8.212243444	8.036816444	8.599954677	8.181574081
MIF	12.88708268	14.08841453	12.9709957	12.66985986	13.10484893
MINK1	7.646953505	8.339546939	7.055635202	7.42612497	7.688370495
MKI67IP	8.531247657	8.908935182	9.451212687	9.120933788	9.216574283
MKL1	9.142159707	8.875711838	7.901561475	9.339095222	8.76626204
MKL2	7.200097273	7.884680111	7.046480485	7.302140758	7.469040515
MKNK1	9.612498475	8.16147404	8.453639616	8.897177899	8.558556152
MKRN1	12.30465105	12.43279956	13.54221797	12.86955734	13.15645152
MLC1	8.934777717	8.964642636	8.917682242	9.943097475	8.208692758
MLF2	8.959665061	8.113745737	7.782462626	8.551751717	8.154705535
MLH1	9.216574283	9.467500556	10.042425	9.57872197	9.715215101
MLL4	8.83948901	8.48093102	7.538207606	8.742635939	8.251125323
MLL5	8.738735232	8.540635232	9.563605545	9.165786899	9.253086273
MLLT11	8.024267525	7.52646604	7.59498599	7.166695253	7.424346313
MMD	10.7532413	11.89130603	10.30058205	11.65052704	10.90192126
MME	10.16073454	8.799348343	10.41330878	9.672623929	9.50710198
MMP1	6.319077384	6.234091788	7.300950758	6.211511121	6.086870354
MMP17	11.44643795	13.76108056	8.980943495	10.29324401	10.9660467
MMP23B	7.238133434	7.107390485	6.691055667	7.744142424	7.332963121
MMP25	14.36070414	12.55625498	12.73731843	13.24400831	12.65819872
MMP28	7.788532485	7.756889051	7.635267465	7.693828071	8.107270071
MMP9	15.449005	13.37822002	14.4335752	14.29173122	13.82538175
MMRN2	7.643628758	7.563439404	7.402015111	7.481960152	7.624735818
MNAT1	7.265083586	7.795116566	7.127365253	7.148452788	7.466342606
MNDA	15.03840752	14.31917195	14.60195877	14.9847254	14.77222727
MOAP1	7.11962903	7.050623333	8.282438737	7.446413172	7.221024394
MOBKL1A	8.186373323	8.604635949	7.537489808	8.612168455	8.484723788
MOBKL2A	10.85923156	10.3494333	9.211846273	10.57996368	10.25220538
MOP-1	9.122783717	11.85617239	6.586196364	7.712213273	8.439112919
MORC3	9.727935707	9.501970121	9.670530667	9.759931222	9.950180667
MOS	7.922041747	8.007640657	7.658148485	8.271795141	8.633848111
MOSC1	7.747112485	6.626873192	7.827504414	7.567065616	6.908606212
MOSPD2	8.246087697	7.696527091	7.356281616	7.904747051	7.875433545
MOV10	7.052765889	6.977817737	6.147877354	7.288727879	7.101573333
MPEG1	9.230202616	9.663106646	9.890135131	9.803356646	9.316888828
MPHOSPH8	8.505287909	8.708030737	9.012760899	9.260607	9.37540303
MPL	7.277849899	7.415013667	8.381309202	7.233346848	6.717503818
MPO	7.806122253	7.071486061	7.791885697	8.215875455	7.588938485
MPP1	14.88238784	14.94534759	15.67809237	14.79546777	15.41210544

MPRIP	7.777661384	7.866306808	8.418553323	8.461795505	8.003147788
MPZL1	8.876209859	8.024267525	7.909412687	8.304169929	8.226500707
MPZL3	11.93975968	11.03163187	11.4090051	11.42562271	11.24646499
MRFAP1	10.86305205	10.33375706	11.99323436	11.16734497	10.66706732
MRFAP1L1	10.20634509	9.807061475	11.13240418	10.49348589	10.1684149
MRGPRE	6.591366	6.549824202	6.718779485	6.604752283	6.785769293
MRGPRF	9.439397061	10.14999886	9.106199707	8.85518403	9.303342798
MRPL12	6.818722313	7.44801498	6.58977503	7.661155364	7.736260545
MRPL13	8.63568099	9.159785394	9.276789111	8.924201657	9.083741606
MRPL18	9.493894545	9.627745303	10.59062542	10.15585749	10.10631712
MRPL22	9.484209455	9.716648152	10.31894523	10.25906505	9.87177298
MRPL24	8.390114414	8.836023374	8.823394192	8.294640202	8.620285051
MRPL28	10.83100199	10.47370876	10.3494333	10.56421743	10.86963124
MRPL3	7.247912131	7.536554758	8.35796399	7.562627657	7.700334879
MRPL42P5	6.467810051	7.077700404	6.926979111	6.467159949	6.269932061
MRPL48	8.180785838	9.009407333	8.796713535	8.484240687	8.702574091
MRPL51	10.52247938	10.80900786	11.27153548	10.9991534	11.02851208
MRPS18B	9.899010949	10.31597323	11.21668249	10.47584162	10.59924244
MRPS2	9.526870273	9.482964828	9.66613299	9.630502939	10.04682541
MRPS23	7.076304455	7.005077596	8.067838768	7.281402384	7.166949475
MRPS24	10.83363242	11.52824	11.65918316	11.07337622	11.30131952
MRPS35	9.014335657	9.708693071	9.333986848	9.387059283	9.658049545
MRPS6	10.06547375	10.11150219	10.3305428	10.09597734	9.881912677
MRPS9	9.445197818	9.619644949	10.37467131	10.03123363	10.25811169
MRTO4	7.13936198	7.343750657	8.39227803	7.554702939	7.456765576
MRVI1	9.329922273	8.234232515	9.037153444	8.100222556	7.815211869
MS4A1	6.973155374	7.013137848	7.410002717	6.818245747	7.165793091
MS4A14	7.142295949	7.71023803	7.448310273	7.584276586	7.539809273
MS4A2	6.785769293	7.153495505	7.464044657	6.618022394	6.165805071
MS4A4A	6.824838515	7.242650333	7.657256192	6.922161646	7.306475687
MS4A6A	9.305189859	9.34289298	9.777520899	9.061649657	9.272374697
MS4A7	8.534897646	9.333361616	9.998242414	9.397189192	9.197870838
MSH2	7.392836818	7.613815293	8.078774939	7.520306162	7.528004313
MSI2	8.34368697	9.005433152	8.918756414	7.973992111	9.21067496
MSL1	9.910960495	8.879851283	10.30695028	9.490338525	9.362770111
MSL2	7.926300626	7.708254091	6.372351394	7.801388869	7.955993172
MSLNL	7.465356566	7.000309313	7.440763838	7.32157099	7.917722121
MSRA	9.669847545	9.870576439	9.267383556	9.476588061	9.186711646
MSRB2	10.86173253	10.71279176	11.26623333	11.14211434	10.76471511
MST150	10.66945215	10.22267877	10.0719806	9.998242414	9.769994879
MT1A	10.15379546	10.2187154	11.01683173	10.23752804	10.75737971
MT1B	9.75438504	9.840616414	10.2026692	9.465659495	10.45754412
MT1E	8.677707162	8.405355091	8.658802051	8.436545051	9.30949301
MT1G	9.29794999	9.190205293	9.86223396	9.207791152	9.666877556
MT1H	9.638309758	9.453743545	10.21769501	9.579413505	10.0719806
MT1L	9.94767803	9.762542495	10.687913	9.837575172	10.70425349
MT1X	9.422377343	9.332795131	10.11760527	9.402230293	9.991229606
MT2A	10.91356479	11.32027501	12.51066587	11.63401423	12.7452022
MTA1	9.118629636	10.07857135	8.775001677	8.870602515	9.053706202
MTHFS	11.90152869	10.85513517	11.50425191	11.63629658	10.99623862
MTMR15	7.801818071	8.288815495	8.971727333	8.045324071	8.573727141
MTMR8	6.815473091	6.235363323	7.582654121	7.094450071	7.309928404
MTPAP	7.283275424	7.427372788	8.049019253	7.307847101	7.70299303
MTPN	11.77305263	11.83072395	11.62739451	12.12692782	11.85301976
MTRR	6.831893455	7.033365899	6.810861495	6.665912939	6.529766576
MTSS1	7.379463242	7.909763434	7.923048354	7.845981061	7.748187364
MTX1	9.954681949	9.733230051	8.57597697	9.436898869	9.094076404
MTX2	7.126519737	7.331457121	8.531247657	7.439506505	7.560118414

MUC2	7.616235111	8.049757475	7.574489596	6.929106091	7.487351192
MUC20	7.585232141	7.946933485	7.049798838	7.176396859	6.968936475
MUC3A	8.192996515	7.188870657	7.708578687	7.660795616	6.82246696
MUC4	9.116279576	9.517771202	9.346206162	9.013830768	9.439397061
MUC6	7.274095485	7.42989803	7.629989283	7.455822202	7.845242687
MVD	6.931701566	7.304428303	6.535075677	7.336756222	7.423442323
MX1	11.73070023	11.29744903	11.75462574	10.78987961	11.2345748
MX2	10.81664077	9.938475404	10.28406896	10.23247836	9.965319232
MXD1	13.02006671	12.14789109	12.54589185	12.91196246	12.61090773
MXD3	8.909404788	7.939228495	8.221010253	8.525321657	8.466446051
MXI1	11.04156144	12.09804166	11.22947614	11.75462574	12.43944324
MYADML	7.533990333	6.628304848	6.506975192	7.102414636	6.512566313
MYB	8.099112939	8.867411152	8.183195545	7.843485626	7.55945201
MYBPC2	6.959689121	7.454572909	6.623632253	6.908859909	7.060512141
MYBPC3	9.860587788	9.203189636	8.773471869	9.145664475	8.851571051
MYBPH	9.377908	10.65871503	9.237664879	8.961327374	8.896578162
MYCN	8.244805758	9.330464051	7.180559343	7.635576919	8.155735586
MYD88	9.009915859	7.68296396	8.229339455	8.868979273	8.186373323
MYEOV	10.07857135	9.694925798	9.062782333	9.425521616	10.13276962
MYH14	9.056533626	9.692793657	8.725113212	8.595703707	8.951989212
MYH9	10.93334198	10.29324401	9.574050242	10.72246175	10.21975253
MYL4	9.106823152	9.971733293	9.148511889	9.474605101	9.739033131
MYL9	7.079910848	8.229734556	6.371420535	7.563777768	6.783369808
MYLIP	9.895061889	8.686679798	10.10093339	9.624391323	9.129654758
MYLK	8.757866131	9.027655758	8.360501364	9.203189636	8.35018502
MYO15B	8.024633293	7.870978364	7.156444778	7.890421556	7.614497747
MYO1F	13.17281418	11.97944932	12.29548604	12.78645268	12.16036216
MYO1G	9.588410576	9.811898121	9.038849273	10.2572153	10.1597097
MYO9A	8.399975414	8.622197111	8.534432515	8.594455404	8.613521394
MYOD1	8.514909313	10.2692332	7.931708101	7.875022121	8.235056909
MYOF	6.729463253	6.998704808	6.756145354	6.661016606	7.01565997
MYOG	9.584803364	11.1060681	8.796283929	8.523456192	10.18000397
MYOM2	8.648450303	8.909945455	6.849836323	8.490531303	8.048299333
MZF1	8.810446485	9.124970051	7.975072535	8.471488636	8.464558212
N4BP2L1	7.919459465	7.973992111	9.078885242	8.301657889	8.265130232
NAAA	8.79576798	8.940148162	9.270407879	9.876096838	9.591133828
NACA2	13.12023156	13.18858184	13.70143341	13.48401144	13.93694444
NACAP1	8.937429899	9.802496737	9.616741535	9.211846273	9.726443586
NACC2	9.277455131	9.540446515	9.903372323	9.117478424	9.234418071
NADK	10.81540317	9.70034896	10.17789322	10.59541023	9.686378667
NAE1	8.865407737	9.384078242	9.039454747	9.018802576	9.06333597
NAGK	11.86922315	11.16888788	11.87445957	11.96636849	11.21493335
NAIF1	8.381754576	8.117339677	8.793874616	8.389243343	8.872352707
NAIP	8.990775909	7.939903758	8.567346869	7.849137545	8.772410586
NAMPT	13.87958112	12.72219022	14.49002723	13.65450155	13.36582475
NAPA	7.97128602	8.099112939	8.097958939	8.153957263	8.221403505
NAPRT1	10.5397012	10.3918566	10.02269473	10.48897537	10.5752557
NAPSA	8.328154263	7.656544636	7.881102323	8.606099333	8.558108202
NAPSB	9.588958727	8.834473929	9.433258586	10.56306707	10.61992902
NARS	9.369545939	9.660200838	9.379180747	9.36748	9.287277576
NAT10	11.56725755	12.37416009	11.34381562	11.57567047	12.17829278
NAT14	10.05320582	9.101613848	9.659537192	9.302103889	9.731150899
NAT15	6.960749798	6.872611121	5.996322707	6.770374545	6.727165222
NAT8B	7.553468394	7.339519202	8.319524414	8.206690121	7.253230737
NAT9	9.805520727	9.816676162	9.494490616	9.990505182	9.707282667
NBEAL2	9.226702434	8.775001677	7.862160495	8.682101313	8.560860505
NBPF10	10.91176188	10.37573078	10.88031852	11.4090051	10.85513517
NBPF14	9.061091758	8.85049999	9.864614818	8.893419636	8.991970091

NBPF15	13.47832004	13.18348162	13.05428976	12.80949032	13.20018245
NBPF20	9.844817354	9.451212687	10.25906505	10.25220538	10.21484784
NCAPG2	6.105179566	6.422006566	6.103424768	6.49370698	6.567185838
NCBP2	7.67197899	8.027802949	8.352537101	7.575132364	8.192996515
NCF1	13.80704919	13.3718573	11.05493438	13.77352346	12.68903729
NCF2	15.50564301	14.47046648	15.06622261	14.91973608	14.65397593
NCF4	11.23116994	10.36687712	10.16165443	10.54950911	10.3380268
NCK2	7.511581475	7.66151798	7.820222687	7.577325354	7.992269263
NCL	10.13958591	10.43440935	10.5152666	10.72900488	10.761049
NCOA4	11.03452834	11.04464523	11.89628941	11.542568	11.24993851
NCR1	8.408296424	7.525550222	8.01423197	8.181963182	7.835434535
NCR2	6.440833869	6.265911495	6.707842586	6.112680475	6.102848495
NCR3	8.457043091	8.522641273	8.919862162	9.214694788	8.610845737
NCRNA00084	9.885156737	9.758431737	10.40701089	9.721175788	9.335891293
NCRNA00094	8.63611798	8.986932636	9.40167604	8.464558212	8.743124222
NCRNA00115	7.114036293	6.774936697	7.261824374	6.718530879	6.719039212
NCRNA00120	7.716479172	7.772503354	6.227863606	7.15072903	7.331138333
NCRNA00168	7.657256192	7.886079293	7.111963455	6.883793818	7.376100929
ND1	10.41330878	10.66105788	10.76239578	10.21375666	10.21583282
ND2	10.72127155	11.07647857	10.88945836	10.82153564	10.41846051
ND3	12.08943903	12.18142186	11.93486375	12.11216258	12.00354228
ND5	8.365106485	8.452737606	8.342773172	8.273483707	8.498167798
ND6	9.709426838	9.753529758	10.95849458	10.20060626	10.60277882
NDEL1	9.187808616	8.197369283	7.937868929	8.793874616	7.916025434
NDOR1	12.41634374	13.56234785	11.58005536	11.76804793	12.58957491
NDRG3	7.398020586	7.096955818	7.512168657	7.540114586	7.658148485
NDUFA12	9.664626495	9.973623707	9.590351455	9.675185677	9.749004051
NDUFA3	9.658049545	11.11230451	10.59292728	10.1421691	10.60161194
NDUFA8	10.26224218	10.81796524	11.35278885	10.44832821	10.53247367
NDUFB2	11.32185019	11.90152869	12.32024961	12.04432	12.07810634
NDUFB5	7.046194354	7.164674212	7.62575301	6.963370131	7.012842909
NDUFB6	10.60161194	10.54190997	9.736957071	10.31997281	10.33146686
NDUFB7	9.777520899	10.86447048	10.06071544	10.20748734	10.31289083
NDUFC1	10.86566339	11.03008027	11.99535117	10.80389925	11.37131374
NDUFC2	8.509065354	8.939630657	8.662363172	8.852576737	8.964642636
NDUFS7	10.77762269	11.50632759	11.13400706	11.30690717	11.59654933
NDUFS8	8.747892899	9.597513697	8.834473929	9.384078242	9.626304798
NDUFV1	10.16165443	10.66706732	10.29101087	11.10035391	11.06573849
NEBL	5.776122162	6.104869535	5.779716121	5.761454253	5.764170636
NECAP1	7.841729333	7.38340502	8.378515131	8.134366646	8.122341
NECAP2	8.53537201	8.33273403	9.466304111	8.83948901	8.721523222
NEDD9	8.157901343	7.539167263	8.397394545	7.958031758	7.890755525
NEFH	7.427999919	7.425828556	7.684965778	6.965182525	7.308145939
NEIL1	8.883414061	8.863375313	9.521192909	9.264483889	9.286739242
NELL2	7.454572909	8.344098929	9.056533626	8.317411434	8.217270354
NET1	7.21931098	8.105207222	7.739314727	7.383711768	7.576037556
NEURL	7.319114313	7.562627657	7.315791616	7.641985	7.661155364
NEUROG1	11.35858946	13.60269863	8.783158111	10.58236152	10.60863639
NEUROG3	11.33477589	12.85048632	9.091299889	11.65698449	11.32721575
NEXN	8.659649677	8.505287909	9.604846424	9.302735434	8.789132293
NF-E4	10.89604789	9.642500081	9.617464455	10.34514895	10.2600862
NFIA	8.133538364	8.775519707	7.446760616	7.842683778	8.626355717
NFIC	6.798121798	6.783830859	6.788765101	6.417030697	6.801276
NFIL3	12.15440913	10.82664672	11.45176908	11.65455063	11.24323027
NFIX	7.711523556	8.270601414	7.846324646	7.944736172	8.421350475
NFKBIA	13.46712151	12.91656586	13.12023156	12.96662998	12.77015535
NFKBIB	8.50473503	8.090488788	8.005727263	8.490053091	8.612685364
NFKBIZ	9.905108616	9.473222667	8.59942799	9.669114061	9.779012071

NFXL1	5.980254848	6.461471737	6.261769939	6.178813899	6.642822768
NGDN	9.241421747	9.509226798	10.80782516	9.701165495	9.862946677
NGFRAP1	11.09565519	12.21991364	12.28105924	11.73070023	11.64848719
NGLY1	8.997224192	8.773988747	9.641007131	9.547339828	9.664626495
NGRN	7.36203996	7.352900667	8.441767061	8.029005444	7.690204818
NHLH1	6.034325333	5.803172081	6.150621626	6.205248909	6.504270879
NHS	7.913871889	7.741148566	7.880363606	7.559118222	7.14635899
NINJ1	13.18858184	12.40735915	11.76804793	12.49170848	12.05489479
NINJ2	9.807061475	9.535633242	10.07107569	9.317615606	9.238221677
NIP7	7.528303323	7.688677182	8.792195697	7.680542242	7.846992646
NIPSNAP3A	7.794719919	8.518144394	8.31214998	7.709511535	7.696527091
NISCH	8.877626323	8.59655801	8.141770949	9.146178758	8.756886111
NKD2	9.566949	10.4450719	7.800624061	8.925229707	9.373397364
NKG7	11.1031408	11.12613667	11.13551505	12.02514953	11.65455063
NKIRAS2	8.573727141	8.215082485	8.003147788	8.38301802	8.409548323
NKX1-2	15.92931651	16.22759729	15.99508933	15.50564301	15.86132141
NKX2-3	7.332963121	8.015388182	7.223856	6.845030273	7.501443394
NKX2-5	8.385303111	8.350987111	8.08972896	8.153159131	8.297630758
NKX2-8	10.32849396	11.62290433	10.24828231	10.10278369	10.54692936
NKX3-1	6.866319192	8.609106909	6.584832545	8.706127333	6.882764848
NLGN2	6.846305687	7.018881949	7.135222657	7.25519196	7.005900293
NLRC4	10.28795108	9.541747455	9.946747141	10.19957169	10.042425
NLRC5	8.549141545	8.196571586	7.61856696	8.498503737	8.355387535
NLRP12	10.87907816	9.880317061	9.768390455	10.90057618	9.879566364
NLRP3	10.2600862	10.77387872	10.23752804	9.976273051	9.996420616
NLRX1	11.11714857	10.31013098	10.60044575	10.47242114	10.07956055
NMB	8.859179455	8.781680606	8.964642636	8.169382444	8.431326525
NME1	8.625920364	9.201355202	9.902579253	9.013314737	9.287819051
NME2	11.52238659	12.02811467	11.96059797	11.79405389	12.19727311
NMI	12.09804166	11.5612151	11.88148631	11.55322016	11.8164662
NMRAL1	9.141588162	9.980047091	9.395177889	9.967178364	9.681893121
NMUR1	8.323157747	8.044608626	8.333227545	9.249868985	8.437457535
NOC3L	7.932066141	8.421973258	8.245649515	7.857447394	8.062539303
NOD2	9.198999222	9.49850403	9.511202364	8.559011545	9.505859384
NOL9	6.794878162	7.132751192	7.616552222	7.183662697	7.262957343
NOMO1	7.338325414	6.890508141	7.332669495	7.322217273	6.69201996
NOP10	13.23341018	12.15752365	12.60022648	12.25844332	12.17513939
NOP16	9.337187111	9.785178838	10.28005063	9.62024901	9.606298465
NOSIP	10.42927797	10.73132595	10.02161965	10.72786832	10.61261659
NOTCH2	12.72972704	12.03310273	12.42616911	12.44611991	12.32024961
NOTCH2NL	8.505797091	8.314030071	9.373397364	9.06333597	9.051957646
NOV	8.334981101	8.316969364	8.995099212	8.194999071	8.006915303
NOVA2	10.82945836	12.58287167	10.07857135	10.05426836	11.41415047
NP	12.052647	12.41634374	12.92081703	12.3843369	12.69316533
NPAS3	10.69470446	11.24147284	9.660861778	10.5029511	10.72384747
NPBWR1	9.303966081	10.33146686	8.653906556	8.970512081	9.46096901
NPBWR2	6.805229596	7.417927455	6.289373162	6.910693596	7.404985061
NPC1L1	8.979298788	9.391460545	8.825312061	8.522156747	9.084401768
NPC2	12.68903729	12.63500423	13.41514643	12.92542832	12.94367069
NPCDR1	6.982654747	7.935875313	7.349811747	6.889757091	7.095808323
NPDC1	8.766842081	8.889922455	8.424471616	8.407442505	9.118059475
NPHP1	7.343750657	6.348622303	7.646666444	7.646953505	7.864535646
NPHP4	7.424653535	7.260583141	7.102116364	6.956131545	7.167804919
NPIP	11.37670858	11.58814301	10.77637516	11.43773109	11.34381562
NPL	11.92937262	11.71895032	12.10951308	11.43380494	12.01191659
NPLOC4	8.742635939	8.649462798	7.459115707	8.806450121	8.370422404
NPPA	5.750703263	5.756206495	5.75338203	5.751544727	7.12541897
NPSR1	7.007051354	6.407880727	5.888908071	6.236632616	6.770374545

NPTN	8.202220545	8.07563398	8.363862202	8.272231939	7.74753198
NQO2	9.748306121	9.472030465	9.020821455	9.857104919	9.395854121
NR1H2	9.048100778	9.264483889	8.059423535	8.589388657	8.864430475
NR4A1	7.041650374	7.091067131	6.864476545	7.36510997	6.868868606
NRBF2	9.169326323	8.662363172	8.717867636	8.531655515	8.121211444
NRBP1	9.419654929	9.224452333	8.493653778	9.53690398	9.048100778
NRGN	12.13244857	13.05987966	10.66229262	12.9851923	11.88909817
NRN1	10.4302696	11.00754401	9.909202788	9.968215758	10.573928
NRSN2	8.226500707	9.333986848	7.082160636	7.483574061	8.104500051
NSMAF	10.8935809	10.11664425	10.74690378	10.4283734	10.45996909
NSUN2	10.0406788	10.31193963	10.52476206	9.867911152	10.06255975
NSUN3	8.308776677	8.717867636	9.362238061	8.517695303	9.660200838
NSUN5	10.33476777	10.23660558	9.545191596	10.32418729	9.962571606
NSUN5B	10.40486524	10.41530743	9.975431768	10.86963124	10.58469265
NSUN6	6.555521899	6.716118697	6.909401626	6.988144556	6.88247998
NSUN7	7.291068525	6.760600303	7.014205707	6.400341677	6.471881182
NT5C1A	6.362493667	6.153812061	8.186741323	7.028571808	7.161605929
NT5C2	9.179819687	8.586230848	10.14631732	8.927019859	8.631321253
NT5C3	9.990505182	9.905108616	10.71031814	11.20660223	10.97054498
NT5M	9.415860727	9.895856061	9.165786899	9.562994313	10.52135963
NTN3	8.483763566	8.925792808	7.97128602	8.568242293	8.849981545
NTNG1	6.512799333	6.686049697	6.456691768	6.434897949	7.29540803
NTNG2	11.17041501	9.776839404	9.237064687	9.715215101	9.739859192
NUAK1	6.333774687	6.159344697	6.677139455	6.529524889	5.980732253
NUAK2	10.50794857	9.884409465	9.793126455	10.04511943	9.49850403
NUB1	7.379145515	7.925642	6.909921869	7.165241424	7.280239242
NUBP2	8.134366646	8.336651323	7.494168788	8.674900657	8.252372808
NUDCD2	8.761177727	9.397864101	9.124449354	9.229082869	9.50650501
NUDT1	9.827596687	10.27338352	10.3810842	10.0178996	10.09994749
NUDT2	7.544716788	8.245222778	7.712213273	8.414282596	8.045722828
NUDT22	9.007843273	10.43122177	8.089360869	8.650384111	8.883414061
NUDT7	6.275054576	6.756647869	6.414872495	6.474296596	6.661815061
NUDT8	7.645321707	7.909412687	7.68740998	7.816254515	7.991026727
NUDT9	7.130177747	7.342871717	8.093077828	7.605411525	7.770465818
NUDT9P1	6.853963192	7.344278889	6.818463323	6.878445485	6.979123747
NUMA1	8.127495596	7.832891081	7.247290424	8.27018399	7.806122253
NUMB	10.96000427	10.46664928	10.73661507	10.59180142	10.53842125
NUMBL	9.341222778	10.40278823	8.663303283	8.510413414	9.856174394
NUP107	9.400967465	9.800904051	9.805520727	9.010430586	9.516398909
NUP133	8.438325768	8.742149222	8.736221495	8.573273283	8.857327323
NUP214	9.637663091	9.243096889	9.061649657	9.453123465	9.285586323
NUP35	7.618887111	8.236633323	8.06213903	7.90505396	8.032499636
NUP54	8.11773804	8.590695768	8.466852202	8.741130788	8.621689657
NUP62	10.83501466	11.41415047	11.30301738	11.24323027	11.20840189
NUP88	7.751275798	8.057063626	8.570262	8.191573747	8.599954677
NUSAP1	7.589252838	8.611286687	7.373983485	8.102124455	8.170586434
NXF3	8.447526838	7.773938747	8.075284384	7.710578727	7.607714535
NXNL1	7.704350384	7.517772121	7.486746556	7.415639222	7.623693616
NXPH4	7.783465061	8.728878424	7.030955384	7.525015414	7.918472515
NYX	14.68633385	15.56435861	14.06476988	13.7211242	14.63205333
OAS1	7.370950798	7.125146293	8.047188455	7.767362636	7.361082222
OAS2	6.23392001	6.469182939	6.195084242	6.402255576	6.291675828
OAS3	6.996297444	6.977525061	7.156711091	6.870354606	6.952529081
OASL	9.801802283	9.706432808	9.250125475	9.255548899	9.836777707
OAT	8.753420071	8.078389566	8.217270354	8.251125323	8.074096707
OAZ2	12.02514953	11.39983314	11.63629658	11.90674449	11.71244686
OBFC2A	9.750460616	8.993505475	9.601568657	9.152615889	9.082448343
OBP2B	6.283373303	6.582941808	6.938883283	6.973893828	7.013654717

OCC-1	7.243557061	7.420458273	8.366003535	7.687096788	7.866656354
OCIAD1	10.54441943	10.70052069	11.3733012	10.82034332	10.73788216
OCIAD2	10.16262583	10.88031852	11.46320141	10.66832974	10.93334198
ODC1	11.33848921	12.54589185	13.2504616	12.34855768	12.84627851
ODF3L2	7.850535525	8.421350475	7.456765576	7.648986848	7.761767192
ODF4	6.775746758	5.954034667	7.124332475	6.781179727	6.747571626
OGFOD2	7.333224889	7.531770848	7.020179424	7.573788949	7.37546403
OGFR	14.93426708	15.88528111	12.80602179	14.05855866	14.78379092
OGFRL1	10.83930015	10.85100254	10.76609029	10.8254891	10.43998577
OGT	9.229670424	9.147897788	9.293710889	9.160413333	9.11510898
OLFM4	6.82246696	6.229099566	7.701029869	8.166602566	6.656675747
OLFML2B	8.671783687	8.657372182	8.498949848	8.731005677	9.001802606
OPLAH	9.251906182	7.926300626	8.690381253	7.705814323	8.878776172
OPN1LW	13.48990673	14.11997744	12.47130519	14.11151028	14.19920898
OPN1MW	6.983407384	6.422427222	6.817996071	6.625311455	6.324531404
OPN4	7.241538444	6.712342556	7.078303869	6.877874525	7.169717707
OPRL1	9.465071586	8.102124455	9.715215101	9.727935707	9.849671929
OPTN	13.55640443	14.11151028	13.90036211	13.58996905	14.09521817
OR10A4	9.333986848	8.920488949	8.844732879	8.60808904	9.229670424
OR10A5	7.283562505	6.84678303	7.82475102	7.721939444	8.317411434
OR10G8	6.818245747	6.118230293	6.555521899	6.925009455	7.319114313
OR10H2	11.92156179	12.90359001	11.67593314	11.44491584	12.05813886
OR11A1	8.060661899	8.002717273	7.38862201	7.210091465	7.882525919
OR1L3	7.621361828	6.652192667	7.717737636	7.040799717	7.674109172
OR1S2	6.439328828	6.403298808	6.753469455	7.009733586	7.656544636
OR2A25	7.323648515	6.334404253	8.120448121	7.572483949	8.616405606
OR2H1	8.130993828	8.498503737	8.147147636	8.077230061	8.263041909
OR2W3	8.358783152	7.528986283	9.143918798	8.882882525	8.739635162
OR4C3	6.789988131	6.376787985	8.245222778	6.979690485	6.97994296
OR4D2	8.485631939	9.022478232	9.137373131	9.648698737	9.169326323
OR4X2	6.234838515	6.587110859	6.392953667	6.415875869	6.615899091
OR52B2	6.675450515	5.988361071	6.550797374	6.429483727	7.093823343
OR52K2	7.291354758	6.887288828	6.344188131	6.977525061	6.888392616
OR52K3P	8.264694495	7.416977434	7.886480404	7.504556444	7.422831253
OR5AP2	6.827413455	6.082207485	6.025707828	5.774889455	6.495899485
OR5H1	7.110215707	6.577521747	6.900739596	6.962034242	7.116341071
OR5L2	6.895709172	6.969919475	6.520809899	6.036996596	6.38666303
OR5V1	7.729749768	8.256499515	7.235668455	6.975683768	7.197763283
OR6K2	6.532632323	6.263522859	6.278089182	6.597486293	6.982654747
OR6W1P	7.784888444	8.719668061	7.16240796	6.987890152	7.352232505
OR6Y1	6.061764141	6.000819535	6.452349424	6.339864232	6.697417434
OR7E24	8.171788646	7.841440677	7.540114586	7.532439838	8.200163707
OR8U1	6.817526586	6.205092606	6.827154646	6.74001704	6.912798808
ORAI2	10.18563382	9.53973704	8.693780273	10.17789322	9.721175788
ORM1	10.11849919	8.139691131	8.967408879	10.13375911	9.57181996
ORM2	8.992957212	7.32489101	8.035288182	8.902484929	8.271795141
ORMDL3	11.07493153	11.49420288	10.20358283	10.71145754	11.3290504
OS9	10.26632574	9.505859384	8.78767504	10.22951336	9.782133848
OSBP2	7.440763838	8.517288667	7.310482798	7.939609869	8.510413414
OSBPL2	9.70034896	8.80133401	9.664626495	9.465071586	8.977005354
OSBPL5	10.35591393	10.63391466	10.65751844	10.91176188	10.2761618
OSBPL8	9.59460096	8.594016788	8.877161475	8.813595202	8.84935699
OSGEP	8.779146061	9.174793152	9.135618929	9.196689051	8.960773354
OSM	9.316888828	8.052077899	8.525321657	7.940254566	7.523756919
OTOF	9.841562192	9.942152202	9.813557354	9.583475303	9.722667242
OTOS	7.764931778	8.490531303	7.053026414	7.174922929	7.876138384
OTP	7.651926253	6.534012141	8.947127939	8.193820172	8.47014499
OTUD6A	6.833358384	6.841827051	6.304387152	6.528194515	6.58977503

OTUD7A	7.919797354	8.367840636	7.738299929	7.610574101	7.74961303
OTX1	7.133298475	6.477937869	6.659807838	6.880211293	7.147555404
OVOS2	6.82018896	6.392119051	6.492652182	6.375715869	6.384310424
OXCT1	6.756883091	7.074397545	7.737346818	7.194952889	7.430156263
OXER1	10.33375706	9.294355394	9.856174394	9.769994879	10.52799495
OXNAD1	7.487078798	8.104133364	8.77298297	8.144908747	8.261007747
OXT	8.203586121	9.475206131	7.841061444	8.15355797	8.76328402
OXTR	7.669657737	7.620272192	7.670275758	8.467263475	8.497692788
P2RX1	8.491490828	8.044191424	7.068665737	8.096128192	7.448630646
P2RX2	11.33292491	9.715215101	10.42409385	10.26541026	9.132651727
P2RX3	8.428176394	8.437457535	8.451550535	8.258465414	9.012031232
P2RX7	7.023140616	7.495088838	7.215770273	7.475955232	7.546918172
P2RY13	9.650789909	9.317615606	8.082337909	8.909404788	9.026117657
P2RY14	8.372772768	9.290785283	8.025864485	7.953126222	8.69919198
P2RY2	8.084275677	8.251492909	8.543686687	7.832217929	7.432695485
P2RY5	8.354947869	8.689808788	8.917069505	8.441767061	8.867918475
P2RY8	9.474605101	9.429506515	9.464440242	9.928455091	10.00886723
P4HB	10.98350754	10.7982674	10.71992703	11.28370047	10.93646713
P704P	6.55412801	5.845420465	5.783851485	6.334799414	5.878796525
PABPC1	13.56886427	13.47327752	14.42673578	14.15092087	14.20688311
PABPC3	10.4687107	10.9991534	10.97631922	10.60635433	11.40145091
PACRG	7.594027929	7.91465098	7.358075697	8.657372182	8.72650097
PACS1	8.72650097	8.330618758	7.729749768	8.682574626	8.37004801
PACSIN2	8.486968232	8.083135202	9.096395636	9.060618192	8.596127869
PADI2	7.638274606	7.613240606	6.663425212	7.773582384	7.122138172
PADI4	8.326162172	7.340147596	6.925244263	7.957018646	7.225301495
PAF1	8.805485081	8.338686465	7.946141838	8.933432389	8.472435657
PAGE2	9.481668929	9.855298394	10.05880999	9.158666485	10.13768673
PAGE2B	7.731871869	7.831854586	7.916025434	7.312252182	8.213832515
PAGE5	9.540446515	9.712341424	9.918671212	9.090721626	10.28005063
PAIP2	12.87819387	12.23769992	13.23341018	12.70787607	12.34855768
PAK1	9.61181703	8.342773172	9.41003803	9.731806091	8.834473929
PAK2	9.323195364	9.094076404	8.903602263	8.834473929	8.768300525
PAK4	7.523206263	8.047553323	7.064538818	6.866852303	7.253764222
PALLD	7.499887141	7.662925475	7.490430596	7.631297323	7.109400202
PALM	7.609232899	7.262118616	7.608647091	7.288128061	7.532439838
PALM2-AKAP2	13.56234785	12.70787607	12.88265743	14.1904887	13.61616406
PAM	7.158043384	7.181699919	8.316570081	7.119927869	7.575754657
PAN3	9.818059192	9.424273283	10.09896607	9.461673394	9.292435667
PANX3	6.977525061	6.628772899	6.325549091	6.125146475	6.455790798
PAPSS1	8.163053768	8.443175586	9.102676354	8.333227545	8.602379626
PAQR6	8.928828384	8.826393101	9.860587788	9.089024899	9.629839465
PAQR9	6.148359626	6.727165222	7.038073394	5.864857505	6.990260788
PARD6G	10.7814083	11.0859227	9.21352099	10.37573078	10.49348589
PARP1	9.53690398	10.01974654	10.47479655	9.634173323	9.969141677
PARP10	11.93770961	12.11562205	10.54692936	11.66441291	11.66708917
PARP12	8.170954818	7.973586899	8.300483646	8.403029636	8.292509485
PARP2	7.189144101	7.478803566	7.45822897	7.334394303	7.44049799
PARP6	8.315227333	8.268510495	7.868335495	8.753420071	8.396532182
PARP9	9.869466131	9.347406172	10.24942024	9.354289121	9.613873859
PARVB	7.007603253	7.022843394	7.012040828	7.616552222	6.90271597
PARVG	9.416442616	8.867918475	8.253639283	9.685649929	8.875206121
PASK	6.890761263	7.680240364	8.994589434	8.242002535	8.412145273
PAX4	7.135537212	7.02909003	7.749943687	7.096340343	7.172125253
PBX4	8.948168879	9.470840162	9.742174424	8.90531298	9.404880263
PBXIP1	9.418269535	9.447242061	8.438325768	9.376667182	9.063885091
PCBP4	8.442251212	9.078885242	8.970512081	8.740195303	8.934777717
PCDH24	7.281402384	6.840661414	7.305287707	7.140902202	7.078018687

PCDHA1	6.825072616	5.862520848	6.40434199	6.416564263	5.936245222
PCDHA5	7.718373848	6.752477677	6.877874525	7.037248667	6.817799576
PCDHA8	6.628047111	6.679714263	5.990636495	6.318093747	6.331115141
PCDHB13	7.690204818	7.693828071	7.572084505	7.551492101	7.788172758
PCDHB4	6.71590199	6.087627818	7.090185162	6.599487732	7.180291929
PCDHB7	7.488608758	7.274624242	6.479663677	6.70156496	7.773938747
PCDHGA2	6.783369808	7.112898869	6.463778677	6.449932949	7.028809465
PCDHGA7	8.736738475	8.790130253	8.766842081	8.019097616	8.069753788
PCDHGB1	6.164144475	6.044883525	6.70156496	6.368728152	6.731752374
PCIF1	8.334587636	7.477539293	7.398696909	8.282900293	7.803193929
PCMTD2	9.675185677	8.792768747	9.260036485	8.805485081	9.370225242
PCNP	10.41022215	10.7442872	10.5752557	10.75600216	10.60635433
PCNX	9.76471501	8.655934636	8.808928141	9.08959502	9.222631263
PCP2	8.812517081	8.975268424	9.046862848	9.069558111	8.974650333
PCSK1N	11.03296099	13.00581854	9.297353606	9.839111	10.5431016
PCSK5	8.602859283	8.453185455	9.140978061	8.822318	8.627322343
PCSK7	7.21289504	7.156966303	7.245884828	7.512168657	7.197446212
PCTP	11.12454893	10.6240143	10.53028115	10.65751844	11.81018832
PDCD1	8.728449242	8.825312061	8.970028374	8.51269104	8.491490828
PDCD5	7.735250384	8.193428737	8.431738505	8.379056919	8.899757485
PDE4C	11.5612151	11.1031408	12.17216641	11.93486375	11.78023213
PDE6D	7.069787606	6.709545626	7.888681737	6.921417374	6.920433444
PDGFA	7.819446808	8.161033919	6.937364475	8.197004869	6.913788323
PDGFRB	6.567922788	6.403697939	6.136533313	6.360466222	6.109379758
PDHA1	9.780583495	9.752732475	10.21375666	9.995698596	10.01340346
PDIA4	7.846324646	8.254919778	8.460863192	8.520243879	8.607126586
PDIA6	8.484240687	8.907915899	9.727055768	8.764299566	8.86024204
PDLIM1	7.202436384	7.042386798	7.689906808	7.735932323	7.062669222
PDLIM4	7.385541293	8.747331152	7.271416848	7.505451222	7.662925475
PDZD11	8.909945455	8.33796001	8.84786598	8.859179455	9.493200828
PDZK1IP1	12.46055504	13.66784462	12.21991364	13.18858184	12.40735915
PEBP1	10.40810668	11.1276132	10.93935201	10.91641235	11.39382595
PEF1	10.29324401	10.080472	10.96162926	10.73911267	10.91356479
PELI1	10.23948175	10.156858	9.447991384	9.562364657	9.426842081
PELI2	10.37346225	9.992988556	9.845595727	9.989520798	9.930141081
PELP1	7.885731566	8.567801737	7.819085697	8.661956242	8.481411768
PEPD	9.07419996	9.721175788	9.459003	9.076382242	9.305189859
PERP	6.510269939	6.872867768	6.587993828	6.848028121	7.165241424
PEX26	8.804952828	9.077100232	9.147897788	9.049629657	9.575401111
PEX6	8.729972586	8.205133263	8.275579182	8.680072242	8.331888859
PF4	13.75520609	13.78737352	14.88238784	14.52085241	13.42888134
PF4V1	7.938531869	9.785983535	9.014335657	9.637663091	9.328020364
PFDN6	6.625965465	6.857502071	6.197355596	6.244028818	6.808747657
PFKFB2	7.169717707	6.470823475	6.672296636	6.932196717	7.028039657
PFKFB3	7.831503424	6.938883283	7.058764576	6.886441253	6.709075525
PFKFB4	10.99186298	9.558255162	8.975838535	10.4549623	9.921211657
PFKL	8.793339475	9.30949301	7.966054535	8.123844788	8.34368697
PFKM	7.217577212	7.432376434	7.590248525	7.063984253	7.325162263
PFKP	8.820759798	9.156208313	9.583475303	9.238221677	8.971727333
PFN1	11.02243393	11.21816154	9.967178364	10.69793218	10.67572435
PGC	6.694471303	6.364484303	7.107390485	6.568654859	6.948313626
PGCP	9.987874111	9.130293909	9.171801828	9.610538374	9.159196141
PGD	11.19447199	10.11452638	10.21157382	11.06261544	10.23850383
PGF	11.76101506	12.36473556	11.72807534	11.02243393	11.74205097
PGLS	9.352487919	8.961327374	9.354289121	9.652939283	9.662314131
PGLYRP1	9.49510903	7.566450929	8.123844788	9.051957646	8.642670768
PGM2	10.18198959	9.360502949	9.014846778	9.451851384	9.530933293
PGM2L1	7.484854374	8.160644788	8.206690121	7.813759899	8.79722998

PGRMC1	7.286424495	7.499887141	8.728449242	7.761378475	7.521572545
PGRMC2	9.482360616	9.657223606	10.42297323	9.632768747	9.750460616
PHB2	14.13521971	15.65613201	13.04381444	13.36582475	14.1904887
PHC2	9.978126798	8.982611727	9.495686737	9.692793657	9.404333879
PHF1	9.038261515	8.89604703	8.102862354	9.399129424	8.934248131
PHF10	9.957374697	10.02362526	10.44094498	9.698896848	10.32946621
PHF11	9.296131263	9.193817232	10.17224075	9.530205505	9.768390455
PHF6	7.164949697	7.236271343	6.617114677	6.972885929	7.017519192
PHLDB3	8.286128505	8.569735879	8.13267903	8.173633596	8.717867636
PHOSPHO1	8.759332636	9.699589758	7.980934222	8.977558859	9.35128199
PHOX2A	15.74256723	16.33688632	14.13865281	15.48449671	15.46469304
PI3	9.088505869	9.685649929	9.031713778	9.551451798	9.515069697
PI4KA	9.647889364	9.406893465	8.82024202	9.739859192	9.105035414
PI4KB	8.484723788	8.344860929	7.579015667	8.50618604	8.071703475
PICALM	8.615330515	7.907924051	9.247274333	8.474228909	8.341977727
PID1	7.927460424	8.15355797	7.855388333	7.74753198	8.562227051
PIGB	8.13932801	8.260520646	8.858269384	8.527198758	8.568768141
PIGC	8.892401576	9.162016475	7.596851556	9.002272697	8.99408802
PIGQ	8.521696414	8.386601707	7.812003626	8.189547909	8.283330131
PIGX	8.348145404	7.891531071	8.767872848	8.077645899	7.983184263
PIGY	9.402814081	9.741357455	10.5397012	9.662314131	9.816676162
PIK3AP1	7.066488111	6.434478081	7.206984404	6.898002222	6.555968283
PIK3C2B	8.942756889	9.132080535	9.009407333	8.725113212	9.081927515
PIK3CD	9.835894606	8.853585596	8.81966502	9.560346242	8.682101313
PIK3R5	8.539328929	7.576687677	7.572483949	8.167813576	7.356542485
PILRA	11.08264222	10.28406896	9.639623879	10.1885747	10.11555853
PILRB	11.4413808	10.83501466	11.14057221	11.54053762	11.15666056
PIM1	13.42153085	13.92894576	13.62106704	13.89331098	14.3093669
PIM2	9.93099102	10.14432724	11.11388317	10.14809211	9.986114121
PIM3	13.36582475	12.22629088	12.71830686	12.52819783	12.34577038
PIN1L	7.556302505	8.179951646	7.193876586	7.266262606	7.473428394
PINK1	11.60069924	11.73710477	10.9889639	11.74670945	11.89876792
PIP4K2A	11.46936418	12.12692782	11.69440164	11.71443529	12.46797904
PIP5K1B	6.693987121	7.110215707	7.610574101	6.418448242	7.476561202
PIP5K1C	9.440510152	10.38329808	7.650531152	8.691458141	8.992957212
PIPOX	7.59498599	7.555333263	7.295094879	7.016713091	7.426469556
PISD	9.698181576	8.304169929	8.929384727	9.064430111	8.106019798
PITPNM1	8.399075535	8.036816444	6.770611535	8.408296424	8.222223545
PIWIL2	6.414426394	5.869415929	6.228213626	6.577521747	6.624843121
PJA1	7.949790515	8.387083515	8.531655515	8.366948404	8.774537848
PJA2	9.497817424	9.231946182	9.408808788	9.331038253	9.366909111
PKIA	7.644959949	8.030614293	8.176718545	7.751275798	7.858214495
PKM2	8.672881101	9.221294354	8.55725397	9.32739897	9.076382242
PKMYT1	7.899333596	7.924885333	7.640655485	7.862831293	7.963030121
PKN1	8.205133263	7.837840162	6.964624475	8.273878929	7.889761727
PKP3	6.772767788	6.665659576	6.924473131	6.810582646	6.996769242
PL-5283	6.746600626	7.091328697	5.857854657	6.569103444	6.836530859
PLA2G12B	6.024470172	6.123014818	6.775501273	6.296516586	6.55251301
PLA2G15	9.486281202	9.41003803	8.871686232	8.953653768	9.048620525
PLA2G16	10.10946026	10.45996909	10.16937171	11.06433979	10.04421951
PLA2G2A	6.316919404	6.482290162	5.973978232	6.381819808	6.731175838
PLA2G4D	8.210602626	7.670902747	7.91707401	7.718373848	7.865549818
PLA2G5	7.364550071	7.12483998	7.038648475	6.931701566	7.297796364
PLA2G6	8.544633697	8.549141545	8.011239071	8.261781515	7.889031475
PLA2G7	7.825767899	8.346585616	8.386601707	8.381754576	7.763504859
PLAC4	8.319524414	8.923576949	8.363019899	8.210186616	8.806957455
PLAC8	10.07956055	10.33890802	10.51650912	10.77890896	10.57028524
PLAUR	9.940265465	8.591651626	9.964343626	9.455711606	8.979298788

PLCL2	8.784649081	9.300983919	8.982611727	8.593558293	9.254890202
PLCXD2	10.70181173	12.3843369	10.03876748	9.847335909	11.36610866
PLD6	7.089371313	6.831619364	6.884558242	6.435745475	7.00817199
PLDN	7.183404394	7.591410606	7.578291899	7.571427586	7.895278253
PLEK2	10.36239309	10.40074116	10.84844593	9.926784556	10.94942311
PLEKHA1	8.739635162	9.226128374	8.267244596	9.253086273	9.161011869
PLEKHA2	7.028039657	6.885618131	7.74961303	7.41345197	7.501110505
PLEKHA3	8.706567556	8.134790222	8.205133263	8.173308798	8.858269384
PLEKHF2	10.67428006	10.17613521	9.698896848	10.28005063	10.30470526
PLEKHG4	6.712828707	6.941216071	6.585042414	7.053922394	7.218159515
PLEKHM2	8.150096222	7.917722121	7.26025397	8.165380303	8.018337859
PLEKHO2	11.91912022	11.06732533	10.70643744	11.51465712	10.88031852
PLK3	11.51867768	11.56291631	11.2518391	10.79284767	11.04156144
PLOD1	9.288419414	8.654464586	7.186028475	8.499404646	8.552208182
PLP2	9.404880263	8.840010495	8.226500707	8.85308204	8.856895172
PLSCR1	10.13862753	9.57872197	9.644080808	9.295516616	9.448606737
PLSCR3	14.27397716	14.99939481	14.53168901	13.87958112	14.36999747
PLTP	6.740256788	6.240116697	6.475960141	6.55006301	7.002659525
PLUNC	6.193578313	6.072916687	6.943550111	6.008494152	6.050578788
PLXDC1	6.459423596	7.097545323	6.896711899	6.974196465	7.14692997
PLXNA3	6.968641576	6.47989397	6.869142343	6.654772152	7.03146702
PLXNB2	9.406122899	9.598893818	8.534897646	10.1309568	9.59460096
PLXNC1	8.637033606	7.858214495	8.447526838	8.000883182	8.32856404
PMAIP1	7.554402606	7.438614949	8.119288848	7.303836939	7.460903263
PMEPA1	7.57318396	7.898205273	6.857741051	7.846688253	7.966054535
PMM2	7.325162263	7.401750182	6.743041313	7.677935313	7.206984404
PMP22	6.257640303	6.289373162	6.796334212	6.361157788	6.021722061
PNMA1	9.661659101	10.49830212	10.87637444	10.24139873	9.926784556
PNMA3	7.825104404	8.175968162	8.295009141	7.518690889	8.141366242
PNMA5	8.489580414	8.474228909	8.348974465	8.614442374	8.918202434
PNO1	8.067132556	7.520306162	8.530333535	7.952386505	8.47107903
PNOC	7.811684818	7.929593313	7.857845061	7.762120596	8.191573747
PNPLA2	8.654464586	9.099925818	7.645943414	8.45577798	8.792768747
PNPLA5	6.339864232	6.228422404	6.139596152	6.187867838	6.428350232
PNPLA6	7.463067838	7.466006556	6.560645212	7.749299101	7.411268949
PNPLA7	7.432695485	6.871904646	6.963620111	6.895709172	7.351945465
POLG2	8.494982343	8.745043505	9.532335293	8.968910758	8.970512081
POLI	7.935081758	7.574140061	8.031407364	7.764199131	7.59498599
POLL	8.558556152	9.278597747	7.759566061	8.574674323	8.366948404
POLM	10.41731261	11.12315281	9.618955576	10.28089537	10.57996368
POLR1A	6.502532889	6.452131838	6.464688212	6.545504717	6.620737576
POLR1C	7.31258798	7.674109172	8.731576192	7.768055657	7.643628758
POLR2A	7.035678848	7.010749455	7.255805293	7.051121485	7.316095192
POLR2C	7.952029848	6.887565899	7.581982414	7.500501152	7.236577444
POLR2H	9.250770152	9.550085313	9.155079848	9.356058495	9.459003
POLR2J2	8.936938576	9.402230293	9.254890202	9.450568111	9.118629636
POM121L1P	7.814874545	6.96124304	7.366006364	7.194433949	7.238770545
POM121L4P	7.289911515	6.712585717	6.855446939	6.733232384	6.40434199
POMC	9.207791152	8.809442889	7.804724687	8.98371896	8.12949599
POMZP3	8.62412801	8.937429899	8.231756293	8.023506091	8.461315273
POP5	6.852936051	7.125696242	7.83046296	7.173324505	7.117635758
POR	9.814415596	9.018299374	7.960547	9.294355394	8.873520545
PORCN	11.39594259	12.83827393	10.84176532	10.96311364	12.03602449
POTEF	10.2572153	9.946747141	9.526200354	10.09161001	9.637663091
POU2AF1	10.06071544	10.30810853	10.04160563	9.829822242	10.26224218
POU3F3	11.29947244	12.7494647	8.466446051	9.908305848	10.76609029
POU5F1P3	7.09298796	6.638362242	6.425984071	6.982384606	6.850882899
PP14571	9.231349182	9.276189121	9.013830768	9.130293909	9.220765354

PPA1	8.885588879	9.30577901	9.874496818	8.645985424	9.410728505
PPAN	8.387507626	8.477505576	8.053207788	8.480158333	8.741655828
PPAP2C	6.922672051	6.516492273	7.12986801	7.191807606	7.359577545
PPAPDC2	7.320252869	7.667962596	8.12156602	7.608934747	7.655304091
PPAPDC3	6.595700424	7.44049799	5.979536485	6.860179071	6.25331604
PPBP	12.6076243	12.41337695	14.48025691	13.47832004	12.71830686
PPID	8.972363838	8.481851586	8.679110091	8.541125101	8.248137606
PPIH	8.777572838	7.332344778	7.895278253	7.825104404	8.787164939
PPIL3	9.674270303	10.56306707	9.899010949	9.95201103	9.976273051
PPIL4	7.289331586	7.738963899	7.790604293	7.37546403	7.659186848
PPM1F	7.552094283	7.30556202	7.191807606	7.839647131	7.180559343
PPM1G	8.439612798	8.178694051	8.225777414	8.967408879	8.631899566
PPM1M	8.819236758	8.305359949	7.543396525	8.589797808	8.066736566
PPM2C	7.250899535	7.494798263	7.520306162	7.478449101	7.252038162
PPP1CA	10.92880859	10.18942995	9.70034896	11.16888788	10.64502212
PPP1CB	8.916479111	8.780633818	8.083806798	8.721054424	8.935320828
PPP1R11	8.402148465	9.305189859	8.069395848	7.949400121	8.523456192
PPP1R12B	8.806450121	8.334981101	8.175288444	8.32939098	8.419470808
PPP1R12C	10.69793218	10.48074239	9.066117364	10.48539779	10.32208546
PPP1R14A	6.466022525	7.171072313	7.404355202	7.03146702	6.875487455
PPP1R15A	9.193817232	8.676791091	8.304169929	8.826393101	8.467263475
PPP1R3B	9.975431768	9.161459798	8.931557869	9.191263899	9.239472869
PPP1R3D	10.59180142	9.923012889	9.49850403	10.22761479	9.640429374
PPP2CA	8.963499596	8.584830636	10.11349335	9.035040848	9.023071899
PPP2CB	9.235179424	9.267383556	9.90675497	8.991410131	8.827377505
PPP2R1A	8.894948323	7.906781182	9.376667182	8.931557869	9.168828475
PPP2R2B	7.770089636	7.79851096	8.758364879	8.378142071	8.532980343
PPP2R3B	8.151663141	7.624016909	6.990260788	8.411285283	7.893273434
PPP2R3C	11.74935785	11.50261564	12.35168631	11.81018832	11.59870577
PPP2R5A	9.711723394	9.335272182	9.256767414	9.826793596	9.443424081
PPP2R5B	9.667708556	10.22557955	9.07419996	9.864614818	9.827596687
PPP3CC	9.186711646	9.889177586	10.36032821	9.728754818	10.05607537
PPP4C	8.954798808	8.474658303	9.129654758	8.662363172	8.694292242
PPP4R1	8.236986626	7.556918475	8.461795505	7.876807081	7.471589222
PPP4R2	7.36510997	7.18283101	7.709201212	7.274888343	7.506434626
PPT1	10.86691694	10.1752482	11.28731295	10.71390019	10.3548828
PPTC7	10.31693391	10.19136651	10.15086094	10.28406896	10.23452156
PPY	6.655235768	6.397600616	6.462162828	6.306878242	6.03119702
PQLC1	9.643287657	10.41330878	9.384078242	10.22668357	10.30810853
PRAF2	9.041236566	9.283145788	8.026271202	8.626833051	8.892401576
PRAGMIN	9.70798304	10.48432644	10.14534091	9.917043434	10.05426836
PRAM1	12.82558839	12.44264271	11.48459908	12.52130815	11.97447062
PRAMEF1	6.593877636	6.480794707	6.878162091	7.129597273	7.449946354
PRAP1	6.496794495	6.246552232	6.584604515	6.852390212	7.254341798
PRB3	9.204345657	11.21493335	7.400588576	9.561667293	9.808672222
PRB4	7.854325061	7.833986465	7.519990596	7.395598354	7.356281616
PRDM1	7.103541778	7.353244222	7.303836939	7.388945192	7.537240869
PRDM7	6.573540778	5.807341535	5.975084869	7.111681374	5.945967919
PRDX1	9.704210374	10.78987961	10.8778129	10.71860391	10.96311364
PRDX2	8.769354545	9.119831202	8.602379626	8.683087172	9.922126404
PRDX5	11.78498903	11.83813795	12.3613163	12.62449502	12.21630458
PRDX6	14.79546777	14.46162431	14.65397593	14.89443037	15.97330865
PREB	9.063885091	8.949784505	8.737323475	9.509226798	8.893968333
PRELID1	11.37847359	11.21668249	10.24227579	11.60069924	10.71503879
PREX1	14.29173122	13.06488572	14.09521817	13.48990673	13.26910509
PRF1	8.864955909	8.251922909	7.516138929	9.187244566	8.514037303
PRIC285	9.083185818	9.30770201	8.292509485	9.200042253	8.981975535
PRIMA1	8.138519566	8.245649515	7.619556354	7.892277636	7.726692222

PRKAR1A	9.876880455	9.196132455	8.843670869	9.741357455	9.31393701
PRKAR2B	9.122138505	9.565032515	9.834210131	10.01974654	9.350012182
PRKCD	11.15503783	10.26332497	10.34193651	10.90324033	10.49140257
PRKCDBP	7.050890818	7.357775939	7.751275798	7.410622798	7.875808798
PRKD2	11.05356995	10.32208546	9.361074667	10.91903182	10.65871503
PRKRA	8.197805768	8.415109929	8.373919657	8.211411717	8.438325768
PRKX	7.56165098	7.764931778	8.682101313	8.232591606	8.201303758
PRKY	7.879657162	7.843485626	7.077096727	8.169730152	6.929106091
PRM1	6.321469101	5.964018485	6.708797384	6.130576222	6.769912384
PRM3	8.155393222	8.472925747	9.046362323	7.878949444	7.660150727
PRO0478	9.392652323	9.363823202	9.963504081	9.538964939	9.627745303
PRO2852	8.088117667	7.629016404	6.095228879	7.448630646	7.334639071
PROK2	13.66158872	12.58957491	12.09497108	12.23505088	12.59668127
PROKR1	10.23660558	9.532335293	10.33989796	10.51886057	9.196689051
PROP1	12.3843369	13.15082863	11.16734497	12.34237091	13.03025597
PRPF39	7.700670768	8.251125323	6.905589111	7.986161141	7.959488687
PRPF6	10.65751844	10.5397012	9.772250768	10.85782556	10.45041513
PRPF8	10.04160563	10.02161965	9.02886299	10.29432836	9.891739697
PRPS1	8.135850465	8.382194192	9.337862091	8.753927596	8.950874253
PRR17	9.190767364	10.67428006	7.998587576	9.30949301	9.537497323
PRR5	14.53168901	14.37933382	14.01682491	14.34357305	14.33555643
PRR7	10.1985269	10.66229262	9.746868596	10.44392641	10.65634987
PRRG3	6.600294051	6.03119702	6.757434848	6.512799333	7.115448364
PRRG4	7.845981061	7.769805091	8.108111747	8.505287909	7.89464897
PRRT1	7.820605525	7.964547263	7.979863071	8.27903296	8.583542788
PRSS2	7.636262586	7.130479657	7.839647131	7.535195111	7.523479626
PRSS23	6.510975394	6.469428232	6.728691949	7.215458101	6.850608899
PRSS3	6.762568667	6.278820495	6.853717071	6.706660646	7.241229071
PRSS33	7.477539293	6.697631071	7.525850576	7.437975364	7.753435232
PRSS36	9.900778232	8.036387556	11.14385742	9.369545939	11.92396137
PRSS8	6.978374222	6.323307657	6.889002121	6.912025515	6.810307121
PRSSL1	6.843774949	7.190368869	7.103815788	7.342557798	6.876638434
PRY2	6.590932293	6.331869444	6.474717424	6.231060354	6.348442212
PS1TP4	7.024510747	7.110844919	8.117339677	7.022261949	7.047335818
PSAP	11.1502402	10.6313464	10.72633412	11.62290433	10.82416773
PSD4	10.37026232	9.501306556	8.613130333	10.34830463	9.80784303
PSEN1	8.598479657	8.159434576	8.85518403	8.377090384	8.002342131
PSENEN	8.416773909	8.315227333	8.334162556	8.118879606	8.605125889
PSG2	7.904381697	7.473129747	7.588284717	6.743573939	7.257820879
PSKH2	6.544566455	5.951479263	7.360451687	6.991264788	7.497307495
PSMA5	9.66613299	9.949411172	11.42371373	10.44094498	10.3896003
PSMB3	10.30358984	9.897493586	10.61133106	10.0053333	10.36239309
PSMB5	9.126080354	9.293710889	10.10631712	9.377908	9.217165182
PSMB6	10.40074116	10.70534521	11.61117883	11.02700786	11.1031408
PSMB9	12.77841715	11.9091429	12.65104366	12.48458958	12.22341713
PSMC4	8.797743222	8.715845505	7.845242687	9.119831202	8.519692939
PSMC5	7.989587677	7.641344242	8.949784505	8.65497299	8.214296636
PSMD2	8.59026096	8.435650677	8.235056909	9.058895657	8.720163182
PSMD6	9.57872197	9.302735434	10.71145754	9.749660394	9.685649929
PSME2	13.10484893	13.28559105	13.85099976	13.57574016	13.76108056
PSMF1	9.715215101	10.10093339	9.567720788	9.915234909	9.834210131
PSMG1	8.43615796	9.004940778	8.690918616	8.257253131	8.623178879
PSMG2	7.609574333	7.814874545	9.142791657	8.020887778	8.13932801
PSORS1C2	8.518144394	9.232683343	7.003455566	7.652952818	8.303356141
PSPH	9.26122399	7.87816304	7.591899823	7.625433667	7.470329323
PSPN	7.037511364	7.996041859	7.165241424	7.218710525	7.496050374
PSTPIP1	12.75396286	11.86922315	11.565007	12.12151457	11.911688
PTAFR	11.80554878	11.38970236	10.7442872	11.74205097	11.1453264

PTBP2	7.956615091	7.816627253	7.612909768	7.833291081	7.963371182
PTCD3	8.591651626	8.070136677	8.460488909	8.57971698	9.049171303
PTCH2	6.565610293	6.215520505	6.714476606	6.833840465	6.91094498
PTCRA	11.55322016	11.7349036	11.43005301	10.95533553	11.80342824
PTDSS2	9.150811232	9.58708204	8.678158051	9.463745455	9.428845576
PTEN	10.88166557	10.20634509	9.916104475	10.12610261	10.53141254
PTGDR	7.922697606	7.686071172	8.489178071	8.279889293	8.070136677
PTGDS	10.08700287	10.93935201	9.729555121	11.96362869	9.907546465
PTGER2	7.714317071	8.137860071	8.912003131	8.003903253	7.961295828
PTGIR	8.373919657	8.932594273	8.25570701	8.713915657	8.549517636
PTGS1	8.806957455	9.550769788	9.620873889	9.509876273	8.377090384
PTGS2	8.465492313	7.595301182	7.664275131	7.976470212	7.874403566
PTH2	8.471488636	10.09079019	8.319901444	7.973227556	7.961659859
PTK2B	8.85049999	7.941320778	7.609574333	8.799825313	7.943134
PTMA	11.89361272	12.08128174	12.0750029	12.99088515	12.72219022
PTMAP7	10.15585749	10.67939021	9.486281202	10.25038968	11.0387546
PTMS	13.3718573	14.78379092	11.68243664	12.3543152	13.68884286
PTOV1	8.415109929	8.372086737	7.396513838	8.641223283	8.216297535
PTPLAD2	9.589694828	9.093526566	9.471411141	9.114524374	8.907915899
PTPN12	11.05493438	10.47584162	10.86691694	10.50057506	10.58689262
PTPN6	10.79564384	10.20964764	8.880853535	10.73384189	10.18198959
PTPRC	12.5521346	12.02240324	13.03496614	12.63888703	12.28105924
PTPRCAP	9.276189121	10.00361533	9.443424081	9.802496737	9.97079498
PTPRE	10.88546605	10.31693391	10.38430498	10.44712342	10.06904603
PTPRK	7.152420131	7.062438556	7.159147152	6.31049204	6.831129859
PURA	9.145078374	9.35128199	8.644542606	9.614560172	9.417037424
PUS3	7.945111576	8.669992818	8.277763646	7.81061403	8.367840636
PUS7	9.302103889	9.177504333	9.361671283	9.029402949	9.281446566
PVALB	7.647964475	8.058246657	8.586731747	6.483554929	7.378842657
PVRIG	9.74383503	10.09339803	10.32418729	10.06904603	9.992988556
PVRL2	7.032313212	6.223876404	6.566291384	6.024205414	6.211831051
PVT1	8.371674091	8.486543404	7.87816304	7.81270001	8.339115859
PWWP2B	8.599954677	8.675348646	8.019097616	8.829061111	8.669992818
PXDN	6.244028818	5.989522455	6.373171747	6.545751121	6.935043828
PXK	9.857940545	9.157435455	8.80133401	9.221294354	9.450568111
PYDC1	9.013314737	8.802385697	8.501579646	8.727989172	8.610337293
PYGL	12.70390423	11.08264222	11.70138789	11.74439188	11.78498903
PYY2	7.800216455	9.693515768	7.40806004	8.037664566	8.575095707
QARS	7.015344242	6.766746646	6.395988485	7.214315212	7.031727121
QDPR	6.686567667	7.359873626	6.638128919	6.737600111	6.893883192
QPCT	11.84529477	10.54692936	11.2345748	11.13400706	11.04316622
QRICH1	9.060017677	9.318836889	9.368224626	9.31327601	9.353057535
QTRTD1	8.85308204	9.145078374	8.634788929	8.673843717	8.808345061
R3HCC1	8.138156535	8.325316889	7.468494556	8.276438626	7.975414556
R3HDM2	9.185497838	9.479751879	9.225515485	9.022000869	9.044608384
R3HDML	7.112259949	7.524403394	7.394983566	7.886480404	8.337550606
RAB11FIP5	6.343092465	6.675226152	6.036027182	6.827413455	7.45998899
RAB15	7.975072535	7.977207111	8.469339576	7.666324061	8.325316889
RAB1A	9.035586242	8.514431737	9.000069889	8.723452192	8.709464434
RAB1B	8.745474636	8.876209859	8.64507902	8.663303283	8.769824364
RAB24	12.43600068	11.23280633	12.10414281	12.31089415	11.88375452
RAB26	6.629727051	6.492854313	6.996297444	6.960463354	6.664678717
RAB27A	9.487680697	8.759757687	9.516398909	9.363823202	9.093526566
RAB2B	7.254341798	7.738613697	8.139691131	7.094132182	8.454049747
RAB31	11.9091429	11.57567047	11.98440811	11.86652814	11.2786408
RAB33A	8.355847778	8.526649808	8.777572838	8.064260859	8.225261616
RAB35	10.38842688	10.00262223	10.05236285	9.811091061	9.793942798
RAB36	8.669992818	7.51310204	7.392218323	8.971160737	7.291982576

RAB3D	9.862946677	8.979921919	8.523871909	9.223302313	9.169862626
RAB5A	8.824381182	8.546982556	8.721926778	8.534897646	8.497259596
RAB7A	8.838020616	7.983184263	8.630877737	8.776574232	8.230996848
RAB8A	7.553773697	7.460903263	7.843855828	7.853350485	7.598252121
RABEP2	8.209418434	8.418553323	7.623386535	8.45451298	8.438712657
RAD23A	8.868452172	9.303342798	8.002011657	8.711304737	8.943861384
RAD51L1	6.801828172	5.980387263	6.448856899	6.935824606	6.536426172
RALB	8.265130232	7.500501152	8.691458141	7.994919717	8.007258162
RALY	9.156840283	8.891390919	7.630934212	9.024707707	8.793339475
RAMP1	7.627997354	8.185177828	8.840010495	8.066736566	7.447389475
RANBP1	9.395177889	9.36439596	10.14809211	9.785983535	9.451212687
RANBP10	9.475206131	10.47702836	10.02549055	9.719830172	10.33989796
RAP1A	11.82112126	11.22456459	12.25572441	11.80554878	11.76336308
RAP1GDS1	7.097212141	7.235967596	7.763186	7.378186273	7.594027929
RAP2A	7.435437465	7.764199131	6.794399263	7.489478697	7.817596162
RAPGEF6	7.724369343	7.575132364	8.462637525	7.934739636	8.308776677
RAPSN	7.29228501	7.016433495	6.715208525	7.147201253	7.170797768
RARA	11.49636188	12.24649354	11.2608846	10.96000427	11.52448673
RARG	8.214678465	8.473802646	7.533663232	7.688677182	8.197004869
RARRES3	10.51650912	11.10761793	11.38025327	11.49839615	11.47496104
RASA4	9.001802606	8.712341879	8.190781646	8.648914657	8.81966502
RASAL1	7.773582384	6.572131525	8.778623323	8.099466788	8.381309202
RASGRP2	13.89331098	13.2158469	13.67554111	13.68884286	13.35919327
RASSF1	10.19528987	9.849671929	9.984347768	10.62647628	10.22668357
RASSF2	14.34357305	13.40302459	13.78737352	13.83173935	13.60269863
RASSF4	8.754441596	8.835493283	9.066605263	9.478383576	8.942313313
RAVER1	10.37124354	11.30690717	9.728754818	9.79615097	10.63752253
RB1CC1	9.880317061	9.455711606	9.558255162	9.746145949	9.597513697
RBBP7	10.10841911	10.40925212	11.31852116	10.75453103	10.687913
RBCK1	10.12697515	9.284395657	8.53628698	10.01524205	9.820433545
RBM12B	7.764609818	7.679634566	6.54019204	7.452458778	7.250545919
RBM26	7.62575301	7.447389475	8.430534091	7.869033162	7.986535808
RBM3	9.766921414	10.60044575	11.45932673	10.15283227	10.75987116
RBM34	7.979092162	8.228946717	8.851018515	8.514431737	8.609511
RBM35B	7.188870657	7.029897788	6.848831242	7.30728296	7.694916828
RBM38	10.54950911	11.47127063	10.58689262	10.35591393	11.09089144
RBM42	9.687758929	9.423026242	9.205404404	9.681893121	9.634173323
RBM6	9.731806091	9.29794999	10.42736719	10.03701736	10.02362526
RBMS2	8.612168455	8.511758747	8.047933768	7.492937667	8.175288444
RBMX2	8.379056919	8.759332636	9.615324495	8.593101434	8.655437626
RBP7	10.74045328	9.983540212	9.690534253	10.4524716	10.19757035
RCAN2	5.80571198	5.902464323	6.402020111	5.785248091	6.237000141
RCAN3	6.992927808	7.810269677	7.403028737	7.453623293	7.797844434
RCC1	10.27804811	9.952967535	9.473222667	9.077686808	9.749660394
RCN3	8.314030071	7.644277273	6.527432758	8.25570701	7.343750657
RCSD1	11.01209098	10.89094316	11.56988377	11.14866374	10.85225864
REC8	9.114524374	8.885078273	8.655437626	8.583542788	8.814101707
REEP1	9.055478465	8.395296687	9.576740677	8.745901071	9.46304199
REEP4	7.531209667	7.412474121	7.160551111	8.138519566	7.320252869
REEP6	8.322342051	8.449337667	8.521696414	7.497675424	8.026630869
REL	7.91465098	7.77977198	7.977207111	7.949790515	7.852302727
RELA	7.869396606	7.507037566	7.421318101	8.387083515	7.831138545
RELB	12.15752365	12.39348455	12.77454263	12.5521346	12.77454263
RELL1	12.38794592	12.20960187	11.41981305	11.21958266	11.25523353
REPS2	10.21375666	9.001272616	9.226702434	9.548618051	9.332795131
RERE	8.625486818	8.256833364	7.784529071	7.545042545	7.668960768
RERG	6.69201996	6.977041091	5.961236343	6.320458162	6.728691949
RETN	7.697220202	7.434501333	7.435437465	8.01423197	7.343153889

REXO1L1	7.267131253	5.784798343	5.766201232	7.091592101	5.89725504
REXO2	8.977005354	9.928455091	9.987077505	8.971727333	10.10946026
RFTN1	9.16271003	9.625041525	9.786685869	9.781348374	9.766131535
RFX2	9.591816061	9.417037424	9.497084828	8.946107263	8.822853162
RGL2	12.09225922	10.62647628	10.36589334	11.92396137	10.77387872
RGL3	6.998508111	6.688467242	6.202443939	6.07129398	6.207117515
RGL4	13.28559105	11.4413808	12.66167346	12.07810634	11.7894918
RGPD1	7.168394253	6.715208525	5.886387566	6.364484303	6.480794707
RGPD2	8.368326869	7.900715717	7.550845909	7.836828747	7.995673758
RGR	6.493490929	6.124346828	6.867621424	6.030522263	6.261209
RGS10	14.5513462	14.72870348	15.70163214	14.88238784	15.34617167
RGS14	9.867911152	9.252443646	7.932453717	9.455015404	9.291911616
RGS18	13.86604428	12.89513437	13.74759592	14.06476988	13.14611959
RGS19	9.491725303	8.741655828	7.992269263	8.960773354	8.526649808
RGS2	16.7804214	15.06622261	16.7804214	16.61084374	15.74256723
RGS9	6.623160525	6.698743475	6.314827061	7.06161799	7.19832496
RHBDD2	8.311754818	7.969793182	7.229587778	8.555429434	8.01013803
RHBDF2	11.72126504	11.61814895	12.23191382	11.70812672	11.72592113
RHBDL1	13.62784329	15.15443825	11.70812672	12.85048632	13.33661988
RHCE	6.360006374	6.871437091	6.863389636	6.413017273	6.992656859
RHD	6.259875061	6.300917343	5.992760111	6.282981828	6.050170374
RHOBTB1	7.294583061	7.870669919	7.731871869	7.923778798	7.435437465
RHOG	11.68243664	11.0035084	10.80091358	11.72592113	11.22294817
RHOH	8.411696677	9.022000869	9.501306556	8.739178636	8.905887879
RHOT2	10.05523038	9.736957071	8.993505475	10.3810842	10.19136651
RHOV	8.981975535	9.024707707	8.669446475	8.553132192	8.516311424
RILP	11.66441291	12.59668127	11.65455063	11.68038318	12.32964397
RILPL2	13.44849295	12.52819783	12.99565877	12.89886333	12.66985986
RIN3	8.584830636	8.135177152	7.659822475	8.352944343	8.107705111
RINT1	8.180413081	8.754900343	7.981709394	8.35752198	8.39308204
RIOK3	10.23850383	10.92485902	11.19966733	10.94786936	11.80554878
RIPK3	8.121954505	7.589252838	7.164102909	8.120795899	7.702311758
RMI1	9.521192909	8.698751323	8.162707626	8.998971485	8.817191758
RNASE2	7.328411747	7.36298202	7.873377879	7.36076402	7.480348535
RNASE3	6.540677515	6.658129061	6.61996199	6.628047111	6.448115818
RNASE6	7.723687384	7.816902374	9.452497313	8.544134919	8.552719747
RNASEN	9.627745303	9.898158111	9.901640636	9.698181576	9.961780586
RNF10	12.68484185	13.74759592	13.49745762	13.27940818	13.47327752
RNF11	11.21493335	11.89876792	12.13538265	11.73271713	12.54268587
RNF114	9.609756293	9.179819687	10.26332497	9.524156586	9.40167604
RNF122	9.548028869	8.870056939	9.390110384	9.147296667	8.539328929
RNF123	8.437457535	9.272374697	7.989204111	8.699656879	9.112697707
RNF125	6.209048788	6.193752182	8.156410909	6.69036997	7.425203606
RNF13	9.925979414	9.486974667	10.78269205	9.994772242	10.01622438
RNF130	13.34730616	12.57107442	13.21058954	12.61449239	12.57107442
RNF14	7.560760293	7.871683444	8.368326869	7.55847901	8.080354404
RNF141	10.34830463	10.32635032	9.836777707	9.958247111	9.703591263
RNF144B	9.945994374	9.328020364	10.98492733	9.145078374	9.62024901
RNF146	9.376046232	8.739635162	9.823678374	8.318250424	8.439612798
RNF149	15.29549959	14.21449649	14.89443037	14.6970922	14.46162431
RNF151	6.60386102	6.155865424	6.706206909	7.303562909	6.442532636
RNF166	8.326884343	7.358963616	7.799196616	8.361327455	7.629989283
RNF182	6.213218293	5.877338586	7.386763141	5.793127394	8.037287293
RNF187	9.175472828	10.39072525	8.213832515	9.297353606	9.742174424
RNF19B	7.459405444	7.580594545	6.166680606	7.123768636	6.944866586
RNF20	7.654890798	7.612909768	8.699656879	7.90290904	7.860764394
RNF207	7.45998899	7.481001515	7.235098212	7.083894141	7.525850576
RNF208	7.091592101	7.31258798	7.303307	7.291354758	7.734896879

RNF214	6.908074727	6.924186162	6.242810566	6.83799099	6.984018879
RNF24	9.781348374	9.056062667	8.747331152	9.69725304	9.164545343
RNF40	8.147857556	8.059423535	6.65032298	7.993077394	7.838908253
RNFT1	7.369172101	7.186960111	8.066736566	6.994489162	6.993463051
RNPC3	9.772980061	10.32309273	8.93587504	9.409395929	9.622300838
RNPEP	7.433629313	6.862375091	7.313451111	7.014842626	7.217862778
RNPEPL1	10.83790729	11.01045685	9.50710198	11.03008027	10.66832974
ROBO3	8.626833051	8.966282283	8.339926909	8.435296202	8.685647889
ROCK1	8.919862162	8.467263475	8.37004801	8.866888202	8.69335002
ROPN1L	9.064430111	7.995673758	8.232591606	7.922041747	8.36093298
RP11-265F14.2	8.025425616	7.679923515	6.783369808	7.292849111	7.881102323
RP11-309M23.2	8.449337667	9.058895657	9.331636121	9.104371838	9.037153444
RP11-529I10.4	8.065602475	8.650384111	9.087255717	8.339926909	8.702074263
RP11-544M22.4	10.6313464	10.38738452	10.68960982	10.26632574	10.32418729
RP11-631M21.2	8.293769586	8.29291099	6.783830859	8.406205495	8.484240687
RP11-94I2.2	11.46539851	11.20660223	11.00053539	11.30301738	11.18636156
RP3-527F8.2	7.824090626	8.397853727	7.948737212	8.048299333	8.207926798
RP4-621O15.2	9.609027141	8.157153081	8.918202434	7.993428586	8.647922808
RP4-691N24.1	6.267641192	5.970980293	6.121756828	6.79736498	6.684320869
RP5-1077B9.4	8.838495424	8.746311293	7.263217283	8.694292242	8.683580283
RP5-886K2.1	7.007889061	6.877410515	6.417030697	6.58977503	6.498851707
RP6-213H19.1	10.63870636	10.51771241	10.19757035	10.23850383	10.13479405
RPA2	9.602112616	9.246644323	10.61480751	10.08521343	10.4549623
RPA4	6.607592929	7.151031394	6.396985434	6.320887909	6.462589889
RPGR	8.650384111	8.547862646	8.740682	8.316081222	8.092691677
RPGRIP1	9.003925646	8.64752602	9.569063343	8.750916455	8.588536172
RPH3AL	7.373717778	6.355561455	6.302130162	7.073141899	6.523626889
RPIA	11.74670945	12.44950322	13.37822002	12.47761486	14.11997744
RPL10A	13.79381649	14.01682491	14.7528858	14.5108222	14.36070414
RPL10L	11.88148631	12.70002263	12.44264271	12.64265677	12.29886409
RPL11	14.74028573	14.89443037	15.97330865	15.74256723	15.61926687
RPL13A	13.80033164	14.05136004	14.38889659	14.26676228	13.99495772
RPL14	13.44126502	13.86604428	14.94534759	14.13521971	14.34357305
RPL15	10.46442497	10.83790729	11.58421758	11.2345748	11.45526933
RPL17	11.80104424	12.04990566	12.85048632	12.70002263	12.85048632
RPL18	12.37087917	12.71520416	13.24400831	13.05987966	13.06967433
RPL18A	12.83405786	13.54965001	12.79024082	13.40990013	13.46005067
RPL19	15.61926687	15.63778652	16.36806566	16.06827159	16.09492117
RPL22L1	10.27905981	10.76983035	11.64044206	10.66465811	11.13879804
RPL23A	12.24351077	12.55983323	13.40990013	12.86086109	12.76207554
RPL23AP7	11.88909817	12.2293359	13.16824615	11.55524693	11.48459908
RPL24	12.39660011	13.00101781	12.25844332	12.9709957	13.27418186
RPL26	14.70594079	14.82007331	16.14978837	15.42966281	15.50564301
RPL27	14.05855866	14.3093669	14.36999747	14.45281427	14.7172184
RPL29	11.36065784	11.96059797	11.72126504	12.04160066	12.08680505
RPL29P2	8.837022465	9.575401111	9.227886646	8.997807768	9.086722374
RPL3	13.37822002	13.62106704	14.39736901	14.04335209	13.98831971
RPL34	13.00101781	13.47832004	13.3718573	13.3718573	13.67554111
RPL36	11.96947069	12.7452022	12.4571909	12.65819872	12.81379045
RPL36A	14.30038792	14.68633385	14.4427031	14.90726461	15.05177986
RPL37A	13.65450155	14.03424604	14.50017253	14.33555643	14.37933382
RPL39	13.39037726	13.89331098	12.93862501	13.87263802	14.16629104
RPL3L	10.9088749	10.14534091	10.22761479	9.314493212	9.912769303
RPL4	13.14611959	13.39037726	14.76275492	13.90036211	13.80704919
RPL41	15.16803204	15.50564301	16.09492117	16.17943014	15.83165575
RPL5	11.25349741	11.52448673	12.35798465	11.96947069	12.08943903
RPL6	12.90783816	13.29117716	14.03424604	13.6823979	13.78027417
RPL7A	12.27101	12.27101	13.87958112	12.81379045	12.50328361

RPL7AP11	10.35917202	10.49466708	11.92396137	11.22947614	10.89094316
RPL9	13.02518615	13.41514643	14.47046648	13.85099976	14.05855866
RPLP0	12.3774088	12.73352188	13.10985871	13.34204398	12.92542832
RPLP0P2	12.58612865	12.93430142	12.7452022	12.89513437	13.17281418
RPLP2	16.4534382	16.57174008	17.23317693	17.36803328	17.29704085
RPS12	15.32551587	15.72204116	16.68963031	16.42659037	16.42659037
RPS14	12.83827393	12.94367069	13.6823979	13.33155169	13.40302459
RPS15	13.7211242	14.27397716	14.74028573	14.50017253	14.35197184
RPS16	13.46005067	13.80033164	14.5108222	14.15863941	14.223913
RPS17	14.85646221	15.12351424	15.58150095	15.54332231	15.42966281
RPS18	14.82007331	15.01206542	15.81050193	15.36066179	15.36066179
RPS19	12.54268587	12.96662998	13.82538175	13.26910509	13.23864174
RPS20	15.05177986	15.41210544	16.04217659	15.92931651	16.06827159
RPS23	13.5360606	13.6823979	15.12351424	14.49002723	14.56207529
RPS24	13.76702458	14.04335209	14.52085241	14.48025691	14.48025691
RPS26	10.27432009	9.178084667	10.3025572	10.46664928	10.79145221
RPS27	16.25437967	16.2036826	17.07010739	16.96059838	16.96059838
RPS27A	14.4427031	14.70594079	14.9727616	15.12351424	14.9591327
RPS28	13.43523914	13.91489381	13.60979651	13.92172837	13.91489381
RPS29	14.49002723	14.90726461	15.61926687	15.18392271	15.22815255
RPS3	12.9709957	13.44849295	14.20688311	13.9071606	13.79381649
RPS3A	12.19398134	12.54268587	13.08418191	13.08418191	13.07454735
RPS4X	13.47327752	13.75520609	15.18392271	14.25042984	14.91973608
RPS4Y1	11.38391359	11.80554878	5.914411111	11.99535117	5.814753253
RPS4Y2	11.68700199	12.06692054	5.765885525	12.3613163	5.768127232
RPS5	10.66229262	11.35278885	12.24097391	11.28731295	11.44319437
RPS6	14.99939481	15.36066179	16.17943014	15.46469304	15.78787594
RPS6KA1	11.7349036	10.93334198	10.59180142	11.51867768	10.74558579
RPS6KA2	8.615838	8.661956242	8.607626293	8.142577798	8.77085696
RPS6KA4	7.74578199	8.412578899	7.152088687	8.252372808	8.180785838
RPS6P1	7.976470212	8.39446903	9.202552434	8.157153081	8.747331152
RPS7	12.2293359	12.37087917	13.78027417	13.0147344	13.03957854
RPS8	16.22759729	15.95256028	17.36803328	16.90924948	17.01900663
RPSA	10.94942311	11.40495249	11.89130603	11.81439214	11.82852227
RPUSD1	9.202552434	9.31393701	8.888766747	9.333986848	9.303966081
RRAGC	11.12151875	10.59062542	11.21493335	10.87235819	10.95374716
RRAGD	7.732550404	7.30728296	8.210994667	7.484516838	7.592388485
RRAS	9.66383	10.06633871	9.286110869	9.724814657	9.949411172
RRBP1	8.559456798	8.219387636	7.726392606	9.179226364	8.476994374
RRM1	8.873520545	9.273022949	8.746767313	8.842109	8.951513343
RRM2B	8.477505576	8.217667525	7.618887111	8.008022949	7.863195747
RRP12	8.986932636	8.736221495	8.090157434	9.445197818	9.079484556
RRP7A	8.056301667	7.334066545	7.190644495	7.696527091	7.762451808
RRP7B	9.863774293	9.985290333	9.149195859	9.519820636	9.70798304
RRS1	8.097222414	8.321919424	8.138519566	8.185563303	8.063008152
RSAD2	7.107390485	7.062669222	7.42989803	6.710284202	7.36298202
RSBN1L	6.489773778	6.116576919	6.119569697	6.847264	6.96619404
RSC1A1	7.101049919	7.927460424	7.546592828	7.291703707	7.743818828
RSPRY1	7.07551896	6.931296273	5.926867677	6.769655182	6.85466502
RSRC1	7.532439838	8.153159131	8.204380404	7.851640596	8.046104414
RTBDN	8.439112919	9.443424081	8.082778869	8.184399818	8.766842081
RTCD1	9.174793152	9.339095222	8.674900657	8.878776172	9.11093099
RTDR1	7.263217283	7.452458778	7.267388222	6.971796172	7.057011424
RTN3	11.05796986	10.79564384	9.535633242	10.43872423	10.52904685
RTN4RL2	7.331138333	7.256041222	6.941709929	7.31991999	7.511838192
RTP4	8.357077121	8.656360697	9.032817303	8.686679798	8.874692596
RUFY1	12.44611991	12.21312889	12.70390423	11.47322198	12.15130062
RUNDC2A	8.320817869	8.443547162	8.321152475	8.145617202	8.407442505

RUNDC3A	8.348974465	8.816703535	7.865908465	9.889177586	9.119222505
RUNX1	9.051957646	9.721958051	8.578858313	8.754900343	9.146732444
RUNX3	9.413321525	9.686378667	9.543825273	10.2026692	9.692043475
RUSC1	7.704713596	7.619556354	7.454572909	7.428304182	7.860050667
RUVBL2	7.785214909	8.024633293	7.141717727	8.221403505	7.939609869
RWDD1	10.08521343	10.51400776	10.73788216	10.46442497	10.90466263
RXRB	9.094076404	8.436545051	8.892970697	9.711723394	10.07764109
RYBP	10.05426836	9.964343626	9.974493182	10.42736719	10.23066525
S100A10	11.54675633	12.19996238	11.60888892	11.42371373	11.96947069
S100A11	11.9577766	10.90606985	10.1250404	11.34892675	10.99345288
S100A12	13.73439002	12.88265743	12.28369472	13.18348162	13.27940818
S100A4	14.46162431	14.17411554	14.02572128	14.80749806	14.53168901
S100A6	16.09492117	15.02501841	16.06827159	15.88528111	15.88528111
S100A8	17.58093558	16.25437967	16.64557528	17.23317693	16.68963031
S100A9	14.52085241	13.29684189	13.45466248	14.83117585	14.47046648
S100B	6.798572747	6.24926196	5.758791525	5.754325131	5.756102667
S100P	15.54332231	13.98831971	13.86604428	13.95167504	13.86604428
S100Z	7.434501333	7.717096556	7.498343283	7.991026727	7.460268556
S1PR1	7.690510313	8.303793737	8.279889293	8.073294879	8.217984283
S1PR4	15.34617167	14.64328928	15.39581235	15.19830883	14.68633385
S1PR5	7.445432899	7.124596343	7.722297051	7.817975838	7.182015707
SAAL1	6.840661414	7.29078701	8.125376242	7.168394253	6.957895121
SAMD10	6.535299384	6.446595263	7.597232061	6.593389535	7.497675424
SAMD3	7.720869455	7.873714818	8.703733141	8.058246657	7.856391758
SAMD4B	6.660563556	6.466725596	6.812677121	7.025264747	7.307847101
SAMD9L	10.45375986	9.432636424	10.88681216	9.932690333	9.415217505
SAMSN1	9.053043889	8.327767232	9.736139061	8.59942799	8.442699879
SAP30	9.347406172	8.879330566	9.610538374	8.573727141	9.175472828
SAPS1	8.497259596	9.683334444	7.536878778	8.366003535	8.762202232
SAR1A	9.946747141	9.734703111	10.16355358	9.966143818	10.25320711
SART1	9.702015293	9.320176919	8.63568099	9.916104475	9.519820636
SAT1	13.27940818	12.26775966	14.05855866	12.95787308	12.71142619
SAT2	11.82616001	11.66441291	12.33907422	11.88649013	11.76101506
SATB1	10.53141254	10.87235819	11.04593523	10.59418919	10.78014639
SBDS	7.370079717	7.661901192	8.361327455	7.826504869	7.996393263
SBF1	8.236633323	8.510413414	7.25519196	8.492322828	8.545617303
SBK1	7.59656001	6.72626003	8.199315667	8.119288848	7.914251515
SBK2	8.104866828	9.095162899	8.186373323	9.014846778	7.819085697
SBNO2	8.722452253	8.164979273	7.03146702	7.835434535	7.698300838
SCAP	11.48840455	11.05959848	9.749004051	11.21493335	10.70299608
SCARA3	7.682588111	7.448310273	7.257525758	7.312858101	7.675893758
SCARA5	6.975978909	6.767482869	6.961503899	7.180020091	7.13105099
SCARF1	8.052783737	7.757833798	7.30673204	7.562285172	7.850180343
SCARF2	8.940763364	9.839882283	8.45451298	8.451550535	8.765233303
SCARNA17	11.87687449	12.38063147	12.66985986	13.33661988	13.18858184
SCCPDH	8.605125889	8.630379313	9.191849071	8.708467242	8.55725397
SCGB1A1	8.112876232	9.322592778	7.400864778	7.587005707	8.280707687
SCGB1C1	5.996460354	7.709201212	7.817596162	7.459405444	6.398440768
SCGB3A1	6.244189293	7.31374401	6.362142859	6.347049828	6.284925121
SCGB3A2	9.763138212	9.943097475	8.789683394	8.994589434	9.189045596
SCLT1	9.971733293	10.07107569	10.35284213	10.58579258	10.60507136
SCML4	8.192266657	8.335861768	8.446641939	8.316570081	8.707057788
SCN3B	7.113720848	6.763870838	6.977817737	6.60520404	6.988144556
SCNN1D	7.234805667	6.702267949	7.097914788	7.164674212	7.097914788
SCO2	9.890135131	9.951175646	10.19332507	10.66105788	9.62302801
SCPEP1	10.28089537	10.29660096	10.41626133	10.54078574	10.22164118
SCRN1	8.146343545	8.299695162	8.813595202	8.287081333	8.23139899
SCRT2	10.16642595	11.93486375	11.00496707	9.226702434	10.99186298

SDCBP	10.80091358	9.95201103	10.92337564	10.47123164	10.15875628
SDCCAG10	8.176718545	8.524356212	7.840343939	8.20265299	8.319901444
SDF2L1	11.08936677	11.71042621	12.19727311	11.57765358	11.64637724
SDHB	9.604175636	9.239472869	10.42622906	9.438094949	9.267923343
SDHC	6.995550687	6.842273677	7.718373848	7.225864141	6.506514626
SDK2	8.252372808	8.751946515	7.925642	7.967113929	8.598980253
SDPR	9.254890202	10.17440526	10.28710845	10.0672481	9.644080808
SDSL	6.950902859	7.518993111	7.267888182	6.956363394	7.350681061
SEC11C	9.680349586	9.998242414	10.63752253	10.2600862	10.53505365
SEC14L1	10.16743096	9.892629697	10.73264046	10.84568098	9.876096838
SEC14L4	7.517436172	8.550897414	7.970926879	6.994245051	7.987573434
SEC16A	8.660166475	8.177528808	9.300367869	8.790654051	8.116577384
SEC16B	9.143279535	9.805520727	8.660608859	8.69919198	9.227275152
SEC22B	8.814101707	8.614442374	8.057864374	8.738283333	8.475210818
SEC24C	6.959189505	6.371798101	6.188743172	7.015941667	6.232553091
SEC31B	10.47820756	10.77890896	10.92060078	10.41846051	10.62280049
SEC61B	11.15183093	11.11856721	11.8358519	11.308834	11.27499716
SECTM1	10.05780386	9.430738808	9.164545343	9.47593803	10.03123363
SEDLP	7.870669919	7.182269455	8.361756343	7.030955384	7.372842667
SELENBP1	11.16579624	11.73070023	11.16888788	11.24646499	11.9875501
SELK	12.41931404	12.79024082	12.91656586	12.16036216	13.33155169
SELL	9.996420616	9.551451798	8.986932636	9.558925838	9.455015404
SELP	7.439228222	7.736619182	7.587005707	7.671246505	6.945903495
SELPLG	12.60396528	11.78023213	10.68687892	12.44950322	12.0838996
SEMA4A	8.362142131	7.558798354	7.553468394	7.94734596	8.040575909
SEMA4B	8.860747333	8.156772778	7.966742939	9.061091758	8.944409727
SEMA6B	7.457632404	9.131560131	6.634850818	7.171072313	7.481960152
15-Sep	11.43565115	11.51867768	11.78498903	11.85889751	11.75662781
1-Sep	7.238770545	7.665987182	6.806487737	7.838558162	7.839647131
14-Sep	10.02821235	8.756396081	8.957944414	8.942313313	9.590351455
SEPW1	10.93494087	11.68038318	11.82852227	11.37670858	11.35657003
SEPX1	13.27418186	12.31089415	11.87687449	12.64708972	12.20960187
SERINC1	10.73661507	10.12610261	10.41530743	10.61743608	10.26832521
SERP2	7.270153101	6.99955204	7.189410667	6.884060404	6.467370697
SERPINA1	12.7494647	11.4481816	11.01531103	12.20640951	11.76588718
SERPINA6	7.313208869	6.80380202	7.238133434	7.118793697	7.442908828
SERPINB1	11.78023213	11.06877356	11.70557621	11.37131374	11.43005301
SERPINB9	7.032569455	7.326042475	7.516462556	7.465036929	7.313451111
SERPINF1	8.077230061	8.154321646	8.323598384	7.835124576	8.185177828
SERPING1	6.182415283	6.280830838	6.025991232	6.141738101	6.098804071
SESN3	9.466304111	10.01168912	9.875361242	9.817379515	10.60383217
SET	8.005727263	8.414282596	7.318539616	8.385691051	8.297224525
SETD3	6.751979343	6.962574283	7.359577545	6.992656859	7.064243152
SETD6	7.286117455	7.62575301	7.431389424	7.486457414	7.936644384
SEZ6	6.644710828	6.418014596	6.762834384	6.539575101	6.894670869
SEZ6L2	7.339214566	6.704918465	8.006915303	7.785940475	8.122747707
SF3A2	13.87263802	15.29549959	12.16036216	12.6076243	13.56234785
SF3B2	11.11560115	10.86173253	10.67081044	11.47682743	11.04464523
SFRS11	10.48310631	10.68452674	11.47871073	11.05651949	10.90057618
SFRS3	11.50261564	11.29076429	11.77305263	12.0838996	12.02811467
SFRS6	7.01811702	7.928481586	6.719301162	7.088019323	6.729176758
SFRS8	9.11211799	9.101086141	8.498503737	9.011546121	9.007167758
SFRS9	9.730279475	9.426142444	9.113305192	9.898158111	9.453743545
SFTPA1	7.566773798	6.540677515	8.575566677	8.420387707	8.833337414
SGK1	11.13240418	11.19447199	10.52904685	10.88430477	10.49466708
SGSH	10.04421951	10.36781996	9.097596465	10.12041459	9.960976253
SGSM2	8.563941778	8.532980343	7.539487212	8.617420111	8.368326869
SGSM3	9.403514808	9.548028869	8.757866131	9.661659101	9.466304111

SH2B1	6.971547919	6.981335535	6.251035687	7.120752303	6.940479424
SH2D1A	7.370383778	7.758961677	8.423588242	7.759936788	7.828260424
SH2D1B	7.589604303	7.277222202	6.954672071	7.910120242	7.066192545
SH2D2A	9.187244566	9.007843273	9.180906778	9.480384485	9.378583414
SH2D3C	9.067241768	8.287888051	7.959080606	9.123297364	8.538919798
SH2D5	7.191229121	6.90271597	6.859461465	6.82385998	6.59504903
SH3BGRL	10.65871503	10.31378621	11.17378617	11.24147284	10.9102587
SH3BGRL2	7.545628697	7.768772758	8.219387636	8.254484434	7.732919727
SH3BGRL3	12.29275278	12.30465105	11.5386618	12.67378338	12.70787607
SH3BP2	10.156858	10.0079343	9.058356636	9.536330707	9.800083455
SH3BP5L	9.784374313	9.021393263	8.265565879	8.30460302	8.624974828
SH3GL3	6.575874879	6.247479727	5.902464323	6.602427343	6.972058485
SH3GLB1	11.95261936	11.14211434	11.54871614	11.58421758	11.71042621
SH3GLB2	7.335878859	8.052417212	6.426632697	7.510907818	7.545335788
SH3TC1	8.372433475	8.247392788	7.787863889	8.622197111	8.074508444
SHANK1	6.980492495	6.223158818	6.923167202	7.292561596	6.570518374
SHARPIN	10.88945836	11.86125412	10.52247938	10.59809845	11.15808934
SHD	9.046362323	8.71485401	8.833963152	8.786134101	9.547339828
SHH	6.071029081	6.552098808	6.929631566	6.26630199	6.31184304
SHISA4	12.24649354	12.42943771	12.2293359	11.93178106	11.95514832
SHISA5	10.72516317	10.32531651	10.49830212	10.7442872	10.6426412
SHKBP1	9.344138121	8.255317818	7.294583061	7.831854586	8.41335802
SHOC2	10.52904685	10.21975253	10.06822268	10.52476206	10.40278823
SHROOM1	7.452458778	6.744878414	6.903511051	7.855731	6.98587896
SIAH1	7.469040515	7.239990273	7.569899606	7.063477202	7.023140616
SIAH2	9.47593803	10.87637444	10.97185531	9.687048303	11.11560115
SIDT2	11.43773109	11.65261158	11.78265022	12.20960187	11.87912023
SIGIRR	10.76737368	11.15503783	11.46539851	11.86922315	11.76804793
SIGLEC10	9.742895778	8.605125889	8.374810828	9.512482222	8.786134101
SIGLEC5	8.543686687	10.31095185	9.542425758	10.3779146	10.08145981
SIGLEC7	8.433468657	7.721939444	7.016197848	8.284876758	7.684965778
SIGLEC9	8.420871687	7.544716788	7.101049919	7.859670455	7.629016404
SIGLECP3	8.345781111	8.297224525	8.572163455	8.302062758	8.968391182
SIK1	9.599465606	9.879566364	8.609511	8.938439909	9.436898869
SILV	7.964914919	7.93027604	7.500795283	7.065103374	7.56409603
SIPA1	11.65455063	11.06573849	10.19528987	11.63837311	11.10035391
SIPA1L1	11.15666056	10.94640834	10.75737971	10.55070525	10.60743282
SIPA1L2	9.143918798	8.254082232	8.137069859	8.212243444	8.58807297
SIRPA	9.217871889	8.160266646	8.348145404	9.21905796	8.22344601
SIRPB1	8.346585616	7.761767192	7.731201121	7.961659859	7.70365599
SIRPB2	9.453743545	9.135096212	8.920488949	8.990775909	8.907429495
SIRT2	8.868979273	8.82789604	7.714642404	8.554528737	8.637033606
SIRT6	7.580266434	7.240898899	6.658129061	7.568310444	7.59656001
SIRT7	10.65246846	9.728754818	9.288999586	10.40278823	9.981718727
SIT1	7.90609097	8.199315667	7.326042475	8.055566596	8.160266646
SKAP1	10.6426412	11.19293398	11.44491584	11.35080354	11.16888788
SKAP2	9.526200354	9.042944374	10.4994288	10.02923217	9.831834717
SKIV2L	9.332795131	8.906910121	7.753435232	8.954798808	8.546545061
SKP1	10.47479655	10.56655729	11.38178745	10.86566339	11.33657706
SLA	12.33576886	10.96886286	11.29947244	11.54675633	11.01951417
SLAMF7	8.831800202	8.98371896	9.717365758	9.667708556	9.756840071
SLAMF8	7.101319909	7.299074768	7.221345788	7.027167101	7.128773535
SLAMF9	8.156772778	8.029836697	8.317411434	8.425403333	7.982442283
SLBP	9.287819051	9.468134182	10.31795252	9.404333879	9.174120707
SLC10A3	10.58469265	11.22119175	11.12935449	10.35917202	10.24828231
SLC11A1	11.47322198	9.838413929	9.118629636	10.81664077	10.20358283
SLC14A1	6.965182525	7.405524869	7.409335747	6.834065141	7.899682051
SLC15A3	11.44319437	10.93646713	10.47123164	11.26446827	10.8193093

SLC15A4	10.24828231	9.442729677	9.627013505	9.904237354	9.772250768
SLC16A11	8.27861104	7.615901626	8.512201869	8.225777414	9.274850788
SLC16A3	14.10328164	13.16824615	13.0147344	13.54965001	13.29117716
SLC16A5	12.76207554	12.06397065	12.28693608	11.33477589	11.81439214
SLC16A8	7.633233848	6.743297485	7.172411071	8.129067798	7.074173121
SLC17A7	8.464558212	9.017695354	8.074904687	7.974325596	8.441767061
SLC19A1	10.05688349	9.282486081	9.591133828	9.469429253	9.607593848
SLC1A5	6.965717616	7.675167556	6.239248505	7.183404394	7.334066545
SLC1A7	7.003455566	6.808747657	6.91094498	7.738613697	7.451183323
SLC20A1	9.092457495	9.280885222	9.377908	9.193817232	9.548028869
SLC20A2	7.697940283	7.897743687	8.187593091	7.517772121	7.646953505
SLC22A10	9.613206141	9.569063343	8.491898242	9.028300162	9.978126798
SLC22A12	6.993183869	7.14692997	6.670140313	6.854415313	6.931501667
SLC22A15	9.736957071	8.77298297	9.047491869	9.190767364	8.936938576
SLC22A17	7.916712838	7.415639222	8.10140802	7.772843323	8.298396535
SLC22A18AS	10.19757035	12.34577038	9.159196141	9.169326323	10.8935809
SLC22A23	8.184050869	9.45706402	7.523206263	7.651593505	8.08112003
SLC22A4	9.189045596	8.287438535	8.266388465	8.136235949	8.589797808
SLC22A5	6.887565899	7.11962903	6.818245747	6.468711152	7.354804152
SLC24A3	7.113452899	7.341966626	7.495424616	7.154661646	6.692767899
SLC24A4	8.51269104	8.960773354	7.826504869	8.233006697	7.775998535
SLC25A20	8.76478299	8.464558212	8.063008152	8.77608297	8.485174677
SLC25A22	8.696280707	9.074692404	8.498167798	8.582586091	8.854095293
SLC25A24	9.888393747	10.11760527	9.094076404	9.449948384	9.355403384
SLC25A29	9.923658606	9.025691242	9.093526566	9.014335657	9.031713778
SLC25A32	7.705454838	8.242431778	7.476561202	7.962004212	8.25570701
SLC25A37	11.30506756	11.29399858	10.84302834	11.33657706	11.43380494
SLC25A38	7.99823603	8.502042192	8.972927475	8.245649515	8.665646899
SLC25A39	14.24162707	15.09418019	13.75520609	15.10875812	14.82007331
SLC25A44	10.76239578	10.0053333	10.43872423	10.52904685	10.42175216
SLC25A6	10.71279176	11.3733012	10.23850383	11.21816154	10.99046505
SLC26A1	7.052522778	7.590558081	6.397416404	7.208855192	7.376746737
SLC26A11	9.492465394	9.910960495	10.39296715	10.16937171	10.31693391
SLC27A1	10.5041569	11.00183361	10.78551178	10.76361324	10.64005918
SLC27A3	8.842109	8.791657869	8.454049747	9.420326576	8.842109
SLC29A1	7.218399283	7.724369343	8.29291099	7.091827414	6.833358384
SLC2A1	9.196689051	9.402814081	9.196132455	8.81572204	9.595333525
SLC2A14	12.13538265	10.75737971	11.24323027	11.9982543	11.46145043
SLC2A3	8.10326497	7.300031424	7.591410606	8.044191424	7.639990515
SLC2A6	7.157253707	7.552833101	7.032569455	7.617189485	7.311327071
SLC31A2	12.10951308	11.11388317	11.80342824	10.56908161	10.72633412
SLC32A1	6.506079909	5.784595657	5.825182131	6.419328293	5.883232879
SLC34A1	8.891390919	8.004282899	8.902484929	8.41552702	8.990220404
SLC34A3	11.81221198	13.88591407	10.72516317	11.29744903	12.54589185
SLC35A1	8.437898414	8.248137606	9.174120707	8.560348606	8.423588242
SLC35B3	7.780448354	7.577325354	8.138908141	7.344278889	7.144617717
SLC35C1	8.809442889	8.347358778	8.831800202	8.904793061	8.848914293
SLC35C2	7.90290904	7.462771485	7.08136002	7.953486636	7.606370131
SLC35E4	16.57174008	15.76560628	17.43906183	17.29704085	16.64557528
SLC36A3	8.795325869	8.374363061	8.257595869	8.187136596	8.750392838
SLC37A2	8.116577384	9.102676354	9.277455131	8.221010253	8.285708747
SLC37A3	8.14677703	7.123768636	7.692888131	7.476910626	7.476910626
SLC38A1	9.552821717	9.890949131	9.752732475	10.12697515	10.26541026
SLC38A5	9.200042253	10.05880999	7.909031556	10.12909315	9.630502939
SLC38A6	7.382214869	7.735932323	7.52646604	7.358688323	7.556609313
SLC39A1	8.188379657	7.469344313	7.944736172	8.177528808	7.223533071
SLC39A10	6.256882495	6.566529242	6.589551859	6.433824646	6.392522354
SLC39A8	7.962004212	8.441767061	8.355847778	7.991889949	8.379056919

SLC40A1	10.42736719	10.13862753	10.10365156	10.53623255	10.03385507
SLC41A3	8.767872848	8.81572204	9.718307192	9.169862626	9.393335687
SLC43A2	9.362238061	8.830117929	8.631321253	9.251353071	9.057828929
SLC44A2	11.64848719	11.32376318	10.49466708	11.88148631	11.22600179
SLC45A3	7.999737535	8.623636919	7.554702939	8.233421293	7.157774636
SLC45A4	8.901935111	8.411285283	7.59859104	8.795325869	7.830100949
SLC46A2	6.351287939	6.577039475	6.876878333	6.700124848	6.377923929
SLC48A1	9.507772687	10.42062148	9.800904051	9.523397737	10.12408141
SLC4A1	8.451947071	9.338458455	8.481411768	8.546056747	9.321890899
SLC5A2	9.626304798	9.793942798	8.836542848	9.677492576	9.76546198
SLC6A10P	8.694840596	9.940265465	7.901142576	9.027136657	9.54312803
SLC6A17	6.98587896	6.036314939	6.215679889	6.987033364	7.412191343
SLC6A18	7.124596343	6.741204455	6.943066869	6.894135505	6.887288828
SLC6A19	8.223899788	7.622739535	8.037475929	8.429582192	7.976858394
SLC6A5	7.567065616	7.596851556	7.646275747	7.869992939	8.290726727
SLC6A8	8.858269384	10.03026493	8.680072242	9.303966081	9.649399939
SLC7A7	9.972781707	9.571117879	10.51771241	10.31013098	9.941224515
SLC8A1	8.251922909	7.733228788	7.411561343	7.705120374	7.51810503
SLC9A3R1	11.80342824	11.22294817	10.48310631	11.68700199	11.47682743
SLC9A3R2	7.736260545	6.987599808	7.111681374	7.278422556	6.964624475
SLCO1B3	6.299210717	5.969095798	6.332476354	6.600799566	6.831619364
SLCO3A1	8.794788636	8.192266657	8.094989869	8.31214998	8.459596535
SLFN5	7.164102909	7.603389051	7.62509899	7.682204525	7.561051303
SLIT1	6.695201899	6.782198505	7.171874212	6.780217071	6.599359394
SLITRK4	8.405727848	7.581657414	7.537817576	7.906781182	7.389870293
SLPI	10.32531651	9.521972354	9.721175788	10.02741959	9.542425758
SLTM	9.325601172	9.133284677	10.08799683	9.390110384	9.503271071
SMAD1	6.826408263	7.014842626	6.729463253	7.052221778	6.765091091
SMAP2	9.673359929	8.667049253	8.443175586	9.277455131	8.968910758
SMARCC2	11.39382595	11.58195332	10.52364746	11.35858946	11.57765358
SMARCD2	8.40783399	7.710578727	7.756889051	8.244006505	8.017539697
SMARCD3	11.09885051	11.15666056	10.88166557	10.51174846	10.27338352
SMCHD1	10.39641485	9.812775374	10.23660558	10.24227579	10.15783547
SMCR5	9.783680727	10.22865659	9.422377343	9.660861778	10.10454015
SMG5	9.257979848	9.634846707	9.371514253	10.36687712	10.46664928
SMN1	10.3305428	10.24038579	10.14713571	9.222631263	10.37026232
SMNDC1	10.3986043	10.06255975	9.818059192	10.11246778	9.997192202
SMOX	9.000672535	9.530933293	9.559682182	9.733950273	9.457741556
SMPD1	7.430156263	7.738299929	7.021742939	7.769805091	7.493565131
SMPD3	8.337550606	9.155079848	9.540446515	9.184000242	9.444057586
SMR3A	11.75662781	12.03068847	11.18636156	10.34418014	11.86125412
SMYD2	7.959080606	8.512201869	9.065009323	8.458697343	8.301264212
SNAI3	8.667049253	8.439612798	7.727390434	8.368780919	8.660166475
SNAP23	8.961854929	8.644074768	9.663106646	8.929972798	8.587623212
SNCA	13.07947925	13.67554111	15.65613201	13.43523914	14.64328928
SNCB	6.832629131	6.885064545	6.958715091	6.925244263	6.766293242
SND1	10.02161965	10.55726192	10.86820493	10.43122177	10.93789932
SNHG1	9.379720636	10.0406788	10.61621963	9.497817424	9.829822242
SNHG12	9.344897333	9.957374697	10.56207051	9.901640636	10.16073454
SNHG5	12.99088515	11.52052132	11.75908761	13.07947925	13.22156044
SNHG8	8.039068889	8.970512081	9.189045596	8.401710747	9.376667182
SNRNP200	11.00183361	10.77507269	10.91903182	11.56988377	11.21958266
SNRNP35	8.637513768	8.360501364	8.927019859	8.460863192	8.291166909
SNRPA1	7.198597879	7.46337499	9.298556212	8.025864485	8.150096222
SNTB1	7.346567394	7.505175253	7.866656354	7.409045616	7.361380131
SNTB2	9.353057535	8.637513768	8.443956768	8.594861495	8.921533879
SNURF	7.394370293	7.729749768	7.792587404	7.695912303	7.684641212
SNX10	11.70138789	11.34732253	11.02851208	11.48840455	11.62739451

SNX11	8.454049747	8.055566596	7.373377929	8.253254586	7.935875313
SNX17	8.181196919	8.268937657	7.809001414	8.63794701	8.365106485
SNX22	7.501110505	8.093860848	7.59785203	7.45822897	8.431738505
SNX26	11.62739451	11.96947069	10.44195016	11.28207759	11.5612151
SNX27	9.269847242	8.458294828	8.557697889	8.663769939	8.282900293
SNX29	8.564366949	8.79722998	9.445852485	8.421742051	8.377562293
SNX3	10.95683616	11.01951417	11.91416468	10.93334198	11.83813795
SNX6	10.36687712	10.20164867	9.972781707	10.09682592	10.11150219
SNX7	6.417447212	5.914902455	6.538200697	6.405686404	6.713757434
SOCS3	8.032499636	7.466342606	7.953840929	7.511581475	7.693828071
SOD1	11.01951417	11.47682743	12.08128174	11.38577951	11.77802923
SOD2	10.50914282	10.18291229	9.656571869	10.01622438	10.03876748
SORBS1	7.195502081	6.672823111	6.928862212	6.92644801	7.200432616
SORCS3	5.925405303	6.512183152	6.66713102	6.397781283	6.559271747
SORD	8.076845152	8.288355808	8.39446903	8.098747556	8.076118121
SORL1	16.17943014	15.05177986	15.38120792	15.83165575	15.12351424
SOS2	9.738398616	8.910531141	9.321890899	9.311440606	9.276189121
SOX1	9.433849505	10.19236777	8.305787747	9.058356636	9.286110869
SOX13	6.778470374	6.475135818	6.283373303	6.891529131	7.680845586
SOX17	9.000069889	9.836777707	7.569572879	8.363862202	8.93638097
SOX18	9.776839404	11.59870577	8.457043091	9.048620525	9.27419499
SOX4	7.555333263	7.799524616	7.802180768	7.902229798	7.744459273
SOX8	9.430738808	10.52135963	9.069043081	9.883591758	9.173615515
SP110	11.40725584	10.91641235	11.27695531	11.28053313	11.29947244
SP5	7.532150707	8.341518747	6.688467242	6.970224646	7.324608717
SPARC	11.34381562	12.4956701	11.80104424	12.11841559	11.29076429
SPATA13	8.842639202	7.916391202	8.789132293	8.548304869	8.758871
SPATA2	7.200432616	6.648393616	7.599211495	7.040256253	7.06536003
SPATA20	8.221403505	8.445759657	7.519364889	7.304711495	8.585836293
SPATA22	7.682204525	6.884322333	7.799854778	7.005354859	7.944406939
SPATA2L	15.02501841	16.06827159	14.36070414	14.25895571	15.27762024
SPDEF	6.669931545	7.169469697	6.506079909	6.279884111	6.653328071
SPEF1	7.215185141	8.05903404	6.953635141	7.101319909	7.31523196
SPEG	7.406826051	7.819838162	7.762120596	7.622357354	8.060252263
SPG21	10.88302044	10.92060078	11.45738434	10.95849458	10.85654613
SPHAR	8.142197384	8.400464071	8.550414556	7.896955242	7.990677606
SPHK1	7.385857192	7.683675283	7.728044798	7.997498828	7.259665485
SPI1	8.606099333	8.099466788	7.15072903	7.710917071	7.803944101
SPIN2B	7.195224778	7.55847901	8.005065495	7.376100929	7.567404636
SPINK7	9.263851586	10.74690378	8.717364515	8.856895172	9.511857455
SPNS3	7.980934222	8.887109626	8.822318	8.483326677	8.404396899
SPOCD1	7.178076949	7.743136949	7.153217667	7.279952616	7.121365859
SPON2	13.62106704	13.09045797	12.97977545	14.57091557	13.3718573
SPOPL	9.725631121	9.10558002	9.003925646	9.044046343	8.878201818
SPP1	7.439837192	7.052522778	6.305155434	6.659312505	6.695720192
SPPL2B	11.71659127	12.35168631	9.938475404	11.31449746	11.45932673
SPRR1A	8.124973859	8.552208182	8.105629576	8.179581869	8.199709495
SPRR2C	6.895207929	6.431827616	6.932737172	6.66276004	6.183137202
SPRR2E	9.46304199	9.599465606	9.250770152	9.320738646	9.639623879
SPRYD4	6.817799576	6.953378071	7.484183869	6.825325	6.995800606
SPSB2	8.235388636	8.625920364	8.825911939	8.297224525	7.891912283
SPSB4	11.25700815	13.57574016	10.05523038	10.60277882	11.07013707
SPTA1	7.541090081	8.627322343	7.910975505	7.482605354	8.310875455
SPTAN1	8.282438737	8.417196576	7.643273222	8.334587636	8.461795505
SQLE	7.116617232	7.524103909	7.08018898	6.912275354	7.551492101
SQRDL	14.11151028	13.45466248	14.23242051	13.32669092	13.63435472
SRC	6.935569505	6.897750404	6.647920455	6.614995778	7.070939121
SRCRB4D	7.10076196	6.955441212	6.681164444	6.938149939	6.982909788

SRD5A1	6.933000202	7.266003808	6.518759273	6.682294404	7.168718192
SRD5A3	7.093254121	7.774617596	7.919459465	7.91707401	7.69973097
SRGAP2P1	6.706660646	6.722483515	6.232724131	6.702734242	6.746340717
SRGN	11.308834	10.22448985	11.02105469	11.29399858	11.00620373
SRP14P1	9.419020677	9.655088667	10.45877561	10.00886723	9.865550636
SRP72	8.050105303	8.426330949	8.408718253	8.198599495	8.57790302
SRP9	8.683087172	8.613985131	9.145078374	8.808928141	8.668931657
SRPK1	12.14191765	10.57028524	11.65261158	11.671419	11.55106122
SRPK3	6.827921778	6.403487758	6.944336869	6.637667434	6.946968899
SRRD	8.93587504	9.577319545	10.22070473	9.436357141	9.980879424
SRRM1	8.904793061	9.030004576	8.184399818	8.985325394	9.057196687
SRXN1	10.55726192	10.55602586	10.8605042	10.57028524	10.98037262
SSB	9.887613414	10.3305428	10.28899471	10.47820756	10.7532413
SSBP4	8.142577798	8.531655515	7.90869301	8.454049747	8.606099333
SSTR3	7.206373697	7.477241869	7.086968939	7.198026636	7.521885657
ST13	8.878776172	9.384699788	9.89348604	9.394567081	9.896649636
ST14	7.835124576	8.217270354	7.109400202	7.985458788	7.537489808
ST3GAL1	11.21016953	11.28207759	10.24727703	11.64044206	11.6137143
ST6GALNAC2	8.366948404	8.112876232	7.516753121	7.737346818	7.539167263
ST6GALNAC3	7.503050071	6.819189677	6.686319576	6.564057303	6.602666131
ST6GALNAC4	8.208296687	9.19736601	7.929187444	8.412956141	8.948168879
ST6GALNAC6	7.88183703	8.858692343	7.384295283	8.569735879	8.524356212
ST8SIA1	6.135884374	6.323943747	6.020522545	6.271799455	6.493490929
STAB1	7.512765949	7.270788949	6.733232384	7.634916323	7.219623727
STAG3	8.011643808	7.754082929	7.970233808	7.631889737	8.718769576
STAG3L1	8.956909848	9.185002364	9.207791152	9.097596465	9.337862091
STAG3L2	8.430929525	8.823889374	8.77962597	8.96575796	9.014846778
STAMBP	7.300950758	7.13936198	8.21550303	7.197446212	7.257203303
STARD10	8.298396535	8.293769586	7.457632404	7.834787162	8.032957859
STARD3	7.829693939	7.312252182	6.643746616	7.847720859	7.445432899
STARD7	9.147897788	9.546645747	9.695719162	9.805520727	9.617464455
STAT1	9.523397737	9.613873859	10.65246846	9.905938879	10.2187154
STAT5A	8.179951646	7.726017717	8.84935699	8.225261616	8.273483707
STAT5B	8.965143333	8.237365566	8.330618758	8.619848374	8.481851586
STAT6	13.64083669	13.48401144	13.39659876	14.14208591	13.89331098
STC1	6.363322586	5.835632263	6.592026141	6.903241343	7.261824374
STEAP4	8.590695768	8.066048727	8.479727162	7.867312374	7.572483949
STK10	13.15645152	12.28369472	12.09225922	12.68097339	12.44950322
STK11	8.449722687	9.572453101	7.997875909	8.350987111	8.932594273
STK16	6.69920203	7.01049403	6.695959364	6.517203657	6.914843414
STK25	8.744081071	8.78214798	7.942409707	9.054860525	8.730448515
STK32C	6.710071909	6.637907929	6.008093566	6.382217859	6.072015283
STK40	14.11997744	13.50997822	13.5160991	13.79381649	13.30795266
STMN1	8.822853162	9.468772697	9.953782444	9.103811545	9.432636424
STOM	8.366462404	8.946107263	9.591816061	9.126663535	9.424985495
STRADB	11.36245739	12.46466592	12.65819872	12.01956714	13.02518615
STRN4	10.15783547	9.781348374	9.677492576	9.74383503	10.34514895
STT3A	8.063898495	8.319901444	6.942257576	8.60413102	8.352537101
STUB1	10.6488688	10.75987116	9.91449603	10.95093974	10.81796524
STX11	9.087255717	8.584413131	8.914720172	9.112697707	8.188779434
STX2	6.998984111	7.391952354	7.531511131	7.28082202	7.825767899
STX3	9.759214424	9.027136657	9.408173051	9.230770667	8.903025495
STX5	8.991410131	9.097596465	8.352134333	8.841575667	8.794361798
STX7	8.712341879	8.027386222	8.614442374	8.832790172	8.768863636
STYK1	5.897922778	5.949701384	6.894670869	6.197522081	6.301115768
STYXL1	7.651593505	7.369172101	8.01602699	7.68569196	7.300950758
SUCLG2	6.997069465	6.792957485	7.332344778	7.226157253	6.661293222
SUDS3	9.299916465	9.286739242	8.518144394	9.408173051	9.801802283

SULF2	13.78737352	13.23864174	12.69676846	13.70143341	12.93862501
SULT1A1	9.53973704	9.192533242	9.694925798	9.598265626	9.225515485
SULT1A2	10.9991534	10.71145754	11.41071085	11.37490296	11.31661678
SULT1A4	10.84974643	10.34514895	10.80239406	11.03452834	10.71860391
SULT1B1	8.926362727	7.55499601	7.728361283	7.956286283	7.599486586
SUMF2	6.648393616	6.800782586	6.178466768	7.085030667	6.785085162
SUMO1	8.000144121	7.619228051	8.298396535	7.973586899	8.07563398
SUMO1P3	9.124970051	8.587623212	8.509502414	8.911610283	8.890373556
SUPT5H	9.042944374	8.988577162	8.313601758	9.496413788	9.144457596
SUPV3L1	6.927779576	7.108229697	7.669946162	7.332669495	7.786298434
SURF4	8.818695101	8.778117444	8.716387192	8.78214798	8.808928141
SUSD1	8.874191697	9.244930505	9.234418071	8.791657869	8.339926909
SUSD3	10.79420974	11.53473316	12.21630458	11.29947244	11.46320141
SVIL	8.898709273	8.179581869	9.051375475	8.625486818	8.06745399
SYAP1	8.930433505	8.758871	9.036569232	8.759332636	9.513164303
SYDE1	6.895973717	6.305339354	6.745632475	6.776493808	6.816006121
SYK	10.40178901	9.837575172	10.02821235	10.13276962	9.752732475
SYMPK	9.559682182	9.611261414	10.62280049	10.32849396	10.04963797
SYN1	9.94402302	10.64738653	8.485631939	9.475206131	9.923658606
SYNGR4	10.21769501	12.47130519	6.808747657	9.473222667	10.46342714
SYNM	6.28356798	6.110613111	6.96124304	6.367075323	6.17582696
SYNPO	7.859293	8.459227657	7.140902202	7.441392919	7.869992939
SYTL1	10.60863639	10.55842197	9.873591818	10.41022215	10.42409385
SYTL3	10.05236285	9.530205505	9.796940758	9.679062616	9.266188263
T	7.041369354	6.723761636	7.225001677	6.995294596	6.906335848
TAAR2	7.972012263	7.082724758	7.850890354	7.543396525	8.489178071
TAAR5	7.495424616	7.814093232	7.557268586	7.775998535	7.901142576
TACC3	9.878686616	9.104371838	9.124970051	9.552159131	9.347406172
TACR1	6.471481465	6.308888444	6.32750801	6.716343364	7.101049919
TACR2	8.560348606	8.810986707	8.281588828	8.19458801	8.719245818
TACSTD2	6.439789939	6.439991313	5.764112707	5.819316182	6.29391397
TAF10	10.3896003	10.34626898	9.329922273	10.39641485	10.21279484
TAF11	9.01707996	9.084401768	10.17030341	9.122783717	9.141588162
TAF15	7.326942212	7.250545919	7.364550071	7.526167626	7.462771485
TAF1B	6.331704263	6.766018101	7.358688323	6.849836323	6.949065
TAF1D	8.929384727	9.053043889	9.713188212	9.457741556	9.760900556
TAF4	7.795765162	7.953840929	8.098747556	7.508036465	7.657256192
TAF6	10.39521192	10.75062885	10.25519474	10.36903954	10.55333109
TAF7	12.48124823	11.67849095	12.43944324	11.53239215	11.96059797
TAGAP	9.27419499	9.194378495	10.34100163	9.106199707	9.122783717
TAGLN2	11.31449746	11.1453264	10.39641485	11.3733012	10.68572341
TANK	9.094691273	9.105035414	9.993897848	9.155079848	9.269224374
TAOK2	10.10631712	10.61133106	9.256085253	9.587719636	9.994772242
TAP1	13.06967433	12.26162026	12.87421343	12.80166365	12.59323492
TARBP1	6.802083788	7.126812859	8.126253929	7.089925434	7.277222202
TARP	7.127947263	7.311629869	8.459596535	7.513736343	8.53537201
TAS1R1	7.54731098	7.924172566	8.040575909	7.791885697	8.189194404
TAS2R44	7.177746566	6.804729515	7.027744404	7.006187899	7.346280818
TAZ	8.967895455	8.831800202	7.872350636	9.092457495	8.846850111
TBC1D10A	9.118059475	9.129114848	9.514496606	9.257324	9.774477667
TBC1D10B	12.68097339	14.13521971	11.32376318	11.98440811	12.35798465
TBC1D10C	14.7528858	14.1830221	15.27762024	14.61202705	14.32804886
TBC1D14	10.60635433	10.16743096	10.70534521	10.36032821	9.822898081
TBC1D17	8.121211444	7.98285402	7.44169298	8.016751283	8.268052495
TBC1D2	8.374810828	8.292509485	7.44801498	8.284505182	8.20265299
TBC1D26	6.357131071	6.083080859	6.228051758	6.088380414	6.313155323
TBC1D3	11.8164662	11.26262606	10.87092264	10.90606985	11.16281786
TBC1D4	7.053922394	7.834787162	7.838558162	7.495088838	7.429246232

TBC1D8	7.424346313	7.782462626	7.545628697	7.36166898	7.402701172
TBC1D9	8.443175586	9.128533354	9.355403384	8.821885465	8.615838
TBC1D9B	9.86223396	10.09896607	10.18000397	9.86223396	9.551451798
TBCEL	7.295980475	8.386133566	8.588536172	7.389558374	8.997807768
TBKBP1	8.794361798	8.840590677	8.432150758	8.631321253	9.109120374
TBL1X	9.769994879	9.441908576	8.786134101	9.12042397	8.908935182
TBL1Y	9.529469889	9.081927515	8.059832677	8.847386828	8.78214798
TBX21	10.6240143	10.97767761	11.10035391	11.58621592	11.28370047
TBXA2R	8.814624313	9.062782333	9.021393263	8.978723121	8.83948901
TBXAS1	8.755421576	8.379884889	8.664725051	9.106823152	8.729485838
TC2N	7.377037515	8.166172404	7.322217273	7.599486586	7.810269677
TCAP	8.624974828	8.825911939	8.151663141	8.13932801	8.389653889
TCEA1	8.588971687	8.647073404	9.067856838	8.631899566	9.147897788
TCEAL3	7.937868929	8.410410394	9.254253192	8.408718253	8.390114414
TCEAL4	8.377090384	9.037153444	9.582110717	8.458294828	8.852576737
TCEAL6	7.540114586	8.012698303	8.873520545	8.050484323	8.032161838
TCEB1P3	7.035420475	7.210091465	7.08160301	7.011288768	7.275765808
TCEB3C	6.709075525	5.819543697	5.776679677	6.074368697	5.78810299
TCERG1	6.753240879	7.173022687	6.56193898	7.091067131	7.12322297
TCF20	9.821195081	9.406122899	8.957404525	8.965143333	9.361074667
TCF3	10.22557955	11.24001011	11.14211434	10.41530743	11.33477589
TCF4	7.460268556	7.823083152	7.079910848	7.626470505	7.708578687
TCF7	9.31327601	10.17030341	10.15875628	10.37686258	10.080472
TCIRG1	13.85817003	13.13007894	12.99088515	13.49745762	13.16151113
TCL1A	12.49170848	12.10414281	12.77841715	11.26623333	12.02514953
TCL1B	8.023506091	7.74961303	8.025051697	7.511268929	8.113343889
TCL6	8.404396899	8.520243879	8.177146606	7.976116929	8.303793737
TCN1	7.583692414	6.741002212	7.091827414	7.275223848	6.416313212
TCP11L2	7.207252646	7.809982667	7.65951298	7.355380455	7.42989803
TCTE1	6.68968302	6.155698343	6.698531303	6.582439566	6.585931495
TCTEX1D2	7.245319404	7.392218323	7.173022687	7.009472515	7.332669495
TDRD7	10.24511785	9.909202788	10.84974643	10.10187506	10.13862753
TDRD9	6.990730697	7.295703091	7.030426444	7.639261768	7.186327495
TEAD3	9.123297364	9.335891293	8.686679798	8.098302202	8.814624313
TEKT5	6.76700904	6.395988485	6.741460808	6.575452697	6.925244263
TELO2	8.195856758	8.53628698	7.874403566	8.473392556	8.555429434
TEP1	7.901561475	7.952386505	9.343457394	7.952774121	8.775519707
TEPP	6.62508102	7.720869455	6.795397778	6.104869535	6.648393616
TERT	7.330546788	6.551390626	6.778194636	7.297158313	6.47989397
TESC	15.19830883	15.67809237	16.39933956	15.39581235	15.76560628
TET2	8.822318	8.302062758	9.036076414	8.944409727	8.785177889
TFDP1	6.524057606	6.920203848	6.857502071	6.582672778	7.353244222
TFE3	8.933684374	8.615330515	7.648319737	8.360501364	8.429582192
TFEB	11.12315281	10.58117749	10.0728998	11.08435313	10.75453103
TFF1	7.775269606	7.450590374	7.344882899	7.454874889	8.166602566
TFF3	7.520648323	7.284692949	7.56320098	7.68740998	8.287438535
TFIP11	8.953653768	8.565878434	7.793372141	8.768863636	8.097566687
TGFB1	9.070117818	8.941359636	8.027802949	9.133918929	8.757405333
TGM2	7.756512818	8.123132566	7.876138384	7.357775939	8.334162556
TH	6.956131545	7.643944697	7.042911333	6.754244737	7.184502222
THBD	11.29076429	10.2026692	10.46342714	10.78014639	10.43122177
THEG	6.969423263	6.651024444	6.662501485	6.451458394	6.619744263
THEM5	11.79615897	13.95167504	9.631972414	10.61133106	11.48655657
THOC3	8.412956141	8.656835556	9.715870253	8.943348051	9.001272616
THOC4	7.893273434	8.186373323	7.247912131	8.157542717	7.956286283
THY1	12.28985985	14.4185289	11.39382595	11.21668249	12.95787308
THYN1	8.12156602	8.341133162	9.35188598	8.006915303	8.403930677
TIAM1	10.47370876	10.05607537	9.799317404	10.04421951	9.76471501

TIAM2	9.311440606	8.383846919	7.69973097	9.569854202	7.727011939
TIGD3	9.235747242	8.576430182	9.081927515	9.166446616	8.230181798
TIGIT	7.546292778	7.87472	8.846850111	7.568036061	7.362680232
TIMM10	7.573484121	8.277405556	8.071703475	8.328154263	8.353342091
TIMM13	10.14534091	10.71731517	11.52645916	10.54190997	10.6313464
TIMM23	7.562928222	7.709908667	8.722452253	8.009440313	8.229339455
TIMP1	9.081281455	9.647013263	8.391460737	9.179819687	8.910531141
TINP1	9.436357141	9.739033131	10.55966675	10.33375706	10.55474917
TIPARP	10.02923217	10.13768673	9.468134182	9.770750222	9.642500081
TJP3	8.472435657	6.60006604	8.22344601	7.893940788	8.780633818
TK1	8.854095293	9.800083455	9.530205505	8.687053253	9.041820404
TKT	13.98081649	13.21058954	13.33155169	13.52959744	12.93430142
TKTL1	8.757405333	8.36093298	8.18481599	9.306330646	8.289212586
TLE3	9.733950273	9.008956374	8.148308414	9.443424081	9.070117818
TLN1	8.493653778	8.124973859	7.133298475	8.084674061	7.70790696
TLR1	11.96636849	11.30506756	11.71443529	11.47871073	11.34732253
TLR2	12.77015535	11.89628941	12.04990566	12.17513939	12.39973853
TLR4	9.081927515	8.123471404	9.387656788	8.610845737	8.341518747
TLR5	8.212243444	7.497675424	6.860671293	6.922922101	7.109111172
TLR6	9.065009323	8.35372597	8.848410616	8.390560192	8.572163455
TLR8	9.746868596	8.626355717	9.354902374	9.167758485	9.052474394
TLX2	7.688370495	8.35667099	7.390752071	7.493254434	7.740855141
TM2D1	10.22361636	10.2861211	9.917863333	9.788274313	9.935661677
TM6SF1	8.778117444	8.22479296	8.471953717	8.585836293	8.851018515
TM6SF2	7.242384899	6.528194515	7.80435998	7.235098212	7.703321374
TMBIM4	10.34717129	10.12041459	10.21583282	9.675904202	9.604846424
TMC5	6.661293222	7.163863636	7.186327495	6.512183152	7.034331232
TMC6	9.339095222	9.326739394	8.568768141	9.576740677	9.693515768
TMCC2	8.053971495	7.948048111	8.275152535	8.665646899	8.915814212
TMCC3	9.590351455	9.341818545	9.537497323	9.21067496	9.071813909
TMCO3	8.396125929	7.646275747	7.969427899	7.810980737	7.770819434
TMED10	9.014846778	9.199527535	10.00886723	9.290152727	9.273022949
TMED2	8.164230707	7.932066141	7.521266808	8.178694051	7.919797354
TMED8	9.727055768	8.846355515	9.481000434	8.780633818	9.337187111
TMED9	7.472500828	7.239727717	6.917518081	8.055916879	7.088821283
TMEM105	7.549557061	6.81164602	6.927522051	7.298118263	6.64590104
TMEM106C	9.293068828	10.02821235	10.05780386	9.684048687	9.696433626
TMEM114	8.406205495	10.02923217	7.831854586	8.083806798	8.250194202
TMEM120A	10.09426568	9.206093556	8.90531298	9.477181455	9.042944374
TMEM120B	6.222757071	6.439547263	6.665912939	6.271221162	6.385648535
TMEM121	8.845785121	9.107945202	9.035040848	8.338686465	8.189547909
TMEM123	10.03385507	10.29949835	10.22668357	10.34100163	10.07388225
TMEM125	6.687475475	6.390360535	7.11316801	6.732525747	6.79323798
TMEM126B	7.761767192	8.123844788	8.583996586	7.936282586	8.10683704
TMEM129	9.515743253	9.857940545	8.82842597	9.216574283	9.368224626
TMEM140	9.993897848	9.070661273	9.477181455	9.772980061	9.436357141
TMEM145	9.607593848	8.880853535	8.846355515	8.537956091	9.736139061
TMEM149	11.61117883	12.13538265	12.75396286	12.05489479	12.25572441
TMEM14B	9.671170444	9.829822242	10.91356479	10.08145981	10.10278369
TMEM14C	9.335272182	9.641007131	9.739859192	9.640429374	9.588410576
TMEM151A	7.834378071	7.821625848	7.155513182	7.726392606	7.876418838
TMEM154	11.91651973	11.40725584	11.14866374	11.17213305	11.33848921
TMEM158	11.00496707	12.15440913	11.55524693	10.74945851	11.14211434
TMEM167A	8.467667768	8.237787788	9.023071899	8.402148465	8.399975414
TMEM170B	9.188424626	8.917069505	8.932594273	9.293068828	9.206631485
TMEM171	6.650108172	6.78758299	5.97184804	6.095353909	6.625311455
TMEM174	6.506758646	6.07011403	6.037606273	7.263531485	6.247784949
TMEM175	14.89443037	15.86132141	13.58209631	14.3093669	15.21350605

TMEM176A	7.287262414	8.928828384	8.448456354	8.996148737	7.21346404
TMEM176B	6.766521566	8.100639	7.748548515	9.195011616	6.173797535
TMEM180	8.179581869	8.054318061	8.123471404	7.980256929	7.699362081
TMEM181	8.401317182	8.585303495	7.357456071	8.124212202	8.475612051
TMEM183B	10.04963797	10.56421743	10.82290783	9.816676162	10.4700758
TMEM184B	12.75784116	11.98999321	12.72219022	12.17829278	12.13867659
TMEM187	7.815532475	7.948408364	7.908308323	7.860764394	8.079553929
TMEM188	8.199709495	7.59785203	7.67303801	7.884680111	7.814874545
TMEM191B	13.07454735	14.7528858	11.20163629	12.41931404	13.09463792
TMEM194A	6.471690111	6.640209303	7.403718889	6.553884717	6.691780525
TMEM201	10.04776272	9.609027141	9.808672222	10.87092264	10.47584162
TMEM204	10.5752557	11.43005301	11.94745099	11.28565422	11.15503783
TMEM214	7.028809465	6.981618121	6.975978909	7.088250616	7.345977091
TMEM217	6.501046121	6.015023111	5.926247798	6.853963192	6.296516586
TMEM218	8.137069859	8.310875455	9.488971182	8.645541747	8.745474636
TMEM219	9.383480283	9.354289121	8.937936253	9.418269535	9.408173051
TMEM222	8.777050384	8.812013889	7.859670455	8.749346626	8.528915121
TMEM40	10.01340346	9.569854202	10.91176188	10.65127749	10.36687712
TMEM42	9.70574997	10.2572153	10.71860391	9.838413929	10.0728998
TMEM45B	8.734806657	7.54731098	8.906407303	8.69723302	8.71485401
TMEM49	10.99763472	10.64379219	10.5029511	10.73788216	10.49713487
TMEM50A	11.19817469	10.64502212	11.51008986	11.3403052	10.95234717
TMEM51	7.296277414	7.341038	6.378573141	6.769202101	7.310724131
TMEM52	6.496990475	6.129775061	6.826879909	6.428567879	6.451227182
TMEM55A	9.941224515	9.560980909	9.435122859	9.540446515	9.207219232
TMEM63A	8.975838535	9.245445293	8.294640202	9.015966535	8.983202444
TMEM63B	8.639417101	9.528176495	7.953126222	8.274698687	8.830660505
TMEM66	10.14999886	9.771430818	11.18800276	9.983540212	9.770750222
TMEM70	8.888204949	8.608532485	9.613873859	9.426142444	9.08614898
TMEM71	11.23641801	10.74172297	11.38775416	11.21187068	10.66465811
TMEM8	10.6820228	10.02269473	9.038261515	10.30151978	10.02549055
TMEM86B	9.292435667	10.13958591	9.11510898	8.949261586	9.89348604
TMEM88	9.851320949	8.592161788	8.442699879	8.52575703	8.45451298
TMEM95	7.047046253	6.826879909	7.088019323	7.196285434	7.74007802
TMEM9B	9.739859192	9.766131535	10.37346225	9.848945263	10.02269473
TMOD1	8.703733141	9.136890505	9.092457495	8.865915939	9.968215758
TMPRSS2	6.866597293	6.438891808	7.091067131	6.84402404	7.157253707
TMPRSS9	7.504269545	8.899757485	7.593038414	7.664275131	7.943747505
TMSB10	11.29597305	12.13244857	12.50328361	11.87687449	11.79405389
TMSL3	16.04217659	15.58150095	16.52993947	16.49551516	16.17943014
TMTC1	6.101540747	6.137509616	5.927094424	6.231404212	6.02432797
TNFAIP1	10.00695165	9.865550636	9.940265465	9.285586323	9.550085313
TNFAIP2	14.78379092	13.85099976	14.16629104	14.23242051	13.95955505
TNFAIP6	9.037616626	8.042161798	9.348136535	8.345353354	8.937429899
TNFRSF10C	14.1904887	12.99565877	13.96779242	13.63435472	13.20536123
TNFRSF17	7.042911333	7.017237545	7.012316737	6.689415172	6.681596343
TNFRSF1A	10.26541026	9.566253768	9.857940545	10.06173934	9.807061475
TNFRSF1B	9.999132495	8.738283333	8.335861768	10.11760527	9.046862848
TNFRSF25	12.53197264	12.95303817	12.55983323	12.797444	13.15082863
TNFRSF9	7.129030091	6.555298505	5.859458737	7.131581374	6.838696091
TNFSF10	11.60680648	11.00496707	11.43208235	11.69658017	11.71443529
TNFSF12	11.71895032	11.75215821	11.01376888	11.53050203	11.23116994
TNFSF13	8.527598192	8.090157434	7.824090626	8.494982343	7.748914677
TNFSF13B	11.16888788	10.64128512	12.10135206	11.43208235	11.19817469
TNFSF14	11.61814895	10.53028115	10.23066525	11.15183093	10.88945836
TNFSF4	7.337386909	7.107120283	7.778752535	7.836508475	6.85163704
TNIK	7.321884212	7.367866364	8.158602879	7.621022111	7.850890354
TNIP1	10.05053825	10.33989796	9.324974768	10.11452638	9.916104475

TNIP2	8.533396525	7.961659859	7.210929778	8.552208182	8.099825778
TNNC2	8.735287485	9.092457495	10.05426836	9.387656788	8.502042192
TNNI2	10.00450786	8.990775909	10.15783547	9.273022949	9.36570098
TNNT1	7.823429646	6.772767788	7.200432616	6.455606303	7.279952616
TNP2	7.033625222	6.113783172	6.312781434	6.923412444	6.822666394
TNR	6.579391333	6.158812283	6.458964152	6.858444758	6.660795162
TNRC6B	9.097596465	9.001802606	7.968691808	8.77962597	8.663303283
TNS1	8.720163182	9.842388081	8.709464434	8.872352707	9.467500556
TNXB	8.661956242	9.411394242	8.316081222	8.371674091	8.687969051
TOB1	7.636533838	7.488273475	6.702513515	7.152687384	7.339519202
TOM1	7.478150263	6.74432	6.146364788	7.471219899	7.248451727
TOMM20L	8.846850111	9.584100455	8.100639	9.415217505	9.778131475
TOMM7	12.15130062	12.797444	13.05987966	13.09977851	12.88708268
TOP1	8.900779434	8.861258687	9.171164091	8.488340929	8.923097444
TOP1MT	9.173615515	10.11246778	10.42175216	9.62302801	9.859664869
TOPORS	9.044046343	8.806450121	7.917722121	8.576891556	8.404822414
TOR1A	7.665645141	7.242080394	8.263866778	7.752687697	7.504269545
TOX3	7.18112796	8.190781646	6.908074727	7.152958919	7.530574707
TP53I11	8.059832677	6.979690485	6.620950929	7.876138384	7.421318101
TP53I13	7.926719768	8.042974687	7.30556202	8.105207222	8.158602879
TP53INP1	12.72219022	11.90384839	11.17213305	12.42943771	12.15752365
TPCN2	7.807594768	7.258429848	6.626176061	7.765298909	7.205488778
TPD52L2	8.36093298	7.483257202	7.551170919	7.784888444	7.443845071
TPM1	8.231756293	9.006527424	8.779146061	8.092691677	8.898709273
TPM2	7.590558081	7.854005263	8.037475929	8.151663141	8.359263212
TPP1	7.708254091	7.888681737	7.45998899	8.034046273	7.719466677
TPPP3	6.523815232	7.394370293	6.693002899	7.199434576	6.552305929
TPSG1	17.23317693	17.49517975	15.449005	16.82803465	17.23317693
TPST1	10.2026692	9.285000091	9.46304199	8.888204949	8.637513768
TPT1	14.9847254	14.9727616	16.25437967	15.70163214	15.95256028
TRA2A	7.684965778	7.391655768	6.598445515	7.626110576	7.329870525
TRABD	11.16423752	12.27421104	10.82945836	11.18636156	11.18471248
TRAF5	9.641787333	9.995698596	9.93663897	9.804037697	9.951175646
TRAK2	7.536179778	8.581169111	8.387083515	7.914965525	8.963499596
TRAM1	10.29432836	10.94496034	11.15666056	10.83363242	10.85923156
TRAP1	7.18313201	7.360165061	6.682819434	7.799524616	7.393766606
TRAPPC1	8.055566596	7.886480404	7.755503707	8.128726778	7.855388333
TRAPPC10	9.724814657	9.378583414	9.25368898	9.528176495	9.483552818
TRAPPC2	7.653305051	7.894965162	7.433629313	7.614146707	7.546292778
TRAPPC3	8.393536232	8.207926798	9.29794999	8.620285051	8.352134333
TRAPPC4	10.12610261	10.15783547	9.75438504	9.734703111	9.805520727
TRAT1	7.48289603	8.351407394	9.384699788	8.487847515	8.864955909
TRBV5-4	12.64708972	13.43523914	14.05136004	13.58209631	13.74093847
TREM1	11.26815786	10.48659409	12.18492446	10.74305691	11.5737214
TREML1	7.718083212	8.454954939	7.900011667	8.149788848	7.487940131
TREML2	11.18636156	10.37346225	10.08145981	10.31193963	9.985290333
TREX1	8.424934535	8.76626204	8.791137414	8.817191758	8.840010495
TRGV5	7.423727141	7.210358909	7.686444293	7.234805667	7.241538444
TRIB2	6.804991424	7.12824003	7.507381747	7.529949465	7.26571197
TRIM10	8.519178475	8.966940586	8.824381182	8.510894717	8.64507902
TRIM22	9.556759333	9.556759333	9.605576293	9.217165182	9.087255717
TRIM24	9.468772697	9.155642525	9.308918081	8.877161475	8.666601818
TRIM25	8.76328402	7.843855828	8.65497299	8.389653889	8.135850465
TRIM27	9.610538374	9.139095455	8.639417101	9.380344141	9.055478465
TRIM28	8.783158111	8.937936253	7.760648222	8.964642636	8.741130788
TRIM35	9.755162434	10.69470446	8.267698071	9.328711051	9.858738737
TRIM41	8.463578343	7.945472162	7.683675283	8.579336081	8.164979273
TRIM46	7.083314707	7.453291162	6.640209303	6.638550414	7.004818101

TRIM58	10.21157382	11.58421758	12.00644476	11.16579624	11.62290433
TRIM66	7.935875313	7.700670768	8.347717242	8.160644788	8.868979273
TRIP10	6.669467525	6.636727626	6.609208404	6.62508102	7.058479485
TRIT1	7.796085586	8.559456798	8.119679141	8.257984293	8.07053003
TRPC4AP	8.568768141	8.248986687	7.055055273	8.012346152	7.842359758
TRPM5	7.896600606	7.356281616	8.396125929	8.210994667	8.747892899
TRPS1	7.294861071	7.470329323	7.20183702	7.424927172	7.446413172
TRUB1	7.343153889	7.685352364	6.773706576	7.711523556	7.533296202
TSC1	9.850553616	9.877792838	9.315088131	9.113305192	9.145078374
TSC2	8.574674323	8.127895253	8.305359949	8.214296636	8.166172404
TSC22D1	7.197763283	7.545628697	8.309668384	8.419470808	7.916712838
TSC22D3	10.99623862	9.929312889	11.0473733	10.53842125	9.589694828
TSC22D4	9.834210131	9.220765354	8.27861104	9.500664495	9.301561
TSEN34	13.71423947	12.65472241	13.84455043	13.13522491	12.8652792
TSEN54	8.924201657	9.098746404	8.941359636	9.499247869	9.465071586
TSHB	6.691055667	6.159847303	6.750367323	6.725499141	6.973155374
TSHZ1	6.502086778	6.785085162	6.684320869	6.720541949	6.580572354
TSHZ3	9.073600626	8.578858313	8.425403333	8.300103657	7.822322859
TSPAN1	7.182546172	6.528629101	7.532708636	7.057330242	6.993183869
TSPAN10	11.42371373	12.10135206	10.07580535	11.53677901	11.54470354
TSPAN13	6.860179071	6.514559495	7.08777197	6.889250929	6.744595263
TSPAN16	7.776293606	7.326350616	6.637264	7.413101485	7.174922929
TSPAN2	7.404985061	7.453021566	7.667962596	7.433328091	6.787032949
TSPAN3	7.911309091	8.043387838	8.583542788	8.176360121	8.386133566
TSPAN33	8.204025576	7.610574101	7.207500556	7.848096879	7.16240796
TSPAN4	7.397737788	8.020096283	7.476255091	7.478803566	7.557268586
TSPAN5	10.62280049	11.94745099	11.61814895	11.68243664	11.87687449
TSPO	11.07158908	10.89224764	10.56548536	11.12613667	10.86691694
TSPYL2	8.048636061	8.997224192	7.898595626	7.89560499	8.249809909
TSPYL4	8.167813576	7.923423949	8.10683704	7.467207283	7.719108384
TSPYL5	6.651970061	6.463509232	5.956194919	6.018680697	6.171005242
TSTA3	8.678658293	9.826793596	7.638981707	8.979921919	9.954681949
TTBK1	8.69919198	9.826032111	8.890373556	9.034403333	9.688441414
TTC16	6.994769535	7.309054838	7.266003808	7.556302505	7.227882465
TTC21A	8.114219303	8.166983455	8.514037303	7.979863071	8.095330333
TTC22	6.839681778	6.847044404	6.912541192	7.317897131	7.966402293
TTC25	8.851571051	8.848410616	9.642500081	8.686126354	9.038261515
TTC32	8.962353222	8.617840212	9.11393401	9.432003333	9.214694788
TTC38	8.190781646	7.689572788	7.33858799	8.778117444	7.720541949
TTC5	6.425312475	6.700124848	7.06891401	6.783830859	6.613716152
TTC9B	9.049171303	9.846508495	8.25927404	9.046362323	9.616741535
TTLL3	9.710054737	9.444645091	9.195011616	9.493894545	9.635582141
TTMA	9.180906778	6.443018677	6.778470374	10.27432009	6.604509566
TTRAP	12.89513437	11.46145043	12.30789527	12.14789109	12.47761486
TTTY12	6.505445727	6.32497801	7.083314707	7.102116364	7.581982414
TTYH3	7.252886707	7.244714616	7.300653717	7.840025778	7.638274606
TUBA1A	10.57282784	9.481000434	10.14131493	10.69470446	9.987874111
TUBA3D	9.910074283	10.56802411	8.981975535	9.992117576	10.12041459
TUBA4A	12.92081703	11.93178106	12.27773509	12.97977545	12.28693608
TUBA8	10.56802411	10.98492733	11.05959848	11.01376888	10.3986043
TUBB1	8.773471869	8.730448515	9.444645091	9.505258828	8.812013889
TUBB2A	10.95093974	11.49839615	8.912452162	11.92156179	12.41931404
TUBB3	7.905781333	8.023099172	7.116617232	7.919201576	7.950819283
TUBB6	9.560980909	10.10365156	9.339095222	9.869466131	10.18563382
TUBGCP2	8.911109919	8.69723302	8.023099172	8.940148162	8.797743222
TUBGCP6	8.508189222	8.59942799	7.410364737	8.948168879	8.754900343
TUFM	10.85361094	11.51260306	10.40178901	11.36423723	10.9889639
TUFT1	8.162329636	7.576363505	8.136235949	7.325469747	7.637262101

TULP4	8.215875455	8.280707687	7.732206152	8.170586434	8.208296687
TWF2	10.63752253	10.13276962	9.053706202	10.58689262	10.01703805
TXN	12.89886333	11.95514832	11.85889751	12.26775966	12.2031413
TXN2	8.698751323	8.809951364	8.707057788	9.321347081	9.241421747
TXNDC1	10.82664672	11.00620373	10.65365596	10.57159251	10.87379507
TXNDC12	8.33024502	7.863195747	8.943861384	8.278157657	8.011239071
TXNDC13	10.48782122	9.634173323	10.16642595	9.887613414	9.580097263
TXNDC17	11.40306525	11.05796986	10.83642757	10.70643744	11.02105469
TXNDC3	9.671912455	8.784649081	9.020337111	8.528512253	8.295436051
TXNDC5	8.683580283	8.880364303	8.856895172	8.517288667	8.616914869
TXNIP	16.52993947	15.34617167	15.52328152	16.12384164	15.72204116
TXNRD1	7.170232747	6.86313897	6.691325636	6.561721071	6.528194515
TYK2	10.09339803	9.496413788	8.629474212	10.02549055	9.280885222
TYMS	6.18856802	6.418448242	6.888392616	6.678316576	6.302130162
TYROBP	16.2036826	15.31193978	16.12384164	15.99508933	15.48449671
U2AF1	10.99469779	10.40592026	11.45002291	11.05191968	11.06877356
UAP1L1	8.185177828	7.69185196	7.789255535	7.942409707	8.405727848
UBA1	8.196571586	7.724673141	7.147872253	8.279462	8.03490996
UBA2	7.840343939	8.460488909	8.881310737	8.075284384	8.098302202
UBA3	8.124212202	7.742436303	8.440878707	8.026271202	7.937009
UBA7	8.567346869	8.680072242	7.445432899	9.019731374	8.816703535
UBAP1	9.538263677	9.792275424	9.257324	9.696433626	10.12314196
UBASH3A	7.275223848	7.686775222	7.622037172	7.754407434	7.649291899
UBB	16.96059838	16.17943014	17.49517975	17.58093558	17.49517975
UBD	7.195783737	6.049314636	5.794122121	7.188870657	6.115636667
UBE2C	8.621272737	7.706168626	8.326884343	8.141366242	8.102124455
UBE2D1	9.613873859	9.398445515	9.32739897	9.211184727	9.139095455
UBE2D3	11.83330316	11.76336308	12.47441306	11.60680648	11.72807534
UBE2D3P	6.890508141	6.995550687	7.12824003	6.711662182	7.054238101
UBE2F	10.78014639	10.96311364	11.44319437	10.4700758	11.14707085
UBE2H	8.826846768	9.005975162	9.455015404	8.77298297	9.185497838
UBE2I	11.19114259	10.59541023	11.18096106	10.70902317	10.4429644
UBE2J1	7.165538758	6.841133828	7.757532545	6.777485293	6.791503909
UBE2L6	9.316319051	8.921011566	8.925792808	9.095162899	9.119831202
UBE2M	9.146178758	9.054276	7.780448354	8.94874	8.686679798
UBE2MP1	8.634788929	8.461795505	7.49893601	8.494130061	8.197805768
UBE2O	8.996148737	9.508442768	8.63611798	9.214111545	9.692793657
UBL4A	11.77552743	12.89113428	11.43565115	11.1502402	12.27421104
UBL4B	7.853350485	7.522869182	7.347141657	7.387388131	8.148308414
UBL7	8.053207788	8.721523222	7.674109172	8.179951646	8.270601414
UBN1	11.24646499	10.35591393	10.43122177	11.15808934	10.62647628
UBP1	9.129114848	9.557589465	9.503271071	9.541041677	9.848227263
UBR5	7.180559343	6.919392394	7.462771485	7.135222657	7.200755828
UBTD1	8.08537204	7.514821949	7.005900293	7.716479172	7.240274404
UBXN2B	8.955359253	8.205892515	8.31049196	8.546982556	8.391869283
UBXN6	13.41514643	14.63205333	13.13007894	13.93694444	14.15863941
UBXN8	7.762120596	8.138519566	7.413101485	7.396513838	7.533663232
UCK2	6.503631525	6.496103798	6.337791596	6.592026141	6.506308818
UCKL1	9.911926424	10.81142627	10.16743096	9.852239606	10.58579258
UCN2	13.85099976	15.27762024	11.51260306	12.56726296	13.88591407
UCP2	9.172421828	9.763138212	8.044608626	9.007843273	9.285000091
UGT2A1	8.961327374	9.221852535	8.215875455	8.228946717	8.927565404
UGT2B15	7.93027604	7.143440162	6.618511313	7.170797768	7.705454838
UHRF1	7.797125465	7.44169298	7.90403798	7.638629778	7.28582396
UHRF1BP1L	7.684641212	6.949608808	7.780116303	7.554402606	7.51340999
ULK3	9.038849273	9.048620525	8.744562374	9.310775172	9.451851384
UMPS	7.511268929	7.870356606	8.301657889	8.197805768	8.094989869
UNC119	9.35796903	8.672881101	7.480348535	9.182204323	8.459227657

UNC13D	8.863920758	8.967408879	7.969046636	8.58807297	8.748883111
UNC50	9.792275424	9.609756293	10.56084718	9.714566535	9.534883657
UNC84B	8.562227051	7.850180343	7.51340999	9.186711646	7.883157929
UNC93B1	8.302900242	8.29927897	7.843485626	8.144161414	8.264240727
UNK	9.41003803	9.435722162	9.18155302	9.438799404	9.477759242
UNQ1945	9.077100232	9.450568111	8.399553939	7.495424616	8.40702104
UNQ6228	9.072403576	9.220211768	9.520598525	9.258612929	9.543825273
UNQ6487	6.413868758	6.339459364	6.510509061	5.890469384	6.640442667
UNQ6493	8.090157434	7.493565131	8.072532475	7.423140192	7.464704768
UNQ9165	6.529097838	6.629471747	6.836753071	7.079662576	6.679485202
UPF1	12.90359001	11.97196754	12.11562205	12.14507673	12.31419772
UPF2	7.472789818	7.228169333	7.739667424	7.477877475	7.668279394
UPF3A	7.490707535	7.840025778	8.760676242	7.828584192	7.78118699
UPK3B	8.884521697	8.410908071	8.915814212	8.93587504	9.585510535
UQCRB	10.1838089	10.70425349	10.09161001	10.64738653	10.93935201
UQCRC1	9.372705202	9.587719636	8.890936242	9.903372323	9.326238727
UQCRC2	10.98037262	11.13400706	10.67572435	11.04593523	11.37847359
UQCRH	11.63629658	12.01191659	12.64265677	12.28693608	12.18792926
URG4	7.202978657	7.543741273	6.499057505	7.547650101	7.462172081
USP1	10.14044783	10.13479405	10.36781996	9.851320949	10.19418334
USP16	10.00886723	10.11849919	10.27432009	10.2187154	10.34830463
USP17	8.143040162	8.053207788	7.326350616	7.907197636	8.034450929
USP18	6.746121293	6.816006121	7.177177071	6.796334212	6.72401001
USP20	9.903372323	10.16642595	8.998420707	10.17122401	9.868690293
USP32	9.03221704	8.800782677	9.584100455	8.853585596	8.975268424
USP53	6.47989397	6.706413566	7.049242879	6.748847646	6.66391404
UTF1	8.559011545	9.773700283	8.473802646	8.489178071	9.021393263
UTP18	7.820222687	8.181196919	9.214111545	8.206288727	8.400464071
UTP3	9.441234879	9.70574997	10.30470526	9.905108616	10.12224713
UTP6	9.57181996	9.509876273	10.38208186	9.596773313	9.602761333
UTS2	6.047425707	6.460074192	6.176006556	6.026785687	6.030805626
UTS2R	15.41210544	16.12384164	12.97555423	14.65397593	15.08134613
UTX	9.378583414	9.09013302	9.479751879	8.74840203	9.228437333
UXS1	8.512201869	8.29635502	8.621689657	7.870669919	8.585303495
VAMP2	8.338686465	8.55725397	7.55847901	7.85299496	7.835124576
VAMP5	8.760291222	8.929972798	8.798247798	8.934777717	8.200539131
VAMP8	11.96362869	12.51766186	12.87819387	13.03496614	12.56726296
VARS	7.158304313	7.413750677	6.883542434	7.850890354	7.759566061
VASH1	8.325717687	8.946596606	8.151247717	7.786298434	8.331458747
VASP	14.02572128	13.12023156	12.23769992	13.16151113	12.72972704
VAV1	12.01191659	11.02851208	10.91641235	11.80342824	10.9991534
VCAN	10.46342714	10.73014715	11.74935785	10.91514001	10.80389925
VCX3A	6.403487758	6.083230091	6.262151687	6.232553091	5.984918404
VDAC2	10.38208186	10.28710845	10.94353341	10.45375986	10.57649171
VDR	9.956562586	9.432003333	10.07479617	10.08325108	9.948421707
VENTX	7.12986801	7.096055535	6.993764091	7.042643192	6.842776727
VGF	7.225864141	7.218710525	7.355080212	7.074987333	7.276635293
VIM	14.40789433	13.82538175	14.7172184	14.78379092	14.4185289
VMO1	6.60520404	6.971547919	7.187484444	6.542753323	6.407444293
VNN1	7.812327374	6.678558889	7.383711768	7.193299919	7.222960121
VNN2	15.52328152	14.20688311	14.9591327	15.95256028	15.03840752
VNN3	8.504327141	7.83046296	7.85268101	7.987188889	7.202436384
VPREB1	7.043955899	6.860671293	6.702978071	6.572131525	7.342268626
VPREB3	7.748187364	7.793372141	7.854685616	7.608041616	7.812327374
VPS11	8.170586434	8.152443121	7.179153636	8.311754818	7.909763434
VPS16	9.810255869	9.227886646	8.666113404	9.778131475	9.417594202
VPS18	10.25127201	10.15379546	9.756840071	10.42175216	10.07670007
VPS37C	10.80499832	10.76737368	10.03026493	10.26832521	10.14631732

VPS41	6.86860504	7.013931768	7.782796596	7.267888182	7.088518818
VRK2	8.305787747	8.286128505	8.625920364	8.079553929	8.246913576
VRK3	7.950449798	7.715032394	7.487940131	8.164575929	7.787525596
VSIG4	6.889002121	6.684320869	6.740749081	6.445432434	6.729687141
VTI1B	10.20164867	10.57282784	11.22456459	10.31480319	11.13085117
VWA1	7.623059667	7.670275758	7.498029465	7.531770848	7.567690545
VWA5B2	7.917403434	8.721926778	8.389653889	7.997183333	8.696739152
VWCE	12.28105924	13.31405593	13.77352346	12.84627851	13.14093405
VWF	7.972411364	7.92237903	7.766738848	7.653651778	7.55499601
WARS	10.43998577	9.966143818	10.40278823	9.825199323	9.918671212
WAS	9.35728196	8.883993253	8.214678465	8.846355515	8.876650909
WASF2	10.4429644	10.88681216	9.580783667	10.36589334	10.59180142
WASF3	6.166137596	6.353889641	7.015941667	6.239584586	6.230489586
WASH1	11.0859227	10.69682519	10.27139209	11.18800276	10.80499832
WASH5P	7.659186848	7.072036626	7.589252838	7.078835444	7.269324172
WBP2	8.261781515	8.101780545	7.281402384	8.150448232	8.079179394
WBSCR17	6.682081222	6.301539283	6.692548323	6.57287004	6.655235768
WDFY3	8.271405859	7.635267465	7.442598384	7.59498599	7.381652111
WDR23	9.983540212	10.25906505	10.08599138	10.43564423	10.38001468
WDR37	8.978723121	8.878201818	9.814415596	9.037153444	8.901935111
WDR40A	10.78551178	12.08680505	12.36473556	11.2518391	12.38063147
WDR41	7.845601525	7.692217293	7.629016404	7.419275485	7.472500828
WDR45	10.29000276	10.77103399	10.3380268	10.62536143	11.0940021
WDR45L	10.33146686	10.22164118	10.94071069	10.18000397	10.14895288
WDR51B	8.20265299	8.376704939	8.078389566	8.086153313	8.946596606
WDR6	10.67081044	11.00053539	11.19293398	11.36065784	11.17750487
WDR61	7.383097848	7.684641212	8.943348051	7.713612677	7.785596848
WDR70	7.888681737	8.137069859	9.152615889	8.464081152	8.679578434
WDR91	9.279768394	8.930963202	9.484209455	8.901935111	9.058895657
WDR93	9.023639636	8.098302202	9.224923687	8.148308414	8.053619525
WDTC1	10.10093339	10.7814083	8.759757687	8.966940586	9.710962273
WFDC10B	7.830796253	8.468064667	7.55178802	7.755503707	7.964914919
WFDC3	7.88791003	8.498167798	7.869396606	7.821971636	7.772177253
WIPF1	10.42175216	10.11941657	9.519820636	10.49592664	10.09254304
WIPI1	11.41981305	10.88166557	10.79145221	10.80644997	10.63391466
WIZ	9.624391323	9.574050242	9.548028869	9.135618929	9.120933788
WNK1	9.044608384	9.388912091	9.230202616	9.09824599	9.829119768
WNT6	11.46724679	11.69891546	10.51291939	10.83100199	11.06433979
WRB	7.790242404	7.746135697	7.867312374	7.938205586	7.139668182
WSB1	11.33657706	10.90192126	11.76101506	11.13551505	11.31267384
WSB2	7.953486636	8.221403505	7.454260455	8.040575909	7.951199808
XAF1	8.462637525	8.378515131	9.280305798	8.591651626	8.749346626
XCL1	6.905589111	7.852302727	8.29927897	7.095534354	8.259704283
XCL2	6.437041	7.103541778	8.14677703	6.846509828	7.40744601
XCR1	7.931306778	7.283275424	7.747112485	7.589604303	7.880750535
XIST	5.754251323	5.76546399	12.48458958	5.765905929	12.89113428
XK	9.756054152	11.58005536	10.4687107	9.263851586	11.3403052
XKR3	5.872561636	6.198948818	5.833420222	5.856640909	6.181905172
XPO6	11.98440811	11.06433979	10.19236777	11.52238659	11.03737422
XRN2	8.199315667	7.770465818	9.129114848	7.960941889	8.192266657
YBX1	12.04432	12.39058663	13.32021166	12.51766186	12.48458958
YEATS4	7.323124384	7.885378	8.11773804	7.491688212	7.759936788
YOD1	8.266388465	8.915814212	8.494982343	8.336651323	9.596088263
YPEL3	10.60383217	10.65246846	9.436357141	10.71279176	10.44195016
YTHDF3	6.772514131	6.531281859	6.715644404	6.490215273	6.387713778
YWHAH	8.742149222	9.007167758	9.123926859	9.497084828	9.069043081
YWHAZ	10.24612484	10.04160563	9.085558838	10.18666124	10.0672481
ZAP70	10.77387872	11.28731295	11.17750487	11.39185194	10.8778129

ZBED1	8.681579566	8.124599212	9.140392293	9.254253192	8.511980308
ZBP1	8.726024485	8.265130232	8.208296687	8.966282283	8.865407737
ZBTB11	8.547862646	8.322806515	8.896578162	8.806957455	8.882882525
ZBTB16	8.656360697	7.773582384	7.36694602	8.171788646	8.134790222
ZBTB17	9.995698596	9.876096838	8.720627081	10.14432724	9.934615404
ZBTB25	6.215679889	6.484888434	6.610658212	6.233403424	6.334611455
ZBTB3	8.216297535	8.687969051	8.284876758	8.044608626	8.162707626
ZBTB32	6.606438778	6.904353566	6.559271747	6.785316677	6.686049697
ZBTB48	8.422643323	8.104866828	7.287262414	8.519178475	8.283707475
ZBTB7A	8.969483	9.23383296	8.273878929	9.366312899	9.468772697
ZBTB7C	6.918565768	6.736375424	7.242650333	6.872867768	6.838224475
ZC3H12A	9.267383556	8.979298788	9.370832535	9.116279576	8.955880859
ZC3H18	8.311348051	8.233838889	6.710284202	8.480536717	7.972012263
ZC3H4	8.976374343	8.909404788	8.268510495	8.744562374	8.598479657
ZCCHC11	8.692466525	8.499836475	9.665316566	8.502042192	8.69723302
ZCCHC8	9.302735434	9.334658071	9.627745303	9.118059475	9.171164091
ZDHHC11	8.10683704	8.453639616	7.183932212	8.630877737	7.228759818
ZDHHC18	10.06356315	8.768300525	7.743818828	9.609027141	8.678158051
ZDHHC2	7.570191354	7.719108384	7.837507091	7.792215626	8.226840717
ZDHHC5	9.761765848	10.27905981	9.463745455	9.470173818	9.911926424
ZDHHC7	10.43564423	10.12697515	10.47242114	10.33146686	10.18291229
ZDHHC8	11.41605585	11.88649013	11.34202326	11.78265022	11.86401838
ZEB2	9.509226798	9.819688798	8.739635162	9.599465606	9.432003333
ZER1	9.058895657	9.833438364	8.721054424	9.212349869	9.484840202
ZFAT	9.421700798	9.640429374	9.281446566	8.735287485	9.003357768
ZFP36	12.12151457	11.36423723	11.308834	11.75908761	11.20345844
ZFP36L2	13.98831971	13.5360606	13.26276274	13.28559105	13.39037726
ZFP62	9.577319545	9.923658606	10.43332542	9.439397061	9.397189192
ZFP91	7.77977198	7.800624061	7.991889949	7.868335495	7.868007081
ZFPM1	13.81266894	14.85646221	12.47761486	12.88708268	13.57574016
ZFYVE21	9.59387599	9.377908	10.62142174	9.552821717	9.566253768
ZFYVE28	6.996769242	7.101573333	7.023397576	7.096649162	6.984268657
ZMAT2	10.72384747	11.39185194	10.85225864	11.51260306	11.67849095
ZMYM2	8.801904485	8.556855828	8.405355091	8.420871687	8.264694495
ZMYM4	7.238429162	7.435155859	7.051418091	7.220780818	7.453623293
ZNF117	7.504556444	7.230593889	7.49864798	7.448962859	7.584620515
ZNF133	8.888766747	9.35067002	9.08614898	9.07526898	9.25368898
ZNF14	8.144584758	7.907197636	7.956286283	8.107705111	8.30460302
ZNF182	9.459664354	9.08959502	9.541747455	9.349388778	9.142791657
ZNF185	8.18280501	8.689422596	7.742830394	8.223899788	7.774617596
ZNF200	9.07526898	8.703733141	9.55462702	9.012031232	9.165150051
ZNF205	9.224452333	11.20840189	7.794025323	8.050105303	8.813595202
ZNF213	9.437491071	9.346872071	8.648914657	8.720163182	9.348735909
ZNF217	11.41071085	10.98749259	10.24414187	10.99469779	10.86447048
ZNF230	7.278747747	6.521234222	7.837840162	7.203788636	6.845030273
ZNF234	7.130479657	7.439506505	7.085030667	7.166060222	7.21289504
ZNF266	8.080354404	7.955993172	8.861258687	9.005433152	8.629474212
ZNF267	9.840616414	9.266188263	9.968215758	9.380979222	9.439917707
ZNF268	8.18481599	8.543234566	8.716864899	8.339115859	8.315227333
ZNF274	7.090449141	6.588475747	7.809644101	7.090758091	6.964897566
ZNF281	9.394567081	8.39227803	9.214694788	9.282486081	8.928157121
ZNF319	8.553595747	7.823737283	6.974671657	7.729749768	7.768402071
ZNF330	7.418946535	7.465356566	8.714332636	7.837507091	7.729067364
ZNF333	7.529686949	7.825104404	7.701686677	7.691535778	7.820222687
ZNF335	8.551311374	8.320357909	7.301506111	8.321152475	8.263866778
ZNF354A	7.93888704	6.535959323	6.330092323	6.601482788	6.958952465
ZNF385A	10.03299879	10.3986043	9.767642525	9.996420616	10.38329808
ZNF418	11.66189432	11.72592113	11.02403513	10.14999886	10.49241362

ZNF429	8.179120646	7.932453717	9.073600626	8.398231626	9.067241768
ZNF438	10.80644997	9.824540899	10.17702971	10.14631732	10.26632574
ZNF467	9.963504081	10.10736711	8.977005354	9.678297182	9.792275424
ZNF480	7.596246919	8.166602566	8.443547162	7.676615465	8.174871818
ZNF488	7.470886646	6.499941	7.135537212	7.004539727	7.362377768
ZNF502	6.921167192	7.126269081	7.272016424	7.310724131	7.464355384
ZNF509	6.436812525	6.204170475	5.750374955	6.089542354	5.983964121
ZNF513	7.924172566	7.774939586	6.782428768	7.748548515	7.606043535
ZNF529	6.584387121	6.520809899	7.025264747	6.894670869	6.814222283
ZNF550	8.006153172	8.477856384	8.336257222	7.98506802	7.74678301
ZNF573	9.259301556	9.744663444	9.387059283	9.853774707	10.11849919
ZNF574	8.674900657	9.039989364	9.026636737	8.886096818	9.517771202
ZNF575	9.733230051	10.80644997	8.712915394	9.534883657	9.890949131
ZNF579	15.97330865	16.61084374	14.08841453	15.63778652	15.92931651
ZNF580	8.339546939	8.579336081	8.495447152	8.322806515	8.468418253
ZNF641	8.586731747	8.31049196	8.265130232	7.795116566	8.056742909
ZNF652	9.384699788	9.602761333	10.6820228	9.522660222	9.655770101
ZNF653	6.616628394	7.335252788	6.461471737	7.063222061	7.026621727
ZNF668	7.535866141	8.724556485	6.854191545	7.561964414	7.759260374
ZNF675	8.452737606	8.257253131	8.708995576	8.479185606	8.703161646
ZNF683	7.279669313	7.943134	7.679285535	8.558108202	9.488337717
ZNF687	8.220625586	7.643628758	6.235727697	7.139090576	6.34228097
ZNF696	6.578936343	6.375508101	6.839464636	7.106848111	7.115757364
ZNF71	7.395882727	7.496684051	7.636934919	7.252580253	7.329290475
ZNF710	10.58689262	10.07388225	9.985290333	9.987077505	10.02741959
ZNF713	11.37131374	11.56725755	11.53473316	11.73972102	11.2608846
ZNF721	9.368875727	9.361074667	8.936938576	9.454390232	9.424273283
ZNF728	6.066342414	5.944238404	6.173797535	6.159847303	6.476196333
ZNF730	6.273265505	6.489542818	6.730193313	6.157827909	6.415480919
ZNF746	12.4956701	11.50814936	11.67849095	11.77053658	11.43938222
ZNF771	9.35188598	10.53842125	8.246087697	9.208368677	9.155079848
ZNF775	10.20532801	10.51291939	9.581418808	9.957374697	9.963504081
ZNF780B	7.531770848	7.573788949	8.854095293	8.139691131	7.929905606
ZNF784	7.97753898	8.216297535	8.073294879	8.755421576	8.387939343
ZNF787	9.256085253	8.770297859	7.808339242	9.04056096	8.354570556
ZNF83	7.196057152	7.364550071	7.663954061	7.140588444	6.901187545
ZNF830	8.427236737	8.063898495	9.282486081	8.085049192	8.057063626
ZNF831	9.161459798	9.529469889	9.36439596	9.155642525	9.335272182
ZNF843	9.239472869	11.62059475	8.181574081	8.637513768	9.172421828
ZNF85	8.035675313	7.243824414	8.272620253	7.644626202	7.859293
ZNF91	8.069395848	8.289599333	8.464081152	8.44446797	8.707515232
ZNF92	8.490053091	8.489580414	8.838020616	8.923576949	8.939630657
ZNHIT3	10.2752886	10.82153564	11.49636188	10.97767761	10.99763472
ZRSR2	9.802496737	9.212349869	10.03611568	9.773700283	9.746145949
ZSCAN10	13.32669092	15.449005	11.40725584	13.76702458	14.21449649
ZSWIM6	10.03796113	9.669114061	9.341818545	9.591816061	9.235747242
ZSWIM7	7.530291586	7.921614596	8.255317818	7.860366808	7.302736424
ZYX	11.14707085	10.17122401	9.268661657	10.39521192	10.43564423
hCG_1641703	11.45176908	12.13867659	11.41238356	11.94745099	12.10694646
hCG_1732469	6.550314424	6.065510788	7.033625222	6.168008697	6.190947343
hCG_1771830	7.182015707	6.366338828	7.109111172	6.638813101	6.556248091
hCG_1776259	9.689783586	9.045755677	9.439917707	9.689783586	9.039989364
hCG_1783494	6.946711626	6.33568397	6.728447091	6.551390626	6.19283997
hCG_1811732	8.379510323	7.601304687	7.825104404	7.25994097	7.965660091
hCG_18290	13.48401144	14.16629104	13.68884286	13.60979651	13.85099976
hCG_19809	10.49830212	11.18291631	11.84736958	11.50425191	11.77053658
hCG_1984468	11.71042621	12.2031413	13.18348162	12.50328361	12.61449239
hCG_2009921	7.652274172	8.566871343	7.483257202	7.286424495	7.448962859

hCG_20426	9.634846707	10.72384747	9.499917182	9.67665103	10.11349335
hCG_2045899	6.603384646	7.28582396	6.528858343	6.648393616	6.400763333
hCG_21078	14.19920898	14.1904887	14.80749806	14.37933382	14.39736901
tcag7.1015	9.321347081	9.179226364	9.852239606	9.886762101	9.610538374
tcag7.1023	9.627013505	11.42787377	8.296772869	8.722452253	9.491725303
tcag7.23	10.17789322	11.10460602	11.4413808	10.43998577	10.70181173
tcag7.873	7.344278889	7.728706424	7.91465098	7.580594545	7.965251131
tcag7.907	12.19996238	11.14707085	11.15808934	11.2786408	11.26990549

GSM518895	GSM518897	GSM518899	GSM518901	GSM518903	GSM518905
6.915399929	6.418014596	6.566529242	6.939171505	7.028039657	6.911201424
9.609027141	9.666877556	8.513601889	9.093526566	8.304980606	9.444645091
7.576996152	6.826151202	6.555968283	6.599129636	6.541618313	7.998937818
8.113343889	8.660166475	8.844262616	8.637513768	8.593558293	7.714004283
7.671593667	7.465665798	6.984268657	6.950902859	6.875911141	7.545628697
7.740855141	7.798826303	7.97753898	7.785214909	7.995673758	7.816902374
8.749346626	8.731576192	8.979298788	8.859715222	8.978204141	8.551751717
7.724369343	7.499887141	7.384295283	7.807908545	7.952774121	7.803554182
10.17030341	10.89912578	11.01209098	11.06261544	10.65500785	9.061091758
8.648914657	7.961659859	7.576687677	7.603719626	7.442271202	8.773471869
8.522156747	8.026630869	7.383097848	7.695912303	6.94985404	8.491490828
7.808339242	8.364710758	7.82717702	7.295980475	7.891531071	8.157153081
8.119288848	7.784529071	8.124212202	7.940978939	7.801818071	7.971654859
8.219832939	8.166602566	8.178694051	8.328154263	8.265965545	8.443175586
8.22479296	8.769824364	8.875206121	8.566415323	8.664254273	8.038729465
8.384333949	8.735287485	8.891888576	8.858692343	8.762742172	7.849137545
7.08136002	7.218710525	7.31991999	7.579628838	7.217577212	7.473428394
7.772177253	7.792995667	6.599129636	7.007603253	7.113452899	8.312729141
8.111336758	7.505451222	6.860671293	7.087210828	7.097914788	8.125818909
8.368780919	7.565422434	8.354182626	8.725113212	8.228946717	8.628208788
10.63014735	10.14999886	10.50166798	10.85923156	10.51886057	10.81142627
11.51867768	11.14057221	10.156858	9.902579253	9.953782444	11.28565422
9.744663444	9.46096901	9.615324495	9.870931818	9.770750222	9.664626495
9.319510202	9.528176495	9.387656788	9.263233333	9.097596465	8.719245818
11.60479972	11.51008986	11.2518391	10.7814083	11.87175588	11.58621592
11.77053658	12.30167305	12.67700968	12.51424883	12.26162026	10.76983035
9.099925818	8.744081071	8.843670869	9.188424626	9.021393263	9.106199707
9.068490586	9.268661657	8.336651323	8.751946515	8.832291949	8.703733141
8.993505475	8.843670869	8.035675313	8.582147707	8.333227545	8.567801737
6.97260104	6.286294667	6.063625505	6.147209687	6.138235202	7.130479657
7.239990273	7.940978939	7.828584192	8.867411152	7.809982667	6.835608667
9.381544081	9.591816061	7.294038515	8.721523222	8.391460737	9.832711535
7.606990414	7.208321485	6.530842141	6.782883697	6.64590104	7.535195111
7.545042545	7.897352323	8.063898495	7.890057202	8.017104828	7.623386535
8.042161798	7.940608939	7.880750535	7.93888704	8.190353414	8.336651323
6.834332747	7.864535646	6.369870263	6.95944596	6.742219182	6.961028939
9.828404283	10.90466263	10.8405808	10.02161965	10.72633412	9.905938879
8.17219503	7.521266808	7.847720859	8.140977576	8.074904687	7.999327475
10.09994749	10.50543333	10.59541023	10.28089537	11.05356995	10.68687892
12.21312889	11.70348036	11.53239215	12.23505088	11.10035391	12.46466592
8.199709495	7.77328201	8.19458801	8.371229848	8.065602475	7.97128602
13.5360606	14.26676228	14.00297944	14.1904887	13.98081649	12.99565877
8.404822414	8.737323475	8.648450303	8.728878424	8.621689657	7.841729333
8.135526051	8.679110091	8.229339455	8.366003535	8.345353354	8.364710758
6.721549354	6.619744263	6.612514242	6.615401818	6.506079909	6.42621898
8.164230707	7.065949717	6.840661414	6.974196465	6.989960667	7.959488687
10.65127749	11.46145043	11.88909817	11.35278885	11.34202326	9.87177298
6.007564444	6.467159949	6.612316919	6.422838717	6.560443758	5.891211081
9.159196141	9.134591697	8.442251212	8.64752602	8.373157505	9.12042397
9.772250768	9.526200354	8.134790222	8.414679525	8.442251212	10.23351878
9.271123182	9.07419996	9.126663535	9.252443646	9.344138121	9.250125475
8.002717273	8.60413102	7.248166182	8.085049192	8.076845152	8.669446475
8.728449242	8.84786598	7.496050374	7.812003626	7.743818828	7.517436172
9.170513566	9.033915556	8.989142232	9.393934131	9.481668929	9.433258586
10.94786936	10.56084718	10.81034167	10.59809845	10.59418919	11.14866374
7.303562909	7.072869879	7.165538758	7.359873626	7.206091434	7.222960121
9.107388182	9.244930505	8.877626323	9.231946182	8.838951081	9.256085253

8.927565404	8.236633323	7.406158394	7.639990515	7.542783869	9.008373061
15.449005	15.06622261	15.86132141	15.74256723	15.38120792	15.31193978
6.102018232	6.705661929	7.005636414	7.016958768	6.790317606	6.008616515
9.150235333	9.348136535	9.199527535	9.285586323	9.08959502	8.686679798
7.473129747	7.078018687	6.548684566	6.664432283	6.573293212	7.697940283
6.96917198	7.199742475	7.188870657	7.259306869	6.857502071	6.335022778
9.513795939	10.13276962	10.17440526	9.729555121	9.540446515	9.827596687
5.851394535	5.79008703	5.848593192	6.310083061	5.894489576	6.574452879
8.099825778	8.750916455	8.48873997	8.613130333	8.628208788	7.400314747
10.20964764	9.815129384	9.127251283	9.302735434	9.220765354	10.27139209
7.46337499	6.259132677	6.364101768	6.29783297	6.27919301	7.896600606
10.77387872	10.7814083	10.8605042	10.96460614	11.0035084	10.66229262
7.119081899	7.400864778	7.414724596	7.639662323	7.509707515	7.819446808
8.120448121	8.030983081	7.935875313	7.983184263	8.157901343	8.424934535
12.95303817	12.75784116	12.05489479	12.63500423	12.29275278	12.82153585
8.036816444	7.991889949	7.910120242	7.860050667	8.217270354	8.136641818
11.60888892	10.68338253	10.75737971	10.81664077	10.62647628	12.15130062
8.379056919	6.555968283	6.608482505	6.501886182	6.514326051	8.524356212
9.227886646	9.732604394	8.265565879	8.340252101	7.966742939	7.286722061
9.848945263	9.759931222	10.31013098	10.5041569	9.422377343	10.19236777
8.502042192	8.385691051	7.106302455	7.480029465	7.286993737	8.800782677
8.653906556	8.487847515	8.69919198	8.589388657	8.598980253	8.273878929
9.140392293	9.888393747	9.31393701	9.642500081	9.512482222	9.372025394
10.29324401	10.52135963	10.70643744	10.46766096	10.48897537	10.13958591
7.029897788	6.966976535	6.66391404	6.803567182	6.858224505	6.864210101
7.811684818	7.769805091	7.651926253	7.724673141	7.866966485	7.860366808
9.408173051	9.619644949	9.050265121	9.431415586	9.116804101	9.325601172
7.087495071	6.44166202	6.482522545	6.407674848	6.411283202	6.207878939
7.667649192	7.694916828	7.902575141	7.826504869	7.388278253	7.242384899
7.644277273	7.552833101	6.877410515	7.144617717	7.102414636	7.259665485
8.267698071	8.028563859	8.57971698	8.25570701	8.864430475	8.323598384
8.545617303	8.703161646	8.708030737	8.614861051	8.737323475	9.152615889
10.89604789	10.82034332	10.60635433	10.91903182	11.18096106	11.4413808
7.279952616	6.364932	6.344827192	6.396791283	6.300565949	7.279952616
14.6970922	14.85646221	15.08134613	14.7172184	14.60195877	14.4427031
9.395177889	9.549314313	9.347406172	9.428190232	9.574050242	9.719830172
8.708030737	8.596127869	7.843077242	8.366462404	8.112161727	8.092218303
8.68840998	8.796283929	8.481411768	8.800782677	8.461315273	8.924201657
8.552719747	9.478383576	8.988033424	9.259301556	7.999737535	8.985325394
9.963923854	9.847335909	9.250770152	9.53690398	9.390834152	9.789981192
9.383480283	9.360502949	9.21905796	9.271123182	9.550769788	9.423026242
8.518658667	8.637033606	8.863375313	8.405355091	9.139095455	8.457489293
8.162707626	7.988251818	8.125376242	7.904381697	8.572701232	8.03179299
8.759332636	9.211184727	9.70798304	9.319510202	9.363823202	8.277405556
7.053635222	7.501443394	7.704008545	7.616235111	7.449265303	6.826879909
8.423139949	8.798247798	8.542429747	8.601386131	8.616914869	8.585836293
9.235179424	7.997498828	8.518658667	8.446184828	8.834473929	8.967408879
8.299695162	8.105207222	8.389243343	8.089360869	8.170129495	8.190353414
6.683063909	6.434478081	6.493490929	6.640705545	6.643064293	6.888155525
17.67797782	17.01900663	15.81050193	16.64557528	15.90458538	17.67797782
7.785596848	8.189895404	8.351752182	8.06213903	8.092691677	7.128773535
10.37026232	10.46766096	10.63752253	10.25038968	10.77762269	10.52476206
6.936097616	7.133007667	6.66692301	7.152958919	7.330801667	7.626470505
14.53168901	15.09418019	14.60195877	14.40789433	14.78379092	14.26676228
9.126663535	9.969141677	10.23351878	10.00985275	10.00985275	7.752360667
9.072998758	9.095762	8.336257222	8.548304869	8.452374444	9.302103889
6.994029394	7.368846081	7.056486293	6.61686503	7.198896253	7.582654121
13.75520609	13.64083669	13.98081649	13.92894576	13.54221797	13.65450155

12.92937388	12.66167346	12.52819783	12.96241124	12.3543152	12.3543152
7.997498828	6.551865586	6.611130667	6.482726162	6.655986263	7.770819434
11.89361272	11.5386618	11.66934136	11.7894918	11.55322016	11.66441291
7.38400901	7.173022687	7.23423804	7.036743576	6.654064545	6.922412606
8.210602626	7.903688606	7.554062253	7.689234404	7.582654121	8.01423197
8.975268424	8.777572838	7.93027604	8.22479296	8.257984293	8.775519707
11.542568	11.38178745	11.67849095	11.59870577	11.65052704	11.38025327
8.460863192	7.574795778	8.456191465	8.398231626	8.548304869	7.989204111
13.5964752	13.73439002	13.94443	13.87958112	13.38447499	13.34730616
7.074173121	6.958952465	7.420741101	7.368198747	7.424927172	7.603389051
9.761765848	9.730279475	8.776574232	9.27419499	9.149684939	9.640429374
9.903372323	10.16454544	11.12613667	10.48310631	10.45375986	9.165150051
9.511202364	9.241421747	9.242526283	9.167114	9.590351455	9.026636737
7.373377929	7.913871889	8.254919778	7.716120232	8.043818737	7.773938747
8.420387707	8.179581869	7.574140061	7.855388333	7.593664717	9.07581704
5.980498747	6.16228202	5.841076919	5.901987303	5.956419384	6.038263586
7.092334828	6.668510737	6.576096444	6.656190667	6.615630737	6.975188859
16.8710039	16.73234105	17.07010739	16.82803465	16.90924948	16.73234105
6.123173242	6.09939598	6.199844141	6.312971121	6.296313384	6.240680768
8.583996586	8.294640202	8.532980343	8.77085696	8.652865111	9.133284677
7.152687384	7.371907727	7.539487212	7.613240606	7.559118222	7.101573333
8.585303495	9.084956182	9.491725303	9.274850788	8.956407626	8.104500051
7.224700556	7.505759091	7.264201869	7.091827414	7.227000899	6.964897566
6.97366599	7.721209061	7.329578283	7.705120374	6.680176879	7.070666535
6.635568162	6.755664455	7.344278889	7.199742475	7.055909434	6.502744273
14.1904887	14.02572128	12.96241124	13.57574016	13.50997822	14.25042984
8.867918475	7.939903758	7.391655768	7.66930299	7.546592828	8.706127333
8.181196919	7.525850576	7.483852838	7.251769333	7.994919717	7.962680434
7.966402293	8.160644788	8.532516465	8.358783152	8.567346869	8.582586091
7.328411747	7.212383152	7.166369152	7.446413172	7.491357222	7.572483949
10.97767761	10.88681216	10.71279176	10.6768734	10.61001127	11.11081747
8.364710758	8.564847253	8.113745737	8.196571586	8.727528626	8.78767504
12.70390423	12.71142619	11.24646499	12.25251148	11.89130603	13.17281418
7.310724131	7.099882485	7.245014414	7.193007566	7.28082202	6.896238273
7.119927869	7.519990596	7.527074182	7.479422939	7.786298434	6.506308818
9.147296667	9.663106646	10.01071956	9.739033131	9.730279475	9.956562586
7.68569196	7.15072903	7.08777197	7.098240939	6.746600626	7.601643838
9.149195859	9.584100455	10.18666124	9.558255162	10.05780386	8.912452162
13.07947925	13.11515098	13.17778072	13.3718573	13.74759592	13.13522491
9.314493212	9.403514808	9.779012071	9.294355394	9.439397061	9.235179424
9.803356646	9.622300838	9.960013465	9.23383296	8.310875455	10.35699559
9.203189636	9.220765354	8.902484929	8.966940586	8.854603364	9.267923343
10.15875628	10.35284213	10.31997281	9.934615404	9.917863333	10.18762619
8.060252263	8.08972896	8.358783152	8.468064667	8.334587636	7.705454838
8.740682	8.782655051	9.031713778	8.980943495	8.984231152	8.554971
7.432999727	7.238133434	7.587331949	7.714317071	7.514494576	7.331457121
8.04259702	8.48093102	8.480158333	8.526186556	8.291610121	7.600942677
8.335861768	8.806450121	9.385290182	8.923097444	8.928828384	7.736964596
6.545978343	7.020975202	6.506079909	6.546440545	6.370955485	6.241225677
10.89224764	11.24993851	11.19817469	11.1060681	11.16579624	10.31095185
6.804033586	6.738245586	6.872867768	6.993183869	6.786032343	6.36270599
6.716586707	6.916922333	6.757183747	6.789025475	6.662952677	6.813963929
9.580097263	9.810255869	9.566253768	9.622300838	9.430143091	9.169326323
7.155245384	7.246997929	7.132449434	6.991849242	7.29228501	7.726692222
10.80499832	10.60161194	10.82034332	10.93334198	10.71503879	10.6488688
8.626833051	8.474658303	8.546545061	8.864955909	8.414679525	8.10326497
8.389884152	8.197004869	7.697611606	7.622739535	8.134790222	8.181196919
10.93494087	10.91356479	11.40495249	11.33657706	11.16423752	11.24323027

8.189194404	8.586230848	8.857327323	8.615330515	8.735755485	7.805068949
8.642670768	8.36093298	8.460488909	8.543234566	8.276438626	8.161911788
11.85033795	12.08943903	12.40735915	12.27101	12.13244857	11.30506756
12.32364634	11.59654933	11.85889751	12.3543152	11.75215821	12.41033676
10.53737043	10.61480751	11.16579624	11.03008027	10.61371787	10.13276962
7.637262101	7.853698798	8.13932801	8.045722828	7.751645273	7.21289504
8.391020323	8.057063626	8.444861909	8.705145061	8.233006697	8.440058758
6.653830545	6.458735293	6.454504667	6.972319545	6.439107101	6.149487535
8.812517081	9.003925646	9.149684939	9.287819051	8.949261586	8.561321323
8.018723424	8.016429949	8.153159131	7.956615091	8.053971495	8.057864374
7.721209061	7.021219939	7.337989111	7.288727879	7.053327354	7.679634566
7.410622798	7.89560499	7.466342606	6.888392616	7.255477111	7.866966485
8.710391727	8.481851586	7.920877394	7.991889949	8.065153556	8.800397414
7.58642403	7.376746737	7.882525919	7.530291586	7.782462626	8.021628354
13.66158872	13.44849295	13.39037726	13.72760196	12.96662998	13.06967433
7.18421398	7.456429202	7.889381343	7.565422434	7.695912303	6.816280253
10.76361324	11.27153548	11.70812672	11.57567047	11.36065784	10.16743096
8.976374343	8.841037687	9.126080354	9.53973704	8.759757687	8.656835556
10.79145221	10.61992902	11.35080354	11.43565115	11.1031408	10.71279176
8.934777717	9.509876273	9.803356646	9.378583414	9.361671283	8.528915121
6.75400198	7.144617717	7.637262101	7.263859848	7.274379929	6.616628394
7.559825556	7.952774121	7.772177253	7.646275747	7.727729838	7.321250939
10.08700287	9.406893465	8.651919778	10.43440935	8.090886384	10.32946621
9.143279535	8.891888576	9.980047091	9.574050242	9.533596364	8.738735232
9.140978061	8.680513909	8.745901071	9.296131263	9.152615889	9.212980727
7.476561202	7.823737283	8.037664566	7.432695485	8.07053003	7.758961677
9.818059192	9.951175646	9.440510152	9.94402302	9.596773313	9.45706402
7.447717303	7.687700545	8.401710747	8.029383646	7.760294414	6.826408263
9.149684939	9.332175414	10.09795649	9.497817424	9.568406747	8.805907646
13.11515098	13.38447499	13.65450155	13.54965001	12.9851923	12.89113428
7.891912283	8.254919778	8.418118394	8.53537201	8.342368121	7.489812667
9.556759333	9.649399939	10.00985275	9.353057535	9.739033131	9.458377051
9.153730283	8.842639202	7.783847071	8.342773172	8.174069929	9.385290182
7.539167263	8.266842545	7.032569455	7.235366172	7.172125253	7.74678301
11.65261158	11.2278827	11.57567047	11.43380494	11.75662781	11.87912023
8.201303758	8.250194202	7.821271606	8.127895253	7.865213	8.50618604
9.312041414	8.841575667	8.443175586	8.471488636	8.369167818	9.281923404
8.80133401	9.436898869	9.737663949	9.453123465	9.038849273	8.329784131
10.2572153	10.04600105	10.61867663	10.57758511	10.68572341	9.667708556
7.269598859	7.787223354	8.06213903	7.924502232	7.671246505	7.124332475
9.194378495	9.163332636	8.82024202	9.256085253	9.257324	9.548028869
7.486088616	7.286722061	7.609574333	7.048974677	8.049019253	7.284105152
10.2752886	10.06997663	10.06547375	10.14131493	10.36349344	9.933588354
9.835894606	10.0406788	11.03452834	10.58689262	10.48659409	9.349388778
10.59292728	10.58689262	10.70902317	10.6240143	10.56306707	11.05796986
8.161033919	7.979092162	8.366462404	8.274698687	8.552208182	8.589797808
8.495447152	8.864955909	8.626833051	8.912452162	8.835493283	8.507703202
8.69335002	8.737817051	8.702574091	8.700142556	8.670447949	8.007640657
6.812677121	6.53573598	6.550797374	6.806225091	6.835608667	6.901187545
7.311327071	7.001377828	7.231529313	7.368501293	7.555333263	7.55945201
11.72126504	12.10694646	12.50328361	12.24933389	12.19727311	12.16627503
14.08841453	14.40789433	14.84412351	14.59173737	14.46162431	14.04335209
15.09418019	14.88238784	15.10875812	15.21350605	15.13975511	14.99939481
12.86086109	12.70787607	12.91196246	12.90783816	12.82558839	13.22730043
8.34368697	8.411696677	8.79576798	8.846850111	8.420387707	9.069558111
7.044506424	7.645645939	8.391460737	7.417927455	7.907924051	6.612991111
12.16279806	12.42616911	12.59668127	12.39973853	12.26475658	11.85301976
9.434425707	9.957374697	10.38430498	9.969141677	9.987874111	9.023071899

8.78214798	8.709464434	8.575566677	8.48742196	8.808928141	7.810980737
8.900236364	9.049629657	9.32739897	9.170513566	9.000069889	7.929593313
7.894965162	7.326942212	7.109678444	7.013654717	7.066192545	7.861535657
7.389558374	6.982909788	6.984849141	7.21289504	7.117401788	7.402347162
7.928481586	7.990313949	7.555661677	7.788923101	7.648616949	7.704008545
9.124449354	8.945002717	8.951513343	9.281923404	9.07526898	8.716387192
7.736964596	7.866656354	7.023936596	7.333224889	7.330546788	7.875433545
8.130616515	8.168196313	8.540635232	8.143040162	8.927565404	8.065602475
8.997807768	9.687758929	9.248396293	9.177504333	9.093001495	9.361671283
7.772843323	8.233421293	7.143440162	7.486088616	7.423442323	8.388817192
8.234636465	8.468418253	8.212685152	7.850180343	7.83046296	8.465000141
11.2345748	11.75215821	11.87687449	11.37670858	11.81439214	11.80554878
9.826032111	9.736957071	9.692793657	9.026117657	10.2572153	9.828404283
7.857447394	8.037664566	8.268510495	8.036387556	8.106019798	7.275765808
8.973478152	9.237664879	8.645985424	9.270407879	8.889380424	8.824381182
7.295703091	7.729367111	6.520559131	6.672296636	6.66713102	7.18313201
10.95093974	10.52904685	10.29324401	11.25349741	10.85654613	11.12001247
7.193571	7.579628838	8.03601997	7.886820596	7.984708455	7.444823313
10.52904685	10.76361324	9.744663444	10.14895288	9.998242414	9.966143818
6.651970061	6.412381	6.863923949	6.857241172	6.953109798	6.987890152
14.94534759	14.4335752	15.09418019	14.90726461	14.82007331	14.88238784
8.35667099	8.871209182	9.137887434	9.160413333	9.560346242	7.674109172
8.706567556	9.015966535	8.903025495	8.984797889	8.858692343	7.923423949
11.33100665	11.78023213	12.38063147	11.41605585	12.22629088	10.14534091
8.680513909	8.868979273	8.398231626	8.670884566	8.876650909	8.901935111
9.036076414	8.545617303	7.932066141	8.663303283	8.547449101	9.136296333
8.325316889	8.668536687	8.195405505	8.207099091	8.08112003	7.894965162
7.477539293	6.881026485	6.85466502	6.936850606	6.951169364	7.807154444
8.478263182	8.776574232	7.98506802	7.953840929	8.040575909	9.081281455
7.19003896	7.873080636	7.950135394	7.758961677	7.713612677	6.604297121
8.693780273	8.950355889	9.597513697	8.835019636	9.230770667	8.870056939
6.573072253	6.828622869	7.239071505	6.945089283	7.170797768	6.69920203
11.68948846	11.37847359	11.57178154	11.66189432	11.50051559	11.78023213
7.628690051	8.233838889	8.355387535	8.247731232	8.180413081	6.805713475
11.22119175	11.51647662	11.20163629	11.19817469	11.41981305	11.58814301
14.34357305	14.31917195	13.93694444	14.11997744	14.14208591	14.5413574
11.00053539	11.0035084	11.18941159	11.11714857	11.30506756	11.1453264
8.064260859	8.031407364	8.531247657	7.718747404	8.11047698	8.523456192
10.54078574	9.826032111	10.44832821	10.11941657	10.24414187	10.77762269
7.44049799	7.236577444	7.492282404	7.515186091	7.479422939	7.45028304
11.51008986	11.9091429	12.15752365	12.05813886	11.90674449	11.1276132
14.04335209	13.45466248	13.42888134	13.68884286	13.58209631	13.74093847
10.71992703	11.17378617	11.18096106	10.89912578	10.73661507	9.974493182
9.065009323	9.119831202	9.067856838	9.056533626	8.995584808	9.257324
11.55928848	11.50051559	11.52448673	11.60069924	11.34576518	11.52645916
9.162016475	8.646531515	9.113305192	8.859179455	8.815152444	9.491039162
9.037616626	8.992473788	9.048620525	8.833963152	8.960218374	9.479084687
11.78265022	11.65455063	12.06964699	11.6157426	11.81887725	11.93486375
10.35385551	9.637663091	8.555833323	8.603276828	8.769824364	10.39521192
7.187484444	6.762066263	7.358375414	7.191807606	7.362377768	7.16240796
6.740481444	6.519889687	6.772272192	6.621412929	6.924186162	6.506079909
8.241801545	8.251492909	7.868335495	7.950449798	8.156772778	8.722452253
7.875808798	9.274850788	7.867312374	8.256833364	8.044608626	9.377330121
8.696280707	8.940148162	8.017539697	8.549517636	8.389653889	8.791137414
8.762202232	9.402814081	9.851320949	9.548028869	9.444057586	8.488340929
8.024633293	7.547922717	7.918848717	7.885378	7.73355204	7.585232141
7.07011003	7.103272273	6.870614657	6.939713242	7.067022475	6.833358384
7.680845586	7.82475102	8.557697889	8.268052495	8.151663141	6.937364475

6.926979111	7.028039657	6.483554929	6.804729515	6.642585051	6.750670859
6.539797657	6.739060495	7.081067566	6.641378788	6.728952394	6.283373303
16.12384164	15.83165575	16.57174008	16.39933956	16.61084374	16.12384164
6.980238687	7.192117626	6.534413101	6.689415172	6.647679424	6.725993303
7.780116303	7.112259949	6.910693596	7.418256545	6.997949788	6.908354899
9.088505869	8.347358778	8.260520646	8.784649081	8.554027364	8.510894717
7.032313212	7.457632404	6.42554104	6.688924818	6.62116196	6.883018111
11.96636849	12.24933389	12.16627503	12.40735915	11.94481318	11.95261936
7.81867298	7.142295949	6.959934293	7.03699504	7.000811505	7.797125465
8.907429495	9.521972354	9.402230293	9.50710198	9.473222667	9.116279576
9.693515768	9.964343626	9.850553616	9.84311402	9.776839404	9.815129384
9.59460096	8.629055081	9.19736601	9.980879424	8.913588253	9.196132455
10.46108665	10.69574272	10.41330878	10.5104744	10.56655729	10.47479655
8.24281297	7.574489596	8.483326677	7.604109192	8.239474646	8.187968455
7.603719626	8.514431737	8.304980606	8.238272808	8.757866131	8.439112919
7.574795778	7.430781919	7.337386909	7.358375414	7.783110646	7.41731598
16.39933956	16.2036826	15.97330865	15.65613201	16.2036826	16.90924948
9.204855576	8.966940586	9.264483889	9.179226364	9.248396293	9.472607081
6.92802298	7.268488485	7.240600646	7.788172758	7.411268949	6.16631502
8.464558212	8.979921919	9.368875727	8.983202444	8.773471869	8.052783737
7.300330596	7.012842909	8.144161414	7.850890354	7.054238101	7.529686949
8.339115859	9.856174394	8.643157737	9.846508495	8.68840998	8.923576949
7.313208869	7.683943101	8.001277303	7.845601525	7.778752535	6.812677121
13.88591407	14.16629104	14.1904887	14.05136004	13.62106704	13.80033164
6.594827192	6.947784242	7.206091434	7.07011003	7.048974677	6.302911687
9.945028121	9.79615097	10.31795252	10.25422487	10.23160947	10.0060683
11.94481318	11.6157426	11.80448851	11.71042621	11.83330316	11.84529477
10.14044783	10.22951336	10.06822268	10.57996368	10.00450786	10.64128512
7.553068374	8.282073859	8.588971687	7.727729838	8.343220798	7.461509737
8.494982343	8.431738505	7.718083212	8.329784131	7.803944101	7.946141838
10.04160563	9.650789909	9.424985495	9.785983535	9.795448596	10.40701089
8.667554273	8.57971698	8.609952768	8.819236758	8.533396525	8.70424704
6.866852303	7.058479485	7.360165061	7.216692263	7.517436172	6.627335
15.70163214	16.04217659	15.01206542	15.31193978	15.29549959	15.41210544
9.574050242	9.763138212	9.763138212	9.511202364	9.735412273	9.168828475
8.593558293	8.911610283	9.459664354	8.989652131	9.01707996	7.934739636
9.750460616	9.639623879	9.37540303	9.484209455	9.511202364	9.285000091
11.83072395	11.46936418	11.5737214	11.66934136	11.22456459	11.79405389
9.283145788	8.967408879	8.901935111	8.747331152	8.786134101	9.068490586
11.92396137	11.73070023	11.9982543	12.01427079	12.12151457	12.18492446
13.48990673	12.71830686	13.27418186	13.44126502	12.78185519	13.79381649
7.788172758	8.066048727	8.276014121	8.239938859	8.21550303	7.524403394
7.915342566	7.922697606	7.509050374	7.262427071	6.269348929	7.856753939
6.556473354	6.407674848	6.714000354	6.689141323	6.765091091	6.368128081
6.797872071	6.736847222	7.564402343	7.257203303	7.084738172	6.862375091
7.037826263	6.617377051	6.305339354	6.459198636	6.31049204	7.545986354
10.04963797	10.94208941	9.415217505	9.960976253	10.03205964	10.20060626
9.40167604	8.967895455	9.103811545	8.936938576	9.680349586	9.582110717
8.050484323	7.704350384	7.615901626	7.855731	7.970926879	8.609511
7.76948301	6.669931545	6.823157384	7.030955384	6.773706576	7.479422939
12.27101	12.67378338	11.72126504	12.03068847	12.052647	11.04464523
7.052765889	6.81164602	7.377037515	6.96124304	7.345267404	6.907588505
11.12315281	10.85782556	11.15966862	11.04593523	10.88031852	11.3290504
9.035586242	9.342353505	9.290152727	9.355403384	9.350012182	8.946107263
9.205404404	8.876209859	9.009407333	9.156840283	9.11510898	9.662314131
10.61743608	10.87235819	10.70425349	10.83363242	10.87235819	11.17750487
6.685554869	6.900957616	6.844744919	6.676440081	6.940977263	7.044743657
7.582311101	9.169326323	8.298004222	9.143279535	8.25570701	7.193876586

9.335272182	9.25368898	9.076382242	9.08614898	9.236429667	9.389466253
11.5612151	11.98196729	11.93178106	11.40145091	11.51647662	11.80791686
13.39659876	13.02518615	12.26475658	13.18858184	12.28369472	14.05136004
7.494798263	8.109636222	8.434412929	8.058592364	7.924885333	7.070381242
7.691187768	7.638629778	7.897352323	7.599486586	7.735932323	8.092691677
7.068375576	7.262654505	6.824345162	7.204042263	6.536426172	7.649291899
10.60161194	10.29949835	10.43998577	10.58117749	10.57649171	10.67081044
13.22156044	13.05428976	12.86086109	12.89886333	12.76207554	13.58209631
7.828260424	7.242080394	7.10076196	7.120752303	7.037248667	7.762451808
7.832891081	7.677613273	7.380686859	7.710578727	7.845981061	8.179581869
8.066374202	8.984231152	8.724556485	8.60808904	8.683580283	7.461239687
11.79615897	10.99469779	9.980879424	10.5029511	10.3779146	12.24933389
6.438891808	6.515423162	5.913680374	5.932593626	6.573540778	6.943317879
11.25700815	10.81142627	9.021393263	9.70034896	9.588410576	10.66587479
10.59418919	9.923012889	10.21975253	10.3810842	10.55474917	10.65634987
8.333749495	8.526649808	8.675789182	8.391020323	8.514037303	8.236633323
7.034081253	7.057011424	7.251769333	7.108809091	7.093254121	7.581657414
14.1830221	13.98081649	14.4185289	14.25895571	14.01682491	14.23242051
10.573928	10.04340808	10.5199714	10.58579258	10.69931975	10.5397012
6.950902859	7.186679828	7.832540091	7.601304687	7.462771485	6.681851
8.092691677	8.355387535	7.980934222	7.945864808	8.150096222	8.580722586
11.53239215	11.6137143	11.87175588	11.86922315	11.96362869	11.9091429
9.804809051	9.588410576	9.066117364	9.333986848	9.805520727	9.724814657
10.75865445	10.23160947	10.3090972	10.83930015	10.41530743	11.01807842
12.60396528	9.772980061	10.19136651	10.11349335	9.54312803	12.65104366
12.63500423	12.31419772	11.83813795	12.2293359	12.16942168	12.8652792
12.27773509	11.86652814	11.87445957	12.19727311	12.32364634	12.01956714
8.068931465	7.577683121	7.85604701	8.002342131	7.949790515	8.18280501
10.72127155	10.84705809	10.92757694	10.22557955	10.71613341	9.785178838
5.780624475	9.746868596	8.882379495	5.792667778	8.676316919	9.580783667
10.65634987	5.861893434	9.528176495	10.81796524	8.990775909	5.819163172
10.29752835	9.961780586	10.1885747	10.31894523	10.39521192	10.78269205
9.396536798	9.20200202	9.363307667	9.304577515	9.214111545	9.560346242
8.349393212	8.264240727	8.135526051	8.241600556	8.382609505	8.710391727
8.835493283	8.208692758	8.813032152	9.031713778	8.31318196	8.466852202
7.728361283	6.510064202	6.397600616	6.680925848	6.500836152	7.97830402
7.803944101	7.648986848	8.082778869	8.04259702	8.049354232	7.765675424
8.026271202	9.141588162	8.265130232	8.248566778	8.314398727	9.323731354
7.295980475	7.218399283	7.011783192	7.328721848	7.799524616	7.939228495
11.95261936	12.46797904	12.92081703	12.63888703	12.62449502	10.90324033
6.192135242	5.768233283	5.805896495	5.902769929	5.752899404	5.757790323
8.24438497	7.951199808	7.943134	8.032499636	8.117339677	8.049354232
12.88708268	13.07947925	13.1944969	12.99088515	13.30255942	13.26276274
8.20265299	7.761029182	6.784856394	6.973416747	6.867131162	8.170954818
10.42297323	10.47370876	9.442729677	9.636296737	9.616741535	10.74945851
10.10946026	9.899969596	9.842388081	9.881912677	10.06633871	10.37686258
10.47820756	10.40810668	10.95374716	10.84176532	10.45754412	10.5041569
7.632188616	7.134092121	7.309336424	7.258429848	7.013137848	7.182269455
10.35178281	9.790672212	9.403514808	9.618256808	9.533033172	10.09339803
8.735287485	8.83948901	8.510413414	8.944409727	9.242526283	9.033394545
6.507801384	7.468494556	6.464011586	6.515215919	6.418892273	6.794527247
11.27695531	10.77507269	11.47871073	11.87175588	11.20660223	11.43380494
10.77507269	10.97054498	10.40387449	10.6426412	11.10460602	11.23116994
10.21975253	10.21583282	9.524763869	9.384078242	10.23850383	10.8405808
7.90869301	8.325316889	7.780448354	8.537544354	7.862160495	8.24122704
7.916025434	7.522209596	8.229734556	7.981345788	8.137069859	8.284505182
8.999492384	7.277482444	7.302435273	7.221024394	7.240898899	8.85518403
7.906425364	7.763186	8.280295667	8.382609505	8.553595747	7.940254566

8.57597697	7.996393263	8.640338566	8.876209859	8.416773909	9.087865687
14.47046648	14.27397716	12.91656586	13.56234785	13.35359128	14.46162431
8.263464364	8.196571586	8.559456798	8.386601707	8.511758747	8.061425949
6.977525061	6.617782121	6.656675747	6.546871707	6.632345869	6.873334899
11.95514832	12.0724233	12.04432	11.94745099	12.16279806	12.38794592
8.394903626	8.396963808	8.29635502	8.412956141	8.496365737	8.348543172
9.546645747	9.488337717	8.308776677	8.744081071	8.657806242	9.731150899
9.142791657	8.600831152	8.33024502	8.411696677	8.363019899	8.549141545
7.564741838	7.414724596	6.651524121	6.807473404	7.099269525	8.757866131
6.149487535	6.089688374	6.819920253	6.252936333	6.648620667	7.41959598
8.401317182	8.204380404	6.940479424	6.95496698	6.995800606	8.308331848
7.584620515	7.410002717	8.019434586	7.865908465	7.75371804	7.801006182
14.21449649	14.52085241	15.06622261	14.53168901	15.09418019	14.10328164
8.222643394	7.986161141	8.222223545	8.357077121	8.064713051	8.130616515
8.168969465	7.652632424	8.11047698	8.505797091	7.906781182	8.248986687
11.16281786	10.98492733	11.81439214	10.71992703	10.86963124	10.94208941
6.739522626	6.609208404	6.651250333	6.917518081	6.694950828	6.578458283
8.200961636	8.274698687	7.760294414	7.641344242	7.724369343	8.344860929
8.811517808	8.298896434	8.567346869	8.59655801	8.963499596	9.622300838
7.755503707	8.446184828	8.090886384	7.985767212	7.825104404	7.692888131
13.81935116	13.66158872	12.47441306	13.08418191	12.99088515	13.75520609
8.520754616	9.165786899	8.135850465	8.409959899	8.598008343	8.938439909
12.32964397	12.03602449	10.50794857	11.13400706	10.82804231	12.43944324
11.61117883	11.72355631	11.0473733	10.95849458	11.16734497	11.56988377
6.188743172	6.69920203	6.896938141	6.776493808	6.773240303	6.017525253
6.76944904	7.179751081	6.565804798	6.507219869	6.564717364	7.580266434
7.175476838	8.012346152	8.41335802	8.349393212	8.564847253	7.200755828
9.568406747	10.05780386	9.857104919	9.980047091	9.647013263	8.81572204
6.921891778	5.943440172	6.082207485	6.076308061	5.99991201	6.089257051
7.216692263	7.36298202	6.902984	7.126018333	7.909031556	7.40806004
10.70425349	10.70425349	10.19528987	10.4524716	10.33375706	10.31013098
6.978374222	7.587331949	7.371907727	6.92669298	7.077700404	6.912025515
7.704350384	7.114036293	6.544818747	6.90271597	6.731476051	7.386173242
7.616552222	8.221010253	7.545335788	7.988882495	7.818322879	7.720197707
9.571117879	9.846508495	9.499247869	9.495686737	9.721175788	9.758431737
9.651465606	9.874496818	10.01257663	10.10093339	9.924448444	8.841037687
8.815152444	8.723452192	8.817758687	8.871686232	8.925792808	8.143040162
11.20163629	12.04990566	10.22557955	11.21668249	11.23116994	12.33907422
8.448008778	9.062782333	9.279768394	9.159785394	9.266188263	7.692552364
10.8443678	8.957944414	8.880853535	8.722953263	8.324880909	10.88031852
7.848096879	6.670809131	6.675987374	6.905826838	6.741974778	6.715418303
9.451851384	9.370225242	8.50473503	9.412044242	9.526870273	10.03701736
6.353011444	6.547549889	6.417447212	6.295493455	6.356085939	7.185712515
10.30695028	10.30151978	10.57028524	10.0178996	10.15283227	10.35385551
9.627745303	9.191849071	8.800782677	8.814624313	9.023639636	9.660200838
15.21350605	15.61926687	15.05177986	15.46469304	15.27762024	14.3093669
9.497817424	9.126663535	8.637033606	9.09013302	9.114524374	10.15283227
10.35591393	10.57885616	8.840590677	9.35188598	9.311440606	10.39747702
7.942409707	8.307483899	8.015705778	7.827504414	7.85507	8.012010707
10.75062885	10.8193093	10.75865445	10.88546605	10.89224764	10.5104744
7.206984404	6.95496698	7.112259949	7.419275485	7.28113	7.439837192
7.747885899	7.454572909	7.573788949	7.664275131	7.586712596	7.70299303
6.185668061	6.176177303	6.462851414	6.21945704	6.233403424	6.083662081
10.79564384	10.7532413	11.23116994	10.86305205	10.69354222	10.3918566
8.966282283	7.876807081	7.381950828	7.680240364	7.508036465	9.060618192
7.71023803	7.758961677	7.74578199	7.744459273	7.782178323	7.552094283
9.585510535	9.826793596	9.662314131	9.626304798	9.660861778	8.63568099
7.091328697	8.064260859	7.311629869	6.909148313	7.506099556	8.966940586

10.71613341	11.03737422	10.76609029	10.66465811	10.58117749	10.51650912
7.744142424	7.739667424	7.212654808	7.526167626	7.36694602	7.640655485
8.932093424	9.486281202	9.144457596	8.760676242	9.384699788	9.174793152
6.500607838	7.018424152	7.204326061	7.152420131	7.034081253	6.111573
10.12610261	10.21484784	9.613206141	9.604175636	9.852239606	10.66465811
8.84935699	8.598980253	7.851640596	7.620272192	8.634342798	8.777050384
6.929929141	7.33379003	7.21150504	6.863389636	6.657655061	6.262348667
6.286294667	6.460516394	6.678790232	6.361759	6.127679485	5.924046616
11.10170724	11.06573849	10.67815746	10.88809182	10.94640834	11.53050203
8.020887778	8.305787747	8.341518747	8.252794354	7.889381343	8.304169929
9.849671929	7.197763283	7.368198747	7.574795778	7.333224889	9.857940545
7.471589222	7.187213162	7.067867747	7.286117455	7.28582396	7.474059606
7.817254242	8.082778869	7.518690889	7.788532485	7.669946162	7.571747222
9.310775172	8.867918475	7.514494576	7.963371182	7.964547263	10.42927797
12.04734694	12.38794592	11.99323436	12.31719428	12.24351077	11.48459908
8.496365737	8.190781646	7.944736172	8.414282596	8.706127333	8.529417101
8.31318196	8.918756414	8.069753788	8.373514202	8.282900293	7.691535778
10.22448985	9.722667242	10.05144087	10.23351878	9.981718727	9.515743253
7.51340999	6.77992704	6.913511828	7.058212263	7.163006	7.468494556
9.55462702	9.406122899	9.719830172	9.662314131	9.992117576	9.600185283
6.828875111	7.471589222	7.421037465	6.57397498	7.419275485	6.777485293
7.418614091	6.528405747	6.601004717	7.001869131	6.515215919	7.256596263
10.22267877	9.247274333	9.320738646	9.07581704	9.17861001	10.51771241
9.332175414	9.316319051	8.954798808	9.080123545	9.151431899	9.239472869
12.04160066	11.90674449	12.77841715	12.20640951	12.51766186	11.69188536
9.145664475	8.937936253	9.062782333	8.810986707	8.894491141	9.650129091
7.805446323	7.07551896	8.096128192	7.695265798	7.839647131	8.698751323
9.237664879	8.346585616	8.875711838	8.854603364	9.143279535	9.382205394
9.786685869	9.564343333	9.763952101	9.842388081	9.790672212	10.03796113
7.453291162	7.318539616	6.606197394	6.452131838	6.389460141	7.540434798
7.424346313	7.233950838	7.250238485	7.07551896	7.202115061	7.396159798
7.124025636	7.932453717	8.658285303	7.652632424	7.680845586	7.562627657
8.859179455	8.726978374	8.661956242	8.817758687	8.754900343	8.892401576
12.93862501	12.54268587	11.33477589	12.19398134	12.31419772	13.03496614
10.042425	9.681142253	8.783584889	9.085558838	9.069558111	9.651465606
7.542783869	7.61856696	7.216973434	7.456122566	7.251769333	7.220780818
13.69473258	12.10135206	13.34730616	13.34204398	13.42153085	13.76702458
11.84027237	11.88375452	12.01427079	11.96636849	12.01673563	12.23505088
10.93646713	10.74690378	10.79145221	11.18096106	10.2761618	11.04156144
7.984708455	6.375910899	6.34460196	6.778966495	6.719301162	8.620790505
7.994228667	8.831201768	7.396513838	7.541090081	7.667962596	8.122341
7.907197636	8.295839707	8.551751717	8.151663141	8.298396535	8.153957263
8.112525414	7.918105152	7.390752071	7.688677182	7.711891616	7.935875313
6.686787465	6.205556535	6.064770778	5.914206222	5.770150273	6.388351374
10.40925212	10.66105788	10.11452638	10.32635032	10.57885616	10.66832974
8.213832515	7.307562737	7.697940283	7.729367111	6.879229626	7.688011354
15.52328152	15.32551587	13.35359128	13.9071606	13.72760196	15.06622261
10.01524205	9.240082808	10.03796113	10.23752804	9.70574997	10.01524205
9.774477667	7.471847707	7.705814323	8.191204879	7.973992111	10.0719806
8.895503556	9.146178758	9.192533242	9.299183616	9.281446566	9.356741384
9.377908	8.886620687	8.83948901	8.838495424	8.782655051	9.322592778
8.742149222	8.637513768	7.825767899	8.326518101	8.097958939	8.609106909
8.559975162	8.613130333	8.522156747	8.706567556	8.85049999	8.770297859
8.838020616	9.174120707	9.433258586	8.963499596	9.106823152	8.483326677
8.479185606	8.285708747	8.292509485	8.316570081	8.093860848	8.53715797
8.228510596	8.408718253	8.154705535	8.474228909	8.387507626	8.533396525
7.902229798	7.736260545	7.658441616	7.784529071	7.90869301	8.211411717
6.874493707	7.614146707	6.791252535	7.247290424	6.74585002	6.809486374

8.685647889	9.235747242	8.890373556	8.780092131	8.862785404	9.289535939
8.474658303	8.435296202	9.053706202	8.258860253	8.147147636	8.643157737
8.286128505	7.996770556	8.018723424	8.307093606	8.228510596	8.419470808
8.756396081	8.657372182	8.346989566	8.879330566	8.85308204	8.947623838
9.623794152	8.987499273	9.600185283	9.586238192	9.824540899	9.704210374
9.655088667	9.387059283	9.268661657	9.804809051	9.528804131	9.167114
8.093077828	8.946596606	9.161011869	8.875206121	8.791657869	7.949400121
8.523871909	8.298004222	7.815532475	8.032957859	7.024510747	8.919862162
10.06547375	9.333986848	6.537747374	6.967663404	6.285518596	6.689933697
10.40074116	9.533596364	9.417037424	9.447991384	9.49510903	10.20532801
8.59655801	8.615330515	9.558925838	8.81966502	8.891888576	8.504327141
9.305189859	9.251353071	9.273640758	9.157435455	9.415860727	9.518503263
10.02161965	10.27338352	10.10365156	10.16262583	10.26729954	10.31378621
8.578393657	7.691535778	8.359263212	8.185177828	8.094218354	8.644542606
5.784265253	5.789534242	5.778670313	5.793066939	5.786246919	5.819207141
11.32185019	11.09565519	11.04886125	10.93935201	11.35278885	11.11560115
10.97896732	10.87379507	10.56421743	10.80091358	9.919491525	11.33292491
10.66229262	10.60863639	10.49592664	10.51771241	10.86820493	10.71613341
11.55524693	11.48459908	10.30058205	9.877792838	10.78269205	12.43279956
10.85225864	10.65987528	11.10035391	11.08107282	11.27306561	10.17613521
7.270153101	7.024222101	7.336145273	7.277849899	7.386763141	7.470886646
7.730154939	7.871272152	6.518759273	6.783595586	6.792730596	8.261781515
7.32157099	7.388278253	7.236914141	6.354771242	7.938531869	7.45822897
11.01531103	10.8605042	11.30690717	10.63752253	11.38025327	10.96311364
10.50057506	7.701029869	7.045942	9.501970121	8.708995576	7.57049196
8.216823323	8.498503737	8.236986626	8.433042727	8.273483707	8.558108202
9.999132495	9.965319232	9.852239606	10.04862183	10.18468509	10.37215733
10.10841911	9.566949	9.643287657	9.907546465	9.625041525	10.3380268
7.233039222	6.841391899	6.331115141	6.270843101	6.179354162	7.412474121
8.295436051	8.366948404	8.204737434	7.774939586	8.380307172	8.879851283
9.815893677	9.043522909	8.450171172	8.532119949	8.370422404	10.09597734
12.00644476	12.29886409	12.27421104	12.04160066	12.47441306	12.35798465
12.33907422	12.03310273	12.18142186	12.14191765	12.32659908	12.28369472
6.537516434	6.158989596	6.085156525	6.184860172	5.999381293	6.499057505
9.027136657	8.908475283	8.923576949	8.872352707	8.912452162	9.373397364
6.392953667	6.129263667	6.13150703	6.687475475	6.423272566	5.77365997
8.583542788	8.44047504	8.439612798	8.379884889	8.700142556	8.76478299
6.273265505	6.380219	6.442532636	6.483130091	6.486163293	6.873999848
7.95873603	7.594662899	7.909763434	7.938205586	7.819085697	8.427236737
9.486281202	9.499247869	9.081927515	9.276789111	9.293068828	9.287277576
8.511758747	8.696739152	8.562227051	8.50618604	8.591651626	8.707515232
9.057196687	9.229670424	8.294640202	8.739178636	8.584413131	8.494130061
12.34855768	12.21630458	12.57107442	12.53197264	12.78645268	12.70390423
9.028300162	8.488340929	8.700606657	8.812013889	8.82024202	8.914720172
6.59799604	6.85163704	6.879943566	6.933584646	6.880211293	6.76551097
7.132177788	7.407756293	6.655986263	6.973893828	6.891529131	7.727729838
7.924502232	7.896600606	8.142197384	7.862831293	8.002342131	8.198946162
8.061425949	7.778362485	7.535530424	7.740469253	7.634532263	8.102124455
9.93187304	10.15283227	10.48432644	10.17613521	9.892629697	9.374121768
8.59942799	9.099925818	8.367422212	8.32939098	8.12949599	8.737323475
7.760294414	8.072110051	6.788320081	6.831376636	6.629471747	6.344188131
8.754900343	9.531623293	9.622300838	9.679062616	8.753927596	8.279889293
9.359783616	9.763952101	9.548618051	10.08893073	9.879566364	8.804477505
8.316081222	8.265130232	7.51061902	7.753046657	7.603389051	8.149032212
9.400327152	9.137373131	9.536330707	9.254253192	8.992957212	9.452497313
12.08943903	12.13244857	10.81664077	11.5737214	11.4535969	12.42233701
5.841200939	5.768873263	5.805546232	5.985453778	5.758070626	5.824390354
7.416977434	7.215770273	7.141446111	7.380085202	7.222444626	7.878518051

7.979449616	7.337098929	6.815206394	7.011036525	7.032313212	8.181574081
12.66167346	13.22156044	12.77015535	12.92937388	13.0147344	12.36759935
8.675348646	8.409959899	6.358295333	7.091067131	6.959934293	6.434266404
7.722652869	7.232151747	7.646953505	7.806845253	7.693213707	8.134366646
13.29684189	12.12436236	12.31719428	12.61449239	10.96162926	13.80704919
7.595878485	8.041000495	7.726692222	7.671593667	7.841440677	7.38340502
9.652169778	9.079484556	9.461673394	9.896649636	10.00262223	9.550769788
11.58814301	11.74439188	10.11051676	10.67428006	10.49009312	12.0089388
7.293748525	7.44112097	6.639726192	6.799617657	6.735607747	6.375038899
8.87289497	8.474228909	9.046362323	9.629101929	9.406122899	9.688441414
7.697220202	8.443956768	8.032499636	8.434806414	7.922041747	7.404051424
9.483552818	9.081281455	9.031155364	9.250770152	9.004940778	9.450568111
7.394370293	7.997875909	8.507703202	8.738735232	8.245649515	7.629016404
11.61814895	10.38208186	9.530933293	9.872596081	9.58708204	11.46936418
6.022865747	6.174981131	6.664911182	6.050896313	6.093889788	5.863405263
7.927460424	7.273224596	7.081850444	7.158855657	7.016433495	7.795765162
10.82153564	11.74205097	11.96362869	11.46724679	11.70348036	10.10365156
6.748092424	7.316406697	7.256041222	7.08160301	7.166695253	6.708308495
11.0387546	11.19966733	11.20498985	10.90754603	11.48840455	10.47820756
6.845568374	7.38340502	7.485191121	7.282365313	7.565422434	6.904054566
7.522869182	6.557678737	7.16240796	6.871904646	7.397737788	7.152420131
9.333986848	8.510894717	8.225777414	8.425847798	8.339926909	9.238221677
7.606370131	6.542314697	6.620195253	6.620737576	6.720541949	7.576687677
7.949790515	7.394370293	8.571676263	7.64030596	8.497692788	8.32400903
8.256076333	6.803094364	6.519889687	6.71121296	6.505445727	8.409548323
7.601643838	8.305359949	8.221010253	8.142197384	8.275152535	7.033114263
8.950355889	8.946107263	7.894965162	8.013487535	8.144584758	10.0841781
7.082724758	7.015941667	7.856391758	7.622357354	7.32489101	6.834332747
8.249350414	8.668062838	9.133284677	8.754900343	8.501579646	7.981709394
7.973227556	8.069395848	7.92597801	7.819838162	7.782796596	7.794719919
9.384078242	10.37215733	8.77962597	9.041236566	9.047491869	9.459003
10.74558579	10.78014639	10.49830212	10.7982674	10.72786832	10.69931975
6.423456465	6.774434798	6.178002222	6.331869444	6.236419566	6.362142859
6.432616596	7.035420475	7.409710545	7.101319909	7.267888182	6.380660929
9.180285111	9.35128199	9.968215758	9.677492576	9.200683333	8.848914293
9.899969596	8.974650333	7.381027636	7.814474586	7.594662899	9.06557301
9.073600626	9.440510152	9.10215096	9.374121768	9.151976485	8.447076788
11.14385742	10.91641235	10.06997663	9.939327313	9.943097475	11.57178154
10.38001468	10.35178281	10.47820756	10.50794857	10.31289083	10.71992703
12.09225922	12.95787308	12.77454263	12.10951308	12.70390423	11.83330316
12.61449239	12.54589185	12.57486869	12.75784116	12.70787607	12.9851923
6.764115131	6.778730485	5.906792141	5.966829434	5.978534414	7.351945465
9.826793596	10.2166926	10.66342196	10.2861211	10.19236777	9.224452333
10.17224075	9.84311402	10.24038579	10.26729954	10.14534091	9.793126455
9.222631263	8.988577162	9.204345657	9.356058495	9.246644323	8.940763364
8.247392788	8.47014499	8.452374444	8.466446051	8.499404646	7.840025778
10.42409385	10.38631174	10.22668357	10.74172297	10.09597734	10.20859114
10.080472	9.813557354	9.742174424	9.822898081	9.387656788	9.982665242
9.160413333	8.494130061	8.767368071	9.346206162	8.826846768	8.633351606
8.989652131	9.843921515	10.19332507	9.214694788	9.569854202	8.918202434
9.530933293	10.31480319	9.995698596	9.612498475	9.664626495	9.340550081
10.95374716	11.11230451	10.91356479	10.87092264	10.8935809	10.43757507
14.01682491	13.80033164	13.81266894	14.09521817	13.70143341	14.19920898
10.78856661	10.69354222	10.58812248	10.84974643	10.5041569	10.71145754
8.907915899	8.882379495	8.770297859	8.875711838	8.702074263	9.360502949
9.917863333	10.1684149	9.746145949	9.895061889	9.885156737	9.721958051
7.153217667	7.245319404	7.204042263	7.279114576	7.220780818	7.300330596
11.37847359	10.7442872	11.32559397	11.09721747	11.33292491	11.64244603

7.459405444	7.586125333	6.696473434	6.901187545	6.804494667	7.40744601
5.937497727	6.939171505	6.261965667	6.300917343	6.393637899	6.149648949
7.516753121	7.71023803	7.618249414	7.161069364	7.382214869	7.818322879
7.370383778	7.389236737	6.778470374	7.137721343	6.357131071	7.497307495
8.921533879	8.693780273	9.232683343	8.915814212	8.638952889	9.159196141
8.236633323	8.323157747	8.728449242	8.381309202	8.188779434	8.686126354
7.307022919	7.486088616	7.56409603	7.423727141	7.44801498	6.871668566
8.984231152	8.577417788	8.746767313	8.846355515	8.959130566	9.096947232
8.538380869	8.166172404	7.792215626	8.056301667	7.940608939	8.684654737
7.262118616	7.335878859	7.552833101	7.795429838	7.483257202	6.580361485
9.837575172	9.383480283	9.423026242	9.415860727	9.828404283	9.979092404
7.574489596	8.02125603	7.940978939	8.189194404	8.071334404	7.382527051
9.027655758	9.321347081	9.288999586	9.010430586	9.365016101	9.387656788
7.008962394	7.050347636	6.123014818	6.497644434	6.409265687	6.006188242
9.066117364	8.647922808	8.765233303	8.771294657	8.978723121	9.376667182
7.697940283	7.708922879	7.040523253	7.367541707	7.242951465	7.535530424
8.825312061	9.538263677	8.937936253	8.940148162	9.122138505	8.680072242
8.169730152	8.372772768	9.359783616	8.96575796	8.792768747	8.712915394
5.984084283	5.994507889	5.825133505	6.02626803	5.921638606	6.123671758
9.168828475	9.536330707	9.831087364	9.705000525	9.851320949	9.143279535
8.949261586	8.78214798	8.985325394	9.273022949	9.244399414	8.758871
6.756409455	7.091328697	6.414036263	6.96434804	7.194952889	7.236271343
8.372086737	7.647964475	7.993428586	8.180785838	7.239398515	8.121211444
9.433258586	6.957403667	6.961758566	7.087495071	6.767482869	7.215185141
7.942071303	8.613521394	7.826840444	7.99823603	7.51247098	7.341038
7.515816273	6.860179071	7.223246162	7.807594768	7.960192616	6.971547919
8.544633697	9.35188598	9.344138121	9.039989364	9.206093556	7.899682051
11.0473733	9.517771202	9.420326576	10.82290783	9.466304111	9.845595727
6.201106192	6.292500646	6.564512374	6.149487535	5.917584465	6.270128909
8.621272737	8.655437626	8.464558212	8.44446797	8.739178636	9.129114848
9.860587788	9.912769303	9.829822242	9.837575172	10.03983393	10.42622906
6.622911394	7.050890818	6.875689354	6.309118495	6.858687869	5.949920465
8.184050869	8.541524636	7.911629535	8.101068051	7.939903758	7.793372141
7.777311636	7.115757364	7.545628697	6.903511051	7.225301495	7.781852747
7.289658101	6.845301404	6.39883498	6.555521899	6.363888626	7.496345141
10.0672481	9.738398616	9.811091061	9.903372323	9.954681949	10.07857135
8.909945455	9.37540303	8.911109919	9.234418071	9.285586323	9.048100778
8.385691051	8.317411434	8.900779434	8.613521394	8.84935699	8.951989212
11.2518391	11.61117883	12.12939166	11.67593314	11.85617239	10.66105788
7.615524929	8.187968455	8.282073859	7.763846354	7.928481586	9.192533242
8.408296424	8.007640657	7.778011646	7.952029848	7.799854778	8.481851586
7.541384646	6.915399929	6.786285848	6.981618121	6.808010384	6.802568323
6.710717586	7.084441778	6.517646071	6.612060818	6.48207598	6.76309904
6.02015296	6.131379717	6.545978343	6.849968616	7.064817384	6.729937747
10.35917202	10.65634987	9.650789909	10.08599138	10.156858	9.972781707
7.843077242	7.676615465	8.492322828	8.207926798	8.054318061	8.281588828
6.306878242	6.434897949	6.821514788	6.727438899	6.693719727	6.340052
10.70181173	10.15186663	10.70181173	10.85361094	10.26445444	10.26445444
11.17571904	11.73972102	11.96636849	12.14507673	11.92937262	10.65871503
11.16888788	11.81439214	11.80104424	11.90384839	11.87912023	10.18000397
8.581169111	8.770297859	9.333361616	9.137887434	9.066117364	8.408296424
13.39037726	12.66627862	12.58612865	12.92081703	12.64708972	13.86604428
7.870356606	7.442908828	7.876418838	7.869396606	7.980256929	8.173633596
6.975188859	7.373983485	7.302140758	7.257820879	7.290204495	6.865318939
7.871683444	7.98285402	7.557268586	7.738963899	7.372238061	8.177146606
7.161342051	7.252299788	7.580904889	7.239990273	7.081850444	7.147201253
9.425521616	9.189045596	9.430143091	9.451851384	9.243096889	9.420326576
8.979921919	8.628622212	9.369545939	8.70424704	8.966282283	8.778623323

7.980568657	7.707585606	7.163006	7.435437465	7.442598384	7.689234404
8.281588828	8.603276828	8.649462798	8.363468485	8.559011545	8.51269104
8.655437626	8.189194404	7.996393263	8.054318061	8.341518747	8.344441616
8.962353222	9.249034202	9.334658071	10.20060626	9.260607	9.413942061
6.755440343	6.874731313	6.778730485	7.12824003	6.763620737	7.039183869
12.95787308	13.32021166	13.29684189	12.00354228	13.83173935	12.91656586
10.25320711	11.31267384	10.98037262	10.91356479	11.32027501	10.52135963
10.57649171	10.88945836	11.61117883	10.66229262	10.87518762	10.04600105
6.116113394	6.629727051	6.872611121	6.592903869	6.514559495	6.112843636
7.353244222	7.058212263	7.113452899	7.309928404	7.06161799	8.100222556
10.16165443	10.02161965	10.76471511	10.31095185	10.27338352	9.680349586
6.146851515	6.516258222	6.161098354	6.107803293	6.037311121	6.186533747
9.647013263	11.96059797	10.28089537	11.41778198	10.1985269	10.40592026
7.33379003	8.241600556	7.528004313	8.253639283	7.851994586	7.452109758
7.164949697	7.362680232	7.476561202	7.319616859	7.717737636	7.25402203
13.10484893	12.65472241	12.73731843	13.33155169	12.8652792	13.11515098
6.489542818	7.596851556	6.966460535	7.303836939	6.958715091	6.757183747
8.185943182	9.076382242	7.993875677	8.777572838	8.447076788	8.274698687
9.532335293	10.06450841	9.122138505	9.560980909	9.436898869	10.24511785
10.54692936	10.26445444	10.21769501	10.55474917	10.69099899	10.60044575
8.325717687	8.02698897	7.027744404	7.165538758	7.813414727	8.210602626
11.24646499	11.62290433	10.761049	11.14211434	11.19966733	11.89876792
6.075996354	6.01801997	6.157329697	6.000571626	6.170362909	6.385400465
8.768863636	8.531655515	8.687053253	8.47107903	9.005433152	9.687758929
11.35657003	11.09885051	11.73070023	11.37847359	10.92608881	11.26815786
6.201274	6.612755929	6.146166717	5.892551778	7.136875657	7.376443525
8.592161788	8.403930677	7.923423949	7.837507091	7.780823677	7.890755525
9.600888859	9.432003333	9.488971182	9.447242061	9.36748	9.35128199
8.52575703	8.44446797	8.161911788	9.095162899	8.31008898	8.276014121
9.511857455	9.512482222	9.719000626	9.257324	9.803356646	9.854472677
9.410728505	9.972781707	10.05523038	10.05236285	9.869466131	9.317615606
10.22557955	10.48185706	9.921211657	10.31193963	10.49830212	10.13768673
10.69574272	10.64502212	10.79420974	10.86566339	10.75178718	11.20498985
12.62449502	12.93862501	12.25251148	12.51766186	12.52130815	12.26475658
8.743617505	8.336257222	8.714332636	8.602379626	8.400903717	8.65497299
7.940608939	7.956286283	7.871272152	8.233006697	7.858612495	7.988582505
13.70143341	14.65397593	14.42673578	14.65397593	14.45281427	13.10985871
8.149448323	8.020887778	8.27018399	8.515350859	8.219832939	8.07563398
10.94353341	10.58935103	10.11349335	10.38208186	10.22951336	10.65127749
9.148511889	9.716648152	10.15086094	9.613206141	9.513164303	8.985821889
8.314850485	7.74961303	8.335861768	8.475210818	8.375668162	8.487847515
7.492282404	8.32856404	7.019424707	8.295839707	6.535531162	7.404355202
6.282012172	7.734550869	6.901187545	7.465356566	6.757183747	6.852150101
8.725555596	9.636296737	9.685649929	9.467500556	9.415217505	8.581684657
14.67552064	14.49002723	12.76207554	13.76702458	13.23341018	14.36999747
7.520992313	9.004940778	8.137069859	8.715328101	7.983913121	7.400864778
9.49510903	10.13862753	10.24414187	10.37026232	10.08978265	8.76626204
10.12314196	10.61743608	10.9102587	10.62142174	10.61992902	9.715870253
7.479422939	8.038354192	7.840025778	8.022720303	7.995268899	8.139691131
7.665987182	7.962004212	9.626304798	7.614497747	8.198946162	7.726017717
11.03737422	10.94496034	10.02061657	11.28370047	10.39641485	10.70775781
8.891390919	8.921011566	8.433939838	9.069558111	8.524356212	8.408718253
7.997183333	8.36556403	8.429582192	8.038729465	8.24122704	8.899254879
9.286110869	8.833963152	9.212980727	8.07651197	8.841575667	8.749346626
8.195405505	7.730154939	7.750957677	8.061425949	8.150448232	8.569735879
12.23191382	12.34855768	13.2158469	13.05428976	12.92081703	11.82112126
9.879566364	10.33375706	10.27714677	10.09506496	10.41626133	9.948421707
9.251353071	10.03796113	9.545191596	9.999132495	9.683334444	8.77962597

9.675904202	9.686378667	9.014335657	9.501306556	9.304577515	9.851320949
8.808345061	8.588971687	7.312858101	7.663954061	7.52530399	9.209526556
10.06450841	9.510536	9.635582141	9.356741384	9.185497838	10.21484784
10.47584162	10.43564423	10.18291229	10.43872423	10.51771241	10.7814083
7.366646525	7.582311101	7.379773919	7.512168657	7.451961045	7.412806768
12.41337695	12.48835313	12.24097391	12.67700968	12.40343679	11.71895032
9.266188263	9.782133848	10.43332542	9.975431768	10.04160563	8.645985424
7.937868929	7.639990515	7.402347162	7.469040515	7.457632404	8.002011657
9.968215758	10.12224713	9.841562192	9.332175414	9.392082222	8.846355515
9.916104475	9.598265626	10.29660096	9.867106131	9.056062667	9.508442768
16.25437967	16.39933956	16.12384164	15.99508933	16.09492117	15.52328152
10.18098661	9.659537192	9.796940758	9.598893818	9.35728196	8.498949848
7.993875677	7.695912303	6.771643182	6.990730697	6.938620788	8.045324071
8.971727333	7.64030596	7.385218303	7.989587677	7.892604899	9.307024131
7.598885727	7.329870525	7.400588576	7.567404636	7.380377788	7.840343939
7.792215626	8.554971	7.842052606	7.998587576	8.45451298	7.687700545
8.047188455	8.213427111	7.836828747	8.167383949	8.030181424	7.653305051
9.19736601	9.368224626	9.265583747	9.308918081	9.306330646	8.694840596
8.509502414	8.727528626	9.063885091	8.825911939	8.758364879	8.283330131
7.878949444	8.23581501	8.116577384	8.118136869	8.183602061	7.49864798
8.540202646	8.131862263	7.00817199	7.269598859	7.149903152	7.677264869
7.020179424	7.291982576	6.947784242	6.56193898	7.21289504	7.97753898
9.038849273	8.975268424	9.185497838	9.489614919	9.112697707	8.710842768
8.618302869	8.813032152	8.421546263	8.293340606	8.336651323	8.401710747
9.809529798	9.568406747	10.11246778	10.07479617	10.07956055	9.272374697
11.18800276	10.72900488	10.89755671	10.41957053	9.945028121	10.91641235
14.50017253	14.09521817	13.81935116	14.21449649	14.17411554	15.22815255
8.225777414	8.135850465	7.160791343	7.363288879	7.243253313	8.397853727
8.159907626	7.706911222	7.464704768	8.023866626	7.917403434	7.918472515
8.235388636	7.975072535	7.21289504	7.334066545	7.318539616	7.274888343
8.586731747	8.510413414	7.979092162	8.157153081	8.272231939	7.787525596
7.469344313	7.714004283	7.654256818	7.476910626	7.451512687	7.189410667
11.0859227	11.47496104	11.32185019	10.90606985	10.86447048	11.109559
6.366338828	6.166137596	5.895512636	5.807759576	6.243346859	6.261769939
7.717447071	6.963370131	6.432616596	6.071029081	7.074173121	7.840676768
11.12613667	11.88649013	11.89876792	11.61814895	11.20163629	11.11388317
13.56234785	12.95303817	13.54965001	13.37822002	13.32669092	13.73439002
12.76207554	12.86955734	13.97489728	13.44849295	13.30795266	12.65472241
8.694840596	8.618800343	9.343457394	8.950874253	9.449254061	8.515864333
8.766842081	8.942756889	8.802954374	8.945002717	9.187808616	9.243679384
10.68687892	8.812013889	8.831201768	9.058895657	9.070117818	10.97476091
7.592388485	8.119679141	7.161605929	7.397472212	6.997357515	7.933221424
8.989142232	9.123297364	8.961327374	9.303342798	9.175472828	8.651919778
12.05813886	12.32024961	12.26775966	12.41033676	12.30167305	12.19996238
7.715405939	7.910975505	7.365664505	7.536179778	7.453956636	7.486457414
6.906817121	5.936362556	6.51319704	6.052054152	6.391201182	6.311152586
9.107945202	9.082448343	9.435122859	9.024212859	9.46304199	9.769223525
7.143149899	7.627670859	7.946541949	7.571747222	7.705454838	7.609232899
6.295918879	7.680542242	7.388945192	7.359287172	7.716120232	7.432999727
14.74028573	12.78645268	15.32551587	15.19830883	15.449005	12.82558839
9.544495798	9.505258828	9.503271071	9.650129091	9.619644949	9.891739697
6.286870242	5.969852939	6.207117515	7.174161374	6.119415222	6.354946212
9.295516616	9.623794152	9.864614818	9.696433626	9.592426051	8.788135667
9.093001495	9.022000869	9.46096901	9.421059	9.008956374	9.702758343
13.71423947	14.01682491	14.05855866	13.96779242	13.99495772	13.47327752
7.658839354	7.151031394	7.287262414	7.391952354	7.334908263	7.249962576
8.580231323	9.010430586	8.08537204	8.325717687	8.340681505	8.598479657
7.821971636	7.470329323	7.199434576	7.069165141	7.33379003	8.331458747

10.31289083	10.54800525	10.37573078	10.28710845	10.38430498	10.67815746
8.731576192	9.271123182	9.054860525	8.937936253	9.30770201	8.192996515
8.322806515	8.401710747	8.318250424	8.254082232	8.463063475	8.541125101
11.41778198	12.06964699	11.60680648	12.21630458	11.94979793	10.92190902
8.425403333	8.140128606	8.183602061	8.298004222	8.341133162	8.555429434
7.306206455	7.460591212	6.937364475	7.071197202	7.000099141	7.172125253
7.650858424	7.25519196	9.582761798	8.260123323	8.639872707	7.751645273
6.501046121	7.341641707	6.884558242	7.641985	7.285574899	6.699888758
7.84876403	8.338355444	8.690918616	8.586230848	8.433042727	6.997628374
9.942152202	10.1752482	10.80499832	10.30810853	10.67939021	9.612498475
9.234418071	8.854095293	9.484209455	9.566253768	9.429506515	9.602112616
9.878686616	9.840249348	10.06633871	9.694213162	9.706432808	9.935661677
10.25627793	8.711304737	8.533908051	8.817191758	8.637513768	10.27905981
6.922922101	6.85895202	6.232903485	6.42385696	6.439328828	7.173324505
8.418553323	7.957660747	8.056301667	8.369612525	8.271795141	8.706567556
10.46442497	9.910960495	8.652865111	8.835493283	9.189045596	10.23247836
7.248451727	7.302435273	6.658852677	6.691780525	6.664911182	7.069444
6.542314697	7.363617495	6.245816848	6.494784798	6.578698909	6.950101798
7.475955232	7.772503354	7.46337499	7.551492101	7.529315566	7.16725497
7.335553323	6.856233343	6.774688626	6.840661414	6.737335172	7.066192545
7.521572545	6.58529104	6.868107182	6.824533111	6.722265657	7.18112796
7.453021566	7.773938747	7.989899475	7.791244131	7.835434535	8.565878434
10.66342196	10.25422487	10.54078574	11.09089144	10.46108665	10.58935103
9.910960495	10.15483313	9.802496737	9.719000626	9.584100455	8.967895455
8.542429747	8.137069859	8.647922808	8.000144121	8.29927897	8.435296202
6.707625232	6.35574901	6.154324444	6.206802879	6.276608434	6.654520283
8.289212586	8.348145404	8.35752198	9.011546121	8.197369283	8.019769071
11.31449746	10.75737971	11.02551486	10.97767761	11.15737495	11.16423752
6.913051818	6.47989397	6.694471303	6.697842384	6.669467525	7.080525788
8.398231626	8.885078273	9.02886299	8.805485081	8.745043505	7.918105152
10.55213977	10.43440935	10.5397012	10.48782122	10.46664928	10.71731517
11.81018832	11.53677901	11.50425191	11.78725933	11.64637724	12.0838996
9.206631485	8.82842597	8.219832939	8.735755485	8.57790302	9.456392222
8.514431737	9.184000242	8.233838889	8.581684657	8.549517636	9.117478424
8.115437899	8.576891556	9.412634061	9.326739394	9.081927515	8.960218374
10.3779146	10.5397012	10.71860391	10.65987528	10.64623997	9.493200828
11.53050203	12.17829278	12.90359001	12.24351077	13.07454735	12.08680505
8.64507902	8.476994374	7.428935273	8.00165403	7.719108384	8.950355889
7.914965525	7.824443343	6.680439192	6.973155374	6.903241343	7.842683778
8.417604414	7.847379061	7.51810503	7.924172566	8.001277303	6.732288444
9.736139061	8.011239071	7.904381697	7.737649848	7.662925475	9.890135131
6.194085455	6.446834051	5.994909444	6.071710828	5.958977909	6.451688303
7.609574333	7.00817199	7.072297535	7.007335798	6.881026485	7.451183323
7.534302667	7.705814323	6.941709929	7.201570444	7.123473687	7.388278253
8.852576737	9.049171303	9.002272697	8.892970697	9.107388182	8.959130566
6.829096384	6.890245747	6.604752283	6.761077899	6.609208404	6.518976111
7.347734202	7.33182303	7.169190859	7.401460495	7.525850576	7.462771485
9.846508495	8.99408802	7.982442283	8.375668162	8.908705232	9.448606737
9.545881535	8.685201879	8.032957859	8.973478152	8.91530399	9.072998758
7.763846354	8.35752198	8.524356212	9.045168101	8.446184828	8.918756414
10.75600216	10.69099899	10.88031852	11.24457024	10.73264046	10.72633412
8.832790172	8.997807768	8.671783687	8.467667768	9.018299374	8.895503556
11.24001011	11.10460602	11.1502402	10.82945836	11.07158908	10.62647628
8.99408802	9.260607	9.470173818	9.567720788	8.803935202	9.337187111
12.83827393	12.17513939	12.36759935	12.36759935	11.93486375	12.39348455
7.770819434	6.882244081	7.420173404	6.417796273	7.629989283	7.125696242
7.456765576	7.177746566	7.854325061	7.731201121	7.294284717	6.698080333
5.842189778	7.308145939	6.972319545	7.136095172	6.228764364	6.446595263

7.657544313	7.063754707	7.618887111	7.791885697	7.557268586	7.102414636
9.811898121	9.077100232	9.701165495	9.883591758	9.526200354	10.31480319
10.83790729	10.8947391	10.87379507	10.72516317	10.98627861	11.12454893
8.897177899	7.99823603	7.418256545	7.387654737	7.406826051	8.720627081
8.452737606	8.793874616	9.024212859	8.554528737	8.746311293	8.311348051
9.524763869	10.44392641	9.990505182	9.345614434	10.6362571	9.853774707
7.908308323	7.628690051	7.533663232	7.527697455	7.916712838	8.218887434
7.346859747	7.36694602	7.394027687	7.143674071	7.406158394	8.175968162
6.221214869	5.917761556	6.421175192	6.531968667	6.083662081	6.489070778
9.905108616	9.647013263	9.904237354	10.66587479	9.699589758	10.03492192
8.898231131	9.204345657	9.393934131	9.112697707	9.169326323	7.93437196
8.956407626	9.221294354	9.095762	9.229082869	9.614560172	8.925792808
11.65455063	11.67371856	11.41778198	11.77552743	11.08264222	11.65918316
7.980256929	8.610337293	8.354947869	7.72538904	8.491490828	8.933180404
7.025021455	6.685804808	6.656891394	6.298192394	6.562721354	6.77698199
9.667708556	9.835081424	9.07581704	9.402230293	9.366312899	9.482360616
9.478383576	9.537497323	9.492465394	9.513164303	9.839882283	9.552159131
6.508927202	7.21466303	6.840143455	7.229324455	6.987320737	6.815473091
10.49241362	10.09597734	10.07764109	10.41330878	9.835081424	10.80239406
6.234440616	6.089395283	6.288760242	5.946866202	6.362324414	6.31049204
9.248396293	10.01257663	10.54950911	10.24942024	9.94767803	9.120933788
7.979863071	8.232591606	8.823889374	8.52305899	8.498503737	7.800216455
10.21583282	9.094076404	9.870221061	9.795448596	9.862946677	10.30569114
8.040575909	7.797507939	8.25570701	8.164979273	8.077230061	7.927460424
9.008373061	9.212349869	9.216574283	9.133284677	9.198999222	9.164000111
7.49864798	7.275223848	7.149668323	7.372842667	7.392563535	7.859293
8.158602879	8.962901121	7.621361828	7.808339242	7.728706424	7.844550919
7.398020586	6.546871707	6.411917909	7.511838192	6.176996141	7.063754707
8.254484434	7.656544636	7.61520499	7.722652869	7.620272192	8.101780545
9.465071586	9.318228697	9.484840202	9.339764051	9.318836889	9.247274333
9.954681949	10.57758511	9.957374697	11.04156144	10.07107569	9.176817596
6.362324414	6.539797657	6.590449828	6.297066051	6.714736646	6.580819444
6.862647808	10.42062148	9.546645747	8.888204949	8.400464071	8.923097444
8.648450303	8.57597697	8.494130061	8.43006898	8.812517081	8.875206121
6.622230818	7.140902202	7.070939121	7.168942192	6.986482212	6.160317818
11.30301738	11.53473316	10.86820493	10.70181173	10.75865445	11.6445291
12.22629088	13.17281418	13.7211242	13.29684189	13.16824615	11.18471248
8.076118121	8.815152444	9.051375475	8.942313313	8.983202444	7.256041222
7.706911222	7.85604701	7.850890354	8.098747556	7.834378071	7.431389424
10.50166798	10.61371787	10.34100163	10.48185706	10.47584162	10.19332507
9.491039162	10.04776272	8.946596606	9.155642525	9.289535939	10.69470446
12.73352188	12.99565877	13.05987966	12.77841715	13.10985871	12.68097339
9.811091061	9.70574997	9.955574404	9.637663091	9.881176566	10.03611568
15.16803204	15.54332231	15.31193978	14.85646221	15.65613201	14.63205333
8.239938859	7.951633808	7.110215707	7.38862201	7.300950758	8.403471909
10.65987528	11.03163187	10.86691694	10.79686873	11.12935449	10.2752886
6.453863293	7.382214869	7.207252646	6.369292	7.10439698	6.482923929
8.280707687	8.367422212	8.762202232	8.114219303	9.06333597	7.870669919
6.523375646	7.173570899	6.235213687	6.484888434	6.306085808	5.887120091
8.956909848	8.275152535	8.459227657	8.643157737	8.486053202	8.409959899
8.714332636	8.077645899	8.876650909	8.746767313	8.528049141	8.399553939
8.809951364	8.451947071	7.729067364	7.996041859	7.868007081	8.410410394
6.893606717	6.789988131	6.113305919	6.232553091	6.197177	7.179153636
8.034450929	7.932066141	8.440878707	8.008400859	7.881102323	7.664565879
10.8193093	12.34577038	10.33596201	11.11388317	11.12001247	11.66934136
7.682204525	6.906335848	6.561106071	6.629471747	6.624146566	7.738613697
7.527074182	7.924502232	7.964547263	7.706911222	8.123132566	6.842028788
8.237365566	9.128533354	9.224923687	8.931557869	8.391020323	7.851640596

10.02269473	10.09994749	10.22448985	9.692793657	9.992988556	9.987874111
8.525321657	8.080354404	6.840910293	7.244122677	7.085629475	8.868979273
6.956636515	6.756145354	6.987890152	7.194677455	6.812168303	7.008962394
8.515864333	7.886079293	7.485478828	7.521266808	7.402701172	8.40783399
8.681067818	8.126700889	7.864535646	7.755503707	7.758192424	8.877161475
7.00817199	7.019424707	6.290463121	6.322511848	6.353596172	6.055906899
8.528512253	7.310197869	7.135537212	7.431389424	7.121055596	8.298396535
10.38531322	10.55966675	9.79615097	9.950180667	9.84311402	10.73264046
10.32208546	9.834210131	9.710054737	10.28005063	9.370832535	10.06547375
8.309247939	8.050484323	8.201745222	8.516311424	8.200163707	8.485174677
7.982442283	7.712540141	7.542431869	7.608934747	7.656877838	8.359263212
10.94071069	9.793126455	9.46304199	9.721175788	9.643287657	10.88681216
9.129114848	9.024707707	8.673363869	8.636577313	8.50473503	9.354902374
10.24612484	9.960976253	9.793942798	10.03983393	10.0719806	10.02061657
9.311440606	9.705000525	8.560348606	8.82789604	8.771856424	9.772980061
8.598008343	7.120482	6.780688182	6.923947081	6.71121296	8.274284293
10.95849458	11.8358519	10.88430477	11.54470354	11.42562271	11.03008027
8.12156602	7.872008071	8.448929687	8.100222556	8.007258162	7.858214495
7.500795283	7.198597879	7.356542485	7.466342606	7.275508121	7.741791646
10.63516273	10.28190294	10.3918566	10.50057506	10.59062542	10.47242114
10.06173934	9.736139061	9.625623061	9.819688798	10.12041459	9.747577162
7.814874545	7.528303323	7.612584303	7.983548525	7.504860717	7.778011646
6.727438899	7.021482505	6.276206182	6.528405747	6.580819444	6.574185192
6.282981828	6.472782788	6.24762501	6.391416354	6.314827061	7.147555404
9.009915859	8.917069505	8.634788929	8.509502414	9.062220818	9.424273283
7.801818071	7.436027273	8.312729141	7.911629535	7.812327374	7.581254909
12.89886333	12.05489479	12.19398134	12.62807191	12.16627503	13.72760196
13.5160991	13.3718573	14.010587	13.15082863	13.61616406	13.09463792
8.672348475	8.734806657	9.110297333	8.568242293	8.602859283	8.991970091
7.575754657	7.36510997	7.527697455	7.321884212	7.310482798	7.923778798
11.08756575	11.28053313	12.03602449	11.82616001	11.542568	9.888393747
8.18280501	8.497692788	7.761029182	7.550195808	8.010523545	8.122747707
7.680542242	7.241813919	7.568310444	7.77328201	7.578628212	8.213079374
8.40702104	9.037616626	9.368224626	9.073600626	8.686126354	8.358418384
7.174677505	7.297796364	6.252758313	6.697631071	6.597742293	7.174677505
11.68038318	11.95261936	11.9875501	11.87912023	11.73070023	11.29076429
9.321347081	9.669114061	9.648698737	9.634846707	9.552821717	8.971727333
9.669114061	9.335272182	9.392082222	9.472030465	9.853774707	9.75438504
8.158321101	8.044191424	6.923688879	7.179153636	7.176396859	8.498167798
9.767642525	9.889177586	10.2026692	9.126663535	10.4283734	8.553595747
8.729972586	9.107388182	9.06557301	8.209418434	9.302103889	7.539167263
8.025425616	7.960192616	8.110881556	8.729485838	8.012010707	7.533296202
11.08435313	11.60888892	11.14211434	11.45526933	11.40725584	11.22119175
5.778232444	5.759993172	5.758486051	6.013925687	5.868827747	5.749692929
9.793126455	9.328020364	10.04862183	9.890949131	9.61181703	9.659537192
11.4090051	11.01807842	11.29230964	11.22600179	10.86305205	11.2608846
6.584832545	6.899489646	6.691055667	6.252448444	6.881752616	7.342871717
6.354167	6.489542818	7.013654717	6.74432	6.578936343	6.07088597
9.905938879	9.802496737	8.690381253	9.255548899	9.024707707	10.74172297
7.695611414	7.911995121	7.503972212	7.954533778	7.726392606	8.033283465
10.70775781	10.96000427	9.523397737	10.07857135	9.957374697	10.59809845
10.09506496	10.61621963	10.80091358	10.18291229	10.68687892	10.25519474
10.4700758	10.64005918	10.56207051	10.30151978	10.30810853	11.21958266
7.855388333	8.495447152	8.514037303	8.302900242	8.122341	7.585815263
6.919663687	6.706413566	6.173134414	6.417030697	6.321469101	7.050890818
9.015412101	9.112697707	8.87289497	9.100524758	9.184000242	8.891390919
9.468134182	8.268052495	7.013396273	6.813695162	8.319524414	8.459596535
11.11714857	11.72126504	11.79615897	10.69793218	11.69440164	11.0035084

8.493155899	8.664725051	8.350619919	8.042161798	8.24438497	9.634173323
11.51260306	11.40725584	11.91416468	11.58005536	11.36423723	11.58195332
7.358075697	7.102982434	7.342268626	8.093077828	7.693828071	6.741460808
6.472536212	7.138289616	6.874493707	7.088518818	6.890508141	6.37731698
7.391952354	7.152088687	6.772767788	6.902232848	6.905091242	7.289331586
8.848914293	8.368780919	8.477505576	8.63794701	8.521230828	9.250770152
7.709511535	7.450888848	7.798177384	7.790242404	7.771540485	8.056301667
13.2504616	12.51066587	13.29117716	12.7494647	12.07810634	12.97977545
10.56306707	10.0672481	10.42409385	10.59541023	9.898158111	10.60161194
7.369510384	6.444835424	6.681164444	6.553214606	6.545042596	7.340448859
7.930615646	7.995268899	9.138451657	7.36510997	8.561321323	8.611710212
10.3896003	10.30810853	9.731150899	10.81423156	9.75438504	10.70299608
6.372952485	6.188743172	6.586196364	6.984268657	6.452957253	6.326100737
7.816627253	6.875689354	7.266813283	7.491688212	7.739667424	7.708922879
7.912365798	8.479727162	8.814624313	8.51269104	8.666113404	7.383097848
8.293769586	8.741130788	8.812517081	8.770297859	8.560348606	8.403930677
6.71142402	6.410100141	6.202989697	6.238699505	6.323529697	6.072482869
7.871272152	7.66151798	8.584830636	8.187593091	8.150892606	7.979449616
7.169469697	6.023927495	6.287217818	6.38878402	6.220336424	7.203497747
11.05191968	10.98627861	9.53690398	10.09161001	9.938475404	10.60383217
9.123297364	8.909945455	8.237365566	8.521230828	8.707057788	9.447242061
7.494510091	7.317304515	7.369773828	7.508747081	7.367866364	7.497675424
6.062490404	6.787317758	5.996965293	6.398440768	6.186899475	6.18704099
12.18792926	12.01956714	11.73710477	11.8358519	11.77053658	12.11562205
8.574218515	9.583475303	9.521192909	9.256767414	9.128533354	8.086153313
9.097596465	9.943097475	10.2187154	9.687758929	9.807061475	9.130293909
8.192266657	7.597541737	7.390207394	7.743136949	7.831138545	8.074096707
7.042643192	7.319616859	7.178076949	6.930222384	7.16240796	7.307847101
8.887109626	8.762742172	7.823737283	8.023099172	7.756512818	8.796283929
10.18762619	9.366909111	10.38842688	10.54190997	10.61743608	10.7532413
7.393167162	7.390207394	6.576569869	6.937364475	6.67667401	7.652274172
6.40434199	5.785204192	5.778138111	5.77677902	6.304940444	5.786362172
7.989587677	6.535531162	6.943550111	6.69201996	6.848551889	6.44166202
8.91530399	9.047491869	7.823429646	7.895278253	7.706911222	9.280885222
7.529686949	7.224700556	7.317590788	7.132751192	7.115131646	7.475313929
7.158304313	7.850535525	6.740256788	7.286993737	7.17606597	7.033845343
7.537240869	7.260895414	6.549596111	6.728207192	6.686049697	7.142295949
7.748187364	7.153217667	6.734989303	6.885064545	6.820457899	7.763846354
7.901561475	8.262181081	8.119679141	8.373919657	8.166172404	7.328721848
9.965319232	9.503271071	8.76626204	10.19957169	9.465071586	9.182731869
7.19681397	7.857447394	7.347416111	7.662925475	7.58864003	7.041650374
7.300950758	7.09469803	6.855182313	7.199434576	7.029897788	6.75222999
11.60680648	11.78498903	12.05813886	11.66708917	11.80554878	10.77637516
13.58209631	13.48990673	13.64789782	13.83173935	13.66158872	14.010587
10.0053333	9.289535939	9.149195859	9.349388778	9.376046232	9.655770101
6.525168444	6.220153717	6.151324646	6.308888444	6.182077616	5.946739657
6.298407384	6.301707192	6.638362242	6.436392303	6.524717879	6.10953001
8.77962597	7.664565879	7.093823343	7.188870657	6.961028939	8.426330949
7.861189172	7.600248455	7.945472162	7.704008545	7.736964596	8.607126586
6.918844495	7.338325414	7.921213404	7.910120242	7.942803596	6.407035273
8.885588879	9.240719434	9.801802283	9.569854202	9.477181455	8.639417101
10.42927797	10.0728998	10.56084718	10.49140257	10.41426339	10.84705809
8.292509485	8.845785121	9.160413333	9.012760899	8.872352707	8.205133263
8.898709273	9.421059	9.682548525	9.597513697	9.543825273	8.191573747
13.92894576	13.62106704	14.1282639	13.85099976	13.94443	13.6823979
7.997875909	7.099574394	7.010236465	6.990487535	7.028313949	8.048299333
7.742830394	8.278157657	8.909945455	8.435650677	8.36093298	7.396823121
9.70034896	9.776839404	8.948168879	9.215946081	9.148511889	9.001272616

7.153761556	6.871904646	8.046784596	7.674109172	7.394027687	7.339805162
9.303966081	9.073600626	8.799348343	9.159196141	9.062782333	10.12314196
7.006478434	6.296112455	6.443449727	6.38452097	6.464934909	7.348317141
8.661956242	8.725555596	7.778752535	8.174069929	8.166983455	8.478760515
8.784105778	8.794361798	9.07526898	9.140978061	8.894948323	8.900779434
9.086722374	8.918202434	8.947623838	8.732044667	8.794788636	8.957944414
6.992392121	6.873999848	7.708254091	7.410944101	7.197763283	6.767959131
10.30569114	9.839111	10.29542336	9.876880455	10.12224713	9.924448444
7.880750535	7.508036465	7.595301182	7.705454838	7.560401535	7.463713737
8.551311374	8.588536172	8.704708384	8.612685364	8.801904485	9.217165182
7.837840162	7.652274172	7.841061444	7.840343939	7.820934747	7.90403798
10.81280665	10.80239406	10.52247938	10.6313464	10.73788216	11.2345748
10.42736719	11.21668249	11.19966733	11.02243393	11.00496707	10.25127201
7.794025323	7.696877364	8.242431778	5.904125172	8.263464364	8.115437899
8.425847798	9.06557301	9.200683333	8.937429899	9.022000869	8.029836697
7.555333263	5.822681152	6.992392121	6.084226273	6.973155374	7.693516556
13.83772389	13.60979651	13.48401144	13.98831971	13.67554111	13.92894576
8.221010253	8.675789182	8.460863192	8.57597697	8.647073404	7.706567859
10.21054893	10.12126634	10.03876748	10.11246778	9.57872197	10.14432724
7.78118699	7.896287434	7.834787162	8.028563859	8.075284384	7.459405444
7.339519202	7.385218303	7.57049196	7.302140758	7.337670899	7.711891616
7.434204455	7.32338401	7.814474586	7.785940475	7.482291222	7.277482444
8.301657889	8.634788929	8.706127333	8.546982556	8.738735232	7.91465098
6.900245465	7.012571859	7.087495071	7.018881949	6.913788323	6.595700424
10.1421691	10.83790729	11.05356995	10.74305691	10.83642757	9.377908
15.46469304	14.7528858	13.23864174	12.29275278	14.48025691	15.09418019
10.36239309	10.13958591	7.280239242	7.557888444	9.768390455	10.92880859
7.419865051	6.799149535	6.775232444	6.630903202	6.582439566	7.208855192
7.583998525	7.845601525	7.084144263	7.392563535	7.350114798	7.926300626
8.510413414	8.363862202	8.290353162	8.401317182	8.227270172	8.719668061
9.499917182	9.758431737	10.15379546	9.792275424	9.815893677	9.23383296
10.52247938	10.53028115	10.3025572	10.70299608	10.56908161	10.76857843
7.852302727	8.227270172	7.930919909	7.962004212	7.95565902	8.214678465
9.788274313	9.445197818	9.727935707	9.588958727	9.627013505	9.903372323
7.820222687	7.548584576	7.846688253	7.521572545	7.763846354	7.935468505
8.302900242	9.130293909	9.298556212	9.038261515	8.833963152	7.970233808
8.672881101	9.018299374	9.34289298	9.191263899	8.81572204	8.484723788
12.11216258	11.97660652	11.96059797	12.12151457	12.06397065	12.36473556
8.514909313	8.960218374	8.908935182	8.724013404	8.948168879	7.957660747
11.88909817	11.55928848	11.82852227	11.81439214	11.80104424	11.88649013
8.84786598	8.550897414	8.301264212	8.503061444	8.467263475	8.784105778
8.499404646	7.885002121	7.443557333	7.577325354	7.481645051	8.638952889
9.838413929	9.332795131	9.271123182	9.241421747	9.359207242	9.873591818
9.019731374	9.300983919	9.143918798	9.176146596	8.622644364	9.600888859
7.990313949	8.106019798	8.106418192	8.071334404	7.885002121	7.907197636
11.38391359	10.53141254	10.64005918	11.20163629	10.67428006	11.45738434
8.585836293	8.31049196	8.877161475	8.335861768	8.088956818	8.334162556
8.041000495	8.549141545	8.855784909	8.346585616	8.743124222	8.098747556
9.548618051	9.157435455	9.36570098	9.455015404	9.515743253	9.84311402
8.396125929	8.923576949	9.035586242	8.904793061	9.11093099	7.895961929
8.442251212	7.55499601	8.270601414	8.122341	8.013857071	8.841575667
10.17336172	10.03611568	10.32093584	9.987077505	10.14432724	10.10454015
9.507772687	9.662314131	10.4994288	10.2829717	10.18762619	9.180285111
6.953914061	6.219076051	6.103600394	6.201106192	6.132115071	7.196285434
8.091725323	8.170586434	7.957321232	8.047553323	7.889761727	7.339519202
7.240274404	7.300330596	6.407880727	6.681596343	6.783136778	7.338887687
11.42178201	9.276789111	8.521696414	10.62764398	10.00886723	9.195534384
8.168587596	7.732919727	8.222994222	7.949071818	7.829325051	8.100639

8.660166475	8.714332636	8.586230848	8.768863636	8.752912545	9.034403333
6.734736768	7.06536003	6.607797263	6.585495889	6.614504818	7.397472212
10.66832974	10.42736719	10.77507269	10.57159251	10.90057618	10.04340808
6.467159949	6.231060354	6.814711535	6.855182313	6.279014293	6.465158313
9.584100455	9.524156586	10.03026493	9.895856061	9.775265525	10.18563382
8.775001677	8.363468485	8.753927596	8.708995576	9.135096212	8.570262
10.33375706	10.16743096	9.655088667	9.667708556	9.31393701	9.945028121
8.769354545	8.77298297	8.876209859	8.934248131	8.730448515	8.853585596
9.06333597	8.509502414	9.03221704	8.939630657	9.006527424	9.196689051
6.907839071	7.205804788	7.901917293	7.39712799	7.050890818	6.810582646
7.430156263	6.842776727	7.345267404	7.478803566	7.157499455	7.384903253
14.30038792	14.79546777	14.39736901	14.79546777	14.52085241	13.47832004
9.455015404	9.092457495	8.582147707	8.829061111	8.658285303	9.037616626
9.000069889	8.761177727	8.346585616	8.537956091	8.287081333	9.596773313
8.055566596	8.193820172	8.473392556	8.23139899	8.59942799	8.037984616
8.491490828	8.142577798	7.226157253	7.486746556	7.646953505	8.682574626
10.59809845	10.79564384	10.2166926	10.49348589	10.40278823	10.83501466
7.629344343	7.392836818	7.413101485	7.369172101	8.063898495	7.733842657
8.431326525	8.146343545	8.644542606	8.272620253	8.571676263	8.201303758
8.896578162	8.400903717	8.193820172	8.183195545	8.214678465	8.574674323
8.83948901	9.08614898	9.193817232	9.145664475	8.863375313	8.724013404
6.402255576	7.041096091	6.025991232	6.516723778	6.328529444	7.290204495
8.76328402	8.628208788	8.387083515	8.347717242	8.339115859	8.870602515
10.63391466	9.359207242	8.806957455	8.886620687	8.783158111	10.85100254
8.498949848	8.590695768	8.873520545	8.57971698	7.833640626	8.273483707
6.912275354	7.108492263	7.394691081	7.262118616	7.063477202	6.947784242
9.823678374	9.384078242	10.23247836	9.868690293	9.896649636	9.93663897
9.54312803	9.53690398	8.588536172	8.647073404	8.893968333	8.372086737
16.68963031	16.68963031	16.82803465	17.07010739	16.68963031	16.09492117
9.759214424	10.34100163	10.6426412	10.58812248	10.41846051	9.191263899
8.045722828	7.632188616	8.386601707	7.592388485	8.216823323	8.12949599
7.196057152	7.046726313	6.653328071	6.73278298	6.789537253	6.676905949
6.680656707	6.372952485	6.537080394	6.693002899	6.797589061	7.126269081
8.057063626	8.341133162	8.045722828	8.15355797	8.143040162	7.884680111
8.414282596	8.877161475	9.588410576	9.072998758	9.270407879	7.838173152
10.50543333	10.6488688	10.40701089	10.60863639	10.31597323	10.63516273
6.809486374	8.123844788	6.619037364	7.664957788	6.917762192	7.205488778
10.3986043	10.52689599	11.02403513	10.87518762	10.76983035	10.01622438
10.25038968	9.62845904	9.843921515	10.05688349	9.541041677	9.620873889
10.89912578	11.11081747	9.859664869	10.39968902	10.36589334	10.40178901
6.919392394	7.271701444	7.613240606	7.345589182	7.199742475	6.523815232
10.50794857	11.05651949	10.73911267	10.91641235	11.03580591	9.736139061
7.766006091	7.249695535	6.299210717	6.647920455	6.359165707	8.013857071
7.521266808	7.439837192	6.692548323	6.890761263	6.743803525	7.51061902
7.193299919	6.307709909	6.391634697	6.473835768	6.329915667	7.406826051
7.924172566	7.891912283	7.955993172	7.800624061	7.98690202	8.197004869
7.456429202	6.585495889	6.228051758	6.391201182	6.565610293	7.812003626
8.341977727	8.862785404	8.950355889	8.757866131	9.049629657	7.824090626
7.90331499	8.019434586	8.145617202	8.396963808	8.38301802	8.518658667
5.864284747	5.873729434	6.081802747	6.16631502	6.379794838	6.047425707
10.4283734	9.490338525	9.810255869	9.818059192	9.905108616	10.12799521
7.202978657	7.534885556	7.606990414	7.523206263	7.334394303	7.336756222
7.83046296	7.183932212	6.823384798	6.914843414	6.949608808	8.077645899
8.666113404	9.266794162	9.453123465	9.249034202	9.262561	8.104133364
6.824345162	7.39712799	7.736619182	7.59498599	7.614831859	6.674468929
11.28565422	11.03580591	11.41981305	11.24993851	11.30690717	11.09885051
6.961028939	7.634532263	6.56887297	6.935043828	6.668064172	7.979863071
10.86820493	10.9088749	9.509876273	9.983540212	9.797856576	10.77507269

7.211808657	7.03146702	6.591813727	6.685554869	6.612514242	7.516753121
8.164979273	7.684267263	7.715032394	8.168969465	7.937009	8.109266465
5.927292939	5.765268505	5.776618111	5.749593626	5.894389394	5.92804901
7.966054535	8.802385697	7.52530399	8.009440313	7.924502232	7.761378475
12.47130519	11.94979793	12.68097339	12.42616911	12.41634374	12.2031413
6.778470374	5.801864813	5.840461798	6.024470172	5.903126667	5.956757394
6.023264646	6.045455434	5.883232879	5.756802828	5.879155152	5.833092162
16.82803465	16.8710039	16.64557528	16.90924948	17.01900663	16.57174008
6.717503818	6.864987879	6.942257576	7.003981192	6.874493707	6.113157071
11.29076429	11.18636156	11.00902763	10.88430477	11.11081747	11.92937262
9.618955576	9.168324747	8.165787747	8.578393657	8.186741323	9.000069889
10.09254304	10.58812248	10.18762619	10.59292728	9.609756293	9.626304798
9.746868596	9.573320202	9.025193869	8.864430475	9.229670424	10.29949835
11.29399858	10.42297323	10.76239578	11.16888788	10.97476091	11.12151875
11.109559	10.87518762	10.97322727	11.15666056	11.31661678	10.9660467
7.572856394	7.536878778	7.85268101	7.580594545	7.797844434	8.118879606
8.807484626	8.914132333	8.554528737	8.788135667	8.71485401	8.07651197
10.48539779	10.55333109	10.64738653	10.38430498	10.65634987	10.80644997
9.84311402	9.459664354	9.200042253	9.46096901	9.226128374	10.19418334
8.57790302	8.346989566	8.195856758	7.97753898	8.412956141	8.846850111
7.634916323	7.925642	8.272620253	8.08112003	7.895278253	7.180020091
9.62302801	9.719830172	9.541747455	9.890135131	10.12909315	9.909202788
6.981850818	6.84607197	7.330546788	7.50339202	7.085333222	6.823157384
9.271759586	9.324974768	8.793874616	9.106199707	8.759332636	9.306330646
9.920430606	10.24942024	9.446464909	9.666877556	9.972781707	10.1838089
9.707282667	10.46232362	10.5431016	10.29324401	10.54441943	9.167758485
7.171589687	7.339519202	7.240274404	7.227000899	7.291982576	6.964119424
11.40145091	11.86125412	11.84027237	11.69891546	11.91912022	11.33477589
6.710946737	7.504269545	6.271221162	6.382999071	6.413017273	8.10140802
13.52959744	13.15082863	12.7452022	12.87421343	13.20018245	14.02572128
12.88265743	12.90359001	12.72972704	12.82558839	13.13007894	13.12562627
13.44126502	13.85099976	13.89331098	13.65450155	13.96779242	13.29684189
10.32750324	10.80900786	11.80791686	10.94496034	11.65261158	9.138451657
7.12322297	6.697631071	6.896472707	6.542971677	7.094997242	7.315524667
9.376046232	7.651593505	6.828400424	7.683318778	7.427086202	9.618955576
9.661659101	9.701165495	8.914132333	9.433849505	9.597513697	9.746868596
8.543234566	8.496814303	8.938439909	8.227270172	8.600356081	8.634788929
10.73517618	10.71031814	10.70052069	10.83501466	10.85225864	10.60277882
9.045755677	7.874063444	7.796747616	7.923423949	8.08972896	8.975838535
9.926784556	10.38430498	10.85225864	10.61001127	10.40178901	8.476085384
6.340472131	6.416313212	6.922922101	6.743041313	7.069165141	6.792458242
9.924448444	10.62895885	10.93334198	10.62647628	10.66342196	9.462343535
10.86963124	10.72127155	11.58421758	10.9889639	11.24323027	11.72592113
8.311348051	7.985458788	8.246470545	8.295009141	8.493653778	8.937429899
16.73234105	16.4534382	16.52993947	16.73234105	16.73234105	16.68963031
10.11051676	9.806332162	9.84311402	7.157774636	10.20964764	10.8605042
11.00754401	10.63516273	10.40592026	10.66342196	10.75987116	11.00620373
7.929187444	8.123471404	7.655304091	7.810269677	7.91307104	8.496365737
7.653651778	7.98690202	8.200961636	8.124973859	7.558798354	7.65784001
10.38208186	9.918671212	9.505559106	9.886023253	9.747577162	10.98492733
7.463713737	7.566773798	7.770465818	7.520648323	7.681826051	7.277849899
7.58864003	7.442598384	7.608041616	7.558798354	7.351279444	7.573484121
12.85048632	12.77454263	13.43523914	13.02518615	13.00581854	13.35359128
10.96311364	11.30301738	10.67428006	10.73661507	10.97054498	10.81664077
11.35080354	11.89130603	11.59427705	11.74670945	11.911688	11.308834
14.02572128	14.05136004	13.95955505	13.88591407	14.13521971	14.15863941
8.636577313	8.936938576	9.016491202	8.733083212	8.944409727	8.66101001
10.40592026	10.89604789	10.68452674	10.51650912	10.81142627	10.3810842

8.11047698	7.984288364	8.058246657	8.163923192	8.092218303	8.127056495
11.82616001	11.29597305	10.53737043	10.25627793	10.06255975	11.70348036
6.761335818	7.122424869	7.34804498	7.095534354	6.79370899	6.339077535
6.809240364	6.515007141	6.176996141	5.948947566	6.801564111	5.972872515
8.575566677	7.158855657	6.317463354	6.416564263	6.520559131	7.135222657
7.041369354	7.024771818	7.035420475	7.005354859	7.219933525	6.95944596
8.003147788	7.328721848	7.888681737	8.035675313	7.800624061	8.096910323
9.07581704	8.39227803	8.552719747	9.012031232	8.532119949	8.917069505
13.92172837	14.010587	13.27940818	13.62784329	13.40990013	13.3718573
8.931557869	8.814624313	8.935320828	9.119222505	9.017695354	9.080123545
9.217165182	9.254253192	8.674281929	8.64224801	8.735287485	9.185002364
9.648698737	9.791460222	9.608350808	9.855298394	9.748306121	10.04963797
8.818695101	8.606099333	7.534885556	7.872350636	7.767042424	8.792195697
12.03068847	11.76336308	12.35168631	11.75215821	12.18792926	12.12692782
7.426803586	8.099825778	6.946711626	7.144887111	6.943550111	7.953126222
7.132449434	6.996297444	6.935569505	7.235967596	6.997069465	7.723009
7.100441616	7.463067838	7.712213273	7.563439404	7.614497747	6.577521747
10.90754603	11.07158908	11.49420288	11.38178745	11.26815786	11.44491584
10.56207051	10.93646713	11.1060681	10.87379507	10.9088749	10.18942995
8.957944414	8.616405606	9.325601172	9.004398101	9.421700798	8.522641273
10.59685822	11.18471248	9.967178364	10.48659409	10.9991534	9.841562192
9.53690398	9.710054737	10.0188097	9.76546198	9.165150051	9.503271071
9.280305798	9.223885747	8.419470808	8.628208788	8.507703202	8.687969051
13.54965001	13.96779242	13.5160991	13.92172837	13.46712151	11.99535117
6.660795162	6.627087081	8.057864374	6.950382242	6.931701566	6.375910899
8.51269104	8.683580283	8.449337667	8.170129495	8.637033606	9.187244566
8.101068051	8.977005354	8.268052495	8.416773909	8.300483646	7.984708455
8.268937657	8.042974687	7.094997242	7.291068525	7.55847901	8.221010253
10.71390019	11.32376318	11.51647662	11.27153548	11.46936418	10.42175216
6.417030697	6.140862859	6.026135475	5.945188566	5.896263404	6.168988222
7.591105343	7.747885899	7.308145939	7.461509737	7.364257101	7.173022687
7.943134	8.066374202	8.00165403	8.579336081	7.914965525	8.228510596
8.796713535	8.533396525	8.57790302	8.072110051	8.226840717	9.684048687
7.177458596	7.095808323	7.569899606	6.977291071	7.143674071	7.372238061
13.24400831	12.43944324	11.56988377	12.18142186	11.91416468	13.64083669
8.36093298	7.945111576	8.014979636	8.010919919	8.169382444	7.775630727
8.452374444	8.356302828	8.51269104	8.443547162	8.422643323	8.354182626
8.463578343	8.143040162	8.171788646	7.483852838	8.710842768	8.43615796
8.996667384	8.773988747	9.226128374	9.118059475	9.450568111	9.10215096
6.521696273	6.525168444	6.09086501	6.265724232	6.204530263	6.762066263
6.713056273	6.868352737	7.522869182	6.993463051	6.827921778	7.163563677
12.57877126	11.08936677	9.945028121	11.17041501	10.19418334	12.04160066
8.124212202	7.689572788	7.553773697	7.625433667	8.24281297	7.524103909
7.030955384	6.942257576	6.469635131	6.513874333	6.414872495	6.240475939
8.826846768	8.781156657	8.150096222	8.284876758	8.265565879	8.366003535
9.085558838	9.752732475	10.00262223	9.526870273	9.727055768	8.85049999
9.214694788	9.068490586	9.120933788	9.580783667	9.505258828	9.436357141
6.935043828	7.106035242	6.709545626	6.762066263	6.708053838	6.529524889
8.418959303	7.660150727	7.837840162	8.355847778	8.11773804	8.185563303
7.955335374	7.374297343	7.759566061	7.078018687	7.446082798	8.492322828
7.487647384	7.372842667	7.311327071	7.475616394	7.697940283	7.347416111
8.802385697	8.493155899	8.528915121	8.491049758	8.755421576	9.301561
5.944857354	6.054005586	7.442271202	7.057330242	6.714999636	6.484447556
10.71860391	10.41530743	11.03163187	10.92757694	10.96748398	10.73132595
10.59180142	10.70181173	11.32376318	10.85782556	10.67815746	10.23452156
7.965660091	7.68569196	7.03599896	7.207252646	7.151822525	8.234232515
12.50706636	11.35080354	10.82804231	10.82804231	10.72900488	12.30465105
12.7494647	12.49965499	10.87518762	11.5386618	11.17213305	12.01673563

6.60386102	6.731476051	6.530610939	6.521458212	6.618022394	6.94985404
14.9591327	15.31193978	14.72870348	14.83117585	14.86987024	14.70594079
9.524156586	9.567720788	9.379180747	9.545191596	9.636296737	9.321890899
9.256085253	8.949261586	9.026636737	9.158023	9.250125475	9.391460545
9.596773313	9.574050242	9.515069697	9.587719636	9.638947465	9.70574997
8.853585596	8.479185606	8.685647889	8.450171172	8.820759798	9.106823152
10.09597734	9.76546198	9.783680727	9.922126404	9.963504081	10.04421951
10.3305428	10.33146686	9.532335293	9.858738737	9.927686616	10.90754603
10.97322727	10.99345288	11.03580591	10.3548828	11.11856721	11.75908761
6.928572354	6.599831253	7.357456071	6.981086404	6.818722313	7.000811505
6.57287004	5.923322192	6.154670293	6.337620818	6.434266404	6.375508101
9.415860727	9.320738646	8.589797808	8.938439909	8.299695162	9.050797061
6.87215099	6.862647808	7.326042475	7.101832475	7.488273475	7.219623727
8.734416606	8.961327374	9.167114	9.262561	8.918202434	8.981479273
8.25927404	8.151663141	7.976116929	8.111336758	7.798826303	7.684641212
7.676615465	7.395300939	7.154007354	7.864535646	7.018669091	7.171589687
6.871904646	7.246699182	7.314300525	7.979863071	7.421647414	7.108809091
8.05903404	7.729749768	7.037248667	7.312252182	7.291068525	8.011239071
7.95565902	8.832790172	8.739635162	8.507703202	8.709941838	7.643273222
8.48873997	8.226500707	8.057063626	8.293769586	8.108111747	8.169730152
14.23242051	13.63435472	13.78027417	13.83772389	13.75520609	14.32804886
10.0143126	10.36465285	10.29851595	10.17336172	10.00094823	9.58708204
12.53197264	12.26162026	12.33273925	12.052647	12.7452022	12.93430142
10.22164118	9.562994313	9.574750414	9.679645121	9.800083455	10.31693391
10.55602586	10.16937171	9.641787333	9.865550636	9.715870253	10.45754412
11.21668249	10.94071069	11.06877356	11.11856721	11.02700786	11.80104424
10.44094498	10.67572435	10.96000427	11.13240418	10.79284767	9.874496818
7.504860717	7.543396525	7.051685909	7.129597273	6.96619404	7.275508121
10.63752253	8.878201818	8.529888505	8.617840212	8.373919657	10.61621963
10.56084718	10.20859114	10.47584162	10.55966675	10.44832821	10.48897537
7.253764222	7.392218323	6.609763515	6.773240303	6.79984598	7.269598859
9.528804131	9.858738737	9.445197818	9.328711051	9.962571606	9.148511889
11.71659127	11.21187068	10.97631922	11.21493335	11.27153548	11.48061491
6.318299141	6.500408909	8.025051697	6.276409495	6.51170598	6.502291131
8.822853162	8.269395232	7.991026727	8.181963182	8.072924455	9.208368677
7.584965596	7.482605354	7.036743576	7.147201253	7.08777197	7.501777556
8.959130566	8.752460404	8.984231152	9.331038253	8.86638896	8.77298297
11.82852227	11.18941159	11.14057221	11.64848719	11.38775416	11.63837311
13.3718573	13.46005067	13.09045797	13.03496614	13.03025597	13.29117716
7.875022121	9.395177889	8.095330333	8.975268424	8.420871687	8.158602879
16.64557528	16.64557528	15.65613201	16.36806566	15.86132141	16.52993947
6.792957485	7.000584848	6.339262212	6.597742293	6.38666303	6.097511051
7.238133434	7.675167556	7.881467646	7.90609097	7.651926253	7.091328697
9.919491525	10.44195016	10.13958591	10.14713571	10.21583282	7.967920667
6.896472707	6.826879909	6.542971677	6.603384646	6.551008899	6.737600111
7.495424616	6.987033364	7.807594768	6.952808172	7.70299303	7.272016424
6.547549889	6.302320576	7.403336687	6.465574545	7.078018687	6.639490384
10.72633412	10.67081044	10.71390019	10.85513517	10.57028524	10.56655729
7.899682051	8.240375404	8.342773172	8.390560192	8.445759657	7.226157253
10.76857843	10.67305197	10.43440935	10.65871503	10.88945836	11.02700786
8.253639283	8.411285283	8.833337414	7.678291495	7.904747051	8.396963808
8.263866778	8.429130081	8.131862263	8.200539131	8.087781242	8.25927404
8.375190152	8.889380424	8.68840998	8.881793333	8.824381182	8.260123323
9.388912091	9.139724848	9.447991384	9.379180747	9.749004051	9.886762101
9.292435667	9.310775172	8.47014499	8.891888576	9.127251283	9.66831603
8.265765712	7.160551111	6.891782758	6.893369586	6.94072599	7.920144808
9.885156737	9.513795939	8.254484434	8.876650909	8.529417101	10.35069541
12.83405786	12.39058663	11.2786408	11.71443529	11.68484165	13.02006671

7.470329323	6.846305687	6.495899485	6.68968302	6.516492273	7.221626808
8.749910828	9.62024901	9.270407879	8.681067818	8.540202646	9.219562515
7.063222061	6.61686503	6.997069465	6.729176758	7.20918101	7.252886707
9.822898081	9.633493677	10.05780386	10.10946026	9.767642525	9.131560131
6.988716071	7.207790596	7.144029273	7.164949697	6.904353566	6.674020838
7.304975899	9.187526591	7.821971636	8.946596606	8.171788646	8.028563859
8.098747556	9.154469939	9.519820636	8.701109788	8.647922808	7.893273434
6.98921798	5.975569111	7.615524929	6.169649212	6.34170502	7.744459273
7.208855192	7.170797768	7.083314707	7.410002717	7.196550485	7.669946162
8.814624313	8.57070398	9.35858599	9.051957646	8.941359636	8.673363869
7.996041859	7.721580051	7.916025434	7.787863889	7.619556354	8.108917222
8.312729141	8.246087697	8.31008898	8.026271202	7.819838162	8.726024485
8.59026096	8.568242293	7.589888475	7.85299496	7.892277636	8.445297313
9.583475303	9.279768394	9.645545465	9.62024901	9.456392222	9.245932475
12.70787607	12.43279956	12.52461444	12.72219022	12.68097339	12.15752365
9.186711646	8.767368071	8.640789455	9.139095455	8.66101001	9.321347081
8.880364303	9.340550081	9.853774707	9.412634061	9.517013525	9.007843273
9.818860343	9.519820636	9.108564253	9.186711646	8.942756889	9.727935707
10.83642757	10.74172297	11.54871614	11.34892675	10.93935201	10.80499832
6.975426091	6.788535525	6.692266071	6.964119424	7.091328697	7.063984253
8.133538364	8.039790061	7.524403394	7.409045616	7.466342606	7.850535525
10.96162926	10.81540317	11.09885051	11.09885051	10.97767761	10.95374716
6.371622788	6.181117949	6.569606636	6.625743253	6.445241798	6.418654788
6.04413602	5.904609677	5.987835697	5.833912	5.829948566	5.850621525
6.417620515	6.405265273	6.268423737	6.29488699	6.212887111	6.172106293
7.619933919	7.257525758	7.813759899	7.776293606	7.733228788	7.060512141
9.531623293	9.216574283	9.385837242	9.488337717	9.261851101	9.785983535
7.626470505	8.217667525	8.129874121	8.265130232	7.820222687	7.712213273
7.161069364	6.59116396	6.872396859	7.200097273	7.110528152	7.218710525
8.541125101	8.054318061	8.872352707	8.508645727	8.871209182	9.090721626
7.787525596	7.107957081	7.550195808	7.762451808	7.400864778	7.870356606
14.4185289	14.21449649	14.07245549	14.15863941	14.29173122	14.83117585
9.228437333	8.93638097	7.728706424	8.037664566	7.786626808	8.065153556
8.85785699	7.983548525	7.482605354	7.651926253	7.527339687	8.885588879
9.27419499	9.08959502	9.451212687	9.027136657	9.669114061	9.709426838
7.711178283	7.315524667	7.138578667	7.583998525	7.736619182	7.844877657
7.382821899	7.196550485	6.710071909	7.03650198	6.848831242	6.728691949
8.788135667	8.134366646	8.559975162	8.410908071	8.99408802	9.478383576
10.07956055	10.23948175	10.07107569	10.15186663	10.14326599	9.484840202
9.94767803	9.827596687	8.519178475	9.023071899	8.903602263	9.400327152
10.14326599	9.894213939	9.979092404	10.10542249	10.21375666	10.05236285
12.18492446	12.24649354	11.92937262	12.26775966	11.98440811	12.06098883
7.249375465	7.553468394	7.726392606	7.749943687	7.671593667	6.668735424
7.233650202	7.805761556	6.893606717	6.914278354	6.982909788	6.662501485
9.476588061	9.253086273	8.682101313	8.943861384	8.867918475	9.629101929
7.193876586	7.389870293	6.909921869	7.962680434	7.295980475	7.208062909
8.754441596	8.960773354	8.76328402	8.893968333	8.789683394	8.727528626
9.75438504	9.569854202	9.571117879	9.848945263	9.86223396	9.699589758
8.581684657	8.495994869	8.447526838	8.734416606	8.228066545	8.639872707
14.5513462	14.15863941	14.16629104	13.99495772	14.39736901	15.32551587
8.81966502	8.762202232	7.951633808	8.260520646	8.344860929	8.388388636
10.58469265	10.48782122	9.885156737	9.993897848	10.60743282	11.27306561
8.051256737	8.053619525	8.140128606	8.05474504	8.121954505	7.656877838
10.76471511	10.90754603	11.46320141	11.49239347	11.33657706	9.833438364
7.054536222	6.630903202	6.708308495	7.01152302	6.594580697	6.826151202
9.439397061	9.300367869	10.14809211	8.756396081	9.891739697	9.002272697
12.23505088	12.8293171	11.69658017	11.90152869	11.84736958	11.77802923
7.68296396	6.900245465	7.460591212	8.079553929	8.107270071	7.309928404

10.71503879	10.54692936	11.238428	10.0079343	9.780583495	10.6426412
6.92164503	7.709201212	7.372842667	7.808676939	7.307022919	7.741148566
16.4534382	15.95256028	16.22759729	16.61084374	16.02065153	16.04217659
11.15666056	10.41626133	10.92337564	10.92337564	10.3548828	11.51647662
8.294235768	8.905887879	8.755916071	8.843148071	8.900236364	7.877119465
7.161889071	7.398977253	8.118473111	7.902229798	7.761767192	7.069165141
6.542523919	7.654256818	6.733232384	7.011288768	6.710494808	6.505895828
12.37416009	13.2504616	13.9071606	13.46712151	13.73439002	12.27773509
6.771393131	8.486543404	7.112898869	8.039790061	7.064538818	7.48289603
6.945626949	7.374842788	6.792957485	7.119927869	6.635568162	6.954672071
7.265354444	8.771856424	7.08018898	7.649926505	6.70156496	8.114611232
6.20351898	7.498029465	6.414872495	7.020439899	6.449932949	6.628556657
10.6820228	10.78551178	10.55602586	10.84568098	10.70775781	10.20634509
7.898974697	7.777661384	7.819446808	7.881467646	8.04259702	8.233421293
8.871686232	8.571194222	8.002717273	8.325316889	8.173308798	9.441234879
7.797507939	7.896955242	7.035145869	7.093254121	7.337386909	6.881752616
9.231349182	9.798623747	10.25127201	9.716648152	9.845595727	8.894948323
10.44195016	11.18800276	11.52052132	11.0859227	11.25898056	9.251906182
7.707585606	6.569345798	6.385183717	6.825072616	6.789988131	8.068200283
8.906407303	9.769994879	10.49140257	9.930141081	9.18155302	8.903025495
9.315088131	8.961854929	7.563439404	7.998937818	7.958393384	9.012760899
7.487940131	7.468171747	6.922161646	7.105750636	6.936612586	7.326942212
9.602112616	9.90675497	10.39296715	10.19136651	10.05426836	9.124449354
8.395713444	8.198599495	9.303966081	8.861728919	8.614861051	8.642670768
12.96241124	13.54221797	11.86922315	12.65472241	12.71520416	13.37822002
6.579152939	6.640705545	6.402881788	6.57287004	6.524488081	6.467159949
11.13400706	10.17440526	10.50677257	10.81142627	10.1250404	10.84844593
8.758364879	7.816902374	8.016751283	8.002011657	8.17219503	8.432150758
7.093823343	7.127674667	7.229587778	6.929106091	6.846509828	6.795634141
12.86955734	13.09977851	13.23341018	12.86086109	12.68484185	12.71142619
10.6313464	10.81034167	11.31053596	11.04464523	10.94786936	10.32309273
9.782133848	10.01340346	9.687048303	9.404880263	9.863774293	9.353057535
9.35858599	8.817191758	9.276189121	8.265565879	8.152443121	9.741357455
6.831376636	7.175214949	8.161033919	7.44169298	7.585232141	7.002156515
8.582147707	8.922082808	9.296718121	9.107388182	8.746767313	8.113343889
7.454874889	7.826151263	6.652192667	7.246997929	7.165538758	6.748092424
7.279114576	6.679485202	6.353400323	6.351456879	6.375288505	7.454874889
11.20498985	11.06877356	10.59418919	11.18941159	10.66465811	11.65698449
8.468418253	8.063008152	8.349393212	8.408718253	8.088528121	8.72650097
8.687053253	8.045722828	8.718769576	8.879851283	8.344098929	7.828991111
9.417037424	9.071813909	8.885588879	8.852576737	9.044046343	9.177504333
8.60560303	8.264694495	8.107270071	8.152087687	8.52575703	8.546056747
11.58195332	11.69891546	11.97944932	12.06964699	11.94234713	11.24147284
7.909031556	7.413750677	6.986482212	7.361082222	6.973893828	7.346567394
12.81735602	11.31661678	12.28369472	12.84627851	12.58957491	12.26775966
10.91766747	10.98350754	10.87092264	10.9660467	11.08936677	10.44832821
11.06261544	10.97631922	10.99046505	11.02403513	11.04030995	10.68960982
8.100222556	7.964914919	7.874063444	7.950135394	7.92597801	7.774939586
6.788765101	6.334611455	6.993463051	6.415480919	6.534012141	6.643553717
10.24139873	10.07764109	9.078331919	9.395854121	9.26122399	10.56802411
8.33024502	7.846324646	7.929593313	8.431738505	8.156410909	7.561051303
9.051375475	9.444057586	9.286739242	9.347406172	9.417037424	9.123926859
14.80749806	15.29549959	14.58095582	15.18392271	15.21350605	13.83772389
13.13522491	12.87421343	13.07947925	12.7452022	12.88708268	13.08418191
10.22668357	10.01974654	10.72516317	10.67190595	10.60863639	9.713188212
7.275223848	6.925244263	7.073657889	7.409335747	7.192707293	7.610260323
6.112073394	6.700323747	6.638550414	6.55251301	6.526955566	6.513004101
7.723687384	7.593664717	6.905316545	7.062438556	7.205228253	8.132293172

6.900739596	6.968393455	6.786546495	6.889990505	6.791503909	6.232724131
10.52364746	9.702015293	8.383429707	9.137373131	8.842109	10.54565144
8.248986687	8.724013404	7.457632404	8.171342424	8.119288848	7.896287434
10.41530743	10.90192126	9.542425758	10.06450841	10.16454544	9.435722162
6.833358384	6.859929394	6.605442354	6.726910778	6.676225182	6.443018677
8.131433848	7.425828556	7.170490939	7.218710525	7.180291929	7.966402293
7.860366808	6.537747374	6.356904333	6.544097556	6.354564141	6.836068354
6.320079859	6.28101901	7.229904545	6.722042727	6.792041707	6.442315303
8.671294253	9.087255717	8.385303111	8.066048727	8.297224525	8.265130232
9.095762	8.877626323	9.381544081	8.783158111	9.288419414	9.249612495
9.326238727	9.768390455	10.21054893	9.775265525	10.06173934	10.12610261
6.857008404	6.704194818	7.166949475	6.879719566	6.920666222	6.498851707
6.545751121	6.437651364	6.295493455	6.271385515	6.303106697	6.902482606
8.230588212	8.010523545	7.906425364	7.637927172	7.415013667	8.29291099
6.414426394	6.366826091	6.090583616	6.14867199	6.045455434	6.756145354
11.06123261	10.50794857	10.43655428	10.71279176	10.99763472	11.72126504
8.436995596	9.519086535	10.20452777	9.617464455	9.583475303	8.34982299
11.14211434	11.32027501	10.41731261	11.1031408	10.84844593	10.75737971
8.930963202	9.008373061	9.171801828	9.108564253	9.07581704	8.875711838
8.838495424	9.413942061	9.766921414	9.195534384	9.316319051	7.944058707
8.870602515	8.869521111	9.592426051	9.503271071	9.514496606	8.641737071
8.550897414	8.880364303	8.879330566	8.854095293	8.639417101	7.976116929
6.625965465	6.218567293	6.899979889	6.82246696	6.501455758	6.160939939
7.161605929	7.460903263	7.642932737	7.36203996	7.235668455	6.995294596
7.904381697	7.616552222	7.277222202	7.743466333	7.241813919	7.81867298
8.661508737	8.449337667	8.593101434	8.495994869	8.600831152	8.651452677
6.338442758	6.358706071	6.366139859	6.288951848	6.528405747	5.763697626
9.11154599	9.419654929	9.774477667	9.732604394	9.451212687	7.914965525
8.227270172	8.951989212	8.824381182	8.960218374	8.780092131	8.798818646
10.11349335	9.457741556	9.925226333	10.01974654	10.07857135	10.27035429
7.800216455	7.690840778	7.262654505	7.188612899	6.987033364	7.895278253
8.609952768	9.761765848	7.629989283	11.58195332	10.67081044	8.085771838
9.020821455	8.580722586	8.926362727	9.295516616	9.149195859	9.721175788
10.92880859	11.43005301	10.14131493	10.97322727	10.61867663	10.67190595
6.817799576	6.552305929	6.766746646	6.513435323	6.563630475	6.439547263
10.89755671	10.86963124	9.706432808	10.26541026	10.20634509	10.67572435
8.706127333	9.077686808	8.863920758	9.050797061	8.901331343	8.288355808
6.967928273	7.08018898	6.826151202	6.901464747	6.791252535	6.56887297
6.941709929	7.937437293	7.275765808	7.047335818	7.142295949	6.605935465
10.98350754	10.66465811	9.444057586	9.917863333	9.736139061	10.98627861
7.013931768	7.331138333	6.395369727	6.60058796	6.630216838	7.553773697
9.463745455	9.055478465	7.556918475	7.905419798	7.538502939	9.730279475
6.694222899	7.149903152	6.32497801	6.399225919	6.482726162	6.925244263
8.836542848	9.214111545	9.244930505	9.124970051	8.865915939	8.27903296
9.247274333	8.82789604	9.439917707	9.228437333	9.225515485	9.460382818
7.845981061	8.40702104	7.521885657	7.56409603	7.563439404	8.300483646
13.72760196	13.82538175	14.5513462	14.34357305	14.11997744	13.30255942
10.67190595	9.927686616	10.09994749	10.97631922	10.687913	10.92337564
6.238895919	6.239756909	6.79370899	6.585042414	6.337204768	6.312971121
12.9709957	12.92542832	13.34204398	12.96662998	12.64265677	13.12023156
8.30620801	8.21550303	8.64224801	8.138519566	8.653906556	8.374810828
7.945864808	6.718256293	6.573293212	6.780426	6.64233597	7.810269677
6.96434804	7.137721343	6.746600626	6.935569505	6.856475414	6.644710828
9.249034202	8.203151141	7.786298434	7.946541949	8.179581869	9.562364657
8.116577384	7.622357354	7.304125475	7.433916687	7.437384141	7.907575465
10.33989796	9.712341424	10.20859114	10.41846051	10.1905069	10.46342714
7.401750182	6.956131545	6.395106727	6.612755929	6.499057505	7.095275111
7.138031798	8.048299333	6.974196465	7.017519192	6.836284939	8.499404646

11.18471248	11.05356995	11.36979611	11.02700786	10.95533553	11.00754401
14.48025691	14.47046648	14.5413574	14.38889659	14.53168901	14.61202705
9.449254061	9.70034896	10.35809493	10.24612484	10.38001468	10.06904603
6.920666222	7.279952616	7.707585606	7.425506818	7.310197869	6.734736768
7.482291222	7.730484909	7.781509414	7.789255535	7.766357737	6.812168303
8.108111747	8.297630758	8.316081222	8.500240172	8.405727848	8.820759798
6.537260646	6.580572354	7.02555202	6.842028788	6.834332747	6.070726232
7.944736172	7.916391202	7.065642788	7.320933798	7.156966303	7.975414556
7.415948364	6.391201182	6.431584121	6.390557101	6.358295333	7.538502939
7.073657889	6.798121798	6.69350096	6.722758141	6.643746616	6.71730499
9.859664869	10.34193651	10.36687712	10.31795252	10.58935103	9.320738646
8.043387838	8.004282899	7.293153485	7.883536596	8.265130232	8.355847778
8.063461202	8.395713444	9.235747242	8.746311293	8.955359253	8.270601414
7.344013535	7.841061444	6.821005303	7.230210646	7.132449434	7.203245626
6.452957253	6.650544455	7.045230232	7.106592788	6.668510737	6.09363498
13.05987966	12.68097339	13.73439002	13.60979651	13.18858184	12.45390404
11.48459908	11.89628941	12.11562205	11.79870316	11.59021288	10.86691694
9.727055768	9.127251283	9.436898869	9.948421707	9.758431737	10.29432836
12.49170848	11.90384839	11.56291631	12.15130062	11.76336308	12.61761735
7.659822475	7.566450929	7.054536222	7.051418091	6.961758566	7.755825222
6.725730828	6.017403131	6.265013455	6.287983818	6.543419131	6.736375424
8.521230828	7.276013455	7.163563677	7.441392919	7.251486172	8.954798808
7.229904545	5.974334111	6.330318636	6.178323768	6.012730253	6.252603323
13.56886427	12.83827393	12.22629088	12.53911948	12.14191765	13.68884286
8.443175586	8.081502051	8.204380404	7.980256929	8.33273403	9.149684939
5.867624293	5.757052172	5.834652768	5.961236343	5.840144535	5.760733545
9.250770152	9.699589758	10.02821235	9.943097475	9.882779162	8.670447949
7.184502222	6.115027343	6.174156556	6.399678273	6.638128919	7.21466303
7.769155828	7.301506111	6.527644212	6.779669273	6.720289051	8.001277303
12.0089388	11.24001011	11.61814895	11.74439188	11.52448673	11.71659127
7.127947263	7.453021566	7.745118444	7.729067364	7.537817576	6.958952465
8.369612525	8.108917222	7.736260545	8.314030071	7.890421556	8.952561111
7.532708636	7.094450071	7.319114313	7.634532263	7.478150263	7.318843495
6.758395253	6.790809081	7.592047838	6.988716071	7.212087616	6.851905687
8.373157505	8.281167657	8.677707162	8.919862162	8.722953263	8.265565879
14.93426708	15.27762024	15.02501841	15.12351424	14.72870348	14.62217632
11.62494396	12.23191382	12.31089415	12.12939166	12.28105924	10.95849458
8.466026899	9.425521616	9.055478465	8.885078273	9.161459798	8.730448515
9.689129545	9.969958172	9.785178838	9.744663444	9.965319232	10.05523038
6.02502097	6.100439616	6.306878242	6.186015354	6.416313212	6.071710828
10.1250404	10.26832521	9.994772242	9.961780586	10.53141254	9.996420616
8.638487899	8.314030071	8.381754576	8.456593071	8.392711081	8.106019798
7.350251869	6.518759273	6.429848434	6.475742909	6.443018677	7.888681737
6.209590788	7.091067131	6.27717298	6.75400198	6.715644404	6.317094394
7.844877657	8.352944343	8.251492909	8.316081222	8.242002535	7.371907727
9.373397364	8.978723121	9.077686808	8.985821889	9.247858869	9.368224626
7.590248525	7.260583141	7.207500556	7.496684051	7.598252121	6.866597293
8.877161475	9.023639636	9.020821455	9.094076404	9.067856838	9.52752797
11.2786408	11.2608846	11.95261936	11.92937262	11.46320141	12.04432
8.947623838	8.302900242	8.747331152	9.180285111	9.041236566	9.01707996
9.296131263	9.391460545	9.609027141	9.001272616	9.230202616	9.393335687
11.80791686	11.80342824	12.13867659	11.97660652	11.63837311	12.25844332
11.4535969	11.83072395	10.71992703	11.37131374	11.13713447	11.55106122
14.78379092	14.32804886	14.70594079	14.9591327	14.37933382	14.78379092
6.177840061	6.661815061	6.471263556	6.405870152	6.073749475	6.543222263
7.866306808	7.693213707	7.948408364	7.815915242	8.473802646	8.927565404
7.736619182	8.498949848	8.522641273	8.068200283	8.381309202	7.30556202
11.66934136	11.66934136	11.63174178	11.73271713	12.06098883	11.93178106

9.87177298	10.24727703	8.941795242	9.391460545	9.211184727	9.34289298
9.800083455	9.455015404	9.584803364	9.653591273	9.547339828	9.973623707
7.842052606	7.835788293	7.060219081	7.165793091	6.909691071	7.186327495
10.02439619	8.781680606	8.412578899	8.935320828	8.613985131	9.994772242
6.763620737	6.860671293	6.34228097	6.587577626	6.498588101	6.618511313
6.833605444	7.785214909	7.490430596	7.481001515	7.289911515	6.588688525
7.917722121	8.008400859	8.490053091	8.223899788	7.647964475	7.308421545
6.933584646	6.741204455	6.658617323	6.964624475	6.71776502	6.969691626
7.716801838	7.97471396	7.994919717	7.473129747	7.950449798	8.118136869
11.04464523	10.58579258	10.96886286	10.90324033	11.05651949	11.03580591
9.907546465	9.93187304	9.540446515	9.681893121	10.27714677	10.48432644
8.285312424	8.608532485	7.645321707	7.822730929	7.654256818	8.078044465
9.543825273	10.00450786	9.427546879	9.588410576	9.572453101	9.248396293
8.025864485	8.491490828	8.130283051	8.025864485	8.268937657	7.264510162
7.128516444	7.765675424	7.291354758	7.404051424	7.609879768	6.684069253
9.780583495	10.56908161	9.355403384	9.370832535	9.872596081	10.32208546
7.989204111	8.43615796	7.759936788	7.956286283	8.317411434	8.041000495
7.681167667	7.695611414	7.477539293	7.505759091	7.676615465	7.371279929
6.778966495	6.930222384	6.629471747	6.684834222	6.691558677	6.550797374
10.73911267	10.50057506	10.46232362	10.67572435	10.5029511	10.89755671
8.159434576	7.943134	7.38400901	7.742830394	7.631297323	8.382609505
8.878776172	8.532980343	8.965143333	8.569209263	8.937429899	8.010919919
7.25632596	7.346859747	7.954939636	7.280239242	7.547922717	7.059956313
9.030619364	8.667049253	7.436640343	7.553068374	7.569223354	9.02886299
8.924680667	9.21352099	8.572701232	8.841037687	8.728878424	8.940148162
10.29208493	10.21054893	10.1838089	10.33146686	10.41731261	10.71503879
10.06822268	9.831834717	10.10841911	10.19649023	10.13958591	10.22164118
12.42943771	12.57107442	12.70787607	12.71830686	12.71142619	12.86955734
9.76471501	10.28089537	10.38631174	10.13193078	10.32208546	10.12408141
9.857104919	9.641007131	9.736957071	9.928455091	9.920430606	10.22557955
12.68903729	12.60022648	13.39659876	12.797444	13.2158469	13.36582475
9.223302313	9.451212687	9.443424081	9.297353606	9.337862091	9.445852485
9.390110384	8.483326677	8.170129495	8.263866778	8.530767747	9.412044242
8.713915657	9.039989364	8.925229707	8.842109	8.555833323	8.554027364
7.420741101	7.225301495	6.889521525	7.009086005	7.237206848	7.414724596
8.399553939	8.728449242	7.874403566	8.026630869	7.979863071	8.755421576
8.526186556	8.354182626	7.546918172	7.817975838	7.396513838	8.640338566
10.73264046	10.12909315	11.10170724	10.78987961	10.55602586	10.07107569
13.77352346	13.68884286	13.80033164	13.89331098	13.5964752	13.96779242
7.585519525	6.445674283	6.45190396	6.296876485	6.319492374	7.466652071
7.815915242	7.567404636	7.133560273	7.20183702	7.262654505	7.583321859
9.240082808	9.086722374	9.156208313	9.180906778	9.338458455	9.445197818
12.19086998	12.13538265	12.06397065	11.81887725	11.8358519	12.30789527
9.625041525	9.618256808	9.372705202	9.416442616	9.501306556	9.648698737
14.57091557	14.08841453	13.08418191	14.07245549	13.66784462	15.65613201
8.319524414	8.212685152	8.227704253	8.287888051	8.025864485	8.601386131
11.80104424	11.77802923	11.83330316	11.47127063	11.37490296	11.19638858
6.471031303	6.287413717	6.528858343	6.204346798	5.912062667	6.016419242
7.412474121	8.144584758	8.576891556	8.12949599	8.272620253	7.686775222
8.863920758	8.706127333	8.464081152	8.482406475	8.264240727	8.433255692
7.356007838	7.349811747	6.449508273	6.697417434	6.665428859	7.639662323
9.36570098	9.275532697	9.018299374	9.156208313	9.072998758	9.101613848
12.67378338	13.10985871	13.18858184	13.22156044	13.42888134	12.24097391
11.68484165	11.02551486	10.83100199	11.18636156	11.28207759	11.96059797
7.145493505	7.038073394	7.107702061	7.039966242	6.877148152	7.03699504
15.59877501	15.59877501	15.92931651	15.95256028	15.46469304	15.39581235
10.68572341	11.05033578	10.98627861	11.23280633	11.02403513	10.04511943
10.08521343	10.11760527	9.260607	9.746868596	9.465659495	10.58812248

9.004940778	8.792195697	8.864430475	8.941795242	8.899254879	8.881310737
6.757183747	7.484854374	6.868868606	7.099574394	7.346280818	7.443192556
6.093319636	6.551639909	7.037511364	6.469182939	6.739060495	5.971096737
8.357077121	8.554027364	9.291911616	8.830117929	8.605125889	8.171342424
9.379180747	9.35796903	9.012760899	8.640338566	9.277455131	9.706432808
8.867411152	9.418269535	10.21279484	9.919491525	9.663106646	7.833986465
8.551751717	8.057421596	8.005065495	8.083806798	8.053619525	8.319901444
12.24097391	11.58195332	11.95514832	12.03310273	11.79870316	12.54589185
11.86125412	12.09804166	11.93770961	11.14385742	11.89876792	12.71520416
7.449616394	7.01811702	6.616118788	6.637667434	6.567411212	7.176878273
8.213427111	7.757833798	7.164102909	6.852686475	7.407119727	7.258107788
9.10215096	9.272374697	9.011040293	9.058356636	9.315088131	9.211846273
9.175472828	8.893968333	8.916479111	9.109120374	9.077100232	8.997224192
11.02851208	9.239472869	9.828404283	10.56207051	10.60044575	10.02741959
8.517288667	8.546056747	8.564847253	8.571194222	8.570262	8.858692343
7.91307104	7.930615646	7.02213399	7.342268626	7.267645253	7.098723051
7.519695475	7.55847901	7.735250384	7.882189879	7.846992646	8.095746162
7.026391172	7.464044657	6.542089697	6.752731616	6.672081333	6.452573444
9.184527788	8.851571051	8.432612263	8.883993253	8.981479273	8.882882525
10.70534521	10.84302834	10.63266161	10.42175216	10.58812248	10.88945836
6.832875889	6.937104768	7.329290475	7.164674212	7.205488778	6.309118495
8.24122704	8.456593071	7.990677606	8.190353414	8.443956768	8.524867051
7.438305869	6.944336869	7.297445616	7.264201869	6.990730697	7.183662697
7.592689374	6.839464636	6.919663687	7.037826263	7.068520657	7.01811702
11.10460602	9.143918798	8.927565404	9.129114848	9.123926859	11.20840189
7.024510747	7.443192556	8.318668263	7.336145273	6.591366	6.682081222
11.82112126	11.87912023	11.94234713	11.89628941	12.20960187	12.11216258
7.018669091	7.781852747	6.671548495	6.90482902	6.894957566	8.163475424
7.517772121	7.736619182	8.155393222	7.90869301	7.617890202	7.001377828
8.407442505	7.229324455	6.94462002	7.110844919	6.936977687	8.722953263
11.3290504	11.47127063	11.911688	11.48655657	10.85100254	11.39382595
8.348543172	8.067132556	8.412145273	8.41598997	8.479727162	7.974325596
10.84844593	9.21905796	9.049629657	9.201355202	9.087255717	10.88302044
8.421350475	8.631321253	7.346567394	7.543396525	7.389870293	7.187484444
9.106823152	8.874692596	7.374297343	7.509707515	7.632536929	9.451212687
7.896955242	7.991026727	7.662925475	7.815211869	8.095746162	8.148308414
8.788663253	7.193876586	6.996042242	7.094132182	7.391342576	8.583542788
14.25042984	14.56207529	14.3093669	14.4185289	14.25895571	13.61616406
15.10875812	15.39581235	15.27762024	15.38120792	15.34617167	14.1282639
12.03912361	11.911688	10.61992902	11.16579624	11.31267384	11.94979793
8.416773909	8.115821848	8.589388657	8.304980606	8.626355717	8.522156747
7.307847101	7.766357737	8.443956768	7.379463242	8.233838889	6.919083737
9.164000111	9.048100778	8.93587504	9.06557301	9.49850403	8.996148737
7.098240939	6.920918808	6.725993303	6.345240111	6.77206196	7.446760616
7.65457904	8.380307172	7.692552364	8.214678465	7.97284099	7.74578199
7.338887687	8.437898414	6.604082657	7.454260455	7.332344778	9.577319545
8.933180404	9.513164303	9.480384485	9.483552818	9.466909636	9.742895778
7.51310204	7.849137545	7.503050071	7.374842788	7.270153101	7.67303801
9.104371838	7.797125465	7.706567859	7.692217293	7.811371919	8.958493495
9.112697707	7.655968232	7.033114263	7.345977091	7.107957081	9.206093556
7.517436172	6.128938879	6.25782001	6.441889697	6.283913465	7.720869455
9.579413505	7.85268101	8.368780919	8.211847566	8.161911788	9.595333525
6.855446939	7.086698091	7.863532101	7.445432899	8.014979636	6.786546495
10.69682519	9.923658606	10.07580535	10.61867663	10.03299879	9.951175646
9.207791152	8.984797889	9.145664475	9.114524374	8.843148071	9.020821455
8.22437102	7.325732596	7.331457121	7.434501333	7.313451111	8.518144394
7.729367111	9.11510898	10.39747702	9.646274414	9.503271071	7.208594677
8.244006505	8.564366949	8.385691051	8.451947071	8.50111796	7.328995606

7.851263121	7.457359414	7.511838192	7.271131505	8.094605657	8.055171202
7.204326061	6.683531697	6.329524818	6.413438525	6.344403182	6.901978929
8.282438737	8.956407626	8.955359253	8.808928141	8.932594273	7.574795778
9.135618929	9.365016101	9.062220818	9.223885747	9.189590182	8.907915899
10.33146686	10.22557955	9.572453101	9.381544081	9.580097263	10.16073454
10.37346225	8.177528808	7.469040515	9.45706402	8.83948901	8.262596626
16.28343863	16.96059838	16.68963031	16.49551516	16.64557528	17.58093558
7.290204495	6.555298505	6.420140667	6.463778677	6.562503909	7.361082222
6.509391576	6.055906899	6.259323444	6.312781434	6.143049838	6.182938475
9.518503263	10.40074116	10.66465811	10.66945215	10.39968902	10.25038968
6.426872343	6.816006121	6.31629898	6.285312596	6.251035687	6.095353909
6.802811828	7.052221778	7.13441502	7.19832496	6.965960869	6.294668192
9.328020364	8.718321818	8.835019636	8.841575667	9.359783616	9.418269535
7.762794545	7.432999727	7.822322859	7.477877475	7.600248455	7.530574707
6.459423596	7.126812859	6.885887889	6.874493707	7.283562505	6.680656707
7.468792788	6.687236677	6.330092323	6.614259212	6.546653242	7.482605354
9.974493182	9.95201103	9.273022949	9.704210374	9.519086535	10.17702971
6.581970424	6.891529131	6.310299848	6.296703525	6.261965667	6.522534343
14.4335752	14.61202705	14.19920898	14.47046648	14.28325373	14.27397716
8.941795242	9.117478424	8.656360697	9.005975162	8.663303283	8.157542717
9.159785394	9.249612495	9.739033131	9.47593803	9.584803364	10.33890802
10.04340808	10.28005063	10.62895885	10.57028524	10.63391466	9.305189859
15.08134613	15.13975511	15.39581235	14.67552064	15.39581235	14.76275492
8.665165081	9.321890899	8.711304737	9.190767364	8.687969051	8.911610283
9.49850403	10.26116661	10.01974654	10.17030341	10.10187506	8.788663253
11.40306525	11.25898056	10.59685822	10.74945851	11.06877356	11.13713447
8.314398727	7.994919717	7.644277273	8.123844788	8.043387838	8.913588253
6.836753071	6.797074616	6.072482869	6.862895808	7.204610747	7.19003896
13.81266894	13.86604428	14.15863941	13.84455043	14.19920898	14.20688311
7.865908465	7.431067202	7.468171747	7.224144889	7.270452455	8.168587596
10.00695165	9.734703111	9.702758343	9.93187304	9.979092404	10.60635433
8.114219303	8.130993828	8.254082232	8.313601758	8.232163879	9.171164091
7.689906808	7.807908545	7.714317071	7.919201576	7.683675283	8.136235949
9.008956374	9.091299889	9.533596364	9.569063343	9.303966081	9.147296667
7.728706424	7.623693616	7.933961929	7.997875909	8.066736566	8.135850465
6.725034798	6.708558414	6.062932939	6.193905333	6.16294199	6.234440616
16.33688632	15.74256723	15.48449671	15.81050193	15.95256028	16.3077943
12.02240324	11.81887725	11.96947069	11.97196754	12.18492446	12.10135206
8.508189222	8.787164939	9.387059283	9.081927515	9.223885747	9.042346232
7.506434626	7.65951298	7.720197707	7.707249808	7.403718889	7.842052606
8.298396535	8.747331152	7.603719626	8.014979636	7.875808798	8.575566677
7.727390434	7.119927869	6.76309904	7.198026636	7.019697232	7.481001515
13.30795266	13.27940818	13.35919327	13.45466248	13.39659876	13.9071606
7.228468091	7.721939444	8.025425616	7.842052606	7.833291081	7.001612657
12.79024082	12.14507673	12.19086998	13.12023156	12.27101	12.42943771
12.69316533	11.25700815	11.54470354	11.72355631	11.79171062	11.5612151
12.44950322	12.4956701	12.0724233	13.2158469	11.88375452	11.88909817
10.42175216	10.37467131	10.47123164	11.76336308	10.6820228	10.8935809
10.6488688	10.37346225	9.879566364	10.68338253	10.18198959	9.722667242
6.172285434	6.716586707	7.099269525	6.748092424	6.361367657	6.219261424
7.696259101	8.260520646	8.852092202	8.45577798	8.923576949	7.144617717
9.835081424	9.818059192	9.835894606	9.898158111	9.895061889	10.21054893
11.00620373	11.41981305	12.17216641	11.4090051	11.54470354	11.10460602
6.899489646	7.046194354	6.183978364	6.157181768	6.046729758	6.984849141
7.340448859	6.740481444	6.42621898	6.457381818	6.448856899	7.266262606
16.14978837	16.14978837	16.36806566	16.28343863	15.99508933	15.90458538
14.010587	13.70717091	14.09521817	14.23242051	14.3093669	14.40789433
9.902579253	9.698896848	9.684886576	9.672623929	9.870931818	10.40925212

12.97555423	12.66985986	12.27773509	12.52461444	12.21991364	12.21312889
7.307562737	7.67303801	7.182546172	7.455517596	7.396159798	6.959689121
7.111135707	7.69185196	7.783465061	8.280707687	7.742436303	7.10439698
6.705415545	6.418892273	6.102848495	6.128938879	6.065202949	6.712342556
7.875433545	7.521885657	7.493254434	7.428605818	7.649926505	7.866656354
10.36032821	10.07956055	10.10187506	10.29101087	10.33476777	10.1752482
16.42659037	16.22759729	15.83165575	16.2036826	15.74256723	16.4534382
7.474938939	7.309928404	6.833605444	7.20129803	6.814222283	7.632861657
17.86179439	16.42659037	16.8710039	17.36803328	15.18392271	16.49551516
16.49551516	14.9847254	15.58150095	16.22759729	15.32551587	17.1822602
8.549517636	8.112876232	8.495994869	8.544633697	8.613130333	8.748883111
17.1822602	17.29704085	16.90924948	16.7804214	17.07010739	16.64557528
10.57159251	9.388912091	6.45473303	6.586655667	7.007889061	7.554062253
9.59387599	10.22164118	10.09254304	10.0672481	9.945994374	9.012031232
7.359873626	7.026621727	7.350388939	7.279952616	7.277222202	7.31258798
8.102124455	7.664275131	8.052417212	7.83046296	7.574795778	8.036387556
8.677215515	7.945472162	8.386133566	9.343457394	8.426780919	8.31912796
8.380307172	6.864210101	6.742219182	7.049507061	6.901464747	8.172571899
10.69793218	11.02105469	11.51465712	10.89604789	10.88166557	10.03876748
14.72870348	14.9727616	15.16803204	14.94534759	14.6970922	15.12351424
17.23317693	17.86179439	17.58093558	17.23317693	17.43906183	16.96059838
16.2036826	16.52993947	16.39933956	16.25437967	16.04217659	15.449005
11.27499716	11.78725933	10.38531322	11.04030995	11.0940021	11.17213305
9.080123545	8.366462404	8.241600556	8.424934535	8.179120646	9.186096828
7.493254434	8.006495333	8.631321253	8.363019899	8.163475424	7.090449141
7.026870727	7.117401788	7.205228253	7.170232747	7.094132182	7.434204455
9.226702434	9.204855576	9.531623293	9.29496	9.163332636	8.925229707
9.57872197	9.454390232	10.65871503	8.684081152	10.3380268	9.578067192
10.67572435	10.19136651	10.58236152	10.73132595	10.41224321	11.16734497
7.987573434	7.048453949	7.085030667	7.179445253	7.208594677	9.110297333
12.72972704	12.27421104	11.74205097	12.35168631	11.98196729	13.44849295
11.44319437	11.38775416	11.94979793	11.79405389	11.62059475	11.73271713
7.168103949	6.927259889	7.303836939	7.40526796	7.359577545	8.08193004
7.786626808	8.29927897	8.606099333	8.555833323	8.484723788	7.607714535
10.00002479	11.19638858	10.52135963	9.603529667	9.881912677	10.89094316
6.869142343	7.134684455	6.386460131	6.819920253	6.804242869	7.835124576
7.561964414	7.220780818	7.700670768	7.653990838	7.550541152	7.913871889
7.392836818	7.662925475	7.89464897	7.560401535	7.707249808	7.598252121
11.01376888	11.00053539	10.12314196	10.08700287	10.3810842	10.68572341
17.07010739	17.12376032	16.7804214	17.12376032	17.23317693	17.12376032
10.07580535	9.960013465	10.26729954	10.2692332	10.40387449	10.3305428
7.690510313	8.739178636	8.553595747	8.465492313	8.418553323	7.360451687
9.831834717	9.428845576	10.11664425	9.37540303	9.660200838	10.13671639
7.562627657	7.327234414	6.423272566	6.748847646	7.038360253	6.918565768
7.427086202	8.155393222	6.899721758	7.263531485	7.145769434	8.254082232
9.093526566	9.329342374	9.097596465	9.953782444	8.986432162	9.434425707
7.156444778	7.752360667	6.790568566	7.010749455	6.758137737	7.20129803
9.554043101	9.140978061	9.240082808	9.526200354	9.827596687	8.816703535
7.304125475	7.269892283	7.318216242	7.385857192	7.59498599	7.568622485
11.93975968	11.86922315	11.94481318	11.79615897	12.19398134	12.15440913
6.798354081	6.696473434	6.683791949	6.66391404	6.666656929	6.913266485
8.35372597	8.934777717	8.55725397	8.868979273	8.732044667	8.608532485
8.652374404	8.366003535	8.744081071	8.622197111	8.689808788	8.354570556
8.488340929	7.666604434	7.907197636	7.781509414	7.588284717	8.743124222
9.236429667	8.912452162	9.085558838	9.203760495	9.307024131	9.426842081
12.00354228	11.17750487	12.49170848	11.60479972	12.0089388	11.68484165
8.760291222	9.060017677	9.580097263	8.848914293	9.605576293	8.936938576
8.324453253	7.946141838	8.771856424	8.23581501	8.602379626	8.481411768

11.73271713	11.67593314	11.54053762	11.82112126	12.12939166	11.96636849
9.164545343	9.095162899	9.57872197	9.661659101	9.856174394	9.541747455
10.77103399	10.77103399	10.32418729	10.43332542	10.74558579	10.83363242
8.663303283	8.499836475	8.382609505	8.48873997	8.642670768	8.912003131
8.118879606	8.410410394	8.982611727	8.473802646	8.690381253	8.533908051
9.951175646	10.09896607	10.6313464	9.910960495	9.980879424	9.815893677
8.739178636	8.77962597	9.212349869	8.69723302	8.882882525	8.653674131
12.55625498	12.51766186	12.71830686	12.57486869	12.43944324	12.42616911
9.200042253	9.142791657	9.382205394	9.312041414	9.510206136	9.363823202
12.44611991	12.17216641	12.44611991	12.5521346	12.61449239	12.7494647
8.35752198	8.373157505	8.819236758	8.641737071	8.753420071	8.368326869
12.81379045	12.74093701	12.87819387	12.74093701	12.61090773	12.13867659
10.25519474	9.877792838	10.14326599	10.36134852	10.51291939	10.60863639
8.140538697	7.834378071	8.01423197	8.068564879	8.384837869	8.096525737
9.773700283	9.49510903	9.714566535	10.17224075	10.08325108	9.811898121
8.883993253	8.941359636	9.078885242	8.995099212	8.884521697	7.996393263
10.92190902	10.40701089	10.11941657	10.71390019	10.73132595	10.6240143
13.91489381	13.42888134	13.64083669	13.69473258	13.80704919	14.1904887
6.585931495	6.668735424	7.479422939	6.890245747	6.998984111	6.697631071
7.799854778	7.291068525	8.426330949	8.259704283	7.621699758	7.851263121
11.97660652	11.35657003	11.25898056	11.70557621	11.73710477	11.87445957
10.87637444	11.15966862	11.05493438	11.41981305	11.63174178	10.74045328
11.08936677	10.81796524	10.93935201	11.28053313	11.29076429	11.45932673
12.71142619	12.56315114	12.95303817	12.92542832	12.90359001	13.18348162
10.60507136	10.52799495	10.34830463	10.49830212	10.72516317	10.89912578
15.18392271	15.03840752	15.41210544	14.72870348	14.4427031	14.90726461
10.65365596	10.6362571	10.60383217	10.42512206	11.03008027	10.80389925
8.632349838	8.853585596	9.430738808	8.648914657	8.69335002	8.147481596
9.157435455	9.746145949	10.22865659	9.957374697	9.776037091	8.120795899
13.40302459	13.88591407	14.53168901	14.10328164	14.30038792	13.09045797
14.9727616	15.52328152	16.4534382	15.78787594	15.59877501	14.28325373
8.745901071	9.644080808	9.804809051	9.26122399	9.638309758	8.527198758
9.135096212	9.474605101	9.577319545	9.244399414	9.656571869	9.146732444
10.28795108	11.27695531	11.02700786	10.59418919	10.85513517	10.1309568
13.17281418	13.99495772	13.78737352	13.32669092	13.40302459	12.4571909
6.816280253	7.663618646	7.25490398	6.55412801	7.60736902	6.320680717
7.404985061	8.197805768	7.693516556	7.128516444	8.30460302	6.859701798
6.701095535	7.789255535	7.841440677	6.537516434	6.792957485	6.319301697
8.125376242	9.436357141	9.432003333	7.893940788	8.840590677	7.583998525
11.39983314	12.41337695	12.3543152	12.04432	11.9577766	11.04316622
12.18142186	13.40990013	13.54221797	12.8293171	13.12023156	12.38063147
9.684048687	10.80644997	11.07013707	10.24511785	10.70425349	10.31795252
10.33596201	11.41778198	11.55928848	11.46936418	11.82852227	10.55842197
11.20840189	12.04432	12.25844332	11.83072395	12.17216641	10.87379507
14.99939481	15.24813401	16.2036826	15.76560628	15.41210544	14.49002723
15.06622261	15.10875812	15.63778652	15.29549959	15.24813401	14.16629104
14.5108222	14.67552064	15.72204116	15.22815255	15.31193978	13.85099976
15.86132141	16.49551516	17.12376032	16.57174008	16.57174008	15.19830883
7.522209596	7.883157929	8.760676242	8.315715768	8.066374202	6.850381687
7.577683121	8.252794354	8.868452172	8.281588828	8.355847778	7.398696909
6.784609556	8.524867051	9.789981192	8.147857556	8.353342091	8.08737399
11.92665931	11.50425191	11.00620373	11.2786408	11.29399858	12.72610186
8.273483707	7.619556354	7.14635899	7.528004313	7.386173242	8.470863394
11.38970236	11.42178201	12.24933389	11.911688	12.08680505	11.88148631
12.67700968	12.35798465	12.54268587	12.88265743	12.79024082	12.71830686
9.955574404	8.638952889	8.287438535	8.513601889	8.35372597	10.28005063
10.30358984	9.985290333	10.39072525	10.10841911	10.44934262	10.85513517
7.922697606	7.869396606	7.967524909	8.190781646	8.050868949	8.143776848

9.665316566	9.517013525	9.417594202	9.821914929	9.811091061	10.21279484
11.85617239	11.24323027	11.68948846	11.21187068	11.21338774	11.95514832
10.83930015	11.10035391	9.324974768	10.29000276	10.25320711	11.33100665
11.54470354	11.50261564	11.37131374	11.53239215	11.19114259	11.60266156
6.935569505	7.257820879	7.274888343	7.09353204	7.227882465	6.984018879
11.56725755	11.34576518	11.34892675	11.3733012	11.67593314	11.52448673
11.04593523	10.75453103	10.73788216	10.83100199	11.0859227	11.15666056
7.001104828	6.923688879	6.871437091	6.970472768	6.791799101	7.680542242
10.93935201	10.73517618	10.71613341	10.85225864	10.87907816	11.06732533
9.801802283	9.905108616	9.47593803	9.830354566	9.960976253	8.962353222
7.249962576	7.763504859	8.156085586	7.768772758	7.618887111	6.442532636
10.01974654	9.870221061	9.831834717	9.940265465	10.08893073	10.53028115
10.53028115	9.988725727	10.30358984	10.43122177	10.66706732	10.64379219
7.532439838	7.207500556	7.494168788	7.708922879	7.303836939	7.928303495
11.57178154	11.30131952	12.00354228	12.17829278	11.77552743	11.59654933
9.260036485	8.249350414	7.917722121	8.65497299	8.185177828	9.384699788
8.049354232	7.210358909	7.652632424	7.962380919	7.16725497	7.873080636
9.302735434	10.44094498	8.998420707	10.31480319	10.31378621	9.887613414
8.375668162	8.754900343	9.419020677	9.178084667	9.226702434	8.322806515
6.043588101	6.631584848	6.241029384	6.651970061	6.665174586	6.103147919
9.431415586	9.192533242	8.760291222	9.583475303	9.029402949	9.182204323
7.38340502	7.167545535	7.411561343	7.00817199	6.983707354	7.784529071
7.147555404	6.247479727	6.124663283	6.27402404	6.255446566	6.571432061
6.243838475	7.401460495	6.788765101	6.364484303	6.658129061	7.609879768
10.45877561	9.807061475	9.549314313	9.675185677	9.50710198	10.11452638
8.279889293	7.403028737	7.026391172	7.073400869	7.000584848	8.127895253
7.118205404	7.353560545	6.754699697	6.69350096	6.586196364	6.496794495
7.857845061	7.825104404	6.971315	7.270153101	7.093823343	8.014589606
5.829948566	5.82849902	6.509844303	6.223158818	6.333774687	6.106166717
9.233272283	9.045168101	8.581684657	8.418959303	9.596088263	8.229734556
7.339214566	7.482291222	7.40744601	7.404680798	7.471589222	7.508747081
12.14789109	11.8164662	12.13538265	12.28985985	12.23769992	12.06397065
6.660321343	6.805470263	6.179675212	6.382405131	6.363322586	6.55251301
9.268661657	9.277455131	9.260036485	9.197870838	9.276789111	9.605576293
7.065949717	7.495424616	6.597017333	6.839237455	6.708308495	7.761767192
11.565007	11.54871614	12.01956714	11.48840455	11.80342824	11.35858946
11.08264222	11.33848921	11.90674449	11.76588718	11.66708917	12.08943903
6.758137737	7.068375576	7.462172081	7.297158313	7.374297343	7.354199768
11.50261564	11.29947244	11.24457024	11.30301738	11.43005301	11.61117883
10.16262583	10.31378621	10.33989796	10.50677257	9.890949131	10.78551178
8.508645727	8.174468182	8.339926909	8.526649808	8.453185455	8.708030737
11.79171062	11.70557621	12.24649354	12.14789109	11.56988377	11.20163629
9.438799404	9.446464909	9.11393401	9.363307667	9.275532697	9.095162899
7.063754707	7.056486293	6.419103354	6.519889687	6.633467848	7.298118263
7.351279444	7.316995263	6.698080333	7.139090576	7.08160301	7.279114576
9.752732475	9.442729677	9.846508495	10.03026493	9.900778232	9.695719162
9.016491202	9.140392293	9.283145788	8.370830747	9.530933293	9.666877556
8.382609505	8.589388657	8.758364879	8.604635949	8.84786598	8.695776131
11.80554878	11.93486375	11.565007	11.80791686	11.93178106	11.92156179
10.26116661	10.21279484	9.499917182	9.951175646	10.15783547	10.33375706
10.08978265	9.890949131	10.11150219	9.949411172	10.28899471	10.08325108
8.914132333	8.314850485	6.976769172	7.246141232	7.037826263	8.120062485
6.595947525	6.16631502	6.71142402	6.58104199	6.464688212	6.448856899
7.969046636	8.331888859	8.383846919	8.251922909	8.319901444	7.652632424
9.666877556	11.00754401	8.197004869	8.672881101	8.466446051	11.83072395
8.683580283	7.919459465	8.051625212	8.059423535	7.97128602	8.753927596
11.49636188	11.49839615	11.31449746	11.32559397	11.56291631	11.50051559
8.138908141	7.989899475	7.853698798	7.947667091	8.085049192	7.892604899

7.862160495	7.487078798	6.977041091	6.92644801	7.042119313	7.908308323
14.77222727	14.83117585	15.36066179	15.06622261	14.68633385	13.49745762
7.668279394	7.360165061	7.49893601	6.910146768	7.37546403	7.754082929
8.151663141	7.671593667	7.743818828	7.668279394	8.396963808	8.33273403
10.14999886	10.41123643	10.18942995	10.21975253	10.48074239	10.52247938
7.597541737	8.310875455	8.623636919	8.348145404	8.048299333	7.173915838
9.417594202	8.892401576	9.308265131	9.263851586	9.231946182	9.606298465
7.698928879	7.983184263	8.953653768	8.287438535	8.779146061	7.721580051
14.58095582	14.35197184	14.62217632	14.70594079	14.4185289	14.89443037
9.208914778	8.257984293	9.207219232	9.151976485	9.509226798	9.347406172
12.68484185	12.97555423	13.01016522	12.94367069	13.04869312	12.12436236
8.892970697	8.669446475	9.224452333	8.886096818	9.395854121	8.50673198
9.883591758	9.715215101	9.62302801	10.1752482	9.971733293	10.17336172
11.45002291	11.45002291	11.60266156	11.57178154	11.34381562	11.50632759
9.755162434	9.479084687	9.598265626	9.512482222	10.16546821	10.25627793
14.3093669	14.19920898	14.90726461	14.28325373	14.5108222	14.31917195
8.212243444	8.45451298	8.893968333	8.298896434	8.249809909	8.072110051
8.980444384	9.245932475	10.24828231	9.504584061	9.424985495	9.719000626
6.94072599	8.095330333	8.978204141	7.735579636	7.837507091	8.047188455
9.447991384	9.707282667	10.16262583	9.206631485	9.41003803	9.216574283
8.705145061	8.798818646	9.165786899	8.773471869	8.928157121	9.041236566
10.45150222	11.11856721	11.90384839	10.81280665	11.00183361	11.02851208
10.34626898	10.3380268	11.2345748	10.84302834	10.7532413	10.49466708
9.004398101	9.493894545	10.17224075	9.524763869	9.492465394	9.251353071
8.627796121	8.72650097	9.042944374	8.583048444	8.614442374	8.347717242
13.06967433	13.39659876	13.91489381	13.48401144	13.60269863	13.13007894
13.27418186	13.83173935	14.30038792	14.37933382	14.07988992	13.10484893
12.70002263	13.49745762	13.62106704	13.60269863	13.45466248	12.58957491
10.98037262	11.82852227	11.89130603	12.04734694	11.93975968	10.99345288
14.7528858	15.50564301	15.21350605	15.26374094	15.26374094	13.87263802
7.179751081	6.424673818	6.519436343	6.542314697	6.305155434	7.034867141
8.863375313	8.821324343	8.645541747	8.927019859	8.90531298	8.780092131
7.376100929	7.453956636	7.920144808	7.644626202	7.531511131	7.628690051
12.57107442	12.68484185	12.88265743	13.00101781	12.59323492	11.90674449
10.01703805	9.566253768	9.627013505	10.09994749	9.620873889	10.26116661
9.161459798	8.995099212	8.498949848	8.701614283	8.789132293	9.423636879
8.584413131	8.644074768	8.281167657	8.63568099	8.120062485	9.21352099
9.701165495	8.18280501	7.448310273	7.75479003	7.563777768	9.848227263
7.655611	8.119288848	7.432999727	7.657256192	8.213427111	8.781156657
7.901142576	8.351407394	8.556855828	8.004655889	8.443547162	8.829514889
9.898158111	10.10841911	10.65127749	10.27714677	9.838413929	9.691258919
7.050073121	9.283145788	8.036816444	7.495088838	7.323994646	8.595703707
9.887613414	8.753420071	9.395177889	8.802954374	9.59460096	9.772250768
9.244930505	10.65500785	10.40074116	10.03492192	10.13580598	9.224923687
7.05790099	7.540114586	7.377888556	7.401155333	7.483574061	7.072297535
6.752477677	7.780823677	7.422831253	7.669657737	7.631889737	6.976233182
6.103600394	6.982135444	6.646173444	6.726701909	6.772767788	6.475135818
9.122138505	12.16036216	10.77103399	11.44491584	10.8405808	9.245445293
7.604463141	7.037826263	7.676257364	7.534885556	7.623693616	7.462172081
9.992117576	10.59685822	9.756840071	9.335891293	10.17702971	10.10187506
7.000584848	8.443547162	7.644959949	7.699362081	7.666980273	7.141989162
7.670902747	8.406604404	7.411868313	7.958393384	7.418614091	7.59498599
6.827661505	7.733228788	7.700334879	7.554062253	7.755503707	7.597232061
7.929905606	8.22437102	6.792730596	7.358075697	7.124596343	7.987912222
7.958031758	8.816703535	8.805907646	8.519692939	8.577417788	7.794365768
9.039989364	10.2026692	9.800083455	9.591133828	9.722667242	8.928828384
9.565660949	9.848945263	10.72384747	9.982665242	9.496413788	9.374690616
7.976858394	6.734736768	6.688467242	6.916684768	6.75222999	7.869992939

6.244571899	6.617114677	7.233950838	6.64187404	6.732525747	6.029867525
9.315671101	9.322592778	9.666877556	9.574750414	9.495686737	9.333361616
8.516793455	8.371674091	8.35018502	8.610337293	8.154321646	8.712341879
8.287438535	8.021984222	7.570191354	7.870356606	7.638629778	8.32939098
10.47242114	9.994772242	10.73517618	10.43757507	10.37124354	10.37124354
8.829514889	8.663303283	9.072403576	8.757405333	8.34368697	8.560348606
6.784076586	7.148452788	6.307278677	6.634635343	6.571898091	6.710717586
11.92937262	12.55983323	13.16824615	12.53563192	12.35798465	11.31267384
7.806122253	7.328995606	6.666656929	6.860179071	6.542523919	6.306085808
7.034867141	6.59799604	6.925960677	7.33858799	6.816006121	7.019921606
11.37131374	11.17571904	11.24323027	11.56291631	10.43122177	12.05813886
11.1060681	10.62647628	10.81280665	10.6820228	10.49241362	10.70425349
10.81034167	10.32635032	10.84176532	10.92880859	10.09896607	10.58117749
6.994489162	6.732525747	7.380377788	7.433629313	7.360451687	6.643746616
8.794361798	8.558108202	8.483763566	8.805907646	8.691979949	8.939019091
9.384699788	8.601896768	9.056062667	9.039454747	9.678297182	9.953782444
12.77015535	10.55070525	9.90675497	12.41337695	11.67849095	10.08521343
7.485802727	8.272231939	6.82197003	7.24440804	7.191807606	8.649462798
10.57885616	9.311440606	9.712341424	10.16165443	9.759931222	10.73014715
9.345614434	10.10631712	9.935661677	10.35385551	9.848945263	9.009915859
8.60808904	7.969793182	8.960773354	8.574218515	7.891912283	8.275152535
6.161616828	6.168490424	7.007051354	6.060042182	6.633705889	5.993783505
7.55847901	7.411268949	8.226165	7.615524929	7.895961929	8.301657889
13.43523914	13.08418191	13.48990673	13.27940818	12.92542832	13.81935116
7.093254121	6.794399263	6.393414081	6.407880727	6.458735293	7.193007566
11.90674449	12.28693608	12.2031413	12.10694646	12.06964699	11.91651973
11.24457024	11.32185019	12.23769992	12.21312889	11.92156179	11.34576518
7.368501293	6.744072859	7.521572545	7.488894061	7.756889051	6.616392283
10.57282784	10.69682519	10.96748398	10.89755671	10.7442872	10.01340346
10.01257663	9.905938879	10.86173253	10.59924244	10.5336638	9.661659101
10.15086094	11.27499716	12.04734694	11.78265022	11.65698449	10.59924244
15.54332231	15.46469304	15.67809237	15.42966281	15.22815255	14.94534759
8.71485401	9.359783616	9.600888859	9.249612495	9.267383556	8.563501616
7.627997354	7.377557566	7.559825556	7.508388697	7.653651778	7.946541949
8.861258687	8.896578162	8.515864333	8.601896768	8.700606657	7.966054535
10.96886286	11.28896545	12.14191765	11.65455063	11.49636188	10.91903182
7.610976808	7.119314485	6.436392303	6.583924121	6.347837848	6.536206333
9.615324495	9.538964939	9.708693071	9.789131121	9.582761798	9.383480283
6.510752152	6.260672758	6.535299384	6.313155323	6.485516717	6.035878667
8.11773804	7.537489808	7.033845343	7.281724869	7.02555202	7.900715717
7.703321374	6.190240242	6.254907455	6.611130667	6.290882545	7.776293606
8.147857556	8.454954939	9.223885747	8.793339475	8.516311424	7.614497747
6.550314424	7.339805162	7.458509535	7.392836818	7.153495505	6.295918879
8.222223545	7.835434535	6.948873394	7.287539616	7.178323677	8.390114414
7.216973434	7.28082202	7.670902747	7.538797101	7.74578199	7.281724869
7.463067838	7.12483998	7.389558374	7.506719576	7.451961045	7.53302901
8.389884152	7.900388485	7.953840929	8.020096283	8.029383646	8.232163879
10.99763472	10.63391466	10.85654613	11.15808934	10.70643744	10.76239578
9.291393586	8.897177899	9.956562586	9.55462702	9.400327152	9.569854202
8.064713051	8.040196323	7.589252838	7.629016404	7.80435998	7.886480404
10.00262223	9.50710198	8.526649808	9.022000869	9.271759586	10.45041513
11.43208235	10.6426412	10.20532801	10.31378621	10.31193963	11.22947614
8.037664566	7.999737535	8.082337909	8.11047698	7.850535525	7.948737212
10.10454015	10.2829717	11.07493153	10.62280049	10.69222123	10.15483313
8.165787747	9.817379515	7.916712838	8.631899566	8.664725051	8.452374444
9.604175636	10.24227579	10.48310631	10.45150222	10.18942995	9.644080808
8.234232515	8.465492313	8.751405818	7.384903253	7.346859747	8.059423535
9.018299374	9.438799404	9.659537192	9.261851101	9.341818545	8.82024202

6.575249545	6.462162828	6.688683899	6.876878333	6.71590199	7.117401788
7.996770556	7.910120242	6.838696091	7.294284717	6.943812717	8.219832939
8.759757687	8.867411152	8.10140802	8.427685838	8.410908071	8.358783152
7.641344242	7.604463141	7.128773535	7.304428303	7.187741232	7.349485707
6.484023485	6.498851707	7.978709162	6.385400465	6.675711333	6.435093051
7.580904889	7.766738848	6.889002121	7.190930768	7.189144101	8.144161414
9.961780586	9.887613414	10.12697515	10.06255975	10.08521343	10.45150222
12.48458958	12.02811467	13.04381444	12.41634374	12.35168631	12.92937388
10.96460614	11.85889751	12.6076243	11.45002291	11.6157426	11.19293398
14.32804886	14.37933382	14.65397593	14.56207529	14.64328928	14.7172184
8.539776737	7.289658101	7.116047475	7.121365859	7.047846657	8.614861051
6.321876909	6.005792444	6.156849111	6.267871525	5.980019909	6.344013859
9.024707707	8.567346869	8.543234566	8.656835556	8.879851283	9.166446616
7.263859848	7.094997242	7.042643192	7.01811702	7.051937434	6.697417434
8.400464071	9.202552434	8.296772869	7.747112485	8.805485081	8.588971687
7.774283859	7.519364889	7.97471396	7.803554182	7.716801838	8.152794889
8.810986707	8.599954677	8.603745778	8.630877737	9.451851384	8.59655801
9.158023	9.482360616	10.16355358	9.711723394	9.29496	8.705608919
10.9889639	10.70299608	10.93171462	11.14057221	10.65246846	11.02551486
10.14631732	9.657223606	10.35699559	10.22164118	9.969141677	9.491725303
9.337862091	8.769354545	9.211184727	9.35858599	9.068490586	9.862946677
9.344897333	8.805907646	8.923097444	8.961327374	8.790130253	9.532335293
14.07988992	13.71423947	14.23242051	14.02572128	13.85817003	13.83173935
6.723002525	6.994769535	7.230210646	6.413218091	7.035420475	7.939609869
7.66151798	7.613240606	7.652274172	7.65457904	8.159907626	7.997183333
7.182546172	7.859670455	7.864224778	7.046194354	7.883157929	7.747885899
12.65819872	12.80166365	12.51766186	12.47761486	12.57877126	12.09225922
6.430913434	6.198948818	5.865959808	6.074631616	5.910561061	6.183644212
8.481851586	8.05474504	8.077645899	8.256499515	8.583048444	8.479185606
9.895061889	9.569063343	8.177906263	9.124449354	8.725113212	10.16165443
8.726024485	8.835493283	9.514496606	10.09079019	9.482360616	9.179819687
12.58612865	12.01673563	12.61090773	12.56726296	12.54906863	12.37416009
7.194433949	7.70790696	7.490096869	7.581657414	7.608647091	7.281402384
10.35809493	9.922126404	9.243096889	9.288419414	9.235747242	10.761049
7.382214869	6.937364475	7.183404394	7.111681374	7.277482444	7.18421398
8.323598384	8.338686465	8.727528626	8.680513909	8.386133566	8.14677703
10.26445444	10.080472	10.52799495	10.39296715	10.15585749	10.32418729
7.255477111	6.59646801	6.120530394	6.297260404	6.162773061	7.350114798
10.66587479	10.36781996	9.616057222	9.899969596	9.812775374	10.32849396
8.709464434	8.244006505	7.816627253	9.372705202	8.101068051	8.78563097
8.105629576	7.917722121	7.67303801	6.073749475	7.900388485	8.116577384
8.53579602	8.200961636	8.251125323	8.724556485	8.067132556	8.127495596
10.90466263	11.35858946	11.28731295	11.56988377	11.1502402	10.05970796
7.06891401	7.240898899	8.148308414	7.525550222	7.396823121	7.311629869
5.897552566	6.219635727	6.599359394	6.284925121	6.213570111	5.757413838
8.735755485	7.998587576	7.844550919	7.812327374	7.794719919	8.916479111
8.140977576	8.206690121	8.441767061	8.457043091	8.102484232	8.428657566
10.58236152	10.01703805	10.49713487	10.38329808	10.4322159	10.61261659
12.77454263	12.97977545	13.02006671	13.35919327	13.18348162	12.97555423
7.488894061	7.000309313	6.980492495	6.808747657	7.140588444	6.395988485
6.825870455	6.612316919	7.02934998	6.525595273	7.231823384	8.024267525
7.406536232	6.948047899	6.703725465	6.698531303	7.019424707	7.668279394
7.780823677	7.715785354	7.631889737	7.682204525	7.636262586	8.289212586
9.161011869	8.743124222	8.693780273	8.708030737	8.774537848	9.47593803
10.71731517	10.24511785	10.91641235	10.69354222	10.94942311	10.39968902
6.16228202	6.973416747	6.322925	6.377923929	6.148191788	6.443888202
8.37004801	7.934739636	7.97128602	8.481411768	8.131862263	8.021984222
7.461834424	6.731175838	6.724256232	6.589360121	6.561106071	7.211210626

9.847335909	9.638309758	10.10631712	9.886762101	10.01071956	10.11555853
7.514105485	6.952529081	6.85895202	6.744878414	7.110844919	7.820605525
7.246435697	7.762451808	6.584387121	7.116743323	7.225001677	7.071771354
7.393766606	7.025021455	6.405686404	6.451227182	6.372542859	6.866055313
6.020401747	5.866835152	6.094348303	6.255621687	6.349789677	6.49370698
11.25898056	11.26446827	11.109559	11.238428	10.58347296	11.19817469
10.40486524	10.15585749	10.48897537	10.3779146	9.794631646	10.05320582
5.749171687	6.413218091	5.979399586	6.36270599	6.02015296	5.935453677
8.773988747	8.169382444	7.886820596	8.057063626	8.041792596	8.671783687
7.186327495	6.700579303	6.474509737	6.575656404	6.404620899	7.269892283
13.33155169	13.40302459	12.19727311	12.57877126	12.6076243	12.72219022
6.89207597	6.215155556	6.79323798	6.253672202	6.751729354	7.036743576
7.296277414	7.139090576	6.413438525	6.560645212	6.552305929	7.325732596
9.178084667	10.3918566	9.596088263	10.2187154	9.742895778	8.361756343
11.14057221	10.86305205	11.29399858	11.42371373	11.37847359	11.34381562
12.24351077	11.13085117	10.32849396	10.47479655	10.38531322	12.20640951
8.248137606	8.755916071	8.081502051	8.316969364	8.101780545	8.578858313
8.534432515	8.47107903	8.984797889	8.576891556	8.736738475	8.177528808
7.872008071	7.411561343	7.680542242	7.467207283	7.455822202	7.839249838
6.268625929	6.443018677	6.315391253	6.400112778	6.398621818	5.997184121
6.608710798	6.805713475	6.200564747	6.321067485	6.319301697	6.437269283
7.125696242	7.007335798	7.133813545	7.172411071	7.441979727	7.277222202
6.116576919	7.013396273	6.265203717	6.924473131	6.455606303	6.184170131
7.740469253	7.531770848	7.807908545	7.967920667	7.939609869	7.742436303
10.13671639	10.33476777	10.48185706	10.55333109	10.33596201	9.745446081
11.76336308	11.37131374	11.82112126	11.75662781	11.72807534	11.66708917
8.468898455	7.698928879	7.688370495	7.531770848	7.463713737	8.391869283
8.03601997	8.209418434	8.713417869	8.400464071	8.148308414	7.498029465
10.96748398	10.35917202	10.36239309	10.47941256	10.31480319	11.20660223
9.617464455	9.070661273	9.553430788	9.41003803	9.777520899	9.821914929
7.813759899	8.144908747	7.742113232	8.066736566	7.845242687	8.994589434
9.229082869	8.552719747	8.167813576	8.441767061	8.476085384	9.494490616
6.567684242	6.762568667	6.854415313	7.19681397	6.830578646	6.281398798
6.751729354	7.229587778	7.520648323	7.252299788	7.217862778	6.520809899
9.86223396	10.04963797	9.436357141	10.03701736	9.750460616	10.13479405
7.751275798	7.698300838	7.624735818	7.286722061	7.896287434	7.639261768
8.307093606	8.44129198	9.566949	8.812517081	9.245932475	8.460053242
7.829325051	7.31189602	8.333749495	8.065602475	7.91465098	8.117339677
9.917043434	10.08231743	10.1597097	10.32946621	10.11760527	9.204855576
8.44446797	8.067838768	8.463063475	8.300483646	8.072110051	7.634532263
9.030004576	9.59387599	7.106035242	8.462236515	8.62991701	8.175288444
7.803554182	7.555661677	8.269818727	8.024633293	6.963895283	8.096128192
8.265765712	8.019097616	7.791533949	7.754407434	7.868335495	8.558556152
10.99469779	11.45526933	11.0035084	11.29076429	11.25523353	10.31894523
7.256041222	7.421647414	7.678291495	7.492282404	7.505759091	7.082435283
9.694925798	9.465659495	9.318228697	9.35728196	9.168828475	9.584100455
9.145078374	9.552159131	9.963504081	9.791460222	9.636929838	9.606954152
7.817596162	7.675893758	7.912742707	7.894965162	7.808339242	7.924502232
9.549314313	9.388273121	9.848945263	9.451212687	9.829119768	9.683334444
9.172421828	9.535633242	9.281923404	9.702758343	9.382882596	9.113305192
8.26142704	8.449722687	8.098302202	8.195856758	8.296772869	8.205892515
7.683318778	8.735755485	7.636262586	8.055171202	7.929905606	8.352537101
7.133560273	7.145208253	6.514326051	6.569606636	6.511476192	6.606438778
6.790568566	6.978602455	6.393862758	6.485740606	6.279884111	6.526793081
12.14507673	12.39973853	12.30789527	12.44611991	12.14789109	10.80782516
9.444645091	9.288419414	9.569854202	9.552159131	9.208914778	8.463578343
7.064817384	7.51061902	7.93437196	7.649291899	7.715032394	6.811363364
7.039966242	6.75222999	7.32489101	7.258984768	7.282945384	7.427086202

6.846305687	6.319492374	6.817526586	6.855446939	6.702513515	6.928307859
8.231756293	8.953164051	8.256499515	8.394903626	8.384333949	8.006495333
8.555833323	8.083485404	7.222702545	7.546918172	7.443192556	8.318668263
6.768970657	6.497193293	6.794655232	6.650783061	6.335255596	6.976769172
8.533908051	8.689422596	8.024267525	8.143419303	8.018337859	8.265965545
6.35978	6.329109535	6.969423263	6.713538929	6.270498212	6.806225091
6.628304848	5.976914455	6.186180192	6.181284283	5.930729465	6.652654
7.665645141	7.722652869	6.929106091	7.138031798	7.160253414	7.884314808
10.78014639	10.90606985	10.1309568	10.37124354	10.4700758	10.46232362
6.754244737	7.542431869	6.662035182	6.756647869	6.550797374	7.121055596
7.507037566	7.527697455	6.990260788	7.300031424	7.444173596	7.680845586
7.778011646	7.397472212	7.742830394	7.845981061	7.64030596	7.445127778
9.411394242	9.121511687	9.246644323	9.591816061	9.576136707	9.425521616
12.16942168	11.4481816	10.49348589	10.58236152	10.54078574	12.10694646
10.45754412	10.42927797	9.895061889	10.16642595	9.982665242	10.22361636
12.48124823	12.57877126	12.62807191	12.80949032	12.65104366	12.94821125
6.702734242	6.770888697	6.925728848	6.692266071	6.484023485	6.885887889
10.26332497	10.49241362	9.94402302	10.04160563	10.17904437	11.03452834
8.559456798	7.803554182	6.984574354	7.365381232	7.22559698	8.290726727
6.895973717	6.279552505	6.164313111	6.131811687	6.764355354	6.792730596
7.21346404	7.269064929	7.755503707	7.173915838	7.133007667	6.490008293
7.782462626	9.143279535	7.573484121	7.932453717	7.741148566	7.436640343
7.581657414	7.813759899	7.961659859	8.367422212	7.963371182	7.845601525
8.507211606	8.308331848	8.127895253	8.40702104	8.609952768	8.655437626
7.30673204	6.578936343	6.264079364	6.366615303	6.359365273	7.463067838
8.44129198	8.30620801	8.12949599	8.033283465	8.46201601	8.627322343
8.014979636	8.049354232	8.099466788	8.25927404	7.855731	7.622739535
8.190353414	8.62991701	7.433916687	7.745118444	7.591410606	8.379056919
9.076382242	9.36439596	9.09178804	9.194378495	9.053706202	8.602379626
8.196571586	8.277405556	7.270153101	7.600942677	7.431389424	8.296772869
8.433939838	9.660200838	7.942409707	8.594455404	8.379884889	10.72384747
10.75178718	10.11246778	10.74819911	10.50543333	10.81034167	10.47123164
8.594455404	7.243824414	8.446184828	7.42612497	8.254919778	7.536179778
6.67377398	7.31523196	7.93888704	7.675893758	8.221010253	6.585931495
8.228946717	6.454504667	7.677613273	6.802083788	7.216973434	7.078835444
9.39974104	8.188779434	8.729972586	8.673363869	8.59655801	8.355387535
8.558556152	7.542783869	7.662615848	8.600831152	7.604463141	7.758641747
9.466909636	7.908308323	9.087255717	9.831087364	9.10215096	9.283736939
7.809338131	7.90331499	8.347717242	8.594861495	7.985767212	7.798826303
7.849821828	7.18421398	7.227304778	8.053971495	7.366006364	7.552833101
12.21991364	12.49170848	12.64708972	12.64708972	12.23505088	12.00086001
9.842388081	9.376046232	9.568406747	9.613873859	9.810255869	9.674270303
7.395300939	7.45523802	6.887565899	6.925728848	6.510752152	7.504556444
10.32309273	10.96886286	10.74558579	10.9088749	11.08435313	9.876880455
12.97977545	12.79024082	12.66627862	13.61616406	13.20536123	14.86987024
10.75737971	11.00183361	11.22456459	10.89224764	11.31053596	10.82034332
6.968393455	7.350114798	6.665174586	6.960749798	6.890761263	7.205228253
7.887186596	6.691325636	6.495692828	6.79225503	6.632781101	8.084275677
5.979536485	5.861153535	5.835234071	5.861397838	5.834101232	5.956868202
11.8164662	12.12151457	12.41931404	11.40306525	10.95093974	10.96748398
8.827377505	8.653441707	7.758192424	8.029005444	7.78118699	8.447526838
8.348974465	10.08799683	8.01602699	8.658285303	8.626833051	9.524156586
8.126700889	7.938205586	6.747074889	6.912798808	6.863661697	8.326162172
7.540114586	6.347049828	6.392953667	6.501455758	6.354771242	7.549557061
8.546056747	10.67190595	8.697732283	7.858214495	9.258612929	7.743818828
5.784506404	5.822012439	5.787295909	5.857602626	5.827323949	5.793304727
12.03602449	11.43208235	10.62142174	10.91766747	11.13551505	11.81018832
7.691535778	8.171788646	7.068665737	7.213178616	7.223246162	7.942409707

9.473962505	9.30577901	8.85308204	9.266794162	9.242021586	8.882379495
9.151976485	8.93587504	8.104500051	8.377562293	8.200539131	9.455015404
7.647631172	8.852576737	7.303307	7.482291222	7.588938485	9.511857455
6.413868758	6.822666394	6.287025869	6.39076103	6.235905444	6.106968071
11.11388317	11.33657706	9.424273283	10.60743282	10.37026232	9.617464455
8.617840212	8.925229707	8.258860253	8.478263182	8.43006898	8.157901343
7.090758091	6.825325	6.312971121	6.448115818	6.485102909	7.330801667
9.246644323	9.326238727	8.903602263	9.185497838	9.192533242	9.094076404
10.67815746	8.708995576	8.80133401	9.480384485	9.224452333	10.57996368
9.07419996	7.630594101	7.367541707	7.110215707	6.956131545	8.945002717
11.30690717	8.909404788	8.185563303	10.56306707	9.907546465	9.092457495
8.697732283	7.981345788	7.598252121	7.531511131	7.496050374	8.362537455
9.156840283	7.732550404	7.529686949	7.51310204	7.426469556	9.099242404
6.534227879	6.784609556	6.415074455	6.767263616	6.572638727	6.232553091
8.89604703	8.216823323	8.579336081	8.197805768	8.556855828	8.464081152
8.276014121	7.789573343	8.211847566	8.250194202	8.231756293	8.10683704
8.356302828	8.827377505	8.363862202	8.889380424	8.142197384	8.020484273
7.585815263	7.309336424	8.225261616	7.652274172	7.101573333	8.285312424
9.91449603	9.61181703	8.47107903	8.918756414	8.725555596	8.576430182
7.836828747	7.188044747	6.714476606	6.788765101	7.229587778	8.045722828
10.86447048	10.67939021	10.84974643	10.75453103	10.63870636	11.12613667
9.776037091	9.441908576	9.748306121	9.979092404	9.793942798	9.221852535
6.601717263	6.463509232	6.931501667	6.495272566	7.017237545	6.526565121
9.054276	9.660861778	9.467500556	9.094691273	9.096395636	8.744081071
11.23116994	11.57567047	11.77802923	11.52824	11.45002291	10.85782556
10.49466708	10.51886057	10.0672481	10.54565144	10.19528987	10.89224764
8.716864899	8.586731747	9.026117657	8.755916071	8.699656879	9.023639636
7.314025869	7.44169298	7.817975838	7.809338131	7.480029465	6.455790798
7.250899535	7.585815263	7.446082798	7.304975899	7.133560273	7.521572545
9.525471414	9.709426838	9.007843273	9.288999586	9.631972414	9.682548525
13.55640443	13.47832004	13.58996905	13.78737352	13.70717091	13.2158469
6.430261071	6.686319576	6.260257263	6.363888626	6.261769939	6.709797242
13.1944969	13.58209631	12.43279956	12.79024082	12.85589012	12.76628311
11.81221198	12.38063147	11.59870577	11.87445957	11.85301976	10.49009312
14.91973608	15.21350605	15.26374094	15.24813401	15.19830883	13.78027417
7.317304515	7.158304313	6.750367323	7.011783192	6.89207597	7.267388222
7.54731098	7.714642404	7.28582396	7.688011354	7.466006556	7.876418838
9.206093556	9.237064687	8.191573747	8.493653778	8.408718253	9.361074667
6.997949788	7.531209667	6.552098808	6.853717071	6.873334899	7.127947263
10.52476206	10.1985269	9.109120374	9.752732475	9.486281202	9.677492576
16.09492117	16.33688632	16.04217659	16.06827159	16.12384164	15.72204116
10.0841781	10.5152666	10.65014074	10.70534521	10.71860391	9.763952101
7.424024737	7.221024394	7.254644071	6.406814333	7.46337499	7.360165061
14.19920898	14.38889659	14.67552064	14.5513462	14.31917195	14.17411554
8.748883111	7.06104997	7.237499505	7.11962903	7.002421121	8.924680667
12.96662998	13.1944969	12.95787308	12.71520416	13.5360606	13.23864174
7.456122566	6.941957697	6.757434848	6.843046434	6.995294596	7.865908465
7.394027687	7.09298796	7.178905394	7.41439101	7.145208253	7.870978364
7.281724869	7.118205404	7.299714313	7.442271202	7.438935576	7.538797101
9.207219232	8.863920758	9.019731374	8.545617303	9.640429374	8.932594273
8.571194222	8.389653889	8.591239465	8.776574232	8.750916455	8.268052495
9.17294002	9.361671283	9.669847545	9.86640601	9.576740677	8.851571051
8.573727141	8.622644364	8.509502414	8.640789455	8.621272737	8.537544354
10.13193078	10.4322159	10.18563382	10.11664425	10.36465285	9.61181703
12.45390404	12.9709957	12.61449239	12.34855768	12.7494647	12.30167305
9.575401111	8.622197111	8.788135667	8.591651626	9.257979848	9.955574404
12.02514953	12.99088515	13.50997822	12.19086998	12.81735602	11.97944932
13.36582475	12.91656586	11.98999321	12.02240324	12.37087917	13.44126502

6.883278424	7.32157099	7.012040828	6.864210101	6.697631071	6.679714263
8.542859455	7.017803152	6.704711566	7.012040828	6.738778758	6.968936475
7.320252869	6.578006293	6.327350374	6.47341896	6.392522354	6.973893828
8.109636222	8.327353333	8.73262002	8.066374202	8.004282899	7.218159515
11.17041501	11.22294817	11.89361272	11.33848921	11.33848921	10.86820493
7.566119051	7.684641212	6.912798808	6.956363394	6.80380202	7.056715131
6.996042242	7.344013535	7.400314747	7.295094879	7.276913788	6.716343364
10.04511943	9.645545465	9.247858869	9.707282667	9.329342374	9.988725727
12.46055504	11.21958266	9.468772697	10.39414857	9.886762101	11.46320141
7.289911515	7.741148566	6.619525869	7.163563677	6.994489162	8.696280707
8.291166909	8.116928465	7.242080394	7.616841162	7.592689374	7.969427899
9.781348374	10.04511943	9.824540899	9.952967535	10.19757035	9.549314313
8.900779434	9.205404404	9.923012889	9.36748	9.034403333	8.346198293
8.320817869	9.586238192	9.118059475	10.0406788	7.842359758	8.246087697
9.35067002	9.992117576	10.5029511	10.26224218	9.488971182	8.708995576
7.291703707	8.521696414	8.217667525	9.023639636	7.282635596	6.983154111
7.059356717	7.256041222	7.804724687	7.301506111	7.098240939	6.99955204
12.73731843	12.63888703	13.86604428	13.23864174	12.66627862	12.54906863
12.80602179	13.15645152	13.62784329	13.10985871	12.797444	12.60022648
9.275532697	9.857940545	10.22267877	9.966143818	9.652169778	8.676316919
7.391342576	7.195224778	7.237206848	7.618249414	7.556609313	7.228468091
10.72384747	10.53842125	9.102956136	9.738398616	9.626304798	9.916104475
9.402230293	9.378583414	9.876096838	9.687048303	9.405539273	9.641007131
9.392082222	9.671170444	9.807061475	9.240082808	9.710962273	10.2829717
8.63611798	8.545091384	8.39227803	8.582586091	8.244006505	8.527598192
9.353057535	8.567801737	8.121211444	8.236217485	8.201745222	9.173615515
10.64379219	9.980047091	10.89224764	9.923012889	11.12315281	10.30358984
6.376277596	6.102326556	6.231781505	5.881206263	6.351287939	6.754943232
11.15183093	11.29076429	11.38025327	11.70812672	11.33100665	10.78014639
7.664275131	7.57318396	7.758641747	7.994919717	7.744777657	6.988716071
7.618887111	6.832629131	6.827661505	6.963620111	6.935824606	7.853350485
11.6295292	11.25523353	11.38775416	11.55524693	11.52824	11.59870577
7.923048354	8.012698303	7.768055657	7.737346818	7.601973414	7.005900293
8.095746162	8.749910828	8.670447949	8.31912796	8.852092202	8.468898455
7.466006556	7.062669222	7.030688768	7.400588576	6.848276364	7.553468394
6.77698199	6.821271707	6.794399263	6.967234434	6.932196717	7.008416879
7.32489101	7.610976808	6.405475687	6.695720192	6.636479121	6.047018687
9.981718727	10.09795649	10.57282784	10.20748734	9.720489222	10.41224321
7.561342071	7.249375465	6.494395354	6.808256808	6.355148343	7.914251515
8.523456192	8.229734556	7.611330343	7.688370495	7.605411525	9.421059
6.700579303	6.776493808	6.957403667	6.576798515	6.588688525	6.81164602
7.90290904	7.715405939	8.201303758	8.110881556	8.152087687	7.663954061
10.65751844	10.77387872	10.35178281	10.91176188	10.65751844	10.69793218
8.844262616	9.514496606	9.815893677	9.505258828	9.412044242	8.458294828
8.992957212	8.24281297	8.038729465	7.911995121	7.903688606	8.732044667
8.135177152	7.859293	9.112697707	8.877161475	8.796713535	8.142197384
9.889177586	11.84027237	11.86401838	11.68243664	11.72592113	8.717867636
9.626304798	9.741357455	9.537497323	9.600185283	9.815129384	9.348136535
6.74783701	5.81589399	5.983325222	6.554821889	5.998584	6.315590828
8.631899566	9.147897788	8.831800202	8.630379313	8.618302869	8.950874253
7.742436303	8.604635949	8.546056747	8.36556403	8.298896434	7.521266808
7.55945201	7.200432616	7.859670455	7.473746677	7.575754657	8.229339455
9.682548525	9.245445293	9.158023	9.380979222	9.724047525	10.042425
10.15186663	10.59180142	9.164000111	9.809529798	9.917043434	9.969141677
8.587234515	9.067856838	8.943348051	8.981975535	9.084956182	8.344098929
10.90606985	11.79171062	11.71042621	11.77802923	11.85889751	10.0079343
7.458795919	8.319901444	7.266003808	7.493254434	7.485478828	7.770465818
7.474059606	7.123473687	6.680656707	6.814458515	6.678083162	7.059676455

13.50997822	13.84455043	13.84455043	13.47327752	13.54965001	12.73731843
9.252443646	10.05880999	9.278597747	9.568406747	9.489614919	9.629839465
13.35359128	13.46712151	13.55640443	13.30795266	13.65450155	13.67554111
8.079973212	7.656238101	6.97366599	6.995294596	7.09469803	8.620285051
7.30556202	7.066488111	6.385648535	6.627578354	6.58104199	7.116341071
7.474397404	8.091725323	7.055635202	7.247912131	7.20666299	7.177177071
11.33848921	11.54470354	11.39382595	11.17378617	11.5737214	11.2278827
13.45466248	13.65450155	13.10484893	13.40302459	13.74093847	13.38447499
8.967408879	9.612498475	8.976374343	9.189045596	9.143918798	9.314493212
8.164575929	7.919201576	8.152443121	8.429582192	7.930919909	8.529888505
16.7804214	16.7804214	16.14978837	16.4534382	16.36806566	16.82803465
9.983540212	9.816676162	9.57181996	9.820433545	10.29101087	10.08145981
7.599486586	6.649897606	6.672562737	6.55275403	6.61398898	7.391952354
6.976769172	6.919943293	6.786032343	7.307562737	6.851123798	6.683791949
6.165117384	5.975084869	6.097663737	5.999647091	6.439991313	6.03924
8.25570701	8.340252101	7.903688606	8.158968566	8.144908747	8.12156602
6.942257576	7.098240939	6.986766929	7.003455566	6.706413566	7.028809465
8.095330333	7.705120374	7.530291586	7.133007667	7.278747747	8.024633293
8.887684293	8.344098929	6.817257404	7.174677505	7.001869131	9.123297364
7.988582505	7.88825004	7.432695485	7.645321707	7.659186848	7.692217293
11.1031408	10.17904437	10.54565144	9.794631646	9.831834717	10.687913
14.28325373	14.14208591	14.06476988	14.1830221	14.26676228	14.48025691
9.831087364	8.216297535	8.191204879	8.049354232	7.825767899	9.750460616
8.695335444	9.257979848	9.835081424	9.198436465	9.220211768	10.09079019
10.8405808	8.927019859	8.826846768	9.499247869	9.430738808	11.24816417
14.79546777	15.18392271	14.86987024	15.13975511	15.03840752	13.98831971
6.647920455	6.506975192	6.728207192	6.838224475	6.719039212	6.522106242
6.471263556	5.896752626	6.518976111	6.723002525	6.282381596	6.783830859
7.672658859	6.994245051	6.341887717	7.082160636	6.812925505	6.793900848
7.180839586	7.410622798	7.275223848	6.796104869	6.764115131	6.906817121
12.31719428	12.61449239	11.35278885	12.08128174	11.6295292	11.76804793
11.54675633	11.96362869	11.46539851	11.5571076	11.63401423	11.54871614
9.852239606	10.1597097	9.826032111	9.912769303	10.29660096	9.031155364
6.969423263	7.323124384	7.686071172	7.370079717	7.259306869	7.260895414
11.43005301	11.71244686	11.41415047	11.31449746	11.2518391	10.42062148
7.129597273	6.415074455	6.120218535	6.525823616	6.380219	7.317304515
7.790242404	8.534432515	7.74007802	8.228946717	8.097566687	8.337114525
9.221294354	7.810269677	8.334162556	8.102124455	8.37004801	9.402814081
9.273022949	9.196132455	9.329342374	9.287277576	9.328711051	9.656571869
7.204610747	7.208855192	6.449932949	6.531066697	6.380424646	7.624419899
7.952386505	8.375668162	8.328992859	8.26142704	8.419866586	8.005727263
7.275765808	6.403079273	6.425764848	6.200189737	6.218237869	7.294038515
10.37215733	10.65014074	9.58708204	10.64623997	9.921211657	8.946596606
7.949400121	8.191909586	7.895961929	8.107705111	7.822730929	7.605411525
10.8254891	10.45996909	8.931557869	9.330464051	8.878776172	10.79956984
11.36793803	11.2518391	10.81540317	11.04316622	11.18800276	10.98186414
13.41514643	13.77352346	14.05136004	13.80033164	13.85099976	13.63435472
6.156655838	6.018805323	6.138834828	6.096608202	6.002892646	5.769052182
8.594016788	6.721088758	6.693279889	6.893115293	6.971547919	9.135096212
7.731542758	7.56165098	7.600615697	7.434204455	7.188612899	7.648616949
14.33555643	14.66395575	14.6970922	14.6970922	14.84412351	14.4335752
6.587380232	6.06264496	5.938137475	6.046867384	6.096447404	5.996965293
7.512765949	6.618022394	7.347141657	7.259665485	7.757234778	7.523206263
8.426780919	8.592622071	9.083185818	8.799348343	8.705608919	7.943747505
6.739790818	7.388945192	7.083013909	7.08589403	7.120197273	6.953109798
7.541750556	7.358075697	7.158590636	7.413101485	7.386471808	7.344278889
6.378366909	6.437041	6.755924879	6.637469778	6.514775394	6.013541848
8.044980354	8.543686687	8.970028374	8.456191465	8.683087172	8.783158111

7.689234404	7.608041616	7.87472	8.112525414	7.748914677	8.137479222
9.593075545	9.479751879	9.473962505	9.58708204	9.490338525	9.566949
7.645645939	7.868007081	7.061330929	7.431067202	7.328126596	7.352900667
10.29542336	10.17702971	9.400967465	9.756054152	10.04862183	10.40486524
7.110215707	8.35018502	7.244714616	7.456765576	7.795765162	8.658802051
11.7349036	12.01427079	11.1612024	11.46320141	11.69891546	11.12315281
8.622197111	9.256767414	9.785983535	9.453743545	9.471411141	8.445759657
6.727962222	7.247912131	6.763342091	6.639045768	6.716824636	6.76944904
6.351456879	6.051299838	5.92647101	6.062490404	6.044883525	6.054709576
9.956562586	9.747577162	10.14631732	9.748306121	10.25627793	9.650789909
7.195502081	6.870354606	6.784609556	6.84607197	6.574738525	6.904630343
10.13479405	10.27139209	10.29949835	10.1300726	10.28089537	9.890949131
6.912541192	7.344278889	6.795847303	7.061330929	6.911201424	6.871904646
10.91514001	11.16734497	10.98350754	11.07158908	11.26446827	11.00053539
8.928157121	8.562227051	8.315227333	8.772410586	8.964642636	9.230202616
11.47127063	11.78265022	11.64244603	11.69658017	12.00354228	11.46145043
11.33477589	11.87445957	11.07337622	11.05033578	11.55928848	11.64044206
8.179581869	8.322342051	8.376704939	7.761767192	8.808345061	7.977207111
8.181963182	8.636577313	8.703733141	8.774537848	8.979298788	8.035675313
12.43600068	12.32659908	12.46466592	12.60022648	12.75396286	12.96241124
11.64848719	11.14211434	11.38970236	11.30506756	11.12454893	11.36065784
11.26815786	10.687913	10.94353341	10.96000427	10.79956984	10.99469779
8.825911939	9.087865687	8.688838939	8.718321818	8.975838535	7.795116566
8.059832677	8.484240687	8.60808904	8.445297313	8.518658667	8.112525414
9.35371997	9.638947465	9.136296333	9.458377051	9.418269535	8.327353333
8.685201879	8.751405818	8.374810828	8.551311374	8.733083212	8.768300525
9.979092404	9.670530667	8.927019859	9.828404283	9.448606737	9.70798304
10.23160947	10.62764398	10.76983035	10.16454544	10.17613521	10.19528987
8.918202434	8.563941778	8.435650677	8.690381253	8.814101707	9.660861778
6.928307859	7.232456929	7.052221778	6.888155525	6.638362242	7.513736343
8.588536172	8.161911788	9.314493212	8.712915394	8.395296687	9.436898869
11.68700199	11.29744903	11.2608846	11.32185019	11.4413808	12.59323492
6.951169364	7.047335818	6.454080828	7.060794313	6.923167202	6.74783701
7.708254091	7.353244222	7.496345141	7.576037556	7.486746556	8.149448323
8.935320828	8.998420707	7.57799899	8.131433848	8.108523939	8.995584808
11.94745099	11.79405389	11.85301976	11.73710477	12.11562205	12.34237091
10.04776272	9.507772687	9.408173051	9.811091061	9.746145949	9.829822242
6.791799101	7.073657889	7.472500828	7.034867141	7.33858799	6.89207597
8.986432162	9.555296242	9.001272616	9.311440606	9.369545939	8.738283333
8.129874121	6.973893828	7.159453505	7.217289505	7.020179424	7.153761556
10.72246175	10.3494333	10.6488688	10.47242114	10.4302696	10.36032821
11.38577951	11.81221198	11.39772243	11.59427705	11.81018832	11.42178201
13.20018245	13.30795266	12.92937388	13.50411017	13.34204398	13.16824615
7.442908828	7.350388939	6.898002222	7.114036293	7.338325414	7.831854586
9.953782444	9.547339828	9.670530667	9.226128374	9.792275424	9.568406747
7.434843313	7.102116364	7.137450808	7.406536232	7.341339747	7.147872253
7.160791343	7.290204495	6.675226152	6.815737535	6.758967323	7.313451111
12.52819783	12.62807191	12.55983323	12.51066587	12.65819872	12.48835313
7.148192263	7.832540091	7.773582384	7.752360667	7.982442283	8.173308798
12.20640951	12.4571909	12.21991364	12.38063147	12.36473556	11.91912022
8.82024202	8.765233303	8.203586121	8.523871909	8.879330566	8.063461202
6.416313212	6.784076586	7.196057152	6.845030273	6.959189505	6.537747374
7.414093131	6.895207929	7.330801667	7.030426444	7.67197899	7.732550404
15.56435861	15.56435861	14.46162431	14.89443037	14.88238784	15.54332231
11.31267384	11.73271713	11.44643795	11.48061491	11.87687449	11.15808934
7.149903152	6.79323798	6.878445485	6.286870242	8.019434586	6.229843364
10.55333109	11.51465712	10.76857843	11.69440164	11.62494396	11.60680648
9.07526898	7.607714535	8.047188455	8.750916455	9.742174424	9.686378667

10.17702971	9.600185283	9.793126455	9.860587788	10.1752482	9.618256808
13.03496614	13.2158469	12.97555423	13.13522491	13.2504616	12.78645268
6.785316677	8.079179394	6.60386102	7.156444778	7.182015707	8.378142071
8.719245818	8.313601758	7.170232747	7.65951298	7.365381232	8.953653768
8.39308204	7.783110646	7.374563525	8.41552702	8.369612525	9.024707707
8.831800202	8.621689657	9.255548899	9.025691242	8.925229707	9.769994879
8.938439909	8.408296424	8.836542848	9.096395636	9.171801828	8.720163182
9.092457495	9.246644323	8.660166475	8.91530399	8.818695101	8.624974828
7.962680434	7.162716495	6.851123798	6.965960869	6.996769242	7.962004212
6.897441	6.507801384	6.826634828	6.872611121	7.126269081	6.893369586
6.186368	6.206802879	6.46756804	6.387499121	6.444835424	6.326506525
7.470009697	7.359287172	6.250664576	6.445674283	6.413679545	7.483574061
8.664725051	8.088117667	9.339095222	7.945111576	8.851018515	8.220225172
6.543902859	6.376882727	7.066488111	7.026621727	6.507425384	7.061925202
10.43440935	10.45041513	10.12408141	10.17904437	10.1300726	10.43564423
6.910146768	6.475338556	6.743573939	6.623921737	7.339519202	7.209485919
7.468171747	7.736964596	7.784888444	7.056715131	7.500501152	7.917722121
8.304980606	8.43006898	8.586731747	8.529417101	8.399553939	7.919201576
15.38120792	15.70163214	15.46469304	15.70163214	15.81050193	15.63778652
9.080683394	8.862225737	9.037616626	8.960773354	9.026117657	9.464440242
9.139095455	8.899254879	8.743617505	8.873520545	9.027136657	9.760900556
7.405524869	7.434204455	7.159962485	7.217862778	7.469040515	7.483852838
5.980959273	5.929687869	5.934799364	5.942271616	5.847349172	5.806384394
7.232737253	6.853717071	6.43322899	6.259132677	6.756647869	7.369510384
14.27397716	14.57091557	14.34357305	14.62217632	14.49002723	14.15092087
12.59323492	12.89113428	12.60396528	12.68097339	12.80949032	12.75784116
8.00874802	8.03601997	7.651222556	8.055916879	7.579015667	7.832217929
13.64789782	13.5360606	13.28559105	13.42153085	13.6823979	13.78737352
9.966143818	9.598893818	8.725555596	9.071276071	9.082448343	9.978126798
12.72610186	13.03496614	13.41514643	12.58612865	13.15645152	12.32364634
10.4450719	9.169862626	6.744595263	10.042425	9.167758485	8.029005444
9.921211657	9.93099102	9.408808788	9.285000091	9.647889364	10.57649171
7.831854586	7.90505396	7.555333263	7.637262101	7.616235111	7.08777197
13.25686564	13.29117716	12.92542832	13.20536123	13.29684189	13.32669092
8.864955909	8.767872848	8.251922909	8.64507902	8.421350475	8.690918616
8.875206121	8.94874	8.716387192	9.435722162	8.62412801	8.384837869
6.263697566	6.809755232	6.308703535	6.718779485	6.535075677	6.726910778
10.74690378	10.69931975	10.21375666	10.93789932	10.78408516	10.51291939
11.15342364	11.66708917	10.84844593	11.11230451	11.26623333	11.89130603
11.18291631	10.95533553	10.78705857	10.8443678	11.04464523	10.72516317
10.21279484	10.30569114	10.07857135	10.08799683	10.42736719	10.75453103
11.66441291	11.99535117	11.79405389	11.84027237	11.90152869	11.58005536
13.73439002	13.43523914	13.40302459	13.50997822	13.71423947	14.11997744
9.225515485	9.420326576	9.300367869	9.139724848	9.378583414	8.982611727
11.33292491	11.22600179	11.45526933	11.40495249	11.24001011	11.28370047
10.77637516	9.883591758	9.412044242	10.20859114	10.66229262	11.24993851
10.39641485	10.96311364	11.00754401	11.04886125	10.88546605	9.993897848
12.92542832	12.96662998	12.71520416	12.73352188	12.91656586	13.41514643
13.47832004	13.69473258	13.63435472	13.54221797	13.7211242	13.42888134
12.46797904	12.84197228	12.80602179	12.78645268	12.83405786	12.4956701
15.95256028	15.76560628	16.25437967	16.12384164	16.28343863	15.88528111
11.69440164	11.61814895	11.18291631	11.96362869	11.71659127	11.64637724
7.614831859	7.163563677	7.230900293	7.311037152	7.154318051	7.853698798
7.488273475	7.601304687	8.841037687	8.08972896	8.327353333	7.960192616
13.68884286	13.89331098	13.83772389	13.71423947	13.87958112	13.04381444
11.48655657	12.06397065	11.90152869	11.70138789	12.0838996	10.93935201
8.520243879	7.861189172	7.716479172	7.884314808	7.880750535	8.556855828
7.220780818	7.408375899	7.923778798	7.679923515	7.55945201	6.908859909

8.102484232	8.157542717	8.670884566	8.119679141	8.176718545	7.813414727
7.28497204	7.170490939	7.664275131	7.447087758	7.510027525	7.349811747
6.98587896	6.890996051	7.724369343	7.292849111	7.330192545	7.945472162
8.880853535	8.287081333	7.165241424	7.511268929	7.249695535	9.356058495
8.939019091	8.908935182	9.295516616	8.945493505	9.786685869	6.997069465
12.38063147	12.70390423	12.85589012	12.4956701	11.96636849	11.7349036
7.785940475	7.962680434	8.93638097	8.177906263	8.326162172	8.141366242
7.626793707	7.149049263	7.355080212	7.352232505	7.430781919	7.096955818
10.41123643	10.09682592	9.895856061	10.00262223	10.17789322	11.05356995
15.97330865	16.25437967	15.61926687	16.17943014	16.22759729	15.83165575
12.12436236	11.38970236	11.06123261	11.3290504	11.54871614	12.57107442
8.903025495	9.380979222	9.132651727	9.136890505	9.162016475	8.335469222
7.028313949	5.890469384	6.013024788	6.191632212	5.997303687	7.374563525
9.482360616	9.954681949	10.06071544	9.847335909	9.69725304	8.630877737
7.420173404	6.674272111	7.237838192	7.184502222	7.264510162	7.273224596
10.64623997	10.45375986	10.45877561	9.850553616	10.96311364	10.46664928
14.1282639	14.223913	13.71423947	14.01682491	13.81266894	13.27940818
14.38889659	14.15092087	14.63205333	14.76275492	14.7172184	14.84412351
13.63435472	14.06476988	14.15092087	14.1282639	14.24162707	13.40990013
9.763952101	10.37686258	9.296131263	9.533033172	9.789131121	9.438799404
8.112876232	8.240781051	8.337550606	7.98690202	8.086153313	8.307483899
7.738299929	7.578291899	7.375790182	7.678591667	7.778362485	7.089925434
7.579959303	7.634226727	6.833840465	7.235668455	7.05481101	7.515531818
12.36473556	12.87819387	12.66985986	12.72610186	12.66985986	12.02811467
8.029836697	8.498167798	8.380828141	8.379510323	8.140977576	8.551311374
11.89876792	12.13867659	12.02514953	11.95514832	12.16036216	11.78265022
6.920433444	6.726910778	6.332263313	6.559042364	6.615401818	6.584604515
7.735579636	8.398231626	8.91530399	8.200163707	8.459227657	8.715845505
8.974650333	9.225515485	9.230770667	8.905887879	9.341222778	9.014846778
10.14432724	10.45150222	10.5752557	10.5397012	10.55726192	9.495686737
15.99508933	15.78787594	15.70163214	15.83165575	16.25437967	16.25437967
10.90057618	10.75600216	10.74945851	10.80644997	10.68338253	10.83930015
8.008022949	6.985632404	6.716118697	7.621022111	7.356007838	6.929106091
7.743818828	7.848096879	7.91707401	7.498343283	7.708922879	7.612909768
13.34204398	13.7211242	12.86955734	13.64083669	12.95303817	12.21991364
11.75662781	11.81018832	10.67572435	10.69682519	11.24457024	12.51066587
8.724013404	8.439612798	9.089024899	8.703733141	8.793874616	9.042944374
13.20536123	13.05987966	12.81735602	12.89513437	13.06488572	13.21058954
11.70812672	11.75462574	12.03912361	11.48459908	12.25572441	12.25572441
7.332669495	6.361759	6.298836091	6.304082879	6.473654737	7.363288879
10.38738452	10.22361636	9.916104475	10.25519474	9.973623707	9.799317404
14.20688311	14.17411554	14.25895571	14.27397716	13.76702458	14.223913
7.681826051	7.688370495	7.724673141	7.559825556	7.684641212	7.696259101
6.871158202	6.676440081	7.09469803	7.214035101	7.195224778	7.247912131
8.229734556	9.545881535	8.126700889	8.361327455	8.141770949	8.19621002
8.258860253	8.044608626	7.487940131	9.011040293	8.069753788	7.560401535
10.60635433	10.41731261	9.567720788	11.14866374	9.570470929	9.228437333
8.050105303	8.743617505	9.071276071	8.31318196	8.096910323	7.567065616
7.269892283	7.092080626	6.605683505	6.849574919	6.755440343	6.926979111
8.390560192	7.90403798	7.675499444	8.053619525	7.624016909	7.850890354
6.523815232	6.537953232	6.486382556	5.897552566	7.053922394	6.386460131
6.825072616	8.378515131	7.44801498	8.215875455	7.295094879	6.758395253
8.516311424	8.593101434	6.941957697	6.712585717	8.181574081	8.383846919
14.39736901	14.53168901	14.77222727	14.29173122	14.99939481	15.56435861
9.300367869	9.523397737	9.318836889	9.511857455	9.525471414	8.897177899
6.175139333	6.419328293	6.800782586	6.587802667	6.396985434	5.989763313
8.218887434	8.299695162	8.170586434	8.498167798	7.910617626	8.390560192
11.72592113	12.18792926	11.92396137	12.01673563	12.3613163	11.84027237

8.194999071	6.79984598	6.758676202	7.025866354	7.025021455	8.45110203
15.36066179	15.65613201	15.38120792	15.50564301	15.58150095	15.36066179
12.68097339	12.9851923	12.97977545	12.87819387	13.00101781	12.66627862
8.909404788	8.783584889	9.130293909	8.833337414	9.045755677	9.296718121
12.15752365	12.44264271	11.76336308	12.09497108	12.09804166	13.07947925
9.146178758	9.243679384	9.091299889	9.585510535	9.333361616	8.339546939
7.935468505	8.611710212	8.731005677	8.247392788	8.716864899	8.147857556
13.03025597	13.12562627	12.99565877	13.06967433	13.32021166	13.69473258
11.45526933	8.435650677	11.24993851	8.449722687	9.065009323	11.85889751
9.011040293	8.760676242	9.392652323	8.131862263	9.878686616	8.08075902
12.40343679	12.37416009	12.04990566	12.23191382	12.47761486	12.67378338
6.29528098	6.004508838	6.770611535	6.485310808	6.575874879	6.240116697
6.893115293	6.085762192	7.473746677	6.432439828	7.580266434	5.950149222
7.845601525	7.883917657	8.565878434	8.344441616	8.667554273	8.286128505
10.27804811	10.4429644	10.36903954	10.08978265	10.33146686	9.886023253
8.851018515	10.36589334	9.328020364	9.273640758	9.007167758	8.911109919
11.17910752	11.542568	11.82346178	11.68700199	11.52238659	10.92485902
13.84455043	14.1830221	14.24162707	14.08841453	14.35197184	13.48401144
14.52085241	14.45281427	14.9727616	14.77222727	14.56207529	14.59173737
12.60022648	12.6076243	12.33907422	12.44264271	12.73731843	12.63888703
6.706906717	7.461509737	6.774175343	6.51319704	6.84909404	8.089360869
8.239474646	8.436995596	8.102124455	8.049019253	8.294235768	7.392836818
8.302062758	8.710391727	9.366312899	9.016491202	8.743617505	8.84935699
7.538207606	6.625311455	6.515423162	6.629250202	6.486382556	7.414093131
14.86987024	15.12351424	15.24813401	15.02501841	15.16803204	14.58095582
14.10328164	13.81266894	13.61616406	13.63435472	13.90036211	14.4185289
5.782680606	5.787675232	5.778826879	5.797202354	5.792870909	5.785760051
12.4956701	12.36759935	12.39660011	12.29886409	12.63102018	12.85589012
8.951989212	8.742635939	8.205892515	8.433468657	8.320357909	8.593101434
7.653990838	7.975414556	7.820222687	7.732206152	7.579350515	7.416977434
12.10694646	12.59668127	12.53197264	12.3774088	12.65472241	12.50328361
13.57574016	13.44126502	13.30255942	13.39037726	13.58996905	13.66158872
9.341222778	9.088505869	8.010523545	8.71485401	8.356302828	9.821195081
6.363508222	6.260478586	6.613208707	6.510269939	6.620737576	6.530171141
11.59654933	11.64848719	11.88148631	11.52645916	11.83072395	11.35080354
11.93486375	12.29275278	11.37847359	12.27773509	11.68948846	10.85361094
9.382205394	9.723272263	10.1250404	9.763138212	9.755162434	10.02644328
13.65450155	13.54965001	13.76108056	13.81935116	13.77352346	13.45466248
9.652939283	9.742174424	9.262561	9.40167604	9.546645747	10.03983393
9.777520899	9.830354566	9.761765848	9.727055768	10.02923217	9.070661273
13.62784329	13.79381649	13.46005067	13.43523914	13.78027417	13.81266894
8.526649808	8.78767504	8.05474504	8.368780919	8.40783399	8.195856758
7.506719576	7.842359758	7.267131253	7.453956636	7.604109192	7.204326061
7.038648475	6.771840333	6.69036997	7.102414636	7.209485919	7.448630646
8.911109919	9.288999586	8.409136758	8.581169111	8.302900242	8.039068889
6.962574283	6.406575848	6.248527828	6.34064704	6.282981828	6.741723879
7.586712596	7.379463242	6.747964717	6.859461465	6.865318939	7.028313949
13.94443	13.90036211	13.80704919	13.74093847	13.95955505	14.34357305
9.980047091	9.861430101	9.472607081	9.835081424	9.293710889	9.258612929
13.07454735	13.35919327	12.59323492	12.99565877	12.68903729	12.27101
7.238429162	8.744802939	7.914251515	8.683580283	7.709201212	7.608647091
7.738963899	7.234805667	7.589604303	7.459405444	7.446413172	7.579015667
6.557678737	6.97366599	6.707842586	6.832875889	6.874988444	6.859220677
7.445127778	7.822730929	8.170954818	7.88791003	8.027386222	7.541090081
6.905316545	6.557407798	6.574738525	6.696930808	6.828622869	6.906092707
12.82153585	13.02006671	12.72610186	12.76628311	12.94367069	12.59668127
10.57758511	10.10278369	8.517695303	9.324974768	8.93587504	9.923012889
12.5521346	12.65819872	12.63888703	12.42233701	12.75784116	12.52461444

12.2293359	12.44950322	12.19996238	12.15752365	12.5521346	12.23769992
6.977041091	6.169292545	7.140009717	6.167683182	6.97366599	5.806757808
6.228213626	6.506079909	6.490008293	6.318496152	6.496103798	6.069800253
10.21157382	10.04421951	10.74172297	10.82664672	10.55966675	10.06356315
6.457381818	5.785111636	6.813169384	5.967318737	7.000309313	6.126729404
15.02501841	15.16803204	14.9591327	15.01206542	14.83117585	15.18392271
15.13975511	15.34617167	14.9847254	15.05177986	15.61926687	15.58150095
15.26374094	15.26374094	15.449005	14.78379092	14.80749806	15.15443825
12.24649354	12.57486869	12.25572441	12.32659908	12.51066587	12.35168631
14.46162431	14.6970922	14.36999747	14.36070414	14.61202705	14.77222727
8.106418192	8.052783737	8.367840636	8.379056919	8.361327455	7.568036061
7.880363606	8.261007747	8.818695101	8.157542717	8.544134919	8.124973859
14.76275492	14.4185289	14.33555643	14.57091557	14.74028573	14.9591327
8.927019859	9.363823202	9.937540263	9.576136707	9.756840071	8.63611798
13.85817003	13.97489728	13.52959744	13.67554111	14.04335209	14.07245549
8.366462404	7.923778798	7.968277051	8.761644081	8.227704253	8.133538364
8.410410394	8.426330949	8.020096283	8.090157434	8.352537101	8.267698071
8.593101434	8.298396535	7.125146293	7.516462556	7.427701768	8.843148071
7.231823384	6.814222283	6.859220677	7.829693939	6.909401626	6.880775747
11.86922315	11.80554878	12.41634374	12.17513939	12.14507673	12.10414281
11.88649013	11.66441291	11.671419	11.86401838	12.0750029	11.98999321
6.764835354	7.16725497	7.101573333	7.262654505	7.154937303	6.557678737
9.675185677	9.766921414	9.61181703	9.491039162	9.899010949	9.588410576
15.01206542	15.05177986	15.13975511	14.9847254	14.89443037	15.13975511
7.684267263	7.410364737	8.345353354	8.134366646	8.372433475	9.046362323
11.44491584	11.56725755	11.26446827	11.29597305	11.57765358	11.1031408
9.591816061	9.01707996	9.177504333	9.219562515	9.368875727	9.575401111
13.32021166	13.18858184	12.75784116	13.04869312	13.27418186	13.58996905
6.561721071	7.075769859	6.373171747	6.548235576	6.389892586	6.04559901
12.66985986	12.85048632	12.67378338	12.69316533	12.89886333	12.66167346
8.067838768	8.771294657	8.305787747	8.528915121	8.77085696	7.52646604
7.74007802	7.681488071	7.795765162	7.571116455	7.488894061	7.60255999
12.35168631	11.92396137	12.20960187	12.08943903	11.98999321	12.11841559
8.727989172	8.807484626	8.020484273	7.885731566	8.323157747	8.843670869
13.28559105	13.26910509	13.00581854	12.97555423	13.03957854	13.1944969
15.58150095	15.81050193	15.95256028	15.58150095	15.83165575	16.42659037
6.955441212	6.521008828	6.596206051	6.651250333	6.461471737	7.304711495
7.970926879	8.025425616	6.970224646	7.364797848	7.141717727	8.734416606
7.365664505	6.877631798	7.909031556	7.747885899	7.193299919	7.394691081
7.693213707	8.015388182	7.481960152	7.661155364	8.02698897	7.76948301
13.78027417	13.67554111	13.87263802	13.85817003	13.81935116	13.52334642
12.03310273	11.91651973	12.14507673	12.0724233	12.03068847	11.82616001
13.70717091	13.81935116	13.66158872	13.77689882	14.02572128	14.00297944
10.27905981	10.25038968	11.11856721	10.76239578	10.84302834	10.91514001
6.569841374	6.688196	6.887875313	6.584832545	7.042911333	6.437041
9.048100778	8.886096818	8.226500707	8.608532485	8.511305758	8.672348475
6.699888758	6.538933859	5.900628	6.284325212	7.026391172	6.180211313
16.57174008	16.57174008	16.73234105	16.68963031	16.96059838	16.61084374
6.951745222	6.802328384	6.917518081	7.337098929	7.606043535	7.385541293
11.9577766	11.76101506	11.33657706	11.59212007	11.55106122	11.62059475
8.476485121	7.693516556	6.839911636	7.167545535	6.983407384	8.037664566
10.12041459	9.721175788	10.02923217	10.08521343	9.662314131	9.538263677
11.98999321	11.82616001	11.86125412	11.78498903	11.27499716	11.36793803
8.150096222	7.88791003	7.12483998	7.131581374	7.247290424	8.043387838
11.50425191	11.64044206	11.43773109	11.51465712	11.71244686	11.91416468
10.21375666	10.36903954	10.3779146	10.20634509	9.542425758	9.803356646
15.12351424	15.02501841	15.34617167	14.80749806	14.94534759	15.46469304
9.582110717	9.655770101	9.578067192	9.35371997	9.739859192	9.586238192

9.338458455	9.935661677	8.633848111	9.030619364	8.94874	9.335272182
11.92156179	12.44611991	11.91651973	12.39058663	12.04160066	11.01209098
12.34577038	12.65104366	12.10414281	12.40343679	12.11841559	11.45176908
9.413942061	9.987077505	10.10736711	9.800904051	10.08700287	8.810986707
9.421059	9.417037424	9.977292899	9.861430101	9.279246253	8.08972896
9.787586646	9.556759333	9.780583495	9.988725727	9.516398909	10.08599138
8.117339677	8.178268222	8.087781242	8.015388182	8.314030071	8.21550303
6.822217707	6.282801404	6.193578313	6.282801404	6.19283997	6.509844303
5.95265099	6.045042838	6.478552354	5.973367646	5.904609677	6.017125566
6.560443758	7.02750098	5.984555323	6.252758313	5.975569111	7.633560879
7.610574101	7.738613697	7.171874212	7.293451808	7.159715333	8.094218354
7.764199131	7.351279444	7.656877838	7.369510384	7.876138384	7.272599475
8.284876758	8.132293172	8.130993828	8.291166909	8.305787747	8.453639616
6.743803525	7.248766182	7.31258798	7.33350399	7.256596263	6.397416404
10.156858	9.917863333	9.907546465	10.24828231	10.23752804	9.344138121
8.845226141	8.620285051	8.356302828	8.970028374	9.009407333	9.280305798
9.406893465	9.23383296	9.605576293	9.454390232	9.095162899	8.785177889
13.99495772	13.62784329	12.78645268	13.28559105	12.92937388	12.6076243
7.062669222	7.145769434	7.253230737	7.341641707	7.298118263	7.238770545
7.118793697	7.267888182	7.214922364	7.221869232	7.472789818	7.315791616
6.908354899	7.635576919	6.55006301	6.769202101	6.669677444	6.351069404
6.522534343	6.94407103	6.383834626	7.185402798	7.390207394	6.554351
7.518430525	7.767362636	7.817596162	7.91465098	7.371573384	7.899333596
7.579015667	7.272016424	7.612909768	7.709201212	7.79851096	7.436336172
8.161911788	8.320817869	7.575132364	7.724369343	7.702311758	8.00874802
7.048729333	8.231756293	7.221626808	7.600615697	7.599870798	7.725703576
7.056486293	7.394983566	6.518533202	7.274624242	7.449616394	6.882764848
11.27153548	11.1060681	11.03737422	11.49636188	10.60507136	10.96460614
8.211411717	7.915639949	7.115757364	7.592689374	7.328721848	7.918848717
6.615630737	6.900488939	6.585712788	6.087012455	6.878694758	6.513435323
10.8947391	10.8254891	11.11714857	11.10170724	10.70534521	10.6362571
7.66930299	7.697940283	7.809001414	7.975072535	7.764609818	7.119927869
9.279768394	9.437491071	9.139095455	9.21905796	9.555296242	8.910531141
9.440510152	9.617464455	9.526870273	9.759214424	9.645545465	9.517771202
11.4481816	11.83330316	11.60479972	11.71659127	11.70138789	11.36423723
10.40178901	10.40278823	10.63014735	10.61133106	10.58689262	10.62764398
13.50411017	13.50997822	13.87958112	13.77689882	13.52334642	12.88265743
7.879657162	6.962574283	7.048974677	7.326942212	7.244122677	7.730484909
13.74759592	14.34357305	14.17411554	14.19920898	13.86604428	13.7211242
8.783158111	9.579413505	8.049019253	8.523456192	8.348974465	10.2861211
12.53563192	12.89886333	13.09463792	12.81379045	12.93862501	12.62449502
15.29549959	15.90458538	16.06827159	15.92931651	16.17943014	15.70163214
8.006153172	7.966742939	8.339546939	8.018337859	8.140128606	7.405823253
7.102982434	7.459115707	6.79225503	7.050073121	6.923412444	6.885064545
6.780968242	6.816827283	7.040799717	6.276797273	6.883542434	7.661901192
7.623693616	7.770465818	7.173324505	7.906781182	7.415309101	7.988251818
8.19458801	8.493653778	9.069558111	8.687053253	8.654464586	7.404680798
8.675789182	8.475210818	8.023866626	6.824345162	8.015705778	9.303966081
7.222702545	6.99155401	7.268778051	6.911201424	7.095534354	6.654772152
8.789683394	8.616914869	8.513135323	8.760291222	8.811517808	9.276789111
8.118136869	7.681826051	8.000521869	8.252372808	8.307483899	8.182386152
7.141146879	7.543124535	7.657544313	7.212383152	7.082724758	6.958444212
8.943861384	8.257595869	8.912003131	8.82024202	8.825312061	8.721054424
10.29432836	11.04464523	10.88809182	10.51400776	10.82664672	10.2187154
11.47682743	11.308834	11.38178745	11.68484165	11.22119175	11.01683173
9.287819051	8.972927475	8.78563097	9.481000434	9.09013302	9.646274414
11.79870316	12.26475658	11.8164662	12.10414281	12.32024961	11.8358519
12.25251148	12.21991364	13.00101781	12.63102018	12.4956701	12.61449239

9.636296737	9.397189192	8.837022465	9.052474394	9.051375475	9.619644949
7.771540485	7.757532545	7.239398515	7.437384141	7.368846081	7.615524929
8.288815495	8.204737434	8.333227545	8.300103657	8.138156535	8.549970081
10.41846051	10.41022215	10.36134852	10.38001468	10.36032821	10.67305197
11.86652814	11.55524693	11.9577766	11.79171062	11.68243664	11.58421758
12.29275278	11.84529477	12.75396286	12.24097391	12.67378338	11.93975968
7.101832475	8.32939098	6.621578222	6.936369818	6.962034242	8.239069586
7.826151263	7.41731598	7.55499601	7.649620657	7.447389475	7.121365859
8.085049192	8.148682091	8.030614293	8.092218303	8.188379657	8.228946717
7.991889949	8.255317818	8.576430182	8.437898414	8.229734556	8.130283051
10.00094823	7.363288879	6.665428859	8.852092202	8.071703475	6.952808172
8.160644788	8.758871	8.754441596	8.559456798	8.953653768	7.471847707
9.997192202	10.23850383	10.46442497	10.08145981	10.23066525	9.056062667
8.391460737	8.41598997	8.274698687	8.436995596	8.525321657	8.023099172
9.449948384	9.643287657	8.761644081	9.135096212	9.296131263	9.469429253
9.876096838	9.70798304	10.41846051	9.814415596	9.757590192	9.918671212
7.470886646	7.534556657	7.086698091	7.062669222	7.028809465	7.26571197
7.108492263	7.005077596	6.502744273	6.531486374	6.481897646	7.164102909
7.000099141	7.432376434	5.957826909	6.018143222	5.938900657	7.785940475
8.970028374	8.648914657	8.638487899	8.635209293	9.184527788	9.263851586
6.830856061	6.855446939	7.388278253	6.895441525	7.221024394	7.037248667
7.242951465	6.607135434	7.381652111	7.284431525	7.32157099	7.169717707
10.88430477	10.65365596	10.57996368	9.925979414	11.0387546	10.83790729
8.967895455	8.502509434	8.879851283	8.874191697	8.819236758	9.240082808
7.362680232	6.912798808	6.631383222	6.940977263	7.211808657	7.644626202
7.751645273	8.198176101	8.143776848	8.104500051	8.23139899	7.168394253
11.09565519	11.79615897	11.80448851	11.47322198	11.73271713	10.56548536
7.600615697	7.869033162	7.90505396	7.569899606	7.678926152	6.882244081
7.912742707	7.584620515	7.526167626	8.147481596	7.608934747	7.817975838
7.236914141	6.677606	6.424247404	6.438298737	6.380003717	6.945626949
8.503061444	9.447991384	8.34368697	8.522156747	8.39227803	7.90505396
8.599954677	8.124212202	8.570262	8.528049141	8.630877737	9.168324747
9.751080646	10.35069541	10.08978265	10.1421691	10.06356315	8.909404788
13.12023156	12.92937388	12.48124823	12.94821125	12.71830686	13.50411017
10.68338253	10.30058205	10.41022215	10.46342714	10.40486524	10.65751844
7.312858101	7.661901192	7.20666299	7.753435232	7.28903701	7.849463182
9.810255869	10.33890802	10.31095185	10.28190294	10.0672481	9.019287687
11.49239347	11.62739451	11.75215821	11.86652814	11.65455063	11.16579624
10.46664928	10.39521192	10.4302696	10.75062885	10.53737043	10.85923156
7.48289603	6.818961091	6.622230818	6.698743475	6.652192667	7.446413172
8.082778869	9.02886299	8.575095707	9.008373061	8.873520545	7.255477111
7.656544636	8.247392788	8.364710758	8.264240727	8.181196919	7.263217283
8.456191465	8.578393657	8.053207788	8.208296687	8.115023838	7.889381343
11.66708917	12.06692054	12.34237091	12.23769992	12.43279956	10.90466263
9.749004051	9.493200828	9.320176919	9.59460096	9.322592778	9.895856061
9.552821717	9.587719636	8.470647758	8.89604703	8.713417869	10.0143126
6.244370242	6.905826838	6.124834283	6.09420704	6.018143222	7.359873626
10.7442872	10.52247938	10.1421691	10.73788216	10.23550694	10.98749259
8.10140802	7.858964434	8.037984616	7.939609869	7.742113232	8.22437102
6.442753616	6.409033687	6.543419131	6.644015919	6.431116323	6.775968394
7.853698798	8.053207788	8.185943182	8.154705535	8.029005444	7.759566061
8.752912545	8.980444384	9.322592778	9.019731374	8.908705232	9.781348374
8.919862162	8.476085384	8.468898455	8.901331343	8.514909313	9.013314737
8.704708384	8.239069586	7.555972677	7.525850576	8.063008152	9.165786899
9.863774293	10.00094823	9.198999222	9.762542495	9.62845904	9.881176566
13.27940818	13.26276274	13.32669092	13.26910509	12.84197228	12.39660011
7.411268949	7.536179778	7.499556727	7.446760616	7.684267263	7.327495141
9.402814081	10.00361533	10.15283227	9.315671101	9.491725303	8.775001677

8.143040162	8.430929525	8.620790505	8.47014499	8.34982299	7.617189485
9.993897848	10.07107569	9.517771202	9.62302801	9.942152202	10.62536143
7.353560545	7.467207283	6.873999848	7.10996603	7.459405444	6.337620818
8.757866131	9.013314737	9.35728196	9.435122859	9.058895657	8.214296636
8.424934535	8.427236737	8.02698897	8.948168879	8.438325768	7.546592828
9.657223606	9.525471414	9.698181576	9.641787333	9.561667293	10.49241362
8.743124222	8.457898556	8.381309202	8.448456354	8.129067798	8.830117929
10.0188097	10.27714677	10.47242114	10.32531651	10.20859114	9.897493586
11.62059475	10.26332497	9.380344141	10.9102587	10.46342714	10.61743608
9.869466131	9.596773313	9.677492576	9.776839404	9.952967535	9.869466131
11.62739451	11.39382595	11.72355631	11.65261158	11.60888892	11.60479972
9.912769303	10.17336172	9.806332162	10.06173934	10.35284213	10.15379546
9.091299889	9.885156737	10.02644328	9.552821717	9.621550919	8.493155899
8.607626293	8.679578434	8.421546263	8.577417788	8.660166475	8.823394192
8.261781515	8.337114525	8.420871687	8.735287485	8.546056747	8.303356141
7.292849111	8.108523939	7.547922717	8.194999071	7.299074768	7.437975364
7.127106434	6.485516717	7.324298343	7.000309313	7.366646525	7.01565997
8.579336081	8.139691131	8.493653778	8.45110203	8.090488788	8.82789604
6.759458	6.922161646	6.661016606	5.819100101	6.636058242	5.800970263
8.208692758	7.532708636	7.03699504	7.245014414	7.127106434	7.83046296
6.625743253	6.703725465	7.172411071	6.936097616	6.783830859	6.358295333
7.805068949	8.226840717	8.571194222	8.374363061	8.223899788	7.250545919
7.846324646	8.358783152	8.00874802	8.227704253	8.286610323	7.279669313
6.965182525	6.742790232	6.567411212	6.879229626	6.736085162	6.803567182
8.817758687	9.16271003	8.282900293	8.524356212	8.35667099	8.155393222
10.94496034	10.03983393	9.012031232	9.537497323	9.321890899	10.67939021
7.755132808	8.112525414	7.734550869	7.694916828	7.552449879	7.403028737
8.63794701	8.700606657	8.829514889	9.065009323	8.955880859	8.59026096
10.20060626	9.93663897	10.07956055	10.32750324	9.783680727	10.09795649
7.893940788	7.272331859	8.179581869	7.21064097	7.821271606	7.457359414
8.121954505	7.843077242	7.859293	8.099825778	7.873714818	8.387083515
8.078044465	8.432150758	8.687969051	8.230588212	8.468898455	7.576996152
9.365016101	9.339764051	9.324388263	9.505859384	9.603529667	9.779012071
7.337989111	7.639261768	7.404355202	7.494510091	7.381652111	7.158043384
6.577039475	6.247784949	5.952164414	5.89831598	6.025570495	6.866852303
7.513736343	7.111428596	7.171336586	7.238133434	7.62509899	7.18832599
9.698896848	10.12610261	10.08599138	10.13580598	9.62024901	9.334658071
11.42562271	11.57178154	11.05112773	11.34202326	11.24993851	10.96162926
7.358688323	6.551008899	6.320079859	6.412381	6.293717162	7.37546403
11.671419	12.25844332	11.77053658	11.94234713	11.09089144	11.67371856
7.42612497	7.381950828	6.990487535	7.130479657	7.096340343	6.821005303
7.877119465	7.909763434	8.244805758	8.376150616	8.044980354	7.377888556
7.945472162	7.595878485	6.781955545	7.122697263	7.021219939	7.964547263
8.864430475	9.125507586	9.005433152	8.775001677	8.675789182	8.460488909
8.397853727	7.936644384	7.471219899	7.420173404	7.368501293	8.30460302
9.227275152	8.551311374	8.477856384	8.860747333	7.898205273	9.489614919
12.85589012	12.00086001	12.08680505	11.84529477	12.04734694	13.05987966
9.193130899	9.136890505	9.435722162	9.286739242	9.372705202	9.232683343
7.192707293	7.266525586	7.571427586	7.341966626	7.325162263	6.936097616
6.861931182	7.042119313	7.090449141	7.086698091	7.127365253	6.553437202
6.853963192	7.150454879	6.953635141	6.874245465	6.876638434	7.356281616
8.498503737	8.53537201	8.844732879	8.620790505	8.366948404	7.969046636
7.288128061	6.783136778	7.31937996	7.875433545	7.801006182	7.60736902
7.439506505	6.342479778	7.438305869	6.756145354	6.975188859	7.303836939
8.378142071	6.465379212	6.894135505	6.656675747	7.861832121	9.005433152
8.01423197	8.383846919	8.574218515	8.352944343	8.562227051	7.735579636
7.667309091	9.247858869	8.250622253	8.40783399	8.793339475	7.369172101
8.301264212	7.709511535	7.666604434	7.283562505	7.961295828	8.39446903

7.214315212	7.25632596	6.568165434	6.604981172	6.710946737	7.603719626
7.629673333	9.152615889	7.073141899	7.654890798	7.68296396	9.685649929
8.833337414	7.763846354	7.486088616	7.314926455	7.190930768	8.902484929
7.174161374	7.08136002	6.979690485	6.948047899	6.823157384	7.10076196
12.16627503	10.26541026	10.042425	10.3986043	10.30058205	12.17829278
13.42888134	13.27418186	11.66189432	12.3843369	12.11216258	12.60396528
6.894957566	6.664678717	6.708797384	7.399358222	7.176635455	6.615162343
7.59656001	7.536554758	7.54731098	7.648986848	7.686444293	7.560760293
11.55322016	11.01683173	10.84705809	11.25898056	11.52645916	12.02514953
6.276975525	6.512183152	6.317094394	6.534648919	6.490840394	6.97809596
8.955880859	9.926784556	9.217871889	8.487847515	9.414554808	9.241421747
11.88375452	11.85033795	12.4956701	12.70002263	12.34577038	11.49420288
7.622357354	7.400015081	7.684965778	7.604779273	7.905419798	7.530291586
10.4322159	11.01531103	10.42736719	10.70902317	10.52364746	10.49140257
8.802954374	8.778623323	8.798247798	9.276189121	8.814624313	8.718769576
7.502072596	6.928572354	7.549557061	7.44049799	7.756175162	6.94614002
10.46342714	11.20498985	11.45932673	11.46539851	11.12613667	9.601568657
8.035675313	7.906781182	8.365106485	7.946141838	7.730154939	8.039451586
8.372433475	8.222643394	7.709201212	7.924885333	7.893631939	8.083806798
7.757532545	7.898205273	8.426780919	8.072924455	8.157542717	7.636934919
13.21058954	13.22730043	13.05428976	13.23341018	13.29117716	13.15082863
7.450888848	7.659186848	7.099574394	7.408681949	7.340147596	6.899979889
9.258612929	9.036076414	9.083741606	9.147897788	9.428845576	9.690534253
8.27861104	8.846355515	9.434425707	8.926362727	8.998420707	7.557551899
7.503972212	7.455822202	7.507037566	7.652952818	7.88825004	7.393766606
8.147481596	8.295009141	8.660608859	8.145194646	7.904381697	7.931708101
12.9851923	12.58287167	11.84529477	12.0089388	12.25844332	13.77352346
9.715870253	8.473802646	8.981479273	8.662363172	9.014335657	9.428845576
7.699362081	8.078389566	8.434806414	8.237787788	8.085771838	7.423140192
9.857940545	9.585510535	9.883591758	9.777520899	9.844817354	9.342353505
7.736260545	8.465000141	8.696739152	8.559975162	8.509936919	7.424653535
9.388273121	8.686679798	9.16271003	8.930433505	9.041820404	9.980047091
8.500240172	7.868722081	7.300950758	7.327234414	7.380085202	8.483763566
10.78269205	10.80389925	11.63401423	11.19114259	11.54675633	11.17378617
9.817379515	9.35728196	9.032817303	9.684886576	8.534897646	10.00002479
5.993422394	6.204170475	6.155698343	5.772825949	6.010748859	5.966829434
10.18000397	10.50166798	9.861430101	10.19528987	10.20452777	9.295516616
7.866656354	7.585519525	7.879985051	7.964914919	7.848409778	7.078303869
12.80166365	13.60269863	13.31405593	13.16824615	12.99565877	13.2504616
8.597538556	7.144299071	6.819416596	7.160253414	7.062959768	8.586731747
14.11151028	14.77222727	13.5964752	14.24162707	13.10484893	14.13521971
7.403336687	7.750957677	6.830578646	7.177177071	7.013931768	7.247605232
7.794365768	7.40526796	7.346280818	7.722297051	7.632188616	7.696527091
14.83117585	14.70594079	15.12351424	15.16803204	14.7528858	14.5513462
7.654890798	7.257203303	7.159147152	7.378508566	7.309928404	8.014979636
8.277405556	8.38301802	8.897751606	8.530333535	8.791137414	7.972012263
10.11452638	10.33596201	10.46108665	10.4283734	10.30695028	9.229670424
7.662925475	8.047933768	7.943433747	7.886480404	7.744459273	6.901740727
9.771430818	9.66613299	9.913634949	9.880317061	9.848227263	9.59387599
7.91707401	8.955880859	7.633913808	7.839249838	7.719466677	8.008400859
6.591596333	7.062438556	6.948047899	7.252580253	6.499744121	7.050347636
7.820934747	7.735250384	7.694916828	7.986161141	7.799196616	7.926719768
6.635108111	6.995550687	7.507381747	6.895973717	7.117115202	6.141879192
9.458377051	9.684048687	9.925979414	9.325601172	9.811898121	9.288419414
9.127872697	9.381544081	9.471411141	9.468134182	9.550085313	9.064430111
6.930987343	6.691055667	6.558602434	6.393414081	6.53573598	7.550845909
8.290726727	7.479422939	7.124332475	6.883793818	7.648986848	7.643628758
15.42966281	14.63205333	14.21449649	14.5413574	14.10328164	16.14978837

7.554702939	7.693828071	7.969427899	7.891531071	7.861189172	7.634916323
7.983913121	7.254341798	8.023506091	7.895961929	7.877487515	7.466342606
11.59870577	11.4535969	11.6445291	11.68038318	11.22947614	11.52238659
10.86566339	10.49592664	10.68072859	10.85100254	10.77890896	11.8164662
10.52799495	10.18000397	10.33696433	10.54950911	10.64502212	11.32185019
6.714000354	6.886756677	6.247099485	6.457625061	6.264838313	6.078134778
9.400967465	9.166446616	8.641737071	8.868452172	8.822853162	9.387059283
7.276913788	8.068931465	8.043818737	7.768402071	7.860050667	6.706206909
9.253086273	9.397864101	9.169862626	9.251353071	9.438094949	9.345614434
10.31693391	10.19957169	10.14999886	10.12610261	10.49592664	10.87235819
10.24942024	10.47479655	10.24612484	10.30358984	10.48310631	10.5199714
8.908475283	8.878776172	8.630877737	8.645985424	8.822318	8.865915939
10.85782556	11.00496707	10.49466708	10.71503879	10.60383217	10.59292728
7.843485626	7.630295798	7.718373848	7.737987747	7.853350485	8.120448121
6.34603899	6.061065343	6.950902859	6.502532889	6.908074727	6.856475414
8.998420707	8.933684374	8.544134919	8.687969051	8.899757485	9.002847848
11.26623333	11.38577951	11.24147284	11.17213305	11.38577951	11.37490296
10.64738653	10.92337564	10.72633412	10.77890896	10.96000427	11.16888788
9.880317061	10.05053825	9.631972414	9.532335293	9.70798304	10.53141254
7.40806004	7.306475687	7.327857495	7.219623727	7.213178616	7.845981061
11.48061491	11.52645916	11.38391359	11.28565422	11.59427705	11.56291631
9.762542495	9.595333525	9.776037091	9.486281202	9.90675497	9.748306121
10.05780386	10.25519474	9.681142253	10.1309568	10.16165443	10.16937171
10.34311059	10.32093584	10.22164118	10.44934262	10.53842125	10.38631174
8.042974687	8.300888657	7.892277636	8.245649515	8.22344601	8.271405859
9.04056096	7.404680798	9.792275424	8.953164051	8.568768141	9.507772687
6.719810283	8.397853727	7.350681061	7.828991111	6.940218677	7.377557566
7.198597879	7.483574061	7.826151263	7.355230333	6.962574283	6.591813727
6.772272192	6.450607838	7.430156263	6.276206182	7.56165098	6.85895202
7.416298071	6.989715687	7.218159515	6.832363455	6.689141323	7.151031394
9.173615515	9.385837242	9.942152202	8.964085616	9.591816061	9.186711646
9.372025394	9.061091758	9.961780586	8.808345061	8.491898242	9.260607
7.768402071	7.578628212	8.128726778	7.955993172	7.871272152	7.976470212
8.475612051	8.074096707	7.689572788	8.415109929	8.145962667	8.876650909
9.493894545	9.009407333	9.142791657	9.413321525	9.174120707	9.723272263
7.058212263	8.134790222	8.074904687	8.207537162	7.957018646	6.585495889
6.804729515	7.867668606	6.453428828	6.792957485	6.944336869	6.875236313
8.945493505	9.147296667	8.738735232	8.957944414	8.711304737	9.175472828
10.62895885	11.05796986	10.26116661	11.22947614	10.82416773	10.85654613
9.824540899	9.374121768	9.739859192	9.146732444	9.652939283	10.45996909
11.02551486	10.45877561	10.2572153	10.80782516	10.30470526	10.31597323
10.10187506	10.05523038	9.917863333	10.42927797	9.646274414	9.707282667
8.491898242	9.104371838	8.252794354	8.731005677	8.217984283	8.431326525
9.721175788	9.291911616	9.169326323	9.614560172	9.045168101	9.588958727
10.1300726	9.733950273	9.759214424	10.13276962	9.440510152	9.829119768
10.65014074	10.41224321	10.38208186	10.82416773	10.01524205	10.4655823
9.927686616	9.605576293	9.746868596	10.10454015	9.438799404	9.608350808
12.27421104	12.19086998	12.32024961	12.70787607	11.76101506	11.7894918
8.868979273	8.910531141	8.65914896	8.815152444	8.985325394	8.643636162
11.05651949	11.14866374	10.52689599	11.50261564	11.14866374	11.62739451
8.438325768	8.753927596	8.304169929	8.264694495	8.611286687	8.544633697
7.798177384	6.508927202	6.808747657	6.838696091	6.894135505	8.277763646
7.817975838	7.715032394	7.65784001	7.646953505	7.893273434	8.11773804
11.54871614	11.63837311	12.16036216	11.71895032	11.92396137	11.49239347
6.71590199	6.724801141	6.725273333	6.829096384	6.862647808	7.548285091
8.251125323	8.129874121	8.168587596	8.243245818	8.037664566	7.959080606
9.012760899	9.304577515	9.354902374	9.306330646	9.376667182	8.583996586
7.840343939	7.719466677	7.709511535	7.793372141	7.963030121	8.404822414

7.611330343	7.186960111	6.875236313	6.959689121	6.852150101	7.476910626
7.288414848	6.921167192	7.245319404	6.782643404	6.479217141	7.604109192
8.005065495	7.005354859	6.840363778	6.835371293	6.618511313	7.466006556
9.169326323	9.118629636	8.258465414	8.742149222	8.740682	9.526870273
7.278747747	7.901142576	6.744878414	7.042643192	6.976769172	7.789573343
7.312252182	7.610574101	7.505451222	7.641683626	7.369773828	6.494595232
11.238428	11.68484165	12.70390423	11.03452834	11.28370047	11.08756575
10.05688349	10.03701736	10.38329808	10.02061657	10.02439619	9.899010949
12.35798465	12.18492446	13.07454735	12.9709957	12.86955734	12.39058663
8.322342051	8.489178071	8.175288444	8.257595869	7.920494253	7.90869301
11.98440811	11.99323436	11.35657003	11.52052132	12.00644476	12.0750029
7.401155333	6.603596232	6.562721354	6.734222485	6.580572354	6.951961606
8.472925747	7.942071303	8.27903296	7.87816304	8.641223283	8.165787747
6.901187545	6.928862212	6.415480919	6.48058603	6.547344253	6.769202101
8.986932636	9.016491202	9.171164091	9.217871889	8.918756414	8.530767747
8.568768141	7.791533949	10.56548536	9.049171303	9.033915556	9.020337111
7.89560499	7.972411364	7.414093131	7.647332323	7.490430596	7.135797152
7.811371919	8.022720303	8.554027364	7.97471396	7.839249838	7.924885333
9.630502939	10.3896003	8.867918475	9.386484303	9.323731354	10.74558579
9.11093099	8.952561111	8.454954939	8.792768747	8.580722586	8.632799758
9.845595727	10.26632574	10.4450719	10.34514895	10.25220538	9.576740677
9.556000646	9.690534253	9.11510898	9.015412101	9.127872697	9.853021111
6.548235576	7.368501293	6.447481424	6.057890444	7.132751192	6.571898091
8.882379495	9.093001495	9.535633242	9.623794152	9.060618192	9.766131535
8.381754576	8.283330131	8.594861495	7.870669919	8.376704939	8.341518747
7.858964434	7.91707401	8.181963182	7.670275758	8.719668061	7.378186273
12.12939166	12.11216258	12.89886333	12.58287167	12.28985985	11.36979611
9.790672212	10.42622906	10.58347296	10.40810668	10.14631732	8.818210192
8.732044667	8.610845737	8.271795141	8.679578434	8.627796121	8.976374343
7.869992939	8.343220798	7.690840778	7.760294414	7.692888131	7.982442283
7.057330242	7.096340343	7.19681397	6.635568162	6.811363364	6.873566444
9.62845904	9.39974104	7.807154444	8.539328929	8.230181798	9.153730283
7.251769333	10.10542249	7.072618697	8.666601818	7.071771354	9.791460222
8.300103657	8.651452677	8.629474212	8.681579566	8.701614283	7.813018717
8.503455465	8.227704253	8.538380869	8.342368121	8.615330515	8.53579602
9.462343535	9.408173051	9.695719162	8.721054424	9.719000626	8.90531298
14.07245549	13.98831971	13.85817003	13.90036211	14.05136004	14.05855866
9.753529758	9.692793657	9.331038253	9.359783616	9.494490616	9.242021586
8.801904485	8.662363172	9.109760465	9.17861001	8.898231131	9.215289101
9.590351455	9.749660394	10.04511943	9.788274313	9.740584727	9.806332162
9.409395929	8.86638896	8.949784505	9.215289101	9.342353505	9.211184727
11.28896545	11.69440164	12.052647	11.51260306	11.49239347	11.38775416
9.101086141	8.65914896	8.230996848	8.558556152	8.337114525	9.496413788
9.290785283	8.289212586	8.467263475	9.035040848	8.314850485	8.554528737
13.82538175	13.07454735	13.20018245	13.82538175	13.26276274	14.21449649
7.939609869	8.086566798	7.828991111	7.568924192	7.692552364	8.264240727
10.52689599	10.84568098	10.75453103	10.14631732	10.63752253	10.27236099
7.774617596	9.362770111	9.105035414	8.657806242	8.705145061	7.755503707
9.566949	11.38391359	11.34202326	10.56908161	10.74045328	9.343457394
9.499247869	9.610538374	9.550769788	9.592426051	9.761765848	9.556759333
11.65918316	12.07810634	10.5152666	11.08435313	11.06573849	11.51008986
10.4549623	9.078331919	8.644074768	8.956909848	8.962353222	10.64502212
6.527907232	6.77206196	7.165793091	6.926979111	6.88247998	6.011815869
7.259665485	7.799196616	7.076844636	7.123768636	7.553773697	8.371229848
9.724814657	9.933588354	9.753529758	9.517771202	9.876096838	9.118059475
8.586230848	8.542859455	8.784649081	8.575566677	8.514431737	8.309247939
10.51771241	10.63266161	11.49239347	10.62536143	10.87637444	10.90606985
9.120933788	8.796713535	8.846850111	8.976374343	8.849981545	9.477759242

13.33661988	12.7494647	13.15645152	13.21058954	13.11515098	13.26910509
9.681142253	9.726443586	10.59062542	9.630502939	10.03026493	10.15585749
6.506514626	6.921417374	6.74432	6.764115131	6.737819111	6.539153576
7.953126222	8.593558293	8.215082485	8.455403657	8.180785838	8.429130081
12.24933389	13.32669092	13.85099976	13.5360606	13.17778072	11.11856721
14.9847254	14.60195877	14.76275492	14.86987024	14.67552064	15.10875812
9.946747141	10.3548828	10.31193963	10.30569114	10.11051676	9.632768747
8.271795141	7.28497204	7.076589626	7.216096788	7.105472313	8.699656879
10.61480751	10.66342196	10.96460614	10.73384189	10.89604789	10.82664672
11.31852116	10.93789932	10.77762269	10.69099899	11.01807842	11.94745099
8.849981545	7.590558081	8.054318061	8.53670504	8.280295667	8.539328929
6.621786404	6.642822768	6.134254273	6.266652758	6.232171323	6.880211293
9.456392222	9.116804101	9.180285111	9.377330121	9.386484303	9.447991384
9.736957071	9.819688798	10.12126634	9.691258919	9.618256808	10.54190997
8.744562374	8.551751717	8.396963808	8.706127333	8.721054424	9.054276
6.639986485	6.296703525	6.857999616	6.723501929	6.75007699	7.068134081
6.768172747	6.938883283	7.802836333	7.759936788	7.369510384	6.474717424
7.536554758	6.482726162	6.381219657	6.529524889	6.538425424	7.36694602
9.503271071	10.00186072	10.68687892	9.829822242	10.23247836	9.927686616
10.0728998	10.57996368	10.8947391	10.25906505	10.71731517	10.4450719
11.58621592	12.25251148	12.30167305	11.74935785	12.06692054	11.62290433
8.004655889	8.31214998	8.676791091	8.292509485	8.348543172	7.732206152
9.932690333	10.01524205	10.65634987	10.36589334	10.49140257	11.02403513
7.878518051	7.84876403	8.120062485	8.250622253	7.929187444	7.958393384
12.31089415	12.47130519	11.77552743	12.18492446	12.0724233	11.81221198
7.500167182	7.608647091	7.686775222	7.648616949	7.509397909	7.54731098
9.629101929	9.848227263	9.867911152	9.875361242	10.05970796	10.01974654
10.58117749	10.60044575	10.1985269	10.32418729	10.41957053	10.18098661
10.85513517	10.63014735	10.70534521	10.40178901	10.64738653	10.94786936
12.29548604	12.21312889	12.33576886	12.13867659	12.39348455	12.3774088
7.121055596	7.229904545	7.198026636	7.194186434	7.15072903	7.448310273
10.55726192	10.51650912	10.42622906	10.52364746	10.48782122	10.61001127
10.27236099	10.51771241	10.01703805	10.19418334	10.28795108	10.26632574
11.49029795	10.93494087	11.30506756	11.10761793	11.50632759	11.88375452
9.169862626	9.148511889	9.048100778	9.030004576	9.150811232	9.103811545
11.24323027	11.7349036	11.34732253	11.35080354	11.44319437	10.88546605
9.47593803	9.908305848	9.710962273	9.680349586	10.00002479	8.890373556
10.98186414	11.65918316	11.49029795	11.45932673	11.72355631	10.49348589
6.92551101	6.729937747	5.759696051	5.763621586	5.769464788	5.808313747
8.091323071	7.97284099	7.366317111	7.784199556	7.774617596	8.691458141
8.894491141	8.858692343	8.937429899	8.752460404	8.586731747	9.25368898
8.000144121	7.828584192	7.653305051	8.094605657	7.837132667	8.088956818
7.164102909	6.949065	7.098484101	7.168718192	7.380686859	7.301216929
9.605576293	9.461673394	8.666113404	8.881310737	9.251353071	9.62024901
8.842109	8.39402401	8.314398727	8.000883182	8.682574626	9.019731374
7.874403566	7.894315778	7.569223354	7.17606597	8.168196313	7.556609313
7.862831293	7.879657162	7.496684051	7.133298475	6.998704808	7.495088838
10.12126634	10.34717129	9.75438504	9.967178364	9.788274313	10.65246846
10.67939021	11.18291631	11.04316622	11.07493153	11.00053539	9.380979222
7.873714818	8.522641273	8.710842768	8.869521111	8.86024204	9.266188263
10.27714677	9.582761798	9.017695354	10.0060683	9.686378667	10.2026692
8.096525737	8.08537204	7.772843323	7.825767899	7.982067081	7.65457904
7.039421667	6.697172354	6.384073253	6.306878242	6.270128909	6.937881404
11.22600179	10.99186298	11.09721747	11.57765358	10.92757694	11.50425191
8.156410909	8.290353162	7.789881242	7.704350384	7.793372141	8.176718545
13.04869312	12.90783816	13.45466248	13.03957854	13.09045797	13.50997822
8.786134101	8.907915899	8.436545051	8.547862646	8.503455465	8.754900343
9.853774707	9.818860343	9.724814657	9.916104475	9.591133828	8.896578162

5.93060098	5.772686101	6.051158222	6.69943696	6.456691768	6.610225071
10.03796113	9.903372323	9.928455091	9.987874111	10.17224075	10.56084718
11.77802923	11.53050203	11.74935785	11.0473733	11.89628941	11.83813795
9.646274414	9.713858919	9.886023253	9.908305848	9.806332162	9.735412273
7.347141657	7.522869182	7.730873535	7.578628212	7.663618646	8.037287293
6.123671758	6.504501838	6.01801997	6.199844141	5.996585424	6.640442667
7.627373273	7.057634667	7.51247098	7.258693172	7.314300525	6.903241343
12.51766186	12.12692782	12.29886409	12.32024961	12.46797904	12.01191659
9.757590192	9.693515768	9.517013525	9.220211768	9.310205828	10.8193093
7.752687697	7.846688253	7.816254515	7.796747616	7.80651396	8.22479296
7.98285402	8.013857071	8.322342051	7.604463141	8.003147788	8.03490996
8.358783152	8.85049999	9.231349182	8.985325394	9.080683394	7.995673758
8.873520545	9.145664475	8.804477505	9.126080354	9.030004576	8.316969364
12.14191765	11.93770961	11.81018832	12.43600068	11.26990549	11.74439188
8.485174677	8.583996586	8.317824071	8.276867707	8.262181081	8.566871343
15.90458538	15.97330865	14.99939481	15.34617167	15.42966281	15.99508933
7.741457929	7.560118414	6.881514152	6.950648455	6.845568374	7.472500828
8.625486818	8.34982299	7.562285172	7.799854778	7.640998838	8.752460404
9.895856061	10.11452638	9.465071586	9.699589758	9.731806091	9.439917707
9.279246253	8.63794701	6.908859909	6.508047626	7.083613121	7.38400901
7.041650374	7.318216242	7.053026414	7.113452899	6.994769535	7.067022475
10.1752482	10.07670007	9.922126404	10.15875628	9.922126404	10.07580535
7.973586899	8.491898242	8.686126354	8.430929525	8.262596626	7.557888444
9.691258919	10.27236099	10.72900488	10.3918566	10.05144087	9.37540303
10.20532801	10.21375666	10.27432009	9.826032111	10.42297323	10.44094498
10.36465285	10.3090972	10.17122401	10.3896003	10.06997663	10.34418014
8.854095293	8.282438737	8.263464364	8.727989172	8.768300525	9.200042253
9.340550081	9.682548525	9.184000242	9.237664879	9.665316566	9.324974768
12.25572441	12.62449502	11.97660652	12.24649354	12.39058663	12.19727311
11.68243664	11.6445291	11.68700199	11.88909817	11.671419	11.9577766
9.698181576	10.03123363	9.896649636	10.25220538	10.22557955	9.137373131
9.587719636	7.501110505	8.373157505	8.205133263	8.376150616	9.528804131
8.465492313	8.181196919	8.090157434	8.235388636	8.504327141	8.707057788
8.980943495	8.893419636	9.133918929	9.248396293	9.263851586	8.803482152
7.436336172	7.399710364	7.338325414	7.341038	7.450888848	7.34804498
6.674020838	6.501455758	7.028809465	6.718256293	6.945089283	6.508699242
12.28105924	12.26775966	12.23505088	12.25572441	12.30789527	12.44611991
9.97079498	9.841562192	9.820433545	9.675904202	9.95201103	10.38430498
10.43757507	10.8935809	10.7442872	10.47370876	11.20498985	9.967178364
12.50328361	12.46466592	12.45390404	12.3613163	12.27773509	12.40735915
8.762742172	8.480158333	9.388912091	9.003925646	8.960773354	9.155642525
8.083135202	7.873714818	7.649926505	7.869033162	7.884680111	7.814093232
10.73384189	11.13551505	8.941359636	9.990505182	9.889177586	11.17910752
12.17216641	11.97196754	12.18492446	12.16627503	12.13867659	13.0147344
10.43872423	10.62142174	10.40486524	10.52247938	10.52476206	10.03026493
9.536330707	9.503858424	8.475612051	8.810446485	8.825911939	9.128533354
6.79984598	7.480029465	6.439789939	6.733232384	6.685084394	6.287217818
9.083741606	8.496365737	8.048299333	8.312729141	8.463578343	8.914132333
12.90359001	13.24400831	13.47832004	13.16151113	13.15082863	12.68484185
6.898002222	7.133560273	7.319616859	6.533325687	7.503692152	6.754460545
8.883414061	9.232683343	9.344897333	9.046362323	8.550897414	8.655934636
6.959189505	8.005371424	6.59116396	7.202115061	7.104662848	8.362142131
7.11489098	6.816537596	7.161342051	7.339519202	7.161605929	7.694540808
10.94942311	11.12151875	11.308834	11.2345748	11.27695531	11.09565519
12.12151457	11.82112126	11.0387546	11.39594259	11.10761793	12.63102018
7.655968232	8.591651626	8.807484626	8.510413414	8.480536717	7.441979727
5.751841727	5.751987646	6.328112374	5.754819929	5.755128288	5.754797667
7.037511364	6.725499141	6.066974626	6.18704099	6.077984505	5.936803283

7.933961929	7.613528424	7.926300626	7.879296202	7.94734596	8.191204879
9.329342374	9.11154599	8.387939343	9.79615097	8.482852263	9.119222505
8.774537848	8.852092202	8.587234515	8.661508737	8.566871343	8.500729384
7.202701576	6.705162535	7.087210828	6.69920203	6.508927202	7.118512586
8.402589283	8.075284384	8.157901343	8.59026096	8.058246657	8.511305758
8.843148071	9.013830768	9.421059	9.271759586	9.195011616	8.432612263
11.21958266	12.41634374	12.32659908	11.21958266	12.29886409	11.03296099
10.48074239	10.30695028	9.463745455	9.807061475	9.772980061	10.22668357
7.763504859	7.711891616	7.219623727	7.477241869	7.400015081	7.300031424
10.47479655	10.4450719	10.28710845	10.79284767	10.45041513	10.28190294
10.39747702	10.11150219	10.02269473	10.21279484	10.31013098	10.59062542
8.686126354	8.028141747	8.008400859	8.50111796	8.289996192	9.476588061
9.769223525	10.24038579	10.27905981	10.22951336	10.44094498	9.510536
10.26224218	10.76983035	11.01683173	10.76737368	10.83930015	9.949411172
6.41609998	6.842273677	7.580594545	7.067591	7.157774636	6.825870455
6.595504071	6.422838717	6.444835424	6.616628394	6.340852071	6.715104081
6.477937869	7.224435778	6.152834101	6.623632253	6.506308818	8.187593091
9.109120374	8.301657889	9.008373061	9.057196687	8.744562374	9.80784303
10.07764109	10.70534521	10.68960982	10.39072525	10.4994288	10.3548828
10.17122401	10.156858	9.799317404	8.956407626	9.859664869	10.84974643
9.087255717	8.800397414	7.552094283	8.305787747	8.120448121	8.448929687
6.483130091	6.992927808	6.113157071	6.503851576	6.240312677	5.869415929
9.731806091	9.967178364	9.918671212	9.998242414	9.55462702	9.461673394
7.079910848	6.141021192	6.624377646	6.201274	6.195589626	6.378366909
10.02741959	9.296131263	9.767642525	9.935661677	9.689783586	9.831834717
7.151567818	6.963620111	7.459115707	7.415948364	7.266813283	6.87893297
8.012698303	8.547449101	8.735755485	8.62991701	8.648450303	7.443557333
9.642500081	9.355403384	9.348735909	9.376046232	9.654354424	9.466909636
10.28710845	10.03299879	9.779738	9.899010949	9.734703111	10.43872423
7.412191343	7.846992646	8.382194192	7.898595626	8.247731232	8.629055081
8.572701232	8.789683394	8.680072242	8.775519707	8.687053253	8.154705535
6.688683899	6.563630475	6.77992704	6.582190566	6.694688636	6.245816848
7.870669919	8.140538697	7.36166898	7.557268586	7.507667889	7.715785354
7.907575465	7.851994586	7.690510313	7.700026646	7.771143222	7.927799515
6.732525747	6.420140667	6.963095333	7.065949717	7.308145939	7.449946354
7.460591212	7.826840444	8.188779434	7.847379061	8.127495596	6.884803323
10.81664077	10.47242114	10.43757507	10.80499832	10.49466708	10.91766747
9.779738	9.352487919	7.755825222	8.459596535	8.170954818	8.907429495
9.741357455	9.429506515	9.639623879	9.761765848	9.675185677	9.917863333
8.846850111	8.875206121	8.800397414	8.933180404	9.140392293	8.691979949
9.356741384	9.430738808	9.153730283	9.054860525	9.020337111	9.233272283
8.102862354	7.904747051	8.013487535	8.095746162	8.295436051	8.523871909
8.765233303	8.730448515	8.974101818	8.954798808	9.126663535	8.873520545
11.41071085	11.20660223	11.58005536	11.49420288	11.62290433	11.61814895
8.528049141	8.859179455	8.402589283	8.532980343	8.430929525	9.180906778
7.810269677	7.798177384	8.055916879	7.647631172	7.928125404	8.025051697
8.589388657	7.041650374	7.599870798	7.231823384	6.991849242	9.052474394
6.938149939	6.848831242	6.157181768	6.275248485	6.253857596	6.668510737
7.940254566	7.928481586	6.890245747	7.380377788	7.259665485	7.329578283
14.84412351	14.30038792	13.21058954	13.66784462	13.56886427	14.7528858
7.638981707	7.649926505	8.635209293	7.501443394	7.455517596	7.005077596
6.245816848	6.325549091	6.801276	6.365147848	6.299035566	6.159501303
6.473835768	7.031727121	7.547650101	6.83507799	6.988716071	6.859461465
8.321919424	9.607593848	9.953782444	9.310205828	9.528176495	8.324453253
8.222994222	7.796432081	7.94734596	8.032161838	8.074508444	8.060252263
11.47871073	11.60680648	11.68484165	11.62739451	11.47682743	11.39983314
9.211184727	8.87289497	9.489614919	9.35796903	9.11211799	9.354289121
6.719552727	7.147872253	6.381616303	6.584154061	6.59116396	7.112898869

8.107705111	7.839249838	7.796432081	8.126253929	7.858214495	8.657806242
11.03163187	10.78269205	10.88302044	10.88031852	11.03737422	11.31053596
11.07797425	11.0859227	10.80239406	11.06732533	11.2345748	11.47682743
12.28369472	11.89361272	11.71895032	12.06098883	12.43600068	12.84627851
7.62575301	7.202701576	6.971547919	6.998704808	7.080799364	7.456765576
6.996769242	6.87945998	6.380424646	6.409033687	6.422427222	6.723002525
7.015344242	7.462771485	7.920494253	7.873080636	7.641683626	6.663676879
14.15863941	14.5413574	13.98831971	14.33555643	14.1282639	13.35919327
10.71279176	10.68572341	10.59924244	10.94071069	10.39072525	10.67428006
9.235747242	9.222631263	9.407554697	9.477181455	9.332175414	9.336479586
6.782428768	6.595947525	5.965211869	6.159847303	6.054582838	8.346585616
9.134591697	8.858269384	8.064260859	8.468898455	8.287888051	8.943348051
8.118473111	8.499404646	7.776293606	9.053043889	8.006495333	8.165380303
12.51066587	12.94367069	11.65261158	12.26475658	11.74935785	12.17513939
6.90271597	6.27602701	6.25331604	6.29391397	6.154487859	6.479663677
6.514559495	7.180020091	6.464462626	6.582439566	6.62508102	7.493863788
8.893968333	10.37026232	8.662363172	8.880364303	8.886096818	10.70902317
14.14208591	13.5964752	13.02518615	13.64789782	13.47327752	14.9847254
8.418118394	9.673359929	7.962380919	8.514909313	8.316081222	7.701686677
8.365106485	8.444861909	7.201570444	7.543741273	7.51810503	8.667554273
7.555661677	7.379145515	6.507609283	6.611373919	6.548684566	7.61520499
12.38794592	11.69658017	10.58689262	11.31852116	10.83100199	12.49965499
8.281167657	7.683675283	7.127674667	6.95442902	6.893369586	7.875808798
8.739635162	6.817048798	6.909148313	7.185712515	7.109678444	9.220211768
6.806225091	8.001277303	6.435745475	6.486601465	6.875236313	6.532632323
9.054860525	8.51269104	6.975188859	6.947784242	6.861163889	9.451851384
8.257595869	7.975784071	7.693828071	7.890421556	7.869709071	8.158968566
8.591651626	7.82717702	7.908308323	7.953486636	8.039451586	9.246644323
7.318843495	6.243838475	6.371798101	6.130885768	6.221607646	8.064713051
8.784649081	9.238865747	7.857447394	8.197369283	8.03364202	9.482964828
6.025175505	6.842028788	6.253672202	6.189761525	6.25331604	5.84033399
7.320933798	6.533083232	6.202273253	6.247784949	6.173981626	7.515186091
6.710494808	6.969919475	6.906092707	7.032313212	6.700579303	6.503631525
6.914843414	7.540776061	7.326634626	7.925642	7.275765808	7.537240869
6.775501273	6.063067495	6.019631313	5.990113141	6.028808465	6.518759273
7.484516838	7.396159798	6.636727626	6.87945998	6.770374545	7.434843313
6.752731616	6.265203717	6.284325212	6.279014293	6.207516667	6.665428859
7.934739636	7.875808798	6.780426	6.983154111	6.772514131	7.91307104
6.494784798	7.223856	6.021467343	6.361367657	6.330318636	6.565175343
7.735932323	6.674468929	6.517856677	6.583409828	6.608710798	7.491688212
6.372351394	6.743803525	6.186736495	6.336250778	6.251220354	7.327234414
8.747892899	8.471953717	7.281402384	7.351625758	7.655611	8.552208182
7.066192545	6.816280253	6.112843636	6.32572904	6.298009505	7.326042475
9.622300838	9.993897848	10.16642595	10.12224713	9.817379515	8.646531515
8.562227051	10.36349344	9.191849071	8.818695101	8.532516465	9.153232364
7.721580051	9.024212859	7.910617626	7.734170172	7.228759818	8.082337909
10.99046505	11.41238356	10.3305428	10.51174846	10.31894523	10.87637444
9.368875727	9.751885293	10.61133106	10.01071956	9.950180667	8.826393101
7.954533778	8.070136677	7.727011939	7.614146707	7.665645141	8.077230061
9.013830768	8.985325394	9.023071899	9.082448343	8.933180404	9.278597747
10.74305691	9.945028121	10.82153564	11.11560115	10.29542336	10.58689262
8.664254273	8.606624646	8.881793333	8.720163182	8.526186556	8.777572838
9.433849505	9.489614919	9.458377051	9.318228697	9.679062616	9.164545343
8.103690354	7.450590374	7.45998899	7.684641212	7.581982414	8.258465414
10.29660096	9.762542495	9.398445515	9.307024131	9.183367525	9.991229606
7.81270001	7.741791646	6.841594566	7.178905394	7.089056525	7.514821949
7.218159515	8.53715797	6.965717616	7.462491444	7.273826505	9.514496606
7.107120283	7.213178616	6.491493939	6.563853182	6.400763333	7.041898303

7.742113232	7.696527091	7.390471152	7.543124535	7.645943414	7.564741838
6.506975192	6.405475687	6.636479121	6.601976232	6.126877838	6.570518374
6.607135434	5.996844343	6.445674283	5.849599879	7.755825222	7.532708636
7.498343283	7.443845071	7.41439101	7.503692152	7.68569196	7.639990515
10.69354222	9.041236566	8.809442889	8.568768141	8.595703707	11.26262606
8.286610323	8.377562293	8.189895404	8.087781242	8.707515232	8.396532182
8.06745399	8.517288667	7.40806004	7.816254515	7.786927414	8.220625586
8.083485404	8.467263475	8.029383646	7.770819434	7.841061444	7.60282502
7.083613121	7.688011354	7.623386535	7.053327354	7.154661646	7.020439899
11.74439188	10.48897537	9.660861778	9.798623747	9.556759333	11.41238356
8.499836475	8.870056939	7.596851556	8.082337909	7.935468505	9.129654758
7.314616131	7.393766606	7.971654859	7.548584576	7.195502081	7.02200603
8.458697343	8.645541747	9.80784303	9.130979939	8.932093424	8.169382444
8.29291099	8.387083515	9.180906778	8.821324343	9.335891293	8.095330333
8.410908071	7.863532101	8.404822414	7.783110646	9.033394545	8.036816444
8.437457535	8.245222778	8.906910121	9.150235333	8.549970081	8.87289497
9.909202788	10.31095185	10.08231743	10.04340808	10.04511943	9.669847545
10.56802411	10.88809182	11.21493335	10.92060078	11.07013707	10.5336638
6.231404212	5.994649556	5.778993465	5.912287212	5.767765101	5.808613101
13.78737352	13.72760196	14.31917195	13.95955505	14.15092087	14.11151028
10.8778129	10.82416773	11.37670858	11.01209098	11.2608846	10.61867663
7.960941889	8.656835556	6.813409273	7.584276586	7.534885556	6.534227879
7.936282586	8.181574081	8.683087172	8.171788646	8.306613333	7.447389475
8.477505576	8.452737606	8.432150758	8.575095707	8.251125323	8.595283071
7.309668525	7.551492101	7.581254909	7.80651396	7.411868313	6.503401212
6.745632475	7.637262101	7.680845586	7.631889737	7.297445616	6.789755162
7.963792818	8.759757687	9.058356636	8.668931657	8.476994374	7.670568384
10.13580598	10.13375911	8.908475283	8.906407303	8.861728919	10.49713487
8.142197384	8.271405859	7.20918101	7.134684455	7.079103131	8.232591606
10.2692332	10.18291229	8.874191697	8.882379495	8.836023374	10.5431016
12.58287167	12.0838996	12.55625498	12.60396528	12.63500423	12.9709957
8.702574091	9.177504333	9.140392293	9.048620525	8.740195303	8.794788636
8.917069505	8.856343687	8.899254879	8.878776172	9.493894545	8.864430475
7.539809273	7.18482696	6.731175838	6.91094498	6.855182313	7.127106434
7.975784071	7.333224889	7.785940475	7.451512687	7.794025323	7.488608758
8.175288444	7.631600293	7.118205404	7.223246162	7.005636414	8.135177152
13.83173935	13.5160991	13.57574016	13.55640443	13.43523914	13.46005067
7.741791646	7.100441616	7.451812333	7.438305869	7.649291899	7.437044505
9.541041677	8.91530399	9.579413505	9.621550919	9.402230293	9.62302801
7.056715131	6.090583616	6.240866273	6.246360182	6.218052667	7.07551896
9.033915556	8.511305758	8.210602626	8.326162172	8.229339455	9.008956374
9.066605263	9.195534384	9.790672212	8.153159131	9.216574283	9.385837242
6.242810566	5.855331576	5.959211788	6.491978192	6.220864051	6.614759768
10.28899471	10.4524716	10.15783547	10.21583282	10.09161001	9.636929838
10.0079343	10.39968902	9.691258919	10.14534091	10.09682592	10.34830463
11.43380494	11.56988377	11.46724679	11.5612151	11.4090051	10.92608881
7.954205616	8.834473929	8.920488949	8.565346707	8.53579602	8.121954505
7.452739495	7.194433949	7.372238061	7.570765313	7.715785354	7.599486586
8.044608626	8.475612051	8.72650097	8.504327141	8.545091384	7.569899606
9.415217505	9.372705202	9.70034896	9.560346242	9.312041414	9.774477667
6.447674828	7.274888343	7.143674071	6.774175343	7.141446111	7.245319404
8.734806657	8.929384727	9.679062616	9.083741606	9.119831202	8.111336758
8.596127869	8.77608297	8.448008778	8.339926909	8.185563303	9.061649657
7.380085202	7.179445253	6.869634889	6.831619364	6.717503818	6.867884374
9.533033172	9.042944374	9.134591697	9.533596364	9.381544081	10.10946026
8.764299566	9.083185818	9.562994313	9.647013263	9.532335293	8.338686465
8.960773354	9.07526898	8.672348475	9.324388263	9.056533626	9.04056096
8.335469222	7.842052606	6.874245465	7.041898303	6.813963929	8.568768141

6.117577232	6.142236505	5.909479596	5.917404455	5.84643499	6.076148545
7.60255999	6.753240879	6.538933859	6.608026545	6.517425313	7.514105485
6.370541444	6.33587599	6.169456131	6.363691192	6.141021192	6.184860172
8.028563859	6.977041091	7.108809091	7.206984404	7.227602475	7.965251131
8.099466788	6.498091727	6.542753323	7.023140616	6.869142343	8.528049141
7.095808323	7.350681061	6.198754162	6.710071909	6.679714263	7.308145939
6.938883283	6.77624102	6.254739111	6.743573939	6.329709869	6.536426172
9.067856838	8.287438535	7.709908667	7.920144808	7.742830394	9.206631485
6.106642303	7.273507545	6.030256101	6.409265687	6.340270525	7.138827606
8.200163707	7.725042051	7.493863788	7.586125333	7.465036929	7.795429838
9.650129091	9.561667293	9.413321525	9.441908576	9.091299889	9.712341424
10.68072859	10.63752253	10.60277882	10.68572341	10.89912578	10.55726192
8.936938576	9.110297333	8.841575667	8.887684293	8.889922455	8.984797889
9.133918929	8.419470808	8.26142704	8.360024717	8.509502414	9.484209455
9.726443586	9.756054152	9.186096828	9.329342374	9.751080646	8.610845737
8.621689657	8.448008778	9.106823152	8.019434586	8.871686232	8.697732283
7.114645596	7.317590788	7.293451808	7.252038162	7.159962485	7.076844636
10.81796524	10.39072525	10.42175216	10.96311364	10.8605042	9.813557354
9.214111545	8.829514889	8.859715222	9.485668061	8.342773172	9.235747242
8.657806242	8.529888505	8.752912545	8.899254879	8.85785699	8.992473788
12.32659908	11.96947069	11.8358519	12.02514953	11.85033795	11.43565115
7.109111172	6.923167202	7.18421398	7.249695535	7.111428596	7.428935273
6.735859162	7.398020586	7.959488687	7.06161799	7.260895414	6.967928273
7.231529313	6.118230293	6.339459364	6.128775343	6.429848434	6.67332503
10.2761618	10.0143126	10.0728998	10.08325108	10.31095185	10.44602022
8.905887879	8.81572204	8.414282596	8.688838939	9.038261515	8.592161788
9.056062667	8.865407737	8.864955909	9.038849273	8.886620687	9.428190232
6.619037364	7.148192263	6.876638434	6.842504626	6.852936051	7.083013909
7.423442323	7.738963899	6.943812717	7.210091465	7.072618697	7.492628778
8.846355515	9.414554808	8.515350859	8.83754499	8.775519707	9.921211657
13.42153085	12.59323492	13.0147344	12.83827393	13.03496614	13.56234785
11.50051559	11.60266156	11.36610866	11.54053762	11.77802923	11.34892675
9.89348604	11.26623333	9.728754818	10.4322159	10.56802411	10.43655428
9.759931222	9.269224374	9.723272263	9.701165495	9.529469889	9.136890505
9.862946677	9.971733293	9.982665242	10.12408141	9.905938879	9.562994313
8.652865111	8.993505475	8.752460404	8.606099333	8.898709273	7.814474586
9.526200354	9.404333879	9.466909636	9.361671283	9.445197818	9.671170444
6.983154111	6.629471747	6.787032949	7.443192556	6.947262939	6.990995828
9.644080808	9.823678374	9.696433626	9.647889364	9.537497323	9.838413929
8.78563097	6.779434273	9.319510202	9.257979848	8.568242293	8.147147636
13.48401144	13.87263802	13.60269863	13.11515098	14.00297944	14.39736901
10.31013098	9.890135131	9.206093556	7.31374401	10.37215733	10.54950911
7.176635455	7.115131646	6.912541192	7.182546172	6.843046434	6.704918465
7.067324051	7.22559698	6.668064172	6.824112909	6.521234222	7.027744404
7.111681374	6.756647869	6.671323232	6.919392394	6.716586707	6.92551101
9.479751879	9.979092404	10.47941256	10.02439619	9.823678374	8.598980253
8.613130333	8.448929687	8.408296424	8.295436051	8.41598997	8.198176101
7.067022475	7.495088838	7.063477202	7.442908828	7.073903182	7.191532202
9.376667182	9.162016475	9.240719434	9.562994313	9.284395657	9.054860525
10.64502212	10.79686873	10.59809845	10.63266161	10.52799495	10.33146686
7.457359414	6.485102909	6.367511859	6.587110859	6.423050626	7.667649192
9.394567081	9.17861001	9.218466939	9.382205394	9.316888828	8.987499273
9.886023253	10.50914282	10.81142627	10.4655823	10.65014074	9.910960495
11.39382595	11.52824	10.35069541	10.93646713	10.93171462	11.92396137
9.597513697	9.895061889	10.03701736	9.968215758	9.714566535	9.304577515
8.538919798	9.044608384	7.994228667	8.583542788	7.088821283	8.234636465
9.710054737	9.570470929	9.809529798	9.554043101	8.988033424	9.771430818
7.525550222	8.043818737	8.007258162	8.045324071	7.880363606	8.471953717

7.601304687	7.252886707	7.487351192	6.958444212	7.549206232	8.640789455
9.805520727	9.574750414	9.520598525	9.753529758	9.772250768	10.24139873
13.98831971	13.74093847	12.68484185	13.0147344	12.93430142	13.62784329
9.278597747	9.287277576	9.477759242	9.57181996	9.195534384	8.951513343
8.445759657	8.932093424	8.952561111	9.061091758	9.193130899	8.090157434
10.55474917	10.29000276	10.16073454	10.3380268	10.73014715	10.29324401
9.756054152	9.74383503	9.730279475	9.803356646	9.580783667	10.11941657
6.977291071	7.02612702	7.25519196	7.231529313	7.243824414	7.219017232
7.883536596	8.680072242	8.422643323	8.341133162	8.53537201	8.175629323
8.603276828	8.594016788	8.123132566	8.584830636	8.440058758	8.184399818
15.19830883	15.42966281	14.66395575	15.09418019	15.06622261	14.53168901
9.664626495	9.868690293	8.709941838	8.765765323	9.003925646	9.162016475
9.048620525	9.130979939	9.863774293	9.291393586	9.435722162	8.829061111
7.666604434	8.097566687	8.487847515	8.146343545	8.083135202	7.448962859
8.462637525	8.165787747	8.284876758	8.443956768	8.274284293	8.783584889
7.795765162	8.369167818	8.359650919	8.090886384	8.131433848	7.384605828
8.641223283	7.65457904	8.012698303	8.265965545	8.338355444	8.417196576
7.192117626	9.739033131	7.378508566	7.227602475	9.834210131	10.10278369
8.679578434	8.289599333	8.122747707	8.157901343	8.235056909	8.353342091
8.192996515	7.924172566	8.123471404	8.189895404	8.207099091	8.235056909
10.05880999	9.565032515	9.889177586	9.757590192	9.87177298	10.46108665
6.315590828	6.400341677	6.77698199	6.59799604	6.538933859	6.619744263
9.216574283	9.061649657	8.815152444	9.36570098	9.393335687	9.190767364
8.557697889	8.461795505	8.925792808	8.676316919	8.494982343	8.354947869
7.175798869	7.206984404	7.715785354	7.549206232	7.349181162	7.092334828
9.717365758	10.20164867	10.88681216	10.67939021	10.45877561	9.376046232
11.02700786	11.56291631	11.74670945	12.25844332	11.82112126	11.67849095
14.00297944	13.52959744	13.2504616	13.62106704	13.95167504	14.30038792
9.492465394	10.08145981	9.983540212	9.713858919	10.0406788	9.950180667
12.63888703	12.5521346	12.51424883	12.66167346	12.86086109	12.69316533
7.242080394	7.44801498	7.340746384	7.398977253	7.357173859	6.956131545
10.63266161	11.24816417	11.26815786	11.28207759	11.07956266	11.69658017
11.76804793	11.64637724	11.22119175	11.4535969	11.59870577	12.07810634
6.750367323	6.995800606	6.96619404	6.999830061	6.718256293	6.978602455
8.736738475	8.981975535	8.491049758	8.707057788	8.48093102	7.928832091
6.736575242	7.75479003	6.748587242	6.955200051	6.826151202	7.230210646
8.363862202	8.137860071	8.621272737	8.199315667	8.146343545	8.115023838
7.752360667	8.125818909	8.552208182	8.563085788	8.184050869	6.761077899
6.638128919	7.121605929	6.160622323	6.204026394	6.173134414	6.41609998
8.733447455	8.335469222	8.422204465	8.674900657	8.499836475	8.804952828
9.453123465	9.333361616	9.470840162	9.731806091	9.295516616	9.207219232
7.864861071	7.584965596	7.632188616	7.330546788	7.835124576	8.305359949
8.747331152	9.302735434	9.335272182	8.901935111	9.201355202	8.334587636
7.983184263	7.799854778	7.215458101	7.412806768	7.431765758	7.748548515
7.546592828	7.869992939	8.305359949	8.046433182	8.135177152	6.962306323
7.33182303	6.795634141	6.330919414	6.590707929	6.428350232	7.297796364
6.545300424	6.262968212	6.759969273	6.475960141	6.529766576	6.515423162
6.468467859	6.753469455	6.063490212	6.164796424	6.142236505	6.771840333
8.79576798	9.263851586	9.038849273	8.461795505	8.635209293	8.647073404
11.308834	11.109559	10.92190902	11.52448673	11.2786408	10.55333109
6.169869697	6.662035182	6.127679485	6.229257394	6.221057081	5.94398602
8.90531298	7.270153101	7.393167162	7.481960152	7.250545919	8.943861384
7.656877838	7.589252838	6.615899091	6.910411354	6.844259293	7.578291899
8.35796399	7.713270545	7.839647131	7.977207111	7.920877394	7.843855828
8.155393222	8.361327455	8.118879606	7.690510313	8.183195545	8.784649081
8.534897646	8.525321657	7.831503424	8.061773232	7.942071303	8.231756293
10.73788216	11.30690717	10.89604789	11.00754401	10.4524716	10.82153564
8.823889374	8.69723302	8.975268424	9.092457495	8.760676242	8.808928141

9.171164091	8.822853162	8.388817192	9.079484556	8.856343687	9.671912455
10.77252405	10.80091358	8.593558293	9.853021111	9.437491071	9.783680727
6.651718455	6.867884374	6.796566808	6.789537253	6.899721758	6.830856061
7.339805162	7.558170465	7.866656354	7.55499601	7.584965596	7.961295828
10.20634509	10.042425	8.842639202	9.473962505	9.134591697	10.76737368
8.856343687	8.672881101	9.202552434	9.12042397	9.285000091	8.740195303
7.390471152	7.250899535	7.664957788	7.561196687	7.353900202	7.679923515
8.913024061	8.08193004	7.90331499	8.128290707	7.956615091	9.017695354
10.31997281	10.31597323	10.25320711	10.47702836	10.55213977	10.0053333
7.264798293	6.906092707	7.115131646	6.696696758	7.518430525	7.037826263
7.90505396	8.090488788	8.331458747	8.120062485	7.996770556	7.390752071
11.02243393	11.41415047	11.58814301	11.308834	11.54053762	10.50057506
11.13879804	10.92608881	10.88546605	10.65127749	11.25700815	11.18291631
7.766738848	8.687969051	8.656835556	8.374810828	7.966402293	7.28582396
8.166172404	8.740682	9.150811232	8.485174677	8.597538556	7.99823603
9.320738646	8.667554273	9.306330646	8.952561111	9.159785394	9.35858599
14.16629104	14.23242051	13.3718573	13.81266894	13.91489381	14.56207529
7.450590374	6.517425313	6.485955434	6.661293222	6.566291384	7.431067202
6.623383182	6.21945704	5.960790525	6.112523455	5.94398602	6.55275403
6.611373919	6.584604515	6.927522051	6.575874879	7.059956313	7.809338131
7.384903253	7.114362495	6.533083232	6.632573485	6.496457697	7.695265798
9.290152727	10.02821235	10.44602022	9.497084828	9.791460222	8.263866778
8.026630869	7.892277636	8.148682091	8.269395232	7.893940788	8.101068051
7.209785606	7.013654717	8.037287293	7.529315566	7.204042263	7.854325061
7.021219939	8.563501616	8.474228909	8.290353162	8.021984222	7.101049919
6.962822313	8.513601889	8.595703707	7.274888343	7.640655485	6.622230818
6.513435323	6.20471398	6.926221848	6.833605444	7.650858424	6.023662929
10.02061657	9.417594202	10.00094823	9.528176495	10.02362526	10.76471511
7.869709071	8.457043091	7.692888131	7.915342566	7.906425364	8.375190152
8.374363061	9.384699788	8.246087697	8.314398727	8.174468182	9.505258828
7.697611606	8.825312061	7.636533838	7.778752535	7.805446323	9.09824599
7.959488687	9.215946081	7.909412687	8.626355717	8.26142704	8.291166909
8.361327455	8.375190152	8.101780545	8.419866586	8.397394545	7.909763434
6.275469879	6.570961455	6.019346071	6.277527444	6.084522657	6.114098768
7.10996603	7.60282502	7.873080636	7.648319737	7.504269545	6.552305929
7.67303801	7.550541152	6.734736768	6.839464636	6.880472354	7.230900293
9.29794999	8.903025495	9.235179424	9.236429667	9.380979222	9.467500556
7.26819499	7.201570444	7.717737636	7.614831859	7.520992313	7.821625848
8.443956768	8.036816444	8.066736566	7.942071303	8.044191424	8.516793455
10.34830463	10.76857843	9.800904051	10.19236777	10.11246778	9.615324495
6.420580374	6.724256232	6.249071646	6.401803212	6.307051293	6.162098859
7.839647131	7.890421556	7.84419497	7.906425364	8.023099172	8.257595869
7.479119	7.397737788	6.570729323	6.774434798	6.632573485	7.742113232
7.039678949	7.593349828	7.36076402	7.326042475	7.280525364	7.307562737
9.67665103	9.449948384	9.451851384	9.611261414	9.744663444	9.613873859
8.799348343	9.279246253	8.53537201	8.74840203	8.697732283	8.927019859
7.415013667	7.382527051	6.941216071	6.84402404	7.068134081	7.189144101
6.560645212	6.581970424	6.569345798	6.493067	6.566085596	6.342479778
8.950874253	8.57790302	8.838951081	9.474605101	8.765233303	9.30577901
8.882882525	8.385303111	7.988882495	7.942409707	7.875433545	8.700142556
7.088250616	7.34804498	7.166695253	7.241538444	7.237499505	7.073400869
8.616914869	8.583048444	9.220765354	9.198999222	9.264989899	7.938531869
11.56291631	11.43380494	10.05320582	10.70052069	10.65987528	11.36245739
9.487680697	9.624391323	9.91449603	9.733950273	9.649399939	9.432003333
9.911926424	11.80791686	9.789131121	11.26990549	9.994772242	10.51400776
10.62280049	10.75865445	10.33252085	10.34717129	10.74305691	8.728449242
6.818722313	6.738515899	6.502086778	6.549138121	6.436392303	7.458509535
9.618256808	8.533908051	8.343220798	8.522641273	8.375190152	9.587719636

9.12042397	9.330464051	9.384078242	8.643636162	9.30577901	9.513795939
8.767872848	8.957404525	8.360024717	8.437457535	8.39867397	8.044980354
7.457936747	7.832217929	6.55275403	6.802811828	6.682537212	8.058246657
7.530907465	7.76948301	7.712908172	8.02698897	7.927460424	7.929187444
6.673038182	6.716118697	6.531968667	6.661016606	6.649399919	7.159715333
12.56315114	13.03957854	12.44950322	12.41931404	12.56315114	14.01682491
9.059459505	8.419866586	8.028563859	8.457489293	8.315227333	8.375668162
9.35128199	8.661508737	7.500501152	7.957018646	7.540776061	7.81270001
9.711723394	9.76471501	9.251353071	9.841562192	10.26832521	10.20358283
7.985767212	7.799524616	7.684641212	7.832891081	7.857447394	8.321919424
6.829579545	6.974373515	7.813414727	7.416641556	7.373116051	6.713303939
8.202220545	8.623636919	8.956407626	8.717867636	8.646531515	8.072924455
8.040196323	8.308776677	8.400464071	8.251492909	8.220225172	7.543396525
6.989443677	7.025866354	7.907575465	7.16725497	7.248451727	7.491977768
10.2600862	10.54078574	11.05112773	10.78856661	10.54800525	9.689783586
8.583048444	8.26142704	8.632799758	8.777050384	8.678158051	8.180413081
8.241801545	8.573273283	8.027802949	8.337114525	8.347358778	7.582966818
8.561789232	8.228066545	8.721926778	8.436545051	8.260123323	8.57790302
9.872596081	10.45628541	10.45150222	10.50914282	10.37894268	9.081927515
6.435963586	7.928832091	7.545986354	7.111428596	7.240600646	7.351279444
8.19621002	8.370422404	8.70424704	8.611710212	8.587623212	7.88825004
9.30577901	9.168828475	9.059459505	9.318836889	9.100524758	9.231946182
9.551451798	9.293710889	10.11760527	9.54312803	9.400967465	9.480384485
9.263233333	8.989652131	9.044046343	9.10558002	9.060017677	9.521192909
9.184000242	8.615838	8.986932636	9.290785283	9.030619364	9.468772697
9.858738737	8.01423197	8.462236515	8.363862202	8.279462	10.1300726
8.968391182	8.253254586	8.21550303	8.996667384	8.090157434	8.954176293
7.45523802	7.949400121	8.18481599	8.213427111	8.118473111	6.98587896
11.97447062	12.28105924	12.00086001	12.26162026	11.78498903	12.29886409
9.559682182	9.423636879	9.456392222	10.04682541	9.088505869	9.238865747
9.722667242	10.07388225	9.131560131	9.461673394	9.411394242	9.614560172
10.32418729	10.27432009	9.811898121	10.39747702	10.24139873	10.64623997
8.513135323	8.786134101	8.578393657	8.721926778	8.546545061	8.821324343
7.606043535	7.391655768	7.355380455	7.504556444	7.378842657	7.955993172
7.142295949	7.097545323	7.46750399	7.28082202	7.232737253	7.729067364
10.49592664	10.54950911	10.53028115	10.04600105	10.30358984	10.20964764
10.22865659	9.582110717	10.55842197	10.11760527	10.4429644	9.858738737
6.786546495	6.503631525	6.243346859	6.305339354	6.258382485	6.895973717
9.855298394	9.314493212	9.646274414	9.604846424	9.694925798	9.585510535
8.546545061	8.534897646	8.490531303	8.515864333	8.472925747	8.008022949
9.766131535	10.17789322	9.992117576	10.18468509	10.62536143	9.860587788
12.11841559	12.68903729	12.65819872	12.22341713	12.44950322	11.13085117
7.129030091	7.314025869	6.404865071	6.765291919	6.659100485	6.70156496
6.821514788	7.141446111	6.425110121	6.522313242	6.616392283	7.724049626
8.201745222	8.684654737	8.471953717	8.609106909	8.313601758	7.086157636
7.98506802	7.25490398	6.938620788	6.887288828	6.877410515	7.740855141
6.766746646	6.754943232	7.646666444	7.266525586	7.031727121	7.252038162
6.380219	5.901987303	5.999796737	6.064770778	5.984457657	6.043588101
10.84705809	11.05493438	10.83930015	10.74558579	11.04156144	11.14057221
9.241421747	9.297353606	8.249809909	8.699656879	8.124599212	8.989142232
12.75784116	12.16942168	13.15082863	12.06692054	11.81221198	11.89628941
15.65613201	14.4427031	13.96779242	14.9727616	14.09521817	15.16803204
8.817191758	9.084401768	9.354289121	9.230770667	9.301561	8.307893798
10.40810668	11.15342364	11.6157426	11.05356995	11.02851208	10.33476777
13.46005067	12.72972704	13.66784462	13.7211242	12.9709957	13.84455043
8.779146061	8.113745737	8.683580283	8.825312061	8.45577798	7.77328201
8.220225172	9.021393263	8.173633596	8.397853727	8.325717687	8.962901121
8.385303111	7.626470505	7.508388697	7.357173859	7.329290475	8.269818727

9.581418808	8.986432162	9.384699788	9.540446515	9.221294354	9.290152727
9.002847848	9.188424626	9.460382818	8.33796001	9.460382818	9.74383503
10.07388225	10.51400776	10.90057618	10.54078574	10.3305428	9.954681949
7.771866444	7.741457929	7.235967596	7.079910848	7.337098929	7.621699758
10.1309568	10.71613341	11.0940021	10.64128512	10.60161194	9.316319051
8.537956091	8.387939343	8.469339576	8.412578899	8.510894717	8.813032152
8.27903296	8.271795141	8.108523939	8.464081152	8.35018502	8.294235768
8.227704253	8.016751283	8.123844788	6.994029394	8.199709495	8.395296687
6.742219182	6.736085162	6.097933475	6.178466768	6.217314636	6.674729293
8.128726778	7.470886646	7.022843394	7.262957343	7.12322297	8.868452172
9.915234909	9.902579253	9.713858919	9.787586646	9.723272263	9.541041677
7.296570525	7.168718192	7.462771485	7.386173242	7.288414848	6.300351141
12.92081703	12.53563192	12.16942168	12.6076243	11.60069924	13.05428976
10.87518762	10.66229262	9.121511687	9.106823152	8.974101818	11.25523353
13.44849295	12.71520416	12.27101	12.34237091	12.37416009	12.58287167
8.071703475	8.086153313	7.954533778	8.17219503	8.354182626	7.622357354
10.06997663	10.64623997	11.09250749	10.77103399	10.91176188	9.710962273
9.441234879	9.998242414	10.3548828	10.07580535	10.23351878	9.070117818
8.712341879	9.137887434	8.323598384	8.525321657	8.560860505	9.047491869
8.823394192	9.345614434	8.437898414	9.190205293	9.074692404	8.154321646
14.36999747	14.13521971	13.75520609	13.91489381	13.88591407	14.57091557
9.923012889	10.23752804	9.69725304	9.879566364	9.948421707	9.66383
7.089652374	6.070580202	6.286107737	6.166896333	6.491978192	7.378508566
7.726017717	7.719835253	7.313208869	7.866306808	7.932066141	7.893631939
8.319901444	7.168394253	6.956636515	7.119314485	7.344278889	8.304980606
7.913871889	7.163006	7.040256253	7.032021525	6.996514071	8.217667525
7.622037172	6.812677121	6.886441253	7.203245626	6.747074889	6.856233343
7.088821283	6.407880727	6.535959323	6.410100141	6.634356909	7.34349297
8.191573747	7.506099556	7.623059667	7.253230737	7.59785203	8.442699879
12.82558839	10.43998577	9.839111	12.50328361	11.77305263	10.05607537
7.120752303	6.819416596	6.703235576	6.754460545	6.680656707	7.092673939
6.930222384	6.736375424	6.914278354	6.507801384	6.670331202	6.860671293
7.223533071	6.842504626	6.243838475	6.293110242	6.30015496	7.128516444
7.509050374	6.834065141	7.18283101	7.351279444	7.182269455	7.802503596
10.4429644	10.97476091	11.69891546	11.10460602	10.9889639	10.33696433
9.221852535	9.984347768	10.00886723	10.15585749	9.904237354	8.661508737
8.069395848	8.382194192	8.068931465	8.193428737	8.045722828	8.017104828
8.876650909	9.045755677	8.389653889	8.336257222	8.263041909	8.773988747
8.624974828	7.176635455	7.119927869	6.750902172	6.880775747	8.701614283
7.491688212	7.292561596	6.279552505	6.435479606	6.358115374	7.727390434
10.69470446	10.71390019	10.68338253	10.61480751	10.78014639	11.3733012
10.56655729	10.35591393	10.27804811	10.16937171	10.14999886	10.38531322
9.69725304	9.434425707	9.359207242	9.476588061	9.551451798	9.842388081
11.09721747	11.47871073	11.21816154	11.27306561	11.32185019	11.53473316
12.16036216	12.42233701	12.85048632	12.58957491	12.4571909	12.28105924
7.990677606	8.738283333	9.030004576	8.615838	8.620285051	7.832540091
8.323157747	8.417196576	8.542859455	8.263041909	8.250194202	8.830660505
8.412956141	8.412145273	8.996667384	8.489580414	8.578858313	8.239474646
9.865550636	9.708693071	9.641007131	9.654354424	9.799317404	10.46766096
13.67554111	13.92894576	14.29173122	13.80704919	13.80033164	13.82538175
10.00886723	10.2752886	9.362770111	9.200683333	9.396536798	10.3494333
8.960218374	8.68840998	8.602859283	8.79722998	8.931557869	9.058895657
8.296772869	8.185563303	8.248137606	8.067838768	8.133993636	8.660608859
7.976470212	8.07053003	7.604109192	7.849821828	7.65784001	7.425828556
9.426842081	9.805520727	8.852576737	9.663106646	7.379773919	9.363307667
7.246141232	7.218159515	6.706413566	7.013137848	6.801007778	6.949335212
11.41981305	12.41033676	12.44264271	12.55983323	12.20640951	11.41071085
11.0035084	10.93935201	11.7349036	11.41238356	11.565007	10.34514895

8.033283465	7.724673141	8.276867707	8.521696414	8.250622253	7.798177384
8.694292242	8.917682242	8.186373323	8.359650919	8.292068253	8.400903717
6.390557101	6.993463051	6.326728293	6.452957253	6.438496414	6.287602071
12.06964699	11.68948846	10.79284767	10.53247367	10.84974643	12.08128174
9.41003803	9.800904051	9.749660394	9.575401111	9.801802283	9.701165495
10.61261659	10.2572153	10.33890802	10.41426339	10.22448985	10.00262223
8.628622212	7.997183333	8.089360869	8.911610283	7.953126222	9.07526898
10.88809182	10.25811169	10.40178901	10.63870636	9.666877556	11.04593523
8.244805758	7.466342606	7.838558162	8.130283051	7.725703576	8.507211606
8.785177889	8.794788636	9.06333597	8.03490996	8.277763646	8.371674091
8.695776131	9.026117657	8.665165081	8.00874802	8.876209859	9.400967465
7.928125404	7.542064859	7.561051303	7.633233848	7.611680859	7.409710545
7.556609313	7.676956101	6.875487455	7.133813545	7.056197182	7.485478828
7.533663232	8.007258162	8.288815495	8.29291099	7.85268101	7.519990596
12.41033676	12.82558839	13.12562627	12.83405786	13.22156044	12.61090773
10.60743282	11.11388317	11.60888892	11.01951417	11.50814936	10.34311059
13.35919327	13.23341018	11.84736958	12.48835313	12.17829278	12.78185519
7.97128602	8.322806515	8.353342091	8.403930677	8.308776677	7.400015081
9.208368677	8.943348051	9.01707996	8.966282283	8.941795242	8.963499596
10.51400776	10.20452777	10.73384189	10.53623255	10.57996368	10.84568098
9.692043475	10.49348589	10.94496034	10.47584162	10.13862753	8.891888576
12.43944324	12.05813886	12.62449502	12.62127559	12.38794592	12.80602179
9.875361242	10.4302696	9.738398616	10.55602586	10.09079019	9.630502939
9.710962273	9.769223525	10.66587479	10.14432724	10.15483313	10.11246778
6.652395162	7.033845343	6.672081333	7.208321485	6.847044404	7.026391172
8.800782677	9.368875727	10.01524205	9.520598525	9.575401111	9.03221704
7.785214909	8.431326525	8.291166909	8.182386152	8.556357949	8.35796399
9.678297182	9.558255162	8.577417788	9.103811545	9.179226364	10.02269473
7.648986848	8.290726727	7.16725497	7.562928222	7.789881242	7.632188616
10.47941256	10.05426836	9.794631646	10.31997281	10.47123164	10.27804811
8.369167818	5.99330002	6.297066051	6.19477003	6.260672758	6.142236505
8.409136758	8.304169929	7.392836818	7.666324061	7.627670859	8.531655515
8.436545051	9.390110384	9.378583414	9.04056096	8.684081152	7.999737535
6.929372162	7.078303869	6.134543798	6.222974525	6.21305104	7.235668455
8.968910758	9.170513566	9.54312803	9.514496606	8.905887879	8.880853535
8.496814303	8.880853535	8.869521111	8.78767504	8.687541707	8.190781646
11.94234713	11.9982543	11.64637724	12.05489479	11.28053313	11.73972102
7.954939636	8.633351606	7.686444293	7.98506802	7.951199808	7.777311636
6.294668192	6.768172747	7.37546403	6.884803323	6.956636515	6.236816515
6.998508111	7.074173121	7.153761556	6.818961091	7.077389495	7.12986801
11.11230451	11.09721747	10.19418334	11.36065784	10.08599138	11.0473733
9.408808788	9.640429374	9.576740677	9.760900556	9.681893121	9.333986848
8.991410131	9.056062667	8.936938576	8.78664797	8.991410131	8.980943495
7.679923515	8.185177828	8.372086737	8.225777414	8.137479222	7.462491444
9.105035414	8.814101707	8.63568099	8.858269384	8.751405818	9.095762
7.897352323	8.402148465	6.669224869	7.170490939	7.056486293	6.319492374
6.780426	6.748847646	6.764355354	7.118512586	6.571432061	6.466266586
7.596851556	8.00165403	7.701686677	7.689906808	8.077645899	8.031407364
9.031155364	8.350987111	8.801904485	8.781680606	8.81966502	8.852092202
8.841037687	8.810986707	8.268937657	8.564366949	8.418959303	8.588536172
11.69891546	11.9875501	12.23191382	11.94979793	11.59654933	11.38391359
7.078835444	6.931701566	6.278820495	6.49370698	6.357923899	6.810307121
9.230770667	8.947623838	9.072998758	9.253086273	8.909945455	9.071813909
8.158968566	7.572856394	7.104930788	7.099269525	7.354804152	8.78664797
11.47496104	11.47682743	11.62290433	11.50051559	11.76588718	11.5737214
8.515350859	8.25570701	8.525321657	8.553595747	8.489580414	8.807959646
10.20859114	9.755162434	9.940265465	9.915234909	9.830354566	10.03205964
8.405355091	7.355380455	7.392218323	7.609879768	7.467207283	7.141146879

8.965143333	9.29794999	9.305189859	9.221852535	9.09178804	8.52305899
8.715845505	8.176360121	8.399553939	8.39867397	8.543686687	8.947127939
7.995673758	8.195856758	9.034403333	8.296772869	8.100639	8.455403657
7.709908667	7.515816273	7.670568384	7.287828434	7.70365599	7.860050667
8.303356141	8.870602515	8.476994374	8.794788636	8.60808904	8.070136677
8.07053003	8.316969364	8.392711081	8.335469222	7.885731566	8.256833364
7.399358222	6.319689081	6.540451323	6.538200697	6.504708505	7.243824414
7.819838162	7.83046296	8.341977727	7.994228667	7.824090626	8.538919798
8.289599333	9.071276071	9.397864101	9.11393401	9.011040293	7.616235111
7.549557061	7.954939636	7.924172566	7.677264869	7.615901626	8.467667768
9.884409465	9.990505182	9.770750222	10.12041459	10.35809493	10.40387449
9.660200838	9.644739414	9.321890899	9.362238061	9.388273121	10.43998577
11.5737214	11.15183093	11.66441291	11.70348036	11.5612151	11.94481318
7.501110505	7.584276586	7.628690051	7.771143222	7.65457904	7.326350616
7.621361828	7.523206263	7.710917071	7.723009	7.700334879	6.803094364
8.259704283	8.472925747	7.918472515	8.014589606	8.385691051	8.29927897
7.630594101	7.427372788	6.593389535	6.848551889	6.879943566	7.246141232
11.35858946	10.93023415	10.21583282	10.687913	10.58469265	11.21016953
8.566871343	8.302498081	7.069787606	7.393766606	7.097212141	7.979092162
11.49420288	11.5737214	11.65052704	11.51008986	11.64044206	11.42371373
8.351752182	9.315671101	8.730448515	8.123471404	9.233272283	8.473802646
8.364284556	8.059832677	6.994489162	7.496977949	7.335252788	9.229082869
13.80033164	13.76108056	13.88591407	13.86604428	13.87263802	13.64789782
10.13958591	10.21975253	10.74045328	10.75600216	10.3918566	9.725631121
13.80704919	13.47327752	13.79381649	13.66158872	13.47832004	13.71423947
8.858692343	9.106199707	9.874496818	8.798247798	9.286739242	8.863920758
10.41626133	10.22070473	9.423636879	9.816676162	9.674270303	10.18666124
9.538263677	9.054276	9.673359929	9.682548525	9.558925838	9.694925798
10.95533553	10.83223099	10.85100254	11.00183361	10.91514001	11.25700815
9.272374697	9.744663444	10.14713571	10.06997663	9.964343626	8.550897414
7.230593889	6.916684768	7.560118414	7.555333263	7.464044657	7.087495071
8.150448232	7.948048111	8.176360121	8.217667525	8.215082485	8.648914657
11.11856721	10.86447048	10.72786832	10.60161194	10.98749259	11.27695531
8.76386996	8.638487899	8.639417101	8.695335444	8.826393101	9.132080535
7.31523196	8.439112919	6.869142343	7.151031394	7.104930788	8.427685838
10.35284213	9.992988556	9.725631121	10.080472	10.18291229	10.35591393
9.497084828	9.836777707	10.01168912	9.817379515	9.814415596	9.21067496
10.02362526	9.953782444	9.652939283	10.01340346	10.40592026	10.4283734
8.48093102	7.791244131	7.465356566	7.608347495	7.521572545	8.513135323
8.831201768	8.630379313	8.607626293	8.717364515	8.823394192	9.432636424
10.47702836	10.94786936	10.31378621	10.45996909	9.987077505	10.05880999
5.942984192	6.591813727	5.945424717	6.450362182	5.882632626	6.199491667
7.652274172	7.573484121	7.681488071	7.332669495	7.98506802	7.56320098
10.74819911	9.305189859	8.549970081	8.553132192	8.482406475	10.6768734
7.378186273	7.704008545	8.163475424	7.782178323	7.602282465	6.521008828
11.38178745	10.9991534	10.74305691	11.07956266	11.39382595	11.34202326
8.50618604	8.738735232	9.247274333	8.899757485	8.867411152	8.317411434
9.658049545	9.661659101	7.919459465	8.281167657	8.371229848	9.920430606
7.550195808	7.881102323	7.694540808	7.618887111	8.114611232	6.955646677
8.509936919	7.891531071	7.453623293	7.718083212	7.660499434	8.492747404
8.155023263	8.283707475	8.33273403	8.291610121	8.31912796	7.909031556
7.461509737	7.629989283	8.391020323	7.961295828	7.873080636	7.113452899
12.52461444	12.80949032	12.74093701	12.59668127	12.89513437	13.02518615
11.77552743	10.50677257	11.47127063	11.73972102	11.79615897	10.69682519
9.380979222	9.159196141	8.994589434	9.437491071	8.72650097	9.016491202
8.099112939	7.596246919	7.66151798	7.66151798	7.548285091	8.164575929
6.523626889	6.188392525	6.135409394	6.046596596	5.993422394	6.231242707
7.317897131	7.809001414	7.471589222	6.984574354	7.93027604	7.780116303

6.752974364	5.770679061	5.807826384	5.900210131	5.791842768	5.764191364
10.32849396	9.878686616	9.274850788	9.408173051	9.634173323	10.6313464
10.00985275	9.242021586	9.969141677	9.977292899	9.861430101	9.843921515
8.988577162	8.672348475	8.158321101	8.740195303	8.767872848	9.022478232
10.72516317	10.81280665	11.58195332	11.50632759	11.01531103	9.818059192
6.396985434	6.490215273	6.426872343	6.582672778	6.124834283	5.91259502
12.26475658	12.15440913	11.63837311	11.88375452	11.97660652	12.94367069
6.322079051	5.924468283	6.276797273	5.989387929	6.710717586	7.07468796
7.823083152	7.327495141	7.558324737	7.444499747	8.279889293	8.91530399
6.868107182	6.212710222	6.111573	6.073352293	6.085914667	6.874493707
14.68633385	14.59173737	14.5108222	14.66395575	14.36999747	15.67809237
8.45451298	9.187526591	9.455015404	9.446464909	9.202552434	7.806122253
13.16824615	12.797444	13.40990013	13.38447499	13.55640443	13.27418186
8.142577798	8.518658667	8.939630657	8.743124222	8.39446903	7.690204818
16.3077943	15.99508933	16.33688632	16.52993947	16.33688632	16.17943014
7.297796364	7.262118616	6.933815889	7.381324374	6.754699697	6.655001313
7.825440475	7.856753939	8.403930677	8.064260859	8.248566778	7.199434576
11.17378617	11.24147284	12.01673563	11.93178106	11.21958266	11.671419
12.37087917	12.88708268	12.69316533	12.52130815	12.46466592	11.0387546
6.488640525	6.652395162	6.334611455	6.428567879	6.512378081	6.920666222
6.279884111	6.30015496	5.768195747	5.884050495	6.193202374	6.353983172
6.999830061	6.956363394	7.641344242	6.761583657	7.480656747	7.970574879
10.91356479	11.42787377	11.59021288	11.30131952	11.17378617	10.41626133
8.996148737	9.231349182	8.739178636	9.166446616	9.255548899	9.582761798
9.882779162	10.15086094	10.78269205	10.39641485	10.5431016	9.003357768
8.366003535	8.221794202	7.339805162	7.697940283	7.74646602	8.31318196
12.09804166	11.2786408	11.34576518	11.6137143	11.11714857	11.84291489
12.77841715	12.77015535	13.12023156	13.09977851	12.91196246	12.80949032
8.019434586	8.281588828	8.653906556	8.319524414	8.40702104	7.440763838
8.981479273	8.418553323	8.610845737	8.668062838	8.533908051	8.574218515
11.38775416	10.7982674	10.41530743	10.66832974	10.77637516	12.05489479
7.489812667	7.68740998	8.308331848	7.847720859	7.929593313	6.817799576
8.86024204	8.74840203	9.052474394	8.947623838	9.06557301	8.295839707
7.381027636	7.383711768	8.951989212	7.264510162	7.358963616	7.688949081
6.532429758	6.385183717	7.537489808	6.243346859	6.476638242	6.541858778
8.446184828	8.874191697	9.100524758	8.345353354	8.874191697	7.701352778
10.0178996	9.980879424	9.877792838	9.955574404	10.24942024	10.06997663
13.34730616	13.10484893	12.26162026	12.66627862	12.52819783	13.91489381
11.77305263	11.39983314	11.21187068	11.12613667	11.47322198	12.16279806
9.534883657	9.541041677	9.393335687	9.432636424	9.573320202	9.763138212
8.556855828	9.175472828	9.249612495	9.102676354	8.696739152	9.086722374
8.522641273	8.634342798	8.115023838	8.393536232	8.149788848	8.461315273
8.03179299	7.618249414	7.002156515	7.606699253	7.470329323	8.111719586
9.832711535	9.849671929	10.11555853	10.36687712	9.857940545	10.09682592
12.71830686	12.39660011	12.66167346	12.65104366	12.45390404	12.70787607
8.093473121	7.855388333	7.510907818	7.536878778	7.698300838	8.721926778
10.05970796	9.937540263	9.817379515	9.917043434	10.08145981	10.16642595
9.992988556	9.588958727	9.633493677	9.479751879	9.441908576	9.992117576
9.324388263	8.362537455	8.498503737	8.962353222	8.436995596	9.623794152
14.63205333	14.25895571	14.37933382	14.64328928	14.25042984	14.60195877
6.639490384	6.608026545	6.546440545	6.688467242	6.572131525	6.588475747
7.370079717	7.557551899	8.107705111	7.880750535	7.826504869	7.197446212
5.919581566	6.911201424	7.289658101	5.854788798	6.421175192	7.391342576
9.725631121	9.135618929	8.838020616	9.115691495	9.010430586	8.86638896
6.438298737	6.789286121	7.246141232	6.781179727	6.952808172	6.300565949
7.802180768	7.717096556	7.81270001	7.772177253	7.882525919	8.410908071
7.660150727	7.142620404	6.887288828	6.87893297	6.841391899	7.540776061
7.357456071	6.643746616	6.726480707	6.867411697	6.731175838	7.681167667

6.839237455	6.669677444	7.01811702	6.969919475	6.832363455	7.239071505
8.699656879	9.223302313	9.168324747	9.397864101	8.996148737	8.450646323
7.483852838	7.57799899	7.925642	7.772843323	7.690204818	6.644250091
7.526779747	7.091827414	7.352232505	7.402347162	7.351625758	7.836138323
9.636929838	9.812775374	9.209526556	9.502644929	9.349388778	9.455711606
6.594376838	7.083613121	7.073400869	7.187213162	6.946968899	7.053922394
10.59924244	11.06732533	10.98186414	11.07797425	10.94208941	9.517013525
9.372705202	8.524356212	7.946933485	8.041792596	8.084674061	9.237664879
8.713417869	8.286610323	8.888766747	8.862225737	8.669992818	8.436545051
8.348145404	8.507703202	8.072110051	8.501579646	7.670902747	8.327767232
7.075769859	8.170129495	6.805470263	7.019424707	6.888392616	6.506975192
8.772410586	8.982611727	8.77608297	8.917069505	9.101613848	8.989652131
9.049629657	8.595703707	7.734896879	7.792587404	7.898974697	9.751885293
10.48897537	10.39747702	10.60044575	10.51886057	10.71031814	10.80900786
7.693516556	7.561051303	7.357775939	8.124599212	7.113720848	6.536865101
11.1612024	10.8778129	11.6137143	11.13085117	11.38178745	11.43938222
7.870978364	7.546292778	7.987188889	8.85785699	8.036387556	8.65914896
9.653591273	7.932792414	7.810980737	8.034046273	7.996041859	10.25422487
6.664432283	6.525371737	6.53935602	6.626438091	6.625743253	6.380003717
8.337114525	8.648450303	8.747892899	8.696280707	8.502509434	7.482291222
6.679485202	6.36270599	6.131811687	6.312227091	6.122852596	6.213570111
10.51650912	9.974493182	10.36032821	10.45041513	10.5199714	10.33989796
10.05426836	10.13580598	10.08521343	10.56421743	10.2187154	10.20452777
6.427915242	6.259517909	7.734170172	6.64964403	6.608273162	6.955873859
8.518144394	7.731542758	8.206690121	8.466852202	8.289212586	8.411696677
8.587623212	8.325717687	9.174793152	9.069043081	8.847386828	8.366948404
6.797074616	6.865784889	6.750670859	6.627087081	6.279712293	6.391874798
13.26276274	12.61761735	11.78023213	12.85048632	12.30465105	13.60979651
14.35197184	14.82007331	14.57091557	14.49002723	15.01206542	14.72870348
12.33273925	12.73731843	12.48458958	12.47130519	12.83827393	12.22341713
15.81050193	16.02065153	15.76560628	15.54332231	15.67809237	15.92931651
14.17411554	14.48025691	14.47046648	14.36999747	14.66395575	14.24162707
14.26676228	14.36999747	14.26676228	14.15092087	14.63205333	14.65397593
11.41415047	11.57765358	11.43380494	11.34732253	11.58195332	11.25349741
12.87421343	13.09463792	12.90783816	12.80602179	13.09463792	12.797444
13.09977851	13.35359128	13.13522491	13.06488572	13.33155169	13.5964752
13.15082863	13.74759592	13.70717091	13.35359128	13.79381649	12.74093701
16.22759729	16.3077943	16.42659037	16.42659037	16.7804214	16.22759729
10.9991534	11.47322198	11.12454893	11.47871073	11.36245739	11.44643795
12.94821125	13.12023156	12.84627851	12.97977545	13.12562627	13.22156044
12.30789527	11.72592113	11.29597305	12.02811467	12.41033676	12.93862501
13.18348162	13.31405593	13.13007894	13.26276274	13.36582475	12.64708972
15.76560628	15.72204116	15.52328152	15.86132141	16.14978837	16.02065153
14.85646221	14.80749806	15.03840752	14.84412351	15.10875812	14.6970922
12.0724233	12.41931404	12.17513939	12.13244857	12.3843369	11.74670945
9.06557301	9.424273283	9.179819687	9.193130899	9.424273283	8.862785404
13.87958112	14.10328164	14.27397716	14.14208591	14.11151028	13.90036211
13.47327752	13.80704919	13.67554111	13.73439002	13.92894576	13.33155169
12.15130062	13.16824615	12.94367069	12.89113428	13.16151113	12.54268587
15.24813401	15.15443825	15.15443825	14.99939481	15.15443825	15.02501841
14.56207529	14.42673578	14.74028573	14.42673578	14.79546777	14.50017253
13.93694444	14.29173122	14.13521971	14.07988992	14.33555643	13.20018245
11.34732253	9.325601172	9.195011616	8.942756889	8.912003131	11.565007
13.64083669	13.66784462	13.76702458	13.5160991	13.84455043	14.06476988
16.04217659	15.92931651	16.09492117	15.63778652	16.06827159	16.06827159
12.15440913	12.11562205	12.12151457	12.12692782	12.49965499	12.29548604
13.97489728	13.94443	13.68884286	13.36582475	14.05855866	14.1830221
12.84197228	12.60396528	12.65472241	12.65819872	12.8293171	13.54965001

11.01807842	10.81423156	10.90606985	10.83642757	11.01376888	11.9875501
14.06476988	14.28325373	13.92894576	13.97489728	14.27397716	14.25895571
12.78645268	13.23864174	13.36582475	13.13007894	13.48401144	12.92081703
13.10985871	13.33661988	13.44126502	13.39659876	13.64789782	12.81379045
17.36803328	17.36803328	17.49517975	17.49517975	17.67797782	17.49517975
16.61084374	16.61084374	16.61084374	16.33688632	16.4534382	16.7804214
13.61616406	13.61616406	13.04869312	13.29117716	13.62784329	13.89331098
14.7172184	14.78379092	14.50017253	14.63205333	15.05177986	14.93426708
14.24162707	14.3093669	14.56207529	14.20688311	14.59173737	14.42673578
15.78787594	15.63778652	15.54332231	15.39581235	15.92931651	15.81050193
15.63778652	14.99939481	14.79546777	15.67809237	15.76560628	15.74256723
13.46712151	13.55640443	13.24400831	13.42888134	13.5160991	13.43523914
15.92931651	16.28343863	16.28343863	15.97330865	16.42659037	15.97330865
14.82007331	14.62217632	14.7172184	14.61202705	15.08134613	15.01206542
14.89443037	14.64328928	14.83117585	14.68633385	14.9591327	14.82007331
11.29947244	11.48655657	10.90466263	11.54871614	10.95849458	10.37573078
17.12376032	17.07010739	17.23317693	16.8710039	17.29704085	17.23317693
15.31193978	15.36066179	15.22815255	15.41210544	15.56435861	15.27762024
13.6823979	14.25042984	14.32804886	14.06476988	14.34357305	13.31405593
15.41210544	15.38120792	15.59877501	15.52328152	15.54332231	15.50564301
13.95955505	13.85817003	13.90036211	13.58209631	13.9071606	14.14208591
13.23341018	13.33155169	12.9851923	13.03025597	13.31405593	13.16151113
14.61202705	14.5108222	14.49002723	15.03840752	14.70594079	14.74028573
12.26162026	12.29548604	12.0750029	5.815307061	12.56726296	12.46055504
12.64265677	12.76207554	12.54589185	5.762608364	12.84627851	12.79024082
11.76101506	11.64244603	10.85923156	11.35657003	11.68700199	11.93770961
16.02065153	16.12384164	16.02065153	16.09492117	16.39933956	16.2036826
10.37894268	10.70643744	11.17910752	10.98492733	10.7814083	10.23160947
8.945002717	8.756886111	8.248986687	8.159434576	8.35752198	9.187808616
7.505451222	8.348974465	8.39308204	8.29927897	8.366462404	7.009733586
8.830660505	9.020821455	8.419866586	8.68840998	8.930433505	8.373919657
13.17778072	13.13522491	13.06967433	13.07454735	13.34730616	13.40302459
17.01900663	17.23317693	17.01900663	17.43906183	17.58093558	17.29704085
11.83330316	12.12939166	11.85617239	12.03912361	12.2031413	11.90152869
9.017695354	9.608350808	8.989652131	9.227886646	9.180906778	8.581169111
10.98749259	10.96460614	10.99186298	11.12454893	10.91766747	11.01045685
7.469040515	7.475313929	7.220780818	7.704713596	7.244714616	7.778752535
9.922126404	9.795448596	9.587719636	9.388273121	9.326238727	9.826032111
7.964179182	8.199709495	9.099925818	8.595283071	8.775001677	7.570765313
9.361671283	9.027655758	8.897177899	9.066605263	9.419020677	9.288999586
7.920494253	7.813414727	7.827906879	8.000521869	8.109266465	7.493565131
8.658285303	8.929972798	9.623794152	8.739635162	8.263866778	8.211847566
7.525850576	7.676257364	7.922041747	7.863195747	7.835788293	7.627670859
9.783680727	9.450568111	9.227275152	9.555296242	9.441234879	9.531623293
8.193820172	8.244805758	8.285708747	8.220225172	8.452737606	8.340681505
6.509135818	7.281402384	7.606370131	6.801564111	7.027744404	6.931948737
6.260478586	7.108229697	6.535075677	6.624843121	6.405265273	7.041096091
7.734170172	7.627373273	7.234805667	7.272599475	7.428304182	8.22344601
6.58104199	6.742510061	7.086390323	6.927779576	6.955873859	6.05413799
7.517077263	7.967113929	7.74678301	8.266842545	8.036816444	8.416400293
8.260520646	8.701109788	7.484516838	8.016751283	7.916391202	9.30949301
9.542425758	9.115691495	8.962353222	9.243679384	9.229082869	9.431415586
7.387061818	7.255805293	6.837484717	7.25118401	6.812677121	6.819920253
10.24414187	10.24316974	10.25811169	10.50166798	10.07388225	9.729555121
7.13441502	7.621022111	6.86214797	6.952257202	6.901187545	6.742790232
8.813032152	8.696280707	9.237664879	8.800397414	8.785177889	9.198999222
11.72355631	11.88909817	11.43565115	11.26815786	11.60479972	12.18142186
7.718747404	8.136235949	8.969483	8.44047504	8.587234515	8.099112939

8.676316919	8.808345061	7.882189879	8.486053202	8.720163182	9.026117657
9.188424626	9.035586242	8.785177889	8.836023374	8.745901071	8.869521111
9.990505182	9.75438504	9.797856576	10.26632574	9.928455091	8.929384727
7.931306778	7.40806004	7.316995263	7.435709141	7.398020586	7.910120242
7.403028737	7.98506802	8.214296636	7.937009	8.179951646	7.110844919
10.84568098	11.09089144	10.53842125	10.8405808	11.09250749	11.10170724
10.32531651	10.0053333	9.070661273	9.212349869	9.027655758	10.53505365
9.958247111	9.677492576	10.44195016	10.24227579	10.34100163	9.945994374
11.96059797	11.77053658	12.11216258	12.00086001	12.15440913	11.73710477
10.58347296	10.85923156	11.10460602	11.13713447	10.6768734	9.946747141
12.91656586	14.04335209	12.83405786	13.93694444	12.74093701	12.20960187
14.49002723	14.89443037	14.91973608	15.15443825	14.77222727	14.36070414
15.83165575	16.09492117	16.17943014	16.04217659	15.88528111	15.95256028
16.96059838	17.1822602	17.36803328	17.86179439	17.1822602	17.07010739
14.42673578	15.01206542	14.85646221	14.52085241	14.40789433	13.97489728
6.579612929	5.951137939	6.187521313	5.939443263	5.757964798	5.755766253
13.52334642	13.87958112	14.48025691	14.93426708	14.223913	13.23341018
7.223246162	7.575754657	8.438712657	7.979092162	7.842683778	7.686071172
8.39402401	8.152443121	7.936644384	8.556357949	8.303356141	8.484240687
14.90726461	14.93426708	14.88238784	15.08134613	15.02501841	15.05177986
7.476910626	7.248166182	7.342871717	7.837840162	7.216363677	7.544716788
7.341339747	7.092673939	6.981335535	7.222163485	7.293451808	7.87816304
8.271405859	6.918279333	6.325549091	7.572084505	7.079662576	6.814458515
8.500729384	7.774283859	8.003903253	8.655934636	8.162707626	8.582147707
6.799383374	7.261824374	6.604509566	6.86214797	6.736575242	7.419275485
10.42512206	10.1885747	10.66229262	10.3305428	10.15086094	10.56908161
8.925229707	8.324453253	8.541524636	8.943348051	8.776574232	9.370225242
9.094691273	8.826393101	8.886096818	9.143918798	8.715845505	8.893968333
8.317411434	8.432612263	8.822318	8.214296636	8.161033919	7.689572788
10.41426339	10.06255975	10.0841781	10.17440526	10.05688349	10.30058205
9.09013302	9.737663949	9.962571606	9.749660394	9.787586646	8.64752602
12.99088515	12.63102018	12.80949032	12.82153585	12.50706636	13.07454735
11.93178106	12.08128174	12.28985985	12.10135206	12.19086998	12.13538265
10.82416773	10.68687892	10.80389925	10.49592664	11.17750487	11.01951417
8.221403505	7.775998535	7.991473828	8.048636061	8.22479296	8.272231939
8.132293172	8.383429707	8.778623323	8.531655515	8.492322828	7.31189602
8.084674061	7.177177071	6.927259889	7.310482798	7.331138333	8.519692939
7.941320778	7.720541949	7.049507061	7.29540803	7.166369152	8.373157505
7.387965737	7.678926152	7.848096879	7.844877657	7.575420636	6.98921798
10.50677257	10.52585606	11.20840189	11.29399858	10.88809182	9.755162434
7.949071818	7.732206152	6.950101798	7.160551111	7.135222657	7.199149121
7.038360253	7.253764222	6.58977503	6.805470263	6.654303071	6.48772704
8.012346152	7.833986465	7.431765758	7.552094283	7.582966818	7.820030424
8.576430182	8.761644081	8.313601758	8.528512253	8.531655515	8.162329636
12.65472241	12.91196246	13.74093847	13.52334642	13.49745762	12.90783816
8.798818646	8.556357949	8.985821889	8.77608297	8.76386996	8.834473929
8.315227333	7.710578727	7.007889061	7.18511501	7.073141899	7.957018646
6.597234394	6.399020677	6.852150101	6.252266879	6.779669273	6.169869697
6.28178403	6.401394333	6.226273667	6.236419566	6.471263556	6.415074455
9.714566535	9.767642525	8.789683394	8.637033606	8.615838	9.546645747
10.33696433	10.4655823	11.28053313	10.52689599	10.59180142	11.01531103
8.487847515	8.331458747	8.14677703	8.832291949	8.583542788	8.318250424
7.498029465	6.737335172	6.603158687	6.674983667	6.662501485	7.591105343
7.56574702	7.385857192	7.021742939	7.195783737	7.192117626	7.13133802
9.509226798	10.15783547	11.36793803	10.09339803	10.02741959	9.393934131
10.55966675	10.60507136	10.77890896	10.35699559	10.81796524	10.3779146
8.71185199	8.907429495	8.453185455	8.625920364	8.276867707	8.779146061
10.29851595	10.78408516	9.881176566	10.12799521	10.46232362	9.736957071

10.1985269	9.658772677	10.42297323	10.47123164	10.15379546	10.52799495
8.575095707	8.530767747	8.273878929	8.538919798	8.698751323	8.662363172
11.91912022	11.91416468	11.76804793	11.99323436	12.09497108	11.97196754
9.11393401	9.338458455	9.488337717	9.174793152	9.264483889	9.818860343
6.719301162	6.502086778	6.632781101	6.544328465	6.495011879	7.220481808
8.256499515	8.192996515	7.675893758	7.858964434	7.813759899	8.084674061
9.117478424	9.12042397	9.892629697	8.968391182	9.760900556	10.38208186
7.155513182	7.68296396	7.197080263	7.051121485	7.222702545	7.156444778
10.54800525	10.67428006	10.54800525	10.67815746	10.73911267	10.99046505
9.789131121	9.431415586	9.683334444	9.724047525	9.729555121	10.87907816
7.993077394	7.510027525	6.932737172	6.531724505	6.940479424	8.446641939
8.397394545	8.015705778	8.390114414	8.422204465	8.236217485	8.77608297
9.342353505	9.196689051	8.48093102	8.762202232	8.671783687	9.025691242
8.254082232	8.594861495	8.578858313	8.71185199	8.715328101	8.248566778
6.013395242	6.011014495	6.743803525	6.45473303	6.561505162	5.978784909
10.43655428	10.48310631	10.6820228	10.4302696	10.86173253	10.86566339
11.3733012	11.40306525	11.47682743	11.43005301	11.48655657	11.64848719
9.563605545	9.49850403	9.880317061	9.450568111	9.486974667	9.243096889
7.56165098	7.437630343	7.509707515	7.007889061	7.741791646	7.809644101
11.6157426	10.69793218	10.00186072	10.36465285	10.14131493	11.4090051
12.797444	12.69316533	11.81887725	12.37416009	11.86922315	12.92542832
9.612498475	9.441234879	9.66831603	9.678297182	9.630502939	8.968391182
7.044969556	7.352900667	7.11962903	6.502086778	7.132177788	7.355080212
11.55106122	11.86401838	12.43600068	12.18792926	12.22341713	10.69354222
7.635576919	8.107705111	7.76948301	7.502072596	7.54731098	7.010236465
8.235056909	9.056533626	8.110083838	8.409548323	8.08537204	8.497692788
7.143440162	7.342268626	7.318539616	7.18482696	7.172666465	6.724801141
11.84291489	11.87687449	11.97447062	11.86125412	11.96947069	11.75662781
7.488608758	7.956615091	7.877487515	7.931306778	8.021628354	6.798354081
9.940265465	10.14534091	9.654354424	9.657223606	9.206631485	10.22761479
11.65052704	11.44643795	11.47322198	11.64637724	11.73972102	11.6295292
12.06098883	12.30465105	12.72219022	12.55625498	12.28693608	11.68243664
10.85361094	10.34626898	10.67190595	10.74045328	10.66832974	10.63870636
7.496977949	6.371622788	6.855692535	6.606197394	6.673038182	7.601973414
11.75462574	11.93178106	12.08943903	11.80342824	11.5571076	11.26990549
7.661155364	6.879943566	6.729687141	6.772767788	6.841827051	7.593038414
11.63837311	11.36423723	11.21016953	11.16423752	11.67371856	11.53239215
7.832540091	7.342557798	7.610976808	7.761378475	7.826840444	7.432695485
8.041332778	8.279462	7.541750556	7.776955808	7.919797354	8.000144121
5.792698465	6.019206505	6.622911394	6.048147646	6.137022141	6.570263495
10.3090972	9.631972414	9.331636121	10.06071544	10.10946026	10.30470526
8.017539697	8.576430182	8.633351606	8.542429747	8.538919798	7.55847901
7.191807606	7.128516444	7.126812859	7.169469697	7.108809091	7.373116051
7.859293	7.670568384	8.073652495	7.745466071	8.169730152	7.626110576
6.387713778	7.104930788	6.316499364	6.348225889	6.309281687	6.990730697
8.43006898	7.660499434	6.821773354	7.373717778	7.19003896	8.728878424
13.32669092	13.20018245	12.79024082	12.8652792	12.72219022	12.77454263
10.46766096	10.97322727	11.53050203	11.0940021	11.09721747	10.63391466
11.09250749	10.573928	11.45176908	11.21816154	11.35475345	11.51260306
12.17513939	12.15130062	12.28693608	12.16942168	12.59668127	12.03602449
6.48207598	7.116341071	7.648616949	7.308421545	7.354199768	6.487932242
8.814101707	9.000672535	9.562364657	9.151431899	9.237664879	8.544134919
9.467500556	9.766131535	9.661659101	9.844817354	9.818059192	9.158023
9.560346242	8.955359253	7.269892283	7.761029182	7.610976808	9.703591263
11.21016953	10.53247367	11.34381562	11.18291631	10.80389925	10.41022215
9.683334444	9.882779162	9.930141081	10.05144087	10.4450719	9.20200202
8.179951646	8.60560303	8.626355717	8.653441707	8.728449242	7.533990333
9.254890202	9.797856576	9.858738737	9.849671929	9.933588354	8.685647889

6.709797242	7.08589403	7.376746737	6.947262939	7.053635222	6.133322131
8.171342424	7.760648222	7.862492343	8.319901444	8.005065495	8.441767061
7.862492343	6.822217707	7.489812667	7.301216929	7.526779747	7.768772758
9.794631646	9.50650501	8.964642636	9.600888859	9.650129091	9.474605101
8.155735586	8.529417101	8.980444384	8.738283333	8.451550535	7.74646602
7.308713444	6.474928879	6.525168444	6.522534343	6.461688586	7.152088687
11.06573849	10.93334198	11.17750487	10.82034332	11.0473733	10.97631922
7.545986354	7.569223354	7.487078798	6.937104768	7.792215626	8.018723424
12.42233701	12.76628311	12.57877126	12.49170848	12.54589185	11.50814936
9.827596687	9.520598525	9.402814081	9.524156586	9.196132455	9.459664354
9.441908576	8.906407303	8.653441707	9.192533242	8.52305899	8.69919198
6.092401404	7.069787606	6.436179667	6.385847152	6.209772707	5.865094778
11.81887725	11.41605585	11.27695531	11.63629658	10.91356479	11.71443529
6.934568707	7.12322297	7.540434798	7.348618394	7.497307495	6.638550414
7.789573343	8.690381253	8.712915394	8.249350414	8.475210818	8.609952768
7.07857001	6.067372778	6.187353162	6.384764838	6.336798838	6.995550687
11.01209098	10.71279176	10.15585749	10.35917202	10.21975253	10.68338253
9.844817354	9.642500081	8.021628354	8.492747404	8.644542606	9.911926424
6.125302162	6.255971394	6.003803061	5.949159778	5.988232071	6.028300838
12.10951308	11.34732253	11.99535117	11.10035391	11.82346178	13.70717091
10.31894523	10.39296715	10.92485902	10.53737043	10.44392641	10.16355358
8.298004222	8.150096222	8.528512253	8.434412929	7.59859104	7.167545535
10.49713487	10.43757507	10.03492192	10.59180142	10.50057506	10.49592664
7.111428596	7.390752071	8.199315667	7.342557798	7.954939636	6.441470343
7.122697263	6.601717263	7.233039222	7.068375576	7.173915838	7.278422556
10.40701089	9.751080646	9.148511889	9.651465606	10.26632574	10.4549623
11.911688	12.28369472	11.69188536	11.98440811	11.76804793	12.12151457
11.75215821	11.96636849	11.7894918	11.94481318	12.10414281	11.24646499
8.253254586	8.447526838	9.711723394	8.695776131	8.755916071	7.883917657
9.46304199	10.57159251	10.3986043	10.21054893	10.32531651	9.21905796
7.359287172	7.51810503	8.443547162	7.877844556	8.325316889	7.255805293
7.36510997	7.820934747	8.049354232	7.877487515	7.613528424	7.244122677
9.023639636	7.836508475	8.234232515	9.199527535	8.483326677	8.689808788
9.457741556	9.27419499	8.878776172	9.110297333	8.936938576	9.127872697
8.015705778	7.453623293	6.87893297	7.098723051	7.013654717	8.063898495
10.79284767	11.58814301	11.49839615	11.80104424	11.58814301	10.21769501
10.43998577	10.39641485	10.04682541	10.41530743	10.16743096	10.50677257
7.909412687	7.716120232	8.325717687	7.910617626	7.739314727	8.512201869
8.698751323	8.291166909	8.440058758	8.498949848	8.36556403	8.462637525
7.576687677	7.303836939	8.344441616	7.662252303	7.426803586	7.17606597
9.001802606	8.937429899	9.642500081	9.02886299	9.228437333	8.456593071
8.264694495	8.263866778	8.261007747	8.395713444	8.208692758	7.88753598
7.192412434	7.723009	7.667962596	7.567065616	7.502072596	6.805229596
9.502644929	10.08599138	10.56306707	10.1838089	9.946747141	9.157435455
7.833813545	8.280707687	8.116928465	8.384333949	8.143419303	7.379145515
11.36245739	11.01045685	11.19638858	11.59021288	11.44491584	11.74205097
10.01168912	9.627745303	9.788274313	10.41224321	9.984347768	10.32750324
8.458294828	8.758364879	9.114524374	9.105035414	8.934248131	7.750957677
11.1453264	10.95093974	10.69470446	10.76471511	10.92190902	11.29597305
10.94640834	10.71860391	11.27306561	11.01376888	10.81664077	10.94496034
10.5104744	9.787586646	9.95201103	10.35591393	9.57181996	9.940265465
7.344278889	7.170007293	7.395300939	7.340448859	7.357456071	7.195502081
8.195856758	8.155735586	7.309928404	7.593038414	7.533990333	8.555833323
9.453743545	9.371514253	9.529469889	9.226702434	9.447991384	9.762542495
10.49009312	10.15379546	10.05880999	10.05320582	10.27905981	11.13551505
9.904237354	10.29432836	10.69682519	10.09682592	9.559682182	8.807484626
7.364550071	7.26025397	6.841827051	6.838435556	7.391081455	8.212685152
10.59541023	11.33292491	11.24001011	11.12935449	10.93334198	10.06633871

9.719830172	9.346206162	9.750460616	9.688441414	9.574750414	9.249034202
9.047491869	8.712915394	7.402701172	7.889761727	7.867312374	9.262561
12.94367069	13.25686564	13.52334642	13.30255942	13.44849295	12.89513437
12.0838996	11.63629658	12.03068847	11.92156179	12.01191659	12.03912361
7.280525364	7.252580253	6.975978909	7.153761556	6.951451	6.704451293
8.690918616	7.255477111	7.456765576	7.584620515	7.516138929	8.695335444
9.129654758	9.379180747	9.150235333	9.332795131	9.404880263	9.174120707
6.77206196	6.95944596	6.845837626	6.954196414	6.870918222	6.55006301
7.74961303	7.101319909	7.164674212	7.297796364	7.121885657	7.487078798
9.79615097	9.466304111	9.713188212	9.698181576	9.598893818	9.404880263
7.827504414	7.743136949	7.71023803	7.702311758	8.020887778	8.146343545
10.39414857	9.940265465	8.715328101	8.85049999	8.565346707	8.867918475
6.992927808	6.601976232	6.443240697	6.48207598	6.387934697	6.887875313
8.738283333	8.85308204	8.708995576	9.118629636	9.153730283	8.741130788
7.626110576	8.326518101	7.241229071	7.549557061	7.562627657	8.669992818
10.04862183	10.18098661	8.384837869	8.862785404	8.656835556	9.471411141
8.212685152	7.877844556	7.419275485	7.376746737	7.708254091	8.119679141
8.207099091	8.102124455	7.523756919	8.212243444	7.774939586	8.562664263
7.086157636	6.963095333	6.672296636	6.875911141	7.203112141	6.395369727
6.805984869	6.886152545	6.650783061	6.431354778	6.95944596	7.469344313
8.344860929	8.032957859	8.207099091	7.841440677	7.885378	8.324880909
8.342773172	7.950449798	7.949071818	8.78214798	8.367840636	8.428176394
8.826393101	8.79576798	8.922082808	9.056062667	8.986932636	9.059459505
9.600185283	9.550085313	9.397189192	9.482360616	9.94402302	9.840616414
9.062220818	9.462343535	9.469429253	9.389466253	9.217165182	7.967524909
8.03490996	7.618887111	7.964179182	8.396125929	8.403029636	8.307093606
11.22947614	10.59924244	10.61621963	10.95374716	10.74690378	11.23280633
8.804477505	8.823394192	8.223899788	8.240781051	8.324453253	9.564343333
14.5413574	14.50017253	14.36070414	14.48025691	14.1830221	14.52085241
10.64128512	10.58236152	10.2861211	10.46108665	10.46442497	10.90192126
10.59062542	11.28731295	11.47496104	10.94208941	11.16888788	10.1421691
6.887565899	7.222702545	7.447717303	7.296570525	7.219623727	6.292091273
10.28005063	9.560980909	10.12799521	10.20164867	10.41123643	10.32093584
10.61001127	10.74305691	10.26332497	10.55726192	10.54190997	10.65500785
8.682574626	9.018802576	9.755162434	9.084401768	9.080123545	8.102484232
7.830100949	7.498343283	8.24281297	8.180413081	8.586230848	8.088117667
9.455711606	9.119222505	8.85785699	9.423636879	9.013830768	9.831087364
11.64244603	11.69188536	11.15183093	11.60680648	10.79564384	11.39185194
7.603389051	7.699362081	7.412191343	7.811684818	7.257525758	7.698638758
7.171072313	7.67441401	7.831138545	7.427372788	7.422831253	6.909921869
11.99535117	10.40387449	11.27499716	10.97185531	11.18636156	10.73788216
6.561505162	5.782722222	5.877775859	6.094616111	6.113926525	5.937924475
9.014335657	8.406205495	7.67441401	8.179581869	8.105629576	9.121511687
11.96362869	11.92665931	10.49009312	11.13551505	10.84705809	11.32376318
8.541524636	8.582147707	8.703161646	8.562664263	8.607126586	8.709941838
7.369172101	7.065103374	7.150454879	7.188044747	7.129597273	7.718373848
9.060017677	9.180906778	8.344860929	8.320817869	8.429582192	9.348735909
7.089056525	7.449265303	8.005371424	7.615901626	7.678291495	6.74001704
16.90924948	16.90924948	17.29704085	17.29704085	17.12376032	17.01900663
8.670884566	8.775001677	7.705120374	7.963030121	7.854005263	8.034450929
8.370830747	8.784105778	8.366003535	8.104133364	8.063461202	8.235388636
7.562285172	7.398696909	7.208855192	7.665314131	7.208321485	7.769155828
10.23752804	9.876880455	9.996420616	10.56084718	10.32849396	10.07764109
9.763138212	9.224452333	9.198436465	9.227275152	9.212349869	8.857327323
7.37546403	7.520306162	7.614497747	7.579350515	7.56574702	7.640998838
7.073400869	7.330192545	7.601973414	7.355706667	7.152687384	7.202701576
6.403079273	6.232171323	6.628772899	6.624585818	6.617782121	6.246148091
8.449337667	7.97830402	7.938531869	8.333227545	8.520754616	8.663769939

10.33890802	9.821195081	10.08325108	10.30695028	10.29432836	9.995698596
9.625623061	9.557589465	9.215946081	9.232683343	9.260036485	10.15875628
8.840010495	9.009915859	9.618955576	9.090721626	9.185002364	8.85785699
10.74045328	11.17041501	11.54675633	11.19293398	11.12151875	10.44934262
7.365381232	7.92237903	8.980943495	7.322217273	8.44047504	7.780823677
7.967920667	8.321919424	8.173308798	8.239069586	8.221403505	7.558798354
6.515007141	6.196296707	6.753240879	6.501046121	6.639045768	6.533325687
10.15585749	9.320176919	9.589694828	9.544495798	10.02821235	9.726443586
8.629474212	8.542429747	7.674109172	8.385691051	8.186373323	8.878776172
9.391460545	9.867106131	8.970512081	9.196689051	9.125507586	8.576891556
8.674281929	9.041820404	8.893419636	8.863920758	9.012760899	7.91707401
6.745111616	7.423140192	6.323529697	6.553437202	6.414426394	6.014049576
7.360451687	6.19477003	6.49328996	6.559739475	6.622230818	7.272331859
8.270601414	8.130616515	8.125818909	7.840676768	7.963792818	7.756512818
8.123471404	8.528512253	7.285574899	7.526779747	7.303562909	8.153159131
9.337187111	9.194378495	8.69335002	8.908475283	9.150235333	9.051957646
9.840616414	10.44602022	10.23452156	9.808672222	9.925226333	9.570470929
7.090185162	7.042386798	7.861832121	7.606043535	7.121605929	7.432070859
11.29744903	11.07647857	11.65918316	11.32376318	10.83501466	10.99186298
7.59435096	7.341339747	6.971796172	7.138289616	7.036743576	7.336476646
7.445746576	7.288727879	6.204908667	6.468965343	6.428820091	7.85710597
8.558108202	8.446641939	7.805068949	7.937437293	7.813018717	8.621689657
7.275508121	7.317897131	8.111336758	7.520992313	7.762120596	7.757234778
6.592693727	6.72401001	6.556698131	6.548684566	6.569103444	6.996297444
8.104500051	7.545628697	7.654890798	7.331457121	7.303307	8.616405606
10.15783547	9.650129091	9.453743545	9.548618051	9.66831603	9.407554697
9.766921414	9.36748	9.606298465	9.710054737	9.565032515	10.08799683
6.601226343	6.730193313	7.300653717	6.881514152	6.861656576	6.479217141
8.859715222	8.694840596	9.449948384	9.472607081	8.919352101	8.000521869
11.40495249	11.51260306	11.50814936	11.44643795	11.38391359	10.99623862
7.812003626	7.978709162	8.407442505	8.113745737	8.009790253	7.855731
10.12224713	10.47584162	10.37894268	10.57885616	10.40701089	10.48539779
10.09896607	9.725631121	10.32208546	10.41626133	10.03385507	10.13580598
9.971733293	9.917043434	8.615330515	9.251906182	9.083185818	8.842639202
9.80784303	10.48659409	10.4700758	10.06547375	10.48539779	9.567720788
10.69931975	10.29851595	10.67081044	10.48432644	10.84568098	10.55213977
10.16743096	9.590351455	10.18000397	10.11051676	10.37686258	10.14044783
9.745446081	9.604175636	9.316319051	8.665165081	9.347406172	10.42512206
7.272914455	7.404985061	7.809338131	7.537489808	7.64232702	6.648141899
9.848227263	9.975431768	9.679645121	9.815129384	10.01622438	10.47941256
12.20960187	11.22119175	10.29101087	10.29542336	10.18563382	12.14191765
8.637513768	8.559011545	8.492747404	8.589797808	8.765765323	9.158666485
8.483326677	8.932594273	8.842109	9.153730283	8.921533879	7.723687384
9.126080354	8.59942799	8.718321818	8.959130566	8.842639202	9.292435667
14.4427031	13.76702458	12.64265677	13.05987966	13.07947925	15.03840752
6.880472354	6.786766677	6.520115535	6.28356798	6.243182121	7.574140061
11.24993851	10.6768734	10.3810842	10.77507269	10.61621963	11.05033578
10.32093584	9.895856061	9.911926424	10.22361636	10.5152666	10.17122401
10.27338352	10.09161001	10.04421951	10.1885747	10.24828231	10.39641485
11.67593314	13.00581854	12.68903729	11.6295292	13.63435472	12.70002263
8.934248131	8.722452253	8.792768747	8.896578162	9.427546879	9.001802606
11.13713447	11.3733012	11.60069924	11.54675633	11.6445291	11.05959848
8.746311293	8.895503556	8.565346707	8.971160737	8.594016788	9.146178758
8.266388465	8.172571899	8.283707475	8.306613333	8.230588212	8.809951364
7.341641707	7.348618394	7.883917657	7.442598384	7.501777556	7.940978939
8.710842768	8.759332636	9.266188263	9.055478465	8.69919198	8.62460002
7.467207283	7.665987182	7.602282465	7.545335788	7.941320778	7.374842788
11.53473316	11.36245739	11.41071085	11.27695531	11.17910752	11.08264222

7.892936636	8.391460737	7.841729333	8.08193004	7.944736172	7.235967596
7.948737212	8.41552702	8.754900343	8.403029636	8.419470808	7.578628212
7.569899606	7.969046636	7.155513182	8.050484323	7.236914141	7.690840778
11.36065784	11.59212007	11.39185194	11.63174178	11.51867768	11.08435313
8.104866828	8.178694051	8.478760515	8.518658667	8.187593091	8.080354404
8.755916071	8.505287909	8.603276828	8.653906556	8.895503556	9.189590182
11.32721575	11.02403513	10.55474917	10.8778129	10.67305197	11.70812672
10.33476777	9.542425758	9.971733293	10.25811169	10.21157382	10.01168912
7.261824374	6.317278899	6.129094697	6.408336172	6.308316374	7.592047838
7.876418838	7.749943687	7.415013667	7.361380131	7.381324374	8.294640202
11.98196729	11.77305263	11.48655657	11.6445291	11.9091429	12.26162026
9.66613299	9.724047525	10.20358283	9.974493182	9.831087364	9.50650501
7.268778051	7.133813545	6.526955566	6.632101727	6.670578394	7.436027273
6.546440545	6.341475172	6.370541444	5.93060098	6.725499141	6.090583616
7.859670455	8.115437899	8.799825313	8.534897646	8.833337414	8.402589283
15.50564301	14.94534759	15.78787594	15.88528111	15.72204116	15.26374094
9.281923404	8.990220404	9.147296667	9.445197818	9.126080354	9.415860727
9.034403333	9.212980727	9.094691273	9.096947232	9.085558838	8.520754616
7.36203996	7.645943414	6.75222999	6.913511828	6.45190396	7.788172758
8.750916455	8.861728919	8.548304869	8.759757687	8.573273283	8.160266646
9.198436465	9.331636121	8.621689657	8.797743222	8.78664797	8.055916879
7.546292778	7.806122253	8.07563398	8.012346152	7.923048354	7.249066101
9.114524374	9.372025394	8.663769939	8.726024485	8.817191758	9.147897788
11.2608846	11.28207759	11.45002291	11.62290433	11.37670858	11.18096106
7.159715333	7.28582396	7.019151556	7.175476838	7.117905152	6.758137737
11.07493153	12.14191765	11.52238659	11.1276132	11.57178154	12.3613163
8.39867397	8.235388636	9.080683394	8.897177899	8.472435657	8.626355717
6.815737535	7.166060222	6.467159949	6.713757434	6.732033313	7.262957343
8.233838889	8.156772778	7.136356818	7.923048354	7.664565879	7.630934212
8.426330949	7.337989111	6.900245465	7.219017232	6.886756677	8.530333535
15.32551587	14.9591327	14.11151028	14.4427031	14.20688311	14.9727616
6.863923949	6.645437646	6.303290121	6.458494616	6.319689081	6.623632253
7.164674212	7.17606597	6.947262939	6.958200131	6.973416747	7.002659525
7.621022111	7.416298071	7.266525586	6.902984	8.562664263	7.968277051
11.01951417	11.4090051	11.15808934	10.90057618	10.8947391	11.11230451
8.097222414	8.049019253	8.085771838	7.701352778	8.192996515	8.282073859
7.103272273	7.552449879	7.799524616	7.368846081	7.38862201	7.096340343
7.595301182	7.701686677	8.10326497	7.912365798	7.703321374	7.141717727
7.629989283	7.492628778	7.417598061	7.576363505	7.529949465	7.736260545
9.395854121	9.102676354	8.262181081	8.652374404	8.493155899	9.331038253
8.994589434	9.044046343	9.438094949	8.929972798	9.877792838	8.86024204
7.130781323	7.548285091	7.030426444	6.834332747	7.348317141	7.624735818
15.03840752	13.04381444	14.38889659	14.31917195	13.83772389	14.33555643
8.721926778	8.65497299	9.145078374	9.11154599	8.70424704	8.681579566
6.070726232	6.218052667	6.57397498	5.859926222	6.35000001	6.304940444
11.52645916	11.75908761	11.55106122	11.64044206	11.71895032	10.2166926
8.31912796	8.055171202	7.797844434	7.842683778	7.791244131	8.50473503
6.974671657	5.998196768	6.306277939	6.331704263	6.221214869	6.655749121
9.673359929	9.409395929	8.647073404	9.043522909	8.73262002	9.529469889
7.182269455	6.946711626	7.180020091	7.063754707	7.228169333	7.178323677
8.377090384	8.000883182	7.960547	8.39308204	8.681579566	8.198599495
10.50914282	10.73788216	9.932690333	10.42736719	10.35917202	9.739033131
7.688370495	7.151822525	7.123768636	7.400864778	7.492628778	8.351752182
8.255317818	8.280295667	8.309247939	8.191573747	8.273878929	7.600615697
7.824090626	7.342871717	7.216692263	7.668638899	7.387061818	7.595597162
13.58996905	13.56886427	13.56234785	13.58996905	13.37822002	13.70143341
7.062959768	7.103815788	6.534648919	6.650108172	6.595947525	7.282635596
6.873334899	7.226157253	6.649399919	6.708053838	6.724801141	6.536641737

6.989715687	6.918844495	7.075225141	6.830079818	6.918565768	6.726701909
8.466446051	7.500795283	7.328721848	7.325162263	7.164102909	7.848096879
6.396609343	6.512378081	7.194677455	6.965182525	6.895441525	6.323529697
11.07647857	10.46664928	11.12315281	11.26446827	11.11388317	11.32721575
10.11941657	9.999132495	9.722667242	9.969958172	9.875361242	9.939327313
8.346198293	8.352537101	8.924680667	8.34982299	8.609511	8.509065354
8.856895172	8.316570081	8.809951364	8.789683394	8.961327374	9.352487919
11.83813795	11.14385742	10.90324033	11.83330316	10.70181173	11.66189432
7.303307	6.419503354	6.278089182	6.443888202	6.47408797	7.326634626
9.695719162	9.872596081	8.677215515	9.041820404	9.182731869	10.65014074
8.73262002	8.854603364	9.336479586	9.193817232	9.238865747	8.309668384
10.70902317	10.61261659	9.929312889	10.49009312	9.312727606	11.47127063
10.55842197	10.56802411	10.79956984	10.71145754	10.85923156	10.71031814
8.377562293	8.63611798	8.790130253	8.855784909	8.549141545	7.82717702
7.077389495	7.573788949	6.740749081	6.982384606	6.766293242	7.01152302
9.718307192	9.518503263	9.051957646	9.300367869	9.35067002	10.17440526
7.340147596	7.835124576	8.050484323	7.973992111	7.84419497	7.101319909
10.76737368	11.36979611	11.542568	11.19638858	11.60680648	10.50914282
7.727011939	7.783847071	7.680240364	7.838908253	7.701991131	7.693213707
6.485740606	6.212018222	6.558365444	6.530842141	6.574452879	6.74585002
8.021628354	7.959488687	7.844877657	8.301264212	7.787223354	7.291703707
7.890057202	8.182386152	8.466446051	8.60560303	8.517695303	7.765298909
6.476638242	6.484023485	6.461008283	6.426421657	6.34228097	7.084144263
7.050890818	7.448310273	7.842359758	7.347416111	7.722652869	6.551212667
7.608934747	7.737649848	7.635907081	8.593558293	8.023866626	7.854005263
9.226128374	9.580097263	9.041236566	9.394567081	9.292435667	8.757405333
8.954176293	9.250770152	9.361074667	9.279246253	9.399129424	8.660166475
7.319114313	7.326634626	7.122697263	7.180839586	7.294583061	7.503972212
8.315715768	8.344860929	8.300103657	8.285312424	8.209801687	7.618249414
6.788076343	7.242951465	7.775998535	7.605113071	7.452458778	6.39883498
9.588410576	10.22267877	9.939327313	9.857940545	9.937540263	9.234418071
9.550085313	9.996420616	10.75987116	9.976273051	9.925979414	10.37467131
8.240781051	8.147481596	8.102862354	7.853350485	7.915342566	8.844262616
8.13932801	8.230588212	8.543686687	8.509936919	8.203586121	8.289996192
13.89331098	11.52448673	13.37822002	13.31405593	11.84027237	12.10951308
7.549206232	7.507667889	6.227696606	6.519665929	6.364484303	7.858964434
7.894315778	7.971654859	7.992662242	8.181196919	7.545335788	8.325717687
12.52130815	12.53911948	12.8293171	12.48124823	12.41931404	12.21630458
8.225261616	8.546545061	8.140977576	8.169382444	8.288815495	8.233006697
6.548684566	6.987599808	6.526325283	6.725499141	6.629250202	6.823384798
8.152443121	8.933180404	9.185002364	8.906910121	8.929972798	7.872350636
6.665428859	6.360244697	6.992927808	6.255807596	6.236087091	6.097185768
13.40990013	13.16151113	13.50411017	13.34730616	13.1944969	13.14093405
9.802496737	9.291393586	9.31327601	9.30949301	9.961780586	9.673359929
9.042944374	8.944409727	8.503061444	8.870602515	8.60560303	9.269847242
12.62127559	12.39348455	10.96162926	11.91416468	11.74670945	13.14611959
10.43122177	9.182731869	9.107388182	9.07526898	9.211846273	10.18291229
8.05474504	8.809951364	8.597538556	8.762742172	8.804477505	6.941957697
10.3494333	11.07493153	11.30301738	10.98186414	11.00754401	9.885156737
8.350987111	8.117339677	8.297630758	8.373157505	8.178268222	8.76328402
7.646666444	7.716479172	8.047553323	7.912742707	7.541090081	7.936282586
9.239472869	8.953653768	8.628622212	9.146178758	8.893419636	9.406122899
8.750392838	8.913588253	8.662816202	8.691979949	8.690918616	8.276867707
8.946596606	8.716387192	8.827377505	8.790654051	8.496814303	8.89604703
6.64233597	6.038947424	6.017254566	7.138578667	5.983964121	6.756409455
7.508747081	7.658441616	7.608934747	7.617533182	7.446760616	7.862492343
7.609879768	7.320933798	6.763620737	6.860943283	6.930743303	8.230996848
9.488971182	9.303342798	9.167758485	9.415217505	9.280885222	8.805485081

13.03957854	12.69676846	13.22730043	13.01016522	13.25686564	13.15645152
8.953653768	9.919491525	10.31480319	9.958247111	9.895856061	9.326739394
11.19638858	12.20960187	12.40343679	12.09804166	11.91651973	11.41981305
10.41957053	11.43773109	11.64848719	11.32721575	11.18471248	10.56421743
7.923778798	8.039068889	7.465665798	7.768055657	7.173022687	7.596851556
6.57823699	6.944866586	7.066776273	6.727165222	6.92551101	6.182782414
8.327767232	7.862160495	7.979449616	8.099112939	8.016751283	8.658285303
9.30949301	8.712341879	8.883414061	9.11510898	8.946596606	9.227275152
8.821885465	9.46304199	9.35128199	9.131560131	9.249612495	8.366462404
7.885002121	7.849463182	7.652952818	7.766357737	7.814093232	7.822322859
8.797743222	8.649944576	8.731576192	8.903602263	8.806450121	8.889922455
8.848410616	8.597027768	8.896578162	8.541125101	8.845785121	9.324388263
11.39185194	11.42562271	11.15342364	11.02551486	11.66189432	11.911688
8.469339576	8.127495596	8.324453253	8.360501364	8.337550606	8.519178475
9.528176495	9.296718121	8.599954677	9.042346232	8.860747333	9.487680697
7.034331232	6.886441253	6.415875869	6.447271273	6.376515232	6.989960667
9.562364657	10.32531651	10.44094498	9.926784556	9.690534253	9.298556212
10.01622438	10.6820228	10.29752835	10.53505365	10.50914282	10.29101087
9.416442616	9.739859192	9.482964828	9.49850403	9.453743545	8.711304737
9.34289298	10.1838089	9.690534253	9.840616414	9.798623747	7.444173596
5.797932899	5.772434121	6.143049838	6.692767899	6.20351898	6.830578646
7.589888475	7.888681737	7.681826051	7.74007802	7.57049196	7.292849111
10.38631174	10.78705857	10.45375986	10.8605042	10.69574272	9.773700283
9.412044242	9.100524758	9.559682182	9.712341424	9.510206136	9.124970051
6.991849242	7.187741232	6.444612172	6.71730499	6.658617323	7.160791343
8.459227657	8.098302202	6.940218677	7.237838192	7.302435273	8.246913576
7.443192556	7.93027604	7.083894141	7.280525364	7.174439091	7.137450808
8.881310737	9.415860727	9.867106131	9.116804101	9.618955576	8.96575796
6.739283293	7.606043535	6.246725566	6.137022141	6.22592999	7.68569196
8.69723302	7.597232061	7.431067202	7.575420636	7.634916323	8.771856424
5.843117535	6.521900899	6.479217141	6.496794495	5.841267899	5.783939091
9.775265525	10.25320711	10.16165443	10.27432009	10.04421951	9.214694788
9.557589465	9.435122859	9.360502949	9.49510903	9.535633242	10.04682541
7.220240556	7.650531152	6.867411697	7.221345788	7.158855657	6.866319192
6.634850818	7.14692997	7.261194687	7.091328697	6.864750879	7.345267404
9.804037697	9.501970121	9.93099102	9.836777707	9.897493586	10.05144087
7.813414727	7.623059667	7.613528424	7.741457929	7.797125465	8.046433182
10.53505365	10.34311059	10.62647628	10.56655729	10.67190595	10.57282784
12.10414281	11.76804793	12.15130062	12.17216641	12.01427079	12.24649354
9.262561	8.86024204	9.287819051	9.291911616	9.32739897	9.742174424
10.18468509	10.48074239	10.7814083	10.80389925	10.97322727	9.835894606
9.137373131	8.901331343	9.179226364	9.133918929	8.995099212	9.542425758
10.01071956	9.94402302	9.002847848	9.549314313	9.329922273	9.35728196
12.41634374	12.56726296	13.10985871	12.68484185	12.66167346	12.39973853
7.550541152	7.13936198	7.301216929	7.500501152	7.616552222	7.593664717
8.868452172	7.849821828	7.792995667	8.844732879	7.950819283	7.672313364
7.652632424	8.251125323	7.382214869	7.631600293	7.53302901	8.480536717
7.380686859	7.502411182	6.832363455	7.024222101	6.971796172	7.480656747
8.746767313	9.014335657	8.983202444	8.909404788	8.877161475	8.04259702
9.621550919	9.886023253	8.995099212	9.399129424	9.160413333	10.28795108
11.78725933	12.02240324	11.59654933	11.96947069	11.70812672	11.28207759
14.66395575	14.84412351	14.89443037	15.10875812	14.91973608	14.79546777
10.03026493	9.909202788	9.917043434	10.27035429	9.868690293	9.86223396
8.094218354	7.850890354	7.754407434	7.972012263	7.829693939	7.489176
7.648616949	8.377090384	8.307483899	8.002717273	8.081502051	7.320252869
6.950648455	6.039785808	6.089542354	6.241411051	6.17582696	6.834834778
11.29597305	10.95234717	11.06732533	11.63837311	11.06261544	11.43208235
7.478150263	7.659822475	7.599211495	7.157499455	7.892936636	7.775269606

7.609232899	7.268778051	7.572084505	7.299385535	7.533663232	7.555972677
8.769824364	9.772250768	9.326238727	9.488971182	9.277992232	9.261851101
9.772980061	9.910074283	10.09339803	9.625041525	9.738398616	9.965319232
8.019097616	7.755503707	7.56320098	7.596851556	7.583998525	8.526186556
9.212349869	8.612685364	8.034450929	8.41335802	8.302498081	8.968910758
8.96575796	9.228437333	8.832291949	9.171801828	8.752460404	8.625920364
8.050868949	8.625920364	9.352487919	9.047491869	8.649944576	7.568310444
12.06397065	11.39185194	11.26623333	12.16279806	11.15503783	11.96362869
9.211846273	9.255548899	8.35796399	8.338686465	8.685647889	9.547339828
8.451947071	8.830117929	8.64507902	8.613985131	8.537956091	8.144908747
7.558170465	7.554702939	7.69185196	7.650231051	7.994566778	7.420173404
8.219387636	8.335861768	7.860366808	8.166602566	8.041332778	7.951199808
8.568242293	9.312041414	8.650965909	8.597538556	8.643157737	8.612685364
8.553595747	8.266388465	8.430534091	8.378142071	8.532980343	8.955880859
8.787164939	8.848914293	8.372772768	8.925792808	9.052474394	9.369545939
8.078774939	7.973586899	8.086153313	8.177528808	8.197004869	8.759757687
7.258693172	7.225001677	7.274379929	7.153495505	7.181436869	7.047335818
5.846995475	5.785134253	5.776079596	5.851984606	5.765373737	5.768471152
7.088518818	6.999830061	7.080799364	7.303562909	7.239990273	7.040523253
9.350012182	9.426142444	9.323731354	9.535633242	9.579413505	9.223302313
10.87907816	10.65871503	10.09597734	10.74690378	10.60635433	11.2786408
7.282065253	8.572163455	8.018337859	8.30620801	7.911309091	7.121885657
10.10278369	10.18942995	10.34626898	9.655770101	10.6426412	10.38738452
13.2158469	13.52334642	13.58209631	13.49745762	13.50411017	12.50706636
11.22294817	10.3025572	11.21338774	11.38577951	12.36759935	12.33576886
7.52646604	7.488894061	8.141770949	7.680845586	7.791533949	7.825104404
8.441767061	8.136641818	7.639662323	7.759260374	7.807154444	8.430534091
7.041096091	6.581253303	6.767959131	6.720541949	6.99155401	7.06536003
7.177746566	7.025264747	7.10996603	6.988144556	7.304125475	8.236986626
6.884803323	7.06161799	6.486601465	6.602666131	6.51696904	6.848551889
7.635267465	7.095534354	7.238133434	7.650858424	7.246141232	7.533663232
10.19136651	9.892629697	10.24316974	10.18098661	10.14044783	10.56207051
6.948873394	7.680240364	7.066192545	6.858444758	6.741460808	7.551170919
9.264989899	8.457489293	7.948048111	8.188779434	8.128726778	8.917682242
6.981086404	6.854415313	6.17582696	6.53354203	6.366615303	6.777231859
8.473392556	8.988033424	8.720163182	8.802385697	8.970512081	7.604463141
9.165786899	9.370832535	8.756396081	9.16271003	8.569735879	9.273022949
6.608949242	6.590707929	6.140075394	6.377507434	6.325917293	6.352616364
6.88247998	6.693987121	6.609208404	6.666656929	6.632101727	6.576798515
15.39581235	15.48449671	14.80749806	15.56435861	15.50564301	16.39933956
8.70424704	8.345781111	9.156840283	8.804952828	8.611710212	8.653674131
6.589168273	6.42385696	6.697417434	6.705661929	6.502532889	6.717050677
8.127495596	8.121954505	8.021984222	8.074508444	7.870356606	8.081502051
10.85654613	11.33100665	10.97476091	11.24646499	10.92485902	10.1250404
8.182386152	7.724049626	7.012316737	7.405524869	7.40526796	8.218433131
7.669946162	8.388388636	7.793372141	7.451183323	7.63758797	7.668638899
7.993428586	8.311754818	8.694840596	8.322342051	8.18481599	7.799524616
8.569735879	8.811517808	9.106199707	8.813595202	8.764299566	7.898974697
7.961295828	7.941721242	7.222163485	7.717737636	7.034331232	8.207537162
6.92644801	6.874245465	6.641155141	6.792041707	6.683531697	6.848028121
10.7814083	10.41846051	10.2829717	9.290152727	9.837575172	9.716648152
7.676956101	7.163863636	6.383422788	6.171005242	6.271013081	7.325162263
10.87379507	11.04593523	10.14534091	10.69222123	10.28506122	10.0406788
8.988033424	8.565346707	9.038261515	8.786134101	9.108564253	9.526200354
7.627670859	8.19621002	8.236633323	8.076845152	8.193428737	7.364257101
12.4571909	12.23505088	10.41626133	11.39983314	11.06433979	11.81887725
8.684654737	8.607626293	8.466026899	8.480536717	8.722452253	8.904793061
10.8605042	9.185002364	8.98371896	9.205404404	9.094076404	10.81540317

9.442729677	8.768300525	8.721523222	9.326238727	9.142791657	8.864955909
8.129067798	8.168969465	8.870056939	8.354570556	8.777572838	8.093077828
7.88791003	8.165380303	7.788923101	8.094989869	7.85299496	8.385691051
8.245222778	8.630877737	8.810446485	8.50473503	8.914132333	7.952029848
11.06433979	10.9889639	10.95849458	10.68452674	10.93494087	11.07797425
8.256833364	8.167813576	8.083806798	7.984708455	8.05903404	8.693780273
8.929972798	9.000069889	9.043522909	9.328020364	9.62302801	8.305787747
10.56421743	10.58117749	10.87637444	10.4994288	10.75062885	10.73384189
9.706432808	9.116279576	9.650129091	9.730279475	9.926784556	9.26122399
8.692466525	7.302140758	7.239990273	7.777311636	7.560760293	9.104371838
9.465659495	9.070117818	8.996148737	8.674281929	9.109120374	10.1597097
13.37822002	13.48401144	13.70143341	13.48990673	13.09977851	13.30795266
9.303342798	8.093860848	8.816703535	8.490053091	9.287277576	8.71185199
8.630379313	8.994589434	9.335891293	9.35128199	9.23383296	8.115821848
7.454260455	7.870356606	7.946141838	7.630934212	7.564741838	7.246997929
11.65698449	11.40495249	11.73271713	11.34576518	11.24646499	11.54053762
12.39348455	12.31089415	12.84197228	12.75396286	11.95261936	11.74935785
8.870056939	8.39867397	8.62460002	8.978204141	8.427236737	9.222631263
7.349485707	7.263531485	7.425828556	7.334908263	7.354460354	7.143674071
8.545091384	8.558556152	8.481851586	8.552719747	8.328154263	8.448008778
9.102676354	8.881310737	8.856343687	8.99408802	8.469339576	9.253086273
7.46750399	7.446760616	7.082724758	7.339805162	7.254644071	7.222444626
10.34514895	9.835894606	10.09079019	10.22448985	10.16073454	10.12697515
8.683087172	8.992957212	8.038354192	8.428176394	8.232591606	9.394567081
7.732206152	7.275765808	6.733937384	7.12541897	6.982135444	8.05903404
9.967178364	9.410728505	10.18468509	9.888393747	9.671170444	9.844817354
6.781710556	6.680176879	6.428567879	6.80380202	6.642136364	7.950449798
9.573320202	9.865550636	9.948421707	9.992117576	9.910960495	8.670884566
8.942756889	8.691979949	7.843855828	7.916712838	7.946141838	9.293068828
9.562994313	8.920488949	8.463578343	8.818210192	8.679578434	9.550085313
7.804724687	7.603389051	7.744142424	7.944736172	7.567690545	7.97284099
9.634846707	9.35067002	9.229670424	9.408808788	9.467500556	9.823678374
8.001277303	7.755132808	7.98690202	8.133993636	8.105207222	7.670275758
9.379720636	9.45706402	9.462343535	9.464440242	8.904793061	9.591133828
6.540929384	7.032313212	7.501110505	6.724536455	7.309336424	6.266853091
7.314300525	6.539575101	6.83774396	6.82385998	6.751435283	7.288727879
9.985290333	10.14044783	9.348136535	9.833438364	9.782868192	9.986114121
8.295009141	8.328992859	7.502729737	7.823083152	7.539487212	8.271029889
8.985821889	9.408808788	9.439397061	9.541041677	9.224923687	8.656360697
6.382217859	6.26253897	6.455163758	6.46756804	6.430701515	6.732525747
8.955359253	8.626833051	8.187593091	7.971654859	9.269847242	9.625041525
10.08893073	9.828404283	10.58579258	10.01257663	10.48185706	10.13375911
7.260583141	6.552098808	6.452349424	6.460516394	6.343092465	7.516462556
8.275152535	7.944406939	8.22479296	8.117339677	8.326518101	8.251125323
9.406122899	9.273022949	9.585510535	9.392082222	9.395177889	9.413321525
8.758871	8.673363869	9.27419499	9.30577901	9.392652323	8.724556485
10.05523038	8.531247657	8.083135202	8.057864374	8.014589606	10.19649023
11.34381562	12.70002263	11.74439188	11.33477589	11.79405389	12.57486869
10.16454544	9.829822242	10.04600105	9.801802283	10.20164867	10.82945836
9.930141081	9.343457394	10.19649023	9.582110717	10.02549055	9.960013465
7.968277051	7.386471808	7.04545701	7.266813283	7.262118616	7.792215626
11.58421758	10.85361094	11.16423752	11.2518391	11.53473316	11.19114259
10.49140257	10.93171462	10.48782122	10.94942311	10.92880859	10.87092264
8.433468657	8.199315667	8.526186556	8.323157747	8.435650677	8.713915657
9.08614898	9.153730283	9.483552818	9.388912091	9.35371997	8.739635162
6.495899485	6.111914111	6.105015131	6.176869667	6.099738869	6.349593626
6.207516667	6.235363323	6.050896313	6.176177303	6.182241051	6.133823313
14.36070414	14.5513462	13.83173935	14.05855866	13.93694444	13.87958112

6.855940818	7.136875657	8.669446475	8.173308798	6.864476545	8.626833051
6.068877949	6.158655535	8.320357909	8.289212586	6.214653717	8.009440313
8.174871818	8.057864374	7.891531071	8.12156602	8.011643808	7.706911222
8.792195697	8.145617202	8.630379313	8.975838535	8.651919778	8.200539131
10.27035429	10.07479617	9.565032515	9.746145949	9.648698737	10.8947391
12.28693608	11.49420288	11.79870316	12.11841559	11.97447062	12.2293359
8.149788848	8.114611232	8.142577798	8.133538364	8.289599333	8.155735586
8.013857071	7.81061403	7.891143768	7.963792818	7.734550869	7.662925475
12.51424883	12.84627851	12.0838996	12.33273925	12.2293359	11.77053658
6.902482606	6.843046434	6.673543687	6.920203848	6.859929394	7.28082202
10.51886057	10.36687712	10.04963797	10.22668357	10.10454015	10.28506122
11.43773109	11.00902763	10.9889639	10.55213977	11.6137143	12.47441306
6.766293242	7.472789818	6.702734242	6.719301162	6.795634141	7.09469803
6.328925929	6.27919301	6.090019909	6.162603707	6.056728475	6.646430939
8.957404525	8.791137414	8.865407737	8.761177727	8.863920758	8.871209182
9.589694828	9.534194535	9.245445293	8.916479111	9.069043081	8.888766747
8.549970081	9.030619364	8.885078273	9.004940778	8.924201657	7.886079293
10.22951336	9.804037697	9.497084828	9.242021586	9.833438364	11.39772243
10.22070473	10.06071544	10.27236099	10.20532801	10.4549623	10.78987961
8.40783399	8.690918616	7.559118222	6.315590828	7.672313364	8.078774939
10.83363242	10.3810842	10.65365596	10.8254891	10.70902317	10.54692936
11.12001247	10.76471511	10.71031814	10.89094316	11.1453264	11.18800276
7.366006364	8.205892515	7.620272192	6.946711626	7.82475102	6.983407384
7.141717727	6.364484303	6.212887111	6.310688818	6.224978131	7.132751192
9.368224626	9.337187111	9.197870838	9.445852485	9.294355394	9.254890202
8.602379626	9.179226364	9.18155302	9.344897333	9.412634061	8.082778869
8.530333535	8.309668384	7.771143222	8.017539697	7.775269606	8.474658303
10.34418014	9.412634061	9.729555121	9.938475404	10.0188097	10.72127155
8.964642636	8.691458141	8.749910828	9.74383503	8.650965909	9.256767414
10.88681216	10.5041569	11.01531103	10.70425349	10.70052069	11.06123261
9.721958051	10.29660096	10.28005063	10.00695165	9.716648152	9.114524374
9.484840202	9.625623061	8.816214848	9.184527788	9.488337717	9.390110384
8.320357909	8.359263212	8.03364202	8.276438626	8.068200283	7.983913121
7.059676455	7.938531869	6.801007778	6.921891778	6.802811828	7.088518818
9.881176566	9.786685869	9.818860343	10.01168912	9.916104475	10.36465285
9.282486081	9.491725303	7.568924192	8.444861909	8.258860253	9.76546198
7.036743576	7.431765758	6.541858778	6.660321343	6.773917293	7.302435273
8.391869283	8.005727263	7.584276586	7.506434626	8.07651197	8.29635502
11.89628941	12.09225922	12.12692782	11.92396137	12.18142186	12.04734694
16.36806566	16.36806566	16.49551516	16.02065153	16.49551516	16.28343863
7.214922364	5.778423091	7.200432616	7.197080263	6.629982465	6.352813182
9.825199323	9.308918081	9.275532697	9.436357141	9.549314313	10.24316974
14.11997744	13.92172837	14.64328928	14.35197184	14.42673578	13.85817003
8.451550535	8.000144121	8.322806515	7.862160495	8.329784131	8.894491141
13.18858184	13.14093405	13.30795266	13.47832004	13.27940818	13.57574016
6.566952202	7.057330242	6.860179071	6.722483515	6.930500768	7.016713091
9.756840071	9.469429253	9.908305848	9.741357455	9.658772677	9.46304199
8.328992859	8.76478299	9.791460222	9.164000111	8.917069505	8.119288848
13.00581854	12.64708972	12.88708268	12.70390423	12.80602179	13.48990673
6.365542475	7.749299101	7.032021525	6.666381515	6.411488202	5.944354475
11.46145043	11.9577766	11.75908761	11.72807534	11.45932673	11.41605585
11.73710477	11.59021288	11.70138789	11.55106122	12.01956714	11.27153548
7.527697455	7.764931778	8.496365737	8.005727263	7.989899475	7.22559698
11.00183361	10.75178718	10.94942311	11.15342364	11.09565519	11.46539851
10.49830212	10.87907816	11.24816417	11.0035084	10.52585606	10.24828231
6.862895808	6.529317889	7.366646525	7.126812859	7.076028737	7.150454879
7.940978939	7.466913717	8.020887778	7.715032394	7.828584192	8.138156535
9.496413788	9.792275424	9.751080646	9.694925798	9.613873859	9.583475303

7.625433667	8.092218303	8.533396525	8.389653889	8.15355797	7.040799717
8.278157657	8.621272737	9.630502939	8.037287293	8.437457535	9.415217505
9.508442768	10.38842688	10.08700287	9.436898869	9.867911152	9.231349182
6.461008283	9.496413788	7.834378071	9.142159707	9.374690616	7.238429162
7.101573333	6.559042364	6.29940503	6.423456465	6.507609283	7.312858101
6.248527828	6.400763333	6.285518596	6.423050626	6.403910828	6.602902657
7.967113929	8.440878707	8.854603364	8.799825313	8.677707162	7.945864808
9.043522909	9.085558838	8.302498081	8.480158333	8.695776131	10.12909315
8.423588242	8.617840212	7.95565902	8.019769071	8.230996848	8.44446797
6.898792364	6.834834778	7.387965737	7.249066101	7.250899535	6.857502071
6.643553717	6.98921798	7.747885899	7.398696909	7.282065253	6.14867199
9.09824599	9.862946677	8.957944414	9.168828475	8.857327323	9.421700798
13.02006671	13.30255942	13.49745762	13.17778072	13.48990673	12.83827393
8.862785404	8.650384111	8.554971	8.98371896	8.668931657	9.313001808
9.640429374	10.03205964	9.534883657	9.620873889	10.07764109	9.925226333
8.352537101	8.311348051	8.620285051	8.811517808	8.471953717	7.772503354
7.665314131	7.324608717	7.353900202	7.366317111	7.106592788	8.246470545
7.010749455	7.427701768	6.698966242	7.006478434	6.941216071	7.103815788
7.164403717	7.688677182	7.299385535	7.590787525	6.873794323	6.780968242
7.663618646	8.086953081	8.17219503	8.453639616	8.246087697	7.386763141
12.47441306	12.24351077	12.41337695	12.76207554	12.76628311	11.79870316
6.74585002	7.071486061	7.47214603	7.413750677	7.290498626	6.314614657
7.402701172	7.019151556	7.334394303	7.395882727	7.349811747	7.557268586
7.988251818	8.030614293	7.705454838	7.849463182	7.836138323	9.286739242
8.494576061	8.111719586	8.438325768	7.97128602	8.144161414	7.752031929
7.836508475	7.907924051	8.088956818	7.691535778	7.988582505	7.337670899
6.946968899	7.006764162	7.368846081	7.205488778	7.637927172	6.532429758
16.52993947	16.82803465	16.96059838	17.01900663	16.82803465	15.86132141
9.025193869	8.527598192	9.726443586	9.402814081	8.907429495	8.491898242
16.06827159	15.58150095	15.56435861	15.449005	15.78787594	16.33688632
7.374842788	7.685352364	7.805446323	7.860366808	7.715405939	6.764835354
11.0940021	11.12315281	10.86305205	10.94786936	10.95374716	10.64738653
10.44934262	9.845595727	9.782133848	10.45628541	10.28190294	10.12041459
8.023099172	8.350619919	7.896287434	7.831503424	8.168587596	8.988577162
10.73661507	10.90324033	10.8193093	10.91514001	11.03452834	10.82804231
6.924473131	6.997628374	7.553068374	7.478150263	7.480348535	6.514109778
7.576037556	7.897743687	8.048636061	7.893631939	7.941721242	7.410364737
9.692793657	9.206093556	9.715870253	9.905108616	9.670530667	9.672623929
7.524103909	7.663284545	7.363617495	7.828584192	7.524103909	7.432376434
8.461315273	8.210186616	8.37004801	8.31049196	8.335469222	8.685201879
10.09682592	9.921211657	10.39414857	10.05053825	9.958247111	10.34100163
8.402148465	8.137479222	8.692466525	8.524867051	8.950355889	9.201355202
13.7211242	13.83772389	13.33155169	13.94443	13.89331098	13.94443
10.36903954	11.02243393	10.47479655	11.14707085	10.99046505	11.00183361
7.287828434	7.647332323	7.466006556	7.044228545	7.706567859	7.590558081
9.808672222	9.760900556	9.855298394	9.717365758	9.609027141	9.543825273
8.554528737	9.285000091	9.257324	8.81572204	9.348735909	8.252372808
7.807594768	7.559118222	6.817996071	7.162148556	6.803567182	7.619228051
7.116341071	7.168942192	7.655968232	7.120482	7.548584576	7.491357222
8.884521697	8.358418384	8.006495333	8.095330333	8.104133364	9.252443646
9.024212859	9.101613848	9.627745303	9.456392222	9.362238061	9.247858869
9.001272616	8.81966502	9.07419996	8.971727333	8.997224192	8.966282283
7.93888704	7.857845061	7.912365798	7.985458788	7.714004283	8.622644364
9.033394545	8.804477505	9.174120707	9.268661657	9.137373131	8.451550535
8.303793737	8.975838535	9.000069889	8.903025495	9.061649657	7.757833798
9.679645121	9.853021111	9.276789111	9.602761333	9.522660222	8.977005354
8.192645727	8.384837869	8.124599212	8.06745399	8.128290707	7.714317071
7.031184939	6.860943283	6.511217374	6.615899091	6.477093667	6.788535525

11.96947069	11.16423752	10.4322159	10.17702971	11.11560115	12.48124823
8.531247657	8.463063475	8.590695768	7.970574879	9.118629636	8.372433475
6.452131838	6.920433444	6.468467859	6.652872152	6.499941	6.218400657
8.011239071	7.803193929	8.12156602	8.327353333	8.291166909	8.505287909
7.481001515	7.762794545	8.165380303	7.804724687	7.784529071	6.956897717
7.677264869	8.921533879	7.114645596	7.670568384	7.653305051	9.359207242
7.159147152	7.42612497	7.868007081	7.836138323	7.641344242	7.096055535
7.588284717	7.535195111	7.498343283	7.627670859	7.936644384	7.688370495
9.547339828	9.144457596	9.297353606	9.224452333	9.315671101	9.465071586
8.388817192	7.869709071	8.221403505	8.096525737	8.028563859	8.275579182
7.413750677	7.21150504	8.263041909	7.360165061	7.670568384	8.287081333
9.749660394	9.133918929	10.0406788	9.90675497	9.899969596	10.10736711
8.820759798	9.19736601	9.663106646	9.644739414	9.334658071	8.254919778
12.93430142	12.67700968	12.89513437	13.10484893	12.88265743	13.01016522
9.545191596	9.354902374	9.323195364	9.790672212	9.672623929	9.151431899
7.352232505	6.428147687	6.340270525	6.451458394	6.462359596	7.757532545
6.454504667	6.394931444	6.86140099	6.74585002	6.72130502	6.297260404
8.937936253	7.986535808	8.569735879	8.546545061	7.973586899	8.240375404
7.572084505	7.302736424	6.531281859	6.769912384	6.621412929	7.980568657
10.97631922	11.54675633	10.47702836	10.8935809	10.74819911	10.49830212
6.574738525	6.395106727	6.709075525	6.655001313	6.638550414	7.242650333
6.248152273	6.843774949	7.438935576	7.418946535	7.114362495	6.310299848
7.183404394	6.818245747	6.786766677	7.294583061	7.546918172	7.246699182
8.321152475	9.382882596	8.316570081	8.922082808	8.330618758	8.809442889
6.515215919	7.293748525	7.370950798	6.736085162	7.367541707	6.807771525
7.748548515	8.317824071	7.965251131	7.738299929	8.057864374	7.094132182
11.46724679	11.18096106	10.64502212	10.78269205	11.49839615	12.52130815
10.77890896	11.55322016	11.20660223	11.42178201	11.2278827	10.69574272
8.087781242	8.090157434	7.617533182	7.783465061	7.766738848	7.949790515
7.822322859	7.451183323	7.847379061	7.799196616	7.989587677	8.069753788
6.448856899	6.587110859	6.512566313	5.844566545	6.769912384	6.660563556
8.725113212	8.666601818	8.678158051	9.051375475	8.703161646	8.546545061
9.005433152	9.558925838	8.096525737	8.572701232	8.520243879	8.302498081
7.227602475	7.472500828	7.500795283	8.01602699	7.502729737	6.715104081
7.724049626	8.823889374	8.32400903	8.669446475	8.433468657	7.962380919
7.25994097	8.104133364	6.785316677	7.117905152	6.97994296	7.576363505
10.35699559	10.16073454	8.163053768	8.137479222	8.852576737	10.40810668
9.318228697	8.925792808	9.175472828	9.032817303	8.890936242	9.286110869
8.072532475	8.01602699	8.639872707	8.558108202	8.115821848	7.519695475
6.631383222	6.540677515	6.984018879	6.909401626	6.733685141	7.054238101
8.717364515	9.395854121	7.849821828	8.211411717	7.951633808	8.131433848
9.437491071	9.672623929	9.377330121	9.577319545	9.231349182	8.826846768
8.928828384	6.618511313	6.340472131	6.669224869	6.464011586	6.553214606
12.59668127	11.95514832	12.09497108	12.20960187	12.25251148	12.53911948
7.490430596	7.85299496	6.400570727	6.916181	6.730688788	7.993875677
7.273507545	7.629673333	7.656544636	7.241229071	7.461509737	7.184502222
9.631182697	9.719000626	10.25519474	9.960013465	9.784374313	9.767642525
9.491725303	9.829119768	9.591133828	9.299916465	9.538263677	9.183367525
12.08680505	12.31719428	12.56726296	12.4571909	12.55625498	12.17216641
10.28506122	10.5752557	10.68572341	9.891739697	10.56207051	11.29399858
8.400903717	8.381754576	8.997224192	8.017104828	8.942313313	9.368875727
11.5386618	10.91514001	10.66832974	11.97447062	10.10278369	8.320817869
7.64232702	7.620680879	7.638629778	7.833640626	7.528986283	7.148192263
9.960013465	10.49466708	9.30949301	10.09597734	9.472030465	9.125507586
8.41598997	8.857327323	9.140978061	8.703161646	8.710391727	7.836508475
8.295839707	8.789132293	8.917069505	8.898231131	8.945493505	7.411561343
10.92485902	11.72807534	11.49636188	11.69188536	11.45526933	10.41426339
7.754082929	7.250238485	7.764609818	7.801818071	7.877119465	7.24440804

8.269395232	8.528915121	8.286610323	8.632349838	8.387939343	7.912365798
9.716648152	10.02362526	10.66706732	10.29752835	10.25906505	8.806957455
12.01427079	12.0089388	11.92156179	12.39660011	12.10694646	12.31089415
8.794788636	9.55462702	9.545881535	9.360502949	9.569063343	8.429582192
11.24147284	10.94353341	11.07797425	10.86691694	10.92060078	10.85225864
8.156772778	8.049757475	8.210994667	8.428657566	8.302062758	8.515350859
9.715215101	9.452497313	9.760900556	9.945028121	9.835894606	9.964343626
11.19293398	11.13879804	11.13713447	11.09565519	11.02551486	11.21816154
9.082448343	7.929187444	8.500729384	9.210065	9.133918929	8.893419636
8.691979949	8.632349838	8.79722998	8.499836475	8.817758687	8.633848111
15.88528111	15.22815255	16.3077943	16.3077943	16.52993947	15.42966281
6.503631525	6.538425424	6.433023606	6.728447091	6.544818747	6.804033586
9.329922273	9.477181455	9.716648152	9.715870253	9.545881535	8.60808904
6.655235768	6.550562869	6.630444394	6.338878222	6.821271707	6.97260104
15.72204116	15.67809237	15.88528111	15.61926687	15.63778652	15.61926687
11.35475345	10.98749259	11.04593523	11.18471248	10.97185531	11.68700199
8.298896434	8.129067798	7.273224596	7.610976808	7.76948301	8.279462
7.67197899	7.873377879	8.03179299	8.027386222	7.847720859	7.283800475
8.446641939	8.164575929	8.079973212	8.338355444	8.321919424	8.881793333
8.08737399	7.777311636	8.050105303	8.022369434	7.919459465	8.203151141
8.376704939	9.022478232	9.214694788	8.991970091	8.982611727	7.444499747
9.493200828	10.08700287	8.878201818	9.35067002	9.190767364	9.31393701
7.44112097	7.462172081	7.394370293	7.731871869	7.900715717	7.395300939
17.49517975	17.43906183	17.1822602	16.96059838	16.8710039	17.86179439
6.428820091	5.970702848	6.010869525	5.949701384	6.050028192	5.992641091
7.929593313	7.830100949	8.986432162	7.791533949	8.345781111	8.321152475
9.331038253	8.736738475	8.772410586	9.000672535	9.176146596	9.242526283
11.79405389	11.49239347	11.16734497	11.48258282	11.40495249	12.62127559
6.737335172	6.701799071	6.485516717	6.671323232	6.549138121	6.893115293
11.12454893	11.07337622	10.31597323	10.65751844	10.42409385	11.59427705
9.050265121	8.720163182	8.405355091	8.483326677	8.541125101	9.826793596
11.52824	10.25906505	10.72246175	10.4429644	10.53028115	11.72807534
6.78758299	6.93243001	7.24440804	6.778730485	6.628047111	7.092080626
8.78767504	8.713417869	9.176146596	8.627796121	8.676791091	8.908475283
8.327353333	8.692870869	8.664725051	8.585303495	8.411696677	7.762794545
7.938531869	8.207099091	8.092218303	8.266388465	8.079973212	7.356007838
9.339764051	8.990775909	8.742635939	8.619848374	8.694292242	9.580097263
12.22341713	11.6295292	10.0079343	10.83223099	10.41022215	11.77552743
8.214296636	8.070920919	6.995294596	7.328126596	7.373717778	8.19458801
8.233421293	8.187593091	7.862831293	8.003903253	7.886079293	7.994566778
10.4994288	10.76609029	10.7532413	10.79145221	10.50543333	10.36589334
10.01340346	9.900778232	9.844817354	9.839111	10.06904603	9.849671929
7.19832496	7.23423804	7.270452455	7.457078101	7.266262606	7.580594545
7.298118263	7.334066545	7.410622798	7.517077263	7.238133434	6.794527247
8.408718253	8.303793737	8.20265299	8.59942799	8.130616515	8.003147788
14.09521817	13.37822002	13.46712151	13.70143341	13.57574016	13.92172837
7.889761727	7.465036929	7.596246919	7.74753198	7.844877657	7.820030424
6.378748303	6.647920455	6.733685141	6.574452879	6.532834	6.510269939
9.712341424	10.60383217	8.52305899	9.538964939	9.499247869	10.09161001
13.30255942	13.34204398	12.53563192	13.04381444	12.62807191	12.56315114
8.604635949	9.182204323	9.356741384	8.954176293	9.024212859	8.287888051
9.504584061	7.796747616	7.477877475	7.820605525	7.707585606	9.154469939
7.200097273	7.641344242	6.526092091	6.877148152	6.825870455	6.367511859
8.024267525	7.387061818	7.063984253	7.104930788	7.076589626	8.164979273
7.480348535	7.394027687	7.489478697	7.573484121	7.246997929	7.697611606
9.11211799	9.096947232	9.433849505	9.254890202	9.238221677	8.677707162
8.34982299	8.443175586	8.207537162	8.41382397	8.406205495	8.495447152
7.956286283	8.315715768	8.914720172	8.844262616	8.44129198	7.189710394

8.297630758	8.729485838	8.338355444	8.585836293	8.47014499	7.781509414
9.978126798	9.466909636	9.742895778	9.565032515	9.76546198	10.14131493
7.433629313	7.787863889	8.859179455	8.178268222	8.126253929	7.149668323
7.907924051	8.517695303	8.126253929	8.074904687	8.152794889	7.31523196
9.606954152	9.665316566	9.620873889	9.671912455	9.692793657	9.499917182
9.273640758	8.354570556	7.28497204	7.470886646	7.358075697	8.844732879
9.238865747	9.067241768	9.747577162	9.424273283	9.677492576	9.199527535
6.771840333	6.36195701	6.172614384	6.454301414	6.349206475	7.218399283
7.927799515	7.865213	7.934739636	8.186741323	7.780116303	7.739667424
7.102414636	6.615899091	7.173570899	7.016713091	6.898276768	6.708797384
12.48835313	11.93975968	12.39058663	12.33907422	12.21312889	12.31719428
7.765298909	7.071197202	7.486746556	7.56165098	7.654890798	7.915342566
7.97284099	8.260123323	7.957660747	7.988251818	8.210994667	8.747892899
8.456593071	9.499917182	8.243637242	8.65914896	8.521696414	9.985290333
10.74172297	11.16579624	10.78551178	10.77387872	11.03163187	10.35809493
9.238221677	9.952967535	9.899969596	9.660861778	9.967178364	8.845785121
11.4413808	11.31449746	11.3403052	11.35475345	11.5386618	11.29744903
12.26775966	12.53197264	12.29548604	12.07810634	12.57107442	12.64265677
7.035420475	7.263217283	7.787223354	7.469344313	7.569572879	6.496343606
10.29949835	9.842388081	10.22361636	10.32309273	10.47941256	10.5752557
10.6768734	10.38329808	10.53141254	10.55070525	10.64005918	10.81796524
8.098302202	7.52469401	7.044969556	7.337989111	7.245319404	8.052077899
6.839681778	6.879719566	7.344013535	6.712342556	6.648141899	7.086698091
9.521192909	9.86223396	9.853021111	9.897493586	10.27236099	9.011040293
8.959665061	8.865915939	8.767872848	8.723452192	8.648914657	9.119831202
7.171336586	6.400997131	6.663425212	6.782198505	7.628336929	6.974926293
8.780633818	8.768863636	7.875808798	8.185943182	8.143776848	8.796713535
8.62991701	8.724556485	8.505287909	8.53715797	8.709464434	9.127251283
10.41731261	10.27035429	10.02549055	10.14999886	10.31693391	10.60507136
9.913634949	9.69725304	9.772980061	9.779012071	9.836777707	10.17789322
6.455790798	6.079743687	6.198948818	6.209590788	6.254907455	8.794361798
14.65397593	14.86987024	14.4427031	14.88238784	14.58095582	14.03424604
9.036569232	8.553595747	8.898231131	9.407554697	8.802385697	9.044608384
9.130293909	8.29291099	8.085049192	8.271029889	8.41335802	9.194378495
7.882525919	7.714317071	7.82475102	7.793711495	7.753046657	7.579350515
8.396963808	8.705608919	9.221852535	8.874692596	8.428657566	8.499836475
12.64708972	12.80602179	13.03496614	12.88708268	13.05428976	12.81735602
7.18112796	7.915342566	7.883157929	7.848096879	7.859293	6.648620667
8.455403657	8.391020323	7.799196616	7.823429646	8.050484323	8.346989566
12.69676846	13.01016522	13.44849295	12.7939548	12.82153585	12.86086109
10.80389925	11.52238659	11.5386618	11.56725755	11.24816417	10.59541023
10.71145754	10.86173253	11.18636156	11.01045685	11.308834	10.9889639
5.861310202	6.433824646	5.879155152	6.384073253	5.82221799	6.03543097
10.68960982	10.4549623	10.62536143	10.4450719	10.56548536	10.8443678
10.17613521	9.749004051	9.974493182	10.03385507	9.79615097	10.48185706
6.967457677	7.101049919	7.423442323	6.73803103	7.449946354	7.056486293
7.350971899	7.406158394	6.492652182	6.676225182	6.734736768	7.140009717
14.15092087	14.00297944	14.61202705	14.3093669	14.15863941	14.35197184
7.04319302	7.099269525	6.368932879	6.281398798	6.387499121	6.277726788
7.241229071	6.514326051	6.686787465	6.748324394	6.399225919	7.441392919
15.67809237	15.08134613	14.7528858	15.36066179	14.85646221	15.34617167
7.25402203	7.643944697	7.168942192	7.996770556	7.946541949	7.400588576
7.355380455	6.570263495	6.263342323	6.474509737	6.363691192	7.13105099
8.175968162	7.786927414	7.263217283	7.876418838	7.779426687	8.297224525
7.482605354	7.853350485	8.395713444	7.866656354	7.923423949	7.126812859
9.354902374	9.631182697	9.981718727	9.689129545	9.707282667	8.671294253
10.20748734	10.6240143	12.18792926	10.31289083	10.59924244	10.09506496
9.986114121	9.833438364	10.2761618	10.00450786	10.1421691	9.715215101

7.379463242	7.090758091	7.427701768	7.342871717	7.170007293	7.447087758
8.261007747	8.002342131	8.348145404	8.409136758	8.341977727	8.308776677
7.324298343	7.635907081	8.139691131	7.892604899	7.998587576	7.091067131
6.792458242	6.271013081	7.102116364	6.925244263	7.41439101	6.309473212
10.58812248	10.03026493	9.86640601	10.23247836	9.955574404	11.08107282
7.333224889	7.66930299	7.178323677	7.382214869	7.34349297	7.614831859
8.529888505	8.50111796	7.348618394	7.519990596	7.494510091	8.543686687
13.66784462	12.02514953	12.28105924	12.64265677	11.47127063	13.39037726
7.097914788	7.387965737	7.252038162	6.795141737	7.288727879	6.909401626
9.93099102	10.16355358	10.60161194	9.346872071	10.08799683	10.08231743
8.790130253	8.778117444	8.788663253	8.669992818	8.517288667	8.370830747
10.08145981	10.13671639	10.69793218	10.29660096	9.976273051	9.997192202
6.168988222	6.354564141	6.056434111	5.764180727	6.161616828	6.401803212
10.44392641	11.13713447	11.28207759	11.08936677	11.28731295	10.14631732
7.867668606	7.013137848	6.871668566	7.013931768	7.07551896	8.040196323
7.737346818	7.858612495	8.100222556	7.773582384	7.681488071	7.565085768
6.475960141	6.761831111	6.275248485	6.269158071	6.228606576	7.588938485
7.459115707	7.270788949	7.514105485	7.453021566	7.282365313	7.490096869
10.61867663	10.58469265	10.28506122	10.28795108	10.32750324	10.48659409
9.288419414	8.998971485	9.159785394	9.283736939	9.21067496	9.50710198
11.91416468	11.68038318	10.41426339	10.78705857	10.91903182	12.5521346
7.88753598	7.373116051	7.463067838	7.385541293	7.87472	8.006915303
10.76609029	10.61867663	10.4524716	10.52135963	10.23948175	10.34717129
10.36134852	9.864614818	10.33375706	10.3494333	10.43564423	10.63266161
8.532516465	8.166983455	8.012010707	8.465000141	8.116577384	8.495994869
11.49839615	11.34892675	11.3290504	11.38391359	11.47496104	10.93494087
8.081502051	7.982067081	7.963792818	7.89560499	8.02125603	8.217984283
8.656360697	8.866888202	8.795325869	8.719245818	8.951989212	8.810446485
9.257324	8.966282283	8.76478299	9.354289121	9.250770152	9.538964939
9.890949131	8.164979273	7.650858424	7.921213404	7.747112485	10.15783547
9.613206141	9.353057535	8.35372597	8.82842597	8.575566677	9.366312899
7.770465818	7.823083152	7.381324374	7.562627657	7.422224808	7.238133434
7.918105152	7.916025434	7.180839586	7.405823253	7.358688323	7.59656001
9.679062616	9.784374313	10.32635032	10.21484784	10.02161965	9.419020677
10.54565144	10.21769501	10.45041513	10.46664928	10.45996909	11.56725755
9.302103889	8.943861384	9.045168101	9.072403576	9.025193869	9.408173051
9.501970121	9.271759586	8.737323475	8.997807768	9.046362323	9.523397737
10.82034332	11.10761793	10.97185531	11.09250749	11.238428	10.51886057
7.935081758	7.325469747	7.245604273	7.385218303	7.300330596	8.066374202
11.69188536	11.12454893	11.9091429	11.89876792	11.29597305	11.71042621
8.136641818	8.093473121	8.310875455	8.246913576	8.097222414	7.559118222
8.502509434	8.821885465	9.775265525	8.887109626	9.116279576	8.833963152
7.753046657	7.469634172	7.457936747	8.722452253	7.448962859	8.178694051
7.235098212	7.114645596	6.824533111	8.176360121	7.012040828	7.847720859
7.747112485	7.801818071	7.103272273	7.349181162	7.159453505	8.148682091
5.761050778	5.768950131	5.810162828	13.12562627	5.761958131	5.764355545
10.24227579	9.713188212	8.607126586	8.961854929	9.462343535	10.73517618
7.126519737	6.473192182	6.956363394	5.773817667	6.468467859	6.535531162
10.7532413	10.92485902	11.11230451	11.39185194	11.00902763	10.4700758
8.412578899	7.90609097	8.029836697	8.093473121	7.983184263	8.840590677
12.66627862	12.47761486	12.32364634	12.27421104	12.19996238	13.17778072
8.334162556	8.068200283	8.147481596	8.051625212	8.394903626	8.351407394
8.662816202	7.838908253	8.056742909	8.35018502	8.205892515	8.930433505
9.656571869	10.00886723	10.10093339	10.37467131	9.983540212	9.545881535
6.394104788	6.353596172	6.494178707	6.490432818	6.381219657	6.36310503
9.03221704	9.432636424	9.205404404	8.796713535	9.015966535	9.071276071
9.672623929	10.13768673	10.33146686	9.972781707	9.959124273	9.226702434
11.37670858	10.97896732	10.94071069	11.39772243	11.51008986	11.17571904

8.238272808	8.441767061	9.17861001	8.713417869	8.756396081	8.861258687
8.03364202	8.658285303	9.190205293	9.18155302	8.368326869	7.879985051
9.009407333	8.836023374	8.870602515	8.977558859	9.048620525	8.998420707
8.337550606	7.973227556	8.627322343	8.511305758	8.427685838	7.518993111
9.447242061	10.14326599	10.09896607	10.06633871	9.989520798	8.991410131
6.066662545	6.313976677	6.211355596	6.376098505	6.530395293	6.521458212
8.657372182	8.245649515	8.38301802	8.310875455	8.492747404	8.48873997
6.919083737	7.902575141	7.305287707	7.884680111	6.741204455	7.231823384
7.838908253	8.381309202	8.551311374	8.442699879	8.597027768	7.287539616
8.990775909	9.667708556	9.702015293	9.734703111	9.523397737	8.601896768
7.141989162	7.007051354	6.486835303	6.716586707	6.640209303	7.68296396
9.045168101	9.198153652	9.182204323	9.154469939	8.968391182	9.315671101
7.278422556	7.775630727	8.694292242	8.110083838	8.079179394	6.655494515
8.504327141	8.540635232	8.790654051	8.744562374	8.693780273	8.26142704
8.548304869	8.390560192	8.899757485	8.806957455	8.896578162	8.978723121
9.538964939	9.06333597	9.376046232	9.510536	9.421059	9.878686616
8.138519566	7.437975364	7.799854778	7.936282586	8.678658293	8.345781111
7.853350485	8.236986626	9.339764051	8.656360697	8.449722687	7.496977949
8.146343545	7.742113232	7.095534354	7.697611606	7.572483949	7.647631172
9.66831603	9.729555121	9.17294002	9.206093556	9.043522909	9.604846424
10.04682541	10.11664425	10.27338352	10.03611568	10.10093339	9.963504081
11.33657706	10.4994288	9.223302313	9.742895778	9.552159131	10.95533553
9.167114	9.339095222	9.972781707	9.810255869	9.515069697	8.731576192
8.972363838	8.527198758	8.514431737	8.692466525	8.625920364	8.833337414
9.18155302	8.70424704	8.59655801	8.787164939	8.579336081	9.561667293
11.07956266	11.1453264	11.62494396	11.3403052	11.50425191	11.09089144
13.38447499	13.41514643	13.6823979	13.70717091	13.60979651	13.46712151
9.733230051	9.36570098	9.426142444	9.649399939	9.986114121	10.22865659
7.591751808	7.816627253	8.176718545	7.887186596	7.772503354	7.779426687
13.02518615	13.50411017	12.96662998	13.20018245	13.14611959	12.41337695
9.66383	9.269847242	9.437491071	9.452497313	9.254253192	9.824540899
6.984574354	6.653830545	6.978374222	7.193571	6.800063838	6.968641576
11.50814936	11.43938222	11.18800276	11.16281786	11.71443529	11.69440164
8.62460002	8.035288182	8.619347838	8.539776737	8.561789232	8.564847253
7.501777556	7.426803586	7.621022111	7.585232141	7.737649848	7.805446323
7.551170919	7.377037515	7.727729838	7.613815293	7.535195111	7.876807081
9.110297333	9.179819687	9.215289101	9.260036485	9.305189859	9.184000242
8.667049253	8.326884343	8.055566596	8.301657889	8.563501616	8.59942799
9.387059283	9.217165182	9.10558002	9.373397364	9.428190232	9.457741556
7.969427899	8.332313525	7.606699253	7.542064859	8.125818909	7.776629576
8.888766747	8.652374404	8.991410131	8.882882525	8.869521111	9.236429667
8.448929687	8.571676263	7.872008071	8.268510495	8.245222778	7.731542758
9.363307667	8.779146061	8.782655051	8.801904485	8.846850111	9.084956182
10.85100254	10.66945215	11.14385742	11.18800276	10.99345288	10.26729954
7.025866354	6.751979343	6.754244737	7.20918101	6.930987343	7.507381747
6.689933697	7.418256545	7.989204111	7.59859104	7.462491444	7.261194687
8.923097444	8.871686232	8.943861384	9.091299889	8.864955909	9.005975162
9.638947465	9.18155302	9.509226798	9.631972414	9.390110384	9.980879424
8.668062838	8.491049758	8.511758747	8.62412801	9.023071899	9.033915556
6.868868606	6.740749081	7.259665485	6.957636657	7.131581374	7.18482696
8.874692596	8.838020616	8.997807768	9.377908	8.806957455	9.284395657
7.814093232	7.454260455	7.706168626	7.701029869	7.561342071	7.919459465
7.838173152	7.503050071	7.972411364	7.900011667	7.915639949	8.280707687
7.674820101	7.916712838	7.240898899	7.542783869	7.479119	8.263464364
7.841729333	8.246913576	8.563941778	8.493155899	8.290353162	7.299714313
7.494168788	7.174439091	6.853963192	7.186327495	6.981618121	7.478150263
10.66465811	10.49140257	9.819688798	9.769994879	10.06071544	10.50166798
12.02811467	10.85654613	10.13276962	9.937540263	10.05880999	12.00644476

9.098746404	8.799825313	8.618800343	9.135618929	8.913024061	9.062782333
10.06904603	9.966143818	9.814415596	10.09795649	9.804037697	10.21975253
9.660861778	9.756840071	9.544495798	9.805520727	9.655770101	9.025193869
8.290353162	8.218433131	8.079179394	8.065153556	8.01602699	8.292509485
8.243637242	6.604509566	6.592903869	6.684542485	6.644015919	8.269395232
6.851375081	7.936282586	7.506099556	6.836969222	7.275223848	6.923688879
6.186015354	5.965483919	6.353796111	6.332645192	6.338878222	6.299035566
7.262427071	7.686775222	8.097222414	7.787223354	7.685352364	6.772767788
7.114036293	6.945089283	6.82018896	7.176878273	7.183932212	6.902984
7.891531071	7.356848556	8.239069586	7.970233808	8.33796001	8.013487535
9.690534253	9.785178838	10.0719806	9.910074283	10.35385551	9.653591273
8.736221495	9.618955576	8.187136596	8.587234515	8.702574091	8.189547909
9.494490616	9.721958051	9.556759333	9.793126455	9.728754818	8.874692596
15.48449671	15.86132141	15.50564301	15.90458538	15.70163214	14.85646221
8.440878707	8.829061111	8.724013404	8.702074263	8.567801737	8.004655889
8.368326869	8.104500051	8.406205495	8.502509434	7.767362636	8.631899566
10.45375986	9.759214424	9.868690293	10.18000397	9.684886576	10.06450841
6.843545424	6.796334212	6.847553121	6.884322333	6.889757091	6.564057303
7.493565131	7.795429838	7.683318778	7.682588111	7.597232061	6.844744919
8.844732879	8.720627081	8.884521697	8.930963202	8.756886111	9.449948384
9.797856576	9.208914778	7.18511501	9.301561	7.278422556	8.370422404
6.087153556	6.348225889	6.584604515	6.491055869	6.385400465	5.995024848
6.889990505	6.76459802	7.58642403	7.13441502	7.210929778	6.853963192
7.358963616	7.283275424	7.323124384	7.354199768	7.469634172	7.752687697
9.934615404	9.950180667	10.25220538	9.767642525	9.813557354	9.625623061
11.12151875	11.23641801	10.4283734	11.00053539	10.71279176	11.01376888
9.607593848	9.502644929	9.570470929	9.482964828	9.825199323	9.441908576
6.510975394	6.501664081	6.67377398	6.480989929	6.55275403	6.548447101
6.333216859	6.113628535	6.506758646	6.339262212	6.350873162	6.651970061
11.39594259	11.16281786	11.69440164	11.68948846	11.41238356	11.31852116
7.809644101	7.957018646	6.593877636	7.088821283	6.866319192	6.330516949
9.654354424	9.691258919	9.172421828	9.374690616	9.297353606	9.031713778
7.915639949	8.211847566	8.072532475	7.999737535	7.979449616	7.903688606
7.799196616	8.133538364	8.099825778	8.044608626	8.055171202	7.505759091
8.133993636	8.931557869	8.727989172	8.790130253	8.494130061	7.831138545
7.720541949	7.361380131	7.582966818	7.487940131	7.641985	6.986766929
8.052783737	8.102862354	8.002342131	8.402148465	8.695335444	9.045168101
9.90675497	9.190205293	9.400327152	9.825199323	9.766131535	9.500664495
9.099242404	9.057828929	7.970233808	8.334162556	8.199315667	8.510413414
7.796747616	7.845242687	7.975414556	7.935875313	7.869992939	8.80133401
9.203760495	8.773471869	8.783158111	9.002847848	8.706567556	8.729485838
9.158666485	8.716864899	9.12042397	9.019287687	8.979921919	9.004940778
11.34892675	11.1276132	11.23280633	11.1502402	11.39983314	11.68948846
9.413321525	9.694213162	9.857940545	10.13862753	9.774477667	9.579413505
13.76702458	14.11151028	13.5360606	14.13521971	13.69473258	12.7939548
9.468772697	8.777050384	9.583475303	9.513795939	9.426142444	9.466304111
7.860764394	7.496977949	7.55945201	7.313208869	8.045324071	8.039790061
10.12909315	10.53737043	10.48539779	10.27804811	9.935661677	9.155079848
11.71042621	12.18142186	12.22341713	11.89361272	12.31719428	11.30301738
6.796566808	6.335449051	6.095658889	6.15603497	6.023382354	7.297158313
6.597486293	6.720541949	6.86313897	6.791503909	6.794655232	7.784199556
9.601568657	9.863774293	9.818059192	9.018299374	8.902484929	9.426142444
6.408579424	6.230855899	6.352434697	6.496103798	6.313742556	6.522975131
8.651452677	7.858214495	7.42989803	7.41959598	7.439506505	8.603745778
13.87263802	13.93694444	13.74759592	13.76108056	13.97489728	13.52959744
11.9982543	11.98999321	11.50051559	11.77305263	12.09225922	11.81439214
12.53911948	12.92081703	12.63102018	12.61761735	12.7939548	12.57877126
7.440186081	7.387388131	7.008962394	7.272331859	7.112259949	7.305287707

9.500664495	9.415217505	8.613985131	8.913024061	8.829061111	9.207791152
6.449076475	6.147877354	6.71776502	6.690165707	6.884803323	5.933931576
14.64328928	14.11997744	14.28325373	14.58095582	14.62217632	14.68633385
9.245445293	9.753529758	9.958247111	9.635582141	9.653591273	9.009407333
8.929384727	9.303966081	8.736221495	8.957404525	8.874692596	8.057421596
10.75453103	10.8443678	10.53505365	10.60277882	10.90324033	11.21668249
8.006915303	8.002011657	8.256076333	8.063008152	8.268052495	7.779041818
11.57765358	11.50814936	11.55524693	11.46145043	11.04316622	11.28731295

GSM518907	GSM518909	GSM518911	GSM518913	GSM518915	GSM518917
7.293153485	6.441255838	6.422232	6.810582646	6.827661505	6.568165434
9.690534253	8.969483	9.481000434	9.480384485	8.008400859	9.049629657
7.294583061	7.045230232	7.113720848	7.524103909	6.592293788	6.620481919
8.331046747	8.896578162	8.874191697	7.77328201	8.94874	8.517695303
7.432376434	7.404680798	7.633233848	7.421928768	6.876372364	6.833605444
7.523479626	7.598885727	7.55847901	7.841440677	7.760648222	7.748187364
8.26142704	9.200683333	9.185497838	8.748883111	9.217871889	8.613130333
7.59435096	7.721939444	7.660150727	8.133538364	8.012698303	7.719108384
9.553430788	10.34100163	10.49241362	9.365016101	10.66832974	10.61261659
8.636577313	8.315715768	8.318250424	8.319901444	7.697611606	7.817975838
7.970574879	7.540434798	7.575420636	8.694292242	8.035288182	8.197805768
8.672348475	8.460863192	8.053971495	8.904793061	8.867918475	8.548679899
7.676257364	7.648319737	7.853698798	7.79851096	8.254919778	7.883917657
8.294235768	8.037984616	7.81061403	8.601896768	8.323598384	8.271405859
8.532516465	8.779146061	8.399553939	8.158321101	8.602859283	8.39227803
7.866306808	8.73262002	8.710842768	7.85268101	8.494982343	8.658802051
7.641344242	7.643944697	7.550195808	7.688677182	8.06213903	7.446760616
7.787223354	7.331457121	6.984849141	8.074096707	6.744072859	7.603101596
8.042161798	7.603389051	7.608347495	7.917403434	6.896472707	7.249375465
8.254919778	8.036387556	8.520243879	8.714332636	8.859179455	8.86024204
10.01257663	10.65871503	10.66229262	10.59418919	11.04464523	10.56207051
11.68700199	11.01951417	11.33657706	10.82804231	10.10093339	10.58579258
9.050265121	9.378583414	9.520598525	9.515743253	10.06173934	9.790672212
9.406893465	9.504584061	9.163332636	8.479185606	9.333986848	9.460382818
11.25349741	10.87092264	10.97185531	11.60680648	10.82945836	11.36245739
12.01191659	12.43944324	12.84627851	10.69793218	12.42616911	12.19086998
9.537497323	8.757866131	8.076118121	9.095162899	8.633848111	9.155079848
10.17224075	8.573273283	9.222631263	8.639417101	8.44446797	9.121511687
8.660608859	8.325717687	9.214111545	9.185002364	8.766842081	8.469779808
6.349593626	6.367696616	6.516492273	6.891782758	5.97484504	6.198389818
8.655934636	7.982442283	7.842052606	7.272599475	8.653441707	8.721926778
10.5431016	9.000672535	9.003357768	8.913588253	8.662363172	9.134591697
8.665165081	6.989443677	7.343153889	7.084738172	6.931501667	6.931948737
7.747885899	8.59942799	8.040196323	7.700670768	7.734170172	7.844877657
8.198176101	7.836828747	7.887186596	8.494982343	7.774617596	8.044608626
7.719466677	7.775998535	6.780688182	8.282438737	6.351069404	6.959689121
11.24457024	10.20060626	10.40701089	10.13193078	9.095762	10.38329808
8.058246657	7.91707401	8.125376242	8.210994667	7.98506802	8.300103657
10.36781996	11.45176908	11.30301738	10.6820228	11.20498985	11.55106122
11.17750487	12.05813886	12.06692054	12.0089388	11.92156179	11.57178154
8.094218354	8.270601414	8.259704283	8.155735586	8.532980343	8.275152535
13.35919327	14.50017253	14.47046648	13.52959744	14.5513462	13.86604428
9.493200828	8.843148071	8.943861384	7.982442283	9.148511889	8.463063475
9.323195364	8.733877859	8.39227803	7.755132808	8.581684657	8.372086737
8.149032212	6.830578646	6.940977263	6.450135586	6.560197424	6.449932949
8.382194192	7.525550222	7.635907081	7.67441401	6.82246696	7.180291929
11.24816417	12.11562205	12.10135206	9.953782444	11.58621592	11.32027501
6.311249485	6.713303939	6.569103444	5.948711596	6.556473354	6.341475172
10.92190902	8.932594273	8.646531515	9.232683343	8.513601889	8.48873997
9.816676162	9.46096901	9.31327601	9.650129091	8.471488636	8.687969051
9.016491202	8.611286687	8.841037687	9.329342374	9.046862848	9.341818545
8.778117444	8.45451298	7.74578199	7.870356606	7.74753198	8.047188455
8.895503556	6.587802667	8.735755485	7.838558162	7.130781323	7.700026646
8.635209293	8.745043505	8.639872707	9.53973704	8.821885465	9.322592778
10.81664077	10.48432644	10.20452777	10.761049	10.82664672	10.70052069
7.377557566	7.060512141	7.229587778	7.29078701	7.639261768	7.226432323
8.995584808	9.637663091	9.435122859	9.067241768	9.315671101	9.231349182

8.617840212	8.324880909	8.434412929	8.484240687	7.314926455	7.858214495
14.9727616	15.81050193	15.74256723	15.52328152	15.97330865	15.50564301
6.556698131	7.016713091	6.942817626	6.383610909	7.038917566	6.612514242
10.09597734	9.451851384	9.461673394	8.894948323	9.050265121	9.144457596
7.101319909	7.369510384	7.168718192	7.519990596	6.67377398	6.859929394
6.878694758	7.103272273	7.358688323	6.329524818	7.313208869	7.109678444
9.264989899	9.915234909	10.00186072	9.679645121	9.918671212	9.405539273
6.362493667	6.131204707	5.771070222	5.776494313	6.286294667	6.03924
8.009790253	8.203586121	8.575566677	7.864224778	8.245222778	8.648450303
9.472607081	9.671170444	9.36570098	9.844817354	9.338458455	9.277455131
6.845030273	6.774936697	7.057011424	7.900715717	6.103276869	6.334611455
10.70181173	10.48185706	10.60863639	10.48659409	10.69470446	10.98350754
7.691187768	7.24440804	6.951451	8.162707626	7.380085202	7.747885899
7.896600606	8.128726778	8.020887778	8.692870869	8.44129198	8.228510596
12.85589012	12.73352188	12.80949032	12.85589012	13.16824615	13.07454735
7.809338131	8.453639616	7.635267465	8.119679141	7.98690202	7.822322859
11.30301738	11.17041501	11.18636156	11.71443529	11.26262606	10.16454544
7.81061403	7.451812333	7.766738848	8.456191465	6.393414081	6.744595263
9.015412101	7.123768636	8.660608859	7.876807081	7.206373697	7.770819434
9.876096838	10.40178901	9.907546465	9.476588061	10.05607537	10.07857135
8.845785121	8.078774939	8.275579182	8.441767061	7.224435778	7.688370495
9.338458455	8.996667384	8.934248131	8.171788646	8.613521394	8.621272737
9.861430101	9.685649929	9.889177586	9.601568657	9.395854121	9.740584727
11.25523353	10.56802411	10.48310631	10.17904437	10.59924244	10.6313464
7.788923101	6.995294596	7.029601354	6.873794323	6.802568323	7.018424152
7.684641212	7.475616394	7.523206263	7.948737212	7.638274606	7.909763434
9.281923404	9.452497313	9.983540212	9.214111545	9.231946182	9.217165182
6.159344697	6.492200283	7.480656747	6.354771242	6.326100737	6.262744758
7.661901192	7.972012263	8.115437899	7.122942091	7.536878778	7.69973097
7.662925475	6.839681778	7.591751808	6.92802298	6.910411354	7.178076949
7.810269677	8.420871687	8.367422212	8.826846768	9.073600626	8.335861768
7.985767212	8.792195697	8.704708384	9.160413333	9.037153444	8.670447949
10.24316974	10.37894268	11.1502402	11.07493153	11.48459908	10.69354222
7.31937996	7.098999354	7.123768636	6.702978071	6.307709909	6.479009949
13.83772389	14.93426708	14.89443037	14.56207529	14.9727616	14.62217632
9.205404404	9.283145788	9.233272283	9.66613299	9.455711606	9.449948384
9.300367869	7.748548515	8.33024502	8.27018399	8.118473111	8.200961636
8.798818646	8.618302869	8.652865111	8.919862162	8.610337293	8.775519707
7.851994586	8.317411434	8.32856404	8.661956242	7.928832091	8.84935699
10.83100199	9.721175788	10.10365156	9.691258919	9.660861778	10.08978265
9.408173051	9.185497838	9.000069889	9.634173323	9.078331919	9.326739394
7.59656001	9.101086141	8.768863636	8.208692758	8.981975535	9.031713778
8.013857071	7.731871869	7.957660747	7.985767212	8.044608626	8.286610323
9.059459505	9.411394242	9.400967465	8.333227545	9.109760465	9.284395657
7.561051303	7.672658859	7.623386535	6.645661505	7.322572717	7.387061818
8.124599212	8.57070398	8.207099091	8.797743222	8.765765323	8.472435657
8.22344601	7.415639222	7.452109758	8.551751717	8.39402401	8.285708747
9.359207242	8.170129495	8.334587636	8.103690354	7.987573434	8.143040162
6.385648535	6.339077535	6.541858778	6.778194636	6.719301162	6.644710828
16.36806566	16.64557528	16.02065153	17.58093558	16.68963031	17.43906183
7.416641556	7.423442323	7.973992111	7.794719919	8.156772778	7.919459465
9.890135131	10.67305197	10.65871503	10.85100254	10.72246175	10.32093584
6.736085162	6.964897566	6.774688626	7.026621727	6.846509828	7.306206455
15.12351424	15.03840752	15.10875812	14.14208591	14.70594079	14.78379092
9.730279475	10.3305428	10.28190294	7.757234778	9.66383	9.737663949
9.399129424	8.909945455	8.892970697	8.981975535	8.352537101	8.747331152
7.894965162	8.040575909	7.407119727	7.592689374	7.558798354	7.499556727
13.40990013	13.94443	13.90036211	13.82538175	13.95167504	13.36582475

12.41337695	13.03957854	13.10484893	12.38063147	12.7494647	12.68903729
8.326518101	7.314616131	7.60255999	7.275223848	6.573072253	6.813695162
10.97631922	11.50051559	11.92665931	11.72592113	11.6137143	11.58421758
7.384903253	7.540114586	8.053207788	7.194677455	7.676615465	6.729687141
8.354570556	7.969046636	8.471953717	7.798177384	7.734550869	7.734896879
9.198999222	8.39227803	8.721926778	8.563085788	7.869033162	8.32856404
11.06123261	11.67593314	11.60069924	11.42787377	11.98196729	11.55322016
8.758364879	8.680072242	8.857327323	8.613130333	7.479706475	8.318250424
12.80602179	13.87263802	13.91489381	13.63435472	13.83173935	13.49745762
7.067867747	6.969691626	6.744595263	7.553773697	7.215770273	7.379773919
11.93975968	9.596773313	9.294355394	9.420326576	9.133918929	9.746145949
9.956562586	11.04886125	10.76609029	9.550769788	11.16281786	10.66832974
9.731806091	9.091299889	9.231946182	9.318228697	9.020821455	8.879330566
7.869709071	7.889761727	7.764931778	7.842359758	8.774537848	7.753046657
8.126253929	7.994566778	7.964179182	8.768300525	7.404985061	8.036816444
6.165987838	6.001077121	5.953449444	5.772292687	5.860536929	5.822958545
8.74840203	6.968936475	6.914843414	6.76551097	6.509844303	6.679485202
15.50564301	17.23317693	17.01900663	17.07010739	16.82803465	16.64557528
6.254184596	6.056307162	6.377507434	6.230315707	6.262348667	6.307278677
8.17219503	8.483326677	8.233006697	9.189590182	8.717867636	8.77608297
7.527339687	7.130479657	7.249695535	7.242080394	7.332963121	7.644626202
8.952561111	9.532335293	9.61181703	8.151663141	9.329342374	9.201355202
7.772503354	6.490634808	7.126812859	7.784529071	6.683324051	6.978866758
6.324763646	6.679264929	7.081850444	6.727165222	6.513435323	7.888681737
7.364257101	6.988144556	7.078303869	6.282012172	7.228759818	7.471847707
14.47046648	13.56886427	13.73439002	14.1830221	13.60979651	13.95167504
9.671170444	8.22344601	8.47014499	8.262596626	7.519990596	8.479185606
7.029601354	7.988251818	7.114362495	7.747885899	7.478150263	7.550845909
7.792215626	7.947667091	7.996041859	8.544134919	8.032161838	8.409136758
7.408375899	7.138031798	6.832629131	7.801818071	7.410944101	6.984268657
10.91356479	11.04030995	11.08264222	10.88809182	10.47941256	10.80644997
8.491898242	8.751405818	8.759757687	8.569735879	8.102124455	9.235747242
13.25686564	12.11216258	12.42943771	12.69316533	12.20640951	12.3543152
7.097545323	6.830856061	7.109400202	7.075644409	7.179153636	7.301216929
7.500167182	7.394983566	7.593038414	6.629727051	7.161342051	7.396823121
9.634173323	9.885156737	9.797856576	9.766131535	9.89348604	10.20964764
7.808676939	7.349181162	7.443557333	7.610260323	7.46337499	6.79984598
10.72786832	10.44934262	10.04862183	8.401317182	10.24942024	9.393934131
12.92937388	12.75396286	13.29684189	13.74759592	13.33155169	13.39037726
8.96575796	9.321347081	9.425521616	9.084401768	9.736957071	9.343457394
9.478383576	8.98371896	9.821914929	9.856174394	9.368224626	8.990775909
8.921011566	8.863375313	8.883414061	9.332175414	9.049171303	9.044046343
9.529469889	10.14326599	9.829119768	10.1885747	10.46342714	10.2600862
7.211210626	8.585303495	8.551751717	7.424346313	8.03490996	8.192645727
8.745474636	9.388273121	9.125507586	8.66101001	9.239472869	8.674281929
7.32489101	8.016429949	7.825440475	7.791533949	8.185177828	7.536878778
8.471953717	8.534432515	8.405355091	7.620680879	8.377562293	8.198599495
9.143918798	9.50650501	9.283736939	7.734170172	9.158023	8.78664797
7.217862778	6.560909667	6.812413525	6.462851414	6.646655162	6.553214606
10.33696433	10.75987116	11.05959848	10.33696433	11.38178745	11.13713447
7.25402203	7.169190859	7.316095192	6.368932879	6.895709172	7.011783192
7.417927455	6.882764848	6.86860504	6.775501273	6.706413566	6.900488939
10.08893073	9.966143818	10.05880999	9.145078374	9.973623707	9.655088667
6.84607197	6.953635141	7.137147091	7.568924192	6.824112909	7.013137848
10.25906505	10.83363242	10.82416773	10.78551178	11.01951417	10.86566339
7.848409778	8.985821889	8.974101818	8.789132293	8.972927475	8.668931657
8.468418253	8.112525414	8.279462	7.98690202	7.502072596	7.949071818
10.21975253	11.54675633	11.51008986	11.13879804	11.31449746	10.92608881

8.404822414	8.810986707	8.928828384	7.810269677	8.887109626	8.43006898
8.663769939	8.633351606	8.216823323	8.489178071	8.572701232	8.340681505
11.68243664	12.39660011	12.50706636	11.32185019	12.39973853	12.24649354
11.38391359	11.80104424	12.15130062	12.53911948	12.11841559	12.22629088
10.43872423	11.29744903	11.20840189	10.39747702	11.25898056	10.55726192
7.421037465	7.876138384	7.639990515	7.099269525	7.606043535	7.841729333
8.152087687	8.222643394	7.877119465	8.222223545	8.492322828	8.993505475
6.665428859	6.490215273	6.10922399	6.097038818	6.806762071	6.711662182
9.044608384	9.267383556	9.071276071	8.443547162	9.268661657	9.096395636
7.094997242	7.936282586	8.137069859	7.936644384	7.888681737	8.454049747
8.220625586	7.501777556	7.545042545	8.677215515	7.105750636	7.726692222
7.624735818	7.900388485	7.889031475	7.691187768	7.137450808	7.33182303
9.470173818	8.558108202	8.692870869	8.407442505	8.086153313	8.26142704
7.525850576	7.943433747	7.678926152	8.328992859	8.096910323	7.272599475
13.22156044	13.52334642	13.78027417	13.42888134	13.46712151	13.10484893
7.799196616	8.057864374	7.646666444	6.831129859	7.974325596	7.418614091
11.21338774	11.85033795	11.85617239	10.44094498	11.64848719	11.35080354
8.951989212	8.985325394	8.882882525	8.485174677	9.189045596	9.465659495
10.49830212	11.5386618	11.43380494	10.98186414	11.06732533	10.99345288
9.496413788	9.927686616	9.902579253	8.93587504	9.647013263	9.356058495
7.004818101	7.512765949	7.409335747	6.560443758	7.432999727	7.138289616
8.24122704	8.059423535	7.930919909	7.374563525	7.539167263	7.740469253
9.646274414	9.776037091	8.586230848	9.221852535	9.694925798	9.087255717
9.136890505	9.400327152	9.155642525	8.440058758	9.788274313	9.719000626
8.845226141	8.423588242	8.529417101	8.836023374	8.940148162	9.324974768
7.890755525	8.060252263	7.907197636	7.521572545	8.106019798	8.126700889
8.648914657	10.12126634	10.04776272	9.74383503	9.763138212	9.366312899
8.007640657	8.410908071	8.316081222	6.966706414	8.691979949	7.787863889
9.228437333	9.872596081	10.07956055	8.921533879	9.593075545	9.254253192
12.47761486	13.72760196	13.47832004	13.00581854	13.77352346	13.14093405
7.999327475	8.637033606	8.647073404	7.640998838	8.37004801	8.340252101
9.264483889	9.715870253	10.05320582	9.221294354	9.894213939	9.37540303
9.349388778	7.906781182	8.997807768	9.127872697	7.801006182	8.091725323
8.785177889	6.958444212	6.951169364	7.340147596	6.722483515	7.352546465
11.58621592	11.6157426	11.43565115	12.01191659	11.73710477	11.51465712
7.804724687	8.604635949	8.050868949	8.606099333	8.59655801	7.797125465
9.519820636	8.957404525	9.04056096	9.081281455	8.445759657	8.608532485
8.751946515	9.629101929	9.846508495	8.50111796	9.167114	9.425521616
10.36589334	10.59062542	9.766921414	9.876880455	10.15585749	9.915234909
7.016713091	8.247392788	8.226165	7.275508121	7.889381343	7.611330343
8.680072242	9.184527788	9.203189636	9.684886576	9.322592778	9.095762
7.216096788	8.10140802	7.945864808	7.506099556	7.20666299	7.145493505
9.960013465	9.760900556	9.785178838	10.1597097	10.20748734	10.04600105
10.02741959	10.47584162	10.05144087	9.239472869	10.73911267	10.31693391
10.27035429	10.45628541	10.59685822	10.94786936	10.80389925	10.52247938
7.444823313	8.043387838	7.912742707	8.430534091	8.331458747	8.482852263
8.094989869	8.598980253	8.747331152	8.578393657	8.345353354	8.600356081
9.723272263	8.787164939	8.930963202	7.945864808	8.498949848	8.524356212
6.887875313	6.244906081	6.094616111	7.083013909	6.598705667	6.725034798
7.249375465	6.900488939	7.000584848	7.743818828	7.309054838	7.533990333
12.14191765	12.82153585	12.47130519	12.15752365	12.96241124	12.06692054
14.06476988	14.79546777	14.59173737	14.25042984	14.57091557	14.35197184
14.76275492	15.34617167	14.93426708	15.03840752	15.32551587	14.90726461
12.58612865	13.2504616	12.89513437	13.12023156	13.2158469	12.80949032
8.571194222	8.687969051	8.065153556	8.584413131	8.509936919	8.591239465
7.198597879	7.92597801	7.959488687	6.616628394	7.939228495	7.85268101
12.08128174	12.93862501	12.76628311	11.78265022	13.09463792	12.21630458
9.884409465	10.27714677	10.00450786	9.493894545	10.0672481	10.05144087

8.762742172	7.817596162	7.95873603	8.671294253	9.214111545	7.745466071
9.018802576	9.472030465	9.583475303	7.823429646	9.012031232	9.062782333
7.98506802	7.714642404	7.711891616	7.718373848	7.216363677	7.378508566
6.643553717	7.245319404	7.420741101	7.353560545	7.348317141	7.23452504
12.75396286	7.894965162	8.039451586	7.579628838	7.65457904	8.069395848
8.247392788	9.096947232	8.818210192	9.126080354	9.418269535	9.117478424
8.595703707	7.435155859	7.760648222	8.116928465	7.372238061	7.60255999
7.592689374	8.18481599	7.591105343	8.553595747	8.095330333	7.843855828
10.03205964	9.839111	10.03026493	9.025691242	9.200042253	9.383480283
9.407554697	7.883157929	7.696259101	7.242384899	7.299714313	7.627030141
8.321919424	8.802954374	8.701109788	8.196571586	8.181963182	7.792215626
10.97185531	11.73271713	11.76101506	11.84027237	12.27773509	10.9889639
10.5752557	10.30810853	10.78014639	9.940265465	9.940265465	9.830354566
8.580722586	8.127056495	8.195856758	7.276635293	8.127056495	7.98690202
8.988033424	8.934248131	8.292068253	8.743617505	9.144457596	8.434412929
8.929384727	6.389669667	6.680176879	7.159715333	6.486601465	6.940977263
11.09885051	10.8605042	10.82945836	11.1612024	10.57996368	11.21958266
7.788172758	7.845981061	7.768055657	7.555972677	7.904381697	7.949790515
12.64265677	10.31013098	10.39968902	10.21375666	10.21975253	10.69931975
6.526092091	6.589360121	6.681596343	6.381019303	7.035420475	6.951961606
14.3093669	15.12351424	15.09418019	15.13975511	15.31193978	14.9847254
8.405727848	9.335272182	9.252443646	7.909412687	8.901935111	9.229082869
9.283145788	9.182204323	9.228437333	8.014589606	8.678158051	8.968391182
11.12001247	12.3613163	12.51066587	10.44602022	11.83330316	11.51647662
8.612168455	8.708030737	8.450646323	8.976374343	8.526649808	8.556357949
7.245604273	8.389653889	8.427236737	9.139095455	8.174871818	8.282073859
9.756054152	8.155393222	8.258860253	7.872350636	7.809644101	8.114611232
7.704008545	7.445127778	7.560760293	7.656238101	6.964119424	7.236914141
8.684654737	8.733083212	8.485631939	8.646531515	8.050105303	8.261007747
7.368501293	7.864861071	7.728706424	6.606673949	7.797125465	7.579628838
10.48539779	9.21352099	9.053043889	8.81572204	8.888766747	9.029402949
8.234232515	7.132177788	7.078835444	6.783369808	6.957403667	6.860671293
11.33848921	11.07956266	11.09721747	11.80104424	11.80791686	11.55928848
8.140977576	8.539776737	8.284876758	6.887014152	8.092691677	8.060252263
10.88809182	11.30131952	11.02851208	11.65455063	11.43208235	11.19638858
13.98081649	14.07245549	14.223913	14.57091557	13.85817003	14.24162707
10.56421743	11.1031408	11.00496707	11.12315281	11.11856721	11.1060681
7.336145273	7.778752535	7.752031929	8.032161838	8.181196919	8.417196576
9.701165495	10.05880999	10.26729954	10.69354222	10.56421743	10.41530743
7.053922394	7.511268929	7.387654737	7.755825222	7.677613273	7.389236737
11.77802923	12.49965499	12.31419772	11.23116994	12.27421104	11.81221198
13.76702458	13.82538175	13.76702458	13.2158469	13.78737352	14.07245549
10.95533553	11.18291631	11.39983314	9.790672212	10.71503879	10.66105788
8.947623838	9.190205293	9.131560131	9.566253768	9.21905796	8.998420707
10.79564384	11.51465712	11.6137143	11.65052704	12.10951308	11.50425191
8.433042727	8.937936253	8.848914293	9.479084687	8.991410131	8.807959646
8.547862646	9.087255717	9.009407333	9.635582141	9.184000242	8.954176293
10.90324033	11.40725584	11.41071085	12.03602449	11.85301976	11.75908761
11.07493153	9.563605545	9.813557354	9.95201103	8.720627081	9.337187111
6.804494667	7.085333222	7.237499505	7.246699182	7.355380455	7.279114576
6.832363455	6.469428232	6.344827192	6.481458475	6.889002121	6.787317758
7.078303869	7.791244131	7.766006091	8.853585596	8.438325768	7.977952253
9.691258919	8.617840212	7.754407434	8.690918616	7.890057202	8.298004222
9.127872697	8.446641939	8.466026899	8.722452253	8.277405556	8.39308204
9.09824599	9.670530667	9.578067192	8.494130061	9.561667293	9.534194535
7.690510313	7.591751808	7.995673758	8.458697343	8.443547162	8.426330949
9.090721626	6.894391313	7.406158394	7.239398515	7.044506424	7.13441502
7.66151798	8.404396899	8.179951646	6.941709929	8.162329636	8.250622253

7.885378	6.714000354	6.94407103	6.841391899	6.617114677	6.707139758
6.916453859	6.82018896	6.653596384	6.279712293	7.229587778	6.405058101
15.10875812	16.2036826	15.86132141	15.86132141	16.28343863	16.22759729
7.748187364	6.254539838	7.095534354	6.498363273	6.439789939	6.648873556
7.93027604	6.87010599	6.335255596	6.554351	7.036210545	7.815211869
9.089024899	8.658802051	8.776574232	9.217871889	9.080123545	8.85049999
7.579628838	7.203245626	6.79323798	6.975188859	6.428147687	6.920203848
11.43005301	12.52130815	12.52819783	11.87445957	11.70138789	11.6295292
8.256499515	7.453021566	7.611680859	7.398020586	6.999830061	7.242384899
9.198436465	9.439397061	9.373397364	9.099242404	9.650789909	9.244930505
10.06356315	9.789131121	9.905108616	9.515069697	9.254253192	9.776037091
8.755421576	9.862946677	9.267923343	10.31013098	8.874191697	9.645545465
10.47941256	10.85654613	10.88546605	10.56908161	10.40387449	10.60635433
7.882189879	8.134366646	7.937009	8.615330515	8.238629646	7.811371919
8.155023263	8.240375404	7.455669899	9.170513566	8.230181798	7.481322818
7.14692997	7.257525758	7.07011003	7.254341798	6.989715687	7.233950838
15.86132141	15.88528111	16.57174008	16.22759729	16.22759729	16.39933956
9.031713778	9.514496606	9.046862848	9.451212687	9.308265131	9.135096212
6.657192121	8.009440313	7.543396525	6.91172998	7.868335495	7.665645141
8.886096818	8.977005354	9.323731354	8.067132556	8.851018515	9.353057535
6.704918465	6.931296273	6.366338828	6.589168273	7.370950798	6.889002121
8.802385697	8.280707687	7.972411364	9.215289101	8.020096283	8.507703202
7.69973097	7.826151263	7.782796596	6.827661505	7.910617626	7.771143222
13.29684189	14.30038792	14.23242051	14.15092087	14.31917195	13.66784462
7.158304313	7.284105152	7.383711768	6.298407384	7.315524667	6.921167192
9.586238192	10.05236285	10.05523038	10.0053333	10.05880999	10.23452156
12.21312889	12.18142186	12.20960187	11.71042621	11.96947069	11.7349036
9.926784556	10.42297323	10.14895288	10.65634987	9.800083455	10.25422487
7.775269606	7.684641212	7.51061902	7.104662848	7.33379003	9.151976485
9.200042253	8.383846919	8.579336081	8.293340606	7.858612495	7.570765313
9.922126404	9.602761333	9.930141081	10.36903954	9.715870253	9.789981192
8.110881556	8.704708384	8.683087172	8.77298297	9.042346232	8.814101707
7.394370293	7.215770273	7.166369152	7.197080263	7.480656747	7.222444626
16.4534382	15.18392271	15.58150095	15.12351424	15.78787594	15.90458538
10.80499832	9.776839404	9.815893677	9.161011869	9.458377051	9.616741535
9.224452333	9.399129424	9.299916465	7.946933485	9.231349182	9.014335657
9.345614434	9.037153444	9.254890202	9.719000626	9.039454747	9.504584061
10.95234717	11.59212007	11.81018832	11.74205097	11.63629658	11.77552743
9.50650501	9.224452333	9.384078242	8.640789455	9.016491202	9.096947232
12.0724233	12.31719428	12.22629088	12.11841559	12.01191659	12.08128174
12.43600068	13.16151113	13.80033164	13.79381649	13.46005067	13.34204398
8.355387535	8.619848374	8.409548323	7.536554758	8.561789232	8.354947869
6.976233182	5.987495828	7.33350399	5.944961081	7.687096788	7.893940788
6.702734242	6.409677646	6.360244697	6.634122485	6.598211152	6.48058603
6.853461566	7.337670899	7.587331949	6.700323747	7.664275131	7.187741232
7.418946535	6.714232707	7.073903182	6.529097838	6.70156496	6.483554929
11.44319437	9.374690616	9.725631121	10.40074116	9.470840162	10.24414187
9.36748	8.743124222	8.955880859	10.03299879	8.733447455	9.424985495
7.236914141	7.655611	7.444823313	8.541972566	7.970926879	8.177528808
7.182269455	6.772767788	6.489773778	6.161766525	7.269598859	7.448630646
14.52085241	11.99535117	12.16942168	11.1453264	11.86125412	12.33576886
6.954196414	7.700670768	7.12541897	7.306206455	7.633233848	7.214922364
11.12151875	11.35657003	11.31267384	11.47496104	11.4413808	11.06433979
9.769223525	9.442729677	9.083741606	8.631321253	9.16271003	9.053043889
8.578393657	8.800397414	9.017695354	9.977292899	9.169862626	9.528176495
10.62280049	9.454390232	10.2829717	11.08107282	9.90675497	10.67081044
7.276635293	6.466965869	6.367075323	6.954196414	6.857502071	6.559271747
8.042974687	8.065153556	7.268488485	7.578291899	7.742830394	7.963030121

8.783158111	8.856895172	8.93638097	9.445197818	9.074692404	9.021393263
11.46320141	11.9875501	11.90384839	11.97660652	11.72592113	11.76804793
13.37822002	12.37087917	13.03025597	13.6823979	12.66167346	13.69473258
7.701686677	8.486968232	8.275152535	7.223533071	8.352134333	7.709201212
7.284431525	7.867668606	8.025051697	8.31214998	8.121211444	7.906781182
6.779669273	7.13105099	7.537489808	7.112620848	7.171072313	7.140009717
10.48074239	10.30058205	10.53737043	10.72246175	10.46442497	10.6488688
12.57486869	12.60396528	13.18858184	13.56886427	13.07947925	13.17281418
7.845981061	7.535866141	7.720869455	7.432070859	6.916453859	6.95496698
7.585815263	7.277849899	7.427701768	8.367422212	7.568036061	7.834787162
8.25927404	8.404822414	8.469339576	7.472789818	8.452374444	8.609511
10.93935201	10.13479405	10.96460614	12.39973853	10.6820228	10.91514001
7.19681397	6.269735929	6.453428828	6.056037747	6.311636323	6.184860172
13.13007894	9.279768394	10.3896003	10.24828231	9.828404283	10.33989796
10.31693391	10.03026493	10.20060626	10.89224764	10.65987528	10.53737043
7.787525596	9.136296333	8.687541707	8.421350475	8.890373556	8.112525414
6.702978071	6.910146768	6.912275354	7.497675424	7.415013667	7.034867141
14.03424604	14.53168901	14.5108222	14.33555643	14.50017253	14.09521817
9.962571606	10.07670007	10.06997663	10.34830463	10.61992902	10.75178718
7.587956192	7.629673333	7.512765949	6.797074616	7.633560879	7.575420636
7.825440475	7.788532485	7.796747616	8.783158111	7.988251818	8.269395232
11.17571904	11.73972102	11.72592113	11.98999321	11.69891546	11.73070023
10.12224713	9.448606737	9.268661657	9.348735909	9.290785283	9.600888859
10.15585749	10.55726192	10.72900488	10.5104744	10.56802411	10.34514895
12.05489479	11.6137143	11.80342824	12.26162026	10.00002479	10.16262583
12.16942168	11.60680648	12.14507673	12.81379045	12.28105924	12.44611991
11.44491584	11.58421758	11.87687449	12.03310273	12.68484185	12.01427079
8.086953081	7.646953505	7.854325061	8.278157657	8.067838768	8.115821848
10.26224218	9.86223396	10.62280049	10.68072859	10.52585606	11.1031408
5.774380697	5.786544101	5.781964232	5.819486	5.793187949	9.102676354
10.48185706	10.68338253	11.39594259	10.31997281	10.56306707	10.01703805
9.982665242	10.23660558	10.00695165	10.63516273	10.55474917	10.27432009
8.742635939	9.331038253	9.247858869	9.619644949	9.345614434	9.266188263
7.800624061	7.840025778	7.900715717	8.767368071	8.295436051	8.419866586
8.642670768	8.366003535	7.846992646	7.999737535	8.260123323	8.543686687
6.622230818	7.154318051	7.262654505	7.235098212	6.499057505	6.781710556
7.599486586	7.968691808	7.968277051	7.899333596	7.975072535	7.957321232
9.80784303	8.904793061	7.598252121	8.559975162	7.807908545	8.308776677
7.156966303	7.209785606	7.411268949	8.210186616	7.095534354	7.304975899
11.90152869	12.88708268	12.75396286	10.8778129	12.5521346	12.48124823
5.756527813	5.797477455	5.917584465	5.759653798	5.867143949	5.763545343
7.516462556	7.66151798	7.859670455	8.128290707	7.953486636	8.193820172
12.68097339	12.97555423	12.86086109	13.10484893	13.16151113	12.83405786
8.466026899	7.840343939	7.886820596	7.744459273	6.982654747	7.240898899
10.27804811	10.31095185	10.35591393	10.37124354	9.122138505	9.602761333
9.446464909	9.723272263	9.739859192	10.38531322	9.95201103	10.08799683
10.1421691	10.36239309	10.44602022	10.6362571	11.28053313	11.70138789
8.043818737	7.813018717	7.659186848	7.197446212	7.042119313	7.352900667
10.75737971	9.957374697	10.13375911	9.766921414	9.556759333	10.14895288
8.848410616	8.549970081	8.448929687	9.11093099	8.930963202	9.033915556
7.180839586	6.258382485	6.551865586	6.341087596	5.911450071	6.387054293
11.05356995	11.46539851	11.42371373	11.36065784	11.58814301	11.44643795
10.96311364	10.61867663	10.60044575	11.3403052	11.11230451	10.91766747
10.00262223	9.471411141	9.667708556	10.98492733	9.610538374	9.837575172
7.621022111	8.295436051	8.254082232	8.237787788	8.23581501	7.910617626
7.892936636	7.666604434	7.634916323	8.443956768	8.079973212	8.082778869
9.742174424	8.188779434	8.507703202	8.545617303	7.466913717	8.22479296
7.789881242	8.132293172	8.422643323	7.882525919	8.198946162	7.999327475

8.189895404	8.453185455	8.480536717	9.050797061	9.005433152	8.678658293
15.54332231	13.47832004	13.6823979	14.13521971	13.96779242	14.15092087
8.276014121	8.328154263	8.222994222	8.051625212	8.614442374	8.421742051
7.107120283	6.969919475	6.965182525	6.842028788	6.415875869	6.671065323
11.66189432	11.69891546	11.85301976	12.46466592	12.06397065	12.06397065
8.274698687	8.058592364	8.086566798	8.269818727	8.493155899	8.382609505
10.21769501	8.581169111	9.549314313	9.390834152	8.389243343	8.863375313
9.35796903	8.558556152	8.675348646	8.565878434	8.631321253	8.575566677
7.991889949	6.677794545	6.992079758	8.420387707	7.348618394	7.38340502
6.619037364	6.021063475	5.780440091	5.928940303	6.02159498	6.352246303
9.824540899	7.953486636	8.10683704	8.049757475	7.039966242	7.439506505
7.202701576	8.011239071	8.247731232	8.198176101	8.368326869	7.466913717
13.74759592	14.60195877	14.24162707	14.45281427	14.78379092	14.52085241
8.27018399	8.171342424	7.976858394	8.233838889	8.903602263	8.204737434
7.859293	7.893940788	8.33796001	8.038354192	8.356302828	8.023866626
11.07158908	11.36065784	11.11388317	11.14707085	11.1276132	10.73661507
7.024771818	6.579844919	6.518333111	6.445241798	6.547766404	6.832125364
7.533990333	8.086153313	8.299695162	8.68840998	7.838173152	8.035288182
9.064430111	8.429130081	8.336257222	9.660861778	8.69335002	8.62412801
12.04990566	8.102484232	8.354570556	7.799196616	7.661901192	8.109636222
14.74028573	13.00581854	13.44126502	13.50411017	12.86955734	13.52959744
9.122783717	8.843670869	8.419470808	8.752460404	8.230996848	8.27903296
14.27397716	11.36245739	11.86922315	11.39772243	11.92937262	12.16036216
11.76588718	11.17750487	11.09089144	10.91176188	10.78551178	11.46936418
6.884322333	7.061330929	6.82018896	6.024205414	6.660088081	7.019697232
6.949065	6.518112475	7.29540803	6.839237455	6.342888505	6.350237535
7.797844434	8.399975414	8.046784596	7.300950758	8.104866828	7.894965162
10.16546821	9.731806091	10.042425	8.886620687	9.646274414	10.09254304
5.820272051	5.96112699	6.611130667	6.10377004	5.875358434	5.869840515
7.494510091	7.85299496	7.410944101	7.772177253	7.473428394	7.357456071
11.35657003	10.32309273	10.40592026	10.30058205	10.45150222	10.62142174
6.771840333	6.670140313	6.843046434	7.005900293	7.193571	7.152958919
7.458509535	6.672562737	7.479119	7.162148556	6.64187404	6.713538929
6.83774396	7.991889949	8.271029889	7.286424495	7.992269263	7.200755828
9.032817303	9.513164303	9.390110384	9.701165495	9.468134182	9.476588061
9.343457394	10.21375666	10.41731261	8.851018515	10.09161001	9.581418808
8.996148737	8.722953263	8.724556485	8.285708747	8.307483899	8.914132333
12.92081703	11.59654933	10.687913	10.8443678	10.94786936	11.50632759
8.345781111	8.994589434	9.196132455	7.780116303	8.887684293	8.977558859
11.04886125	10.27139209	10.28089537	10.16073454	9.052474394	9.174793152
6.688924818	6.686787465	8.083135202	7.032021525	6.704194818	6.741723879
9.724047525	8.959130566	9.152615889	10.07857135	9.312041414	9.505859384
7.066192545	6.271385515	7.340448859	7.471219899	7.534556657	7.257820879
9.974493182	9.870221061	10.3305428	10.33375706	9.586238192	9.965319232
10.05688349	8.793339475	9.236429667	9.099925818	8.858692343	9.385290182
16.28343863	15.27762024	15.46469304	14.48025691	15.50564301	15.46469304
9.679062616	8.840010495	9.546645747	9.971733293	9.526870273	9.169326323
12.54906863	8.942756889	10.18198959	9.609027141	9.326739394	9.985290333
7.644277273	7.546592828	7.532708636	8.183195545	7.836828747	8.100639
11.50814936	10.82153564	10.84302834	10.52585606	10.85782556	10.80782516
7.175214949	7.152088687	7.246435697	7.600942677	7.682588111	7.356542485
7.508388697	7.353560545	7.496050374	7.658441616	7.748914677	7.552094283
5.953231333	6.471881182	6.227696606	6.262348667	6.439547263	6.258005697
11.47322198	11.64637724	11.51465712	10.42409385	11.17750487	10.92880859
9.217871889	8.490531303	8.542429747	8.528049141	7.609574333	8.052783737
9.083741606	7.833291081	7.777311636	7.545628697	7.738963899	7.733228788
10.72127155	9.682548525	9.651465606	8.385303111	9.393934131	9.828404283
8.947127939	8.992473788	9.09013302	8.64507902	7.369510384	7.796432081

10.27236099	10.92485902	11.17213305	10.45628541	10.98350754	10.71992703
8.757405333	7.548584576	7.591410606	7.664957788	7.20183702	7.247605232
9.414554808	8.967895455	9.755162434	8.888766747	8.573727141	8.733447455
6.801276	7.158043384	7.069787606	6.073896293	6.962034242	6.968393455
10.94640834	10.00094823	10.08893073	10.45877561	10.02644328	9.627745303
7.424653535	7.574795778	8.166172404	8.019434586	8.811517808	7.618249414
6.516723778	6.960463354	6.47300398	6.204908667	6.716586707	6.953378071
6.071423232	6.427492121	6.231060354	6.240475939	5.889752596	6.369666687
10.65751844	10.39968902	10.62647628	11.22947614	10.74558579	10.96460614
7.977207111	8.232163879	8.556855828	8.82024202	8.349393212	8.165787747
9.145078374	8.703161646	8.827377505	9.574750414	7.541750556	7.657256192
6.804242869	7.165538758	7.035678848	7.447389475	7.325469747	7.161889071
9.420326576	7.877487515	7.920494253	7.401155333	7.681167667	8.039068889
8.776574232	7.756512818	8.017912586	10.10631712	8.246470545	8.64224801
11.84529477	11.97196754	12.05489479	11.96362869	12.04432	11.65052704
8.177146606	7.563439404	7.988882495	8.373157505	8.583996586	8.621689657
11.45526933	8.340252101	8.493155899	7.469634172	8.284876758	8.426780919
9.781348374	10.03876748	9.689129545	9.728754818	10.02549055	10.0672481
8.145617202	7.304975899	7.438614949	7.512765949	6.983407384	7.264201869
9.804809051	10.23351878	9.904237354	9.832711535	10.25220538	9.804037697
7.974325596	7.486746556	7.426803586	7.894965162	7.620680879	7.228759818
6.393637899	7.097212141	6.917518081	6.911201424	7.119081899	7.406826051
10.61743608	9.995698596	9.992988556	10.01340346	10.02923217	9.613873859
8.454954939	8.925792808	8.915814212	9.236429667	8.997807768	9.226702434
11.90674449	12.85589012	12.24097391	12.0838996	13.0147344	12.32964397
8.808928141	9.145078374	9.013314737	9.633493677	9.588410576	9.058895657
7.170007293	7.655304091	7.584620515	8.523456192	7.796432081	7.861832121
8.933180404	8.86638896	8.814624313	9.536330707	8.944409727	9.123926859
9.341222778	9.642500081	9.842388081	9.894213939	10.12041459	10.0188097
7.267645253	6.938411576	7.185712515	7.440186081	6.491978192	6.590229374
7.550845909	7.23452504	7.436027273	7.180559343	7.496050374	7.212654808
7.562928222	7.978709162	7.359287172	7.419275485	8.030614293	7.036743576
8.639417101	8.459227657	8.769824364	9.266794162	8.749346626	8.700142556
12.24097391	12.20640951	12.23191382	13.27418186	12.57877126	12.29886409
10.94071069	9.281923404	9.630502939	9.613873859	9.107388182	9.538263677
7.773938747	7.465036929	8.171788646	7.325732596	7.30728296	7.360451687
13.58209631	12.91196246	13.04869312	13.39659876	13.24400831	13.1944969
11.94979793	12.09804166	12.0089388	11.80342824	11.99535117	11.77802923
11.00754401	11.02700786	11.24323027	10.91514001	11.2278827	10.6820228
6.867884374	7.829325051	7.304125475	7.940978939	6.537080394	6.689141323
8.887684293	7.487647384	7.472789818	7.695611414	7.251769333	7.685352364
7.022558657	8.204737434	8.295436051	8.44047504	7.965660091	7.854685616
9.555296242	7.960941889	8.046433182	7.97471396	7.761767192	8.054318061
5.96112699	6.053149253	5.772359657	5.785043273	6.115805333	5.77304901
10.24612484	10.43332542	10.29208493	10.59062542	10.54190997	9.960013465
7.322217273	7.884314808	7.02909003	7.520648323	7.08136002	7.855731
16.17943014	14.16629104	14.39736901	14.61202705	14.53168901	14.89443037
9.126080354	9.735412273	9.767642525	10.3918566	10.86963124	10.24511785
9.348136535	9.507772687	9.321890899	9.465659495	8.195856758	8.112161727
8.894948323	9.438799404	9.284395657	9.293068828	9.051375475	9.032817303
9.34289298	9.009915859	9.008956374	9.143918798	8.889922455	9.153730283
10.10454015	8.517288667	8.461315273	8.051256737	8.590695768	8.803935202
8.32939098	8.208296687	8.467263475	9.037153444	8.430534091	8.480158333
8.788135667	9.455015404	9.455711606	8.491049758	9.490338525	8.954798808
8.898709273	8.583048444	8.602859283	8.281588828	8.512201869	8.351752182
7.829325051	8.189547909	8.181574081	8.599954677	8.436995596	8.372433475
7.326634626	7.494798263	7.599211495	8.168196313	8.137069859	7.820222687
6.498851707	7.059956313	7.419275485	6.179526626	6.415480919	7.03599896

8.376704939	8.857327323	8.821324343	9.41003803	9.071813909	8.903602263
8.105207222	8.885588879	8.700606657	8.385691051	8.524356212	8.101068051
8.525321657	8.099825778	7.925642	8.440878707	7.993875677	8.108917222
8.43615796	8.127895253	7.927113394	8.945493505	8.222994222	8.911610283
9.350012182	9.407554697	9.172421828	9.815129384	9.36748	9.450568111
9.26122399	9.696433626	9.863774293	9.503271071	9.436357141	9.555296242
9.054276	9.139724848	9.155079848	7.954533778	8.003147788	8.922082808
8.345353354	8.149788848	9.16271003	9.098746404	7.52469401	7.419865051
6.242810566	9.870931818	6.669224869	6.750670859	6.728207192	6.435745475
10.80091358	9.781348374	9.993897848	10.12314196	9.59387599	9.615324495
8.699656879	8.773471869	8.989652131	7.857845061	9.408173051	8.674900657
8.565878434	8.879851283	9.057828929	9.316319051	9.361074667	9.377908
9.960976253	10.56084718	10.29949835	10.77890896	10.05320582	10.16355358
8.802954374	8.771856424	8.502509434	8.366948404	8.550414556	8.092218303
5.79388998	5.785903404	5.791635343	5.831583848	5.783650737	5.799149535
10.91641235	10.81540317	10.87235819	11.28896545	10.66342196	11.05191968
10.66229262	10.84705809	11.08936677	11.26262606	10.88809182	10.41846051
10.30569114	10.20634509	10.16355358	10.78269205	9.797856576	10.39641485
11.74439188	10.83501466	10.82153564	13.03957854	10.74305691	10.81034167
10.21054893	10.68572341	10.86447048	10.54078574	11.36979611	10.88809182
6.972885929	6.957895121	6.974373515	7.584620515	7.273826505	7.406536232
8.409959899	7.612584303	6.995294596	7.143149899	6.659100485	6.794655232
7.401750182	6.884060404	7.397472212	7.41959598	7.221345788	7.534556657
10.86963124	10.64379219	10.65987528	11.18800276	10.86691694	11.0940021
7.623693616	7.110215707	7.489812667	6.974373515	6.871904646	8.677215515
8.122747707	7.92237903	8.217270354	8.327353333	7.899333596	8.586731747
9.159196141	9.552821717	9.528804131	10.43655428	9.861430101	9.918671212
9.608350808	9.625041525	9.579413505	10.53505365	9.451212687	10.05053825
6.460794313	6.807214747	6.888392616	6.885618131	6.194435071	6.697842384
8.215875455	8.461795505	8.716387192	8.761177727	8.852092202	8.545617303
9.892629697	9.29496	9.564343333	9.455711606	8.432612263	8.63568099
11.45932673	11.94481318	12.02514953	12.73731843	12.08128174	12.11841559
11.62739451	11.91651973	12.17513939	12.54589185	12.62807191	12.33907422
6.183644212	6.406575848	6.795397778	6.208223859	6.04944802	6.063625505
8.402148465	8.53715797	8.64224801	9.273640758	8.948168879	8.872352707
7.030955384	5.861799182	6.219635727	6.566529242	6.995800606	6.677368899
8.23581501	8.256833364	8.25570701	8.768863636	8.560348606	8.534432515
6.167372141	6.471031303	6.505445727	6.872611121	7.198026636	6.651024444
7.849137545	7.837840162	7.308145939	8.613985131	8.35752198	8.189194404
8.928828384	8.903025495	9.244399414	9.556000646	8.84786598	9.411394242
8.278157657	8.486053202	8.455403657	8.882882525	8.593101434	8.578858313
11.69440164	9.07419996	9.098746404	8.514909313	8.823889374	9.35067002
12.29886409	12.57877126	12.35798465	12.67700968	12.86086109	12.50706636
9.186711646	8.498167798	9.018299374	9.596773313	9.220765354	8.838020616
6.294668192	6.860943283	6.769655182	6.521458212	6.247479727	6.792041707
7.782178323	6.391201182	7.149903152	7.258984768	6.569841374	6.84678303
7.308713444	8.027386222	7.80651396	8.064713051	8.061425949	7.963792818
8.296772869	7.850535525	8.035288182	7.619228051	7.584620515	7.850890354
9.156840283	10.49830212	10.36239309	9.551451798	9.538263677	9.939327313
8.870602515	8.943861384	8.648914657	8.940763364	8.541524636	8.271795141
7.853350485	5.846920253	7.142620404	7.326042475	6.390967051	6.592293788
8.551311374	9.76471501	9.694213162	8.220225172	9.35128199	9.108564253
9.418269535	9.698896848	9.609027141	8.772410586	9.595333525	10.01257663
8.773471869	8.082778869	8.244805758	8.061773232	7.576363505	7.728044798
8.883993253	9.580097263	9.533596364	9.333361616	9.751885293	8.914720172
13.99495772	11.72126504	11.38025327	11.9577766	11.64244603	12.29275278
7.46337499	5.826825303	5.883426525	5.935564899	5.757830722	5.764504919
7.466913717	7.136356818	7.229904545	8.212243444	7.425828556	7.509050374

8.341977727	7.617533182	7.843855828	7.826151263	7.069165141	7.52646604
14.82007331	12.95787308	13.15645152	12.52130815	12.55983323	12.8652792
8.364284556	6.801828172	6.677139455	6.780968242	6.650108172	6.993764091
7.233650202	7.318843495	7.873080636	7.812327374	8.126700889	7.928832091
12.25251148	11.88909817	12.31719428	12.9709957	12.93430142	12.25844332
7.226681909	7.692552364	7.848096879	7.523479626	7.845981061	8.094989869
9.033394545	9.159785394	9.465071586	10.10454015	9.321890899	9.421700798
12.27773509	10.49241362	11.26262606	12.30465105	10.82153564	11.28053313
7.535530424	6.275469879	7.010749455	7.178076949	6.500186222	6.732989909
8.192645727	9.269847242	8.874692596	9.306330646	9.344138121	9.614560172
8.031407364	8.290726727	8.961854929	7.646275747	7.803944101	7.732206152
8.590695768	8.832790172	8.990220404	9.430738808	9.190767364	9.133284677
8.074096707	8.457043091	8.197004869	7.977952253	8.210186616	7.995268899
12.8652792	10.65987528	10.97054498	11.00053539	10.3810842	10.71860391
6.391874798	5.986652889	6.046729758	5.939868061	6.057743394	6.129955152
8.887109626	7.549557061	7.689572788	7.606370131	7.018881949	7.23119303
14.77222727	12.03602449	11.65698449	8.916479111	10.86305205	11.38577951
7.18482696	6.634356909	7.40806004	6.377717758	6.50518599	6.994029394
11.9577766	11.22456459	11.29597305	10.77387872	11.03452834	11.1502402
6.813409273	7.249695535	7.424346313	7.473129747	7.730154939	7.09298796
7.535866141	7.243253313	6.95944596	6.735607747	7.274095485	7.220240556
9.702758343	8.778623323	9.026636737	8.878776172	8.472435657	8.890373556
7.290498626	7.400314747	7.29228501	7.21064097	6.651024444	6.872396859
8.187968455	7.682204525	7.996393263	8.549970081	8.425847798	8.046784596
7.258984768	7.601643838	7.584276586	7.956615091	6.466965869	6.893883192
7.821971636	8.004655889	7.997183333	7.04319302	7.735250384	8.246087697
10.23160947	8.80133401	9.512482222	8.914132333	8.333227545	8.677707162
7.745466071	7.740469253	7.534302667	7.073141899	8.023099172	7.631600293
9.122138505	8.653441707	8.537544354	8.088117667	9.147296667	8.559975162
7.991473828	8.344860929	8.127895253	8.191204879	8.055171202	8.061773232
10.34830463	10.26729954	9.366312899	9.196132455	8.964085616	9.060618192
10.79145221	10.40592026	10.56421743	10.88945836	10.64738653	10.90192126
7.554702939	6.835371293	6.484023485	6.515215919	6.255971394	6.304082879
7.12322297	7.272331859	7.106035242	6.644710828	7.589252838	6.858224505
8.846355515	9.847335909	9.943097475	9.657223606	9.511857455	9.525471414
10.59292728	7.646666444	7.829325051	9.521192909	8.979921919	7.710917071
9.457741556	9.606954152	9.237664879	8.831800202	8.926362727	9.041820404
11.28370047	10.01340346	11.1453264	10.75062885	10.74045328	10.156858
10.09506496	10.83930015	10.41846051	10.64623997	11.09250749	10.26445444
12.09497108	12.43600068	12.90359001	12.02514953	12.3774088	11.94481318
12.51766186	12.3543152	12.35168631	12.82558839	12.84627851	12.61449239
7.323648515	6.577039475	5.960790525	6.826879909	5.755808879	6.054582838
10.38631174	10.68072859	10.39747702	9.220765354	10.59062542	10.33375706
9.857940545	10.23550694	10.22557955	9.522660222	10.71992703	10.47479655
8.70424704	8.929972798	9.221852535	9.269847242	9.606298465	8.769824364
7.958393384	8.438712657	8.147481596	7.839249838	8.289996192	8.397853727
9.526200354	10.58689262	10.67428006	10.34100163	10.53737043	10.26116661
9.717365758	9.771430818	9.580097263	9.386484303	9.74383503	9.303342798
8.193428737	8.523871909	8.757405333	8.823394192	9.223302313	8.767872848
9.633493677	9.881176566	9.500664495	8.909945455	10.04682541	9.276789111
8.939019091	10.4655823	9.982665242	9.462343535	9.573320202	10.33252085
11.85889751	11.0035084	11.04464523	10.57282784	10.90754603	10.96000427
13.78027417	14.08841453	14.02572128	13.81935116	14.40789433	13.94443
10.66342196	10.65634987	10.81280665	10.81664077	10.98186414	10.63014735
8.800397414	8.580231323	8.35796399	8.833963152	8.406604404	8.944409727
11.11714857	9.79615097	10.24139873	10.07388225	10.3380268	9.777520899
8.174069929	7.104098253	7.106848111	7.149049263	7.33182303	7.507037566
11.05959848	11.49029795	10.91356479	11.64044206	11.39382595	10.84974643

8.354182626	7.073141899	7.108229697	7.047335818	6.697417434	7.040523253
6.913788323	6.356712303	6.410700434	6.400570727	5.881579646	6.08802603
7.300330596	7.315791616	7.236914141	7.546592828	7.163006	6.960198707
7.309668525	6.258944949	7.079662576	7.509050374	6.896711899	7.069787606
8.588971687	8.594861495	8.752912545	8.930433505	9.180595944	8.957944414
7.833640626	8.321509909	8.491898242	8.302900242	8.57070398	8.355847778
7.44801498	7.811371919	7.667962596	6.900245465	7.446760616	7.342557798
8.597538556	8.08737399	8.650384111	9.149195859	8.817758687	9.109120374
7.960192616	7.587635919	8.436995596	8.336651323	7.893940788	7.931708101
7.427701768	7.452109758	7.194186434	6.575249545	7.363617495	7.463067838
9.288419414	9.316888828	9.45706402	10.04421951	9.311440606	9.658772677
8.316570081	8.091725323	7.757833798	7.44169298	7.649926505	8.105629576
8.700606657	8.869521111	8.926362727	9.31327601	8.846850111	8.973478152
7.002156515	6.102200535	6.307709909	6.339262212	6.00930701	6.37731698
8.550414556	8.741655828	8.492322828	9.384699788	9.264483889	8.924680667
8.631321253	7.348317141	7.54731098	7.247605232	7.465036929	7.806845253
9.168828475	9.333986848	9.582110717	8.715845505	8.853585596	9.239472869
8.374363061	9.205404404	9.052474394	8.477505576	8.745043505	9.007167758
5.75937802	5.980732253	5.883690545	6.173623303	6.039785808	5.786272646
8.425403333	9.480384485	9.541747455	8.956407626	9.417037424	9.549314313
8.597027768	8.567801737	8.634342798	9.008956374	8.707515232	8.956407626
6.020401747	6.727438899	6.859929394	7.048974677	6.324159687	6.883793818
7.846324646	7.680542242	8.634788929	8.416773909	8.086953081	8.680513909
7.155245384	7.224435778	9.078885242	7.066488111	6.94407103	6.834332747
7.232151747	8.121954505	8.610337293	8.100639	7.983913121	7.967920667
7.870669919	8.018723424	7.445432899	8.096128192	7.973586899	7.954939636
8.541524636	8.920488949	9.142159707	7.909763434	8.789683394	9.04056096
10.75865445	9.54312803	11.565007	10.13276962	9.378583414	9.881912677
6.762066263	7.216096788	7.219933525	6.25331604	5.993549475	6.443240697
8.184399818	8.400464071	8.041000495	9.013830768	8.911610283	8.541125101
9.474605101	9.773700283	9.650129091	10.2572153	9.870931818	9.835081424
6.286107737	6.543222263	6.717503818	6.193905333	6.898538505	6.937881404
9.785178838	7.987573434	8.163923192	7.479422939	7.757833798	8.297630758
7.845601525	6.437041	8.248137606	7.98285402	8.613130333	6.966460535
6.798855303	6.59250899	6.955200051	6.762066263	6.262968212	6.602666131
9.662314131	9.582110717	9.514496606	10.5336638	9.774477667	10.02741959
9.31327601	9.196689051	9.041236566	8.943348051	8.908475283	9.497817424
8.398231626	8.608532485	8.555833323	8.795325869	9.014335657	8.686679798
11.15342364	11.911688	12.02240324	10.86305205	11.54675633	11.75662781
7.92597801	8.594455404	8.238629646	7.706567859	8.441767061	8.130283051
9.226128374	8.469779808	8.554971	8.091323071	7.766357737	8.086153313
6.898792364	7.192117626	7.360165061	6.581970424	6.797074616	6.738778758
7.113878571	6.76309904	6.589168273	6.581763828	6.322925	6.540677515
6.576569869	6.33399804	6.010087253	7.617189485	6.114423687	6.29940503
11.6137143	10.25519474	10.19418334	10.00450786	9.834210131	10.17440526
9.895856061	7.527697455	7.387388131	8.36556403	10.91176188	8.503455465
8.33796001	6.492854313	6.336798838	6.822217707	9.494490616	6.899234475
10.3918566	10.21054893	9.46304199	10.29949835	10.48185706	11.03737422
11.72126504	12.48835313	12.04432	10.08145981	11.65918316	12.19398134
11.70348036	12.18492446	12.11216258	10.18098661	11.4535969	11.83813795
8.832790172	9.413321525	9.18155302	8.298896434	9.667708556	9.009915859
12.9709957	12.94367069	12.85048632	13.5964752	13.29117716	13.13007894
7.496050374	7.590248525	7.627997354	8.179581869	8.381754576	8.021628354
7.149903152	6.97366599	7.154661646	7.032569455	7.124596343	7.046480485
7.913459869	8.117339677	8.236633323	8.125818909	7.728044798	7.603719626
7.091328697	7.632536929	7.38400901	7.404051424	7.795429838	7.15072903
8.951513343	9.292435667	9.473962505	9.288419414	9.50650501	9.273640758
8.740682	9.323731354	8.89604703	9.380979222	9.885156737	9.151431899

9.368224626	7.746135697	7.88753598	7.721209061	7.418614091	7.653990838
8.268052495	8.301657889	8.444861909	8.529888505	8.694840596	8.291610121
8.116928465	7.825767899	8.758364879	8.572163455	8.285708747	8.011239071
9.716648152	9.659537192	8.78664797	9.077686808	9.583475303	10.1885747
6.996641657	6.529524889	6.455606303	7.453956636	6.697172354	6.712342556
12.48124823	12.41033676	12.81735602	13.25686564	12.24351077	12.71142619
10.45877561	11.03452834	10.75062885	10.86173253	10.90192126	10.61480751
11.14866374	11.13713447	11.21016953	10.10841911	11.19817469	10.62647628
6.414872495	7.472789818	7.608934747	6.15200799	6.158655535	6.856746788
6.782883697	7.803944101	6.705893697	7.180291929	7.299385535	7.326042475
9.846508495	9.980047091	10.24414187	10.2761618	10.74172297	9.869466131
6.064326273	6.104594101	7.346859747	6.558602434	6.598910859	6.124346828
9.635582141	9.649399939	8.825312061	10.22448985	9.599465606	9.443424081
7.369172101	7.258429848	7.471589222	7.997341081	7.237206848	7.445432899
7.196550485	7.33379003	7.218159515	7.457359414	7.788923101	7.220481808
13.2504616	12.7452022	12.38063147	12.90359001	12.77841715	13.24400831
6.410521475	6.816006121	6.303681202	6.579612929	6.446595263	6.404620899
8.197805768	7.567065616	7.400015081	8.131862263	7.957660747	7.600615697
10.32309273	10.04600105	9.147897788	9.45706402	9.567720788	9.753529758
10.36032821	10.04682541	9.853774707	10.37686258	10.11150219	10.11452638
7.672658859	7.928481586	7.929905606	8.095330333	7.256041222	7.789881242
12.21630458	11.05493438	11.01209098	11.6137143	10.67190595	11.48258282
6.074946343	5.977265909	6.089542354	5.972994556	5.965348202	6.350873162
8.77085696	8.112161727	8.292509485	9.785178838	8.513135323	8.925792808
11.46724679	11.46145043	11.44643795	11.2518391	11.52448673	11.58814301
6.785592192	6.213570111	7.104930788	6.480989929	6.647920455	6.103600394
9.053706202	7.85710597	8.557697889	7.514105485	7.753046657	7.655304091
9.037616626	9.133284677	9.632768747	9.242021586	9.353057535	9.484840202
8.412578899	8.295839707	8.609952768	8.097222414	8.409959899	9.243096889
8.872352707	9.516398909	9.884409465	8.952561111	10.01974654	9.575401111
9.356741384	10.11150219	9.868690293	9.299916465	9.655088667	9.706432808
9.874496818	9.93663897	9.821195081	10.35699559	9.465071586	10.38208186
10.24227579	10.75062885	10.13958591	11.07956266	10.1752482	10.48897537
14.91973608	12.58287167	12.78645268	12.14191765	12.89886333	12.86086109
7.916391202	8.269395232	8.205133263	8.45451298	8.85049999	8.822318
8.245649515	8.188379657	8.093860848	7.898974697	7.478449101	7.955335374
13.66784462	14.45281427	14.11151028	13.44126502	13.54965001	14.28325373
7.912365798	8.436995596	8.313601758	8.346989566	8.723452192	8.25927404
11.5571076	10.59180142	10.80499832	10.62764398	10.14326599	10.83223099
9.636929838	9.782133848	9.684048687	8.899757485	10.2572153	9.189590182
8.023506091	7.609879768	7.879296202	8.363468485	8.102484232	8.574674323
7.489478697	6.949335212	6.699888758	7.932792414	7.146637616	7.355080212
6.527189616	6.579612929	6.101540747	6.505895828	6.494178707	6.460273586
9.106199707	9.529469889	9.280305798	8.596127869	9.724047525	9.305189859
15.59877501	13.67554111	13.82538175	14.04335209	14.01682491	14.48025691
7.545628697	7.673764162	7.200432616	7.298118263	7.317304515	7.496345141
9.460382818	10.05523038	9.721958051	8.753927596	9.76471501	9.819688798
10.63266161	11.05651949	10.75737971	9.254253192	10.10946026	10.45996909
7.686071172	7.756889051	7.284692949	8.236633323	7.351279444	7.762120596
7.902229798	7.667649192	7.867312374	7.880750535	7.486457414	7.473746677
10.56548536	11.21187068	10.11941657	9.579413505	10.54800525	10.49713487
8.981975535	9.710962273	8.35372597	7.626470505	8.35372597	8.27861104
7.861832121	8.354570556	8.618302869	9.018802576	8.935320828	8.659649677
8.77298297	9.756054152	9.480384485	8.589388657	9.182731869	8.905887879
8.120795899	7.878949444	7.637262101	8.614442374	7.752360667	8.194999071
12.84197228	13.61616406	13.05987966	12.26475658	13.45466248	12.73352188
10.29324401	10.48782122	10.36903954	10.05144087	10.42736719	10.1421691
9.923658606	9.977292899	10.63870636	8.77962597	9.504584061	9.638309758

9.097596465	9.142159707	9.257979848	10.08325108	9.038849273	9.417594202
8.457898556	8.192996515	8.53715797	8.934248131	6.677139455	7.610574101
10.25627793	9.783680727	9.710054737	9.869466131	9.390110384	9.257324
10.04862183	10.16743096	10.01340346	11.04886125	10.36239309	10.5752557
8.307093606	7.362680232	7.291703707	7.671246505	7.394691081	7.787223354
12.82558839	12.67700968	12.89113428	11.72355631	11.94745099	13.27418186
9.497817424	10.25422487	10.11555853	8.639872707	10.23752804	9.910074283
8.497259596	7.708254091	7.881102323	7.988882495	7.431067202	7.500795283
9.291393586	10.36349344	10.20964764	9.291911616	10.28089537	10.05607537
9.710054737	10.25320711	10.26832521	8.961327374	9.974493182	9.727055768
17.29704085	16.61084374	16.36806566	15.70163214	16.3077943	16.25437967
9.409395929	9.967178364	9.742174424	8.902484929	10.11849919	9.524156586
8.458697343	7.611330343	7.572084505	7.651593505	7.006478434	7.143440162
8.157153081	8.623636919	8.272231939	8.848410616	7.315791616	7.975784071
7.315524667	7.289911515	7.341038	7.886079293	7.815532475	7.568622485
7.564741838	7.993428586	7.622357354	8.083135202	7.492937667	7.488894061
9.638309758	7.959080606	8.172571899	7.701352778	8.112525414	8.068564879
10.50794857	9.624391323	9.449254061	8.769824364	9.096947232	9.415860727
8.858269384	9.193817232	9.140392293	8.197805768	9.136890505	8.881793333
8.109266465	8.152443121	8.187593091	7.870669919	8.390114414	8.304169929
9.515743253	7.661155364	7.660795616	7.750590293	7.207500556	7.612584303
7.129307667	6.953109798	6.890245747	7.594027929	7.158304313	7.299714313
9.034403333	9.565660949	9.469429253	9.011546121	9.481668929	9.36570098
9.196689051	8.609511	8.989142232	8.400464071	8.554528737	8.848914293
9.624391323	9.782868192	9.953782444	9.427546879	9.961780586	9.839882283
10.40810668	10.17613521	9.965319232	10.3305428	11.54871614	10.76361324
14.07988992	14.5108222	14.4185289	15.36066179	14.52085241	14.1904887
8.523456192	8.123471404	8.016751283	7.732206152	7.21346404	7.312252182
8.242431778	7.87816304	8.205457293	8.250194202	8.251492909	7.868007081
8.496814303	6.507609283	7.933573889	7.300330596	6.999250869	7.464044657
8.663303283	8.44129198	8.37004801	7.937868929	8.152443121	8.551311374
7.645645939	7.65951298	7.911995121	7.099882485	7.372842667	7.342268626
10.89912578	11.64244603	11.69891546	11.71244686	10.79564384	10.8605042
6.629250202	5.994507889	5.865328414	5.977376444	6.282201616	6.067709717
8.033283465	6.50518599	6.689141323	6.622230818	7.298462212	6.491278737
12.33907422	11.76336308	12.06098883	10.69574272	12.35168631	11.97196754
13.31405593	13.47327752	13.3718573	13.70143341	13.92894576	13.01016522
12.87421343	13.95167504	13.58996905	12.55983323	13.78027417	13.22730043
8.856343687	9.436898869	8.995099212	9.07526898	9.201355202	8.456593071
8.883414061	8.708995576	8.656360697	9.396536798	8.701109788	8.951513343
10.41846051	10.18098661	10.29101087	10.56655729	9.331038253	9.329342374
8.239938859	7.138827606	7.016713091	7.533663232	7.821271606	7.089652374
8.897751606	9.235747242	9.122783717	9.029402949	9.229082869	8.990220404
11.51260306	12.24351077	12.12692782	12.36759935	12.35798465	12.10135206
9.18155302	7.484854374	7.633560879	7.482605354	7.316095192	7.562928222
6.599129636	5.784529535	6.929929141	7.278115626	6.292304869	7.165538758
8.903025495	9.285000091	9.223302313	9.352487919	9.572453101	9.036076414
7.317590788	7.280239242	7.439506505	7.679634566	8.267698071	7.445127778
6.252603323	7.90403798	7.692552364	6.324531404	7.736619182	7.367541707
14.07245549	13.38447499	14.9847254	14.99939481	14.04335209	13.29117716
8.397394545	9.426842081	9.50650501	9.93663897	9.543825273	9.699589758
6.95496698	6.388351374	6.224237061	6.535299384	7.018424152	6.978374222
9.280305798	10.06997663	9.849671929	8.759332636	9.796940758	9.449254061
8.69335002	9.582761798	9.625623061	9.627013505	9.792275424	9.394567081
13.43523914	14.17411554	14.19920898	13.48990673	14.14208591	13.74093847
6.94985404	7.020691646	7.250899535	7.538207606	7.106302455	7.431067202
8.568768141	8.703733141	8.312729141	8.542429747	8.098747556	8.540202646
7.567065616	7.912365798	7.880363606	8.07563398	7.343153889	7.576687677

9.525471414	10.24316974	10.17440526	10.71860391	10.20634509	10.20358283
8.843148071	9.187244566	9.137887434	7.977207111	8.76386996	8.984231152
8.166172404	8.154321646	8.191204879	8.643157737	8.550897414	8.183602061
14.39736901	11.66708917	11.91416468	10.73661507	11.91912022	11.81887725
8.207099091	8.26142704	8.205892515	8.778623323	8.628622212	8.272620253
7.88791003	6.997069465	7.172666465	7.232737253	6.995550687	7.088518818
8.408296424	8.929384727	9.598893818	7.939228495	8.89604703	9.340550081
6.58104199	6.610013051	6.98587896	6.474296596	6.506514626	6.809755232
7.893940788	8.550414556	8.592161788	6.941467394	8.47014499	8.154321646
9.882779162	10.28795108	10.39641485	9.562364657	10.51650912	10.22448985
8.520754616	9.282486081	9.361074667	9.35188598	9.889177586	9.455015404
9.428190232	9.768390455	9.952967535	9.638309758	9.604846424	9.395854121
9.541747455	9.779738	9.762542495	10.0719806	8.35018502	8.853585596
6.733685141	6.960749798	6.393637899	6.902984	6.191632212	6.343264394
8.125376242	7.922697606	7.989899475	8.70424704	8.299695162	8.424934535
9.663106646	9.26122399	9.719000626	10.68338253	9.560346242	9.94767803
7.555661677	7.276277889	7.00317901	7.183932212	6.535531162	6.756409455
7.402701172	6.268423737	6.555298505	6.61398898	6.311444323	6.511217374
8.554027364	7.75479003	7.741148566	6.889002121	7.281402384	7.675499444
6.971315	7.136875657	7.249066101	6.85466502	6.719039212	6.747331182
7.757833798	7.488273475	7.427086202	7.02200603	6.671065323	6.850608899
8.027802949	7.963371182	8.072532475	8.627796121	8.315227333	7.599870798
9.745446081	10.48310631	10.01257663	10.28710845	10.21484784	10.30569114
11.16423752	9.90675497	10.52135963	9.285586323	10.12610261	9.632768747
8.036387556	7.694916828	8.206288727	8.00874802	8.48873997	7.97128602
7.509050374	6.437442354	6.61686503	6.261209	6.252266879	6.491055869
8.417196576	8.845226141	8.238272808	8.479727162	8.854603364	8.539776737
11.1612024	10.4700758	10.48185706	11.17571904	11.22456459	10.93646713
6.491493939	6.57287004	6.586881556	7.046726313	7.271416848	6.650783061
8.606624646	9.088505869	9.133284677	7.93437196	8.808928141	8.552719747
9.167758485	10.70181173	10.37124354	10.69099899	10.6426412	10.41426339
11.24323027	11.42371373	11.68948846	12.09497108	11.91651973	11.84529477
8.692870869	8.368326869	8.468418253	9.596088263	8.481851586	8.970512081
9.465071586	9.06557301	8.642670768	8.287438535	8.514431737	8.624974828
9.544495798	9.249612495	8.816947646	8.913024061	9.867911152	9.019731374
11.18291631	10.86691694	10.58347296	9.819688798	10.51174846	10.76983035
12.03602449	12.09225922	12.08128174	12.78185519	12.19727311	12.38794592
8.842374101	7.917403434	8.380307172	8.941359636	7.560401535	8.223899788
7.307847101	7.712540141	7.781852747	7.471589222	6.482290162	6.789755162
7.683943101	7.639261768	8.012698303	6.921417374	7.667309091	8.000883182
10.10841911	9.159196141	9.300983919	9.428845576	8.243637242	8.017539697
6.447481424	6.211688465	6.089395283	6.216389465	5.882632626	6.175655576
7.544716788	7.383097848	7.537240869	7.273507545	6.958715091	7.083894141
8.576891556	7.07011003	7.436640343	7.210929778	6.971547919	7.447087758
8.871686232	9.227886646	9.819688798	9.470173818	9.003357768	9.241421747
6.912275354	6.92644801	7.150169929	6.733937384	6.631383222	6.577737222
7.523206263	7.477877475	7.44801498	7.771540485	7.358075697	7.298118263
8.507703202	9.196132455	9.386484303	9.207219232	8.116577384	9.276189121
8.569735879	8.736738475	9.014335657	8.927565404	8.019097616	8.977005354
7.954533778	8.973478152	9.010430586	8.687969051	8.186741323	8.650384111
10.17440526	10.79956984	11.24816417	10.66945215	10.85225864	11.28896545
8.542859455	8.498503737	8.881793333	8.409959899	8.829514889	8.69723302
10.49140257	11.02551486	11.06261544	11.10170724	11.32027501	11.2608846
8.837022465	10.89912578	10.06255975	9.478383576	8.928828384	9.927686616
11.59654933	12.39973853	12.00354228	12.08943903	12.71520416	12.41634374
7.28903701	7.236914141	7.575132364	7.135797152	7.361380131	6.928307859
7.26819499	7.387388131	7.402015111	7.103272273	7.527339687	7.594662899
5.990113141	6.35186004	6.293110242	6.815206394	7.030426444	6.374790061

7.527074182	7.217289505	7.072618697	6.685804808	7.328126596	7.680845586
9.179819687	9.154469939	9.454390232	10.27139209	10.21054893	10.01974654
10.81540317	10.78856661	10.6488688	11.0940021	10.83930015	10.89755671
9.057828929	8.260520646	8.541972566	8.365106485	7.512168657	7.945472162
7.930919909	8.726024485	8.661956242	8.499404646	8.626833051	8.546545061
10.31289083	11.37847359	11.40145091	9.772980061	11.01683173	10.90466263
7.391081455	7.11316801	7.589888475	8.236217485	7.481960152	7.295980475
7.305287707	7.252886707	7.227000899	8.094218354	7.608647091	7.451812333
6.109379758	6.129955152	6.547766404	6.210980111	6.599616071	6.365723081
9.658772677	9.751885293	9.099925818	9.356741384	9.913634949	10.29851595
11.01683173	9.271759586	9.209526556	8.156410909	8.936938576	9.103811545
9.419020677	9.06333597	9.215289101	9.212980727	9.618955576	9.503858424
11.29399858	11.63174178	11.40495249	11.74935785	11.06573849	10.94640834
9.109760465	9.362770111	9.090721626	8.513601889	8.480158333	9.145078374
7.227000899	6.50824402	7.170490939	7.321884212	6.744878414	6.779669273
10.88166557	9.374121768	9.439917707	9.568406747	9.429506515	9.654354424
9.447242061	8.953164051	9.162016475	9.190205293	9.488337717	9.453123465
8.099466788	7.302736424	6.984268657	6.77672004	6.549368909	7.049242879
9.910960495	10.04511943	10.2187154	10.31894523	10.1421691	10.43440935
6.09363498	6.741204455	6.425110121	6.557137687	6.522313242	7.030426444
9.381544081	10.1597097	9.999132495	9.165786899	10.31289083	9.793942798
8.439112919	8.876209859	8.509936919	7.676257364	8.619848374	8.44047504
9.814415596	9.428845576	9.265583747	10.02061657	10.37026232	9.781348374
7.684267263	7.516607838	7.398696909	8.135526051	7.709908667	7.638981707
8.499836475	8.880364303	8.897751606	9.203189636	9.310775172	9.172421828
6.982384606	7.424024737	7.176635455	7.905781333	7.224144889	7.34349297
9.593075545	7.19681397	7.510343899	7.889381343	7.356542485	7.910120242
6.493067	7.28113	7.630934212	6.816827283	7.556918475	6.912798808
8.84786598	8.156085586	8.111719586	7.877487515	7.737346818	7.933221424
9.352487919	9.805520727	9.643287657	8.924680667	9.31393701	9.363307667
9.980879424	10.72246175	10.65751844	9.827596687	10.69793218	10.5104744
6.572638727	6.383199758	6.330516949	6.389669667	6.870614657	6.381819808
8.435296202	10.11246778	10.00094823	7.222702545	8.468898455	9.254890202
8.06745399	8.118473111	8.328154263	8.663769939	8.722452253	8.524867051
6.953635141	7.382821899	7.265083586	6.165117384	7.583507136	7.162716495
10.92757694	10.66945215	10.67815746	11.16579624	11.14057221	11.02105469
12.72610186	13.54965001	13.5160991	11.17910752	13.30255942	13.03496614
8.979921919	9.007167758	9.077100232	7.255477111	8.906407303	8.630877737
7.576687677	7.832891081	7.997498828	7.485802727	8.321509909	7.916712838
10.46108665	10.90754603	10.80644997	10.2600862	10.63014735	10.42297323
10.97054498	9.269224374	9.039454747	10.18942995	9.368875727	9.384078242
12.69316533	12.7494647	12.9709957	12.44611991	13.80033164	12.50328361
9.706432808	9.811091061	9.580783667	9.950180667	9.857104919	9.602112616
16.49551516	15.56435861	15.50564301	14.62217632	15.46469304	15.42966281
7.727390434	7.935081758	7.897743687	8.121211444	6.923947081	7.213178616
10.70052069	10.74045328	10.62142174	10.41224321	10.24316974	10.82153564
7.144617717	7.192707293	7.52469401	7.442598384	7.67197899	6.786766677
8.509502414	8.205133263	8.373919657	8.863920758	8.737817051	8.304980606
7.915342566	5.777438939	6.284925121	6.057302141	6.099109354	6.465379212
8.671294253	8.514909313	8.705608919	8.446641939	8.515350859	8.719668061
8.52305899	8.652865111	8.686126354	8.585303495	8.725555596	8.716864899
9.763952101	8.217667525	8.36093298	8.341133162	8.090157434	8.569735879
6.342479778	6.995042596	6.745632475	7.118205404	6.265371141	6.353400323
7.952774121	8.50111796	8.158321101	7.616235111	7.645943414	7.398020586
11.41981305	11.32185019	11.46145043	11.88649013	11.28565422	11.84027237
7.542431869	7.186327495	7.315791616	7.540776061	6.503631525	6.773917293
7.511838192	7.869396606	8.077230061	6.850882899	8.000144121	7.900011667
8.649462798	9.216574283	8.983202444	8.202220545	9.142791657	9.140978061

9.013314737	9.32739897	9.574750414	10.03492192	10.64379219	9.529469889
8.065602475	7.595301182	7.861832121	8.654464586	6.489542818	7.170490939
6.845301404	6.817996071	6.87893297	7.058212263	7.374297343	6.745348657
8.661956242	8.184399818	8.175288444	8.114611232	7.577683121	7.684267263
8.621272737	8.414282596	8.467667768	8.617840212	7.827504414	7.920494253
7.261194687	6.422006566	6.896711899	6.420979687	6.250843515	6.319865313
7.963371182	7.639662323	7.826840444	8.078044465	7.107702061	7.208321485
12.68484185	10.42512206	10.66832974	10.43332542	9.865550636	10.36589334
9.994772242	10.32208546	10.4549623	10.02439619	10.23160947	9.051957646
8.854095293	8.560860505	8.39867397	8.32939098	8.651209293	8.328154263
7.571116455	7.494510091	7.464704768	8.453639616	7.790242404	7.751645273
10.10187506	9.816676162	9.629101929	10.81796524	10.37124354	10.1597097
8.957944414	9.002847848	9.019731374	9.052474394	8.261007747	8.554528737
12.5521346	10.0188097	10.03492192	10.15379546	10.1684149	9.956562586
9.763138212	9.232683343	9.080123545	9.377330121	8.871209182	9.126663535
9.257324	7.762794545	7.772177253	7.855731	6.85163704	7.406158394
11.23116994	11.07797425	10.88681216	11.43380494	11.24457024	11.13240418
8.226840717	8.173633596	7.986535808	7.943134	8.255317818	8.131433848
7.054238101	7.404051424	7.442271202	7.719108384	7.956286283	7.538502939
9.963504081	10.32418729	10.51650912	10.39296715	10.83501466	10.56421743
10.20452777	9.663106646	9.652939283	10.0672481	9.920430606	10.12041459
7.531770848	7.975414556	7.386173242	7.116047475	7.306475687	7.557268586
7.928832091	6.415689222	6.75400198	6.523626889	6.326924687	6.475742909
6.627335	6.211511121	7.269598859	7.74646602	6.302690465	6.392742677
9.397864101	9.067856838	8.978204141	9.130293909	9.435122859	8.985325394
7.03599896	8.176718545	8.122747707	7.612294131	7.92597801	7.95873603
12.25844332	12.24097391	12.47761486	13.68884286	12.14507673	12.47761486
13.23341018	13.5360606	13.22156044	13.42153085	14.16629104	13.25686564
8.771294657	9.31327601	9.243679384	9.096395636	9.744663444	8.553132192
8.068564879	7.678291495	7.816254515	7.803944101	8.07651197	7.519695475
11.49239347	12.2031413	12.04990566	9.881176566	12.19996238	11.565007
7.412191343	8.103690354	7.814093232	7.626793707	8.273483707	7.557551899
7.268778051	7.304711495	7.612584303	8.065602475	7.950449798	7.701029869
9.203189636	9.62302801	9.449948384	8.283330131	9.379720636	8.780633818
7.348618394	6.970224646	7.335553323	7.004539727	6.422006566	6.732525747
12.62127559	12.06098883	11.97944932	10.98037262	12.0750029	12.01191659
9.636296737	9.838413929	9.629839465	8.932594273	9.281446566	9.361671283
9.932690333	9.240082808	9.132651727	9.874496818	9.566253768	9.482360616
7.243253313	7.88753598	7.919201576	8.221794202	6.56887297	6.881752616
9.385290182	10.31693391	10.28710845	8.557697889	10.1885747	9.330464051
8.732044667	9.163332636	9.220765354	7.552449879	9.041820404	8.276438626
8.550897414	8.204380404	8.00165403	7.117635758	8.064713051	8.142197384
11.49636188	11.16734497	11.46539851	11.17750487	11.09089144	11.28370047
5.82497602	5.757575374	5.817790192	5.76208101	5.980254848	5.75716304
9.634846707	9.865550636	9.935661677	9.839111	10.12799521	9.846508495
10.71031814	11.13240418	11.17041501	11.49029795	11.66189432	11.27499716
6.897750404	7.148452788	7.210091465	7.161605929	7.168103949	7.053635222
7.205804788	6.874988444	6.857741051	6.582190566	6.943812717	6.436179667
9.165150051	9.063885091	9.950180667	11.13400706	9.40167604	9.600185283
7.901142576	7.386471808	7.279669313	7.716479172	7.705814323	7.742113232
13.05987966	9.924448444	10.22361636	10.20532801	10.29101087	10.62280049
10.44094498	11.04464523	10.90754603	10.25627793	10.75062885	10.67190595
11.5737214	10.3494333	10.4450719	10.62280049	10.88302044	10.22761479
7.520648323	8.43615796	8.742149222	7.042119313	8.091725323	8.066048727
6.726480707	6.056874828	6.559504222	6.835608667	6.300351141	6.651250333
9.481000434	8.672348475	9.005433152	8.817758687	9.043522909	8.950355889
9.09178804	7.570765313	8.035675313	7.984288364	7.994228667	8.441767061
11.11856721	11.19817469	11.55928848	11.15966862	11.25700815	11.12935449

8.913024061	8.307893798	8.724013404	9.523397737	8.197369283	8.575095707
9.939327313	11.38025327	11.54675633	11.59212007	12.08680505	11.36065784
7.696259101	7.927799515	7.243557061	6.594827192	7.742113232	7.704713596
8.254484434	7.003981192	7.20666299	6.612991111	6.825325	6.891782758
7.487351192	7.341038	7.349181162	7.327234414	6.728691949	7.032569455
7.946541949	8.458697343	8.438325768	9.303342798	8.927019859	8.746311293
6.898538505	7.68740998	7.497307495	8.391869283	7.939609869	7.750279556
12.4956701	12.84627851	12.33576886	12.16942168	12.78645268	12.7939548
10.87907816	10.40387449	10.60161194	9.649399939	10.77890896	10.29208493
7.997498828	6.972885929	7.062669222	7.081850444	6.758395253	7.055635202
7.707585606	8.432612263	8.975838535	9.007167758	8.136235949	9.209526556
10.88945836	9.993897848	9.341818545	10.43998577	10.31013098	10.41957053
6.396200636	6.439107101	6.264245596	6.449508273	7.088250616	7.019921606
7.399358222	7.092673939	6.973155374	7.25688896	7.529686949	7.56165098
8.334587636	8.749346626	8.370422404	7.379773919	8.661956242	8.395296687
8.396532182	8.449337667	8.084275677	8.001277303	8.331888859	8.233838889
6.430485283	6.157001545	6.565610293	6.184352576	6.444612172	6.214135505
7.337386909	8.403930677	8.096128192	8.171342424	8.220625586	8.296772869
7.415309101	7.058479485	7.077700404	6.894670869	6.328925929	6.402478545
13.14611959	10.06450841	10.57028524	10.28899471	9.827596687	10.40925212
8.552719747	8.408718253	8.480158333	9.355403384	8.579336081	8.898231131
7.228169333	7.076844636	7.11316801	7.547650101	7.830100949	7.600248455
6.085914667	6.353983172	7.207252646	7.767042424	5.906078172	6.707842586
12.63888703	12.0750029	11.94481318	11.97447062	12.0838996	11.94745099
9.08614898	9.280885222	8.955359253	8.097566687	9.038261515	8.794361798
9.044046343	9.869466131	9.746868596	9.147897788	9.739859192	9.576740677
8.212243444	8.008400859	7.847379061	8.341977727	7.552094283	7.364797848
6.875689354	6.893115293	7.064243152	7.015344242	7.31189602	6.985120646
9.484209455	8.342368121	8.52305899	8.666113404	7.948408364	8.498167798
9.746145949	9.890949131	9.925979414	10.59809845	10.64623997	11.16281786
7.984288364	7.065949717	7.346567394	7.633560879	6.698966242	6.767752323
5.784110273	5.777324343	5.780165717	6.660563556	5.790651242	6.063755374
6.992656859	6.57823699	6.406383182	6.433428778	7.392218323	6.722265657
8.898231131	8.206288727	8.00874802	8.498503737	7.463713737	7.601643838
7.464704768	7.446413172	7.766357737	7.362377768	7.116617232	7.325732596
7.840676768	7.220481808	7.285287818	7.638629778	6.800553495	7.134684455
9.135618929	6.894957566	7.066776273	7.116341071	6.749805475	7.155245384
8.453639616	7.506099556	7.471847707	7.494798263	7.003981192	7.16725497
9.489614919	8.29927897	8.439112919	7.315524667	8.097958939	7.987912222
9.617464455	9.438094949	10.10542249	8.990220404	9.736139061	9.61181703
7.553068374	7.732206152	7.872008071	7.344596535	7.709201212	7.151291707
6.551212667	6.678790232	6.92669298	6.862375091	7.083613121	6.913788323
11.91416468	11.68484165	11.49636188	11.01683173	11.5612151	11.48459908
13.27418186	13.6823979	13.61616406	14.5108222	13.87263802	13.81266894
10.29660096	9.666877556	9.766131535	9.538263677	9.19736601	9.530933293
6.367286556	6.341087596	6.508699242	7.031727121	6.759969273	6.637667434
6.630903202	6.685804808	6.305750758	6.188392525	6.511217374	6.603596232
9.408808788	8.047933768	8.129874121	8.194999071	7.119314485	7.569572879
8.204380404	8.191573747	8.11773804	8.361756343	8.309668384	8.113745737
7.326942212	7.709511535	7.822730929	6.472300566	7.722297051	7.546918172
9.142791657	10.35591393	10.14044783	9.234418071	9.938475404	9.177504333
9.859664869	10.23066525	10.41626133	10.70299608	10.88945836	10.79420974
8.992957212	9.413942061	8.87289497	8.210602626	8.972363838	8.707057788
9.692043475	9.303966081	9.339764051	8.396125929	9.096395636	9.53973704
13.58996905	13.79381649	13.81935116	13.64083669	13.90036211	13.92172837
7.939903758	7.621361828	7.634532263	7.786626808	7.052221778	7.047573414
8.209054152	8.824381182	8.775001677	7.420173404	8.69723302	8.239938859
10.7982674	9.18155302	9.529469889	8.960773354	9.217165182	9.750460616

8.137069859	7.532150707	7.30673204	7.21346404	7.330801667	7.501777556
9.176146596	9.036569232	8.71185199	9.486974667	9.195534384	8.860747333
6.699648101	7.193876586	7.014205707	6.840910293	6.536641737	6.475135818
9.580783667	8.181196919	8.285708747	8.426780919	8.456191465	8.469339576
8.924680667	9.328020364	8.929972798	8.975838535	8.891390919	8.864955909
9.067241768	8.758871	8.720163182	9.112697707	8.752460404	8.738735232
7.462491444	7.599870798	7.515186091	6.832363455	7.07857001	7.107120283
9.415860727	9.542425758	9.709426838	9.758431737	10.07479617	9.897493586
7.573484121	7.885002121	7.949790515	7.009472515	7.346859747	7.771540485
8.046104414	8.386133566	8.399975414	9.053043889	8.605125889	8.679110091
7.296570525	7.30556202	7.486457414	8.009072212	7.546292778	7.817596162
10.43440935	10.41022215	10.18291229	11.31053596	10.67815746	10.60161194
10.49713487	10.57996368	10.71145754	10.30810853	10.34100163	10.74819911
7.728044798	7.824090626	7.948408364	7.994566778	8.063461202	5.91905298
8.733083212	9.217165182	9.17861001	8.043387838	9.000069889	8.903025495
6.516258222	5.816289535	6.763620737	7.172125253	6.404865071	5.974964202
12.83405786	13.62784329	13.54965001	14.11997744	13.92172837	13.89331098
8.998420707	8.582586091	8.329784131	7.715405939	8.581169111	8.496365737
10.2761618	9.858738737	9.559682182	10.080472	9.609027141	9.867106131
7.841440677	7.81270001	7.880750535	7.599211495	7.92237903	7.766357737
7.176396859	7.254644071	7.044969556	7.433629313	7.221869232	7.727390434
7.335252788	7.545042545	7.696877364	7.221626808	7.738299929	7.659822475
9.365016101	8.635209293	8.666113404	8.039451586	8.514037303	8.566871343
6.962306323	7.311629869	7.193876586	6.599616071	7.342871717	6.973155374
10.55842197	11.31267384	11.0859227	9.362770111	10.70052069	10.65871503
15.27762024	13.85817003	13.26276274	13.32669092	14.1830221	13.56234785
10.40701089	8.894948323	8.379056919	9.625623061	9.560980909	9.184527788
6.331316808	7.256041222	7.21346404	7.488273475	6.334208879	6.364101768
7.980568657	7.508036465	7.418614091	7.970926879	7.358688323	7.272331859
8.01013803	8.200163707	8.534897646	8.671783687	8.687053253	8.335469222
10.12909315	10.12314196	10.06071544	9.477181455	9.964343626	9.596773313
10.06633871	10.52364746	10.88166557	10.84302834	10.89094316	10.35178281
7.514105485	7.735579636	7.809982667	8.502042192	7.91707401	7.918472515
9.704210374	9.333361616	9.103811545	9.807061475	10.09896607	9.665316566
7.546592828	7.544047919	7.354804152	7.795116566	7.750957677	7.387388131
8.394903626	9.562994313	9.394567081	8.057063626	9.116804101	9.038261515
9.162016475	9.537497323	9.417594202	8.497692788	9.598265626	8.62460002
11.51008986	11.95514832	12.01673563	12.3774088	12.36759935	11.77053658
8.677215515	8.647922808	8.856895172	8.167813576	8.604635949	8.822853162
11.53050203	11.48061491	11.32559397	11.93770961	11.81221198	11.58621592
9.372025394	8.980943495	8.937429899	8.683580283	8.403930677	8.641223283
8.630877737	7.923778798	8.185563303	8.39227803	7.540434798	8.925229707
10.10946026	10.02161965	9.909202788	9.811091061	9.273640758	9.625623061
9.324974768	9.161011869	9.526200354	9.604175636	9.216574283	9.166446616
8.050105303	8.479727162	8.48873997	7.931708101	8.40783399	7.713270545
10.28506122	10.77103399	10.72127155	11.35080354	11.1060681	10.71390019
7.988251818	9.038849273	8.892401576	7.972411364	9.066605263	8.919862162
8.922632727	9.013830768	8.981975535	8.427685838	8.438712657	8.248137606
8.762202232	8.917069505	9.127872697	9.706432808	9.473962505	9.69725304
9.014335657	8.803482152	8.976374343	7.987573434	8.812517081	8.978204141
8.499404646	8.807959646	8.414679525	8.319524414	8.412578899	8.239474646
10.11664425	10.18942995	9.674270303	10.16937171	10.09682592	9.691258919
9.103811545	10.32750324	10.02161965	9.271123182	10.09506496	10.08599138
6.623383182	6.560443758	6.558838808	6.933815889	6.135409394	6.157508333
7.74678301	7.741148566	8.071703475	7.837132667	7.940608939	7.449946354
6.415279879	6.905316545	6.863389636	7.259665485	6.286870242	6.333400636
9.896649636	8.638952889	9.174120707	8.468898455	8.530333535	10.19236777
7.590248525	8.05903404	8.063008152	8.160266646	8.188379657	8.138156535

8.520243879	8.618800343	8.638487899	9.11510898	8.609106909	8.620285051
6.947262939	7.013931768	6.443888202	7.173570899	6.413438525	6.479663677
10.4283734	10.46766096	10.5431016	10.29432836	10.4524716	10.39747702
6.362142859	6.66276004	6.460273586	6.517203657	6.437269283	7.064538818
8.784649081	9.585510535	9.736139061	9.976273051	10.04340808	9.748306121
7.709201212	9.030004576	8.606099333	8.344441616	8.522641273	8.443175586
10.67815746	9.896649636	10.23550694	9.913634949	9.643287657	9.404880263
7.868335495	9.486974667	9.306330646	8.707515232	8.53715797	8.740682
8.777050384	8.520243879	8.632799758	9.164000111	9.108564253	8.927019859
6.571183485	7.784888444	7.66151798	6.67377398	7.850180343	7.294583061
7.56165098	7.34804498	6.891782758	7.831854586	7.575132364	7.246997929
16.09492117	14.78379092	14.57091557	13.85099976	14.37933382	14.67552064
10.6426412	9.101613848	9.337862091	9.080683394	8.658285303	9.059459505
8.363468485	8.263464364	8.546056747	9.440510152	8.639872707	8.618800343
8.272620253	8.333227545	8.104500051	8.156085586	8.510413414	8.254484434
7.794365768	7.344013535	7.836508475	8.600356081	7.676257364	7.892604899
10.74558579	10.68960982	10.83100199	10.71613341	10.23247836	10.78269205
7.475955232	7.990677606	7.549557061	8.392711081	8.217667525	7.954205616
7.411868313	8.50618604	8.350619919	8.444861909	8.086566798	8.268052495
9.182731869	8.629474212	8.844262616	8.277405556	8.24122704	8.214296636
8.981479273	9.103235919	9.402230293	8.870056939	9.007843273	8.955359253
7.344278889	6.930743303	6.275469879	6.149648949	6.230489586	6.348442212
7.282945384	8.538919798	8.477505576	9.054860525	8.348145404	8.205892515
11.17910752	10.05053825	10.25422487	10.41731261	8.832291949	9.113305192
7.906425364	8.934777717	8.829514889	8.594455404	8.70424704	8.705145061
7.270153101	7.372842667	7.626793707	6.909401626	7.61520499	6.868868606
9.818860343	9.526870273	9.324974768	10.1309568	9.707282667	9.774477667
8.208296687	8.246470545	9.548618051	8.729485838	8.702574091	8.527198758
17.67797782	16.96059838	16.96059838	16.36806566	17.01900663	17.23317693
9.993897848	10.71992703	10.62895885	9.12042397	10.4450719	10.47242114
8.232591606	8.587234515	8.928157121	8.428657566	8.480536717	8.718769576
8.092218303	6.47944304	6.994029394	6.772767788	6.567684242	6.807473404
6.532834	6.261014313	6.504270879	7.18421398	6.435479606	7.005354859
7.831503424	7.841729333	8.120795899	7.622357354	7.819838162	8.083806798
9.137887434	9.497084828	9.179819687	7.893273434	9.366312899	8.94874
10.37124354	10.72900488	10.90606985	10.76239578	10.82804231	10.39521192
6.657655061	6.267412545	6.129440091	7.415948364	6.621412929	7.051685909
10.58469265	11.08264222	10.8947391	10.5752557	11.19114259	10.78408516
10.39641485	10.32093584	10.13580598	9.924448444	10.1250404	9.911926424
11.26623333	10.40810668	10.70534521	10.42175216	10.06071544	11.4481816
7.879985051	7.572483949	7.486746556	6.533083232	7.349181162	7.05790099
10.71279176	10.74558579	10.83223099	10.01524205	10.60044575	10.91903182
6.767752323	7.247290424	7.086968939	7.288414848	6.395988485	6.583924121
7.853698798	7.262118616	7.296570525	7.21931098	6.720058788	7.12483998
6.285113455	6.816827283	7.354199768	7.069787606	6.410887212	6.322298141
7.515186091	7.686775222	7.742436303	8.268937657	8.058246657	7.796085586
7.099269525	7.041369354	7.023680051	7.774283859	6.212186293	6.472536212
8.585836293	8.774537848	9.117478424	8.004655889	8.584413131	8.776574232
7.601808626	8.196571586	7.910975505	8.756396081	8.046433182	8.299695162
6.458735293	6.428350232	5.802100768	5.879627889	5.848029121	5.867545636
10.08799683	9.647889364	9.660200838	9.888393747	10.28710845	10.45628541
6.969423263	7.349485707	7.300031424	7.348317141	7.459115707	7.492628778
7.837132667	7.390471152	7.573788949	7.476910626	6.735859162	7.178633636
9.16271003	9.379180747	9.243096889	8.113745737	9.198999222	9.165786899
7.789573343	7.272914455	7.415309101	6.677139455	7.461239687	7.51810503
10.81034167	11.13400706	11.54053762	11.33292491	12.12939166	11.27306561
6.879943566	6.934796758	7.644626202	7.135222657	6.685554869	7.290204495
12.71830686	10.11664425	10.7814083	10.23452156	9.94767803	10.3494333

7.304428303	6.951451	7.32338401	6.777950687	6.53573598	6.714000354
8.387083515	8.138519566	7.916391202	7.90403798	8.675348646	7.790916071
5.84823203	5.925077677	6.110927697	6.446140919	6.137685717	6.282981828
9.196132455	7.393766606	7.928481586	7.351945465	7.843485626	7.980256929
12.2031413	12.57486869	12.50328361	12.22629088	12.85048632	12.57107442
5.841974071	5.980387263	7.148452788	5.951827778	5.769844778	5.922892646
5.898604606	5.964156717	6.287413717	5.754432677	5.754315273	5.911651081
17.43906183	16.7804214	16.7804214	16.61084374	16.96059838	17.01900663
7.128516444	7.051418091	6.838696091	6.385847152	7.10076196	6.819416596
10.84568098	11.28370047	11.08435313	11.62290433	11.48258282	11.26446827
10.08978265	8.392711081	9.116279576	8.986432162	7.994566778	8.332313525
10.25811169	10.80782516	11.19638858	9.573320202	10.40925212	9.861430101
9.684886576	9.050265121	9.681142253	9.84311402	9.017695354	9.187808616
10.66465811	10.72633412	10.83501466	10.81280665	10.97476091	10.77103399
11.33477589	10.74172297	10.84176532	10.96886286	11.03737422	11.2278827
7.124332475	7.579959303	7.291068525	8.14677703	7.813414727	7.734170172
12.44611991	8.960773354	9.074692404	8.229734556	8.743124222	9.024707707
10.24828231	10.2572153	10.06450841	10.87092264	10.27432009	10.45375986
9.435722162	9.365016101	9.20200202	9.929312889	9.227275152	9.444645091
8.446641939	8.626355717	8.198599495	8.898231131	7.970574879	8.371229848
8.045324071	8.615330515	8.394903626	7.307022919	8.474228909	8.013857071
9.109120374	9.611261414	9.503271071	9.94767803	9.320738646	9.647889364
7.049507061	7.412191343	7.066192545	6.73278298	7.245014414	7.116869414
9.232683343	9.003925646	9.023639636	9.300367869	9.203760495	9.256767414
10.34311059	9.845595727	10.08599138	9.818860343	9.285000091	9.967178364
9.945994374	10.48897537	10.49466708	9.489614919	10.2187154	9.920430606
7.088019323	7.139090576	7.326634626	7.036210545	7.580904889	7.337386909
11.08936677	11.77053658	11.77552743	11.47871073	11.40725584	11.60888892
6.801007778	7.172125253	6.255110242	7.602282465	6.221395899	6.351647616
12.26475658	12.58957491	12.62449502	13.98831971	13.28559105	12.97977545
12.47130519	12.60022648	12.54589185	13.17778072	12.72972704	12.70390423
13.54965001	13.77352346	13.74759592	13.34204398	13.62106704	13.62784329
12.23191382	11.58814301	11.45738434	8.977005354	11.04593523	10.84705809
6.915952788	7.149903152	7.02934998	7.61520499	7.45523802	6.948313626
7.842052606	8.862225737	8.056301667	8.857327323	7.282635596	7.332669495
9.817379515	9.043522909	9.207219232	9.46096901	9.292435667	8.77298297
8.744562374	8.770297859	8.722452253	8.689422596	8.713915657	8.82024202
10.37215733	10.38738452	10.45041513	10.4429644	10.63870636	10.75737971
9.070661273	8.625920364	8.559011545	8.655437626	8.053207788	8.179951646
10.14713571	10.58935103	11.04593523	8.486543404	10.35917202	10.50057506
6.683791949	6.442114232	6.563407434	6.931501667	6.909691071	7.012316737
9.282486081	10.94640834	10.94353341	9.707282667	10.55333109	10.35069541
10.32418729	11.07158908	10.73384189	11.49839615	11.308834	11.08264222
8.329784131	7.98506802	8.017104828	8.742149222	8.322806515	8.379510323
15.76560628	16.57174008	16.39933956	16.52993947	16.52993947	16.7804214
9.899969596	9.654354424	9.980047091	10.76983035	10.14999886	7.17606597
10.39521192	10.33696433	10.5397012	10.92337564	10.32849396	10.92337564
8.226165	7.871683444	7.885731566	8.33273403	7.990313949	7.976116929
7.420173404	8.319901444	8.227704253	7.655304091	7.832217929	8.012010707
10.83363242	9.946747141	10.35069541	10.54950911	10.02269473	9.948421707
7.543396525	7.916712838	7.576363505	7.104098253	7.426803586	7.659186848
7.32157099	7.419865051	7.522559465	7.470612081	7.817596162	7.462172081
12.53563192	13.21058954	12.67700968	13.34730616	13.35359128	12.83827393
11.42178201	11.05959848	10.99345288	10.69931975	10.59418919	11.03452834
11.56291631	11.64044206	11.73271713	11.30216845	11.4090051	11.82346178
13.89331098	13.99495772	13.75520609	14.223913	14.29173122	14.1282639
8.804477505	8.709464434	8.808345061	8.77608297	8.899757485	8.748883111
10.99186298	10.4549623	10.6426412	10.59292728	10.30569114	10.64738653

8.057063626	7.888681737	7.876138384	8.097958939	8.166172404	8.013081051
12.13244857	11.30690717	11.43773109	11.28565422	10.49830212	10.66706732
7.164403717	7.007889061	7.394027687	6.729937747	7.208321485	6.829579545
6.71776502	6.271799455	6.063192515	5.991626222	6.344188131	6.32793802
7.964547263	6.937881404	6.808256808	6.856746788	6.585495889	6.870918222
6.627087081	6.760181828	6.714999636	7.428304182	6.972058485	7.177458596
7.898205273	7.766006091	7.790242404	7.720541949	7.945111576	7.869709071
8.410410394	8.966282283	8.942313313	8.919352101	8.836542848	8.321509909
15.39581235	13.3718573	13.77352346	12.99565877	13.71423947	15.56435861
8.731005677	9.257979848	9.139095455	9.290785283	9.153730283	8.945493505
9.042346232	9.008956374	9.01707996	9.202552434	8.493653778	9.206093556
9.239472869	9.484209455	9.36748	9.925226333	9.558255162	9.59460096
9.995698596	8.256076333	8.53670504	8.401710747	7.692217293	8.078774939
11.65052704	11.85617239	11.95514832	12.20960187	12.3843369	11.58005536
8.223899788	7.784529071	7.491021444	7.054238101	6.800063838	7.187484444
6.602902657	7.492937667	7.223246162	7.561964414	7.180291929	6.650544455
7.337989111	7.856391758	7.616235111	6.582672778	7.626470505	7.434843313
10.59062542	11.03580591	10.74945851	11.20163629	11.16888788	11.10035391
10.3810842	10.96748398	10.89912578	10.22267877	10.77507269	10.97631922
9.07419996	10.03492192	8.39446903	9.711723394	9.756840071	8.696280707
10.78856661	9.658049545	11.16579624	10.8193093	10.21157382	10.70181173
9.899010949	10.19418334	10.04600105	9.626304798	10.042425	9.486281202
10.56306707	9.118629636	9.337187111	8.700606657	8.941359636	8.90531298
15.83165575	13.62106704	13.62784329	12.3843369	13.35919327	13.62106704
7.168394253	6.573737152	6.407880727	6.858224505	7.158855657	6.738515899
8.724013404	8.954176293	8.836542848	9.384078242	8.981479273	8.838495424
9.179226364	8.135850465	7.931306778	8.130616515	7.965251131	8.003479939
8.287888051	7.896287434	8.057063626	8.076845152	7.36076402	7.593664717
10.97476091	11.56291631	11.45002291	10.52476206	11.3733012	10.97054498
6.299772566	6.113628535	6.500186222	6.054301051	5.895332323	5.805059394
7.56320098	7.354460354	7.745118444	7.463067838	7.402015111	7.506434626
8.282073859	8.431326525	7.31937996	7.600248455	7.595301182	8.231756293
8.540635232	8.254082232	8.623178879	9.025193869	8.294235768	8.348145404
6.84402404	6.952257202	7.092080626	7.652952818	7.798826303	7.311327071
13.32021166	12.01191659	12.39660011	13.15082863	11.47682743	12.52130815
9.12042397	8.382609505	8.702574091	7.346859747	7.949071818	8.05903404
7.957018646	8.209054152	8.203151141	8.524867051	8.109636222	8.275579182
8.623178879	7.942409707	8.519692939	8.649944576	7.664957788	8.331458747
8.786134101	9.176146596	8.534432515	9.901640636	9.339764051	9.479751879
6.787827455	6.24729001	6.332476354	6.567684242	6.070726232	6.191632212
6.475338556	6.87893297	6.544818747	7.06104997	6.610013051	7.584965596
12.32024961	10.51174846	10.92190902	12.5521346	10.32946621	11.67593314
7.072297535	8.04259702	7.489478697	8.254082232	7.849463182	8.264694495
7.124596343	5.946081162	6.464011586	6.498851707	6.140397172	6.487263202
9.296131263	8.02698897	8.822853162	8.088956818	7.997498828	8.410908071
9.831087364	9.521192909	9.554043101	9.005433152	8.959665061	9.605576293
8.927019859	8.993505475	9.355403384	9.669114061	9.23383296	9.294355394
9.072403576	6.927522051	7.141989162	6.448364717	6.593634414	6.815206394
7.951633808	8.156772778	8.509502414	8.48873997	7.30673204	8.368326869
8.046433182	7.877844556	8.006915303	8.53670504	7.654256818	7.70299303
7.693828071	7.155827141	7.220780818	7.68296396	7.388278253	7.70365599
8.441767061	8.458294828	8.33273403	8.852092202	9.299183616	8.859179455
6.583924121	6.652395162	7.198597879	5.993149869	6.167536404	7.928125404
10.15186663	10.83223099	10.77762269	10.31693391	10.99186298	10.87637444
9.802496737	10.95234717	10.93935201	10.13671639	10.80782516	10.78014639
8.072110051	7.008962394	7.859293	7.720197707	6.96545202	7.318843495
13.12023156	11.78023213	12.03068847	12.00086001	11.14866374	11.76336308
14.99939481	11.82112126	12.04734694	11.58814301	11.65261158	12.39973853

6.873794323	6.858444758	6.871437091	6.491736758	6.484023485	6.768970657
15.74256723	14.91973608	15.01206542	14.50017253	14.94534759	15.10875812
9.725631121	9.467500556	9.35188598	9.742174424	9.250125475	9.157435455
8.909945455	8.630877737	8.806957455	9.495686737	9.032817303	9.132651727
8.829061111	9.107388182	9.118629636	9.716648152	9.06333597	9.537497323
8.87289497	7.973992111	8.207926798	8.895503556	8.631899566	8.726978374
9.62845904	9.969958172	10.23066525	10.47123164	10.05688349	10.16642595
10.83930015	10.44094498	9.773700283	10.77103399	9.867106131	10.07388225
11.13240418	11.35278885	11.34892675	11.79171062	11.60479972	11.75462574
6.809240364	6.813169384	6.827413455	6.737098263	6.995294596	7.077096727
6.363508222	6.299210717	6.254380545	6.326728293	6.792458242	6.751435283
9.244399414	8.687053253	9.109760465	9.509226798	8.460863192	8.219832939
6.961503899	6.564945162	6.704194818	6.904054566	6.559965303	7.029601354
8.708030737	9.124970051	9.296718121	8.758871	9.251353071	8.887109626
7.68296396	8.147481596	8.022720303	7.567690545	7.939903758	7.530574707
7.727729838	7.983913121	7.41731598	7.182269455	7.081850444	7.087495071
7.395300939	7.676956101	7.331457121	7.001377828	7.735579636	7.364550071
7.706168626	7.21931098	7.267131253	8.231756293	7.34804498	7.174439091
8.738283333	8.446184828	8.240375404	7.660795616	8.41552702	8.346198293
8.823889374	8.308776677	8.278157657	7.893631939	8.164230707	8.339546939
13.70143341	13.80033164	13.98831971	14.01682491	13.94443	13.74759592
9.972781707	10.58469265	10.69354222	9.404880263	10.31480319	10.3090972
12.12151457	11.92396137	12.14789109	13.18858184	12.03912361	12.35168631
10.50166798	9.898158111	10.03385507	10.03123363	9.623794152	10.03983393
10.81142627	10.0672481	10.35178281	11.02105469	9.857940545	10.35591393
10.74819911	11.13551505	11.20163629	11.86652814	11.34732253	11.20498985
10.74172297	11.12935449	10.76983035	9.821914929	11.0035084	11.02243393
8.181574081	7.382527051	7.369172101	7.137450808	7.104930788	7.096055535
10.66832974	9.934615404	9.979092404	10.03205964	8.736738475	9.198999222
9.631972414	10.22164118	10.33890802	10.16165443	10.91514001	10.761049
7.470886646	7.280525364	7.429246232	7.114645596	6.396985434	6.573737152
9.402814081	9.35067002	9.640429374	9.159196141	8.79722998	9.436898869
11.73070023	11.55322016	11.21187068	11.1031408	11.24323027	11.57765358
6.215155556	7.608934747	6.199844141	6.257640303	6.390148364	6.27383702
8.888766747	8.151247717	8.302062758	8.727528626	8.491898242	8.574218515
8.227270172	7.300330596	7.432695485	7.401460495	7.249375465	7.233650202
8.581684657	9.033915556	8.963499596	8.978723121	9.49510903	8.978723121
11.32721575	11.51260306	11.46936418	11.7349036	11.72807534	11.21816154
13.95167504	13.23341018	13.36582475	13.03496614	13.37822002	13.32021166
7.585519525	8.219387636	7.447389475	8.216297535	7.923778798	7.890755525
17.23317693	15.95256028	16.49551516	16.49551516	16.4534382	16.4534382
7.245014414	6.072015283	6.583924121	6.653596384	6.302516242	6.604082657
7.275223848	7.742830394	7.714317071	7.268488485	7.777661384	7.74678301
11.57765358	10.51771241	10.73661507	8.431738505	9.212349869	9.90675497
7.552833101	6.657892071	6.590229374	6.641378788	6.732033313	6.847553121
7.173570899	7.34349297	8.143040162	7.890421556	7.224851116	7.374842788
6.808010384	6.912541192	7.571747222	6.845568374	6.836530859	6.872867768
10.60277882	11.06877356	11.03296099	10.4655823	10.48539779	10.57996368
8.175629323	8.303793737	8.116212253	7.230210646	7.97471396	8.382194192
10.42512206	10.31193963	10.21769501	11.04156144	10.31378621	10.70775781
8.257984293	8.544134919	6.900245465	7.09353204	7.000811505	8.35372597
7.603101596	8.115821848	8.056742909	8.657372182	8.257984293	8.065153556
8.301657889	8.650965909	8.682101313	8.154139455	8.510894717	8.730448515
9.808672222	9.430143091	9.460382818	9.851320949	10.10542249	9.427546879
9.366312899	8.970512081	9.333986848	9.240082808	9.026636737	9.402814081
7.637927172	7.67197899	7.74753198	7.670568384	6.840661414	7.104930788
10.38842688	8.609106909	9.590351455	9.281923404	8.25570701	8.83948901
13.87958112	12.05489479	12.39973853	12.58957491	11.9091429	12.21312889

7.949071818	6.896472707	7.059356717	6.94462002	6.526092091	6.705893697
9.887613414	8.85785699	8.971727333	8.674281929	8.600831152	8.57971698
6.539797657	6.661293222	6.433023606	7.067022475	6.790317606	6.745632475
8.647922808	9.363823202	9.247274333	9.389466253	10.13193078	9.968215758
7.098723051	7.544716788	7.205228253	6.917222737	6.891782758	7.118512586
7.677613273	7.801006182	7.339805162	8.727989172	7.455517596	7.566450929
8.457043091	8.78664797	8.716864899	8.080354404	8.834473929	8.324453253
6.620737576	6.833605444	7.301216929	7.535530424	6.443018677	6.397781283
6.933584646	6.737819111	6.60520404	7.286117455	7.362377768	7.262427071
8.494576061	9.07526898	8.922082808	8.448008778	9.028300162	8.841037687
7.33858799	7.962380919	7.89464897	8.493653778	8.290353162	7.893273434
8.551751717	7.952386505	8.384837869	8.239474646	8.458697343	8.433939838
9.831834717	8.381754576	8.349393212	8.079973212	7.500795283	7.962004212
9.243679384	9.990505182	9.833438364	9.698896848	9.983540212	9.419654929
12.40343679	12.40343679	12.55625498	12.21991364	12.90783816	12.56726296
9.246644323	8.900779434	9.078331919	9.294355394	9.187808616	8.547862646
9.728754818	9.742174424	9.277992232	9.417594202	9.856174394	9.295516616
10.85225864	9.57181996	9.669114061	9.525471414	9.324974768	9.435722162
10.85923156	11.3733012	11.11560115	10.83363242	11.36793803	11.11081747
7.28113	7.386173242	7.413750677	6.741723879	7.097545323	7.004539727
7.944406939	8.014979636	8.067838768	7.532708636	7.33858799	7.468171747
10.33476777	10.88546605	10.61261659	10.95234717	11.01531103	11.00620373
7.409710545	6.610225071	6.671065323	6.647920455	6.988399394	6.988144556
6.05329202	6.079466192	6.026785687	6.000819535	6.05329202	5.970368172
6.435479606	6.329524818	6.634356909	6.27159296	6.425312475	6.281203727
7.791885697	7.851640596	7.636262586	7.335252788	7.857447394	8.079179394
9.237064687	8.946107263	9.120933788	9.49850403	9.437491071	9.299183616
8.191909586	7.457632404	7.652274172	7.90331499	7.606990414	8.136235949
6.906335848	7.321250939	7.371573384	7.648986848	7.592388485	7.498343283
8.183602061	8.430534091	8.614442374	8.76478299	8.997224192	9.094076404
7.382527051	7.303562909	6.908859909	7.108809091	7.08777197	7.769155828
13.62784329	14.1830221	14.16629104	14.84412351	14.25895571	14.15863941
9.547339828	6.688683899	8.70424704	8.951513343	7.193299919	7.866306808
9.904237354	8.447076788	8.600831152	8.561789232	7.80651396	7.775630727
9.590351455	9.710054737	9.459664354	9.94402302	9.698896848	9.214694788
7.644626202	7.886480404	7.721209061	7.969793182	7.99823603	7.02612702
8.029005444	7.108809091	7.059956313	7.052221778	6.955441212	7.098484101
9.103235919	8.531655515	8.594861495	9.857104919	8.707057788	8.644542606
11.31053596	10.14044783	10.13276962	9.655088667	10.01071956	10.24139873
12.02514953	8.875206121	9.278597747	9.045168101	9.033394545	9.406893465
9.650129091	9.868690293	9.989520798	10.23550694	10.22267877	10.06633871
11.57567047	12.10951308	12.32364634	11.99535117	12.24097391	11.97447062
7.242080394	7.782796596	7.659822475	6.677368899	7.671593667	7.589252838
7.51810503	6.446595263	8.188779434	6.95496698	6.495692828	6.847044404
10.42927797	9.383480283	9.398445515	9.321347081	8.956407626	9.066605263
7.362377768	7.25118401	6.923947081	7.167804919	6.649399919	7.65457904
8.63794701	8.759332636	8.925229707	8.881310737	8.683580283	8.810446485
9.750460616	9.633493677	9.684886576	9.925979414	9.685649929	9.868690293
7.550541152	8.660608859	8.595703707	8.664254273	8.587234515	8.363468485
14.08841453	14.37933382	14.46162431	14.86987024	14.4185289	14.60195877
8.601896768	7.832540091	8.433939838	8.508189222	8.259704283	8.152443121
10.62142174	10.2752886	10.49592664	10.687913	10.21769501	10.4322159
8.540202646	8.352537101	8.369167818	7.527339687	8.303356141	8.099825778
10.17030341	11.41071085	11.35657003	9.904237354	11.10170724	11.41778198
6.530171141	6.803567182	6.951961606	6.576344929	6.964624475	6.874245465
9.571117879	9.301561	9.826032111	9.775265525	10.17904437	9.728754818
14.35197184	11.78498903	11.93178106	11.53239215	11.79405389	12.03068847
8.574674323	7.428605818	8.253639283	7.752360667	8.082133975	8.179581869

9.187808616	10.4450719	11.03452834	10.19957169	9.516398909	11.61117883
7.805068949	7.469634172	7.186327495	7.08018898	7.36510997	8.307893798
14.93426708	16.42659037	16.28343863	16.12384164	16.2036826	15.92931651
10.49466708	11.05356995	11.04316622	11.25349741	11.78023213	11.01683173
8.501579646	9.145664475	8.965143333	8.149788848	8.81572204	8.600831152
7.477877475	7.460268556	7.480348535	6.983154111	7.731201121	7.823429646
7.450888848	6.984849141	7.362680232	6.871158202	6.523815232	6.803094364
12.97555423	14.15863941	13.69473258	12.43944324	13.48401144	13.09463792
6.80380202	6.766018101	6.698966242	7.228468091	6.963620111	6.762314343
6.479217141	6.966976535	6.498588101	6.393175788	6.773917293	6.476859778
6.976769172	6.411697737	7.09469803	7.084144263	7.185402798	7.083314707
6.538425424	6.233403424	6.031850414	6.486163293	6.223508929	6.253499364
11.5612151	10.61480751	10.50543333	10.1838089	10.78856661	10.82416773
7.759566061	7.696527091	7.679634566	8.262181081	7.341038	8.029005444
8.827377505	8.163053768	8.433042727	9.512482222	8.208296687	8.622644364
7.433328091	7.214315212	7.752360667	7.419865051	7.834378071	7.438935576
9.748306121	10.64738653	10.29324401	8.832291949	10.47123164	9.726443586
10.58935103	11.39772243	10.87637444	9.379720636	11.52645916	11.11388317
6.745348657	7.709201212	7.204610747	7.45523802	6.658852677	6.707366768
8.85308204	10.59292728	10.42622906	8.572701232	10.51886057	9.59387599
10.3779146	8.361327455	8.726024485	8.62460002	7.530907465	8.47014499
8.233421293	7.103541778	7.20918101	7.102116364	7.023397576	7.255805293
10.16743096	10.32531651	10.2752886	9.118059475	10.22613156	9.908305848
7.55945201	9.168828475	8.578393657	8.873520545	8.929972798	8.476485121
15.65613201	12.73731843	12.48458958	12.34237091	12.81379045	13.07947925
8.20265299	6.316707768	6.567185838	6.953914061	6.330516949	6.542971677
10.65365596	10.1300726	9.649399939	9.880317061	10.5397012	10.84844593
8.655437626	8.553132192	8.13267903	8.376150616	8.205133263	8.461795505
7.101573333	7.332344778	7.481322818	6.350448798	7.437384141	6.938883283
11.74670945	12.97977545	13.33155169	12.97977545	13.15645152	13.20018245
11.08756575	11.62739451	11.60888892	10.51400776	12.01673563	11.09089144
10.27714677	9.673359929	9.839882283	9.493200828	9.608350808	9.725631121
8.961854929	8.349393212	8.717364515	9.978126798	9.137887434	9.250770152
7.61520499	7.566119051	7.357173859	7.064538818	7.422534091	7.467830485
8.987499273	9.295516616	9.115691495	8.126253929	9.113305192	8.920488949
7.89464897	6.841594566	7.559118222	7.140269465	6.91172998	7.505175253
7.142620404	7.124025636	6.991849242	7.195224778	6.339864232	6.461688586
10.77507269	10.9991534	10.94208941	11.44643795	10.19649023	11.238428
8.965143333	8.214678465	8.732044667	8.817191758	8.427236737	8.438712657
8.737323475	8.836542848	7.816902374	7.995268899	8.744562374	8.12156602
9.93099102	9.019731374	9.591816061	8.796283929	8.960218374	9.341222778
9.076382242	7.802503596	7.895278253	8.475612051	8.124212202	8.065602475
11.10460602	11.71443529	11.80791686	11.40145091	12.08943903	11.85617239
6.898002222	7.485478828	7.390207394	7.505175253	7.048974677	7.066192545
12.11216258	12.72219022	12.93430142	13.05987966	13.32021166	12.9851923
11.54675633	10.96311364	10.96886286	10.59180142	10.80499832	11.22600179
10.84176532	11.22119175	11.50425191	11.4413808	11.52824	11.3403052
8.063898495	7.912742707	8.105629576	7.80651396	7.911995121	7.831854586
6.241562485	6.078134778	6.256334263	6.643553717	6.174477505	6.82018896
10.09339803	9.290785283	10.15186663	10.24727703	9.419020677	9.509226798
7.055909434	8.101068051	8.099112939	7.236914141	7.365381232	8.290353162
9.247274333	9.812775374	9.895061889	9.116279576	9.382882596	8.867918475
16.52993947	15.02501841	14.9591327	14.05855866	14.89443037	14.83117585
12.70390423	12.83827393	13.23341018	13.16151113	13.61616406	12.58612865
9.569854202	10.75737971	10.73517618	10.35917202	10.19528987	10.46108665
6.431116323	7.082160636	7.115757364	7.462491444	7.252038162	7.297796364
6.250090798	6.424673818	7.005354859	7.28113	6.742790232	6.679026717
8.537544354	7.911629535	7.531770848	7.59785203	6.889250929	7.283562505

8.460488909	6.773462798	7.038360253	6.329109535	6.558838808	6.912275354
9.826032111	8.212243444	9.752732475	9.800083455	8.248566778	9.035040848
8.83948901	7.698638758	8.055566596	8.071703475	7.886480404	8.165380303
12.75784116	9.829119768	10.13862753	9.699589758	9.860587788	10.46442497
8.002342131	6.539575101	6.836284939	6.58529104	6.608482505	6.774936697
8.519692939	7.596246919	7.830796253	7.736619182	7.399358222	7.838558162
6.365312818	6.72130502	7.496977949	6.920203848	6.384073253	6.356712303
5.900311293	6.817526586	6.864476545	6.503188616	6.590229374	6.352813182
8.350619919	8.051256737	7.996770556	8.395713444	8.166602566	8.188779434
8.720163182	9.077100232	9.061091758	9.326739394	9.264989899	9.352487919
9.078885242	9.660861778	9.573320202	9.920430606	9.515069697	9.730279475
6.747571626	6.742510061	7.275765808	6.550797374	7.094997242	6.977817737
6.412595495	6.343446081	6.449700465	7.069165141	6.391201182	6.470121364
8.757866131	7.893631939	7.784199556	8.037287293	7.40806004	7.780116303
6.18159802	6.16126504	6.372158566	6.540677515	6.117409697	6.355148343
10.98749259	10.56655729	10.74305691	10.50794857	11.13713447	11.20345844
9.649399939	10.1985269	10.10841911	8.559011545	9.955574404	9.376667182
13.72760196	10.99046505	11.11856721	11.15666056	10.41530743	11.00902763
8.173308798	8.773988747	9.029402949	9.123926859	8.727528626	8.935320828
9.421700798	9.678297182	9.530205505	7.957660747	9.666877556	9.409395929
8.879851283	9.817379515	9.608350808	8.344860929	9.441908576	8.942756889
8.598479657	9.018802576	9.159785394	8.069753788	8.916479111	8.689808788
6.639986485	6.636272576	5.990249465	6.185353808	7.066488111	6.522975131
7.839249838	7.351625758	7.144887111	7.369172101	7.892604899	7.582966818
7.555333263	7.690840778	7.736619182	7.587956192	7.574795778	7.525015414
8.095746162	8.235056909	8.363468485	8.752912545	8.417604414	8.22344601
7.279410566	5.859544788	6.524057606	7.686444293	5.980387263	6.371193616
9.564343333	10.25127201	10.01524205	8.057421596	9.694213162	9.595333525
8.212685152	8.680513909	8.221010253	9.106199707	8.740195303	8.653906556
9.848945263	9.278597747	9.476588061	10.49830212	9.720489222	10.09597734
7.581254909	7.427372788	7.581657414	7.877844556	6.945089283	7.022843394
8.553595747	8.930433505	9.941224515	10.91356479	7.763504859	8.768300525
9.173615515	9.267923343	9.273022949	9.828404283	9.910960495	9.183367525
13.57574016	10.4524716	10.78705857	10.06450841	10.72127155	12.28693608
7.301506111	7.21346404	6.916922333	6.264838313	6.658617323	6.824112909
13.14093405	10.18762619	10.60277882	10.39521192	10.20060626	11.04316622
8.819236758	8.890936242	9.19736601	8.665165081	8.454049747	8.933684374
8.64507902	6.978602455	6.97366599	6.631857323	6.687730495	6.610225071
7.701991131	7.391655768	7.936644384	7.285287818	7.15072903	7.166369152
11.75462574	9.983540212	10.63516273	10.24139873	9.726443586	10.3896003
7.137147091	7.400588576	7.209485919	6.891529131	6.442114232	6.521696273
9.669114061	8.782655051	9.149684939	8.956909848	7.638981707	8.327767232
7.522869182	6.529766576	6.544566455	7.020691646	6.583167424	6.782883697
9.03221704	9.481000434	9.123926859	8.729972586	8.55725397	8.583996586
9.208368677	9.694213162	9.47593803	9.19736601	9.917863333	9.659537192
8.735755485	8.644542606	8.02125603	7.498029465	7.25402203	7.469344313
13.97489728	15.08134613	14.36070414	13.47832004	14.36070414	14.19920898
10.00094823	10.31597323	10.41957053	10.79145221	11.17378617	10.74045328
6.888722505	6.942817626	6.679264929	6.457134202	7.034331232	6.586881556
12.78185519	13.36582475	13.31405593	12.91656586	13.34204398	12.46055504
8.354947869	8.500240172	7.970926879	8.451550535	8.23139899	8.665165081
8.318250424	7.433916687	7.520648323	7.29687102	6.798855303	7.099882485
7.582966818	6.80697597	7.059676455	6.587802667	6.851123798	6.905316545
9.733230051	8.895503556	9.192533242	9.172421828	7.982442283	8.45110203
7.879657162	7.723009	8.02698897	7.842683778	7.538502939	7.215185141
9.948421707	10.12041459	10.23452156	10.72900488	10.35591393	10.38531322
6.605935465	7.021482505	7.204326061	7.309336424	6.365723081	6.623383182
7.389236737	7.750279556	8.445759657	8.25927404	7.400588576	8.08193004

11.17213305	11.47871073	10.96311364	11.24001011	11.51465712	10.82290783
14.49002723	14.63205333	14.65397593	14.29173122	14.64328928	14.38889659
8.58807297	10.25906505	9.642500081	9.878686616	9.841562192	9.859664869
7.531209667	7.714317071	7.396159798	6.934051768	7.44112097	7.281724869
7.603719626	7.739314727	7.630594101	6.899006192	7.672313364	7.623386535
8.108523939	8.559011545	8.152087687	9.297353606	8.970512081	8.242431778
7.243557061	7.170490939	7.186960111	6.343830616	7.327857495	6.676440081
8.52575703	7.393489313	7.654256818	7.794365768	6.951745222	7.489478697
7.311037152	6.904054566	7.032021525	7.491021444	6.405058101	6.475338556
7.723687384	7.137721343	7.408681949	6.453863293	6.552973232	6.432015545
10.12408141	10.32635032	10.61480751	9.263851586	10.20452777	10.13768673
8.035288182	7.01565997	7.842359758	7.953126222	7.008962394	7.896287434
8.079973212	8.283707475	8.249809909	8.228066545	8.388817192	8.447526838
7.889381343	6.359365273	7.300653717	7.123768636	6.940479424	7.19003896
7.222960121	7.07551896	6.937881404	6.046027313	6.562721354	6.546440545
11.98999321	13.39037726	12.10414281	13.66784462	13.70717091	13.0147344
11.51647662	11.9982543	12.06964699	11.04593523	11.53239215	11.79615897
9.746868596	9.67665103	9.671170444	10.10365156	10.18666124	9.923658606
11.69658017	11.76804793	12.03912361	12.71142619	11.88148631	12.22341713
7.560401535	6.971547919	7.587956192	7.395882727	6.970224646	7.145769434
6.597234394	6.558125737	6.443240697	6.27402404	6.437442354	6.748324394
8.005065495	8.411696677	8.31214998	8.50673198	7.617189485	7.648616949
6.073749475	6.526565121	6.895709172	6.203676525	6.161435566	6.190240242
13.77352346	12.69676846	13.29117716	13.46005067	12.47441306	12.47441306
8.086153313	8.145962667	8.060661899	9.109760465	8.577417788	8.322342051
5.776898404	5.896855071	5.981454889	5.922804	5.763926939	5.758476465
9.588410576	10.156858	9.995698596	8.741655828	9.896649636	9.762542495
6.178813899	6.981086404	6.676905949	6.821773354	6.124834283	6.332476354
7.18313201	6.843046434	6.587993828	7.879657162	6.536426172	7.025021455
11.51465712	11.63629658	11.77053658	11.70812672	11.64637724	11.4535969
7.629673333	7.941721242	8.065602475	6.965960869	7.890421556	7.665987182
7.907197636	8.781680606	8.337114525	8.466446051	8.08537204	6.992392121
7.665987182	7.189710394	7.434843313	6.901978929	7.504860717	7.650531152
6.760181828	6.990995828	6.988399394	6.340472131	7.008416879	7.034331232
8.386133566	8.503061444	8.592622071	8.39446903	8.648914657	8.761177727
16.22759729	15.38120792	15.39581235	14.60195877	15.34617167	15.08134613
12.10694646	12.43279956	12.37416009	10.94640834	12.12692782	12.12939166
9.108564253	8.690381253	9.322592778	9.350012182	9.225515485	8.704708384
9.003925646	9.544495798	9.591133828	10.15086094	9.601568657	9.74383503
6.104272606	6.595312657	6.920433444	6.157664758	6.957146182	6.195589626
9.828404283	9.997192202	10.27035429	10.41123643	9.785178838	10.36465285
8.859715222	8.713915657	8.881310737	8.016751283	8.445297313	8.888766747
7.036210545	7.420173404	7.357456071	7.611975737	6.648620667	6.458494616
6.249071646	6.068167424	5.981818636	6.401596919	6.732288444	6.441255838
8.380828141	8.64507902	8.638952889	7.360451687	7.967920667	7.864224778
9.235179424	9.118059475	9.150811232	9.238221677	9.430738808	9.190205293
7.486088616	7.569899606	7.710578727	6.755203192	7.571427586	7.387654737
8.683580283	8.116577384	8.808928141	9.116804101	8.57971698	10.27139209
10.85782556	11.82852227	11.76336308	11.77305263	11.74670945	12.08943903
8.309247939	8.601896768	8.331888859	8.893968333	8.718769576	9.231946182
8.880364303	9.036076414	9.037153444	8.996148737	8.625230823	9.876096838
11.41605585	11.46320141	11.59427705	11.85033795	11.26446827	13.21058954
11.65918316	11.15808934	12.13538265	12.18142186	11.77802923	11.59870577
13.88591407	14.61202705	14.79546777	14.6970922	14.9847254	14.5513462
6.273265505	7.12541897	6.861656576	5.976785828	6.725730828	6.438718404
7.850890354	7.534302667	7.365664505	8.476085384	7.744777657	8.806957455
8.074904687	8.411285283	8.333227545	7.306475687	8.339546939	8.210186616
11.89130603	11.44319437	11.26446827	12.00354228	11.71659127	11.77305263

11.29597305	9.203760495	9.381544081	9.325601172	9.348735909	9.76546198
9.493894545	9.681893121	9.867911152	9.987874111	9.252443646	9.563605545
7.488894061	6.555084162	7.442908828	7.330192545	6.76309904	6.906092707
9.66383	9.056062667	9.870931818	9.54312803	8.742149222	8.960218374
8.57597697	6.446140919	6.53935602	6.686319576	6.767263616	6.540451323
7.59498599	7.504556444	7.499556727	6.591596333	7.368501293	7.047846657
7.774283859	7.994228667	8.076845152	7.217862778	8.000521869	8.354182626
6.659312505	7.000309313	6.304241131	6.659312505	6.789025475	6.962034242
7.997875909	8.40702104	8.106418192	8.090886384	7.977952253	7.956286283
10.82034332	10.77252405	9.94767803	11.31449746	10.80091358	10.4700758
10.35284213	9.62845904	9.969141677	9.794631646	9.123926859	9.831087364
9.080123545	7.766357737	7.994228667	7.692888131	7.522869182	8.041792596
12.09804166	9.792275424	9.805520727	9.151976485	9.642500081	9.756054152
8.473392556	8.289212586	8.390560192	7.311327071	7.820605525	8.110881556
7.248166182	7.611975737	7.605113071	6.652192667	7.167545535	7.347416111
9.929312889	10.62895885	10.1250404	10.97322727	10.87907816	10.15283227
8.328154263	8.373919657	8.304169929	8.045324071	7.816902374	8.207537162
7.982442283	7.881467646	7.937868929	7.174677505	7.34349297	7.945111576
8.206690121	6.819416596	6.874988444	6.494395354	6.680176879	6.900957616
10.25519474	10.30695028	10.35284213	10.81540317	10.58689262	10.57282784
8.594861495	7.759936788	7.817254242	7.942803596	7.673764162	8.430929525
8.77962597	9.425521616	8.512201869	8.057864374	8.62991701	9.430738808
7.44049799	7.520648323	7.390752071	7.095534354	7.439228222	7.802180768
9.316319051	8.521696414	8.66101001	8.634342798	7.622739535	7.940608939
10.2752886	8.897751606	8.650965909	8.611286687	8.815152444	9.149684939
10.19757035	10.33252085	10.38738452	10.57028524	10.49713487	10.19528987
9.388273121	9.465071586	9.669847545	9.912769303	10.18762619	10.31597323
11.96636849	12.44611991	12.19398134	12.63102018	12.71142619	12.66627862
10.51174846	10.03983393	10.07670007	10.19418334	10.12909315	10.16743096
9.702015293	9.424985495	9.458377051	10.00002479	10.04776272	9.841562192
12.13538265	12.80949032	12.75784116	13.22730043	12.94367069	12.90783816
9.237664879	9.266794162	9.501970121	9.411394242	9.617464455	8.897177899
9.543825273	9.174793152	9.251353071	9.214694788	8.266388465	8.478263182
9.277455131	9.134591697	8.804477505	8.317824071	8.989652131	9.135618929
7.046194354	6.961758566	7.455669899	7.535866141	6.710494808	6.971315
8.975268424	8.304169929	8.199709495	8.651919778	7.696877364	7.676257364
8.203151141	8.154705535	8.294640202	8.418553323	7.196285434	7.673376626
10.1752482	10.92880859	11.17750487	10.53247367	10.81423156	10.93023415
13.69473258	13.96779242	13.95167504	14.10328164	14.010587	13.68884286
7.881102323	7.172411071	7.364257101	6.915665626	6.396397444	6.572402919
11.34202326	7.38862201	7.734170172	7.356007838	7.354199768	8.118473111
8.927565404	8.549517636	8.767872848	9.463745455	8.974650333	9.290785283
11.82346178	11.93178106	11.96947069	12.42943771	11.94979793	12.23769992
8.946596606	9.237664879	9.263233333	9.734703111	9.370832535	9.451851384
14.09521817	13.75520609	13.92894576	15.01206542	13.39037726	14.33555643
8.307893798	8.047553323	7.940254566	8.480158333	8.429130081	8.289599333
11.16888788	12.01673563	12.19086998	11.47127063	11.88649013	11.62059475
6.181440384	6.014747222	6.038145939	6.004879293	6.412381	6.439991313
7.969427899	8.508645727	8.450171172	7.425203606	8.614861051	7.997498828
9.320176919	8.855784909	8.918756414	8.249350414	8.294640202	8.639417101
7.730873535	6.84909404	7.271701444	7.024771818	6.559042364	7.206091434
9.453743545	8.949261586	9.53973704	9.528176495	9.12042397	9.020821455
13.02518615	13.18348162	12.99088515	12.24097391	12.57107442	12.85589012
10.54800525	10.93334198	11.27695531	12.15130062	11.55928848	11.29399858
8.426780919	7.398977253	7.298118263	6.930500768	7.340147596	7.416977434
14.7528858	15.76560628	15.67809237	15.50564301	15.59877501	15.54332231
10.75987116	11.37131374	11.48655657	10.36349344	11.04156144	10.94208941
10.73384189	10.12799521	10.25220538	10.6426412	9.749004051	10.20532801

8.838495424	8.720627081	8.540202646	8.920488949	8.928157121	8.742635939
7.838908253	7.283275424	7.726392606	6.929106091	7.396513838	7.546292778
6.986482212	6.840143455	6.860943283	5.852355242	6.928862212	6.628304848
8.85785699	9.641007131	9.164545343	8.029005444	9.387656788	8.672881101
9.139724848	9.53690398	9.733950273	9.854472677	9.392082222	9.335272182
10.05970796	10.68687892	10.37026232	7.841061444	10.17122401	9.528804131
7.571747222	7.81867298	7.948048111	8.527198758	7.802180768	7.890421556
11.75908761	11.8164662	11.83813795	12.17829278	12.62127559	11.82852227
11.85617239	12.55625498	12.18492446	12.88265743	12.51066587	12.70002263
7.218399283	6.966706414	7.249375465	6.854415313	6.616118788	6.741002212
9.509876273	7.294861071	7.112620848	7.021482505	6.842028788	7.911629535
8.871209182	8.727528626	8.872352707	9.409395929	8.920488949	9.071813909
8.228946717	9.189045596	9.107388182	8.918202434	9.012760899	8.753420071
10.8605042	9.923012889	9.876096838	8.725113212	10.20964764	11.07013707
8.138908141	8.475210818	8.429130081	9.042944374	8.696280707	8.405355091
8.045722828	7.127947263	7.90869301	7.525015414	7.005636414	7.291068525
7.309336424	7.332669495	7.163863636	8.086953081	7.549206232	7.802503596
8.608532485	6.477739737	7.149049263	6.617567333	6.478358667	7.063754707
8.622644364	8.737817051	8.663303283	8.500240172	8.531247657	9.057828929
10.50914282	10.98627861	11.03737422	10.58469265	10.66706732	10.573928
7.020691646	7.391342576	7.236271343	6.481897646	7.105472313	7.11316801
8.665646899	8.140128606	7.836138323	8.467667768	7.909412687	8.359650919
7.126269081	7.435709141	7.596246919	7.070939121	7.104662848	7.111135707
7.204880737	7.345977091	7.47214603	7.709908667	6.680439192	6.82246696
11.30131952	10.38208186	10.5336638	10.68687892	9.242526283	9.713858919
6.945903495	7.514494576	7.909763434	6.504501838	7.780823677	7.220780818
11.79615897	11.84291489	11.77305263	12.27101	12.10135206	11.74670945
7.732919727	7.022558657	7.016958768	7.107390485	6.868352737	7.002421121
7.558798354	8.072924455	7.889381343	7.012571859	7.827906879	7.748914677
8.289599333	7.72538904	7.741791646	8.386133566	7.136586475	7.044743657
11.42787377	11.89876792	11.98196729	10.60863639	10.93646713	11.41605585
8.543234566	8.571676263	7.801006182	8.35752198	8.495447152	8.189547909
11.87445957	9.960976253	10.31997281	10.42062148	9.619644949	9.870931818
9.331636121	6.643064293	8.689808788	7.102982434	6.79225503	7.591410606
8.93638097	8.944409727	8.908935182	8.59942799	7.652632424	7.994566778
8.059423535	8.144161414	8.293769586	8.205133263	8.160644788	8.02698897
7.667962596	8.425847798	8.493653778	8.089360869	6.947784242	7.171874212
15.92931651	14.46162431	14.42673578	13.95167504	14.32804886	14.70594079
16.73234105	15.50564301	15.449005	14.03424604	15.27762024	15.63778652
12.90359001	11.12151875	11.74439188	11.90152869	10.45996909	11.49636188
7.69185196	8.052417212	8.712915394	8.348974465	8.456593071	8.728449242
7.190644495	8.234636465	8.187968455	7.153761556	8.533908051	8.427236737
9.082448343	8.835493283	9.096395636	9.183367525	8.979298788	9.146732444
6.217165485	6.157664758	6.967663404	7.813759899	7.390752071	6.621412929
7.578291899	7.590558081	7.421928768	7.643944697	7.630594101	7.898974697
8.122341	8.029005444	6.72626003	7.574489596	7.156105475	7.241538444
8.760291222	8.66101001	8.966940586	9.850553616	9.185002364	9.202552434
8.069753788	7.706168626	7.375117525	7.373717778	7.608934747	7.583692414
9.983540212	8.383429707	8.840010495	8.679578434	7.651222556	8.459227657
9.471411141	8.147147636	8.307893798	8.254919778	7.42989803	8.523456192
6.542753323	7.059062808	6.972319545	7.309928404	6.189422515	6.417030697
9.320738646	9.005975162	9.121511687	9.374690616	8.241600556	8.560860505
7.443845071	7.802836333	7.319114313	7.514821949	7.911309091	7.231823384
10.58812248	10.2692332	9.332795131	9.079484556	10.16743096	10.3986043
8.492747404	8.724556485	8.977005354	9.062782333	8.869521111	9.586238192
7.854685616	7.993875677	7.980256929	7.919201576	7.36694602	7.556302505
9.480384485	9.835081424	9.976273051	7.156105475	7.134684455	8.643157737
8.648450303	8.915814212	9.276789111	7.328995606	8.499404646	8.180413081

7.353900202	6.924473131	7.55178802	8.161911788	7.20129803	7.654890798
7.344013535	6.815737535	7.041898303	6.696696758	6.267021808	6.369292
8.607626293	9.01707996	8.863920758	7.59498599	8.68840998	8.940763364
10.07388225	9.427546879	9.487680697	8.988577162	9.036569232	9.269847242
10.48897537	10.1250404	10.21975253	9.858738737	9.297353606	9.702758343
8.82789604	7.735250384	8.123132566	7.842052606	7.553468394	9.095162899
16.39933956	16.68963031	16.64557528	17.67797782	16.61084374	16.52993947
7.629344343	7.095808323	7.082435283	6.924744576	6.507609283	6.760820788
5.954034667	6.11854299	7.015941667	6.279014293	6.261563212	6.36310503
9.811898121	10.48659409	10.21054893	10.3380268	10.11246778	10.52689599
7.096955818	5.991022919	6.231781505	6.647161081	6.126072101	6.219635727
6.920433444	7.460903263	7.581982414	6.291307172	7.332669495	7.105750636
8.893968333	8.901935111	8.633848111	9.835081424	8.955359253	8.929384727
8.451947071	8.135177152	7.626470505	7.940254566	8.000883182	7.603389051
7.094132182	7.201570444	6.461008283	7.287539616	7.464044657	7.335252788
7.294284717	7.297796364	7.123473687	7.083314707	6.510064202	6.641378788
11.50632759	9.698181576	10.04963797	9.922126404	10.02821235	9.984347768
7.047573414	6.244028818	6.572402919	6.561106071	6.188910586	6.426421657
16.04217659	14.36999747	14.5513462	14.27397716	14.58095582	14.82007331
8.868452172	8.928828384	8.985821889	8.002717273	8.44047504	9.348735909
9.37540303	9.689783586	9.139724848	10.22865659	9.786685869	9.860587788
10.35917202	10.6313464	10.82664672	9.366312899	10.66465811	10.34626898
14.94534759	15.58150095	15.56435861	14.83117585	15.61926687	14.77222727
8.27861104	8.978204141	8.747892899	9.065009323	8.877161475	8.824851909
9.310205828	9.992988556	10.33146686	8.925792808	9.541747455	10.34830463
11.18636156	11.22600179	11.35080354	11.00754401	10.82034332	10.8405808
8.217667525	7.607714535	7.923048354	8.602379626	8.096525737	8.446641939
6.136373525	6.857741051	6.416786646	7.243557061	6.062343	6.893606717
13.5360606	14.1282639	13.85099976	14.30038792	14.36999747	13.92894576
7.286722061	7.406826051	7.535530424	7.897352323	7.418256545	7.256041222
9.801802283	9.739033131	9.732604394	10.56306707	10.03205964	9.957374697
7.955335374	8.003479939	7.944736172	8.216823323	8.271029889	8.263464364
7.513736343	7.568924192	7.441979727	8.277763646	7.911629535	7.768055657
8.53537201	9.758431737	9.217165182	9.195534384	8.861258687	8.827377505
7.732550404	7.907197636	7.970574879	8.316570081	7.787525596	7.901917293
8.144584758	5.990004354	6.398440768	6.102018232	6.296313384	6.583409828
15.29549959	15.78787594	16.3077943	16.17943014	15.39581235	16.36806566
11.53239215	11.97447062	11.87445957	11.94745099	12.36473556	11.80104424
8.424471616	9.346872071	8.644542606	9.087865687	9.290152727	8.454954939
7.266003808	7.558798354	7.68569196	7.878518051	7.733842657	7.689906808
9.790672212	8.339115859	8.130616515	7.868335495	7.604463141	7.93888704
7.726017717	6.933276949	7.132449434	7.555661677	7.204610747	7.153761556
14.4335752	13.50411017	13.60979651	13.45466248	13.73439002	14.10328164
7.704713596	8.049354232	7.857447394	7.005077596	7.371907727	7.637262101
12.04734694	12.14507673	11.32376318	12.01673563	12.41033676	13.18858184
11.57178154	10.99345288	10.40387449	10.87637444	11.92665931	12.21991364
12.48458958	12.7939548	11.4090051	11.42371373	12.37416009	12.797444
10.25127201	10.35385551	9.665316566	10.30695028	10.60863639	11.11714857
9.737663949	10.34418014	9.945028121	9.485668061	10.28506122	10.43564423
6.184352576	7.393167162	7.290498626	5.929579768	7.28582396	6.14156601
8.092691677	8.806450121	8.625486818	7.055055273	8.507703202	8.381309202
9.692793657	9.702758343	9.616057222	10.25320711	9.912769303	9.721958051
11.03737422	11.89361272	11.58621592	11.13713447	11.75662781	11.27695531
7.380377788	6.61398898	6.743297485	6.403910828	6.157827909	6.397600616
7.451812333	6.980492495	7.211808657	6.945089283	6.600799566	6.691780525
15.38120792	16.52993947	16.4534382	16.04217659	16.12384164	16.02065153
13.6823979	14.1904887	14.10328164	14.28325373	14.49002723	13.88591407
9.58708204	9.690534253	9.29794999	10.19136651	9.985290333	9.758431737

13.26276274	12.57107442	13.04381444	12.48458958	12.48458958	12.94367069
9.645545465	7.402015111	7.396823121	6.878162091	7.040256253	7.297158313
6.997069465	7.665645141	7.222960121	7.365381232	8.082778869	7.97284099
6.41609998	6.450135586	6.427081586	6.625506687	5.936577404	5.978649424
8.598008343	8.136641818	8.058246657	7.916025434	7.227000899	7.390207394
10.02061657	9.929312889	10.00361533	10.33146686	9.998242414	10.25627793
16.14978837	15.90458538	15.90458538	16.64557528	15.88528111	16.33688632
7.737649848	7.064538818	7.373717778	7.930919909	6.959689121	7.020439899
16.68963031	14.47046648	17.49517975	17.86179439	17.43906183	16.14978837
15.97330865	15.72204116	14.90726461	16.73234105	15.52328152	16.8710039
8.014589606	8.260123323	8.443175586	8.78664797	8.893968333	8.655934636
16.96059838	17.07010739	17.23317693	17.01900663	17.12376032	16.68963031
9.433849505	6.323721596	8.568242293	10.90606985	6.647679424	6.784856394
10.04682541	10.31378621	10.2761618	8.947127939	9.704210374	10.13580598
7.329290475	6.912275354	6.876372364	7.170490939	7.359577545	7.32338401
7.489176	7.622357354	7.592388485	7.370950798	7.118205404	7.424346313
8.288815495	8.59026096	8.845226141	8.618302869	9.37540303	8.870056939
8.363862202	7.784199556	7.817975838	7.770465818	6.83799099	7.131863879
11.22294817	11.88375452	11.69440164	10.08799683	11.94234713	10.88166557
14.31917195	15.52328152	15.15443825	15.22815255	15.29549959	14.58095582
17.86179439	17.67797782	17.86179439	16.96059838	17.67797782	17.86179439
17.49517975	16.73234105	16.61084374	15.81050193	16.25437967	16.42659037
12.25572441	10.69470446	11.17910752	10.92880859	11.02403513	11.3733012
8.85518403	8.93587504	8.843670869	8.85049999	8.314398727	8.115437899
7.959488687	8.510413414	8.478263182	7.323124384	8.570262	8.043818737
6.841827051	7.193571	7.144299071	7.428935273	7.803193929	7.091328697
9.797856576	9.928455091	9.928455091	8.773471869	9.528176495	9.286110869
10.66105788	9.907546465	10.45628541	10.68960982	11.04316622	10.21975253
10.98037262	10.93789932	11.16423752	11.03452834	11.00620373	10.70534521
7.000811505	7.770819434	7.906425364	8.193428737	7.196057152	7.374563525
12.54268587	11.26990549	12.16627503	12.86086109	12.32659908	12.44950322
11.11560115	11.94745099	12.09225922	10.68572341	11.671419	11.66934136
6.708053838	7.174677505	7.159962485	8.238629646	7.277849899	7.371907727
8.317824071	8.574674323	8.48093102	7.742830394	8.151247717	8.457489293
9.925226333	10.26224218	10.51886057	10.59685822	10.55602586	10.48432644
7.465665798	6.737600111	6.884060404	7.536878778	6.962822313	7.18283101
7.503692152	7.330192545	7.258984768	7.26819499	7.711523556	7.762451808
7.647332323	8.026630869	7.310482798	7.529686949	7.358963616	8.036387556
12.65104366	10.35699559	10.67305197	10.67815746	10.58935103	10.8254891
17.58093558	17.36803328	17.12376032	16.82803465	17.29704085	17.1822602
9.377330121	10.2166926	10.09161001	10.46664928	10.57649171	10.05320582
8.29635502	8.451947071	8.801904485	7.363924808	8.462637525	8.437898414
10.08145981	10.34514895	10.3548828	10.5041569	10.07670007	10.24316974
7.488273475	6.051732566	7.890755525	6.485516717	7.584965596	7.339519202
9.641787333	7.727011939	7.203497747	7.809338131	7.141146879	7.548285091
8.259704283	9.474605101	10.47370876	9.398445515	9.330464051	9.636296737
8.186373323	6.713757434	6.698319919	6.515007141	6.635354444	7.116341071
9.104371838	9.039454747	9.135096212	9.151431899	9.484840202	9.55462702
7.539167263	7.399358222	7.165793091	7.865213	7.449265303	7.144029273
11.4481816	11.6295292	11.62290433	12.28693608	11.69440164	11.79405389
6.547549889	6.684834222	6.899979889	6.833840465	6.84678303	6.612991111
8.430534091	8.487847515	8.094989869	8.648914657	8.265130232	8.425847798
8.873520545	8.844262616	8.889922455	8.515350859	8.724013404	8.753927596
8.313601758	7.659186848	8.009440313	8.659649677	7.726692222	7.511268929
9.287819051	8.852092202	8.890373556	9.412634061	9.373397364	9.591816061
11.80104424	11.81887725	11.28207759	12.03912361	11.81018832	12.15752365
8.284073869	9.060017677	8.702074263	8.995584808	8.924680667	8.85308204
7.813414727	8.376150616	8.170129495	8.582147707	8.371674091	8.53628698

11.60680648	11.64848719	11.82346178	11.80791686	11.78725933	11.88909817
9.459664354	9.69725304	9.374121768	9.629839465	9.717365758	9.829822242
10.37467131	10.3810842	10.52585606	10.97896732	10.83100199	10.65246846
8.994589434	8.567346869	8.561789232	8.805485081	8.578393657	8.740195303
8.990220404	8.712341879	8.319524414	8.745474636	8.615838	8.708467242
10.09896607	10.13193078	9.759931222	9.86223396	9.882779162	10.08325108
8.743124222	8.972363838	8.612685364	8.961854929	8.780633818	8.909945455
12.79024082	13.14093405	12.82558839	12.57107442	12.47761486	12.62807191
9.234418071	9.27419499	9.342353505	9.545191596	9.660200838	9.334658071
12.30167305	12.50706636	12.57877126	12.7452022	12.95787308	12.30789527
8.241600556	8.494982343	8.745043505	8.156772778	8.549141545	8.549517636
12.57107442	12.71142619	12.67378338	12.37416009	12.32964397	12.38063147
9.853774707	10.26832521	10.37686258	10.5431016	10.62647628	9.996420616
7.91465098	8.268052495	7.98506802	8.331458747	8.491049758	8.485174677
9.607593848	9.750460616	9.891739697	10.05688349	9.968215758	8.921011566
9.354289121	8.765233303	8.806450121	8.594016788	8.372772768	7.679285535
10.63014735	9.886762101	10.2600862	10.89604789	10.0406788	10.81423156
13.55640443	13.60979651	13.41514643	13.95955505	13.40302459	13.61616406
6.473192182	6.880775747	6.720840657	6.288561919	6.582941808	6.82197003
8.211847566	7.966054535	7.522869182	7.662615848	7.822730929	8.234636465
11.21958266	11.00053539	11.33477589	11.94234713	11.26815786	11.51008986
11.68038318	11.29947244	11.35475345	10.49241362	11.49636188	11.5571076
11.07956266	11.36423723	11.29399858	11.46320141	11.77053658	10.88546605
12.30465105	12.84197228	12.93862501	13.06967433	13.27418186	12.67378338
10.50677257	10.5029511	10.53247367	10.80389925	10.55726192	10.84176532
14.4427031	15.13975511	15.05177986	15.05177986	15.22815255	14.79546777
10.39968902	10.58236152	10.71390019	11.06573849	10.2861211	10.71031814
8.197369283	8.988033424	9.336479586	8.354182626	8.778623323	8.453185455
9.599465606	10.19757035	10.51771241	8.166602566	10.15086094	9.530205505
13.22730043	14.39736901	14.62217632	13.39037726	13.95955505	14.46162431
14.78379092	16.39933956	16.12384164	14.40789433	16.33688632	15.95256028
9.281446566	9.456392222	9.180906778	8.628622212	9.39974104	9.222631263
8.697732283	9.193130899	9.009915859	9.388912091	9.575401111	9.388273121
10.42409385	10.42736719	10.65365596	10.52135963	11.21816154	10.71279176
12.58287167	13.27418186	13.34730616	12.96241124	13.52334642	13.6823979
7.568310444	6.953914061	7.041650374	6.685554869	7.605113071	6.894957566
7.832217929	7.288414848	7.389236737	6.788076343	8.03179299	6.898538505
7.390471152	7.68569196	7.152088687	7.858612495	6.636479121	7.728706424
8.840590677	9.183367525	8.687969051	9.343457394	8.740682	9.751885293
11.2518391	11.7894918	11.82852227	11.60888892	11.62290433	11.37490296
12.52130815	13.08418191	12.87421343	12.90783816	13.26910509	12.74093701
9.734703111	10.13671639	10.13193078	10.88546605	10.74819911	10.55474917
10.64738653	11.02851208	11.02105469	10.63752253	11.77552743	10.1309568
11.3733012	12.10414281	12.01191659	11.91912022	12.16942168	11.65455063
14.84412351	16.28343863	15.76560628	14.39736901	15.99508933	15.65613201
14.30038792	15.74256723	15.26374094	14.32804886	15.36066179	15.27762024
14.58095582	15.65613201	15.41210544	13.83772389	15.67809237	15.39581235
15.36066179	16.82803465	16.68963031	15.32551587	16.64557528	16.28343863
8.096525737	8.892401576	8.663769939	6.806487737	8.809951364	8.348543172
7.664565879	8.72650097	8.756396081	7.873377879	8.329784131	8.460053242
8.29927897	8.473802646	8.030614293	8.042161798	8.168196313	8.279889293
12.65472241	11.23280633	11.59870577	12.3543152	11.35858946	12.00644476
8.978723121	7.71023803	7.257525758	8.263464364	7.26025397	7.604109192
10.73132595	12.16036216	11.61814895	12.04160066	11.96636849	11.73972102
11.97660652	12.61449239	12.77454263	12.95787308	12.82558839	12.58957491
10.11051676	9.197870838	9.638309758	10.16546821	8.799348343	8.842639202
9.738398616	9.849671929	9.829822242	10.66587479	10.28795108	10.52135963
7.394027687	8.041332778	7.703321374	8.151247717	7.877119465	7.75371804

9.036076414	9.412044242	9.421059	10.41846051	9.847335909	10.20452777
11.66934136	11.8358519	11.31661678	12.13538265	12.13538265	11.04156144
12.18142186	10.27338352	10.10631712	10.53737043	10.35809493	10.81280665
11.19447199	11.89628941	11.85033795	11.89876792	11.76101506	11.31661678
6.789988131	6.807771525	7.202978657	7.158043384	7.436640343	7.022261949
10.65871503	10.93646713	10.99046505	11.58621592	11.44491584	11.46539851
10.20748734	10.41530743	10.52689599	11.17041501	10.92608881	11.0387546
6.537080394	6.740256788	6.685804808	7.690510313	7.148774485	6.67332503
10.18942995	10.53028115	10.53623255	10.95849458	10.78014639	10.7982674
9.004398101	9.370225242	9.597513697	9.230202616	9.721175788	9.823678374
7.950449798	8.088117667	7.787525596	6.526565121	7.917722121	7.394027687
9.877792838	9.560346242	9.478383576	10.34514895	10.11452638	10.06547375
9.759214424	9.876880455	9.872596081	10.7442872	10.23550694	10.40387449
7.042643192	7.187484444	7.209785606	7.807594768	7.675893758	7.482605354
11.26990549	12.09497108	12.13867659	11.46936418	11.71443529	11.38970236
8.685201879	8.452737606	7.935081758	8.468064667	8.816214848	8.911109919
7.836828747	7.436640343	7.473129747	7.415639222	7.462771485	7.711523556
9.70034896	9.444057586	8.986432162	10.4524716	10.81664077	9.792275424
9.190205293	9.655770101	9.635582141	8.130283051	9.592426051	9.167758485
6.466965869	6.295706747	6.137864596	6.680656707	6.905316545	6.489542818
9.254890202	9.162016475	9.403514808	9.429506515	9.21067496	8.980943495
6.720058788	7.282065253	7.719835253	7.60282502	7.263859848	6.757434848
6.810023535	6.392953667	7.032840081	6.278635202	6.341087596	8.063461202
6.429848434	7.237499505	7.272599475	7.667309091	7.138578667	7.044506424
9.844817354	9.912769303	10.31480319	10.32946621	9.789981192	9.577319545
8.754900343	7.763186	7.991026727	7.623059667	7.091827414	7.431389424
7.137721343	6.959689121	6.899721758	6.817048798	6.721088758	6.649399919
8.228510596	7.619228051	7.675499444	7.795765162	6.99155401	7.494168788
6.236816515	6.128300636	6.264838313	6.804991424	6.69036997	6.173448717
10.51400776	8.373157505	8.507211606	9.618256808	9.698181576	8.664254273
6.627578354	7.130177747	7.168942192	7.56165098	7.117115202	7.133813545
11.60479972	12.10135206	12.18142186	12.15440913	11.75215821	12.12151457
6.830856061	6.824345162	6.60386102	6.421175192	6.014327434	6.359365273
8.638487899	9.150811232	8.975268424	9.632768747	9.447991384	9.599465606
7.646275747	7.495088838	6.857241172	7.241229071	6.812413525	6.648141899
11.11230451	12.17513939	12.00086001	11.43208235	12.27101	11.40145091
10.6313464	11.67371856	11.27499716	11.84291489	11.79615897	11.69188536
6.496001641	7.136586475	6.839237455	7.054536222	7.153495505	7.125696242
11.55928848	10.92337564	11.07647857	11.71659127	11.48655657	11.63629658
10.1684149	11.1060681	10.73911267	11.05191968	11.33477589	9.899969596
8.057421596	8.038354192	8.043387838	8.837022465	8.676316919	8.314030071
11.77305263	12.5521346	12.66985986	11.78498903	12.17216641	11.98440811
9.923012889	9.201355202	9.605576293	9.242526283	9.000672535	9.258612929
7.029897788	7.039966242	7.10439698	7.068134081	6.384961778	6.560443758
8.907429495	7.243824414	7.117905152	7.171589687	7.157253707	7.108492263
9.223885747	9.70034896	9.507772687	9.963504081	9.908305848	9.881176566
9.600888859	9.265583747	9.560346242	9.910074283	9.650129091	9.544495798
7.431765758	8.516793455	8.583542788	8.69919198	8.363019899	8.460488909
10.91903182	11.48840455	11.50814936	11.93975968	11.49029795	11.79870316
10.07670007	9.477759242	9.636929838	10.22164118	10.05144087	10.01524205
9.798623747	9.848227263	9.786685869	10.4687107	10.0079343	10.02061657
8.523871909	6.757884566	8.417196576	7.513736343	6.45473303	6.965182525
7.146069808	6.563853182	7.082724758	6.896238273	7.142895121	6.237753616
8.05903404	8.474658303	8.257595869	7.747112485	8.178694051	8.039790061
9.838413929	10.83790729	9.521972354	11.53677901	8.563501616	7.812327374
8.192266657	8.433042727	8.643636162	8.104133364	7.874063444	7.953840929
11.00053539	11.28053313	11.07013707	11.62494396	11.2608846	11.32376318
7.750957677	7.600942677	7.476910626	8.238272808	7.946541949	7.927113394

7.186327495	7.306475687	7.365381232	7.938531869	6.824533111	6.873999848
14.46162431	15.42966281	15.38120792	13.36582475	15.21350605	14.91973608
7.283800475	7.510343899	7.595597162	7.74678301	7.533296202	7.316719848
7.77977198	7.221345788	7.783465061	8.607126586	7.557268586	7.641344242
9.97079498	9.973623707	9.897493586	10.44392641	10.12697515	10.21484784
8.285708747	8.464558212	8.387083515	7.177458596	8.574674323	8.371674091
8.656835556	8.777572838	8.84935699	9.349388778	9.693515768	9.420326576
6.650783061	8.161033919	7.811371919	8.966940586	8.378515131	8.452374444
13.92894576	14.90726461	14.77222727	15.02501841	14.77222727	14.37933382
9.115691495	9.123926859	9.204855576	8.841037687	8.543234566	8.397394545
14.010587	12.87421343	12.8293171	12.10135206	13.02518615	13.00581854
8.728449242	9.271123182	9.551451798	9.041236566	9.372705202	8.830660505
9.793126455	9.627013505	9.556759333	10.1752482	9.742174424	10.03611568
10.87518762	11.44643795	11.28370047	11.5737214	11.24001011	11.5737214
9.174793152	9.36748	7.663618646	10.18762619	8.947127939	9.877792838
13.91489381	14.58095582	14.4335752	14.24162707	14.84412351	14.04335209
8.231756293	8.788663253	8.645541747	7.85710597	8.131433848	8.468418253
9.098746404	9.683334444	8.708995576	9.070661273	9.329922273	9.848227263
7.25519196	8.552719747	7.387965737	7.274379929	7.742436303	8.211847566
9.482964828	10.53623255	9.675904202	8.865915939	9.009915859	9.822898081
8.687541707	8.909404788	8.564847253	8.732044667	8.648450303	9.224452333
10.29432836	11.82346178	10.45877561	10.04682541	10.64502212	11.70348036
10.1597097	11.14385742	10.72384747	10.06547375	10.49140257	10.92485902
8.831201768	9.948421707	9.382882596	8.788663253	9.263851586	10.25811169
8.160644788	8.79722998	8.371229848	8.15355797	8.539328929	8.810986707
13.30255942	13.81266894	13.97489728	13.06488572	13.44849295	13.85817003
13.62106704	14.34357305	14.7528858	13.09463792	14.07245549	14.06476988
13.03025597	13.76108056	14.1904887	12.58612865	13.31405593	13.52334642
11.6157426	12.06397065	12.44264271	11.02551486	11.57178154	11.88148631
17.01900663	15.16803204	15.29549959	13.76702458	15.09418019	15.16803204
6.718779485	7.132449434	6.955873859	6.919083737	6.32572904	6.096608202
9.07526898	8.886620687	8.783584889	8.968391182	8.643157737	8.719245818
6.32793802	8.087781242	7.791244131	7.533296202	8.115821848	7.339214566
12.50706636	12.75784116	12.70390423	12.23769992	12.87819387	12.49170848
9.705000525	9.729555121	10.17224075	10.16454544	10.2761618	10.22668357
9.661659101	8.733447455	8.942756889	9.135618929	8.751405818	8.971727333
9.644080808	8.298396535	9.161459798	8.886096818	8.396125929	8.569209263
9.186096828	8.964642636	8.917682242	8.993505475	7.697940283	8.256076333
8.08737399	7.931708101	8.359650919	8.188779434	7.581982414	7.980568657
8.062539303	8.837022465	8.562227051	8.144161414	8.758871	8.949261586
9.648698737	10.52799495	10.71279176	9.802496737	10.5029511	10.18563382
7.856391758	7.977952253	10.07580535	8.447076788	7.422224808	8.235388636
9.08959502	8.77962597	8.368326869	9.580097263	9.174793152	9.180906778
10.1985269	9.62024901	9.389466253	9.811898121	8.679578434	9.250125475
7.630934212	7.490096869	7.05790099	6.940977263	6.986217889	6.973893828
7.060219081	7.545335788	6.674272111	6.877148152	7.126519737	6.374400737
6.813695162	6.59116396	6.330092323	6.244753081	6.552098808	6.304770596
10.47584162	10.47941256	9.607593848	8.721926778	10.2600862	9.844817354
7.23119303	7.02555202	7.318843495	7.918105152	7.084738172	7.455517596
9.913634949	9.518503263	8.742635939	9.757590192	9.782133848	9.297353606
7.500795283	7.421318101	7.055635202	7.174922929	6.878694758	7.291703707
8.538380869	7.371573384	7.342871717	7.440763838	7.44169298	7.418256545
7.230900293	7.553468394	6.958444212	6.973155374	6.961028939	7.012842909
8.289996192	8.066736566	7.475313929	8.793874616	6.977817737	8.045324071
8.389243343	8.655934636	8.449337667	7.814093232	8.145194646	8.130993828
9.637663091	9.650789909	9.212349869	8.864955909	8.961854929	8.966282283
9.505258828	10.40925212	10.43757507	9.182731869	9.886023253	9.95201103
7.241538444	7.764609818	7.617189485	7.632188616	6.673543687	6.832629131

6.525595273	6.804991424	6.716586707	6.121897343	6.87215099	6.614995778
9.554043101	10.06904603	9.815129384	8.906407303	9.778131475	9.513795939
8.132293172	8.496814303	8.511758747	8.663303283	8.915814212	8.060661899
8.641223283	8.016751283	8.179120646	7.898205273	7.475616394	7.869396606
10.20859114	10.7814083	10.15483313	10.12697515	10.74690378	10.34193651
8.57070398	8.396532182	8.629474212	8.449722687	9.125507586	8.683087172
7.758641747	5.910561061	6.753729404	6.28178403	6.519436343	6.609208404
12.0089388	13.04869312	13.06488572	11.28731295	12.87421343	12.52461444
7.659186848	6.790317606	7.235967596	6.595947525	6.513659232	6.669467525
6.796566808	7.028039657	6.961028939	7.241538444	7.300950758	7.397472212
10.6240143	11.91912022	11.89130603	12.01956714	12.34855768	12.20960187
10.4687107	11.03737422	10.83642757	10.81034167	10.90466263	10.56655729
10.52799495	10.39296715	10.12224713	10.25038968	10.61480751	10.12697515
7.329870525	7.438935576	7.051937434	6.815473091	7.359287172	7.26819499
8.807959646	8.645985424	8.235388636	8.645985424	8.606624646	8.567346869
8.816214848	8.532516465	8.832291949	10.09079019	8.929384727	9.195534384
10.15483313	9.173615515	10.3494333	8.638487899	9.285586323	9.925979414
8.120062485	7.796085586	7.374563525	7.347416111	7.123473687	7.196285434
9.832711535	9.489614919	9.054276	10.17613521	9.992117576	10.27714677
10.3986043	10.04160563	9.916104475	9.004398101	10.03796113	9.93663897
8.403471909	8.243637242	8.209054152	9.428190232	9.128533354	8.732044667
6.494595232	6.355908172	6.728207192	6.166487869	6.741723879	6.791029141
8.172571899	8.079179394	7.74678301	7.737346818	7.833640626	8.415109929
12.71142619	13.52959744	13.56886427	13.87263802	13.65450155	12.70787607
6.396609343	7.022261949	6.814975071	7.25118401	6.195417717	6.154670293
11.52824	12.51066587	12.53563192	12.31419772	11.98999321	11.911688
11.62059475	11.75908761	11.78725933	11.42562271	11.46724679	11.78498903
7.989899475	6.148519293	6.231976475	5.813789717	7.560118414	6.945089283
10.96748398	11.14707085	11.48459908	9.918671212	10.99623862	10.76239578
10.35385551	10.87518762	10.761049	9.748306121	10.92485902	10.44602022
11.50261564	11.96059797	12.01956714	10.74305691	11.89628941	11.68243664
13.46005067	15.63778652	15.48449671	15.46469304	15.10875812	14.7172184
9.360502949	9.954681949	9.76546198	8.580722586	9.611261414	9.440510152
7.107390485	7.32338401	7.322879626	7.992269263	7.626793707	7.540776061
9.096947232	8.681067818	8.863375313	8.476485121	8.366003535	8.709941838
11.31661678	11.72592113	11.70557621	10.80091358	11.72355631	11.39382595
7.426469556	6.787317758	7.166060222	6.161098354	6.180964455	6.376277596
8.51269104	9.687048303	9.596088263	9.529469889	9.271123182	9.01707996
6.36310503	6.306085808	6.99155401	6.809755232	6.816827283	6.635568162
8.023099172	7.782178323	7.841729333	7.731201121	7.066192545	7.398370889
6.781434051	7.219933525	6.982384606	7.134092121	6.554351	6.506514626
9.072998758	9.233272283	9.091299889	7.686071172	8.794788636	8.519178475
7.517772121	7.705814323	7.417598061	6.292304869	7.425203606	7.070939121
8.479727162	7.381027636	7.714642404	8.295436051	6.817799576	7.375117525
6.358115374	7.225864141	7.212654808	7.769805091	7.216692263	7.474397404
7.258107788	7.214035101	7.230593889	7.698638758	7.453291162	7.427999919
8.39402401	7.603101596	7.633913808	8.276014121	7.947506525	7.992269263
10.23948175	10.79284767	11.07337622	10.88681216	11.09565519	10.68338253
9.267383556	10.05780386	9.137373131	9.454390232	10.05523038	9.565660949
8.567801737	7.762120596	8.038729465	7.975072535	7.345977091	7.59859104
9.795448596	9.306330646	9.35796903	10.24316974	8.817191758	9.696433626
12.09225922	10.8254891	11.02551486	10.92485902	10.53623255	10.82804231
7.641985	8.067132556	7.79851096	8.232163879	8.071334404	7.784888444
11.38577951	11.14057221	10.84844593	9.965319232	11.23641801	10.97896732
10.13671639	7.777311636	7.736260545	7.75479003	8.149032212	8.539328929
9.008956374	10.33375706	10.05607537	9.876096838	9.710962273	10.30151978
8.453185455	7.687096788	8.500240172	7.938205586	7.808339242	8.558108202
9.084401768	9.620873889	9.486974667	8.800782677	9.335891293	9.547339828

6.235536455	6.509391576	6.375508101	6.811363364	7.246435697	6.678316576
8.202220545	6.577737222	7.962004212	7.372532788	6.468260505	6.887288828
10.54078574	8.469339576	8.679110091	8.546056747	8.791657869	8.610845737
8.782655051	7.576363505	7.670902747	7.229324455	7.229324455	7.400314747
6.42732104	6.276608434	6.599129636	6.636058242	6.173981626	6.436392303
7.691535778	7.017519192	7.223533071	8.374363061	6.907093253	7.274888343
9.812775374	10.19957169	9.870221061	10.67572435	9.969141677	10.2761618
11.9875501	12.30465105	12.07810634	12.61449239	12.54589185	12.3843369
10.87235819	12.23191382	11.29230964	10.43122177	10.93789932	12.27101
14.1282639	14.66395575	14.35197184	14.52085241	14.5108222	14.66395575
8.71485401	8.035288182	8.116928465	8.012010707	7.169469697	7.280525364
6.06334998	6.240680768	6.47989397	6.305549929	5.990876141	6.128938879
8.10683704	8.025864485	8.262181081	9.156840283	9.053706202	8.854095293
7.865908465	7.123473687	7.274095485	6.935824606	6.935824606	7.073657889
8.918202434	9.59387599	9.387059283	8.391020323	8.804477505	9.379180747
8.135526051	8.553595747	8.177906263	7.456765576	8.124599212	7.897743687
8.708467242	8.578858313	8.798247798	8.911109919	8.411285283	9.070117818
9.505859384	10.1752482	9.784374313	8.588536172	9.977292899	9.588958727
10.60743282	11.04316622	10.99469779	11.29230964	11.18941159	10.49241362
10.10736711	10.52135963	10.51174846	9.837575172	10.85923156	9.707282667
8.486053202	9.014846778	8.956407626	9.661659101	9.675904202	9.556000646
8.407442505	8.572701232	8.86638896	9.373397364	9.146178758	9.339095222
13.18348162	13.91489381	14.05136004	13.89331098	14.24162707	13.76108056
7.030688768	7.718747404	7.692888131	7.426469556	7.563439404	7.455822202
7.508747081	7.356281616	7.819446808	7.834378071	7.839249838	7.843485626
7.722652869	8.52305899	8.439612798	7.738613697	8.232163879	8.214678465
12.63102018	12.27421104	12.0724233	12.11216258	12.30789527	12.51424883
5.997922384	6.285113455	6.936097616	6.03107604	5.801733202	5.826032343
8.335861768	7.802180768	8.173308798	8.717867636	8.074508444	8.498503737
10.54565144	8.678158051	8.896578162	10.34717129	9.17294002	8.221010253
9.045755677	9.499917182	9.542425758	9.624391323	8.880853535	9.566949
12.63500423	12.21991364	12.43600068	12.59323492	13.04869312	12.58287167
7.261502495	6.934568707	7.009733586	7.219017232	7.851994586	7.380377788
10.17613521	8.948168879	9.812775374	10.32750324	8.808345061	9.145664475
6.521008828	6.862895808	7.161069364	7.579350515	7.491688212	7.18832599
8.654464586	8.549141545	8.170586434	8.060252263	8.592161788	8.653441707
10.83790729	10.69931975	10.57282784	10.20452777	10.73132595	10.1985269
6.328112374	6.930500768	6.556698131	6.737600111	6.240116697	6.192299
10.99469779	10.08893073	10.58689262	10.3494333	9.76546198	10.16073454
9.644739414	8.055171202	7.795765162	9.238865747	9.248396293	9.212980727
7.985458788	7.621699758	7.771540485	8.315227333	7.963371182	5.906078172
8.911109919	8.379056919	8.177528808	8.352537101	8.178268222	8.784105778
10.69793218	11.45002291	11.38391359	10.57649171	11.45932673	11.19447199
6.881752616	7.708578687	7.474688838	7.154937303	7.599870798	7.334639071
6.387499121	6.374400737	6.441889697	5.755594273	6.588475747	6.040474222
9.856174394	8.561789232	8.78214798	8.633351606	7.715405939	8.531247657
7.288727879	8.339926909	7.844877657	7.954939636	8.268052495	8.041332778
10.09795649	10.54441943	10.70775781	10.31289083	10.75453103	10.25320711
12.11562205	13.01016522	13.50411017	13.30255942	13.05428976	13.15645152
7.062195576	6.893606717	6.712828707	6.488853636	8.32939098	6.664178717
6.985416596	6.391874798	7.219017232	7.302140758	6.760820788	7.091827414
6.953109798	6.891782758	7.242384899	7.424653535	6.798121798	6.747571626
7.465036929	7.651593505	7.58864003	8.150892606	8.00874802	7.89464897
8.710842768	8.692466525	9.271123182	9.584100455	9.093526566	9.19736601
10.35591393	10.80499832	10.72516317	11.00496707	10.9660467	10.49140257
6.733937384	6.000571626	6.534227879	6.259517909	5.978026141	6.30015496
7.930615646	8.343220798	8.353342091	8.741130788	8.368780919	8.649944576
7.021742939	7.342871717	7.358075697	7.014842626	6.524057606	6.710717586

10.15783547	10.04340808	10.19528987	9.959124273	10.07388225	10.02362526
7.820934747	7.420458273	7.199434576	7.319616859	6.86214797	6.73278298
7.916025434	7.286993737	7.091592101	7.737987747	6.759458	7.13936198
7.534302667	6.075380768	6.805984869	7.070666535	6.225711566	6.533325687
6.338442758	6.102684848	5.810341747	6.151324646	6.368932879	5.830914333
10.98492733	11.12315281	11.8164662	11.29744903	10.72900488	10.88681216
10.11941657	10.49009312	10.42409385	10.54441943	10.61371787	10.40810668
5.748915707	5.817790192	6.121104576	6.025445606	6.06447001	6.490634808
9.335272182	8.362142131	8.518658667	8.496814303	8.158602879	8.389243343
6.929929141	6.948873394	7.037826263	6.929631566	6.570036263	6.637264
14.29173122	12.63888703	13.20018245	12.54906863	12.73731843	12.96241124
6.432817071	6.561721071	6.69201996	6.913266485	7.256596263	6.55251301
7.287539616	6.928307859	7.040256253	6.997949788	6.314167232	6.471263556
9.53973704	9.940265465	9.49850403	8.990775909	9.183367525	9.271759586
11.08435313	11.24816417	11.29076429	11.34576518	11.39594259	11.19817469
12.91656586	11.43938222	11.79405389	11.86401838	10.65500785	11.11856721
8.714332636	8.108523939	8.400903717	8.069395848	8.004282899	7.993428586
8.310875455	8.380307172	8.658285303	8.078774939	8.980943495	8.817758687
8.518144394	8.076845152	8.052417212	7.477877475	7.683943101	7.848096879
6.786032343	6.232346939	6.481897646	6.253125879	6.42554104	6.402667606
7.678291495	6.352246303	6.460074192	6.652872152	6.443449727	6.439789939
7.760294414	6.432225313	6.597017333	6.867884374	7.107390485	7.075225141
6.474296596	6.124663283	5.79490397	6.690572232	6.180045333	6.604509566
7.223856	7.738613697	7.779041818	8.040575909	7.975784071	7.821271606
10.27338352	10.39414857	10.47479655	9.843921515	10.4700758	10.36349344
11.04156144	11.9091429	11.76588718	11.88148631	12.00644476	11.74439188
8.695776131	8.150448232	8.314850485	8.175968162	7.634226727	7.881102323
7.625433667	8.670884566	8.752460404	7.554702939	8.554971	8.344441616
10.57282784	10.8778129	10.74045328	10.90466263	11.05959848	10.20060626
9.682548525	9.015966535	9.132080535	9.613206141	9.393335687	9.47593803
8.494982343	8.063008152	7.516753121	8.503920141	7.725703576	8.038729465
9.344138121	8.977558859	9.039989364	9.468772697	8.05903404	8.708030737
6.631584848	7.062438556	6.903241343	6.286294667	7.082160636	7.060512141
7.276013455	7.58642403	7.39712799	6.529766576	7.46750399	7.274095485
9.772250768	9.372705202	8.766842081	10.01703805	9.759931222	9.673359929
7.966742939	6.995800606	7.28497204	7.51061902	7.029897788	7.797844434
9.012760899	9.609027141	9.378583414	8.682101313	8.76478299	9.207219232
7.237838192	8.328992859	7.987188889	8.313601758	8.386133566	7.968691808
10.04776272	10.06633871	10.11349335	9.426142444	10.06904603	10.17789322
8.165787747	7.715785354	8.194244232	7.740469253	8.343220798	7.979092162
7.524403394	9.570470929	9.961780586	8.261007747	8.878776172	10.03492192
7.647631172	7.504860717	8.031407364	7.981709394	8.149448323	8.381754576
8.656360697	7.983548525	8.03179299	7.991026727	7.686071172	7.757833798
12.60396528	10.86305205	10.98627861	10.50914282	10.97054498	11.05796986
7.409335747	6.90271597	7.437044505	7.229904545	6.960463354	7.084738172
10.19957169	9.641787333	9.793126455	9.293710889	9.728754818	9.342353505
8.852092202	9.803356646	9.536330707	9.419020677	9.522660222	9.717365758
7.334394303	7.688370495	7.701991131	8.223899788	7.914965525	7.943433747
8.459596535	9.540446515	9.109120374	9.470840162	9.521192909	9.652169778
9.35858599	9.066117364	8.782655051	9.897493586	9.625041525	10.0053333
7.798826303	8.010919919	7.989587677	8.651452677	8.197805768	8.14677703
9.041820404	7.787223354	7.48289603	7.679285535	7.865213	7.926300626
6.917222737	6.305155434	7.341339747	6.856233343	6.334404253	6.445028586
6.842776727	6.397781283	6.35069898	6.899979889	6.093889788	6.206101182
12.66627862	12.39058663	12.05813886	10.87235819	11.43938222	11.87912023
9.129114848	9.746868596	9.517771202	8.47014499	9.658049545	9.280885222
7.484183869	7.843077242	7.466006556	6.833099889	7.585232141	7.405524869
6.376277596	7.152420131	6.906597848	7.202701576	7.285574899	7.384903253

6.850100909	6.847553121	6.534648919	7.387061818	6.887288828	7.382214869
9.524156586	8.111719586	8.415109929	7.761029182	8.066736566	8.519692939
8.692466525	7.94734596	8.10326497	8.059423535	7.18482696	7.572856394
6.656675747	6.308509485	6.448856899	6.510269939	6.895441525	6.658365848
8.930963202	7.790916071	8.463578343	7.783847071	7.767362636	8.128726778
6.668984303	6.214653717	6.048440505	6.155001141	7.004539727	6.724256232
5.905089657	6.654772152	6.515779859	6.704711566	6.161098354	6.063889717
7.880750535	7.556302505	7.352900667	7.635267465	6.915952788	7.269598859
14.02572128	10.52476206	10.69793218	10.04340808	10.36589334	11.28207759
7.658839354	6.544097556	6.448608091	6.918565768	6.379794838	6.649151414
7.637262101	7.207500556	7.534885556	7.766738848	6.862895808	7.633560879
7.730154939	7.227882465	6.936612586	7.697940283	7.616841162	7.692888131
9.23383296	8.899254879	8.917069505	9.312041414	9.154469939	9.452497313
13.17778072	11.36979611	11.76804793	11.7894918	10.57028524	11.28731295
11.21016953	10.20532801	10.30695028	10.43564423	10.29851595	10.33890802
12.14507673	12.99088515	12.69676846	13.09977851	13.13522491	12.59668127
6.590106323	6.929631566	6.923167202	6.848028121	7.331457121	6.824838515
10.57885616	9.591816061	9.924448444	10.05426836	9.773700283	10.11150219
10.16355358	7.740855141	7.960547	8.117339677	7.524103909	8.185563303
7.02555202	6.55251301	6.70133698	6.011815869	6.703493626	7.08136002
7.186028475	7.748914677	7.961295828	6.746340717	7.493254434	7.578291899
8.756396081	7.31258798	7.487351192	8.021984222	7.212654808	7.814474586
8.445297313	8.100639	8.208296687	8.143040162	8.197004869	8.191204879
8.38301802	8.271405859	8.316969364	8.706567556	8.366948404	8.465492313
9.339095222	6.887565899	7.109678444	6.729687141	6.314827061	7.373717778
8.36556403	8.318668263	8.40783399	9.388273121	8.463063475	8.515864333
7.244714616	8.31912796	8.118136869	7.770819434	8.074096707	8.076118121
9.204855576	7.960547	8.378142071	8.218433131	7.441979727	7.808339242
9.987077505	9.152615889	9.191263899	8.778117444	9.002847848	9.210065
8.44129198	8.096910323	7.723384495	8.169730152	7.288414848	7.577325354
9.604175636	9.214111545	8.472435657	10.18468509	7.959830364	8.34982299
10.82945836	10.6362571	10.23160947	10.82290783	10.19418334	11.02551486
7.325162263	7.160791343	7.195783737	7.38862201	7.901917293	8.762202232
6.150288283	7.145493505	7.172125253	6.610658212	8.155393222	8.729972586
6.714000354	6.590449828	6.317278899	6.819920253	7.054536222	7.870669919
7.836508475	8.022720303	7.786626808	8.17219503	8.984231152	9.417037424
7.776955808	7.88825004	7.153217667	7.061925202	7.629016404	8.36556403
8.844732879	8.48742196	7.894965162	8.410908071	8.830117929	9.228437333
7.728361283	8.242002535	7.583998525	7.982067081	8.207926798	8.630379313
6.993764091	7.453623293	7.000309313	6.931701566	7.759936788	7.737987747
11.72807534	12.81735602	12.64708972	11.95261936	12.52819783	12.40735915
8.912452162	9.463745455	9.585510535	9.885156737	9.900778232	9.682548525
7.060512141	7.035678848	7.789881242	7.274888343	7.243824414	6.971045939
10.58236152	10.71279176	10.6768734	9.815893677	10.48782122	10.87518762
13.02006671	13.43523914	12.7452022	14.89443037	13.14611959	14.36070414
10.52585606	10.76239578	10.70643744	11.16734497	10.97185531	11.06732533
7.986161141	6.782428768	6.94531798	6.998984111	6.907322414	7.033365899
7.469344313	7.560118414	7.470886646	7.986535808	6.747331182	6.830079818
5.77191799	5.845132939	6.447271273	5.943750677	5.906381636	5.76707798
11.08264222	11.47127063	12.63888703	11.61814895	11.911688	12.35798465
10.78014639	8.388388636	8.566415323	8.342368121	7.87472	8.108523939
10.76609029	9.153232364	8.088117667	8.371674091	8.29635502	8.868452172
7.423727141	7.833986465	7.76948301	7.612584303	6.336250778	6.758137737
7.045715273	6.931501667	7.154007354	7.36166898	6.264444141	6.532200808
6.247954444	7.910120242	8.392711081	8.197369283	7.535530424	9.892629697
5.767014091	5.782429475	6.896238273	5.826032343	5.792143606	5.819713545
13.09045797	11.22947614	11.84529477	11.65698449	11.15503783	11.69658017
9.255548899	7.765675424	7.366317111	7.622739535	7.097212141	7.422224808

10.00002479	9.057196687	9.038849273	9.044046343	8.841037687	9.162016475
8.213832515	7.788172758	9.022000869	9.296718121	7.740469253	8.085049192
8.772410586	8.686679798	7.645321707	7.495088838	6.981335535	7.202978657
6.992079758	6.166896333	6.244028818	6.444123263	6.218725646	6.298612879
12.24351077	9.250125475	10.70902317	11.14385742	10.00186072	10.74690378
9.921211657	8.755916071	8.588536172	8.303793737	8.397394545	8.526186556
7.08589403	7.086390323	6.321876909	7.030955384	6.35978	6.990260788
10.42622906	9.218466939	9.178084667	9.228437333	9.283145788	9.229670424
10.90754603	10.04421951	10.30151978	10.3779146	8.671783687	9.566253768
9.517771202	8.299695162	8.648450303	8.632349838	7.446082798	7.630934212
9.318836889	8.370830747	8.835493283	8.372086737	7.959488687	9.816676162
8.589797808	8.307483899	8.410410394	8.127495596	7.520992313	7.812003626
9.177504333	8.555833323	8.834473929	8.710391727	7.452739495	7.664957788
6.252758313	6.484023485	7.036210545	6.632781101	6.326506525	6.472300566
8.01423197	7.979449616	8.23581501	8.608532485	8.369612525	7.903688606
7.564402343	7.554402606	7.787223354	7.612909768	7.761378475	8.554027364
8.749346626	9.080683394	8.179581869	7.638274606	8.22479296	8.738283333
8.609106909	7.872008071	7.002890535	8.21550303	7.223533071	7.302736424
10.41530743	8.752460404	9.694925798	8.189895404	8.088117667	8.790654051
8.899254879	7.790242404	7.899682051	7.496977949	6.635568162	7.343750657
10.24414187	11.109559	10.63391466	11.12454893	11.27499716	10.56908161
9.49510903	9.625623061	9.837575172	9.504584061	9.874496818	9.867911152
6.663676879	6.761335818	6.160622323	6.859220677	6.589360121	7.067867747
8.522156747	8.781156657	8.771294657	8.773988747	9.186711646	9.271123182
11.20163629	11.72355631	11.62739451	11.11856721	11.85033795	11.43380494
10.3380268	10.79564384	10.62536143	11.07158908	11.05796986	10.33146686
8.733877859	9.245445293	9.028300162	9.448606737	9.469429253	8.476085384
6.994245051	7.389870293	7.173915838	6.544328465	7.186960111	7.123768636
6.595947525	7.457936747	7.238133434	7.090185162	7.177177071	7.616841162
10.5041569	9.459003	9.497817424	9.452497313	8.962353222	9.224923687
12.83827393	13.64083669	13.56234785	13.07947925	13.6823979	13.48401144
6.918565768	6.577521747	6.495692828	6.521234222	6.140075394	6.531066697
14.61202705	12.88265743	13.10985871	12.7494647	12.71830686	13.20536123
14.66395575	12.04160066	12.21312889	10.71031814	11.87175588	12.04160066
16.90924948	15.29549959	15.31193978	13.92172837	14.99939481	15.22815255
6.705893697	7.019424707	7.420458273	7.451812333	6.77624102	6.895973717
7.022261949	7.50339202	7.812327374	8.095746162	7.431765758	7.57049196
11.59870577	8.919862162	8.279889293	9.249034202	8.897177899	8.579336081
6.876638434	6.808520727	6.945089283	6.936850606	6.543222263	6.77698199
12.93862501	9.756840071	10.20634509	9.942152202	9.84311402	9.992988556
17.1822602	16.3077943	16.33688632	15.76560628	16.17943014	16.3077943
10.76471511	10.67572435	10.29542336	9.709426838	10.31795252	10.68687892
8.10140802	7.158590636	7.023140616	6.543651707	7.398696909	7.338887687
14.05855866	14.88238784	14.6970922	14.34357305	14.74028573	14.31917195
8.517695303	8.213832515	8.266388465	8.405355091	7.071197202	7.33379003
12.45390404	12.58612865	12.76207554	13.66158872	12.48835313	12.92081703
7.384605828	7.678926152	7.391342576	7.695265798	6.874731313	7.002156515
7.190368869	6.942257576	6.839681778	7.354460354	7.381652111	7.358375414
7.166369152	6.977041091	7.061925202	7.915342566	7.371279929	7.310724131
8.861258687	8.563941778	8.846850111	9.207791152	8.823394192	9.109760465
8.349393212	8.292509485	8.449722687	8.39867397	8.450646323	8.644074768
8.725113212	8.705145061	9.281923404	9.224452333	9.550085313	9.650129091
8.125818909	8.466026899	8.383846919	8.672348475	8.448456354	8.484723788
11.65261158	10.17904437	10.25038968	9.93187304	10.15283227	10.31894523
12.35168631	12.54589185	12.65104366	12.25844332	12.77454263	12.49965499
9.918671212	9.251906182	9.295516616	9.831087364	9.080683394	9.064430111
12.17216641	13.03025597	13.99495772	12.75784116	13.30795266	12.60022648
13.20536123	12.21312889	12.83827393	13.31405593	12.46466592	12.82153585

7.491021444	7.398370889	7.283562505	6.745111616	6.71121296	6.925009455
6.56887297	6.989715687	7.969046636	7.243824414	6.756883091	6.782198505
7.272016424	6.629250202	6.996297444	7.028313949	6.455606303	6.668064172
8.181963182	8.546982556	8.598479657	7.411868313	7.983548525	7.967113929
11.05651949	11.79870316	11.65052704	11.04030995	11.25523353	11.33477589
8.030983081	7.347416111	6.99955204	7.203245626	6.520315747	6.899489646
7.172411071	7.248451727	7.341641707	6.907322414	6.839237455	7.363617495
10.09426568	9.106823152	9.428845576	9.614560172	9.355403384	9.780583495
13.73439002	10.72127155	11.19293398	11.03163187	10.47584162	10.94786936
7.711523556	7.369773828	7.326942212	8.005727263	7.166060222	7.353244222
10.19332507	7.891912283	7.984708455	8.138908141	7.893273434	7.762794545
9.456392222	9.653591273	9.571117879	9.898158111	9.480384485	9.943097475
9.363823202	10.09339803	9.763952101	8.461795505	9.870221061	9.349388778
9.229670424	9.433849505	10.38329808	8.687541707	9.386484303	9.495686737
9.689129545	10.58347296	10.51291939	8.845226141	10.33989796	10.03123363
8.527198758	8.665646899	8.947127939	7.397472212	8.433468657	8.418553323
7.329578283	7.290498626	6.949335212	6.845837626	7.470009697	7.168718192
12.18492446	13.23864174	13.0147344	13.33155169	13.32669092	13.40990013
12.76207554	13.73439002	13.66158872	12.38794592	13.76702458	13.05428976
9.52752797	10.54950911	10.38531322	8.694840596	10.26632574	9.904237354
7.449616394	7.183932212	6.990730697	7.254644071	7.017519192	7.294284717
10.97767761	9.369545939	10.44392641	9.30949301	9.165786899	9.794631646
9.500664495	9.740584727	9.93663897	9.554043101	10.11555853	9.515743253
9.226702434	10.01524205	9.991229606	10.23660558	9.971733293	10.16165443
8.529417101	8.485631939	8.812013889	8.304169929	8.200163707	8.01423197
9.988725727	8.634342798	8.805907646	8.929384727	8.490531303	8.601386131
10.03385507	10.54565144	9.946747141	10.5152666	10.70643744	10.95683616
6.245100394	6.28178403	6.577737222	6.981086404	6.244370242	6.458964152
10.99623862	11.98196729	11.88649013	10.59541023	11.60888892	11.21668249
7.699362081	7.838173152	7.586712596	6.855182313	7.51061902	8.013487535
7.52646604	7.694540808	7.683318778	7.538502939	6.842504626	6.913266485
10.86305205	11.33477589	11.46724679	11.70348036	11.80554878	11.59427705
8.57971698	7.980568657	7.85507	7.372842667	7.501777556	7.592047838
8.729972586	8.976374343	9.189045596	8.792195697	8.787164939	8.711304737
7.209485919	7.51247098	7.816627253	7.388278253	7.186327495	6.974671657
6.857008404	6.630696545	6.517425313	7.191807606	6.836284939	6.709075525
7.676956101	6.596206051	6.709075525	6.530395293	6.293717162	6.687730495
10.33596201	10.41224321	10.27236099	10.08599138	10.33696433	10.05970796
7.270788949	7.263859848	7.303562909	7.657544313	6.427915242	6.617114677
9.419654929	8.476085384	8.097958939	8.875206121	7.565422434	7.771866444
6.736847222	7.531209667	7.374297343	6.612316919	6.208078222	6.433428778
7.886820596	7.918472515	8.008022949	8.04259702	7.636533838	8.139691131
10.0060683	9.956562586	10.25906505	10.99469779	10.01257663	10.89912578
9.151976485	9.788274313	9.562994313	8.625920364	9.548618051	9.430143091
9.626304798	8.933684374	8.952561111	8.37004801	7.982067081	8.291166909
8.603745778	8.830117929	8.789132293	8.295839707	9.530933293	8.650965909
11.88909817	11.13085117	11.59021288	8.701614283	11.15666056	11.54470354
9.850553616	9.36439596	9.235179424	9.486281202	9.159785394	9.176817596
5.810202495	5.82821701	5.901889	6.452349424	6.471881182	5.815697556
8.876650909	8.912452162	8.958493495	8.708995576	8.868979273	8.665646899
8.185177828	8.389243343	8.228066545	7.735250384	8.102862354	7.861535657
7.605411525	7.499261545	7.407756293	8.025425616	8.035675313	7.925098076
9.169862626	8.883993253	9.050265121	10.07580535	9.812775374	9.641787333
12.17829278	10.06255975	9.774477667	9.749660394	9.733950273	10.31095185
9.189590182	9.028300162	9.005975162	8.338686465	8.668536687	8.468898455
11.14385742	11.43208235	11.8358519	10.65751844	10.65365596	11.29230964
8.511305758	7.114362495	7.102414636	7.31258798	7.421037465	7.611680859
7.466006556	7.168103949	7.373116051	6.96619404	6.722265657	6.69036997

15.41210544	13.71423947	13.83772389	12.61090773	13.5160991	13.82538175
10.05053825	9.405539273	9.825199323	9.859664869	9.708693071	9.655770101
13.04869312	13.49745762	13.35359128	13.76108056	13.36582475	13.35919327
7.650858424	7.895278253	7.883536596	7.764931778	7.094132182	6.987890152
7.870356606	6.825565162	6.986217889	7.244122677	6.310299848	6.723002525
9.148511889	7.087495071	7.46337499	7.236271343	7.182269455	7.757532545
10.84302834	10.90606985	11.20660223	11.21668249	10.79420974	11.15666056
13.33155169	13.13522491	13.22730043	13.61616406	13.11515098	13.28559105
8.913588253	8.922082808	9.218466939	9.548028869	9.388912091	9.315088131
7.675893758	8.391460737	8.418553323	8.193820172	8.457489293	8.162329636
17.07010739	16.36806566	16.73234105	16.7804214	16.57174008	16.96059838
9.416442616	9.221852535	9.561667293	10.27714677	9.369545939	9.901640636
7.032021525	7.186960111	7.395598354	7.13441502	6.612514242	6.857741051
7.043463859	7.125696242	6.925244263	6.743297485	6.77992704	6.619744263
6.227143626	5.846995475	6.246725566	6.312971121	6.187353162	6.29391397
9.36439596	8.286128505	8.208692758	8.320357909	8.276867707	8.428176394
6.908606212	7.361380131	7.366006364	7.249066101	7.086390323	6.646430939
8.118879606	8.08193004	7.600942677	7.984708455	7.100167172	7.264798293
7.757532545	8.061773232	8.015705778	8.532980343	6.64694096	6.957403667
8.633848111	7.663618646	7.894315778	7.748187364	7.640998838	7.592689374
11.35475345	10.94786936	10.37346225	10.00361533	9.988725727	9.976273051
13.41514643	13.90036211	13.89331098	14.59173737	14.13521971	14.25042984
9.999132495	9.344138121	9.420326576	9.570470929	7.952029848	8.391020323
9.951175646	10.35809493	9.535633242	10.05320582	9.848227263	9.989520798
10.5104744	10.54190997	10.4994288	10.92608881	8.896578162	9.347406172
16.42659037	15.09418019	14.9727616	13.96779242	14.7172184	15.09418019
6.836068354	6.716118697	6.777231859	6.571432061	6.925244263	6.706906717
6.33399804	6.438718404	5.963785667	6.387713778	6.137509616	6.243838475
6.518976111	6.637000293	6.754460545	6.659807838	6.560443758	6.87945998
6.743041313	7.063754707	7.363288879	7.071197202	7.538207606	6.750902172
14.80749806	11.73070023	12.20640951	11.46145043	11.59654933	12.24933389
11.83072395	11.20498985	11.5571076	11.73271713	11.20660223	11.49029795
10.18000397	9.048100778	9.353057535	9.530205505	9.240719434	9.35371997
6.790809081	7.405524869	7.12986801	7.320252869	7.13936198	6.941957697
14.26676228	11.26262606	11.58195332	10.07670007	10.77103399	13.57574016
6.469182939	6.886441253	6.84607197	6.918279333	6.343264394	6.489773778
8.347717242	8.03364202	8.326162172	8.766842081	8.342773172	8.201303758
9.140392293	8.911109919	8.871686232	8.841575667	8.350619919	8.393536232
9.249612495	8.930963202	8.954798808	9.684048687	8.727989172	9.475206131
6.56193898	7.341966626	7.010236465	7.195502081	6.324763646	6.644450293
7.521885657	8.360024717	8.517288667	8.417196576	8.256076333	7.953126222
7.342871717	7.202701576	7.122424869	7.118793697	6.565610293	6.23392001
10.68338253	7.562627657	9.526870273	9.231349182	7.326634626	10.4687107
8.03364202	8.139691131	8.024633293	7.942071303	7.938531869	7.772503354
11.29230964	9.969141677	10.43872423	10.35591393	8.912003131	9.865550636
11.43380494	10.36465285	10.85100254	11.01531103	10.60383217	10.73014715
13.95955505	14.19920898	13.60269863	13.49745762	13.81935116	13.87263802
6.115970333	6.150782192	6.250292737	5.768914616	5.863786071	6.015419475
7.332963121	8.057063626	8.044608626	8.708467242	6.761583657	6.983707354
7.976470212	7.914965525	7.877487515	7.264510162	7.279669313	7.288727879
14.34357305	14.40789433	14.37933382	14.68633385	14.80749806	14.53168901
6.266099303	6.026785687	7.030955384	6.02690996	5.957120131	6.044454283
6.302911687	7.303019677	7.033625222	6.471263556	6.69350096	6.998704808
7.959830364	9.099242404	8.893968333	8.093473121	9.089024899	8.681067818
6.730688788	6.895973717	6.982135444	6.937881404	7.034867141	6.623632253
7.282065253	7.469040515	7.780448354	7.271701444	7.132177788	6.982384606
6.430701515	6.812925505	6.674468929	6.021467343	6.462851414	6.505445727
7.167804919	9.04056096	8.532119949	8.851571051	8.747331152	8.230588212

7.618887111	7.870978364	8.063461202	7.760294414	7.74007802	7.875808798
9.417594202	9.348735909	9.466909636	9.895061889	9.790672212	9.613206141
9.361671283	7.374842788	7.60736902	7.580594545	7.122138172	7.21150504
10.53737043	9.796940758	9.981718727	10.40810668	9.491725303	9.870221061
8.262181081	7.743466333	7.434501333	8.660608859	7.387061818	8.047933768
12.70002263	11.44491584	11.5737214	11.238428	11.0387546	11.86125412
8.738735232	9.390110384	8.867411152	8.74840203	8.883414061	9.023639636
7.179445253	6.836068354	6.796334212	6.795141737	6.511476192	6.665912939
6.010338545	6.223508929	7.020179424	6.213970697	6.146166717	5.971096737
9.145664475	9.984347768	8.985325394	9.395177889	9.350012182	9.034403333
7.252580253	7.170007293	7.151031394	6.573540778	6.774175343	7.093823343
10.17122401	9.903372323	9.772250768	9.974493182	9.850553616	9.909202788
7.830100949	7.416641556	7.058212263	6.608710798	7.121885657	7.136586475
10.64379219	10.75600216	10.55213977	11.18471248	10.60635433	10.94353341
8.127056495	8.19621002	8.379510323	9.330464051	8.891888576	8.849981545
11.86125412	11.78265022	11.63629658	11.4535969	11.42371373	11.65698449
11.70138789	11.24457024	11.17571904	11.75462574	11.29947244	11.04593523
8.041000495	8.297224525	8.731005677	8.450171172	7.873377879	8.338686465
8.765233303	7.915342566	8.08737399	8.431326525	8.092218303	7.726392606
11.76804793	13.06488572	13.05428976	12.74093701	12.52130815	12.34855768
10.41123643	11.01683173	10.92757694	11.40725584	11.34892675	11.44319437
10.48659409	10.65500785	10.56306707	11.08936677	11.00496707	11.09721747
9.17294002	7.767362636	7.865213	8.466026899	7.928481586	8.171788646
6.50518599	8.814624313	8.59655801	8.276867707	8.240781051	7.843077242
11.911688	9.61181703	9.482964828	8.524356212	8.705608919	9.070661273
8.476994374	8.314030071	8.452737606	8.992957212	8.466852202	8.635209293
9.260607	8.866888202	9.481668929	8.074508444	8.991970091	9.625041525
10.6488688	10.62142174	10.81540317	10.24942024	10.57282784	10.18291229
8.53628698	8.211411717	8.264694495	9.70798304	8.616914869	8.620790505
6.713538929	6.436812525	7.03146702	6.692548323	6.376693242	6.710284202
8.290726727	9.273640758	9.359783616	8.950874253	10.35699559	8.918756414
11.41238356	11.57178154	11.78023213	12.56726296	12.23505088	11.30131952
6.01628699	6.739060495	6.666157333	6.611655717	6.912541192	6.753240879
7.572084505	7.249375465	7.046726313	8.050484323	7.586712596	7.543741273
9.222631263	7.571116455	8.870602515	8.325717687	7.483852838	8.39402401
11.78725933	11.63401423	11.46320141	12.44264271	12.09804166	11.78725933
9.732604394	9.377908	9.158023	10.06071544	9.461673394	10.00002479
6.979690485	7.273507545	7.250545919	6.829819869	7.059676455	7.082160636
10.47242114	9.470840162	9.568406747	8.859179455	9.131560131	9.362770111
6.755924879	7.37546403	8.008400859	7.121885657	6.981618121	6.79225503
9.778131475	10.10365156	9.951175646	10.52247938	10.43757507	10.41123643
11.40725584	10.92190902	11.13551505	11.83072395	10.97767761	11.63401423
12.59668127	12.70787607	12.90783816	13.43523914	12.65472241	13.58209631
7.087495071	6.494595232	7.295094879	7.136875657	6.833840465	7.40806004
9.312727606	9.558925838	9.521192909	10.042425	9.631972414	9.093526566
7.753046657	7.308145939	7.712540141	7.549884232	7.377269152	7.655968232
7.025866354	7.469344313	7.278115626	7.314025869	6.592026141	6.762066263
13.56234785	12.64265677	12.51766186	12.34855768	12.56315114	12.72972704
7.617189485	7.158855657	7.154318051	8.398231626	7.011036525	7.076589626
12.89513437	12.48458958	12.44611991	11.91651973	12.39660011	12.43944324
8.928157121	7.999737535	8.286128505	8.350987111	8.056742909	8.030983081
6.818463323	6.743573939	6.782883697	6.630444394	7.039678949	7.256596263
6.915128061	6.649399919	6.916181	8.245649515	7.089925434	7.229587778
16.06827159	14.86987024	15.27762024	15.39581235	15.26374094	15.19830883
11.38025327	11.48258282	11.58005536	11.21816154	11.28370047	11.52238659
7.375117525	6.154487859	5.861397838	8.311348051	7.978709162	6.387713778
11.27153548	10.85225864	9.940265465	11.48840455	10.44712342	9.987077505
8.22437102	8.593558293	8.74840203	7.952386505	7.794365768	9.013830768

9.41003803	10.33146686	9.814415596	10.6488688	9.405539273	10.60383217
12.33576886	13.12023156	12.99565877	12.73352188	12.91196246	12.91196246
8.249350414	7.836508475	6.810023535	7.566773798	7.054238101	7.391655768
8.381309202	7.368501293	8.301264212	8.743124222	7.337989111	7.51247098
8.835493283	7.85604701	8.736221495	8.917682242	8.068382581	8.38301802
7.872008071	8.769824364	9.168324747	9.361671283	9.261851101	8.997224192
8.563085788	9.153730283	8.825911939	9.078331919	9.449254061	9.281446566
10.26832521	8.996148737	9.159196141	8.601386131	8.795325869	8.952561111
7.919797354	7.482291222	7.640998838	7.458509535	6.997949788	6.939947626
6.710071909	6.548684566	6.467810051	7.402701172	6.628772899	6.794878162
6.77992704	6.464688212	6.498851707	6.424911465	6.347837848	6.41992303
6.445028586	7.470886646	7.126519737	6.959934293	6.14736504	6.465818222
8.572701232	9.090721626	9.004940778	8.583542788	8.923576949	8.529417101
6.959689121	6.449700465	6.225711566	6.92669298	6.881026485	6.75007699
11.3290504	10.28089537	10.42297323	9.718307192	10.48432644	10.66945215
6.123808535	6.31770101	6.39883498	7.237206848	6.387934697	6.594580697
8.141366242	7.965251131	8.070920919	7.800216455	8.110083838	7.863195747
8.096910323	8.221403505	8.601386131	7.931306778	8.350987111	7.652632424
14.57091557	15.41210544	15.42966281	15.61926687	15.16803204	15.41210544
8.713417869	9.018299374	9.049171303	9.569063343	9.142159707	9.053706202
9.147296667	8.886096818	9.006527424	9.166446616	9.058356636	8.897751606
7.281724869	7.340746384	8.29635502	7.476255091	7.220780818	6.917518081
5.880601303	5.783226717	6.319301697	5.845488535	5.81111603	5.824282758
7.332078838	7.020179424	6.790568566	6.614759768	6.54114802	6.301337414
14.1904887	14.10328164	14.14208591	14.5413574	14.30038792	14.05855866
12.39973853	12.32964397	12.29886409	12.88708268	12.14789109	12.63102018
7.945111576	7.946541949	7.662615848	8.01423197	8.099112939	7.706168626
12.80166365	12.94821125	13.1944969	13.85817003	13.27940818	13.54965001
10.46232362	8.838020616	9.749004051	9.779012071	9.022000869	9.650789909
12.22629088	12.00086001	12.30465105	12.45390404	11.93770961	11.48655657
7.300031424	9.424273283	9.593075545	10.78987961	10.5431016	7.652274172
9.30577901	9.415860727	9.818860343	10.56802411	9.171164091	9.698896848
7.734550869	7.515186091	7.953840929	7.291068525	7.533663232	7.571747222
13.10484893	12.90359001	13.06967433	13.44849295	12.81735602	13.17778072
11.77552743	8.790654051	8.844732879	8.459596535	8.390560192	8.870602515
8.582586091	8.443547162	8.45577798	8.709464434	8.762742172	8.78214798
6.388134263	6.36070104	6.303290121	6.662035182	6.416786646	6.581763828
10.37573078	10.09426568	10.30358984	10.98350754	9.999132495	10.31997281
11.82616001	11.21958266	10.61992902	11.67371856	10.60743282	11.18800276
11.19293398	9.991229606	10.21484784	10.82945836	10.29324401	10.59292728
9.935661677	10.05144087	10.58579258	10.27432009	9.899010949	10.19757035
11.5386618	11.62059475	11.671419	11.71895032	11.41415047	11.72807534
13.06967433	13.40302459	13.23864174	14.11151028	13.97489728	13.67554111
8.53670504	9.103811545	9.237064687	9.153232364	9.079484556	9.188424626
10.77762269	11.3403052	11.13240418	11.51647662	11.10460602	11.18636156
9.512482222	9.264483889	9.647013263	10.12799521	10.48659409	10.51291939
11.23280633	11.35475345	11.37847359	10.17122401	11.60266156	10.26632574
12.10951308	12.24933389	12.31089415	13.27940818	12.48124823	12.73731843
12.53911948	13.41514643	13.30795266	13.64789782	13.12023156	13.44849295
12.86086109	12.45390404	12.58612865	12.40735915	12.40343679	12.75784116
15.15443825	16.22759729	16.09492117	15.95256028	15.92931651	15.83165575
11.33657706	11.07493153	11.31852116	11.78725933	11.42787377	12.07810634
7.419275485	7.293451808	7.287539616	8.34368697	7.530574707	7.322572717
7.499261545	7.932792414	8.305787747	8.021628354	8.788135667	7.530907465
12.86955734	13.20536123	13.32669092	13.54965001	12.83405786	13.23341018
11.52645916	11.55524693	11.70138789	10.86820493	11.37131374	11.6137143
9.925979414	8.253639283	8.367840636	8.564366949	7.840025778	7.786626808
7.884680111	7.882850313	7.629989283	6.990995828	7.872350636	7.553468394

7.374842788	7.628690051	7.790604293	8.178268222	8.08972896	7.962380919
7.767042424	7.359873626	7.264201869	7.244714616	7.220481808	7.531770848
6.734989303	7.229904545	6.739060495	7.796085586	7.132449434	7.232456929
8.05474504	8.613985131	8.233421293	8.549141545	7.329290475	7.777311636
8.818210192	9.109120374	9.488971182	7.010236465	9.328020364	9.922126404
12.3543152	13.24400831	13.00101781	11.9875501	12.88708268	12.01673563
7.628690051	8.187136596	8.051256737	8.243245818	7.953126222	8.274698687
7.055367566	7.071486061	7.232456929	7.465036929	7.486746556	7.259665485
10.10365156	10.19136651	10.22448985	10.77252405	10.42927797	10.28005063
16.7804214	16.12384164	16.17943014	15.92931651	16.14978837	16.57174008
10.44934262	10.70902317	10.80239406	12.49965499	11.62739451	11.32185019
8.269818727	8.672881101	9.108564253	8.305787747	9.229670424	9.054860525
6.149311434	6.637667434	6.435479606	6.958715091	6.129601242	6.197694323
10.0841781	10.13375911	10.12314196	8.50618604	9.782868192	10.02269473
7.015941667	7.166060222	7.200755828	7.705814323	7.548584576	7.452458778
9.881176566	9.975431768	10.48782122	10.60044575	9.777520899	10.48782122
15.88528111	13.89331098	14.07988992	12.99088515	13.9071606	14.84412351
14.10328164	14.5413574	14.60195877	14.85646221	14.79546777	14.42673578
13.61616406	14.11151028	14.04335209	13.55640443	13.41514643	13.83772389
10.93494087	9.226128374	9.363307667	9.35858599	9.409395929	9.472607081
8.526186556	8.314850485	8.351752182	7.761378475	7.986535808	8.063898495
6.909921869	6.844744919	7.075769859	7.728361283	7.402701172	7.035145869
7.905419798	7.02934998	7.803193929	7.413750677	6.890761263	6.995550687
14.5513462	12.80602179	12.97977545	12.10694646	12.38794592	13.02006671
7.792995667	8.357077121	8.585303495	8.358783152	7.876138384	8.039451586
11.7349036	11.69188536	11.84027237	11.92156179	11.52052132	11.92665931
8.166983455	6.528629101	6.585042414	6.944866586	6.567922788	6.713303939
7.217289505	8.616914869	8.161911788	8.35372597	7.90505396	8.197004869
8.985821889	9.020821455	8.80133401	9.197870838	8.79576798	9.083185818
10.29101087	10.30151978	10.42175216	9.698181576	10.33476777	10.43655428
14.88238784	15.70163214	15.61926687	16.25437967	16.09492117	15.88528111
10.23351878	10.70299608	10.64623997	11.01045685	10.5152666	10.63266161
7.214035101	6.749084333	6.941467394	6.765793475	6.691055667	7.320933798
6.987890152	7.385541293	7.679923515	7.881102323	7.291354758	7.628336929
13.54221797	10.64128512	12.59323492	12.35168631	10.20358283	13.5964752
11.92396137	11.50632759	11.63837311	12.41337695	11.06877356	11.53677901
8.299695162	8.894491141	8.865407737	8.888204949	9.397189192	8.619347838
12.43279956	12.32364634	12.68484185	13.26276274	12.70002263	13.05987966
11.36979611	11.07013707	11.51867768	10.77762269	11.5386618	11.71443529
7.614831859	6.900739596	7.038073394	6.917762192	6.499296646	6.659312505
10.61133106	10.36032821	10.50677257	9.336479586	10.15875628	10.58347296
13.44849295	13.83772389	13.83173935	13.71423947	13.79381649	14.00297944
7.105472313	7.23119303	7.692217293	7.773938747	7.127365253	7.706911222
7.187213162	6.617114677	7.421037465	7.173022687	7.080525788	7.583998525
9.598265626	8.380828141	8.046104414	7.741148566	7.524403394	8.513601889
8.348145404	8.236217485	8.119679141	8.63568099	8.760676242	7.959488687
10.45041513	9.737663949	10.03123363	9.692793657	9.959124273	9.685649929
7.776629576	8.663303283	8.440878707	7.764199131	8.319901444	7.954533778
8.39227803	6.80380202	7.011288768	6.76944904	6.994769535	6.982909788
6.923167202	8.193428737	8.107705111	7.97830402	7.805446323	7.673764162
6.067372778	6.243182121	6.508047626	6.440388828	6.190947343	6.463301323
6.962034242	7.124596343	6.79984598	7.110844919	6.792957485	6.671548495
6.523815232	6.59799604	7.913459869	6.951961606	8.644074768	6.633467848
14.19920898	14.31917195	14.53168901	14.64328928	14.59173737	14.94534759
9.950180667	9.03221704	9.253086273	9.318836889	8.796713535	8.859715222
7.011036525	6.722483515	6.713056273	6.07088597	6.553437202	6.358502424
8.16147404	7.838908253	8.282900293	8.318250424	7.777311636	8.184399818
12.29275278	12.13538265	12.04160066	11.98440811	11.80104424	11.91651973

7.979092162	8.020887778	7.896600606	7.837507091	6.935285707	7.29228501
14.50017253	15.10875812	15.21350605	15.54332231	15.08134613	15.13975511
12.41634374	12.66985986	12.7939548	12.80166365	13.03957854	12.64708972
8.625920364	8.862785404	8.574218515	9.34289298	9.139724848	8.929972798
12.81379045	12.03310273	11.72126504	12.67378338	12.23191382	12.10414281
9.975431768	9.493200828	9.70798304	8.541524636	9.657223606	9.561667293
8.342773172	8.675789182	8.679578434	8.082778869	8.176718545	8.366948404
12.62449502	13.33155169	12.9851923	13.91489381	13.22730043	12.84627851
11.00620373	8.85518403	8.868452172	10.9991534	8.782655051	8.568242293
8.19621002	9.926784556	9.835081424	8.481851586	7.922697606	8.096128192
11.59427705	11.65261158	11.91651973	12.64708972	11.78265022	12.14507673
6.213400495	6.723255293	6.608026545	7.120752303	6.925960677	6.774175343
6.928307859	6.587577626	6.467370697	6.391874798	7.028571808	6.427915242
7.580594545	8.510894717	8.182386152	8.311754818	8.404396899	8.200539131
9.614560172	9.793942798	9.886023253	10.32635032	9.965319232	9.977292899
10.47820756	9.430738808	9.511202364	9.566949	8.39227803	8.953164051
11.64244603	12.052647	11.98440811	10.91903182	12.26775966	11.38775416
12.99088515	13.83173935	14.03424604	13.90036211	13.50997822	13.91489381
14.32804886	14.82007331	14.67552064	14.70594079	14.82007331	14.30038792
12.0838996	12.0838996	12.16036216	12.60396528	12.07810634	12.55625498
7.940254566	6.661815061	6.879719566	7.846324646	6.436610747	6.97366599
8.281588828	7.46337499	7.683943101	7.383711768	7.724049626	7.608647091
8.396963808	9.29794999	8.855784909	8.647922808	8.565878434	8.41552702
7.469040515	7.128773535	7.281724869	7.271131505	6.484888434	7.299385535
14.4185289	14.52085241	14.74028573	14.91973608	14.38889659	14.63205333
12.95787308	13.58996905	13.42888134	14.49002723	13.76108056	13.70717091
5.810341747	5.778636677	5.785877899	5.822896859	5.781781232	7.121885657
11.90384839	12.25251148	12.2031413	12.95303817	12.30167305	12.25572441
10.14326599	8.543234566	8.854603364	8.720627081	8.483326677	8.595703707
8.302062758	7.571747222	8.05474504	7.439837192	7.74646602	7.466342606
12.34237091	12.18792926	12.13244857	12.57486869	12.052647	11.9875501
12.94367069	12.81379045	13.14093405	13.77352346	13.21058954	13.34730616
9.443424081	8.613130333	8.685647889	9.986114121	8.391869283	8.821324343
6.439328828	6.461688586	6.739790818	6.816537596	6.930222384	6.382806586
11.02700786	11.66934136	11.54470354	11.30216845	11.82616001	11.42371373
11.92937262	9.223302313	11.27153548	10.99763472	8.759332636	12.16279806
9.508442768	10.0406788	9.921211657	9.809529798	9.967178364	10.23550694
13.42153085	13.22156044	13.48401144	13.69473258	13.03496614	13.77352346
9.429506515	9.429506515	9.648698737	10.20859114	9.284395657	9.776839404
9.515069697	9.352487919	9.380979222	9.465071586	9.267923343	9.182204323
13.44126502	13.30255942	13.40302459	13.98081649	13.25686564	13.35359128
11.52052132	8.313601758	8.655934636	8.102124455	8.339115859	8.372772768
6.791029141	6.659312505	6.935043828	7.071771354	6.919663687	6.851905687
6.855692535	6.842028788	6.931701566	7.361082222	7.010236465	6.996769242
9.992117576	8.528915121	9.033915556	8.056301667	8.598479657	8.686126354
6.721795586	6.789988131	6.89207597	6.492200283	6.313338182	6.239068939
7.723384495	7.039678949	7.406826051	7.178905394	6.884322333	7.066488111
12.89113428	13.31405593	13.38447499	14.25895571	13.57574016	13.75520609
13.12562627	10.04963797	10.11051676	9.124449354	9.556000646	9.742174424
14.6970922	12.44950322	12.96241124	12.10414281	12.69316533	13.37822002
7.834787162	7.696877364	7.154937303	7.266813283	7.262427071	7.221345788
8.510413414	8.040196323	7.960941889	7.290498626	7.083314707	7.555661677
6.763870838	6.885064545	6.873566444	6.593141616	6.490432818	6.647920455
7.327495141	8.113343889	7.394983566	7.544047919	7.680542242	7.391342576
6.254539838	6.401214677	6.799383374	6.419328293	6.27383702	6.762834384
12.76628311	12.46055504	12.58957491	12.60022648	12.06964699	12.63888703
12.15752365	9.211846273	9.543825273	9.631972414	9.482360616	10.0178996
12.24933389	12.36759935	12.26162026	12.62127559	12.26162026	12.48458958

11.77053658	12.10694646	12.11562205	12.28105924	12.21312889	12.09225922
5.782454909	6.338442758	6.722042727	6.574185192	7.179445253	6.447674828
6.407245091	6.261769939	6.692767899	6.140691525	7.227882465	6.243490263
9.766131535	10.42622906	10.46108665	10.15483313	10.32750324	10.85923156
6.40434199	6.039925859	6.096900687	6.338230899	6.538664657	6.46756804
14.21449649	14.4185289	14.64328928	15.449005	14.63205333	14.9591327
14.40789433	14.56207529	14.7172184	15.65613201	14.9591327	15.05177986
14.5108222	15.15443825	14.85646221	14.72870348	15.01206542	15.18392271
12.31089415	11.74205097	11.88375452	12.4571909	11.75908761	12.32659908
13.5964752	14.223913	14.1282639	14.65397593	14.15092087	14.27397716
6.531968667	8.337550606	8.187136596	7.775630727	8.206690121	8.140128606
8.225777414	8.573727141	8.373157505	8.111719586	7.825104404	8.434806414
14.15092087	14.25042984	14.27397716	14.9591327	14.68633385	14.56207529
8.921533879	9.719000626	9.260036485	8.705145061	9.271759586	9.09178804
13.08418191	13.66158872	13.7211242	14.06476988	13.64789782	13.80033164
8.230181798	8.145617202	8.243245818	8.03490996	8.083485404	7.945864808
9.729555121	8.387939343	8.524867051	8.096525737	8.192266657	8.326518101
8.602379626	8.115023838	8.368780919	8.369612525	7.18313201	7.780823677
6.889002121	6.760385848	6.727688212	6.788535525	6.860943283	6.885618131
11.05493438	12.40735915	11.99323436	12.25572441	12.02240324	12.01956714
10.8405808	11.58621592	11.47871073	12.14789109	11.76336308	11.89361272
7.928481586	6.626873192	6.86214797	6.712585717	6.688196	6.828875111
9.194378495	9.481668929	9.618955576	9.533596364	9.588958727	9.533033172
13.7211242	14.59173737	14.66395575	15.15443825	14.62217632	14.93426708
6.526325283	8.371229848	7.920877394	9.067856838	8.200539131	8.102484232
11.2608846	10.90466263	10.96000427	11.39594259	10.7982674	11.20163629
9.315088131	9.041236566	8.970028374	9.120933788	9.291393586	9.245445293
12.64708972	12.65104366	12.71520416	13.70717091	12.92937388	12.95303817
7.956615091	6.361580141	6.501886182	6.36070104	6.311249485	6.354771242
12.32964397	12.46466592	12.52461444	12.8293171	12.32364634	12.6076243
8.330618758	7.653305051	7.864224778	8.558556152	7.47214603	8.066374202
7.279952616	7.429246232	7.487647384	7.723687384	7.937009	7.587005707
11.27306561	11.79171062	11.74935785	12.12692782	12.17829278	12.2031413
8.645541747	8.531247657	8.927565404	8.538919798	8.045722828	7.891143768
12.28693608	12.39348455	12.70002263	13.33661988	12.62449502	13.09977851
14.85646221	15.46469304	15.70163214	15.72204116	15.74256723	15.86132141
6.592026141	6.582941808	6.618749384	7.044228545	7.057634667	6.612755929
7.755825222	7.691187768	6.980492495	8.648450303	7.144887111	7.392563535
7.074173121	7.797507939	7.969427899	7.476561202	8.214678465	7.649620657
7.926719768	7.507667889	7.392218323	7.60255999	7.693516556	7.861189172
13.50411017	13.13007894	13.47327752	13.83173935	13.05987966	13.64083669
12.00354228	11.41981305	11.69658017	12.06397065	11.32721575	11.86401838
13.66158872	13.46005067	13.50997822	14.07988992	13.52959744	13.63435472
9.977292899	10.99623862	10.56084718	10.92060078	10.72633412	10.67939021
6.881279879	6.923412444	7.334394303	6.78758299	6.984268657	6.438496414
12.50328361	8.918202434	8.991970091	8.750392838	8.773988747	8.789683394
6.923412444	5.816773424	6.756647869	6.307278677	6.656675747	6.503401212
15.24813401	16.4534382	16.25437967	16.57174008	16.73234105	16.49551516
6.895207929	6.524488081	6.725034798	7.431765758	7.412191343	7.31189602
11.23641801	11.51008986	11.74205097	11.55322016	11.18800276	11.82112126
10.10631712	7.617890202	7.790916071	7.571747222	7.356848556	7.508747081
10.0188097	9.600185283	9.35128199	9.860587788	9.365016101	8.996148737
11.75662781	12.08680505	11.92396137	11.68700199	11.9875501	11.12151875
7.616235111	7.033625222	8.118879606	8.064260859	6.897441	7.301506111
11.18800276	11.38970236	11.42562271	12.09225922	11.41778198	11.42178201
10.77387872	10.9889639	10.81142627	10.00886723	10.5336638	10.45754412
14.20688311	14.83117585	15.06622261	15.24813401	15.19830883	15.32551587
8.851018515	9.300367869	9.558255162	9.464440242	9.454390232	9.501306556

11.03580591	8.660166475	9.094076404	8.586731747	9.008373061	9.127251283
14.9591327	12.12151457	12.27101	11.33657706	12.01956714	12.43600068
14.7172184	12.12939166	12.49170848	11.66441291	11.95514832	12.61090773
9.578067192	9.742895778	9.599465606	9.138451657	9.637663091	9.807061475
9.11510898	9.918671212	10.18468509	8.488340929	9.557589465	9.255548899
9.238865747	9.636296737	9.557589465	9.731150899	9.904237354	9.711723394
7.619556354	7.339214566	7.759260374	8.32856404	7.862831293	8.302062758
6.479663677	6.521900899	6.527432758	6.398440768	6.087012455	6.123014818
6.362324414	6.016419242	6.386460131	6.396200636	7.275508121	6.34603899
7.60255999	6.81164602	6.342094838	7.073903182	6.075996354	6.107491061
7.576363505	7.789255535	7.311327071	7.694916828	7.152088687	7.173022687
7.390207394	7.545986354	7.178633636	7.329870525	7.503050071	7.562285172
7.663954061	8.152794889	8.285312424	8.593558293	8.104133364	8.339115859
6.902482606	7.347141657	7.470009697	6.536641737	6.698319919	7.172666465
10.30358984	10.41626133	10.25811169	9.443424081	9.997192202	9.395177889
8.467263475	8.296772869	8.686679798	9.369545939	8.735287485	8.627322343
9.468772697	9.757590192	9.818059192	8.737817051	9.534194535	9.378583414
15.449005	13.26910509	13.17778072	12.46055504	12.73352188	13.41514643
7.821625848	7.062195576	7.028039657	7.399358222	7.393167162	7.309336424
6.74001704	6.974373515	6.909148313	7.635907081	7.21289504	7.217289505
8.130283051	6.611837919	6.639289253	6.761583657	6.442532636	6.784076586
6.369292	7.422224808	7.068134081	6.780426	7.116047475	7.157499455
7.112620848	8.213079374	8.098302202	8.044980354	7.85710597	7.451512687
8.019434586	7.104662848	7.259665485	7.604109192	7.610574101	7.810980737
9.012031232	8.107270071	7.919797354	7.783465061	7.650531152	8.170954818
7.07551896	7.298118263	7.136356818	7.800624061	7.294583061	7.457632404
7.267131253	7.111135707	8.150096222	6.957636657	7.313451111	6.800553495
10.42297323	10.79420974	11.34576518	10.84705809	11.19447199	10.92757694
9.302735434	7.476910626	8.042161798	7.710917071	7.390207394	7.667649192
6.580572354	6.290463121	7.013137848	6.415074455	6.347438828	6.810861495
10.52904685	11.65052704	11.47127063	10.37215733	11.28207759	11.03580591
7.448962859	8.08075902	8.176718545	7.179445253	7.812327374	7.432070859
9.051957646	8.728878424	8.890936242	9.219562515	8.78214798	9.189045596
9.627013505	9.393934131	9.612498475	9.421059	9.746145949	9.739859192
11.39185194	11.37670858	11.37131374	11.52238659	11.33848921	11.50814936
10.16642595	10.23247836	10.23850383	10.70181173	10.40592026	10.60743282
13.04381444	13.98831971	13.86604428	13.14611959	13.60269863	13.40302459
8.479185606	7.77977198	7.690840778	7.567404636	7.295980475	7.301802939
13.60269863	14.28325373	14.50017253	14.35197184	14.11997744	13.93694444
9.756840071	8.968910758	7.792215626	9.410728505	8.337550606	9.204345657
11.93178106	13.09977851	12.24351077	12.76207554	12.58957491	12.57486869
14.86987024	15.83165575	15.92931651	15.99508933	15.56435861	15.29549959
7.939609869	8.061012707	8.261007747	7.164403717	7.918105152	7.860366808
7.287828434	6.890508141	7.101319909	7.437630343	6.763870838	7.239071505
7.346567394	7.025866354	7.356848556	7.67197899	7.422831253	7.404680798
8.251922909	7.783465061	7.526167626	7.395598354	7.352900667	7.606699253
7.962680434	9.125507586	9.21905796	7.406536232	8.833963152	8.604635949
9.321890899	7.871272152	7.965251131	7.945472162	8.583542788	7.541750556
8.005727263	7.531770848	7.237838192	6.897750404	7.368846081	7.518690889
8.48093102	8.201745222	8.327353333	9.267383556	8.663769939	8.837022465
7.674820101	8.038729465	8.090157434	8.792768747	8.454954939	8.710842768
7.221869232	7.448630646	6.985120646	6.932196717	7.122424869	7.357775939
8.489580414	8.052077899	8.988577162	8.703161646	8.685647889	8.972927475
10.3896003	10.46342714	10.78408516	10.60743282	10.61867663	10.22267877
11.4535969	11.68700199	11.7894918	11.19817469	11.35278885	11.66189432
8.932093424	8.808345061	8.743617505	9.521972354	9.341222778	9.290152727
12.26775966	11.87175588	12.25572441	11.54053762	11.38577951	12.23505088
11.80554878	13.0147344	12.56726296	12.52461444	12.8293171	12.25251148

10.54950911	9.473962505	9.452497313	9.35128199	9.11393401	9.257979848
9.303966081	7.540776061	7.410002717	7.478803566	7.415309101	7.585519525
7.707249808	7.870356606	8.036816444	8.509502414	8.318250424	8.528049141
9.883591758	10.29949835	10.33252085	10.50543333	10.51400776	10.52476206
11.63401423	11.4481816	11.79171062	11.51008986	12.15440913	11.80342824
11.36065784	10.47123164	10.69470446	9.469429253	12.99088515	12.02240324
9.390110384	7.993077394	6.69350096	6.924473131	6.811363364	7.050347636
7.670902747	7.700026646	7.69973097	6.741974778	7.433629313	7.638629778
7.736260545	7.939609869	7.850180343	8.59655801	8.405727848	8.078389566
8.496365737	9.016491202	8.765765323	8.172571899	8.90531298	7.942071303
6.334208879	6.658852677	7.064538818	6.80380202	6.765793475	7.357173859
8.115437899	8.736221495	9.045168101	8.06745399	8.335861768	8.835019636
10.04511943	10.03611568	10.2861211	9.132080535	10.04421951	10.06822268
8.530767747	8.321919424	8.516793455	7.672658859	8.562664263	8.440058758
10.95093974	9.11510898	9.455015404	9.50710198	8.911109919	9.332795131
9.468134182	9.739859192	9.682548525	9.648698737	9.835894606	9.952967535
7.90505396	6.842504626	7.731201121	7.242650333	7.303836939	6.995294596
7.795429838	6.867411697	7.039678949	7.040523253	6.399871131	6.631383222
6.734222485	7.092334828	6.669677444	7.162716495	5.799658879	6.051732566
8.707515232	8.222223545	8.545617303	9.534883657	8.503061444	8.793874616
7.987573434	7.216692263	6.930743303	6.735859162	7.064817384	7.306475687
6.927522051	7.753435232	7.612909768	6.977291071	7.845242687	7.511838192
10.43757507	10.08145981	10.79686873	11.63401423	9.939327313	10.35917202
8.545617303	8.853585596	8.777050384	9.317615606	9.415860727	8.904239657
7.378186273	6.831129859	7.018669091	7.855388333	7.188612899	7.208062909
8.200163707	8.165787747	8.175629323	7.40806004	7.924172566	8.186741323
11.02105469	11.77305263	11.52824	10.40701089	11.66708917	11.40306525
7.695912303	7.63758797	7.582654121	6.885064545	7.665314131	7.636533838
7.452458778	8.093860848	8.134790222	7.970233808	8.258860253	7.54731098
6.369108576	6.749332859	7.121605929	6.705162535	6.500408909	6.700579303
9.785983535	8.651452677	8.484240687	7.853698798	7.806845253	8.53670504
8.400464071	8.463063475	8.252794354	9.372705202	9.084401768	8.63611798
10.55070525	10.37346225	10.43440935	8.932093424	10.16642595	10.55602586
13.52959744	13.15645152	13.07454735	13.14093405	12.68903729	13.04381444
10.53141254	10.3918566	10.60507136	10.7814083	10.82416773	10.61867663
7.589604303	7.612294131	7.328126596	7.832891081	7.158043384	7.490430596
10.07107569	10.40486524	10.66342196	9.211846273	10.19757035	10.31289083
11.39983314	12.15752365	11.82112126	11.21958266	11.85889751	11.65918316
10.21375666	10.2761618	10.67081044	10.96748398	10.76471511	10.66465811
7.306206455	7.298462212	7.284105152	7.015108687	6.682537212	6.845030273
8.678158051	8.738735232	8.560860505	7.298462212	8.39308204	8.729485838
8.080354404	8.471488636	8.459227657	7.269598859	8.344441616	8.295009141
9.348735909	7.799524616	7.926719768	8.396532182	8.061773232	8.35018502
12.05813886	12.37416009	12.3774088	10.95374716	12.29275278	12.09804166
9.404333879	9.679645121	9.710962273	10.03385507	9.632768747	9.399129424
9.324388263	9.610538374	9.545881535	9.991229606	8.125818909	8.85518403
6.456479596	5.885582222	6.112843636	7.096955818	5.942271616	6.091920121
10.59541023	10.34193651	10.64128512	10.37573078	10.6362571	10.35699559
8.209418434	8.070136677	8.319901444	8.028141747	7.706911222	7.629344343
6.267641192	6.228051758	6.181284283	6.779180051	6.796334212	6.516723778
8.075284384	8.492322828	8.21550303	7.651222556	8.301657889	7.985767212
9.967178364	9.559682182	9.368224626	8.134366646	9.530205505	9.041236566
8.561321323	8.384333949	8.800782677	8.898709273	9.222631263	8.739178636
8.32856404	7.620272192	8.047553323	8.99408802	7.854325061	8.383429707
10.30810853	9.648698737	9.702015293	9.865550636	9.339095222	9.839111
12.77015535	12.92542832	13.34204398	13.12562627	13.42888134	12.81735602
7.303836939	7.654890798	7.849821828	7.737649848	8.154321646	7.275223848
8.543686687	10.01257663	9.023071899	8.548679899	9.459003	9.568406747

8.682574626	8.911610283	8.738735232	7.69973097	8.607626293	8.347358778
9.853021111	10.06547375	9.97079498	10.5397012	9.759214424	10.39968902
8.640338566	7.293153485	7.414093131	6.90271597	6.897750404	7.237206848
8.885588879	9.829822242	9.602112616	8.448456354	9.886762101	9.081927515
8.335469222	8.346198293	8.428657566	7.281724869	8.157542717	8.833963152
9.576136707	9.939327313	9.890949131	10.55602586	9.747577162	9.509876273
8.265965545	8.559975162	8.946596606	9.019287687	8.540202646	8.774537848
10.40925212	10.82664672	10.55070525	9.923658606	10.54078574	10.2829717
9.905108616	8.988577162	9.964343626	9.848227263	9.443424081	10.70643744
9.678297182	9.477181455	9.390834152	9.752732475	9.519820636	9.799317404
11.06261544	11.77802923	11.7349036	11.84736958	12.03310273	11.60479972
10.39296715	9.716648152	9.485668061	10.05607537	10.1905069	10.05426836
9.299183616	9.759214424	9.404333879	8.497259596	9.569854202	9.546645747
8.27903296	8.243245818	8.346198293	8.813032152	8.578858313	8.734416606
9.092457495	8.581684657	8.851018515	8.038729465	8.414282596	8.187136596
6.908859909	7.218159515	6.801828172	7.675499444	7.13133802	7.609879768
7.02909003	7.441392919	7.36510997	6.597486293	7.481322818	6.678083162
8.160266646	8.456593071	8.543686687	8.591651626	8.729485838	8.439612798
6.384310424	6.168008697	5.942858242	5.788907172	6.571898091	5.78329004
9.774477667	7.711891616	7.828584192	7.670902747	7.377557566	7.736964596
7.041650374	7.299714313	7.196057152	6.392119051	7.336756222	7.03650198
8.142197384	8.362537455	8.384333949	7.251769333	8.159434576	8.19458801
7.873714818	8.384837869	8.436545051	7.51340999	7.739667424	7.880363606
5.772068798	6.989960667	6.786766677	6.682819434	7.173570899	6.432817071
10.34626898	8.465000141	8.703733141	8.099112939	8.495994869	8.717364515
11.73271713	10.09682592	10.27139209	10.20964764	9.634846707	10.19418334
7.630295798	8.168587596	8.274284293	7.34349297	8.361327455	7.658148485
8.925792808	8.966940586	9.011546121	8.964085616	8.932093424	8.598479657
9.894213939	10.02821235	10.19136651	9.911926424	10.35069541	10.09896607
6.960198707	7.509707515	7.653305051	7.518993111	7.799854778	8.019434586
7.795116566	7.734550869	7.87472	8.354947869	8.028141747	8.116212253
8.39446903	8.780092131	8.733877859	7.803193929	8.561321323	8.47107903
8.985325394	9.095762	9.110297333	9.780583495	9.406893465	9.481668929
7.528004313	7.336756222	7.930615646	7.194186434	7.051418091	7.344013535
7.09469803	6.964119424	6.638813101	6.389460141	6.248876929	6.324159687
6.795847303	6.944866586	6.976769172	7.576687677	7.222444626	7.338325414
8.659649677	9.867911152	9.937540263	9.852239606	10.08893073	9.282486081
12.82153585	11.11388317	11.36610866	10.97476091	11.38025327	11.52448673
7.312858101	7.005636414	7.163287778	6.99155401	6.436392303	6.81111
11.22456459	11.33292491	12.33273925	11.90674449	12.11562205	11.69891546
7.588284717	7.529315566	7.549206232	6.939713242	7.02200603	7.021742939
7.689906808	8.282900293	8.10140802	7.377888556	8.298004222	8.166172404
7.583321859	7.432070859	7.388945192	7.974325596	6.838435556	7.159715333
8.424934535	8.566871343	8.798818646	8.298396535	8.857327323	9.147296667
8.104866828	7.960192616	8.098747556	8.679110091	7.467830485	7.649291899
8.324453253	8.768863636	9.234418071	9.058895657	9.356741384	8.691458141
15.09418019	12.61090773	12.7494647	12.31089415	12.49965499	12.29548604
8.486543404	8.902484929	8.961327374	9.018299374	9.362770111	9.306330646
7.450590374	7.782462626	7.963792818	6.953109798	7.762794545	7.418946535
7.361082222	7.771866444	7.789255535	6.512183152	6.885887889	7.642932737
6.875487455	7.18283101	6.540929384	6.955441212	6.615899091	6.788320081
8.303356141	9.021393263	8.849981545	8.30620801	8.660608859	8.516793455
7.425203606	7.822322859	7.503050071	7.541090081	7.84419497	7.839647131
6.630696545	6.072186929	6.092545697	6.493950384	6.270309222	6.559739475
6.723501929	6.29528098	8.281167657	7.492628778	7.900388485	7.198896253
7.566773798	8.53537201	8.311754818	7.768055657	8.442251212	8.365106485
7.710917071	8.133538364	7.606043535	7.336476646	7.792995667	8.181574081
7.913871889	7.380377788	7.514105485	8.680513909	7.856391758	7.093254121

9.782868192	6.689141323	7.242080394	6.601976232	7.124332475	11.25349741
8.573273283	8.950874253	7.221345788	9.231946182	7.271131505	7.778011646
8.770297859	8.545091384	8.622197111	8.510413414	7.419275485	7.531511131
6.673038182	7.091827414	7.240600646	6.737335172	6.526565121	6.91172998
12.3843369	11.565007	11.66441291	11.68484165	10.3548828	10.73517618
15.58150095	12.49170848	12.68097339	12.33907422	12.49170848	13.16151113
6.369870263	6.605935465	6.747331182	6.268062758	6.676225182	6.611837919
7.307022919	7.378186273	7.369773828	7.568310444	7.867668606	7.733842657
10.88031852	10.687913	10.9088749	11.43005301	11.56725755	11.52824
5.827547343	6.47408797	6.318093747	6.777231859	6.119415222	6.269348929
9.299916465	10.05970796	10.14326599	9.496413788	9.662314131	10.37124354
11.98196729	12.33273925	12.30789527	11.54871614	12.96662998	12.10694646
7.301802939	7.318216242	7.735579636	7.708922879	7.91307104	7.517077263
10.42062148	10.5199714	10.3025572	10.8254891	10.22448985	10.34311059
8.826846768	8.746767313	8.393536232	8.971727333	8.708995576	8.701614283
6.87945998	6.873088737	7.316995263	6.624377646	6.171325172	7.07468796
10.84974643	11.53677901	11.52448673	9.907546465	10.95683616	11.20840189
8.331458747	8.125818909	8.83948901	9.473962505	8.591651626	8.309668384
8.789132293	8.068931465	8.124599212	8.073652495	7.608041616	7.872350636
8.06213903	8.544633697	8.342368121	7.678591667	9.031713778	8.168196313
13.93694444	13.25686564	12.91656586	13.04869312	12.54906863	13.04869312
8.284505182	7.417927455	7.561051303	7.108229697	7.482605354	7.501276949
8.89604703	8.775519707	8.885078273	9.450568111	9.121511687	9.220765354
8.491049758	9.070117818	9.193130899	7.875808798	9.215289101	8.884521697
7.837507091	7.105472313	7.181699919	7.629989283	7.474688838	7.67441401
8.836023374	8.957944414	8.209418434	8.257984293	8.621689657	7.972012263
12.55625498	12.42233701	12.62127559	13.58209631	12.56726296	12.65472241
8.838951081	8.682574626	8.643157737	8.749910828	8.733083212	9.139095455
8.11773804	8.749910828	8.347358778	7.432999727	8.559975162	7.988251818
9.424985495	9.541747455	9.258612929	9.835894606	9.671912455	9.681893121
8.260123323	8.817758687	8.692466525	7.65951298	8.625920364	8.266388465
8.393536232	9.376667182	9.189590182	9.600888859	8.848914293	9.047491869
8.892401576	7.844550919	7.949400121	8.13932801	7.436027273	7.677613273
10.64005918	11.20660223	11.38775416	11.12151875	11.93486375	11.93770961
8.468898455	9.346206162	9.269224374	9.434425707	9.509876273	8.942313313
6.409482747	6.898538505	6.232553091	6.006498545	6.16346097	6.403487758
14.33555643	10.56306707	10.48432644	9.019731374	10.06822268	10.72786832
6.706906717	7.825104404	7.378186273	7.309054838	7.518690889	7.825959581
12.96662998	13.7211242	13.76108056	12.54268587	13.56234785	12.68097339
8.865915939	7.860050667	8.082778869	8.023099172	7.158590636	7.496050374
13.82538175	14.04335209	14.84412351	13.13522491	13.84455043	13.70143341
8.267244596	7.636533838	7.538207606	7.725703576	6.902232848	7.068665737
7.783847071	7.19832496	7.099574394	7.750957677	7.498343283	7.630594101
14.05136004	15.26374094	15.18392271	14.7172184	15.12351424	14.7528858
7.086390323	7.222444626	7.184502222	8.011643808	7.66151798	7.332078838
8.432612263	8.36556403	8.649462798	8.326162172	8.598008343	8.912003131
10.51771241	10.39747702	10.56655729	9.353057535	10.35284213	10.31013098
12.20640951	8.155735586	8.037287293	6.843046434	7.320933798	7.88825004
9.513795939	9.584100455	9.919491525	9.694213162	9.638309758	9.607593848
9.260036485	7.413750677	7.456122566	7.804724687	7.398370889	7.686775222
6.276975525	7.074173121	7.047573414	7.134684455	7.156444778	7.191229121
7.687096788	7.66930299	8.168196313	7.781509414	7.969046636	7.528986283
7.377888556	7.102685081	7.074397545	6.149813737	7.180839586	7.211808657
9.189045596	9.841562192	9.565032515	9.402814081	9.844817354	9.422377343
7.77328201	9.496413788	9.287277576	9.249612495	9.196689051	9.286739242
6.833840465	7.145769434	6.869142343	7.598252121	6.897195879	6.982654747
8.374810828	7.284692949	7.078018687	6.959189505	7.687700545	7.528303323
14.64328928	14.48025691	14.49002723	15.97330865	15.06622261	14.85646221

7.819446808	8.249809909	8.011239071	7.988582505	8.257253131	7.872008071
7.743818828	8.175629323	8.058592364	8.612685364	8.519178475	8.307093606
11.0940021	11.43773109	11.87912023	11.59021288	11.73972102	11.67849095
10.156858	10.59418919	10.61867663	11.671419	11.54053762	10.90606985
9.686378667	10.18666124	10.18563382	11.19447199	10.7814083	10.65987528
6.782198505	6.439991313	6.322079051	6.244028818	6.052054152	6.230489586
10.16073454	9.025193869	9.17294002	9.136296333	8.903025495	9.101613848
7.229324455	7.587956192	8.005727263	6.859461465	7.606699253	7.865213
9.118629636	8.941359636	9.03221704	9.488337717	8.930433505	9.310205828
9.665316566	9.905108616	10.1985269	10.60161194	10.36687712	10.19957169
9.758431737	10.08599138	9.939327313	10.22070473	10.14895288	10.31193963
8.977005354	8.707515232	8.580722586	8.883414061	8.53670504	8.634788929
11.48258282	10.78551178	10.81796524	10.41426339	10.09994749	10.65365596
7.370383778	7.347734202	7.506434626	8.123844788	7.916712838	7.829325051
6.245298859	6.573072253	6.358115374	6.807771525	6.561505162	6.899979889
8.795325869	8.595283071	8.554027364	8.984231152	8.623636919	8.687541707
10.72900488	10.94071069	11.02243393	11.58005536	10.96311364	11.10761793
10.32750324	10.32946621	10.34193651	11.13085117	10.53247367	10.67815746
9.969141677	10.0060683	10.21375666	9.910960495	9.439397061	9.553430788
7.155827141	7.064243152	6.909401626	7.798826303	7.401155333	7.427086202
11.43938222	11.36610866	11.20345844	11.64244603	11.42178201	11.42562271
9.49850403	9.321890899	9.298556212	9.66831603	9.829822242	9.631182697
9.604846424	9.856174394	9.652169778	10.21583282	9.923658606	10.12224713
9.804037697	10.02061657	9.858738737	10.58347296	10.02061657	10.17030341
7.363924808	7.966742939	8.189895404	7.937437293	7.998937818	7.85604701
8.230996848	8.559456798	8.162707626	8.662363172	8.152794889	9.216574283
6.517646071	6.614759768	6.27216099	6.984018879	6.630444394	6.630903202
7.310724131	7.604109192	7.453623293	7.392836818	7.934739636	7.183404394
7.561964414	6.738515899	7.014842626	7.656877838	7.39712799	7.487940131
6.87893297	7.073903182	7.135537212	7.257820879	7.222960121	6.802328384
9.018299374	9.503858424	9.615324495	9.35796903	9.831087364	9.218466939
8.373514202	9.598265626	8.824851909	9.377908	9.854472677	8.930963202
7.470009697	7.326634626	7.283800475	7.851640596	7.658441616	7.830796253
8.803482152	7.868007081	7.860050667	8.604635949	8.039068889	8.542859455
8.914720172	9.224923687	9.383480283	9.934615404	9.690534253	9.390110384
7.854325061	8.061425949	8.138519566	6.625743253	7.898205273	7.878518051
8.112525414	6.395106727	6.657192121	6.778470374	6.839681778	7.126812859
10.57758511	9.014335657	9.251906182	8.98371896	9.171801828	9.370832535
10.56908161	11.11560115	11.2786408	10.74045328	11.05651949	10.67428006
9.806332162	9.826032111	9.731806091	10.23752804	10.16355358	10.14326599
10.71145754	10.17030341	10.5104744	10.33252085	9.929312889	11.31449746
10.67572435	9.909202788	10.20532801	9.565660949	9.140392293	11.13085117
9.84311402	8.101780545	8.057864374	8.303356141	7.763186	9.136296333
9.803356646	9.410728505	9.66383	9.474605101	8.83948901	10.28190294
9.958247111	9.784374313	10.05426836	9.773700283	9.244399414	10.75987116
10.7532413	10.29542336	10.61621963	10.30470526	9.600185283	11.68700199
9.829822242	9.766921414	9.926784556	9.47593803	9.062220818	10.69470446
12.12939166	11.96947069	11.84736958	11.44319437	11.1502402	13.55640443
10.06450841	8.986932636	8.974650333	8.673363869	8.82024202	8.826393101
10.99345288	11.24993851	11.63174178	11.43565115	11.50814936	11.34576518
7.995673758	7.943134	8.672348475	9.081927515	8.052077899	8.559011545
7.595301182	7.771143222	7.587005707	8.003903253	6.713538929	7.015344242
7.133560273	7.764199131	7.567065616	8.366003535	8.032957859	7.619556354
11.04316622	11.84027237	12.08680505	11.48459908	11.82346178	11.94979793
6.887014152	6.556900818	7.276013455	7.930615646	7.630295798	6.94072599
7.924885333	7.661901192	7.519990596	8.215875455	8.129067798	7.825440475
9.330464051	9.726443586	9.87177298	8.747892899	9.314493212	9.291911616
7.204326061	7.641344242	7.519364889	8.239938859	7.732919727	7.799854778

8.676791091	7.187213162	7.364550071	7.492937667	6.968936475	7.091592101
7.49864798	7.225001677	6.767752323	7.033625222	7.199149121	7.474059606
7.086968939	7.454260455	7.849463182	7.671593667	6.823157384	6.654303071
10.34418014	8.96575796	8.762202232	9.171164091	8.511758747	9.067856838
8.478760515	7.460591212	6.962306323	7.233650202	6.845030273	7.01049403
7.013137848	7.223246162	7.330546788	6.496794495	7.241538444	7.621022111
11.20498985	12.27101	10.68687892	10.80644997	10.75987116	11.21187068
9.809529798	10.49592664	10.33989796	10.05780386	10.30058205	10.02439619
12.02240324	13.07454735	12.83405786	12.43600068	12.61761735	12.51766186
7.935081758	8.528512253	8.879851283	7.908308323	8.462236515	8.471953717
11.8358519	11.76101506	12.18792926	12.21630458	11.96059797	11.67371856
6.906597848	6.975978909	7.215185141	6.884803323	6.623921737	6.726701909
8.442251212	8.066048727	8.112525414	8.336257222	8.379056919	8.156772778
7.918472515	6.487263202	6.714232707	6.495011879	6.411697737	6.662501485
9.171164091	8.980444384	9.229670424	8.786134101	8.990220404	9.251906182
8.809951364	9.241421747	10.02923217	10.70052069	9.241421747	10.63870636
9.379180747	7.275223848	7.517772121	7.240898899	7.409045616	8.246913576
8.030614293	8.530767747	8.304980606	7.794025323	8.93638097	7.717447071
11.11388317	8.919352101	9.424273283	9.226702434	8.783584889	9.592426051
10.04160563	8.78767504	9.169326323	8.709941838	8.351752182	8.602379626
10.4429644	10.77387872	10.62764398	9.636929838	10.70425349	10.042425
10.06547375	8.971160737	9.417037424	9.50650501	9.300983919	9.70574997
6.780688182	7.467830485	7.240898899	6.830578646	7.246699182	7.661155364
8.334981101	9.530205505	9.283145788	9.491039162	10.01622438	9.11093099
8.434412929	8.825911939	8.869521111	8.876650909	8.960773354	9.012031232
7.822322859	8.130616515	8.130993828	7.154661646	7.996041859	7.937437293
11.81221198	13.06967433	12.69316533	11.45176908	12.63500423	12.2293359
9.582761798	10.41846051	10.27804811	9.012031232	10.10841911	10.28710845
8.891888576	8.409136758	8.219387636	8.923576949	8.575095707	8.588536172
10.31597323	7.915639949	7.970233808	7.862492343	7.559825556	7.820605525
6.788535525	6.836753071	7.045230232	6.767263616	7.081067566	7.108809091
11.67849095	8.554278051	8.749346626	8.818695101	8.431738505	9.336479586
6.356904333	6.76944904	6.430701515	8.827377505	10.70775781	7.620272192
9.080683394	8.650384111	8.741130788	7.865549818	8.494576061	8.560348606
7.875433545	7.879985051	7.91307104	8.553132192	8.461315273	8.52575703
9.266794162	9.137373131	9.959124273	9.439397061	9.767642525	9.293068828
13.27940818	13.80704919	13.92172837	14.1282639	13.85099976	13.81935116
9.95201103	8.542859455	8.899757485	9.348136535	8.969483	9.025691242
9.133284677	9.286739242	9.385290182	9.642500081	9.627013505	8.739635162
9.841562192	10.6768734	10.54692936	10.07107569	10.60507136	9.786685869
9.40167604	8.60560303	8.700142556	9.29794999	8.865407737	9.386484303
11.28731295	12.02514953	11.57567047	10.90057618	11.73271713	11.01376888
8.39308204	8.926362727	8.614861051	9.124970051	8.446184828	8.420871687
7.515531818	9.403514808	8.344098929	8.941795242	7.849137545	7.828260424
12.69676846	13.29117716	13.42153085	13.80033164	14.1904887	13.43523914
8.165380303	8.000883182	8.128726778	8.107270071	7.711891616	7.803554182
10.29000276	10.95849458	10.65014074	10.53141254	10.25422487	10.52585606
7.638981707	8.499836475	8.999492384	7.226157253	8.188779434	7.504556444
8.817758687	10.27905981	11.00902763	8.532516465	10.06450841	9.184000242
9.488337717	9.335891293	9.379180747	9.789131121	9.545191596	9.583475303
12.61449239	11.7349036	11.42787377	11.06732533	10.84974643	11.70812672
10.60863639	9.961780586	9.888393747	9.990505182	8.905887879	9.16271003
6.76309904	6.879719566	6.901464747	5.998729768	7.007335798	6.777485293
7.304125475	7.896955242	8.190353414	7.987188889	7.721939444	7.538207606
9.930141081	9.606298465	9.844817354	9.208914778	9.466909636	9.574050242
9.091299889	8.946596606	9.088505869	8.160644788	8.515864333	8.534897646
10.15379546	10.82416773	10.65634987	10.94071069	10.65751844	10.47123164
8.612685364	8.878201818	8.96575796	9.905108616	9.442729677	8.871686232

12.70787607	12.77015535	13.07947925	13.78027417	13.29684189	13.03957854
9.071276071	9.876096838	9.830354566	10.29660096	9.629101929	9.642500081
7.33379003	6.553884717	7.157253707	6.952257202	6.621786404	6.765091091
8.097566687	8.036816444	7.965660091	8.077645899	8.012010707	8.261781515
12.81735602	13.92172837	13.80704919	11.1060681	13.00581854	13.47327752
14.67552064	15.01206542	14.94534759	15.26374094	15.38120792	14.74028573
10.36349344	10.45041513	10.52904685	9.730279475	10.56207051	9.902579253
7.845242687	8.096128192	7.924502232	8.002342131	7.302140758	7.366646525
9.863774293	10.63266161	10.4302696	10.80499832	10.68960982	10.62536143
10.83501466	10.89604789	10.86820493	11.64848719	11.86401838	11.29744903
7.942071303	7.933221424	7.901142576	7.837840162	8.076845152	8.313601758
6.765793475	6.557543268	6.639490384	6.550562869	6.333400636	6.315590828
9.650789909	9.445197818	8.78563097	9.010430586	8.423588242	9.412044242
9.463745455	9.702015293	9.698181576	10.03796113	9.895061889	9.934615404
8.889922455	8.603276828	8.383429707	9.01707996	8.626355717	8.607626293
7.31258798	6.567684242	6.518533202	7.071486061	6.972885929	6.753729404
6.988144556	7.329870525	7.852302727	6.326292525	7.094450071	7.521885657
8.377562293	6.895709172	7.050890818	7.249695535	6.638362242	6.844259293
10.12314196	10.70534521	9.93099102	9.591816061	10.24227579	10.29101087
10.31480319	10.8947391	10.4322159	10.28190294	10.65127749	10.77637516
11.71042621	12.25844332	11.97196754	11.43773109	12.00086001	11.85301976
8.293769586	8.377562293	8.135526051	7.994228667	8.094218354	8.396963808
9.160413333	11.32559397	9.702758343	10.67428006	11.35080354	10.70425349
8.308776677	8.345353354	8.389243343	7.851263121	8.925792808	8.019097616
13.67554111	12.13244857	12.21630458	11.88375452	11.93178106	12.13538265
6.866319192	7.583321859	7.502729737	7.634532263	7.59498599	7.552449879
9.733950273	9.675185677	9.758431737	9.769994879	9.776037091	9.903372323
11.55322016	10.15875628	10.34830463	10.28506122	10.22951336	10.44832821
10.71860391	10.33596201	10.24612484	10.93023415	10.61261659	10.56548536
11.74935785	12.08128174	12.01427079	12.27773509	11.88909817	12.04990566
6.811363364	6.981850818	6.987320737	7.495760485	7.381027636	7.344278889
10.26541026	10.73661507	10.63266161	10.70425349	10.45628541	10.47820756
10.85513517	10.06822268	10.31894523	10.06173934	9.942152202	10.36239309
11.15966862	11.29076429	10.93023415	11.82112126	11.21338774	11.3290504
8.899757485	8.842109	8.933180404	8.904239657	8.913588253	9.086722374
11.53677901	11.27695531	11.39772243	11.08264222	11.03163187	11.56291631
9.254253192	9.833438364	9.631182697	9.069043081	9.274850788	9.669847545
10.65500785	11.33657706	11.38178745	10.72384747	10.7532413	11.35858946
5.758280051	6.018680697	6.627836202	5.764992293	6.655986263	6.378573141
7.970926879	7.842359758	7.499887141	8.706127333	7.67303801	7.889381343
8.343220798	8.738283333	8.675789182	9.094076404	9.07419996	8.749346626
7.194186434	7.626110576	7.795116566	8.334981101	8.316969364	8.071334404
8.016429949	7.223856	7.048729333	6.621412929	7.382821899	6.889250929
9.600185283	9.302735434	8.694292242	9.003925646	9.050797061	9.142159707
8.427685838	7.819085697	8.375668162	8.707057788	7.870669919	8.208296687
7.515816273	7.101573333	7.684641212	7.993077394	7.301506111	7.595301182
7.70299303	7.56574702	7.302140758	7.214035101	8.494130061	7.365381232
13.86604428	10.042425	11.01045685	9.526200354	10.68338253	10.4524716
12.97977545	11.26446827	11.19966733	9.094691273	10.42622906	11.59021288
8.644074768	9.051375475	9.024212859	9.140978061	9.126663535	9.203760495
10.53505365	10.02439619	10.32418729	9.951175646	10.2692332	8.949784505
7.97284099	8.396963808	8.273061586	7.892936636	7.989587677	8.494982343
7.081850444	6.320887909	6.645215414	6.781179727	6.277726788	6.37918304
11.04464523	11.17378617	11.45526933	11.39382595	11.48840455	11.09565519
7.655611	7.958031758	8.083485404	7.886820596	7.582966818	8.07563398
13.15082863	12.90783816	13.33661988	12.89886333	13.62784329	13.2158469
8.482406475	8.283330131	8.868979273	8.595283071	8.248986687	8.375668162
9.207791152	9.692043475	9.913634949	8.840010495	9.825199323	9.80784303

6.191967758	5.975084869	6.326292525	6.284133697	6.465818222	6.197177
9.332795131	9.59460096	9.650789909	10.48897537	9.839111	9.980047091
11.30690717	12.02240324	11.60266156	12.29548604	11.84027237	11.89130603
8.668536687	9.647013263	9.618256808	9.751080646	9.701165495	9.698181576
7.387965737	7.468494556	7.368501293	8.013857071	8.065153556	7.461509737
6.075089566	6.084661768	6.564945162	6.785769293	5.988232071	6.028808465
7.157774636	7.218399283	7.787863889	6.261563212	7.87816304	7.862160495
12.28105924	12.52819783	13.16824615	11.20498985	12.28985985	12.36473556
9.453123465	9.179226364	8.979921919	8.668931657	9.423026242	10.09795649
7.647964475	7.456765576	7.463713737	8.113343889	7.802836333	7.74753198
7.905781333	7.823083152	7.912365798	8.129067798	8.20265299	7.796747616
8.616405606	9.046362323	9.012031232	8.068931465	9.023639636	8.932594273
10.42175216	8.914132333	9.096947232	8.19621002	9.035040848	8.787164939
12.08680505	12.0724233	11.18941159	10.92190902	11.71244686	12.28369472
8.741130788	8.29635502	8.810986707	8.144584758	8.097566687	8.104133364
16.25437967	15.22815255	15.63778652	15.78787594	15.63778652	15.72204116
8.437457535	7.279114576	7.402347162	7.356542485	7.033114263	7.370079717
9.087865687	7.937437293	8.416773909	8.323157747	7.444823313	7.846992646
11.59021288	9.703591263	10.12041459	9.564343333	9.571117879	10.19649023
7.160253414	7.809001414	6.656891394	6.547113253	6.944866586	6.825565162
6.551639909	7.226157253	7.647332323	7.376100929	6.780688182	6.780426
9.257979848	10.16546821	10.17122401	10.43440935	9.780583495	9.597513697
8.084275677	8.32856404	8.549970081	7.835788293	8.534432515	8.627796121
9.895061889	10.6488688	10.77387872	9.210065	10.59541023	9.941224515
10.43655428	10.87379507	9.691258919	9.792275424	10.49348589	9.812775374
10.16454544	10.55842197	10.68572341	10.40387449	10.53141254	10.14713571
8.877626323	8.50473503	8.248986687	9.164545343	8.288815495	8.69335002
9.559682182	9.17294002	9.303342798	9.17861001	9.162016475	9.25368898
12.08943903	12.04432	12.2293359	11.95514832	12.19086998	12.15130062
11.37847359	11.55106122	11.55524693	11.79405389	11.59427705	12.0724233
9.767642525	9.688441414	9.880317061	9.072998758	10.09426568	9.973623707
8.253639283	8.381309202	7.771866444	8.136235949	8.473392556	8.486543404
8.362537455	7.747112485	7.717737636	8.308776677	8.347358778	8.229734556
9.534194535	9.252443646	9.293710889	9.724047525	8.964642636	9.441908576
6.852936051	7.206373697	7.156711091	7.316995263	7.247912131	7.312858101
6.949608808	7.25994097	6.984018879	6.597017333	7.571747222	6.749084333
11.96059797	12.62449502	12.32024961	12.2293359	12.31089415	11.96947069
9.810255869	9.645545465	9.718307192	10.31480319	9.687048303	9.825199323
10.68452674	10.82804231	10.80389925	10.11150219	10.25519474	10.82945836
11.76336308	12.69316533	12.54268587	12.75396286	12.63888703	12.12436236
7.900715717	8.984231152	8.823394192	9.339764051	9.263233333	8.804477505
8.383846919	8.095746162	7.981345788	8.028563859	8.222643394	8.143776848
12.99565877	10.26632574	9.730279475	10.42297323	10.25627793	10.60863639
12.80949032	12.16627503	11.99535117	12.94367069	11.9982543	12.57877126
11.24646499	10.43655428	10.46442497	10.1985269	10.41957053	10.47941256
10.83642757	8.998971485	9.388912091	8.946596606	7.89464897	8.805485081
8.017912586	6.350448798	6.571898091	6.497416687	6.418892273	6.80697597
9.494490616	8.694840596	8.797743222	9.009407333	8.31049196	8.819236758
12.26162026	13.30795266	13.09045797	12.72219022	13.18348162	13.18348162
6.96434804	7.198026636	7.509397909	6.73803103	6.471263556	6.891529131
8.917069505	9.20200202	8.514909313	9.205404404	8.546545061	8.703161646
7.697940283	7.631889737	6.742510061	8.033283465	6.834834778	6.884060404
7.154661646	6.758395253	6.792041707	7.724673141	7.64030596	7.133007667
11.88375452	11.2518391	11.04156144	10.78856661	11.15342364	11.30301738
11.8164662	11.54470354	11.71443529	11.70557621	11.50425191	11.55524693
8.821324343	8.668062838	8.548679899	7.454874889	8.502509434	8.632349838
5.751208162	5.754657061	5.752755323	7.130177747	5.749934667	5.750817394
7.531511131	5.930000051	6.583409828	6.034603152	5.986014515	6.188910586

7.730484909	7.924172566	7.924885333	8.094605657	8.687541707	8.268510495
9.206631485	9.273022949	8.69919198	9.969958172	9.55462702	8.176718545
9.400967465	9.053043889	8.907429495	8.254484434	8.734416606	8.701109788
7.85507	6.626676333	6.737335172	7.392563535	7.579015667	6.908074727
8.417604414	8.32400903	8.670884566	8.394903626	8.640789455	8.500240172
9.084956182	9.479751879	9.408173051	8.451947071	9.363307667	9.072998758
11.72592113	13.2158469	13.18348162	10.85654613	12.38063147	12.62449502
11.22947614	9.706432808	10.00886723	9.957374697	10.08521343	10.18666124
9.212349869	7.44112097	7.447087758	7.245014414	7.360451687	7.809338131
9.721175788	10.51291939	10.57996368	10.49140257	10.68072859	10.51771241
10.0178996	10.33890802	9.853021111	10.58812248	10.26116661	10.28899471
8.419470808	8.032957859	8.252372808	9.345614434	8.585836293	8.654464586
10.27432009	10.23948175	10.27905981	9.509876273	9.839882283	10.05236285
10.41731261	10.6240143	10.68960982	9.948421707	10.29432836	10.43122177
6.817526586	6.667348717	7.174161374	6.429483727	7.096649162	7.120197273
6.327126061	7.048974677	6.748324394	6.617377051	6.922412606	6.605935465
6.758967323	6.66759498	6.135589202	7.019921606	6.431827616	6.539575101
8.249809909	8.970028374	9.07419996	9.799317404	9.444057586	8.746767313
9.683334444	10.73517618	11.14385742	10.17336172	10.77252405	10.55966675
9.749660394	10.18563382	10.01071956	9.916104475	9.980047091	10.18468509
9.331038253	8.048299333	8.923097444	8.60413102	8.118879606	8.59942799
7.634532263	5.988108535	5.906986101	6.069331687	6.207117515	6.651718455
9.957374697	10.11349335	10.39072525	9.733950273	9.876096838	9.733230051
6.951169364	6.397207283	6.135409394	6.327126061	6.654064545	6.452349424
9.545191596	9.875361242	10.0728998	9.771430818	10.14044783	9.656571869
8.517288667	7.358375414	7.148774485	7.12322297	7.354804152	7.251769333
8.010919919	8.700142556	8.653906556	7.589252838	8.41382397	8.443547162
9.337187111	9.062220818	9.033394545	9.482360616	9.341818545	9.379720636
9.870221061	9.789981192	10.06173934	10.18666124	9.816676162	10.0406788
7.630594101	8.090488788	8.164230707	8.622644364	8.079553929	7.838173152
10.55333109	8.889380424	8.63611798	8.173633596	8.449337667	8.713417869
6.516492273	6.651970061	6.365312818	6.669677444	6.819189677	6.985416596
8.373157505	7.955993172	7.719108384	7.639261768	7.220240556	7.629016404
7.527697455	7.099574394	7.488894061	7.990313949	7.505451222	7.800216455
7.118793697	6.59504903	6.751729354	7.407119727	6.690572232	7.008962394
7.989204111	8.003147788	7.991473828	6.888722505	8.072924455	7.759936788
10.20634509	10.69793218	10.78269205	11.13551505	10.8935809	10.64623997
11.17378617	7.927113394	9.087865687	8.609952768	8.465000141	9.150811232
9.612498475	9.298556212	9.359207242	10.13580598	9.596088263	9.91449603
8.736738475	8.493155899	8.568768141	8.854603364	8.757866131	8.768863636
9.736957071	9.572453101	9.477759242	8.91530399	8.98371896	9.036569232
7.943747505	7.670568384	7.726017717	8.020484273	7.915639949	7.987573434
8.727989172	8.495994869	8.603276828	8.982611727	8.700142556	9.128533354
11.21187068	11.17213305	11.1060681	11.62739451	11.57567047	11.33100665
8.135177152	8.285312424	8.228946717	8.867918475	8.221010253	8.571194222
7.729749768	7.656238101	7.916025434	7.370383778	8.210602626	7.883157929
9.116279576	8.006495333	8.08193004	8.294235768	7.470329323	7.447717303
6.59116396	6.600294051	6.01113301	6.29488699	6.121897343	6.09177099
9.009915859	7.262957343	7.856753939	7.295094879	7.143440162	7.681488071
15.81050193	13.87958112	14.29173122	14.36999747	14.19920898	14.5413574
7.213178616	7.790604293	7.802180768	7.089925434	7.865549818	7.884314808
6.331869444	6.535299384	6.133322131	6.11789698	6.394492141	6.265203717
6.92551101	7.32157099	6.769202101	6.874988444	6.891529131	7.126519737
9.606954152	9.397189192	9.433849505	8.349393212	9.305189859	9.381544081
7.660499434	7.707249808	8.05903404	8.027802949	8.030181424	8.244006505
11.02243393	12.14789109	12.03310273	11.69440164	11.83072395	11.41981305
8.620790505	9.404333879	9.393934131	9.224923687	9.484209455	9.348136535
6.92802298	6.704451293	6.931948737	6.678083162	6.348442212	6.779180051

7.481645051	8.185563303	7.566773798	8.242002535	8.451947071	7.585232141
10.16937171	10.71031814	10.68338253	11.33477589	10.92060078	10.96162926
10.61867663	10.60635433	10.44195016	11.45002291	10.75178718	11.06123261
11.95261936	11.81439214	12.10951308	12.91196246	12.18792926	12.82558839
8.346989566	7.518430525	7.56574702	7.427086202	6.965717616	7.349181162
6.839681778	6.059487707	7.125696242	6.574452879	6.120069909	6.279712293
7.323994646	7.897743687	7.668279394	6.889757091	7.79851096	7.492937667
16.02065153	14.36070414	14.40789433	13.30795266	14.33555643	14.47046648
10.2600862	10.59924244	10.5752557	10.17789322	10.73264046	10.54078574
9.117478424	9.133918929	9.320738646	9.32739897	9.544495798	9.344897333
7.348317141	6.117232192	5.760389909	6.585495889	7.23423804	6.379570131
9.153730283	7.846688253	8.809951364	8.718769576	8.205892515	8.514431737
7.97128602	8.144584758	8.000883182	7.468494556	7.456429202	7.894315778
12.95303817	11.671419	12.4571909	11.94481318	11.76588718	13.46712151
6.256172131	6.537260646	6.669931545	6.36270599	6.222147	6.290882545
7.657544313	7.060219081	6.471031303	6.968393455	6.585931495	6.652654
9.94402302	9.953782444	8.655437626	10.36781996	8.684654737	9.084956182
14.13521971	13.32021166	13.85817003	14.58095582	13.68884286	14.23242051
10.05780386	6.936612586	8.048299333	9.275532697	7.754407434	8.476994374
7.67197899	8.505287909	7.502072596	8.516311424	7.321884212	7.536554758
7.116617232	7.373116051	7.257820879	7.245604273	6.271979525	6.408579424
13.56886427	11.55928848	11.80104424	11.74670945	11.65455063	13.5160991
8.969483	7.635267465	7.881467646	7.843855828	6.967457677	7.759566061
8.293340606	8.462236515	8.232163879	8.212685152	7.029601354	7.252886707
7.21064097	8.012346152	7.008416879	6.69943696	6.163109394	6.391416354
8.102484232	8.27861104	8.387507626	8.798818646	6.710071909	7.20129803
8.935320828	8.085049192	8.250194202	8.143419303	7.964547263	7.984708455
8.594455404	8.611710212	8.597027768	9.051375475	8.230588212	8.251125323
7.197763283	6.715208525	6.997949788	7.726017717	6.270843101	6.561505162
9.273640758	9.049629657	8.925792808	8.424471616	7.858214495	8.369167818
6.810861495	6.039785808	5.995703788	6.309281687	6.111914111	6.249618616
6.524953909	6.980238687	6.785769293	6.973416747	6.137175152	6.029217242
6.648620667	7.28082202	7.111135707	6.520115535	6.674729293	6.864987879
7.997183333	7.342557798	8.265965545	7.822730929	8.404822414	8.171342424
7.410944101	6.487487818	6.282381596	6.539575101	6.012078172	6.110289768
7.551492101	7.491021444	6.972058485	7.438305869	6.419328293	6.701799071
7.423442323	6.748324394	6.563630475	6.502086778	7.368198747	6.410700434
8.466446051	7.618887111	7.820934747	7.57318396	7.267388222	7.435155859
7.382821899	6.522975131	6.159167768	6.100757848	6.153019566	6.216046091
9.479084687	7.180291929	7.36694602	7.297445616	6.808256808	7.271131505
6.802811828	6.744072859	6.030939667	6.631584848	6.31049204	6.139596152
8.908475283	7.649926505	8.454049747	8.003479939	7.439506505	7.514494576
7.07011003	6.866319192	6.356285141	6.685307768	6.12238997	6.320680717
9.371514253	10.20748734	10.33696433	8.432150758	9.957374697	9.885156737
7.675499444	9.464440242	10.74690378	9.580783667	9.083185818	8.882882525
7.06161799	8.272620253	9.35858599	8.352134333	7.805761556	7.876807081
12.10414281	10.78269205	10.68452674	10.79564384	10.39641485	10.53505365
9.322592778	10.63391466	10.26541026	8.698751323	10.22613156	9.508442768
8.480536717	7.671246505	7.863195747	7.556302505	7.85268101	7.897352323
8.842374101	9.207219232	9.23383296	9.315671101	9.447242061	8.856895172
10.95683616	10.46108665	9.90675497	10.14713571	10.75600216	10.64128512
8.790130253	9.39974104	9.415217505	8.71485401	8.949784505	8.576891556
9.223302313	9.40167604	9.422377343	9.704210374	9.030004576	9.542425758
8.187593091	7.776293606	8.014979636	8.036816444	7.973227556	7.440763838
10.22668357	9.718307192	9.857104919	9.663106646	9.410728505	9.311440606
8.929972798	7.444823313	7.798826303	7.215185141	7.014842626	7.412806768
8.059832677	8.030614293	6.760600303	8.938439909	6.924186162	7.376100929
7.671246505	7.018881949	7.005077596	6.914278354	6.388351374	6.636058242

8.60560303	7.636262586	7.784529071	7.77977198	7.334908263	7.850180343
6.627836202	6.719039212	6.841827051	6.515423162	7.258984768	6.632573485
7.180559343	7.072297535	6.710494808	6.010087253	5.916494313	6.209212768
7.157499455	7.007051354	7.020975202	7.729367111	7.314025869	7.560401535
10.91176188	10.26445444	10.68072859	10.33989796	8.996667384	9.438094949
7.936282586	7.919797354	8.160644788	8.642670768	7.883917657	8.359263212
10.18563382	8.167813576	7.903688606	7.811684818	7.937437293	8.134790222
8.549517636	7.141446111	7.59859104	7.901561475	7.575420636	7.853350485
7.00317901	7.995673758	7.898974697	6.827154646	7.424927172	7.404051424
11.12315281	10.60161194	11.3290504	10.93494087	9.625623061	10.54950911
9.885156737	8.526649808	7.788532485	8.825911939	7.701991131	8.360501364
7.147872253	7.712213273	6.526793081	6.890996051	7.569899606	7.774939586
8.781680606	9.337862091	9.264989899	8.177528808	9.453743545	9.444057586
9.081281455	8.769354545	8.019769071	9.095762	8.781680606	9.917043434
8.604635949	8.228946717	8.004655889	9.191849071	8.840590677	8.145617202
8.292509485	7.95873603	8.430929525	8.87289497	8.563941778	8.69919198
9.536330707	10.12224713	9.832711535	9.972781707	9.951175646	9.677492576
11.09089144	11.09721747	11.03580591	10.58117749	11.1453264	10.85654613
5.769083091	5.882450778	6.289157818	6.35978	5.784943869	5.782370646
13.30795266	14.13521971	13.81266894	13.99495772	14.35197184	13.84455043
11.11081747	10.7442872	10.45150222	10.81423156	10.88166557	10.23948175
9.455015404	7.175476838	7.741457929	7.628690051	7.471589222	8.183195545
8.098747556	8.05474504	8.387939343	7.341038	8.148682091	8.128290707
7.828584192	8.691458141	8.559456798	8.808928141	9.100524758	8.470647758
7.376746737	7.996041859	8.32400903	6.861163889	7.942071303	7.542064859
7.281402384	7.677264869	7.728044798	6.606438778	7.762451808	7.247290424
8.221794202	8.94874	8.787164939	7.878949444	8.521696414	8.533396525
9.965319232	9.284395657	9.41003803	10.44712342	9.407554697	9.520598525
8.279889293	7.172666465	7.511838192	8.430929525	7.679634566	7.666324061
10.05144087	9.318836889	9.479751879	10.62536143	9.417594202	9.552159131
11.97196754	12.06964699	12.24933389	12.72972704	13.07454735	12.71520416
8.690381253	9.210065	9.31393701	8.794361798	9.636929838	8.910531141
8.882882525	9.093526566	8.851831626	9.400967465	8.844262616	8.795325869
8.30460302	7.267645253	7.31189602	7.311037152	6.790568566	7.090758091
7.404355202	7.348914263	7.282945384	6.857502071	7.318216242	7.372842667
7.765298909	8.000144121	7.826504869	7.78118699	7.125146293	7.058212263
12.28985985	13.27940818	14.08841453	14.08841453	13.80704919	13.12562627
7.193299919	6.996042242	7.520992313	7.69185196	7.328995606	7.253486596
9.028300162	9.190767364	9.37540303	9.727055768	9.919491525	9.66831603
7.464044657	6.698966242	6.681366485	6.916922333	6.306085808	6.449076475
8.233006697	8.422204465	8.721054424	9.002272697	8.739178636	8.671294253
9.297353606	9.220211768	8.526649808	9.59387599	9.34289298	8.151247717
6.226967616	5.795144121	6.498363273	7.170232747	6.286107737	6.361759
11.07013707	10.24828231	10.24727703	9.824540899	10.18000397	10.36781996
10.03611568	9.704210374	9.466304111	10.40925212	9.719000626	9.752732475
12.06692054	11.60479972	11.66189432	10.95533553	11.37490296	11.52645916
7.920877394	8.723452192	8.401710747	7.962680434	8.373514202	8.448929687
7.494798263	7.181699919	7.227304778	7.490096869	7.594027929	7.714004283
8.437898414	8.466852202	8.549517636	7.886480404	8.409548323	8.427685838
9.024212859	9.168324747	9.437491071	9.344138121	9.36439596	9.935661677
7.091592101	7.945472162	7.676615465	6.845030273	7.359873626	6.965717616
9.094691273	9.66613299	9.438094949	8.180413081	9.312727606	8.881310737
7.933221424	7.908308323	8.286610323	9.278597747	7.706168626	7.441392919
6.830079818	6.810307121	6.975426091	6.868107182	6.672562737	6.722042727
9.272374697	9.029402949	9.015966535	10.21769501	9.275532697	9.559682182
8.841575667	9.923658606	9.831834717	9.177504333	9.823678374	9.11154599
9.305189859	8.864430475	8.667049253	9.158023	9.020337111	9.198436465
7.796747616	7.647332323	7.869396606	7.940608939	6.770611535	6.905091242

5.804599545	5.945080303	6.315590828	5.794853798	5.837564172	5.77831096
7.632188616	7.359287172	7.481960152	7.218159515	6.46756804	6.780217071
6.751169162	6.462162828	6.475135818	6.221395899	6.127679485	6.11854299
8.207537162	7.710578727	7.821271606	7.808339242	7.304711495	7.531209667
6.737819111	8.068564879	7.542431869	7.64232702	6.746121293	7.048124798
8.686679798	6.243651848	6.648620667	6.712342556	6.494395354	6.977041091
7.729067364	6.491493939	6.724536455	6.490008293	6.423456465	6.373918293
9.266188263	8.577417788	8.69335002	8.855784909	8.120795899	8.163923192
6.791503909	6.843545424	6.012730253	6.688683899	6.260478586	6.174981131
7.731871869	7.948048111	7.976116929	7.198026636	7.386173242	7.458509535
8.45110203	9.588958727	9.749660394	9.382205394	9.491039162	8.830117929
10.66945215	10.31795252	10.42736719	10.71145754	10.9991534	10.86820493
8.919862162	9.318228697	9.432003333	8.946107263	8.959130566	8.864430475
9.085558838	9.011546121	8.815152444	9.020821455	8.807484626	8.71185199
12.39348455	9.220765354	9.513795939	8.858269384	9.026117657	9.368875727
8.003903253	8.447526838	8.923576949	8.695335444	8.305787747	8.451947071
7.302140758	7.41439101	7.204880737	7.185402798	7.608347495	7.251486172
11.00183361	10.53247367	10.85654613	11.04316622	10.63516273	11.43005301
9.757590192	8.300103657	8.628622212	8.949784505	8.680072242	8.553595747
8.41552702	8.189895404	8.533396525	9.042346232	8.551751717	8.345353354
10.88430477	11.95261936	11.89628941	11.78023213	11.39772243	11.45932673
6.675987374	6.908606212	6.922161646	7.480348535	7.469040515	7.186028475
7.070666535	7.730484909	7.812003626	6.807473404	7.850535525	8.213079374
6.624585818	6.013395242	5.825403596	6.045111384	6.065061232	6.586655667
10.00886723	9.850553616	9.91449603	10.62647628	9.829119768	10.22070473
7.896287434	8.293340606	8.484723788	8.962353222	8.677707162	8.671783687
8.593101434	8.689808788	8.745474636	9.567720788	9.270407879	8.991410131
7.096340343	7.561342071	7.035145869	7.138578667	7.739314727	7.106302455
9.966143818	7.80651396	7.571427586	7.106035242	6.76459802	7.389870293
10.15875628	9.334658071	8.282073859	9.339095222	8.57790302	8.876209859
12.54589185	12.91656586	12.91196246	13.15645152	12.60022648	13.14611959
11.38970236	11.05033578	10.9889639	11.77053658	11.0473733	11.17910752
11.69188536	9.994772242	9.715870253	9.517013525	10.04963797	10.29324401
9.715215101	9.58708204	9.505859384	9.18155302	10.03299879	9.824540899
9.447991384	10.09254304	10.19957169	9.552821717	10.36134852	10.1905069
8.869521111	8.440878707	8.82842597	7.817975838	8.382194192	8.705608919
9.708693071	9.169862626	8.91530399	9.507772687	9.658772677	9.004940778
7.556302505	7.05790099	6.672296636	6.766293242	7.263531485	7.485478828
8.890373556	9.524763869	9.314493212	9.739033131	8.904793061	9.473962505
7.827504414	9.312727606	8.680513909	8.070136677	8.256499515	8.028563859
13.40302459	14.6970922	14.58095582	14.37933382	14.26676228	14.40789433
9.36570098	9.019287687	10.11664425	11.39185194	10.17440526	8.562227051
6.810307121	7.40526796	7.280239242	6.87945998	6.864987879	6.760600303
6.86214797	6.876372364	6.79736498	6.981618121	6.446375626	6.57397498
6.705415545	6.935285707	7.210929778	6.935285707	6.869371495	6.619037364
9.934615404	10.84176532	10.47584162	8.736738475	10.31894523	9.712341424
10.02439619	8.514037303	8.554528737	8.327767232	8.149788848	8.157901343
7.350681061	6.914008404	7.021874485	7.474059606	7.114645596	7.442908828
9.744663444	9.499247869	9.54312803	9.188424626	8.942756889	9.063885091
11.06573849	11.11714857	10.88809182	10.26832521	10.72516317	10.61743608
7.770465818	7.230593889	7.073141899	7.198896253	6.514559495	6.582941808
9.212980727	9.556759333	9.750460616	9.372025394	9.746868596	8.988577162
10.34514895	11.10460602	10.95234717	9.905938879	10.6313464	10.11760527
12.73352188	11.08107282	10.85513517	11.5612151	10.81796524	11.5612151
9.116804101	10.0719806	10.09339803	9.461673394	9.838413929	9.473222667
8.162707626	8.502042192	8.914132333	7.723009	8.528512253	7.696877364
9.024707707	9.146732444	9.413321525	9.466304111	9.70574997	9.466304111
7.855731	7.659822475	7.926300626	8.528915121	7.789573343	8.187593091

6.691558677	7.355080212	7.403028737	8.474228909	8.101068051	7.966054535
9.488971182	9.332175414	9.354902374	10.18000397	9.921211657	9.809529798
15.70163214	13.15082863	13.32021166	13.02006671	13.47327752	13.58996905
8.911610283	9.724814657	9.624391323	9.209526556	9.683334444	9.227886646
8.971160737	9.305189859	9.525471414	8.364710758	9.097596465	9.179819687
10.03876748	10.01703805	9.751080646	10.80239406	9.982665242	10.06071544
9.019731374	9.595333525	9.50710198	10.20164867	9.265583747	9.853021111
7.239071505	6.909148313	6.960749798	7.168103949	6.926221848	7.18313201
8.260520646	7.721580051	8.100222556	8.495447152	8.007258162	8.153957263
8.812013889	8.612685364	8.718321818	8.157901343	8.863375313	8.995099212
16.33688632	15.31193978	15.19830883	14.80749806	15.48449671	15.59877501
9.065009323	8.701109788	9.406893465	8.372433475	8.486053202	8.59026096
9.761765848	9.53973704	9.588410576	8.984797889	9.752732475	9.296718121
8.275152535	8.496365737	8.289212586	7.487940131	8.355847778	7.997183333
7.891912283	8.295009141	8.447526838	8.832790172	8.927565404	8.402589283
8.333227545	7.480029465	8.460488909	7.941721242	8.59942799	7.788923101
8.024267525	7.938531869	8.06213903	8.818210192	8.521230828	8.309247939
9.204345657	7.084144263	9.397189192	10.17030341	9.806332162	7.413750677
8.856895172	8.554971	8.651919778	8.106019798	8.08112003	8.361327455
7.713612677	8.354947869	8.30620801	8.530333535	8.679110091	8.248986687
9.558925838	9.517013525	9.39974104	10.28005063	10.07580535	9.778131475
6.304082879	6.780688182	6.66759498	6.408811828	7.035145869	6.574185192
9.001802606	9.069043081	8.597538556	9.159785394	8.597538556	8.793339475
8.919352101	9.023071899	8.911109919	8.538380869	9.316888828	8.497259596
7.888681737	7.83046296	7.686775222	7.186327495	8.348543172	7.400864778
10.22164118	10.75453103	10.74558579	9.153730283	10.96460614	10.49348589
10.96000427	11.24323027	11.14707085	11.32376318	11.82112126	11.74205097
13.45466248	13.17281418	13.65450155	13.97489728	13.34730616	14.05136004
9.886762101	9.996420616	9.856174394	10.36134852	10.1838089	10.21769501
12.23769992	12.63102018	12.5521346	12.76628311	12.94821125	11.93975968
8.62991701	7.445746576	7.28582396	6.988399394	7.403028737	7.639662323
11.55106122	11.73710477	11.47682743	11.50261564	11.22947614	11.22947614
11.71895032	11.49239347	11.67593314	11.70138789	11.33100665	12.06964699
6.799149535	6.53485901	6.738245586	7.272016424	6.866852303	6.853717071
10.52135963	8.399075535	8.584413131	8.053619525	8.593558293	8.533908051
8.205457293	6.396985434	6.866852303	7.537489808	6.656456051	7.000309313
8.516311424	9.104371838	8.78767504	8.383429707	9.165150051	7.935081758
7.36166898	8.242431778	8.194999071	6.773008051	8.475210818	8.116928465
6.625311455	5.792205879	6.265371141	6.828875111	6.045730283	6.067236707
8.570262	8.251492909	7.946933485	8.65497299	8.307893798	8.399975414
8.840010495	9.392082222	9.584100455	9.424985495	9.58708204	9.572453101
7.847720859	7.771540485	7.545628697	8.063461202	7.370661465	7.478449101
8.560348606	9.409395929	9.34289298	8.464081152	9.161459798	8.89604703
8.171788646	7.678591667	7.606699253	7.719466677	7.323124384	7.341641707
7.85299496	8.390560192	8.013081051	6.970224646	8.116928465	7.891912283
6.634850818	6.929106091	7.104662848	7.238770545	6.337018061	6.531724505
6.837226	6.573540778	6.397416404	6.719552727	6.555968283	7.072297535
6.327726343	6.436179667	6.388134263	6.535531162	6.090741131	6.410292616
9.451212687	9.042346232	9.333361616	8.698215303	9.070117818	8.731005677
10.57996368	10.88031852	10.16165443	10.75453103	10.67305197	10.34100163
6.821773354	5.828925566	6.155174505	6.287602071	6.100757848	6.330092323
9.492465394	8.568768141	8.443547162	8.406205495	7.44049799	7.755132808
7.641683626	6.297649162	7.45028304	7.264201869	6.841827051	7.242951465
8.901935111	8.326162172	8.388388636	7.871272152	7.971654859	7.976470212
8.206288727	7.929187444	8.572701232	7.956286283	8.477505576	8.379056919
9.374690616	7.945111576	8.255317818	8.274284293	8.169382444	8.32939098
10.1309568	10.38531322	10.27714677	10.56548536	10.34626898	10.72246175
8.736221495	9.319510202	9.511857455	9.071276071	9.436898869	8.961854929

8.999492384	8.572163455	8.619347838	9.681893121	8.656360697	8.875206121
12.8293171	8.908475283	9.656571869	9.670530667	9.62024901	10.52799495
6.941957697	6.363322586	6.572638727	7.196057152	6.627087081	6.858687869
6.931701566	7.490707535	7.807594768	7.55178802	7.707585606	7.857447394
10.71992703	9.402230293	10.15283227	10.54692936	9.862946677	10.19332507
9.150235333	8.661956242	8.698215303	8.728878424	8.719668061	9.143918798
6.317463354	7.44169298	7.251769333	7.571427586	7.619228051	7.289331586
8.653441707	8.41552702	8.490531303	8.673843717	8.034450929	8.146343545
9.941224515	9.793126455	10.07479617	9.803356646	10.43998577	10.21375666
6.532632323	6.607353172	6.96917198	7.391081455	6.771146798	6.907322414
8.079553929	8.424934535	8.35018502	7.310482798	8.164979273	8.03490996
11.05796986	11.94979793	11.48840455	10.74558579	11.5737214	11.01951417
11.93486375	10.60383217	10.79145221	11.48061491	11.109559	10.95374716
8.618800343	8.726978374	9.286739242	7.192707293	8.531655515	8.318668263
8.783584889	8.68840998	9.026117657	8.005065495	8.635209293	8.433468657
9.286110869	8.831201768	9.105035414	9.150811232	8.901331343	9.468134182
15.05177986	13.85099976	13.64083669	14.02572128	14.03424604	14.20688311
6.829338141	7.350681061	7.011036525	6.922161646	6.56193898	6.611373919
6.330710616	6.11485799	6.409033687	6.510509061	5.79820198	5.795624788
6.779180051	6.85895202	7.064817384	7.055635202	6.961503899	7.067324051
7.65951298	7.046480485	7.301802939	7.528640475	6.613716152	6.855182313
9.522660222	10.11452638	10.14131493	8.264240727	10.39296715	9.660861778
8.065153556	8.208692758	8.178694051	7.966742939	8.383429707	8.032957859
7.213710414	7.470329323	7.614497747	7.685352364	7.866966485	7.722652869
7.327234414	7.733228788	8.061425949	7.152958919	7.31991999	7.666604434
7.586712596	7.841440677	7.493863788	6.983407384	7.36298202	7.627373273
6.694950828	6.575452697	6.273654202	7.323648515	6.976233182	6.741204455
9.864614818	9.417037424	9.57872197	10.72633412	10.33375706	10.10736711
7.93888704	7.568036061	8.226840717	7.979863071	7.727011939	7.580594545
9.321347081	8.881793333	8.866888202	9.038261515	8.226165	8.226500707
8.557697889	8.47014499	7.18511501	8.583996586	7.489176	8.049354232
7.510343899	8.030181424	7.600248455	8.300888657	7.749943687	7.606043535
8.379056919	8.561321323	8.657806242	7.53302901	8.361756343	8.29291099
6.720840657	6.018409434	5.90208997	6.275054576	6.030256101	6.038814121
7.624419899	7.676257364	7.57049196	6.800553495	7.490096869	7.429246232
7.722297051	6.840910293	7.378842657	6.988144556	6.718256293	6.788076343
8.673363869	8.940763364	8.788663253	9.62845904	9.07581704	9.240719434
7.81270001	7.463713737	7.377557566	7.762794545	7.609879768	7.532708636
8.881793333	8.39308204	8.523871909	7.942409707	7.964914919	8.362142131
11.29076429	10.30470526	10.59180142	9.710054737	10.38631174	10.02923217
6.732989909	6.351287939	6.302690465	6.710946737	6.193905333	6.297649162
7.461239687	7.651926253	7.643628758	8.530767747	8.014589606	7.799196616
7.925310818	7.092080626	7.098240939	7.481645051	6.632973788	6.660321343
6.854415313	7.260895414	7.617533182	6.889990505	7.172411071	7.037248667
9.640429374	9.250770152	9.312041414	9.772250768	9.398445515	9.470840162
8.775519707	8.554278051	9.198999222	8.835019636	8.761644081	8.970028374
7.529315566	6.805229596	7.636934919	7.230900293	7.059356717	6.87010599
6.632573485	6.661555384	6.811868152	6.541858778	6.578936343	6.427081586
8.025425616	8.717867636	8.862785404	8.374810828	8.85785699	9.051375475
9.141588162	8.424058939	8.68840998	8.500729384	8.157153081	8.387083515
6.989960667	6.871158202	7.030688768	7.332344778	7.16725497	7.258107788
8.801904485	9.276789111	9.203760495	8.007258162	9.226128374	8.906910121
13.17281418	10.84302834	10.5152666	10.95683616	10.46232362	11.59654933
9.843921515	10.14999886	10.01168912	9.424273283	10.24828231	9.670530667
9.839882283	9.479084687	8.715328101	10.69222123	9.493894545	9.558255162
13.01016522	10.37026232	10.39414857	8.900236364	9.905108616	10.88031852
7.664957788	6.783595586	6.76551097	7.172666465	6.521458212	6.77992704
10.2026692	9.251353071	9.509876273	9.323195364	8.48742196	9.186096828

9.143279535	8.936938576	9.368875727	9.599465606	8.918756414	9.766921414
9.095762	8.29291099	8.873520545	8.33024502	8.070920919	8.467263475
7.55847901	8.039451586	7.580594545	7.25402203	6.341887717	6.740481444
7.59859104	7.632861657	7.631600293	8.329784131	7.576687677	7.804724687
6.277331707	6.788535525	6.855446939	6.969691626	6.525823616	6.406383182
12.36473556	13.44849295	13.71423947	13.93694444	13.67554111	13.45466248
8.368780919	8.327767232	8.781680606	8.587234515	8.296772869	8.175968162
9.675185677	7.473129747	8.681067818	7.62509899	7.113720848	7.601304687
10.08231743	9.005433152	9.242021586	9.265583747	9.616057222	10.04340808
7.447389475	7.336145273	7.540434798	8.432612263	7.72538904	7.573484121
7.041369354	7.510027525	7.650858424	6.801007778	7.476255091	7.246699182
8.643157737	8.890373556	8.77298297	7.95565902	8.885078273	8.672348475
8.427236737	8.643636162	8.561321323	7.491688212	8.458294828	7.984288364
6.897195879	7.13936198	7.198026636	7.55945201	7.513736343	7.175214949
10.33989796	11.10035391	10.87518762	9.653591273	11.03580591	10.59685822
8.355847778	8.393536232	8.494576061	8.125376242	9.178084667	8.720163182
9.655770101	8.443956768	8.358418384	7.978709162	7.832540091	7.862492343
8.265130232	8.57790302	8.57597697	8.324453253	8.634788929	8.271029889
10.85100254	10.46442497	10.56908161	9.167758485	10.50166798	10.38842688
7.252038162	7.925642	7.835788293	7.247912131	7.063754707	7.086390323
8.626833051	9.10215096	8.909404788	7.967113929	9.199527535	8.899757485
9.135096212	9.102676354	9.224452333	9.085558838	9.529469889	9.143279535
9.459003	9.855298394	9.800083455	9.595333525	9.909202788	9.598893818
8.404396899	8.789683394	8.898709273	9.546645747	9.483552818	9.094691273
9.69725304	8.628208788	8.77962597	9.382882596	9.425521616	9.163332636
9.038849273	9.096395636	9.107945202	9.87177298	8.517695303	8.10140802
8.79576798	8.711304737	7.922697606	8.534432515	8.628208788	8.99408802
7.851640596	8.617420111	8.654464586	7.527697455	7.920144808	7.810269677
11.49839615	11.46936418	12.3613163	12.28985985	12.12436236	12.05813886
9.015966535	9.887613414	10.00262223	9.713858919	9.731806091	9.441234879
10.10542249	9.899969596	9.896649636	9.285000091	9.282486081	9.512482222
9.981718727	9.815893677	9.613873859	10.63870636	9.769223525	10.23247836
8.359650919	9.180906778	8.821885465	8.742635939	9.151431899	8.626833051
7.548584576	7.547922717	7.776955808	8.204380404	8.353342091	7.543124535
6.785769293	7.178633636	7.220240556	7.588938485	7.531511131	7.223533071
10.13375911	10.41731261	10.50057506	9.755162434	10.8947391	10.40074116
9.91449603	10.32849396	10.23752804	10.00695165	10.38329808	10.51886057
6.756409455	6.798354081	6.710946737	6.553884717	6.280078727	6.265911495
10.0719806	10.1885747	10.1309568	9.048100778	9.46096901	9.727935707
9.069558111	8.744562374	8.736738475	8.071334404	8.460053242	8.615330515
10.00361533	9.818860343	9.869466131	9.892629697	9.735412273	10.22865659
11.81887725	12.6076243	12.85589012	11.22600179	12.24649354	12.41033676
7.473129747	6.544566455	6.750902172	7.208321485	6.490634808	6.568654859
6.682537212	7.113720848	6.57287004	7.148192263	6.261209	6.532632323
9.304577515	8.748883111	9.019287687	7.232456929	7.415948364	8.303793737
8.50673198	7.677935313	7.879657162	7.668638899	6.97260104	7.194677455
7.056863278	7.459405444	6.907588505	7.279410566	7.508388697	7.139090576
5.880154242	5.884050495	6.561305909	5.96355196	6.06334998	5.857682343
10.79284767	10.761049	10.69682519	11.03737422	11.00053539	10.71613341
9.026636737	8.316081222	8.910531141	9.109120374	8.282900293	8.913588253
12.36759935	12.32024961	12.92542832	12.08680505	12.15752365	12.20640951
15.01206542	14.67552064	15.22815255	15.38120792	14.39736901	14.9727616
8.991410131	9.337187111	9.156840283	8.32400903	9.384699788	9.237664879
11.06433979	11.47322198	11.21493335	10.18198959	11.36065784	11.109559
13.35359128	13.48401144	13.11515098	13.58996905	13.98831971	13.23864174
9.036569232	8.816214848	7.987573434	7.423727141	9.334658071	8.814624313
9.602112616	8.621272737	8.503061444	7.834787162	8.211847566	8.486968232
8.701109788	8.07053003	8.177146606	7.911309091	7.505759091	7.544716788

9.04056096	9.236429667	9.483552818	9.274850788	9.949411172	9.708693071
9.284395657	8.962901121	9.736957071	9.889177586	9.713188212	10.21157382
10.53623255	11.01045685	10.91176188	10.09597734	10.87637444	10.26541026
7.76948301	7.167545535	7.609232899	6.99955204	7.200432616	7.079103131
10.38531322	11.1502402	10.87092264	9.286110869	10.41022215	10.86173253
8.360024717	7.91307104	8.055916879	8.846850111	8.567801737	8.406205495
8.034046273	7.934739636	7.898205273	8.509936919	7.972411364	8.700606657
7.797125465	7.879296202	7.950819283	8.471488636	8.144908747	7.11489098
6.339262212	6.503188616	6.61996199	6.506079909	6.098532758	6.221214869
8.593558293	7.116869414	7.617890202	8.484723788	7.623386535	7.97753898
9.216574283	9.732604394	9.894213939	9.692043475	9.524763869	9.576136707
7.22559698	7.190930768	7.281402384	6.526092091	7.01811702	7.386173242
12.89886333	12.29548604	13.2504616	12.49170848	13.2504616	11.90674449
11.15666056	10.20358283	10.49140257	11.06877356	9.059459505	9.658049545
13.10985871	12.26162026	12.77841715	12.41931404	11.75462574	12.5521346
8.431326525	7.876807081	8.019434586	7.816627253	8.088528121	8.27018399
10.45996909	11.0473733	10.81664077	10.04963797	10.59809845	10.60507136
10.21157382	10.2861211	10.15783547	9.09178804	10.03026493	9.946747141
8.384333949	8.955880859	8.372086737	8.985325394	8.478263182	8.862785404
11.03452834	9.261851101	9.335891293	8.157153081	8.216297535	9.710962273
15.19830883	14.21449649	14.34357305	14.21449649	14.23242051	14.17411554
10.94942311	10.22668357	10.12408141	9.575401111	9.837575172	9.969958172
6.955646677	6.849574919	6.913511828	6.873999848	6.202273253	6.316499364
7.454874889	8.021628354	8.229339455	7.872718384	8.142577798	7.41439101
8.897177899	7.738299929	8.090488788	8.05474504	6.899721758	8.040196323
7.942409707	7.758641747	7.801388869	8.047553323	7.140009717	7.132449434
6.691055667	6.492436768	6.539153576	6.66759498	6.779669273	7.23423804
7.530574707	7.052221778	7.138289616	7.049507061	6.479217141	6.789988131
8.077645899	7.583692414	7.785596848	8.242431778	7.055367566	7.169717707
10.3025572	8.753420071	10.0079343	8.164575929	9.208914778	11.72355631
6.797589061	7.202436384	6.751435283	7.016713091	6.361157788	6.542314697
6.696224707	7.104930788	6.971045939	6.431827616	6.669677444	6.921891778
7.15072903	6.806225091	6.904054566	6.806762071	6.393637899	6.460794313
6.733232384	6.952808172	7.233950838	7.995673758	7.357173859	7.354199768
10.41626133	11.68948846	11.31053596	10.30358984	12.11216258	10.88302044
9.830354566	10.11555853	10.34717129	8.803935202	9.993897848	9.618955576
8.131433848	8.33024502	8.468064667	8.425847798	8.334587636	7.990313949
8.152443121	9.027136657	8.779146061	8.246087697	8.175288444	8.316969364
8.678658293	8.164979273	8.165380303	8.124973859	7.245604273	7.374297343
6.474717424	7.041650374	6.242331465	7.468171747	6.10298801	6.128300636
9.596773313	10.56548536	10.43655428	11.21338774	10.69574272	10.62895885
9.731150899	10.57649171	10.29851595	10.27338352	10.37467131	10.15483313
8.997224192	9.431415586	9.440510152	9.881912677	9.415217505	9.550769788
10.7442872	11.05191968	10.98186414	11.52824	11.30506756	11.16579624
11.48840455	12.56315114	12.46797904	12.29886409	12.26475658	12.91656586
8.962353222	8.880853535	8.76328402	7.891531071	8.893419636	8.609106909
7.694540808	8.202220545	8.099466788	8.923097444	8.612168455	8.409959899
8.487847515	8.93638097	8.723452192	8.191573747	9.061091758	8.610337293
9.379720636	9.852239606	9.671912455	10.46766096	9.943097475	9.755162434
12.94821125	13.68884286	13.57574016	13.57574016	13.40990013	14.11997744
10.23066525	9.763138212	9.84311402	9.638947465	9.508442768	9.932690333
9.118059475	8.664254273	8.500729384	9.244399414	8.837022465	8.775001677
7.455822202	7.97284099	7.972012263	8.765765323	8.418553323	8.222643394
9.511202364	7.650231051	7.891531071	7.25490398	7.440186081	7.816254515
9.314493212	7.285287818	7.632536929	10.0143126	7.607714535	7.283275424
7.445746576	7.053635222	7.090758091	6.825870455	6.725499141	6.900245465
11.97944932	12.34855768	12.80602179	12.0750029	12.3613163	12.19727311
11.35858946	12.00354228	11.56725755	10.5029511	11.58005536	11.58195332

7.747112485	7.809338131	7.869709071	8.000883182	8.731576192	8.282438737
8.954176293	7.649291899	8.386601707	8.824381182	8.043387838	8.107705111
6.922412606	6.598705667	6.938883283	6.071423232	6.266464606	6.35186004
12.29548604	11.78725933	11.87175588	11.81221198	11.03296099	11.88649013
9.10558002	8.815152444	9.552159131	9.945994374	9.591816061	9.516398909
10.38738452	10.28406896	10.31597323	10.09254304	10.70299608	10.31480319
8.312729141	8.076118121	7.934739636	7.952029848	8.204737434	8.770297859
11.21493335	10.16165443	9.633493677	9.472607081	10.37686258	10.07670007
8.129067798	7.582966818	7.70365599	8.399975414	8.403029636	8.111336758
8.804952828	9.020337111	9.248396293	8.344098929	8.673843717	8.724556485
8.719245818	9.155079848	9.263851586	9.58708204	9.249034202	9.033394545
7.408681949	7.851263121	8.209801687	7.608347495	8.231756293	8.049757475
9.570470929	7.287262414	7.438305869	7.587331949	7.279952616	7.403336687
8.114611232	8.65914896	8.569209263	7.558798354	8.623178879	8.050105303
11.95514832	12.76628311	12.72219022	12.63500423	12.63102018	12.8293171
9.900778232	10.8935809	10.94640834	10.47242114	10.45041513	10.59541023
15.08134613	12.28105924	12.88265743	12.68484185	12.51766186	13.22156044
8.340681505	8.444861909	8.563085788	7.403028737	8.376704939	8.066736566
8.651919778	9.289535939	9.069043081	9.068490586	9.177504333	9.204855576
10.47702836	9.941224515	10.34100163	10.48539779	10.97896732	10.85361094
10.36134852	10.84844593	10.71613341	8.879851283	10.49466708	10.14044783
11.80342824	12.3843369	12.45390404	12.7939548	13.09977851	12.43279956
9.888393747	10.15379546	9.801802283	9.683334444	9.721958051	10.17336172
10.1885747	10.42927797	10.27432009	10.1421691	10.22164118	10.21583282
6.645215414	6.503631525	6.228422404	6.413017273	6.476196333	6.224237061
9.268661657	9.879566364	9.395177889	8.862785404	9.439917707	9.535633242
8.644542606	8.217270354	8.152794889	8.174069929	7.818322879	8.736738475
9.576740677	9.355403384	9.600888859	9.788274313	8.907429495	9.080123545
6.776493808	8.287438535	8.550897414	7.797507939	7.726392606	8.350987111
10.3090972	10.10093339	9.610538374	10.15783547	9.687758929	10.25038968
6.646430939	6.924744576	6.329109535	5.985642434	6.235363323	6.923289823
11.49029795	7.589888475	8.280707687	7.903688606	7.613528424	8.50473503
8.861728919	9.151431899	9.042346232	8.014979636	8.949261586	8.690918616
6.499296646	7.21150504	6.741002212	6.936369818	6.098804071	6.210392253
8.81572204	9.071276071	9.126663535	8.740195303	9.003925646	9.168324747
8.418118394	8.991970091	9.077686808	8.118473111	8.86638896	8.788135667
11.2278827	11.93486375	11.96059797	11.97196754	11.73070023	10.99469779
10.08700287	7.689906808	7.850890354	7.771866444	7.499556727	8.160644788
6.987320737	7.007603253	6.777485293	6.205556535	7.278115626	6.686567667
7.294861071	6.641619879	6.595947525	7.027167101	7.079103131	7.095534354
10.38430498	10.4429644	11.06877356	10.76857843	10.78408516	10.51650912
9.220765354	9.36570098	9.404880263	9.530933293	9.576136707	9.694925798
8.561789232	8.669446475	8.734806657	9.089024899	8.91530399	8.697732283
8.238629646	8.565346707	8.338355444	7.516138929	8.340681505	8.242002535
8.863375313	8.702074263	9.081281455	9.189045596	9.256767414	8.889380424
8.039451586	6.510509061	7.151822525	6.825565162	6.681596343	7.177746566
6.831129859	7.09353204	6.571183485	6.106642303	7.170797768	7.06104997
7.634916323	7.161889071	7.757234778	8.314398727	7.759260374	8.573727141
8.305787747	8.591239465	8.710391727	8.838951081	9.136296333	8.861728919
9.415217505	8.412145273	8.822318	8.562664263	8.406205495	8.735755485
11.12454893	12.26775966	12.36759935	11.29399858	12.21991364	11.93178106
6.894135505	6.643553717	6.844744919	7.061330929	6.413218091	7.129030091
8.364710758	8.775001677	9.060618192	8.901935111	9.029402949	8.943861384
7.349181162	7.091328697	7.515531818	8.519692939	7.881467646	7.616235111
10.75453103	11.58195332	11.94745099	11.39983314	11.9577766	11.5386618
8.592161788	8.375668162	8.029383646	9.091299889	8.179951646	8.337550606
9.996420616	10.08978265	9.954681949	9.987077505	10.01340346	9.763138212
7.179751081	7.959830364	8.462637525	7.748548515	7.838908253	7.347141657

8.984797889	9.569063343	9.35371997	8.699656879	9.245445293	9.221852535
8.129874121	8.009790253	8.339926909	8.868452172	8.831201768	8.640789455
8.082778869	8.884521697	8.58807297	8.301657889	8.708467242	8.042974687
7.011288768	7.640998838	7.511268929	7.772503354	8.489580414	7.663284545
8.255317818	8.593101434	8.459596535	7.864861071	8.232591606	8.489178071
8.988577162	8.731005677	8.511305758	7.821971636	8.468064667	8.57790302
7.023397576	7.173324505	7.18283101	6.992392121	6.402020111	6.621578222
7.379145515	8.429582192	8.085049192	8.053207788	8.632349838	7.788172758
8.726978374	9.215946081	9.227886646	7.634916323	8.732044667	8.908935182
7.611975737	7.147201253	8.658802051	7.498343283	8.024633293	7.92597801
9.056533626	9.825199323	9.827596687	10.74172297	9.958247111	9.926784556
10.02821235	9.599465606	9.601568657	9.838413929	9.396536798	9.733950273
10.82664672	11.20345844	11.25523353	11.81887725	12.41931404	11.71042621
7.233039222	7.384605828	7.366646525	7.551170919	7.550845909	7.629673333
8.034450929	7.442908828	7.501110505	7.052522778	7.968277051	7.97830402
7.421318101	7.846324646	8.007640657	8.629474212	7.615524929	8.150892606
7.642932737	7.135797152	7.343750657	7.228169333	6.570263495	7.035420475
12.22341713	10.85100254	10.86566339	11.24993851	10.92337564	11.12001247
9.462343535	7.418256545	8.158602879	7.658148485	7.108492263	7.964914919
11.49420288	11.52824	11.22456459	11.25523353	10.79145221	11.87687449
7.949790515	7.966402293	8.600356081	8.421742051	7.870978364	8.74840203
8.252794354	7.916391202	7.044743657	8.944409727	6.782198505	7.383097848
12.56315114	14.02572128	13.96779242	13.94443	13.72760196	13.64789782
10.20060626	10.64623997	10.30058205	9.915234909	10.97322727	10.48185706
13.47327752	13.70717091	13.70143341	13.80704919	13.98081649	13.5360606
8.64752602	9.379720636	9.260607	8.65914896	9.477759242	8.702074263
11.18096106	9.837575172	9.986114121	9.967178364	9.932690333	10.31795252
9.159785394	9.37540303	9.576740677	9.445852485	9.878686616	9.608350808
10.33375706	10.59809845	10.54950911	11.4090051	10.84176532	10.98186414
9.373397364	10.36781996	10.10093339	8.554528737	9.975431768	9.983540212
7.391952354	7.31991999	7.308713444	7.277482444	7.464704768	7.509397909
7.459710222	7.889031475	7.916712838	8.810986707	8.269395232	8.257984293
10.00695165	10.26541026	10.20164867	11.03008027	11.25349741	10.86691694
8.020484273	8.434806414	8.401317182	9.251906182	8.858269384	8.780092131
8.361756343	8.124599212	7.152958919	7.559118222	6.808010384	6.95944596
11.13879804	10.2829717	10.6313464	10.46108665	10.16454544	10.58469265
9.397189192	10.01622438	9.811091061	9.043522909	9.478383576	9.684886576
9.29794999	10.27035429	9.957374697	10.63391466	10.58579258	10.23752804
9.240082808	8.118879606	8.341977727	8.268052495	7.734896879	8.118136869
8.35372597	8.497259596	8.496365737	9.393335687	9.004398101	8.831201768
9.989520798	10.46664928	10.93171462	9.724814657	9.966143818	10.15186663
6.696930808	6.305750758	6.105514909	6.123671758	7.044228545	6.778966495
6.963370131	7.245884828	7.56165098	7.994919717	7.267888182	7.430156263
11.14707085	9.904237354	10.11246778	10.16355358	8.728878424	9.488971182
7.187741232	8.005065495	8.336651323	6.962574283	7.814093232	7.941721242
10.75062885	11.07647857	10.55842197	11.30506756	10.81540317	10.73384189
8.69508802	8.962353222	8.771856424	8.338355444	8.484723788	9.129654758
9.857104919	9.350012182	8.049019253	9.363823202	7.657544313	8.483326677
7.91707401	7.701352778	7.655304091	6.850100909	8.485174677	8.125818909
8.939630657	7.957660747	8.135850465	8.355387535	7.579959303	7.901142576
7.347734202	8.213427111	8.110083838	8.053971495	7.855731	7.982442283
7.686444293	8.166172404	7.975072535	7.140009717	8.051256737	7.729067364
11.86401838	12.76207554	12.63102018	12.71520416	12.70787607	12.69676846
11.56725755	12.00644476	11.13085117	11.69188536	12.25844332	11.90384839
9.02886299	9.657223606	9.534883657	8.908475283	9.497817424	9.328711051
8.924201657	8.228066545	8.136641818	7.854325061	7.783465061	7.739667424
7.359577545	6.390360535	6.339459364	6.122087354	6.063889717	6.303681202
7.962380919	7.532708636	8.044980354	7.484516838	7.439837192	7.432376434

5.82585701	5.777942717	6.757183747	5.773857909	5.825080838	5.766399495
9.497084828	9.174120707	9.277455131	10.62895885	9.204855576	9.565032515
9.67665103	9.555296242	9.285586323	9.662314131	9.934615404	9.815129384
9.200683333	8.83948901	8.943348051	9.35371997	9.167758485	8.835493283
11.0859227	11.70557621	11.52238659	10.06997663	11.87445957	11.19293398
6.02015296	6.568654859	6.463086606	6.151838859	6.352052949	6.358115374
11.78023213	12.38794592	12.46466592	12.68097339	12.06098883	11.92156179
6.570036263	5.894855384	6.070436798	6.178002222	5.82923402	7.409335747
7.591105343	7.166369152	7.197080263	7.911995121	7.059062808	8.893419636
6.847553121	6.423050626	6.38666303	6.742510061	6.088380414	6.190765768
14.56207529	15.06622261	14.56207529	15.31193978	14.90726461	14.88238784
9.214111545	9.543825273	9.724814657	7.895961929	9.487680697	9.337862091
13.3718573	13.92894576	13.46005067	13.40990013	14.223913	13.78027417
8.910531141	9.09013302	8.944409727	7.706168626	9.112697707	8.59655801
14.9847254	16.14978837	16.22759729	16.3077943	16.7804214	16.09492117
6.828400424	7.334066545	6.575874879	6.389892586	7.210358909	7.427701768
8.014979636	8.417196576	8.341133162	7.183662697	8.105629576	8.262181081
11.41778198	11.60266156	11.41981305	12.05813886	11.21016953	11.9982543
15.22815255	12.51424883	13.09463792	11.10761793	12.60396528	12.80166365
7.107702061	6.417239485	6.778470374	7.384605828	6.512799333	6.845837626
6.32750801	5.759653798	5.781781232	6.179354162	6.150621626	5.977758596
7.145493505	7.776955808	7.335252788	6.968641576	7.704008545	7.695265798
11.04593523	11.62290433	11.77802923	10.40178901	11.43380494	11.01209098
8.917682242	8.989652131	8.795325869	9.505258828	8.847386828	9.071276071
9.514496606	10.43440935	10.4283734	9.000069889	10.3986043	10.3810842
8.534897646	7.884680111	8.077645899	8.11047698	7.304428303	7.794719919
12.00644476	11.43005301	11.65455063	11.50632759	11.28731295	12.052647
12.73731843	13.05428976	12.797444	13.16824615	12.90359001	13.03025597
8.104500051	8.551311374	8.409136758	7.456122566	8.216823323	8.227704253
8.696739152	8.177146606	8.19458801	8.979921919	8.741130788	8.93638097
10.65634987	10.67815746	10.92880859	11.62059475	10.73517618	11.26262606
7.613528424	8.209418434	7.967920667	7.224144889	7.912365798	8.064260859
8.559011545	9.135618929	8.859715222	8.382194192	9.761765848	8.821885465
7.495760485	7.695265798	7.729067364	9.584803364	7.366317111	7.912365798
6.777950687	6.565610293	6.649897606	7.965660091	6.856475414	6.935569505
7.471219899	8.575566677	8.382194192	7.575420636	8.585303495	8.77962597
10.03026493	9.766131535	9.589694828	10.36589334	9.783680727	10.23160947
13.46712151	13.12562627	13.28559105	13.7211242	12.82153585	12.77454263
11.31852116	11.31053596	11.79870316	12.42233701	11.69188536	12.10951308
9.121511687	9.280305798	9.288999586	9.727935707	9.644739414	9.558925838
8.813032152	9.567720788	9.195011616	9.087255717	9.240082808	8.832790172
8.955880859	8.410410394	8.59026096	8.314850485	8.116212253	8.563085788
6.084661768	7.433328091	7.546918172	8.084674061	7.477877475	7.412191343
9.296718121	9.965319232	9.960013465	9.958247111	10.27035429	9.854472677
12.52461444	12.62807191	12.57107442	12.70002263	13.20018245	12.44264271
7.731542758	7.450590374	7.478449101	8.456593071	7.910120242	7.834378071
10.06904603	9.826793596	9.915234909	10.05970796	10.08799683	9.782133848
8.80133401	9.414554808	9.685649929	9.882779162	10.09339803	9.374121768
8.717867636	8.803935202	8.846355515	9.608350808	9.226702434	8.564847253
14.36999747	14.62217632	14.48025691	14.7528858	15.02501841	14.4335752
6.273097626	6.616118788	6.964119424	6.995294596	6.466725596	6.341887717
7.783465061	8.123132566	7.954939636	7.272331859	8.333749495	7.581982414
6.615899091	7.09469803	6.637000293	7.777661384	9.332175414	5.950485919
9.601568657	8.965143333	9.324388263	8.376704939	8.871686232	9.245932475
6.790568566	7.316406697	7.101832475	6.277331707	7.090185162	6.909401626
7.378842657	7.658839354	7.719466677	8.331046747	8.189895404	7.832217929
7.979449616	7.322217273	7.429567525	7.404355202	6.941467394	7.219933525
7.285574899	7.141717727	7.367244707	7.59859104	6.822905505	7.072618697

6.860390606	6.375508101	6.636479121	7.262654505	6.953109798	6.927259889
8.982611727	9.509876273	9.705000525	8.271405859	9.235747242	9.232683343
7.832891081	7.996393263	8.052077899	6.74001704	7.957321232	7.65951298
6.996297444	6.925244263	7.308421545	7.90609097	7.600615697	7.59785203
10.11246778	9.519086535	9.751885293	9.250770152	9.29794999	9.793126455
7.383097848	7.350971899	7.381950828	7.049242879	7.924885333	6.807771525
10.78987961	11.18941159	11.22600179	9.545881535	10.83363242	10.93171462
9.376046232	8.320357909	8.690918616	8.755916071	8.384333949	8.703733141
8.204737434	8.417604414	8.609511	8.43006898	9.055478465	8.866888202
8.518658667	8.341977727	8.567346869	8.181196919	8.114219303	7.970233808
8.406604404	6.543651707	6.76700904	7.199149121	6.69920203	7.028809465
9.21905796	8.76478299	8.85308204	9.175472828	8.711304737	8.781156657
8.904239657	7.976858394	8.35752198	9.511202364	8.037287293	8.789132293
10.20532801	10.63870636	10.12610261	10.8935809	10.30695028	10.36903954
6.709797242	7.155245384	7.951199808	8.942313313	7.841729333	7.119081899
10.86820493	11.0940021	11.09250749	11.60069924	11.12935449	11.08435313
8.242002535	8.22479296	7.860764394	8.892401576	8.043818737	8.803482152
8.809442889	9.513795939	9.369545939	9.519820636	7.614146707	8.144161414
6.315805677	6.22946	6.096295556	6.408579424	6.167372141	6.240866273
8.387939343	8.71485401	8.854095293	7.475616394	8.266842545	8.623178879
6.369478939	6.200027596	6.537260646	6.405058101	6.298407384	6.179354162
10.5152666	9.905938879	9.799317404	10.22668357	10.47702836	10.53028115
9.339764051	9.802496737	10.1684149	10.29752835	9.994772242	9.954681949
7.195224778	6.492652182	6.58104199	6.703235576	6.675987374	6.527432758
8.102862354	8.275152535	8.457489293	8.927019859	8.710842768	8.660166475
8.022720303	9.384078242	8.946107263	8.35018502	9.509226798	9.389466253
6.536206333	6.762314343	7.167545535	6.272928495	6.522975131	6.544097556
12.31719428	12.19996238	12.65819872	13.78737352	12.44264271	12.92937388
13.92172837	14.27397716	14.13521971	14.67552064	14.34357305	14.5108222
12.46466592	12.28693608	12.48835313	12.36473556	12.05813886	12.66985986
14.90726461	15.54332231	15.65613201	16.09492117	15.58150095	15.81050193
14.04335209	14.29173122	14.28325373	14.3093669	14.11151028	14.26676228
13.75520609	14.09521817	14.05855866	14.94534759	14.20688311	14.21449649
10.80389925	11.34202326	11.25898056	11.38775416	11.34381562	11.32559397
12.3774088	12.65472241	12.62807191	13.02518615	12.51424883	12.72219022
12.44950322	13.11515098	13.39659876	13.20536123	12.75396286	13.06488572
13.15645152	13.46712151	13.43523914	12.59668127	13.22156044	13.42153085
15.63778652	16.25437967	16.14978837	16.42659037	16.04217659	16.04217659
10.77637516	10.80900786	10.58236152	11.35278885	10.95093974	11.10460602
12.37416009	12.63500423	12.61761735	13.13007894	12.66627862	12.92542832
11.36793803	11.26815786	11.59654933	12.02240324	12.22341713	12.31719428
13.23864174	13.00101781	13.03957854	12.85048632	12.69676846	13.16824615
14.53168901	15.449005	15.34617167	16.06827159	15.81050193	15.67809237
14.24162707	14.9847254	14.61202705	14.66395575	14.56207529	14.68633385
11.71443529	12.13867659	12.17216641	11.83813795	11.89361272	12.04432
9.334658071	9.132651727	9.262561	9.028300162	9.001802606	9.08614898
13.80704919	13.93694444	13.9071606	14.05136004	13.69473258	13.98081649
13.47832004	13.50997822	13.54221797	13.48401144	13.17778072	13.60979651
12.6076243	12.72610186	12.80166365	12.66627862	12.18142186	12.84197228
14.60195877	14.94534759	14.82007331	15.10875812	14.93426708	15.15443825
13.48401144	14.4427031	14.31917195	14.46162431	14.25042984	14.32804886
13.85099976	13.84455043	13.98081649	13.22156044	13.47832004	13.98831971
11.51867768	10.69222123	10.77252405	10.94942311	9.288419414	9.515069697
13.39037726	13.63435472	13.37822002	14.19920898	13.89331098	13.50997822
15.21350605	15.97330865	16.06827159	16.02065153	16.06827159	15.97330865
11.66708917	11.76588718	11.82616001	12.32964397	11.97447062	11.7894918
13.32669092	13.55640443	13.5964752	14.23242051	13.58996905	13.80704919
12.39058663	12.80166365	12.43279956	13.47327752	13.10484893	12.51066587

10.53028115	10.78408516	10.55602586	11.82616001	11.53050203	10.69793218
13.50997822	13.78027417	13.84455043	14.31917195	13.83772389	13.90036211
12.72972704	13.18858184	13.00581854	12.92081703	13.02006671	12.90359001
13.18858184	13.42888134	13.15082863	13.32021166	13.14093405	12.85048632
16.3077943	17.49517975	17.29704085	17.29704085	17.23317693	17.36803328
15.72204116	16.49551516	16.52993947	16.8710039	16.36806566	16.61084374
12.61090773	13.33661988	13.27940818	13.50997822	13.50411017	13.31405593
14.01682491	14.42673578	14.52085241	14.88238784	14.4427031	14.6970922
13.52334642	14.25895571	14.15092087	14.5513462	14.08841453	14.29173122
15.06622261	15.59877501	15.54332231	15.59877501	15.42966281	15.58150095
15.03840752	14.99939481	15.36066179	15.88528111	15.41210544	15.36066179
13.11515098	13.35919327	13.26910509	13.52334642	13.1944969	13.2504616
15.48449671	15.86132141	16.04217659	16.14978837	15.65613201	16.12384164
13.48990673	14.4335752	14.3093669	15.09418019	14.42673578	14.36999747
13.64789782	14.49002723	14.4427031	14.78379092	14.61202705	14.57091557
11.36423723	9.256085253	10.66706732	10.57996368	8.922082808	11.47127063
16.12384164	17.01900663	16.8710039	17.12376032	16.90924948	17.29704085
14.62217632	14.80749806	15.03840752	15.41210544	14.67552064	15.26374094
13.78737352	14.24162707	14.09521817	13.5360606	13.66158872	14.13521971
14.70594079	15.21350605	15.16803204	15.42966281	14.88238784	15.31193978
13.00101781	13.57574016	13.48990673	13.86604428	13.66784462	13.79381649
12.68903729	12.9851923	12.95787308	13.1944969	12.88265743	12.9709957
13.81266894	14.3093669	14.32804886	14.90726461	14.17411554	14.99939481
11.82852227	11.96362869	11.97447062	12.51424883	12.09225922	5.84150103
12.06964699	12.41634374	12.3543152	12.80949032	12.43279956	5.766484141
11.01209098	10.57282784	11.25700815	12.06098883	10.9889639	11.53473316
15.42966281	15.99508933	15.95256028	16.28343863	15.70163214	15.99508933
11.18941159	11.5737214	11.34732253	10.48432644	11.36245739	10.7814083
9.380344141	8.804477505	8.640789455	8.892970697	8.179120646	8.361756343
8.31008898	8.129067798	8.372433475	7.017237545	8.312729141	8.136641818
9.391460545	8.137860071	8.27861104	8.630877737	8.103690354	8.273878929
12.66985986	13.10985871	12.94821125	13.54221797	12.95303817	13.00101781
15.95256028	16.8710039	16.82803465	17.23317693	17.1822602	16.90924948
11.542568	11.62494396	11.71244686	12.12151457	11.49839615	11.96636849
9.805520727	8.982611727	9.289535939	8.517695303	8.873520545	9.273022949
10.31095185	11.12454893	11.07493153	11.18941159	10.95234717	10.9088749
7.237206848	7.618249414	7.821625848	7.682204525	7.906781182	7.460591212
10.19528987	9.727935707	9.887613414	9.541041677	9.474605101	9.631972414
8.267698071	9.164545343	8.708030737	7.876138384	8.946596606	8.34368697
9.395177889	8.830660505	8.546982556	9.260607	8.898709273	9.119831202
8.158968566	7.861535657	8.282438737	7.962380919	8.031407364	8.075284384
8.615838	9.083185818	9.382205394	8.48742196	8.712341879	8.230181798
7.670568384	7.937009	7.825104404	7.186960111	7.937868929	8.098302202
10.51886057	9.495686737	9.778131475	9.222631263	9.380344141	9.751080646
8.800782677	8.084275677	8.068200283	8.056742909	8.257595869	8.270601414
6.929106091	7.422534091	6.814458515	6.739060495	6.981086404	7.621699758
6.53485901	6.878162091	6.284543212	6.895441525	6.272739152	6.301916869
7.681167667	7.527074182	7.566119051	7.809982667	7.322217273	7.488273475
6.815206394	6.919943293	6.83457999	6.126729404	6.951169364	7.030162636
8.35752198	7.582311101	8.037664566	8.263866778	8.264240727	8.238272808
9.879566364	8.476994374	7.893273434	8.547449101	7.879985051	8.161911788
9.119831202	8.959665061	9.051375475	9.606954152	9.156840283	9.350012182
7.708922879	7.001612657	7.677935313	7.106592788	7.19681397	7.138827606
10.70643744	10.60863639	10.71031814	9.777520899	10.52247938	10.27236099
7.948737212	6.615162343	6.891253768	6.88247998	6.637000293	6.893369586
8.781156657	8.822318	8.575095707	8.838495424	8.655934636	9.70034896
12.14789109	12.47761486	11.97660652	12.77841715	12.28693608	12.28985985
7.442598384	8.713417869	8.277405556	7.860764394	8.457043091	8.104866828

8.953653768	8.369612525	9.104371838	8.435296202	8.302498081	9.110297333
9.782133848	8.712915394	8.956909848	8.972927475	8.736221495	9.042944374
10.19236777	9.97079498	9.285000091	9.61181703	9.793126455	10.10187506
8.041332778	7.834787162	7.78118699	7.722652869	7.363924808	7.350971899
8.038729465	8.119288848	8.167813576	7.117115202	7.826151263	7.840343939
10.55474917	10.59541023	10.37467131	11.28207759	10.78987961	10.77252405
9.359783616	9.894213939	9.859664869	10.22951336	8.879851283	8.989652131
9.380979222	10.35178281	10.23247836	9.793126455	10.42297323	10.11051676
12.27421104	11.86125412	11.84291489	12.13244857	12.05489479	11.97660652
10.9991534	11.6445291	11.45932673	10.11246778	11.12151875	10.67572435
13.60979651	13.20018245	14.01682491	11.89628941	11.59212007	12.17513939
14.42673578	14.84412351	14.83117585	14.38889659	14.4335752	14.4185289
14.89443037	15.92931651	16.2036826	15.83165575	15.76560628	15.61926687
16.64557528	17.43906183	17.58093558	16.68963031	16.8710039	16.73234105
13.79381649	14.70594079	15.08134613	13.92894576	13.82538175	14.25895571
5.874835889	6.410887212	5.88773696	6.794655232	6.660795162	5.755626687
12.7494647	14.57091557	14.30038792	13.40302459	13.43523914	13.47832004
6.743803525	8.276867707	8.034046273	7.704713596	8.189547909	7.959080606
8.291166909	8.110881556	8.037984616	8.447526838	8.291610121	8.376150616
14.65397593	15.32551587	15.13975511	15.19830883	15.13975511	15.02501841
7.733228788	7.683318778	6.963895283	6.986482212	7.632188616	7.756889051
7.28082202	6.852150101	7.054536222	8.027386222	7.56165098	7.161069364
6.831893455	6.906817121	6.524488081	6.374173354	6.336448141	6.987320737
8.287081333	8.07563398	7.553773697	8.300483646	8.345781111	8.737817051
7.041898303	6.955646677	6.893369586	6.833605444	6.613466586	6.759458
9.784374313	9.828404283	10.02269473	10.52904685	10.06997663	10.70902317
8.237365566	8.126700889	8.43615796	9.471411141	8.966940586	8.800397414
8.886620687	8.893968333	8.813595202	8.953164051	9.471411141	8.907915899
9.800083455	8.198599495	8.412578899	7.727390434	8.21550303	8.288355808
9.660200838	10.03701736	10.02821235	10.36465285	10.08231743	10.34717129
9.35371997	9.998242414	9.789131121	8.641223283	9.75438504	9.590351455
12.23505088	12.71520416	12.84197228	13.20018245	13.12562627	12.79024082
11.32559397	12.01427079	12.10694646	12.0724233	12.04990566	11.85033795
10.60383217	10.57758511	10.70425349	11.31852116	10.69682519	10.98627861
7.672313364	7.595878485	7.675893758	8.548304869	7.860050667	8.31214998
8.173633596	8.827377505	8.539328929	7.503692152	8.547449101	8.500729384
7.973586899	7.916025434	7.997875909	8.260520646	7.249695535	7.377888556
9.739033131	7.705120374	7.933961929	7.751275798	7.564402343	8.150096222
7.88825004	8.145194646	8.025864485	7.06536003	7.923423949	7.554702939
10.90466263	11.29597305	11.02403513	10.06255975	11.18291631	11.05356995
8.118473111	6.493067	7.667309091	7.261194687	6.987320737	7.25519196
7.166060222	6.394492141	6.847044404	6.593634414	6.466022525	6.612316919
8.056742909	7.596851556	7.854685616	7.223856	7.901142576	7.768772758
9.898158111	8.481851586	8.553132192	8.475210818	8.471953717	8.755421576
12.18792926	13.48990673	12.92937388	12.52819783	13.09045797	13.27940818
8.756886111	8.578393657	8.94874	8.918756414	9.200683333	8.773988747
9.362238061	7.652274172	7.862492343	7.722297051	7.240898899	7.985458788
7.050347636	7.835124576	7.576037556	7.179751081	6.84607197	7.852302727
7.292561596	6.853963192	7.194433949	7.387654737	6.413868758	6.275248485
10.43332542	9.515069697	9.843921515	9.196689051	9.007167758	8.991970091
9.449254061	10.69354222	10.72633412	10.42622906	10.63266161	10.88430477
8.82842597	8.193820172	8.377562293	8.757866131	7.922041747	8.699656879
7.363288879	7.314300525	7.206091434	7.314616131	6.624843121	6.875911141
6.738778758	7.523479626	7.457632404	7.375790182	6.794126838	6.988974929
9.86223396	10.01071956	10.75865445	9.488971182	10.2026692	10.55213977
10.01340346	10.67428006	10.8254891	10.71279176	10.75865445	10.44712342
8.721523222	8.347358778	8.03364202	8.824851909	8.870602515	8.728878424
11.93770961	10.09079019	10.12799521	10.17440526	9.61181703	9.62302801

9.713188212	10.15585749	10.31013098	10.35385551	10.67572435	10.30058205
8.573727141	8.194999071	8.318668263	8.821324343	8.428657566	8.465000141
11.308834	11.61814895	11.51647662	12.17513939	11.79171062	11.97944932
8.983202444	9.324388263	9.221294354	9.738398616	9.603529667	9.219562515
6.808998949	6.582439566	6.423456465	6.516258222	6.778470374	6.620195253
8.877161475	7.873714818	8.039068889	7.919459465	8.088956818	8.164979273
9.156208313	9.959124273	10.01622438	10.31378621	10.00361533	10.37215733
7.388945192	7.191807606	7.295703091	7.091827414	6.975683768	7.31258798
9.907546465	10.20452777	10.08700287	10.98627861	10.29000276	10.60044575
9.575401111	9.874496818	9.823678374	9.964343626	9.656571869	9.891739697
7.814874545	7.078835444	7.415013667	8.068200283	7.151567818	7.062438556
8.316081222	8.631321253	8.298896434	9.002847848	8.814624313	8.339926909
9.997192202	8.350987111	9.036076414	9.014846778	8.776574232	8.994589434
8.156772778	9.039989364	8.647922808	8.039068889	8.961327374	8.641737071
6.482726162	6.878694758	6.525823616	5.933607051	6.997628374	6.344188131
10.55602586	10.18000397	10.42927797	11.00183361	10.51771241	10.72384747
11.01951417	11.34732253	11.26815786	11.66934136	11.44319437	11.49839615
9.658049545	9.475206131	9.908305848	9.192533242	9.661659101	9.775265525
7.481001515	7.10439698	7.491357222	7.731542758	7.855388333	7.286993737
11.71244686	10.53842125	10.84568098	11.54675633	10.61001127	11.15503783
13.03496614	11.97660652	12.15752365	13.24400831	12.42233701	12.80602179
9.215946081	9.692793657	9.807061475	9.073600626	8.738283333	9.191263899
7.918848717	7.717737636	7.671246505	7.673376626	7.273224596	7.385541293
12.06397065	12.38063147	12.24649354	10.67939021	12.33907422	11.91416468
8.415109929	7.848409778	7.98285402	7.314926455	7.723384495	7.150169929
8.941359636	8.27018399	8.442251212	8.170954818	8.233421293	8.06745399
8.796713535	7.242080394	7.355380455	6.998704808	6.962306323	7.228468091
11.45002291	11.61117883	11.6295292	11.87687449	11.83813795	11.9091429
7.507667889	7.878518051	7.723687384	6.900488939	7.493863788	7.870978364
8.592622071	10.21484784	10.36589334	9.555296242	9.876880455	8.804952828
11.99535117	11.59021288	11.54871614	11.65261158	11.47127063	11.68948846
12.28369472	12.93430142	12.88708268	11.68243664	12.44950322	12.27773509
10.19418334	10.29324401	10.573928	10.7982674	10.62280049	10.82034332
7.621361828	7.226432323	7.196550485	7.228759818	7.001869131	6.916453859
11.84027237	12.14191765	12.46055504	11.26446827	12.01427079	11.56988377
7.90331499	7.350114798	7.495760485	7.517436172	6.858444758	6.939171505
11.37490296	11.75662781	11.93975968	11.565007	11.3290504	11.17041501
7.400588576	7.190644495	7.729367111	7.328721848	7.637262101	7.708922879
8.755916071	7.326042475	7.88791003	7.729749768	8.090886384	7.983913121
6.005249616	6.00822299	5.977641838	5.811609788	6.229994768	6.707625232
10.30470526	9.597513697	9.462343535	10.35284213	9.809529798	10.64005918
8.31214998	8.136235949	8.220625586	7.782178323	8.597027768	8.431326525
6.507609283	7.085030667	7.085030667	7.482291222	7.303019677	7.113452899
7.42989803	7.695611414	7.386471808	7.774939586	7.589604303	7.826840444
7.143149899	6.396397444	6.318299141	6.212710222	6.196644333	6.398234566
7.837840162	7.732919727	8.066736566	8.357077121	6.86140099	7.190930768
15.32551587	13.14611959	13.27418186	12.47761486	12.84197228	12.7494647
10.7814083	11.52052132	11.15808934	10.64128512	11.40145091	10.81664077
10.26729954	10.74690378	10.83790729	11.38025327	11.32559397	11.34892675
11.64637724	11.84529477	11.81221198	11.96947069	12.03602449	12.14191765
7.270452455	7.372532788	7.019424707	6.245816848	7.423727141	6.804494667
9.009407333	9.297353606	8.996148737	8.598479657	8.745474636	8.865407737
8.950355889	9.667708556	9.782133848	8.968910758	10.33596201	9.863774293
8.245222778	8.51269104	8.799825313	9.712341424	6.987599808	7.45822897
10.36239309	10.86820493	10.72246175	10.77507269	11.13879804	10.87235819
10.35069541	10.12697515	10.02644328	9.406893465	9.976273051	10.10093339
8.495447152	8.860747333	8.712341879	7.689234404	8.609511	8.572701232
9.242021586	9.662314131	9.728754818	8.842109	9.545881535	9.641007131

6.925960677	7.381950828	7.200097273	6.215863646	7.126018333	6.923688879
7.889031475	8.018337859	7.409045616	8.041000495	7.883536596	8.267698071
7.142295949	6.749564212	6.738778758	7.062669222	7.329870525	7.56320098
9.954681949	9.491039162	8.748883111	9.376046232	9.247858869	9.679645121
8.291610121	9.308918081	9.142791657	7.749299101	9.071276071	8.744081071
7.440186081	7.107957081	7.13936198	6.965182525	6.37918304	6.496990475
10.4700758	10.91176188	10.77507269	11.04464523	11.27695531	11.17571904
7.377037515	7.851994586	7.758961677	7.910975505	7.946141838	7.967524909
11.96362869	12.83405786	12.86955734	11.60266156	12.39348455	12.59323492
10.33890802	9.908305848	10.14534091	9.360502949	9.511202364	9.66383
9.835894606	9.492465394	9.729555121	9.737663949	8.860747333	8.289212586
6.601482788	6.266853091	6.775968394	7.152088687	6.108765172	6.081659081
11.15503783	11.52448673	11.68243664	11.45526933	11.33657706	11.32721575
7.366317111	7.432999727	7.93437196	6.523375646	7.488273475	7.198026636
8.428657566	8.666601818	8.514037303	7.552094283	8.804952828	8.091323071
6.178323768	6.536641737	7.203788636	6.912275354	6.248351606	6.222757071
11.52238659	10.61992902	10.69099899	10.33476777	10.4549623	10.75600216
9.567720788	9.238221677	9.320176919	9.505859384	8.108523939	8.893968333
6.716118697	6.044454283	6.087779919	6.214981424	6.060613939	7.219623727
12.51424883	11.47682743	13.13522491	12.9851923	11.37847359	12.53563192
10.26116661	10.70425349	10.52476206	10.31193963	10.77387872	10.5041569
8.821885465	8.069395848	9.018802576	7.21289504	8.947623838	7.669657737
10.06255975	10.22557955	10.53505365	10.38738452	10.6240143	10.51174846
7.376100929	6.35978	7.514821949	6.903822293	7.379463242	6.94531798
6.967663404	6.913511828	6.852390212	7.200097273	7.536179778	7.221869232
9.968215758	9.600888859	10.09994749	10.84568098	9.672623929	10.3025572
11.9091429	11.81221198	11.16888788	11.92937262	12.21630458	11.39594259
11.10761793	11.71042621	11.68484165	11.4481816	11.43565115	12.03602449
8.825911939	9.779012071	9.089024899	8.641737071	10.11051676	8.662816202
10.11452638	10.4322159	10.91514001	8.776574232	10.3090972	10.40592026
7.631889737	8.356302828	8.195405505	7.598885727	8.145617202	7.989204111
7.764609818	8.428657566	8.363019899	7.079662576	7.862160495	7.574795778
9.025193869	8.533396525	7.754082929	8.331888859	9.46304199	9.315671101
10.29542336	9.187808616	9.246644323	8.957404525	9.033915556	9.270407879
8.91530399	7.561964414	7.795429838	7.790604293	7.245319404	7.572084505
10.93334198	11.70812672	11.85889751	10.25906505	11.68484165	11.45002291
11.09250749	10.61621963	10.49348589	10.23160947	10.4322159	10.46232362
7.931306778	8.564366949	8.026271202	8.358418384	8.553132192	7.911309091
8.53715797	8.583996586	8.903025495	8.348145404	8.681067818	8.092691677
7.335553323	7.987188889	7.911629535	7.076589626	7.868722081	7.449265303
8.834473929	9.470173818	9.393335687	8.382609505	9.153232364	9.08959502
8.941795242	8.495447152	8.460053242	7.797844434	8.380307172	8.467667768
7.314300525	7.928125404	7.61520499	6.659100485	7.395300939	7.500167182
9.889177586	10.50543333	10.45375986	9.145664475	10.31693391	9.876880455
8.408718253	8.276438626	7.919459465	7.457078101	7.822322859	8.253639283
11.35080354	11.07337622	10.73014715	11.84529477	11.10761793	11.23280633
9.377908	10.24942024	10.05236285	10.69470446	10.34717129	10.4994288
8.954798808	9.317615606	9.083185818	8.040196323	8.976374343	8.969483
10.55726192	10.35284213	10.77890896	11.24147284	10.94208941	11.41415047
10.95374716	11.35080354	11.24646499	10.93789932	11.41238356	10.79564384
9.622300838	9.840616414	9.174793152	9.432636424	9.682548525	10.29000276
7.500501152	7.194677455	6.907839071	7.05790099	6.982384606	7.865549818
8.251492909	8.122747707	7.88825004	8.199709495	7.590787525	7.721939444
9.007843273	9.380979222	9.508442768	9.890135131	9.633493677	9.636929838
10.5336638	10.44602022	10.38631174	10.96311364	10.71390019	10.68572341
10.0143126	10.86173253	10.43332542	8.949261586	10.42062148	9.833438364
7.593038414	6.905589111	7.353244222	7.85604701	6.977041091	6.935285707
10.73661507	11.15183093	11.37490296	10.26445444	10.88681216	11.15183093

9.541041677	9.818059192	9.769223525	9.302103889	10.24414187	9.704210374
9.004940778	8.788135667	8.799348343	8.814101707	7.314300525	8.205133263
13.87263802	13.35359128	13.52959744	12.8652792	12.89513437	13.08418191
11.81439214	12.04734694	11.9875501	12.27421104	12.52461444	11.66708917
6.722265657	7.20918101	7.761029182	6.927259889	6.761831111	6.883542434
9.886023253	8.268937657	8.407442505	8.507211606	7.483257202	8.015705778
9.342353505	9.819688798	9.666877556	9.425521616	9.775265525	9.069043081
7.998937818	6.868868606	7.109111172	6.436179667	6.899979889	7.038648475
7.504556444	7.01049403	7.091827414	7.062959768	6.912275354	7.433328091
9.274850788	9.331636121	9.303966081	9.625041525	9.745446081	9.93187304
7.942803596	7.41959598	7.578628212	8.326884343	7.75479003	7.718083212
10.85361094	7.783847071	9.900778232	8.589797808	8.251125323	8.879851283
7.312252182	6.729463253	6.847553121	6.475338556	6.525168444	6.625965465
8.45451298	9.061091758	9.170513566	8.739635162	9.295516616	9.026636737
8.558556152	8.185943182	7.231823384	8.305359949	7.107120283	7.723687384
12.55983323	8.964085616	9.003925646	9.204855576	8.892970697	9.806332162
9.491039162	8.050105303	8.080354404	7.868722081	7.393766606	7.582654121
8.666113404	8.083485404	8.346989566	8.750916455	7.926719768	7.939903758
6.977817737	7.210091465	6.998508111	6.33399804	6.924744576	6.963095333
7.093254121	7.118512586	6.881514152	7.509707515	7.031184939	6.967234434
7.490096869	7.528640475	8.389653889	7.882189879	8.752912545	8.21550303
8.192996515	8.359263212	7.953486636	8.346585616	8.143776848	8.633351606
8.996667384	8.762742172	8.739635162	9.077100232	8.841575667	9.106199707
9.890949131	9.031155364	9.219562515	9.399129424	10.1309568	9.502644929
9.022478232	9.721958051	9.517013525	8.017104828	9.574050242	8.542429747
7.912742707	7.489812667	7.74961303	8.072532475	8.355387535	8.31049196
10.98186414	11.16281786	11.11081747	11.34381562	10.98627861	11.15342364
8.641737071	8.342773172	8.330618758	9.036569232	8.13932801	8.450171172
14.45281427	14.68633385	14.91973608	14.010587	14.45281427	14.86987024
10.45628541	10.58117749	10.65246846	10.97631922	10.50543333	10.03299879
11.05191968	11.15666056	11.12613667	10.14131493	11.17910752	10.99046505
7.591751808	7.409335747	7.444173596	6.353596172	6.755203192	7.206373697
10.05607537	9.821914929	10.15379546	10.5199714	10.31095185	10.14999886
10.61261659	10.4283734	10.82034332	10.86566339	10.86566339	10.49009312
9.190767364	9.30770201	9.360502949	8.112161727	9.291911616	9.07526898
7.952029848	8.280295667	7.485478828	8.93638097	8.338686465	7.562627657
9.657223606	8.739635162	8.657372182	9.744663444	9.161011869	9.298556212
11.13713447	11.68038318	11.83330316	11.36423723	11.37670858	11.14866374
7.356542485	7.688677182	7.763504859	7.523756919	7.842052606	7.487647384
7.446760616	7.508388697	7.665987182	6.85163704	7.647964475	7.647631172
10.66706732	11.45738434	11.31449746	12.01427079	12.2031413	12.0089388
6.107491061	6.351069404	6.365147848	5.873898899	5.985642434	6.043729879
9.687048303	8.594016788	8.948168879	9.061091758	7.638629778	8.323157747
14.25042984	11.17910752	11.47322198	11.15808934	11.14707085	11.86922315
8.128290707	8.264240727	8.541524636	8.693780273	8.843670869	8.676316919
7.194433949	6.922922101	7.060512141	7.551492101	7.710578727	7.454260455
8.69723302	8.903602263	8.973478152	8.959130566	8.135850465	8.41335802
7.571427586	7.875022121	7.889761727	6.754943232	7.819266253	7.549206232
15.61926687	17.1822602	17.1822602	17.1822602	17.36803328	17.07010739
9.445197818	7.662615848	8.750916455	8.12156602	7.710917071	8.168969465
8.625486818	8.663769939	8.496814303	8.995099212	9.388273121	8.153159131
7.191229121	7.233039222	7.764609818	7.712540141	7.601643838	7.425203606
9.823678374	9.84311402	9.644739414	10.02269473	9.684886576	9.912769303
10.00186072	9.655088667	10.01703805	8.965143333	9.395177889	9.803356646
7.584965596	7.451183323	7.398020586	7.452458778	7.967113929	7.601973414
7.689234404	7.946933485	7.782178323	7.267645253	8.177906263	7.347734202
6.523206192	6.067526697	6.111914111	6.244370242	6.928572354	6.694950828
8.485174677	7.775269606	7.820222687	8.847386828	8.106418192	8.384333949

9.76471501	10.06071544	10.4655823	10.03611568	10.43872423	10.48074239
9.114524374	9.534194535	9.119831202	9.660200838	9.317615606	9.355403384
8.65497299	9.447242061	9.317615606	9.021393263	9.413321525	8.958493495
11.02851208	11.42562271	11.43005301	10.55333109	11.41071085	11.07493153
8.243245818	8.727989172	8.951513343	8.211411717	9.048620525	8.781680606
8.240781051	8.450646323	8.681579566	7.973586899	8.669992818	8.544134919
6.419328293	6.636727626	6.804494667	6.876146636	7.32338401	6.470357717
10.20964764	9.962571606	9.790672212	9.630502939	9.815129384	9.736957071
9.499247869	8.35796399	8.547862646	8.373919657	7.831503424	8.714332636
10.40178901	8.858269384	9.328020364	9.199527535	8.802385697	9.105035414
9.382205394	8.878776172	9.409395929	7.848096879	8.34368697	9.221294354
7.488608758	6.199121808	6.440833869	7.227000899	6.011944242	6.276206182
7.705454838	7.274379929	7.199149121	7.090603616	6.492200283	6.749564212
7.318216242	7.390752071	7.989204111	8.105629576	8.047933768	7.349485707
9.035586242	8.24281297	7.779426687	8.253639283	7.017803152	7.319114313
9.62302801	9.19736601	9.538263677	8.861728919	8.645985424	9.176146596
9.451851384	10.08325108	10.37215733	9.935661677	10.17613521	10.14534091
7.137450808	7.952774121	7.750957677	7.511581475	7.900715717	7.387965737
11.44643795	11.48655657	11.2345748	10.73014715	11.86652814	11.53239215
7.636934919	7.279669313	7.463067838	6.864750879	7.122942091	6.791252535
6.679714263	6.261563212	7.061330929	7.159453505	6.369666687	6.449263919
9.157435455	7.964179182	8.335469222	8.438325768	8.482406475	8.07651197
7.485478828	7.461239687	7.266525586	7.626110576	7.617890202	7.645943414
6.740256788	6.678083162	6.668303455	6.822905505	6.629982465	6.59646801
7.719835253	7.74007802	8.326884343	8.090488788	7.410622798	7.605411525
8.703161646	10.25220538	10.28506122	9.602761333	9.505859384	9.612498475
8.963499596	9.342353505	9.25368898	9.998242414	9.960013465	9.689129545
6.721549354	6.981335535	6.885064545	6.418892273	7.045715273	6.657421566
8.83754499	9.734703111	8.924201657	8.274698687	9.391460545	8.982611727
11.79870316	11.50425191	11.47496104	10.99046505	11.41981305	11.61814895
8.004282899	8.597538556	8.242002535	7.713612677	8.516311424	7.780448354
10.71731517	10.63752253	10.93789932	10.65500785	10.61133106	10.4302696
9.392082222	9.978126798	9.942152202	10.06356315	10.14713571	10.08893073
11.09565519	9.007843273	9.563605545	9.187244566	9.194378495	9.318228697
9.66831603	10.35069541	9.977292899	9.680349586	9.922126404	9.894213939
10.56207051	10.21157382	9.93187304	10.4994288	9.385290182	10.18000397
10.44712342	9.749660394	9.984347768	10.12909315	10.08700287	10.15783547
9.833438364	9.589694828	9.638947465	9.890949131	9.616741535	9.459664354
7.439837192	7.614146707	7.893940788	7.02612702	7.569223354	7.660499434
10.40278823	9.736957071	9.311440606	10.49466708	9.734703111	9.354902374
12.88265743	11.38178745	11.79615897	11.75215821	10.55213977	10.95849458
8.403029636	8.200961636	8.412145273	9.300983919	8.62412801	8.824381182
8.508189222	8.722452253	9.167758485	7.925310818	8.724556485	8.614442374
8.484723788	8.442251212	8.784649081	9.163332636	9.518503263	8.941359636
13.38447499	12.8293171	13.35919327	15.27762024	14.10328164	14.14208591
7.300950758	6.897750404	7.046194354	6.478787505	6.394104788	6.746121293
10.80900786	10.48074239	10.10278369	10.61133106	10.52689599	10.69682519
9.796940758	9.304577515	9.600185283	10.48310631	9.750460616	10.24828231
9.813557354	9.851320949	9.70034896	10.42512206	9.843921515	10.2752886
12.53197264	13.42153085	11.66934136	11.85889751	12.43944324	11.81018832
8.539776737	8.30620801	8.790654051	9.11211799	8.629474212	9.007843273
11.19114259	11.59427705	11.29744903	11.18096106	11.32376318	11.2786408
8.545091384	8.882882525	9.007167758	9.316888828	8.646531515	8.60808904
7.373377929	7.758961677	7.828991111	8.57597697	8.517288667	8.169382444
7.444173596	7.399710364	7.42612497	7.756175162	7.750590293	7.516462556
8.569209263	8.989142232	8.761644081	8.754900343	8.803935202	8.888204949
7.716479172	7.539809273	7.628336929	7.550541152	7.65951298	7.640655485
10.98627861	11.308834	11.56291631	10.97054498	11.49420288	11.39983314

8.048299333	8.461315273	8.653441707	7.252299788	8.191909586	7.907924051
8.30620801	8.677215515	8.523456192	7.557888444	8.738735232	8.302498081
8.676316919	7.358963616	7.83046296	7.518690889	7.238429162	7.646666444
11.99323436	11.50261564	11.5612151	11.01951417	11.18636156	11.50051559
8.142577798	8.780633818	8.62460002	8.099825778	8.574218515	8.395713444
8.710391727	8.403471909	8.357077121	8.986932636	8.682101313	8.331046747
10.73014715	10.62764398	10.75453103	11.8358519	11.238428	11.02700786
10.03701736	9.664626495	9.975431768	10.03876748	10.4283734	10.13375911
6.71121296	7.052522778	6.709545626	7.345589182	6.327126061	6.437269283
7.843485626	7.698300838	7.520306162	8.22344601	7.26571197	7.183662697
11.238428	11.39382595	11.53677901	12.31719428	11.53473316	11.78265022
9.740584727	10.11760527	10.03796113	9.562994313	10.01703805	10.00886723
7.051685909	7.049242879	7.111963455	7.073400869	6.541370778	6.772767788
6.18856802	5.962981202	6.464252303	6.324159687	6.148849848	6.160016172
7.967113929	8.546545061	8.153957263	9.013314737	8.596127869	8.474658303
14.68633385	16.06827159	15.81050193	15.67809237	15.83165575	15.38120792
8.813595202	9.048620525	9.456392222	9.643287657	9.304577515	9.23383296
10.25422487	9.299916465	9.230770667	8.665646899	8.879330566	9.259301556
7.407756293	6.898276768	7.721939444	7.007335798	6.356712303	6.469862081
10.06997663	8.580722586	8.713915657	8.461315273	8.51269104	8.765233303
12.91196246	8.785177889	9.002272697	8.457489293	8.391460737	9.09824599
7.232456929	8.013857071	7.958031758	7.404680798	8.06745399	7.731871869
10.76737368	8.603745778	8.904239657	8.843670869	8.878201818	9.67665103
10.95849458	11.238428	11.44319437	11.19966733	11.02105469	11.43565115
8.470647758	7.140269465	7.148192263	6.905589111	7.092673939	7.315791616
11.60069924	12.15440913	12.23505088	12.23505088	11.8358519	12.02811467
8.285312424	9.006527424	8.76386996	8.442251212	8.342368121	8.458697343
7.368846081	6.349206475	6.715644404	6.913051818	6.627836202	6.699888758
9.54312803	7.620680879	7.845601525	7.464704768	7.186028475	7.864861071
8.337550606	7.82475102	7.945291869	8.529417101	7.341641707	7.399710364
15.99508933	14.5513462	14.88238784	14.76275492	14.60195877	15.06622261
7.507381747	6.821271707	6.851905687	6.586881556	6.575249545	6.450820343
8.346198293	7.204326061	7.249962576	7.012316737	6.863923949	7.07011003
6.987599808	7.847720859	7.927460424	8.434806414	7.286722061	8.276014121
10.5397012	11.2278827	11.10460602	11.24646499	11.08756575	10.97767761
7.981709394	7.973586899	8.010919919	8.23581501	8.382609505	8.239069586
7.286117455	7.879657162	7.966054535	7.553468394	8.045324071	7.694540808
7.714642404	8.285708747	8.092691677	7.24440804	7.799524616	7.675167556
7.396159798	7.533990333	7.451183323	7.904747051	7.728706424	7.604463141
10.71613341	8.899757485	9.157435455	8.678658293	8.923097444	9.272374697
9.180285111	9.290152727	9.173615515	9.679062616	9.197870838	8.963499596
7.170490939	7.240600646	6.696224707	7.576037556	7.32157099	7.688011354
13.34204398	12.72972704	12.56315114	13.26910509	13.86604428	14.07988992
8.421742051	8.885078273	9.064430111	8.931557869	9.185497838	9.126080354
5.857854657	7.18511501	6.737098263	6.413218091	7.77328201	7.037826263
12.11841559	11.542568	11.58421758	10.45754412	11.23116994	11.48061491
8.40783399	8.175288444	8.181196919	8.036387556	7.817975838	8.40702104
6.549138121	6.946968899	6.533739475	6.365312818	6.095984313	6.267871525
9.928455091	8.655437626	9.679062616	9.157435455	8.716387192	8.892970697
7.019151556	6.907093253	6.943066869	7.558170465	6.975426091	7.164674212
8.281167657	8.223899788	7.840025778	8.82842597	8.193428737	7.899333596
13.84455043	10.43564423	10.59292728	9.814415596	10.3896003	10.5397012
8.246913576	7.069787606	7.586125333	7.921614596	7.909031556	7.807154444
8.552208182	8.40783399	8.183195545	7.752031929	8.165380303	8.115023838
7.659822475	6.70133698	7.272331859	7.335553323	7.469344313	7.413101485
13.33661988	13.65450155	13.58209631	13.62106704	13.70143341	13.56886427
7.655304091	7.037511364	7.009472515	6.908606212	6.606886212	6.792458242
7.285287818	7.660499434	6.989443677	6.436610747	6.562503909	6.441470343

6.943550111	6.806487737	6.903511051	6.626438091	7.140902202	6.975188859
8.178268222	8.122341	7.177177071	8.052783737	8.252372808	7.285574899
6.329319202	6.850608899	6.684069253	6.36310503	7.020179424	7.057634667
10.18666124	10.91903182	11.00053539	11.02403513	11.31267384	10.90057618
9.727055768	9.922126404	9.788274313	10.10542249	9.636296737	9.580097263
8.109636222	8.245649515	8.070136677	8.35796399	8.45110203	8.088117667
8.375668162	8.251125323	8.47107903	9.121511687	9.20200202	8.845226141
10.80239406	10.88166557	11.75462574	11.32721575	11.2518391	10.95093974
7.220481808	6.882244081	6.889002121	7.145208253	6.390557101	6.567922788
9.901640636	9.076382242	9.547339828	10.54565144	9.208368677	9.621550919
8.584830636	9.127872697	8.980444384	8.368326869	8.912452162	9.074692404
10.8443678	9.443424081	10.46342714	11.542568	10.36903954	10.09079019
10.35809493	10.09896607	10.32750324	10.75178718	10.50914282	10.71731517
8.747331152	8.616405606	8.403930677	7.949400121	8.556357949	8.81966502
7.923778798	6.652872152	6.751169162	7.040256253	6.705162535	6.865784889
9.355403384	9.242021586	9.438799404	9.891739697	9.428845576	9.421059
7.735579636	8.00874802	7.758192424	7.147201253	7.940978939	7.760648222
10.83223099	11.54053762	11.70812672	10.47820756	11.41605585	11.17378617
7.70790696	8.152087687	8.115023838	7.222444626	8.01013803	8.028141747
6.66391404	6.635354444	6.620195253	6.649151414	6.746340717	6.671323232
8.881310737	8.000521869	8.550414556	7.32157099	8.234232515	8.580722586
8.611710212	8.556357949	8.397394545	7.717096556	8.347717242	8.445759657
6.490008293	5.933733586	6.109845465	6.714232707	6.358295333	6.258944949
7.201570444	7.346280818	7.663954061	6.476638242	7.689234404	7.230210646
8.076118121	7.731542758	7.112898869	7.765675424	7.218399283	7.39712799
9.369545939	8.868979273	9.421700798	8.914720172	8.939019091	9.203189636
8.23139899	9.157435455	9.056533626	8.883993253	9.094691273	9.248396293
7.024510747	7.194433949	7.246997929	7.861535657	7.553068374	7.100167172
8.012010707	8.412956141	8.577417788	7.876418838	8.235056909	8.173308798
7.278747747	7.692217293	7.602282465	6.612060818	7.675499444	7.496977949
9.476588061	9.857104919	9.639623879	9.548618051	9.807061475	9.522660222
9.180906778	9.785178838	10.07388225	9.949411172	9.716648152	10.73264046
7.899333596	8.339546939	8.269818727	8.72650097	8.167813576	7.856391758
8.185563303	8.808928141	8.632349838	8.000521869	8.64224801	8.155735586
13.21058954	14.05855866	13.24400831	14.07245549	12.17513939	13.9071606
6.814222283	7.333224889	6.528629101	7.833640626	6.241411051	6.382025798
7.735932323	8.124212202	8.518144394	8.345353354	8.145962667	7.645645939
12.40735915	12.68903729	12.66167346	12.42616911	12.53563192	12.32364634
9.250770152	8.44047504	8.690381253	8.101068051	8.270601414	8.233421293
7.697220202	7.096649162	6.739522626	6.658365848	6.629250202	6.593877636
8.792768747	9.11093099	8.916479111	7.901142576	8.975838535	8.680072242
6.68968302	6.786546495	6.380219	6.09420704	6.459603455	6.083800768
13.29117716	13.76702458	13.63435472	13.56234785	13.99495772	13.3718573
9.581418808	8.799825313	8.722953263	9.324974768	9.057828929	9.453743545
8.85049999	8.796283929	8.871209182	9.484209455	9.058895657	9.266794162
12.10135206	11.74670945	12.29275278	13.29684189	12.04160066	12.33273925
10.84705809	10.02644328	10.08799683	9.956562586	9.495686737	9.432636424
8.336651323	8.501579646	8.703161646	6.960198707	8.437898414	8.267244596
10.63752253	11.11081747	10.96162926	10.0406788	10.90606985	10.64379219
8.527598192	8.454954939	8.461795505	8.812517081	8.875711838	8.823394192
7.141146879	7.964914919	7.684267263	7.889031475	7.496977949	7.650231051
9.06333597	9.362238061	9.451851384	9.376667182	9.306330646	9.015412101
9.231946182	8.975268424	9.067241768	8.246470545	8.446641939	8.764299566
7.996393263	8.070920919	8.981479273	9.007843273	8.825312061	8.785177889
6.501249222	6.563203242	5.923322192	6.481687929	6.451227182	6.871668566
6.928572354	7.715032394	7.678291495	7.276277889	7.851640596	7.545335788
7.316995263	7.308713444	7.179153636	7.574140061	6.913511828	6.768428949
8.908935182	9.598893818	9.516398909	9.541747455	9.562994313	9.438799404

12.85048632	13.40990013	13.30255942	12.66167346	13.63435472	12.89113428
9.621550919	10.36903954	10.41022215	9.558255162	9.298556212	9.907546465
10.97322727	12.44264271	12.34855768	11.82346178	10.54692936	11.60266156
10.67081044	11.42178201	11.6157426	10.64379219	10.62536143	11.1453264
7.840343939	7.541750556	8.41598997	7.666324061	7.555972677	7.576996152
7.083894141	7.195783737	6.759694808	6.217501929	7.168394253	7.137147091
7.274095485	7.927460424	7.97128602	8.528512253	8.566871343	8.067132556
8.645985424	8.813595202	8.913588253	9.290152727	9.245932475	9.104371838
8.942756889	9.353057535	9.302103889	8.378515131	9.127872697	9.022478232
7.165793091	7.379463242	7.416977434	8.058246657	7.510907818	7.823083152
8.912003131	8.750916455	8.524356212	9.11393401	8.898231131	8.891888576
9.276189121	8.693780273	8.693780273	9.132651727	9.036076414	8.934248131
11.38775416	11.18800276	11.44491584	12.24933389	11.22119175	11.38178745
8.208692758	8.340681505	8.791657869	9.437491071	8.649462798	8.777572838
9.247858869	8.668931657	8.743124222	9.586238192	8.978204141	9.142791657
6.857502071	6.903241343	6.512183152	6.731752374	6.34064704	6.414872495
9.531623293	10.43757507	10.1752482	9.460382818	9.791460222	9.582761798
8.977558859	10.27804811	10.34418014	10.68452674	10.06633871	10.28406896
11.0473733	9.615324495	9.588958727	8.721054424	9.092457495	9.57181996
13.07947925	9.952967535	10.22761479	7.416977434	9.389466253	10.5199714
6.062791	6.537953232	6.468467859	6.319689081	6.55365803	6.571183485
8.588536172	7.506434626	7.589252838	7.367244707	7.384605828	7.489812667
10.3494333	10.61133106	10.80900786	10.14809211	10.37346225	10.72900488
9.580097263	9.730279475	9.274850788	8.939630657	9.715215101	9.571117879
7.254341798	7.402701172	6.956636515	6.939947626	6.564057303	6.508047626
8.853585596	7.844877657	8.153159131	7.771143222	6.566725596	7.302140758
8.945002717	7.249066101	7.102116364	7.149668323	7.08160301	7.352232505
9.164000111	9.968215758	9.820433545	9.103235919	9.710054737	9.09013302
6.752477677	7.626793707	6.81111	7.537240869	6.155865424	6.130576222
9.466304111	8.23581501	8.593101434	8.521230828	7.549557061	8.024267525
6.026660879	5.887825424	6.532200808	5.751033333	5.807291182	5.799847364
10.10093339	10.5336638	10.55333109	9.208368677	10.22761479	10.37026232
8.892970697	9.184000242	9.185002364	10.05053825	9.624391323	9.490338525
8.341518747	6.987599808	7.116869414	7.375117525	6.872611121	7.151567818
6.468965343	6.93243001	6.597234394	7.367866364	6.983154111	6.910146768
8.730448515	9.400967465	9.40167604	10.02161965	9.869466131	9.955574404
7.587005707	7.447717303	7.552833101	8.081502051	7.551170919	7.805761556
10.4302696	10.42175216	10.30569114	10.73132595	10.5041569	10.57028524
11.69891546	11.98999321	11.49839615	12.39348455	12.40735915	12.18142186
9.001272616	9.25368898	9.000672535	9.726443586	9.875361242	9.554043101
10.8778129	11.5612151	11.22294817	10.31597323	11.24147284	11.04886125
8.600831152	8.937429899	9.031155364	9.419654929	9.53973704	9.185497838
10.81423156	9.217871889	9.803356646	9.583475303	9.492465394	9.817379515
12.19086998	12.78185519	12.4956701	12.33273925	12.53911948	12.99565877
7.063754707	6.967928273	7.141446111	7.830100949	7.454260455	7.327857495
8.482852263	8.509936919	7.809001414	7.82475102	8.186373323	8.449337667
8.59026096	7.981345788	7.656238101	7.552833101	7.41731598	7.763846354
7.083613121	7.637927172	7.473746677	7.627670859	6.678790232	6.998984111
8.628208788	9.311440606	9.347406172	7.936282586	8.977005354	8.906407303
9.440510152	10.00695165	9.532335293	10.13479405	9.173615515	9.114524374
14.16629104	11.91416468	11.96362869	11.25898056	11.86922315	11.92937262
13.96779242	14.7172184	14.80749806	15.06622261	14.76275492	14.72870348
10.03983393	10.20859114	10.0719806	10.39968902	10.14432724	9.883591758
8.352134333	8.220625586	8.168969465	7.242951465	7.951633808	7.921614596
8.369612525	8.17298499	8.581684657	7.209785606	8.099466788	7.819085697
7.053327354	6.605442354	6.813695162	6.723501929	6.229099566	6.545504717
11.34732253	11.49420288	10.99186298	11.81439214	11.53677901	11.31852116
7.393489313	6.99155401	7.582966818	7.920494253	7.349485707	7.493565131

7.456765576	7.310724131	7.330192545	7.537817576	7.876418838	7.356848556
8.805907646	8.598008343	8.82789604	9.400327152	9.421700798	9.442729677
10.042425	9.70798304	9.687048303	10.2166926	10.48310631	9.810255869
7.636262586	7.301802939	7.655968232	8.460863192	7.55178802	8.207099091
9.751080646	8.69335002	9.171164091	8.887684293	8.240375404	8.568768141
9.59460096	9.380344141	9.302735434	8.767872848	8.794361798	8.986432162
8.433939838	9.260036485	9.118059475	7.810980737	8.571676263	8.771294657
11.55524693	11.59870577	10.56802411	11.08435313	11.45738434	11.95514832
9.081927515	9.359207242	9.348735909	9.433258586	8.360024717	8.725113212
8.700142556	8.673843717	8.613130333	8.222643394	8.603745778	8.518658667
7.987188889	7.517077263	7.494798263	7.684641212	7.688370495	8.129067798
8.698751323	7.954939636	8.101068051	8.203151141	8.161033919	8.284073869
8.934777717	8.395713444	8.361756343	9.084956182	8.571194222	8.63794701
8.193820172	8.511305758	8.284073869	8.834473929	8.548304869	8.455403657
8.780633818	8.709941838	8.546545061	9.542425758	8.863920758	8.733877859
7.823083152	8.372433475	7.871683444	8.540202646	8.31912796	8.137860071
7.077700404	7.024222101	7.006478434	7.158855657	7.111428596	7.046726313
5.766778045	5.766548566	6.008494152	5.771639616	5.781531051	5.778343354
6.87215099	6.789755162	6.845837626	6.725273333	7.588284717	7.334066545
8.622197111	9.421700798	9.560980909	9.404333879	9.257979848	9.356741384
10.89094316	10.40701089	10.40278823	11.49420288	10.38208186	10.61001127
7.436640343	7.70365599	7.409710545	7.194433949	7.878518051	7.754082929
10.03796113	10.19332507	10.17789322	10.44832821	9.709426838	9.986114121
13.0147344	13.98081649	13.78737352	12.92937388	13.5360606	13.39659876
11.13551505	12.17829278	10.25127201	11.308834	11.565007	9.880317061
7.369510384	7.805068949	7.664957788	7.647964475	7.309928404	7.298462212
8.824851909	8.01602699	8.19621002	8.260123323	7.895961929	8.13932801
7.744142424	6.780217071	7.531511131	6.84678303	6.862647808	7.117905152
7.510027525	7.126519737	7.367866364	7.806845253	7.019697232	7.003455566
6.555968283	6.943812717	6.363322586	6.686049697	6.330092323	6.36270599
7.574489596	6.797589061	6.800553495	7.515816273	7.046480485	7.262654505
9.789131121	10.3090972	10.05688349	10.51291939	10.41224321	10.38738452
7.172125253	7.107390485	8.064713051	6.943812717	7.54886499	6.454504667
9.910074283	8.540635232	8.728878424	8.811517808	8.170586434	8.507211606
7.311327071	6.685554869	6.831893455	6.969919475	6.07088597	6.499744121
8.358418384	8.812013889	8.85518403	7.624735818	8.640338566	8.321152475
7.651222556	9.466909636	9.555296242	9.149684939	9.081927515	8.670884566
7.967524909	6.337204768	6.450820343	6.708797384	6.38878402	6.509844303
6.432015545	6.647920455	7.143440162	6.797872071	6.60058796	6.578458283
15.67809237	15.24813401	15.59877501	16.2036826	15.24813401	15.449005
7.963792818	9.001272616	8.783158111	8.790654051	8.971727333	8.573273283
6.690165707	6.659807838	6.865784889	7.019424707	6.736847222	6.766521566
8.595283071	8.584413131	8.660166475	8.020887778	8.221794202	8.022720303
10.73788216	11.15966862	11.33848921	10.43872423	11.01807842	10.79284767
8.464558212	7.772843323	7.992269263	7.836508475	6.921891778	7.446082798
8.217984283	6.976769172	7.385541293	8.449337667	7.485478828	7.315524667
8.429582192	8.338355444	8.474228909	7.718747404	8.665165081	8.347717242
8.857327323	9.221294354	9.146178758	7.893940788	8.992473788	8.861258687
8.414282596	7.554062253	7.532150707	7.955335374	7.423442323	7.867668606
7.638274606	6.794878162	7.018881949	6.697524253	6.702978071	6.929106091
9.584803364	10.91356479	11.07797425	10.00985275	10.98749259	10.32946621
7.545986354	6.86313897	7.159715333	7.105225354	6.47300398	6.659578697
13.9071606	10.54692936	10.81034167	9.980879424	10.31997281	10.42175216
8.693780273	9.030619364	8.884521697	9.500664495	8.627322343	8.433042727
8.08193004	8.141770949	8.03490996	7.393766606	8.402589283	7.925098076
14.59173737	11.15503783	11.64848719	11.50051559	11.70812672	12.24351077
8.040575909	8.422643323	8.466446051	9.02886299	8.408296424	8.585836293
11.37131374	10.24511785	10.43122177	10.35809493	9.383480283	9.618256808

8.327767232	9.592426051	9.099242404	10.60507136	9.039989364	8.946596606
8.388817192	9.473222667	8.841575667	8.85308204	9.456392222	8.778623323
7.792587404	7.648616949	7.183662697	8.234232515	7.682204525	7.730484909
8.156410909	8.291610121	8.131862263	8.048299333	7.951199808	8.66101001
10.61001127	10.65751844	10.75178718	11.05493438	10.78705857	10.83790729
7.568924192	7.979092162	7.901917293	8.593101434	8.130616515	8.137069859
9.307024131	9.731150899	9.416442616	8.592161788	9.056533626	9.058356636
9.641007131	10.07580535	10.27338352	11.03580591	10.79686873	10.54565144
9.86640601	9.629839465	9.495686737	9.49510903	10.33890802	9.972781707
9.188424626	8.309247939	7.071771354	8.815152444	7.326942212	7.784199556
9.976273051	8.563501616	8.498167798	9.795448596	8.773471869	8.521696414
13.20018245	13.78737352	13.74093847	13.03025597	13.38447499	12.55983323
8.370422404	8.290353162	8.246470545	9.622300838	8.542429747	9.346872071
8.674900657	9.398445515	9.348136535	8.192645727	9.438094949	8.766842081
7.840025778	8.265565879	7.990313949	7.163287778	8.039451586	7.756512818
11.26446827	11.51647662	11.52645916	11.2786408	11.46145043	11.2518391
12.04160066	12.86955734	12.39348455	11.41778198	12.42943771	12.14789109
8.011239071	8.45577798	8.478760515	9.062220818	9.103811545	8.681579566
7.299385535	7.487940131	7.715032394	7.320933798	7.241229071	6.775746758
8.379510323	8.615838	8.662363172	8.626833051	8.702074263	8.481411768
8.513135323	8.834473929	9.245445293	9.035040848	9.30949301	8.867411152
8.416400293	7.57049196	7.60282502	7.252038162	7.109400202	7.38400901
10.08521343	9.753529758	9.840616414	10.10093339	10.29208493	10.20859114
9.412044242	8.756886111	8.426330949	8.168969465	8.620285051	8.265130232
7.284105152	7.864224778	7.541750556	7.964914919	6.990730697	7.156966303
9.412634061	9.744663444	9.641787333	9.430143091	10.26224218	9.757590192
6.833358384	6.583924121	6.683063909	7.378842657	6.834332747	7.001104828
9.306330646	9.867106131	9.699589758	9.103811545	9.422377343	9.741357455
8.858692343	8.662363172	8.696739152	8.969483	8.153957263	7.958031758
9.681142253	8.967408879	9.164000111	9.368224626	8.957944414	9.569063343
7.661155364	7.458795919	7.665645141	7.961659859	8.109266465	7.462491444
9.178084667	9.340550081	9.29496	9.945028121	9.542425758	9.418269535
7.613815293	7.627373273	7.807908545	7.614146707	8.548679899	8.182386152
8.716864899	8.960218374	9.11393401	10.01257663	8.900779434	8.91530399
8.150892606	7.079103131	6.992656859	6.408336172	6.603596232	7.25994097
7.040256253	7.226681909	7.214035101	7.138031798	6.894391313	6.582190566
9.870931818	9.711723394	9.923658606	9.76471501	9.714566535	10.2692332
10.42736719	7.185402798	8.178268222	7.973992111	7.525015414	7.712213273
9.31393701	9.646274414	9.739033131	8.843148071	9.230770667	9.225515485
6.615162343	6.278635202	6.257095384	6.471690111	6.376882727	6.378965828
8.438325768	8.505797091	8.15355797	8.448929687	7.724673141	8.337114525
9.699589758	10.15783547	10.0143126	10.17702971	10.41123643	10.5029511
6.429246364	6.955441212	6.913051818	7.524403394	6.463301323	6.309659747
7.694191859	7.700334879	7.760294414	7.88791003	8.137479222	8.176360121
9.631182697	9.322592778	9.273640758	9.640429374	9.299916465	9.366909111
8.962901121	9.394567081	9.371514253	8.566871343	9.269847242	9.214111545
10.34100163	9.412634061	9.622300838	9.433849505	8.359263212	8.390560192
11.56988377	12.47130519	12.44950322	12.44950322	12.44611991	12.48835313
9.467500556	9.638947465	9.550085313	10.73911267	10.13768673	10.01071956
9.656571869	9.49850403	9.796940758	10.44195016	10.13862753	9.731806091
8.091323071	7.619556354	7.868007081	7.608041616	7.180020091	7.477877475
11.48655657	11.27499716	11.33100665	11.20840189	11.50051559	11.46320141
12.19727311	10.73014715	10.72786832	10.61261659	11.30690717	11.13879804
7.922041747	8.120448121	8.322342051	8.561321323	8.684081152	8.435296202
8.634788929	9.10558002	9.141588162	8.785177889	9.441234879	9.076382242
7.481960152	6.287983818	6.300565949	6.462162828	6.231242707	6.449700465
6.088843848	6.49328996	6.687730495	6.296516586	6.114553465	6.076308061
15.90458538	14.20688311	14.25895571	13.5160991	14.3093669	14.50017253

7.762794545	7.325732596	6.817996071	7.038648475	7.88753598	8.167383949
6.862647808	7.45998899	6.251735919	6.410100141	8.217984283	8.185943182
7.697611606	7.814474586	8.302900242	8.268510495	8.029005444	8.152087687
8.833337414	8.323157747	8.050105303	8.542859455	8.306613333	8.64507902
10.2187154	9.823678374	10.00985275	10.8405808	10.01524205	9.963504081
11.61814895	12.24649354	11.9091429	12.3613163	12.61090773	11.80554878
8.235388636	7.913459869	8.047933768	7.583998525	8.112876232	8.08537204
7.275508121	7.818322879	7.941721242	7.593349828	8.258465414	7.974325596
14.72870348	12.27773509	12.48124823	11.58421758	12.18492446	12.56315114
6.591366	6.658365848	6.808747657	7.295703091	6.757884566	6.765291919
9.509226798	9.786685869	10.71992703	10.90324033	10.2752886	9.97079498
11.28207759	10.81423156	10.7442872	12.4956701	11.65698449	11.00183361
8.070920919	6.621786404	6.741460808	6.68968302	6.608710798	6.663676879
6.015889354	6.214493687	6.354564141	6.257467596	5.802251848	6.014458293
8.268510495	8.545617303	8.765233303	9.182204323	8.921533879	8.76328402
10.11849919	9.245932475	9.623794152	8.658285303	9.206631485	9.278597747
8.430929525	9.010430586	9.031713778	8.077230061	8.469779808	8.924201657
10.48782122	10.50166798	10.71860391	10.76737368	9.794631646	10.53141254
10.05880999	10.08700287	9.929312889	10.70534521	10.12314196	10.1684149
8.21550303	7.503972212	9.082448343	8.722953263	8.576891556	7.978709162
10.09254304	10.59685822	10.77637516	10.97767761	11.29399858	10.65127749
10.72516317	10.62280049	10.60635433	11.26990549	11.36423723	10.91176188
6.919392394	6.821514788	7.265354444	6.051732566	7.057330242	7.36298202
7.033845343	6.867621424	6.828146828	6.755924879	6.27402404	6.572638727
8.849981545	9.230202616	9.658049545	9.311440606	9.562364657	9.718307192
9.131560131	9.070661273	9.370832535	8.25570701	8.818210192	9.263233333
9.158023	8.199315667	8.551311374	8.436545051	7.944058707	8.106418192
9.46304199	9.55462702	9.69725304	10.74690378	10.59180142	10.12799521
8.716387192	8.307093606	8.847386828	9.212349869	9.52752797	8.657806242
10.22865659	10.73132595	10.94942311	11.1276132	11.05033578	11.02851208
9.855298394	10.5152666	10.63014735	8.988033424	9.996420616	9.991229606
9.727935707	9.302103889	9.482360616	9.616741535	8.692870869	9.735412273
9.382882596	8.41598997	8.775519707	8.292068253	8.539776737	8.137479222
7.656877838	7.379773919	6.94614002	6.990260788	6.316133697	6.877148152
9.585510535	9.656571869	9.738398616	10.38208186	10.25127201	10.0143126
9.286739242	8.576430182	8.848410616	9.378583414	8.391020323	9.249612495
7.310197869	7.200432616	7.120482	6.974926293	6.500607838	6.636727626
9.182204323	7.608041616	8.321509909	8.436995596	7.206984404	7.996041859
11.89628941	11.94234713	12.03602449	12.12436236	11.67849095	12.03310273
15.34617167	16.33688632	16.42659037	16.33688632	16.39933956	16.2036826
5.788779394	6.648141899	7.673764162	5.807688101	5.816773424	6.755924879
9.749004051	9.613206141	9.392082222	10.25519474	9.799317404	9.455711606
13.39659876	14.77222727	14.72870348	14.1904887	14.86987024	14.49002723
8.434806414	8.513601889	8.814101707	7.654256818	8.409136758	8.616914869
13.05428976	13.56234785	13.5360606	13.60269863	13.64083669	13.29684189
7.303307	6.631383222	6.251390545	6.888392616	6.502086778	6.361157788
9.698181576	9.761765848	9.873591818	9.639623879	9.669847545	9.35188598
9.326238727	9.797856576	9.376667182	8.235056909	9.946747141	8.946107263
12.90783816	12.42943771	12.71830686	13.28559105	12.7452022	12.93862501
6.000047212	6.988974929	7.756889051	6.072186929	6.866319192	6.809240364
10.96162926	11.75462574	11.94979793	11.31267384	11.51008986	11.73710477
11.25700815	11.82616001	11.45176908	11.37490296	11.61117883	11.64044206
8.154321646	8.702574091	8.404822414	7.338325414	8.549517636	7.851263121
10.36465285	11.32376318	11.238428	11.5386618	11.55524693	11.11230451
10.4322159	11.41238356	11.64637724	10.26332497	10.73014715	10.92190902
7.273507545	7.233950838	7.30556202	7.295980475	7.182546172	7.204042263
7.528986283	7.511581475	7.396513838	8.207099091	7.847379061	7.738613697
10.13768673	10.12408141	10.19757035	9.135096212	9.629839465	9.710054737

7.766006091	8.57971698	8.218433131	7.081067566	8.556855828	8.070136677
8.484240687	9.749004051	9.101086141	9.334658071	9.072403576	9.000069889
8.667554273	10.00361533	9.945994374	9.818059192	9.702758343	9.731150899
8.420871687	9.16271003	7.529315566	6.875487455	7.339805162	7.356281616
7.406536232	6.921167192	6.825325	6.714000354	6.238117788	6.574738525
6.308316374	6.629727051	6.672081333	6.424467222	6.241562485	6.227503758
9.165786899	8.718321818	8.617420111	7.969046636	8.407442505	8.694292242
9.917863333	8.923576949	8.959130566	9.408173051	8.313601758	9.023071899
9.550769788	8.430929525	8.598008343	8.482406475	8.170129495	8.408718253
7.396513838	7.231823384	7.159147152	6.725730828	7.677935313	7.390752071
6.762314343	7.269598859	7.579015667	6.369108576	7.257203303	7.172411071
9.680349586	8.494130061	8.615838	8.880364303	8.673363869	9.146178758
12.71520416	12.96241124	13.16151113	12.94821125	12.68097339	13.32669092
8.418959303	8.600831152	8.71485401	9.117478424	9.152615889	9.014846778
9.775265525	9.453123465	9.695719162	10.84844593	9.450568111	9.879566364
8.336257222	8.599954677	8.598980253	7.985458788	8.749910828	8.599954677
7.373983485	7.332963121	7.275508121	7.791885697	7.792587404	7.392218323
8.372772768	7.275765808	7.076589626	6.739283293	6.826879909	7.158590636
6.940479424	7.575420636	8.052783737	6.645215414	7.453623293	7.263531485
8.124212202	8.352134333	8.105207222	7.479119	8.159907626	8.026271202
11.80791686	12.59668127	12.40735915	11.96059797	12.67700968	12.39660011
6.994489162	7.154661646	7.610976808	6.311055687	7.51340999	7.055055273
7.562627657	7.536179778	7.483852838	7.426803586	7.756512818	7.250545919
8.339115859	8.233421293	7.990677606	9.174793152	8.372433475	8.376704939
8.456593071	8.233006697	8.085771838	7.954205616	7.794719919	7.864535646
7.715405939	7.900011667	7.814874545	7.366481818	7.921614596	7.648319737
6.316499364	7.662925475	7.370950798	6.980492495	7.761029182	7.040256253
17.36803328	17.12376032	17.07010739	15.90458538	16.49551516	16.82803465
10.0053333	9.191849071	7.88183703	8.912452162	8.967895455	9.321890899
15.02501841	15.39581235	15.52328152	16.4534382	15.86132141	16.06827159
7.083314707	7.709908667	7.649620657	6.956363394	7.958031758	7.935468505
12.31419772	10.95374716	11.08107282	11.05356995	10.86447048	10.99186298
9.674270303	9.387059283	9.354289121	10.24612484	9.581418808	10.080472
8.271029889	8.032161838	8.074508444	8.876209859	7.842683778	8.127495596
10.74690378	10.53505365	10.52247938	10.85225864	10.89604789	10.95234717
7.019424707	7.372238061	7.32157099	6.673038182	7.585519525	6.945903495
7.631297323	8.30460302	8.079179394	7.340746384	7.986161141	7.871272152
9.35728196	9.347406172	9.325601172	9.675185677	9.739033131	9.872596081
7.635907081	7.524103909	7.778362485	7.858214495	7.909763434	7.729367111
7.784199556	8.378142071	8.878776172	8.822318	8.537956091	8.852092202
10.14044783	10.3380268	10.12126634	9.877792838	9.995698596	10.3380268
7.892277636	7.823429646	8.16147404	9.271759586	8.372086737	8.645541747
13.63435472	13.70143341	13.44849295	14.00297944	13.48990673	13.60269863
10.72384747	11.1612024	11.36423723	10.7532413	10.73788216	10.82664672
7.478150263	8.216823323	8.134366646	7.725042051	7.758192424	7.760294414
10.28089537	10.41330878	10.26445444	9.899969596	10.19957169	9.702015293
8.767368071	9.339764051	9.217871889	8.527598192	8.917069505	8.951989212
8.107270071	7.418946535	7.636533838	7.540434798	6.804242869	7.09353204
6.872611121	7.042119313	7.055909434	7.402015111	7.335878859	7.189410667
9.130293909	8.311754818	8.635209293	9.251353071	8.274284293	8.462236515
9.466909636	9.139095455	9.066605263	9.006527424	9.051957646	9.913634949
9.422377343	8.816703535	9.316319051	9.341818545	9.191849071	8.79722998
8.213079374	8.39446903	8.237787788	8.168587596	8.204025576	7.783465061
9.250125475	9.694925798	9.558925838	9.001272616	9.671170444	8.976374343
8.623636919	9.089024899	8.865915939	7.760648222	8.797743222	8.845785121
10.85654613	9.416442616	9.493894545	9.280305798	9.481000434	9.692793657
8.503920141	8.500729384	8.490053091	7.664275131	8.359650919	8.379884889
8.025864485	6.753469455	6.795847303	6.583409828	6.542971677	6.747074889

10.31013098	10.73264046	11.05796986	12.14507673	9.960976253	10.97476091
8.157542717	8.33796001	7.75371804	8.501579646	8.367422212	8.431738505
7.203112141	6.168165919	6.864210101	6.309118495	6.414229091	6.470823475
8.362142131	7.967524909	7.724369343	8.568768141	8.826393101	8.148308414
8.210186616	8.17219503	8.072110051	6.961028939	8.129874121	7.942409707
9.639623879	8.791137414	7.312858101	7.681488071	7.248451727	7.766006091
7.083013909	7.265354444	7.539809273	7.193007566	7.778362485	7.512765949
8.061425949	7.181436869	7.256041222	7.617890202	7.811684818	7.701991131
9.618256808	8.931557869	9.10215096	9.672623929	9.433849505	9.312041414
8.381754576	8.519178475	8.400464071	7.890755525	8.08075902	8.152794889
6.950648455	7.586125333	7.661155364	7.776955808	8.400903717	7.892277636
10.0406788	10.37686258	9.917043434	10.34626898	11.7349036	9.994772242
8.984231152	9.748306121	9.737663949	8.263041909	9.535633242	9.426842081
12.77454263	13.44126502	13.12023156	13.09045797	13.23341018	13.02518615
9.574050242	9.500664495	9.041820404	9.201355202	9.301561	9.79615097
6.67377398	7.153761556	6.981618121	7.364797848	6.369108576	6.740749081
6.743297485	6.732989909	6.597742293	6.247954444	6.721795586	6.550797374
8.566415323	8.274284293	8.295839707	8.546545061	8.803482152	8.440878707
6.950101798	7.324298343	7.312252182	7.520992313	6.50824402	6.71590199
12.87819387	10.51886057	10.66587479	10.0841781	10.93171462	11.71244686
6.568383192	6.819920253	6.376277596	6.184687263	7.159962485	6.322731394
7.634226727	7.255805293	7.651926253	6.054865192	7.01152302	6.908606212
7.043955899	7.117401788	7.45822897	7.880363606	7.514821949	7.450436707
7.802180768	8.017539697	8.014589606	8.511758747	8.344098929	8.055566596
7.34349297	7.593038414	7.605411525	6.916684768	7.856753939	7.383711768
7.471589222	7.459115707	7.496684051	7.122138172	8.19458801	7.559825556
11.60266156	10.88809182	11.13713447	11.73070023	11.01209098	11.60069924
10.6768734	11.29230964	11.66708917	10.57885616	11.11560115	10.87907816
9.220211768	7.711523556	7.767362636	7.85507	7.81270001	8.386133566
8.060252263	7.492282404	7.456765576	8.186741323	7.880363606	8.007640657
6.244571899	6.031446939	6.458058747	6.339864232	6.430485283	6.177173253
8.691979949	9.175472828	9.206631485	8.091725323	8.498167798	9.285000091
10.29208493	8.445297313	8.762742172	8.664725051	8.412956141	8.967895455
8.050868949	7.935875313	7.561342071	6.684542485	7.282065253	8.135177152
7.869992939	8.300888657	7.797507939	7.883917657	8.021984222	8.041000495
8.266388465	7.038073394	7.30728296	6.939486697	6.565400606	7.106848111
9.495686737	9.12042397	9.316888828	9.817379515	9.124449354	9.679062616
8.467667768	8.649462798	8.887109626	8.998420707	9.106199707	9.518503263
7.93437196	8.376704939	7.532439838	7.176396859	8.10326497	8.117339677
6.369666687	6.653328071	6.436392303	6.696224707	7.170232747	6.638128919
10.38001468	8.24438497	8.176360121	8.041792596	7.730484909	8.631899566
9.681893121	9.465659495	9.776839404	9.012760899	9.356058495	9.541041677
6.048147646	6.583688333	8.76626204	6.857008404	6.268423737	6.456232283
11.82112126	11.90674449	12.12939166	12.10951308	12.4571909	11.75215821
7.838558162	7.510907818	6.970472768	7.359873626	6.406814333	6.86986903
7.401155333	7.755503707	7.519695475	7.101319909	7.755503707	7.300031424
9.971733293	10.61743608	10.3918566	9.689783586	10.4302696	9.772250768
10.75600216	10.15186663	10.17336172	8.903025495	9.501970121	9.760900556
12.052647	13.05987966	12.79024082	12.30789527	12.58287167	12.42233701
10.43998577	11.14211434	11.1031408	11.01209098	10.99469779	10.85100254
8.574218515	9.244399414	9.207791152	9.477759242	9.538964939	9.148511889
8.477856384	7.99823603	11.18096106	12.78645268	10.93935201	8.556855828
7.679634566	7.569223354	7.810980737	8.059832677	7.642932737	7.389558374
9.521192909	9.608350808	9.933588354	10.09426568	10.10454015	9.039454747
8.878776172	8.829514889	8.875711838	7.949790515	8.924201657	8.698215303
8.50618604	8.871209182	8.957944414	7.416641556	8.587623212	8.808928141
11.26262606	11.89130603	11.34202326	10.40486524	11.15808934	11.25523353
7.269324172	7.518993111	7.835124576	7.539809273	7.589888475	7.969793182

8.226500707	8.104866828	7.982442283	7.759936788	8.123844788	8.175288444
10.37346225	10.66706732	10.55474917	8.802385697	10.30358984	10.03701736
11.88649013	12.22341713	12.41033676	12.07810634	11.80342824	12.11216258
8.610845737	9.445852485	9.484209455	8.691979949	9.198436465	9.235179424
10.58579258	10.92060078	10.60743282	10.58935103	11.18471248	11.03296099
7.537489808	8.157153081	8.066374202	8.716387192	8.630379313	8.123844788
9.911926424	10.03385507	9.855298394	10.15875628	10.2829717	9.862946677
10.9889639	11.08936677	11.18291631	11.27153548	11.11081747	11.21016953
8.303793737	8.831800202	8.435650677	8.695776131	8.35667099	9.124970051
8.836542848	8.628622212	8.322806515	8.760291222	8.725113212	8.370830747
15.46469304	16.02065153	15.72204116	15.63778652	16.42659037	15.76560628
6.468467859	6.551212667	6.523815232	7.041650374	7.168942192	6.676225182
9.390834152	9.809529798	9.841562192	8.638952889	9.899969596	9.35728196
6.784856394	5.971734434	6.066974626	6.479217141	6.727688212	6.481897646
15.16803204	16.17943014	15.97330865	15.58150095	16.02065153	15.52328152
10.14809211	11.00183361	10.91641235	11.40306525	11.05493438	11.02403513
8.724556485	7.807594768	8.137860071	8.025864485	7.760294414	7.698638758
7.865549818	8.142197384	8.090886384	7.238429162	8.003479939	8.023506091
8.203586121	7.897352323	7.918848717	8.980943495	8.352944343	8.301264212
7.603389051	7.694191859	7.899333596	8.273878929	8.375668162	8.228066545
8.553132192	9.237064687	9.182204323	7.508388697	8.73262002	9.136890505
10.59685822	9.105035414	9.538964939	9.416442616	9.235179424	9.447242061
7.814474586	7.754407434	7.535866141	7.60736902	7.535195111	7.858964434
16.8710039	16.90924948	16.90924948	17.49517975	17.07010739	17.58093558
5.931088616	5.863023434	6.207714091	6.012482273	6.007287677	6.282592313
8.338355444	7.885378	7.746135697	8.477856384	8.094605657	7.91707401
9.333986848	9.147897788	8.838951081	8.829061111	9.472030465	9.283145788
11.76101506	11.1276132	11.48258282	12.21312889	11.90152869	11.71659127
7.048729333	6.169649212	6.474509737	6.836284939	6.678083162	6.543651707
11.22600179	10.8193093	11.05191968	11.63837311	10.52799495	10.77507269
8.746311293	8.497692788	8.596127869	9.379180747	8.870056939	8.84786598
11.58814301	11.56725755	11.3733012	11.3733012	10.4655823	10.47370876
6.866597293	6.992927808	6.955646677	7.175214949	7.096340343	6.689933697
9.275532697	8.995099212	9.095762	8.414679525	8.880364303	9.230202616
8.761177727	8.940148162	8.940148162	7.98506802	8.589388657	8.642670768
8.366003535	8.525321657	8.591651626	7.660499434	8.295839707	8.225777414
9.473962505	8.516311424	9.011040293	9.174120707	8.705145061	8.919352101
13.74093847	10.93494087	11.30506756	11.32559397	11.00902763	11.87445957
8.274284293	7.616552222	7.879985051	7.989204111	6.755924879	7.711891616
8.658802051	7.97830402	7.995268899	7.814474586	7.943747505	8.097958939
10.57159251	11.11856721	11.17378617	10.50057506	10.761049	10.41022215
9.372705202	9.565032515	9.726443586	9.962571606	9.552159131	9.856174394
6.934295525	7.025021455	7.117401788	7.684267263	7.588938485	7.359873626
7.200755828	7.77328201	8.059423535	6.857999616	7.537240869	7.473428394
8.380307172	8.319524414	8.510413414	8.248566778	8.236986626	8.420387707
13.81935116	13.81935116	14.07245549	13.3718573	13.5964752	13.99495772
7.965660091	7.474688838	7.270153101	7.828260424	7.68296396	7.934739636
6.245450939	6.410100141	6.543651707	6.05895999	6.858687869	6.50518599
11.53473316	10.22361636	9.06557301	9.223302313	9.221294354	9.694213162
15.52328152	12.53563192	12.82153585	12.48124823	12.66985986	12.68484185
9.154469939	9.074692404	8.902484929	8.621272737	9.218466939	9.465071586
10.26445444	8.77298297	8.757866131	8.849981545	7.906425364	8.532516465
8.429130081	6.150983909	7.120752303	6.962034242	6.57397498	6.702042636
8.147147636	7.560760293	7.653651778	8.046784596	7.097914788	7.076304455
7.079662576	7.447087758	7.58642403	7.758961677	7.731542758	7.371573384
9.270407879	9.169326323	9.062220818	8.719668061	9.424273283	9.468772697
7.868722081	8.153957263	8.118473111	8.459227657	8.144584758	8.181196919
8.012346152	9.243096889	9.045755677	7.219623727	9.049629657	8.549970081

9.332175414	8.457489293	8.744562374	7.943747505	8.387083515	8.530767747
9.197870838	9.293710889	9.343457394	9.863774293	10.02161965	9.669114061
8.358783152	8.809442889	8.492747404	7.36203996	8.775001677	7.789255535
8.59942799	8.236633323	8.22437102	7.561051303	8.25927404	8.195405505
9.501970121	9.450568111	9.318228697	9.89348604	9.423636879	9.567720788
10.78408516	8.266842545	8.514431737	8.576891556	7.653651778	8.237365566
8.271795141	9.108564253	9.182731869	8.81966502	8.798818646	9.439917707
6.27919301	7.091067131	6.635108111	6.82246696	6.07988801	6.29449701
7.326042475	8.173308798	8.335861768	7.802836333	8.538380869	7.724369343
6.439547263	7.112620848	6.784856394	6.314369343	6.957895121	6.734478838
11.79171062	12.33576886	11.9982543	12.47130519	12.43600068	12.13244857
6.976489687	7.247912131	7.431389424	7.848409778	7.775269606	7.662925475
7.516138929	7.762451808	7.866656354	9.105035414	7.987912222	7.927460424
9.876880455	9.160413333	8.155393222	9.280885222	8.389653889	8.894491141
10.67939021	10.82290783	10.6820228	10.25220538	10.57159251	11.17213305
9.667708556	9.877792838	9.956562586	8.915814212	9.811898121	9.975431768
11.29744903	11.28896545	11.16734497	11.49636188	11.27153548	11.35657003
11.89876792	12.31419772	12.27773509	12.63888703	12.37087917	12.40343679
7.466652071	7.538502939	7.460903263	6.531968667	7.62509899	7.493863788
9.943097475	9.790672212	9.867106131	10.51886057	10.43564423	10.32309273
9.920430606	10.00186072	10.19236777	10.83930015	10.39521192	10.59418919
9.027655758	7.55945201	8.082337909	7.90869301	7.419865051	7.574489596
6.807473404	7.06161799	6.466965869	6.772514131	6.754699697	7.196057152
10.20164867	9.888393747	10.19649023	9.168324747	9.851320949	10.00361533
8.575095707	8.842639202	8.945002717	9.508442768	8.982611727	8.777050384
6.612514242	6.490432818	6.567411212	7.217577212	6.48207598	7.370950798
10.53842125	8.701614283	8.585836293	8.399553939	8.010919919	8.673843717
7.940608939	8.364284556	7.933221424	9.031155364	8.594016788	8.459596535
9.655088667	9.632768747	9.79615097	10.71992703	10.04862183	10.40278823
9.184527788	9.917863333	9.895856061	10.51174846	10.02741959	9.898158111
6.055906899	6.309118495	6.056037747	6.342479778	8.140128606	6.111258566
16.2036826	14.85646221	14.86987024	14.26676228	14.83117585	14.80749806
8.923097444	8.483763566	8.767368071	9.030619364	9.135096212	9.434425707
8.904793061	8.657372182	8.735287485	9.051957646	8.158321101	8.341133162
8.876209859	8.130993828	8.101780545	7.689906808	7.66930299	7.774617596
8.768300525	8.529417101	9.036569232	8.428176394	8.543686687	9.000672535
12.41931404	13.02518615	12.71142619	12.77015535	12.80166365	12.72610186
7.522559465	7.769155828	7.845242687	6.654520283	7.578291899	7.709908667
9.149195859	8.178268222	8.260520646	8.356302828	7.999737535	8.174069929
12.72219022	13.60269863	13.40990013	13.18348162	12.7939548	12.89886333
11.16281786	11.83330316	11.78498903	10.86691694	11.63401423	11.07337622
9.852239606	10.95093974	11.308834	10.96460614	11.30301738	10.52364746
6.025312283	5.868059303	6.260478586	5.995169737	5.930241818	5.938569596
10.37686258	10.2600862	10.21583282	10.92757694	10.76361324	10.43757507
9.229082869	9.933588354	10.33375706	10.46442497	9.86640601	9.749660394
6.77698199	7.537817576	7.525850576	7.102685081	7.570765313	7.006187899
7.410622798	7.029897788	7.094450071	7.114036293	6.399455636	6.955441212
13.90036211	14.7528858	14.45281427	14.4185289	14.6970922	14.08841453
7.195502081	6.382613596	6.483554929	7.398370889	7.071486061	6.463778677
6.265013455	6.127520364	6.733451596	7.483574061	7.217577212	6.566952202
14.48025691	15.48449671	15.32551587	15.48449671	15.449005	15.12351424
7.422224808	7.954533778	7.478803566	7.677264869	7.942803596	7.915639949
8.07563398	6.890245747	6.948610667	6.826408263	6.534648919	6.672562737
8.14677703	7.962680434	7.698638758	8.211847566	7.266262606	7.286722061
8.225261616	8.336651323	7.964547263	7.220780818	8.190353414	7.877119465
9.238221677	9.810255869	9.809529798	8.821885465	9.864614818	9.739033131
10.41426339	10.39521192	10.71503879	10.12041459	10.05970796	10.40701089
10.69931975	10.14631732	10.30470526	10.05236285	10.42175216	10.23351878

6.67667401	7.242384899	7.269892283	7.394027687	7.587331949	7.437384141
8.017539697	7.93888704	8.192645727	7.825767899	8.326162172	8.623636919
7.732206152	8.378515131	8.200961636	7.019151556	7.916025434	7.794365768
6.892837172	6.510064202	6.176869667	6.695720192	6.575452697	6.821514788
10.69099899	10.24227579	10.36781996	11.12935449	10.24612484	10.81540317
8.008022949	7.565422434	7.145769434	7.344882899	7.201015091	7.271701444
9.005433152	7.989587677	8.169730152	8.100222556	7.295094879	7.545986354
13.28559105	13.45466248	13.72760196	13.11515098	11.46539851	12.76628311
7.526167626	7.90290904	8.045722828	7.396823121	7.304125475	7.736619182
9.684048687	9.566949	9.673359929	10.02923217	10.17336172	9.949411172
8.879330566	9.354289121	8.997224192	8.362142131	8.651209293	8.501579646
9.556759333	10.70643744	10.81423156	9.588958727	10.15379546	10.07479617
5.88728801	6.189605273	6.141351889	6.304387152	6.286506818	6.340852071
10.61480751	11.45932673	11.03008027	10.20634509	10.77637516	10.88945836
7.665314131	7.939903758	7.737649848	7.877119465	7.047335818	7.183932212
7.793372141	8.214296636	7.872350636	7.206091434	8.096128192	7.759260374
6.886152545	6.370541444	6.804729515	7.107702061	6.107803293	6.16346097
7.305875394	7.549884232	7.652952818	7.487647384	7.723009	7.285287818
10.18098661	10.50677257	10.61371787	10.37026232	10.21375666	10.51400776
8.950874253	9.172421828	9.134591697	9.658772677	9.158666485	9.185002364
11.6295292	11.25898056	11.2608846	12.41033676	11.21668249	11.62739451
7.758192424	7.313451111	7.491977768	7.904381697	7.833291081	7.836508475
10.9088749	10.37215733	10.4524716	10.25811169	9.950180667	10.89094316
10.04421951	9.831087364	9.857940545	10.55070525	10.60277882	10.07107569
8.472925747	7.781852747	8.354182626	8.794788636	8.283330131	8.387507626
10.70534521	11.19638858	11.06123261	11.34732253	11.16579624	11.12613667
7.421647414	7.788923101	7.733842657	8.378142071	7.870356606	7.949400121
7.966054535	8.466446051	8.517695303	8.96575796	8.813595202	8.759757687
8.846850111	9.428190232	8.962353222	9.423026242	9.11093099	8.964642636
9.666877556	8.939019091	9.122138505	9.641787333	7.962680434	7.981345788
11.45176908	9.003357768	8.939630657	8.928828384	8.668062838	9.196132455
8.668931657	7.472500828	7.729749768	7.358375414	7.635576919	7.749943687
8.580231323	7.628336929	8.083806798	7.831503424	7.510027525	7.539167263
9.826793596	10.45150222	10.43998577	9.397189192	10.3305428	9.890135131
10.8193093	10.78705857	10.37573078	11.14211434	10.63391466	11.01045685
9.455711606	8.907915899	9.024707707	9.405539273	9.438799404	9.011546121
9.542425758	8.819236758	9.053706202	9.667708556	8.82789604	9.335891293
11.68484165	11.00620373	10.95849458	10.6240143	10.8778129	10.8193093
7.430512737	7.612909768	7.448310273	7.709201212	7.955335374	8.074508444
10.47479655	11.4413808	11.43208235	11.66189432	11.63837311	11.52052132
8.290353162	8.266388465	7.81867298	7.983913121	8.569209263	8.243637242
8.110083838	9.094076404	8.520754616	8.724556485	8.290726727	9.132080535
7.695611414	8.372772768	7.116341071	7.459405444	7.860366808	7.788532485
6.92669298	7.791533949	6.775746758	7.493863788	7.361082222	7.701352778
8.136641818	7.817975838	7.254644071	7.979092162	7.062195576	7.345589182
5.75495597	5.759479182	5.758423828	5.763122222	5.773245404	13.12023156
10.61992902	9.204855576	9.62024901	11.06123261	9.647889364	10.06255975
6.058183636	5.822681152	5.779555172	6.29940503	5.954705364	6.476450515
11.64848719	11.24147284	11.12001247	10.67305197	11.31661678	10.76737368
7.990313949	7.892604899	7.984288364	8.896578162	8.638952889	8.057421596
12.32659908	12.22629088	12.73352188	13.08418191	12.78185519	12.54906863
7.547922717	7.235668455	7.392563535	8.240375404	7.891531071	8.084674061
8.47014499	8.179120646	8.445297313	9.516398909	8.297630758	8.76626204
10.13276962	10.60507136	10.59062542	9.588410576	10.20164867	10.38631174
6.246552232	6.485516717	6.450607838	6.386235697	6.78436696	6.543222263
8.671783687	9.451212687	9.319510202	9.537497323	9.615324495	9.316319051
9.762542495	10.31480319	10.22951336	9.484840202	10.16073454	10.23066525
11.40306525	10.95533553	11.0473733	11.01376888	10.76609029	11.49420288

7.881467646	8.805485081	8.641223283	8.517288667	9.098746404	8.297224525
8.304980606	9.033394545	9.127251283	8.02698897	8.551311374	9.02886299
8.514909313	8.592161788	8.802954374	9.046362323	8.789132293	8.751405818
8.199709495	8.491490828	7.752687697	7.469344313	8.286128505	8.320817869
9.722667242	10.04776272	10.09506496	8.738735232	9.842388081	9.919491525
6.343830616	6.287809646	6.050726626	6.34603899	6.567411212	6.61996199
8.251125323	8.269818727	8.241600556	8.465492313	8.667554273	8.192266657
7.224700556	6.484666182	6.484447556	7.291703707	6.934295525	7.330801667
8.213427111	8.450171172	8.530767747	7.288128061	8.282438737	8.302900242
9.32739897	9.631972414	9.58708204	8.567801737	9.203189636	9.415217505
7.049798838	6.950101798	7.25994097	7.235967596	6.864210101	6.850100909
9.01707996	8.872352707	9.051957646	9.359783616	9.214694788	9.238221677
7.993077394	8.39867397	8.093473121	6.851905687	8.05474504	7.940254566
8.562227051	8.714332636	8.72650097	8.295009141	9.090721626	8.722452253
8.690918616	8.402148465	8.293340606	9.056062667	8.64507902	8.997807768
9.30770201	9.482360616	9.331038253	9.926784556	9.533033172	9.510536
7.682588111	8.398231626	7.533663232	8.562227051	7.948737212	8.675348646
9.221852535	9.382205394	9.054860525	7.508036465	9.143918798	8.663303283
7.304711495	7.546918172	7.767702525	8.115437899	8.150448232	7.837840162
11.02403513	9.553430788	9.318836889	9.520598525	9.321347081	9.244399414
9.736139061	10.15283227	10.2692332	10.15186663	10.56655729	10.20634509
11.58421758	10.02923217	10.54565144	10.60635433	9.499247869	10.58935103
9.235747242	9.547339828	9.574050242	8.725555596	9.846508495	9.49850403
9.383480283	9.140392293	9.356741384	8.771294657	8.83754499	9.050265121
9.302103889	8.666113404	8.555429434	9.227886646	9.067241768	8.922632727
11.14057221	11.57765358	11.74670945	11.0035084	11.79870316	11.18291631
13.68884286	13.69473258	13.62106704	13.46712151	13.7211242	13.71423947
9.799317404	9.126663535	9.408808788	9.938475404	9.600888859	9.66613299
7.350114798	7.946141838	7.748914677	7.805761556	7.935081758	7.851994586
15.13975511	13.37822002	13.39037726	12.66985986	12.89113428	13.54221797
9.337862091	9.449948384	9.503858424	9.933588354	9.813557354	9.617464455
7.403718889	6.792458242	6.710284202	6.937104768	7.451183323	7.30728296
10.78705857	11.40495249	11.5386618	11.76336308	11.82852227	10.83501466
8.269395232	8.293769586	8.327767232	8.556855828	8.464558212	8.545091384
7.202115061	7.321884212	7.324298343	7.690204818	7.750279556	7.510907818
7.36076402	7.079910848	7.458795919	7.707585606	7.684965778	7.228169333
9.21067496	9.186711646	8.920488949	9.481000434	9.150235333	9.169862626
8.006153172	8.176360121	8.186373323	8.661508737	8.077230061	8.341518747
8.34982299	9.195011616	9.145664475	9.453123465	9.540446515	9.380344141
8.647073404	8.455403657	8.257984293	8.310875455	8.204380404	7.950449798
8.694292242	8.898709273	8.837022465	9.119831202	9.127251283	9.208368677
11.36245739	8.288355808	8.385303111	8.009790253	8.069395848	8.511305758
9.490338525	9.371514253	9.385837242	8.865407737	9.066117364	8.943348051
10.77252405	10.76983035	11.09885051	10.27804811	11.70557621	11.07956266
6.478787505	6.930987343	7.03599896	7.343153889	7.391655768	7.168103949
7.030426444	7.411868313	7.352232505	6.972885929	7.334639071	7.294038515
8.344860929	8.908935182	8.276014121	9.115691495	8.820759798	8.983202444
9.166446616	9.114524374	9.346872071	9.732604394	9.933588354	9.507772687
8.833963152	8.363468485	8.364710758	8.970028374	8.620790505	8.741655828
6.769912384	6.976233182	6.782643404	7.144617717	7.514105485	6.961503899
8.198599495	8.971727333	9.290785283	9.240719434	9.119222505	8.880853535
8.559975162	7.485191121	7.380085202	7.641985	7.950135394	7.789573343
7.190930768	7.523206263	7.536878778	8.128726778	8.395296687	7.973992111
8.263464364	7.749943687	7.585815263	7.444173596	7.438305869	7.683943101
8.087781242	8.420387707	8.354947869	7.299714313	8.175968162	8.209801687
6.717503818	6.718530879	7.14635899	6.987320737	7.063222061	6.967457677
10.93646713	9.618955576	10.35699559	10.12224713	9.907546465	9.917863333
12.78645268	11.17571904	11.53473316	11.59427705	10.36465285	10.60277882

7.770819434	8.784649081	9.058895657	9.324388263	9.099925818	8.965143333
9.610538374	9.658772677	10.16642595	9.846508495	10.11760527	9.858738737
10.4450719	9.674270303	9.828404283	8.94874	9.723272263	9.634173323
8.565346707	8.039790061	7.803554182	8.18481599	7.845601525	8.166983455
7.810980737	7.662252303	7.657256192	7.802503596	6.607592929	6.925244263
7.105750636	7.371907727	7.07468796	7.044743657	6.526955566	7.386471808
6.505895828	6.140075394	6.043063202	6.395369727	6.424467222	6.060926152
7.525550222	7.843485626	7.764199131	6.781955545	7.981709394	7.81061403
6.593634414	6.596746364	6.909921869	6.912541192	7.129597273	7.149049263
8.221403505	7.364257101	7.782462626	7.621022111	8.059423535	8.290726727
9.677492576	9.517771202	9.701165495	9.90675497	9.566949	9.802496737
10.21484784	8.155023263	8.499404646	8.724013404	8.555429434	8.771856424
10.92608881	9.549314313	9.644080808	8.979298788	9.230202616	9.716648152
16.61084374	15.61926687	15.78787594	14.9727616	15.72204116	15.78787594
8.409136758	8.848914293	8.622644364	8.57790302	7.993077394	8.333227545
7.643273222	8.127495596	8.386133566	8.250622253	8.316570081	8.072924455
8.664725051	10.10187506	10.40486524	10.36239309	10.17030341	9.660200838
6.993463051	6.872611121	6.965717616	6.402881788	6.687012212	6.946968899
9.123297364	7.842052606	7.832217929	6.867621424	7.542431869	7.666980273
8.450171172	8.218433131	8.645985424	8.731005677	8.802954374	8.692466525
10.78551178	8.472925747	8.246087697	8.035675313	8.680513909	10.27804811
7.998587576	6.407444293	6.382025798	6.17279397	6.464252303	6.362324414
6.426872343	7.272016424	6.668984303	6.62508102	7.048124798	6.736375424
7.692888131	7.068665737	7.195502081	7.758641747	7.497307495	7.326942212
10.23452156	9.972781707	10.11150219	9.937540263	10.26729954	9.964343626
11.01376888	10.69099899	11.63401423	11.53473316	11.35657003	10.98037262
9.292435667	9.11154599	9.427546879	9.273022949	9.460382818	9.361074667
6.105514909	6.191799808	6.152199374	6.336448141	6.752477677	6.256334263
6.360934626	6.115636667	6.292920576	6.226273667	6.320458162	6.448115818
11.45738434	11.70138789	11.57765358	11.38391359	12.06692054	11.22119175
8.01602699	7.146069808	6.895207929	6.575452697	6.694688636	7.773582384
9.986114121	9.50710198	9.502644929	8.925229707	9.35188598	9.464440242
7.264798293	7.90505396	7.698300838	8.299695162	8.396963808	7.948408364
7.596246919	7.866656354	8.497259596	8.148682091	7.790604293	7.898595626
8.721054424	9.149195859	9.280885222	8.000144121	8.986932636	8.737323475
7.462771485	7.320252869	7.225864141	6.891253768	7.084144263	7.814874545
8.133993636	8.051625212	7.791533949	8.55725397	8.43615796	8.511758747
9.719830172	9.310205828	8.912003131	9.735412273	9.303966081	9.627013505
11.75215821	8.582147707	8.950355889	8.406604404	7.926300626	8.552208182
7.882850313	7.291703707	7.975784071	7.887186596	7.91465098	7.928481586
8.967408879	8.614861051	8.897177899	8.630379313	8.573273283	8.613521394
8.351407394	8.867918475	8.794361798	8.992473788	8.900236364	8.953653768
10.53247367	10.88302044	10.82804231	11.68948846	11.08107282	11.1612024
9.613873859	9.714566535	9.491725303	9.436898869	9.903372323	9.951175646
15.56435861	13.5964752	13.94443	13.07454735	13.56886427	14.39736901
9.392652323	9.045168101	9.332175414	9.191263899	9.79615097	9.478383576
7.320933798	7.863532101	7.097545323	7.84876403	8.127895253	7.920144808
10.46766096	10.90324033	10.89755671	9.206631485	10.71731517	10.23660558
11.94745099	11.90384839	11.98999321	11.27695531	11.54470354	12.05489479
6.410887212	6.491055869	6.494178707	6.75222999	6.005117737	6.184687263
6.709545626	6.414872495	6.794399263	6.571643687	6.743297485	7.02909003
8.807484626	9.999132495	9.489614919	9.776037091	9.507772687	9.517771202
6.292920576	6.570036263	6.543902859	6.978602455	6.910146768	6.646173444
9.078331919	8.32939098	8.311348051	8.354570556	7.572483949	7.684965778
14.1830221	13.58209631	13.66784462	13.62784329	13.39659876	13.66158872
11.40145091	11.21493335	11.42178201	11.91416468	11.43005301	11.68484165
12.42943771	12.16942168	12.21991364	12.797444	12.12151457	12.66167346
8.907915899	7.256596263	7.391655768	7.421647414	7.237499505	7.28903701

10.68572341	8.874692596	9.166446616	8.948168879	9.070661273	9.864614818
7.003715121	6.58104199	6.771393131	6.549596111	6.909148313	7.314300525
14.36070414	14.23242051	14.26676228	14.9847254	14.00297944	14.76275492
9.053043889	9.777520899	9.657223606	9.144457596	9.689129545	9.436357141
11.41071085	8.823889374	9.08959502	8.185563303	8.617420111	9.107388182
11.19966733	10.65365596	10.99623862	10.83790729	10.3918566	10.687913
7.878518051	7.962004212	7.588284717	8.452374444	8.005065495	7.414724596
11.1031408	11.85889751	11.72807534	11.21187068	11.60680648	10.95533553

GSM518919	GSM518921	GSM518923	GSM518925	GSM518927	GSM518929
6.987599808	7.460591212	6.912541192	6.990730697	6.852936051	7.050890818
9.012031232	8.647922808	9.592426051	8.888204949	9.223302313	9.600888859
6.842504626	6.421576576	7.130781323	6.496990475	6.540451323	6.989443677
8.43006898	8.471953717	8.674900657	8.430929525	8.218887434	8.349393212
7.095275111	7.123768636	7.383711768	7.422831253	7.570191354	7.21064097
8.003903253	7.771540485	7.543396525	7.675167556	7.774617596	7.737649848
8.020484273	8.182386152	8.719668061	9.272374697	9.127251283	8.425847798
7.74007802	7.734550869	7.49864798	7.789573343	7.923778798	8.189547909
10.30695028	10.22448985	9.391460545	10.61621963	9.650129091	10.29851595
8.24438497	7.88753598	8.586731747	7.842359758	8.230181798	8.468064667
7.901917293	7.724369343	8.24122704	7.054536222	8.280295667	7.374297343
8.458697343	8.33273403	8.308776677	8.039790061	8.742149222	7.352546465
7.737346818	8.028141747	7.793372141	7.988582505	7.764931778	8.217270354
8.386133566	8.598479657	7.925642	8.412578899	8.439612798	8.47107903
8.489178071	8.30620801	8.589797808	8.236217485	8.332313525	8.376150616
8.145962667	7.948048111	8.052077899	8.166983455	8.063898495	8.227704253
7.180020091	8.266842545	7.328126596	7.988251818	7.568622485	8.104133364
7.728044798	7.032840081	8.348145404	6.584387121	8.096910323	6.855182313
7.787525596	7.278115626	7.856753939	7.220240556	7.73355204	8.158602879
8.352944343	8.882882525	7.858214495	8.630379313	8.392711081	8.320357909
10.03205964	10.57885616	9.846508495	10.72384747	10.29949835	10.042425
11.19293398	10.67939021	11.55106122	10.3918566	11.18291631	11.49839615
9.205404404	9.418269535	8.942313313	9.638309758	9.379180747	9.241421747
9.453123465	9.150811232	9.591816061	9.21905796	9.323195364	9.206631485
11.96636849	12.00354228	11.37670858	11.46320141	11.60479972	11.79615897
11.90152869	12.06692054	12.02811467	12.16942168	11.89628941	11.96636849
9.444645091	9.601568657	9.245932475	9.227275152	9.480384485	9.429506515
10.21583282	9.723272263	10.27714677	9.799317404	10.27804811	9.361074667
8.697732283	9.454390232	9.062782333	8.674900657	10.34193651	8.496365737
6.179163152	5.922132222	6.513004101	5.946866202	6.217872192	6.292091273
7.839647131	8.64224801	7.488273475	8.273483707	7.261824374	7.330546788
9.76471501	9.694213162	10.03299879	8.049019253	9.959124273	10.77507269
7.85268101	7.608647091	8.265965545	7.190930768	8.152794889	8.086153313
7.411868313	7.442908828	7.710917071	7.593038414	7.711523556	7.403718889
8.253639283	8.53715797	7.918105152	8.051256737	8.221403505	8.165380303
7.249066101	6.804494667	7.413750677	6.081219061	7.035678848	7.224700556
10.19957169	10.84176532	10.12697515	10.97631922	10.93171462	11.16281786
8.16147404	8.346198293	8.024633293	8.203586121	8.242002535	8.01423197
10.61261659	11.05191968	11.05191968	10.37686258	11.12001247	10.66706732
11.11230451	12.40343679	11.33100665	12.30789527	11.81439214	11.41981305
8.198946162	8.686679798	8.058592364	8.677215515	8.319524414	8.088956818
13.49745762	13.57574016	13.60979651	13.62784329	13.60979651	13.61616406
9.474605101	9.526870273	9.366909111	9.123926859	9.417037424	8.98371896
9.106199707	8.836023374	9.173615515	8.549517636	9.151431899	8.761644081
8.134790222	7.404355202	8.153957263	7.620272192	7.775269606	6.925728848
7.834787162	7.106035242	8.278157657	7.258693172	7.828260424	8.19621002
11.15342364	11.34892675	11.25898056	11.51867768	10.96460614	11.42787377
6.11470702	6.410887212	6.104434657	6.582439566	6.067102263	6.186180192
10.11555853	9.331636121	10.87092264	9.302735434	9.399129424	8.782655051
9.321890899	8.741130788	9.804809051	8.951513343	9.340550081	9.708693071
9.35728196	9.432636424	8.668931657	9.036569232	9.092457495	9.425521616
8.47014499	8.191204879	8.745043505	7.726692222	8.561321323	8.726978374
8.173308798	7.938531869	8.53537201	8.419866586	8.422643323	8.109266465
9.080683394	9.072998758	8.563501616	8.858692343	8.914720172	9.104371838
10.8935809	11.13085117	10.41626133	10.45754412	11.16579624	10.81423156
7.261502495	7.638981707	7.084144263	7.927799515	7.484854374	7.180839586
8.735755485	8.626833051	8.991410131	9.455015404	8.99408802	9.120933788

8.108111747	7.608934747	8.639417101	7.69185196	8.181963182	8.566415323
14.85646221	15.449005	14.68633385	15.92931651	14.88238784	15.02501841
6.739283293	6.403487758	6.346259394	6.611373919	6.540929384	6.967234434
9.983540212	9.896649636	10.00886723	9.665316566	9.910960495	9.482964828
6.945903495	6.747571626	7.243973545	6.957636657	7.072618697	6.643064293
6.749564212	6.874245465	6.934295525	7.112259949	6.70156496	6.584604515
9.678297182	9.459003	9.972781707	9.789981192	9.481668929	9.602761333
6.150288283	5.820378232	6.095228879	5.803845323	6.112680475	6.59250899
8.053619525	7.946933485	8.128290707	7.958031758	8.214296636	8.143419303
9.410728505	9.217871889	9.346872071	9.087865687	9.185002364	9.334658071
6.697631071	6.349593626	6.906597848	6.454943192	6.70133698	6.575656404
11.13400706	11.11081747	10.62536143	11.02403513	10.94786936	11.20840189
7.442598384	7.74007802	7.680542242	7.68569196	7.347141657	7.799524616
7.990313949	8.118136869	7.784888444	7.882850313	7.79851096	7.712540141
13.04869312	12.55625498	13.26276274	12.43600068	13.03025597	12.64708972
8.164979273	8.175288444	8.477856384	8.157901343	8.240375404	7.993875677
10.48897537	11.16579624	10.70052069	11.671419	11.16734497	10.90324033
7.310197869	6.453428828	7.797125465	6.662952677	7.082435283	7.60282502
8.248986687	7.755503707	8.663769939	7.660150727	8.307893798	8.169382444
9.675185677	10.32635032	9.814415596	10.29949835	10.22164118	9.687048303
8.160266646	7.630934212	8.755421576	7.741148566	8.331458747	8.712341879
8.999492384	8.958493495	9.35067002	9.042346232	8.998420707	9.015412101
9.745446081	10.28795108	9.419020677	9.644080808	9.696433626	9.617464455
11.15666056	11.22947614	11.12454893	11.00183361	11.01807842	10.97054498
7.645645939	7.358688323	7.697611606	7.131581374	7.717447071	7.267131253
7.570191354	7.913871889	7.529315566	7.803554182	7.986161141	7.651926253
9.198436465	9.407554697	9.190767364	9.639623879	9.383480283	9.444057586
6.118072525	6.362894646	6.276409495	6.446595263	6.576096444	6.106166717
7.172125253	7.257203303	7.77977198	7.851263121	7.749943687	7.331138333
7.422224808	7.134964384	7.589604303	7.126269081	7.354460354	7.237838192
7.952774121	8.00874802	8.136235949	8.379510323	8.127895253	8.047553323
8.177528808	8.114611232	8.256833364	8.331888859	8.375190152	8.345781111
11.2278827	10.18291229	10.75062885	10.33696433	10.75865445	11.01376888
6.941709929	6.476638242	7.383097848	6.58891503	6.812677121	7.239071505
14.07988992	14.05855866	14.02572128	14.89443037	13.95955505	13.92172837
9.46304199	9.480384485	8.925792808	9.303342798	9.281923404	9.58708204
8.832790172	8.63568099	9.062220818	8.388817192	8.856895172	8.552208182
8.76328402	8.91530399	8.700142556	9.050797061	8.881310737	8.680513909
7.756175162	8.314030071	7.960547	8.375668162	8.40702104	8.620285051
10.48659409	9.983540212	10.95234717	9.562364657	10.70299608	10.03385507
9.641007131	9.656571869	9.355403384	9.453743545	9.439917707	9.611261414
8.583048444	8.073294879	7.595597162	8.631321253	7.642608182	8.469339576
8.669992818	8.52305899	8.226840717	8.336257222	8.301264212	8.45577798
9.09824599	8.985325394	8.962353222	8.826846768	8.558556152	9.335272182
8.008022949	7.618887111	7.869992939	7.926300626	7.567404636	7.336145273
8.183195545	8.223899788	7.954205616	8.161033919	8.179120646	8.254919778
8.606624646	8.44129198	8.554528737	8.08193004	8.417604414	7.846688253
9.146732444	8.914132333	9.581418808	9.480384485	9.469429253	8.482406475
6.440388828	6.503851576	6.289561192	6.494395354	6.357131071	6.72401001
16.42659037	15.58150095	17.12376032	15.42966281	16.4534382	16.3077943
7.40526796	7.542431869	7.271416848	7.195224778	7.416298071	7.114362495
10.47479655	10.27714677	10.12909315	10.31095185	9.984347768	10.31289083
7.183404394	6.685554869	7.148452788	7.012842909	7.561964414	6.758967323
14.94534759	15.01206542	15.13975511	15.01206542	14.90726461	15.06622261
9.541747455	9.617464455	9.838413929	9.556759333	9.254253192	9.465071586
9.20200202	8.956909848	9.376667182	8.715328101	9.280305798	9.321347081
7.963030121	7.947667091	8.301657889	7.387654737	7.799196616	7.173324505
13.45466248	13.98081649	13.27940818	14.42673578	13.50997822	13.70143341

12.32659908	12.9851923	11.87175588	12.55625498	12.43944324	12.53911948
7.74678301	6.92802298	8.040575909	6.965182525	7.419865051	8.183602061
10.97896732	11.31449746	11.17378617	12.01673563	11.308834	11.1276132
6.848028121	7.561051303	7.303019677	8.609106909	7.269598859	7.149343495
8.157901343	7.964179182	8.315227333	7.791244131	8.721523222	8.073652495
8.893968333	8.363019899	8.856895172	8.183195545	8.873520545	8.953653768
11.04156144	11.40145091	10.92337564	11.4481816	11.03737422	11.30301738
8.39227803	7.967524909	8.741130788	7.301802939	8.218433131	7.391655768
12.72972704	13.1944969	12.60022648	13.47327752	12.72972704	13.36582475
7.356848556	7.313208869	7.119314485	7.071197202	6.919392394	7.723009
11.72807534	10.98186414	11.81018832	9.960976253	11.56725755	11.04316622
9.962571606	10.03796113	10.29752835	10.50914282	10.01703805	10.01168912
9.666877556	10.0060683	9.633493677	10.21054893	9.790672212	9.30949301
7.933573889	8.016429949	7.88825004	8.214678465	8.229339455	7.647631172
8.23139899	7.758961677	8.62460002	7.596246919	8.541524636	7.701686677
6.102848495	6.021063475	6.367075323	5.944749283	5.923846051	6.036027182
8.768300525	8.210186616	8.773988747	8.304169929	8.453185455	7.37546403
15.58150095	15.76560628	16.02065153	17.01900663	15.99508933	15.83165575
6.272556111	6.537953232	6.26313998	6.311055687	6.405475687	6.297066051
8.496365737	8.468418253	8.098747556	8.587623212	8.500240172	8.370422404
7.723009	7.757532545	7.144029273	7.088019323	7.355706667	7.306206455
8.894491141	8.980444384	9.129654758	9.460382818	8.613985131	9.046862848
7.435437465	6.881514152	7.962004212	6.748847646	7.230593889	7.417927455
6.516258222	7.16240796	6.749805475	6.474928879	6.558125737	6.466266586
7.494168788	7.393489313	7.285574899	7.357775939	7.253486596	7.458795919
14.10328164	14.1282639	14.13521971	13.6823979	14.57091557	14.67552064
9.143918798	8.538919798	9.241421747	8.396125929	9.172421828	9.501970121
7.115448364	7.327857495	7.220780818	7.826151263	7.242650333	7.429567525
7.721209061	7.667962596	7.552833101	7.566450929	7.514494576	8.186373323
7.572084505	7.763846354	7.268488485	7.763504859	7.584620515	7.725703576
10.6768734	11.26990549	10.84176532	10.81423156	10.65751844	11.06573849
8.703733141	7.719466677	9.774477667	7.998587576	9.133284677	7.906425364
12.86086109	12.46797904	13.32669092	11.99535117	13.08418191	12.63888703
7.418256545	7.278422556	6.696930808	7.212383152	6.970224646	7.395300939
7.855731	7.544047919	7.404051424	7.399710364	7.29228501	7.758961677
9.53690398	9.284395657	10.05426836	9.163332636	9.336479586	9.368875727
7.70299303	7.899682051	7.770465818	8.020096283	7.877844556	7.517077263
9.370225242	10.11452638	10.25811169	10.15875628	10.18942995	9.990505182
13.15645152	13.04381444	12.56315114	12.83827393	12.92937388	12.93430142
9.234418071	8.895503556	9.269224374	9.097596465	9.077686808	9.330464051
9.215289101	10.47702836	9.218466939	10.69574272	9.94767803	9.223885747
8.64752602	9.118629636	8.871686232	8.649944576	9.108564253	8.806957455
9.994772242	9.431415586	9.658772677	10.1885747	10.19957169	9.567720788
7.946541949	7.528303323	9.250770152	9.247858869	9.283145788	8.021984222
8.684654737	8.743617505	8.867411152	9.161011869	8.698751323	8.589797808
6.933584646	7.382527051	7.248166182	7.609574333	7.220481808	7.387654737
8.531247657	8.457898556	8.53670504	8.361327455	8.498167798	8.580231323
9.034403333	9.074692404	9.067856838	9.113305192	8.673843717	8.905887879
6.943317879	6.583167424	7.016433495	6.592903869	6.684834222	6.61686503
10.66105788	10.4322159	10.73264046	10.48897537	10.4655823	10.68452674
6.918043889	6.824533111	7.404985061	6.94462002	7.203245626	6.890245747
7.272599475	7.175798869	7.883157929	7.437975364	7.612909768	6.76944904
9.526200354	8.989652131	10.4687107	9.501306556	9.775265525	9.692793657
7.141717727	7.214315212	6.777231859	7.06104997	7.652952818	7.032840081
10.38001468	10.61867663	10.25038968	10.67190595	10.39747702	10.60743282
7.85710597	8.07563398	8.212243444	9.061649657	8.546056747	7.878949444
8.275579182	7.816627253	8.60808904	7.619556354	8.369612525	8.315715768
10.27035429	10.18762619	10.38329808	10.96886286	10.24038579	10.8778129

8.78214798	8.530333535	8.854095293	8.941359636	8.464558212	8.670447949
8.723452192	8.579336081	8.53579602	8.935320828	8.751405818	8.391460737
11.58621592	11.69658017	11.78023213	12.29548604	11.66189432	11.79171062
10.6820228	12.04160066	11.91651973	12.45390404	11.00496707	11.49239347
10.29000276	10.73788216	10.43332542	11.29597305	10.16165443	9.951175646
7.266003808	7.111963455	7.868722081	7.215770273	7.640998838	7.482291222
7.771866444	8.099112939	8.186373323	8.163923192	8.34982299	8.154321646
6.271013081	6.344188131	6.27383702	6.17295696	6.666656929	6.308509485
8.925792808	9.245932475	9.176146596	8.830117929	8.52305899	8.668536687
7.074397545	7.082724758	8.135177152	7.663284545	7.736964596	6.974926293
7.654890798	7.041898303	7.536179778	7.008962394	7.923048354	6.933000202
7.541750556	7.224700556	8.048299333	7.174677505	7.969427899	7.427999919
9.099242404	8.845785121	9.385837242	8.504327141	9.026117657	8.903602263
6.876372364	7.439506505	7.635907081	8.163053768	7.935875313	8.178694051
13.14093405	13.70717091	12.81735602	14.223913	13.26910509	13.10985871
7.638981707	7.647631172	7.920494253	7.796432081	7.544367232	7.623386535
11.19114259	11.32185019	11.11388317	11.47127063	10.9660467	11.25700815
9.118059475	9.273640758	8.733447455	8.868452172	9.227886646	9.022478232
10.58935103	10.5199714	10.40387449	11.21668249	10.88166557	10.66945215
8.497259596	9.250125475	9.449948384	9.453123465	9.208914778	9.299916465
6.936097616	6.906597848	7.032840081	6.902232848	6.495899485	6.840910293
8.000144121	7.871683444	8.158968566	7.898974697	8.150892606	7.900388485
9.169862626	10.17224075	10.6768734	11.15666056	10.33890802	9.088505869
9.081927515	9.042346232	8.503061444	8.832291949	8.668062838	9.552159131
9.332795131	9.315088131	8.742149222	9.060017677	9.37540303	9.117478424
8.08737399	8.369167818	8.061425949	8.120448121	8.439112919	7.763504859
8.530333535	8.689808788	9.532335293	10.25220538	9.440510152	8.522156747
7.782462626	7.863889121	8.00874802	8.167383949	7.720541949	7.693516556
9.569063343	9.556000646	9.486974667	9.581418808	8.985325394	9.723272263
12.51766186	12.72610186	12.50328361	13.42888134	12.75396286	13.12023156
8.08112003	8.128726778	8.231756293	8.325316889	7.968691808	8.042161798
9.227886646	9.094076404	9.339764051	9.174793152	9.235179424	9.470173818
8.903602263	8.158968566	9.265583747	8.065153556	8.552719747	8.556855828
8.057864374	7.636262586	8.176360121	7.307847101	7.740855141	8.157901343
11.87687449	12.01191659	11.62059475	12.09497108	12.01427079	12.12692782
7.912742707	7.845601525	7.787525596	8.528512253	7.776955808	8.005065495
9.252443646	8.973478152	9.439397061	8.741655828	9.176817596	9.266794162
8.666601818	9.064430111	8.652865111	9.09824599	8.481411768	9.208368677
10.20452777	10.62764398	10.22668357	10.53028115	10.52585606	10.1309568
6.850608899	7.129597273	7.206373697	7.634532263	6.945089283	7.144617717
8.163475424	8.577417788	8.462637525	8.488340929	8.714332636	8.723452192
7.885378	8.137860071	7.846688253	7.862831293	7.519990596	7.448962859
10.26445444	10.23752804	9.926784556	10.35284213	10.31193963	10.08893073
9.790672212	9.487680697	9.93663897	9.529469889	9.603529667	10.13276962
10.08231743	10.05144087	10.23948175	10.19528987	10.56084718	10.6768734
7.604109192	7.505175253	7.808339242	7.795429838	7.869396606	8.210994667
7.930615646	7.97753898	8.29927897	8.427685838	8.371229848	8.091323071
9.466304111	8.818210192	9.733950273	8.465492313	9.271123182	8.762742172
7.151822525	7.097545323	6.873999848	7.121605929	7.088821283	7.032313212
7.546918172	7.596246919	7.192117626	7.314616131	7.328411747	7.436336172
11.67593314	12.06397065	12.34237091	12.41033676	12.00086001	12.20960187
14.05136004	14.27397716	13.80704919	14.30038792	13.78737352	14.11151028
14.9591327	15.16803204	14.7528858	15.39581235	14.9727616	14.82007331
12.5521346	12.58612865	12.24351077	12.96662998	12.25844332	12.96241124
8.644074768	8.752460404	8.350987111	8.586731747	8.525321657	8.740682
6.899489646	6.826879909	7.154007354	6.980492495	6.686567667	7.275508121
11.88375452	12.16279806	12.18492446	12.53197264	12.21312889	12.2293359
9.811091061	9.425521616	9.708693071	9.417594202	9.419020677	9.66831603

8.29291099	8.452374444	9.217871889	8.771856424	8.677707162	8.917069505
8.997224192	8.972363838	9.262561	9.125507586	8.937936253	9.196689051
7.648616949	7.24440804	8.049757475	7.328995606	7.537817576	7.893631939
6.56193898	6.591596333	7.094450071	7.216973434	6.859929394	6.630216838
12.28985985	11.93486375	12.78645268	12.13538265	12.02811467	8.641737071
8.675789182	8.484240687	8.009072212	9.031155364	8.572701232	8.099825778
7.969427899	8.257595869	8.078044465	7.374842788	8.458697343	7.902575141
8.070136677	8.432612263	7.949790515	8.014979636	7.110528152	8.494982343
9.47593803	8.962901121	10.36687712	8.878201818	9.31327601	9.585510535
9.084956182	8.31214998	9.453123465	8.105207222	9.028300162	8.52575703
7.776629576	7.620680879	8.271405859	8.109266465	7.651926253	8.152443121
10.94496034	10.93789932	11.31053596	11.41778198	11.05796986	11.25523353
9.938475404	10.09597734	9.892629697	10.09079019	9.495686737	9.86640601
8.157153081	8.074096707	8.71185199	8.275579182	8.280707687	8.374810828
9.417594202	9.048100778	8.980943495	9.041236566	9.441908576	8.52305899
7.859670455	7.230593889	8.169382444	6.94072599	7.504556444	7.828991111
11.20660223	11.18636156	10.8935809	11.88375452	11.77305263	10.87907816
7.945864808	8.061425949	7.565422434	7.901142576	7.812327374	8.052417212
12.49170848	12.35168631	12.49965499	11.38178745	12.12939166	11.5737214
6.583167424	6.693719727	6.34460196	6.754460545	6.468711152	6.704451293
14.06476988	14.56207529	14.16629104	14.91973608	14.20688311	14.79546777
9.058356636	8.555833323	8.407442505	8.481411768	8.994589434	8.458294828
9.052474394	8.970512081	9.528804131	8.768300525	9.209526556	8.840010495
10.573928	11.43380494	11.5386618	11.53677901	11.38775416	11.91416468
8.775519707	8.802954374	8.583996586	8.721926778	8.696280707	8.587623212
7.553468394	7.330801667	8.342773172	8.454954939	8.522156747	7.354804152
9.735412273	9.46096901	9.832711535	9.088505869	9.532335293	8.972363838
7.387654737	7.154937303	7.702676091	7.216692263	7.606990414	7.688370495
8.354570556	8.617420111	8.657806242	8.414282596	8.205892515	8.253639283
7.633913808	7.544716788	7.748914677	7.556918475	7.518993111	7.731871869
10.49592664	10.17702971	10.44934262	10.14809211	9.938475404	9.746868596
8.226840717	8.046104414	8.137860071	7.973227556	7.824090626	7.484516838
11.6157426	11.46320141	11.08936677	11.43938222	11.55322016	11.33848921
8.120795899	8.149448323	8.187136596	8.016751283	7.859670455	8.149448323
11.30690717	11.12001247	11.12935449	10.88809182	11.12151875	11.0940021
13.83772389	13.76108056	14.05855866	13.74759592	14.23242051	13.92894576
10.78269205	10.70643744	10.66342196	10.60635433	10.48782122	10.87235819
7.597232061	7.430156263	7.693213707	7.383711768	7.671246505	7.796432081
9.854472677	9.926784556	9.521972354	9.902579253	9.777520899	9.982665242
7.326634626	7.288128061	7.059356717	7.326634626	7.194433949	7.248451727
11.68038318	11.76101506	11.48459908	12.09225922	11.40495249	11.69891546
13.71423947	13.2504616	14.1904887	13.32669092	13.93694444	13.85099976
10.57996368	10.5336638	10.95683616	10.71731517	10.53842125	10.55070525
8.790654051	9.240082808	8.876650909	9.235179424	9.060017677	9.168324747
10.69470446	11.03008027	10.68960982	11.48459908	10.71031814	11.01209098
8.273061586	8.48093102	8.289996192	8.671783687	8.452737606	8.591651626
8.352134333	8.660608859	8.386133566	8.695335444	8.508645727	8.568242293
11.18636156	11.02700786	11.05651949	11.18096106	10.86305205	11.24646499
10.48185706	9.939327313	10.60635433	9.789131121	10.54441943	10.43564423
7.144617717	7.018669091	6.728691949	7.21931098	6.794399263	7.037248667
6.726910778	6.753469455	6.632573485	6.914278354	6.701799071	6.661293222
7.098999354	7.060794313	6.919663687	6.959934293	6.908074727	7.20183702
9.161011869	8.328992859	9.412634061	7.967920667	9.191849071	9.235747242
8.968910758	8.811517808	9.186711646	8.497692788	9.244930505	8.89604703
9.070117818	9.027136657	9.341818545	9.193130899	9.093001495	9.524763869
7.719835253	7.642608182	7.370383778	8.310875455	8.145962667	7.955335374
8.378515131	9.262561	8.871209182	9.206631485	9.276189121	7.384295283
7.729749768	7.495088838	7.718747404	7.692552364	8.003479939	7.496345141

7.296277414	6.64329102	7.825104404	7.093823343	7.667309091	7.889761727
6.60520404	6.823615333	7.06891401	6.971045939	6.921891778	6.715208525
15.09418019	15.54332231	15.18392271	15.72204116	15.32551587	15.50564301
7.622357354	7.292849111	7.747885899	7.270153101	7.291703707	6.707842586
6.589983273	6.683063909	6.299590657	6.221214869	6.331115141	6.220153717
8.727989172	9.109760465	8.569735879	9.586238192	8.549970081	8.283330131
7.062195576	6.728447091	7.396823121	6.417030697	7.192412434	7.398977253
11.07493153	11.62059475	11.71443529	12.38063147	11.35657003	11.52238659
7.881467646	7.416977434	8.143776848	7.168942192	7.810980737	7.917403434
9.09178804	9.096947232	9.282486081	9.299183616	9.282486081	9.205404404
9.740584727	9.452497313	10.19332507	9.385290182	9.565660949	9.979092404
9.247858869	9.161011869	9.273640758	10.31894523	9.650789909	9.214694788
10.30569114	10.60635433	10.3305428	10.94786936	10.4302696	10.3986043
7.69185196	7.863532101	7.66930299	7.813414727	7.624419899	8.022369434
7.882850313	8.416400293	7.98690202	7.875433545	7.239727717	8.390790258
7.588938485	7.783847071	7.519990596	7.548584576	7.939903758	7.333224889
15.97330865	15.92931651	15.95256028	15.88528111	15.76560628	16.06827159
9.387059283	8.963499596	8.916479111	8.984231152	8.912452162	9.370832535
7.535866141	6.578458283	6.584387121	7.610260323	6.747074889	7.282945384
8.541524636	8.659649677	9.068490586	8.846850111	8.783158111	8.879851283
6.299210717	7.291068525	6.665428859	8.023099172	7.38400901	6.977041091
8.904793061	9.647889364	8.799348343	8.810986707	9.86223396	9.189590182
7.494798263	7.480029465	7.742436303	7.633560879	7.373116051	7.606699253
13.48401144	13.92172837	13.05987966	14.27397716	13.62784329	13.32669092
7.162148556	7.304428303	7.157774636	7.577683121	7.199149121	7.205488778
9.552159131	9.542425758	9.41003803	9.517013525	9.392652323	10.18468509
12.04734694	11.86401838	12.46055504	11.94481318	12.15130062	11.94979793
9.511857455	10.25320711	9.783680727	10.25519474	9.927686616	9.874496818
8.131433848	8.998971485	8.622644364	7.670902747	7.494168788	7.116047475
8.64507902	8.924201657	9.833438364	9.849671929	9.888393747	8.453185455
9.560980909	9.705000525	9.844817354	10.12041459	9.741357455	9.750460616
7.11489098	7.866966485	8.216823323	8.964642636	8.563941778	8.635209293
7.704008545	7.266003808	7.373116051	7.608347495	7.183404394	7.45523802
16.25437967	16.22759729	16.64557528	15.58150095	16.42659037	16.4534382
10.5431016	10.02362526	10.71503879	9.844817354	10.29208493	10.13479405
8.806957455	9.119831202	9.287819051	9.280305798	8.705145061	8.949784505
9.75438504	9.481668929	9.159785394	9.12042397	9.267923343	9.062782333
11.06261544	11.70348036	10.87637444	11.64044206	11.28565422	11.2518391
9.640429374	8.796283929	10.16743096	8.840590677	9.687758929	9.230770667
11.62494396	12.45390404	11.60888892	12.63888703	12.11841559	11.85301976
12.48458958	13.02006671	11.97196754	13.13007894	12.23505088	12.60396528
8.356302828	8.309668384	8.303356141	8.15355797	8.230996848	8.120795899
7.656877838	7.196285434	7.277849899	7.116341071	7.334066545	8.267244596
6.673038182	7.028571808	6.656190667	6.909691071	6.635819404	6.695470869
6.880211293	7.040256253	7.020691646	7.365664505	6.642822768	6.981335535
7.052522778	6.520559131	7.394983566	6.496343606	6.835608667	7.262957343
11.05493438	10.81280665	10.85100254	10.15585749	10.85513517	11.21493335
9.996420616	9.435122859	8.759757687	8.940148162	9.056533626	9.446464909
7.62575301	7.611330343	7.325732596	7.651593505	7.683943101	7.579015667
7.067591	6.812677121	7.033845343	7.110844919	7.567690545	6.816280253
14.52085241	14.223913	14.40789433	14.03424604	14.15092087	13.2158469
7.01811702	7.067867747	6.589168273	7.392563535	6.620195253	6.83894704
11.06732533	11.28053313	10.83790729	11.72807534	10.97767761	11.05033578
9.729555121	9.388912091	9.459664354	9.201355202	9.485668061	9.616057222
8.710842768	8.721523222	8.76386996	8.953653768	8.769824364	8.735755485
11.32185019	11.43773109	10.67081044	10.53247367	11.48258282	11.27695531
6.884558242	7.250238485	6.875911141	7.213710414	7.33858799	7.271701444
7.515186091	8.649462798	8.406205495	7.893273434	8.723452192	8.759332636

8.854095293	8.678658293	8.453639616	8.942756889	8.871686232	8.99408802
11.64244603	11.57567047	11.93178106	11.60479972	10.9889639	11.43773109
12.78185519	11.88148631	13.37822002	12.21991364	13.30795266	12.80949032
7.916712838	7.750590293	7.674109172	7.925310818	7.373983485	7.701029869
7.360451687	7.585815263	7.541750556	7.651222556	7.809001414	7.021219939
7.083894141	7.457078101	6.834332747	7.224144889	7.237206848	6.992927808
10.58812248	10.95683616	10.58117749	10.92060078	10.58117749	10.49241362
12.99565877	12.25844332	12.9851923	12.26162026	12.85589012	12.28369472
7.379463242	7.250899535	7.911309091	7.348618394	7.410622798	7.676956101
7.622037172	7.627373273	7.396159798	7.600248455	7.773938747	7.660795616
8.316570081	8.208296687	8.60560303	8.463578343	8.318250424	8.388817192
11.29399858	10.22361636	11.58621592	10.24828231	11.91912022	10.26332497
6.15234603	6.567684242	6.656456051	6.304587333	6.170676111	6.490634808
12.77454263	11.88375452	12.09804166	10.76857843	12.48458958	12.49170848
10.54800525	10.83100199	10.2026692	10.51886057	10.81540317	10.57282784
7.454572909	7.406826051	8.521696414	8.261007747	7.94734596	8.309247939
6.796334212	6.968641576	6.56193898	6.691558677	6.593634414	6.934796758
14.03424604	14.5513462	14.010587	14.66395575	14.06476988	14.45281427
10.61992902	10.43998577	10.03796113	10.52364746	10.42175216	10.48310631
7.547786409	7.542064859	7.523479626	7.508388697	7.20666299	7.561342071
7.890755525	7.941721242	7.497307495	7.770819434	7.947667091	7.966402293
11.35657003	11.10761793	11.02851208	11.37131374	11.21187068	11.36979611
9.862946677	9.486281202	10.53737043	9.251353071	10.6426412	9.763952101
9.789981192	10.73014715	10.1752482	10.6768734	9.969958172	10.41626133
11.52645916	10.13862753	11.9982543	10.18942995	11.02700786	11.53677901
12.13867659	11.94481318	12.12436236	11.90674449	12.35168631	12.01427079
11.70557621	11.89130603	11.41778198	12.06692054	11.95261936	11.82852227
8.011239071	8.278157657	7.796085586	8.371229848	7.98285402	7.928481586
10.41846051	10.31013098	10.2761618	9.388912091	9.50650501	10.3779146
8.70424704	5.829596535	8.472435657	10.17224075	5.787806556	8.942313313
9.80784303	10.72246175	9.433258586	5.803126111	10.43332542	9.710054737
10.46442497	10.53505365	10.07956055	10.49592664	10.47820756	10.56908161
8.736738475	8.720163182	8.631899566	8.716864899	8.59655801	8.68840998
8.174069929	8.271795141	7.67197899	7.865213	8.081502051	8.214296636
9.039989364	8.707515232	8.050484323	8.06213903	8.728449242	8.279462
6.257640303	6.115970333	6.59799604	6.21252797	6.218052667	6.476638242
7.364257101	7.383711768	7.22559698	7.709511535	7.392218323	7.614831859
9.283145788	7.896600606	9.530205505	7.975784071	8.959130566	9.598893818
7.425203606	7.486746556	6.771393131	7.304125475	7.31991999	7.638274606
11.69188536	11.8164662	11.95514832	12.13867659	11.54871614	11.75908761
5.751668596	6.128153374	5.762677354	5.882450778	6.816537596	5.757688131
7.504269545	7.789881242	7.292561596	7.807594768	7.391952354	7.582311101
12.9709957	13.15645152	12.84627851	13.05987966	12.96241124	13.12562627
7.751645273	7.154318051	8.247731232	7.286117455	7.760294414	7.990313949
9.861430101	9.535633242	10.24727703	9.684886576	9.954681949	10.37467131
9.671170444	9.862946677	9.62302801	9.753529758	9.808672222	9.912769303
10.52247938	10.53842125	10.44832821	10.18468509	10.22070473	10.13671639
7.689572788	7.539809273	8.073294879	7.335878859	7.85268101	7.643628758
10.3090972	9.932690333	10.68687892	9.66831603	10.50057506	10.51400776
9.212349869	9.266188263	8.859715222	9.004398101	9.437491071	8.962353222
6.612991111	6.407245091	6.712342556	5.906592949	6.284925121	7.082435283
10.96886286	11.542568	11.05493438	11.69440164	11.35475345	11.51260306
11.16281786	11.19817469	10.9088749	10.87637444	11.20498985	11.39983314
10.47370876	9.778131475	10.37894268	9.667708556	9.969141677	10.44934262
7.904747051	8.219832939	7.751275798	8.209801687	8.326884343	7.938205586
7.950819283	8.268052495	7.490430596	7.961659859	7.716801838	8.117339677
8.979921919	8.308331848	9.523397737	8.150096222	9.025691242	9.605576293
8.286610323	8.628208788	7.739667424	8.30620801	8.226165	8.456191465

8.593558293	8.886620687	8.054318061	8.882379495	8.436995596	8.411285283
15.16803204	14.88238784	15.29549959	14.32804886	15.34617167	15.36066179
8.332313525	8.438712657	8.959130566	8.940763364	8.972363838	8.132293172
6.75222999	6.71590199	7.185402798	6.79225503	7.091328697	6.846509828
11.98440811	12.27421104	11.76101506	11.98440811	11.98440811	12.12939166
8.499836475	8.725113212	8.341133162	8.597538556	8.73262002	8.806450121
9.642500081	9.286110869	10.11760527	9.101086141	9.840616414	9.318836889
9.550085313	9.099925818	9.679062616	9.023071899	9.451851384	9.849671929
7.957660747	7.265354444	8.046433182	7.378186273	7.764609818	6.710071909
6.001983586	6.668064172	6.808747657	6.632573485	6.122852596	6.353208646
8.705608919	8.151663141	9.187808616	7.699362081	8.598008343	9.18155302
7.329290475	7.349811747	7.26571197	7.613240606	7.006478434	6.978602455
13.86604428	13.95955505	13.76108056	13.78027417	13.81935116	14.3093669
8.432612263	9.081927515	8.149788848	8.553132192	8.310875455	8.372433475
7.663954061	7.905781333	7.657256192	8.432612263	8.032499636	8.211847566
11.07647857	11.31053596	11.22947614	11.67371856	10.70643744	10.98749259
6.966460535	7.09353204	6.884060404	7.230210646	7.45028304	6.970769556
7.747885899	7.728044798	8.086566798	7.452458778	8.007258162	7.819446808
9.144457596	9.324388263	8.870602515	9.044046343	9.084401768	9.281446566
12.01427079	10.93023415	11.99535117	11.05356995	11.26815786	9.642500081
14.4185289	14.08841453	14.57091557	13.47832004	14.53168901	14.65397593
8.904239657	8.630379313	9.223885747	8.769354545	8.836023374	9.082448343
14.11151028	13.44849295	14.15863941	12.797444	13.69473258	13.66784462
11.60479972	12.09225922	12.0750029	11.10761793	12.40343679	11.28565422
6.576096444	7.05481101	6.693987121	6.928862212	6.594827192	6.632101727
6.602427343	6.533739475	6.855940818	6.563630475	6.739060495	6.732033313
8.773988747	8.417604414	7.790916071	7.966742939	8.155393222	8.072924455
9.715215101	9.681893121	9.867106131	9.951175646	9.69725304	9.62302801
5.821766576	6.135589202	5.926867677	6.147877354	6.670140313	5.759604707
7.614831859	8.126700889	7.672658859	7.667962596	7.74753198	7.850180343
11.36065784	11.15503783	11.31449746	10.96311364	11.17910752	11.20163629
7.522559465	7.057634667	6.873794323	6.770142061	7.246435697	6.853178192
7.507667889	7.393766606	8.150892606	7.279669313	8.226500707	6.815206394
6.470600061	6.494595232	7.637927172	6.960463354	6.669677444	6.738515899
9.123297364	9.149684939	9.132080535	9.247274333	9.114524374	9.512482222
9.244399414	9.607593848	9.663106646	9.999132495	9.492465394	9.440510152
9.366312899	9.382882596	8.947127939	9.026117657	9.303966081	9.050797061
12.43600068	11.58421758	12.54589185	11.18941159	12.44950322	12.40735915
8.836023374	8.662816202	8.387083515	8.415109929	8.755916071	8.630379313
10.16546821	9.448606737	10.94786936	9.284395657	9.978126798	10.76983035
6.461249374	6.982654747	6.994029394	7.176396859	7.633913808	6.440833869
9.601568657	9.235747242	9.227886646	8.765765323	9.438799404	9.211846273
6.337620818	7.116869414	7.34349297	6.248152273	6.042269061	7.013396273
9.967178364	10.09994749	10.17224075	10.11664425	10.16073454	10.44602022
10.57649171	9.562994313	9.875361242	9.702758343	9.858738737	10.32531651
16.28343863	16.33688632	16.36806566	16.25437967	16.28343863	15.90458538
9.428190232	9.203189636	10.16642595	8.949784505	9.746868596	9.210065
11.68948846	11.1612024	11.75462574	10.06173934	11.4413808	11.71443529
7.84419497	7.91307104	7.540776061	7.90505396	7.850180343	7.865549818
11.55524693	11.28565422	11.52448673	11.34732253	11.33292491	11.31852116
7.225301495	7.417598061	7.194952889	7.815211869	7.258429848	7.19832496
7.554702939	7.70365599	7.361380131	7.697220202	7.607714535	7.227000899
6.188826879	6.191297606	6.32497801	6.478141798	6.027354717	6.213218293
11.52238659	11.30131952	11.36979611	10.53505365	10.43564423	11.28896545
8.527598192	7.860366808	9.272374697	7.900388485	8.597538556	9.139095455
8.903025495	8.679578434	9.071813909	8.380307172	8.721926778	8.207537162
10.94942311	10.28005063	11.10035391	10.31193963	10.73132595	10.1905069
8.036816444	8.696280707	8.77962597	7.514494576	9.193817232	7.622357354

10.3548828	10.31378621	10.63870636	10.41731261	10.27236099	10.54950911
8.431326525	8.219387636	8.765765323	7.890057202	8.466026899	7.933221424
8.805485081	9.846508495	9.27419499	9.749660394	9.236429667	8.91530399
6.87893297	7.485802727	6.682537212	6.933276949	6.783830859	6.689933697
10.54078574	9.969141677	10.687913	9.839111	10.40387449	10.41224321
7.977952253	7.873080636	8.175968162	8.186373323	7.735250384	8.192996515
6.785592192	6.579152939	6.409900091	7.14635899	6.475338556	6.444123263
5.995948626	6.066060394	6.042010263	6.623921737	6.078299758	6.222355778
10.77762269	10.66229262	10.54441943	10.57996368	10.92485902	10.62764398
7.777661384	7.984288364	7.540434798	8.643157737	7.826151263	7.896287434
8.344098929	7.353560545	8.978204141	7.422534091	7.963371182	8.863375313
7.204042263	7.154661646	6.968393455	7.134684455	7.061925202	7.162148556
9.438094949	9.024212859	9.35128199	8.669992818	9.134591697	8.794788636
8.824851909	7.82717702	9.21905796	7.764199131	9.146178758	8.050105303
11.86652814	12.30465105	11.47682743	11.85889751	11.7894918	11.71042621
8.29927897	8.560860505	7.595878485	7.491357222	8.083806798	8.344098929
11.4090051	10.16642595	11.47127063	9.822898081	10.81280665	9.823678374
9.957374697	9.969958172	9.707282667	9.867911152	9.848945263	10.10187506
7.595301182	7.294284717	8.155023263	7.368846081	8.105207222	7.743466333
9.360502949	9.484209455	9.678297182	10.04511943	9.647889364	9.663106646
6.735859162	7.130479657	6.726480707	7.962680434	6.610905657	7.531209667
6.813169384	6.572402919	6.792041707	7.062669222	6.604297121	6.558838808
10.22865659	10.18468509	10.70425349	9.390110384	10.40074116	10.02362526
8.815152444	8.723452192	8.807484626	8.928828384	8.727528626	8.864430475
11.72592113	11.98999321	11.89361272	12.48124823	11.67849095	11.86125412
9.083741606	9.071276071	8.856343687	8.908935182	8.929384727	9.066117364
7.048729333	7.189144101	7.125696242	7.482291222	7.098999354	7.54731098
9.274850788	9.146178758	9.206093556	9.400967465	9.055478465	9.30577901
9.544495798	9.553430788	9.151431899	9.717365758	9.564343333	9.625623061
6.908354899	6.514109778	7.204326061	6.637000293	6.799149535	7.01152302
7.525550222	7.685352364	7.625433667	7.510907818	7.574140061	7.119081899
7.661901192	7.750279556	7.641985	7.429567525	7.950449798	7.652952818
8.667554273	8.796713535	8.379884889	8.820759798	9.037153444	8.463578343
12.53911948	11.67371856	12.48835313	11.23280633	12.28693608	11.87175588
10.48782122	10.15783547	10.60863639	9.511857455	10.58236152	10.72384747
7.61856696	7.623386535	7.941721242	7.97284099	8.185943182	7.137147091
13.35359128	13.14093405	13.70143341	13.06488572	13.68884286	13.0147344
11.8164662	12.19996238	11.68038318	12.10694646	11.70812672	11.88909817
10.77637516	11.27499716	10.57282784	11.41415047	10.97631922	10.39968902
6.561505162	6.515007141	7.157253707	6.189914495	6.76700904	6.882244081
8.262596626	7.803554182	8.733083212	7.562928222	8.514431737	8.799825313
7.059062808	7.04545701	7.455517596	7.634226727	7.112898869	8.102124455
9.189045596	8.672348475	9.340550081	8.265565879	9.388273121	8.951989212
6.092696515	6.013541848	5.773471525	6.227319707	6.413017273	5.897740091
10.43564423	10.40178901	10.33476777	10.17122401	10.31095185	10.5104744
8.289212586	7.45028304	7.894315778	7.377557566	7.593664717	7.508036465
15.86132141	15.13975511	14.90726461	14.28325373	15.67809237	16.49551516
9.135618929	9.598265626	10.10278369	9.990505182	10.44392641	9.439397061
8.767872848	7.451183323	9.453743545	7.608041616	8.634342798	9.06333597
8.392711081	8.669446475	8.554971	8.60413102	8.425847798	9.004940778
9.439917707	8.830660505	9.609756293	8.876209859	9.226702434	8.956407626
9.469429253	9.053043889	9.715215101	8.291166909	9.518503263	9.497084828
8.708995576	8.718321818	8.130616515	8.59655801	8.584413131	8.59942799
8.830660505	8.841037687	9.196132455	9.010430586	8.825312061	8.892970697
8.682101313	8.554528737	8.977558859	7.973992111	8.559456798	8.335469222
7.952029848	8.293769586	7.752687697	8.350987111	7.97753898	8.321152475
7.518430525	7.593038414	7.153217667	7.453623293	7.429246232	7.575420636
6.780968242	6.827921778	6.690165707	7.023140616	6.892562737	6.84402404

8.726978374	8.760676242	8.667554273	8.607626293	8.631321253	9.063885091
8.76626204	8.448929687	8.532980343	8.910531141	8.298004222	8.03490996
8.889380424	8.738735232	8.251922909	8.629474212	8.296772869	8.872352707
8.962901121	9.054860525	8.546545061	8.708467242	9.019287687	8.870056939
10.17030341	9.609027141	9.584100455	9.256767414	9.710962273	9.792275424
8.818210192	9.069558111	8.812517081	9.883591758	9.007843273	8.816214848
8.656835556	8.50673198	9.737663949	8.745901071	9.476588061	8.65914896
8.72650097	7.535195111	8.384333949	7.288727879	8.758871	6.635568162
9.060017677	6.614259212	6.437269283	6.939947626	6.624843121	6.567922788
10.46664928	9.922126404	10.58236152	9.627745303	10.35917202	10.4302696
8.876209859	8.967408879	9.278597747	8.870602515	8.344860929	8.32400903
8.784105778	8.755421576	8.564366949	8.989652131	8.979921919	8.718321818
9.956562586	10.07956055	10.23850383	10.42297323	10.12126634	10.2829717
8.309247939	8.024267525	8.752912545	8.784649081	8.321919424	8.631321253
5.79008703	5.781277556	5.795853727	5.791383172	5.811704121	5.808613101
11.25700815	11.02403513	10.9991534	10.87518762	10.97476091	11.16423752
10.20964764	10.94353341	10.47941256	12.00086001	10.99345288	10.3810842
10.66832974	10.43564423	10.30569114	10.33252085	10.56421743	10.5152666
11.76101506	10.69682519	11.68700199	10.80091358	12.87819387	10.22361636
10.52476206	10.38001468	10.02821235	10.8605042	10.65634987	10.36465285
7.069165141	7.073400869	6.808010384	7.032569455	6.95496698	6.855692535
7.742436303	7.055909434	8.252372808	6.858224505	7.586712596	7.969427899
8.32400903	7.867668606	7.148192263	7.891143768	7.319616859	7.517436172
11.15183093	11.12454893	10.72384747	11.15183093	10.98037262	11.11856721
9.669847545	9.491725303	6.883542434	8.644542606	9.499247869	8.902484929
8.31049196	8.366948404	7.875808798	8.223899788	7.924502232	9.024707707
9.532335293	9.466304111	9.042346232	9.396536798	9.225515485	9.57872197
10.52689599	10.19236777	10.50543333	10.1421691	10.61992902	10.31095185
6.29783297	6.283168747	6.601717263	6.161098354	6.270309222	6.34126302
8.549970081	8.746311293	8.824381182	8.112525414	9.013830768	7.74753198
9.382205394	8.943861384	9.689783586	9.051957646	9.623794152	9.404880263
11.94979793	11.61814895	11.43565115	11.71244686	11.53473316	12.24097391
11.83813795	11.62290433	11.78265022	12.01427079	12.0724233	12.18142186
6.110155364	6.182782414	6.165805071	6.17195304	6.548447101	6.218725646
8.701109788	8.84935699	8.336651323	8.667554273	8.546545061	8.907915899
5.774169051	6.363322586	5.774247364	6.128153374	6.47300398	6.437856202
8.724556485	8.589388657	8.239474646	8.385691051	8.79722998	8.541524636
6.363691192	6.226461556	6.145507687	6.339077535	6.121456293	6.279712293
8.254484434	8.690381253	7.944058707	8.577417788	8.349393212	8.084275677
9.042346232	9.004398101	9.150811232	8.930433505	9.110297333	9.272374697
8.355387535	8.45110203	8.326518101	8.491898242	8.251125323	8.50673198
11.40145091	10.61743608	11.69188536	9.406893465	10.91176188	10.17440526
12.52461444	12.77454263	12.22341713	12.54589185	12.51424883	12.85048632
8.83948901	8.911109919	8.728878424	9.576740677	9.791460222	8.995584808
6.800063838	6.574452879	6.286294667	6.562194212	6.387713778	7.039183869
7.499261545	6.811363364	7.817975838	6.651524121	6.941216071	6.874493707
7.438305869	7.689234404	7.523206263	7.854005263	7.461834424	7.485478828
7.849463182	7.511581475	8.20265299	7.545335788	7.803554182	8.242002535
8.817758687	9.143918798	9.447991384	9.896649636	8.832790172	9.257324
8.696739152	8.50473503	8.963499596	8.640338566	8.709941838	8.473392556
7.240898899	6.630696545	7.746135697	6.734989303	7.04545701	7.373983485
8.687541707	8.39867397	8.805907646	9.369545939	8.578393657	8.745043505
9.384699788	9.300983919	9.354902374	9.262561	9.588410576	9.396536798
8.358418384	8.364284556	8.679110091	8.191204879	8.62991701	8.420871687
9.000672535	9.251906182	9.432636424	9.602112616	8.975268424	9.045755677
13.17778072	12.7494647	13.41514643	11.84736958	13.33155169	13.54965001
7.156105475	7.426469556	7.444823313	7.106848111	6.95442902	5.811428889
7.577325354	7.406158394	7.770819434	7.562627657	7.811371919	7.126269081

7.669657737	7.251769333	8.296772869	7.25519196	7.828991111	7.94734596
14.62217632	14.79546777	15.01206542	14.86987024	14.94534759	13.40990013
7.845242687	7.435155859	8.017912586	7.25118401	8.03601997	9.337862091
7.44112097	7.322217273	7.325469747	7.435155859	7.419275485	7.139090576
12.81379045	12.20640951	12.93862501	12.25844332	12.59668127	12.0838996
7.445746576	7.708254091	7.622037172	7.509397909	7.45822897	7.491977768
9.434425707	9.037616626	8.78664797	8.680513909	9.035040848	8.662816202
11.59870577	11.30690717	12.11841559	10.61992902	12.32964397	11.12001247
6.991264788	6.853178192	7.110528152	6.637667434	7.326350616	6.778194636
8.975268424	8.951513343	9.105035414	9.140392293	8.913588253	8.787164939
7.840025778	8.002011657	7.890057202	8.061012707	7.601973414	7.882850313
8.428176394	8.732044667	8.430534091	8.937936253	8.456191465	8.968391182
8.41335802	7.969793182	7.942803596	7.870356606	7.675167556	7.757234778
11.98999321	11.47322198	12.52130815	10.82804231	11.96947069	12.3843369
6.026528364	6.140691525	5.829475475	6.064326273	6.180794455	6.015743101
8.498949848	8.401710747	8.911806707	8.288355808	8.562227051	7.739667424
14.78379092	13.83173935	14.89443037	13.61616406	14.37933382	13.03957854
6.898276768	6.546440545	7.966054535	6.938620788	7.026870727	6.833099889
12.14191765	11.78265022	11.67593314	11.75908761	11.57765358	11.911688
7.28903701	7.071197202	7.369510384	7.127674667	6.626873192	7.023397576
7.328126596	7.177458596	7.195502081	7.092334828	7.21466303	7.386471808
9.395854121	9.002847848	9.626304798	8.856895172	9.503858424	9.447242061
6.994245051	6.517425313	7.21466303	6.397207283	6.956131545	7.416977434
7.130177747	7.696527091	8.226165	9.162016475	7.076304455	8.433468657
6.607353172	6.431354778	7.230593889	6.373918293	6.563853182	7.077389495
8.230996848	7.988582505	7.989204111	7.914251515	8.124973859	8.333749495
9.966143818	9.113305192	10.21769501	9.315671101	9.824540899	9.493200828
7.649926505	7.90609097	7.805446323	8.095746162	7.681167667	7.547922717
8.690918616	8.69919198	8.891390919	8.927565404	8.198599495	8.823394192
7.802503596	8.230996848	8.405355091	8.066736566	8.204380404	7.949790515
9.929312889	9.254253192	10.39072525	8.708995576	9.466304111	10.40387449
10.58347296	10.89224764	10.95374716	11.06123261	11.03580591	10.9102587
6.861656576	6.501046121	7.181436869	6.271799455	6.29940503	7.07011003
6.918565768	7.070939121	7.276635293	7.292561596	6.351647616	7.079103131
8.531655515	8.804477505	8.990220404	10.10365156	8.829514889	9.189045596
10.31693391	10.86447048	8.77085696	10.63516273	11.00183361	9.651465606
9.109120374	9.198999222	9.766131535	9.811091061	9.384699788	8.865407737
10.35069541	10.27035429	11.2345748	10.5104744	9.990505182	10.80239406
9.823678374	9.894213939	9.965319232	10.57028524	9.762542495	10.0728998
12.01956714	11.93178106	12.30789527	12.33576886	12.08128174	12.32964397
12.54906863	12.69316533	12.40735915	12.50706636	12.78185519	12.58612865
6.898002222	5.827664404	7.206091434	6.049314636	6.238699505	6.023530485
10.27905981	10.20859114	10.69099899	10.17789322	10.29752835	10.17702971
9.76546198	10.1905069	9.834210131	10.3896003	9.997192202	9.968215758
8.863920758	8.965143333	8.527598192	9.515069697	9.136890505	8.671783687
8.088528121	7.936644384	8.046104414	7.924885333	8.137069859	8.146343545
9.732604394	9.960013465	9.895856061	10.48782122	9.828404283	9.958247111
9.558255162	10.24511785	10.01257663	10.32208546	10.01071956	9.638309758
8.264694495	8.44047504	8.065602475	8.962901121	8.907915899	8.643636162
9.84311402	9.62024901	10.00450786	9.662314131	9.343457394	9.969958172
8.890373556	8.877161475	10.37026232	9.583475303	10.11150219	9.694925798
11.78265022	11.80104424	11.95261936	11.65261158	11.82616001	11.56725755
13.6823979	14.19920898	13.73439002	14.47046648	13.83173935	13.60979651
10.76983035	11.16888788	10.6362571	11.43208235	10.87235819	10.30569114
8.987499273	9.395854121	8.389653889	8.93587504	9.079484556	8.653906556
11.08264222	10.72900488	11.51867768	10.55474917	11.5571076	9.890135131
7.247912131	7.212654808	7.378842657	6.93243001	7.237838192	8.001277303
10.82804231	11.34381562	11.28207759	12.06964699	10.31894523	11.17041501

7.757833798	7.18283101	8.115437899	6.799149535	7.556302505	7.815211869
6.343092465	6.308316374	6.781710556	6.582190566	6.629250202	6.563407434
7.251486172	7.571427586	7.347141657	7.468171747	7.272331859	7.224435778
6.217165485	7.446760616	6.834065141	7.815532475	7.581982414	7.434843313
8.520243879	8.438325768	8.753420071	8.471488636	8.474228909	8.499404646
7.992269263	7.925642	8.275579182	8.35796399	8.096128192	8.012698303
7.192707293	7.317897131	7.812327374	7.576363505	7.816902374	7.554702939
8.872352707	8.710391727	8.311348051	8.554027364	8.595703707	8.848410616
7.837507091	7.805761556	8.102862354	7.803193929	8.234636465	8.093860848
7.418614091	7.338887687	7.29687102	7.206373697	7.490707535	7.376443525
9.528804131	9.430738808	8.790654051	9.248396293	9.133918929	9.36748
8.412578899	8.213427111	8.555833323	8.594861495	8.644074768	8.35796399
9.077100232	8.988033424	8.594016788	8.686679798	8.795325869	9.123926859
6.606438778	6.456009747	6.531968667	6.342479778	6.404128626	6.502086778
8.783584889	8.948168879	8.717364515	8.688838939	8.776574232	8.899757485
8.20265299	8.000883182	8.327767232	7.640655485	8.31912796	8.185177828
8.974650333	8.785177889	9.064430111	8.810446485	8.998971485	9.468772697
8.626833051	8.306613333	8.472925747	8.94874	8.272620253	8.85785699
5.809183828	5.781179475	5.832104141	5.867810081	5.767607182	5.759872354
8.456191465	8.339926909	8.836542848	8.782655051	8.371674091	9.007843273
9.030004576	8.823394192	8.539328929	8.814101707	8.964642636	8.986932636
6.626438091	6.484447556	6.807214747	6.323943747	6.412381	6.523815232
8.188379657	8.147857556	7.611680859	7.534302667	7.666604434	8.08112003
6.683324051	7.539167263	7.286424495	7.20183702	8.361756343	7.209785606
7.851263121	7.257525758	7.518430525	7.920494253	7.148452788	7.742113232
7.12824003	7.02612702	6.957636657	8.128726778	6.988399394	6.85895202
8.740682	8.738283333	8.791137414	8.57597697	8.625486818	8.731576192
9.533596364	10.41426339	9.402230293	9.394567081	9.554043101	11.74439188
6.204530263	6.094493333	6.236816515	6.002532	7.690510313	6.500186222
8.553132192	8.681067818	8.181196919	8.433042727	8.621272737	8.754900343
9.538263677	9.565660949	9.363823202	9.606298465	9.497084828	9.62024901
6.174477505	6.902232848	6.665659576	6.787317758	6.731175838	7.159962485
8.896578162	8.687053253	8.965143333	7.835788293	8.947623838	9.36439596
7.250545919	8.104133364	7.601973414	8.647922808	7.597232061	7.833986465
6.419720182	6.185516475	6.770142061	6.304241131	6.503631525	6.571898091
9.840616414	9.94767803	9.508442768	9.75438504	9.826793596	10.13768673
9.136296333	9.320738646	9.515743253	9.203189636	9.219562515	9.166446616
8.340681505	8.063461202	8.490053091	8.403471909	8.028563859	8.492747404
11.27499716	11.16734497	11.20498985	11.21016953	10.687913	11.308834
8.041792596	8.123471404	8.227270172	7.750590293	8.171788646	7.591410606
8.85518403	8.348543172	9.209526556	8.383429707	8.451947071	8.415109929
6.57823699	6.957403667	6.938883283	6.860390606	8.262181081	6.912025515
6.574738525	6.61686503	6.869142343	6.511476192	6.733937384	7.187484444
6.272928495	6.581253303	6.825072616	7.151822525	5.903336	7.233650202
11.33100665	11.08435313	11.46936418	10.16454544	11.11560115	11.28207759
7.622739535	8.473392556	7.451183323	7.494798263	7.564402343	7.353900202
6.513435323	7.13105099	6.526325283	6.483130091	6.366826091	6.437041
9.874496818	10.32093584	10.02549055	9.958247111	10.42927797	9.267383556
11.44319437	11.36423723	11.71895032	10.76983035	11.6157426	10.81280665
11.46145043	11.45738434	11.36065784	11.82616001	11.07797425	11.60479972
8.869521111	9.044608384	9.128533354	9.081927515	8.773471869	8.499836475
13.12023156	13.34730616	13.2504616	13.13522491	13.70717091	12.797444
7.644959949	7.955335374	7.291982576	7.825767899	7.595878485	7.805761556
7.257820879	7.153217667	7.205488778	7.23119303	6.935043828	7.462491444
7.539809273	7.877119465	7.743466333	8.215082485	7.584276586	7.840025778
7.041898303	7.075769859	7.050073121	7.411868313	7.007889061	7.263217283
9.016491202	9.35188598	8.888766747	9.616057222	9.239472869	9.135096212
8.030181424	8.684654737	8.86024204	8.898709273	8.052783737	8.705145061

8.658285303	8.144908747	9.316888828	8.252794354	8.869521111	8.281588828
8.302900242	8.455403657	8.356302828	8.634342798	8.298396535	8.312729141
7.747112485	8.496814303	8.012346152	8.161911788	8.333227545	8.149788848
9.326739394	9.857104919	9.981718727	9.445852485	10.30569114	9.348735909
6.97994296	7.470886646	6.284925121	6.892302889	7.198026636	7.045715273
13.24400831	13.07947925	12.66985986	13.14093405	12.66627862	13.57574016
10.8947391	10.70425349	10.68572341	10.52247938	10.70902317	10.53623255
10.77890896	10.95374716	11.47871073	11.20840189	10.56655729	10.58689262
6.448856899	6.62508102	7.123473687	6.888392616	6.242654081	6.501046121
6.794655232	6.718779485	6.200189737	6.735607747	6.671548495	6.821005303
9.571117879	9.980879424	10.07857135	10.30151978	9.80784303	9.892629697
6.559271747	6.301539283	6.238895919	7.680542242	6.108765172	6.212345727
10.12799521	11.12151875	10.19528987	10.18666124	11.21958266	10.31597323
7.374297343	7.098723051	7.251769333	7.506719576	7.337670899	7.500167182
7.555972677	7.277849899	7.430512737	7.269064929	6.996297444	7.29078701
12.76628311	13.07454735	12.61449239	12.80949032	13.20536123	13.04869312
6.727165222	7.063477202	6.880775747	6.729176758	7.376746737	6.544818747
7.560401535	8.39402401	8.191573747	8.180785838	7.677613273	8.406205495
9.926784556	9.507772687	10.02161965	9.530933293	9.773700283	10.03123363
10.67815746	10.35069541	9.86640601	9.853021111	10.32093584	10.30358984
7.726692222	7.158590636	8.347358778	7.518430525	7.851640596	8.207099091
11.74439188	11.51008986	12.01956714	11.33477589	12.03602449	12.10414281
6.268823394	6.536865101	6.207516667	6.587802667	6.276975525	6.016143081
9.449948384	9.383480283	8.597027768	8.829061111	9.211184727	9.621550919
11.08936677	11.37490296	11.22294817	11.19817469	10.68452674	11.26815786
6.835859525	5.91840196	6.271221162	6.745348657	7.140588444	5.900311293
8.522156747	7.957660747	8.83754499	7.963792818	8.283707475	8.450171172
9.193130899	9.379720636	9.479751879	9.803356646	9.121511687	9.136890505
8.040575909	8.203151141	8.303793737	8.17298499	8.513135323	8.282438737
9.011040293	9.691258919	9.35371997	9.16271003	8.734806657	9.226128374
9.523397737	9.718307192	9.762542495	9.856174394	9.886023253	9.604175636
10.08521343	9.821914929	9.673359929	9.732604394	9.804809051	9.922126404
10.2861211	10.09795649	9.917043434	10.23452156	10.41022215	10.68960982
14.88238784	14.1904887	15.08134613	14.05136004	14.63205333	13.77352346
8.508645727	8.298896434	8.230996848	8.437898414	8.476485121	8.593101434
8.260123323	7.79851096	7.762794545	7.753435232	7.666980273	8.174069929
14.14208591	13.9071606	13.98831971	13.33661988	13.64083669	14.10328164
7.579350515	7.896287434	7.660150727	8.305787747	8.111336758	8.006495333
11.3733012	10.80782516	11.15503783	10.52476206	10.86566339	11.51647662
9.535633242	9.062782333	10.36032821	9.985290333	9.604175636	9.503858424
8.130616515	8.443175586	7.767362636	8.254082232	8.685201879	7.855388333
6.20939903	7.351625758	6.460273586	7.317304515	7.486088616	7.915639949
6.858444758	7.379145515	6.939486697	7.076304455	7.312858101	6.917518081
9.293068828	9.929312889	9.616741535	9.436357141	9.490338525	9.556000646
15.15443825	15.15443825	15.54332231	14.53168901	15.39581235	15.31193978
7.896287434	8.013857071	8.08193004	7.601304687	8.265565879	7.892277636
9.503858424	9.472607081	9.457741556	9.426842081	9.664626495	9.479084687
10.25127201	10.14044783	10.15783547	10.43440935	9.807061475	10.50677257
7.979449616	8.006495333	7.435155859	8.037287293	7.951199808	7.611975737
6.987890152	7.293748525	6.881752616	7.02909003	7.75371804	7.531770848
9.867911152	9.907546465	9.863774293	10.31795252	10.6313464	10.38531322
8.851018515	8.08193004	8.825911939	8.69335002	8.683087172	8.990220404
8.503920141	8.454954939	8.798247798	8.33796001	8.314850485	7.819838162
8.517695303	9.165786899	8.197369283	9.652169778	8.529417101	9.059459505
8.385691051	8.221403505	7.766738848	8.075284384	8.278157657	8.350987111
12.44950322	12.54589185	12.3774088	13.46712151	12.51066587	12.38794592
10.24828231	10.2761618	10.17702971	9.857940545	10.042425	9.921211657
9.394567081	9.660200838	9.408808788	9.84311402	9.658772677	9.62845904

9.07419996	8.750392838	9.145664475	8.729485838	8.885078273	8.82842597
7.985767212	7.082160636	8.577417788	7.206984404	7.912365798	8.090157434
9.839111	9.35067002	10.0188097	9.066117364	9.606954152	10.02644328
10.11664425	10.28506122	10.0079343	10.30810853	10.17336172	10.25906505
8.311754818	8.35667099	8.251125323	8.494576061	8.592161788	7.539487212
12.60396528	12.12692782	13.49745762	11.77053658	12.19398134	12.83827393
9.557589465	9.58708204	9.763138212	9.560346242	9.544495798	9.613206141
8.452374444	7.774617596	8.560860505	7.749943687	8.05474504	7.675499444
9.043522909	9.369545939	9.31327601	10.32531651	9.230770667	9.560346242
9.576136707	10.55602586	9.847335909	10.48185706	9.785983535	10.02269473
17.23317693	17.1822602	17.29704085	17.1822602	17.1822602	16.90924948
9.455015404	9.62302801	9.324388263	10.05320582	9.315671101	9.795448596
7.827906879	7.378186273	8.090488788	7.153761556	7.734170172	8.187593091
7.681488071	7.386173242	7.563777768	7.018881949	7.499261545	7.844550919
7.369510384	7.718083212	6.953914061	7.848409778	7.439837192	7.391952354
7.791244131	8.708995576	8.222643394	8.143040162	8.490053091	7.935468505
9.634846707	9.211846273	9.597513697	8.965143333	9.49510903	8.821885465
10.51400776	10.28899471	10.4994288	10.22761479	10.53141254	9.888393747
8.469779808	8.446184828	8.941795242	8.453185455	8.662363172	8.085049192
7.993077394	8.16147404	8.308331848	8.333749495	8.342368121	8.072532475
8.970512081	8.359263212	8.826846768	7.573484121	8.725113212	8.758871
6.765793475	6.555084162	7.377557566	6.630216838	7.071197202	6.709075525
8.942313313	9.35371997	8.816214848	9.710962273	9.136296333	8.904793061
8.927565404	8.583048444	9.300367869	8.358783152	9.044608384	8.638487899
9.836777707	9.753529758	9.57872197	9.917043434	9.671170444	9.632768747
11.28731295	10.15875628	11.09089144	10.79564384	10.42409385	10.84302834
14.09521817	14.37933382	14.19920898	14.50017253	14.26676228	14.00297944
7.91465098	7.601304687	8.269395232	7.607714535	7.68740998	8.043387838
8.02125603	8.120795899	7.899333596	9.01707996	8.19458801	7.738963899
8.181574081	7.28582396	8.502042192	7.212654808	7.662252303	7.651593505
8.654464586	8.287081333	8.762202232	8.257253131	8.718769576	8.79576798
7.332963121	7.494510091	7.688949081	7.737649848	7.175214949	7.225001677
10.17440526	11.15342364	10.92880859	11.66934136	10.28710845	10.77890896
5.915758535	6.250664576	6.395106727	5.893121081	5.807381162	6.301337414
6.471031303	6.943317879	7.891912283	6.577277596	6.385648535	7.453291162
12.32024961	12.41634374	12.52461444	12.33273925	11.89130603	11.95261936
13.09463792	13.66158872	13.00581854	13.96779242	13.21058954	13.06967433
12.66985986	12.9709957	13.00101781	13.50997822	12.8293171	12.97555423
8.620790505	9.251353071	9.030619364	9.446464909	8.433939838	8.615330515
9.072403576	9.264989899	8.907915899	9.137887434	9.273022949	9.071276071
9.884409465	9.08959502	10.50794857	9.079484556	10.0406788	10.50166798
7.228468091	7.371907727	7.500795283	8.44446797	7.500501152	7.176635455
9.018802576	8.758364879	8.72650097	9.253086273	9.124970051	8.853585596
11.7894918	11.92937262	11.58005536	11.76336308	11.69658017	12.2031413
9.062782333	8.618800343	9.043522909	8.196033389	8.760291222	8.564366949
7.354460354	6.413868758	6.617377051	6.839681778	6.229681899	6.951745222
8.559011545	8.829061111	8.599954677	9.287277576	8.414679525	9.08959502
7.380686859	7.496977949	7.024771818	7.356007838	7.437044505	7.437975364
7.150169929	6.267641192	7.638629778	7.163006	6.540677515	7.020975202
12.71830686	13.17281418	12.94821125	13.48990673	14.79546777	12.91656586
8.393536232	8.503061444	8.426780919	8.987499273	8.807959646	8.929384727
6.471690111	6.467159949	6.360244697	6.442532636	6.541618313	6.442753616
9.374690616	9.562364657	9.468134182	9.505859384	9.353057535	9.511857455
8.438712657	8.606099333	8.634342798	9.608350808	8.662816202	8.939019091
13.17281418	13.47832004	13.50411017	13.92894576	13.09463792	13.78027417
7.052765889	6.901740727	6.496990475	7.382214869	7.013931768	7.003455566
8.408296424	8.300483646	8.612168455	8.290726727	8.666113404	8.716387192
8.231756293	7.80435998	7.885378	7.248451727	8.204025576	7.371907727

9.618256808	9.749660394	9.518503263	9.681142253	9.695719162	10.00002479
8.962353222	8.995584808	9.271123182	9.118059475	8.990220404	9.023639636
8.173633596	8.091323071	8.029005444	8.31214998	7.955335374	8.203151141
14.57091557	13.62784329	14.56207529	13.22156044	14.1904887	13.05987966
8.361756343	8.560348606	8.041792596	8.618302869	8.31049196	8.543686687
7.469040515	7.531209667	7.993428586	7.444173596	8.165787747	7.298462212
8.978723121	8.617840212	8.109636222	8.510413414	6.760385848	7.249962576
6.543222263	6.560197424	6.399871131	6.649151414	6.654520283	6.32750801
7.862160495	8.124212202	7.707249808	8.188379657	7.623386535	7.863532101
10.0406788	10.18198959	10.21484784	10.10542249	9.902579253	9.814415596
8.604635949	8.907429495	8.639872707	9.250125475	8.96575796	9.147897788
9.659537192	9.806332162	10.87907816	10.92880859	10.91641235	9.393934131
8.930963202	8.385303111	9.67665103	8.24438497	8.781680606	9.285000091
6.506308818	6.301337414	6.685554869	6.340852071	6.461904303	6.547766404
8.252794354	8.356302828	7.90609097	8.483326677	8.472435657	8.217984283
10.59809845	9.341818545	10.59541023	8.664725051	10.04862183	9.721958051
7.120482	7.048453949	7.356848556	6.914008404	7.212383152	7.367244707
7.02750098	6.746600626	7.403028737	6.455374485	6.971315	6.812677121
8.166983455	7.635576919	8.636577313	7.821971636	8.235388636	7.793711495
6.931501667	7.118512586	7.169190859	6.937881404	7.081067566	7.178633636
7.264798293	7.059676455	7.61856696	7.311629869	7.440763838	7.121885657
7.927113394	7.973227556	8.440878707	8.020887778	7.870978364	7.981709394
9.616057222	9.635582141	9.951175646	9.86223396	10.11452638	9.620873889
10.88945836	10.687913	11.07956266	9.967178364	10.88945836	10.43655428
8.025864485	8.010919919	8.295839707	7.873080636	7.99823603	7.696259101
6.950648455	6.805470263	7.453021566	6.487487818	6.956636515	7.095534354
8.234636465	8.335861768	8.171342424	9.334658071	8.061012707	8.200961636
10.72246175	11.55322016	10.78705857	11.45738434	11.238428	10.48659409
6.587380232	7.015344242	6.438496414	6.918043889	6.69943696	6.625311455
8.809442889	8.705145061	8.705608919	9.035040848	8.473392556	8.720163182
9.276189121	9.356058495	9.86223396	10.1597097	10.19649023	9.560980909
11.09885051	11.49636188	10.77762269	11.72355631	11.07158908	11.37847359
8.673363869	8.583996586	9.208368677	8.409959899	9.194378495	8.48742196
9.080123545	8.540635232	9.472607081	8.437457535	9.107388182	9.198999222
9.11393401	9.721175788	8.767872848	9.612498475	9.164000111	9.052474394
11.38970236	11.0035084	11.3290504	11.24646499	11.12454893	10.72127155
11.41415047	12.44264271	11.9091429	12.36473556	10.24828231	12.47441306
8.29635502	7.931306778	8.923097444	7.862492343	8.568242293	8.096525737
6.911480061	6.526955566	7.386471808	6.737600111	7.01811702	7.204610747
7.657256192	7.74753198	7.969046636	7.970574879	7.442598384	8.384837869
9.507772687	8.706567556	9.870221061	8.649462798	9.384078242	9.757590192
6.161616828	6.132562212	6.409677646	6.097038818	6.134392101	6.517646071
7.316995263	6.981850818	7.759566061	7.19681397	7.57318396	7.311327071
8.193428737	7.824443343	8.607626293	7.698300838	8.286610323	7.648986848
8.687053253	9.171801828	9.429506515	9.155642525	8.965143333	8.29927897
6.630696545	6.687730495	6.889521525	6.923167202	6.931501667	6.645437646
7.668279394	7.274624242	7.511838192	8.143776848	7.117115202	7.666980273
8.742635939	8.71185199	7.337670899	9.845595727	8.817191758	8.743124222
9.722667242	8.782655051	8.240375404	9.735412273	9.019731374	8.864955909
8.197805768	8.134366646	8.479727162	7.22559698	8.388817192	7.68296396
10.31597323	10.63391466	10.40074116	11.35475345	10.29660096	10.24828231
7.969046636	8.822318	8.755916071	8.539776737	7.840343939	8.964642636
10.63391466	10.76857843	10.66706732	10.84568098	10.55602586	10.5431016
8.983202444	10.01257663	10.00002479	9.988725727	9.381544081	8.779146061
11.0940021	11.85889751	11.44643795	12.17829278	11.73271713	11.96059797
7.527697455	7.468494556	7.070939121	7.332078838	7.28082202	6.797872071
7.329578283	7.439837192	7.119927869	7.954533778	7.149668323	7.163006
6.64233597	6.557897323	6.107645465	6.428567879	6.088380414	6.475135818

7.483574061	7.617533182	7.486088616	7.082724758	7.801006182	6.956131545
9.472607081	9.497084828	9.014335657	9.573320202	9.645545465	9.724047525
11.10170724	10.96748398	10.76609029	10.93171462	10.8947391	11.08264222
8.462637525	7.940978939	8.914720172	7.767702525	8.669446475	8.86638896
8.216297535	7.942409707	7.801818071	7.769155828	8.458294828	8.135177152
10.83642757	10.61992902	11.06732533	9.813557354	10.84974643	9.224452333
7.860366808	8.010523545	7.310724131	7.983548525	7.41731598	7.92597801
7.352546465	7.230900293	7.272599475	7.073400869	7.386763141	7.143674071
6.718530879	5.972872515	6.109379758	5.917971949	5.840210848	6.069475828
9.993897848	9.769223525	9.525471414	9.724814657	9.993897848	9.359783616
11.03580591	10.72633412	10.99186298	10.42622906	10.82804231	10.09079019
9.689783586	9.690534253	9.392652323	9.318228697	9.724814657	9.277992232
11.07158908	11.49839615	10.86173253	11.48840455	11.25349741	11.28053313
9.323195364	9.267383556	10.24511785	8.906407303	10.25038968	7.694540808
6.976233182	7.567404636	7.130479657	7.119081899	6.66692301	7.916712838
10.36589334	10.17440526	10.49241362	9.682548525	10.27139209	10.06173934
9.685649929	9.877792838	9.422377343	9.633493677	9.675185677	9.66613299
7.812003626	7.790242404	8.081502051	7.418614091	8.149448323	7.235098212
10.08978265	10.76983035	10.38842688	10.28406896	10.37573078	10.30470526
6.072635697	6.404620899	5.84728504	6.608273162	6.578458283	5.883593636
9.470173818	9.701165495	9.285586323	10.30470526	8.934777717	10.07479617
8.214678465	8.267244596	8.131862263	8.148308414	7.862831293	8.727989172
9.694925798	10.09896607	9.613206141	9.798623747	10.06255975	10.01524205
7.811684818	8.065153556	7.369773828	7.679923515	7.540776061	7.798177384
8.757866131	8.856343687	8.534432515	8.85308204	8.718321818	9.019287687
7.564402343	7.339214566	6.985632404	7.260583141	7.020179424	7.636533838
8.950355889	8.522156747	9.028300162	7.711523556	9.129654758	8.825312061
6.774936697	6.702267949	6.329109535	8.179951646	7.228468091	6.911201424
8.453639616	8.209418434	8.782655051	7.949790515	8.39446903	8.587234515
8.993505475	8.719668061	9.648698737	9.290785283	9.382882596	9.684048687
10.25422487	10.40074116	9.699589758	10.24227579	9.534883657	10.64005918
6.737335172	7.253764222	7.038648475	7.063477202	6.832363455	6.958579652
6.315590828	9.150235333	8.446641939	8.713417869	9.526200354	9.138451657
8.297630758	8.45577798	8.179581869	8.099825778	8.317411434	8.162329636
6.935285707	7.286424495	6.898276768	7.33350399	6.993764091	7.150454879
10.88302044	10.88430477	10.75865445	10.65751844	10.96000427	11.12315281
12.63102018	12.6076243	12.72219022	12.73352188	12.22341713	12.88265743
8.788663253	8.800397414	8.707515232	8.860747333	8.431738505	8.65497299
7.57318396	7.715405939	7.42612497	7.907197636	7.762451808	7.443845071
10.21975253	10.2752886	10.71390019	10.25320711	10.05970796	10.74558579
10.41626133	9.739859192	10.54078574	9.121511687	10.80900786	10.65365596
13.16151113	12.96662998	13.18348162	12.76207554	13.28559105	12.56726296
9.821914929	10.08521343	9.798623747	10.00361533	9.805520727	9.699589758
16.4534382	16.57174008	16.82803465	16.57174008	16.49551516	15.97330865
7.476910626	6.96124304	7.842359758	7.07857001	7.474059606	7.647964475
11.11714857	10.95533553	10.90754603	10.73384189	10.67305197	10.67815746
6.907588505	7.508036465	6.816006121	7.672658859	6.914534515	6.737819111
8.254082232	8.376150616	8.911109919	8.390560192	8.845785121	8.676791091
7.004269242	6.740481444	7.290498626	6.618269616	6.561305909	6.84909404
8.273878929	9.196689051	8.197004869	9.122783717	8.593101434	8.396963808
8.345353354	8.602859283	8.30620801	8.843670869	8.603745778	8.325717687
9.206093556	9.067241768	9.380979222	8.416400293	9.311440606	9.404333879
6.178002222	6.285312596	6.303106697	6.243017283	6.945626949	6.687954879
7.805761556	7.678591667	8.065153556	7.25490398	7.718083212	7.740855141
11.04593523	11.81887725	11.82346178	11.11081747	12.07810634	11.02851208
7.022843394	6.691780525	7.525850576	6.858687869	7.145208253	7.18832599
7.302736424	7.473746677	7.682204525	7.762451808	7.574489596	7.663618646
9.134591697	8.39227803	8.859179455	8.736221495	7.842052606	8.994589434

9.470840162	9.001272616	9.45706402	9.421059	8.876650909	9.409395929
7.428304182	6.757659566	8.316969364	7.153217667	7.471847707	7.668279394
6.743573939	7.236914141	6.796104869	7.755132808	6.859220677	6.812168303
8.597027768	8.068931465	8.675348646	7.935468505	8.31214998	8.350619919
8.360501364	8.082337909	8.651919778	8.198599495	8.385303111	8.617840212
6.901464747	6.677606	7.173022687	6.575452697	7.077389495	7.047046253
7.97830402	7.722652869	8.111336758	7.505175253	7.901917293	7.904381697
12.65104366	11.71895032	12.2293359	11.44491584	12.57486869	12.28985985
9.911926424	10.24038579	9.748306121	11.12001247	10.68960982	9.805520727
8.76386996	9.047491869	8.510894717	9.320176919	8.918756414	8.744081071
7.775630727	7.575132364	7.574489596	7.579015667	7.51310204	7.722297051
10.27804811	10.20060626	10.49592664	9.435122859	10.09079019	9.857940545
8.87289497	8.491898242	9.050265121	8.698215303	8.961854929	8.900779434
12.52819783	11.79405389	12.3543152	11.51647662	12.052647	10.69931975
9.337187111	9.027655758	9.591133828	8.800397414	9.821914929	9.49850403
8.429130081	7.805446323	9.320738646	7.620680879	8.287888051	8.652374404
11.07956266	10.45150222	10.79145221	11.20498985	10.86691694	11.22947614
8.396125929	8.291610121	7.957660747	9.279768394	9.100524758	8.226500707
6.938620788	7.110844919	6.921417374	7.479706475	7.106848111	7.078303869
10.08325108	10.27139209	9.933588354	10.61001127	10.24227579	10.31480319
10.4700758	10.1684149	9.817379515	9.901640636	10.34418014	10.85225864
7.45998899	7.335878859	7.518690889	7.715405939	7.719466677	7.627030141
6.818245747	6.729937747	7.199742475	6.714232707	7.303307	7.092673939
6.395106727	6.442753616	6.487487818	6.353983172	6.624146566	6.368510475
9.755608293	9.842388081	9.759214424	9.435722162	9.662314131	9.373397364
7.232737253	7.411561343	8.097566687	8.107270071	8.304980606	7.420173404
12.0724233	11.50261564	12.73352188	11.56291631	12.27773509	11.99535117
13.63435472	13.39659876	13.80033164	13.70143341	13.04869312	13.50997822
8.463063475	8.342773172	8.731576192	9.186711646	8.207099091	8.38301802
7.495424616	8.156085586	7.900715717	8.493653778	8.064260859	7.765675424
11.26262606	11.58005536	11.37490296	11.97447062	11.33848921	11.5612151
7.723687384	7.618249414	6.939713242	8.620285051	7.31523196	7.620680879
7.354199768	7.55178802	7.134684455	7.66930299	7.427086202	7.467207283
9.057196687	9.022000869	9.057196687	9.189590182	8.635899485	8.862225737
7.030426444	6.400997131	7.477539293	6.517425313	6.871904646	7.548285091
12.60022648	12.34855768	12.24933389	11.71042621	12.10414281	12.72610186
9.550769788	9.582110717	9.675904202	9.55462702	9.582110717	9.426842081
10.00695165	10.05970796	9.680349586	9.687758929	9.912769303	9.964343626
6.895441525	6.598910859	7.506719576	6.644450293	6.949335212	6.947262939
9.404880263	9.398445515	9.954681949	9.414554808	8.915814212	9.510536
8.517288667	8.633848111	9.096947232	8.451947071	8.123471404	8.448008778
7.825440475	8.896578162	8.390560192	9.221852535	8.887109626	7.669946162
11.53239215	11.79615897	11.34202326	11.50425191	11.24993851	11.15666056
5.820161667	5.80542502	8.772410586	5.979399586	7.144299071	5.762029485
9.660200838	10.05053825	9.292435667	10.33476777	9.535633242	9.587719636
10.97185531	10.90324033	10.59924244	11.03163187	10.7442872	10.93171462
7.182269455	7.326942212	7.348317141	6.590932293	7.055055273	6.283373303
6.937104768	6.715644404	7.406536232	7.208855192	6.935824606	6.47408797
9.608350808	9.143279535	10.61621963	9.023639636	9.826032111	8.44129198
8.142577798	7.824090626	7.587331949	7.899333596	7.835788293	7.864861071
12.89113428	12.03068847	12.72610186	11.4535969	12.61761735	12.13867659
10.43122177	10.76361324	11.0035084	10.42409385	10.41846051	10.21484784
10.93935201	10.53028115	11.39594259	10.57649171	10.82290783	10.74172297
7.692888131	7.654890798	7.967524909	7.672313364	7.691535778	7.74646602
6.521234222	6.367511859	6.885309253	6.128300636	7.309668525	6.155511101
9.091299889	8.893968333	8.838020616	8.963499596	8.978723121	9.283736939
7.580594545	8.595703707	8.816703535	7.428935273	6.801007778	9.253086273
11.3290504	11.39983314	11.40495249	11.14057221	10.89094316	11.20498985

8.933684374	8.568768141	9.136890505	8.397853727	8.364284556	8.550897414
9.784374313	10.10093339	10.39747702	11.02551486	10.11664425	10.15379546
7.415013667	7.493565131	7.249375465	7.370661465	7.975784071	7.336476646
8.744562374	8.445759657	7.105225354	7.660499434	6.79984598	7.076844636
7.145493505	7.117115202	7.628336929	7.228759818	7.630934212	7.208855192
7.951199808	8.290726727	7.949400121	8.45577798	7.98506802	8.280707687
7.171072313	7.208855192	6.816537596	7.332344778	6.982654747	7.30556202
12.6076243	12.66985986	12.51066587	12.60396528	12.40735915	12.28693608
10.40178901	11.08756575	9.987077505	10.73911267	10.69793218	10.44832821
7.994566778	7.491357222	8.019097616	7.088250616	7.700026646	7.464044657
7.885002121	8.119288848	8.232163879	7.599486586	9.203760495	7.799854778
10.26224218	10.70299608	9.876096838	9.987874111	10.93789932	11.02403513
6.097038818	6.456921838	6.497193293	6.207714091	6.838224475	6.158146455
7.862492343	7.713612677	7.238770545	7.368198747	7.318216242	7.502072596
8.40783399	8.449337667	8.63794701	8.228510596	8.291166909	8.341133162
8.352537101	8.287438535	8.524867051	8.096910323	8.395296687	8.626833051
6.240680768	6.345431182	6.638813101	6.31629898	6.393414081	6.048882657
7.40806004	7.462771485	7.366006364	7.748187364	7.309928404	7.49893601
6.899006192	6.382806586	7.23423804	6.422427222	6.624377646	7.267645253
12.97977545	12.01673563	13.09045797	11.32185019	12.7452022	12.16036216
8.514431737	8.670884566	8.764299566	8.332313525	8.995584808	8.598479657
7.291703707	7.543741273	7.059062808	7.010236465	7.28582396	7.533990333
6.041192253	6.278089182	6.535075677	6.04186799	6.234266364	6.443018677
12.62449502	12.3543152	12.70787607	12.3613163	12.62127559	12.34855768
9.015412101	8.946107263	9.346206162	9.036076414	9.03221704	8.848914293
9.219562515	9.142791657	9.522660222	9.077686808	8.843670869	9.422377343
8.050484323	7.69185196	8.109266465	8.136641818	7.81270001	7.695265798
6.991849242	7.03650198	6.865784889	7.173324505	6.564307798	6.756647869
9.172421828	8.849981545	9.345614434	8.528049141	9.18155302	9.395177889
9.554043101	9.605576293	9.971733293	9.776839404	9.768390455	10.26541026
7.300031424	6.940977263	7.640655485	7.226681909	7.604779273	6.910411354
5.77310201	5.782454909	5.805546232	5.777686707	5.779800404	5.821925616
6.610658212	6.653328071	6.798121798	6.995800606	6.587380232	7.107390485
8.460863192	8.361327455	8.810446485	7.381950828	8.76328402	8.494130061
7.25632596	6.714232707	7.390752071	7.07468796	7.098723051	7.165241424
7.427999919	7.335553323	7.671246505	7.065103374	7.433629313	7.437044505
8.617420111	8.095746162	8.152443121	7.308145939	8.764299566	8.598008343
7.85507	7.417927455	8.370422404	7.175798869	7.815532475	8.050868949
9.587719636	9.051375475	9.638947465	9.29794999	9.833438364	8.532119949
9.706432808	9.515069697	8.69919198	10.02439619	9.195534384	9.991229606
7.533990333	7.499261545	7.274379929	8.048636061	8.041000495	7.644277273
6.855446939	6.589551859	6.831893455	7.178323677	6.930500768	6.674468929
12.02514953	11.96362869	11.88909817	11.67849095	12.05489479	11.42562271
13.37822002	13.72760196	13.08418191	13.66784462	13.29117716	13.64789782
9.974493182	9.362238061	10.10631712	9.255548899	9.771430818	10.2026692
6.55365803	6.506758646	5.912175495	5.933111222	6.674272111	6.538664657
6.405475687	6.395369727	6.475338556	6.363322586	6.379374253	6.455163758
8.657806242	7.958031758	9.383480283	7.820605525	8.5055425	8.751946515
8.090886384	8.807959646	8.580231323	7.99823603	8.968391182	8.145194646
7.516753121	7.09469803	7.572856394	7.510343899	7.300950758	7.213178616
9.093001495	9.581418808	9.061649657	9.509876273	8.901935111	9.513795939
9.852239606	10.09506496	10.14432724	10.38208186	10.25627793	9.983540212
8.969483	9.115691495	8.922082808	8.808928141	8.738735232	8.887684293
10.09161001	10.11849919	9.639623879	9.927686616	9.857104919	10.0841781
13.46005067	13.87958112	13.38447499	13.79381649	13.38447499	13.71423947
7.683943101	6.941216071	7.859293	7.102414636	7.473746677	7.773582384
8.121954505	8.388817192	8.576891556	8.35372597	8.154321646	8.260123323
10.44712342	10.1752482	10.3779146	9.208368677	10.06822268	10.62647628

7.870669919	8.282438737	7.896600606	7.48289603	8.269395232	7.520648323
9.295516616	9.631182697	9.372025394	9.063885091	9.70798304	9.569063343
6.502291131	6.47341896	7.172411071	6.512566313	6.763342091	6.759694808
9.481668929	9.155079848	9.20200202	8.550414556	9.466909636	9.395854121
8.765233303	8.768300525	8.552208182	9.513164303	8.833337414	9.035040848
9.044608384	8.557697889	9.321347081	8.609511	8.977005354	8.760676242
7.103272273	7.294038515	7.068665737	7.690840778	7.060512141	7.60255999
9.044046343	9.039989364	9.739033131	10.24511785	10.26224218	9.274850788
7.838173152	7.525850576	7.714004283	7.170490939	7.971654859	7.992662242
8.31008898	8.350619919	7.969793182	8.227270172	7.932453717	8.531247657
7.596851556	7.487351192	7.126018333	7.60282502	7.445746576	7.514494576
10.70902317	10.92757694	10.31894523	10.38631174	10.80091358	10.81796524
10.84568098	10.61621963	10.58812248	10.43757507	10.65127749	10.69793218
7.865908465	8.007640657	7.830796253	8.03179299	8.192645727	7.907575465
8.689422596	8.655437626	8.760676242	8.423588242	8.550897414	8.802385697
6.529317889	7.007335798	7.443557333	6.230315707	7.950135394	6.638362242
12.94367069	13.12023156	12.89113428	13.1944969	12.96662998	13.17778072
8.806450121	8.375668162	8.988033424	8.109636222	8.529888505	8.838495424
10.60044575	10.80389925	10.62647628	10.4687107	10.25906505	9.576136707
7.878518051	7.639662323	7.377269152	6.905091242	7.038648475	7.797507939
7.42612497	7.765675424	7.987912222	7.504556444	7.307562737	7.224144889
7.190368869	7.264201869	7.709908667	7.901917293	7.360451687	7.324608717
9.499247869	9.393335687	9.365016101	9.244399414	9.326739394	8.773988747
6.857008404	7.038917566	6.620950929	7.004818101	6.961028939	6.887288828
10.64623997	10.71731517	10.43757507	10.8778129	10.46342714	10.59541023
13.10985871	14.46162431	15.09418019	12.80602179	12.51766186	14.93426708
8.732044667	10.00262223	10.27236099	8.230588212	8.203151141	10.43440935
6.439789939	6.264444141	6.960749798	6.804494667	6.58529104	6.399225919
7.521266808	7.068665737	7.991889949	7.331138333	8.078389566	7.678926152
8.133538364	8.423139949	8.029383646	8.341518747	8.258465414	8.177146606
10.31997281	10.26632574	10.13375911	10.20452777	10.080472	10.05780386
10.33375706	10.36032821	10.06904603	10.9660467	10.32750324	10.35284213
7.64030596	7.911309091	7.339214566	7.83046296	7.658441616	8.250622253
9.846508495	10.33146686	9.683334444	9.974493182	9.97079498	9.870931818
8.094989869	8.072924455	7.450888848	7.678926152	7.782796596	8.093473121
8.861728919	9.052474394	8.311754818	8.966940586	8.852576737	9.009407333
8.840010495	9.117478424	8.935320828	9.812775374	8.753420071	8.750916455
11.42562271	11.78498903	11.28731295	12.06397065	11.68700199	11.671419
8.607626293	8.391020323	8.815152444	8.76328402	8.213427111	8.710842768
11.70812672	11.89361272	11.43380494	11.58421758	11.79405389	11.71659127
8.956407626	8.691979949	9.399129424	8.725113212	9.123926859	9.121511687
8.204737434	7.873377879	8.498949848	7.788172758	8.419470808	8.097566687
9.845595727	9.481000434	10.06450841	9.223885747	9.91449603	10.16546821
9.49850403	9.831087364	9.377908	9.848945263	9.217165182	9.424273283
7.643944697	8.135177152	7.713270545	8.361756343	7.777661384	7.662925475
10.41957053	10.43332542	10.22164118	10.71390019	10.51650912	10.55474917
8.402589283	8.133538364	8.813032152	8.619347838	8.419866586	8.602379626
8.780633818	8.883993253	8.592622071	8.761644081	8.684654737	8.434412929
8.985325394	9.069043081	8.769354545	9.005433152	9.008373061	9.250770152
9.232683343	8.90531298	9.000672535	8.813595202	8.917069505	9.11093099
8.431738505	8.187136596	8.450171172	8.050868949	7.991026727	8.274284293
9.826032111	10.23351878	11.00183361	10.36134852	10.14999886	10.3918566
9.031713778	9.31393701	9.149684939	9.811898121	9.132080535	9.717365758
6.361580141	6.056037747	6.637667434	6.070726232	6.092545697	6.478141798
7.530574707	7.927460424	7.855731	8.234636465	7.271416848	7.306475687
6.533739475	6.57397498	6.617114677	6.801276	6.795634141	6.443657091
11.40306525	11.05356995	9.25368898	10.19136651	11.45738434	10.72786832
7.783465061	8.288815495	7.847720859	8.705145061	8.020096283	7.796747616

8.399553939	8.559975162	8.832790172	8.765233303	8.681067818	8.430534091
6.961758566	6.562194212	6.954196414	6.418892273	6.907322414	7.001612657
10.25320711	10.60383217	10.76983035	10.19757035	9.66383	10.6313464
6.778194636	6.916181	6.460794313	6.290108071	6.444612172	7.079662576
8.895503556	8.820759798	8.747331152	8.843148071	8.749346626	9.378583414
7.59498599	7.410944101	7.196057152	7.924172566	7.570765313	8.189194404
10.09896607	10.36903954	10.22865659	10.26332497	10.26332497	9.826793596
7.845601525	7.775998535	7.952386505	8.597027768	7.787525596	7.764931778
8.924201657	9.008956374	8.44129198	9.202552434	8.974650333	8.752460404
6.506975192	6.736847222	7.203245626	7.404051424	6.752477677	7.217862778
7.546592828	7.428605818	7.144617717	7.407756293	7.51810503	7.738613697
16.14978837	16.04217659	16.17943014	16.02065153	16.14978837	15.41210544
9.896649636	9.66831603	10.42297323	9.473222667	10.20859114	9.93663897
8.394903626	8.30460302	8.629055081	8.225777414	8.53715797	8.205892515
8.530767747	8.258860253	8.042974687	7.955335374	8.191204879	8.257984293
7.955335374	7.453291162	8.287438535	7.421318101	8.061425949	7.803193929
10.23247836	9.839111	11.33657706	10.40074116	10.56908161	10.38430498
7.243824414	7.382214869	7.335252788	8.211847566	7.834378071	7.405823253
8.006495333	8.295436051	8.463578343	7.884680111	7.440186081	7.960547
8.835019636	8.453185455	9.174793152	8.228946717	8.69335002	9.081927515
8.991970091	9.015412101	8.823394192	9.143279535	8.703733141	8.765233303
6.802328384	6.309118495	7.212383152	6.430045889	6.625506687	6.943550111
7.274888343	7.387654737	9.482964828	9.94767803	9.538263677	8.474228909
10.51771241	9.579413505	11.08264222	9.781348374	10.41530743	11.21958266
7.956286283	7.837840162	7.895278253	7.954205616	7.846688253	8.299695162
6.896238273	6.773917293	7.798826303	7.352546465	6.986482212	6.920203848
9.49510903	9.746868596	9.264483889	9.439397061	9.67665103	10.25811169
8.391020323	8.233838889	8.32856404	8.289996192	8.365106485	9.302103889
17.67797782	17.58093558	17.67797782	17.67797782	17.67797782	17.36803328
9.680349586	9.973623707	10.15086094	10.39641485	10.00186072	10.10841911
8.498167798	8.852576737	8.615838	8.03490996	8.725555596	7.686444293
8.229339455	7.636934919	8.038729465	7.423442323	7.687096788	6.975188859
6.724536455	6.698319919	6.313338182	6.554351	6.637667434	6.802811828
7.523206263	7.269064929	8.047188455	7.461834424	7.68296396	7.653990838
9.088505869	9.058356636	9.168324747	8.928157121	8.879330566	9.058895657
10.25627793	10.88681216	10.34626898	10.90754603	10.59809845	10.5041569
6.601717263	7.059062808	6.512566313	6.456009747	7.170797768	7.100167172
10.73788216	10.65500785	10.60507136	11.06261544	10.24942024	10.93494087
10.1985269	10.12314196	10.23351878	10.38531322	10.20748734	10.23247836
10.90192126	10.16937171	11.39772243	10.05607537	10.99763472	11.09565519
7.627373273	7.575754657	8.034450929	7.737987747	7.892936636	7.468494556
10.75600216	10.35284213	10.73788216	10.41224321	10.89224764	10.97896732
6.369108576	6.177173253	6.579612929	6.219076051	6.366615303	6.597742293
7.45822897	7.196057152	7.693828071	7.019424707	7.545986354	7.63758797
6.156509616	6.312584202	6.436392303	6.506758646	6.890761263	6.168008697
7.800624061	7.854325061	7.623693616	7.601973414	7.477241869	7.635907081
6.683791949	6.083800768	7.243253313	5.991626222	6.840910293	6.634850818
8.337550606	8.80133401	8.544134919	8.824381182	8.046104414	8.398231626
7.444173596	7.675499444	7.401460495	8.000144121	7.559825556	7.548584576
7.176396859	6.575452697	6.312584202	6.817257404	7.017519192	5.984555323
10.17904437	10.36239309	9.796940758	10.16642595	10.34626898	10.38329808
7.061925202	7.519364889	7.21931098	7.544047919	7.248451727	7.18112796
7.451512687	7.511268929	7.550195808	7.436336172	7.464704768	7.341339747
9.235179424	8.952561111	9.152615889	8.891888576	8.863920758	9.085558838
7.716801838	7.305875394	8.052417212	7.582654121	7.58864003	7.368501293
10.67428006	11.05959848	10.46766096	12.0838996	11.39983314	10.59809845
7.020691646	7.651593505	7.439506505	7.238133434	6.850381687	7.45822897
12.43279956	11.94745099	12.27101	11.42371373	12.04432	11.80554878

6.919663687	6.939171505	7.163863636	7.050073121	7.12986801	7.210929778
7.911995121	7.939228495	8.302900242	7.689906808	8.656360697	7.941721242
5.837264263	5.877009727	5.839692404	5.881038758	5.958288828	5.74913202
8.716864899	8.065602475	9.167114	8.006915303	8.908935182	9.027655758
12.37416009	12.18492446	11.98196729	12.87421343	11.66441291	12.62449502
5.812753323	6.870354606	5.927094424	6.255110242	7.725042051	5.763252606
5.853798667	6.051299838	5.869663172	5.968970758	6.458735293	5.760741364
17.36803328	17.49517975	17.36803328	17.43906183	17.49517975	17.43906183
7.203788636	7.048729333	6.893369586	7.057330242	7.077096727	6.950101798
11.13085117	11.09721747	11.15666056	11.05651949	11.34202326	10.96748398
9.285586323	8.912452162	9.813557354	8.62412801	9.081927515	9.329922273
9.998242414	10.35699559	10.3380268	11.28053313	10.43655428	9.973623707
9.952967535	9.184000242	10.04421951	9.452497313	9.767642525	9.474605101
10.57758511	10.99763472	10.45375986	11.24457024	10.86820493	10.94208941
11.43773109	11.71443529	11.07337622	11.54470354	11.42562271	11.36610866
7.202701576	7.42612497	7.140902202	7.252299788	7.368198747	7.59435096
12.41337695	11.56725755	12.47130519	11.30301738	12.03912361	10.67572435
10.61133106	10.56802411	10.33146686	10.25906505	10.4283734	10.52476206
9.264483889	9.108564253	9.333986848	9.007843273	9.08614898	9.640429374
8.685647889	8.715328101	8.332313525	8.972363838	8.582147707	8.404396899
7.921614596	8.204380404	8.045722828	8.630877737	8.026271202	7.690510313
9.689129545	9.519820636	9.249034202	9.571117879	9.509226798	9.821195081
7.244714616	7.663618646	7.604109192	7.484854374	7.14692997	7.235366172
9.159196141	9.204345657	8.652374404	9.464440242	9.069043081	9.101613848
9.991229606	9.584803364	10.09161001	9.353057535	9.827596687	10.09506496
10.00450786	9.93099102	10.05688349	9.959124273	9.865550636	9.516398909
7.18313201	7.309054838	7.104098253	7.280239242	7.24440804	7.406536232
11.29230964	10.85100254	11.2786408	11.12935449	11.07013707	11.34576518
6.605935465	6.074210909	7.025021455	6.070436798	6.303290121	6.658365848
12.47761486	12.71830686	12.13867659	12.61090773	12.57107442	12.70002263
12.64708972	12.73352188	12.34855768	12.75396286	12.58957491	12.92542832
13.70143341	13.46005067	13.39659876	13.35919327	13.35919327	13.65450155
11.82112126	11.62739451	12.79024082	12.64708972	12.57877126	11.82616001
6.886441253	6.833605444	6.930987343	7.32489101	7.449616394	7.258693172
7.03650198	6.204346798	7.769155828	6.316919404	7.451183323	7.813759899
9.811898121	9.769994879	9.573320202	9.785178838	9.699589758	9.987077505
9.017695354	8.986932636	8.751946515	8.855784909	8.686679798	8.550414556
10.55726192	10.40486524	10.43564423	10.49009312	10.4549623	10.17789322
8.851571051	8.181963182	8.960773354	8.099466788	8.796713535	9.133918929
10.16165443	10.3305428	10.25220538	10.52135963	10.02269473	10.26832521
6.792458242	7.138289616	6.768970657	6.985632404	6.636058242	6.803567182
9.374121768	9.596773313	9.854472677	10.55213977	9.569854202	9.220765354
10.18000397	9.925226333	10.27035429	9.953782444	9.995698596	11.07956266
8.269395232	8.146343545	8.03364202	8.399975414	8.399553939	8.331888859
16.12384164	16.2036826	15.88528111	16.22759729	15.88528111	16.42659037
10.09426568	9.966143818	9.946747141	10.23948175	10.47702836	9.721175788
10.71503879	10.29660096	11.15808934	10.31289083	10.78987961	9.856174394
8.163053768	7.578291899	8.482852263	7.802180768	8.074096707	7.627670859
7.678926152	7.750957677	8.300103657	8.065602475	7.577683121	7.701991131
10.46766096	10.08893073	10.71145754	10.11051676	10.78408516	10.24139873
7.902575141	7.776629576	7.672313364	7.551492101	7.554702939	8.055916879
7.268488485	7.646953505	7.17606597	7.680240364	7.282365313	7.425828556
12.56726296	12.62127559	12.25251148	12.39058663	12.24351077	12.99088515
11.58421758	10.81423156	11.65455063	10.70902317	11.14707085	11.22456459
11.77305263	11.70557621	11.72592113	11.5571076	11.71659127	11.65261158
14.25895571	14.26676228	13.78737352	14.01682491	14.04335209	14.28325373
8.544134919	8.64752602	8.376150616	8.379884889	8.290353162	8.742635939
11.05651949	10.75600216	10.75453103	10.69222123	10.64502212	10.78987961

8.292068253	8.541972566	8.174069929	8.434806414	8.396532182	8.282900293
11.58814301	11.13879804	12.0724233	10.70052069	11.61117883	12.0750029
7.425828556	7.094450071	7.584620515	7.059956313	7.133813545	7.314025869
6.020767303	6.351647616	6.760181828	5.919581566	5.964018485	6.64694096
7.313208869	7.179445253	7.282365313	6.897750404	7.469634172	9.206093556
6.864210101	6.759203	6.492854313	6.776493808	6.575656404	6.924744576
7.917403434	8.290353162	8.033283465	8.696739152	8.383429707	7.596246919
8.566871343	8.737817051	8.911806707	9.528804131	9.356741384	8.251922909
15.42966281	15.21350605	15.50564301	15.06622261	15.36066179	14.77222727
8.595283071	9.131560131	8.530767747	9.250770152	8.539776737	9.043522909
8.072924455	8.184399818	9.154469939	8.374363061	8.76386996	8.824381182
9.477759242	9.444057586	9.337862091	9.390834152	9.562994313	9.627013505
8.967408879	8.780633818	10.03385507	9.502644929	9.245932475	8.532516465
11.53473316	11.63401423	11.99323436	12.28985985	11.50261564	11.82346178
7.601973414	7.550541152	7.85299496	7.20129803	7.813018717	7.88825004
6.331869444	6.576569869	7.441392919	7.71023803	7.723687384	6.597234394
7.428605818	7.407756293	7.528004313	7.453956636	7.387388131	7.310482798
10.59292728	10.89912578	10.67939021	10.95849458	10.98350754	11.46320141
10.58469265	10.32418729	10.73517618	10.75737971	10.4994288	10.7442872
9.074692404	9.299916465	9.575401111	9.194378495	8.603276828	9.35728196
10.02644328	9.902579253	10.01168912	10.34830463	9.412044242	9.756054152
9.761765848	10.24139873	9.604175636	10.61480751	9.372025394	10.24227579
10.04600105	9.934615404	10.50914282	9.652939283	10.14809211	9.957374697
15.90458538	15.63778652	15.90458538	15.50564301	15.90458538	14.9591327
6.238699505	7.104930788	6.243017283	6.529317889	6.204026394	6.604082657
8.641223283	8.656360697	9.153232364	8.31049196	9.350012182	8.01602699
8.825312061	8.420871687	8.844262616	7.90403798	8.880853535	8.798247798
8.053971495	7.503050071	8.621031621	7.683943101	8.418118394	8.304169929
10.89604789	10.98627861	11.13400706	10.99046505	10.69574272	10.72900488
6.059347778	6.176506162	6.420979687	6.088521525	6.328925929	6.166487869
7.412191343	7.164102909	7.457078101	7.319616859	7.680542242	7.119314485
7.730154939	7.923048354	7.941320778	7.819085697	8.134366646	7.542431869
8.336651323	8.221010253	8.740682	8.031407364	8.286128505	8.808928141
7.280239242	7.336756222	7.334639071	7.458795919	7.025264747	7.334066545
12.76207554	11.77053658	13.42888134	11.8358519	13.23341018	12.04734694
8.928828384	8.389243343	9.589694828	8.338355444	9.047491869	8.204737434
8.04259702	8.154321646	7.873080636	8.201303758	7.823083152	8.168196313
9.170513566	8.494982343	8.201745222	8.249809909	7.838558162	8.861258687
8.77608297	9.548028869	9.033915556	9.544495798	8.825911939	9.857104919
6.356488616	6.153502808	6.774434798	6.305750758	6.213769515	6.160939939
6.76551097	7.086698091	7.632861657	6.876638434	6.611130667	6.534227879
11.18096106	10.79564384	12.82558839	10.60743282	12.62807191	11.1060681
8.287438535	7.030955384	8.11773804	8.27018399	7.252886707	7.796085586
6.83507799	6.577737222	7.035420475	6.708558414	6.810861495	6.910146768
9.110297333	8.653906556	9.156840283	8.491049758	8.660166475	8.737323475
10.20164867	9.956562586	10.1300726	10.03205964	10.04421951	10.01257663
9.497817424	9.408808788	9.176817596	8.585836293	9.048620525	9.147296667
8.79576798	7.842052606	9.069043081	8.055566596	9.10558002	7.249066101
8.419866586	8.495994869	7.93437196	8.679110091	8.225777414	7.882189879
7.914965525	7.861189172	8.174468182	7.274624242	8.256076333	7.289331586
7.835124576	7.952386505	7.434204455	7.819838162	7.738613697	7.668638899
8.598980253	8.880364303	8.495994869	8.314030071	8.76626204	8.599954677
6.079466192	7.245319404	7.02555202	6.512378081	6.082667919	6.264444141
10.12041459	10.23550694	10.13479405	10.761049	10.37686258	10.54565144
9.663106646	9.904237354	9.515069697	9.97079498	9.546645747	10.57885616
7.725042051	7.422534091	8.18481599	7.309928404	7.520306162	7.327495141
12.54589185	11.98440811	13.02518615	11.84291489	12.64708972	12.89513437
14.65397593	13.85099976	14.9847254	13.45466248	14.28325373	14.1830221

6.76309904	6.698080333	7.545628697	6.539575101	7.14635899	6.758137737
15.83165575	15.41210544	15.78787594	15.449005	15.65613201	15.76560628
9.899010949	9.855298394	9.618955576	9.637663091	9.853774707	9.669114061
9.285000091	9.307024131	8.858692343	8.959665061	9.191263899	9.239472869
8.838951081	8.77298297	8.814624313	8.680072242	8.713915657	8.942756889
8.858269384	9.29496	8.560348606	8.818210192	9.199527535	9.08614898
9.733950273	9.991229606	9.801802283	10.4549623	10.25320711	9.53690398
10.4524716	9.583475303	10.64379219	9.918671212	10.31693391	10.89755671
11.36979611	11.5737214	11.39983314	10.5041569	11.19638858	10.36239309
6.755664455	6.645437646	6.473192182	6.250843515	6.597234394	6.253672202
6.164625879	6.435093051	6.231976475	6.604509566	6.121104576	6.195084242
8.918202434	9.467500556	8.985821889	10.12799521	9.287277576	9.161011869
7.566919707	7.008962394	6.92644801	6.800782586	6.584154061	7.135222657
8.056301667	8.405355091	8.959665061	9.230770667	8.665646899	9.071813909
8.236217485	7.557551899	8.282073859	8.247731232	7.568924192	7.683943101
7.617890202	7.020179424	7.763846354	7.372238061	7.400015081	8.086953081
7.386763141	7.477241869	7.601304687	6.957146182	7.374297343	6.819920253
7.788532485	7.193876586	7.437975364	7.122697263	7.245884828	7.239727717
8.630877737	8.880853535	9.056062667	8.618800343	9.186096828	8.812517081
8.576430182	8.12949599	9.002847848	8.239938859	8.887684293	8.294235768
13.30795266	13.50411017	13.52334642	14.08841453	13.67554111	14.05136004
9.739859192	10.19136651	9.406122899	9.875361242	9.455711606	10.28005063
12.62807191	12.87421343	12.04160066	12.47761486	12.27101	12.77841715
10.24942024	10.06255975	10.22361636	9.810255869	10.17224075	10.3896003
10.54950911	10.10187506	10.97896732	10.0178996	10.85361094	10.49140257
10.85361094	10.9991534	10.56084718	11.16281786	10.83100199	11.11388317
10.63516273	10.71503879	10.56421743	10.56084718	10.65014074	10.85361094
7.679285535	7.489176	8.138156535	7.222702545	7.864535646	7.792587404
9.932690333	9.443424081	10.65634987	9.300367869	10.03983393	10.95849458
9.924448444	10.2026692	9.819688798	9.981718727	10.16642595	10.03701736
7.139090576	6.780968242	7.469040515	6.761583657	7.141989162	7.418614091
9.968215758	9.637663091	9.571117879	9.486974667	9.618955576	9.880317061
11.79405389	10.82153564	11.81439214	10.58117749	11.3403052	11.10460602
7.46750399	7.482605354	6.099109354	6.431116323	6.31184304	6.392742677
8.848914293	8.769354545	8.874692596	8.381754576	8.728878424	8.491049758
7.976858394	7.744777657	7.838908253	7.326942212	7.911995121	8.004282899
8.581169111	9.193130899	8.200163707	9.450568111	8.747331152	8.993505475
10.91766747	11.68243664	10.76857843	12.27773509	11.43938222	10.93935201
13.94443	13.85817003	14.01682491	13.83772389	14.05855866	13.80704919
8.089360869	8.638952889	8.220625586	8.284505182	8.971160737	8.260520646
17.12376032	17.07010739	16.7804214	16.82803465	17.12376032	17.12376032
6.781434051	6.560645212	6.997949788	6.354564141	7.01152302	6.644015919
7.337670899	7.244122677	6.817526586	7.62575301	6.974926293	7.041650374
11.32376318	10.4549623	11.77802923	10.86447048	11.68038318	10.03026493
7.357456071	7.239398515	7.514494576	7.246997929	7.508036465	7.181699919
7.404985061	7.472500828	7.314926455	7.375117525	7.186327495	7.609879768
7.170797768	7.096955818	7.107390485	6.847553121	6.619525869	7.124025636
10.6240143	10.25038968	10.53247367	10.45996909	10.35699559	10.52247938
8.299695162	8.372772768	8.34368697	8.03601997	8.216823323	8.371229848
10.67190595	10.81034167	10.156858	10.19957169	10.42736719	10.83930015
8.057421596	7.771143222	8.262596626	6.67667401	7.074173121	8.799348343
7.729367111	7.743466333	7.454260455	7.681826051	7.515186091	7.698928879
8.284505182	8.675348646	8.48742196	8.555833323	8.508189222	8.638952889
9.413942061	10.29432836	9.593075545	10.05053825	9.987874111	9.789981192
9.122783717	9.067856838	9.378583414	8.908475283	9.053043889	8.858692343
7.147555404	6.992656859	7.607714535	6.886441253	7.474397404	7.471219899
9.793126455	9.180285111	10.30810853	9.02886299	9.431415586	9.21067496
13.26910509	13.0147344	13.29684189	12.70390423	13.40302459	13.03496614

7.316406697	6.859461465	7.64030596	7.194186434	7.314025869	7.970233808
9.266188263	8.93638097	9.503271071	8.80133401	8.804477505	9.113305192
7.32338401	6.885064545	6.747074889	6.759969273	7.28497204	6.881279879
9.457741556	8.234232515	9.561667293	9.087255717	9.19736601	9.048620525
7.252038162	8.053207788	7.273224596	6.977525061	7.609232899	7.310197869
7.843077242	8.816214848	8.073652495	8.323157747	8.774537848	7.776629576
8.747892899	8.534897646	8.276014121	8.727528626	8.426780919	9.122783717
6.285720283	6.452957253	7.456429202	6.35978	6.77992704	7.590558081
6.989443677	7.377557566	6.857502071	7.039678949	7.374563525	7.002421121
8.27903296	8.755916071	8.119679141	8.762742172	8.248986687	8.290353162
7.276277889	7.760648222	7.502729737	7.928832091	7.269892283	7.689906808
8.601386131	8.08112003	8.703733141	7.920877394	8.905887879	8.479185606
9.625623061	9.214111545	9.826032111	8.711304737	9.576740677	9.128533354
9.408173051	9.36748	9.348136535	9.983540212	9.149195859	9.499247869
12.50706636	12.93862501	12.09497108	13.07947925	12.55983323	12.56315114
9.000069889	9.419654929	8.906407303	9.838413929	9.366909111	9.058356636
9.721958051	9.636929838	9.574050242	9.363823202	9.243679384	9.521972354
10.28406896	10.0841781	10.88546605	9.766921414	10.49713487	10.27035429
10.36903954	10.89755671	10.58935103	11.41605585	10.68338253	10.61133106
7.738963899	7.274379929	7.90869301	7.271131505	8.027802949	7.063222061
7.599486586	7.272331859	7.848096879	7.089371313	7.328995606	7.911995121
10.34311059	10.63014735	10.54190997	10.81664077	11.01531103	10.74305691
6.894391313	6.936097616	6.890761263	6.638128919	7.186679828	7.027744404
5.997184121	6.223694404	5.950718343	6.020522545	6.447481424	5.873648667
6.334799414	6.543419131	6.323721596	6.464934909	7.069787606	6.237000141
8.049757475	7.600615697	7.202978657	7.090449141	7.578291899	7.809338131
9.407554697	9.69725304	9.022478232	9.314493212	9.665316566	9.658049545
8.065602475	8.377090384	8.199709495	8.438712657	8.210602626	7.761767192
6.688467242	6.731476051	6.616628394	7.465036929	6.771643182	6.73803103
8.423588242	8.443547162	8.206690121	8.899254879	8.047933768	8.23581501
7.211210626	7.604463141	6.972885929	6.923947081	7.335553323	6.897441
13.76108056	13.80704919	13.81266894	13.87263802	13.80033164	14.11997744
8.602379626	7.916025434	8.954176293	8.094218354	8.409136758	7.974325596
9.115691495	8.530767747	9.435722162	8.255317818	9.169326323	9.461673394
10.00186072	10.0719806	9.830354566	9.672623929	9.472607081	9.820433545
6.888392616	7.850535525	7.409335747	8.079553929	7.956615091	7.809644101
7.734896879	7.787863889	7.927113394	7.446760616	8.113343889	8.491898242
9.484840202	9.582761798	8.534897646	9.145078374	8.930963202	9.821914929
11.20345844	11.06877356	11.25523353	11.22600179	11.38025327	10.75987116
11.33292491	10.77387872	11.19966733	9.206093556	11.38970236	11.03452834
9.949411172	10.00186072	9.400327152	10.17336172	9.750460616	9.515743253
11.61117883	12.10135206	11.42371373	12.4571909	11.78498903	11.75662781
7.391342576	7.345977091	7.488894061	7.276635293	7.135797152	7.215185141
7.005077596	6.818463323	7.31523196	6.749332859	6.977525061	7.242080394
10.14131493	9.721958051	10.5029511	9.760900556	10.24414187	9.899969596
7.314300525	7.573484121	6.899979889	7.363924808	7.074397545	7.223856
8.664254273	8.875206121	8.640338566	9.11093099	8.773988747	8.425403333
9.772250768	9.913634949	9.469429253	10.06904603	9.801802283	9.566253768
7.633560879	7.865549818	8.018723424	8.475612051	7.938205586	7.643944697
14.33555643	13.89331098	14.5413574	13.69473258	14.4427031	14.14208591
8.529888505	8.226165	8.483326677	8.256076333	8.381309202	8.348145404
10.63014735	9.91449603	10.72246175	9.905108616	10.45754412	10.37686258
8.505287909	8.294640202	8.771856424	8.041000495	8.327767232	7.960192616
10.41426339	10.46766096	10.58347296	10.5152666	10.35591393	10.73517618
6.42554104	6.748092424	6.623383182	7.062959768	6.860671293	6.880211293
9.591133828	9.482964828	9.551451798	10.28795108	9.267383556	9.018802576
13.78737352	13.20536123	13.64789782	12.4956701	13.7211242	13.62784329
8.404822414	8.281588828	8.31049196	8.006153172	8.574218515	8.396125929

9.445197818	10.19528987	9.94767803	11.28207759	9.496413788	9.398445515
6.961028939	7.705814323	7.279410566	7.827504414	7.744142424	7.389236737
15.36066179	15.39581235	15.06622261	15.97330865	15.18392271	15.16803204
10.45150222	10.77103399	10.31289083	11.07956266	10.72246175	10.58812248
8.48742196	8.588536172	8.398231626	8.767368071	8.667554273	8.641223283
7.286117455	7.577325354	7.081067566	7.722652869	7.372238061	7.478449101
7.048124798	6.569345798	7.330546788	6.596206051	6.916181	6.897750404
13.15082863	12.8652792	13.14611959	12.77454263	12.71520416	13.22730043
6.982909788	7.834787162	7.140269465	7.302140758	8.005065495	7.406826051
7.056197182	6.775501273	6.827921778	6.617782121	7.403718889	7.294038515
6.784076586	7.125696242	7.002156515	7.019921606	7.422224808	7.670568384
6.490215273	7.233346848	6.526793081	6.799617657	7.221024394	6.622442465
11.53050203	11.53677901	11.38577951	11.32027501	11.5612151	11.28731295
7.911629535	7.910617626	7.711523556	7.695611414	7.724369343	7.790916071
8.677215515	8.169730152	9.21067496	7.918472515	8.868452172	8.063461202
7.612584303	6.931501667	7.735932323	7.192412434	7.718747404	7.567065616
9.607593848	9.587719636	9.979092404	9.694213162	9.718307192	9.261851101
10.66229262	10.81664077	10.80499832	10.86963124	10.37894268	10.93646713
6.485740606	6.569841374	6.874988444	6.346660364	7.08160301	6.768172747
8.627796121	8.69335002	9.805520727	10.10278369	8.85049999	8.544134919
9.613206141	8.742149222	9.956562586	8.527198758	9.613206141	9.942152202
7.561342071	7.474688838	7.442598384	6.94531798	7.840676768	7.797125465
10.17702971	10.15379546	9.912769303	10.42062148	9.760900556	10.14713571
7.542431869	7.742830394	7.973227556	8.32856404	7.850890354	8.726024485
15.05177986	14.45281427	14.67552064	13.27940818	14.78379092	14.78379092
7.883917657	7.118793697	8.205892515	7.160551111	7.546592828	6.74683296
10.06997663	10.13768673	9.713188212	9.658049545	10.30058205	9.402814081
8.203586121	8.881793333	7.679634566	8.933180404	8.904793061	8.385303111
6.941216071	6.94072599	7.328411747	7.234805667	6.650783061	6.506975192
12.37087917	12.47130519	11.7349036	12.44264271	11.81018832	12.34237091
11.04464523	11.28731295	11.24147284	11.66189432	10.87907816	10.92608881
10.50057506	10.17613521	10.30695028	10.42175216	10.06997663	10.22448985
9.518503263	9.870931818	9.394567081	9.042944374	9.491725303	8.209418434
6.845030273	7.000099141	7.143674071	7.136586475	6.695470869	7.608041616
9.061091758	8.928157121	9.049629657	9.015966535	8.792768747	9.016491202
7.377269152	7.246997929	7.484854374	6.903241343	7.580904889	7.505759091
6.696473434	6.490215273	7.000584848	6.587577626	6.668064172	6.861931182
10.88031852	10.37894268	11.4481816	10.37026232	11.07956266	10.72633412
8.981479273	8.149788848	8.971160737	8.417196576	8.682574626	8.238272808
8.228946717	8.838495424	8.931557869	9.07419996	8.487847515	8.16147404
9.244930505	9.242526283	9.999132495	9.173615515	10.06356315	9.449948384
9.014846778	8.752912545	8.343220798	8.811517808	8.493155899	8.135850465
11.32721575	11.47871073	11.16734497	11.6157426	11.45002291	11.35475345
6.786032343	6.676440081	7.553773697	7.398977253	7.606370131	6.773917293
12.04160066	12.36759935	11.49239347	13.40302459	12.39660011	12.09225922
11.671419	11.39772243	11.65918316	11.36793803	11.48061491	11.56291631
10.76857843	10.86173253	10.62142174	11.44319437	10.41426339	10.65987528
8.241600556	8.426780919	8.023866626	7.97471396	8.569735879	8.504327141
7.127106434	6.489542818	6.224422343	6.769912384	6.735177838	6.867131162
9.830354566	9.337862091	10.20748734	9.569063343	10.02362526	9.889177586
7.466652071	7.741148566	7.69185196	8.007640657	7.936282586	7.304125475
9.616741535	8.992473788	9.377330121	9.102676354	9.078331919	9.741357455
16.52993947	16.36806566	16.49551516	16.4534382	16.33688632	15.74256723
12.70787607	13.29117716	12.71830686	13.26910509	13.01016522	12.57107442
9.114524374	9.500664495	9.542425758	10.71279176	9.045168101	9.681142253
6.668735424	6.665912939	7.174922929	7.39712799	7.266813283	6.849342131
6.513659232	6.550314424	6.465574545	6.609208404	6.409677646	6.428820091
7.978709162	7.314616131	8.462236515	7.159715333	8.014589606	8.094989869

8.390114414	7.611680859	8.59026096	7.891531071	8.340252101	7.191229121
8.989142232	8.256833364	9.710962273	8.049757475	8.35752198	8.738283333
8.547862646	8.367840636	8.821324343	8.252372808	8.702074263	8.41598997
12.71520416	11.97660652	12.31719428	11.5612151	12.26775966	11.32721575
8.543234566	7.448962859	8.537544354	7.682588111	8.575095707	7.182269455
8.024267525	7.829325051	8.368326869	7.66151798	8.208296687	8.248566778
6.222974525	7.158855657	6.695959364	6.692548323	7.896955242	6.309863182
5.952893525	6.186180192	6.128775343	6.075380768	6.307933879	6.200189737
8.430534091	7.923778798	8.349393212	8.517695303	7.981709394	8.376704939
8.976374343	9.135096212	8.926362727	9.004940778	9.212349869	9.068490586
8.840590677	8.710842768	8.997224192	8.733447455	8.996667384	9.406122899
6.752477677	7.119927869	6.706206909	6.789286121	6.758676202	6.890996051
6.251390545	6.511476192	6.496571788	7.323124384	6.669224869	6.338442758
8.175968162	7.389558374	8.696739152	7.558170465	7.946933485	8.303793737
6.116267859	6.014458293	6.277726788	6.164144475	6.271799455	6.121456293
11.20840189	11.1276132	11.74935785	10.34514895	10.56306707	11.48459908
9.176146596	9.771430818	9.263233333	10.23066525	8.967895455	8.952561111
13.66158872	12.91656586	13.75520609	13.16824615	13.63435472	11.90384839
8.326518101	8.420387707	8.06745399	8.745043505	8.651452677	8.924201657
9.346206162	9.469429253	9.70798304	9.238221677	9.543825273	9.109760465
8.798247798	9.277992232	9.109760465	9.381544081	8.778623323	8.701109788
8.651919778	8.79576798	8.82789604	9.291393586	8.876209859	8.578393657
6.423272566	6.486382556	6.349206475	6.775232444	6.027790576	6.173797535
7.344013535	7.212383152	7.050347636	7.84419497	7.814093232	7.125146293
7.491977768	7.398696909	7.616552222	7.25688896	7.738299929	7.624419899
8.338686465	8.354947869	7.902575141	8.196571586	8.041792596	8.498949848
7.219623727	5.960922172	5.950149222	6.940977263	7.053026414	7.240600646
9.430738808	9.591816061	9.693515768	9.998242414	9.599465606	9.473222667
8.719668061	8.551751717	8.079179394	8.434412929	8.623178879	8.293769586
10.19418334	10.14631732	9.390110384	9.698181576	10.05880999	10.33696433
7.309336424	6.880211293	7.770089636	7.072618697	7.18283101	7.363924808
8.257253131	9.631972414	11.86125412	9.70034896	9.109120374	9.248396293
8.992957212	9.543825273	8.481851586	9.854472677	9.445852485	9.103811545
13.5360606	12.99565877	13.40990013	12.51424883	13.00581854	12.02811467
7.11962903	6.980785162	7.718083212	6.855446939	7.276635293	6.882010616
13.09977851	13.03025597	12.797444	11.58005536	12.72610186	12.23191382
8.880853535	8.600356081	9.165150051	8.802954374	9.207219232	8.583996586
8.577417788	8.286610323	8.630877737	8.245649515	8.438712657	7.893940788
7.712540141	7.347734202	7.720869455	7.471219899	7.667649192	7.060219081
11.13713447	10.64379219	11.50261564	10.54078574	11.4090051	10.85513517
6.922161646	6.653596384	7.08136002	6.583688333	6.767482869	6.969691626
9.066605263	8.185943182	9.474605101	8.092691677	9.054276	9.399129424
6.879943566	6.655235768	7.30728296	6.523815232	7.037511364	7.496977949
8.922082808	9.258612929	8.734806657	8.958493495	8.194244232	8.982611727
9.396536798	8.592622071	9.267383556	8.593558293	9.445197818	9.341818545
8.367840636	7.831854586	8.883993253	7.777311636	8.209054152	8.24281297
13.50997822	14.25895571	13.25686564	14.59173737	13.12562627	13.80033164
10.11452638	10.51771241	9.524763869	11.01209098	10.07956055	10.00262223
6.458058747	6.583688333	6.754699697	7.447389475	6.60058796	6.741460808
12.57107442	13.20018245	12.3843369	13.5160991	12.7494647	12.99565877
8.412956141	8.076118121	7.531770848	7.772503354	8.094218354	8.275152535
7.627670859	7.262118616	8.027386222	7.157499455	7.711178283	7.80651396
7.626110576	7.522209596	7.636533838	7.582966818	7.610260323	7.132177788
9.158666485	8.361756343	9.53973704	8.364284556	9.052474394	9.352487919
7.486088616	7.624016909	7.648986848	7.428605818	7.787863889	7.626470505
9.965319232	10.22557955	9.368224626	10.156858	9.408173051	10.15186663
6.487487818	6.513659232	6.772514131	6.678083162	6.738245586	6.55365803
8.013857071	7.455822202	8.002717273	6.955646677	7.895278253	6.976489687

11.05191968	10.92485902	10.97476091	11.59212007	10.55726192	10.83223099
14.50017253	14.36999747	14.64328928	14.74028573	14.60195877	14.36070414
8.816703535	8.665165081	9.423636879	9.229082869	9.286110869	9.069043081
6.67377398	7.305287707	8.084674061	7.745292258	7.143149899	7.438935576
7.760648222	7.984708455	7.521885657	7.676257364	7.590248525	7.481001515
8.19621002	8.565878434	8.222994222	8.176360121	8.079553929	8.229734556
7.127365253	6.888155525	7.479119	7.085333222	6.901978929	6.735177838
7.896955242	7.578628212	8.418959303	7.508747081	8.082337909	8.316969364
6.96545202	6.522209742	7.340147596	6.546653242	6.725273333	7.086390323
7.174439091	7.189710394	7.841061444	7.850180343	7.635576919	7.208594677
10.47941256	10.04511943	10.29324401	10.14131493	10.04776272	10.41123643
8.195405505	8.258465414	7.430781919	8.078389566	7.452109758	7.390471152
8.389653889	8.312729141	8.316081222	8.427236737	8.480536717	8.71185199
7.491021444	7.075225141	7.912365798	7.02934998	7.734550869	6.97809596
6.992656859	7.135222657	7.559118222	7.576687677	7.335878859	6.546653242
12.89886333	13.17778072	12.7939548	12.94821125	12.70390423	13.04381444
11.35080354	11.36793803	11.62739451	11.38970236	11.45176908	11.36245739
9.699589758	9.837575172	9.682548525	9.892629697	9.788274313	9.482360616
11.84736958	11.47682743	12.19086998	11.60266156	12.19727311	11.4535969
7.557551899	7.152088687	7.588284717	6.97260104	7.251769333	7.729749768
6.804033586	7.293153485	6.885064545	6.792041707	7.902575141	6.570518374
7.490430596	7.420173404	7.876807081	7.585815263	7.911629535	8.150096222
6.14156601	6.150782192	6.16785301	6.264079364	6.618022394	6.154817253
13.29684189	12.42233701	13.66784462	12.07810634	13.14093405	12.8293171
8.204025576	8.510894717	8.32939098	8.482852263	8.394903626	8.163053768
5.754422949	6.045455434	5.853061354	5.805896495	6.01168199	5.764000515
9.647013263	9.602761333	9.59387599	9.546645747	9.454390232	9.649399939
6.034468727	6.046443465	6.326506525	5.952164414	6.318902889	6.701095535
6.827413455	6.33587599	7.204880737	6.459603455	6.871158202	6.94072599
11.59212007	11.71659127	11.33848921	12.25251148	11.54470354	11.55524693
7.626793707	7.669946162	7.646275747	7.65951298	7.278747747	7.653651778
8.08193004	9.45706402	8.578858313	10.08893073	7.848096879	8.865915939
7.584965596	7.932792414	7.405823253	8.131433848	7.869033162	7.283275424
6.258005697	6.233249556	7.141989162	6.396397444	6.133495202	6.392953667
8.598479657	8.78214798	8.586230848	8.840010495	8.716387192	8.435650677
16.2036826	16.25437967	16.28343863	16.3077943	16.17943014	16.12384164
11.87175588	12.34237091	12.19996238	12.20640951	11.93178106	12.06397065
9.35188598	9.70574997	9.17294002	9.739859192	9.177504333	9.477181455
9.397189192	9.312727606	8.902484929	9.178084667	9.292435667	9.492465394
6.012078172	6.168988222	6.176996141	6.386235697	6.366139859	6.060471061
10.37573078	10.05688349	10.02923217	10.15483313	9.797856576	10.62536143
8.443547162	8.27861104	9.315088131	7.947667091	8.754441596	8.300103657
6.710494808	6.31869002	7.077389495	6.423272566	6.603158687	6.896938141
6.435745475	6.66784103	6.18347903	6.327350374	6.256172131	6.526092091
8.046104414	8.289996192	8.469339576	8.433939838	8.10683704	8.095746162
8.686126354	9.789981192	9.186096828	9.679062616	9.572453101	9.320176919
7.224144889	7.18112796	8.026271202	7.439837192	7.244714616	7.325732596
9.023639636	9.891739697	8.955359253	8.925792808	9.142791657	8.833337414
11.12613667	11.53050203	11.32559397	11.77305263	10.92190902	10.69574272
7.923778798	9.293710889	8.698751323	8.08737399	8.307093606	8.82789604
9.048100778	9.296718121	9.32739897	8.348145404	8.707515232	9.192533242
11.54675633	12.57107442	11.83072395	11.16579624	11.70557621	11.30131952
11.30131952	12.37416009	11.66708917	10.95683616	12.89886333	11.32376318
13.85099976	14.70594079	13.91489381	14.77222727	14.11997744	14.04335209
7.641344242	6.441470343	7.361082222	6.229681899	7.688370495	6.79370899
8.35372597	8.341977727	7.744777657	7.960941889	8.115437899	8.399553939
8.126700889	8.098302202	8.217984283	7.874063444	7.787223354	8.200539131
12.06692054	12.11841559	11.5612151	11.60888892	11.90152869	12.22341713

11.00620373	10.52247938	11.00902763	9.736139061	10.93023415	10.86305205
9.463745455	8.82842597	9.436898869	9.101613848	8.990775909	9.133284677
7.077389495	6.691558677	7.321250939	6.954672071	7.220240556	6.914008404
9.201355202	9.244399414	9.533033172	9.227886646	10.03385507	9.356741384
8.480536717	8.03364202	7.983548525	7.313451111	8.593558293	7.317590788
7.456765576	7.28497204	7.18511501	7.938205586	7.481001515	7.604463141
7.850180343	8.020484273	8.006153172	7.646953505	7.808339242	7.984288364
6.510269939	6.853963192	6.451016717	6.399871131	6.598705667	6.328723111
7.924172566	7.949071818	8.358783152	7.702676091	8.155023263	7.748548515
10.73911267	11.11230451	11.17571904	11.11856721	10.59685822	10.67305197
9.954681949	9.503271071	10.45628541	9.995698596	9.821195081	10.18762619
8.484723788	8.276867707	8.629474212	7.943134	8.803935202	8.644074768
12.03310273	11.33292491	11.69440164	10.93023415	11.65261158	10.66465811
8.318250424	8.563501616	8.687541707	8.37004801	8.263866778	8.027802949
7.206091434	7.798177384	7.445746576	7.40526796	7.266003808	7.11489098
11.46936418	10.10841911	10.37573078	9.630502939	10.52799495	9.054860525
8.232591606	9.053706202	8.574218515	8.63568099	8.370830747	8.207926798
7.787223354	7.942071303	8.014979636	7.571747222	7.761029182	7.869709071
8.185943182	7.762451808	7.997183333	7.391342576	7.797125465	7.603389051
10.49830212	10.29324401	10.27905981	10.41957053	10.50166798	10.39641485
8.308331848	8.144161414	8.334981101	7.801388869	8.581169111	8.226840717
8.129067798	8.838951081	9.066605263	8.937429899	8.541972566	8.356302828
7.758192424	7.447389475	7.467207283	7.962004212	7.346567394	7.801388869
8.468064667	7.959080606	9.037616626	7.759566061	8.57971698	8.519178475
9.828404283	9.612498475	10.06255975	9.266188263	9.886762101	9.835081424
10.50914282	10.24942024	10.20532801	10.3090972	10.23452156	10.60383217
9.746145949	9.684886576	9.461673394	9.469429253	9.651465606	9.84311402
12.39973853	12.0089388	12.04432	12.62807191	12.36759935	12.30789527
10.87907816	10.08700287	10.24139873	10.0728998	10.17440526	10.48185706
9.987874111	9.887613414	9.431415586	9.666877556	9.781348374	10.03611568
12.39348455	12.13244857	12.04990566	12.18492446	11.97944932	12.92937388
9.447991384	8.925792808	9.408173051	8.976374343	9.281446566	9.165150051
9.135096212	8.569209263	9.704210374	8.58807297	9.303342798	9.266188263
9.35796903	9.600888859	9.095762	9.191849071	9.007167758	9.541041677
7.008416879	6.717050677	7.675499444	7.010749455	6.914278354	6.837484717
8.631899566	8.398231626	9.058356636	8.197369283	8.74840203	8.129874121
7.852302727	7.414093131	8.185177828	7.566119051	7.765675424	8.205457293
10.5336638	10.48539779	10.40592026	10.65365596	10.63516273	10.70643744
13.42153085	13.97489728	13.46005067	14.07988992	13.66784462	13.87958112
7.431389424	6.822217707	7.734896879	6.997069465	7.362680232	7.491021444
11.34732253	10.02644328	10.40178901	9.62024901	9.779738	9.614560172
9.152615889	9.211184727	8.739635162	8.901935111	8.914132333	9.060017677
11.71895032	12.2031413	11.50814936	11.92156179	11.87687449	12.02514953
9.273022949	9.06557301	8.809442889	8.800782677	9.167758485	9.008373061
13.83173935	13.55640443	14.58095582	13.12562627	13.84455043	13.74759592
8.183602061	8.548304869	8.008400859	8.226840717	8.170954818	8.276867707
10.92880859	11.39382595	11.16888788	12.38794592	11.18800276	11.34202326
6.424911465	6.471690111	5.934153242	5.805852343	5.773793404	6.579152939
8.368780919	7.641985	7.882850313	7.827906879	7.381950828	7.963792818
9.176817596	8.932594273	9.550769788	8.875206121	9.173615515	9.026117657
7.163287778	6.697417434	7.665314131	6.495692828	7.202436384	7.487940131
9.26122399	9.151431899	9.483552818	9.182731869	9.679645121	8.81966502
13.18348162	13.10985871	12.89886333	13.21058954	12.89513437	13.33155169
10.69222123	10.47479655	10.47479655	11.12315281	11.00053539	10.38738452
7.87816304	7.909763434	8.239938859	7.337989111	7.913871889	7.901142576
14.66395575	14.99939481	14.84412351	15.48449671	14.7172184	14.86987024
10.65500785	10.99623862	10.70299608	11.17571904	10.67428006	10.73264046
10.28506122	9.744663444	10.65014074	9.346206162	10.20358283	9.747577162

9.183367525	9.133918929	8.802954374	8.900779434	9.174120707	9.081281455
7.629673333	7.602282465	8.164575929	7.263531485	7.478150263	7.122942091
6.548235576	6.331316808	6.787827455	6.473192182	6.485102909	6.328925929
8.475612051	8.841575667	8.952561111	9.134591697	8.636577313	8.877626323
9.256085253	9.727935707	9.543825273	9.537497323	10.17702971	8.717867636
9.766921414	10.00985275	10.10736711	10.22448985	9.701165495	9.546645747
7.640998838	7.723687384	7.868007081	7.845981061	7.984708455	7.424024737
11.58005536	12.48835313	11.42178201	12.84197228	11.92665931	11.72807534
11.97196754	12.06098883	12.26475658	11.87912023	12.16627503	11.32559397
6.954672071	7.138578667	7.401155333	7.001612657	7.792587404	6.936612586
8.423139949	8.248566778	9.018802576	8.404396899	8.460863192	9.264989899
9.185497838	9.179819687	8.771294657	9.030619364	9.179226364	9.207219232
8.321919424	8.217270354	9.236429667	8.915814212	8.643157737	8.344860929
9.501306556	10.40278823	10.22761479	9.820433545	10.75062885	8.498503737
8.306613333	8.173308798	8.08537204	8.339926909	8.164575929	8.078044465
7.549557061	7.386471808	7.901917293	7.264798293	7.774939586	7.202978657
7.727390434	7.65951298	6.996042242	7.262957343	7.329578283	7.490096869
7.566919707	7.324608717	7.681826051	6.992079758	7.731871869	7.002659525
8.467667768	8.550897414	9.160413333	8.352944343	8.940148162	8.748883111
10.27714677	10.38430498	10.96748398	10.52585606	10.74172297	10.31894523
6.900957616	6.913266485	7.059676455	6.877148152	6.85895202	6.82385998
8.603745778	8.86024204	8.689808788	8.72650097	8.942313313	8.572701232
7.340147596	7.276913788	7.239990273	7.253230737	7.612294131	6.994245051
7.549884232	7.582654121	7.654256818	7.786927414	7.720869455	7.266262606
10.65871503	9.798623747	11.3403052	9.825199323	10.6240143	11.29230964
7.103815788	7.883536596	7.337386909	7.717096556	6.688196	7.657256192
11.78498903	11.97196754	11.74205097	11.92396137	11.86922315	11.87912023
7.407756293	6.907093253	7.798177384	7.007335798	7.538797101	7.59859104
7.495760485	7.488894061	7.599870798	7.610976808	7.443845071	7.469634172
7.631297323	6.767263616	8.391869283	7.262427071	7.463067838	7.707249808
11.10460602	10.98749259	10.98350754	11.61117883	10.91514001	10.66229262
8.154321646	8.511305758	8.189194404	8.806957455	8.154705535	8.147147636
11.13551505	10.48432644	11.6137143	9.997192202	11.05191968	11.47322198
8.395713444	7.694916828	8.52305899	7.720541949	8.126700889	7.843077242
8.244805758	8.108111747	8.818210192	7.950819283	8.393536232	8.488340929
8.366462404	7.395882727	9.584803364	7.494168788	8.254484434	8.068931465
7.401155333	6.387713778	7.789881242	6.560443758	7.166369152	7.997183333
15.92931651	15.97330865	15.99508933	15.76560628	15.95256028	15.24813401
16.68963031	16.42659037	16.52993947	16.73234105	16.39933956	16.2036826
12.30465105	12.24351077	12.92081703	12.05813886	12.9851923	12.12151457
7.92597801	8.615838	7.732550404	8.354947869	8.168196313	8.064260859
7.279669313	7.571116455	7.537240869	7.522869182	6.880775747	7.163863636
9.366909111	9.304577515	8.956407626	8.852576737	9.229670424	9.142791657
5.974219697	6.244370242	6.375508101	7.535530424	6.410521475	6.653092081
7.841440677	8.066736566	7.62509899	7.728361283	8.162707626	7.708254091
7.589888475	6.697842384	8.157901343	6.866597293	7.482605354	7.866306808
9.095162899	8.977558859	8.649462798	8.887109626	9.070117818	9.531623293
8.514037303	7.865908465	8.361327455	7.923778798	8.289599333	7.649620657
9.473962505	8.614861051	9.654354424	8.394903626	9.521972354	9.544495798
8.509065354	8.194244232	9.548618051	7.971654859	8.64507902	9.098746404
6.355908172	6.013925687	6.577039475	5.997922384	6.574185192	6.376693242
9.056533626	8.391460737	9.46096901	8.443956768	8.820759798	9.303342798
7.301506111	7.67441401	7.604463141	7.877844556	6.382217859	7.932066141
10.0053333	9.968215758	9.836777707	9.816676162	10.43998577	9.203189636
8.501579646	8.790654051	8.345353354	8.511758747	8.838020616	8.432612263
7.439506505	7.51310204	7.867312374	7.831138545	7.668960768	7.706911222
9.21352099	7.391655768	8.517288667	8.842639202	8.989142232	7.893273434
8.596127869	8.942313313	8.573273283	9.35371997	8.515350859	8.747331152

8.272231939	7.901561475	7.219623727	7.731542758	7.755503707	7.726017717
6.917222737	6.878445485	7.211808657	6.840661414	7.140009717	7.093823343
8.547449101	8.594016788	8.761644081	8.565878434	8.611710212	8.752912545
9.599465606	9.829119768	10.09506496	9.941224515	9.980879424	9.452497313
10.10841911	9.811898121	10.57758511	9.576136707	10.21279484	10.5752557
10.43440935	10.08231743	8.473392556	9.159785394	10.39072525	9.50650501
16.90924948	16.14978837	16.14978837	16.12384164	16.73234105	16.61084374
7.127674667	6.716824636	7.609574333	6.709290939	7.384903253	7.353244222
6.072015283	6.447271273	5.981959495	6.018274323	6.785592192	5.838080424
10.31480319	9.613873859	10.06633871	9.972781707	10.27035429	10.30058205
6.567185838	6.496990475	6.730429919	6.35000001	6.684069253	6.555734869
6.870918222	6.975188859	6.982909788	7.367866364	6.675226152	6.951961606
9.348136535	9.310775172	9.055478465	9.242526283	9.616741535	9.731150899
7.595597162	9.116279576	8.019769071	8.806450121	8.047553323	7.654256818
6.972885929	6.662284414	7.153495505	6.956131545	7.188870657	6.265724232
6.891529131	6.576096444	7.454572909	6.577737222	7.174922929	7.058479485
11.31053596	10.85654613	11.44319437	10.55602586	11.38391359	10.77762269
6.580361485	6.411488202	6.891782758	6.16631502	6.518976111	6.674729293
16.06827159	16.06827159	16.06827159	15.67809237	16.04217659	15.48449671
8.748883111	8.245649515	9.451851384	8.329784131	8.782655051	8.849981545
9.769223525	10.14809211	9.576136707	9.521192909	9.988725727	9.443424081
10.44934262	10.24414187	10.49009312	10.4322159	9.895856061	10.36349344
14.89443037	15.05177986	15.19830883	14.9847254	14.83117585	14.9727616
8.707057788	8.434412929	8.655934636	8.631899566	8.742635939	8.871686232
9.426842081	9.585510535	9.661659101	9.675185677	9.533596364	9.39974104
10.91356479	10.79420974	11.12151875	10.59418919	10.84302834	10.78705857
7.972411364	7.90505396	8.197805768	7.755825222	8.102484232	7.671593667
6.797589061	6.182415283	6.625965465	7.026621727	6.595312657	6.912541192
13.56234785	13.69473258	13.90036211	13.86604428	13.66158872	13.64083669
7.224435778	7.192117626	7.590558081	7.199149121	7.219933525	7.372842667
9.810255869	10.20964764	9.842388081	10.20532801	10.20452777	9.727055768
8.074096707	8.262181081	7.833640626	8.181574081	7.93027604	8.06745399
7.491688212	7.706567859	7.239398515	7.56574702	7.310197869	7.736619182
8.287888051	9.741357455	8.257253131	8.668931657	9.188424626	8.55725397
7.939903758	8.101780545	7.70299303	8.135850465	7.988582505	8.058592364
7.214315212	6.853717071	8.150448232	6.892837172	7.143674071	7.112620848
15.54332231	15.38120792	15.76560628	15.31193978	15.52328152	15.61926687
11.81887725	11.74439188	11.62494396	11.65052704	11.63401423	11.78498903
8.680513909	8.899757485	8.553132192	8.192266657	8.560860505	8.80133401
7.335252788	7.66151798	7.307022919	7.762120596	7.430781919	7.235967596
9.246644323	9.018299374	9.703591263	8.448008778	9.29794999	9.033394545
7.772503354	6.944336869	8.163053768	7.069444	8.207537162	6.888392616
13.23341018	12.94821125	13.99495772	13.30795266	13.85817003	13.76108056
7.637262101	7.65457904	7.699362081	7.637927172	7.568310444	7.663284545
11.68243664	12.14789109	11.73070023	11.22294817	12.41931404	11.63401423
11.12454893	11.41238356	11.20345844	10.77103399	11.49420288	10.47479655
11.37131374	11.38577951	10.94942311	11.83072395	11.9982543	10.47242114
10.28710845	10.36687712	10.61992902	9.624391323	11.76588718	10.42297323
9.334658071	9.176146596	10.00186072	9.482964828	10.12799521	9.555296242
7.478150263	6.280830838	7.919201576	7.217862778	7.848409778	6.582439566
8.371229848	8.160266646	8.414679525	8.082337909	7.909031556	8.297224525
9.748306121	10.01071956	9.261851101	9.70798304	9.706432808	9.916104475
11.308834	11.01531103	10.72516317	10.99186298	10.60863639	11.38970236
6.925244263	6.304587333	7.080799364	6.281598899	6.928572354	7.144029273
7.143440162	6.971796172	7.431389424	6.783136778	7.759260374	7.314300525
15.52328152	15.99508933	15.449005	16.17943014	15.50564301	15.78787594
13.74093847	14.15092087	13.72760196	14.3093669	13.64789782	13.9071606
9.890949131	9.982665242	9.488337717	9.641787333	9.891739697	9.763138212

13.3718573	12.39973853	13.61616406	11.911688	12.93862501	12.82558839
9.448606737	8.955880859	9.859664869	9.053043889	9.841562192	7.530907465
7.524103909	7.688370495	7.006478434	8.035675313	7.507037566	7.74678301
6.226606111	6.006351737	6.498091727	6.098658566	6.039096606	6.351287939
8.184050869	8.015388182	8.363862202	7.806122253	8.428657566	8.323598384
10.14432724	10.21157382	9.909202788	10.30358984	10.21484784	10.02923217
15.99508933	15.27762024	16.2036826	15.26374094	16.3077943	15.34617167
7.451812333	8.006915303	7.592689374	6.665912939	7.610574101	7.560401535
16.64557528	17.67797782	16.8710039	15.10875812	17.43906183	17.67797782
15.41210544	15.50564301	16.22759729	15.03840752	16.06827159	14.59173737
8.016751283	8.324880909	7.783465061	8.307093606	8.004655889	8.148682091
16.82803465	16.73234105	16.4534382	16.36806566	16.7804214	17.29704085
9.029402949	6.444123263	10.59062542	8.968910758	11.48840455	10.01703805
10.17224075	9.688441414	10.30358984	9.687048303	9.862946677	9.804037697
7.566450929	7.477877475	7.151031394	7.389236737	7.589252838	7.511581475
7.962680434	8.247731232	7.512168657	7.891912283	7.994566778	7.648319737
8.013081051	8.326162172	8.110881556	8.607126586	8.330618758	8.749910828
7.632188616	7.101832475	8.415109929	7.056486293	7.603101596	8.068200283
11.09089144	11.28896545	11.38775416	11.53239215	11.03452834	10.70775781
14.3093669	14.84412351	14.36070414	14.7528858	14.40789433	14.58095582
17.86179439	17.86179439	17.86179439	17.86179439	17.86179439	17.86179439
17.58093558	17.36803328	17.58093558	17.36803328	17.36803328	16.7804214
11.82616001	11.48459908	12.0838996	11.17041501	12.13867659	12.37087917
8.47107903	8.399975414	8.583542788	8.737817051	8.342773172	8.50618604
7.627030141	7.792995667	7.935875313	7.746135697	7.584965596	8.144908747
7.041369354	7.032569455	7.002659525	7.057011424	7.018881949	7.065949717
9.652939283	9.278597747	9.731806091	9.424985495	9.132651727	9.50710198
9.908305848	10.88546605	9.257324	10.44832821	9.877792838	10.04421951
10.65246846	10.12408141	11.18636156	10.05523038	10.61621963	10.26445444
7.037511364	6.537516434	7.121055596	6.556698131	6.685084394	6.991264788
11.28370047	12.41931404	11.27695531	12.11216258	12.42943771	11.83072395
10.98492733	11.27695531	10.56655729	11.15808934	10.16743096	11.62739451
6.61398898	6.945903495	7.100441616	7.187213162	7.416641556	7.044969556
8.754900343	8.584413131	8.198176101	8.445759657	8.343220798	8.472435657
9.921211657	9.078331919	10.27139209	9.913634949	10.25127201	9.854472677
7.441392919	6.656456051	7.608647091	6.798855303	7.403028737	6.344188131
7.700670768	7.871272152	7.296277414	7.533663232	7.858214495	7.97753898
7.881102323	8.386601707	9.546645747	8.316081222	7.996041859	7.535530424
12.00354228	11.70812672	12.28105924	10.87907816	12.15440913	11.88649013
17.49517975	17.43906183	17.49517975	17.58093558	17.58093558	17.58093558
9.553430788	9.678297182	9.362770111	9.873591818	9.620873889	9.635582141
8.35018502	8.136641818	8.312729141	7.95565902	7.998587576	8.297630758
9.864614818	10.58935103	9.982665242	10.08325108	11.17571904	9.535633242
7.611975737	8.324453253	7.387965737	7.392218323	6.754943232	7.31258798
8.848410616	8.22479296	9.26122399	7.455517596	8.812517081	8.775519707
8.659649677	8.793874616	8.720163182	8.741130788	8.882882525	8.856895172
7.713612677	7.323994646	7.845981061	6.662035182	7.637262101	8.561789232
9.393335687	9.645545465	8.978723121	9.30770201	9.176146596	9.110297333
7.527339687	7.74678301	7.464355384	7.844877657	7.511838192	7.587005707
11.80104424	11.68038318	11.35657003	11.565007	11.69440164	11.92396137
6.529766576	6.85163704	6.389892586	7.08589403	6.59504903	6.651250333
8.818695101	9.024707707	8.478263182	8.59026096	8.996148737	8.831800202
8.914132333	8.705608919	8.972363838	8.896578162	8.976374343	8.916479111
7.803554182	8.085771838	9.489614919	9.494490616	9.524156586	7.995268899
9.210065	9.820433545	8.979921919	9.252443646	9.237064687	8.947127939
12.06098883	12.17829278	11.84291489	11.65918316	11.30301738	11.84529477
8.745043505	8.578393657	8.751405818	8.960218374	8.380307172	8.699656879
8.045324071	8.003479939	8.266842545	7.98285402	7.404680798	7.880750535

11.35278885	11.4481816	11.48061491	11.99323436	11.73972102	11.26990549
9.548028869	9.627013505	9.481668929	9.312727606	10.00094823	9.584803364
10.62895885	10.39521192	10.6488688	10.14895288	11.08264222	10.34514895
8.717364515	8.960773354	8.995584808	9.17294002	9.238865747	8.39867397
8.932093424	9.466909636	9.188424626	8.644074768	9.027136657	8.605125889
10.11849919	10.58117749	10.27432009	9.805520727	10.23066525	9.825199323
8.606099333	9.370832535	9.110297333	8.422643323	8.676316919	8.667049253
12.45390404	13.06488572	12.72972704	13.42153085	12.86955734	12.92081703
9.451212687	9.711723394	9.397189192	9.615324495	9.332175414	9.089024899
12.34237091	12.7939548	12.16279806	12.92542832	12.17216641	12.43944324
8.31912796	8.824381182	8.288815495	8.772410586	8.455403657	8.191909586
12.36759935	12.74093701	12.01427079	12.93430142	12.2293359	12.59323492
9.978126798	10.37346225	9.91449603	10.57159251	9.86640601	10.19649023
8.133179586	8.375190152	8.411696677	8.455403657	8.021628354	7.783465061
9.716648152	10.13479405	9.30770201	10.07956055	9.380344141	9.430738808
9.116279576	9.336479586	8.361756343	9.307024131	8.730448515	8.85049999
10.80900786	10.57758511	10.63391466	10.54800525	10.39296715	10.16743096
13.74759592	13.58209631	13.54221797	13.33155169	13.54221797	13.45466248
6.536206333	6.552305929	6.545300424	6.544097556	7.453623293	6.706413566
8.315715768	7.90290904	8.428176394	7.796747616	8.148682091	8.300888657
11.54871614	11.40306525	11.35858946	11.21958266	11.27499716	11.19114259
11.28053313	11.55106122	11.40725584	12.10414281	11.72807534	11.23116994
11.38577951	11.53473316	11.02551486	11.53050203	11.07647857	11.19638858
12.42616911	12.797444	12.30167305	12.90359001	12.32024961	12.61761735
10.29660096	10.3986043	10.573928	10.78856661	10.79284767	10.20748734
14.48025691	14.66395575	14.47046648	14.76275492	14.47046648	14.64328928
10.57282784	10.61480751	10.34514895	10.76609029	10.55842197	10.78269205
8.271795141	8.610337293	8.212685152	8.713915657	8.434412929	9.240082808
9.572453101	9.674270303	10.04340808	9.919491525	9.359783616	9.490338525
13.32021166	14.09521817	13.60269863	14.16629104	13.54965001	13.37822002
14.76275492	15.19830883	15.16803204	15.59877501	14.91973608	15.26374094
9.524156586	9.478383576	9.674270303	9.372025394	9.149684939	9.339764051
8.952561111	9.206093556	9.000069889	8.687969051	8.979298788	9.014846778
10.97054498	10.70902317	10.88809182	10.54692936	10.45375986	10.58236152
13.21058954	12.79024082	13.34730616	12.89513437	12.80949032	12.73352188
7.209785606	6.182576	6.666157333	6.419328293	6.443888202	7.329870525
7.506434626	6.055906899	7.562285172	7.045715273	7.094450071	7.929187444
7.90290904	6.452749343	7.288414848	6.368128081	6.26630199	6.893883192
9.529469889	8.35796399	9.011040293	7.693828071	8.161911788	8.242431778
11.91416468	11.81221198	11.39382595	11.31661678	10.89755671	11.01951417
12.99088515	12.78185519	12.91196246	12.61761735	12.44264271	12.35168631
10.14631732	9.986114121	10.2166926	9.93099102	9.738398616	9.41003803
10.24316974	10.61001127	9.59460096	10.59924244	10.67190595	10.18942995
12.10694646	11.52052132	11.92665931	11.28565422	11.12613667	11.59870577
14.86987024	15.03840752	15.10875812	15.38120792	14.93426708	15.10875812
14.31917195	14.7528858	14.50017253	14.85646221	14.36070414	14.6970922
14.49002723	14.83117585	14.83117585	15.05177986	14.62217632	14.84412351
15.46469304	15.81050193	15.65613201	16.09492117	15.46469304	16.02065153
7.739667424	8.097222414	8.253254586	7.953126222	7.986535808	8.168969465
7.675499444	7.813414727	7.918472515	7.861535657	7.484516838	7.88791003
7.853698798	8.18481599	7.871683444	8.374810828	8.415109929	6.943812717
12.03912361	11.36610866	12.54906863	11.1276132	12.26162026	11.76101506
8.335469222	7.809338131	8.724013404	7.648319737	8.492747404	8.646531515
10.90057618	10.84844593	10.6240143	10.7982674	10.65500785	11.45932673
12.19996238	12.68484185	12.2031413	12.52461444	12.2031413	12.25251148
9.558925838	9.022478232	10.24612484	8.941795242	9.812775374	9.200042253
9.87177298	10.0178996	9.675185677	9.887613414	9.952967535	10.23351878
7.935468505	7.568036061	7.436027273	7.491021444	7.593038414	7.836508475

9.516398909	9.683334444	9.081281455	9.746868596	9.682548525	9.438799404
11.55928848	11.33848921	12.28985985	12.83405786	12.14507673	10.99186298
11.57567047	11.06732533	11.58814301	10.33596201	11.72355631	11.58195332
10.9991534	11.67593314	10.82945836	11.79405389	10.98749259	11.22119175
7.021219939	6.880775747	7.099882485	7.173570899	6.794126838	7.031184939
11.05033578	11.0940021	10.59292728	10.69470446	10.76361324	11.20345844
10.63266161	10.67305197	10.22951336	10.43564423	10.47479655	10.78856661
6.205248909	6.445432434	6.541618313	6.839237455	6.644015919	6.546871707
10.48074239	10.45996909	10.04862183	10.3305428	10.3548828	10.52585606
9.494490616	9.400327152	9.182731869	9.481000434	9.684886576	9.66383
8.314030071	7.728706424	7.799854778	7.905419798	7.967113929	7.977207111
10.25906505	10.16165443	9.634173323	9.926784556	10.12909315	10.06547375
9.980047091	10.02439619	9.647013263	9.938475404	9.850553616	10.08978265
6.939486697	7.202978657	6.830332131	6.975683768	7.056197182	7.067022475
10.74045328	11.2608846	11.46320141	11.26623333	10.90606985	11.77305263
7.995673758	8.13932801	8.214678465	7.882189879	8.674281929	8.213079374
7.873714818	7.462172081	7.306475687	7.621361828	7.298769596	7.630934212
9.609027141	9.513795939	10.63516273	11.79171062	9.326238727	10.49713487
8.972927475	8.927019859	9.156208313	9.509226798	9.04056096	9.083741606
6.306277939	6.438496414	6.815737535	7.694916828	6.087012455	6.676440081
9.033394545	8.651452677	9.147296667	8.425403333	8.418959303	8.83754499
6.83894704	7.063984253	7.057011424	7.571116455	6.718530879	6.995294596
6.525168444	7.064538818	6.793493859	6.516492273	7.742436303	6.622230818
7.436336172	7.115757364	7.54731098	7.090185162	6.69920203	6.548235576
9.584100455	9.465071586	9.858738737	9.419654929	10.24511785	9.76546198
8.048299333	7.633560879	8.806957455	7.530291586	8.159907626	8.495447152
6.874493707	6.794878162	7.179751081	6.72626003	7.037248667	6.760820788
7.856753939	7.631297323	8.02698897	7.497307495	7.949071818	7.948737212
6.266652758	6.811868152	6.228422404	6.809755232	5.954837061	6.649151414
9.899969596	10.17030341	9.562364657	10.05688349	9.816676162	9.722667242
6.961503899	6.706906717	7.019697232	6.887288828	6.825072616	6.934295525
11.76804793	12.02514953	11.41605585	12.34577038	11.75908761	11.94481318
6.555298505	6.287217818	7.249695535	6.400112778	6.60520404	6.621578222
8.597538556	8.773988747	8.395296687	8.773988747	8.605125889	8.886620687
7.195783737	6.92551101	7.494798263	6.709545626	6.813963929	7.532708636
11.15966862	11.02105469	11.87912023	11.91912022	11.36423723	10.89604789
10.56306707	10.56421743	10.47370876	10.70775781	10.74945851	11.67849095
6.364283747	6.290280232	6.399020677	6.571432061	6.338230899	6.848276364
11.24646499	11.1060681	10.93935201	10.56306707	11.06573849	11.68948846
10.04340808	10.46342714	10.08700287	10.96748398	10.13580598	10.22668357
7.955993172	8.360024717	8.002342131	8.656360697	8.295009141	8.151247717
11.47682743	12.10951308	11.70812672	12.50328361	11.49636188	11.58621592
9.390834152	8.981975535	9.963504081	9.365016101	9.627745303	9.468134182
6.685307768	6.60058796	6.934051768	6.630444394	6.876878333	7.074987333
8.692870869	8.630877737	8.768863636	8.23581501	8.840010495	8.02125603
9.462343535	9.757590192	9.057828929	9.779738	9.361671283	9.408173051
9.293710889	9.853774707	9.58708204	9.290152727	9.976273051	8.663769939
7.238133434	7.172125253	7.487647384	7.645943414	7.359577545	8.014589606
11.31449746	11.17378617	10.99623862	11.21187068	11.29076429	11.81887725
10.45996909	10.59685822	10.08893073	10.72246175	10.85225864	10.41022215
10.19236777	9.948421707	9.747577162	9.778131475	9.815893677	9.829119768
7.678591667	6.842273677	8.51269104	6.996042242	7.462491444	7.487078798
6.59250899	6.532834	6.660088081	7.290204495	6.795847303	6.628772899
8.217270354	8.064260859	8.25570701	7.961295828	8.104866828	8.066736566
9.486974667	8.76328402	9.828404283	7.730484909	8.530767747	9.400967465
7.862831293	7.777311636	8.120062485	7.916712838	7.505175253	8.141770949
11.18291631	11.25898056	11.12613667	11.2278827	11.22294817	11.60266156
8.056742909	7.921614596	7.849821828	7.920144808	7.795429838	8.063898495

6.849836323	6.564307798	7.067591	6.564057303	6.697417434	6.307051293
14.5413574	14.5413574	14.11997744	14.79546777	14.14208591	14.4427031
7.233039222	6.756145354	7.896287434	7.158590636	7.424346313	7.470329323
8.282438737	8.273483707	7.799196616	8.214296636	7.818322879	8.417196576
10.58117749	10.30470526	9.962571606	10.12314196	10.22267877	10.49009312
8.223899788	8.070136677	8.030614293	7.893940788	7.786298434	8.049019253
8.836542848	9.160413333	8.457898556	8.519178475	9.003357768	8.673363869
6.858224505	8.592161788	8.044608626	7.486088616	6.903822293	7.704713596
13.87958112	14.1830221	13.96779242	14.80749806	14.1282639	14.30038792
8.615330515	9.192533242	9.246644323	8.690381253	8.448008778	8.576430182
13.98831971	13.81266894	13.87263802	13.63435472	13.97489728	13.39659876
8.53579602	8.196571586	8.784105778	9.376046232	8.429582192	8.547862646
9.925979414	9.885156737	9.553430788	9.737663949	10.00262223	9.884409465
11.06123261	11.59212007	11.43005301	11.70138789	11.31661678	10.94786936
9.094076404	9.493894545	12.38063147	9.059459505	11.15183093	8.669992818
14.1904887	14.3093669	14.36999747	14.63205333	13.99495772	14.26676228
8.052417212	8.414679525	9.037153444	8.276438626	8.151663141	8.419470808
9.208368677	9.899010949	10.91176188	9.531623293	9.443424081	9.010430586
7.326042475	8.030983081	10.06822268	8.347358778	7.849821828	7.348317141
9.200042253	9.404333879	11.14385742	9.574750414	9.471411141	9.463745455
8.488340929	8.790130253	9.141588162	8.902484929	8.58807297	8.460053242
10.46342714	11.65918316	13.09463792	11.84027237	11.04156144	10.4687107
9.786685869	10.69099899	11.1453264	10.82945836	10.01524205	10.27714677
8.57790302	9.485668061	10.69682519	9.523397737	8.864955909	8.462236515
8.190781646	8.668062838	9.143918798	8.719245818	8.334587636	8.481411768
13.5160991	13.98831971	13.57574016	14.06476988	13.56886427	13.66158872
13.41514643	13.86604428	13.94443	14.60195877	13.48401144	13.74093847
12.95303817	13.70143341	13.63435472	14.13521971	13.23864174	13.38447499
11.66189432	12.15752365	12.03602449	12.71520416	11.55928848	11.76336308
17.01900663	16.68963031	17.07010739	16.52993947	16.61084374	16.28343863
6.545751121	6.519217414	6.971547919	6.915952788	6.806762071	6.555084162
8.609952768	9.043522909	8.841037687	9.312041414	8.797743222	8.546056747
6.330318636	6.378748303	6.935824606	7.325732596	6.612991111	6.390557101
12.3613163	12.77015535	12.39973853	13.12023156	12.54268587	12.29275278
9.622300838	10.01340346	9.320176919	10.20964764	10.03796113	9.817379515
9.400327152	8.770297859	9.69725304	8.604635949	9.433258586	8.967895455
8.862225737	8.968910758	9.499917182	8.498503737	9.40167604	8.859715222
8.554528737	7.780448354	9.093001495	7.646666444	8.254082232	8.72650097
8.384333949	7.783465061	8.622197111	7.846324646	7.855388333	7.692217293
8.307093606	8.533396525	8.84786598	8.072924455	8.522641273	8.074904687
9.096947232	9.488337717	9.368875727	10.30695028	9.391460545	10.05970796
8.634342798	7.48289603	8.517695303	7.137721343	9.057828929	8.246913576
8.951513343	9.082448343	9.256767414	8.882882525	9.264483889	8.918202434
9.513795939	10.22668357	9.366312899	10.47242114	11.36793803	11.70812672
7.089056525	7.875022121	7.410944101	8.124212202	8.589797808	7.932453717
7.895278253	8.519178475	7.928832091	7.506099556	8.324453253	7.202115061
6.606197394	7.36510997	6.913511828	7.376100929	7.867312374	7.195502081
10.71613341	11.34732253	11.44491584	10.92608881	12.31089415	10.90466263
7.113452899	7.826328066	7.858964434	7.436027273	7.795765162	8.133179586
9.099925818	10.38329808	9.119222505	11.00754401	11.77552743	11.0473733
7.363288879	8.030614293	7.462771485	7.968277051	8.626355717	7.832217929
8.097958939	8.246470545	8.090886384	7.75371804	8.78767504	8.44047504
7.253764222	7.759260374	7.526167626	8.193428737	8.958493495	7.597232061
7.774939586	7.437384141	8.156085586	6.81111	8.044980354	7.648616949
7.891531071	8.866888202	8.461315273	8.892970697	8.657372182	8.941795242
8.888766747	10.19332507	9.537497323	10.11246778	10.3380268	10.54441943
9.038849273	9.368224626	9.074692404	9.61181703	8.790654051	10.02439619
7.002659525	6.313976677	7.224700556	6.518533202	7.047573414	7.583692414

6.66276004	6.679485202	6.547344253	6.628556657	6.434266404	6.786032343
9.643287657	9.424985495	9.922126404	9.715215101	9.652169778	9.411394242
8.050868949	8.519692939	7.780116303	8.86024204	8.29927897	8.036816444
8.240781051	7.840343939	8.594455404	7.684267263	8.078044465	8.044608626
9.655770101	10.41731261	9.996420616	10.75600216	10.00361533	9.728754818
9.182204323	8.78767504	8.507211606	8.749346626	8.830660505	9.011546121
7.098240939	6.786285848	7.843077242	6.735177838	7.005636414	6.810582646
11.75215821	12.03602449	12.46797904	12.86955734	12.14191765	11.93486375
7.200097273	7.095808323	7.483852838	7.168394253	7.62509899	7.891531071
6.622442465	7.329578283	6.565400606	6.659578697	6.848028121	6.571183485
10.72127155	12.61449239	11.565007	10.92757694	12.30789527	11.46539851
10.53737043	10.58812248	10.4322159	10.67815746	10.36465285	10.3025572
9.731150899	10.31997281	10.38430498	10.94640834	10.27338352	10.63870636
6.977041091	7.500167182	7.034613909	7.809338131	7.202115061	6.980238687
8.935320828	9.169862626	8.932093424	9.096395636	9.436357141	8.795325869
9.222631263	9.260036485	8.482406475	9.066605263	9.000069889	9.690534253
12.28105924	12.12151457	9.224452333	11.39594259	12.39973853	11.70557621
7.63758797	7.260583141	8.11047698	7.145208253	7.636934919	7.64030596
10.08700287	10.07764109	9.427546879	9.165150051	9.897493586	9.712341424
10.40810668	10.23066525	10.09597734	10.28899471	10.14131493	10.63391466
8.500240172	7.825104404	8.118473111	8.751946515	8.303356141	8.274698687
6.413868758	6.760181828	6.291307172	6.734222485	6.257640303	6.364484303
8.074904687	7.945472162	8.214296636	7.481645051	7.424653535	7.791533949
13.13522491	14.04335209	13.29117716	14.19920898	13.62106704	13.17281418
6.097663737	6.004070414	6.322925	6.262744758	6.076457273	6.336448141
11.34202326	11.24001011	11.17213305	11.94745099	10.88031852	11.44319437
11.72126504	11.43005301	11.21493335	11.03580591	11.13085117	12.48835313
7.322879626	7.435437465	7.016713091	7.00817199	6.708797384	6.413679545
10.4655823	10.75737971	10.62764398	11.17213305	10.4429644	10.21279484
10.0143126	10.32849396	10.13768673	10.84844593	10.20164867	9.915234909
11.17571904	11.30506756	11.32185019	11.76804793	11.26446827	11.04593523
13.65450155	13.76702458	14.38889659	15.19830883	14.223913	13.85817003
9.280305798	9.347406172	9.505258828	9.160413333	9.453123465	8.86024204
7.207790596	7.11316801	7.055055273	7.134964384	7.084144263	7.472500828
8.918756414	8.677707162	9.144457596	8.50111796	8.802385697	8.792768747
10.86305205	10.90192126	11.05959848	11.78498903	10.84844593	11.09250749
6.706660646	6.606197394	7.233650202	6.589168273	6.708308495	7.396823121
8.698215303	8.598980253	9.143279535	9.595333525	8.991970091	8.833963152
6.154487859	6.291508596	6.197005899	6.692266071	6.207516667	6.337791596
7.497307495	7.294583061	7.860764394	7.21466303	7.794025323	7.802503596
6.455374485	6.321469101	6.741723879	6.212710222	6.357735	6.668064172
9.038261515	8.886096818	9.268661657	9.046862848	8.757405333	8.846850111
7.653305051	7.630594101	7.258107788	7.781852747	7.20918101	7.141446111
7.750957677	7.238429162	8.480536717	7.421647414	7.791533949	7.55945201
6.664911182	6.580572354	7.120482	7.191807606	6.998984111	6.60058796
7.311327071	7.769805091	6.971315	7.576037556	7.402015111	7.400015081
8.590695768	8.938439909	7.933221424	8.246087697	8.617420111	8.682101313
10.29101087	10.76471511	10.19649023	11.41981305	10.75737971	10.60863639
8.816214848	8.905887879	8.476485121	8.432150758	8.771856424	9.522660222
8.210994667	8.428657566	8.667049253	7.980256929	8.681579566	7.873377879
9.760900556	8.575566677	9.721175788	8.918202434	9.205404404	8.970028374
11.5571076	11.08107282	11.96059797	10.98492733	11.6295292	11.89130603
7.629989283	8.052783737	7.972012263	8.602379626	7.827906879	7.741457929
11.14866374	11.18800276	11.26446827	11.38577951	10.95374716	10.58935103
9.648698737	8.610845737	10.06071544	7.492628778	9.158023	10.40925212
9.428845576	9.071813909	9.632768747	9.326238727	9.232683343	9.644739414
8.628622212	8.40783399	8.90531298	8.53670504	8.41552702	8.549517636
9.090721626	9.533033172	9.829119768	9.584803364	9.143918798	9.054276

6.716118697	7.000811505	6.550562869	6.575656404	6.814711535	6.709545626
7.389870293	6.733232384	8.069395848	6.81164602	7.092673939	6.624843121
10.40387449	10.38842688	10.38208186	10.36032821	10.26632574	9.606954152
8.746311293	8.094218354	8.794788636	8.387507626	8.460053242	7.759936788
5.924154566	6.21305104	5.827829808	5.902196364	6.721088758	6.608949242
7.591410606	7.084738172	7.88753598	7.159147152	7.466652071	7.338325414
10.16073454	10.59292728	10.0728998	10.40178901	10.33476777	10.40178901
12.23769992	12.33576886	11.87687449	11.95514832	12.04734694	12.19996238
11.00496707	12.04734694	13.30795266	10.68960982	11.20840189	11.1612024
14.36999747	14.67552064	14.3093669	14.61202705	14.5413574	14.70594079
8.174468182	7.471589222	8.662363172	7.529315566	7.952774121	8.382609505
6.058799919	6.096900687	6.036996596	6.178175414	6.216210374	6.121756828
8.617840212	8.916479111	8.318250424	8.63611798	8.954176293	8.354947869
7.702311758	7.770089636	8.025864485	7.843485626	8.052417212	7.171589687
9.287819051	8.990775909	9.717365758	8.116577384	9.629839465	7.656238101
7.87472	7.904747051	8.112161727	8.563501616	7.943747505	8.120062485
9.287277576	9.195534384	8.838951081	9.09013302	8.749910828	9.119831202
9.30577901	9.063885091	9.411394242	9.149195859	8.951989212	9.285586323
10.38329808	10.84974643	10.31013098	10.98037262	10.67572435	10.69354222
9.704210374	10.3918566	9.919491525	10.80900786	10.43440935	9.809529798
8.616405606	8.720627081	8.29291099	8.302062758	8.838951081	8.719668061
8.207926798	8.904239657	8.788663253	8.934777717	8.837022465	9.156840283
13.23864174	13.54965001	13.03957854	13.65450155	13.41514643	13.70717091
7.263531485	7.227000899	7.651593505	6.481687929	7.589888475	6.369478939
7.709908667	8.090488788	7.736964596	7.864861071	7.637927172	7.476910626
8.064713051	7.877844556	8.291610121	7.223856	7.937437293	7.026621727
12.44264271	12.53197264	12.32364634	12.28369472	12.62449502	12.69676846
5.797768909	6.207117515	5.950042576	5.803646263	6.608710798	5.875432919
8.674281929	8.57597697	8.024267525	8.395296687	8.477856384	8.735287485
9.397864101	7.875808798	9.349388778	8.08972896	10.32531651	9.238221677
8.966940586	9.070661273	8.735755485	8.701109788	9.354289121	9.441234879
12.58287167	13.22156044	12.43279956	13.26276274	12.70002263	12.7494647
7.026870727	7.258107788	7.217577212	7.714642404	7.618887111	7.709511535
9.815129384	9.041820404	10.3810842	9.143918798	9.421059	10.16165443
6.916181	6.845030273	6.766018101	6.907588505	7.118512586	6.712107606
8.619347838	8.575095707	8.523871909	8.562227051	8.855784909	8.619848374
10.44392641	10.71860391	10.23550694	10.80644997	10.3090972	10.70181173
5.992397141	5.960154798	6.22255399	5.946081162	6.095072758	6.157664758
10.62280049	10.37124354	10.7814083	10.16743096	10.51291939	10.46342714
8.549141545	9.501970121	7.938531869	8.41552702	9.55462702	8.721926778
8.251125323	8.260520646	7.890755525	8.042161798	8.136641818	8.236633323
8.826393101	8.492747404	9.060618192	8.404822414	9.014335657	8.10683704
9.961780586	10.29752835	10.94208941	11.77802923	11.0940021	10.96311364
6.895973717	6.881279879	7.079375364	6.919943293	6.75222999	7.114645596
6.276206182	6.330710616	6.273265505	6.940218677	6.181905172	6.366826091
9.306330646	8.672881101	9.578067192	8.479185606	9.270407879	9.306330646
6.984268657	7.334908263	7.447389475	7.657544313	7.152687384	8.468898455
9.969141677	10.21583282	10.12126634	10.51771241	10.12697515	10.27236099
12.14507673	12.31089415	12.29275278	12.48835313	12.6076243	12.22629088
8.097222414	6.992392121	7.603389051	7.187484444	7.268778051	8.486053202
7.344596535	7.376746737	6.184535	7.279952616	6.988974929	7.343750657
7.190644495	6.451458394	7.590248525	6.523206192	6.890508141	6.817048798
7.568310444	7.535866141	7.301802939	7.424024737	7.359873626	7.767042424
8.920488949	8.472435657	9.552159131	9.018299374	9.530205505	8.467667768
10.86173253	10.86566339	10.83930015	11.00620373	10.54078574	10.81142627
6.559965303	6.304940444	6.402478545	5.881116071	6.424467222	6.741204455
7.937009	8.094989869	7.631600293	8.528915121	8.094989869	7.964914919
6.899234475	6.74683296	7.067324051	6.857008404	7.29540803	6.885309253

9.772980061	10.33375706	9.665316566	10.29208493	9.982665242	9.742895778
7.706168626	7.696259101	8.127895253	7.281724869	8.029383646	7.797844434
7.577683121	7.561342071	7.466006556	6.69036997	7.316095192	7.873080636
6.812677121	6.490840394	7.189710394	6.565400606	6.701095535	6.31049204
6.760181828	7.213178616	6.684542485	6.449508273	7.267388222	6.380003717
10.84705809	11.99535117	10.09079019	11.68700199	11.19966733	11.17571904
10.10187506	10.69931975	10.20452777	10.99623862	10.26541026	10.11452638
5.95863198	5.817312737	5.751365293	6.011944242	6.014458293	6.257467596
8.960218374	8.704708384	9.214111545	8.418959303	9.068490586	9.116804101
6.725730828	6.718256293	7.19681397	6.847264	6.938411576	6.774688626
13.77352346	13.15082863	13.98081649	12.39973853	13.98081649	13.46712151
6.746121293	6.889250929	6.66713102	6.870918222	6.29391397	6.558365444
6.844744919	6.635108111	7.177177071	6.661815061	7.095275111	7.029601354
9.352487919	9.341222778	8.81572204	9.209526556	9.376667182	10.27338352
10.92608881	11.24646499	10.92190902	11.02851208	10.64379219	11.1031408
12.31719428	11.84736958	12.82153585	11.73710477	12.47761486	12.76207554
8.124212202	7.796085586	8.757866131	8.073294879	8.236986626	7.910617626
8.230588212	8.352134333	8.940763364	8.975838535	8.633848111	8.395713444
8.408718253	7.894315778	8.605125889	7.872718384	8.075284384	8.091725323
6.27402404	6.212018222	6.753240879	6.171621859	6.485740606	6.217314636
7.675167556	7.36203996	7.629016404	7.031727121	7.515531818	6.798855303
7.505451222	7.958393384	7.125146293	7.532708636	7.617533182	7.748187364
6.414036263	7.056486293	6.410292616	6.443657091	7.051121485	6.378748303
7.121055596	7.505451222	7.141717727	7.665314131	7.394027687	7.436027273
10.31795252	10.48659409	9.983540212	10.50166798	10.21375666	10.7814083
11.03452834	11.20660223	11.03163187	11.87445957	11.22600179	11.21816154
8.39867397	7.777661384	8.383846919	8.00165403	8.149032212	8.613985131
7.605113071	7.587005707	8.055916879	8.357077121	7.592689374	8.142577798
10.27338352	10.8254891	10.38531322	11.25349741	10.79956984	10.29949835
10.042425	10.03983393	9.392082222	9.669114061	9.989520798	9.863774293
8.182386152	7.569572879	8.540635232	7.576996152	8.155735586	8.133538364
8.81966502	8.460863192	9.228437333	8.479727162	9.135618929	9.198436465
6.868352737	6.82385998	6.809755232	6.693002899	6.749805475	7.214922364
7.263859848	7.159147152	7.328721848	7.220481808	6.857008404	7.072869879
9.870221061	10.40592026	9.779012071	9.982665242	10.86963124	10.12314196
8.168196313	7.69973097	7.645943414	7.64232702	7.131581374	7.645321707
8.721926778	8.824851909	9.032817303	8.621272737	8.502042192	9.250125475
7.112620848	7.258693172	6.975978909	7.676956101	7.026391172	7.595301182
10.13479405	10.29101087	9.709426838	10.3779146	10.18468509	10.50543333
8.564366949	8.256499515	8.047933768	8.049354232	8.315715768	8.358418384
8.755916071	8.912003131	7.069444	10.19236777	8.975838535	8.741655828
7.506099556	7.838173152	7.50339202	8.469779808	7.422534091	7.864224778
8.253254586	8.066374202	9.016491202	8.029383646	8.537544354	8.163475424
12.77015535	12.66167346	12.26775966	12.12939166	12.46055504	12.41337695
7.213710414	6.798855303	6.961028939	7.525015414	7.07551896	6.669224869
9.94402302	9.876096838	10.11849919	9.782133848	10.07857135	9.890949131
8.882379495	8.995099212	9.206631485	9.008956374	8.643636162	9.361671283
7.705120374	7.39712799	7.33379003	7.583692414	7.365664505	7.567690545
8.592622071	7.975072535	9.311440606	8.835019636	8.607626293	9.910074283
9.667708556	9.629101929	9.624391323	9.323195364	9.977292899	9.626304798
7.970926879	7.962380919	7.614497747	8.122747707	7.758192424	8.228510596
9.030619364	8.234636465	8.947623838	7.996041859	8.490531303	8.82024202
6.454080828	6.303106697	7.102116364	6.517203657	6.547113253	6.485955434
6.509135818	6.285908	6.908074727	6.336448141	6.392742677	6.749805475
12.26162026	12.01956714	13.0147344	12.11841559	12.97977545	11.85617239
9.087865687	9.084401768	9.03221704	9.260607	9.11093099	9.129654758
7.565085768	7.383097848	7.293451808	7.252886707	7.222702545	7.674109172
6.324345747	6.314614657	6.255807596	6.377923929	6.27919301	7.116869414

6.819920253	6.819189677	6.417796273	6.700124848	6.96434804	6.864210101
9.138451657	8.62460002	9.006527424	8.229339455	9.067241768	8.988577162
7.990677606	7.641683626	8.730448515	7.631889737	8.143419303	8.612685364
6.914843414	6.787317758	6.82197003	6.762066263	7.663954061	6.339770293
8.525321657	8.103690354	8.933180404	8.284073869	8.499404646	8.353342091
7.102982434	6.986217889	6.909401626	6.547549889	7.84419497	6.310688818
5.967318737	5.79006096	6.02751096	6.355365798	5.991248111	6.061217212
7.60255999	7.167804919	7.733842657	7.038917566	7.621361828	7.80435998
14.04335209	13.40990013	14.10328164	13.29684189	13.74093847	12.28105924
7.168103949	7.101049919	7.351279444	6.855940818	7.06891401	7.362377768
7.341339747	6.968156515	8.340681505	7.108809091	7.821625848	7.525015414
7.307022919	7.563777768	7.611330343	7.964179182	7.622739535	7.563777768
9.636296737	9.525471414	8.900236364	8.96575796	9.547339828	9.420326576
12.47441306	11.99323436	12.75784116	11.54675633	12.52819783	12.84197228
11.26623333	11.23641801	11.28565422	11.18800276	11.29947244	10.95374716
11.86125412	12.052647	11.83330316	12.55983323	11.84291489	12.01673563
6.392953667	6.499744121	6.414650828	6.959189505	6.577039475	6.792041707
10.29851595	10.04776272	10.85513517	9.903372323	10.52135963	9.960013465
9.17861001	8.82024202	9.845595727	8.367840636	9.397864101	9.667708556
6.843295	6.121104576	6.493490929	6.552973232	6.063889717	6.401803212
7.158304313	7.340448859	7.04319302	7.295094879	6.738778758	7.449616394
8.025425616	7.628336929	8.486053202	7.161342051	7.634916323	8.339115859
8.046433182	8.966940586	8.163923192	9.26122399	8.447076788	8.089360869
8.708467242	8.711304737	8.475612051	8.956909848	8.87289497	8.997224192
8.766842081	8.051625212	9.102676354	7.902575141	8.516793455	7.356848556
8.778623323	8.898709273	8.660608859	8.44047504	8.41598997	8.645985424
7.222444626	7.442271202	7.583321859	8.116212253	7.404985061	7.444499747
8.558108202	8.538380869	8.954798808	8.237787788	8.794361798	8.885588879
9.886023253	9.633493677	9.993897848	9.622300838	9.731806091	9.538263677
7.980934222	7.853350485	8.399553939	7.603719626	8.616914869	8.215875455
9.122138505	7.92237903	10.01622438	7.754082929	8.425403333	9.287277576
10.71992703	10.82664672	10.92757694	10.26224218	11.21016953	10.0143126
7.352232505	7.731201121	8.154321646	7.670568384	9.165150051	6.931948737
6.768428949	7.309928404	7.179445253	7.642932737	8.972927475	6.798572747
6.819189677	7.296277414	7.235366172	6.908074727	7.834787162	6.220682606
7.846688253	8.233421293	8.338355444	8.124973859	9.168828475	7.589252838
7.546292778	7.850890354	7.521572545	7.405823253	8.423588242	6.760600303
8.989652131	9.045168101	8.610845737	8.314398727	8.906910121	8.140128606
7.539167263	7.526779747	7.629673333	7.630594101	8.448456354	7.387061818
6.930987343	7.090185162	6.86313897	6.866055313	7.438305869	7.047573414
11.54470354	11.97944932	11.77552743	12.15130062	11.57567047	11.9091429
9.239472869	9.46304199	8.913588253	9.477181455	9.049171303	9.320738646
6.864476545	7.385541293	7.225001677	7.900011667	7.192707293	6.945089283
10.75178718	10.24727703	10.77637516	10.49830212	10.36781996	10.89094316
12.16036216	12.26162026	13.12023156	11.6137143	13.98831971	11.99323436
10.94208941	10.95234717	10.39521192	10.67428006	10.70181173	10.8935809
7.768402071	7.608041616	8.084275677	7.228169333	7.940608939	7.494510091
7.074173121	6.248708515	7.772843323	6.458735293	6.931296273	7.272914455
5.784641202	6.347049828	5.816439131	5.876742788	6.485310808	5.77189203
11.35858946	11.54470354	11.1276132	12.65819872	11.74670945	11.2278827
10.7442872	9.430143091	10.74819911	9.020821455	10.28089537	9.172421828
10.05880999	8.977005354	10.4429644	8.606624646	9.956562586	10.71860391
7.030955384	6.328112374	7.345267404	6.432439828	6.654303071	7.595878485
6.625743253	6.350448798	7.116047475	6.364484303	6.698531303	6.96434804
8.493155899	9.023639636	8.211847566	9.027655758	9.404880263	9.35188598
5.775106808	5.778926242	5.788151778	5.767446949	5.792970636	5.845283899
12.80949032	12.32364634	13.07454735	11.59654933	12.90359001	12.58287167
8.78664797	7.965251131	9.145078374	7.656544636	8.644542606	8.17298499

10.00361533	9.875361242	10.05236285	9.688441414	10.17030341	9.387059283
8.038354192	7.876138384	8.427236737	8.114611232	8.578858313	8.210602626
8.071703475	7.652274172	8.601896768	6.788535525	7.579959303	8.612168455
6.555521899	6.422006566	6.921167192	6.265013455	6.42385696	6.527644212
11.41238356	10.91176188	11.49029795	10.08145981	11.24646499	11.01531103
9.842388081	9.624391323	9.883591758	9.528176495	9.804037697	9.127872697
6.776493808	6.423456465	6.838224475	6.227696606	6.784856394	7.180559343
10.4322159	10.32309273	10.41123643	10.06071544	10.4322159	9.736957071
10.20060626	9.282486081	10.54565144	8.996148737	9.960976253	10.84844593
9.053043889	8.222994222	9.501970121	8.131862263	9.062782333	9.31393701
11.29947244	11.07797425	8.681579566	10.13768673	11.41071085	10.4450719
8.159907626	8.311754818	8.556855828	8.147147636	8.640338566	8.279889293
8.526649808	8.106019798	9.042944374	7.967319419	8.672348475	9.000069889
6.143547697	6.261209	6.21305104	6.464462626	6.363691192	6.030805626
7.986161141	8.063898495	7.935468505	7.877119465	8.125376242	8.228066545
8.513601889	7.837132667	7.62575301	8.138156535	7.708254091	7.588938485
8.059423535	8.470647758	8.559456798	8.115437899	8.997224192	7.766738848
8.325717687	7.929593313	7.452109758	9.000672535	8.163923192	8.548679899
9.812775374	8.897751606	10.09254304	8.734416606	9.174793152	9.100524758
8.191909586	7.129307667	8.565346707	7.138578667	8.000521869	8.379510323
10.14999886	10.43440935	10.24942024	10.75178718	10.3494333	10.57028524
9.299183616	9.531623293	9.237664879	10.04600105	9.321347081	9.551451798
6.577039475	7.533663232	7.782796596	7.210091465	6.923947081	6.986217889
8.55725397	8.903025495	9.784374313	8.867411152	8.848410616	8.690381253
11.0473733	11.41605585	11.10170724	11.5737214	11.1031408	11.17750487
10.22448985	10.42622906	9.939327313	10.82153564	10.13671639	10.31193963
8.582586091	8.822853162	8.465492313	9.038261515	8.486053202	8.741130788
7.152958919	6.970472768	7.189410667	7.192707293	7.182015707	7.382214869
6.641378788	6.504968949	6.997357515	6.805713475	6.764115131	6.961758566
10.32849396	10.19957169	10.23066525	10.03876748	10.73661507	9.397864101
13.06967433	13.52334642	12.80602179	13.41514643	13.22730043	13.28559105
6.567922788	6.153328596	6.977525061	6.211158404	6.472782788	6.546202222
14.25042984	13.83772389	14.42673578	13.2158469	14.010587	14.21449649
14.63205333	13.78737352	14.7172184	13.74093847	14.33555643	13.32021166
16.96059838	16.4534382	17.1822602	16.2036826	16.52993947	16.25437967
6.663185626	6.890761263	6.889002121	6.872867768	7.451812333	6.677606
7.086157636	7.260895414	7.381324374	7.687700545	7.665987182	7.073400869
11.11081747	11.35657003	10.2861211	10.23160947	11.1060681	10.55842197
6.501664081	6.638813101	6.801007778	6.463301323	6.69036997	6.908606212
12.7939548	11.88649013	12.96662998	11.60069924	12.3774088	11.75462574
17.1822602	16.96059838	17.23317693	17.07010739	17.23317693	16.64557528
10.8605042	10.60743282	10.40278823	10.32849396	10.59541023	10.96000427
6.985416596	7.350388939	8.144161414	7.300950758	7.225301495	7.597541737
13.87263802	14.40789433	13.87958112	14.38889659	14.02572128	14.01682491
8.06213903	7.217577212	8.403471909	7.252038162	7.701686677	7.972012263
13.01016522	12.85589012	12.28693608	12.68097339	12.67378338	13.16824615
6.951745222	6.549368909	7.55847901	6.762834384	7.275765808	7.378186273
7.332669495	7.303562909	6.993764091	7.12322297	7.313451111	7.493254434
7.357173859	7.53302901	6.945903495	7.176878273	7.340746384	7.395882727
9.415860727	9.438094949	8.910531141	9.324974768	9.005975162	9.508442768
8.575566677	8.595283071	8.245649515	8.399075535	8.246087697	8.472925747
8.929384727	9.34289298	9.087255717	9.152615889	8.555429434	9.179226364
8.440058758	8.288355808	8.19621002	8.378515131	8.150096222	8.33273403
11.64044206	11.35475345	11.11714857	10.7532413	11.17378617	10.92190902
12.59323492	12.18792926	12.14507673	12.32964397	12.29548604	12.32364634
9.381544081	9.073600626	10.07388225	8.863375313	9.246644323	9.495686737
11.65455063	11.82346178	13.06967433	12.32659908	11.34892675	12.57877126
12.94821125	11.9875501	12.66167346	11.72126504	12.33576886	13.15082863

7.003715121	6.903241343	7.731542758	6.871158202	7.272016424	7.232151747
6.360934626	6.755203192	6.794399263	6.728447091	7.471219899	6.50824402
6.770142061	6.581970424	7.299385535	6.64329102	6.950382242	6.851905687
7.843485626	8.276438626	8.830117929	8.273878929	8.222818808	8.08193004
11.23641801	11.34202326	11.47322198	11.65455063	11.20163629	11.50425191
7.318843495	7.266525586	7.893631939	7.02750098	7.480029465	7.599486586
7.079910848	6.992079758	7.408375899	7.030162636	7.128773535	6.796831899
9.851320949	9.651465606	9.818059192	9.32739897	9.94402302	9.692043475
12.7452022	12.49170848	13.06488572	11.50632759	12.95303817	13.25686564
7.469634172	6.756409455	7.700670768	6.694471303	7.411561343	7.686775222
10.18098661	9.692043475	9.901640636	9.054276	10.18000397	8.878776172
9.198999222	9.277455131	9.49850403	9.79615097	9.848227263	9.848227263
9.30770201	9.618955576	9.440510152	9.95201103	9.224452333	9.458377051
8.645541747	9.694925798	8.675789182	8.652865111	8.765765323	9.466909636
9.505258828	10.12799521	10.08799683	10.25422487	9.519820636	10.08145981
8.416400293	8.816703535	8.352537101	8.474658303	8.115821848	8.308776677
7.418946535	7.120482	7.363617495	7.419275485	6.719039212	7.001104828
12.35798465	12.97977545	12.12692782	12.80166365	12.12436236	12.68097339
12.4956701	13.05987966	12.9709957	13.66158872	12.70787607	13.07454735
9.580097263	9.971733293	9.960976253	10.23752804	9.635582141	9.785983535
7.666980273	7.732206152	6.827413455	7.46337499	7.533296202	7.190644495
10.45041513	9.758431737	10.78856661	9.627013505	10.23850383	10.17122401
9.365016101	9.895856061	9.468772697	10.09597734	9.363823202	9.332175414
9.383480283	9.473222667	9.446464909	8.879330566	9.600185283	9.187244566
8.247392788	8.757866131	8.27861104	9.411394242	8.432612263	8.263866778
9.822898081	9.344897333	9.554043101	8.755421576	9.660200838	9.773700283
11.27695531	11.08264222	10.5336638	10.89224764	10.761049	10.5397012
6.981335535	6.356085939	6.152673566	6.107803293	6.897441	6.147877354
10.80782516	11.11560115	10.83100199	11.78265022	10.9088749	10.87637444
7.488273475	7.403028737	7.544047919	7.21064097	7.573484121	7.691535778
7.252299788	7.13441502	7.544716788	7.153495505	7.443192556	7.359577545
10.91903182	11.17750487	10.65127749	11.48258282	11.01209098	11.24323027
8.468898455	7.732550404	8.613130333	8.005065495	8.086153313	7.894965162
8.85785699	8.688838939	8.936938576	8.546056747	8.600831152	8.304980606
7.724673141	7.799196616	10.18762619	10.17904437	10.21054893	7.403336687
6.998209475	7.069444	6.643064293	7.242080394	6.921167192	7.183662697
7.118793697	6.799617657	7.249066101	6.71121296	6.797872071	6.956363394
10.20748734	10.26832521	10.22070473	10.41626133	9.815129384	10.16937171
6.972058485	6.582672778	7.655611	6.755664455	6.98587896	7.220240556
8.712915394	7.996393263	9.238865747	7.797125465	8.43615796	8.855784909
6.626176061	6.305549929	7.312858101	6.453641556	6.314167232	6.470600061
8.196571586	8.166172404	7.800216455	7.946541949	8.406205495	8.447526838
10.13862753	9.95201103	9.887613414	10.01071956	10.1905069	10.39072525
9.321347081	9.298556212	9.150235333	9.077100232	8.941795242	9.243096889
9.227275152	8.727989172	9.482360616	8.447526838	9.477759242	8.688838939
8.45577798	8.544134919	8.248137606	8.780092131	8.247731232	8.325316889
12.03068847	11.79870316	11.63401423	11.46724679	11.47127063	11.53050203
10.02923217	10.01168912	9.53690398	9.638947465	9.672623929	9.785178838
6.214653717	6.384961778	6.963095333	5.772170828	5.780513333	5.79278196
8.344860929	9.172421828	8.414282596	8.986932636	8.76478299	9.025691242
8.300103657	8.11773804	8.250622253	7.754407434	7.907197636	8.104866828
7.688370495	8.00165403	7.655968232	7.351625758	7.626470505	7.892936636
9.415217505	9.652169778	9.002272697	9.320738646	9.36570098	9.739859192
12.21312889	11.41981305	12.24649354	11.20660223	12.3613163	11.39772243
9.078331919	9.165150051	8.886620687	9.078885242	8.789132293	8.755916071
10.84176532	10.66465811	12.10694646	11.09721747	11.65698449	10.24038579
8.156085586	8.029383646	8.708030737	7.427701768	8.210186616	7.97830402
7.270788949	7.25118401	7.447087758	7.145769434	7.790604293	7.241538444

15.50564301	15.24813401	15.56435861	15.13975511	15.48449671	14.72870348
9.988725727	10.33890802	9.975431768	9.828404283	10.15875628	9.793942798
13.32669092	13.18858184	13.15082863	13.16151113	13.02006671	13.58209631
7.523479626	7.569223354	7.688677182	7.432376434	8.530333535	7.52646604
7.75479003	7.263859848	7.877119465	7.398020586	7.796085586	7.083894141
8.764299566	8.407442505	8.780092131	8.006495333	8.751946515	7.912365798
10.86691694	10.6768734	10.78269205	10.80499832	10.77387872	10.52364746
13.58209631	13.43523914	13.22730043	13.35359128	13.39659876	13.35919327
8.982611727	9.279246253	8.706127333	8.883414061	9.31393701	9.211184727
7.560760293	8.039068889	7.374297343	8.093860848	7.710578727	7.954939636
16.61084374	16.64557528	16.39933956	16.61084374	16.8710039	16.8710039
9.818860343	9.740584727	9.296131263	9.759931222	9.782868192	9.55462702
6.756883091	6.813409273	7.271131505	6.889002121	7.276013455	7.034331232
6.512378081	7.384295283	6.81164602	6.895973717	7.144617717	6.710494808
6.333774687	6.624377646	5.768491222	6.097185768	6.20351898	6.034734515
9.290785283	9.299183616	9.424985495	8.899757485	9.514496606	8.997807768
6.706906717	7.374297343	6.95496698	7.931708101	7.174439091	6.534413101
7.569899606	8.138519566	7.567404636	7.553468394	7.273826505	7.560118414
7.362680232	6.453863293	7.375790182	6.808520727	7.033114263	7.544367232
8.379510323	8.371674091	8.861728919	8.251492909	8.86024204	7.957660747
10.92337564	10.85225864	10.88302044	11.13400706	11.00902763	11.36423723
13.70717091	13.50997822	13.48401144	13.49745762	13.58996905	13.95167504
9.501970121	8.41552702	9.872596081	8.485174677	9.162016475	9.547339828
9.439397061	9.960976253	10.24828231	8.865407737	10.06547375	9.660200838
9.903372323	8.957404525	10.51886057	9.012760899	9.981718727	10.48539779
16.39933956	16.3077943	16.57174008	16.39933956	16.57174008	15.81050193
6.930500768	7.180839586	7.159962485	7.036743576	6.754244737	6.900739596
6.229843364	6.010338545	6.013925687	6.447481424	6.564717364	6.674983667
7.218710525	7.146069808	6.527907232	6.739283293	7.246997929	6.918565768
6.662501485	6.762314343	6.950101798	7.099574394	7.619933919	7.254341798
14.83117585	14.23242051	14.62217632	13.80704919	14.24162707	13.5964752
11.89876792	11.72807534	11.65698449	11.38025327	11.60069924	11.50051559
10.30358984	9.945994374	9.332175414	9.727055768	9.515069697	9.841562192
6.825072616	6.767959131	6.447869455	7.052765889	6.591366	7.128773535
14.23242051	13.52959744	14.26676228	13.18858184	14.03424604	13.07947925
6.415074455	6.330919414	6.508047626	6.341087596	6.253125879	6.383610909
8.032161838	8.43615796	8.59942799	8.27903296	8.813595202	7.871272152
8.60808904	7.959488687	9.009915859	7.708578687	8.649944576	9.278597747
9.27419499	9.646274414	8.901935111	9.153730283	8.984797889	9.380979222
6.389669667	6.237206657	6.600799566	6.201602636	6.37918304	6.524488081
7.686444293	7.799854778	7.665987182	8.097566687	7.475313929	7.787223354
6.922922101	6.816537596	7.194677455	6.857241172	7.079910848	6.982909788
9.882779162	9.733230051	10.36781996	9.488337717	9.592426051	9.561667293
7.88825004	7.960941889	8.195405505	8.598980253	7.757234778	7.975072535
10.70181173	9.940265465	11.06573849	9.650789909	10.82945836	10.83501466
11.43380494	11.21493335	11.10460602	11.07158908	11.24457024	11.13713447
13.82538175	13.87263802	14.04335209	13.91489381	13.87958112	14.02572128
6.235363323	6.761335818	6.182782414	6.25331604	6.852686475	6.311249485
7.02200603	6.343264394	7.349181162	6.171476646	6.821005303	7.320933798
7.608647091	8.236986626	7.695912303	8.78214798	8.567346869	7.510343899
14.68633385	14.63205333	14.48025691	14.5513462	14.5513462	14.7528858
6.273265505	7.698928879	5.962749869	6.565804798	7.797507939	6.836530859
6.483554929	5.967434616	5.808778929	6.022727263	6.156331323	6.633467848
7.593664717	7.993428586	8.383429707	8.41382397	8.428176394	8.326162172
6.857999616	7.434501333	6.933000202	7.377888556	8.083485404	7.257203303
6.94407103	7.278747747	7.308421545	7.344596535	7.60282502	6.957636657
6.657655061	7.425828556	6.547992737	6.602666131	7.044506424	6.687236677
7.01049403	7.029897788	7.511581475	7.706567859	7.742113232	6.887014152

7.720541949	7.893631939	7.45028304	8.678158051	8.076845152	8.112525414
9.545881535	9.759931222	9.237064687	9.492465394	9.563605545	9.794631646
9.424985495	8.633351606	9.174120707	8.297630758	8.680513909	7.845601525
10.38208186	10.32750324	10.19957169	10.0143126	10.44934262	10.45041513
8.073652495	7.44169298	6.786546495	7.433328091	7.689572788	7.492282404
12.3843369	12.50706636	13.30255942	12.5521346	13.74759592	12.01956714
9.216574283	9.342353505	8.853585596	8.273061586	8.309247939	9.580097263
6.900488939	7.144029273	7.269598859	7.084441778	7.782178323	7.168942192
5.825182131	6.674020838	6.172106293	6.37731698	7.473129747	5.923214808
9.188424626	9.428190232	8.964085616	8.549141545	8.563501616	9.666877556
7.593038414	6.772514131	7.258693172	6.665659576	6.996769242	7.378508566
10.43998577	10.20164867	9.780965934	9.851320949	10.07764109	10.12041459
7.616235111	7.391081455	7.719108384	7.28582396	7.71023803	7.681826051
10.78014639	10.72786832	10.56306707	10.69793218	10.72127155	10.89912578
8.387083515	8.472925747	7.994743247	8.322806515	8.200539131	8.345353354
11.95261936	11.6137143	11.63174178	11.55928848	11.69188536	11.92156179
11.82852227	11.64244603	11.62290433	11.09089144	11.37490296	11.68484165
8.702074263	8.200539131	8.199315667	8.402148465	8.019769071	8.530767747
8.620285051	8.41382397	7.967113929	8.391869283	7.926300626	7.564402343
11.52824	11.41415047	12.12151457	12.32024961	11.86652814	11.64637724
10.39641485	10.78856661	10.48539779	10.86820493	10.29851595	10.48782122
10.33146686	10.79145221	10.53141254	10.94496034	10.22668357	10.56548536
9.303342798	8.906910121	8.246913576	8.777050384	8.679110091	8.927565404
6.495796157	6.521900899	7.377037515	7.585519525	6.967234434	6.810307121
11.92396137	11.21016953	12.14191765	11.26815786	12.16279806	10.33596201
8.758364879	8.94874	8.333227545	8.848410616	8.897177899	8.906910121
9.679062616	9.730279475	9.11211799	9.50650501	9.872596081	9.786685869
10.58579258	10.4994288	10.32635032	10.84302834	10.41123643	10.33989796
9.027136657	9.096395636	8.465000141	8.781156657	9.140978061	9.09178804
6.666656929	6.763870838	6.698743475	6.688196	7.33379003	6.875911141
8.474228909	8.765765323	8.280295667	9.281923404	7.946141838	8.39446903
11.51647662	11.30301738	11.1612024	11.01951417	11.29597305	11.65455063
5.962749869	6.104726626	6.521234222	6.69920203	6.539797657	6.038531545
7.970233808	8.117339677	7.721580051	8.050484323	8.344441616	8.396532182
8.790130253	8.302498081	9.240719434	7.969046636	8.861728919	9.284395657
12.02811467	11.85301976	11.671419	11.76101506	11.64244603	12.16279806
9.891739697	9.682548525	9.588958727	9.842388081	9.774477667	9.788274313
7.106592788	6.948610667	6.861656576	6.90482902	7.081850444	7.197080263
9.923658606	10.17789322	10.70902317	10.31013098	10.89604789	9.021393263
6.88247998	7.185712515	7.191229121	7.084738172	7.730154939	7.18511501
9.759931222	10.02741959	9.660861778	9.955574404	9.617464455	9.595333525
11.58195332	11.51465712	11.21668249	11.34892675	11.37131374	11.58814301
12.73352188	12.66627862	12.61090773	12.81379045	12.87421343	12.87421343
7.430512737	7.285574899	7.430156263	7.085030667	7.196057152	7.574489596
9.543825273	9.139724848	9.513164303	9.430143091	9.147296667	9.397189192
7.284431525	7.436336172	7.092673939	7.44801498	6.995042596	7.465665798
6.768722596	6.618269616	6.979123747	6.627087081	7.57049196	7.098999354
13.30255942	13.12562627	13.44849295	12.90783816	13.22156044	13.43523914
7.770465818	7.271701444	7.775998535	7.958393384	7.094132182	7.867312374
13.07454735	12.75784116	13.11515098	12.96241124	13.03957854	12.65104366
8.958493495	8.878201818	8.223899788	8.48873997	8.38301802	8.269818727
7.013137848	7.233039222	7.36203996	7.283275424	6.929106091	6.698531303
7.28582396	7.252299788	6.86860504	7.258107788	7.304428303	8.078389566
15.88528111	15.95256028	15.74256723	15.27762024	15.97330865	16.22759729
11.59021288	11.44491584	11.5571076	11.542568	11.51008986	11.60888892
6.468711152	7.143674071	5.919756818	7.258429848	6.416313212	7.057330242
11.79171062	12.24649354	11.4090051	11.58814301	12.59323492	11.97944932
8.670447949	7.567065616	7.78118699	6.846509828	8.009072212	8.991410131

9.45706402	9.789131121	9.57181996	9.680349586	9.411394242	9.521192909
12.82558839	12.58287167	11.911688	12.41634374	12.37087917	12.88708268
7.76948301	7.276277889	8.090157434	7.106302455	7.889761727	7.806845253
7.847720859	7.425506818	8.272231939	7.446082798	7.780448354	7.568036061
8.113745737	7.819838162	8.77608297	8.012346152	9.423636879	7.783110646
7.866656354	8.174871818	7.924502232	8.569735879	8.217984283	8.255317818
8.379056919	8.418959303	8.346989566	9.295516616	8.811517808	8.702574091
10.13276962	9.814415596	10.14895288	9.710054737	10.09426568	9.678297182
7.467207283	7.174439091	8.070136677	7.209485919	7.921614596	7.693213707
7.266813283	7.269892283	6.96434804	6.77698199	6.796104869	7.06104997
6.738245586	6.840143455	6.580819444	7.012316737	6.582439566	6.586655667
6.254539838	6.022435242	6.652395162	5.890745525	7.379773919	6.343830616
8.59655801	8.582586091	8.289212586	8.614861051	8.133993636	8.96575796
6.643064293	6.770888697	6.209212768	6.674468929	6.86986903	6.856746788
11.24001011	10.99345288	11.32027501	11.05796986	11.09250749	10.96886286
6.609208404	7.329290475	6.378366909	6.406814333	7.361082222	6.601482788
8.035288182	8.475612051	8.406604404	7.773938747	8.481851586	7.238429162
7.903688606	7.515816273	7.950449798	8.07651197	7.685352364	7.90290904
15.01206542	15.12351424	14.65397593	14.7172184	14.82007331	15.12351424
8.406205495	8.749910828	8.41335802	8.791657869	8.526186556	8.57070398
8.99408802	8.498503737	8.875711838	8.818695101	8.821324343	8.698215303
7.075225141	7.55499601	7.265354444	7.280525364	8.31318196	7.225864141
5.845763465	6.128446525	5.905392192	5.976914455	6.323307657	6.133153667
6.775232444	6.302516242	7.355080212	6.444377596	6.684320869	7.028809465
14.27397716	14.35197184	13.81935116	14.010587	14.07988992	14.16629104
12.61090773	12.64265677	12.19727311	12.31089415	12.34577038	12.67378338
7.926719768	7.733228788	7.528986283	8.125818909	7.677264869	8.400464071
13.09045797	12.73731843	12.85589012	12.89886333	12.88708268	13.26910509
10.04776272	9.530933293	10.41022215	9.204345657	10.17789322	9.748306121
12.27421104	11.84291489	12.18142186	11.90384839	12.10135206	12.37416009
9.340550081	7.313451111	11.29744903	7.456122566	8.483763566	9.473962505
9.291393586	9.126663535	9.839882283	8.950355889	9.615324495	8.931557869
7.619933919	7.686071172	7.739314727	7.531770848	7.979449616	7.349811747
13.2158469	13.14611959	13.10484893	13.03496614	13.09045797	13.21058954
11.76588718	10.90057618	11.71659127	10.21583282	11.46724679	10.14534091
9.035586242	8.541524636	8.557697889	8.659649677	8.652865111	8.556357949
6.301115768	6.46756804	6.433428778	6.539797657	6.660563556	6.402667606
10.42175216	10.22761479	10.03611568	10.41530743	10.5104744	10.24612484
11.57178154	10.97896732	11.80342824	11.06877356	11.69891546	11.76588718
11.36793803	10.9660467	10.28005063	10.91903182	10.31597323	11.1502402
10.1597097	9.988725727	10.0178996	9.829119768	9.763138212	10.43998577
11.79615897	11.61117883	11.45738434	11.43005301	11.53050203	11.80791686
13.34730616	13.28559105	13.17778072	13.44849295	13.5160991	13.83173935
9.032817303	8.608532485	8.833337414	8.541125101	8.720627081	8.858269384
10.93646713	11.01376888	10.72900488	11.26446827	10.82034332	10.61001127
10.37124354	10.29949835	10.8947391	9.503858424	9.223885747	10.09161001
11.40725584	11.32721575	11.10761793	11.24993851	11.11230451	10.79284767
12.65819872	12.36473556	12.42616911	12.29275278	12.43600068	12.70390423
12.75784116	12.69676846	12.46466592	12.69316533	12.60396528	12.76628311
13.03957854	12.76207554	12.86955734	12.56315114	12.56726296	12.61449239
15.12351424	15.59877501	15.21350605	15.95256028	15.19830883	15.65613201
11.37670858	11.29076429	11.26990549	11.21338774	11.49839615	11.73271713
7.553068374	7.524403394	7.532708636	7.907575465	7.586125333	7.33182303
7.964179182	7.869033162	7.814093232	7.44169298	7.358688323	7.848409778
13.10484893	13.04869312	12.43944324	12.66627862	12.69316533	13.23864174
11.7349036	11.54053762	11.48258282	11.2786408	11.45526933	11.84736958
9.917863333	9.212980727	9.89348604	9.132651727	9.887613414	8.495994869
7.674820101	7.728361283	7.689234404	7.712908172	7.415948364	7.555972677

7.448630646	7.507381747	6.938620788	7.678591667	7.033625222	8.336257222
7.988582505	8.078774939	8.046784596	8.156410909	8.092218303	7.683318778
6.896472707	6.58977503	6.610458242	6.57823699	6.580819444	7.283562505
7.695265798	7.31374401	7.892604899	7.218159515	7.641985	7.851994586
7.335553323	7.037511364	8.664254273	10.1905069	8.891888576	9.670530667
12.11562205	12.67378338	12.08943903	12.88265743	12.32659908	11.7894918
7.579628838	7.520648323	7.568622485	7.814474586	7.258984768	8.248137606
7.066192545	7.239071505	7.076304455	7.617533182	7.229587778	7.462771485
10.00886723	9.654354424	10.28406896	9.945028121	9.917863333	9.730279475
16.73234105	16.82803465	16.90924948	16.8710039	16.82803465	16.39933956
10.6488688	10.86691694	10.33890802	10.55726192	10.87637444	11.2345748
8.561321323	8.714332636	8.269818727	8.582147707	8.40783399	8.676316919
6.002250273	5.823446444	6.194435071	5.761322101	6.010600899	6.127366242
9.766131535	9.548618051	10.05320582	9.562994313	9.727935707	9.624391323
6.955646677	6.96619404	6.664911182	7.584276586	7.280525364	6.901978929
10.87379507	10.30810853	9.770750222	10.59292728	9.963504081	10.45754412
15.74256723	15.67809237	15.86132141	15.36066179	15.70163214	15.27762024
14.17411554	14.50017253	14.21449649	14.83117585	14.21449649	14.4335752
13.96779242	13.80033164	13.69473258	13.95167504	13.83772389	14.07988992
10.0841781	10.02061657	10.51291939	9.387059283	10.07107569	10.6426412
8.277405556	8.137069859	8.508189222	8.019769071	8.554027364	8.308331848
7.165793091	7.08589403	6.506514626	6.842028788	6.787317758	6.905316545
7.44801498	7.30556202	7.774617596	7.431067202	7.638981707	7.650531152
14.67552064	14.28325373	14.70594079	14.45281427	14.72870348	13.87263802
7.907575465	8.409548323	7.827504414	8.692466525	7.885002121	8.082337909
11.97944932	11.71244686	11.59870577	11.57178154	11.55106122	11.93975968
7.623693616	7.951199808	8.147857556	7.775630727	8.190781646	6.752974364
7.253230737	7.009472515	7.281402384	7.575420636	7.062669222	7.949400121
9.325601172	9.286739242	8.903602263	9.057828929	8.857327323	9.057828929
10.56207051	10.27804811	9.929312889	10.33146686	10.46232362	10.61480751
15.06622261	15.18392271	14.9591327	15.24813401	15.09418019	15.54332231
10.40486524	10.52904685	10.2752886	10.6362571	10.2761618	10.10454015
7.983548525	7.870356606	6.809240364	7.527074182	8.066736566	7.983548525
7.149049263	7.013931768	7.457632404	7.989204111	7.166060222	7.585519525
12.81735602	12.95787308	13.47327752	12.72219022	12.65472241	12.71520416
11.9577766	10.97185531	12.23769992	10.52799495	11.76804793	11.66708917
8.53670504	8.20265299	8.627322343	8.76626204	8.122341	8.41382397
12.64265677	12.40735915	12.31089415	12.53911948	12.38063147	12.82153585
11.41778198	11.06573849	11.24816417	11.11714857	11.37847359	11.21016953
7.235366172	6.832363455	7.613528424	6.647414293	7.19003896	6.922922101
10.52799495	9.818059192	11.27499716	10.02061657	10.84705809	10.36781996
13.61616406	12.97555423	13.85099976	13.58996905	13.58209631	13.02006671
7.492937667	7.176878273	7.59435096	8.119288848	7.935468505	7.29540803
7.385541293	7.540434798	6.723002525	7.644277273	6.973155374	7.617890202
9.194378495	8.651919778	9.095162899	7.548285091	9.389466253	10.34100163
8.277763646	8.157153081	8.295009141	9.091299889	7.815211869	8.630877737
10.1309568	9.555296242	10.63266161	9.413321525	9.979092404	9.634846707
7.581982414	7.466652071	7.28903701	8.132293172	7.231823384	7.516138929
8.006915303	7.689906808	7.90331499	7.194433949	7.934739636	7.677613273
7.412806768	7.199742475	8.619848374	8.694292242	8.526649808	7.25490398
6.873088737	6.460273586	6.205939172	6.664178717	5.961025949	6.766018101
7.008714091	7.639990515	7.103815788	7.74961303	8.251922909	7.495760485
6.812413525	6.720541949	7.324608717	6.973155374	6.698319919	7.949071818
14.223913	14.14208591	14.31917195	14.23242051	14.49002723	14.36999747
9.951175646	9.843921515	9.441234879	9.626304798	9.331636121	9.232683343
7.040256253	7.251486172	6.669467525	7.248766182	6.508927202	6.393414081
8.489580414	8.337114525	7.743136949	7.809982667	8.018337859	7.677935313
12.34855768	12.33273925	12.13538265	12.04990566	12.06098883	12.44611991

7.301216929	6.955441212	8.07563398	6.936097616	7.533990333	7.816627253
14.72870348	14.68633385	14.4185289	14.64328928	14.66395575	14.91973608
12.84197228	12.81735602	12.71142619	12.7494647	12.77841715	12.96662998
9.006527424	8.735755485	8.476085384	8.530767747	8.606624646	8.620790505
12.63888703	12.32024961	12.93430142	11.94979793	12.55625498	12.66627862
10.06633871	10.25627793	10.08521343	10.61867663	10.66105788	9.099242404
8.503455465	8.178694051	8.431326525	8.546982556	8.305787747	8.84786598
12.82153585	12.87819387	12.68097339	12.94367069	12.77015535	12.94367069
9.156840283	9.083185818	9.01707996	9.231349182	8.739635162	11.84027237
9.241421747	9.195011616	9.531623293	9.511202364	8.185177828	8.363468485
11.93178106	11.60069924	11.64244603	11.66441291	11.85033795	12.07810634
6.165284626	6.484232444	5.868384869	6.513004101	5.939661242	6.248708515
7.100167172	6.518976111	6.53573598	7.132449434	6.857241172	5.870245838
7.477241869	7.552833101	7.300653717	7.520992313	7.019424707	8.061773232
9.783680727	9.815129384	9.701165495	9.545191596	9.819688798	9.686378667
9.573320202	9.361671283	10.18198959	8.859715222	9.299183616	10.18098661
11.71244686	11.80791686	11.66189432	11.69891546	11.51867768	11.68038318
13.47832004	13.26276274	13.16824615	13.09463792	13.07454735	13.76702458
14.24162707	14.62217632	14.17411554	14.90726461	14.31917195	14.46162431
12.30167305	12.47441306	12.35798465	12.02811467	12.42616911	12.65472241
7.489176	6.979690485	8.147147636	6.650783061	7.90609097	7.238133434
8.336257222	8.088117667	7.476910626	7.781509414	7.601643838	8.052783737
7.864861071	7.944406939	7.864861071	8.389653889	7.737987747	8.908935182
6.974373515	6.702734242	7.553468394	6.799383374	7.034867141	7.543396525
14.4427031	14.47046648	14.06476988	14.37933382	14.35197184	14.66395575
13.44849295	13.33155169	12.96241124	13.24400831	13.09977851	13.67554111
7.183932212	5.802713354	5.792455384	6.966976535	5.822496848	5.940786737
12.18792926	12.26775966	11.84529477	11.98999321	12.11216258	12.052647
10.1905069	9.377908	10.34311059	9.242021586	10.34311059	9.518503263
8.058246657	7.936282586	8.168196313	7.866306808	7.990677606	7.908308323
12.80166365	12.63102018	12.06397065	12.10135206	11.99535117	12.54589185
13.28559105	13.06967433	12.97555423	13.11515098	13.05428976	13.18348162
8.883993253	8.458294828	9.495686737	8.559011545	9.221852535	8.94874
6.500836152	7.481960152	6.487263202	7.038073394	7.099882485	6.599359394
11.47127063	11.06261544	11.238428	11.00496707	11.00754401	11.4413808
11.29597305	11.13240418	11.79405389	10.90192126	11.05959848	11.1453264
9.368875727	8.996148737	9.921211657	8.996667384	9.029402949	9.292435667
13.5964752	13.10484893	13.43523914	13.10484893	13.60269863	13.44126502
9.751080646	9.519086535	9.84311402	9.49510903	9.903372323	9.421700798
9.67665103	9.589694828	8.813595202	9.421700798	9.062220818	9.566949
13.52334642	13.30795266	13.35919327	13.22730043	13.24400831	13.56886427
11.51867768	10.52364746	11.14211434	10.06997663	11.06261544	9.828404283
6.828146828	6.638362242	6.383422788	6.625743253	6.448608091	6.595504071
6.816537596	7.11962903	6.620737576	6.58977503	7.52530399	7.040256253
9.441908576	9.123297364	9.698181576	8.927019859	9.320176919	9.130293909
6.377923929	6.362493667	6.751729354	6.165805071	6.538425424	6.490215273
7.428935273	7.392563535	7.716801838	7.382527051	7.931708101	7.681488071
13.36582475	13.09463792	13.20018245	13.02006671	13.12023156	13.50411017
13.18858184	12.29886409	13.20536123	12.23769992	12.83405786	11.11714857
14.70594079	14.39736901	14.66395575	13.70717091	14.27397716	13.79381649
7.714642404	8.311348051	7.668638899	8.418118394	8.969483	7.85507
7.971654859	7.721209061	8.837022465	7.957018646	8.398231626	7.963371182
6.578006293	7.091827414	6.787032949	6.947784242	7.409335747	6.74585002
7.608934747	7.412806768	7.600942677	7.376443525	7.569899606	7.428605818
6.352246303	5.888813515	6.014190303	6.504708505	6.347049828	6.12417499
12.96662998	12.77841715	12.73731843	12.65472241	12.78645268	12.57486869
11.43005301	11.04316622	11.86401838	10.080472	11.48655657	11.48258282
12.48124823	12.38794592	12.03068847	12.22341713	12.28369472	12.67700968

12.11841559	12.00086001	12.00354228	12.14789109	11.87445957	12.21630458
6.677606	7.646666444	6.444123263	6.779669273	7.593349828	5.789534242
6.400570727	7.221626808	6.423272566	7.060219081	6.895207929	6.319077384
10.03492192	9.808672222	9.740584727	9.818059192	9.517013525	10.73132595
7.25490398	5.95300801	5.88666196	6.79736498	6.696473434	6.104434657
14.38889659	14.53168901	14.07988992	14.4335752	14.36999747	14.39736901
14.60195877	14.74028573	14.25895571	14.70594079	14.68633385	14.94534759
14.82007331	14.29173122	14.91973608	14.9727616	14.77222727	14.47046648
12.51424883	12.47761486	11.97447062	12.08680505	12.12151457	12.5521346
13.84455043	13.73439002	13.54965001	13.80033164	13.82538175	14.15863941
6.563203242	6.409677646	7.597232061	7.828991111	7.60576001	6.407880727
8.176718545	7.997183333	8.198946162	7.944736172	7.801818071	8.452737606
14.46162431	14.25042984	14.1282639	14.40789433	14.3093669	14.5513462
9.362770111	9.311440606	9.067241768	8.678658293	8.690381253	9.454390232
13.34204398	13.13007894	13.3718573	13.27418186	13.46712151	13.42888134
8.116577384	8.225261616	8.190781646	8.222643394	8.566415323	8.508645727
9.707282667	9.419020677	9.855298394	9.222631263	9.523397737	9.002272697
8.136641818	7.734896879	8.531247657	7.636262586	8.299695162	8.384333949
7.189710394	7.413750677	6.876372364	6.878445485	6.957895121	7.226681909
11.1453264	11.10035391	10.94640834	11.10460602	10.96311364	11.64044206
11.28207759	11.44643795	11.05796986	11.30506756	11.21493335	11.25898056
7.925310818	7.512168657	7.161889071	7.401155333	6.859461465	7.214315212
9.595333525	9.246644323	9.423026242	9.229670424	9.215946081	9.592426051
13.97489728	13.91489381	13.83173935	13.99495772	13.95167504	14.23242051
6.518976111	6.814711535	7.095275111	7.419865051	6.559271747	6.654772152
11.45932673	11.15183093	10.92485902	11.12151875	11.10170724	11.39382595
9.551451798	9.512482222	9.614560172	9.726443586	9.759214424	9.333361616
12.90359001	12.89113428	12.38794592	12.71142619	12.73731843	12.8652792
7.556918475	6.909148313	7.765298909	6.79323798	7.420173404	7.899333596
12.50328361	12.4956701	12.21630458	12.39660011	12.42233701	12.51066587
8.472925747	8.296772869	8.085049192	8.163475424	7.958393384	7.855731
7.378508566	7.545335788	7.281724869	7.342871717	7.526167626	7.381652111
11.19817469	11.54871614	11.3733012	11.79870316	11.19114259	11.40495249
8.237365566	8.887684293	8.626355717	8.993505475	8.858692343	8.555429434
12.56315114	12.05813886	12.052647	12.27101	12.23191382	12.66167346
14.9847254	14.85646221	15.26374094	15.18392271	15.13975511	15.22815255
6.478552354	6.601226343	6.552305929	6.662284414	6.614504818	6.437442354
7.353560545	6.866852303	7.863532101	6.881752616	7.458795919	7.358075697
6.921891778	6.83457999	7.139668182	7.534885556	6.952257202	7.051418091
7.744459273	8.168587596	7.836138323	8.091725323	7.701029869	7.789573343
13.64083669	13.01016522	13.48990673	13.18348162	13.57574016	13.7211242
12.2031413	11.50425191	11.59021288	11.33100665	11.64848719	12.05489479
13.9071606	13.77352346	13.64083669	13.60269863	13.81266894	13.84455043
10.11051676	10.18563382	9.980047091	10.0053333	9.905108616	10.55213977
6.607797263	8.22437102	6.824838515	8.268937657	7.828584192	6.865318939
12.38794592	12.0838996	12.52819783	11.2345748	12.46797904	11.06877356
6.595312657	6.426872343	5.800174758	6.871668566	6.611655717	6.384073253
15.70163214	15.83165575	15.42966281	15.81050193	15.63778652	16.33688632
7.391655768	7.432999727	6.667348717	7.412191343	7.542064859	7.223246162
11.51465712	10.95849458	11.31661678	11.06732533	11.09089144	11.13879804
9.433258586	8.789132293	9.352487919	7.499887141	9.198999222	9.256767414
10.05970796	10.41626133	9.07419996	10.38329808	9.525471414	9.776839404
11.56291631	12.08680505	11.14866374	12.39348455	11.71443529	11.29597305
7.351279444	7.616841162	7.508388697	7.424653535	7.890755525	7.44049799
11.50814936	11.40725584	11.26262606	11.27695531	11.41605585	11.61117883
10.29542336	11.01807842	10.48185706	11.21493335	10.87092264	10.18198959
14.40789433	14.16629104	14.4335752	14.25042984	14.61202705	14.60195877
9.354902374	9.138451657	9.280305798	8.873520545	8.94874	9.263233333

10.31894523	9.763138212	10.95533553	9.283736939	10.12224713	9.885156737
14.93426708	14.52085241	15.12351424	14.00297944	14.48025691	13.96779242
14.7528858	14.32804886	14.82007331	13.85817003	14.5108222	13.78737352
9.910960495	9.658049545	10.11664425	9.589694828	9.653591273	9.779012071
9.308265131	8.877626323	9.572453101	10.1300726	9.250125475	9.159196141
9.174793152	9.495686737	9.233272283	9.853774707	9.786685869	9.629470697
7.712213273	7.543124535	7.438305869	7.546592828	7.654890798	7.708922879
6.259701051	6.367286556	6.629727051	6.413438525	7.21346404	6.403697939
6.429246364	6.159344697	6.100439616	6.017403131	5.981334404	6.729463253
7.076589626	6.430485283	7.432070859	6.395106727	6.430701515	5.764495354
7.328995606	7.12986801	7.647332323	7.511268929	7.383097848	7.522869182
7.300653717	7.367541707	7.051937434	6.522534343	6.944336869	7.928832091
7.715405939	7.897352323	7.826151263	7.791533949	7.942071303	7.527697455
6.871437091	6.670578394	7.300950758	6.98587896	7.140902202	6.711872081
9.955574404	10.65014074	9.890135131	10.23351878	10.09161001	9.410728505
8.661508737	8.907915899	8.52575703	8.410908071	8.91530399	9.155642525
9.196689051	9.546645747	9.507772687	9.795448596	9.46304199	9.307024131
15.39581235	14.90726461	15.58150095	14.49002723	15.41210544	14.89443037
7.414093131	7.56574702	7.331138333	7.480348535	7.632861657	7.376746737
7.017519192	7.100167172	6.854191545	7.104930788	7.152958919	7.032021525
7.321884212	6.896472707	7.434843313	6.946968899	7.280239242	7.183932212
6.64329102	6.51319704	6.466965869	6.910693596	6.339459364	6.277527444
7.07551896	7.140269465	7.088250616	7.862160495	6.987890152	7.15072903
8.245649515	8.396532182	7.957018646	7.943433747	8.135850465	8.011239071
8.575095707	8.33024502	8.943348051	8.064713051	8.492322828	8.837022465
7.612294131	7.675893758	7.834787162	7.516462556	7.44112097	7.680845586
7.333224889	7.354199768	6.492200283	7.843855828	7.087210828	6.772272192
10.34626898	10.68452674	10.19418334	11.22947614	10.8405808	10.23752804
8.504327141	8.163053768	9.298556212	8.170954818	8.766842081	8.314030071
6.988399394	6.839237455	6.748092424	7.555333263	6.538200697	7.072036626
10.21484784	10.93494087	11.06433979	11.42787377	11.31267384	10.91641235
7.247605232	7.518430525	7.470329323	7.950449798	7.295094879	7.401750182
9.521972354	9.354289121	8.999492384	9.271759586	9.142159707	9.237664879
9.934615404	9.993897848	9.726443586	9.773700283	9.806332162	9.938475404
11.68484165	11.71042621	11.51008986	11.46936418	11.5737214	11.89876792
10.54190997	10.60507136	10.10542249	10.34100163	10.2829717	10.62142174
13.03496614	13.32021166	13.35359128	13.9071606	13.30255942	13.49745762
7.986535808	7.714004283	8.41598997	7.581982414	8.174871818	8.409136758
13.27940818	13.44126502	13.17281418	13.98081649	13.32021166	13.62106704
9.21067496	8.057421596	9.769223525	8.079973212	9.094691273	10.00695165
12.21991364	12.51766186	12.40343679	12.23505088	12.72219022	12.17829278
15.10875812	15.29549959	14.61202705	15.41210544	14.86987024	15.52328152
7.118512586	7.236577444	8.295436051	8.216297535	8.070136677	7.967920667
6.968936475	6.964624475	7.256041222	6.920666222	7.317897131	6.853461566
7.475955232	7.722297051	7.256596263	6.894391313	7.418614091	6.365147848
7.888681737	7.674820101	8.233006697	7.68296396	7.937009	8.073294879
7.743136949	8.165380303	8.203151141	8.39867397	7.784888444	7.968691808
7.901142576	7.640998838	9.635582141	7.18283101	7.496977949	8.522641273
7.311629869	7.435709141	7.910617626	7.244122677	7.726017717	7.252038162
8.887684293	8.675789182	8.379510323	8.516311424	8.637513768	8.695335444
8.015705778	8.289599333	7.916391202	8.670884566	7.841440677	8.098747556
7.125696242	7.21150504	8.24281297	7.395300939	7.141717727	6.992656859
8.307893798	8.926362727	8.290726727	9.052474394	8.503455465	8.637033606
10.37894268	10.34626898	10.59418919	10.6313464	10.2600862	10.41330878
11.36610866	12.21630458	11.50425191	12.0750029	11.76336308	11.55322016
8.718321818	8.815152444	8.348974465	8.318668263	9.222631263	8.988033424
11.81018832	11.50814936	12.39348455	11.62059475	11.86125412	11.8164662
11.65918316	11.81439214	12.08680505	12.60022648	11.68243664	12.13538265

10.34193651	9.899969596	10.52799495	9.850553616	10.30358984	10.16642595
9.337862091	8.831800202	9.29794999	8.545617303	9.058356636	8.478760515
7.587956192	8.197805768	7.774939586	8.001277303	8.01013803	7.823737283
10.10542249	10.09339803	10.2187154	10.45628541	9.895061889	9.903372323
11.64637724	12.08943903	11.60266156	11.88909817	11.77053658	11.542568
11.95514832	10.25127201	11.34732253	10.26729954	12.53197264	10.69099899
8.662816202	7.57049196	8.93638097	7.038360253	8.106019798	9.132651727
7.439228222	7.261194687	7.852302727	7.46750399	7.582311101	7.751275798
7.924885333	8.017912586	7.450590374	7.824443343	7.65784001	7.994566778
8.342773172	8.422204465	8.511305758	9.019287687	8.228946717	8.080354404
7.973992111	8.341518747	6.257095384	7.552094283	8.52575703	9.038849273
8.53537201	8.140977576	8.323157747	8.77608297	8.93587504	7.907197636
9.920430606	9.734703111	10.00985275	9.514496606	9.693515768	9.848945263
8.373919657	7.83046296	9.230202616	8.037984616	8.356302828	8.628622212
10.24511785	10.65246846	11.18471248	10.63391466	11.36979611	9.549314313
10.12314196	9.811091061	10.16165443	9.879566364	9.692043475	9.738398616
7.510343899	7.147201253	7.681488071	7.381324374	7.436336172	7.246997929
7.166695253	6.583924121	7.508747081	6.469182939	6.998209475	7.24440804
6.195258121	6.183644212	6.615899091	5.800862303	5.835234071	6.617782121
9.149195859	9.145078374	8.817191758	8.610845737	8.872352707	9.299183616
8.27861104	7.93027604	8.338686465	8.617420111	8.35796399	7.488273475
6.87945998	7.288414848	6.987599808	7.807154444	7.083314707	6.978866758
11.19966733	11.00620373	10.3986043	10.90606985	10.50543333	11.15503783
8.733447455	9.136296333	8.460053242	9.001802606	8.790130253	8.822853162
7.133007667	6.922412606	7.459115707	7.325469747	7.276277889	6.925960677
7.964547263	7.963371182	8.35372597	8.198946162	8.060252263	7.821625848
11.17213305	10.73911267	11.29076429	11.14707085	10.86447048	11.33477589
7.928832091	7.627670859	7.892936636	7.550845909	7.588938485	7.576363505
7.149343495	7.502072596	7.51340999	8.586230848	7.476561202	7.273224596
6.465818222	6.052626778	6.766293242	6.670809131	6.655494515	6.343446081
8.949784505	8.439612798	9.004940778	7.639662323	9.051957646	9.97079498
8.645985424	8.57971698	8.182386152	8.600356081	8.648450303	8.582147707
9.91449603	9.921211657	10.36465285	10.01257663	10.02161965	10.27905981
13.33661988	12.43600068	13.65450155	13.00101781	13.03496614	13.20536123
10.57885616	11.03452834	10.23752804	11.1502402	10.66945215	10.687913
7.594027929	7.793711495	7.382821899	7.892277636	7.920144808	7.402347162
9.701165495	9.802496737	10.38001468	10.60863639	10.15783547	9.533033172
11.17910752	11.29597305	11.41071085	11.71659127	11.18636156	11.43208235
10.35385551	10.62142174	10.080472	10.70534521	10.38531322	10.17224075
6.941957697	7.028313949	7.298118263	6.916684768	7.132177788	7.308145939
8.572163455	8.433042727	8.643636162	8.529417101	8.647073404	8.657806242
8.297224525	8.31049196	8.365106485	8.162707626	7.972411364	8.230588212
8.773471869	8.759332636	8.765233303	8.219832939	8.890373556	8.661956242
11.94234713	11.90674449	11.65261158	12.49965499	11.70348036	11.73070023
9.315671101	9.316319051	9.191849071	9.890949131	9.35067002	9.387656788
8.841037687	8.586731747	9.638309758	8.660166475	8.777572838	9.520598525
6.261014313	6.02432797	6.384961778	5.880950253	6.013673768	6.301916869
10.4549623	11.15966862	10.69222123	11.308834	10.74558579	10.46442497
7.667309091	7.400015081	8.180785838	7.719466677	7.702311758	7.863889121
6.341475172	6.58529104	6.116267859	6.330516949	6.570263495	6.558125737
8.060661899	8.023099172	8.091725323	7.995673758	7.985458788	7.858214495
10.06450841	9.716648152	10.18942995	9.412044242	10.28795108	10.30695028
8.509502414	9.081281455	8.188379657	9.286110869	8.877626323	8.667554273
8.003479939	7.493254434	8.840590677	7.380085202	8.165380303	8.397394545
10.36134852	9.857940545	10.39296715	9.715870253	10.35385551	10.2761618
12.8293171	12.90783816	12.88265743	13.83173935	12.91656586	12.85589012
7.540776061	7.651926253	7.350971899	7.913459869	7.295703091	7.866966485
8.300483646	9.175472828	8.849981545	9.609756293	8.756886111	8.545617303

8.527198758	8.482406475	8.393536232	8.494982343	8.170586434	8.692466525
10.07580535	9.184527788	10.07479617	9.170513566	9.319510202	9.169862626
8.180413081	8.268510495	8.637033606	8.285708747	8.657806242	7.633913808
9.02886299	9.128533354	9.308918081	9.643287657	9.301561	8.987499273
7.965660091	7.756889051	7.906425364	7.643273222	8.34368697	7.716801838
9.263233333	9.572453101	9.486281202	9.991229606	9.429506515	9.715215101
8.138156535	8.358418384	8.152087687	8.625920364	8.030181424	8.128726778
10.15186663	10.35178281	10.19136651	10.64738653	10.10187506	10.71731517
10.89224764	10.54800525	9.373397364	10.0060683	10.96886286	10.29542336
9.964343626	10.12610261	9.541041677	9.762542495	9.846508495	9.93187304
10.47820756	10.92190902	10.74305691	11.7349036	11.02403513	11.19447199
10.57159251	10.5431016	10.04776272	10.2026692	10.47941256	10.32849396
9.459664354	9.403514808	9.538263677	9.354289121	9.507772687	9.574050242
8.288355808	8.585836293	8.397853727	8.717867636	8.933684374	8.573273283
8.494130061	8.200961636	9.017695354	8.298896434	8.520243879	8.711304737
6.915665626	6.72626003	6.94985404	6.822905505	7.147555404	6.793900848
6.411917909	7.428304182	6.292920576	7.447717303	6.990260788	6.923412444
7.913871889	8.831201768	8.01602699	9.017695354	8.503920141	8.515864333
6.384310424	6.607010823	6.873334899	5.888636667	6.628047111	6.791503909
8.923576949	8.344860929	9.776839404	8.861258687	8.921011566	9.026636737
7.08018898	6.959689121	7.074987333	7.003715121	6.927779576	7.107702061
8.214296636	8.093077828	8.196571586	8.343220798	8.111719586	8.239474646
7.232151747	7.118205404	7.723687384	8.009440313	8.077230061	7.286117455
5.76351104	5.869182889	6.88247998	7.205228253	6.892837172	5.76213197
9.849671929	9.412634061	9.679645121	8.700606657	9.867106131	9.49510903
11.11560115	10.53737043	11.69658017	10.11849919	11.13240418	11.50632759
7.599870798	7.688011354	7.876138384	8.158321101	7.735579636	7.664275131
8.735287485	8.936938576	8.898709273	9.140978061	8.696739152	8.805485081
10.06255975	10.34514895	9.753529758	10.6240143	10.18563382	10.04511943
7.241813919	8.023506091	7.336476646	7.507667889	7.398020586	6.671323232
7.93437196	8.21550303	7.619228051	8.11047698	8.03364202	8.07651197
8.510413414	8.395296687	8.429582192	8.341977727	8.325316889	8.267698071
9.236429667	9.158023	8.844732879	9.195011616	9.231349182	9.204345657
7.155513182	7.203788636	7.608347495	7.390752071	7.659186848	7.034613909
6.497644434	6.178813899	6.708053838	6.092696515	6.417620515	7.127947263
7.334066545	7.412474121	7.045230232	6.951451	7.078303869	7.32338401
8.495447152	8.639417101	9.470840162	9.623794152	9.666877556	8.945002717
12.58612865	12.4571909	12.69316533	12.15440913	12.71830686	12.24933389
6.834834778	6.537747374	7.014842626	6.322079051	6.647414293	6.919083737
11.911688	11.79171062	11.65052704	12.09804166	12.16036216	11.4090051
7.424653535	7.451812333	7.561051303	7.165241424	8.276438626	7.342557798
7.666324061	7.819446808	7.624016909	7.860050667	7.875022121	7.998937818
7.060512141	6.915952788	7.695265798	6.997357515	7.275223848	7.51061902
8.220225172	9.291393586	8.546056747	8.671294253	8.373514202	8.611286687
7.787863889	7.840025778	8.213079374	7.575754657	8.019434586	8.012010707
8.363468485	9.168828475	7.865908465	9.324388263	8.612168455	8.768863636
14.84412351	14.59173737	14.6970922	14.1282639	14.58095582	13.09977851
8.808345061	9.107388182	8.520754616	8.718769576	8.85785699	9.015966535
7.512168657	7.544367232	7.760294414	7.321250939	7.645645939	7.159453505
7.472789818	7.378508566	7.429246232	7.287262414	7.801388869	7.089371313
7.074987333	6.564717364	6.816827283	6.927779576	6.561721071	6.850381687
8.243637242	8.186741323	8.371674091	8.998971485	8.006915303	8.077645899
7.550195808	7.520992313	7.314300525	8.094989869	7.792995667	7.454874889
6.932737172	6.863661697	7.20918101	6.473654737	6.966460535	6.407245091
8.137069859	7.25402203	7.471219899	8.204737434	6.875689354	7.636934919
7.493565131	7.185402798	8.055566596	7.677935313	8.291610121	8.074096707
7.688949081	8.108523939	7.850535525	8.633848111	8.949784505	8.095330333
8.105207222	8.251922909	7.943134	8.130993828	8.478760515	8.418959303

9.723272263	8.959130566	9.011546121	8.133538364	8.877161475	8.567801737
8.053207788	7.561964414	8.547862646	7.849463182	8.198946162	8.36093298
8.422204465	7.816254515	8.890373556	7.976858394	8.655437626	8.559456798
6.594580697	6.530171141	6.839911636	6.808010384	6.886756677	6.722483515
11.73972102	11.07493153	12.31419772	10.93789932	11.77802923	12.33576886
15.24813401	14.86987024	15.70163214	14.78379092	15.15443825	14.83117585
6.583688333	6.809240364	6.605935465	6.216559596	6.276797273	6.315191152
6.982135444	7.308145939	7.378186273	7.872008071	7.344596535	7.375790182
11.35475345	11.36979611	11.59212007	10.65246846	10.63870636	11.39594259
5.97237099	5.879067162	6.105835768	5.990876141	5.97184804	5.868660727
9.728754818	9.517771202	9.719830172	8.702574091	9.259301556	8.340252101
11.69440164	11.97447062	11.74439188	12.12151457	11.58421758	12.17513939
7.28082202	7.320252869	7.686444293	7.701686677	7.068134081	7.339519202
10.49713487	10.14713571	10.4549623	10.1309568	10.6362571	10.37894268
8.328992859	8.913024061	8.263866778	8.762202232	8.510413414	9.171164091
7.456429202	7.659186848	6.174477505	6.990995828	6.152834101	6.153019566
11.00053539	10.62280049	10.76471511	10.77507269	10.70534521	10.88546605
8.612168455	7.856391758	8.422643323	7.950135394	8.099112939	8.517695303
8.333227545	8.021984222	8.611710212	8.236986626	8.488340929	8.665165081
8.047188455	7.954939636	7.65457904	8.639872707	7.918105152	8.093077828
13.91489381	13.6823979	14.23242051	13.73439002	14.25895571	13.48401144
7.989204111	7.937868929	8.128726778	7.554702939	7.854005263	7.84876403
9.392652323	9.315671101	9.041236566	9.245445293	9.354902374	9.193817232
8.420387707	8.379056919	8.445297313	8.384837869	8.082778869	8.666601818
8.271405859	7.973586899	7.665645141	7.84876403	8.063461202	7.985767212
8.097566687	8.534432515	8.546982556	9.139724848	8.130283051	8.218887434
12.9851923	12.02240324	13.2158469	11.75662781	12.90783816	12.44950322
9.440510152	9.039454747	9.046362323	8.552208182	8.882379495	8.614442374
7.99823603	8.131862263	7.959488687	8.181963182	7.718373848	8.111719586
9.592426051	9.566949	9.248396293	9.359783616	9.421700798	9.710962273
8.351752182	8.532119949	8.355847778	8.466446051	8.282900293	8.33796001
8.312729141	8.055916879	8.708467242	8.484240687	8.333749495	8.789132293
8.246087697	7.695611414	8.609511	7.418256545	8.057421596	8.683580283
11.00183361	11.23280633	11.15966862	10.96000427	11.21338774	10.43757507
8.660608859	9.281923404	8.653906556	9.428845576	9.163332636	8.838951081
6.468041212	6.244753081	6.524259657	5.947893172	6.687954879	5.788961677
14.32804886	13.48990673	14.37933382	13.03957854	13.6823979	12.26475658
6.948313626	7.010236465	7.107702061	7.257203303	7.431765758	6.796104869
12.78645268	13.74093847	12.85048632	13.93694444	12.77454263	12.97977545
8.485631939	7.84876403	8.767368071	7.75479003	8.141366242	7.868007081
12.8652792	14.34357305	13.32021166	14.02572128	13.42153085	13.86604428
7.641683626	7.664275131	7.764199131	7.154937303	7.896287434	7.919201576
8.266388465	8.254082232	7.810980737	7.832217929	8.178694051	7.999327475
13.93694444	14.64328928	14.03424604	14.67552064	14.07245549	14.53168901
7.100441616	7.219623727	6.992392121	7.218399283	7.253764222	6.976769172
8.563941778	8.767872848	8.442699879	8.639417101	8.608532485	8.210186616
10.13671639	10.33696433	10.34193651	10.86173253	10.1684149	10.1985269
12.41033676	11.05651949	12.47441306	10.88302044	11.70138789	9.877792838
9.62302801	9.779738	9.517013525	9.968215758	9.646274414	9.382882596
8.595703707	8.169382444	8.616914869	7.807908545	8.337114525	8.292068253
6.552973232	7.01811702	6.296516586	6.712828707	6.585712788	6.736085162
8.006153172	7.957018646	7.429567525	8.078044465	7.534302667	7.546918172
7.317590788	7.352546465	7.36076402	6.931701566	6.957403667	7.154007354
9.435122859	9.202552434	9.443424081	9.341818545	9.257979848	9.415217505
7.773938747	7.726017717	8.419866586	8.740682	8.381754576	8.228946717
7.000811505	7.019921606	7.047046253	6.698531303	7.304711495	6.366338828
7.320621818	7.800624061	8.134366646	7.080525788	6.482290162	8.247392788
14.64328928	14.76275492	15.05177986	14.52085241	15.31193978	14.34357305

8.347717242	7.945111576	7.85507	7.724369343	7.694916828	7.946541949
7.774283859	8.321919424	7.342557798	8.279462	7.77977198	7.940254566
10.98037262	11.60266156	10.67428006	11.63401423	11.50425191	11.52645916
10.45754412	10.77890896	10.13580598	10.41123643	10.58469265	10.50914282
9.922126404	10.19757035	9.603529667	10.04963797	10.02439619	10.08325108
6.538425424	6.491493939	6.845301404	6.667348717	6.775968394	6.515215919
10.14534091	9.845595727	10.11246778	9.548618051	10.10454015	9.954681949
7.138827606	7.10439698	7.178076949	7.233039222	6.901187545	7.295703091
9.311440606	9.361074667	9.179819687	9.109760465	9.102676354	9.01707996
9.831834717	9.950180667	9.610538374	9.556000646	9.721958051	9.946747141
9.863774293	10.07479617	9.878686616	9.748306121	9.800083455	9.669847545
9.010430586	8.858269384	9.101086141	9.006527424	8.983202444	8.949261586
11.00902763	10.51291939	11.38391359	10.35069541	10.83223099	11.29399858
7.724049626	7.706168626	7.431067202	7.528640475	7.632536929	7.695912303
6.201940414	6.514559495	6.530171141	6.850608899	6.007160364	6.249071646
9.01707996	9.072403576	8.621689657	8.971160737	9.187808616	8.968910758
11.08435313	10.86305205	10.64128512	10.74172297	10.8193093	11.07647857
10.60743282	10.77507269	10.5104744	10.4655823	10.61371787	10.49830212
9.824540899	9.439397061	9.989520798	9.510536	9.783680727	10.09682592
7.274624242	7.338325414	7.025264747	7.083894141	6.927259889	7.279669313
11.59654933	11.7349036	11.53473316	11.63837311	11.6137143	11.69658017
9.904237354	9.87177298	9.598893818	9.406122899	9.626304798	9.759214424
9.885156737	10.042425	9.436357141	9.881912677	9.874496818	9.969141677
10.33476777	10.2187154	9.992117576	10.01703805	10.1309568	10.07388225
7.510027525	7.370661465	7.985767212	7.810269677	7.538207606	7.562627657
7.265083586	7.854005263	7.478150263	8.845785121	8.104133364	8.362142131
6.655986263	7.218159515	6.618022394	6.793900848	7.356542485	7.073657889
7.355080212	7.280525364	7.503692152	7.303836939	7.783110646	7.326942212
7.150454879	7.51247098	7.033114263	7.917403434	7.290498626	7.047335818
7.624419899	7.19832496	7.882525919	7.51340999	7.044743657	6.602227
8.972363838	8.908475283	9.356058495	9.190205293	8.892970697	8.911610283
9.065009323	8.187968455	9.189590182	9.000069889	8.733083212	9.056533626
7.638629778	7.804724687	7.367866364	7.677613273	7.909412687	7.59656001
8.320817869	8.297224525	8.57597697	8.185177828	8.741130788	8.39402401
8.635209293	9.10215096	8.690381253	9.551451798	8.827377505	9.187808616
7.977207111	8.054318061	7.781509414	8.282438737	7.893631939	7.832540091
7.464704768	6.978374222	7.879657162	6.506514626	7.45523802	7.189144101
10.49140257	10.27236099	10.56548536	10.15783547	10.1985269	9.586238192
10.15086094	10.94640834	10.8405808	11.07337622	10.17122401	10.70425349
9.808672222	10.79686873	10.0053333	10.16262583	10.52364746	9.834210131
11.03008027	10.77637516	10.49140257	10.6488688	10.85782556	10.52799495
10.59924244	10.30695028	10.19236777	10.10454015	10.38738452	10.06904603
9.329342374	9.016491202	9.299916465	8.563941778	9.139724848	9.365016101
9.698896848	9.460382818	9.596088263	9.526870273	9.659537192	9.37540303
9.963504081	9.715215101	9.719000626	10.00002479	9.945028121	9.591816061
10.75865445	10.3810842	10.3025572	10.37346225	10.61743608	10.0406788
9.876880455	9.707282667	9.557589465	9.815129384	9.917043434	9.445197818
12.25572441	12.24933389	11.19817469	11.75215821	11.94234713	11.34381562
10.26116661	9.847335909	10.03492192	9.607593848	9.955574404	9.701165495
10.81540317	11.64848719	10.62895885	11.29230964	10.73788216	11.18800276
8.502509434	8.269818727	8.159907626	8.208692758	8.416773909	8.957404525
7.116617232	6.626873192	7.416977434	6.84778002	7.142620404	7.113720848
7.338325414	7.247912131	7.308713444	7.395598354	7.219017232	7.408681949
11.08107282	10.69354222	11.11081747	11.00902763	10.81664077	11.54470354
7.864224778	7.864861071	6.826151202	7.061925202	7.339214566	7.022843394
8.380307172	8.022720303	8.173308798	8.076118121	8.137860071	8.110083838
9.217871889	9.095762	9.359207242	9.899969596	9.317615606	9.246644323
7.557888444	7.61520499	7.160253414	7.473746677	7.487078798	7.593664717

8.391460737	8.087781242	8.513601889	7.911629535	8.234232515	8.051625212
7.59435096	8.009440313	7.492628778	7.910617626	7.609879768	7.924502232
6.940479424	7.672658859	7.627997354	7.56409603	8.794788636	7.074397545
9.89348604	9.416442616	10.14131493	8.891390919	9.880317061	9.981718727
7.909031556	7.465665798	8.257984293	6.920918808	7.763504859	8.169730152
7.176878273	7.100441616	7.23119303	7.247290424	7.25402203	7.142895121
10.83223099	11.29399858	12.81379045	12.16279806	11.27695531	11.15183093
10.09254304	10.37026232	10.49348589	10.53842125	9.907546465	10.12909315
11.49839615	11.96636849	11.64637724	12.52130815	11.59654933	11.97196754
8.042161798	7.993077394	8.110083838	8.633351606	8.094605657	7.971654859
12.13538265	11.05033578	12.01673563	11.08936677	11.90384839	11.81018832
6.662284414	6.912798808	6.958715091	6.782883697	7.95873603	6.693002899
8.48093102	9.189590182	8.357077121	8.559975162	7.650231051	8.984797889
7.130781323	7.256596263	7.935081758	6.887014152	7.49893601	7.401460495
8.900236364	9.058895657	9.089024899	9.069558111	8.823394192	9.140978061
9.495686737	8.876209859	8.552719747	9.303966081	7.073141899	7.044506424
9.39974104	8.951989212	9.359783616	8.906910121	9.137373131	8.465000141
8.036387556	8.069395848	8.273483707	8.59942799	7.771540485	7.898974697
10.78705857	9.935661677	10.93494087	9.660200838	10.84176532	10.84705809
9.923012889	9.491039162	10.36134852	9.656571869	10.14534091	9.227886646
10.44195016	10.52585606	10.42062148	10.5336638	10.47123164	10.20164867
9.829119768	9.142159707	9.968215758	9.133918929	10.05688349	8.78563097
6.76700904	7.482291222	7.46337499	6.778470374	7.90505396	6.099259566
8.170586434	8.552719747	8.292509485	9.037153444	8.255317818	9.412634061
8.749910828	8.910531141	9.097596465	8.807484626	8.814101707	7.744142424
8.305787747	8.042974687	8.140538697	7.991473828	7.819446808	8.18280501
11.60888892	11.5612151	11.8358519	12.3543152	11.66934136	11.91651973
9.793942798	9.490338525	10.02061657	9.768390455	9.825199323	9.771430818
9.062220818	9.013314737	8.718769576	8.885078273	9.095162899	8.907429495
10.26832521	9.704210374	10.31193963	9.593075545	10.25811169	8.878201818
7.012571859	6.912541192	7.122424869	6.999250869	6.590229374	6.981086404
10.70425349	10.31894523	11.21816154	9.195534384	10.72900488	11.27306561
8.971160737	8.932093424	6.476196333	7.272016424	6.282012172	6.819416596
9.102676354	8.797743222	9.163332636	8.921011566	8.801904485	9.12042397
8.147481596	8.025864485	7.667309091	7.98690202	8.140128606	7.929905606
9.370832535	8.865915939	9.898158111	9.081281455	8.957404525	9.35858599
13.57574016	13.33661988	13.40302459	13.30255942	13.34204398	13.2504616
9.975431768	9.611261414	8.975838535	9.347406172	9.112697707	9.362770111
9.119222505	9.152615889	8.665646899	9.804037697	9.392082222	9.384699788
9.880317061	10.11555853	9.90675497	10.41426339	9.581418808	9.770750222
9.690534253	9.590351455	9.227275152	9.373397364	9.57872197	9.650129091
11.14707085	11.26446827	10.90192126	11.1060681	11.13400706	11.24147284
8.276014121	8.326884343	8.404396899	8.323598384	8.411696677	8.385691051
7.551492101	8.319901444	8.184399818	9.559682182	9.010430586	7.81270001
12.48835313	13.38447499	12.54268587	13.82538175	13.11515098	12.7452022
7.830796253	7.629344343	8.325717687	7.526167626	8.010523545	7.875808798
10.50166798	10.66105788	10.81280665	9.52752797	10.48310631	10.1684149
8.782655051	8.434806414	8.950874253	8.695776131	9.056062667	8.572163455
9.933588354	9.423026242	10.35284213	9.744663444	10.13375911	9.878686616
9.583475303	9.660861778	9.343457394	9.36570098	9.444645091	9.584100455
12.06397065	11.72126504	12.19398134	11.36065784	12.3543152	12.87819387
9.860587788	9.085558838	10.55213977	8.992473788	9.928455091	10.5199714
6.827921778	6.924744576	6.618749384	7.016958768	6.586655667	6.753729404
7.31937996	7.550195808	7.801388869	7.016433495	8.262596626	7.455822202
9.93663897	9.742895778	9.935661677	9.821914929	9.975431768	9.94767803
8.997807768	8.808928141	9.026117657	8.922082808	8.892401576	8.804952828
9.950180667	9.826793596	10.33252085	10.40592026	9.878686616	10.97767761
8.800782677	9.008373061	8.616405606	9.039989364	9.013314737	9.150811232

13.16824615	13.39037726	12.92542832	13.57574016	13.61616406	13.5160991
8.776574232	8.777050384	9.06557301	9.520598525	8.931557869	9.798623747
6.433023606	6.370955485	6.252758313	6.68968302	6.430485283	7.098484101
8.293340606	8.404822414	8.210602626	8.111336758	8.112161727	8.388388636
12.43944324	12.89513437	12.92937388	13.25686564	12.38794592	12.80166365
14.56207529	15.06622261	14.5513462	15.56435861	14.7528858	14.85646221
9.776839404	10.34193651	10.10454015	10.65987528	9.911926424	10.13958591
7.477539293	7.380085202	7.687700545	7.317590788	7.925310818	7.666324061
9.992988556	9.998242414	10.2600862	10.44094498	10.37467131	10.74819911
10.71390019	10.87637444	11.19447199	10.78014639	11.08435313	10.52689599
7.762451808	7.847720859	7.822322859	7.977207111	8.470647758	7.383097848
6.435479606	6.266853091	6.619525869	6.395789414	6.382613596	6.584832545
9.509876273	9.626304798	9.381544081	9.080123545	9.913634949	8.543234566
9.182731869	9.709426838	9.806332162	10.12697515	9.290785283	9.937540263
9.019731374	9.333986848	8.65497299	9.223302313	9.356058495	8.911109919
7.11316801	7.248451727	6.703725465	6.846305687	6.911480061	7.042386798
7.282365313	7.302736424	6.897750404	7.133298475	6.789537253	7.536554758
7.674109172	7.241813919	8.02125603	7.12483998	7.731542758	8.126253929
10.5041569	10.98350754	10.57996368	10.73014715	10.52476206	10.34626898
11.0859227	11.58621592	11.22600179	11.4413808	11.11714857	10.48897537
12.46055504	12.22629088	12.63500423	12.68484185	12.45390404	11.88375452
8.690381253	8.955359253	8.951989212	8.526649808	8.835493283	8.064713051
9.277992232	10.30358984	10.4700758	10.42512206	10.99469779	9.568406747
8.272620253	8.702574091	8.063008152	8.760676242	8.149788848	8.088528121
13.40990013	13.02518615	13.46712151	12.86086109	13.15645152	13.23341018
7.070939121	7.176635455	6.788765101	7.081850444	6.934051768	7.226432323
10.14713571	9.962571606	9.843921515	10.02362526	9.749660394	9.800083455
11.5612151	10.3548828	11.56291631	10.32946621	10.87518762	10.55602586
10.77103399	10.83501466	10.43998577	10.56908161	10.4687107	10.94640834
11.85617239	11.76588718	11.76588718	11.83330316	11.87175588	12.14191765
7.215458101	7.220481808	7.016958768	7.170007293	7.310482798	7.216096788
10.53141254	10.73517618	10.37346225	10.77637516	10.40701089	10.42622906
10.80239406	10.33476777	10.8605042	10.20164867	10.76609029	10.37026232
11.26815786	11.2786408	11.0859227	10.97767761	10.90466263	11.41778198
9.070661273	9.120933788	8.650965909	9.224923687	9.085558838	8.959665061
11.43938222	11.12935449	11.64848719	10.97476091	11.2278827	11.14057221
9.294355394	8.906407303	9.347406172	9.291911616	8.951513343	9.558255162
11.02700786	10.72384747	11.02700786	10.64502212	10.8254891	10.64128512
5.94215104	5.762637848	6.139423374	6.830332131	5.766330091	5.767242404
7.82475102	7.469634172	7.998937818	7.466342606	7.781852747	7.996393263
8.581684657	8.656835556	8.505287909	8.60560303	8.586230848	8.774537848
7.324608717	7.614497747	7.266262606	7.776629576	7.364550071	7.407756293
7.690510313	7.989899475	7.807154444	7.245604273	7.899682051	7.632188616
9.490338525	9.37540303	9.50650501	8.929972798	9.983540212	9.485668061
8.554027364	8.150892606	7.877487515	8.50473503	8.443547162	9.086722374
7.915639949	7.841729333	7.698300838	7.879657162	7.388278253	8.015705778
8.134366646	7.449616394	8.018337859	7.938531869	7.635267465	7.805068949
13.81935116	13.36582475	13.78027417	12.72610186	13.50411017	12.6076243
12.797444	12.55983323	13.1944969	12.71830686	13.13522491	11.52824
8.384837869	8.721926778	8.572701232	7.663954061	8.200961636	8.571676263
9.658772677	10.57028524	9.728754818	11.11560115	11.17750487	9.589694828
8.507211606	7.327495141	8.139691131	7.830100949	8.369167818	8.363862202
6.602666131	6.586655667	7.053327354	6.615401818	6.562194212	6.957146182
10.83501466	11.46724679	10.77103399	11.71895032	11.21668249	11.00754401
7.759936788	7.043720485	8.079553929	7.453021566	7.599486586	7.457078101
12.92937388	13.27418186	12.59668127	13.38447499	13.02518615	12.81379045
8.152794889	7.956286283	8.684081152	8.238629646	8.35018502	8.53579602
9.402230293	9.280305798	9.40167604	9.956562586	9.625623061	9.329342374

6.150133152	5.882632626	6.072186929	6.411067717	6.251546414	5.799885707
9.687048303	9.793126455	9.475206131	9.613206141	9.698181576	9.644080808
11.70138789	12.26475658	11.48655657	11.63174178	12.10951308	11.80104424
8.82789604	8.829514889	8.64224801	9.146178758	8.817758687	8.817758687
7.254644071	7.188870657	7.031727121	7.051685909	7.127947263	7.131863879
6.100757848	5.935919465	6.083800768	5.987835697	6.099546667	6.069800253
7.21064097	7.284431525	6.778730485	7.320621818	6.663425212	7.045230232
12.39660011	12.43279956	12.61761735	12.76628311	12.24933389	12.3613163
9.713858919	8.894491141	9.450568111	8.942313313	9.829119768	9.484840202
7.640655485	7.738613697	7.459710222	7.797844434	7.631600293	7.763186
8.445759657	8.155023263	7.998587576	8.265965545	8.090157434	8.049354232
8.822318	8.766842081	8.572163455	8.572163455	8.434806414	8.733447455
10.54692936	10.10454015	10.46232362	9.831087364	10.36349344	9.799317404
11.74670945	11.84027237	11.8164662	11.10170724	11.89361272	11.35080354
8.354182626	8.058592364	8.880853535	8.339115859	8.213832515	8.402589283
16.09492117	16.12384164	15.97330865	15.78787594	16.12384164	16.57174008
7.984288364	7.708922879	8.204737434	7.302736424	8.03490996	7.927113394
8.481851586	8.229734556	8.878776172	7.882525919	8.64752602	8.194999071
11.05796986	11.22456459	11.83813795	11.0859227	11.60266156	10.46108665
7.603389051	7.907197636	7.320252869	7.977952253	7.664275131	8.03179299
6.763870838	6.595504071	6.983154111	6.830578646	6.929929141	6.718530879
9.34289298	9.540446515	9.570470929	10.14044783	9.588958727	9.530205505
8.373514202	8.34368697	8.539776737	8.457898556	8.138519566	8.629474212
9.567720788	9.666877556	9.899969596	9.973623707	9.803356646	9.500664495
10.24139873	10.55474917	10.14809211	10.78705857	10.24727703	9.985290333
10.44094498	10.88809182	10.39968902	10.78551178	10.31480319	10.63516273
8.823394192	9.156208313	8.62991701	8.978723121	8.875206121	8.957944414
9.615324495	9.002272697	9.329922273	9.043522909	9.275532697	9.326238727
12.25844332	11.59021288	11.88649013	11.48655657	11.88909817	12.27421104
11.34381562	12.19398134	11.52645916	12.16036216	11.78265022	11.63629658
9.682548525	9.382205394	9.692043475	9.689783586	9.266794162	9.505859384
8.185177828	8.205892515	7.685352364	7.583321859	8.334981101	7.819085697
8.568242293	8.788663253	8.31318196	8.271795141	8.711304737	8.448929687
9.55462702	9.650789909	9.079484556	9.20200202	8.853585596	9.416442616
7.286722061	7.144299071	6.906817121	7.076028737	6.94614002	7.192117626
6.93243001	7.090758091	7.140009717	7.327495141	6.601717263	6.842776727
12.08680505	12.52461444	11.92937262	12.67700968	12.15752365	12.35798465
9.86223396	10.00094823	9.800083455	9.869466131	10.06633871	9.945028121
10.98186414	10.77252405	10.56802411	10.80389925	10.50677257	11.0859227
11.83330316	11.98196729	11.88375452	12.92937388	11.88649013	11.96362869
7.98506802	8.073652495	8.271795141	8.760291222	8.357077121	8.471953717
8.001277303	8.090886384	7.853698798	7.994228667	8.069753788	8.099112939
12.32364634	11.64637724	12.53911948	10.58469265	12.25251148	12.42616911
12.49965499	12.54906863	12.58287167	12.03602449	12.76628311	12.26162026
11.21958266	11.14707085	11.21338774	11.08756575	11.19817469	10.98350754
10.81664077	10.42736719	10.77890896	10.17702971	10.75178718	9.532335293
7.552094283	7.054536222	7.966402293	6.771393131	7.550195808	6.915128061
9.086722374	9.203760495	9.242526283	8.652374404	9.058895657	9.509226798
12.70002263	12.84627851	12.87819387	12.99088515	12.49170848	12.49965499
8.002717273	7.411868313	6.924186162	7.231823384	6.728691949	7.116617232
9.028300162	9.673359929	9.604846424	9.297353606	8.580231323	9.681893121
7.414724596	6.924186162	7.693516556	7.039421667	7.211808657	7.466006556
7.503972212	7.881102323	7.082435283	7.608647091	7.367866364	7.36510997
11.66441291	11.5571076	11.66441291	11.19447199	11.59427705	11.76804793
11.45002291	11.36065784	11.73271713	11.54053762	11.66708917	11.67371856
8.721523222	8.509065354	8.729485838	8.36556403	8.268052495	8.417604414
5.762443596	5.908375212	5.749045222	5.751241515	5.759639268	5.756015182
6.860943283	6.185014717	7.353560545	5.980622596	6.715418303	6.782883697

7.715785354	8.043387838	7.726017717	7.955993172	7.927799515	7.65784001
9.537497323	9.287819051	9.116279576	9.257979848	9.465071586	8.974101818
9.129114848	9.167114	9.625623061	8.949261586	9.245445293	9.119222505
8.426780919	7.41731598	7.549557061	7.609232899	7.397737788	7.407119727
8.177146606	8.786134101	8.261007747	9.071813909	8.576891556	8.286128505
8.948168879	8.964085616	9.180285111	9.254253192	8.930433505	9.136296333
12.17216641	12.12939166	12.87421343	11.3290504	12.19086998	11.13240418
10.99046505	10.80239406	10.8254891	10.24414187	10.90057618	11.06123261
9.166446616	8.772410586	9.243679384	8.789132293	9.293710889	8.551751717
9.792275424	9.981718727	9.733230051	10.37573078	9.973623707	9.933588354
10.63870636	10.13375911	9.997192202	10.03701736	10.09994749	10.32093584
8.795325869	8.424058939	8.946596606	8.532119949	8.995099212	7.964179182
10.36349344	10.13671639	10.38631174	10.24942024	10.05607537	10.53842125
10.59062542	10.28190294	10.45877561	10.31997281	10.26729954	10.79686873
7.591105343	7.787525596	6.691780525	7.381652111	7.076028737	7.328411747
6.547992737	6.661815061	6.27919301	6.907839071	6.550562869	6.455790798
6.601976232	5.99606497	7.039421667	6.197177	6.396609343	6.613208707
8.201303758	8.476485121	8.347717242	9.029402949	8.29291099	8.454954939
10.23452156	10.15186663	10.07764109	9.992988556	10.24139873	10.41731261
9.626304798	9.07526898	10.16073454	8.64224801	9.585510535	9.745446081
8.821885465	7.795429838	9.323731354	8.081502051	9.090721626	9.254253192
6.981618121	6.67332503	7.149903152	6.421175192	6.67332503	6.902232848
9.504584061	9.751885293	9.984347768	10.2600862	9.462343535	10.20634509
6.233249556	6.15200799	6.260841061	6.225179949	6.317094394	5.961588475
9.61181703	10.0728998	9.435122859	10.27236099	9.751080646	9.665316566
8.030614293	8.201745222	8.221010253	7.755503707	7.819838162	7.711891616
8.101068051	8.185563303	8.088956818	7.910975505	8.000883182	8.199315667
9.686378667	9.625623061	9.302735434	9.316888828	9.625041525	9.565032515
9.739033131	9.414554808	9.886762101	9.618256808	9.763952101	9.830354566
7.780448354	7.31937996	7.26025397	7.638981707	7.53302901	8.040196323
10.61371787	10.13193078	10.55842197	9.772980061	10.18666124	9.451851384
6.709075525	7.126018333	6.618269616	6.819670818	6.544818747	6.79225503
7.809644101	7.572483949	8.116928465	7.262118616	7.953486636	8.145962667
7.543124535	7.660150727	7.066488111	7.47214603	7.46337499	7.487351192
7.226432323	7.410002717	6.964624475	7.161889071	7.200097273	7.26819499
8.193820172	8.084674061	7.99823603	7.876807081	7.807594768	8.066048727
10.03123363	10.62647628	10.17904437	10.74819911	10.3986043	10.23066525
10.27236099	9.791460222	10.761049	8.832790172	10.38329808	10.26116661
9.879566364	10.06904603	9.684886576	9.909202788	10.08893073	10.03205964
9.009915859	9.101613848	8.646531515	8.73262002	8.875711838	8.893968333
9.489614919	9.302735434	9.657223606	9.215946081	9.30949301	9.694213162
8.144908747	8.31318196	7.884314808	8.090886384	8.406604404	8.412578899
8.959665061	9.026117657	8.644074768	8.52305899	8.68840998	8.559975162
11.38178745	11.42371373	11.15342364	11.40725584	11.47682743	11.34892675
8.261781515	8.149032212	7.926719768	8.069395848	8.23581501	8.243245818
7.639261768	7.162716495	7.803193929	7.712213273	7.341641707	8.183195545
8.418553323	7.74646602	8.891888576	7.592689374	8.267698071	8.679110091
6.427492121	6.293534101	6.553884717	6.284746758	6.450362182	7.679634566
8.679110091	8.023866626	9.008956374	7.486457414	8.411285283	8.127056495
15.32551587	15.22815255	15.36066179	14.57091557	15.59877501	15.59877501
7.380085202	7.34349297	8.47014499	7.546918172	6.947497737	7.219933525
6.533325687	6.557137687	7.087495071	6.485102909	6.290108071	6.459603455
6.918844495	7.304125475	8.396532182	7.144617717	7.132449434	6.826151202
9.373397364	9.504584061	10.78551178	10.0841781	9.883591758	9.303966081
7.819838162	8.147147636	7.592388485	8.346198293	8.163475424	8.055171202
10.80091358	10.70052069	11.43938222	12.05489479	11.35278885	11.19966733
8.647073404	9.201355202	8.726024485	9.484840202	9.17861001	9.106199707
6.612060818	6.424247404	7.13441502	6.380219	6.673658833	6.870614657

7.473129747	7.595301182	7.138031798	7.341038	7.445432899	7.247290424
10.82416773	10.82416773	10.51174846	10.8443678	10.95533553	10.67939021
11.1612024	11.20163629	10.55333109	10.86566339	11.0473733	11.42178201
12.05813886	11.69891546	12.48124823	11.37670858	12.4571909	11.44643795
8.142197384	7.51061902	8.217667525	7.353244222	8.19621002	8.289599333
6.437442354	6.165805071	6.903241343	6.349789677	6.607135434	6.046867384
7.323648515	7.191229121	7.786927414	7.334908263	7.268488485	7.288128061
16.02065153	15.78787594	16.04217659	15.63778652	15.92931651	15.32551587
10.22267877	10.83790729	9.976273051	10.96460614	10.3918566	10.46664928
9.355403384	9.394567081	8.944409727	9.362770111	9.369545939	9.608350808
6.642136364	6.686567667	7.495088838	5.800064576	6.389669667	6.899979889
8.945493505	8.330618758	9.098746404	8.289212586	8.841037687	8.403930677
7.558798354	8.429582192	8.643157737	8.541524636	8.30620801	7.733228788
12.52130815	12.28105924	12.76628311	11.77552743	12.94367069	12.71830686
6.295706747	6.337620818	6.377717758	6.510509061	6.464688212	6.287413717
7.168942192	6.77206196	7.424927172	6.792730596	7.273224596	7.484183869
9.361074667	8.740682	9.973623707	8.922632727	9.379720636	9.562994313
13.54221797	13.94443	14.52085241	13.64083669	14.25042984	13.16151113
9.230770667	8.648450303	9.279246253	8.766842081	9.601568657	9.488971182
7.393167162	6.969691626	7.579015667	6.885887889	7.501777556	7.794025323
6.765291919	6.232903485	7.214035101	6.444835424	6.529968303	7.149668323
12.68484185	12.41337695	13.01016522	11.52645916	12.65104366	12.63500423
8.226500707	8.028563859	8.915814212	7.796085586	8.715845505	8.251125323
7.583692414	7.037826263	8.069753788	7.219933525	7.38340502	7.724673141
6.884803323	6.556248091	7.315524667	6.259701051	6.42554104	7.685352364
7.32157099	6.526325283	8.125376242	6.756647869	6.73278298	7.628690051
8.792768747	8.532516465	8.962901121	8.257595869	8.816703535	8.334162556
8.366003535	7.847379061	8.76328402	7.997498828	9.104371838	8.26142704
6.737098263	6.320458162	7.113452899	6.245605929	6.638813101	6.868107182
8.777050384	8.357077121	9.121511687	8.157542717	8.82024202	9.160413333
6.415279879	6.35000001	6.428147687	6.141351889	6.452573444	6.458058747
6.173134414	6.004508838	6.418240687	6.17767296	6.125621596	6.265013455
6.702042636	6.580361485	6.725499141	6.606197394	6.957146182	6.814222283
7.735932323	7.493863788	7.632188616	7.031184939	7.74646602	7.461834424
6.632573485	5.904221111	6.957146182	6.062932939	6.497193293	7.341641707
7.147201253	6.828622869	7.507667889	6.801007778	7.56165098	7.419865051
6.892562737	6.852936051	7.380686859	6.82385998	7.057330242	7.287539616
7.636533838	7.485191121	8.104133364	7.311327071	7.799524616	8.195856758
6.938149939	6.563203242	7.388278253	5.991248111	6.557897323	7.539167263
8.672348475	7.963030121	9.235179424	7.591751808	8.553132192	8.908475283
6.471481465	6.203838343	6.525371737	6.099738869	6.256680889	6.474717424
8.364710758	7.876418838	8.76478299	7.801818071	8.177906263	8.379056919
6.717503818	6.183290606	6.989715687	6.269932061	6.521008828	7.083314707
8.805907646	9.387656788	9.620873889	9.921211657	9.126663535	9.450568111
7.820605525	9.11393401	9.477759242	9.147897788	9.537497323	7.435709141
6.939947626	8.329784131	8.359650919	8.039451586	8.420387707	6.734478838
11.6445291	11.34576518	11.79171062	10.60507136	11.41981305	12.08680505
9.288999586	9.221852535	9.617464455	9.733950273	9.161011869	9.376046232
8.273483707	7.491688212	8.491049758	7.631600293	8.093860848	7.991889949
8.741655828	8.892401576	8.543686687	9.317615606	8.834473929	8.950874253
10.72900488	10.573928	10.74045328	10.47584162	11.03296099	10.32309273
8.294640202	8.527198758	8.432612263	8.890373556	8.50618604	8.557697889
9.156208313	9.11154599	8.842109	9.203760495	9.153730283	9.195534384
7.777311636	8.40702104	7.784199556	8.645985424	8.276867707	7.633560879
9.93099102	9.568406747	10.13958591	9.35728196	10.04340808	10.07764109
8.240375404	7.835124576	8.57790302	7.410944101	8.368326869	8.109636222
7.673376626	6.531724505	7.976116929	6.770611535	7.389558374	7.961659859
7.083013909	6.796831899	7.644626202	6.661293222	7.039183869	7.117635758

8.390560192	8.380307172	8.593101434	8.705608919	8.743124222	7.954533778
6.69201996	6.709290939	6.449263919	6.654520283	6.969691626	6.838696091
6.913266485	6.604752283	6.662035182	6.067236707	6.073202434	6.405058101
7.41345197	7.230210646	6.859461465	7.120752303	7.311629869	7.198597879
10.16454544	9.238221677	10.86691694	9.356058495	9.935661677	10.83790729
8.399975414	8.119679141	7.997498828	8.149788848	7.945472162	8.166983455
9.958247111	8.868979273	10.1597097	8.435296202	9.715215101	8.996148737
8.058592364	7.697940283	8.827377505	7.328721848	7.719108384	7.638981707
8.067838768	7.466006556	7.613240606	7.268778051	7.499556727	7.358375414
10.62764398	10.17122401	10.85782556	10.1250404	11.14211434	10.93334198
9.248396293	8.451550535	9.66613299	8.013081051	9.234418071	9.176817596
7.164102909	6.857999616	7.221345788	6.906597848	6.963370131	7.151822525
8.457043091	8.773471869	8.464558212	9.003357768	8.494576061	9.06557301
8.292509485	9.865550636	8.843148071	9.323731354	8.69723302	9.204855576
8.012010707	8.939630657	8.744562374	8.484723788	8.025425616	8.650965909
8.909404788	8.611710212	8.427685838	8.77298297	8.518658667	8.631899566
9.738398616	9.520598525	9.257979848	9.196132455	9.289535939	9.836777707
11.09250749	11.04156144	10.8443678	10.87235819	10.71279176	10.85782556
5.766802545	6.590707929	5.781781232	5.988108535	6.640442667	5.990249465
13.60269863	13.45466248	13.52959744	13.56234785	13.27940818	13.89331098
11.26446827	11.01951417	10.65500785	11.01045685	10.57649171	11.22294817
8.424471616	8.085049192	8.401710747	7.341339747	8.029005444	8.08737399
7.962004212	7.843077242	7.59785203	7.864535646	7.521885657	8.334587636
7.607714535	7.817786	7.823737283	8.699656879	8.377090384	7.877844556
7.516462556	7.778011646	7.723009	8.233421293	7.105225354	7.279114576
7.314926455	7.464704768	7.297796364	6.917222737	7.034081253	7.295094879
8.267698071	8.216823323	8.158602879	8.259704283	7.924172566	8.245649515
9.564343333	9.106823152	10.03026493	8.853585596	10.21769501	9.20200202
7.840343939	7.460903263	8.005065495	7.152088687	8.233838889	7.568310444
9.619644949	9.193817232	9.920430606	8.663303283	10.30151978	9.251353071
12.26475658	12.50328361	11.94481318	12.62127559	12.3843369	12.43279956
8.539328929	8.657372182	8.491490828	8.796713535	8.417196576	8.598980253
8.944409727	9.061091758	8.740195303	9.21352099	8.830117929	9.89348604
7.868722081	7.587635919	8.307483899	7.528303323	8.22344601	7.667962596
7.404051424	7.283275424	6.866852303	6.987320737	6.544097556	6.543419131
7.475616394	7.167545535	8.101780545	7.437630343	7.775998535	7.612584303
12.27101	12.59323492	12.57877126	12.59323492	13.17281418	12.50328361
7.164949697	7.907575465	7.748187364	7.940978939	7.180291929	7.422534091
9.223885747	9.445852485	9.039454747	9.70574997	9.482964828	9.487680697
6.90482902	6.682819434	7.453291162	6.687236677	7.134964384	7.101049919
8.083135202	8.510413414	7.95873603	9.149684939	9.094076404	8.445297313
8.830117929	8.035675313	8.683580283	9.814415596	9.35796903	8.992957212
6.28178403	6.422838717	6.611837919	6.088380414	6.706660646	5.992264404
11.10035391	11.15808934	11.06877356	10.90324033	11.13551505	10.85923156
10.18666124	10.5029511	9.865550636	10.1838089	10.61133106	10.35178281
12.13244857	12.02811467	12.18792926	12.0724233	12.09804166	11.86922315
8.110881556	7.985767212	8.808928141	8.29927897	8.071334404	8.013487535
7.81270001	7.785596848	7.424653535	7.640998838	7.629344343	7.69973097
8.663769939	8.351752182	8.425403333	8.257984293	8.215875455	8.31318196
9.11510898	9.844817354	9.631182697	9.693515768	9.393934131	8.998971485
6.992927808	7.016958768	7.541384646	6.524953909	7.233950838	6.609208404
9.142159707	8.865407737	9.286110869	8.954176293	8.669992818	8.77608297
8.722452253	7.807154444	7.42989803	7.400864778	8.048636061	8.261781515
6.730429919	6.96434804	6.891529131	7.032021525	7.536179778	6.821773354
9.565032515	9.776839404	9.293710889	9.606954152	10.14044783	10.03983393
8.372772768	8.421742051	8.63568099	9.443424081	8.888204949	9.003925646
9.25368898	9.509226798	8.798818646	9.135096212	9.456392222	9.432003333
7.465036929	6.747331182	7.718373848	7.012040828	7.243557061	7.325469747

5.91279402	6.005914232	6.182077616	6.252266879	6.468467859	5.815256505
7.122138172	6.770374545	7.877844556	6.949065	7.395300939	7.476255091
6.587993828	6.41992303	7.152088687	6.53573598	6.923167202	6.222974525
7.869709071	7.429567525	8.086153313	7.282365313	7.963030121	8.068564879
6.419103354	6.396609343	7.011288768	6.237576566	7.009988212	6.63116302
7.886480404	7.261824374	8.533908051	6.556473354	6.917222737	7.274379929
7.359577545	7.012571859	7.287539616	6.583924121	7.533663232	7.091067131
9.119831202	8.719245818	9.038849273	8.421742051	9.018802576	9.105035414
6.498363273	6.383422788	6.615630737	5.979783929	6.183978364	6.598445515
7.620272192	7.833986465	7.913871889	7.74646602	8.928828384	7.665645141
8.303356141	8.504327141	8.868979273	9.597513697	8.945002717	8.576891556
11.04886125	11.1031408	10.89094316	10.75453103	11.09885051	10.78408516
8.913588253	9.170513566	8.748883111	8.783158111	8.919862162	8.859179455
9.06557301	9.657223606	9.465659495	9.188424626	9.07419996	8.897177899
12.15752365	11.82852227	12.32024961	11.76588718	12.28985985	11.24993851
8.664725051	8.474228909	8.353342091	8.301264212	8.087781242	8.568768141
7.124332475	7.163863636	7.145493505	7.117115202	7.129030091	7.198896253
11.39772243	11.51260306	11.78725933	11.08264222	11.10761793	10.72516317
8.907915899	9.321890899	9.756054152	8.545091384	9.630502939	9.70034896
8.537956091	8.175629323	8.215875455	8.155564404	8.303793737	8.643157737
10.95093974	10.88945836	11.34576518	11.86652814	11.42178201	10.9889639
6.757884566	7.017519192	6.685804808	6.92802298	6.736375424	6.986766929
7.51247098	7.473428394	7.854005263	7.214315212	7.901352025	7.386763141
6.47341896	6.582190566	5.924891646	6.538200697	6.139284859	5.781922
10.2752886	10.29542336	9.888393747	10.09426568	10.15585749	10.24942024
8.022720303	8.271405859	7.754082929	8.419470808	8.412956141	8.307483899
8.566415323	8.693780273	8.654464586	8.917682242	8.896578162	8.422204465
7.124596343	7.718373848	7.437630343	6.948610667	7.492628778	6.75400198
9.971733293	8.823889374	9.70034896	7.957660747	9.153232364	8.026271202
9.581418808	8.736221495	9.808672222	8.523456192	9.487680697	9.610538374
12.69676846	12.42943771	12.41337695	11.35657003	12.85048632	12.91196246
11.66708917	11.60680648	11.19638858	11.47682743	11.52645916	11.63837311
11.22119175	10.50166798	11.64044206	10.47702836	11.33477589	11.51867768
9.681142253	9.849671929	9.835894606	10.11452638	9.669847545	9.515069697
9.488971182	9.89348604	9.39974104	10.02269473	9.598893818	9.846508495
8.799348343	8.888766747	8.817758687	8.81966502	9.078885242	8.76386996
9.646274414	9.565032515	9.511857455	9.433849505	9.428190232	9.564343333
6.849574919	7.119314485	6.665174586	7.013137848	7.591105343	7.064817384
8.940148162	8.950874253	9.473222667	8.985821889	9.373397364	9.286739242
8.410908071	7.869396606	7.335878859	7.347141657	8.562664263	9.167114
13.66784462	13.64789782	13.66158872	12.88708268	13.89331098	13.39037726
9.08614898	9.158666485	8.12949599	8.458697343	9.580097263	8.320817869
7.011036525	6.997628374	8.873520545	8.978204141	9.009407333	7.021482505
6.424050455	6.463301323	6.753469455	6.452749343	6.483554929	6.786546495
6.64187404	7.047573414	7.01049403	7.33182303	7.854685616	6.736375424
10.00002479	9.917863333	10.11452638	10.20060626	9.663106646	9.505258828
9.555296242	9.35128199	10.15283227	9.519820636	9.793942798	8.63611798
7.574795778	7.51810503	7.226157253	7.282945384	7.050623333	7.558170465
9.629101929	9.679062616	9.735412273	9.876880455	10.26832521	9.761765848
11.08756575	11.04886125	11.0387546	10.90057618	11.15503783	11.29947244
7.611330343	7.141717727	7.85268101	7.184502222	7.135222657	6.593634414
9.207219232	9.164000111	9.078885242	9.454390232	9.105035414	8.989142232
10.26729954	10.51174846	10.37686258	10.49713487	9.870221061	10.12610261
12.16942168	11.82616001	12.32964397	11.49029795	12.53563192	12.52130815
8.940763364	8.874191697	9.667708556	9.382882596	8.956909848	9.103235919
8.019434586	8.764299566	8.189547909	8.68840998	8.973478152	8.264694495
8.854603364	9.386484303	9.013314737	9.193817232	9.339764051	9.296718121
7.727729838	7.270153101	8.000144121	7.379145515	7.87472	7.313208869

7.056486293	7.235668455	7.233039222	7.016197848	7.331138333	6.862375091
9.560346242	9.687758929	9.414554808	9.569854202	9.717365758	9.703591263
15.31193978	14.78379092	14.85646221	13.71423947	15.22815255	15.42966281
8.781680606	9.145664475	9.212980727	9.696433626	9.186711646	8.854095293
8.981975535	9.205404404	8.804477505	8.851018515	9.009915859	9.032817303
10.56421743	10.70534521	9.894213939	10.5029511	10.62895885	10.82804231
9.319510202	9.397189192	9.393335687	9.619644949	9.394567081	9.276789111
7.233650202	7.418256545	7.199149121	7.356848556	7.507667889	7.518430525
8.383846919	8.921533879	8.770297859	8.907429495	8.105629576	8.917682242
8.341518747	8.440058758	9.702015293	8.696280707	9.237664879	8.975838535
16.33688632	16.52993947	16.42659037	16.49551516	16.64557528	15.95256028
8.403471909	8.561321323	9.441908576	9.481668929	8.847386828	8.342773172
9.610538374	9.502644929	9.472030465	9.311440606	9.540446515	9.705000525
8.063008152	8.102862354	8.264240727	8.08112003	7.856391758	8.137069859
7.814474586	8.154705535	7.704350384	8.557697889	7.962004212	7.935081758
7.942071303	7.928481586	8.469779808	8.325717687	7.390752071	8.577417788
7.966402293	7.95565902	7.788172758	8.78563097	8.340681505	8.020887778
7.017803152	8.264240727	9.463745455	7.122942091	9.302103889	8.990775909
8.499404646	8.191573747	8.957404525	8.209418434	8.614861051	8.663303283
7.644277273	7.757234778	7.400015081	8.498949848	7.434501333	7.875433545
9.876096838	9.916104475	9.541747455	9.582761798	9.854472677	9.905108616
6.465379212	6.761077899	6.422232	6.675711333	6.330710616	6.557678737
9.018299374	9.293068828	8.663303283	9.243679384	9.731150899	9.183367525
8.69919198	9.035586242	8.40783399	8.846355515	8.352537101	8.898709273
7.601304687	7.779041818	7.661901192	7.923048354	7.539487212	7.499261545
10.23850383	10.27905981	10.4302696	10.48539779	10.03299879	10.21583282
11.25898056	11.21338774	10.82804231	10.99469779	11.17041501	11.38775416
14.01682491	13.47327752	14.05136004	13.52334642	13.71423947	13.55640443
9.930141081	10.14534091	9.514496606	9.770750222	9.698896848	9.779738
11.86922315	12.37087917	11.96947069	12.82558839	12.41337695	12.48458958
8.486968232	8.305787747	8.735287485	8.426780919	8.347717242	7.587331949
11.36245739	10.98492733	11.57567047	10.75062885	10.85654613	11.15966862
11.70348036	11.09089144	12.14789109	11.15503783	11.84027237	11.48655657
6.864750879	6.635819404	6.962822313	6.656891394	6.612755929	6.636058242
10.49348589	10.34717129	10.46342714	10.13862753	10.11760527	9.713188212
7.710917071	7.311037152	8.116577384	7.152958919	7.785596848	7.756175162
8.189194404	8.870056939	8.148682091	8.911610283	8.114219303	8.119679141
7.246435697	7.344882899	8.068931465	8.147481596	8.088956818	8.15355797
6.23939997	6.158989596	6.575452697	6.031725677	6.289157818	6.439991313
8.862785404	8.867411152	8.064260859	8.331046747	8.663769939	8.527198758
9.014335657	9.224923687	8.862785404	9.403514808	9.043522909	9.031155364
8.229734556	8.266388465	7.579350515	7.896600606	8.22437102	8.288355808
8.734416606	8.678158051	9.036569232	8.664254273	8.815152444	8.47014499
7.875433545	7.606370131	8.134790222	7.469040515	8.072532475	7.911309091
8.072532475	8.011643808	8.10683704	8.083806798	7.805068949	7.853698798
6.344188131	6.599616071	6.569841374	6.420140667	6.900957616	6.472300566
6.873794323	6.752731616	6.839464636	6.733232384	6.574738525	6.480794707
6.198948818	6.060926152	6.315805677	6.215353354	6.022565525	6.307933879
9.385290182	8.922082808	9.455711606	9.047491869	9.420326576	9.179819687
10.4994288	10.05426836	10.52585606	10.46442497	10.82416773	10.21769501
6.510975394	6.38666303	6.920918808	6.156331323	6.833605444	6.435479606
8.833337414	8.273878929	9.333361616	8.244805758	8.627322343	8.399075535
7.221626808	7.039966242	7.609232899	6.995550687	7.639990515	7.196682227
8.485174677	8.36093298	8.759332636	8.159907626	8.824381182	8.756396081
7.972012263	8.695335444	8.373919657	8.292509485	7.879985051	7.752031929
9.025691242	8.818695101	9.084401768	8.436545051	9.018299374	8.922082808
10.65014074	10.50543333	10.6426412	10.34418014	10.83790729	10.54692936
8.745474636	8.71485401	8.746767313	9.136296333	8.682101313	8.48093102

9.051375475	8.730448515	9.276189121	8.612168455	8.691458141	8.675348646
12.01673563	11.565007	12.41634374	10.55842197	11.8164662	12.19727311
6.933815889	7.239990273	6.606886212	6.858444758	6.842776727	7.394027687
7.373116051	7.053922394	7.402347162	7.160791343	7.269064929	7.432070859
10.02362526	9.776037091	10.98037262	9.836777707	10.42622906	10.31693391
9.271759586	9.093001495	8.656835556	8.721523222	9.049629657	9.441908576
6.353983172	6.449076475	6.865560778	7.208321485	7.073657889	6.654064545
8.519178475	8.161033919	8.596127869	8.213427111	8.300888657	8.53537201
10.28899471	10.74819911	9.961780586	10.40486524	10.55474917	10.16262583
7.407119727	7.122138172	6.916181	6.597017333	6.982384606	7.393766606
7.953126222	7.991889949	8.207926798	8.008400859	7.989587677	7.872350636
10.96460614	11.109559	11.08107282	11.55322016	10.61261659	10.82290783
11.55106122	11.5386618	11.60680648	11.72592113	11.60888892	11.45176908
8.745901071	8.817191758	8.796713535	8.733877859	8.628622212	8.383846919
8.712341879	8.89604703	8.544633697	8.648450303	8.412145273	8.835493283
9.23383296	10.42409385	9.822898081	10.39968902	9.680349586	9.184527788
14.80749806	14.61202705	14.74028573	14.09521817	14.89443037	15.05177986
6.446595263	6.214815889	6.882010616	6.121295172	6.38666303	6.550797374
6.207516667	6.073202434	6.477093667	6.140862859	6.310299848	6.100273545
7.592047838	6.739283293	6.794126838	7.046194354	7.495424616	6.883278424
7.187741232	6.807473404	7.569572879	6.83774396	7.281724869	6.953109798
9.803356646	9.162016475	9.988725727	9.430738808	9.145664475	9.596088263
7.821271606	8.183602061	7.702311758	8.539328929	7.830100949	7.717737636
6.862647808	7.494798263	7.041369354	7.363617495	6.827661505	7.294583061
7.624735818	7.737649848	8.010523545	7.769805091	8.121954505	7.448310273
8.079179394	7.358375414	7.729067364	7.347734202	7.159453505	7.313451111
6.692266071	7.000309313	6.608273162	6.555521899	5.932483798	6.818961091
10.2026692	10.49713487	9.967178364	9.915234909	10.13276962	9.917863333
8.235056909	7.870669919	7.605411525	8.063898495	8.174069929	7.97284099
8.812517081	8.158321101	9.09013302	8.228066545	8.585836293	9.215946081
8.147857556	7.490707535	8.329784131	7.660795616	8.102862354	8.69723302
7.869396606	8.280707687	7.792995667	7.851994586	8.476994374	8.118136869
8.522641273	8.14677703	8.885078273	8.199315667	8.432150758	8.273878929
6.58891503	6.463778677	6.783136778	6.445904515	6.386030697	6.354946212
7.264201869	7.396513838	7.472500828	7.461509737	7.388945192	7.392836818
7.275508121	7.004269242	7.794025323	6.959689121	7.279952616	7.347141657
9.050265121	8.803935202	8.464081152	8.804477505	8.899757485	9.076382242
7.635267465	8.242431778	7.096340343	7.416641556	7.132751192	7.850535525
8.481411768	8.301264212	8.960218374	7.999737535	8.564847253	8.571194222
10.86820493	10.50794857	11.07647857	10.21279484	10.69470446	10.64738653
6.522760475	6.356712303	6.66276004	6.348225889	6.364101768	6.410700434
7.751275798	7.858612495	7.452458778	7.420458273	7.733228788	7.814093232
7.730873535	7.321884212	7.689572788	7.115757364	7.625433667	7.115448364
7.039183869	7.326634626	7.442908828	7.59498599	7.499887141	6.86986903
9.818059192	9.878686616	9.444057586	9.703591263	9.93663897	9.925226333
8.583996586	8.837022465	8.890936242	8.493155899	9.325601172	8.683087172
7.07011003	7.757833798	7.493254434	7.623059667	7.682588111	7.090758091
6.53573598	7.366646525	6.79323798	7.310724131	7.21064097	6.609763515
9.322592778	9.518503263	9.387656788	8.91530399	9.873591818	7.938531869
8.908935182	8.543234566	8.971727333	8.543234566	8.838495424	9.099925818
7.256596263	7.022261949	6.900957616	6.964624475	6.951961606	7.174439091
8.466026899	8.983202444	8.571676263	9.221294354	8.39402401	8.664725051
12.3774088	11.77305263	12.75396286	10.81142627	12.50706636	12.72219022
9.813557354	9.959124273	9.857104919	9.925979414	9.894213939	9.790672212
9.694213162	10.88302044	9.917863333	10.21484784	11.42787377	10.91356479
12.95787308	12.76628311	12.94367069	12.57486869	12.89113428	11.69188536
7.023936596	7.291703707	7.663618646	6.754244737	6.922922101	6.949335212
9.70798304	9.190205293	9.885156737	8.835493283	9.859664869	9.514496606

9.356741384	9.552821717	8.858269384	9.196689051	9.560980909	9.417594202
8.715328101	8.125818909	9.003357768	8.179581869	8.594016788	9.176146596
7.096340343	6.949335212	7.389870293	7.058479485	6.997357515	7.286722061
8.002342131	7.889031475	7.358075697	7.870669919	7.67303801	7.790242404
6.825565162	6.613208707	7.215185141	6.478358667	6.474296596	6.358502424
12.67378338	12.80166365	12.62127559	11.82346178	13.15082863	11.67593314
8.289599333	9.140978061	8.587234515	8.914720172	9.844817354	8.220225172
8.864955909	8.008400859	9.505859384	8.045324071	8.210994667	8.457489293
10.156858	9.933588354	9.789131121	10.14999886	10.1300726	10.56421743
7.670568384	7.770819434	7.460591212	7.730873535	7.966742939	7.842052606
6.762314343	7.012842909	6.943066869	7.490096869	6.634356909	7.024510747
8.749346626	8.353342091	8.512201869	8.501579646	8.372086737	8.332313525
8.401317182	8.363468485	8.336257222	8.514909313	8.139691131	8.363019899
6.930743303	6.766293242	6.626438091	6.47944304	6.414650828	7.380377788
10.36687712	10.48310631	10.36239309	10.40925212	10.2572153	10.51771241
8.603276828	8.979921919	8.635209293	8.580722586	8.678158051	8.492322828
9.386484303	9.269847242	9.730279475	9.264989899	9.424985495	8.336651323
7.944058707	7.963792818	7.779041818	8.629055081	8.092691677	8.294640202
10.687913	10.45375986	10.70775781	10.23850383	10.40178901	10.80091358
6.983407384	7.517436172	7.340746384	6.664432283	7.487940131	6.747074889
8.250622253	8.488340929	8.591651626	9.009407333	8.528915121	8.515350859
8.979298788	9.40167604	8.830660505	9.585510535	9.196132455	8.930433505
8.647922808	9.093526566	9.157435455	9.177504333	8.689808788	10.15875628
8.556855828	8.744081071	8.412145273	8.785177889	8.50473503	8.714332636
9.206631485	9.447242061	8.953164051	9.263233333	9.302735434	9.318228697
8.800397414	8.171788646	9.053043889	8.085049192	8.430929525	9.07526898
8.458294828	8.549141545	8.012010707	8.12949599	8.997807768	7.988882495
7.983913121	8.355847778	7.60282502	8.490053091	7.705120374	8.239069586
11.62739451	11.92396137	11.50051559	11.81439214	11.45932673	11.6445291
9.108564253	9.505258828	9.048620525	9.752732475	8.992473788	9.269847242
10.2187154	9.643287657	10.59685822	9.441908576	9.994772242	9.945994374
10.38631174	10.66587479	9.93099102	10.07580535	10.65871503	10.38001468
8.357077121	8.587623212	8.362537455	8.985325394	8.390560192	8.483763566
7.517077263	7.576996152	7.058764576	8.324453253	7.225001677	7.332669495
6.895709172	6.909921869	6.759694808	7.169190859	6.917518081	7.095275111
9.986114121	10.06173934	10.21157382	10.71503879	9.876880455	10.14631732
9.973623707	9.881912677	9.202552434	9.923012889	9.468134182	10.15483313
6.557137687	6.33568397	6.760600303	6.416313212	6.429246364	6.701799071
9.910074283	9.627745303	10.34717129	9.258612929	9.707282667	9.576740677
8.829061111	8.882379495	9.015412101	8.63794701	8.950874253	8.715845505
10.42062148	10.50057506	10.0406788	10.42736719	10.10542249	10.73661507
12.04990566	11.83813795	12.08128174	11.80791686	11.58195332	12.06964699
6.862895808	6.720840657	7.262957343	6.813695162	7.040256253	6.549596111
6.62508102	6.323128667	6.94407103	6.267412545	6.746600626	6.570961455
8.813595202	8.25570701	10.25906505	8.448456354	9.095762	8.975268424
7.850890354	7.684267263	8.157542717	7.600615697	8.156772778	8.000144121
6.998508111	7.174161374	6.751979343	7.271701444	6.770888697	7.264510162
5.829781091	6.056434111	5.759716798	6.02134404	6.277726788	5.767296556
10.88809182	10.73264046	10.73014715	10.77387872	11.02551486	10.97476091
8.594861495	8.143419303	9.253086273	7.952386505	9.203189636	8.922632727
12.47130519	11.43208235	11.85617239	11.34381562	11.43005301	12.27101
14.26676228	14.72870348	14.80749806	13.54221797	15.78787594	14.76275492
9.003357768	8.771856424	8.948168879	8.780633818	8.916479111	8.885078273
11.01376888	10.99046505	11.18941159	10.92337564	10.71145754	11.09721747
12.63500423	13.62106704	13.02006671	13.92172837	13.37822002	12.9851923
8.887109626	8.888204949	8.396125929	8.068564879	8.767872848	7.968277051
9.191263899	8.518658667	9.579413505	8.483763566	9.260036485	9.219562515
8.532119949	7.927799515	8.671783687	7.758961677	8.367840636	8.582586091

9.07581704	9.287277576	8.822853162	9.451851384	9.16271003	9.094076404
9.385837242	9.638947465	9.516398909	8.874191697	9.794631646	8.298004222
10.29324401	10.44195016	10.68338253	10.60277882	10.27714677	10.20859114
7.593349828	6.82197003	7.870669919	6.962306323	7.283275424	7.455517596
10.39747702	10.45754412	10.71613341	10.48310631	10.17613521	10.3494333
8.680072242	8.668536687	8.294640202	8.53715797	8.560348606	8.542859455
8.140538697	7.990313949	7.794719919	7.949071818	8.285708747	7.879985051
7.960192616	7.954205616	7.694540808	7.964914919	8.034450929	8.119288848
6.114553465	6.164313111	6.443888202	6.211831051	6.452349424	6.437269283
8.334981101	8.408718253	8.170954818	8.309247939	8.777050384	8.13932801
9.165786899	9.133284677	9.544495798	9.669847545	9.419654929	9.16271003
7.485478828	7.509397909	7.13105099	7.706911222	7.599870798	7.534556657
12.4571909	13.56234785	11.96362869	14.24162707	13.06967433	13.00581854
10.61001127	9.937540263	11.25700815	9.767642525	10.57996368	10.49466708
12.29886409	10.76737368	13.15645152	11.5386618	12.76207554	12.53563192
8.516793455	8.523456192	7.968277051	7.966054535	8.283330131	8.352537101
10.64379219	10.55966675	10.57159251	10.57282784	10.20964764	10.6488688
10.26632574	10.0143126	10.32418729	10.00985275	9.961780586	10.14809211
8.331046747	7.842359758	8.961854929	8.457489293	8.631899566	8.918756414
10.55602586	9.874496818	11.27153548	9.993897848	10.73517618	9.311440606
14.9727616	14.93426708	14.46162431	14.58095582	15.02501841	14.90726461
10.84974643	10.65987528	11.09565519	10.67939021	10.99046505	10.60277882
6.661815061	6.329319202	6.892302889	6.383834626	6.612316919	6.880472354
6.908859909	7.098240939	7.732919727	8.471953717	7.590787525	7.218710525
8.27018399	7.44801498	8.600831152	7.315524667	8.382609505	8.298396535
7.518993111	7.159453505	7.957321232	7.196550485	7.70790696	7.585232141
6.587577626	6.652192667	6.286506818	6.325342263	6.327726343	6.16126504
6.971045939	6.611837919	7.207252646	6.390148364	6.994769535	7.186028475
7.424024737	7.30728296	8.279889293	7.453291162	7.133298475	8.121954505
12.18492446	12.03912361	9.214694788	11.18636156	12.23769992	11.41238356
6.632345869	6.489773778	6.998209475	6.594580697	6.983407384	6.633233677
6.928862212	6.618022394	7.08018898	6.731175838	6.745111616	6.47989397
6.594376838	6.27402404	6.997628374	6.248708515	6.741002212	6.748324394
7.144887111	7.170007293	7.194186434	7.586712596	7.432070859	6.861163889
10.29208493	10.26729954	10.74690378	10.45041513	10.12041459	10.45996909
9.600888859	9.762542495	9.70574997	9.980879424	9.584803364	9.709426838
8.048636061	8.343220798	7.992269263	8.428657566	7.898974697	7.930615646
8.022369434	7.983913121	7.997875909	8.406205495	8.346585616	8.114611232
8.014589606	7.568310444	8.658802051	7.372532788	8.007640657	8.276014121
6.267871525	6.017778747	6.459423596	6.039785808	6.210980111	6.759203
9.806332162	9.90675497	9.908305848	10.12126634	9.951175646	10.02741959
9.731806091	10.46664928	9.746868596	10.20358283	9.759931222	9.768390455
9.07526898	9.07581704	9.136296333	9.330464051	9.413321525	9.348136535
10.89094316	10.97476091	10.76737368	10.88430477	10.94071069	10.71503879
11.53677901	12.06964699	11.75215821	11.97944932	11.542568	12.04432
8.951989212	8.805907646	8.85049999	8.482406475	8.633351606	8.668062838
7.779041818	7.680845586	7.542431869	7.700334879	7.572483949	8.067838768
8.405727848	8.228066545	8.693780273	8.047553323	7.869992939	8.148308414
9.533033172	9.609756293	9.232683343	9.524156586	9.262561	9.419020677
13.12562627	13.40302459	13.58996905	13.28559105	13.36582475	13.34204398
9.939327313	9.151976485	10.45150222	9.488971182	10.03492192	9.963504081
9.305189859	9.474605101	8.941359636	9.335272182	9.344897333	9.049171303
7.652952818	7.64232702	7.498343283	7.70790696	7.644959949	7.895961929
9.289535939	9.09178804	9.382882596	8.726978374	9.260607	8.940763364
7.592689374	9.768390455	7.564741838	9.273640758	8.350619919	9.287819051
7.257525758	7.059956313	7.423140192	6.889250929	7.342268626	7.028313949
11.80342824	11.96947069	11.54470354	12.36759935	11.94481318	12.19086998
10.96748398	11.02243393	11.30301738	11.74205097	10.76857843	10.94353341

7.728361283	8.08075902	7.767042424	8.230181798	8.174468182	8.037664566
8.856895172	8.380828141	8.845785121	8.297224525	8.499836475	8.81572204
6.54019204	6.440833869	6.728207192	6.425312475	6.735607747	6.379570131
11.83072395	11.70138789	12.37087917	11.37847359	12.20960187	12.03310273
8.759332636	8.978204141	8.876209859	9.296718121	9.631972414	9.689783586
10.20634509	10.74690378	10.30058205	10.98186414	10.49348589	10.15086094
8.038729465	8.03179299	7.819085697	7.643944697	8.372433475	7.67197899
11.30506756	9.787586646	10.36903954	9.872596081	11.02243393	10.34193651
7.932453717	8.086953081	7.827906879	7.936644384	7.97284099	7.782796596
8.650384111	8.621272737	9.070117818	8.373514202	8.671294253	8.436995596
8.93587504	8.904793061	9.027655758	8.507703202	9.002847848	7.969046636
7.754082929	8.10140802	7.579628838	8.424471616	7.545042545	7.715785354
9.155642525	8.864430475	9.122783717	8.468064667	9.244399414	8.502509434
8.189547909	8.18280501	8.40702104	8.411285283	8.281588828	7.702676091
12.27773509	12.23191382	11.90152869	11.95261936	11.78725933	12.42233701
10.15483313	9.699589758	9.831087364	9.713188212	9.390110384	10.90754603
14.61202705	14.01682491	14.63205333	13.32021166	14.29173122	14.33555643
8.07651197	7.919201576	8.245222778	8.400903717	8.000144121	7.932792414
8.717867636	9.267923343	8.757405333	9.072998758	8.814624313	9.10215096
10.04682541	10.75453103	10.2692332	10.55966675	10.63391466	10.18666124
10.35809493	10.36465285	10.47702836	10.51400776	9.986114121	10.2166926
11.81221198	12.17216641	11.76804793	12.56726296	11.9577766	11.9875501
9.867106131	10.00450786	9.990505182	9.935661677	10.51174846	10.10278369
10.09079019	10.06071544	9.574750414	9.992117576	9.159196141	10.42512206
7.136586475	7.693213707	7.07857001	6.918279333	7.760648222	7.020179424
9.238865747	9.187244566	9.211184727	8.778117444	8.553595747	10.04160563
8.671294253	8.657806242	8.418118394	8.477856384	8.652374404	8.717364515
9.389466253	9.033394545	9.434425707	9.050265121	9.569063343	9.789131121
7.741148566	7.307022919	8.281167657	7.719108384	8.345353354	8.173308798
10.02161965	10.56306707	10.20358283	9.855298394	10.77252405	10.40278823
6.100592202	6.845568374	6.229843364	6.225711566	6.061217212	6.036610232
10.7814083	10.20634509	11.21958266	9.756054152	10.08325108	8.365106485
9.069043081	8.597538556	9.119831202	9.095162899	8.502509434	8.707515232
6.339262212	6.199670273	6.474717424	5.843776152	5.871703929	6.291675828
8.769824364	9.254890202	8.668536687	9.436898869	9.017695354	9.055478465
8.446641939	8.597027768	8.575566677	8.748883111	8.289996192	8.62412801
11.18800276	11.77552743	11.33292491	12.3843369	11.58005536	11.52052132
9.687758929	9.048620525	10.02644328	8.712341879	10.10365156	8.753420071
7.055635202	6.922672051	7.083013909	6.809240364	6.481897646	6.929106091
7.073400869	6.966976535	6.967457677	7.229324455	7.348914263	7.344882899
10.40278823	10.93935201	10.01974654	11.13551505	10.83363242	10.4524716
9.471411141	9.554043101	9.023071899	9.374690616	9.320738646	9.537497323
8.93638097	9.019287687	8.582147707	8.807959646	8.862785404	9.025193869
8.274698687	8.214296636	8.121954505	8.052417212	7.979863071	8.263041909
8.702574091	9.156840283	8.749910828	8.746767313	9.35188598	9.000672535
7.488608758	7.145208253	7.569899606	7.02612702	7.243824414	7.508747081
6.295918879	6.303681202	6.332263313	6.518759273	6.282592313	6.262348667
8.480158333	8.59655801	7.975414556	8.116928465	8.582586091	8.179581869
8.369612525	8.68840998	8.242002535	8.64752602	8.507703202	8.528512253
9.314493212	9.110297333	9.390834152	8.886620687	9.148511889	8.691458141
11.29744903	11.45002291	11.42787377	11.66708917	11.3290504	11.54871614
6.508927202	6.506975192	6.913051818	6.928307859	6.737098263	6.561106071
8.559456798	8.898231131	8.449337667	9.176146596	8.72650097	8.708467242
7.390207394	7.056715131	8.163475424	7.415309101	8.352944343	7.114036293
11.10761793	10.8947391	11.16579624	11.53473316	10.76737368	11.0387546
8.788135667	9.210065	8.391020323	8.827377505	8.762202232	8.921011566
9.727935707	9.93663897	10.29660096	10.70181173	10.42297323	10.09339803
7.063754707	7.612584303	7.299074768	8.205457293	7.699362081	7.410944101

8.897177899	9.303966081	8.800397414	9.477759242	8.821885465	9.062220818
8.212243444	8.444212369	8.335469222	8.692870869	8.474658303	8.31214998
7.891912283	8.072532475	7.819838162	8.302498081	7.824443343	8.152087687
7.129030091	7.56165098	7.235098212	7.299714313	7.197446212	7.400864778
8.528512253	8.459227657	8.516793455	8.429582192	8.679578434	8.372772768
8.781156657	8.091725323	9.36570098	7.962380919	8.700606657	9.017695354
6.777231859	6.191123778	7.158304313	6.290882545	6.563407434	6.980492495
7.159147152	7.665645141	7.221024394	7.885378	7.285287818	7.42989803
8.411696677	8.70424704	8.913024061	8.877161475	8.468418253	8.762202232
8.38301802	8.024633293	7.814874545	7.107957081	7.495088838	7.565422434
9.296718121	9.588958727	9.291911616	10.0188097	9.586238192	9.430143091
10.23550694	9.230770667	10.59809845	9.333361616	9.885156737	9.581418808
10.66465811	11.13400706	10.60161194	11.16734497	10.8935809	11.38391359
7.210358909	7.21466303	7.164949697	7.41345197	7.450888848	7.28582396
7.834378071	7.838558162	7.679285535	7.741791646	7.653990838	7.587635919
7.538797101	7.562285172	7.330192545	8.099112939	7.552833101	7.750957677
7.172411071	6.893369586	7.705120374	6.870354606	7.421037465	7.55178802
11.54053762	11.65052704	11.28370047	10.94071069	11.9875501	11.89628941
8.519692939	7.946141838	9.112697707	7.761767192	8.438325768	8.854603364
11.65261158	11.57765358	11.42562271	10.8405808	11.24147284	11.31053596
8.813032152	8.660166475	8.458697343	8.71185199	8.135177152	8.512201869
7.803193929	6.751729354	8.294235768	6.848551889	7.608347495	7.902229798
12.54268587	12.8293171	13.62784329	14.29173122	13.96779242	13.98831971
10.23948175	10.10542249	10.30151978	10.15086094	10.28506122	9.939327313
13.07947925	13.5160991	12.89513437	13.40990013	13.07947925	13.52959744
8.826846768	8.301657889	8.884521697	8.448929687	7.987912222	8.992473788
10.94353341	10.82034332	10.84705809	10.3548828	10.95093974	11.03296099
9.403514808	9.59460096	8.991970091	9.788274313	9.486281202	9.436898869
10.67939021	10.6240143	10.33596201	10.47820756	10.74045328	10.47702836
9.332175414	9.564343333	9.602112616	9.534194535	9.324974768	9.346872071
7.582654121	7.790604293	7.091592101	7.459115707	7.353560545	7.775630727
7.613240606	7.799524616	7.333224889	7.681488071	7.728361283	7.757833798
10.69574272	10.17904437	10.17030341	10.27338352	10.35069541	10.22557955
8.184399818	8.432150758	7.927460424	8.17219503	8.277405556	8.39227803
7.957321232	7.644277273	8.299695162	7.501777556	7.741457929	8.057063626
11.17378617	10.3025572	11.69891546	10.34193651	11.11388317	10.71613341
9.520598525	9.458377051	9.493894545	9.293389859	9.11393401	9.538964939
9.35067002	9.800904051	9.293068828	9.46096901	9.711723394	10.00361533
8.570262	8.284505182	9.081927515	8.127495596	8.945493505	8.970512081
8.569735879	8.946596606	8.307093606	8.720163182	8.648914657	8.808345061
10.10278369	9.990505182	10.01524205	10.0672481	9.766131535	9.956562586
6.021063475	6.244906081	6.033044697	6.259323444	5.924154566	5.765826162
7.459710222	7.268778051	7.123768636	7.401460495	7.111135707	7.993428586
10.40925212	9.551451798	10.97767761	9.519086535	10.2692332	11.11230451
7.084441778	7.169190859	7.288727879	7.837840162	7.223856	7.279410566
10.8443678	10.6426412	10.41426339	10.87379507	10.43872423	11.33657706
8.886096818	8.799825313	9.438094949	9.169326323	8.623636919	8.719245818
9.100524758	7.998937818	9.370832535	8.272620253	9.021393263	9.859664869
7.656544636	7.809982667	8.321152475	7.403718889	7.49864798	8.013081051
8.463578343	8.399553939	8.826393101	8.210186616	8.759332636	8.524356212
7.580904889	7.342557798	7.760648222	7.521266808	7.933221424	7.631889737
7.738613697	7.599870798	7.775630727	7.709201212	7.619556354	7.332963121
11.96059797	12.09497108	12.10135206	12.91196246	12.46466592	11.97660652
11.11856721	11.51867768	11.16423752	12.40735915	11.58814301	11.74935785
8.907429495	9.269224374	8.781156657	9.786685869	9.21352099	8.77298297
8.479185606	8.177906263	8.566415323	8.362537455	8.35372597	8.57597697
6.840910293	6.668303455	7.227882465	6.600799566	7.216692263	6.912275354
6.967663404	7.943433747	8.581684657	7.322879626	7.255477111	7.549557061

5.774380697	6.466965869	5.812465879	5.934799364	7.337386909	5.776663677
9.671912455	9.462343535	9.751080646	9.220211768	9.633493677	9.677492576
9.953782444	10.0188097	9.823678374	9.780583495	10.16262583	9.70798304
8.706127333	8.85049999	8.433042727	9.886023253	8.977558859	8.989652131
11.04316622	11.03163187	10.17122401	11.36423723	10.80389925	11.18941159
6.78436696	6.33399804	5.829833838	6.160151404	6.008883727	6.352813182
11.99535117	12.81379045	11.66934136	12.34855768	11.96059797	12.11841559
6.405686404	6.843046434	6.372542859	6.321269657	6.822666394	7.027167101
8.123844788	8.142197384	7.555333263	7.397472212	7.899333596	8.383429707
6.515594273	6.101227323	6.87945998	6.262968212	6.402255576	6.614259212
14.39736901	14.60195877	14.78379092	14.26676228	14.9847254	14.62217632
8.891390919	9.182204323	9.077100232	9.331636121	9.014846778	9.317615606
13.22730043	13.88591407	13.44126502	13.84455043	13.94443	13.5360606
8.623178879	8.284073869	8.96575796	8.983202444	8.527598192	8.583048444
15.02501841	15.86132141	14.94534759	15.99508933	15.12351424	15.46469304
6.537080394	6.781955545	6.606673949	6.899721758	6.862375091	6.432817071
7.931708101	7.960547	8.129067798	8.206690121	7.861832121	7.954205616
11.41605585	11.85033795	11.17910752	11.37490296	11.68948846	11.31449746
15.27762024	14.58095582	15.34617167	14.34357305	15.03840752	14.1282639
6.653830545	6.426632697	7.378508566	6.38452097	6.761583657	6.320887909
6.164463909	5.969585909	6.570263495	5.82210002	6.189267869	5.825744636
6.777724495	7.104098253	7.412474121	6.864476545	6.905589111	6.441889697
10.93023415	11.31852116	10.92060078	11.31267384	10.90192126	10.9991534
9.302103889	9.356741384	8.785177889	9.144457596	9.296131263	9.447991384
10.10631712	9.732604394	9.966143818	9.679645121	9.551451798	10.05053825
8.228066545	7.869709071	8.255317818	7.854325061	8.45110203	8.749346626
11.73271713	11.63174178	12.37416009	11.06433979	11.82852227	11.45738434
12.42233701	12.70390423	12.67378338	13.14611959	12.63888703	12.62807191
8.095330333	8.124973859	8.351407394	8.300483646	7.973992111	8.208692758
8.924680667	9.046362323	8.542859455	8.62460002	8.901331343	8.958493495
10.34514895	9.851320949	10.96311364	10.22557955	10.95849458	10.49348589
7.644626202	7.60736902	7.56320098	7.679285535	7.459115707	7.57318396
8.649462798	9.17861001	8.750392838	9.011040293	8.615330515	8.713417869
8.072110051	8.749346626	7.527339687	7.14692997	6.902984	7.089652374
7.076028737	7.716479172	6.668735424	6.526325283	6.16346097	6.421368414
8.179581869	8.340681505	7.898974697	8.028141747	7.303019677	7.427372788
10.23660558	10.28406896	9.840616414	10.02821235	10.19332507	10.33252085
13.46712151	12.58957491	13.76702458	12.78645268	13.47327752	12.60022648
11.76336308	11.42787377	11.94979793	11.04030995	11.89876792	10.88031852
9.335272182	9.468134182	9.054276	9.517771202	9.278597747	9.251906182
8.921533879	8.942756889	9.643287657	10.20748734	9.593075545	8.888766747
8.63794701	8.095330333	8.980444384	7.883917657	8.93638097	8.092218303
6.133648939	6.109691141	7.232151747	7.271416848	7.33350399	6.261376788
9.261851101	9.294355394	8.989142232	10.05144087	9.210065	9.634173323
12.53197264	12.83827393	12.25572441	12.9851923	12.63102018	12.66985986
7.948408364	7.487078798	7.777311636	7.531209667	7.890421556	7.282635596
9.798623747	10.08599138	9.718307192	10.16355358	9.691258919	9.972781707
8.809951364	9.447991384	9.093526566	9.837575172	9.761765848	9.19736601
8.377090384	8.899254879	8.921533879	9.769994879	9.385837242	8.698751323
14.05855866	14.89443037	13.89331098	15.09418019	14.42673578	14.5108222
6.148359626	6.357735	6.195258121	6.545751121	7.547650101	6.768722596
7.892277636	7.859670455	7.331457121	7.904564374	7.503050071	7.645645939
6.828875111	5.796353323	10.44712342	7.684641212	6.66759498	6.911480061
9.179226364	9.014846778	10.13193078	9.071276071	9.427546879	9.09824599
6.792041707	6.933584646	6.698531303	7.278422556	6.417796273	6.625506687
7.403336687	7.574795778	7.284431525	7.758192424	7.401155333	7.572084505
7.611680859	7.438935576	7.970233808	7.387388131	7.748187364	7.611680859
6.897195879	6.895207929	7.415639222	7.01565997	7.260895414	6.965717616

6.922672051	7.065642788	6.51170598	6.579391333	6.761831111	7.198026636
8.609106909	9.012031232	8.919352101	9.555296242	8.971727333	8.845785121
7.726392606	7.761767192	8.074096707	7.802836333	7.688011354	7.728361283
7.10996603	7.273224596	7.184502222	7.426803586	7.47214603	7.353560545
9.855298394	9.644739414	9.902579253	9.596088263	9.799317404	10.07107569
7.226681909	7.314300525	7.247290424	7.739667424	7.136586475	6.978374222
10.72633412	10.8443678	10.82034332	10.69931975	10.66587479	10.68572341
9.083185818	8.632349838	9.318836889	8.424058939	8.980943495	9.249612495
8.132293172	8.428176394	7.960941889	8.526186556	8.384333949	8.402148465
7.876138384	8.724556485	7.886480404	8.852092202	7.954939636	8.147857556
7.83046296	7.52530399	8.039451586	7.569572879	7.643273222	7.869396606
9.204345657	9.036076414	8.912452162	8.979298788	8.765233303	9.093526566
8.563085788	7.931708101	8.988577162	7.726017717	8.955880859	8.83948901
10.6362571	10.55213977	10.38738452	10.50794857	10.42512206	10.74690378
8.110083838	7.922697606	9.556000646	6.808998949	8.27018399	7.740469253
11.0035084	11.05493438	10.93646713	11.50051559	11.14385742	11.66441291
8.22479296	8.017539697	8.127495596	7.68740998	8.208692758	8.230181798
8.166172404	7.559118222	8.939630657	7.704008545	8.250622253	9.331636121
6.374790061	6.307051293	6.103600394	6.557678737	6.54891203	6.392119051
8.267244596	8.358783152	8.885588879	8.399553939	8.424934535	8.414282596
6.315191152	6.89207597	6.590707929	6.566952202	7.673764162	6.415279879
10.6313464	10.78269205	10.18291229	10.53737043	10.69222123	10.60635433
9.423636879	9.785983535	9.251353071	9.277455131	9.294355394	9.545881535
6.564057303	6.579612929	6.357347162	6.497644434	6.840143455	6.632781101
8.19458801	8.436545051	7.501777556	8.803482152	8.108917222	7.940608939
8.420871687	8.134790222	8.083485404	8.334587636	7.835434535	8.581169111
6.466965869	6.813695162	6.555734869	6.605442354	6.779434273	6.443888202
12.51066587	11.92665931	12.95303817	11.55106122	12.75784116	12.18792926
14.42673578	14.24162707	13.84455043	14.11151028	14.09521817	14.52085241
12.80602179	12.68097339	12.53197264	12.42233701	12.61449239	12.73731843
15.13975511	15.02501841	14.86987024	15.08134613	14.9591327	15.13975511
14.1830221	14.13521971	14.15092087	14.11997744	14.11151028	14.38889659
13.95955505	14.05136004	13.68884286	13.94443	13.87263802	14.25042984
11.22600179	10.96162926	10.90324033	10.79420974	10.79686873	11.06433979
12.55983323	12.48458958	12.25844332	12.31419772	12.41033676	12.55983323
12.87421343	12.71520416	12.58612865	12.66167346	12.54906863	13.27940818
13.39659876	13.29684189	13.28559105	12.9709957	13.18858184	13.63435472
15.95256028	16.09492117	15.59877501	16.06827159	15.81050193	16.52993947
10.88430477	11.2278827	10.81664077	10.62536143	11.06433979	10.98037262
12.69316533	12.65819872	12.50706636	12.6076243	12.61090773	12.83405786
12.19727311	12.17513939	12.63102018	11.49839615	11.24323027	12.24351077
13.42888134	13.34204398	12.99565877	13.09977851	12.94821125	13.1944969
14.7172184	14.6970922	14.53168901	14.93426708	14.80749806	15.15443825
14.53168901	14.48025691	14.30038792	14.46162431	14.39736901	14.7172184
11.93975968	11.76336308	11.93486375	11.78023213	11.83813795	11.92665931
9.480384485	9.473962505	9.50710198	9.491039162	9.497817424	9.344897333
13.92894576	13.93694444	13.50997822	13.58209631	13.40990013	14.13521971
13.72760196	13.68884286	13.31405593	13.34204398	13.29684189	13.60269863
12.70390423	12.59668127	12.68484185	12.58612865	12.60022648	12.90783816
14.79546777	14.9591327	14.5108222	14.62217632	14.65397593	15.01206542
13.80033164	13.56886427	13.71423947	13.60979651	13.49745762	14.37933382
14.21449649	14.07988992	13.9071606	13.88591407	13.9071606	14.15092087
10.87637444	9.898158111	11.46539851	9.952967535	10.60743282	11.57178154
13.64789782	13.35359128	13.23341018	13.2504616	13.1944969	13.83772389
15.449005	15.61926687	15.46469304	15.54332231	15.58150095	15.63778652
12.0750029	11.84529477	11.67371856	11.6445291	11.76101506	12.00354228
13.52959744	13.30255942	13.21058954	13.31405593	13.44126502	13.58996905
12.65472241	12.60396528	12.27773509	12.43279956	12.39348455	12.86955734

10.9102587	10.73661507	10.2829717	10.46232362	10.44712342	11.05651949
13.67554111	13.82538175	13.39037726	13.78737352	13.65450155	13.6823979
13.08418191	12.7452022	12.7494647	12.78185519	12.68484185	13.10484893
13.43523914	13.42888134	12.95787308	13.23341018	13.2158469	13.29117716
16.49551516	16.8710039	16.33688632	16.96059838	16.25437967	17.07010739
15.78787594	15.72204116	15.63778652	15.86132141	15.72204116	15.72204116
12.83405786	12.88708268	12.65819872	13.10985871	12.80166365	13.42153085
14.15092087	14.02572128	14.223913	14.1904887	14.32804886	14.32804886
13.76702458	13.60269863	13.83772389	13.5964752	13.72760196	14.08841453
15.22815255	15.36066179	14.93426708	15.29549959	15.06622261	15.21350605
15.29549959	15.42966281	15.02501841	15.32551587	15.16803204	15.67809237
13.2504616	13.22730043	13.03496614	13.04869312	13.17778072	13.30255942
15.81050193	15.74256723	15.39581235	15.74256723	15.42966281	15.86132141
13.90036211	13.78027417	13.70717091	13.81935116	13.86604428	14.17411554
13.81266894	13.7211242	13.92894576	13.97489728	14.05136004	14.05855866
10.91514001	10.85513517	11.28053313	10.64005918	10.72786832	10.92485902
16.3077943	16.61084374	16.09492117	16.7804214	16.2036826	17.01900663
14.74028573	14.7172184	14.59173737	14.88238784	14.74028573	14.9847254
13.98081649	13.81935116	13.5360606	13.64789782	13.52959744	14.29173122
14.99939481	14.9727616	14.76275492	14.9591327	14.70594079	15.09418019
13.20018245	13.21058954	13.10985871	13.15645152	13.05987966	13.69473258
13.04381444	12.92081703	12.66627862	12.91656586	12.797444	12.84627851
14.02572128	13.90036211	13.92172837	13.95955505	14.08841453	14.19920898
12.09225922	11.93975968	11.7894918	12.00644476	11.99323436	12.27773509
12.29548604	12.41033676	12.07810634	12.14507673	12.28105924	12.50706636
11.1031408	10.87907816	10.86305205	10.63870636	11.27153548	11.64848719
15.65613201	16.02065153	15.38120792	15.90458538	15.61926687	16.14978837
10.99469779	11.17041501	11.05356995	11.12454893	10.61480751	10.75062885
8.939019091	8.437457535	9.193817232	8.640789455	8.897751606	9.558925838
8.271029889	8.140538697	7.758192424	7.610574101	7.583998525	8.257595869
9.466909636	9.365016101	8.633351606	9.161459798	8.992957212	8.600356081
12.92081703	12.93430142	12.6076243	12.7939548	12.81735602	12.89113428
16.36806566	16.7804214	15.92931651	16.64557528	16.09492117	16.73234105
11.75908761	11.66934136	11.45932673	11.60680648	11.59870577	11.83813795
9.431415586	8.947127939	9.831834717	8.998420707	9.339095222	9.167758485
10.18468509	10.26224218	10.15379546	10.85361094	10.33375706	10.72246175
7.350971899	7.248166182	6.97940904	7.621022111	6.869142343	7.148774485
10.07956055	9.55462702	10.26445444	9.564343333	9.966143818	10.14326599
8.284073869	8.32400903	8.226500707	8.239474646	8.088117667	8.213832515
9.542425758	9.760900556	9.196689051	9.417037424	9.710054737	9.578067192
8.017104828	8.307093606	7.87816304	8.514037303	8.019097616	7.98285402
8.988033424	8.562664263	8.39402401	9.156208313	8.639872707	8.665646899
7.569572879	7.263531485	7.791244131	7.55178802	7.468792788	8.202220545
10.19136651	9.86640601	10.21583282	9.76546198	10.21583282	10.19957169
8.873520545	8.666601818	8.793874616	8.868979273	8.719668061	8.632799758
6.55006301	7.20918101	8.339546939	7.06161799	6.958715091	6.824345162
6.337204768	6.00930701	6.398440768	6.142072515	6.039925859	6.377923929
7.653990838	7.430512737	8.05474504	7.463067838	7.610976808	7.12541897
6.977525061	7.144617717	6.978374222	7.168718192	6.858224505	6.734736768
7.409045616	7.703321374	8.600356081	7.59435096	8.148308414	7.644626202
9.705000525	8.843670869	9.881176566	8.476085384	9.612498475	8.894948323
9.253086273	9.424273283	9.094691273	9.27419499	9.439397061	9.289535939
7.18511501	7.201570444	7.289658101	7.474688838	7.31189602	7.023680051
10.70775781	10.68960982	10.55070525	10.89912578	10.61001127	10.58117749
7.466342606	7.360451687	7.554702939	7.003455566	7.761767192	7.355706667
8.84786598	9.124970051	9.384699788	8.558556152	8.933180404	8.71485401
12.02240324	12.35798465	12.17216641	11.80554878	12.18792926	11.77802923
7.788172758	7.710578727	8.105207222	7.716120232	7.719835253	8.420387707

8.614861051	8.212243444	9.526200354	8.320817869	8.808928141	8.841575667
9.653591273	9.508442768	9.52752797	9.176817596	9.516398909	9.528804131
9.805520727	9.873591818	9.485668061	9.601568657	9.964343626	9.759931222
7.769805091	7.60576001	8.230588212	7.665645141	8.093077828	7.457359414
8.243245818	8.047553323	8.291166909	8.076845152	8.02698897	8.020484273
10.79420974	10.75178718	10.37124354	10.58812248	10.81796524	10.76857843
8.874692596	8.456593071	9.397864101	8.709941838	8.78563097	9.050265121
9.565660949	9.339095222	9.497084828	10.18000397	9.74383503	9.767642525
12.30789527	12.11562205	11.86922315	12.25572441	12.0750029	12.15130062
10.95608584	11.11388317	10.99046505	11.58195332	10.66465811	11.05493438
12.96241124	13.24400831	12.97977545	14.10328164	12.84197228	12.34577038
14.08841453	14.21449649	14.33555643	14.65397593	14.17411554	14.010587
14.91973608	15.32551587	15.03840752	15.52328152	15.01206542	15.38120792
16.22759729	17.01900663	16.3077943	17.49517975	16.36806566	16.68963031
13.50411017	13.67554111	13.7211242	14.35197184	13.52334642	13.47832004
7.60282502	7.720541949	6.542523919	7.501110505	8.227270172	6.568654859
13.20536123	14.5108222	13.33155169	14.05855866	14.13521971	14.50017253
7.114645596	6.848551889	6.992656859	7.277849899	6.784609556	7.507037566
8.456593071	8.682574626	8.265565879	8.36093298	8.83948901	8.410410394
14.5108222	14.91973608	14.35197184	15.21350605	14.84412351	14.88238784
7.248451727	7.447717303	6.922161646	7.032840081	7.343750657	6.885887889
7.248766182	7.415309101	6.925960677	7.06536003	7.282065253	7.397737788
8.411285283	8.189547909	6.858444758	7.587635919	8.267244596	7.428935273
7.920877394	8.450171172	7.843855828	8.13267903	8.561789232	8.053971495
6.764835354	6.403910828	6.917518081	6.321067485	6.646173444	7.055909434
9.987077505	11.00902763	10.85225864	10.41022215	10.7982674	10.1597097
8.475210818	8.919862162	7.988882495	8.903025495	8.802954374	8.455403657
8.859179455	9.009407333	9.082448343	8.788135667	9.033915556	8.566871343
9.409395929	8.99408802	9.350012182	8.459596535	9.208368677	9.060618192
9.677492576	9.901640636	9.749660394	9.964343626	9.948421707	9.902579253
9.483552818	9.642500081	9.417594202	9.678297182	9.243096889	9.271123182
12.31419772	12.25251148	12.21991364	12.68903729	12.27421104	12.29886409
11.68700199	11.58814301	11.72126504	11.84529477	11.79870316	11.98196729
10.85654613	10.82290783	10.37467131	10.66832974	10.27432009	10.97185531
7.987573434	7.889381343	7.503972212	7.820222687	7.896600606	7.860764394
7.91707401	8.235056909	8.247392788	8.496814303	8.494130061	8.519692939
7.519364889	6.973893828	8.01423197	7.121055596	7.707249808	7.76948301
9.071276071	8.486053202	8.969483	7.952774121	8.893968333	8.594455404
7.704350384	8.302900242	7.940978939	8.40783399	7.972012263	7.842683778
10.80499832	10.63752253	10.59180142	11.01683173	10.58689262	10.97631922
7.689906808	7.337670899	8.082778869	7.308421545	7.756889051	7.435437465
6.92551101	6.699888758	7.322217273	6.791252535	6.889250929	6.705415545
7.50339202	7.743136949	7.301216929	8.31912796	7.87816304	7.559825556
9.886762101	9.573320202	9.903372323	9.663106646	9.90675497	8.895503556
11.99323436	11.87175588	11.89130603	11.9982543	12.01673563	12.72972704
8.866888202	8.93587504	8.691979949	8.845226141	8.609511	8.559011545
8.451550535	8.094605657	9.519820636	8.31318196	8.796283929	8.85518403
7.064243152	7.103541778	6.463509232	6.827921778	7.379463242	6.426421657
6.297066051	6.145037	6.452749343	6.283913465	6.300351141	6.363691192
9.887613414	9.345614434	10.01703805	9.444057586	9.562364657	9.940265465
9.588958727	9.483552818	9.687758929	9.731150899	9.436898869	10.08231743
8.60560303	8.24122704	7.964179182	8.445297313	8.302062758	8.503920141
7.000309313	6.808010384	7.332078838	6.813409273	7.083613121	7.276635293
6.729937747	6.898792364	7.482605354	7.228468091	7.089056525	7.053026414
10.05607537	9.895061889	10.77387872	10.11941657	9.541747455	10.08599138
10.18198959	10.49466708	10.04682541	10.17613521	10.04682541	10.30810853
9.103811545	8.894948323	8.904793061	8.792768747	8.722452253	8.449722687
11.39594259	11.46936418	12.43600068	11.50261564	12.47441306	10.25220538

9.621550919	9.94402302	9.402814081	10.29000276	9.732604394	9.869466131
8.807959646	8.700142556	8.441767061	8.710842768	8.680072242	8.608819697
10.37026232	11.0473733	11.68243664	11.93486375	11.911688	11.75215821
8.996148737	9.034403333	9.107945202	9.315088131	8.987499273	9.159785394
6.604297121	6.834065141	6.920666222	7.477877475	7.262118616	6.946968899
8.678158051	8.531655515	8.705145061	8.115023838	8.629474212	8.800397414
9.339764051	9.550769788	9.476588061	9.039454747	9.721175788	9.061649657
7.235668455	6.961758566	7.332963121	6.680925848	7.232151747	6.653328071
10.3380268	10.16743096	9.850553616	10.05880999	10.36589334	10.28406896
9.436898869	9.350012182	9.379720636	9.632768747	9.402230293	9.324388263
7.74646602	7.035145869	8.690918616	8.055171202	8.348543172	6.973416747
8.269818727	8.362142131	8.012698303	8.102124455	8.136235949	8.231756293
9.86640601	9.49510903	9.688441414	9.057196687	9.800904051	9.992988556
8.207099091	8.760291222	8.493155899	8.841575667	8.601386131	8.524867051
6.47989397	6.534227879	6.478552354	6.382613596	6.310688818	6.293110242
10.99763472	10.82945836	10.40701089	10.61261659	10.78014639	10.8254891
11.23280633	11.27153548	10.9660467	11.16423752	10.99623862	11.36065784
9.256767414	9.756054152	9.66831603	9.862946677	9.410728505	9.199527535
7.845981061	7.900715717	7.47214603	7.442908828	7.671593667	7.775269606
11.45176908	10.05780386	11.98999321	10.32309273	11.74935785	10.99345288
12.93430142	12.43944324	13.62106704	12.65104366	13.48990673	12.53197264
8.730448515	9.261851101	9.239472869	9.809529798	9.193130899	9.646274414
7.446082798	7.775630727	7.897743687	7.126519737	7.74578199	6.674272111
11.75462574	11.83330316	11.79615897	12.24649354	11.56988377	11.70348036
7.932066141	7.730154939	8.314398727	8.184050869	8.107705111	7.527339687
8.472435657	8.213832515	9.024212859	8.104866828	8.543234566	8.671294253
8.141770949	7.845242687	9.122138505	8.295009141	8.970028374	7.671246505
11.62059475	11.89628941	11.68948846	11.69658017	11.79171062	11.46145043
7.520306162	7.480656747	7.674820101	7.598885727	7.661901192	7.859293
7.949071818	8.427236737	9.155642525	9.942152202	9.024212859	8.510413414
11.97447062	12.11216258	11.84027237	11.93975968	11.86401838	12.18492446
11.94745099	12.51424883	12.20640951	12.63102018	11.88375452	12.20640951
10.33252085	10.3380268	9.916104475	10.83790729	10.36687712	10.06450841
7.242650333	6.836969222	7.471589222	6.883018111	7.021219939	7.480348535
11.22294817	11.75908761	11.85033795	12.10951308	11.15666056	11.63174178
7.445127778	6.860390606	7.993077394	7.009988212	7.52238453	7.509397909
11.47871073	12.00644476	11.41238356	11.67593314	11.39185194	10.99046505
7.392218323	7.529315566	7.712213273	7.409045616	7.668279394	7.106035242
8.437457535	7.886480404	8.611286687	8.152087687	8.501579646	8.644542606
5.879961677	6.28064496	6.504049202	6.184170131	5.940904263	5.853647859
10.00094823	9.604175636	10.10841911	9.214694788	10.36903954	9.571117879
8.324880909	8.323157747	7.785214909	8.123844788	8.010919919	8.301264212
6.824533111	6.868352737	6.449508273	6.720840657	6.582941808	6.755924879
7.59859104	7.325469747	7.174161374	7.460268556	7.247290424	7.923048354
6.668303455	6.364283747	7.053922394	6.384073253	6.508494242	6.97260104
7.460268556	6.987320737	7.843485626	6.986766929	7.467207283	7.598885727
15.34617167	14.80749806	15.41210544	14.36999747	14.99939481	14.61202705
10.83100199	10.54692936	10.75600216	10.76471511	10.32208546	10.93023415
10.52585606	10.37467131	10.26224218	10.27432009	10.44094498	10.92060078
11.94481318	12.09804166	11.31267384	11.8164662	12.03068847	12.0724233
7.40744601	7.566773798	6.977041091	6.893115293	7.400588576	7.332344778
8.865915939	8.683087172	8.48873997	8.522156747	7.93437196	9.07419996
9.298556212	9.599465606	9.118059475	9.45706402	9.314493212	9.526870273
7.714004283	6.630903202	8.304169929	6.843046434	7.181436869	7.836138323
10.42297323	10.72127155	10.09896607	11.03452834	10.34830463	10.7982674
10.44832821	10.28089537	10.42409385	10.14432724	10.51771241	10.35591393
8.602859283	8.491490828	8.626833051	8.589388657	8.409959899	8.832790172
9.341222778	9.222631263	9.671912455	9.463745455	9.555296242	9.698181576

7.114362495	6.922161646	7.139090576	6.76309904	6.644450293	7.06161799
7.995268899	8.121954505	7.60736902	7.793711495	8.274284293	8.06213903
7.705454838	7.634532263	7.446760616	7.042911333	7.512765949	7.206984404
9.580783667	9.54312803	9.725631121	8.904793061	10.01168912	9.174793152
8.246470545	8.469339576	8.428657566	8.918756414	8.302498081	8.198946162
7.065103374	6.628556657	7.36298202	6.597486293	7.00817199	7.426469556
10.7982674	10.59541023	10.75737971	10.57758511	10.73384189	10.23850383
7.831854586	8.041000495	7.821271606	7.464704768	7.969793182	6.984849141
11.97660652	11.92156179	12.35168631	11.74935785	12.06692054	11.81439214
10.03026493	9.882779162	10.4283734	9.928455091	10.16937171	9.949411172
9.719830172	10.03492192	10.42512206	10.38430498	10.38430498	8.721054424
6.304241131	6.006053525	6.330516949	6.141021192	6.075380768	6.483130091
11.13240418	11.32559397	11.36423723	12.19727311	11.56291631	11.0035084
7.470329323	7.408375899	7.945472162	7.193876586	7.528004313	7.659822475
8.69723302	8.112161727	8.41382397	8.258860253	8.211847566	8.338686465
6.129263667	6.283373303	6.461904303	6.084226273	6.977041091	6.019346071
10.96000427	10.78551178	11.54675633	10.51291939	11.29399858	11.58005536
9.140978061	8.193820172	9.360502949	8.414679525	9.157435455	9.808672222
6.478358667	6.446834051	6.436711636	6.271385515	6.327126061	5.864197879
13.7211242	11.45526933	12.63888703	11.35080354	12.05813886	12.42943771
10.2166926	10.44832821	10.65246846	10.59685822	10.26445444	10.17030341
8.279462	8.59942799	8.710391727	8.995099212	8.205457293	8.063008152
10.18762619	10.40387449	10.02269473	10.65127749	10.32946621	10.12799521
6.926979111	7.272599475	7.063222061	7.650531152	7.605411525	6.975978909
7.08136002	7.12322297	6.674983667	7.216096788	7.164102909	7.09298796
10.0079343	9.249034202	10.57028524	9.120933788	9.853021111	9.660861778
12.22629088	12.38063147	12.12939166	12.03310273	12.18142186	11.85033795
11.31267384	11.23116994	11.13085117	11.17378617	11.12935449	11.55928848
9.151431899	8.520243879	9.328711051	8.986432162	8.036816444	8.775001677
10.34418014	9.511202364	9.385290182	10.22361636	9.830354566	9.853774707
7.531209667	7.849137545	7.562928222	7.805761556	7.301216929	7.519990596
7.461509737	7.587331949	7.700026646	7.935081758	7.519695475	7.711178283
8.414282596	8.307893798	7.960192616	7.937437293	9.190767364	7.970926879
10.06547375	9.908305848	10.25127201	9.769223525	10.04963797	9.824540899
8.22437102	7.794719919	8.658285303	7.503050071	8.217270354	8.433042727
10.92060078	11.21958266	10.86566339	11.05191968	10.93334198	11.00620373
10.84844593	10.85361094	10.89755671	10.68572341	10.91766747	11.02700786
8.152087687	8.689422596	7.75479003	8.972927475	8.440058758	8.240375404
8.23581501	8.598008343	8.33796001	8.962353222	8.685647889	8.002717273
7.384295283	7.450590374	7.193007566	7.714317071	7.492937667	7.307562737
8.59942799	8.629474212	8.337550606	8.793874616	8.780633818	8.791137414
8.59026096	8.653441707	8.852576737	8.588536172	8.813032152	8.696739152
7.181699919	7.166695253	7.398977253	7.296277414	6.986217889	7.30728296
10.11246778	9.883591758	9.758431737	9.996420616	9.515743253	10.00985275
8.283707475	8.126253929	8.022720303	8.192996515	8.400464071	8.565878434
11.5737214	11.58195332	11.01209098	11.41071085	11.49029795	11.62290433
9.005433152	9.185002364	9.258612929	9.59460096	9.033394545	9.655088667
8.905887879	8.968391182	8.994589434	8.997807768	8.746767313	8.920488949
10.74945851	10.56655729	10.99763472	10.65014074	10.92757694	10.4700758
10.68960982	11.06433979	10.66945215	10.85654613	10.55070525	11.09885051
9.338458455	9.359207242	9.276789111	8.98371896	9.87177298	8.910531141
7.175214949	7.760294414	7.142295949	7.279114576	7.227304778	6.805470263
8.034450929	7.902575141	8.22479296	7.615524929	8.619347838	8.08075902
9.053706202	9.238865747	8.897751606	9.451212687	9.179819687	9.087865687
10.59180142	11.14866374	10.70181173	10.93494087	11.10035391	10.58579258
10.08145981	10.12909315	10.29851595	10.53141254	10.03123363	10.03876748
7.366646525	6.954196414	7.660499434	6.867131162	7.805446323	6.762568667
10.82664672	10.76239578	10.93789932	11.05959848	10.55213977	10.90057618

9.279768394	9.596088263	9.123297364	10.02161965	9.180906778	9.260607
8.510894717	7.690840778	8.70424704	7.476910626	8.442699879	9.018299374
13.78027417	13.99495772	13.82538175	14.4427031	13.85099976	13.09463792
11.80554878	12.16036216	11.74670945	12.66985986	11.671419	12.06098883
6.441470343	6.817799576	6.887288828	6.979690485	7.067324051	6.527432758
9.31393701	8.645985424	9.886023253	8.334981101	9.464440242	9.024212859
8.878201818	9.528176495	9.244399414	9.950180667	9.503271071	9.385837242
7.151031394	6.800063838	8.01013803	6.819920253	7.357173859	7.226157253
7.301802939	6.968393455	6.906092707	6.796334212	6.952529081	7.000099141
9.250770152	9.576740677	9.026636737	9.038849273	9.276789111	9.34289298
8.085049192	8.150448232	7.744142424	7.63758797	7.952386505	8.206690121
10.35284213	9.378583414	10.46442497	9.058356636	9.971733293	9.803356646
6.897750404	6.79323798	7.257525758	6.723761636	7.21289504	7.002156515
8.478263182	8.978723121	8.550414556	9.106823152	8.62460002	8.496814303
8.143776848	7.456429202	8.355387535	7.191532202	8.187968455	8.552719747
11.5386618	10.96000427	11.55524693	9.416442616	11.34732253	12.10951308
9.402814081	8.920488949	9.325601172	8.712915394	9.187244566	8.879330566
8.163923192	8.634342798	8.460863192	9.119831202	8.412578899	8.278157657
6.958444212	7.050347636	7.004269242	6.747571626	7.117635758	7.381027636
6.97366599	7.351945465	7.358688323	6.941216071	7.287539616	6.638813101
8.219832939	8.643157737	8.473802646	8.39308204	8.350987111	8.687053253
7.940978939	8.419470808	8.071334404	8.093473121	8.22479296	8.624974828
9.229670424	9.566253768	9.056533626	9.234418071	9.644739414	9.182204323
10.04511943	10.51886057	9.952967535	10.2187154	10.30470526	9.999132495
8.729485838	8.24438497	9.051375475	8.833337414	9.126080354	9.503271071
8.141366242	8.559011545	7.91307104	8.137069859	7.993428586	7.572483949
11.12151875	10.65365596	11.39185194	10.91356479	11.15342364	10.77252405
8.677707162	8.118473111	8.598980253	8.24122704	8.430534091	8.268510495
14.37933382	13.79381649	14.88238784	13.67554111	14.34357305	14.24162707
9.907546465	10.41224321	10.18000397	10.84974643	10.19418334	10.04600105
11.1502402	10.7442872	11.46145043	10.78408516	10.88681216	11.238428
7.387388131	7.096340343	7.668960768	7.341641707	7.355380455	7.022261949
10.080472	10.01524205	9.580097263	9.870221061	10.23247836	10.54078574
10.39521192	10.81796524	10.64005918	10.44934262	11.14866374	10.69682519
8.875711838	8.871686232	9.084956182	8.957404525	8.620285051	9.10558002
7.693516556	8.57790302	7.74646602	8.653441707	6.819416596	8.845226141
9.454390232	9.684048687	9.356741384	9.329922273	9.881176566	9.383480283
10.76471511	11.4090051	10.83501466	11.47322198	11.28370047	11.18636156
7.088821283	7.632188616	7.084738172	7.79851096	7.336756222	7.463067838
7.557268586	7.066192545	7.834378071	7.516753121	7.425828556	7.385541293
10.66706732	10.8193093	10.68452674	11.56725755	10.93646713	11.21668249
6.117409697	6.57287004	6.196823949	6.300754434	7.669946162	6.074946343
9.063885091	8.041332778	9.334658071	8.041792596	9.039454747	9.719000626
13.68884286	13.09977851	14.14208591	12.2293359	13.26276274	13.18858184
8.382194192	8.458697343	8.193820172	8.35752198	8.24438497	8.464081152
7.370661465	7.759566061	7.253230737	7.438305869	7.688677182	7.552449879
8.539776737	8.381754576	8.53715797	8.534432515	8.687053253	8.672348475
7.686071172	7.426803586	7.645321707	7.545986354	7.25519196	7.651222556
15.72204116	15.88528111	16.12384164	16.68963031	16.02065153	16.09492117
9.027655758	8.467263475	9.159196141	8.08537204	9.36439596	8.381754576
9.446464909	8.842639202	9.027136657	9.060618192	8.947127939	8.404822414
7.501443394	7.964914919	7.231529313	7.740469253	7.488894061	7.525850576
10.07670007	10.26445444	9.752732475	10.11760527	9.999132495	10.37124354
10.05144087	9.098746404	10.4450719	8.905887879	10.56802411	8.654464586
7.762794545	7.828584192	7.617890202	7.868722081	7.778362485	7.889031475
7.272331859	7.701029869	7.818322879	7.74753198	7.187213162	7.091328697
6.580062899	6.585712788	6.41609998	6.450135586	6.528858343	6.739790818
8.694840596	8.765233303	8.166983455	8.418553323	8.798818646	8.554027364

10.2761618	10.24612484	9.807061475	10.45375986	9.905938879	9.652169778
9.429506515	9.191263899	8.893419636	8.946107263	9.277992232	9.146178758
8.57971698	8.709941838	8.593558293	9.126080354	8.376704939	8.625920364
11.00754401	10.80499832	10.79284767	11.31852116	10.49466708	11.07493153
8.042974687	8.52575703	8.086953081	8.691458141	8.032957859	7.837132667
8.25570701	8.631899566	8.219387636	8.520243879	7.949790515	8.174468182
6.45826596	6.065202949	6.571183485	6.374400737	6.252603323	6.212018222
9.562994313	9.515743253	10.05970796	9.654354424	9.899010949	9.613873859
9.093526566	8.049757475	9.640429374	8.362142131	9.549314313	7.921213404
9.702758343	9.526200354	9.889177586	9.130293909	9.832711535	9.570470929
9.097596465	8.03601997	9.803356646	8.035288182	9.057196687	8.531655515
6.824345162	6.232724131	6.900739596	6.331316808	6.507801384	6.789286121
7.214035101	6.598211152	7.730873535	6.629250202	7.172666465	7.389870293
7.494510091	7.456122566	7.28497204	7.264201869	7.953840929	7.354199768
8.239069586	7.789255535	8.904239657	7.614146707	7.907575465	7.959080606
9.496413788	8.457043091	10.03876748	8.532516465	9.318228697	8.919862162
9.639623879	9.216574283	9.66383	9.758431737	9.26122399	9.341222778
7.309668525	7.445127778	7.229904545	8.372433475	7.380085202	7.704008545
11.21187068	11.308834	11.12001247	11.24001011	11.0035084	11.17213305
7.519695475	7.537817576	7.538502939	7.463713737	8.265965545	7.180291929
6.454504667	6.223158818	6.614504818	6.261563212	6.514775394	6.660088081
8.724013404	8.320817869	8.964642636	8.236633323	8.570262	8.830117929
7.362377768	7.427372788	7.043463859	7.297158313	7.03146702	8.187136596
6.644999202	6.73278298	6.876638434	6.751729354	6.893883192	6.723761636
7.531770848	7.547922717	7.756512818	7.897743687	7.262427071	7.380085202
9.082448343	9.785178838	10.12041459	11.1031408	9.298556212	8.595703707
9.013830768	9.260607	8.847386828	9.237664879	9.101613848	9.437491071
7.068665737	6.764115131	6.825565162	6.977291071	6.733232384	6.791252535
8.412145273	8.788135667	8.701109788	9.072403576	8.594861495	8.449337667
11.71443529	11.59870577	11.89876792	11.47871073	11.50814936	11.86401838
7.882189879	7.819085697	8.144584758	7.954939636	7.676956101	8.006915303
10.85923156	11.17571904	10.73384189	11.07797425	10.57758511	10.75178718
9.461673394	9.522660222	9.318228697	9.977292899	9.368224626	9.52752797
10.59418919	10.20358283	10.80782516	9.831834717	10.83501466	9.868690293
9.59460096	9.132080535	9.609027141	9.963504081	9.550769788	9.543825273
10.45375986	10.92608881	9.977292899	10.63266161	10.71503879	11.02105469
10.37346225	10.55842197	10.15483313	10.36903954	10.58579258	10.14432724
10.05780386	9.183367525	10.46664928	9.165786899	9.611261414	9.070117818
7.725703576	7.555972677	7.524103909	7.246435697	7.097545323	7.097545323
9.604846424	9.584100455	10.35069541	9.293389859	9.041236566	10.29101087
12.17513939	11.59654933	12.64265677	11.33657706	12.24097391	12.63102018
8.417604414	8.798247798	7.943433747	8.299695162	8.651919778	8.588536172
8.200539131	8.334162556	8.250194202	8.632799758	8.06213903	8.541972566
8.474658303	8.825911939	8.421742051	8.752686475	8.86638896	8.486543404
13.99495772	12.82153585	14.4427031	13.0147344	14.59173737	13.33661988
6.871904646	6.730688788	7.160791343	6.601717263	6.92164503	7.059356717
10.98749259	10.80900786	10.79420974	10.687913	11.04030995	10.573928
10.41731261	10.54441943	10.12224713	9.975431768	10.35284213	10.19418334
10.04160563	10.00886723	9.815893677	9.800904051	10.10736711	10.31795252
12.86955734	12.92542832	12.84197228	11.93770961	13.10985871	13.03025597
9.328020364	9.196132455	8.604635949	8.849981545	9.003925646	9.229670424
11.40495249	11.10460602	10.96000427	11.06573849	11.05651949	11.13400706
8.496814303	8.640789455	8.614442374	8.920488949	8.784649081	8.437898414
7.652632424	7.392218323	7.317590788	7.252580253	7.201015091	7.769805091
7.480348535	7.609879768	7.356281616	7.560760293	7.175476838	7.605113071
8.464081152	8.722953263	8.32400903	8.669446475	8.556855828	9.031713778
8.200961636	7.747885899	7.651222556	7.594662899	7.712540141	8.005371424
10.95849458	11.31661678	10.82290783	11.68243664	11.19447199	11.07013707

7.81061403	8.364710758	8.267698071	8.406604404	7.655611	7.892604899
8.397394545	8.236217485	8.387507626	8.091323071	7.962680434	8.237365566
8.265565879	7.332344778	8.642670768	7.362377768	7.873377879	8.367422212
12.10951308	11.80554878	11.9875501	11.9577766	11.98196729	11.81221198
8.10140802	8.225777414	8.187968455	8.509502414	7.981345788	8.273061586
8.86638896	8.967895455	8.673363869	8.728449242	9.131560131	8.801904485
11.1060681	10.78014639	11.20660223	10.69682519	11.30506756	10.80782516
10.11760527	10.68338253	10.02439619	10.72900488	10.61867663	10.47370876
6.514775394	6.337018061	6.654064545	6.224797737	6.430045889	6.503188616
7.401460495	7.489478697	7.762120596	7.627030141	7.517772121	7.58864003
11.49029795	11.37847359	11.38025327	11.43380494	11.53677901	11.72355631
9.703591263	9.925979414	9.428845576	10.25627793	9.595333525	9.726443586
6.829819869	6.499057505	6.982135444	6.499941	6.789755162	7.155245384
6.431584121	6.253499364	5.759408909	6.469635131	5.89783198	6.378573141
8.011643808	8.665646899	8.063461202	8.476485121	7.444823313	8.476994374
14.59173737	14.9847254	14.60195877	15.61926687	14.67552064	15.03840752
8.936938576	9.214694788	8.252794354	9.587719636	9.041820404	9.028300162
10.31013098	10.1300726	10.32208546	9.969141677	10.32849396	9.749004051
6.836753071	6.508047626	7.097914788	6.443018677	6.606886212	6.906817121
10.07479617	9.869466131	9.987874111	9.651465606	9.943097475	9.432636424
12.87819387	12.31719428	12.65472241	11.73070023	12.01956714	10.4549623
7.425506818	7.331457121	7.555972677	7.628336929	7.334908263	7.578628212
10.93171462	10.57159251	10.71279176	10.2861211	10.55966675	10.29208493
10.96311364	11.24993851	11.24323027	11.52052132	10.67815746	10.90192126
8.466446051	8.322342051	8.497259596	8.128290707	8.435296202	7.844877657
11.8358519	11.47496104	12.11216258	11.03737422	11.83072395	11.08936677
8.386601707	8.562227051	8.263041909	8.50673198	8.08075902	8.392711081
7.081850444	6.548235576	7.475955232	6.657421566	6.905316545	7.391342576
8.681579566	8.050484323	9.400967465	7.561964414	8.326162172	8.610337293
7.712908172	7.134092121	8.492747404	7.011036525	7.737649848	7.658839354
15.63778652	15.48449671	15.81050193	15.02501841	15.83165575	16.04217659
7.133298475	6.758395253	7.498029465	6.757884566	7.444499747	6.651718455
8.208692758	7.917722121	8.367422212	7.813759899	8.387507626	7.387388131
8.450171172	8.349393212	8.114611232	7.942803596	7.516138929	8.690918616
10.73661507	10.70775781	10.5397012	11.04316622	10.60635433	10.61743608
8.135177152	8.507703202	8.029836697	7.925642	8.443175586	7.85268101
7.356007838	7.650231051	7.874063444	7.685352364	7.540434798	7.097914788
7.587331949	7.865213	7.581254909	8.345353354	7.576363505	7.626793707
7.711523556	7.758192424	8.484240687	7.472789818	7.507381747	7.782462626
9.977292899	9.496413788	10.58689262	9.084956182	10.04600105	10.00886723
8.779146061	9.084956182	8.803482152	9.438094949	8.225261616	9.593075545
7.169717707	7.478803566	7.542064859	7.626793707	7.207790596	6.618511313
13.38447499	13.64083669	13.04869312	12.97977545	13.73439002	12.81735602
8.625486818	8.78563097	8.511758747	9.164545343	8.618800343	8.87289497
6.461471737	5.786127747	6.079607434	6.602227	5.812465879	5.851911838
12.2293359	12.15440913	12.45390404	12.3774088	12.34855768	11.92937262
8.560348606	8.198946162	8.549970081	7.984288364	8.538380869	8.147481596
6.361367657	6.387499121	6.568654859	6.412595495	7.064817384	6.347255636
9.589694828	9.393934131	9.949411172	9.185002364	9.89348604	9.127251283
7.194433949	7.418614091	7.178323677	7.23423804	7.380686859	7.065642788
8.722953263	8.508645727	8.263464364	8.907915899	9.036569232	8.283707475
13.79381649	12.92937388	13.88591407	12.47441306	13.3718573	12.10694646
8.137860071	7.643273222	8.259704283	7.913871889	8.452374444	7.738299929
8.630379313	8.444212369	8.410908071	8.682101313	8.77962597	8.521696414
8.296772869	7.901142576	7.937868929	8.217667525	7.92237903	7.678291495
13.1944969	13.84455043	13.47832004	14.15863941	13.47832004	13.30795266
7.273507545	7.105472313	7.55945201	6.785769293	7.393167162	7.134964384
7.001869131	6.862375091	7.535530424	6.825325	7.001869131	7.064243152

7.035678848	7.14635899	6.982654747	7.186679828	6.773917293	6.963370131
8.037984616	8.083485404	7.567690545	7.370383778	7.269324172	7.470612081
6.320458162	6.531281859	6.64187404	6.626873192	6.308316374	6.520809899
9.912769303	10.40701089	10.42927797	10.76361324	10.29324401	10.51174846
9.633493677	9.620873889	9.61181703	9.478383576	9.406122899	9.845595727
8.135850465	7.773938747	7.609879768	7.723687384	7.56409603	8.621272737
8.71185199	8.806450121	8.36556403	8.674281929	8.668536687	8.692870869
10.55474917	11.19114259	10.66105788	11.85301976	11.25700815	10.99623862
6.799149535	6.435963586	7.165538758	6.461249374	6.586881556	6.796566808
9.850553616	9.009915859	10.22448985	8.930963202	9.567720788	9.565660949
8.729972586	8.745043505	8.450646323	8.812013889	8.475612051	9.190205293
10.1752482	9.800083455	10.72633412	10.24139873	9.996420616	9.87177298
10.41123643	10.32208546	10.4524716	10.32635032	10.49140257	10.61261659
8.497692788	8.612685364	8.372433475	8.307893798	8.47014499	8.627796121
7.421647414	7.38862201	7.534556657	7.180020091	7.681826051	7.34349297
9.363307667	8.982611727	9.330464051	9.129654758	9.296718121	8.938439909
7.680542242	7.692552364	8.015705778	7.355706667	7.443557333	8.042974687
10.81280665	10.41530743	11.09721747	10.66587479	10.68072859	11.24001011
7.731542758	7.732919727	7.377888556	8.112876232	7.26025397	7.916025434
6.923412444	7.065103374	6.40434199	6.890245747	7.517436172	6.567185838
8.553595747	7.571747222	8.895503556	7.636533838	8.485631939	8.223899788
8.325316889	8.243245818	8.430929525	8.00874802	8.127495596	8.39308204
6.386460131	6.430261071	6.019069505	6.191967758	6.299590657	6.808010384
6.619037364	7.083013909	7.453623293	7.44049799	7.042643192	7.186327495
7.895961929	8.821324343	8.102124455	7.875808798	8.141770949	7.863195747
9.095762	8.953164051	9.404880263	9.236429667	9.332795131	9.315088131
8.523871909	8.152794889	8.774537848	8.551311374	8.563085788	8.685201879
7.087210828	7.246141232	6.872611121	7.352232505	6.989960667	7.145769434
7.650858424	8.092691677	8.176718545	8.440878707	7.955993172	7.862831293
7.21289504	7.286722061	7.18482696	7.318539616	7.056715131	7.369773828
9.538964939	9.613206141	9.380344141	9.578067192	9.463745455	9.603529667
9.606954152	9.799317404	9.761765848	9.260036485	9.11211799	9.384078242
7.711178283	7.861832121	7.821625848	7.828584192	7.532439838	7.937009
7.975414556	8.130993828	8.35752198	8.917069505	8.229734556	8.285708747
12.41931404	12.86955734	12.41931404	11.64244603	13.18348162	13.52334642
6.472536212	6.031725677	6.877148152	6.042406616	6.274191354	6.513435323
7.462172081	8.493653778	7.434501333	8.349393212	7.314616131	7.604109192
12.33273925	12.65104366	12.30465105	13.05428976	12.48124823	12.55625498
9.195011616	8.685201879	9.477181455	8.610337293	9.160413333	8.560860505
7.191807606	6.948047899	7.837132667	7.626470505	7.698638758	6.803094364
8.834473929	8.875711838	8.778623323	8.621689657	8.587623212	9.004398101
6.135884374	6.158302111	7.216692263	7.233650202	6.512183152	6.615899091
13.13007894	13.37822002	13.04381444	13.72760196	13.0147344	13.26276274
9.717365758	9.667708556	9.07581704	9.471411141	9.694213162	9.70574997
8.941795242	8.708030737	9.243096889	8.354182626	9.422377343	8.30620801
12.0089388	11.33100665	12.62449502	11.45526933	12.35798465	11.50814936
10.2829717	9.669847545	10.64623997	9.447242061	10.2861211	10.63266161
8.556357949	8.715845505	8.077230061	8.315227333	8.108111747	8.611710212
11.05356995	10.58469265	10.65871503	10.52689599	10.54565144	10.81664077
8.503061444	9.07419996	8.568768141	9.035586242	8.523871909	8.316081222
7.156966303	6.790809081	7.240898899	7.247912131	6.911201424	7.580266434
8.801904485	9.271123182	8.549141545	9.782868192	9.120933788	8.841037687
9.046362323	8.794788636	9.302103889	9.033915556	9.072403576	8.913588253
8.256499515	8.386133566	8.016429949	8.553595747	8.047188455	7.868335495
6.115970333	6.197177	6.062490404	6.312227091	6.59250899	6.082207485
7.440186081	7.31189602	6.935569505	7.348317141	7.505451222	7.351945465
7.073657889	6.923688879	7.546292778	7.219017232	7.168718192	7.064538818
9.209526556	9.049171303	9.109120374	9.241421747	9.432636424	8.883414061

12.91196246	13.13522491	12.68903729	13.36582475	12.88265743	12.77015535
9.279246253	9.256767414	9.326739394	9.711723394	9.050797061	9.787586646
10.75737971	10.94208941	10.61867663	11.10035391	10.49592664	11.41415047
10.60635433	10.33252085	10.48782122	11.02105469	10.2187154	11.17910752
7.958031758	8.426330949	7.870978364	8.932093424	8.040196323	7.592047838
6.988974929	6.791252535	6.840661414	6.913051818	6.804494667	6.892562737
7.359873626	7.737346818	7.25519196	7.933961929	7.844877657	7.709908667
8.607126586	8.77085696	8.533396525	9.054860525	8.907429495	8.562227051
8.852576737	8.717867636	8.908475283	8.64507902	8.457898556	8.824851909
7.170232747	7.117635758	6.894391313	7.263217283	7.218710525	7.152687384
8.73262002	9.011546121	8.649944576	8.794361798	8.966940586	8.778623323
9.245445293	9.571117879	9.335891293	9.214111545	9.279246253	8.700606657
11.51008986	11.73070023	11.23280633	11.22119175	11.52448673	11.94234713
8.206288727	8.425403333	7.871272152	8.69919198	8.361327455	8.134790222
9.007843273	8.743124222	8.945002717	8.925229707	9.280885222	8.703733141
6.574185192	6.516723778	7.09353204	6.744072859	7.274888343	6.612635086
9.576740677	9.497817424	9.314493212	9.516398909	9.230202616	9.740584727
8.930433505	9.04056096	9.706432808	9.916104475	9.675904202	9.423636879
10.85782556	10.49009312	11.03296099	10.43122177	10.8778129	10.15585749
13.03025597	11.74670945	13.07947925	11.57567047	12.30167305	11.03737422
5.95448197	6.481687929	5.963902626	6.080625747	6.459423596	5.771787424
8.555833323	8.316969364	8.496365737	8.264694495	8.468064667	8.151663141
10.66342196	10.5041569	10.35385551	10.51174846	10.45041513	10.58469265
9.531623293	9.641007131	9.114524374	9.475206131	9.528804131	9.233272283
7.016197848	7.047846657	7.373983485	6.888722505	7.488273475	7.115757364
8.305359949	7.140902202	8.670447949	7.269892283	8.100639	8.505287909
8.941359636	8.401317182	8.945493505	7.833640626	8.963499596	7.767362636
8.593101434	9.250770152	9.009407333	9.485668061	8.693780273	9.07581704
6.62116196	6.326728293	6.931501667	5.992527384	6.143547697	6.639986485
8.950874253	8.372086737	9.212349869	7.982442283	9.180285111	8.944409727
5.761464111	5.810702081	5.95595804	5.813397263	5.756894919	6.033623869
10.2572153	10.12697515	10.32849396	10.10631712	10.01257663	10.1421691
9.179819687	9.263233333	9.179226364	9.360502949	9.300983919	9.051957646
8.311348051	8.485174677	8.592161788	8.515864333	8.622644364	7.453623293
6.680656707	6.582439566	6.227696606	6.530395293	6.512566313	7.004269242
9.001272616	9.061649657	8.713417869	8.989142232	9.124449354	9.501306556
7.609574333	7.836508475	7.553068374	8.129874121	7.820605525	7.682588111
10.62142174	10.49592664	10.35178281	10.56207051	10.57885616	10.70299608
12.14789109	12.32659908	11.48840455	12.24097391	11.82346178	12.05813886
9.007167758	9.003357768	8.761177727	9.47593803	9.202552434	9.084956182
10.65365596	10.67572435	10.61480751	11.27306561	10.573928	10.35069541
8.767368071	8.798818646	8.687053253	9.124970051	9.030619364	8.803935202
10.30470526	9.828404283	10.36589334	9.685649929	10.23948175	10.77387872
12.09804166	12.62449502	12.51766186	12.77841715	12.34237091	12.43600068
7.468171747	7.357775939	7.092080626	7.266525586	7.12483998	7.461239687
7.442908828	8.163923192	7.970574879	7.760294414	8.650965909	7.468792788
8.126253929	7.797507939	8.411285283	7.45523802	8.079973212	8.348543172
6.953914061	6.748847646	7.398020586	6.839911636	7.177177071	7.291703707
8.739178636	8.642670768	8.924680667	9.011546121	8.745901071	8.85308204
9.631182697	9.377330121	9.362238061	9.245932475	9.348735909	9.572453101
14.16629104	13.3718573	14.1830221	13.23864174	13.79381649	12.89886333
13.95167504	14.36070414	14.08841453	14.56207529	14.38889659	14.5413574
10.03876748	10.45877561	9.694213162	10.4700758	10.02549055	9.935661677
8.248137606	7.828991111	8.64507902	7.941320778	8.193428737	8.070136677
8.00874802	7.904381697	8.17298499	7.888856606	7.847379061	8.348974465
6.645437646	6.352052949	7.262118616	6.381423636	6.672562737	6.808256808
11.2345748	11.33657706	11.11856721	12.03068847	11.65918316	11.6157426
8.019769071	7.88825004	7.356007838	7.805446323	7.728706424	8.07053003

7.783110646	7.686775222	7.681167667	7.837132667	7.481645051	7.744459273
9.377908	8.970028374	8.431738505	8.564847253	8.607126586	8.889380424
10.0728998	10.37686258	10.08325108	10.10946026	10.22761479	10.22865659
8.57070398	7.645794677	8.548304869	7.836508475	8.293340606	7.147201253
9.092457495	8.609511	9.600185283	8.352537101	9.293068828	9.283145788
9.344897333	9.328711051	9.287277576	9.653591273	9.184000242	9.139724848
8.382609505	8.347717242	8.439112919	8.808345061	7.990313949	8.59026096
11.18941159	11.45932673	11.05033578	11.24323027	11.85889751	10.68072859
8.94874	8.975268424	9.307024131	8.725555596	9.407554697	8.899254879
8.461795505	8.217984283	8.627796121	8.346989566	8.266388465	8.137479222
8.301657889	8.157542717	7.842052606	7.687096788	8.173308798	8.096910323
8.169382444	8.397394545	8.470647758	8.881793333	9.006527424	8.77962597
8.825911939	8.631321253	9.005975162	8.611286687	8.818210192	8.921533879
7.529686949	7.573788949	8.094605657	8.13932801	8.378142071	8.265130232
9.068490586	8.781156657	8.789132293	8.967895455	8.943348051	8.884521697
7.222702545	7.304711495	7.761378475	7.87816304	7.948048111	7.962680434
7.258107788	7.215185141	6.907093253	7.359287172	7.020439899	7.03650198
5.774595131	5.966369889	5.777431414	5.769964838	5.930349131	5.776867566
6.856233343	7.130177747	6.533739475	6.878162091	7.054238101	7.063754707
8.649944576	9.200042253	8.949261586	9.491725303	9.459003	9.36570098
10.95234717	10.79284767	10.93023415	10.7814083	11.28731295	10.82945836
7.563439404	7.752687697	7.649291899	7.696259101	7.695912303	7.962004212
10.66945215	10.11150219	10.27338352	10.27139209	9.924448444	10.76609029
13.00101781	13.26910509	13.09977851	13.48401144	12.92081703	13.27418186
12.26775966	13.16824615	12.57486869	12.70002263	13.25686564	10.48074239
7.708922879	7.898595626	8.016751283	8.447076788	8.545091384	7.537240869
8.528049141	8.387507626	8.701614283	8.200163707	8.675789182	8.729972586
6.652872152	6.825870455	7.749299101	6.895207929	6.575018	6.637667434
7.277222202	6.875236313	7.52469401	7.638274606	7.405396414	6.914534515
6.408811828	6.343678303	6.893115293	6.600294051	6.791252535	6.450820343
7.571747222	7.920494253	6.930222384	7.529949465	7.479706475	7.545042545
9.941224515	10.26332497	10.28089537	10.30569114	10.21157382	9.79615097
7.474397404	7.243253313	7.188612899	8.382194192	7.377037515	6.935285707
9.316319051	8.947623838	9.62845904	8.786134101	9.521192909	9.530933293
6.86140099	6.504049202	7.33350399	6.885618131	6.943317879	7.067324051
8.217984283	7.930615646	8.587623212	8.298396535	7.891912283	8.776574232
7.600615697	7.767362636	9.125507586	9.310205828	8.786134101	7.714004283
7.943433747	7.707585606	7.650531152	7.407119727	8.006153172	6.69201996
6.504270879	6.72130502	6.610225071	6.718256293	6.999830061	6.483340232
15.18392271	14.94534759	15.15443825	14.5108222	15.10875812	15.58150095
7.907197636	8.088528121	8.124599212	8.537544354	8.119679141	8.181963182
6.459603455	6.369108576	6.518976111	6.332645192	6.605935465	6.477093667
8.298896434	8.240781051	8.341977727	8.45451298	8.282438737	8.57790302
10.42927797	10.64005918	10.52247938	11.28370047	10.54692936	10.39296715
8.028563859	7.28903701	8.293340606	7.354804152	7.949400121	8.090488788
7.765298909	7.510027525	8.08112003	7.361082222	7.686071172	8.263464364
8.731576192	8.887109626	8.742635939	8.582586091	8.41382397	8.544633697
8.551311374	8.739178636	9.047491869	8.83754499	8.912003131	8.466852202
8.098747556	7.622739535	8.236633323	7.688677182	8.44129198	7.825440475
7.506719576	7.21064097	7.701352778	7.065949717	7.692888131	7.307847101
9.569854202	10.41330878	9.130293909	10.81034167	9.831834717	10.08799683
6.851375081	6.798121798	7.619745136	6.651718455	7.317304515	7.382821899
13.88591407	12.86086109	13.93694444	12.84627851	13.27418186	12.29548604
9.165150051	8.540202646	8.784649081	9.003925646	8.783584889	9.310775172
8.295839707	8.490053091	8.104500051	8.030181424	7.84876403	8.113745737
14.15863941	13.5360606	14.00297944	12.28105924	14.16629104	13.99495772
8.349393212	8.344098929	8.026630869	8.056301667	8.032161838	8.573727141
10.67081044	9.965319232	11.18096106	9.757590192	10.66229262	11.10035391

8.799825313	9.435722162	8.681067818	9.533596364	8.632349838	8.14677703
8.283330131	8.334981101	7.909031556	8.961854929	7.778752535	8.324880909
7.484854374	8.359650919	7.688370495	7.798177384	8.450646323	7.967524909
8.32856404	8.331458747	8.363019899	8.05903404	8.448929687	8.007258162
10.65634987	10.66342196	10.73661507	10.62142174	10.56207051	10.76239578
7.772177253	7.601973414	7.93027604	7.753046657	7.708922879	7.752687697
8.967895455	9.09013302	9.274850788	9.69725304	9.161459798	8.836023374
10.04862183	9.923658606	9.772980061	10.09896607	9.909202788	10.56802411
9.809529798	10.03701736	9.595333525	10.35385551	9.923012889	9.606298465
8.457489293	7.295980475	9.267923343	7.426469556	8.302900242	8.959130566
9.067241768	9.641787333	9.336479586	9.211184727	9.890949131	9.680349586
13.05987966	13.23864174	13.22156044	13.7211242	13.04381444	13.14093405
9.323731354	9.290785283	9.070661273	8.655437626	8.734416606	9.177504333
8.673843717	9.006527424	8.316570081	9.212349869	8.358783152	8.818695101
7.897743687	7.974325596	8.113343889	8.125376242	8.143776848	7.719108384
11.21742202	11.68484165	11.22456459	11.89130603	11.24816417	11.37490296
11.48258282	12.04432	11.98440811	12.89113428	11.80554878	11.54053762
7.976470212	8.471488636	8.160266646	9.292435667	8.038729465	8.156085586
6.98587896	7.332669495	7.259665485	7.806845253	7.193007566	7.452109758
8.30620801	8.655934636	8.448456354	9.169862626	8.701109788	8.401317182
8.276438626	9.207791152	8.260123323	9.363307667	8.826393101	8.761177727
8.290726727	8.082778869	8.400903717	7.956615091	8.591239465	7.790604293
10.32635032	10.4450719	10.12799521	10.4302696	10.48432644	10.28899471
8.911610283	8.332313525	9.104371838	8.387083515	8.635209293	9.415860727
7.047046253	6.673543687	7.529949465	6.777724495	7.369510384	7.394370293
9.649399939	9.946747141	9.569854202	9.961780586	9.512482222	9.913634949
6.561721071	6.579391333	6.857241172	6.617114677	7.079375364	6.386235697
9.858738737	9.310205828	9.618256808	9.535633242	9.459664354	9.469429253
8.432150758	7.766738848	8.886096818	7.849821828	8.45451298	8.112876232
9.218466939	9.188424626	8.992473788	9.329342374	9.479751879	9.294355394
7.562285172	8.034450929	7.390471152	8.26142704	7.874403566	7.667649192
9.187244566	9.374121768	9.099242404	9.354902374	9.426842081	9.153232364
7.771540485	7.989204111	7.446413172	7.917722121	7.944058707	7.918105152
8.881793333	9.464440242	8.900779434	9.048100778	9.359207242	9.046362323
7.028571808	7.363288879	8.78563097	8.561321323	8.810446485	7.335553323
6.903241343	6.891253768	7.042119313	7.307022919	7.057011424	6.786766677
10.1250404	10.37573078	9.811091061	9.352487919	9.949411172	9.806332162
10.4283734	9.223302313	10.41224321	8.971727333	9.681893121	8.408296424
9.046862848	9.549314313	9.08959502	9.37540303	8.85308204	9.057196687
6.680176879	6.669677444	6.244028818	6.579844919	6.550797374	6.419103354
8.774537848	9.511857455	8.373514202	8.524867051	9.504584061	9.273640758
10.09795649	10.1985269	10.2572153	10.15283227	9.851320949	10.32208546
6.27159296	6.108107747	6.112680475	6.254001758	6.261965667	6.56193898
8.010919919	7.952029848	7.666604434	7.662615848	7.863889121	7.944058707
9.620873889	9.890949131	9.564343333	9.876096838	9.648698737	9.918671212
8.731005677	8.897177899	9.224923687	9.109120374	8.771294657	8.475210818
9.613873859	8.781680606	10.25519474	8.665165081	9.355403384	10.19136651
12.28693608	12.04990566	12.29548604	11.89628941	11.58621592	12.59668127
9.604175636	9.47593803	9.491725303	9.456392222	9.584100455	10.01974654
9.865550636	9.918671212	9.895061889	9.692793657	9.793126455	9.844817354
7.621022111	7.485478828	8.237787788	7.595301182	8.239938859	7.791244131
11.37490296	11.67849095	11.2518391	11.96636849	11.68484165	11.20660223
11.74205097	11.75662781	12.24097391	11.70557621	11.81221198	10.96460614
8.061773232	8.287888051	8.032161838	8.458294828	8.053619525	8.133993636
9.001802606	9.013830768	9.283736939	8.981479273	9.204345657	8.836542848
6.951169364	6.760600303	7.161605929	6.391634697	7.044969556	7.400588576
5.990004354	6.182077616	6.274896838	6.055022081	6.137175152	6.130885768
15.76560628	15.46469304	15.72204116	14.6970922	15.74256723	15.56435861

9.333361616	7.02555202	7.713612677	7.096340343	8.200163707	6.935824606
9.031155364	6.258944949	6.847044404	6.682294404	7.725703576	6.153328596
7.821971636	7.622037172	7.939609869	7.839249838	8.017912586	7.78118699
8.633351606	8.835019636	8.531655515	8.728878424	8.71185199	8.583542788
10.3025572	10.04862183	10.49466708	9.699589758	10.24612484	9.93099102
11.55322016	11.80342824	11.45002291	12.28693608	11.80104424	12.03912361
8.135526051	8.345781111	7.905781333	8.335469222	7.847720859	8.134366646
7.286424495	7.551170919	7.315791616	7.879985051	7.524403394	7.525550222
14.77222727	14.06476988	14.72870348	13.46005067	14.56207529	13.75520609
6.615899091	6.705893697	6.42554104	6.599616071	6.45826596	6.823157384
9.197870838	9.521192909	9.445852485	9.588958727	10.11941657	9.376667182
11.84529477	12.08128174	11.19114259	11.49239347	12.01191659	11.71244686
7.459115707	7.002890535	8.079973212	6.688683899	7.566450929	7.122138172
5.96355196	6.241411051	6.140075394	5.954837061	6.734222485	5.85341901
8.589797808	8.325717687	8.147481596	8.246913576	8.239069586	8.549141545
9.574750414	9.230202616	9.856174394	8.939019091	9.288419414	9.35067002
8.467263475	8.625920364	8.606099333	8.799348343	8.397394545	8.537956091
10.13768673	9.759214424	10.58579258	9.151431899	10.38001468	10.20358283
10.49009312	10.49140257	9.980879424	10.14713571	10.33252085	10.59685822
7.988251818	7.180559343	7.233950838	8.718321818	8.004282899	8.251492909
10.18291229	10.60161194	10.28899471	11.04156144	10.51400776	10.40701089
10.55213977	10.90754603	10.65987528	10.8193093	10.65987528	10.52135963
8.102124455	7.197446212	7.341339747	6.863389636	7.444173596	6.186899475
6.584387121	6.455374485	6.916922333	6.415279879	6.289741071	6.590932293
8.879330566	9.308265131	8.823889374	9.372705202	9.349388778	9.149684939
9.443424081	9.332175414	8.961327374	9.131560131	9.346206162	9.517771202
8.789683394	8.548679899	9.466909636	8.547862646	9.556759333	8.355847778
9.660861778	9.951175646	9.370225242	10.00450786	9.814415596	9.997192202
8.605125889	8.736738475	8.677707162	8.708030737	8.613521394	9.007167758
10.33989796	10.6313464	10.20634509	10.88945836	10.40592026	10.61371787
10.25519474	10.52689599	10.52364746	10.31480319	9.898158111	9.707282667
9.623794152	9.255548899	10.04511943	9.007167758	9.690534253	9.333986848
8.796713535	8.848914293	9.019731374	9.801802283	9.042346232	9.035586242
7.149903152	7.074397545	7.432695485	6.661555384	7.078835444	7.718373848
9.50650501	9.541041677	9.750460616	9.675904202	9.770750222	9.831087364
8.892401576	8.346989566	9.332795131	8.508645727	9.146732444	8.107270071
6.723255293	6.60386102	7.057634667	6.633233677	6.637264	7.194433949
7.783847071	6.892562737	8.521230828	7.357173859	8.309668384	7.68569196
12.09497108	11.91912022	12.15752365	11.91651973	11.72592113	12.03602449
15.48449671	15.52328152	15.61926687	15.65613201	15.56435861	15.99508933
6.725499141	6.222974525	6.87215099	6.535959323	6.667348717	6.187353162
9.762542495	10.10631712	9.715870253	9.939327313	10.01974654	9.948421707
13.44126502	13.75520609	13.5964752	14.17411554	13.45466248	13.73439002
8.586731747	9.050797061	9.039989364	9.302103889	9.023071899	7.815915242
12.84627851	13.58996905	12.76207554	13.68884286	12.79024082	13.08418191
6.685084394	7.80651396	6.789025475	7.705454838	8.305359949	7.874063444
9.455711606	9.567720788	9.277455131	9.969958172	9.310775172	9.095162899
9.348735909	9.088505869	9.200042253	9.294355394	8.532516465	8.881793333
13.26276274	13.09045797	12.7452022	13.02518615	13.13007894	13.81935116
6.171621859	6.04944802	6.303290121	6.931948737	6.027652303	6.517203657
10.89755671	11.3733012	11.63837311	11.62290433	11.22456459	10.8443678
11.22947614	11.51647662	11.1502402	11.41238356	11.2786408	11.47682743
7.934739636	8.020096283	8.39308204	7.976470212	7.495760485	8.002342131
10.87518762	10.62895885	11.07158908	11.54871614	10.35809493	10.4429644
10.15585749	10.0672481	10.28710845	10.82290783	9.673359929	10.70534521
7.135537212	7.794365768	7.502072596	7.36076402	7.553468394	6.812925505
7.336145273	7.648319737	7.202115061	7.367244707	7.536554758	7.707585606
9.916104475	9.773700283	10.32531651	9.827596687	9.921211657	9.449254061

7.389236737	7.68740998	8.229734556	8.174468182	7.853698798	8.000521869
9.204855576	9.233272283	8.724556485	7.988882495	8.859715222	7.977952253
9.249034202	9.610538374	8.877626323	9.420326576	8.948168879	9.217871889
6.712342556	7.039183869	7.721939444	6.919083737	7.336476646	7.04319302
6.997357515	6.218725646	7.420173404	6.356285141	6.745632475	7.394691081
6.108920869	6.284543212	6.329319202	6.285113455	6.077855707	6.219828162
9.272374697	9.417037424	8.707057788	9.344897333	8.699656879	9.197870838
9.318228697	8.803482152	10.10093339	8.798818646	9.681142253	8.754441596
9.342353505	9.225515485	9.565032515	9.013830768	9.609756293	8.777050384
7.050073121	7.152687384	7.028039657	7.587331949	7.318843495	7.56574702
6.632973788	6.681596343	7.060512141	6.841133828	6.475742909	6.828875111
9.237064687	8.930433505	8.909404788	8.460053242	9.122783717	9.124449354
13.22156044	12.84197228	12.71520416	12.97555423	12.58287167	13.15645152
8.509936919	8.731005677	8.423588242	8.970028374	8.800397414	8.694840596
9.900778232	9.731150899	9.714566535	9.724047525	9.606298465	10.13193078
8.14677703	8.498167798	8.10326497	8.903602263	8.263041909	8.411696677
7.394027687	7.527074182	7.173915838	7.522209596	7.225864141	7.094132182
8.106418192	7.725042051	8.377090384	7.394370293	8.086566798	7.720197707
6.968156515	7.020691646	6.980492495	7.155827141	7.11489098	7.093254121
8.098302202	8.059423535	8.124973859	7.994566778	8.158602879	8.395296687
12.07810634	12.25572441	11.53677901	12.46797904	11.92396137	12.16627503
7.292849111	6.856475414	7.232737253	7.13936198	7.021482505	6.785085162
7.491357222	7.924172566	7.229046354	7.937009	7.441392919	7.635576919
8.037287293	8.291166909	8.588536172	8.218433131	8.752912545	7.963030121
7.646666444	7.689572788	8.075284384	7.850890354	7.88825004	8.158321101
7.801818071	7.756512818	7.974325596	7.717737636	7.656877838	7.370950798
7.063477202	6.39883498	6.776493808	6.899006192	6.798121798	6.788076343
17.43906183	17.23317693	17.43906183	17.29704085	17.29704085	17.1822602
8.127056495	9.288999586	9.204345657	9.890135131	9.086722374	8.44446797
15.21350605	15.08134613	14.99939481	15.15443825	15.08134613	15.19830883
7.474688838	7.660499434	7.352900667	7.836138323	7.246141232	7.629989283
12.24097391	11.94234713	12.47761486	11.89876792	12.19996238	11.80342824
10.21157382	10.60044575	9.821195081	9.965319232	10.72633412	10.47123164
8.490053091	7.723009	8.78767504	7.91707401	8.300103657	7.876138384
11.07337622	11.00496707	10.80091358	10.79145221	10.94208941	10.64379219
7.388945192	7.262654505	7.234805667	7.078018687	6.943812717	7.339805162
7.629016404	7.805068949	8.015388182	7.855731	7.815915242	7.569572879
9.404333879	9.662314131	9.19736601	9.794631646	9.679062616	9.550769788
7.947667091	8.164575929	7.582966818	7.80651396	7.95565902	7.760648222
7.789255535	7.797125465	8.148308414	8.373919657	8.179951646	7.837840162
10.2692332	10.57282784	10.01071956	10.32093584	10.24316974	10.14895288
8.676791091	8.463578343	8.032957859	8.187968455	8.228066545	8.480536717
13.62784329	13.61616406	13.23864174	13.29117716	13.76702458	13.91489381
9.960976253	10.44934262	10.29432836	11.88649013	10.59418919	10.27804811
7.543741273	7.584965596	8.006495333	7.441392919	8.07651197	7.000309313
10.14044783	10.71031814	9.900778232	10.81540317	10.01622438	9.955574404
8.704708384	8.721054424	9.291393586	9.142791657	8.921533879	8.451550535
7.619556354	7.168718192	7.70365599	7.154661646	7.919201576	7.624016909
7.297158313	7.18482696	6.703943808	6.983707354	6.985632404	7.304975899
9.023071899	8.295839707	9.331636121	8.554528737	9.254890202	8.493155899
9.796940758	10.34418014	10.21975253	9.833438364	9.834210131	9.465659495
9.085558838	9.245445293	8.932594273	9.701165495	9.333361616	9.230202616
7.799196616	8.267698071	8.115821848	8.282073859	7.978709162	8.047933768
9.178084667	9.129654758	9.170513566	9.763952101	9.11154599	8.996667384
8.727528626	8.616914869	8.790130253	8.912452162	8.708995576	8.985325394
10.94786936	10.67428006	10.60383217	10.34311059	10.87379507	10.34717129
8.029836697	8.235388636	8.69723302	8.233838889	8.284876758	8.436545051
8.03490996	7.688949081	8.044980354	7.485802727	7.416977434	7.174677505

11.12001247	9.752732475	11.70557621	10.25811169	11.22119175	10.46232362
8.268510495	7.985458788	7.673764162	8.002342131	7.525850576	7.895278253
6.664178717	6.484023485	6.991849242	6.519436343	6.889990505	6.953914061
8.469339576	8.365106485	7.805761556	8.281588828	8.31008898	8.296772869
8.020096283	8.200163707	8.275152535	8.187593091	7.651593505	7.947667091
8.927019859	7.740469253	9.448606737	7.540114586	8.598479657	9.405539273
7.479422939	7.293451808	7.284692949	7.519990596	7.179751081	7.271131505
8.08537204	7.979449616	7.737987747	7.871272152	8.07563398	7.732550404
9.937540263	10.07857135	9.305189859	9.618955576	9.847335909	9.971733293
8.101780545	8.045324071	8.532516465	8.110083838	8.220625586	8.232591606
6.984018879	7.348618394	7.166060222	6.979123747	7.706911222	7.236271343
9.779738	9.989520798	9.779738	10.85782556	10.15483313	9.911926424
8.77962597	9.025691242	9.072998758	9.232683343	8.900236364	9.006527424
12.61761735	13.31405593	12.70002263	13.3718573	12.80602179	12.80602179
9.349388778	9.166446616	9.185497838	9.118629636	9.458377051	9.298556212
6.399871131	6.31049204	6.565175343	6.354167	6.714736646	6.751979343
6.884322333	6.659312505	6.374790061	6.362324414	6.259323444	6.759458
8.567801737	8.751405818	8.474658303	9.186096828	8.666601818	8.533396525
6.814975071	6.832125364	6.971045939	6.584604515	6.97366599	6.928862212
12.66167346	12.0750029	12.8293171	11.62494396	12.54589185	12.38063147
7.091827414	7.28082202	6.771643182	6.690165707	7.496345141	6.653830545
7.670275758	7.778362485	7.163563677	7.833986465	7.050890818	7.13133802
7.10439698	6.831893455	6.724801141	7.795765162	7.182546172	7.122697263
8.44047504	8.483763566	8.049019253	8.392711081	8.422204465	8.072110051
7.364797848	7.592047838	7.481645051	7.136356818	7.421318101	6.972058485
7.521572545	7.444173596	8.201303758	7.797507939	7.758961677	7.426803586
11.69658017	10.78408516	12.05489479	10.82034332	11.73710477	11.14707085
10.5752557	10.83642757	11.04156144	11.26990549	10.96162926	10.97322727
8.994589434	8.564847253	8.85785699	8.135177152	8.69919198	8.720627081
8.316969364	8.292509485	7.802836333	8.040196323	8.246913576	8.293340606
6.821773354	6.805713475	6.394931444	5.846630354	6.569103444	6.463509232
8.309668384	8.524356212	8.920488949	8.372086737	9.171801828	8.285312424
9.418269535	9.237064687	10.042425	8.756396081	9.937540263	9.116279576
7.44169298	7.328411747	7.461834424	7.338325414	7.517077263	7.388278253
8.167813576	8.410410394	8.162329636	8.068200283	7.992662242	8.162707626
7.474938939	7.221869232	7.823429646	6.560645212	7.520648323	7.991026727
9.767642525	8.647073404	9.857940545	8.57790302	9.857940545	9.066605263
9.013314737	9.388273121	8.238629646	8.736738475	8.729485838	8.703161646
7.815211869	7.981345788	7.738299929	7.52469401	7.999737535	7.575132364
6.603596232	6.58104199	6.37274698	6.436610747	6.400112778	6.662035182
9.376046232	8.795325869	9.492465394	7.869992939	9.478383576	9.994772242
9.508442768	9.209526556	9.552821717	9.333986848	9.719830172	9.433258586
6.086870354	7.117905152	6.340852071	6.467159949	7.838173152	6.415480919
11.46724679	11.78023213	11.54053762	12.48458958	11.71895032	11.90674449
7.392563535	6.604981172	7.969427899	6.377125556	7.114645596	7.263859848
7.295094879	7.244714616	7.571427586	6.982384606	6.991264788	7.562928222
9.742174424	10.09682592	9.907546465	10.58579258	9.860587788	9.847335909
10.10093339	10.24227579	11.04886125	10.15379546	10.48897537	10.01340346
11.93486375	12.30789527	12.22629088	12.51766186	12.21991364	11.77053658
10.53505365	10.71992703	10.80644997	9.733230051	10.77890896	9.953782444
8.890936242	9.169326323	9.166446616	8.662363172	9.103235919	7.995673758
11.27306561	10.58689262	12.60396528	8.024633293	10.9991534	10.59292728
7.925642	7.882525919	8.400464071	7.597232061	7.940254566	7.690840778
9.632768747	9.348735909	10.1985269	9.021393263	9.84311402	9.671912455
8.916479111	8.422643323	8.613985131	8.398231626	8.205133263	8.647073404
8.899254879	8.754441596	8.645541747	8.505797091	8.683580283	8.924680667
11.65052704	11.47127063	11.38970236	10.82416773	11.23641801	11.43938222
7.562928222	7.403718889	7.38400901	7.841440677	7.670902747	7.502411182

8.540202646	8.775519707	8.187593091	8.627322343	8.676791091	8.693780273
10.23160947	10.22951336	10.32750324	10.22070473	9.766921414	10.10093339
11.84027237	12.22341713	12.00086001	12.21630458	11.9091429	11.84291489
8.787164939	8.724013404	8.861258687	8.591651626	8.534897646	8.783584889
10.8193093	10.83363242	10.74558579	10.65634987	10.95234717	11.14385742
7.761378475	8.236633323	7.537817576	7.97830402	7.845242687	7.570765313
9.724814657	9.777520899	9.413321525	10.16937171	9.590351455	9.895061889
10.89912578	11.04464523	10.90466263	11.31449746	10.98492733	11.16888788
8.213832515	8.673363869	8.692870869	8.988577162	8.216297535	8.254484434
8.721054424	9.442729677	8.695335444	9.387656788	9.811091061	9.588410576
15.38120792	15.90458538	15.52328152	16.42659037	15.54332231	15.88528111
6.584154061	7.026870727	6.326100737	7.071486061	6.640921556	6.653596384
9.382882596	9.329342374	9.509876273	9.625623061	9.087255717	9.290785283
6.199844141	6.797589061	6.25331604	6.398234566	6.602227	6.536641737
15.19830883	15.70163214	15.31193978	16.04217659	15.29549959	15.39581235
9.819688798	10.080472	10.30470526	10.84705809	10.5397012	10.85654613
8.213427111	7.721939444	8.443175586	8.240781051	8.397853727	8.379884889
7.735579636	7.91465098	8.173633596	8.157153081	7.980934222	7.667309091
8.478760515	8.539776737	7.937009	8.327767232	8.386133566	8.477856384
7.775269606	8.056301667	7.516753121	7.77977198	7.714004283	7.869992939
8.733877859	8.762742172	9.077686808	8.473392556	8.554528737	8.537544354
10.05523038	9.839882283	10.0841781	9.319510202	9.813557354	9.898158111
7.929905606	7.759936788	7.20666299	7.411268949	7.428304182	7.664565879
17.07010739	16.39933956	16.61084374	16.33688632	17.07010739	16.82803465
5.849527242	6.211158404	5.975842192	6.115154242	5.985516596	5.950809091
7.998937818	8.395713444	8.118136869	8.369167818	8.509936919	8.019434586
9.359783616	9.408173051	9.135618929	9.53690398	9.168324747	8.809951364
11.6295292	11.73271713	11.82852227	11.81221198	11.94979793	11.60680648
6.998704808	6.877410515	6.777950687	6.975426091	6.693719727	6.57397498
11.01045685	10.30058205	11.04030995	10.26541026	10.98186414	10.95234717
8.695776131	8.606624646	8.768300525	8.583048444	9.022478232	8.586731747
11.33848921	10.97054498	11.73972102	11.43565115	11.41238356	11.6295292
6.698319919	7.061330929	6.656675747	7.495088838	6.637907929	6.706660646
8.902484929	9.094691273	9.677492576	8.797743222	8.954798808	9.038261515
8.430929525	8.424471616	8.678158051	8.687541707	8.466852202	8.838020616
8.10326497	8.127056495	8.423139949	8.218887434	8.177528808	8.418118394
9.199527535	8.641223283	9.684048687	8.552719747	9.643287657	8.941359636
12.90783816	12.54268587	12.67700968	11.51008986	13.00101781	13.06488572
7.883157929	7.272016424	8.320817869	7.346859747	7.975072535	8.430929525
8.26142704	8.08737399	8.848410616	8.074508444	8.851018515	8.104500051
10.24038579	10.4283734	10.50677257	10.79956984	10.10631712	10.38631174
9.724047525	9.712341424	9.534194535	9.448606737	9.822898081	9.599465606
7.085629475	6.994029394	6.677606	7.178905394	6.711872081	7.219017232
7.156711091	7.481645051	7.313451111	7.785940475	7.373717778	7.293451808
8.350987111	8.547449101	7.994743247	8.892401576	8.547862646	8.215082485
13.48990673	13.16151113	14.09521817	13.34730616	13.88591407	13.47327752
8.120062485	8.326518101	7.946541949	8.063008152	8.328992859	7.993077394
6.342690566	6.301707192	6.177536939	6.387054293	6.189914495	6.309659747
10.91176188	10.16355358	11.23641801	9.66613299	10.93494087	11.57765358
15.59877501	15.34617167	15.32551587	14.68633385	15.38120792	14.99939481
9.853774707	9.385837242	9.549314313	9.380344141	9.53973704	9.724814657
9.441234879	8.891390919	10.0719806	8.372772768	9.591133828	9.838413929
7.861535657	7.195502081	8.13267903	7.364797848	7.304125475	7.31523196
7.893273434	7.514105485	8.198599495	7.622739535	7.998937818	7.986161141
7.197080263	7.206091434	6.832629131	7.527339687	7.094997242	7.158304313
9.379720636	9.21352099	9.319510202	9.277992232	9.283736939	9.445852485
8.12949599	7.900388485	7.680240364	7.695912303	7.833640626	7.886079293
8.222223545	8.242002535	8.339115859	8.261781515	8.005371424	8.271029889

8.926362727	8.729972586	9.286739242	8.608532485	8.824851909	8.880364303
9.757590192	9.670530667	9.338458455	9.605576293	9.565032515	9.733950273
7.776293606	7.746135697	8.036816444	8.19458801	7.548584576	8.045722828
8.205457293	8.263464364	8.864430475	8.317824071	8.401710747	8.292509485
9.46096901	9.576136707	9.288999586	9.674270303	9.457741556	9.831834717
9.751885293	9.240719434	10.49713487	8.830660505	9.736957071	10.12408141
8.750916455	8.62412801	8.674281929	8.697482652	8.512201869	9.438094949
6.158655535	6.038947424	6.122087354	6.079319192	7.195783737	5.909195384
7.356281616	7.568924192	8.272620253	8.567801737	8.13932801	7.41345197
6.517203657	6.427081586	6.644710828	6.713538929	6.508699242	6.618269616
11.65698449	12.20960187	11.70348036	12.54906863	12.02240324	12.06692054
6.993183869	7.271131505	6.980238687	7.294284717	7.012316737	7.317304515
7.638274606	7.744142424	7.688011354	7.647964475	7.713270545	7.743136949
9.478383576	8.683580283	9.710054737	8.194244232	9.568406747	9.483552818
10.95374716	10.61133106	10.88166557	10.52904685	10.74819911	10.1300726
9.66613299	9.632768747	9.816676162	9.759214424	9.509876273	9.742174424
11.49636188	11.52448673	11.18800276	11.36610866	11.36245739	11.32027501
11.96362869	12.01427079	12.04734694	11.92937262	12.17829278	12.03068847
7.667962596	7.464044657	7.478449101	7.204042263	7.250899535	7.586712596
10.21279484	10.34311059	9.739859192	10.12610261	10.30695028	10.24414187
10.23752804	10.27432009	9.945028121	10.07764109	10.31289083	10.20452777
8.482406475	8.048636061	8.65914896	7.523479626	8.878201818	8.191573747
6.869142343	7.137721343	8.096910323	7.002890535	7.027167101	6.862647808
10.53028115	10.21769501	10.33696433	10.01168912	10.07580535	10.33476777
8.686679798	8.524867051	8.769824364	9.009915859	8.588971687	8.508189222
7.018881949	7.55945201	7.287262414	6.698743475	7.462172081	6.990260788
10.49466708	9.742174424	9.478383576	9.415860727	10.55333109	8.704708384
8.218433131	8.062539303	7.872718384	8.142577798	8.035675313	8.290726727
9.976273051	9.975431768	9.606298465	9.721958051	9.820433545	9.974493182
9.451851384	9.521972354	9.195534384	9.802496737	9.395854121	9.693515768
6.227863606	8.751946515	6.096119394	6.320458162	6.155698343	6.526955566
16.17943014	16.28343863	16.25437967	16.14978837	16.22759729	15.92931651
9.11154599	9.370225242	8.647922808	9.226128374	8.944409727	9.030004576
8.762202232	8.572163455	9.04056096	8.558108202	9.080123545	8.844262616
8.751946515	8.133993636	8.975268424	8.344860929	8.584830636	8.017104828
8.871686232	9.029402949	8.715328101	8.661956242	8.516311424	9.013830768
12.57486869	12.63888703	12.33576886	12.51066587	12.37416009	12.69316533
7.542783869	7.467207283	7.76948301	7.377269152	7.590558081	7.439228222
8.791657869	8.528512253	8.953653768	8.249350414	8.692870869	9.093001495
12.24351077	12.53563192	11.91416468	12.8293171	11.43773109	13.31405593
11.19638858	11.21816154	11.21016953	11.39185194	11.13879804	10.94071069
9.917043434	10.09426568	10.5431016	10.62647628	10.43757507	10.51650912
5.875020768	6.430701515	6.06149796	5.888088566	6.353208646	5.938785545
10.69354222	10.7532413	10.39414857	10.56802411	10.6820228	10.83363242
9.438799404	9.529469889	9.35728196	9.521972354	9.502644929	9.02886299
7.019151556	6.988399394	7.041096091	7.238770545	6.916922333	7.531511131
7.021482505	6.736375424	7.591410606	6.737098263	7.366006364	7.518993111
13.73439002	13.96779242	13.56234785	14.07245549	13.5964752	14.31917195
7.327857495	6.277904687	6.983407384	6.681164444	6.648393616	6.461008283
6.766293242	7.003981192	6.08048902	6.393414081	6.703725465	6.828622869
14.20688311	14.77222727	14.11151028	15.22815255	14.6970922	14.223913
7.66151798	7.379463242	7.563439404	8.260520646	7.843855828	6.936850606
7.488894061	6.920918808	8.078774939	6.579612929	7.240898899	7.504860717
8.107705111	8.170129495	8.437457535	7.740855141	8.48093102	8.167813576
8.298004222	8.227270172	8.282900293	7.949400121	7.868335495	8.023866626
9.547339828	9.317615606	9.520598525	9.405539273	9.077100232	9.435122859
9.515743253	9.803356646	9.778131475	9.806332162	10.25220538	10.06997663
10.81142627	10.83930015	10.28506122	10.86305205	10.76471511	10.53247367

6.833605444	6.938149939	6.994245051	7.404985061	7.055909434	6.780688182
8.492322828	8.809442889	8.352944343	8.589797808	8.282073859	8.029383646
7.621699758	7.645794677	8.293769586	7.57318396	7.301506111	7.496684051
6.614995778	6.826151202	6.514559495	7.841729333	6.353796111	6.532834
10.40592026	9.995698596	10.89912578	9.777520899	10.36239309	10.1752482
7.696527091	7.373116051	7.912742707	7.342557798	7.741791646	7.747885899
8.167383949	8.047933768	8.998420707	8.051625212	8.775001677	8.373919657
12.41634374	12.48124823	13.27418186	12.82153585	13.39037726	13.14611959
7.734550869	7.961295828	8.028141747	7.08160301	8.195856758	6.778470374
10.03701736	9.49850403	10.06356315	9.276789111	9.155079848	9.769223525
8.632799758	8.844262616	9.044046343	9.095762	8.787164939	8.804477505
9.391460545	9.090721626	10.5152666	9.994772242	9.925979414	10.28089537
6.274531253	6.420979687	5.76208101	5.901784697	6.187521313	5.766484141
10.76239578	10.87235819	10.97185531	10.90466263	10.62536143	10.87379507
7.286993737	7.378842657	7.678591667	7.158304313	7.695611414	7.539809273
7.829693939	7.662925475	8.051625212	7.670275758	7.771143222	7.792215626
6.50518599	6.274531253	6.927522051	6.245450939	6.385847152	6.479009949
7.144029273	7.537489808	7.260895414	8.005727263	7.33182303	7.299714313
9.847335909	9.681142253	10.52135963	10.02741959	10.20060626	9.980047091
9.203189636	8.908935182	8.744081071	9.10215096	8.84935699	8.977558859
12.01191659	11.26815786	12.17513939	10.65500785	12.04990566	11.58421758
8.080354404	7.956615091	7.560118414	7.909412687	7.714642404	7.808676939
10.74819911	9.997192202	11.35278885	9.884409465	10.54950911	10.41846051
10.36239309	10.47584162	9.874496818	10.27905981	10.23752804	10.38208186
8.459227657	8.367422212	8.630379313	8.494130061	8.548304869	8.017912586
11.02105469	11.04593523	10.84568098	10.73264046	11.32185019	11.33100665
7.738299929	7.593349828	7.483257202	7.718083212	7.459405444	7.782178323
8.227704253	8.402589283	8.049354232	8.176718545	8.228510596	8.466446051
9.03221704	9.028300162	8.829514889	9.235747242	8.868979273	9.304577515
8.995099212	8.096525737	9.560346242	8.175629323	8.735287485	9.223302313
10.73014715	10.22070473	11.26623333	9.878686616	11.0387546	10.34830463
8.454049747	8.111336758	8.55725397	7.784888444	8.26142704	8.125818909
8.245222778	8.304980606	8.477505576	8.059423535	8.436545051	8.028563859
9.720489222	9.815893677	9.780965934	10.21975253	9.75438504	9.840616414
10.99186298	11.27306561	11.11230451	10.93334198	11.2518391	10.2692332
9.696433626	9.663106646	9.240082808	9.455711606	9.660861778	9.744663444
9.481000434	8.881310737	9.659537192	8.945002717	9.489614919	9.001272616
11.66934136	11.65261158	11.71244686	11.73972102	11.81887725	11.45526933
7.391952354	7.481322818	7.638274606	7.457936747	7.498029465	7.383711768
10.57028524	10.59924244	10.9889639	11.22456459	10.48659409	11.04886125
8.157542717	8.461795505	8.104866828	8.44129198	7.948408364	8.281167657
8.342368121	8.634788929	9.303342798	8.641223283	8.213079374	8.408718253
7.290498626	8.098747556	7.708922879	7.104662848	8.264240727	7.174922929
6.772767788	7.196550485	7.168103949	6.802811828	7.804724687	6.586196364
7.848409778	7.512765949	8.219832939	7.204326061	8.17219503	7.994919717
5.753371596	5.75404296	5.76119101	5.760541414	5.754529717	5.767004
10.69099899	10.23160947	11.59654933	9.924448444	11.47871073	9.382205394
6.320887909	6.100439616	5.896855071	6.182938475	6.007036778	5.779008051
11.23116994	11.63629658	11.29947244	11.87175588	11.40145091	10.91176188
7.693828071	8.030181424	7.631297323	8.440058758	7.776629576	7.933961929
12.08943903	11.6445291	12.33273925	12.19086998	12.16942168	12.15752365
8.221010253	7.590558081	7.696259101	7.572084505	8.018723424	7.696877364
8.888204949	8.040196323	9.208914778	8.210602626	8.874191697	8.328154263
10.07764109	9.823678374	10.41846051	10.04160563	9.922126404	10.0719806
6.367511859	6.525371737	6.08802603	6.777950687	6.449076475	6.344013859
8.838495424	9.000672535	8.893968333	8.783584889	8.955359253	8.771294657
10.01622438	10.02923217	9.820433545	10.16546821	10.06173934	9.998242414
11.41981305	11.57178154	11.38178745	11.42178201	11.63629658	11.72126504

8.024633293	8.197369283	8.559975162	8.836542848	8.259704283	8.110881556
8.222643394	8.145962667	8.928157121	8.396532182	8.144908747	8.253254586
8.760291222	8.792195697	8.211411717	8.739635162	8.848914293	8.528049141
7.796432081	8.199709495	8.514909313	8.043387838	7.906781182	7.726392606
9.870931818	9.532335293	9.755162434	9.507772687	9.393335687	9.719830172
6.500607838	6.508927202	6.05895999	6.287217818	6.53485901	6.610658212
8.339115859	8.643636162	8.421350475	8.603276828	8.35667099	8.409548323
6.301916869	7.149903152	6.832363455	6.833099889	7.227602475	7.680542242
8.156410909	8.207537162	8.141366242	8.100222556	7.954205616	8.177906263
9.251353071	9.204855576	9.371514253	9.230202616	8.98371896	9.335891293
6.654064545	6.623383182	6.933276949	6.706906717	6.894670869	6.99955204
8.864430475	9.320176919	8.716864899	9.116804101	9.064430111	9.203760495
7.935875313	8.086566798	7.728044798	7.585232141	7.418256545	7.941320778
8.585303495	8.878776172	8.468418253	8.939630657	8.629055081	8.728449242
8.968391182	9.176817596	8.286128505	8.605125889	8.889922455	9.247274333
9.447242061	9.479084687	9.083741606	9.377908	9.374690616	9.536330707
8.550414556	7.556918475	7.513736343	7.928125404	7.904381697	8.138519566
8.844262616	9.031155364	8.57971698	9.356741384	8.645541747	9.019731374
7.702676091	7.491977768	7.911995121	7.653990838	7.724049626	7.537817576
9.942152202	9.708693071	10.85361094	9.580783667	10.06904603	10.35699559
9.894213939	9.93187304	9.815129384	10.18762619	9.856174394	10.05523038
11.06433979	10.31480319	11.33477589	9.979092404	11.1612024	11.71895032
9.570470929	9.533596364	9.300983919	9.899010949	9.256085253	9.54312803
9.472030465	8.698215303	9.825199323	8.754900343	9.412634061	8.625486818
9.413321525	9.25368898	9.576740677	9.673359929	9.556000646	9.020337111
11.03163187	11.49239347	11.29399858	11.91416468	11.39772243	11.23641801
13.56886427	14.010587	13.26910509	14.25895571	13.78027417	13.82538175
10.15783547	10.65751844	9.644739414	9.895061889	10.09795649	10.37346225
7.386471808	7.287539616	7.36694602	7.690510313	7.238770545	7.713612677
15.08134613	15.09418019	15.22815255	14.99939481	15.21350605	14.07245549
9.627013505	9.477759242	9.438799404	9.550769788	9.526870273	9.288999586
7.344278889	7.343750657	6.705661929	6.778966495	7.023140616	7.171874212
10.5397012	10.19418334	11.1031408	10.8254891	11.64637724	11.28370047
8.385303111	8.316570081	7.993875677	8.150892606	7.866966485	8.639417101
7.276635293	7.381652111	7.334394303	7.486746556	7.44049799	7.555661677
7.294583061	7.348914263	6.818463323	7.334639071	7.109678444	7.189710394
9.356058495	9.346872071	9.069558111	9.298556212	9.271759586	9.370225242
8.151247717	8.203586121	7.860050667	8.191573747	8.090886384	8.197805768
7.767042424	8.10683704	8.632349838	9.129114848	9.026636737	9.362238061
8.213079374	8.687969051	8.520243879	8.277763646	8.189194404	7.405524869
8.691979949	9.032817303	8.354570556	9.013314737	8.463063475	8.706127333
11.34892675	10.91514001	10.94496034	10.40387449	11.38178745	9.760900556
9.290152727	9.212349869	9.629101929	9.153232364	9.494490616	9.312727606
11.06573849	11.12315281	10.47584162	11.18291631	10.82153564	11.23280633
6.649151414	6.807771525	6.373747848	7.151031394	6.601482788	6.614504818
7.38862201	7.940254566	6.82018896	7.005077596	7.158590636	7.688949081
8.85049999	8.868452172	8.712341879	8.722452253	8.328154263	8.505797091
9.076382242	9.561667293	8.754900343	9.727935707	9.337862091	9.331038253
9.069558111	8.984797889	8.543234566	8.602859283	8.704708384	8.51269104
6.907322414	7.157774636	6.555298505	6.881026485	6.671323232	6.853963192
8.484240687	8.69723302	8.180413081	8.851571051	8.53579602	8.403471909
8.103690354	8.567801737	7.930615646	8.579336081	8.298896434	7.734550869
7.443192556	7.649291899	7.196285434	7.442271202	7.464044657	7.371279929
7.867312374	7.576687677	8.156772778	7.618887111	7.794719919	8.248986687
8.115821848	8.190781646	7.917722121	8.057421596	8.029836697	8.424471616
6.679714263	7.045715273	6.611373919	7.015108687	6.903241343	6.448856899
10.47242114	9.967178364	10.90606985	9.904237354	10.5199714	10.59062542
12.10135206	11.48840455	12.44950322	11.34202326	12.0838996	12.39660011

7.427372788	7.895278253	8.424471616	8.794788636	8.706127333	7.553468394
9.843921515	10.52799495	9.586238192	10.39296715	10.13958591	9.519086535
10.28795108	10.46442497	10.02362526	10.3986043	10.38842688	9.638947465
8.462236515	8.627322343	8.072924455	7.999327475	7.933573889	8.405727848
7.233950838	6.615899091	7.790604293	6.759203	6.893115293	7.396159798
7.416298071	7.249066101	7.227602475	7.009472515	7.42989803	7.503972212
6.378136	6.408579424	6.06264496	6.423050626	6.32497801	6.460794313
7.538207606	7.598252121	7.616235111	7.623693616	7.402701172	7.655968232
6.933000202	6.837226	6.354946212	6.743041313	6.893369586	6.858687869
8.709464434	8.857327323	8.003903253	8.462236515	8.433042727	8.639872707
10.03796113	10.01622438	9.329342374	9.793126455	9.941224515	10.22761479
9.530205505	8.856895172	10.00695165	8.465000141	9.534194535	8.950355889
11.06877356	10.67190595	10.95849458	10.71031814	10.97322727	10.19757035
16.57174008	16.49551516	16.73234105	16.28343863	16.68963031	16.36806566
8.427685838	8.448008778	8.540202646	8.76478299	8.840590677	8.528915121
7.841061444	8.571194222	7.986535808	7.983184263	8.12949599	7.771540485
8.713417869	8.929972798	10.14631732	10.32750324	9.916104475	8.651919778
6.700323747	6.520809899	6.934568707	6.676225182	6.410887212	7.046726313
9.128533354	8.128290707	9.123926859	8.509065354	8.816214848	7.822322859
8.400464071	8.286128505	7.866656354	8.245222778	8.067132556	8.177528808
7.910975505	9.08614898	8.881793333	8.502042192	10.47242114	7.288727879
7.744142424	8.163475424	7.411868313	7.536179778	7.199434576	7.515816273
6.307933879	6.286107737	6.812677121	6.580819444	6.454504667	6.648620667
7.588284717	7.775269606	7.304975899	7.658441616	7.555972677	7.70299303
10.1838089	10.42062148	10.11555853	10.38842688	10.11849919	10.20532801
10.85513517	11.69188536	11.21187068	10.67305197	12.49965499	10.95093974
9.57181996	9.686378667	9.219562515	9.501970121	9.613873859	9.511202364
6.060042182	6.056307162	5.919581566	6.145681525	5.83550402	6.052478566
6.315959424	6.09939598	6.006906101	6.043460929	7.107957081	5.798342222
11.34576518	11.53239215	11.24993851	12.02514953	11.34381562	11.38178745
7.642932737	7.822322859	7.951633808	7.681167667	8.08972896	8.636577313
9.898158111	9.755162434	9.849671929	9.649399939	9.809529798	10.2187154
7.532439838	7.735579636	7.261502495	7.364257101	7.28903701	7.536179778
7.423727141	7.409045616	8.105629576	7.785214909	8.027386222	7.237499505
8.572701232	8.941359636	8.530333535	9.001272616	8.574674323	8.759757687
7.70790696	7.801818071	7.461509737	7.51810503	7.854325061	7.610574101
8.41552702	8.453639616	7.723384495	8.496365737	7.985767212	8.397853727
9.491039162	9.850553616	9.223302313	9.377330121	10.09682592	9.966143818
11.74935785	10.6820228	11.73710477	10.2166926	11.09721747	9.900778232
7.816254515	7.595597162	7.148774485	7.356281616	7.02934998	7.662615848
8.719245818	8.405727848	8.100639	8.062539303	8.167813576	8.532980343
8.317411434	8.65914896	8.261781515	8.601386131	8.451550535	8.458697343
10.94640834	10.94786936	10.69793218	10.84176532	10.93935201	10.86447048
9.363823202	9.112697707	9.741357455	9.273022949	9.204855576	9.819688798
15.61926687	15.31193978	15.67809237	15.16803204	15.449005	14.74028573
9.534194535	9.728754818	9.413942061	9.800083455	9.692793657	9.548028869
7.814874545	8.168969465	8.070920919	7.517772121	7.34349297	7.829325051
10.19528987	10.31795252	10.78987961	11.27499716	10.51886057	10.16355358
12.21630458	12.16942168	12.03912361	11.81018832	11.93975968	12.33907422
6.250843515	6.200757434	6.53354203	6.121897343	6.32750801	6.11300404
6.695959364	6.504270879	6.251735919	6.608026545	6.311249485	6.649399919
9.224452333	8.889922455	9.795448596	10.21157382	9.156208313	8.794361798
6.328529444	6.49370698	6.052626778	6.586196364	6.280078727	6.42771002
8.436995596	7.895961929	8.986932636	7.88825004	8.449337667	8.633848111
14.36070414	14.31917195	13.97489728	13.85099976	14.01682491	14.20688311
11.60266156	11.37670858	11.17041501	11.3403052	11.32559397	11.62494396
12.67700968	12.62807191	12.13244857	12.34237091	12.25572441	12.78645268
8.901935111	8.586230848	8.78214798	8.533908051	8.634788929	7.870669919

10.22557955	9.954681949	10.07670007	9.11154599	10.09506496	10.41426339
6.463509232	6.731175838	6.389669667	7.334394303	7.062195576	6.246552232
14.34357305	14.4335752	14.07245549	14.39736901	13.90036211	14.80749806
9.254253192	9.229670424	9.361074667	9.251906182	9.178084667	9.191263899
11.33477589	10.97767761	11.09250749	10.78987961	11.1453264	9.826032111
11.09721747	10.85782556	10.97054498	10.57885616	10.95683616	11.25349741
8.099825778	7.975414556	7.536878778	7.616552222	7.628336929	7.632536929
10.82153564	11.52645916	10.97322727	12.01956714	11.27306561	10.93789932

GSM518931	GSM518933	GSM518935	GSM518937	GSM518939	GSM518941
7.362680232	7.246997929	6.982384606	6.802811828	6.682819434	6.989443677
9.153730283	9.134591697	9.186096828	9.568406747	8.66101001	9.728754818
6.77698199	6.609208404	6.31770101	6.678316576	6.450135586	7.355080212
8.678158051	8.625920364	8.613130333	8.660608859	8.617420111	8.510413414
7.101319909	6.971547919	6.572131525	6.751169162	6.928862212	7.18313201
7.934739636	8.195856758	8.060252263	7.713270545	7.977952253	8.063461202
8.295436051	8.544633697	8.575095707	8.946596606	8.844732879	8.648450303
7.549884232	8.594861495	8.150448232	8.348974465	8.534897646	8.282438737
10.83363242	10.07670007	9.400327152	10.50543333	10.41123643	9.785178838
8.052783737	7.984288364	8.426330949	7.395598354	7.171336586	8.498503737
7.559118222	7.587956192	7.757234778	7.246997929	7.714004283	8.302498081
8.251922909	8.12949599	8.17219503	8.402589283	8.53579602	8.237787788
7.851263121	8.138908141	8.153957263	8.095330333	8.230181798	7.957018646
8.44047504	8.807959646	8.627322343	8.175968162	8.26142704	8.702574091
8.884521697	8.790654051	8.64507902	8.457898556	8.471488636	8.489580414
8.508189222	8.469779808	8.470647758	8.27903296	8.544134919	8.340681505
7.252038162	7.946933485	8.141770949	8.514431737	7.821971636	8.395713444
7.004539727	6.855446939	7.802836333	7.163563677	7.899333596	8.241600556
7.425506818	7.418614091	7.819838162	7.703321374	7.560118414	7.90869301
8.447076788	8.875206121	8.404822414	8.409548323	8.500729384	8.418553323
9.834210131	10.6488688	10.56306707	10.45877561	10.29851595	10.43757507
10.80239406	9.715870253	10.10454015	10.8193093	10.68072859	11.60680648
9.159785394	10.21054893	9.675185677	9.511202364	9.727935707	9.492465394
9.732604394	9.340550081	9.456392222	9.453743545	9.285000091	9.295516616
11.70348036	11.70348036	11.89361272	11.20840189	11.4413808	11.34892675
12.28369472	11.34576518	11.69658017	12.2293359	12.16036216	11.69891546
9.546645747	8.874692596	8.674900657	9.007167758	8.631899566	8.918756414
9.926784556	8.281588828	8.69335002	9.097596465	9.019731374	9.203760495
8.661508737	7.917403434	8.215082485	8.415109929	9.10215096	9.187808616
6.151165091	6.397207283	6.702042636	6.188910586	6.195084242	6.358706071
7.889031475	6.97809596	7.416641556	7.886820596	7.205228253	6.804494667
9.580097263	8.564847253	9.959124273	10.3090972	9.712341424	10.26332497
7.549557061	6.429021788	6.430045889	6.792458242	6.714476606	7.75371804
7.274624242	7.727729838	8.553595747	8.316969364	8.621272737	8.372086737
7.814874545	8.746311293	8.622197111	8.071334404	8.27903296	8.406604404
7.043720485	7.009988212	7.342871717	8.096128192	7.927113394	7.094997242
11.1031408	9.208368677	9.32739897	9.488337717	10.37894268	10.41123643
8.121211444	8.308331848	8.06745399	7.753435232	7.936644384	7.831138545
10.39072525	11.21958266	11.58621592	11.62059475	11.58814301	10.58935103
11.55322016	12.82558839	12.23769992	13.20018245	12.77454263	12.31719428
7.910617626	8.211847566	7.916391202	8.168196313	8.485631939	7.803193929
13.86604428	14.4427031	14.27397716	14.36070414	13.76108056	14.59173737
9.605576293	8.611710212	8.974650333	9.466909636	9.083741606	8.970028374
8.903025495	8.381754576	8.757405333	8.978204141	8.780092131	8.794788636
7.491357222	6.194435071	6.138681313	6.321269657	6.441889697	6.57823699
7.576363505	7.098723051	7.263531485	6.883542434	7.193876586	8.17219503
11.36610866	11.25349741	11.34202326	12.08128174	11.74935785	11.20660223
6.276797273	6.267412545	6.305155434	6.808998949	6.715644404	6.411697737
10.87092264	8.62991701	9.152615889	9.160413333	9.015412101	8.806450121
8.909945455	9.929312889	10.85513517	8.251922909	7.943134	9.986114121
9.215946081	8.768300525	8.859179455	8.48093102	8.845226141	8.600831152
8.256833364	8.588536172	8.805907646	8.768863636	8.587234515	8.627796121
7.838908253	9.103235919	9.168828475	9.138451657	8.714332636	8.915814212
8.973478152	8.796713535	8.838951081	8.526649808	8.813595202	8.65497299
11.42178201	11.29597305	11.54053762	11.06433979	10.99763472	10.97631922
7.248166182	8.166983455	7.914965525	8.108111747	7.647964475	7.661901192
8.818210192	9.530933293	9.452497313	9.277455131	8.927019859	9.807452253

7.714004283	7.85268101	8.212243444	8.408296424	8.257595869	8.676316919
15.61926687	15.90458538	15.88528111	16.12384164	15.90458538	15.15443825
6.891529131	6.803094364	7.243557061	6.710717586	7.047335818	6.995294596
9.876096838	7.996393263	8.436545051	8.876209859	9.04056096	8.827377505
6.808010384	6.694950828	6.699648101	6.539153576	6.507609283	6.751169162
7.326350616	6.62116196	6.779434273	7.168718192	7.088019323	6.788535525
9.776839404	10.22267877	9.86640601	10.25127201	9.776037091	10.17702971
6.138360374	6.487487818	6.616628394	5.785713545	5.808355051	6.034205162
8.509502414	7.758192424	7.72538904	7.688370495	7.933961929	7.767702525
9.179226364	9.565032515	9.703591263	9.848945263	9.689129545	9.229670424
6.485516717	6.22946	6.709290939	6.325145273	6.496571788	6.642822768
10.77762269	10.12909315	10.21375666	10.03701736	10.31997281	9.851320949
7.707249808	7.869709071	7.103272273	7.072618697	7.319616859	7.36694602
7.799524616	8.334587636	8.445759657	8.403029636	8.074096707	8.171788646
13.16151113	12.30167305	13.47832004	13.06967433	13.25686564	13.87958112
7.81270001	7.829325051	7.789881242	7.904747051	8.201745222	8.497259596
11.18636156	11.60479972	10.56548536	11.88375452	11.75908761	11.65261158
6.939713242	6.193398343	7.411561343	6.607353172	6.463778677	7.224700556
7.889381343	9.041236566	9.76546198	9.652939283	9.227275152	9.11211799
10.72246175	10.53623255	10.22448985	10.08325108	9.848945263	9.116804101
7.750957677	8.023099172	8.443547162	8.733447455	8.291610121	8.522641273
9.258612929	8.576891556	8.606624646	8.714332636	8.594861495	8.805485081
10.37026232	9.695719162	9.455711606	9.621550919	9.225515485	9.755162434
11.28896545	9.951175646	10.39747702	10.54950911	10.54078574	10.59292728
7.78118699	6.556248091	6.899489646	7.175476838	7.164403717	7.391342576
7.645943414	7.215770273	7.144887111	6.955441212	7.338887687	6.915128061
9.211846273	9.756054152	9.419654929	9.666877556	9.881912677	9.472030465
6.134723333	6.093889788	6.006630626	6.141021192	6.572131525	6.157664758
7.820222687	7.843077242	7.516138929	8.032499636	8.029005444	7.591105343
6.998508111	7.003715121	7.195224778	7.647332323	7.371279929	7.352546465
7.474938939	8.86638896	7.964547263	7.889761727	8.271405859	8.267244596
8.266388465	9.187244566	8.991970091	8.823394192	8.588971687	8.928828384
10.69793218	10.80782516	10.85923156	10.41626133	10.59180142	10.83930015
6.94407103	6.228213626	6.373366101	6.373747848	6.249788222	7.046480485
14.26676228	14.80749806	14.15092087	14.45281427	14.4335752	14.23242051
9.457741556	9.912769303	9.865550636	9.505859384	9.465659495	9.717365758
8.799348343	8.376704939	8.767368071	8.361327455	8.166983455	8.843148071
8.842639202	9.112697707	8.704708384	8.756396081	8.767872848	8.647922808
8.075284384	9.399129424	8.436995596	8.86024204	9.450568111	8.687541707
10.62142174	8.706567556	9.376667182	9.815893677	9.407554697	10.22557955
9.50710198	9.591816061	9.631182697	9.024707707	9.231349182	9.388912091
8.078389566	8.942313313	8.91530399	8.337114525	8.866888202	8.916479111
8.60808904	7.933573889	7.869709071	7.453021566	7.951199808	7.588284717
9.21067496	8.986932636	9.394567081	9.470840162	9.437491071	9.325601172
8.055916879	7.359577545	7.596851556	7.990313949	7.59785203	7.582311101
8.602379626	9.014335657	9.187244566	8.638952889	8.746311293	8.927019859
8.149032212	7.539167263	7.839249838	8.334981101	8.207537162	7.52469401
9.349388778	7.81867298	8.198946162	8.133993636	8.106019798	8.174069929
6.563407434	7.119081899	7.251769333	7.001612657	6.927522051	6.997949788
16.28343863	16.12384164	16.09492117	16.73234105	17.67797782	17.58093558
7.426803586	8.385303111	7.845981061	7.879985051	7.994919717	7.568924192
10.1885747	10.18098661	10.74690378	10.85361094	10.43872423	10.85361094
6.892302889	6.530395293	6.863923949	6.685307768	7.376100929	7.032569455
15.10875812	14.17411554	14.67552064	15.10875812	15.01206542	15.01206542
9.791460222	8.53628698	8.996667384	9.454390232	9.727055768	8.934248131
8.956407626	8.877626323	9.207219232	9.135618929	8.392711081	9.074692404
7.56320098	7.816627253	7.609232899	8.052417212	7.243557061	7.232737253
13.68884286	14.07988992	13.93694444	14.3093669	13.88591407	13.58996905

12.24097391	13.29684189	13.34730616	13.16151113	12.78185519	13.33155169
7.08136002	6.711872081	6.821773354	6.658129061	6.477937869	8.042161798
11.21338774	11.55524693	10.93935201	11.4090051	11.61117883	11.08107282
7.997183333	7.065103374	7.326350616	8.370422404	7.687096788	7.133813545
8.063008152	7.470612081	7.725703576	8.160266646	8.140977576	8.358783152
8.442251212	7.943747505	8.435296202	8.543686687	8.401317182	8.790654051
10.87518762	11.39382595	11.70812672	11.53677901	11.67593314	11.31661678
8.12156602	7.866656354	8.217984283	7.52530399	8.345781111	9.347406172
12.92542832	13.78027417	13.69473258	13.92894576	14.08841453	13.63435472
6.819920253	7.629344343	7.474938939	7.013654717	7.381324374	7.10439698
11.19817469	8.290353162	9.464440242	10.04682541	9.763138212	10.70775781
10.03385507	9.724814657	10.19236777	10.16546821	10.21975253	9.744663444
9.530205505	8.809951364	9.151431899	9.146732444	9.095162899	9.146178758
7.901142576	7.966742939	8.056301667	8.22437102	7.835124576	8.068564879
8.020887778	7.553468394	8.277405556	8.094218354	7.88183703	8.267698071
5.989890596	5.859458737	5.959324364	6.051908586	6.19477003	5.95059998
8.724013404	6.504968949	6.682081222	7.043955899	6.924744576	7.008962394
15.76560628	16.57174008	15.65613201	16.2036826	16.39933956	15.76560628
6.277726788	6.678790232	6.472782788	6.32572904	6.836753071	6.332053182
8.300483646	9.029402949	8.682101313	8.373919657	8.264694495	8.552208182
7.649926505	7.320933798	7.277222202	7.216096788	7.116617232	7.077389495
8.747331152	8.997224192	9.078331919	9.156208313	9.362238061	8.732044667
7.081850444	7.177458596	7.59656001	7.696259101	7.653305051	7.891912283
6.780217071	9.125507586	6.880472354	7.137450808	7.31374401	6.815473091
7.354460354	8.023866626	7.444499747	7.762120596	7.63758797	7.371573384
13.33155169	13.98831971	14.53168901	14.56207529	14.4185289	14.91973608
8.545617303	7.630594101	8.630877737	8.523456192	8.139691131	9.132080535
7.218399283	7.809338131	7.828260424	8.107270071	7.870669919	7.948048111
7.395882727	8.030614293	8.203151141	7.803944101	8.239474646	8.342368121
7.59785203	7.856753939	7.445432899	7.11962903	7.665645141	7.241813919
10.73661507	11.43208235	11.32559397	11.58421758	11.16734497	11.308834
8.434412929	7.852302727	8.597027768	8.478263182	8.863920758	8.284876758
12.92937388	11.21338774	11.64637724	11.84529477	11.71659127	12.27773509
7.270452455	6.657192121	6.521696273	6.846305687	6.906092707	7.202701576
8.168969465	7.063984253	6.858224505	6.727438899	7.043720485	6.866055313
9.334658071	9.543825273	9.526870273	9.337862091	9.751080646	9.543825273
7.495760485	7.434843313	6.886756677	7.062438556	6.917762192	7.062669222
10.3986043	9.464440242	10.00450786	10.73788216	10.65014074	10.02821235
13.14611959	12.89513437	12.74093701	12.39660011	13.09045797	12.35798465
9.385290182	9.883591758	9.848945263	9.992117576	9.816676162	9.832711535
9.865550636	10.50543333	9.453123465	9.958247111	9.860587788	9.590351455
9.136890505	9.799317404	9.489614919	9.547339828	9.255548899	9.377330121
9.985290333	10.73661507	10.57996368	10.46342714	10.55842197	10.82416773
8.39867397	7.90403798	7.643628758	7.846688253	7.784888444	7.488894061
9.131560131	9.540446515	9.165786899	9.153232364	8.937936253	9.188424626
7.288414848	7.948737212	7.452109758	7.524403394	7.29540803	7.723009
8.904239657	8.056742909	8.124599212	8.231756293	8.249809909	7.954939636
9.270407879	8.217270354	8.408296424	8.550414556	8.597538556	8.272231939
6.556473354	6.62508102	7.037248667	6.922161646	6.873334899	7.219933525
10.53247367	10.97185531	11.19293398	10.78408516	10.86691694	11.27153548
7.185402798	6.412800202	6.833099889	6.943317879	6.762314343	6.920433444
7.340746384	6.408579424	6.590707929	6.472092505	6.42554104	6.501249222
9.816676162	8.634342798	9.590351455	9.742174424	9.582110717	9.408173051
6.93243001	6.943812717	6.770611535	6.61686503	6.888155525	6.914534515
10.2166926	11.78265022	11.58195332	11.35475345	11.05959848	11.44319437
7.708254091	8.83754499	7.617890202	7.869992939	7.950449798	7.858612495
8.116212253	7.512933995	7.31258798	7.975414556	7.974325596	8.057864374
10.32309273	11.46539851	11.45932673	11.66441291	11.5386618	11.4535969

9.251906182	8.257253131	8.405355091	8.53670504	8.473802646	8.368780919
8.504327141	8.459227657	8.709464434	8.791657869	8.891888576	8.553132192
11.81439214	11.86125412	11.62290433	12.33576886	12.24351077	11.80791686
11.29230964	12.75784116	12.34237091	12.79024082	12.4571909	12.52819783
10.84705809	10.95234717	10.66832974	11.38178745	11.50814936	10.72786832
7.449946354	7.124332475	7.249695535	7.25519196	7.243253313	7.401460495
8.245649515	9.210065	9.041236566	8.549970081	8.823889374	8.999492384
7.009209616	6.67332503	6.714232707	6.078577444	6.517425313	6.330710616
9.444057586	9.844817354	8.907429495	9.230202616	9.523397737	8.85049999
7.118205404	8.69335002	7.385541293	7.521572545	8.044608626	7.826840444
7.066488111	7.227000899	7.453956636	7.848409778	7.18313201	7.790242404
7.697940283	8.140128606	7.56320098	7.989899475	8.005727263	7.027744404
8.826393101	7.771540485	8.174871818	8.480536717	8.476994374	8.793874616
6.924744576	7.617189485	7.152687384	7.015344242	7.252299788	7.187213162
13.24400831	13.31405593	13.06967433	13.5160991	13.57574016	13.05987966
7.993077394	7.269892283	7.505451222	7.532439838	7.626470505	7.232151747
11.63837311	11.10460602	10.96311364	11.47682743	11.43565115	10.99469779
9.424273283	9.270407879	9.746145949	9.426142444	9.405539273	9.564343333
10.71503879	10.91903182	10.73911267	11.04886125	10.78987961	10.69931975
8.505797091	9.385290182	9.063885091	9.234418071	9.218466939	8.913024061
7.451183323	7.12483998	7.174161374	7.222702545	7.109678444	7.221024394
8.12949599	7.195502081	7.441392919	7.514105485	7.562928222	7.378186273
9.696433626	10.26729954	8.621689657	9.478383576	9.991229606	9.123926859
9.171801828	9.142159707	9.476588061	9.663106646	9.763952101	9.711723394
9.303966081	9.171164091	8.947623838	8.565346707	8.582586091	8.443956768
7.723009	7.526167626	7.47214603	7.656238101	7.911995121	6.812413525
8.648914657	9.307024131	8.503920141	8.700606657	8.974101818	8.563941778
8.370830747	7.68569196	7.848409778	8.372086737	8.208296687	7.57318396
10.18942995	9.182204323	9.377908	9.783680727	9.581418808	9.346206162
12.76207554	13.78737352	13.38447499	13.5360606	13.44126502	13.15645152
8.670884566	7.770089636	7.651926253	7.72538904	7.801818071	7.569223354
9.387656788	9.70034896	9.739033131	10.03796113	9.633493677	9.959124273
8.471488636	7.865908465	8.199315667	8.618800343	8.60560303	8.72650097
7.803193929	8.191741667	8.961327374	8.773471869	8.37004801	9.238221677
12.03912361	12.21630458	12.23191382	12.16942168	11.92156179	11.92937262
8.063898495	8.683087172	8.366948404	8.591239465	8.24438497	8.41598997
9.035586242	8.327767232	8.556855828	8.78767504	8.649462798	9.302103889
8.816703535	9.266794162	9.429506515	9.689129545	9.452497313	9.503271071
10.52364746	9.839111	9.667708556	10.11452638	10.14631732	9.587719636
6.990730697	7.716479172	7.487351192	7.882525919	7.907197636	7.492628778
8.366462404	9.50710198	9.028300162	8.930963202	9.032817303	9.041820404
8.204737434	7.677264869	8.508645727	8.418553323	7.712213273	7.831503424
10.20358283	10.07764109	10.14895288	9.857940545	9.897493586	9.985290333
10.05523038	9.508442768	9.917863333	9.882779162	10.18468509	9.656571869
9.927686616	11.31852116	11.26815786	10.86691694	10.79420974	11.15808934
7.31991999	8.341133162	7.803944101	7.682204525	7.62575301	7.624016909
8.484723788	8.389243343	8.198599495	8.127895253	8.271029889	8.168969465
9.324974768	8.040196323	8.283330131	8.631899566	8.575566677	8.759757687
6.935569505	7.30556202	7.048453949	6.943550111	6.570036263	7.013654717
7.60576001	7.689906808	7.597541737	7.094997242	7.326942212	7.496684051
11.90674449	13.18348162	13.15082863	13.24400831	12.96241124	12.86086109
14.28325373	14.56207529	14.83117585	14.70594079	14.82007331	14.27397716
14.72870348	14.88238784	14.61202705	14.7528858	14.79546777	14.4335752
12.21312889	13.75520609	13.70143341	13.33661988	13.43523914	13.27940818
8.754900343	9.320176919	9.25368898	9.133918929	9.199527535	8.953164051
7.457359414	7.328995606	7.542431869	7.868722081	7.734170172	7.552449879
12.34237091	12.81379045	12.71142619	12.86955734	12.82558839	12.50706636
10.33696433	9.258612929	9.50710198	9.392652323	9.57872197	9.312727606

8.251125323	8.342368121	8.343220798	8.567801737	7.893273434	7.98690202
9.17861001	8.595703707	8.50673198	9.084401768	9.11154599	8.245222778
7.396159798	7.063477202	6.963095333	6.877631798	6.946417495	7.95873603
6.66391404	7.683943101	7.193299919	7.014542111	7.055635202	7.149049263
12.70787607	7.601643838	8.146343545	8.338355444	8.483326677	8.456191465
7.969046636	9.034403333	8.985325394	8.710842768	8.893419636	9.144457596
7.874063444	7.058212263	7.436640343	7.919201576	7.648319737	8.621272737
7.959488687	8.131862263	8.301657889	8.176718545	8.454049747	7.967920667
9.772250768	8.324453253	9.831834717	10.05688349	9.55462702	9.825199323
8.443547162	7.474688838	7.894315778	7.979449616	7.865213	8.134366646
7.730154939	8.125376242	8.372086737	8.628208788	8.262181081	8.34368697
10.99345288	12.52130815	11.96636849	12.14789109	11.80791686	12.30167305
10.23660558	10.37215733	9.629839465	10.26832521	10.21484784	10.18563382
8.341518747	7.776629576	7.91307104	8.108523939	8.08112003	8.057063626
8.909404788	9.229082869	8.68840998	9.029402949	8.956407626	9.184000242
7.685352364	7.420741101	8.35018502	8.169730152	7.567404636	8.539776737
11.23280633	11.96362869	11.29597305	11.66934136	12.00644476	11.11230451
8.039451586	8.310875455	8.334162556	8.236217485	7.853350485	7.932453717
11.50425191	10.57649171	11.14707085	11.12935449	10.83790729	11.09250749
6.545300424	7.561964414	7.473746677	7.101573333	6.831893455	7.134684455
14.66395575	15.39581235	15.05177986	14.91973608	14.93426708	14.58095582
9.028300162	8.861728919	8.803935202	8.630877737	8.651452677	8.582586091
9.35188598	8.143419303	8.736738475	8.733083212	8.992957212	8.53670504
11.83330316	10.92485902	11.01376888	11.20163629	11.48258282	11.1453264
8.851018515	8.348543172	7.938205586	7.981709394	8.146343545	8.013487535
7.463713737	8.256833364	7.314300525	7.26571197	7.424024737	7.448962859
9.470840162	8.199709495	8.427236737	8.549517636	8.720163182	8.442251212
7.29228501	7.528640475	7.45028304	7.576363505	7.308145939	7.794025323
8.499404646	8.428657566	8.514037303	8.660166475	8.430929525	8.409959899
7.900715717	7.255477111	7.208321485	7.286993737	7.488273475	7.064538818
10.43998577	8.597027768	9.424273283	9.116279576	9.449948384	9.451212687
8.210994667	6.779180051	7.233950838	7.173022687	7.098240939	6.992927808
11.29744903	12.15752365	12.07810634	11.60479972	11.47682743	11.58814301
8.447526838	7.744459273	8.177146606	8.156410909	8.279889293	7.745466071
11.22294817	11.53473316	11.30301738	10.83790729	11.0035084	11.07158908
13.58209631	13.03957854	13.27418186	13.78027417	13.85817003	14.15092087
10.67815746	11.38178745	11.34892675	11.11230451	11.11230451	11.38970236
7.326634626	8.469339576	8.199709495	8.554528737	8.285312424	8.845226141
9.488337717	10.46664928	10.36239309	10.15483313	10.26729954	10.21769501
7.219623727	7.998587576	7.666324061	7.720541949	7.524103909	7.648319737
12.04432	12.16279806	12.3843369	12.32964397	12.03310273	12.07810634
13.69473258	13.47327752	13.87958112	13.78737352	13.82538175	13.94443
10.71031814	10.43872423	10.61867663	10.83223099	10.85225864	10.52476206
8.952561111	9.867911152	9.661659101	9.698181576	9.58708204	9.682548525
10.8778129	12.38794592	12.09225922	12.02811467	11.81018832	11.83072395
8.316570081	9.253086273	9.232683343	9.35728196	9.201355202	9.426142444
8.641223283	8.551751717	8.59942799	8.563085788	8.943861384	8.498949848
10.73132595	12.08128174	11.55928848	11.45002291	11.72355631	11.87175588
10.10093339	8.634788929	9.281923404	9.566253768	9.240082808	10.83790729
7.08777197	6.423272566	6.458735293	6.584387121	6.669931545	6.376693242
7.339214566	7.093823343	7.010749455	6.941467394	6.577737222	6.767959131
7.043955899	8.351407394	8.118136869	7.763846354	7.897743687	8.327767232
8.391020323	8.167383949	8.731576192	8.68840998	8.323157747	8.996148737
8.932594273	7.931708101	8.250622253	8.533396525	8.598008343	8.524867051
9.592426051	9.157435455	9.260607	9.423026242	9.318228697	9.073600626
7.753435232	7.943134	7.655611	7.431765758	7.551492101	7.502411182
8.204380404	7.741148566	7.141989162	7.431067202	7.373983485	7.269598859
8.109266465	7.729067364	8.038729465	7.968691808	8.100639	7.540434798

7.206373697	7.378186273	7.391081455	7.542431869	7.157774636	7.731201121
7.110215707	6.949065	6.983707354	7.304125475	7.000584848	7.012842909
15.34617167	16.4534382	16.12384164	15.95256028	16.36806566	15.65613201
7.442598384	7.081067566	7.271131505	7.082160636	6.927259889	6.974926293
8.080354404	7.784199556	7.650531152	7.141146879	7.220780818	7.554062253
8.254919778	8.116928465	7.950135394	8.773988747	9.016491202	7.893631939
6.817799576	6.725730828	7.182269455	7.318539616	7.101573333	7.106592788
11.55928848	11.90384839	11.39594259	12.02240324	11.4090051	11.38178745
7.597541737	6.994245051	7.289331586	7.291068525	7.033114263	7.738299929
9.125507586	9.706432808	9.773700283	9.867106131	8.978204141	9.715870253
10.02061657	8.980444384	9.634846707	9.444057586	9.600888859	9.823678374
9.151976485	9.277992232	8.960773354	8.949784505	8.855784909	9.473222667
10.59062542	10.56802411	10.32418729	10.53141254	10.63391466	10.40925212
7.979449616	8.734806657	8.341977727	8.208296687	8.147857556	7.951199808
7.439837192	7.113720848	8.292509485	8.32400903	7.949071818	8.137860071
7.456122566	7.332963121	7.102414636	7.212654808	7.387654737	7.057011424
16.06827159	16.02065153	16.8710039	16.64557528	16.17943014	16.4534382
9.455711606	9.721175788	9.548618051	9.287819051	9.287819051	9.441234879
7.023140616	6.272928495	6.44166202	6.190072818	6.664432283	6.477937869
8.799825313	8.740195303	8.402148465	8.740682	8.981975535	8.313601758
6.612316919	7.179751081	7.057330242	7.395882727	7.959080606	6.564512374
8.550414556	8.067838768	8.409548323	8.528512253	8.399553939	7.725703576
7.624735818	7.294284717	7.539487212	7.563777768	7.706567859	7.292849111
13.5360606	13.86604428	13.34204398	13.92172837	14.01682491	13.12562627
7.49893601	6.676440081	6.642585051	6.790568566	6.878694758	6.490634808
9.271123182	10.49592664	10.52476206	10.47242114	10.40701089	10.66832974
12.28693608	11.40495249	11.70348036	11.65918316	11.63837311	11.80554878
9.447991384	11.38970236	10.40278823	10.36134852	10.41846051	10.09161001
8.41552702	10.05523038	7.789255535	8.236633323	8.73262002	8.137479222
8.434806414	8.275579182	7.041898303	7.21150504	7.150454879	7.171874212
9.666877556	9.647889364	9.361671283	9.565660949	9.616057222	9.431415586
7.134964384	9.283145788	8.980444384	8.94874	8.703161646	9.084956182
7.346567394	6.895441525	6.73278298	7.046194354	6.706660646	6.448364717
16.22759729	14.61202705	15.81050193	15.99508933	15.63778652	16.42659037
10.23850383	9.820433545	9.992988556	9.935661677	9.951175646	10.09254304
9.253086273	8.611286687	8.573727141	9.093001495	9.217871889	8.65914896
9.116279576	8.963499596	8.475210818	8.418959303	9.209526556	8.840010495
10.82945836	11.66708917	10.99469779	11.4535969	11.30131952	11.05191968
9.593075545	8.625486818	8.923097444	9.182731869	9.21905796	9.193817232
12.19996238	12.85589012	12.53197264	12.83405786	12.30167305	12.36759935
12.82558839	13.81935116	13.14611959	13.55640443	12.9709957	12.57877126
8.60560303	8.079553929	8.186741323	8.13932801	8.145962667	7.844550919
7.330546788	6.888392616	7.524103909	8.08737399	8.33796001	8.03490996
6.575249545	6.860179071	6.612316919	6.512378081	6.466266586	6.509135818
6.955441212	7.382821899	7.154661646	7.527074182	7.626110576	6.932196717
6.754699697	6.525595273	7.580594545	6.572131525	6.101382848	7.293153485
10.91903182	10.65246846	11.24646499	10.98749259	10.68338253	11.11856721
9.613873859	9.413321525	9.370832535	8.721523222	8.960773354	9.839882283
7.162148556	8.443175586	8.067838768	7.783847071	7.953486636	7.90609097
8.386601707	7.819446808	7.60736902	6.922672051	6.931701566	7.357775939
13.89331098	10.28795108	10.96162926	12.31719428	12.44950322	12.15440913
7.210091465	7.550845909	7.928481586	7.714317071	7.604779273	7.646666444
11.20840189	11.95514832	11.47682743	11.80791686	11.43380494	11.48258282
9.79615097	8.994589434	9.191263899	9.190205293	9.320176919	9.269847242
8.844262616	9.116279576	8.968910758	8.96575796	9.109760465	9.556759333
11.05191968	10.38001468	10.39414857	10.16355358	10.53028115	10.24227579
7.147872253	7.010236465	7.762451808	6.963620111	6.850381687	6.726480707
7.695265798	7.70790696	8.185563303	8.326518101	8.334981101	7.60736902

8.783158111	9.346206162	9.055478465	8.568768141	8.967895455	8.890373556
11.35475345	12.33907422	11.7349036	11.97944932	12.01673563	11.84529477
12.58287167	12.32659908	13.87263802	13.00581854	12.84197228	13.76108056
8.06745399	7.586125333	7.551492101	7.993875677	7.680542242	7.736260545
7.26025397	7.608934747	8.236217485	8.102484232	8.588536172	7.557551899
6.894670869	7.612294131	7.229904545	7.565085768	7.591410606	6.876878333
10.35699559	10.26445444	10.1905069	10.25811169	10.31013098	9.925979414
12.49965499	12.34855768	12.9851923	12.4956701	12.7494647	13.33661988
7.327234414	7.022261949	6.669224869	7.178905394	6.964119424	7.491688212
7.681488071	8.061012707	7.883157929	7.52646604	7.644626202	8.152794889
8.30460302	8.08537204	7.950819283	8.248566778	8.207926798	8.113343889
10.95849458	10.06255975	11.06732533	10.761049	11.06573849	12.40343679
6.829338141	7.155513182	7.734550869	6.283736071	5.821140475	6.508494242
11.29399858	8.754900343	9.328020364	10.75178718	10.10365156	11.92396137
10.88546605	10.66465811	10.50543333	10.17702971	9.971733293	10.25038968
7.427999919	8.831800202	8.494576061	8.836542848	8.722953263	8.399975414
6.769202101	7.645943414	7.481645051	7.136586475	7.432070859	7.305287707
14.19920898	15.16803204	14.85646221	15.01206542	14.6970922	14.60195877
10.48185706	10.6820228	10.90754603	10.38738452	10.42927797	10.57159251
7.89560499	7.205488778	7.504556444	7.473129747	7.389870293	7.172411071
7.935875313	8.327353333	8.087781242	8.167813576	8.063461202	8.326884343
11.39185194	11.83330316	11.89130603	11.63401423	11.44491584	11.61117883
9.478383576	8.867918475	9.741357455	9.481000434	9.815893677	10.03876748
10.19649023	11.27695531	11.00183361	11.31661678	11.15666056	10.81796524
10.7814083	8.940763364	9.250770152	9.45706402	9.332795131	11.68243664
11.9091429	11.54675633	11.59212007	11.4413808	12.04160066	11.94745099
11.7349036	12.59323492	12.29548604	12.052647	12.06692054	12.04734694
7.885378	7.90290904	7.439506505	8.200163707	8.004282899	7.871272152
10.65751844	10.74945851	10.32309273	10.17030341	10.74819911	9.432636424
9.023071899	9.191263899	8.930963202	9.235747242	9.045168101	5.824177354
9.372705202	9.602112616	9.54312803	9.895856061	9.679645121	10.9660467
10.32093584	10.97476091	10.83501466	10.58117749	10.33989796	10.70299608
8.898231131	9.459003	9.370225242	9.311440606	9.07581704	9.541747455
7.980934222	8.57070398	8.394903626	8.181196919	8.409548323	8.326518101
9.139724848	8.39308204	8.455403657	8.490531303	8.443547162	8.104133364
6.068447646	6.180964455	6.271013081	6.765091091	6.27919301	6.963620111
7.143674071	7.415013667	7.259306869	7.304428303	7.559825556	7.266813283
8.342773172	7.662925475	8.231756293	8.695776131	8.404396899	8.77962597
7.626470505	7.394983566	6.955646677	6.759203	6.973416747	7.368501293
12.15440913	11.98999321	12.04432	12.19398134	12.51066587	11.98196729
5.831669364	5.761927394	5.758593485	5.762992657	6.416564263	5.766924545
7.584276586	8.347717242	8.037664566	7.681488071	7.669657737	8.431738505
12.97555423	13.64789782	13.25686564	13.2158469	13.06488572	13.18348162
7.407119727	7.350681061	8.21550303	6.710494808	6.601004717	7.946933485
9.623794152	10.55842197	10.75600216	10.59292728	10.28406896	10.3986043
9.822898081	10.51771241	10.10542249	9.987874111	10.04340808	10.08231743
10.48539779	11.91416468	11.60266156	11.17750487	11.38577951	11.109559
7.917403434	7.006187899	7.199149121	7.438935576	7.440763838	7.600942677
10.20634509	9.662314131	10.07107569	9.988725727	9.745446081	10.55842197
9.078331919	9.336479586	8.844732879	8.317824071	8.457043091	8.803482152
6.445028586	6.839237455	7.387654737	7.422534091	7.129030091	6.817996071
10.7982674	11.22600179	10.97476091	11.08936677	11.50051559	10.84568098
11.06123261	11.64044206	11.21816154	10.37686258	10.48185706	10.94353341
9.992117576	9.658049545	10.49592664	9.801802283	10.40074116	11.21016953
8.099112939	7.743466333	7.870356606	7.912742707	7.785596848	7.63758797
7.682204525	7.691535778	7.923778798	7.663284545	8.130993828	7.744142424
8.53628698	8.175629323	8.78563097	8.516793455	7.969793182	9.599465606
8.567346869	8.210994667	8.322806515	8.476994374	8.45110203	8.192996515

8.655934636	8.682574626	8.59026096	8.715845505	8.810446485	8.279889293
14.90726461	13.04381444	14.36999747	14.37933382	13.92172837	15.38120792
8.206690121	8.842639202	7.708578687	7.676615465	7.737649848	7.334066545
6.617377051	6.635108111	6.658617323	6.681596343	6.688467242	7.045942
11.9982543	12.26475658	11.86922315	11.85617239	11.86125412	11.78498903
8.874191697	9.153730283	9.155079848	8.640338566	8.331458747	9.179226364
9.513795939	9.639623879	10.00361533	10.05970796	9.625623061	10.42175216
9.30770201	8.184399818	8.239474646	8.807959646	8.78214798	8.887109626
7.590558081	6.834834778	8.197369283	6.90482902	7.592388485	8.721523222
6.220682606	6.087779919	7.386173242	6.221954697	7.421318101	5.943094152
8.369612525	6.789025475	7.921213404	7.74961303	7.18112796	8.83754499
6.96124304	8.121211444	7.58642403	8.049019253	7.716479172	7.841440677
14.17411554	15.10875812	15.22815255	14.77222727	14.7528858	14.80749806
8.191909586	9.239472869	8.600356081	9.055478465	8.308776677	8.337114525
7.639261768	7.993875677	7.732206152	8.001277303	8.462236515	7.878949444
11.81887725	11.08264222	11.0940021	11.58814301	10.96162926	11.22119175
6.996769242	6.770374545	6.845030273	6.787317758	6.583688333	6.382999071
7.468171747	8.507211606	8.123844788	8.00165403	8.077645899	7.965251131
9.081927515	9.069558111	9.204855576	8.947127939	8.914132333	9.344897333
11.65261158	7.241538444	7.490096869	8.209418434	8.298896434	8.173308798
13.95955505	13.54221797	14.37933382	14.223913	13.90036211	14.78379092
8.680513909	8.871686232	9.09013302	9.119222505	8.775001677	9.089024899
13.79381649	9.488337717	11.13085117	12.18492446	11.86652814	13.30795266
11.49839615	10.41957053	12.09497108	11.98999321	10.82804231	11.59654933
6.751979343	6.639490384	7.085333222	6.97366599	6.907322414	6.66692301
6.472092505	6.960198707	7.240274404	7.269892283	7.022261949	6.999250869
8.236217485	7.90609097	7.467207283	7.74646602	7.671593667	7.944736172
10.29949835	9.981718727	9.485668061	10.13768673	10.1300726	9.821914929
6.009166495	5.816346071	5.807087303	5.856338071	6.459198636	5.975206535
7.355706667	6.917762192	7.962380919	7.945111576	7.841440677	7.684965778
11.21816154	9.404333879	10.59685822	10.70052069	10.57996368	11.05796986
7.351279444	6.978602455	7.117115202	6.74432	6.735859162	7.230593889
7.357775939	6.772272192	6.781434051	7.244122677	7.085629475	7.098240939
6.478141798	8.127056495	6.842028788	7.144887111	7.753435232	7.485191121
9.306330646	9.869466131	9.650129091	9.660200838	9.689783586	9.821195081
9.860587788	9.725631121	9.574750414	10.11555853	9.930141081	9.562994313
9.348735909	8.476994374	8.615330515	8.482406475	8.508645727	8.457898556
11.71244686	11.18941159	12.00354228	11.74205097	11.39382595	12.12692782
9.087865687	8.046784596	8.185177828	8.295839707	8.300888657	8.166602566
9.713858919	8.282073859	8.496814303	9.448606737	9.257324	10.71031814
6.573293212	6.396609343	6.347255636	6.376277596	7.321884212	6.407674848
9.080683394	9.341222778	10.08799683	9.223885747	8.607126586	9.987077505
7.580266434	6.59504903	7.470612081	7.399710364	6.307278677	7.537489808
10.0728998	10.4524716	10.12610261	9.938475404	10.06255975	10.2166926
9.70798304	8.976374343	9.405539273	9.432636424	9.239472869	9.701165495
16.3077943	13.2158469	14.03424604	15.32551587	15.32551587	15.16803204
9.119831202	8.428176394	9.200683333	9.239472869	9.033394545	9.491725303
11.21668249	9.559682182	10.65500785	11.00620373	10.45996909	11.89361272
8.097222414	8.43615796	8.532119949	8.096525737	8.404822414	8.342773172
11.50051559	10.17122401	10.8605042	10.91641235	10.94353341	10.7814083
7.191532202	8.204025576	7.628336929	7.587635919	7.412806768	7.557888444
7.212654808	6.595504071	6.372952485	7.12824003	7.256041222	6.888392616
6.409482747	7.143149899	6.488188354	6.617782121	6.426632697	6.963370131
11.93770961	10.22668357	11.57178154	11.81221198	11.33100665	11.8164662
8.288355808	8.435296202	8.483326677	9.038261515	8.442251212	9.320176919
8.752460404	7.689234404	7.780823677	7.937437293	8.037664566	7.907924051
10.5397012	8.500729384	8.932594273	9.821914929	10.0143126	9.905108616
7.701029869	8.613521394	8.751946515	8.883414061	7.993428586	7.544367232

10.6313464	11.3403052	11.0035084	11.25700815	10.95683616	11.2345748
8.71185199	7.484183869	7.569223354	7.719835253	7.465665798	7.644626202
9.436357141	8.911109919	8.453639616	9.421059	8.755916071	9.053706202
7.112620848	6.421771838	6.513435323	6.736575242	6.850100909	6.494395354
10.52904685	9.584100455	10.31193963	10.22164118	10.04776272	10.27035429
7.739667424	7.407119727	7.630934212	7.940608939	7.721209061	8.525321657
7.591751808	7.062195576	6.857999616	7.058479485	6.95496698	6.71730499
6.685084394	6.957403667	6.639726192	6.573737152	6.382999071	6.337620818
10.48432644	11.16423752	10.90466263	10.48185706	10.71860391	10.99046505
7.398020586	8.118473111	7.723009	8.027386222	8.283707475	7.580594545
8.21550303	7.720869455	8.057421596	6.931501667	6.600294051	8.620285051
6.806225091	8.18280501	7.784199556	7.535866141	7.540434798	7.890755525
8.93587504	7.258429848	7.501443394	8.217667525	8.109266465	8.276867707
8.304980606	8.240781051	9.377330121	8.670447949	8.483763566	9.880317061
12.40735915	11.67371856	11.41415047	11.81887725	11.45738434	11.93770961
8.31912796	8.234232515	8.187593091	7.809644101	7.814874545	7.948408364
10.96311364	7.437630343	7.975784071	8.692466525	8.734416606	8.587234515
10.09506496	11.03580591	10.76361324	10.48659409	10.13958591	10.74045328
7.600942677	7.160253414	7.631600293	7.449946354	7.409710545	7.580904889
9.299183616	9.647013263	9.540446515	9.816676162	9.937540263	9.745446081
6.862895808	6.601976232	7.653651778	7.068665737	6.663425212	7.183932212
7.54886499	7.23423804	6.864210101	6.913051818	6.899006192	7.039421667
10.42927797	9.756840071	10.24414187	10.10187506	9.844817354	9.973623707
8.601896768	9.637663091	9.511857455	9.076382242	8.958493495	8.966940586
12.39973853	12.26775966	12.25572441	12.58612865	12.39058663	12.02514953
9.172421828	9.669847545	9.654354424	9.479751879	9.490338525	9.569063343
7.062669222	8.518144394	8.476994374	8.240781051	7.928832091	8.516311424
9.141588162	9.243096889	9.009915859	8.847386828	9.182204323	9.323731354
9.187244566	10.23550694	10.06255975	9.94767803	9.861430101	10.05053825
6.783830859	6.170864828	6.37731698	6.623383182	6.366826091	7.005354859
7.423442323	6.807771525	6.935285707	6.895973717	7.528004313	6.848276364
7.554062253	8.390114414	7.73355204	8.041332778	7.780448354	7.143149899
8.628622212	8.628208788	8.544633697	8.802385697	8.827377505	8.598479657
12.10694646	11.12001247	12.34855768	12.00086001	12.3543152	12.72610186
9.864614818	8.812013889	9.521192909	9.739033131	9.445197818	10.6362571
7.612909768	7.042119313	7.061925202	7.306206455	7.714642404	7.346859747
13.20536123	12.06692054	12.96662998	13.15645152	13.20536123	13.29684189
11.90384839	12.76207554	12.32964397	12.42943771	12.34855768	12.24097391
11.05651949	11.41981305	11.25700815	11.28565422	11.13879804	10.74945851
6.194435071	6.184860172	6.555084162	7.606043535	7.264510162	7.205228253
7.650858424	9.060618192	9.376046232	9.230770667	8.865915939	8.486968232
6.943317879	7.926719768	7.605411525	7.552833101	7.638981707	7.823083152
8.876209859	7.463067838	8.209801687	8.225777414	7.895278253	8.576891556
5.777924636	6.046293505	6.607353172	5.84635299	6.288561919	5.948711596
10.3779146	10.69931975	11.07493153	10.61261659	10.47584162	11.06123261
7.660499434	8.05474504	7.454572909	6.873566444	7.344882899	7.731871869
15.63778652	12.71830686	14.19920898	14.84412351	14.59173737	16.39933956
9.044046343	10.61743608	10.75987116	10.0841781	10.12408141	10.04511943
8.293340606	7.438305869	8.102124455	8.755421576	8.378142071	9.290152727
8.816214848	9.826032111	9.760900556	9.204855576	9.807061475	9.645545465
9.430738808	8.409959899	9.051375475	8.920488949	9.060618192	9.215289101
9.285586323	7.974325596	8.925792808	9.439397061	9.038849273	9.766921414
8.813032152	8.636577313	8.573273283	8.383846919	8.521230828	8.720163182
9.101613848	9.015966535	9.103811545	9.350012182	9.269847242	9.264483889
8.757866131	8.157901343	8.829061111	8.938439909	8.825911939	9.136890505
8.104866828	8.972927475	8.662363172	8.466852202	8.285708747	8.832790172
7.604109192	8.009790253	7.980568657	7.862831293	7.884314808	7.945472162
6.714999636	7.077096727	6.687730495	7.082435283	6.803320101	6.69943696

9.000672535	9.823678374	9.840616414	9.517771202	9.292435667	9.839111
9.127872697	8.889922455	8.569209263	9.397864101	8.695776131	8.974650333
8.966940586	8.417196576	8.275152535	8.124212202	8.491898242	8.346989566
8.906910121	8.456593071	8.379510323	8.039790061	8.522156747	8.407442505
9.768390455	9.704210374	9.517013525	8.944409727	9.436357141	9.684048687
8.560348606	8.514431737	7.758641747	8.541125101	8.47107903	7.784529071
8.989652131	7.88183703	8.461795505	8.675789182	8.739178636	8.653906556
9.450568111	6.922161646	7.532439838	6.860390606	8.25927404	8.664725051
9.647889364	7.168103949	8.942313313	6.550314424	6.683324051	6.559271747
10.00985275	9.425521616	9.509876273	9.714566535	9.684048687	10.29208493
8.421742051	9.159196141	9.477759242	9.261851101	9.533596364	8.881310737
8.47014499	9.17861001	9.002847848	9.072403576	8.91530399	8.985325394
10.16262583	10.88430477	10.42409385	10.20859114	10.32635032	10.35178281
7.810269677	8.147481596	8.416400293	8.757866131	8.641223283	8.650384111
5.787901081	5.775592576	5.771685111	5.768021929	5.767158131	6.92802298
11.00183361	10.73517618	10.84302834	10.29949835	10.51291939	10.67190595
10.89094316	10.70299608	10.73661507	11.06573849	10.65500785	10.73788216
10.6488688	10.48539779	10.30569114	9.790672212	10.3779146	10.32093584
11.58421758	11.61814895	12.90783816	11.9875501	11.73972102	12.51424883
9.602761333	10.07580535	10.44934262	10.31795252	10.57282784	9.75438504
6.975188859	7.580266434	7.300950758	7.090758091	7.446413172	7.28582396
7.182015707	7.283562505	7.897743687	7.952386505	7.657256192	7.88825004
7.394027687	7.736964596	6.782883697	7.32489101	7.328411747	7.799524616
10.94071069	12.03310273	11.50632759	11.37490296	11.36065784	11.56291631
8.812517081	8.714332636	6.997357515	6.646430939	6.843774949	8.784649081
8.416400293	8.284876758	7.979863071	7.817975838	8.460053242	8.202220545
9.285000091	9.80784303	9.601568657	9.290152727	9.557589465	9.540446515
10.3090972	10.39296715	10.54800525	9.977292899	10.22164118	10.27139209
6.450820343	5.925521111	6.059213889	6.827413455	6.613208707	7.466006556
8.785177889	9.012760899	8.970028374	9.158023	8.86024204	8.265565879
9.069558111	8.257984293	7.991473828	8.84786598	9.009407333	9.775265525
11.68243664	12.54906863	12.52461444	12.12939166	12.13244857	12.39660011
11.91912022	12.88708268	12.84627851	12.40343679	12.4956701	12.76207554
6.044011556	6.138681313	6.353400323	6.264653758	6.633928414	6.199670273
8.467667768	9.398445515	9.266794162	9.030004576	8.80133401	9.153730283
6.410887212	6.890245747	6.786285848	6.700834859	6.628304848	6.537747374
8.48093102	9.050797061	9.045755677	8.613985131	9.018802576	8.898231131
6.121756828	6.799617657	6.881026485	6.914843414	6.893369586	6.64590104
8.061012707	9.074692404	8.860747333	8.248986687	8.217984283	8.60560303
9.235179424	8.899757485	8.758871	8.318668263	8.680513909	8.574674323
8.403029636	9.106823152	9.08959502	8.798818646	8.668536687	9.015412101
10.80499832	7.671593667	8.204380404	9.185002364	8.942756889	9.969958172
12.89513437	12.72610186	12.85048632	12.70787607	12.68097339	12.72219022
8.703161646	8.742149222	8.330618758	8.884799985	8.810986707	8.431326525
7.198597879	6.710717586	7.690204818	7.428304182	7.356281616	7.619556354
6.923947081	6.527432758	6.614995778	7.262118616	7.037248667	7.006478434
7.301506111	8.207099091	8.119679141	7.958393384	8.105629576	8.074096707
7.755503707	7.841061444	8.009072212	8.699656879	8.06213903	8.363468485
8.843670869	9.987874111	9.495686737	9.756054152	10.00695165	9.529469889
8.771294657	8.247731232	8.643157737	9.030619364	8.532980343	8.85785699
6.598705667	6.769655182	6.970769556	8.609952768	7.823737283	8.189547909
8.636577313	9.161011869	9.126663535	9.323195364	9.208368677	8.935320828
10.04340808	9.573320202	9.488971182	9.099925818	9.435722162	9.283145788
8.399075535	7.786626808	7.910617626	8.203586121	8.052077899	8.416773909
9.575401111	9.784374313	9.497817424	9.793126455	9.416442616	9.394567081
12.59668127	11.88148631	13.04381444	12.92081703	12.32659908	13.47832004
7.452739495	5.850261818	5.968264535	5.94936203	5.927828667	5.75903501
7.309054838	7.309668525	7.68296396	7.265083586	7.526779747	8.047188455

7.711891616	7.343750657	7.369510384	7.969427899	7.471847707	8.459227657
14.50017253	13.28559105	12.8293171	13.5964752	13.74093847	13.22156044
7.404680798	7.358963616	7.637262101	7.700670768	7.556918475	6.526325283
7.441392919	7.953840929	8.074508444	7.961659859	7.59859104	7.795116566
12.0724233	11.50051559	13.14093405	12.04734694	12.67700968	13.5964752
7.392563535	8.574218515	7.812003626	7.927460424	7.789881242	8.102124455
9.296131263	8.84935699	8.733083212	8.357077121	9.254890202	8.671294253
11.15503783	10.29000276	11.97660652	11.19966733	11.49636188	12.40735915
6.723501929	6.917222737	7.11489098	7.278115626	7.242080394	7.461239687
8.214296636	9.326739394	9.643287657	9.735412273	9.785178838	8.852576737
8.300103657	8.134366646	7.987573434	8.363019899	8.314030071	7.647332323
8.400903717	9.478383576	9.436357141	9.486281202	9.480384485	9.412044242
8.226500707	7.813759899	7.788172758	7.410944101	8.100222556	7.687700545
11.4090051	10.042425	11.45176908	11.02851208	10.34311059	12.47761486
5.95459801	6.07690697	7.665645141	6.197177	6.384073253	6.235905444
8.762202232	7.151291707	7.17606597	7.26819499	7.056715131	7.729367111
14.47046648	8.861258687	10.05053825	11.41071085	11.69188536	11.00620373
6.895441525	6.756647869	6.968393455	7.092673939	7.016713091	6.969423263
11.80791686	10.52585606	11.08756575	11.02403513	11.46936418	11.21816154
6.951451	7.126018333	7.495088838	6.989443677	7.07857001	7.541750556
7.207790596	7.634532263	7.082435283	7.207252646	7.212087616	7.151567818
9.271759586	8.22479296	8.629055081	8.732044667	8.520243879	9.410728505
6.668510737	6.738515899	7.19681397	7.362377768	7.018881949	7.729749768
7.358963616	7.522209596	8.943348051	8.435296202	8.135177152	8.063008152
6.704918465	6.431584121	6.370113596	7.499887141	7.204042263	7.253486596
8.483326677	7.763846354	7.813759899	7.931708101	7.97471396	7.817975838
9.260036485	7.896955242	8.243637242	8.852576737	8.545617303	9.329342374
8.066048727	7.478150263	7.489176	7.543741273	7.509050374	7.289658101
8.840010495	8.783158111	8.937936253	8.907429495	8.731576192	8.597027768
7.838558162	8.64752602	8.534432515	8.855784909	8.72650097	8.514909313
9.426142444	10.44195016	10.96748398	10.72246175	10.49009312	10.24414187
11.06573849	11.37131374	11.06433979	10.80644997	10.73788216	11.10170724
6.791503909	6.684069253	7.040523253	6.999250869	6.722265657	6.836068354
7.199149121	6.831376636	6.825072616	7.126269081	7.123768636	6.947262939
8.683580283	9.648698737	9.349388778	9.687758929	9.393934131	9.383480283
9.422377343	9.829119768	9.633493677	9.531623293	9.385837242	9.885156737
9.571117879	8.921533879	8.975268424	9.747577162	9.542425758	9.320738646
10.94353341	10.89094316	12.18492446	10.36349344	9.805520727	10.4700758
10.02269473	10.87379507	10.62895885	10.90606985	10.73264046	10.55966675
11.76588718	12.74093701	12.64708972	12.90783816	12.53563192	12.3613163
12.66167346	13.09977851	12.8652792	12.50706636	12.47761486	12.54268587
6.469428232	5.875358434	5.962183081	6.38878402	6.253125879	5.956757394
10.66229262	10.06450841	10.30151978	10.36781996	10.1684149	10.11246778
10.11349335	11.1502402	10.687913	10.87637444	10.90754603	10.65365596
8.593558293	8.715845505	8.437898414	8.802954374	8.845785121	8.60413102
8.24438497	7.889761727	7.802180768	7.766738848	8.018723424	7.639662323
10.07956055	10.57159251	9.538964939	10.62895885	10.573928	10.36589334
9.784374313	10.61001127	9.934615404	10.58935103	10.45628541	10.06547375
8.528512253	9.217165182	8.602379626	8.416773909	8.802385697	8.881793333
9.724814657	10.26541026	10.25127201	10.23247836	10.49348589	10.51400776
8.847386828	9.736957071	10.23948175	10.4700758	10.03026493	9.93187304
11.88375452	10.69793218	11.22456459	11.32185019	11.37490296	11.12613667
13.95167504	14.76275492	14.1282639	14.52085241	14.66395575	13.99495772
10.69222123	11.58195332	11.10460602	11.49420288	11.80554878	11.17378617
8.961854929	9.321347081	8.778623323	8.670884566	8.795325869	8.569209263
10.66587479	9.271759586	9.742174424	10.28795108	10.3305428	10.93171462
7.190644495	6.843545424	7.303836939	7.993428586	7.571747222	8.201745222
10.45877561	11.28053313	11.09565519	11.80342824	11.65918316	11.4481816

7.34349297	7.376443525	7.856391758	7.708578687	7.417598061	7.824443343
6.275469879	6.69943696	6.716343364	6.995294596	6.842273677	6.154324444
7.486457414	8.203586121	7.437044505	7.526167626	7.619392202	7.787525596
7.334066545	7.288128061	6.943066869	7.251486172	7.323648515	6.833840465
8.390560192	9.354902374	8.721926778	8.906407303	8.841575667	8.831201768
7.892277636	8.857327323	8.241600556	8.339115859	8.267698071	8.409136758
7.487940131	7.347416111	7.622197263	7.728706424	7.683943101	7.361082222
8.548679899	8.116212253	8.083806798	7.925642	8.232163879	7.828991111
7.747885899	7.856391758	7.875433545	8.160644788	8.450171172	8.111719586
7.770819434	7.447717303	6.971796172	7.342871717	7.367244707	7.200755828
9.107388182	8.788135667	8.657372182	8.440058758	8.840590677	8.805907646
8.699656879	7.588938485	7.784888444	7.924502232	8.008400859	8.01602699
8.827377505	9.457741556	9.185497838	8.878776172	9.073600626	9.241421747
6.540929384	6.562503909	6.747074889	6.88247998	6.559271747	6.116729
9.042346232	9.397189192	9.368224626	9.058895657	9.022000869	9.287277576
7.877844556	7.032840081	7.291068525	7.681167667	7.534885556	8.056742909
9.11510898	8.373157505	9.119831202	9.035586242	8.933180404	9.253086273
8.339926909	9.183367525	9.277455131	9.079484556	9.326238727	9.618256808
5.764132455	6.053020636	6.015598859	6.113628535	5.965974051	5.913577182
8.295009141	9.37540303	9.130979939	9.054860525	9.271123182	9.030004576
9.106823152	8.508189222	8.687969051	8.282073859	8.707515232	8.361327455
6.482290162	7.057011424	6.908606212	6.441889697	6.647414293	6.345431182
8.155023263	7.668638899	7.529315566	7.706567859	8.23139899	7.708578687
6.756145354	6.799149535	6.729687141	6.826634828	8.408718253	7.437630343
7.592047838	7.66151798	7.588284717	7.581254909	7.546592828	7.932792414
7.572084505	7.058764576	7.02934998	7.354199768	7.236577444	7.120752303
8.810446485	8.388817192	8.489178071	8.521230828	8.718769576	8.528049141
9.431415586	9.046862848	10.31997281	10.06255975	10.69222123	10.61261659
6.665428859	6.564057303	6.435093051	6.258944949	6.066662545	6.232724131
8.53715797	9.006527424	8.856343687	8.626833051	8.814101707	8.741655828
9.641007131	10.65500785	10.34100163	10.20164867	10.01974654	10.31480319
6.568165434	6.420580374	6.065799515	6.337413646	6.666157333	6.353596172
9.198436465	7.628690051	8.166602566	8.60413102	8.350987111	9.112697707
7.729067364	7.285574899	7.245604273	8.873520545	7.541384646	7.556609313
6.391201182	6.727165222	6.921167192	6.882010616	6.637264	6.637907929
9.979092404	10.14895288	10.02061657	9.904237354	10.00002479	10.13862753
9.287277576	9.630502939	9.406893465	9.361671283	9.291911616	9.016491202
7.712908172	8.430534091	8.74840203	8.728878424	8.836023374	8.669446475
11.37670858	11.57765358	11.21493335	11.59654933	11.60266156	11.01531103
8.486053202	8.465000141	8.474228909	8.563941778	8.377090384	8.002011657
9.211184727	7.74578199	7.906425364	8.387939343	8.187780773	8.400464071
6.444123263	6.86860504	7.184502222	7.155245384	7.862831293	7.064243152
6.752731616	6.97260104	7.151567818	7.097545323	7.02750098	6.708558414
5.952773323	6.080185455	6.212710222	6.826151202	6.23359303	6.165641212
11.08107282	9.751885293	10.2829717	10.46442497	10.4700758	10.80389925
10.94208941	8.955880859	10.82416773	10.51886057	8.159434576	8.022369434
9.504584061	6.96917198	9.214694788	9.005975162	6.625506687	6.519665929
11.03008027	10.66105788	9.861430101	9.812775374	9.824540899	9.459003
12.27773509	11.27153548	11.5571076	11.0940021	11.68700199	10.77890896
11.86125412	10.99186298	11.24147284	11.33657706	11.42787377	10.57282784
9.206093556	8.823394192	8.73262002	9.035040848	9.167114	8.507211606
12.99088515	13.70717091	13.5964752	13.47327752	13.42888134	13.62784329
7.661901192	8.271795141	8.444861909	8.393536232	8.227704253	8.131862263
7.186028475	7.68296396	7.42989803	7.477539293	7.089925434	7.858214495
7.858214495	7.695611414	7.562627657	7.918472515	7.683318778	7.884680111
7.328126596	8.44446797	8.31318196	8.200539131	7.926300626	8.277763646
9.004940778	9.071813909	8.944409727	9.116804101	9.217165182	8.946107263
8.210602626	9.226702434	9.019287687	9.636296737	9.051375475	8.875711838

9.350012182	7.08018898	7.352900667	7.175214949	6.994769535	7.987912222
8.303793737	9.423636879	8.889922455	8.750392838	8.851571051	8.904239657
7.477539293	8.021984222	7.89464897	7.837840162	8.486053202	7.914965525
10.32750324	10.24942024	10.0053333	9.162016475	10.03123363	9.835894606
6.563203242	6.663425212	6.504968949	6.758676202	7.053922394	6.988974929
12.80166365	13.50411017	12.91656586	12.30465105	12.76628311	13.04869312
11.13713447	10.47941256	10.25519474	10.12408141	10.16165443	10.12041459
11.02551486	10.70181173	10.75453103	11.23641801	10.93171462	10.87235819
6.76551097	6.334404253	7.06161799	6.588688525	7.012316737	6.811363364
6.860390606	7.105472313	6.693719727	7.398696909	6.922672051	6.674729293
9.89348604	11.00620373	10.77762269	10.54190997	10.81034167	10.54441943
6.841827051	6.731476051	6.12333103	7.356542485	6.674272111	6.091152697
10.28089537	9.926784556	10.761049	10.76239578	10.21375666	9.90675497
7.171589687	7.041369354	7.798826303	7.088821283	7.454260455	7.373717778
7.411868313	8.32939098	8.210186616	7.819838162	7.774617596	8.256076333
13.70717091	13.46005067	12.92542832	12.04160066	13.00581854	12.63888703
6.579844919	6.480359747	6.511476192	6.77992704	6.524953909	6.303681202
8.011239071	7.712213273	8.583996586	7.759936788	8.352944343	7.55847901
9.705000525	9.671912455	10.0143126	10.17904437	9.899969596	10.21054893
10.69682519	10.45628541	10.31378621	10.12126634	10.12314196	10.30569114
7.551170919	7.291982576	7.71023803	7.499556727	7.005636414	7.507381747
11.44319437	11.542568	11.911688	11.74670945	11.36793803	12.03310273
6.298407384	7.489176	6.279360232	6.463086606	6.793493859	6.353983172
9.188424626	9.143279535	8.978723121	8.372433475	8.776574232	8.695776131
11.3290504	11.8164662	11.44319437	11.89361272	11.86401838	11.34202326
6.84909404	6.928307859	7.220481808	7.06161799	6.950101798	7.108809091
8.023099172	7.735250384	8.049354232	8.829514889	8.48873997	8.359650919
8.928157121	9.118059475	8.886096818	9.151976485	9.328711051	8.816214848
8.999492384	8.39867397	8.47107903	8.185563303	8.611710212	8.29635502
8.795325869	9.629101929	8.851571051	9.581418808	10.14326599	9.118059475
9.93099102	9.679645121	9.755162434	9.818860343	9.679062616	9.646274414
10.05236285	10.14432724	9.728754818	8.595703707	9.634173323	9.39974104
10.20859114	11.00183361	10.88945836	9.574400328	10.33252085	10.43998577
14.39736901	11.5386618	11.83072395	13.26910509	13.09977851	13.92172837
8.307483899	9.39974104	9.177504333	8.909945455	8.612168455	8.980444384
8.135177152	8.031407364	7.935875313	7.242650333	7.437975364	7.795765162
14.45281427	13.76702458	14.11151028	13.94443	13.97489728	13.73439002
7.611680859	8.440878707	8.382194192	8.502509434	8.412145273	8.552719747
11.17750487	10.51400776	10.79284767	9.93187304	10.2166926	11.05033578
9.519086535	10.02161965	9.902579253	10.42175216	10.16937171	9.835081424
8.442699879	8.273483707	7.507381747	7.463713737	7.73355204	7.525850576
7.111963455	8.298396535	7.882525919	7.482605354	7.950819283	7.385541293
6.789025475	6.483130091	7.057634667	6.967457677	6.527907232	6.388351374
9.363307667	9.93663897	10.28190294	10.29752835	9.920430606	9.917043434
15.24813401	12.95787308	15.03840752	14.9847254	14.53168901	15.74256723
7.905419798	7.149343495	7.747112485	7.933221424	7.771866444	7.23423804
10.09339803	9.660200838	9.762542495	9.502644929	9.884409465	9.442729677
10.37894268	10.31795252	10.48310631	10.86173253	10.74172297	10.47941256
8.005065495	7.875022121	7.929187444	7.940254566	7.516753121	7.618249414
7.050890818	8.13932801	9.698181576	7.999737535	8.216297535	8.300103657
11.43380494	11.38577951	11.29230964	8.826393101	10.24511785	9.573320202
9.558255162	9.11211799	9.331636121	7.432999727	7.967113929	7.805446323
8.865915939	8.941795242	9.421059	9.259301556	8.130616515	8.397394545
7.948048111	9.219562515	9.276189121	9.144457596	9.271759586	9.421059
8.359263212	8.307093606	8.164575929	7.645943414	7.899682051	7.886079293
12.50328361	12.62449502	12.48124823	12.93862501	12.71520416	12.31089415
10.86963124	9.977292899	10.53623255	10.27432009	10.25038968	10.42297323
9.703591263	8.664725051	8.948168879	10.06904603	9.368875727	9.036569232

9.218466939	8.876209859	8.77085696	8.596127869	8.927565404	8.919862162
7.273507545	7.622037172	8.093860848	8.39867397	8.126700889	8.111336758
10.40701089	9.047491869	9.584100455	8.868452172	9.23383296	9.494490616
10.21484784	10.70902317	10.34193651	10.2692332	10.40592026	10.53247367
8.328992859	7.589888475	7.491688212	7.455822202	7.18482696	7.606370131
12.72610186	11.22294817	12.31719428	12.39058663	12.89513437	12.99088515
10.09254304	9.562994313	9.619644949	9.720489222	9.903372323	9.490338525
8.077645899	7.286722061	7.503692152	7.646666444	7.487078798	7.924502232
9.109760465	9.435122859	9.052474394	9.505258828	9.204345657	9.200683333
9.75438504	10.17224075	9.963504081	10.73014715	10.0672481	9.930141081
17.29704085	15.50564301	16.04217659	16.61084374	16.64557528	16.3077943
9.981718727	9.379180747	9.515069697	9.894213939	9.47593803	9.773700283
7.350388939	7.112259949	7.615524929	7.069165141	6.893606717	8.134790222
7.772177253	7.527339687	7.748187364	8.522641273	8.701109788	8.362537455
7.383097848	8.11047698	7.855731	7.803193929	7.654890798	7.718083212
7.836138323	8.619848374	8.681579566	8.715328101	8.402589283	8.149032212
9.410728505	7.31258798	7.55945201	8.180785838	8.337114525	8.118879606
10.42175216	8.598479657	9.133284677	9.472607081	9.505859384	9.340550081
8.81966502	8.558108202	8.825312061	8.984231152	9.065009323	8.884521697
8.541972566	7.741457929	7.904747051	7.936644384	8.055171202	7.909763434
8.431326525	7.310724131	8.215875455	8.196571586	7.792995667	8.180413081
7.02750098	7.268778051	7.291354758	6.966976535	6.995800606	7.204326061
8.892401576	9.077686808	8.794788636	9.003925646	8.822318	8.792768747
8.794788636	8.295436051	8.616914869	8.911109919	8.826393101	8.874191697
9.70034896	9.123297364	9.180906778	9.157435455	9.548618051	8.798247798
10.99046505	10.97322727	11.41071085	11.6157426	10.70052069	11.44491584
14.13521971	14.99939481	15.24813401	15.16803204	14.83117585	14.83117585
8.286610323	6.85163704	7.724049626	7.503050071	6.808010384	7.635907081
7.977207111	8.644542606	8.363019899	8.188779434	8.213427111	8.405355091
7.352900667	7.750590293	8.123132566	8.165380303	7.93437196	7.734550869
8.568242293	8.244805758	8.546056747	8.644074768	8.597027768	8.374363061
7.491021444	7.506434626	7.57799899	7.647964475	7.656238101	7.276277889
10.58689262	11.38775416	11.05493438	11.16281786	11.34381562	10.67081044
6.146031657	6.405475687	7.370383778	5.909380081	5.871523485	6.346474485
7.637262101	7.917722121	9.404333879	6.361157788	5.987385263	7.291703707
12.55625498	10.81423156	11.52238659	12.24649354	12.02240324	11.85301976
13.50997822	14.5108222	14.09521817	14.30038792	13.9071606	14.11997744
13.09045797	14.25895571	13.98831971	14.32804886	14.37933382	14.36070414
8.959130566	8.923576949	9.038849273	9.35796903	9.274850788	8.89604703
8.606624646	8.340252101	8.316969364	8.086953081	8.314850485	7.901561475
9.390110384	9.087255717	9.090721626	9.917863333	9.214694788	10.70534521
7.480348535	7.421928768	8.327767232	8.751405818	8.346198293	8.005371424
9.249034202	8.915814212	9.167114	9.226702434	9.534194535	8.812013889
11.7894918	11.95261936	11.38391359	11.76336308	11.5612151	11.58005536
8.429582192	7.263531485	7.533296202	7.883536596	7.713612677	7.614497747
6.933584646	6.309118495	6.141738101	5.802028253	5.979287131	5.850261818
8.530767747	9.189045596	9.283736939	9.560346242	9.569854202	9.376667182
7.544367232	8.147857556	7.794025323	7.759566061	7.441979727	7.84876403
7.944058707	7.347734202	6.949608808	7.830796253	6.767482869	7.450888848
14.56207529	14.72870348	13.7211242	13.30255942	13.40990013	14.84412351
8.377090384	9.592426051	9.426142444	9.25368898	9.242021586	9.58708204
7.157253707	6.796334212	6.27402404	6.086549182	6.584832545	6.400763333
9.802496737	9.320738646	9.569854202	9.645545465	9.725631121	9.403514808
8.811517808	9.691258919	9.430143091	9.847335909	9.587719636	9.562364657
13.40990013	14.40789433	14.04335209	14.20688311	13.95955505	14.010587
6.726701909	7.384295283	6.53354203	6.647679424	7.110215707	6.645661505
8.352134333	8.35372597	8.515350859	8.86638896	8.541125101	8.736221495
7.962004212	8.285708747	8.038354192	8.19621002	7.663954061	7.483852838

9.638947465	10.62536143	10.39968902	10.13479405	10.12610261	10.49592664
9.100524758	8.63611798	8.640789455	8.531655515	8.785177889	8.575566677
7.965660091	8.633351606	8.339926909	8.233006697	8.170586434	8.30620801
14.11997744	10.80499832	11.0387546	11.88649013	12.36759935	11.69658017
8.380307172	9.013314737	8.81966502	8.638487899	8.481851586	8.639417101
7.251769333	7.463713737	7.472500828	7.615901626	7.44049799	7.561051303
8.964085616	6.767482869	8.469779808	9.376667182	8.35667099	7.629344343
6.466486121	6.653092081	6.868352737	6.392337434	6.224978131	6.248527828
7.77977198	8.436545051	8.273878929	8.373157505	8.251125323	8.448929687
10.61867663	9.675904202	9.670530667	9.688441414	9.886023253	9.53973704
8.892970697	9.30577901	9.012031232	9.319510202	9.394567081	8.948168879
9.667708556	9.216574283	9.049629657	9.437491071	9.29496	9.453743545
8.803935202	8.701614283	8.706567556	9.310205828	8.946596606	9.20200202
6.20471398	6.962822313	7.020975202	6.881514152	6.771393131	6.618269616
7.915639949	8.076118121	7.871683444	7.610260323	7.75479003	7.88753598
9.775265525	9.011546121	10.0841781	9.058356636	9.941224515	10.3548828
7.050073121	7.03650198	7.304125475	7.313451111	7.275223848	7.276013455
6.560197424	6.881279879	7.187741232	7.303836939	7.013654717	7.413101485
8.509936919	6.652872152	6.967234434	7.377888556	7.329578283	7.645645939
6.749805475	7.286117455	7.391655768	7.016958768	7.206373697	7.28497204
7.329870525	6.823615333	6.884060404	7.122424869	7.102685081	7.052765889
7.421037465	8.73262002	8.34368697	8.456191465	8.368326869	8.684081152
10.23066525	10.66706732	10.3918566	9.877792838	10.24414187	10.03983393
10.79145221	8.78563097	9.339095222	10.08145981	10.31378621	10.20060626
7.680542242	8.348145404	8.359263212	8.499836475	8.060252263	7.66151798
6.854191545	5.990495727	6.113628535	6.679264929	6.606438778	7.130177747
8.644542606	8.50673198	8.222643394	8.29927897	8.261007747	8.35752198
11.13240418	11.60888892	11.69440164	11.51008986	11.26446827	11.32376318
6.445432434	7.40744601	7.213178616	7.142295949	7.248766182	7.066776273
9.202552434	8.385691051	8.65497299	8.665646899	8.854095293	8.447076788
9.561667293	10.60044575	10.13862753	10.23351878	9.890949131	9.969141677
10.65246846	11.65261158	11.18471248	11.58195332	11.47322198	11.24993851
8.374363061	8.500240172	9.116279576	8.532516465	9.105307717	9.748306121
8.879851283	8.433939838	8.934777717	9.187808616	8.947127939	9.02886299
8.874692596	8.624974828	9.627745303	9.779012071	9.779738	9.897493586
11.02243393	9.376667182	10.06071544	10.76609029	10.69574272	10.37467131
11.33292491	10.90606985	11.64848719	11.91416468	12.27101	11.19447199
8.399975414	7.028313949	7.167804919	7.860764394	7.614831859	8.209801687
6.56193898	6.957146182	7.442271202	7.522559465	7.370950798	7.208594677
7.937868929	7.992269263	7.745118444	7.856391758	7.745466071	7.357173859
8.830660505	7.157499455	8.833963152	8.48873997	7.122138172	9.393934131
6.128015677	6.311249485	6.433824646	6.733937384	6.498091727	6.47989397
7.300950758	6.863923949	6.945089283	6.930743303	7.146069808	6.992392121
7.744777657	7.306475687	7.851263121	7.788532485	7.537817576	8.13932801
8.894948323	8.951989212	9.143279535	9.237064687	9.387059283	8.470647758
6.753469455	6.892837172	6.83799099	6.910411354	7.120197273	6.733232384
7.417927455	7.587331949	7.813018717	7.689234404	7.59656001	8.037287293
6.881279879	9.154469939	8.964085616	9.291911616	7.692217293	7.263859848
7.85507	8.721523222	8.563501616	9.252443646	7.774939586	7.421647414
7.494798263	8.863920758	8.616405606	9.004940778	8.215082485	7.220481808
10.56207051	11.09565519	10.1597097	10.49830212	10.72127155	9.89348604
8.038354192	8.706127333	9.004398101	9.296131263	8.498949848	8.890936242
10.53028115	10.64005918	10.39641485	10.55726192	10.89912578	10.19649023
9.603529667	9.735412273	10.5104744	9.878686616	10.57159251	9.849671929
10.98749259	14.13521971	12.3774088	12.3774088	12.89113428	12.54589185
7.516753121	7.676615465	6.999830061	7.112620848	6.789537253	7.357456071
7.02612702	6.985120646	6.772767788	7.324298343	7.610976808	7.159453505
7.289658101	6.160317818	6.531486374	6.366826091	6.280283596	6.35978

7.760294414	6.942817626	7.129030091	7.222960121	7.129307667	7.098484101
9.501306556	9.580783667	9.638309758	9.523397737	9.93663897	9.445197818
10.95234717	11.47496104	11.25898056	10.94496034	10.94496034	11.10035391
8.112876232	7.090449141	7.641344242	7.818322879	7.323124384	9.172421828
8.808928141	8.51269104	8.69723302	8.414282596	8.662363172	8.904793061
11.01683173	10.58812248	10.71613341	11.18941159	10.74945851	9.751885293
7.859670455	8.114219303	7.752687697	7.477877475	7.641344242	7.906425364
7.163287778	7.206373697	7.339519202	7.545628697	7.36076402	7.741148566
6.421576576	6.169649212	6.503851576	6.390148364	6.075089566	7.409045616
9.848227263	9.864614818	9.532335293	9.46304199	9.631972414	9.388273121
10.73264046	7.954205616	8.760291222	9.572453101	9.726443586	9.195011616
9.257324	8.794788636	8.955880859	8.63794701	8.870056939	8.561789232
10.78551178	11.4481816	11.07956266	11.24993851	11.72592113	11.12151875
9.476588061	8.829514889	8.649462798	9.036569232	8.732044667	7.645943414
7.054238101	7.793372141	7.223533071	6.986217889	7.622357354	7.93888704
10.81034167	9.197870838	9.665316566	9.900778232	9.505258828	10.05970796
9.657223606	9.296131263	9.45706402	9.242021586	9.074692404	9.29794999
7.886480404	6.637264	6.892562737	7.155513182	7.286993737	7.002659525
10.33989796	11.0940021	10.56908161	10.73661507	10.67305197	10.40278823
6.211158404	6.977291071	6.266099303	6.48207598	6.714232707	6.426632697
9.895856061	9.924448444	9.582110717	10.687913	10.47123164	10.53842125
8.220225172	8.010919919	8.344860929	8.173633596	8.376150616	8.129067798
9.671912455	10.27035429	10.4429644	10.00886723	9.948421707	10.02549055
7.800624061	7.864861071	7.785940475	7.319114313	7.484183869	7.566450929
8.661956242	9.670530667	9.584803364	9.203189636	9.156840283	9.570470929
7.779426687	7.859293	7.824090626	7.616841162	7.50339202	8.051625212
8.560860505	8.760676242	9.317615606	9.472030465	9.007843273	9.879566364
7.525015414	7.104930788	6.420787636	6.465158313	6.745348657	6.632101727
8.465000141	7.512168657	7.991026727	8.081502051	7.914965525	8.411285283
8.914132333	9.283736939	9.348735909	9.553430788	9.379720636	9.436357141
10.15186663	10.00094823	9.547339828	9.97079498	10.41530743	9.781348374
6.464252303	6.944866586	6.711662182	7.03650198	6.614759768	6.901187545
9.124970051	9.742895778	8.283707475	10.17122401	9.033915556	9.609756293
8.192645727	8.612168455	8.104500051	7.935468505	8.049019253	8.047933768
7.457936747	7.243557061	7.163563677	7.178633636	6.883793818	7.282945384
10.71860391	11.04156144	11.31449746	10.97322727	10.96748398	11.54053762
13.01016522	12.70002263	12.77454263	13.36582475	13.39659876	12.77015535
8.622644364	7.515186091	7.655968232	8.551311374	8.423139949	8.183602061
7.55847901	8.30620801	8.280295667	8.222223545	8.127495596	8.161911788
10.53623255	10.09506496	10.20358283	10.44712342	10.16642595	10.33476777
9.821914929	9.428845576	10.0188097	10.0728998	9.827596687	10.41626133
13.26276274	13.66784462	13.23341018	13.20536123	13.30255942	12.80166365
9.835081424	10.52904685	10.61480751	10.22951336	10.18563382	10.26116661
16.64557528	13.85817003	14.88238784	15.59877501	15.86132141	15.05177986
6.953378071	7.593349828	7.869396606	7.869709071	7.603389051	7.686444293
10.6820228	10.28899471	10.31795252	10.0060683	10.26541026	10.03385507
6.704711566	6.951961606	7.708922879	7.188612899	6.983707354	6.796334212
8.458294828	8.274698687	8.63611798	8.995584808	9.062782333	9.255548899
6.661293222	6.413438525	7.197763283	7.704713596	7.316406697	8.12949599
8.402589283	8.462236515	8.104866828	8.252794354	8.509065354	7.898974697
8.272426096	8.360024717	8.515864333	8.727989172	8.760291222	8.393536232
9.054276	7.69185196	8.57597697	8.662816202	8.412578899	9.384699788
5.970980293	6.531968667	6.561305909	6.748092424	6.718530879	6.48058603
8.254082232	7.576363505	7.668279394	7.875022121	8.003479939	7.265354444
11.21493335	11.88909817	11.70138789	11.92665931	11.42178201	10.687913
7.007051354	6.411067717	6.549596111	6.634635343	6.451688303	7.028809465
8.059832677	7.660150727	8.178694051	7.991026727	7.763504859	8.031407364
8.650384111	8.681067818	8.235056909	9.001272616	8.979298788	8.807484626

9.363823202	10.83790729	10.70299608	10.78987961	10.46442497	10.53505365
6.953914061	7.16240796	7.427372788	7.682588111	7.504860717	7.70365599
6.666656929	7.545986354	7.400588576	7.730484909	7.589604303	7.263531485
8.089360869	7.199434576	7.59785203	7.485191121	7.381950828	8.212243444
8.158321101	8.364710758	7.828584192	7.785214909	7.618249414	8.710842768
6.467159949	7.068375576	7.379773919	7.491357222	7.140588444	6.430261071
7.748187364	7.000811505	6.938883283	7.086698091	7.214035101	7.627030141
12.20960187	9.232683343	10.00186072	10.40486524	10.21157382	11.2608846
9.899010949	10.16073454	10.18666124	10.40074116	10.5752557	9.658049545
8.78563097	8.946596606	8.650384111	8.928157121	8.708995576	8.527598192
7.619556354	7.722297051	8.064260859	7.497675424	7.594027929	8.249809909
10.02923217	10.6240143	10.46664928	10.06450841	10.62536143	11.18291631
8.880853535	8.222223545	8.076118121	8.152443121	8.236633323	8.297630758
12.51066587	10.05053825	10.11849919	10.46232362	10.36465285	10.3810842
9.112697707	8.78214798	8.993505475	9.529469889	9.661659101	9.842388081
8.029383646	6.810023535	7.650858424	6.815473091	6.875911141	8.672881101
11.21187068	10.78987961	10.96886286	10.69682519	10.9102587	10.91903182
8.534897646	8.027386222	9.279768394	9.211184727	8.388388636	8.216823323
7.096649162	8.201303758	7.822322859	7.813018717	7.614146707	7.817254242
9.907546465	10.59685822	10.45375986	10.23948175	10.29000276	9.981718727
10.58812248	9.749660394	10.24511785	10.38430498	9.926784556	10.41846051
7.489478697	7.729749768	7.692217293	7.147201253	7.449616394	7.138578667
6.60386102	7.561051303	8.110083838	6.844493475	6.752974364	7.323994646
6.430261071	6.284133697	6.378366909	6.515007141	6.501886182	7.495424616
9.621550919	9.846508495	9.79615097	9.433849505	9.318836889	9.652939283
7.528004313	7.913459869	7.303307	7.358375414	7.385218303	7.050347636
11.84736958	12.13867659	13.18348162	13.05987966	12.34577038	13.81935116
13.76702458	14.1830221	14.15863941	14.25895571	13.66784462	14.03424604
8.465492313	9.238221677	8.745474636	9.290785283	8.938439909	8.745474636
7.957018646	8.45577798	8.375190152	8.559011545	8.058246657	8.321919424
11.94745099	10.9889639	11.24323027	11.76101506	11.79870316	11.17213305
7.771540485	7.886480404	8.101780545	7.806845253	7.920877394	8.105629576
7.318216242	7.98690202	7.84419497	7.882850313	7.68569196	7.819446808
9.577319545	9.113305192	9.238221677	9.689783586	9.294355394	9.056533626
6.788765101	6.660321343	7.103815788	6.857008404	6.719039212	7.02612702
12.24933389	11.49239347	12.06692054	12.37416009	12.11216258	12.7939548
9.748306121	8.910531141	8.998971485	9.216574283	9.082448343	8.972927475
9.910960495	9.817379515	9.576136707	9.418269535	9.374690616	9.516398909
6.676440081	6.822217707	6.97260104	7.374563525	7.195224778	7.211210626
9.484840202	9.533596364	9.782133848	9.864614818	9.513164303	9.66831603
8.236633323	8.449722687	8.680072242	9.027136657	8.626833051	8.874692596
9.007167758	7.767702525	7.972411364	8.06745399	8.55725397	7.222960121
11.20660223	11.02700786	10.69354222	10.90057618	11.2608846	11.02403513
5.916980778	5.754487242	5.775076687	5.900311293	5.946203788	7.179445253
9.465659495	9.992988556	10.53842125	10.5029511	10.57758511	10.45996909
11.17378617	11.70138789	11.71443529	11.34381562	11.17571904	11.40725584
7.117115202	7.322217273	7.21289504	7.630295798	7.147872253	6.447869455
6.949335212	6.483340232	6.137175152	6.135075747	6.091285889	6.358115374
9.221294354	8.559456798	9.537497323	9.312041414	10.20358283	10.61480751
7.964179182	8.19458801	7.985458788	7.651926253	7.444173596	7.759936788
11.94234713	9.237664879	10.08521343	10.89912578	10.63014735	11.82112126
10.82153564	10.62142174	10.55966675	10.92060078	10.74305691	10.01340346
10.46108665	10.56421743	11.05796986	11.14707085	11.0940021	11.32559397
8.039790061	7.872350636	7.894965162	8.228066545	7.996393263	7.845242687
6.332645192	6.225547869	6.868107182	6.594143485	6.594580697	7.067324051
9.789131121	8.992473788	8.909945455	8.806957455	8.741130788	8.923097444
8.62991701	9.702015293	10.65014074	7.026621727	7.133813545	8.417604414
11.51008986	11.74935785	11.38970236	11.62739451	11.5737214	11.49420288

8.482406475	9.321890899	9.015412101	9.009407333	8.310875455	9.161459798
9.843921515	12.05813886	11.40306525	11.38970236	11.37847359	11.37670858
8.020484273	7.490707535	7.624735818	7.636934919	7.694191859	7.443192556
8.704708384	6.77624102	6.59504903	6.787032949	6.71121296	6.864210101
7.188044747	7.121885657	7.072869879	7.433328091	7.203788636	7.462491444
7.950819283	9.104371838	8.869521111	8.851018515	8.676791091	8.927565404
6.803094364	7.988582505	7.781852747	7.579350515	7.710917071	7.500501152
13.23341018	13.81266894	13.89331098	13.58209631	13.2158469	12.97977545
10.56908161	10.85100254	10.63014735	11.01951417	10.85654613	10.37573078
7.506099556	6.513659232	6.556900818	6.988399394	6.707366768	7.452458778
7.60282502	8.865915939	8.041792596	8.718769576	8.349393212	8.123471404
11.03452834	10.97767761	10.48074239	10.18942995	10.4549623	10.8947391
6.46039499	7.108809091	6.184687263	6.223508929	6.320458162	6.352434697
7.728361283	7.802503596	7.578628212	7.372238061	7.159147152	7.367866364
8.885078273	7.937868929	8.173633596	8.181963182	8.263866778	7.865908465
8.33273403	8.239474646	8.695776131	9.352487919	8.854603364	8.607126586
6.634356909	5.976543899	5.941698303	6.000571626	6.384310424	5.93490399
7.267388222	8.096525737	7.792995667	8.179581869	8.433468657	8.115023838
6.622014	6.165477899	6.030805626	6.881279879	6.487932242	7.086157636
12.43279956	9.038261515	9.901640636	10.59809845	10.39968902	11.60069924
8.677707162	8.409136758	8.740195303	8.537956091	8.929972798	9.191263899
7.433328091	8.392711081	8.40783399	8.084275677	7.660150727	7.975784071
7.342557798	6.806487737	6.325549091	6.801564111	6.406575848	6.365147848
12.42233701	11.52645916	12.16627503	12.56315114	12.34237091	12.34855768
9.225515485	8.415109929	8.66101001	8.874191697	9.008373061	8.385303111
9.224923687	10.23850383	9.971733293	9.814415596	9.670530667	9.874496818
7.510027525	7.246699182	7.527074182	8.302062758	7.876807081	8.602379626
7.16240796	7.656877838	7.30556202	7.372532788	7.218399283	7.69185196
8.833337414	7.87816304	8.833337414	8.708467242	8.35372597	9.257324
9.433849505	10.41426339	10.18291229	10.41731261	10.65871503	10.20634509
7.287262414	7.209785606	7.212087616	7.344882899	7.033365899	7.465665798
6.500607838	6.662284414	5.769634793	5.787115051	5.766310808	5.781597485
6.921167192	6.908606212	7.113720848	6.908354899	7.21150504	7.236271343
7.869033162	8.399975414	8.941795242	8.997224192	8.932093424	8.316081222
7.314300525	6.934295525	6.860179071	6.909401626	6.852936051	7.126269081
7.244122677	8.203151141	8.261781515	8.364284556	8.121211444	6.626438091
8.110083838	6.563630475	7.000811505	7.629989283	7.409335747	8.533396525
7.617890202	6.843046434	7.19832496	7.676257364	7.150169929	8.150892606
8.85518403	7.623059667	7.779041818	8.444861909	8.149448323	7.923778798
9.582761798	8.900236364	8.809442889	9.068490586	9.054276	8.941359636
7.670568384	7.574489596	7.663954061	8.121954505	7.423442323	7.597232061
6.814975071	7.476255091	7.013137848	6.897441	6.810861495	7.096649162
12.23505088	11.0473733	11.11856721	11.21958266	11.6157426	11.08936677
13.78737352	14.02572128	13.95167504	13.81935116	13.54221797	13.56234785
9.828404283	9.694213162	9.541041677	9.649399939	9.300367869	9.712341424
6.610225071	6.719552727	6.042010263	6.296876485	6.216046091	6.946968899
6.316919404	6.762568667	6.638362242	6.454943192	6.581970424	6.655749121
8.015705778	6.632781101	6.925728848	7.192707293	7.012842909	8.686126354
8.002342131	8.362142131	8.540635232	8.776574232	8.614861051	7.960192616
7.580594545	6.797074616	6.975683768	7.014842626	7.078303869	6.878694758
9.458377051	9.319510202	9.558925838	9.638309758	9.096395636	9.489614919
9.713188212	11.3290504	11.04886125	11.0387546	11.22119175	10.42927797
9.365016101	8.787164939	8.835493283	8.862785404	8.986932636	8.715328101
10.10542249	9.677492576	10.18942995	9.968215758	9.932690333	10.00186072
13.58996905	14.37933382	14.223913	14.19920898	14.28325373	13.85099976
7.314926455	6.921417374	7.401750182	7.401155333	7.108229697	7.929593313
8.658285303	8.222643394	8.402589283	8.422204465	8.599954677	8.226500707
10.18563382	8.896578162	10.09254304	9.829119768	9.62024901	10.41957053

7.661155364	7.456429202	7.310724131	7.794025323	7.190644495	6.859461465
9.584803364	9.761765848	10.02161965	9.480384485	9.656571869	10.32531651
6.654772152	6.260672758	6.242654081	6.360934626	6.509391576	6.951745222
8.85785699	7.678291495	8.352134333	8.546056747	8.358418384	9.260036485
8.852576737	8.439612798	8.265565879	9.457741556	8.787164939	8.724013404
9.076382242	8.758364879	9.026117657	8.664254273	9.030619364	8.967895455
7.353900202	7.193571	7.589252838	8.061425949	7.658441616	7.682588111
9.435122859	11.14866374	9.482964828	9.672623929	9.746145949	9.974493182
7.782462626	7.318539616	7.575132364	7.84876403	7.296570525	7.60282502
8.018723424	9.133918929	8.882379495	8.507703202	8.757405333	8.799825313
7.579350515	7.550195808	7.477539293	7.300653717	7.512168657	7.489478697
10.8193093	11.28207759	11.32721575	10.91514001	10.94640834	11.0940021
11.07797425	10.61621963	10.61743608	10.27035429	10.60383217	10.22267877
7.907924051	5.890469384	7.851640596	7.649926505	5.909195384	7.571747222
9.126080354	8.458294828	8.551311374	8.739635162	8.710391727	8.721926778
6.868868606	6.948610667	6.70156496	7.010749455	6.477093667	6.76459802
13.46005067	13.49745762	13.45466248	13.18348162	13.32669092	13.40302459
8.681579566	8.194244232	8.403930677	8.361756343	8.490531303	8.251125323
10.19957169	11.01807842	9.598265626	10.37346225	10.5336638	10.23850383
7.600615697	7.630934212	7.608934747	6.934568707	7.846688253	7.612294131
7.222960121	8.345353354	7.360451687	7.496050374	7.876138384	7.445432899
7.291982576	8.044191424	7.575754657	7.923778798	7.910120242	7.411268949
9.535633242	8.34368697	8.380307172	8.575095707	8.698751323	8.403471909
7.762120596	7.702676091	7.546592828	7.717737636	7.491977768	7.788532485
11.07158908	10.48432644	10.56655729	11.04316622	11.05493438	10.32635032
14.85646221	15.86132141	16.68963031	13.07947925	11.3290504	14.37933382
10.09426568	10.85782556	12.36473556	7.841440677	7.338325414	9.636929838
6.303290121	6.36070104	6.072768485	6.278274667	6.455374485	6.596206051
7.483574061	7.026621727	7.369172101	7.865549818	7.718373848	7.854685616
8.277405556	9.061649657	8.945002717	8.933684374	8.752460404	8.729972586
10.59924244	9.876880455	10.03123363	10.1300726	10.1838089	9.819688798
10.54565144	11.11230451	10.60044575	10.89224764	10.70534521	10.64502212
7.919201576	8.404396899	8.372433475	8.134790222	7.982067081	8.176718545
9.850553616	10.30569114	10.2752886	10.15585749	10.08700287	9.888393747
7.822322859	8.299695162	8.133179586	7.962004212	7.929905606	8.100222556
9.380979222	8.878776172	8.753420071	8.813595202	8.944409727	8.471953717
9.446464909	8.76386996	8.698751323	9.328711051	9.149195859	8.661508737
11.6295292	12.63888703	12.3613163	12.32364634	12.19398134	12.25572441
8.881793333	8.432612263	8.351407394	8.515864333	8.657806242	8.578858313
11.68948846	12.10414281	12.02514953	11.99323436	11.87445957	11.68948846
8.99408802	8.797743222	8.788663253	9.008373061	8.664725051	9.048620525
7.98506802	6.919943293	7.282635596	8.894948323	8.43006898	9.363823202
9.731806091	9.797856576	9.647013263	9.946747141	9.66613299	10.03123363
9.080123545	10.24038579	8.984231152	9.512169838	9.497084828	8.921011566
8.334162556	7.821271606	8.01423197	8.700142556	8.318250424	7.998587576
10.32635032	10.98492733	10.70643744	10.98627861	10.89755671	10.55070525
8.591239465	9.510536	7.990313949	8.443175586	8.537544354	8.120062485
8.756886111	8.386133566	8.075284384	8.707057788	8.319901444	8.366462404
8.971160737	9.909202788	9.802496737	9.501970121	9.606298465	9.596773313
9.338458455	8.665646899	8.718321818	8.473392556	8.803935202	8.426780919
8.715328101	8.157153081	8.305359949	8.493155899	8.41382397	8.119288848
10.27905981	11.27499716	10.76609029	10.67572435	10.61867663	10.81423156
9.071276071	10.58579258	10.48782122	10.53028115	10.57028524	10.75600216
6.198948818	6.122552818	5.941390505	6.395789414	6.154487859	6.629471747
7.307562737	7.634226727	7.310482798	7.556302505	7.538797101	7.827906879
6.505895828	6.302320576	6.543222263	6.735404798	6.640209303	6.559042364
10.49830212	10.49348589	9.21352099	8.571676263	8.686126354	10.5029511
7.720869455	8.480536717	8.371229848	8.329784131	8.198176101	8.170129495

8.674281929	8.316969364	8.165380303	7.886480404	7.960192616	8.248986687
6.500836152	6.854191545	7.170232747	7.220481808	7.213710414	6.951169364
10.36239309	10.5041569	10.73384189	10.34717129	10.75453103	10.34514895
6.725993303	6.587993828	6.867411697	6.746600626	6.714999636	6.851905687
9.122138505	9.787586646	9.788274313	9.713188212	9.810255869	9.710054737
7.342871717	8.205133263	7.791244131	8.183195545	8.715845505	8.186741323
10.18000397	9.75438504	9.90675497	10.3986043	10.3986043	10.27432009
8.13267903	9.21905796	8.500240172	8.686126354	8.959130566	8.837022465
8.791137414	9.023639636	8.633351606	8.41382397	8.747892899	8.811517808
6.777231859	7.361082222	7.466342606	7.825104404	7.724673141	7.299074768
7.916391202	7.264201869	7.730154939	7.169469697	7.76948301	7.622037172
16.14978837	13.23341018	14.20688311	14.79546777	14.99939481	14.63205333
9.790672212	8.164575929	8.441767061	8.961327374	8.878776172	9.698181576
8.373514202	8.813032152	8.967408879	8.616914869	8.805907646	9.35067002
8.418118394	8.555833323	8.504327141	8.266842545	8.492747404	8.580231323
7.609232899	7.357456071	8.182386152	8.090488788	7.857447394	8.39227803
10.09896607	9.994772242	10.83223099	10.7442872	10.66945215	11.01045685
7.907575465	7.760294414	7.331457121	7.224700556	7.12322297	7.506099556
8.10326497	8.655437626	8.242002535	8.572701232	8.144161414	7.763846354
8.626833051	7.904381697	7.856753939	8.321152475	8.168196313	8.925792808
8.943348051	8.384837869	8.222223545	8.459596535	8.449337667	8.451550535
6.345850475	6.19263099	6.456009747	7.041650374	6.723255293	6.959934293
7.277222202	8.831201768	8.066736566	8.141770949	8.088117667	8.287081333
9.984347768	9.513795939	8.248137606	9.271759586	9.439917707	11.18471248
8.268510495	8.933180404	8.851018515	9.17294002	9.078331919	9.233272283
7.120197273	6.977041091	7.236914141	7.782462626	7.550195808	7.248166182
9.624391323	9.331636121	9.927686616	9.166446616	9.297353606	9.616741535
8.287438535	8.464558212	8.262181081	7.911309091	7.981709394	7.960941889
17.67797782	16.04217659	16.90924948	17.36803328	17.36803328	16.7804214
10.16355358	9.852239606	9.903372323	10.05607537	10.19757035	9.852239606
7.561964414	8.302498081	8.727989172	8.53579602	8.325316889	7.489812667
8.017104828	6.469635131	6.543419131	7.090449141	6.972319545	6.842028788
6.71730499	7.286424495	6.92551101	6.666381515	6.804991424	7.118793697
7.430512737	7.559825556	8.006153172	7.775998535	8.022720303	7.725042051
9.35371997	8.810446485	8.815152444	9.256085253	9.008956374	8.675789182
10.48897537	10.61480751	10.71390019	11.11856721	10.77252405	10.77507269
6.285113455	6.813963929	7.541750556	7.496977949	6.860179071	6.933815889
10.89912578	10.41731261	10.2761618	10.33696433	10.42062148	10.14131493
10.12126634	9.446464909	9.606298465	9.987077505	9.532335293	9.752732475
10.62764398	9.493547687	11.43208235	10.59924244	10.77890896	11.4090051
8.096910323	6.880472354	7.101049919	6.988144556	7.192707293	6.781955545
11.14057221	10.22448985	10.54078574	10.62280049	10.65987528	10.61621963
6.246552232	6.322511848	6.527189616	7.318843495	6.88247998	7.142620404
7.234805667	6.981086404	7.277849899	7.434501333	7.363288879	7.527697455
6.286870242	6.002391646	6.156509616	6.475338556	6.696473434	6.408336172
7.66151798	8.312729141	8.134790222	7.831138545	7.84876403	8.115437899
6.617567333	6.58529104	6.421175192	6.917762192	6.936369818	6.640921556
8.652374404	9.513164303	9.50650501	9.521192909	9.534883657	9.455015404
7.323648515	8.114611232	8.005065495	7.593664717	7.500795283	7.603389051
6.620195253	5.828275323	5.980732253	5.785572697	6.256680889	5.836536374
10.47479655	10.5752557	10.81280665	10.44094498	10.30695028	10.26632574
7.250238485	8.65914896	7.832540091	7.966402293	7.979449616	8.083485404
7.653305051	6.668303455	6.841827051	6.797589061	6.866319192	7.397737788
9.376667182	8.687541707	8.852576737	8.744562374	9.014846778	8.708467242
7.928481586	7.335252788	7.424927172	7.511581475	7.51310204	7.186960111
10.75987116	12.39660011	11.40495249	11.59427705	11.58421758	11.70557621
7.028809465	7.351279444	7.141446111	7.487351192	7.17606597	7.275765808
11.51867768	9.581418808	10.35284213	10.71279176	10.45754412	10.90057618

6.851905687	7.304125475	7.341966626	7.336756222	7.09353204	7.148192263
8.249350414	7.825104404	7.582311101	7.797844434	8.065602475	7.963030121
5.749016939	5.869263475	5.810481848	5.931956253	6.027652303	5.753828808
8.234232515	7.724049626	8.136235949	8.221794202	8.073294879	8.295436051
11.73710477	12.24097391	12.53911948	12.69676846	12.2293359	12.16627503
5.845624949	5.865169354	5.901094202	5.817411	7.591751808	5.889266646
5.846700859	5.94215104	5.981573081	5.903924828	6.284543212	5.885687576
17.58093558	15.74256723	17.12376032	17.12376032	17.07010739	17.29704085
7.204042263	6.449932949	6.539153576	6.541618313	6.303290121	6.542753323
10.83223099	11.63837311	11.72807534	11.16888788	11.2518391	11.37131374
9.038261515	8.481851586	9.536330707	9.40167604	9.031155364	9.078331919
10.5152666	10.29208493	9.805520727	10.74172297	10.35284213	9.984347768
9.660200838	9.08959502	9.873591818	9.711723394	9.742895778	9.993897848
10.57649171	10.93171462	11.20498985	11.43773109	10.92337564	10.7442872
11.46724679	11.02243393	11.36979611	11.22947614	11.12315281	11.13879804
7.200432616	8.341977727	8.054318061	7.626793707	7.859293	8.06213903
11.64637724	8.389653889	9.094691273	9.990505182	9.945028121	10.00985275
10.37467131	10.67190595	10.55070525	10.35385551	10.61371787	10.39414857
9.490338525	9.570470929	9.559682182	9.86223396	9.598893818	9.899969596
8.749910828	9.230202616	8.938439909	8.824381182	9.036569232	8.565878434
8.383846919	7.980934222	7.735250384	8.221010253	8.091725323	7.586712596
9.512482222	9.481000434	9.636929838	8.932594273	9.215946081	9.265583747
7.369172101	8.213832515	7.361082222	7.296277414	7.88825004	7.407756293
9.209526556	9.94767803	9.455015404	9.08959502	9.296131263	9.263851586
9.849671929	9.557589465	9.779738	9.491725303	9.302735434	10.17030341
10.04421951	9.172421828	9.241421747	9.341818545	9.586238192	9.153232364
7.322572717	8.230996848	8.200163707	8.078389566	7.856753939	8.046433182
11.35080354	11.15666056	11.08107282	10.77103399	11.11081747	11.0387546
6.143547697	6.444612172	6.874731313	7.014205707	6.818245747	6.615401818
12.51766186	13.13007894	12.66627862	12.15440913	12.41337695	12.68097339
12.57877126	13.00581854	12.7452022	12.14507673	12.45390404	12.35168631
13.72760196	13.47832004	13.52334642	13.14611959	13.50411017	13.14611959
12.18142186	9.859664869	10.91766747	10.74305691	11.07956266	10.86963124
6.651524121	7.740855141	6.752974364	7.479706475	7.001104828	6.944866586
7.030426444	7.572856394	8.012346152	9.318836889	8.441767061	8.214678465
9.634173323	9.061091758	9.005433152	8.736221495	8.906910121	9.008373061
8.717364515	8.779146061	8.912003131	8.726978374	8.786134101	8.666601818
10.41022215	9.895856061	9.645545465	9.503271071	9.794631646	9.486974667
8.576430182	8.32400903	8.323157747	8.656835556	8.168969465	8.939019091
10.63391466	9.702758343	9.942152202	10.25422487	10.06822268	9.602761333
6.827413455	7.188044747	7.363924808	7.072869879	7.067591	7.187484444
9.992988556	9.984347768	9.457741556	10.09426568	9.857940545	9.420326576
9.881176566	11.6157426	11.99323436	11.5612151	11.64044206	11.96636849
7.688370495	8.436995596	8.057864374	7.792995667	7.782462626	7.898205273
16.12384164	17.01900663	16.96059838	16.52993947	16.57174008	17.01900663
9.749660394	6.944336869	9.869466131	9.724814657	7.167804919	10.28795108
10.22951336	9.911926424	10.36032821	9.923658606	10.35809493	10.84974643
7.972411364	7.74961303	8.585303495	7.964914919	8.295839707	8.550897414
7.527697455	8.420387707	7.931306778	8.434412929	8.379056919	8.076118121
9.97079498	9.456392222	10.49140257	10.29208493	9.980879424	10.63014735
8.057421596	8.063898495	8.830117929	8.741130788	8.327767232	8.848914293
7.428304182	8.124973859	8.056742909	8.047933768	7.727390434	7.820222687
12.01956714	13.44126502	13.44849295	13.2504616	13.33155169	13.28559105
11.16888788	10.573928	11.1060681	10.85654613	10.78705857	11.03296099
11.81221198	11.77552743	11.73070023	11.56725755	11.54470354	11.41415047
13.76108056	14.5413574	14.5513462	13.9071606	13.96779242	14.24162707
8.94874	9.242526283	9.458377051	9.125507586	9.376667182	9.368224626
10.88031852	10.3380268	10.37215733	10.03492192	10.09161001	10.07479617

8.347358778	8.572701232	8.522641273	8.326884343	8.027802949	8.230181798
11.36979611	10.22164118	10.86305205	11.18096106	10.79284767	11.92665931
7.372532788	6.55006301	7.622197263	7.46337499	7.444499747	7.752360667
6.592903869	7.09353204	7.970926879	6.102491535	6.043588101	6.529524889
6.710494808	6.694222899	7.145493505	6.66391404	6.485516717	7.519695475
6.935285707	7.278115626	7.334908263	6.917518081	7.091067131	7.342871717
7.805068949	7.898974697	7.738299929	7.622739535	7.901917293	7.362680232
8.481851586	8.718321818	6.605935465	6.809755232	6.878445485	6.622014
15.18392271	11.16281786	12.57877126	15.15443825	14.9847254	15.27762024
9.126663535	9.396536798	9.29496	9.372705202	9.197870838	9.135096212
8.53579602	8.01013803	8.750392838	8.421742051	8.128726778	9.400967465
9.67665103	9.833438364	9.711723394	9.324388263	9.491039162	9.584803364
8.744562374	8.017104828	8.528915121	8.793339475	8.47014499	9.014846778
11.71443529	12.58957491	12.29886409	12.53911948	12.25251148	12.30465105
7.603101596	7.591105343	8.016751283	7.97753898	7.81867298	7.832540091
6.285312596	7.351945465	6.191632212	6.289939313	6.215353354	6.04559901
7.656238101	6.96124304	6.808010384	7.122138172	7.08136002	6.873999848
10.68072859	11.63629658	11.77305263	11.77053658	11.51008986	11.73271713
10.84568098	10.72246175	11.11230451	11.02105469	10.86820493	10.96748398
9.704210374	9.569063343	9.053706202	9.644739414	9.423636879	8.991970091
10.44832821	9.927686616	10.10365156	10.02061657	9.493894545	9.675904202
10.22865659	9.370832535	9.325601172	10.09597734	9.370225242	9.468134182
10.29208493	7.944736172	8.233421293	9.382205394	8.897751606	9.867106131
15.26374094	11.80554878	12.75784116	14.02572128	14.45281427	13.48401144
6.410100141	6.871158202	6.927522051	6.777231859	6.86140099	6.800063838
8.580231323	9.123926859	9.292435667	9.161011869	8.966940586	8.628208788
8.627322343	8.501579646	8.852092202	9.046362323	8.903602263	9.315088131
7.733842657	7.688677182	7.56409603	7.881467646	7.876418838	8.363862202
11.26990549	10.4687107	10.4655823	10.68960982	10.87637444	10.4322159
6.113305919	5.867980414	6.053149253	5.909779	6.182782414	6.156655838
7.148452788	7.457359414	7.502411182	8.032957859	7.985767212	7.784199556
8.014589606	7.82475102	7.638981707	7.162148556	7.487351192	7.414093131
8.253639283	8.479727162	8.800782677	8.734806657	8.498167798	9.232683343
6.982909788	8.208692758	7.656877838	7.700334879	7.57799899	7.660499434
12.23769992	11.13713447	12.78185519	12.10414281	12.41634374	12.95303817
8.804477505	7.119314485	7.919797354	8.175629323	7.817975838	8.001277303
8.035288182	7.735579636	7.623693616	7.525015414	7.870356606	7.555661677
8.947127939	8.217984283	8.045324071	7.626470505	8.029383646	8.19621002
8.980943495	9.217871889	9.716648152	9.010430586	9.619644949	9.249034202
6.227319707	6.355365798	6.42554104	6.825072616	6.434478081	6.770888697
7.280525364	8.434806414	6.547344253	7.259665485	7.273224596	6.949065
11.04316622	10.78408516	10.95093974	11.09721747	11.10170724	11.50632759
7.116047475	7.613240606	8.167813576	7.940978939	7.837507091	7.811684818
6.618511313	6.964119424	7.194433949	7.296570525	7.107702061	7.088250616
8.739178636	8.460488909	8.863920758	8.886096818	8.700606657	8.645985424
10.1684149	9.107945202	9.604846424	9.450568111	9.473962505	9.447991384
8.63611798	8.996667384	9.332175414	9.629101929	9.580783667	9.277455131
8.187136596	6.257467596	6.637907929	6.518333111	6.559042364	6.71142402
7.842359758	7.656544636	7.936644384	7.977207111	8.034450929	7.359287172
7.653651778	7.444173596	7.588938485	7.942409707	7.759260374	7.584965596
7.645321707	7.085030667	7.110215707	6.96545202	7.023680051	6.94072599
8.446184828	9.533033172	9.145078374	9.46096901	9.186096828	9.184527788
7.377557566	8.318250424	6.760600303	7.624016909	8.095746162	6.379570131
9.873591818	10.53505365	10.29208493	10.54692936	10.68960982	10.08893073
9.486281202	10.32750324	10.3986043	10.63014735	10.84974643	10.32309273
7.728044798	7.011036525	7.175476838	7.482291222	7.267388222	7.527339687
12.25251148	11.13085117	11.92396137	11.61117883	11.28207759	12.81735602
13.78027417	9.804809051	12.03068847	12.43279956	11.96636849	13.85817003

6.682819434	6.919083737	6.631857323	7.009733586	6.914843414	6.718038374
15.67809237	14.223913	14.74028573	15.19830883	15.21350605	15.29549959
9.867911152	9.250125475	9.064430111	8.692870869	8.848410616	8.520243879
9.169326323	9.528804131	9.200042253	8.962901121	9.103235919	9.04056096
8.84935699	9.023071899	8.770297859	8.285708747	8.690381253	8.484723788
9.155079848	9.285000091	9.463745455	9.106823152	8.967408879	9.037616626
9.576136707	10.00450786	9.67665103	9.966143818	9.94767803	9.766131535
9.755162434	9.932690333	10.59180142	10.58812248	10.25422487	10.75178718
11.34732253	12.51424883	11.93770961	12.32024961	11.89628941	11.30301738
6.377923929	6.537516434	6.985416596	6.689933697	7.238429162	6.768970657
6.281203727	7.389870293	6.691055667	7.028039657	6.80697597	6.714999636
9.162016475	9.280885222	8.950355889	9.262561	9.474605101	8.765765323
6.914278354	6.48207598	6.454504667	6.231060354	6.561106071	6.511217374
8.041000495	9.380344141	9.366312899	9.513795939	9.203760495	9.242021586
7.885731566	7.784529071	7.731871869	8.150448232	7.850180343	7.603101596
7.202978657	7.078018687	7.394691081	8.117339677	7.345267404	7.658148485
7.537489808	7.358075697	7.659822475	7.588938485	7.593349828	7.331138333
7.247290424	7.249962576	7.738613697	7.319616859	7.486457414	8.003147788
8.877626323	8.003903253	8.216823323	8.205892515	8.615838	8.215875455
8.543234566	7.64232702	8.108917222	7.900715717	8.092218303	8.101068051
13.60979651	14.10328164	13.91489381	14.40789433	14.223913	14.28325373
9.838413929	10.79956984	11.00620373	10.91356479	10.62895885	10.44195016
12.25844332	12.70787607	12.41931404	11.63629658	12.01191659	12.23769992
9.998242414	10.33696433	10.76857843	10.76361324	10.50914282	10.86305205
10.36589334	9.649399939	10.7442872	10.57282784	10.48782122	10.8605042
11.07337622	11.69658017	11.60479972	11.32721575	11.19817469	11.38025327
11.17041501	10.90754603	11.22947614	11.18291631	11.19966733	10.77252405
7.436640343	6.946417495	7.266003808	7.344596535	7.422831253	7.545986354
9.719000626	8.939630657	9.656571869	9.524763869	8.804952828	10.98186414
10.48310631	11.56988377	10.58689262	10.55602586	10.51174846	10.95849458
6.759969273	6.873334899	7.262957343	7.154007354	7.02934998	7.191807606
9.759931222	9.187808616	9.048100778	8.625486818	9.09178804	8.765233303
11.70138789	11.09885051	11.94234713	11.35278885	11.57765358	12.27101
6.560443758	6.484447556	6.276608434	6.088521525	6.209772707	6.463509232
8.507211606	8.368326869	8.85049999	8.439612798	8.586230848	8.981975535
7.67441401	6.810861495	7.072297535	7.475616394	7.280525364	7.998937818
8.793874616	8.749910828	9.034403333	9.482964828	9.312041414	9.09013302
11.18096106	11.43380494	11.13879804	11.82852227	11.67849095	11.26990549
13.93694444	12.63500423	13.43523914	13.95955505	13.63435472	14.07988992
8.22437102	7.958393384	8.189194404	8.753927596	8.315227333	7.90331499
16.8710039	15.54332231	16.39933956	16.96059838	16.82803465	17.43906183
6.501455758	6.417796273	6.703725465	7.239398515	7.030162636	6.458964152
7.659186848	7.040256253	7.107390485	7.064243152	7.220240556	6.901464747
10.90192126	8.057063626	8.669992818	9.89348604	10.22761479	9.768390455
7.201570444	6.535075677	6.700834859	6.827661505	6.693279889	6.92164503
6.874731313	7.309336424	7.380377788	6.349593626	6.661815061	7.293451808
6.713538929	6.789988131	7.196285434	6.029615	6.321876909	7.055635202
10.69470446	10.57282784	10.54441943	10.60743282	10.47702836	10.08325108
8.608532485	7.893940788	7.677264869	7.756889051	8.005065495	7.809982667
10.40486524	10.2166926	10.32093584	9.960976253	10.1309568	10.13276962
8.17298499	8.097958939	8.779146061	7.987573434	7.896955242	6.829819869
7.579015667	8.522156747	8.418118394	7.891531071	7.947667091	8.099466788
8.800782677	8.790130253	8.96575796	8.573273283	8.667049253	8.55725397
9.588958727	9.885156737	9.835081424	9.263233333	9.759214424	9.224452333
9.265583747	9.044046343	9.223885747	9.269224374	9.236429667	9.206631485
7.018669091	6.854959677	7.067022475	7.218399283	6.999830061	7.404051424
9.384078242	8.349393212	8.82789604	9.167114	8.901331343	8.549517636
12.85048632	11.15966862	11.78023213	12.95303817	12.32964397	13.74759592

6.981086404	6.544328465	6.902984	7.261502495	6.661293222	7.688677182
9.217871889	9.627745303	9.695719162	9.792275424	9.363307667	9.746145949
6.591366	6.905826838	7.067324051	6.692767899	6.912275354	6.601482788
8.333749495	10.65127749	9.650789909	9.552821717	10.2026692	10.31693391
7.982442283	7.099269525	7.370661465	7.558170465	7.661901192	7.449616394
7.732550404	7.790242404	8.041332778	8.233421293	8.079973212	7.32489101
9.804037697	8.745474636	8.729972586	9.141873934	8.672881101	8.907915899
6.370742091	7.194677455	5.987269162	6.274896838	7.668960768	7.585519525
6.977525061	7.462172081	7.474688838	7.342557798	7.272914455	7.208062909
7.823083152	8.852092202	8.880364303	8.629474212	9.139095455	8.376150616
7.159962485	8.17298499	7.943134	8.026271202	8.104133364	7.719835253
8.568768141	8.35667099	9.814415596	9.500664495	9.257979848	10.21375666
9.279768394	7.881467646	8.564847253	8.681067818	8.629474212	8.795325869
9.402814081	10.09161001	9.563605545	9.903372323	9.967178364	9.870221061
12.39660011	12.93430142	12.69316533	12.87819387	12.73731843	12.42943771
8.949261586	8.836023374	8.586230848	9.306330646	9.118059475	9.027655758
9.933588354	9.009915859	9.520598525	9.242526283	9.521192909	9.151976485
10.23948175	8.703161646	9.002272697	9.782133848	9.492465394	10.33252085
10.25906505	11.23641801	10.94942311	11.13085117	11.21816154	10.56306707
7.194677455	7.21466303	6.918844495	7.164674212	7.349811747	7.016713091
7.521572545	7.568036061	7.891143768	8.051256737	7.971654859	7.818322879
10.39747702	11.33848921	10.74305691	10.65871503	10.59809845	10.87092264
6.310688818	6.77698199	6.719301162	6.499296646	6.712828707	6.254739111
6.083662081	5.867299758	5.916285152	5.89802204	6.756145354	5.830366293
6.40434199	6.307278677	6.199491667	6.270128909	7.195502081	6.068447646
8.072532475	8.484240687	7.901561475	7.539809273	8.245222778	8.026630869
9.686378667	9.800083455	9.793126455	9.215289101	9.282486081	9.469429253
8.202220545	8.639417101	8.428657566	8.473802646	8.316969364	7.62509899
6.947784242	7.221869232	7.08136002	7.095534354	6.777724495	7.011288768
8.725113212	9.284395657	8.380828141	8.814624313	9.139724848	8.696280707
7.807594768	7.675167556	7.394027687	7.293748525	7.258107788	6.950648455
13.81935116	14.64328928	14.26676228	13.87263802	13.89331098	14.00297944
8.217667525	7.772177253	8.135177152	8.552719747	8.277763646	8.769824364
8.533396525	7.477241869	8.874692596	7.756175162	7.751645273	9.273022949
9.439397061	9.763952101	9.556000646	9.656571869	9.37540303	9.662314131
7.412806768	7.854325061	7.752360667	7.772177253	7.91465098	7.580266434
7.535866141	7.050347636	7.121055596	7.262957343	7.182269455	7.606699253
8.907429495	8.960773354	8.995099212	8.645541747	8.902484929	9.166446616
11.07956266	9.136890505	9.841562192	10.24316974	10.32208546	10.10278369
10.92060078	7.952029848	8.892970697	9.629839465	9.410728505	10.81142627
9.615324495	9.423026242	9.390110384	9.263851586	9.576740677	9.017695354
11.65052704	12.03912361	11.76101506	11.9982543	12.02811467	11.51008986
7.870978364	7.133298475	7.122942091	7.119314485	7.164949697	7.025866354
6.554821889	8.309247939	8.411696677	8.063008152	7.617189485	7.116047475
9.839111	8.687053253	9.158666485	9.011040293	8.948168879	9.76471501
7.970926879	7.654890798	7.072618697	7.343750657	7.38862201	6.745632475
8.589388657	8.193820172	8.084674061	8.256499515	8.35796399	7.902575141
9.57181996	9.242021586	9.10215096	9.012031232	9.361074667	8.945002717
7.702311758	8.85049999	7.876807081	7.899333596	8.211411717	7.711891616
14.05136004	14.27397716	15.09418019	14.5413574	14.58095582	14.77222727
8.136641818	8.84786598	9.074692404	9.070661273	8.893968333	7.844877657
10.17440526	9.619644949	10.59292728	10.12909315	10.12799521	10.63266161
8.714332636	7.764199131	8.086566798	7.930919909	7.843077242	8.133179586
10.81664077	10.6426412	10.44832821	10.37467131	10.72900488	10.21484784
6.516723778	7.438614949	7.197080263	7.684267263	7.616552222	7.353560545
9.273640758	8.677707162	10.52689599	9.023071899	9.453123465	9.495686737
12.80602179	11.86652814	12.61449239	12.7939548	12.26775966	13.80704919
9.366312899	7.103815788	7.604779273	8.293769586	7.148192263	7.925642

9.66383	11.84529477	9.868690293	11.17571904	11.26623333	11.35080354
7.333224889	8.554027364	7.311037152	8.391869283	7.561051303	7.433916687
15.83165575	16.7804214	16.14978837	16.25437967	16.3077943	15.70163214
11.12935449	11.92396137	11.93486375	12.49170848	12.27421104	11.76336308
8.957944414	8.24438497	8.282900293	8.593558293	8.268052495	8.27903296
7.245884828	7.145208253	7.784529071	7.586712596	8.102484232	7.530291586
6.881026485	7.159147152	7.189710394	7.501110505	7.336476646	6.684834222
13.56886427	13.84455043	13.48401144	13.84455043	13.70717091	13.40990013
7.005900293	6.882764848	7.276013455	7.595597162	7.269064929	6.830856061
7.298462212	6.703943808	6.887288828	7.206984404	6.710071909	6.31049204
7.023397576	7.084738172	7.571116455	7.338325414	7.095275111	6.879943566
6.607353172	6.360006374	6.826408263	6.654520283	6.364101768	6.366139859
11.59654933	9.865550636	10.5431016	10.82034332	10.8947391	10.89224764
7.899333596	8.314850485	8.221794202	7.815211869	7.861535657	8.049354232
8.341977727	7.820222687	9.043522909	8.476485121	8.710842768	9.247858869
7.69973097	7.352900667	7.947667091	8.012010707	7.673376626	8.591239465
10.05426836	9.753529758	9.829119768	10.43757507	10.08599138	9.647889364
10.92757694	10.48897537	10.4322159	10.97767761	11.03163187	10.49009312
6.291307172	7.109111172	6.931501667	7.406826051	7.05790099	7.127674667
9.27419499	9.867106131	9.223302313	10.19757035	10.27905981	9.095762
8.974650333	8.45451298	9.319510202	9.304577515	8.998420707	9.651465606
7.397737788	6.839464636	7.073400869	7.440186081	7.256596263	7.905419798
10.39296715	9.793126455	9.744663444	10.06071544	10.09795649	9.696433626
7.543396525	9.235747242	9.560980909	9.575401111	9.703591263	9.264989899
14.29173122	12.27773509	13.36582475	13.49745762	13.06967433	14.52085241
8.166983455	6.407245091	6.507219869	6.915665626	6.597234394	7.072618697
11.38970236	10.72127155	10.85361094	10.4687107	10.59292728	10.3779146
8.385691051	8.228946717	7.975072535	8.030614293	8.070920919	8.087781242
7.368501293	6.315191152	6.306471071	6.938149939	6.748587242	6.695470869
12.28985985	13.65450155	12.97977545	12.96241124	13.03025597	12.89113428
11.75662781	11.2608846	11.26262606	11.62290433	11.06732533	11.03163187
10.2829717	9.372025394	9.673359929	9.631972414	9.711723394	9.742895778
8.991970091	9.223885747	9.486281202	9.925226333	9.939327313	9.739033131
6.808520727	6.900739596	6.886152545	6.963895283	7.211210626	7.513736343
9.494490616	8.769354545	8.871686232	9.145664475	9.127872697	8.693780273
7.154937303	6.922412606	7.379463242	7.656877838	7.230210646	7.135797152
6.517425313	6.115337182	6.341887717	6.471690111	6.08150304	6.807473404
10.85654613	10.69574272	10.75865445	10.00450786	10.59418919	10.70425349
8.920488949	7.977207111	8.685647889	8.682574626	8.808345061	8.565346707
8.179951646	9.403514808	8.256499515	8.695335444	9.298556212	8.503061444
9.061649657	8.323598384	9.066117364	9.270407879	9.029402949	9.182731869
8.155393222	7.175214949	7.134092121	7.412474121	7.523756919	7.996041859
11.28731295	11.9875501	11.7894918	11.86125412	11.54675633	11.81887725
6.757659566	7.330546788	6.734478838	6.788535525	6.894670869	6.894135505
12.49170848	12.30465105	12.10694646	12.66985986	11.63629658	11.99535117
11.72592113	10.23452156	10.87235819	11.17213305	11.07797425	11.15342364
10.78014639	10.24727703	9.987077505	10.22557955	10.31597323	10.14044783
8.466026899	7.858612495	8.149032212	8.325316889	8.197369283	8.491490828
6.535075677	6.490215273	6.346259394	6.585712788	6.156655838	6.515215919
9.625041525	9.02886299	9.749004051	9.876096838	9.415860727	9.804037697
7.612584303	7.771143222	7.032840081	7.165793091	7.522869182	7.243253313
9.848945263	8.474658303	8.891888576	8.914132333	9.12042397	9.27419499
16.49551516	13.6823979	14.68633385	15.34617167	15.48449671	14.9847254
12.89886333	13.71423947	13.06488572	13.62784329	13.23341018	13.13522491
9.301561	9.968215758	9.811898121	9.606954152	9.917863333	9.500664495
6.60058796	7.568924192	6.901187545	6.854415313	6.861163889	7.322572717
6.386235697	6.617114677	6.167536404	6.843046434	6.503631525	6.491736758
7.437975364	7.370079717	7.805446323	8.100222556	7.648616949	7.644277273

8.29291099	6.177536939	6.31629898	6.752477677	6.826408263	6.904353566
8.853585596	8.529417101	8.846850111	9.455711606	9.123297364	8.941795242
8.44129198	8.691458141	8.896578162	8.913024061	8.736221495	7.995673758
11.97660652	9.652169778	10.25320711	11.19638858	10.81540317	10.83363242
7.428605818	6.469862081	6.42732104	6.837484717	6.838696091	6.666656929
7.753046657	7.067324051	7.129307667	7.749943687	7.441392919	8.327353333
6.256334263	6.131811687	6.118230293	6.388351374	7.397737788	6.560909667
6.074500414	6.072015283	6.070436798	5.982799404	6.449263919	6.146031657
8.619848374	8.755421576	8.38301802	8.465492313	8.427685838	8.499836475
9.042944374	9.76546198	9.674270303	9.49510903	9.671170444	9.424985495
8.552719747	9.001272616	9.492465394	9.380979222	9.907546465	9.999132495
6.848028121	6.848551889	6.847553121	6.733232384	7.302736424	6.864987879
6.387499121	6.611130667	6.548235576	6.515423162	6.267641192	6.182782414
7.721939444	7.657256192	8.150096222	8.098747556	8.155023263	8.563501616
6.083513323	5.951037616	6.056170545	6.149009475	5.969349343	6.503401212
10.85513517	12.04734694	10.25038968	10.32208546	12.01956714	11.13240418
9.039454747	8.367840636	7.607714535	8.712341879	9.305189859	8.149448323
12.71830686	9.40167604	10.14131493	10.65500785	10.78014639	10.95374716
8.665646899	9.371514253	8.854095293	8.863920758	8.859179455	9.18155302
10.03026493	9.028300162	9.153232364	9.423636879	9.406893465	8.77608297
9.224452333	9.264483889	8.681067818	9.341222778	8.964642636	8.889922455
8.941359636	8.331458747	8.425847798	8.795325869	8.690918616	8.274698687
6.256512535	6.793900848	6.50824402	6.46756804	6.681596343	6.136227182
7.76948301	8.105629576	7.787223354	7.378508566	7.241538444	7.240274404
7.651593505	7.851263121	7.865908465	7.841061444	7.770089636	7.990313949
8.213832515	8.698751323	8.660166475	8.234232515	8.36556403	8.588971687
6.893606717	5.891497667	6.009700404	6.303290121	6.193202374	5.864779111
10.13862753	8.846850111	8.934248131	9.20200202	9.223885747	8.713915657
8.691458141	8.777050384	9.035586242	8.578858313	8.339926909	8.645541747
10.18098661	9.770750222	9.57872197	9.218466939	9.432636424	9.373397364
7.354804152	7.632188616	7.522209596	7.706168626	7.391081455	7.45822897
9.428190232	8.343220798	8.880853535	9.264989899	11.58195332	8.773471869
9.405539273	9.976273051	9.596088263	9.899010949	9.533033172	9.692793657
12.82153585	8.426330949	9.568406747	10.65634987	10.41022215	11.27499716
7.174439091	6.277726788	6.367286556	6.681851	6.618022394	6.791252535
12.65472241	9.272374697	10.41846051	11.25523353	10.65365596	11.87445957
8.889922455	7.556609313	7.770089636	8.333227545	8.269818727	8.217984283
8.337114525	6.369478939	6.413679545	7.184502222	7.369172101	6.737335172
7.086698091	7.649291899	7.494510091	7.712213273	8.07563398	6.894391313
10.52799495	10.38531322	11.10170724	11.109559	10.73132595	11.38391359
6.647920455	7.500795283	7.608647091	7.29078701	7.272016424	7.115448364
8.275152535	8.188779434	8.756886111	9.596088263	7.962004212	9.30577901
6.551865586	7.120197273	7.775998535	7.324608717	6.94531798	7.487647384
8.820759798	9.151431899	9.769994879	9.93663897	9.699589758	9.739859192
9.560346242	9.384078242	10.33696433	10.09795649	9.950180667	10.46766096
8.298896434	7.475313929	7.868335495	8.088528121	8.078044465	8.254484434
13.77352346	14.1904887	14.56207529	14.62217632	14.85646221	14.49002723
10.35284213	11.38391359	10.52364746	11.18471248	11.14866374	10.46108665
6.459423596	6.524717879	6.615630737	6.652654	6.65032298	6.28101901
13.21058954	13.35359128	13.13007894	13.62106704	13.29684189	13.03025597
7.796747616	7.481960152	7.670275758	7.478803566	8.250194202	8.030614293
7.517772121	7.236271343	7.617189485	6.64329102	6.566529242	7.848409778
7.568924192	6.187206293	6.163811747	6.927259889	6.960749798	6.914278354
8.671294253	7.916712838	8.50473503	9.281923404	8.942313313	9.35796903
7.363924808	7.224700556	7.613815293	7.179445253	7.609232899	7.473746677
9.642500081	10.72900488	10.57028524	10.45375986	10.44195016	10.66229262
6.533325687	6.775746758	6.940218677	6.702042636	6.487487818	6.422838717
7.53302901	8.313601758	7.933573889	8.290353162	7.050073121	7.199149121

11.47127063	11.33477589	11.53473316	11.77305263	11.90152869	11.77053658
14.48025691	14.06476988	14.23242051	14.39736901	14.25042984	14.1904887
8.444861909	9.287277576	9.252443646	9.231946182	9.664626495	9.126080354
7.318539616	7.620272192	7.140009717	7.135797152	7.332963121	6.863389636
7.565422434	7.502411182	7.516753121	7.353244222	7.749943687	7.376100929
8.097566687	9.053706202	8.693780273	8.844262616	8.709941838	8.818695101
7.154661646	6.320274152	6.563630475	6.821005303	6.82197003	6.73278298
7.695912303	7.605411525	7.918472515	7.922697606	7.718747404	8.432150758
6.983407384	6.171777677	6.209048788	7.352232505	6.74432	7.06104997
7.34804498	6.240475939	6.512566313	6.584832545	6.693002899	6.484023485
10.34311059	9.835081424	9.870931818	10.19332507	10.02821235	9.915234909
8.016429949	9.254253192	8.381754576	8.388817192	8.434806414	8.271795141
8.615838	8.55725397	8.822853162	8.893419636	8.829061111	8.619848374
7.016433495	7.526779747	7.836508475	7.51310204	7.347141657	7.557268586
7.539487212	6.268823394	6.137509616	6.261376788	6.263522859	5.996715586
13.03957854	13.06967433	13.04869312	13.48990673	13.13522491	12.29886409
11.56988377	11.12315281	11.32376318	11.39382595	11.55524693	11.20840189
9.711723394	10.02644328	9.791460222	9.453123465	9.504584061	9.593075545
11.76804793	11.31053596	11.1276132	11.24816417	11.38775416	11.39185194
7.470612081	7.525015414	7.99823603	7.884314808	7.535195111	7.876807081
6.762066263	6.044454283	6.083954222	7.585815263	7.118205404	7.434501333
7.132751192	7.961659859	7.954205616	7.34349297	6.908859909	8.531247657
6.063192515	5.818332182	6.042269061	5.892180121	8.025051697	5.836991424
13.08418191	10.31480319	10.97896732	11.34732253	11.29597305	12.61449239
8.126700889	9.11510898	9.261851101	9.096395636	8.995584808	9.337187111
5.753285636	5.820594646	5.859926222	5.788047859	6.385847152	5.902862586
9.890949131	9.198436465	9.268661657	9.446464909	9.519086535	9.279768394
6.191632212	6.587802667	6.666381515	6.430485283	7.411868313	6.579152939
6.508494242	6.628304848	6.839237455	7.032569455	6.777950687	7.294583061
11.46539851	12.16036216	11.9577766	12.05489479	11.71895032	11.72807534
7.904747051	7.636533838	7.271416848	7.576996152	7.855388333	7.514821949
8.458697343	8.248137606	7.866306808	8.935320828	8.119288848	8.550414556
7.461509737	8.033283465	7.458509535	7.754407434	7.875022121	7.202436384
6.212186293	6.922672051	7.478449101	6.961758566	7.086968939	7.272914455
8.564847253	8.256076333	8.213079374	8.24438497	8.284505182	7.952386505
16.25437967	13.48990673	14.86987024	15.09418019	15.19830883	14.85646221
12.34577038	12.10694646	12.15440913	12.57877126	12.24097391	11.92156179
9.062782333	9.687758929	9.295516616	8.731005677	8.835019636	8.757405333
9.262561	10.29542336	9.960976253	9.657223606	9.858738737	9.886762101
6.915128061	6.714000354	6.182077616	6.723002525	6.516723778	6.237576566
10.11051676	9.484209455	9.477181455	9.185497838	9.910960495	9.592426051
8.596127869	7.409710545	8.631899566	8.78563097	8.662816202	9.084401768
6.386847838	6.798121798	7.055909434	7.443557333	7.032840081	6.829096384
6.448364717	6.318299141	6.636479121	6.498851707	6.64187404	6.640442667
8.476085384	7.880750535	7.707585606	7.85299496	7.710578727	7.335252788
9.186711646	9.870221061	9.766921414	9.67665103	9.565032515	9.271123182
7.790242404	7.152088687	7.177746566	7.577683121	7.555333263	7.173022687
9.252443646	10.6362571	8.83754499	9.329922273	9.798623747	9.122783717
11.37847359	12.24933389	10.89912578	11.71443529	11.99323436	11.14057221
9.203760495	9.653591273	8.578393657	9.115691495	8.940148162	8.574218515
9.082448343	10.1985269	8.940763364	9.179226364	9.603529667	9.211846273
12.27421104	13.40990013	11.78498903	12.11841559	12.99565877	12.02240324
11.84529477	11.20498985	11.671419	11.73972102	11.91416468	12.48458958
14.08841453	14.25042984	13.9071606	14.1904887	14.23242051	13.45466248
7.356848556	6.032022283	6.219076051	7.057330242	5.954705364	6.047759404
8.220625586	8.197805768	7.857447394	7.507037566	8.259704283	7.69973097
8.247392788	7.949071818	7.870669919	7.811371919	8.040575909	7.67197899
12.31419772	11.86401838	11.87687449	11.55322016	11.47496104	11.83330316

10.54800525	8.816703535	9.979092404	10.26729954	9.943097475	11.0035084
8.822853162	9.171801828	9.441908576	8.991410131	9.103811545	9.439917707
6.733451596	7.337670899	7.270788949	7.809338131	7.519990596	7.240898899
8.946596606	9.576136707	9.545881535	9.680349586	10.0053333	9.898158111
7.057330242	6.551212667	6.720840657	7.439837192	7.100441616	7.490430596
7.415639222	7.562627657	6.71121296	7.193007566	6.965717616	6.783595586
8.402148465	7.601973414	7.859293	7.410002717	7.864861071	7.629989283
7.241813919	7.410364737	7.219623727	6.290882545	6.61398898	7.068375576
7.814093232	7.733842657	8.08972896	8.226165	8.227270172	7.766006091
10.4549623	10.45996909	10.64128512	10.89604789	10.73014715	10.33989796
9.778131475	8.879851283	9.890949131	9.625623061	9.459003	9.757590192
8.33024502	8.302062758	8.432150758	8.951989212	8.538380869	8.81572204
11.13879804	8.835493283	9.315088131	10.12610261	9.975431768	9.945994374
8.374810828	8.036816444	8.221010253	8.410410394	8.388817192	8.249350414
7.316995263	7.291703707	7.390471152	7.391655768	7.511838192	7.373983485
9.895061889	10.94942311	10.76471511	10.42297323	10.57649171	10.60507136
8.493653778	8.387083515	8.32856404	8.530333535	8.297224525	8.705145061
7.80651396	7.489478697	7.680542242	8.221403505	8.003903253	8.57070398
7.722297051	6.364484303	6.412800202	6.998209475	6.891529131	7.129597273
10.29851595	10.98627861	10.72246175	10.52364746	10.43757507	10.87379507
8.069395848	7.635267465	7.829693939	8.186373323	7.930919909	8.692466525
8.634788929	9.522660222	8.789132293	8.962353222	9.159196141	9.179819687
7.863532101	8.494130061	8.437457535	8.181574081	8.185563303	8.147481596
8.451550535	7.693213707	7.873080636	7.879657162	7.802180768	8.79722998
9.609756293	8.786134101	9.766131535	9.631182697	9.339764051	9.958247111
10.51400776	10.7442872	10.37026232	10.11760527	10.11150219	10.47479655
9.882779162	10.5397012	10.04963797	9.760900556	9.999132495	9.924448444
12.43600068	12.79024082	12.11841559	11.49239347	12.28693608	12.19996238
10.95683616	10.56655729	10.53247367	10.21769501	10.19418334	10.56421743
10.24942024	10.81142627	10.27338352	10.2600862	10.22448985	10.57649171
11.77552743	13.62784329	13.05428976	12.69316533	13.02518615	13.03957854
9.08959502	9.395177889	8.591239465	8.545617303	8.718321818	8.857327323
8.871209182	8.967408879	8.498949848	9.060618192	9.184527788	9.344138121
9.565032515	9.251906182	9.217871889	9.335272182	9.627013505	9.333361616
6.992927808	7.039678949	7.140588444	7.258984768	7.324608717	7.178076949
8.368780919	7.544367232	7.825440475	8.263866778	8.372086737	8.264240727
7.506719576	7.608347495	8.282073859	7.616552222	7.111135707	8.358418384
10.36465285	10.44832821	10.44392641	10.24227579	10.47370876	10.5752557
13.82538175	14.84412351	14.46162431	14.59173737	14.07988992	14.25042984
6.95496698	6.002675707	6.603596232	6.352052949	6.413218091	7.572084505
11.308834	7.078835444	6.898538505	8.270601414	7.886820596	8.663303283
9.196689051	9.142791657	9.083741606	8.492322828	8.740195303	8.951989212
11.86401838	12.76628311	12.28985985	12.63888703	12.58287167	12.46466592
8.925229707	9.281446566	9.239472869	9.03221704	9.150811232	9.251906182
13.97489728	13.68884286	15.29549959	14.13521971	14.7172184	15.34617167
8.008022949	9.085558838	8.626833051	9.014846778	8.849981545	8.76626204
11.54871614	11.91651973	11.59654933	12.03912361	11.88649013	11.6295292
6.821773354	6.417620515	7.128773535	7.037248667	6.637000293	6.968393455
7.886079293	8.630877737	8.582586091	8.628622212	8.341133162	8.611286687
8.96575796	7.962004212	8.287081333	8.668931657	8.575095707	8.744562374
6.955646677	6.637667434	7.010236465	7.233950838	6.879719566	7.620680879
9.321890899	8.476085384	8.532516465	8.648914657	8.78767504	8.871686232
13.33661988	12.9851923	12.99565877	12.52130815	12.83405786	12.55625498
10.40387449	11.58421758	11.51260306	11.06123261	10.72633412	11.52645916
7.701352778	6.607135434	7.107957081	7.589252838	7.577683121	8.164230707
14.89443037	15.95256028	15.48449671	15.46469304	15.58150095	15.09418019
11.08435313	10.54800525	10.3810842	10.77637516	10.74045328	10.29851595
10.41530743	9.020821455	9.500664495	9.818059192	10.23850383	10.12697515

8.781680606	9.060017677	9.014335657	8.534897646	8.596127869	8.425847798
7.724673141	7.183662697	6.941216071	7.160253414	7.075225141	7.399710364
6.84607197	6.455374485	6.807473404	6.833840465	6.808520727	6.324763646
9.048100778	8.777572838	8.715845505	9.159785394	8.970512081	8.814624313
9.208368677	9.881912677	9.652169778	9.959124273	9.793942798	9.225515485
10.48074239	9.080683394	9.404880263	10.05320582	9.992988556	9.182204323
7.697220202	8.397853727	7.45998899	7.393167162	7.812327374	7.594027929
12.02240324	13.05428976	12.73731843	13.3718573	13.05428976	12.56726296
11.66441291	12.97977545	12.70002263	12.86086109	12.48835313	11.54470354
7.019921606	6.57397498	6.674983667	6.810023535	6.794878162	7.058212263
8.917682242	7.097914788	7.657256192	7.80651396	7.599870798	7.99823603
9.014846778	9.231349182	9.027136657	8.614861051	8.872352707	9.015966535
8.324880909	8.903025495	8.428176394	8.497692788	8.487847515	8.373919657
11.41071085	10.75600216	10.32750324	10.05780386	10.60635433	10.41426339
8.346198293	8.806450121	8.547449101	8.375668162	8.492322828	8.673363869
7.371907727	7.194186434	7.170490939	7.671593667	7.593038414	6.980785162
7.431067202	7.566119051	7.872350636	7.329290475	7.839249838	7.928832091
7.662925475	6.82246696	7.220780818	7.404680798	7.342557798	7.03650198
8.319524414	9.390834152	9.089024899	9.008956374	9.146178758	9.067241768
10.38842688	11.12151875	11.00754401	11.13240418	11.03737422	10.63516273
7.216363677	6.646655162	6.612514242	6.962822313	7.073141899	6.624377646
8.774537848	9.07419996	8.670884566	8.149448323	8.273061586	8.323598384
7.448630646	6.841827051	7.095534354	6.997949788	7.374842788	7.041898303
7.521266808	7.145493505	6.611373919	6.693987121	6.931296273	6.79323798
10.12799521	9.182731869	9.706432808	10.21279484	9.724047525	11.23116994
6.731476051	8.155023263	7.107120283	7.801006182	7.882850313	7.640655485
12.04160066	12.64708972	12.58612865	12.34237091	12.12151457	12.61090773
6.904353566	7.070666535	7.294284717	7.754082929	7.530907465	7.845981061
7.910975505	7.554702939	7.441979727	7.85604701	7.766357737	7.653990838
7.679285535	7.345977091	7.083894141	8.119288848	7.517436172	8.110881556
11.55106122	11.43005301	10.97767761	11.77802923	11.58005536	10.48074239
8.924680667	8.362537455	8.379884889	8.335469222	8.334587636	8.164575929
10.81796524	8.90531298	9.815129384	10.48782122	9.804037697	11.52238659
7.946141838	8.251492909	8.742635939	9.061091758	8.560860505	8.469339576
8.002011657	7.831503424	7.797844434	8.178694051	7.871683444	8.460488909
8.02125603	7.078303869	7.942803596	8.277763646	8.193820172	8.609511
6.732033313	6.845301404	7.860366808	7.533663232	7.963371182	7.835434535
15.92931651	13.11515098	14.1830221	14.46162431	14.64328928	14.5413574
16.61084374	13.60269863	14.78379092	15.72204116	15.70163214	15.31193978
12.33907422	11.51008986	11.62739451	11.97447062	11.96947069	12.21312889
7.959080606	9.683334444	9.243096889	9.616741535	8.655437626	8.742149222
7.215185141	7.202701576	7.56574702	7.241538444	7.627373273	7.389558374
8.841037687	9.380979222	8.804952828	8.299695162	9.276189121	9.186096828
5.83769397	7.619933919	6.85466502	7.13441502	7.289331586	7.137721343
7.815532475	7.845981061	7.613240606	7.38340502	7.428304182	7.265083586
7.094450071	7.572483949	8.025864485	8.177146606	7.817254242	7.954533778
9.149195859	8.641737071	8.719245818	8.228946717	8.735755485	8.515350859
8.241600556	7.888681737	7.807154444	8.176360121	7.786298434	8.213079374
8.867411152	7.718083212	8.818695101	8.429130081	8.159907626	9.221852535
8.664254273	7.071486061	8.11773804	9.146178758	8.352134333	10.07107569
6.202273253	5.953231333	6.062791	6.399678273	7.123473687	6.536865101
8.733083212	8.615838	8.940148162	8.528915121	8.234636465	9.262561
7.24440804	6.748847646	7.281402384	7.133560273	7.310482798	6.598705667
11.12454893	10.61371787	10.72633412	10.19418334	10.15186663	10.25320711
8.609952768	9.147897788	8.799348343	8.587234515	9.055478465	8.559456798
7.648616949	7.531770848	7.742436303	7.766357737	7.697940283	7.678291495
9.762542495	6.581970424	6.661555384	7.55178802	7.963792818	7.650858424
8.930433505	8.39446903	8.611710212	8.821885465	8.542859455	8.070920919

7.727390434	7.723009	7.429567525	6.737098263	7.093254121	7.314025869
6.734478838	6.221214869	6.334404253	6.36195701	6.525168444	7.063754707
8.906407303	8.487847515	8.554027364	8.554971	8.685647889	8.482406475
9.636296737	9.35067002	9.198999222	9.419020677	9.451851384	9.402230293
10.11760527	9.192533242	9.588958727	9.576740677	9.749660394	10.32418729
9.522660222	9.842388081	8.298896434	8.18481599	8.181196919	9.165786899
16.17943014	16.8710039	17.29704085	17.29704085	16.61084374	16.73234105
6.854415313	6.580062899	7.088019323	7.409335747	7.015941667	7.476255091
6.105514909	5.872561636	5.929254283	5.936026384	6.827413455	5.987495828
10.25320711	9.66613299	9.569063343	9.843921515	9.842388081	9.481000434
6.353796111	6.829579545	7.186327495	6.974196465	6.79225503	7.028571808
7.432999727	6.760600303	6.774936697	7.312252182	7.140902202	6.678790232
9.161011869	9.117478424	8.959130566	8.955880859	8.773471869	9.208368677
7.980568657	8.133179586	7.996041859	8.588971687	8.247731232	7.659822475
6.529968303	7.18313201	6.857502071	7.268778051	7.154937303	7.023397576
6.845301404	6.547992737	6.696930808	7.086390323	6.887288828	7.043463859
10.95093974	8.460053242	9.596773313	9.885156737	9.740584727	10.22761479
6.406130717	6.617782121	6.883793818	6.736847222	6.58104199	6.994245051
16.02065153	13.52959744	14.64328928	15.06622261	15.02501841	15.08134613
8.660166475	8.077645899	8.57790302	8.918756414	8.889380424	9.086722374
9.356741384	10.18942995	10.15483313	10.20532801	9.908305848	9.758431737
10.76471511	10.05144087	10.10841911	10.48432644	10.38631174	10.10093339
15.06622261	15.97330865	15.67809237	15.74256723	15.42966281	15.18392271
8.716387192	8.597538556	8.314850485	8.362142131	8.415109929	8.324880909
9.689783586	9.393335687	9.17294002	9.499247869	9.468772697	8.829061111
10.62647628	11.24816417	11.43938222	11.54675633	11.3733012	10.90192126
7.90609097	8.179120646	8.656360697	8.129874121	8.289212586	8.933684374
7.013931768	7.079103131	6.455790798	6.583924121	6.913266485	6.82018896
13.80704919	14.82007331	14.49002723	14.26676228	14.02572128	14.26676228
7.149668323	7.404680798	7.409335747	7.570765313	7.307847101	7.812003626
9.55462702	10.04160563	9.451851384	9.384699788	9.705000525	9.341222778
8.198599495	8.438712657	8.175629323	8.028563859	8.163053768	8.068200283
7.40744601	8.193428737	8.08112003	7.823429646	7.858214495	7.942409707
8.230588212	9.036076414	9.100524758	8.879851283	9.329922273	8.708995576
8.57070398	8.573273283	8.417604414	8.187968455	8.087781242	8.164979273
6.486163293	6.11789698	6.415279879	6.530171141	6.366615303	7.183404394
15.81050193	15.27762024	15.76560628	15.67809237	15.54332231	15.92931651
11.96362869	12.12151457	12.05813886	11.85033795	11.81221198	11.86652814
8.498503737	9.093526566	9.423026242	8.309247939	8.57971698	9.220765354
7.70790696	8.14677703	7.705814323	7.871272152	7.762451808	7.721939444
9.596088263	8.396125929	8.867918475	8.974650333	8.750392838	8.823394192
6.976769172	6.480794707	6.937881404	6.773917293	6.912025515	7.457078101
13.1944969	12.46466592	14.25042984	14.08841453	13.78027417	13.93694444
7.898595626	7.390752071	7.836828747	7.639990515	7.890057202	7.623386535
12.91656586	12.55983323	12.28693608	11.85301976	12.35798465	11.88148631
11.88148631	12.44264271	12.62807191	11.54470354	11.97944932	11.76588718
13.41514643	13.70143341	12.95787308	10.82416773	11.64848719	12.06964699
11.3403052	10.1838089	9.170513566	9.033915556	10.34193651	9.515743253
9.717365758	10.31693391	10.53737043	10.03611568	9.782868192	9.948421707
7.150454879	6.147209687	6.401803212	6.784609556	6.350237535	6.731175838
8.474658303	7.685352364	7.872718384	7.972411364	8.302900242	7.739314727
9.505859384	10.08599138	10.35917202	9.875361242	9.800904051	10.06255975
10.79956984	11.94745099	12.08943903	12.06964699	11.83072395	11.86125412
6.526092091	6.043460929	6.470357717	6.26630199	6.168988222	6.780217071
6.780968242	6.653596384	7.24440804	6.320680717	6.513435323	7.457359414
15.70163214	16.52993947	16.4534382	16.3077943	16.14978837	15.72204116
13.94443	14.78379092	14.70594079	14.58095582	14.36070414	14.16629104
9.798623747	9.95201103	9.990505182	9.642500081	9.831834717	9.795448596

12.93862501	11.53677901	12.80949032	12.63500423	12.85048632	12.99565877
8.65914896	6.744878414	6.947784242	7.22559698	7.221869232	6.908606212
7.691187768	7.992662242	7.619556354	7.905419798	7.84419497	7.461834424
6.116729	6.203838343	6.285908	6.31770101	6.227503758	6.380424646
8.030614293	7.416641556	8.030614293	7.279952616	7.595301182	7.874403566
10.0178996	9.611261414	9.518503263	9.320176919	9.588958727	9.329922273
15.72204116	15.26374094	15.70163214	15.54332231	16.02065153	16.17943014
7.435437465	7.303562909	7.412191343	7.62509899	7.495424616	7.80651396
16.4534382	16.49551516	14.6970922	15.12351424	16.42659037	17.86179439
15.32551587	15.36066179	16.28343863	15.76560628	16.04217659	16.14978837
8.047553323	8.594016788	8.583048444	8.691458141	8.678158051	8.541972566
16.36806566	16.25437967	16.82803465	17.01900663	17.1822602	16.8710039
6.759694808	7.23452504	9.453743545	8.353342091	8.003147788	7.015108687
10.15283227	9.293710889	9.790672212	9.954681949	9.750460616	9.493894545
7.450590374	7.717737636	7.634532263	7.404985061	7.185712515	7.583321859
7.984288364	7.924172566	7.960941889	7.963371182	7.721939444	7.58864003
8.390114414	8.754441596	8.595703707	8.316081222	8.876650909	8.802954374
7.445432899	7.221626808	7.487940131	7.254341798	7.024222101	7.988882495
11.57567047	11.25523353	11.35475345	12.26775966	11.80342824	11.19114259
14.74028573	16.28343863	15.59877501	16.06827159	15.50564301	15.58150095
17.86179439	17.12376032	17.86179439	17.86179439	17.86179439	17.49517975
17.43906183	15.08134613	15.95256028	16.39933956	16.52993947	16.02065153
11.40495249	11.29744903	11.74205097	11.70138789	11.53239215	11.91651973
8.433468657	8.176718545	8.050868949	8.682101313	8.539776737	8.748883111
7.983184263	7.870356606	8.291610121	8.297630758	8.480158333	7.864535646
7.331138333	8.120448121	7.944406939	7.969793182	7.563439404	7.864861071
9.520598525	8.978723121	9.303342798	9.759931222	9.613206141	9.423026242
10.28899471	9.09824599	11.29076429	10.31378621	9.935661677	9.86223396
10.39641485	10.17440526	11.03296099	11.19293398	10.89224764	11.21187068
6.742790232	7.271416848	7.604109192	7.790604293	7.214315212	7.56409603
11.09565519	12.797444	13.58996905	13.27418186	12.62807191	13.67554111
10.73014715	11.89876792	12.48835313	12.47441306	12.47441306	12.69676846
6.805713475	7.437214323	6.614759768	6.682081222	7.008416879	7.018424152
8.693780273	8.539776737	8.571194222	8.519178475	8.394903626	8.649944576
9.403514808	9.819688798	10.29660096	10.24727703	10.56421743	11.45932673
7.073400869	6.649897606	7.033625222	6.604082657	7.116869414	7.553773697
7.586712596	8.232163879	7.461239687	7.163006	7.509707515	7.593349828
7.715785354	8.000144121	7.059676455	7.861832121	7.957321232	7.506434626
11.49420288	9.802496737	10.76239578	11.2345748	10.71613341	11.84027237
17.36803328	16.96059838	17.49517975	17.67797782	17.43906183	17.67797782
9.438094949	10.32635032	10.15379546	10.25320711	10.06633871	10.18666124
8.649462798	8.08737399	7.890057202	7.954205616	8.177906263	7.850535525
9.607593848	11.11081747	10.39521192	10.32750324	11.17750487	9.878686616
7.28082202	7.747112485	7.116047475	6.775232444	7.751275798	7.329870525
8.275579182	7.26025397	7.875022121	8.141366242	7.854325061	8.59942799
8.669992818	10.12610261	10.1752482	9.468772697	10.03385507	9.801802283
7.44169298	7.107390485	8.029383646	8.423139949	7.916712838	7.28082202
9.300367869	9.086722374	9.022478232	9.072998758	9.138451657	9.280885222
7.52469401	7.555333263	7.743136949	7.530574707	7.377037515	7.317897131
11.68700199	12.11841559	11.77802923	11.24147284	11.66189432	11.79171062
6.716824636	7.060219081	6.727688212	6.821773354	6.657192121	6.908354899
8.968910758	8.450646323	8.481851586	8.500240172	8.40783399	8.351407394
9.019731374	8.989142232	9.403514808	9.353057535	8.950874253	9.300367869
7.297158313	8.807484626	7.736964596	7.471847707	7.548584576	7.344013535
9.038849273	9.178084667	9.439917707	9.227275152	9.01707996	8.777050384
11.33100665	10.79686873	11.96362869	11.46936418	12.17216641	11.19966733
8.559456798	8.219387636	8.495447152	8.569209263	9.241421747	8.588536172
7.68296396	7.295094879	7.698300838	7.592689374	8.31049196	7.488273475

11.42371373	11.65918316	11.33477589	11.99535117	12.21312889	11.48459908
8.986932636	9.150811232	9.331038253	9.838413929	10.0719806	9.333986848
10.22267877	10.13580598	10.58236152	10.95683616	10.99623862	10.75987116
8.63568099	8.502042192	8.592161788	8.897177899	9.002847848	8.558108202
9.021393263	8.731005677	8.628622212	8.902484929	8.910531141	8.203151141
10.32946621	10.35385551	10.28795108	10.49241362	10.66706732	10.24942024
9.073600626	9.01707996	8.959665061	9.195011616	9.123926859	8.638487899
12.7452022	13.50997822	13.30255942	13.66158872	13.55640443	13.11515098
9.302103889	9.139095455	9.286739242	9.996420616	9.93187304	9.511857455
12.64265677	13.34730616	13.29684189	13.61616406	13.35359128	13.20536123
8.340252101	8.284073869	8.35372597	8.929972798	8.936938576	8.39402401
12.14789109	11.59870577	11.48655657	12.73731843	12.66985986	12.41033676
9.876880455	10.94071069	10.93646713	11.29399858	11.01951417	10.82804231
7.906781182	7.975072535	8.140538697	8.324453253	8.558556152	8.220225172
8.814101707	7.737987747	7.727390434	9.017695354	9.133918929	8.864430475
8.659649677	8.359650919	8.165787747	8.76478299	8.861728919	8.034450929
10.11246778	9.230770667	10.0728998	10.09506496	10.07956055	10.43872423
13.13007894	12.78185519	13.46005067	13.11515098	13.38447499	13.29117716
6.749564212	6.748587242	6.926979111	6.864210101	6.945089283	6.806487737
7.373717778	8.053619525	7.883536596	7.994566778	8.010919919	7.142895121
10.87907816	10.38631174	11.28565422	10.6488688	11.0859227	11.18096106
11.48258282	11.48061491	11.38025327	11.64637724	12.08128174	11.06877356
11.43938222	10.66587479	10.91903182	11.50051559	11.22947614	10.97054498
12.71520416	13.66158872	13.58209631	13.79381649	13.52959744	13.42153085
10.2761618	10.98350754	10.90192126	11.03296099	11.22294817	10.78705857
14.5413574	15.58150095	15.31193978	15.58150095	15.56435861	15.41210544
10.31997281	10.23247836	9.781348374	9.634846707	9.917043434	9.69725304
8.382609505	9.269224374	9.449254061	9.599465606	9.095762	9.608350808
9.961780586	9.393934131	9.454390232	10.16165443	10.01257663	9.200042253
14.16629104	13.39037726	12.80166365	13.88591407	14.17411554	13.02518615
15.50564301	15.81050193	15.32551587	15.78787594	16.2036826	15.03840752
8.761177727	9.590351455	9.955574404	9.588410576	9.815129384	9.565660949
8.756396081	9.556000646	9.617464455	9.289535939	9.185497838	9.336479586
10.21054893	10.53737043	11.06877356	10.93171462	10.96000427	10.86691694
12.47130519	12.83405786	13.28559105	13.35359128	13.93694444	13.20018245
6.446375626	7.516753121	7.495760485	7.398020586	7.87472	7.180839586
6.973893828	7.882189879	8.063898495	7.506719576	8.173633596	7.889031475
6.638550414	6.687236677	6.84778002	7.868007081	7.815532475	7.05790099
7.843077242	8.316081222	8.479727162	9.761765848	9.575401111	8.742635939
10.37573078	11.76804793	11.41981305	11.05191968	11.62739451	11.35475345
12.17829278	13.43523914	12.99088515	13.17281418	13.12023156	12.75396286
9.033394545	11.46320141	10.2861211	10.33146686	10.17702971	10.02644328
10.20964764	11.19114259	11.58005536	11.26262606	11.04464523	10.69682519
11.11081747	12.01427079	12.01427079	11.74439188	12.33576886	11.66934136
15.39581235	15.83165575	15.42966281	15.83165575	15.88528111	15.26374094
14.78379092	15.03840752	14.94534759	15.02501841	15.46469304	14.4185289
15.27762024	15.38120792	15.12351424	15.50564301	15.67809237	14.72870348
16.04217659	16.73234105	16.52993947	16.17943014	16.73234105	15.88528111
7.948737212	7.783465061	7.656238101	8.098302202	8.281588828	7.493565131
7.84876403	7.696527091	7.733842657	7.854685616	8.092691677	7.846992646
7.900388485	8.530767747	10.38329808	8.513135323	9.183367525	8.924680667
11.91416468	10.8947391	11.82346178	11.57178154	11.671419	12.03068847
7.678291495	7.612584303	8.012698303	8.265565879	7.848096879	8.424471616
10.58236152	12.02811467	12.20960187	12.15130062	12.14507673	12.04160066
12.10951308	13.2504616	12.94367069	12.59668127	12.81735602	12.44950322
9.541747455	7.696259101	8.150892606	8.109266465	7.842052606	8.947127939
9.786685869	10.55213977	10.60161194	10.19136651	10.14713571	10.31597323
7.791244131	8.27018399	8.388817192	8.320357909	8.363468485	8.538919798

9.163332636	10.21484784	9.905108616	9.791460222	9.946747141	9.990505182
11.4535969	12.49170848	10.28089537	10.69470446	9.996420616	10.22668357
10.61743608	9.646274414	10.48659409	10.65127749	10.17904437	11.28053313
11.0473733	11.93486375	12.08680505	11.97196754	11.8358519	11.97196754
6.728691949	7.461509737	7.063984253	6.968156515	7.074173121	7.23452504
10.86447048	11.68038318	11.53239215	10.88302044	11.25898056	11.18636156
10.3494333	11.07337622	10.96000427	10.47370876	10.76609029	10.70902317
6.221607646	7.472789818	7.052522778	6.757434848	6.742219182	6.697631071
10.29752835	11.17750487	11.06123261	10.59418919	10.48539779	10.81540317
9.337862091	10.0719806	10.11349335	9.905108616	9.867106131	9.961780586
8.235388636	8.264240727	7.706911222	7.167804919	7.695265798	7.973227556
9.669847545	10.23752804	10.2187154	9.593075545	9.497817424	9.899010949
10.03123363	10.11051676	10.12799521	9.800083455	9.821195081	10.07956055
7.206091434	8.028563859	7.573788949	7.44801498	7.565085768	7.677613273
11.44491584	11.87175588	11.72592113	11.9577766	11.49420288	11.81439214
8.781156657	8.968391182	9.129114848	8.834473929	8.818695101	9.002272697
7.672313364	7.799196616	6.840910293	7.67441401	7.487940131	7.543124535
10.88681216	10.57885616	10.67305197	11.05796986	10.3918566	9.753529758
9.49850403	8.719668061	9.118629636	9.266188263	9.293068828	8.559975162
7.189410667	6.593389535	6.506079909	7.007469525	6.712585717	6.150621626
8.712341879	8.837022465	8.966940586	8.848410616	9.149684939	9.30949301
6.773240303	7.766006091	7.34804498	7.563439404	6.977525061	7.314300525
6.426872343	6.079607434	6.392742677	7.987912222	8.352537101	8.968910758
7.000099141	7.393489313	6.629250202	7.188870657	6.568165434	7.067591
9.470173818	8.617420111	8.937429899	8.872352707	9.244399414	9.609027141
7.989587677	7.130177747	7.927460424	8.388388636	7.935081758	8.710391727
6.900957616	7.065949717	7.190644495	7.353900202	7.099269525	6.696224707
7.634226727	7.720197707	7.858612495	7.991889949	7.409045616	7.964179182
6.215520505	6.338442758	6.593877636	6.338878222	6.298192394	5.981818636
9.260607	8.584830636	9.450568111	8.876650909	8.812013889	8.719668061
6.734222485	7.337386909	7.106592788	6.789755162	6.883542434	6.660795162
11.64244603	11.44643795	11.64244603	11.83072395	11.93178106	11.56988377
6.478787505	6.630903202	6.672823111	6.581970424	6.632101727	6.425764848
8.425403333	9.206631485	9.044046343	8.83754499	8.765765323	8.883993253
6.909401626	7.096649162	7.404680798	7.466652071	7.191807606	7.313451111
11.37131374	12.04990566	11.46320141	11.68038318	11.73271713	11.42787377
10.38329808	11.5612151	11.81018832	11.7894918	12.06397065	11.72592113
6.23939997	7.020975202	7.019697232	6.97940904	7.148452788	7.33350399
10.8947391	11.22119175	11.63837311	11.42371373	11.31053596	12.10694646
10.46766096	11.51465712	11.48459908	12.02514953	11.12151875	11.51647662
8.211847566	9.000069889	8.738283333	8.838495424	8.759757687	8.984231152
12.75784116	11.88375452	11.97196754	12.38794592	12.18492446	12.67378338
9.547339828	8.939019091	9.077100232	8.754900343	9.231946182	9.790672212
6.556248091	6.861931182	7.11316801	7.386763141	6.996514071	6.953378071
8.316969364	7.013137848	7.354199768	7.680240364	7.473746677	7.592388485
9.45706402	9.996420616	9.657223606	9.766921414	9.938475404	9.644080808
9.377330121	9.89348604	9.785983535	10.39968902	9.829119768	8.909404788
7.164403717	7.914965525	7.649926505	7.53302901	7.952029848	7.958393384
11.23641801	11.9982543	11.52448673	11.23116994	11.47127063	11.38775416
10.16454544	10.29432836	9.846508495	9.395177889	9.511202364	9.59460096
10.17122401	9.720489222	9.719830172	9.406893465	9.789981192	9.723272263
7.123768636	7.669657737	7.942071303	8.247392788	7.846324646	7.152687384
7.30728296	7.143674071	6.427492121	7.488894061	6.768722596	6.806225091
8.421350475	7.969427899	8.072924455	7.976116929	8.099466788	7.761767192
8.554027364	9.47593803	10.40810668	11.03452834	10.79686873	10.48310631
8.084275677	7.715405939	7.826840444	8.282438737	8.179951646	8.516793455
11.26623333	12.00086001	11.74439188	11.46145043	11.52238659	11.55524693
7.836508475	8.531247657	8.285708747	7.864861071	8.091323071	7.982442283

6.741974778	6.429848434	6.691325636	6.984018879	7.339805162	7.291982576
14.84412351	14.6970922	14.72870348	14.74028573	14.90726461	14.05136004
7.133298475	6.963620111	7.572483949	7.719466677	7.199742475	7.589888475
7.940608939	8.303793737	7.773582384	7.529949465	7.679285535	7.726392606
10.36687712	10.67939021	10.59809845	10.30058205	10.3090972	10.66342196
8.667554273	8.554528737	8.457898556	8.610845737	8.516311424	8.397853727
8.753420071	8.975838535	9.096395636	9.240719434	9.629101929	9.170513566
7.506434626	8.956909848	7.698928879	8.066048727	8.965143333	7.738613697
14.4427031	15.29549959	14.99939481	14.88238784	14.74028573	14.65397593
8.778117444	9.61181703	7.925310818	8.170129495	8.209054152	7.988582505
13.9071606	11.62059475	12.14789109	13.22156044	13.03496614	13.0147344
8.918202434	8.543234566	8.924680667	9.388912091	8.805485081	9.036076414
10.13580598	10.2026692	9.945028121	9.530933293	9.819688798	9.868690293
11.02700786	11.73070023	10.87637444	11.60888892	11.76804793	10.85923156
9.36748	8.190781646	9.122138505	9.925979414	9.874496818	11.76101506
14.46162431	15.46469304	15.02501841	15.26374094	14.86987024	14.93426708
8.35018502	9.122138505	8.421350475	8.726024485	8.429130081	8.404822414
9.552159131	11.08756575	9.372025394	10.2026692	10.30470526	9.817379515
7.577683121	9.404880263	7.313451111	8.803935202	8.591239465	8.38301802
9.751885293	10.07107569	9.046362323	10.32531651	10.22361636	10.17224075
8.748883111	9.894213939	8.396963808	8.90531298	9.242526283	8.757866131
11.19293398	11.90152869	10.35385551	12.0750029	11.90674449	11.29230964
10.06173934	11.09089144	10.20859114	11.25898056	11.31661678	10.86173253
9.099925818	10.4994288	8.532980343	9.589694828	10.06450841	8.987499273
8.469339576	9.454390232	8.872352707	9.312727606	9.143918798	8.871209182
14.04335209	14.89443037	14.05855866	14.61202705	14.38889659	13.98831971
14.23242051	14.16629104	13.82538175	14.5513462	14.31917195	13.65450155
13.96779242	14.03424604	13.46712151	14.21449649	13.80704919	13.66158872
12.44611991	11.71895032	10.94786936	12.36473556	11.55106122	11.48061491
17.01900663	13.17281418	14.60195877	15.41210544	15.39581235	14.99939481
6.489542818	6.111914111	6.186368	6.269735929	6.285312596	6.524057606
8.53537201	8.477505576	8.166983455	8.57597697	8.897177899	8.092691677
6.34064704	7.973992111	7.055635202	7.267888182	7.060219081	6.902984
12.3543152	12.62127559	12.39348455	12.52819783	12.85589012	12.18792926
9.677492576	9.747577162	9.736139061	9.972781707	9.945994374	9.544495798
9.101086141	8.277763646	8.906407303	8.77962597	8.65914896	9.176817596
9.164000111	8.41382397	8.705145061	9.117478424	8.693780273	9.319510202
7.848409778	6.979690485	7.333224889	7.751275798	7.418614091	8.718769576
8.065153556	7.322572717	7.780116303	7.586125333	7.606699253	7.964914919
7.696877364	9.205404404	8.893419636	9.194378495	8.603745778	8.003479939
9.244930505	10.55070525	10.93171462	10.66465811	10.48310631	10.44934262
9.769223525	9.59460096	8.152443121	9.347406172	8.210994667	8.238272808
9.154469939	9.276189121	9.167758485	9.636929838	8.667554273	9.130293909
9.180906778	10.40387449	10.81142627	10.1250404	10.9660467	8.608532485
7.114362495	7.055055273	7.502072596	8.055916879	7.905419798	6.571432061
8.147857556	7.027744404	7.350114798	7.919459465	7.458509535	6.922412606
6.646655162	6.445904515	6.882010616	7.238429162	7.221024394	6.225711566
11.02851208	9.166446616	10.11760527	11.05493438	10.96460614	9.263233333
7.370079717	7.431389424	7.757532545	7.235668455	7.490096869	7.198896253
8.897751606	10.08893073	10.15086094	11.05033578	10.75062885	9.473962505
7.563777768	7.253486596	7.60576001	8.386601707	7.957018646	6.970472768
7.802180768	7.154937303	7.654256818	8.130616515	7.841061444	7.826151263
7.034613909	6.929929141	6.947262939	7.766006091	7.307022919	6.509613394
7.831854586	6.973416747	7.265083586	8.542859455	8.317824071	8.76478299
8.494982343	7.918472515	8.276014121	8.400464071	8.569209263	7.581254909
9.432636424	9.145664475	9.777520899	10.01257663	10.31795252	8.534897646
8.719245818	10.74558579	10.70902317	10.83501466	10.80499832	10.77387872
6.473192182	7.072036626	7.41439101	7.679285535	7.406536232	7.293748525

6.751435283	6.368728152	6.351287939	6.364283747	6.25331604	6.269932061
9.781348374	9.509876273	10.01071956	9.751885293	9.715870253	9.267923343
8.072110051	8.437898414	8.002717273	7.77977198	8.022369434	7.943134
8.217984283	8.185563303	8.401317182	8.346198293	8.246470545	8.532980343
9.61181703	11.07956266	10.56421743	10.73517618	10.88430477	10.58236152
8.852092202	9.582110717	8.618800343	8.972927475	8.888766747	8.448456354
6.778730485	6.415875869	6.860943283	7.429246232	6.987033364	7.528303323
12.18792926	13.16151113	12.43279956	12.92937388	12.89886333	12.13538265
7.102982434	6.64964403	6.834065141	7.29540803	7.055909434	6.860179071
6.957895121	7.009733586	6.914008404	7.08018898	7.168103949	6.976769172
11.60888892	12.59668127	11.8358519	12.84627851	11.85301976	11.52448673
10.68687892	10.47370876	10.18098661	10.83642757	10.78408516	10.45754412
9.746145949	10.91766747	10.38631174	10.75062885	10.78551178	10.51771241
7.174922929	6.796104869	6.769202101	6.990730697	7.433629313	6.795141737
9.263233333	9.130979939	8.778117444	8.954176293	8.611286687	8.630877737
8.992473788	8.808928141	8.929972798	8.598479657	8.802954374	9.006527424
11.77802923	11.96059797	10.57758511	8.508645727	9.25368898	10.23660558
7.324298343	7.053922394	7.376443525	7.697220202	7.507381747	7.741791646
10.24612484	10.27139209	10.03611568	9.992988556	9.766131535	9.843921515
10.77890896	9.505859384	9.487680697	9.174793152	9.741357455	9.21905796
8.787164939	8.762742172	9.562994313	9.395854121	8.682101313	8.817758687
6.467370697	6.059903081	7.056197182	6.347255636	6.409900091	6.179354162
8.228066545	9.041820404	8.103690354	8.070136677	7.909412687	8.379510323
13.5160991	13.83772389	13.81935116	14.24162707	13.69473258	13.36582475
5.889471707	6.303681202	6.548684566	6.561721071	6.591366	6.659100485
11.11714857	12.04160066	11.77053658	11.93486375	11.91912022	11.81018832
11.69658017	11.78725933	11.98440811	11.10460602	12.29548604	12.46055504
6.696473434	6.526565121	6.301539283	6.575656404	7.55499601	6.616392283
10.85100254	10.15283227	10.33596201	10.79145221	10.92485902	10.33596201
10.12408141	10.1752482	10.20164867	10.5104744	10.3810842	10.03492192
11.25523353	10.89604789	11.12151875	11.46320141	11.46724679	10.97185531
13.48401144	14.30038792	13.23864174	14.00297944	14.77222727	13.50411017
9.608350808	9.109120374	9.033394545	9.379180747	9.312727606	8.768300525
7.23452504	8.10683704	7.885002121	7.550541152	7.426469556	7.847720859
9.017695354	8.242002535	8.400903717	8.530767747	8.342368121	8.389243343
11.19966733	11.76336308	11.18800276	11.74935785	11.65261158	11.65918316
6.537080394	7.011783192	7.802503596	8.061773232	7.271701444	7.143674071
8.291166909	9.407554697	8.639872707	8.984797889	9.447991384	8.823889374
6.176177303	6.389043091	6.237206657	6.254539838	6.363888626	6.341887717
7.279410566	7.106592788	7.44112097	7.746135697	7.165793091	7.821971636
6.218902788	6.294120212	6.480794707	6.402478545	6.168490424	6.861931182
9.37540303	8.570262	8.458294828	8.689808788	8.58807297	8.120795899
7.770465818	7.31937996	7.23452504	7.376100929	7.308421545	7.136586475
8.170129495	7.088518818	7.250899535	7.370079717	7.170490939	7.400588576
6.674468929	6.941709929	6.540929384	6.639726192	6.606673949	6.477739737
7.548285091	7.383097848	7.211808657	7.370950798	7.223856	7.420173404
8.701109788	8.422204465	8.472925747	8.099466788	8.000521869	8.109636222
10.23550694	10.85513517	10.68452674	10.69793218	10.55602586	10.52364746
8.325717687	9.227275152	9.399129424	9.772980061	9.89348604	8.746311293
8.273878929	7.238133434	7.372532788	7.628690051	7.736260545	7.812327374
8.83754499	8.41335802	9.258612929	8.900779434	9.084956182	9.809529798
11.38178745	11.02551486	11.1612024	11.48459908	10.98350754	11.74439188
7.773938747	8.520754616	7.920494253	8.273483707	8.418959303	8.192266657
11.5737214	11.54871614	10.83930015	11.06732533	11.16423752	10.60044575
8.845226141	8.490053091	9.146732444	9.30577901	8.890373556	8.562227051
9.300983919	10.04600105	9.772980061	9.465071586	9.826793596	9.623794152
8.807484626	8.589388657	7.510343899	8.706567556	8.527598192	8.660608859
9.459664354	9.675185677	9.008956374	9.559682182	9.560346242	9.142159707

6.576096444	7.42612497	7.166060222	7.393489313	7.162716495	7.051418091
7.041898303	7.186327495	7.506099556	7.576687677	7.54731098	7.790604293
10.33252085	8.297224525	8.686126354	9.310775172	9.35858599	9.374690616
8.502509434	6.607797263	7.048729333	7.344013535	7.347416111	7.582654121
5.913077222	6.424467222	7.967524909	6.409677646	6.943317879	6.760820788
7.268488485	7.105225354	7.460591212	7.019921606	7.447087758	8.100639
10.16743096	10.80239406	10.65634987	10.3494333	10.3380268	10.17789322
12.16942168	12.41033676	12.63888703	13.21058954	12.32364634	12.3543152
11.63174178	12.80949032	11.75908761	12.61090773	12.42233701	11.95261936
14.62217632	15.31193978	14.7528858	14.6970922	14.89443037	14.42673578
7.849463182	7.134092121	6.552305929	7.073657889	6.961503899	8.18481599
6.015743101	6.892562737	6.319077384	6.247479727	6.723761636	6.283168747
8.526649808	8.912452162	8.716387192	8.541524636	8.528049141	8.147857556
7.918472515	6.969423263	6.752731616	6.920433444	7.035678848	6.957765889
9.54312803	9.140392293	8.998420707	9.690534253	9.385290182	8.049757475
8.324453253	7.459405444	7.698638758	8.110881556	7.87816304	7.92237903
8.540635232	8.21550303	8.009790253	7.890057202	8.32939098	7.991889949
9.826032111	9.151976485	9.146178758	9.329342374	9.491725303	9.06557301
10.83642757	11.40145091	11.04156144	11.55106122	10.92608881	11.36610866
10.57996368	10.60161194	9.929312889	10.72127155	10.39521192	10.39296715
8.804952828	9.922126404	9.831087364	9.493200828	9.602112616	9.438094949
9.089024899	9.09178804	9.36439596	8.947623838	9.259301556	8.882379495
13.16824615	14.36999747	13.6823979	14.36999747	13.84455043	14.14208591
7.581657414	7.558170465	7.470329323	7.995268899	7.391952354	6.853461566
7.722652869	7.186028475	7.203497747	7.048729333	7.407119727	7.091067131
8.130283051	8.298004222	8.285312424	8.836023374	8.53715797	7.613240606
12.94367069	12.61449239	12.56315114	12.32659908	12.39973853	12.06692054
5.912062667	5.862760364	5.996844343	6.176996141	6.988399394	6.072324222
8.614442374	8.335469222	7.997183333	7.487647384	8.049354232	7.948737212
9.282486081	9.076382242	9.712341424	8.958493495	9.193817232	9.954681949
9.186096828	9.411394242	9.267383556	8.483763566	8.541972566	8.863375313
12.45390404	12.65104366	12.63500423	12.7452022	12.74093701	12.64708972
7.128516444	7.550541152	7.889381343	7.896600606	8.069753788	7.706168626
9.634846707	9.212980727	9.725631121	10.02161965	9.638947465	10.10542249
6.802811828	7.483852838	7.237838192	7.307847101	7.334639071	7.262957343
9.283736939	8.328154263	8.234636465	8.12156602	8.511758747	8.053207788
10.28710845	10.99469779	10.88302044	11.1502402	11.19293398	10.91641235
6.139904273	6.516018636	6.507801384	6.944866586	6.528405747	6.538933859
10.26541026	10.16546821	10.29851595	10.30810853	9.865550636	10.61371787
10.0188097	9.529469889	10.03796113	8.708995576	9.030004576	9.435722162
8.226165	6.118809313	8.317411434	8.065153556	6.276206182	8.253254586
8.601386131	8.006153172	7.996770556	8.08193004	8.195856758	7.810980737
10.09682592	12.32364634	11.51008986	11.86401838	11.51647662	11.91416468
6.706660646	7.894965162	8.005727263	7.956286283	7.772843323	7.74007802
6.293534101	6.508047626	6.512183152	6.444835424	6.38878402	6.518333111
8.993505475	7.849137545	8.861728919	8.487847515	8.211847566	9.380979222
6.985120646	8.162707626	8.642670768	8.345781111	8.249350414	8.099112939
10.50794857	10.62280049	10.80389925	11.04156144	10.56207051	10.84302834
12.62449502	12.42233701	13.12562627	13.02518615	12.38794592	13.50997822
6.956131545	6.993463051	7.192707293	7.448962859	7.144887111	7.041650374
6.751729354	7.913871889	6.907588505	7.035678848	7.033625222	7.584276586
7.159147152	6.694471303	7.240600646	6.980238687	6.942817626	7.819085697
7.618887111	8.347358778	8.159907626	7.727390434	7.798826303	8.004282899
8.667049253	8.421742051	8.757866131	8.976374343	8.816703535	9.155642525
10.52135963	10.68452674	11.19638858	10.90754603	10.77103399	10.20164867
6.700834859	6.421175192	6.639045768	6.860179071	6.687954879	6.093319636
7.806845253	7.901917293	8.010523545	7.629344343	7.771540485	7.627373273
6.788076343	6.539575101	6.992927808	7.024510747	7.180559343	7.273507545

9.737663949	9.769223525	9.347406172	9.601568657	9.425521616	9.150811232
7.662615848	6.974671657	6.919392394	7.526779747	7.151291707	7.413750677
7.169469697	6.720058788	7.096340343	7.281724869	7.159962485	7.126519737
6.608949242	6.66784103	6.897750404	7.02555202	6.548235576	7.411868313
6.87945998	6.203185697	6.215353354	6.540929384	6.355908172	5.921030929
11.06433979	12.31719428	11.15966862	11.87175588	11.96362869	10.99623862
10.33375706	12.31089415	11.53050203	11.9091429	12.0089388	11.68484165
5.913884121	6.477312747	5.78592496	5.847759152	6.26253897	6.465818222
8.742149222	7.765675424	8.31049196	8.469339576	8.445297313	9.127872697
6.624377646	6.869634889	7.155245384	6.408579424	6.554596747	6.881026485
13.45466248	10.82804231	12.0724233	12.58287167	12.12939166	13.44849295
6.730688788	6.794399263	6.808998949	7.114645596	6.614504818	6.498091727
6.612755929	6.536426172	6.734736768	6.832125364	6.692767899	7.094132182
9.400327152	8.427236737	8.302498081	8.760291222	8.908935182	9.395854121
10.78269205	11.47871073	11.66189432	11.31449746	11.42371373	11.33100665
12.22629088	11.26815786	11.55106122	12.27773509	11.56988377	12.97555423
7.991026727	8.24122704	8.433939838	8.495447152	8.200961636	8.685647889
8.150892606	9.439397061	9.084956182	9.143279535	8.96575796	8.920488949
8.339546939	7.473746677	7.751275798	8.146343545	7.832217929	8.643636162
6.209590788	5.969349343	6.611655717	6.391416354	6.278463949	6.546202222
6.980492495	6.793493859	6.694471303	6.948610667	6.785769293	7.051121485
7.356281616	7.222163485	7.952774121	7.173915838	7.283800475	6.855446939
6.511948545	6.195258121	6.226967616	6.22397	6.215679889	6.032452212
7.10996603	7.976116929	7.85268101	7.692217293	7.664565879	7.797844434
10.23351878	10.81540317	11.05356995	11.04030995	10.60161194	11.15183093
11.31267384	12.37087917	11.67849095	11.89130603	11.65455063	11.78023213
8.156410909	7.749299101	7.925642	8.119679141	8.174069929	8.819236758
7.533990333	8.004282899	7.820605525	7.915639949	8.063898495	7.861832121
10.48659409	11.14385742	10.72900488	11.29744903	10.95849458	10.79564384
9.681893121	9.606954152	9.693515768	9.387059283	9.502644929	9.437491071
7.892604899	7.715785354	7.929593313	8.025864485	7.861832121	7.940978939
8.492322828	9.087865687	9.35728196	9.363307667	8.928828384	9.361074667
7.20918101	7.433328091	7.503050071	7.237206848	7.088938904	7.620272192
7.353560545	6.855940818	7.08160301	7.078835444	7.145208253	6.995550687
10.1838089	9.768390455	9.924448444	9.608350808	9.852239606	9.554043101
7.914965525	7.55499601	7.307562737	7.047335818	7.295094879	6.939486697
8.527198758	8.889380424	8.972363838	8.868979273	9.091299889	8.638952889
6.911480061	7.965251131	7.974325596	8.004282899	8.287888051	7.74646602
10.08700287	10.46232362	10.58935103	10.31193963	10.11349335	10.45375986
8.994589434	8.443956768	8.208692758	8.207537162	7.972012263	8.248137606
7.592689374	10.20060626	9.20200202	10.21484784	7.208321485	10.20859114
6.932737172	8.240375404	7.335553323	8.289212586	7.278115626	7.792587404
8.27903296	7.578628212	8.098302202	8.276438626	8.076118121	8.233006697
12.62127559	9.698896848	10.28506122	11.30506756	11.32721575	11.28370047
7.077700404	6.95944596	6.284925121	6.912025515	7.447717303	6.522534343
9.885156737	9.545191596	9.830354566	9.734703111	9.448606737	10.08521343
9.155642525	10.9102587	10.29324401	10.46664928	10.58689262	10.36032821
7.325162263	8.178268222	8.296772869	8.114219303	8.153159131	8.173633596
7.933221424	9.615324495	9.75438504	9.627745303	9.753529758	9.013830768
9.183367525	9.901640636	9.123297364	8.613130333	8.806450121	9.35728196
7.817596162	8.325316889	8.207537162	8.024267525	7.847379061	8.31049196
8.392711081	8.150448232	8.440058758	8.485174677	8.321152475	8.339546939
6.445904515	6.889757091	6.877874525	7.20183702	6.886441253	6.655235768
6.353208646	6.702267949	6.807214747	7.121885657	6.819670818	6.441255838
12.47441306	10.67081044	10.70775781	10.84705809	10.92190902	10.79686873
9.753529758	9.221852535	9.257324	9.349388778	9.219562515	8.96575796
7.942071303	7.171589687	7.492628778	7.240898899	7.553773697	7.503972212
6.304241131	7.571747222	7.81270001	7.220780818	7.555972677	7.536878778

6.781434051	6.482522545	6.919943293	6.25331604	6.442315303	6.413218091
9.146178758	7.704350384	8.239938859	8.397853727	8.151663141	8.774537848
7.766006091	7.422534091	8.338686465	8.716387192	8.580722586	8.733083212
7.272016424	6.785769293	7.219933525	7.132751192	7.188870657	6.933276949
8.452737606	8.417604414	8.689808788	9.002847848	8.85518403	8.375190152
7.502411182	6.658852677	7.242080394	6.812168303	7.240600646	6.886152545
5.954254778	6.067102263	5.813789717	6.111258566	6.053020636	6.081802747
7.263531485	7.084441778	7.321884212	7.724673141	7.560760293	7.846324646
13.99495772	9.402230293	10.16454544	11.07337622	10.8405808	11.42178201
6.952257202	7.439228222	7.791533949	7.718373848	7.473428394	7.770819434
7.334639071	6.767959131	8.006915303	7.722652869	7.672658859	8.059423535
7.312858101	7.227602475	7.045715273	7.261194687	7.143674071	7.247912131
9.678297182	8.983202444	9.044608384	8.494576061	9.039454747	8.591651626
11.94979793	10.54950911	12.00086001	11.43380494	10.94071069	12.84627851
11.01951417	10.15875628	10.63752253	10.63391466	10.42736719	10.61743608
12.04734694	12.78645268	12.61761735	12.62127559	12.6076243	12.28693608
6.737098263	7.238429162	7.119927869	7.365381232	7.072618697	6.97809596
9.945994374	9.907546465	9.853021111	9.95201103	10.07764109	9.928455091
8.832790172	7.476561202	8.378142071	8.815152444	8.129874121	9.759214424
6.301337414	6.247784949	6.370955485	6.521900899	6.547549889	6.663676879
7.082724758	7.083013909	6.977291071	7.284105152	7.122942091	6.638128919
7.878949444	9.249034202	9.789981192	9.659537192	9.151976485	8.483326677
7.93888704	8.293340606	7.891912283	8.366003535	8.496814303	7.834378071
8.915814212	8.738283333	8.899757485	8.269818727	8.238629646	8.64507902
9.322592778	6.15234603	6.723255293	6.861656576	6.81111	7.956615091
8.472435657	9.077100232	8.612168455	8.676791091	8.724013404	8.468898455
7.232737253	7.864224778	7.482605354	7.639662323	7.969427899	7.601304687
8.43006898	8.352134333	8.786134101	8.78664797	8.725555596	8.844262616
9.763952101	8.184050869	8.663303283	9.436898869	9.40167604	9.415860727
7.872350636	7.373983485	7.689234404	8.189547909	8.021628354	7.999327475
8.696280707	8.350619919	8.735287485	9.253086273	9.061091758	9.189590182
10.67190595	10.0672481	9.845595727	9.897493586	10.32309273	10.15875628
7.898205273	7.60282502	7.478150263	7.949071818	7.364797848	7.040799717
7.36510997	6.997357515	7.100167172	7.357775939	8.171788646	8.406205495
7.180559343	6.849574919	6.998984111	6.985416596	6.849836323	6.95944596
8.73262002	8.218433131	8.933684374	8.598980253	8.984797889	9.050797061
8.068931465	8.054318061	7.821625848	7.363288879	7.719108384	7.436027273
9.41003803	8.099466788	8.420387707	9.227886646	9.240719434	8.488340929
8.397853727	8.358418384	8.229339455	7.129597273	8.088528121	7.757833798
7.548584576	7.431067202	7.327234414	6.79621954	7.02555202	7.087210828
11.6137143	12.62807191	12.04734694	12.23769992	12.28985985	11.79615897
9.09178804	9.881176566	9.472607081	9.348735909	9.734703111	9.463745455
7.765675424	7.883157929	6.954196414	8.166602566	8.161033919	7.548584576
10.66945215	10.34626898	10.66105788	10.3380268	10.64502212	10.36134852
11.44643795	11.18471248	13.56234785	12.41337695	14.76275492	14.68633385
10.82290783	11.16579624	11.38577951	10.93646713	11.45002291	11.28207759
7.992269263	6.832363455	7.099269525	7.359287172	7.210358909	7.528986283
6.930987343	6.141021192	6.271221162	6.544328465	6.654064545	7.224144889
5.889266646	5.807472949	5.849231121	5.830483111	6.737819111	5.761617717
11.49029795	11.72355631	11.5386618	12.51424883	11.99535117	12.33576886
10.04160563	7.675893758	7.937868929	8.435650677	8.233006697	8.592622071
9.16271003	9.62845904	10.45996909	10.1838089	9.772980061	9.655088667
6.45190396	6.94531798	7.121605929	7.416977434	7.427701768	7.111681374
6.512183152	6.31629898	6.483130091	7.243557061	6.852390212	6.590449828
9.129114848	7.941320778	7.871272152	7.561964414	7.094132182	6.785769293
5.785368111	5.771766717	5.788590465	5.769391505	5.77816901	5.797031404
12.09225922	10.42409385	11.74935785	11.43565115	11.37670858	12.21630458
8.551751717	7.387388131	7.858964434	7.958031758	7.742113232	8.058246657

9.673359929	9.126663535	8.864955909	9.405539273	9.438799404	9.537497323
7.768402071	8.305787747	8.341518747	8.402148465	8.692870869	8.242002535
7.623059667	8.200163707	8.885078273	8.650384111	8.616405606	8.158602879
6.593877636	6.453641556	6.580361485	6.689141323	6.569103444	6.42621898
10.86173253	10.06633871	10.75737971	11.44643795	11.05356995	11.07493153
9.70574997	7.966402293	8.356302828	8.914720172	8.843670869	8.87289497
6.293110242	6.75007699	7.29540803	7.256596263	6.908074727	7.042643192
10.21583282	8.845226141	9.303966081	9.561667293	9.54312803	9.555296242
9.565660949	10.09597734	11.04316622	10.85513517	10.33890802	10.79420974
8.439112919	6.950648455	7.121365859	8.864430475	7.729749768	9.000069889
10.56306707	11.04464523	9.474605101	8.08537204	8.425847798	9.883591758
8.122341	7.377269152	7.454874889	8.286128505	8.188779434	8.468064667
8.101780545	7.659186848	7.978709162	8.817758687	8.630379313	9.124449354
6.193578313	6.589360121	6.370541444	6.676440081	7.011783192	6.251546414
8.019097616	9.27419499	8.662816202	8.918202434	9.337862091	8.980943495
8.244006505	7.877487515	7.289911515	7.673376626	7.818322879	7.663618646
9.231349182	8.773988747	8.061425949	7.387654737	8.127056495	8.320817869
8.984797889	7.495088838	5.827722626	8.151663141	7.954939636	8.973478152
8.794361798	8.359263212	9.136296333	9.171164091	8.81572204	9.022478232
7.951633808	6.933815889	8.05474504	7.889031475	7.606043535	7.873080636
10.27338352	11.71443529	11.56988377	11.28370047	11.1060681	11.20163629
9.369545939	9.853021111	9.311440606	9.607593848	9.686378667	9.776839404
6.965960869	7.540434798	6.41992303	6.766746646	7.414724596	6.738515899
8.995584808	10.31095185	9.235747242	9.62302801	9.267383556	9.552159131
11.21958266	12.0089388	11.79405389	11.95514832	11.59427705	11.58421758
10.57159251	11.42371373	11.22600179	11.38775416	10.94942311	11.24646499
8.902484929	9.834210131	9.776037091	9.691258919	9.168324747	9.550769788
7.167545535	6.794655232	7.137147091	7.235366172	6.974196465	6.683063909
6.431354778	7.610976808	7.034081253	7.004539727	7.415639222	7.08018898
10.02741959	9.056533626	9.488337717	9.556000646	9.308265131	10.1250404
13.27418186	13.90036211	13.92172837	13.67554111	13.56886427	13.48990673
6.520315747	6.593877636	6.542523919	6.612060818	6.476450515	6.703235576
13.83173935	12.53911948	13.5360606	13.44126502	13.18348162	14.1282639
14.40789433	10.61261659	11.57567047	12.48458958	12.21630458	12.48124823
16.82803465	12.96241124	14.52085241	15.21350605	15.36066179	15.10875812
6.555298505	6.921891778	6.86986903	6.841133828	7.266813283	6.823384798
7.239990273	7.918105152	7.673764162	7.28082202	7.432999727	7.508388697
10.12909315	9.276789111	9.615324495	10.85225864	10.29660096	11.46320141
6.525371737	7.387654737	7.764609818	7.445746576	7.381652111	7.207790596
12.19727311	8.77085696	9.387059283	10.44195016	10.14432724	11.18941159
17.12376032	15.48449671	16.17943014	16.68963031	16.4534382	16.33688632
11.33477589	10.53141254	10.50057506	10.14895288	10.58117749	10.31378621
7.854005263	6.884322333	7.804724687	6.468965343	7.141146879	6.70156496
14.15863941	15.05177986	14.63205333	14.38889659	14.40789433	14.31917195
7.421928768	6.779669273	8.177528808	7.372842667	7.132177788	7.732550404
12.66985986	12.90783816	12.54906863	11.91651973	12.55625498	12.26775966
7.061925202	6.939947626	7.506719576	7.644277273	7.394370293	7.433328091
7.66930299	7.920494253	7.576363505	7.274624242	7.519695475	7.674109172
7.291703707	7.457078101	7.402015111	6.956131545	7.092673939	7.133560273
9.094076404	9.421059	9.273022949	8.651919778	9.005975162	9.080123545
8.359650919	8.047553323	7.935081758	7.950819283	8.267244596	7.757234778
9.418269535	9.333986848	9.94767803	10.14534091	9.529469889	9.218466939
8.59942799	8.904793061	8.617840212	8.195856758	8.552719747	8.701109788
11.05493438	9.49510903	10.16937171	10.62536143	10.27139209	10.95093974
12.65819872	13.20536123	13.22156044	13.13007894	12.9851923	13.27418186
9.200683333	8.897751606	9.061649657	9.245445293	9.101613848	9.391460545
12.31089415	11.54053762	12.88708268	12.21312889	13.48401144	12.39058663
12.19398134	12.03068847	13.80033164	13.10985871	12.43944324	13.69473258

7.135797152	6.817996071	6.784609556	6.986766929	7.018669091	7.09298796
6.587993828	6.641378788	6.376515232	6.534012141	7.159453505	6.61686503
6.798572747	6.389460141	6.566291384	6.744595263	6.535075677	7.005077596
8.431738505	7.853350485	7.694191859	7.815532475	8.12156602	7.695611414
11.60069924	11.73271713	11.34576518	11.27306561	11.49029795	11.35858946
7.36166898	7.47214603	7.546292778	7.640998838	7.507037566	7.123473687
7.073657889	6.221607646	6.234838515	6.64187404	6.592903869	6.606886212
9.591816061	9.605576293	9.815893677	9.888393747	9.445852485	9.927686616
12.17216641	9.829822242	11.81221198	11.27695531	11.62059475	12.81379045
6.949608808	7.354804152	7.864861071	8.158321101	7.748548515	8.443175586
8.913024061	7.759260374	8.414282596	8.328992859	7.959488687	8.560860505
8.864430475	9.752732475	9.516398909	9.260607	9.421059	9.141588162
9.361671283	9.257979848	9.66613299	10.31997281	10.05144087	9.409395929
8.438325768	9.455711606	9.439397061	9.995698596	9.315671101	8.236986626
9.912769303	9.689783586	9.733230051	10.38842688	10.22951336	9.239472869
8.825312061	8.214296636	8.389653889	8.72650097	8.381754576	7.986161141
7.305875394	7.528004313	7.156711091	7.671246505	7.314025869	7.840676768
12.96662998	13.7211242	13.42888134	13.44849295	13.47327752	13.12023156
12.83405786	13.42888134	13.37822002	13.86604428	13.64083669	12.93430142
9.771430818	9.278597747	9.52752797	10.2761618	10.08978265	9.392082222
7.572483949	6.810307121	6.986482212	6.539575101	6.806487737	6.465158313
9.960013465	9.663106646	10.156858	10.49713487	10.20748734	10.23948175
9.966143818	10.12224713	9.908305848	10.43655428	10.21769501	9.950180667
8.639417101	10.10093339	9.882779162	10.08231743	9.675185677	9.025193869
8.289599333	8.652374404	8.188779434	8.452374444	8.328154263	8.096128192
9.168324747	8.049354232	8.963499596	8.555429434	8.223899788	9.276789111
10.15585749	11.43938222	11.2608846	10.84974643	11.20163629	11.1502402
6.333400636	6.546871707	6.355148343	6.789537253	6.644999202	6.047425707
11.06877356	11.71244686	11.42787377	11.68700199	11.11856721	11.36245739
8.012698303	7.670568384	7.945472162	7.888681737	8.041792596	7.673764162
7.214922364	7.04545701	7.423442323	7.880363606	7.542064859	7.420458273
10.85225864	11.4090051	11.27695531	11.10035391	11.2786408	10.99345288
8.310875455	7.871683444	7.709201212	8.005727263	7.92597801	7.608347495
8.936938576	8.503061444	8.679110091	8.898231131	8.862225737	8.234636465
7.583692414	7.74007802	7.126812859	7.677935313	7.165538758	7.203497747
6.818722313	7.102414636	6.82018896	6.839911636	6.780968242	7.243557061
6.800063838	7.059956313	7.280525364	7.540776061	7.080799364	6.279712293
9.908305848	10.86173253	10.47941256	10.82945836	10.50543333	10.41330878
7.098999354	6.713056273	6.872396859	7.348618394	6.610905657	7.139090576
8.592161788	7.871272152	7.673376626	8.295436051	7.960941889	8.786134101
6.993183869	6.202799535	6.188743172	6.209590788	6.057581384	6.397996646
8.60413102	8.319524414	8.610845737	8.07651197	8.344441616	8.452737606
9.777520899	10.57028524	9.647889364	9.624391323	10.56908161	10.15283227
9.433258586	9.184000242	9.173615515	9.286739242	9.284395657	8.964085616
8.959665061	7.706567859	8.59655801	8.282900293	8.231756293	8.566871343
8.963499596	8.791657869	8.703733141	9.06333597	9.070661273	8.665165081
11.84291489	10.83501466	11.61117883	11.36065784	11.68038318	11.33477589
9.654354424	9.895061889	9.981718727	9.776037091	9.992117576	9.946747141
6.410521475	5.795117556	6.357923899	6.503188616	5.93846297	5.991022919
8.508645727	9.489614919	9.222631263	9.336479586	8.999492384	9.022000869
8.39227803	7.951199808	8.115437899	7.460903263	8.081502051	7.849463182
7.610260323	8.598980253	8.425403333	8.315227333	7.71023803	8.084275677
9.103235919	10.02741959	9.900778232	9.685649929	9.885156737	9.942152202
11.20345844	8.854603364	9.466304111	10.70775781	10.49713487	10.81280665
8.977558859	7.809001414	7.647964475	8.239474646	8.104866828	8.106019798
11.02105469	8.984797889	8.919862162	9.862946677	10.37686258	9.443424081
7.94734596	7.836828747	8.307093606	8.207926798	7.986161141	8.168587596
7.218159515	6.810582646	7.209485919	6.767959131	6.938149939	7.309928404

15.05177986	12.09804166	13.12023156	14.16629104	14.27397716	14.06476988
10.14713571	9.961780586	10.5752557	10.19236777	10.23160947	10.79284767
13.17281418	13.32669092	13.10985871	12.83827393	13.26276274	12.90359001
7.115131646	7.02200603	7.356542485	7.418256545	7.596246919	7.569899606
7.594027929	6.625506687	6.579391333	6.893883192	6.674983667	6.738245586
7.863195747	7.472500828	8.046433182	8.308776677	7.946541949	8.667049253
10.65127749	10.54692936	10.41957053	10.33596201	10.76737368	10.68338253
13.18348162	12.19727311	12.09804166	12.07810634	12.60396528	12.19398134
9.11393401	10.07479617	10.31013098	9.890949131	9.752732475	10.45877561
7.566119051	8.24281297	7.97830402	8.254484434	8.061773232	8.184050869
16.39933956	16.64557528	17.01900663	17.23317693	16.8710039	17.23317693
9.315671101	9.264989899	9.02886299	8.791137414	9.229670424	9.12042397
6.842776727	6.512682823	6.509844303	6.928307859	7.051685909	7.304711495
6.841594566	7.123473687	6.775968394	7.02200603	6.836530859	6.86860504
6.643553717	6.653328071	6.482290162	5.878796525	6.125146475	5.982220909
8.943861384	8.31318196	8.65914896	8.705145061	8.60808904	8.57971698
7.161342051	7.116047475	6.685554869	6.953378071	7.514105485	6.840910293
7.127106434	7.001612657	6.913511828	6.72401001	6.777485293	7.350971899
6.514109778	7.279952616	7.765298909	8.074508444	7.688011354	7.637927172
8.362142131	7.535195111	7.57318396	7.849463182	7.722652869	7.864224778
11.06261544	10.29752835	10.94496034	10.69354222	10.19957169	11.10460602
13.63435472	13.85099976	13.57574016	13.29117716	13.79381649	13.5160991
8.976374343	7.488273475	8.473802646	8.164979273	7.917403434	9.349388778
9.90675497	10.06997663	10.73517618	10.74045328	10.40387449	9.002847848
9.21905796	10.87518762	11.37670858	11.19114259	10.75178718	10.80499832
16.57174008	12.42943771	13.60269863	15.18392271	15.13975511	14.70594079
6.892837172	6.611373919	6.783369808	6.905589111	6.403298808	6.773706576
6.727438899	6.720541949	6.147039899	6.722042727	6.468260505	6.582941808
7.104930788	6.565804798	6.328925929	6.517856677	6.520559131	6.909148313
7.240274404	6.763620737	7.585232141	6.978866758	7.239071505	7.069444
14.88238784	10.38329808	11.54470354	12.54906863	12.25844332	13.05428976
11.54053762	10.94786936	10.92880859	10.99345288	11.07647857	11.44643795
9.394567081	7.326634626	7.293153485	8.097222414	8.624974828	8.690381253
6.490634808	7.656238101	8.041000495	7.937868929	7.70790696	7.97753898
13.43523914	8.995584808	9.014846778	13.64083669	13.15082863	13.1944969
6.381819808	6.247954444	6.206101182	6.477312747	6.657655061	6.525823616
8.269395232	7.940978939	8.194999071	8.000521869	8.00874802	8.52305899
7.786927414	8.439112919	8.721054424	9.054276	9.044608384	9.402814081
9.582110717	9.870931818	9.191849071	9.048620525	9.200683333	9.10215096
6.099738869	6.808010384	6.82385998	7.132177788	6.96917198	6.66391404
7.555661677	7.641985	6.962822313	7.579015667	7.442908828	7.517772121
6.843295	6.164144475	6.840363778	6.490008293	6.465379212	6.861163889
7.677613273	8.380307172	8.183602061	8.220625586	7.227882465	8.612685364
7.851994586	7.84419497	7.834787162	7.857447394	8.035288182	7.616552222
10.0060683	9.67665103	10.33375706	10.45041513	9.361671283	10.57996368
10.92608881	10.39521192	10.24727703	10.48310631	10.66587479	11.05959848
13.83772389	13.26276274	13.63435472	13.52959744	13.81935116	13.43523914
6.69920203	6.069331687	6.290108071	5.923214808	6.17734602	6.25782001
6.431116323	6.587110859	6.814711535	8.578393657	7.991026727	7.754082929
7.974325596	7.948408364	7.701352778	7.894965162	8.419470808	7.199742475
14.93426708	15.22815255	15.15443825	14.53168901	14.61202705	14.5513462
6.289561192	6.07011403	6.072324222	5.938088202	8.34982299	5.844055899
5.777908919	6.356285141	6.207516667	6.413679545	7.130479657	6.635568162
7.588284717	8.445297313	8.393536232	8.799825313	8.961327374	8.307893798
6.708308495	6.775232444	6.914843414	7.487078798	7.543741273	6.56618849
7.016713091	7.363288879	7.071771354	7.218710525	7.624016909	7.02934998
7.284692949	6.713421434	6.857241172	6.360466222	6.569606636	6.746600626
6.864750879	7.573788949	7.30728296	8.020887778	8.203586121	7.352900667

8.414679525	7.924885333	7.953840929	8.005065495	7.804724687	7.402701172
9.368875727	9.969958172	9.930141081	9.945028121	9.710054737	9.876880455
8.242002535	7.690510313	7.70299303	7.827906879	7.752031929	7.583998525
10.03796113	9.733950273	9.974493182	9.803356646	9.589694828	10.28406896
7.436027273	7.153495505	7.808339242	7.079662576	7.726692222	8.124599212
11.89130603	11.13240418	10.83642757	12.47761486	12.35168631	12.43944324
8.423139949	8.380828141	9.338458455	9.018802576	8.781680606	9.055478465
6.872867768	6.426421657	6.683531697	7.078303869	6.96434804	6.830079818
5.851534677	5.960790525	5.951037616	6.158302111	7.203245626	5.83769397
8.453185455	9.452497313	10.2026692	8.204380404	9.230770667	10.3494333
7.149903152	6.631584848	7.666980273	7.443845071	7.494798263	7.683318778
10.14999886	9.426142444	9.519086535	9.31393701	9.873591818	9.519086535
7.400588576	6.672562737	6.984849141	7.290204495	7.128516444	7.28113
10.86820493	10.75737971	10.37124354	10.3896003	10.55474917	10.54565144
8.021984222	8.03601997	7.835434535	7.957321232	8.167383949	8.271029889
11.78725933	10.72633412	10.86820493	10.8254891	11.05191968	10.94496034
11.32027501	11.59654933	11.71895032	11.27499716	11.06877356	11.86401838
8.479185606	8.315227333	8.034046273	7.776955808	7.961659859	7.667649192
7.923778798	6.852686475	6.830079818	6.86214797	7.475313929	7.29078701
11.39594259	12.4956701	12.42233701	12.40735915	11.9875501	12.14507673
10.57758511	11.43565115	10.99345288	10.96748398	10.90606985	11.11560115
10.40278823	11.20163629	10.78014639	10.93935201	10.76361324	11.00496707
8.528915121	7.273826505	7.367866364	7.609574333	7.927799515	7.997498828
6.593389535	8.015705778	7.512765949	7.789881242	7.756512818	7.601973414
10.42297323	7.281402384	7.960547	9.077686808	9.249612495	8.897177899
8.511305758	9.419654929	9.397864101	8.746767313	8.77085696	8.977558859
9.581418808	9.860587788	9.374121768	8.671294253	9.308918081	9.598265626
10.86566339	10.7532413	10.31095185	10.79284767	10.65246846	10.37026232
8.887109626	8.925792808	8.772410586	8.200961636	8.635209293	8.809442889
7.32157099	7.158043384	6.900957616	6.92802298	6.883018111	7.063222061
8.280295667	9.930141081	9.739859192	9.019731374	10.00886723	9.17294002
11.42562271	11.58005536	11.60069924	11.35858946	11.29947244	11.75908761
5.987975404	6.577737222	5.990636495	6.010869525	5.998584	5.965348202
8.044608626	8.267244596	8.036816444	7.58864003	7.554062253	7.955335374
8.218887434	7.790916071	8.395296687	8.571194222	8.275152535	8.886096818
12.0089388	12.14191765	12.33273925	11.85889751	11.88375452	11.9982543
10.40074116	9.749004051	9.389466253	9.558925838	9.553430788	9.606954152
6.810861495	7.189144101	7.522869182	7.028571808	7.587635919	7.571427586
9.977292899	9.383480283	9.164000111	9.768390455	9.655088667	9.49510903
6.886441253	6.69201996	6.489070778	6.661293222	7.579628838	6.558602434
9.793126455	9.990505182	9.824540899	10.02549055	9.964343626	10.17904437
11.41238356	11.34892675	11.40725584	10.73132595	11.28370047	11.06433979
12.67378338	12.86955734	11.88649013	11.83813795	12.67378338	12.19727311
7.812327374	7.387061818	7.092673939	7.384605828	7.126269081	7.297796364
9.526870273	9.782868192	9.214111545	9.639623879	9.513795939	9.312041414
7.817975838	7.181436869	7.134684455	7.509397909	7.204610747	7.210358909
6.633467848	7.112620848	7.520992313	7.364550071	7.36203996	7.013396273
13.26910509	12.10135206	12.67378338	12.73352188	12.65819872	12.89513437
7.697611606	8.601896768	8.137479222	8.048636061	7.896600606	7.829325051
13.11515098	11.80791686	12.45390404	12.64265677	12.57877126	12.36473556
8.228510596	7.172411071	7.14692997	7.606370131	7.744777657	8.169730152
7.044743657	7.522869182	7.201015091	7.165538758	7.20183702	7.134092121
6.707842586	7.394027687	7.381652111	6.737600111	6.925009455	6.958200131
15.36066179	15.19830883	15.83165575	15.92931651	15.59877501	16.36806566
11.45002291	11.16734497	11.01045685	10.69931975	11.03452834	10.80091358
6.206101182	5.870674374	6.938620788	6.403697939	6.036610232	5.932593626
11.59021288	10.74045328	10.99623862	11.53239215	11.3403052	10.50057506
6.816280253	8.129067798	7.815915242	7.736964596	9.020821455	8.220625586

9.239472869	9.718307192	10.02549055	8.60560303	9.87177298	10.03026493
12.70002263	12.25251148	12.29275278	11.91912022	12.23191382	12.52461444
7.145208253	7.188870657	7.706168626	7.770089636	7.592047838	7.753435232
7.853350485	7.581657414	7.914251515	7.913459869	7.700670768	7.938205586
8.838951081	7.681826051	8.184399818	8.347717242	7.939903758	8.521230828
7.850890354	9.286110869	9.471411141	9.370225242	9.226702434	9.214111545
8.821885465	8.63794701	8.584830636	8.59655801	8.421742051	8.617420111
9.853021111	8.108917222	8.603276828	9.300367869	9.229082869	9.568406747
7.450888848	7.165538758	7.312858101	7.593349828	7.382527051	7.763504859
7.28582396	6.926979111	7.148192263	6.645437646	6.602427343	6.752974364
6.762314343	6.282381596	6.215520505	6.622687202	6.317094394	6.559965303
6.113783172	6.279712293	5.975206535	6.994245051	6.672081333	6.529766576
8.937936253	8.50473503	8.555833323	8.899254879	8.326884343	8.189895404
7.175476838	7.032313212	6.58529104	6.386235697	6.732525747	6.522760475
11.109559	9.295516616	9.941224515	10.81280665	10.48074239	11.26623333
6.670809131	6.249071646	6.146851515	6.241411051	6.100439616	6.320680717
8.214678465	7.583321859	7.392836818	8.008400859	8.128290707	7.196550485
8.237365566	7.695265798	7.398977253	7.083613121	7.251486172	7.581657414
15.03840752	14.91973608	15.01206542	14.47046648	15.03840752	14.74028573
8.445759657	8.548304869	8.486968232	8.367422212	8.647922808	8.152087687
8.283707475	7.991026727	8.267698071	8.722452253	9.023071899	9.763952101
6.968936475	7.690840778	7.365381232	7.290498626	8.416773909	6.830332131
5.883690545	5.929151939	5.886760646	5.851911838	6.778730485	5.873312909
6.885618131	6.256512535	6.224797737	6.245100394	6.376693242	6.57287004
13.7211242	13.20018245	13.32669092	13.46005067	13.66158872	13.88591407
12.57486869	12.0750029	12.16036216	11.51465712	12.09497108	11.82346178
8.042974687	8.254484434	8.254484434	8.314850485	8.462637525	8.700142556
12.57107442	13.22730043	13.03025597	12.54268587	13.10985871	12.94367069
9.699589758	8.728449242	9.161459798	9.905938879	9.460382818	10.24139873
10.79686873	10.78705857	10.37573078	11.2786408	11.98999321	11.41605585
10.20532801	6.844259293	7.040256253	7.355380455	9.561667293	8.347717242
9.096395636	8.644074768	9.235179424	9.031713778	8.811517808	9.417037424
7.797507939	7.20183702	7.274095485	7.510343899	7.770465818	7.432070859
13.12562627	13.07454735	13.1944969	12.70390423	12.72972704	12.85589012
10.79284767	7.768055657	8.708467242	9.214694788	9.226128374	9.464440242
8.321509909	9.045755677	8.378515131	8.977005354	8.972927475	8.667554273
6.456009747	7.232151747	6.515594273	6.654064545	6.71776502	6.773240303
10.07580535	9.613206141	8.600831152	8.603745778	9.784374313	9.251353071
11.15183093	11.70812672	11.87445957	11.64848719	11.41238356	11.86922315
10.4302696	8.726978374	8.705608919	9.370832535	10.1421691	10.02161965
10.06633871	10.42622906	10.4524716	10.13375911	10.19649023	10.62142174
11.52824	11.32559397	11.13240418	10.92337564	11.34202326	11.31852116
13.22156044	14.66395575	14.34357305	13.98081649	13.76702458	14.29173122
8.721523222	9.025193869	8.683580283	8.251125323	8.377562293	8.571676263
10.61261659	10.97054498	10.88166557	10.78551178	11.09089144	10.74819911
9.223302313	11.47682743	8.898709273	8.985821889	11.48459908	10.11452638
11.58005536	10.67305197	10.94353341	11.72592113	11.04593523	11.21338774
12.38794592	13.73439002	13.26910509	12.62449502	12.99088515	13.34730616
12.70390423	12.87421343	12.69676846	11.96636849	12.42616911	12.32024961
12.87819387	12.28985985	12.31419772	11.87445957	12.17513939	12.32364634
15.449005	16.33688632	16.22759729	16.36806566	16.22759729	15.95256028
11.46145043	11.46936418	10.70052069	10.46108665	11.30506756	10.88302044
7.408681949	7.420173404	7.222163485	7.44169298	7.437044505	7.44112097
7.614497747	7.488608758	8.291166909	8.223899788	8.347717242	8.143776848
12.51424883	12.15130062	12.40343679	12.19996238	12.88265743	12.47130519
11.41778198	11.07797425	11.1502402	10.99763472	11.23641801	11.32185019
8.809951364	7.802836333	7.830100949	8.336257222	8.332313525	8.225261616
8.125376242	7.072297535	7.278422556	7.407119727	7.394983566	7.059062808

6.947262939	7.326942212	7.012842909	7.918105152	8.024267525	8.065153556
8.23139899	7.276277889	7.787863889	7.558798354	7.671246505	8.039451586
6.583924121	8.034046273	8.276867707	7.640655485	7.838173152	8.118473111
7.424024737	8.411285283	9.099242404	9.298556212	8.660166475	8.008022949
8.886096818	8.643157737	9.560346242	9.132080535	7.505759091	8.475210818
12.85589012	12.04432	11.96059797	12.46466592	12.87421343	11.95514832
7.464044657	7.915342566	8.132293172	7.983184263	8.178694051	8.53715797
7.332344778	7.803554182	7.491357222	7.364257101	7.215770273	7.610574101
10.13375911	9.509226798	9.919491525	9.881176566	10.17789322	10.88430477
16.73234105	15.449005	16.06827159	16.4534382	16.33688632	16.52993947
10.7442872	11.39594259	10.98627861	10.47479655	10.66832974	11.0859227
8.546545061	9.945994374	9.605576293	9.483552818	9.341222778	9.600185283
6.109379758	5.892070051	6.067372778	6.621578222	6.438718404	6.660321343
9.702015293	8.937429899	9.187808616	9.435122859	9.583475303	9.414554808
6.925728848	7.524103909	7.398696909	7.160551111	7.158855657	7.345977091
10.01622438	10.18666124	9.593075545	9.039454747	9.775265525	9.308265131
15.48449671	12.28369472	13.95955505	14.63205333	14.62217632	14.9591327
14.3093669	15.63778652	15.52328152	15.36066179	15.12351424	14.88238784
14.00297944	13.94443	13.64789782	13.34730616	13.7211242	13.39659876
10.33476777	9.59387599	10.20532801	10.39414857	10.16355358	10.23351878
8.483763566	7.510343899	7.799524616	7.796432081	8.010523545	8.145194646
6.700124848	6.484888434	6.429667919	6.445432434	6.531724505	6.428350232
7.785214909	7.257203303	7.540776061	7.815915242	7.418256545	7.576037556
14.33555643	12.01673563	12.73352188	13.34204398	13.44849295	13.02006671
7.755132808	8.097222414	7.643273222	8.25570701	8.397394545	8.052783737
11.89628941	12.22341713	12.06098883	11.59212007	11.96059797	11.84291489
7.609879768	6.904353566	6.622687202	6.921891778	6.890996051	6.705415545
6.990260788	8.547449101	8.438712657	8.276867707	8.559975162	8.297224525
8.929384727	9.048620525	9.067241768	8.788663253	8.894491141	8.951513343
10.70643744	10.69354222	10.86173253	10.92880859	11.04316622	11.12935449
15.22815255	16.82803465	16.57174008	15.97330865	15.92931651	16.25437967
10.00695165	10.69682519	10.4302696	10.6426412	10.83363242	10.55602586
7.415948364	7.496977949	6.916453859	6.797074616	6.934051768	7.517436172
7.205804788	8.893419636	7.872008071	7.851640596	7.656544636	7.705120374
10.69574272	12.29548604	12.24097391	11.67593314	10.24612484	11.93178106
11.29076429	10.69222123	11.9091429	11.36793803	11.33292491	12.01427079
8.416773909	9.938475404	9.478383576	9.951175646	9.374121768	9.578067192
12.32024961	12.64265677	12.44264271	12.06692054	12.63500423	12.49965499
10.03611568	10.01622438	9.822898081	10.69574272	10.6362571	10.88166557
7.383711768	6.457381818	6.494595232	6.443657091	6.345639859	6.892837172
10.35809493	9.499917182	9.818059192	9.920430606	10.02362526	10.28899471
13.35359128	13.64083669	13.67554111	14.010587	13.99495772	14.15863941
7.45028304	8.93638097	7.83046296	7.893631939	7.638629778	7.689906808
7.741148566	6.840253616	7.176878273	6.808256808	7.161342051	6.575656404
8.489580414	8.679110091	9.337187111	9.788274313	9.316888828	9.293068828
9.024212859	8.959665061	8.645985424	8.477505576	8.546982556	8.344441616
9.86640601	10.18563382	9.855298394	10.39747702	10.48659409	10.03701736
8.032499636	7.445746576	7.214922364	7.667309091	7.885731566	8.021628354
7.604463141	6.420399182	6.652192667	7.108809091	6.938620788	7.579350515
7.21466303	8.10326497	7.043720485	6.920918808	7.029601354	7.004539727
6.290108071	6.396397444	6.260257263	5.840394071	5.921747646	6.093161949
7.18283101	7.019424707	7.109400202	8.217270354	7.800624061	6.554351
6.566291384	6.385648535	6.712342556	8.07053003	6.990487535	7.953486636
13.91489381	14.86987024	14.57091557	14.49002723	14.46162431	14.38889659
9.911926424	7.611975737	7.591410606	7.970233808	8.457489293	8.05903404
6.894391313	6.664178717	6.958715091	7.259306869	7.480029465	6.775232444
8.226840717	7.905781333	7.426803586	7.034331232	7.572856394	7.21931098
12.30789527	11.7894918	11.84529477	11.5386618	11.74439188	11.49636188

7.328995606	7.717447071	7.928125404	7.989587677	7.799854778	7.995268899
14.76275492	14.45281427	14.1904887	13.72760196	14.49002723	13.92894576
12.94821125	11.92665931	11.87912023	11.84027237	11.97447062	12.15752365
8.82789604	8.783584889	8.668062838	8.895503556	8.953653768	9.194378495
12.61090773	12.02240324	12.02240324	12.29275278	12.23505088	12.17216641
9.904237354	8.735755485	9.312041414	9.193130899	9.000069889	9.386484303
8.57597697	9.392652323	9.285000091	9.164000111	8.800397414	9.198436465
12.96241124	13.56886427	13.0147344	13.0147344	13.11515098	12.91656586
9.31327601	8.794361798	11.47322198	9.005433152	8.546056747	11.41071085
9.921211657	8.182386152	9.244930505	8.960773354	8.292068253	8.583048444
11.74205097	12.12939166	11.89628941	11.6137143	11.82112126	12.41931404
6.17734602	7.024771818	6.598705667	6.622230818	6.50518599	6.479009949
6.777950687	7.453956636	6.437651364	6.226606111	6.469862081	6.609470354
6.974671657	7.778362485	8.143776848	8.341518747	8.335861768	8.341518747
9.62302801	10.08145981	10.00094823	9.694925798	9.585510535	9.689129545
9.193817232	10.20964764	10.89755671	10.74558579	10.29542336	8.836542848
12.20640951	12.28105924	12.43600068	12.78185519	12.43279956	12.26475658
13.66784462	13.25686564	13.27940818	13.08418191	13.34204398	13.08418191
14.36070414	14.4335752	14.33555643	15.03840752	14.9727616	14.48025691
12.48124823	12.23505088	11.97944932	11.58621592	11.79615897	11.87687449
6.681851	7.063754707	7.681167667	7.60736902	6.894391313	7.387388131
7.391655768	6.739790818	6.730193313	7.113720848	7.234805667	7.520648323
7.772503354	8.408718253	8.448456354	8.767368071	8.911109919	8.687053253
6.710946737	6.318902889	6.464934909	7.95873603	7.557268586	8.53579602
14.10328164	14.00297944	13.86604428	13.56886427	13.77352346	13.66784462
13.22730043	14.08841453	13.77352346	13.09463792	13.48990673	13.55640443
5.808918475	5.790114182	5.77279096	5.768307667	5.78750101	5.874415586
11.98196729	12.15440913	11.76336308	11.30690717	11.60069924	11.77802923
9.400967465	7.79851096	8.013487535	8.85785699	8.711304737	9.065009323
7.903688606	7.375790182	7.711523556	8.587623212	8.142197384	8.281588828
12.26775966	11.65052704	11.66934136	11.2608846	11.83813795	11.77305263
12.77454263	12.66167346	12.41634374	12.10951308	12.50328361	12.33907422
9.04056096	8.093473121	8.510894717	8.903025495	8.625486818	9.07581704
6.519665929	7.133560273	6.882764848	7.055909434	7.250545919	7.434204455
11.542568	12.22629088	11.93975968	11.65052704	11.6137143	11.97447062
9.011546121	10.56548536	10.3779146	9.955574404	8.791657869	10.2752886
9.084401768	9.739033131	9.611261414	9.557589465	10.042425	9.907546465
13.15082863	13.33155169	13.13522491	12.75396286	13.15645152	13.25686564
9.392082222	9.180906778	9.361074667	9.118059475	9.402814081	9.749660394
8.772410586	7.759936788	7.627373273	8.622197111	9.041236566	8.960773354
12.92081703	12.95303817	12.78645268	12.49965499	12.83827393	12.86955734
10.14326599	7.491357222	7.797507939	8.923576949	8.798247798	9.018802576
6.319492374	6.191297606	6.15200799	6.190947343	6.419503354	6.376277596
6.957403667	7.247290424	7.561964414	7.116869414	7.021742939	7.213178616
9.034403333	8.906407303	9.470840162	9.780583495	9.344897333	8.862225737
6.382025798	6.250090798	5.987975404	6.408811828	6.629471747	6.367075323
7.346280818	6.758967323	7.178905394	7.01565997	6.952257202	7.164403717
13.15645152	14.68633385	14.01682491	13.46712151	14.06476988	14.25895571
13.05428976	8.369612525	8.733877859	10.54565144	10.32849396	10.56802411
14.61202705	10.7814083	12.03310273	12.94367069	12.8293171	13.49745762
7.716801838	7.556302505	7.639261768	8.475612051	7.978709162	6.980238687
7.983548525	7.55178802	7.754407434	7.70365599	7.561342071	8.078774939
6.622911394	6.552305929	6.751169162	6.975188859	7.103272273	6.368128081
7.269064929	7.599211495	7.51810503	6.969919475	7.310063136	7.723687384
6.307709909	6.223694404	5.923322192	6.100129919	6.073202434	6.405870152
12.5521346	11.30131952	11.23116994	11.28896545	11.75662781	11.9875501
10.91176188	7.956286283	9.554043101	9.842388081	9.395854121	10.98037262
12.48458958	12.16942168	11.73972102	11.69188536	12.31089415	12.23505088

12.24351077	12.25572441	11.96947069	11.66189432	11.70348036	12.24351077
7.427372788	7.993428586	7.718747404	6.958444212	6.89207597	7.715405939
6.276206182	6.409677646	6.987033364	6.610658212	6.916453859	6.620481919
10.06071544	10.11760527	10.10946026	10.21375666	10.4302696	10.1905069
6.537516434	7.014205707	6.193752182	5.793358091	6.307499242	6.043315566
14.14208591	14.49002723	14.40789433	13.70143341	14.25895571	14.01682491
14.58095582	15.13975511	14.93426708	14.27397716	14.78379092	14.6970922
14.6970922	15.52328152	15.27762024	15.449005	15.34617167	15.39581235
12.31719428	11.81439214	11.84027237	11.30301738	11.92665931	11.59870577
13.64083669	14.31917195	13.98081649	13.63435472	14.3093669	14.08841453
6.397416404	7.484854374	7.528986283	7.641985	7.795116566	7.599870798
8.163475424	8.729972586	9.220211768	9.051957646	8.731005677	9.219562515
14.24162707	14.05136004	13.85099976	13.43523914	13.81266894	13.64083669
8.931557869	8.846355515	9.340550081	8.949261586	9.066605263	9.19736601
13.12023156	13.83173935	13.17778072	12.96662998	12.91196246	13.26910509
8.581169111	8.066374202	7.796432081	7.405823253	8.007640657	7.997183333
9.414554808	7.513736343	7.993428586	8.445297313	8.478760515	8.508645727
7.723384495	7.922041747	8.521230828	8.45577798	7.925642	8.585836293
7.258693172	7.012571859	6.898002222	6.780426	7.141717727	6.760181828
10.90754603	12.28693608	12.58957491	12.29886409	12.20960187	12.20640951
11.2786408	11.90674449	11.47127063	11.01045685	11.4535969	11.01209098
7.265354444	6.252448444	6.221954697	6.520315747	6.64694096	6.859220677
9.420326576	9.613873859	9.190205293	9.108564253	9.301561	9.563605545
13.87958112	14.94534759	14.50017253	13.54965001	14.35197184	14.07245549
6.681366485	8.304980606	8.462236515	7.793372141	7.882525919	8.386133566
10.90606985	10.02923217	9.923012889	9.860587788	10.50166798	10.56084718
9.165786899	9.593075545	9.565032515	9.168324747	9.215289101	9.244399414
12.62807191	12.80166365	12.59668127	12.08943903	12.44264271	12.57107442
6.982135444	6.593634414	6.834834778	6.910146768	6.880775747	7.073141899
12.41634374	11.92937262	11.72126504	11.38025327	11.88148631	11.73972102
7.720197707	6.688467242	6.736375424	6.825565162	7.062438556	6.941216071
7.26819499	8.534897646	8.550414556	8.194999071	8.150892606	8.266842545
11.39983314	12.57877126	12.10951308	11.92396137	11.77802923	11.94234713
7.622357354	8.505797091	8.593101434	8.921011566	8.977005354	9.037153444
12.02811467	12.53563192	12.46055504	12.01427079	12.56726296	12.59668127
14.80749806	15.92931651	15.61926687	15.48449671	15.41210544	15.24813401
6.443240697	7.105750636	7.127365253	7.357456071	7.188612899	7.561964414
7.227000899	6.836530859	7.109111172	7.239990273	7.214922364	7.277482444
7.38862201	7.57318396	7.350681061	7.574795778	7.660499434	7.01811702
7.468494556	6.967234434	6.82246696	7.205488778	7.367866364	7.166369152
13.20018245	13.35919327	13.03957854	12.77015535	13.23864174	13.32021166
11.62739451	11.03737422	11.16281786	11.6445291	12.12436236	12.60022648
13.90036211	13.93694444	13.81266894	13.54221797	13.62784329	14.09521817
9.726443586	10.81034167	11.11081747	11.12613667	11.15503783	10.85100254
6.457134202	7.559118222	6.85895202	7.421037465	7.732919727	7.510027525
11.49239347	7.668279394	8.208296687	10.080472	9.966143818	9.87177298
6.343678303	6.235213687	6.904353566	6.75222999	6.960198707	6.874493707
15.86132141	17.07010739	16.73234105	16.33688632	16.49551516	16.61084374
6.868352737	6.909148313	6.754699697	6.601226343	6.857741051	7.009988212
10.88166557	10.93935201	11.04464523	11.11560115	11.21493335	12.08943903
9.060618192	6.733232384	8.141366242	7.42989803	7.12824003	8.712341879
9.811898121	8.793339475	8.836542848	9.155642525	9.548028869	9.147296667
11.96059797	11.00496707	10.72127155	11.51867768	11.87912023	11.15666056
6.949065	7.589252838	7.717096556	8.143419303	7.820222687	7.930615646
11.29597305	11.21016953	11.2278827	10.86820493	11.38391359	10.90466263
11.04593523	10.14999886	10.05880999	11.06261544	11.14385742	10.61992902
14.09521817	15.09418019	14.90726461	14.60195877	14.63205333	14.57091557
9.305189859	9.625623061	9.248396293	9.11211799	9.30770201	9.631182697

10.00450786	8.012346152	8.587623212	9.721958051	9.426842081	10.3305428
14.7528858	10.76857843	11.80791686	12.54589185	12.78645268	12.29275278
14.31917195	10.43757507	11.85301976	12.47130519	12.52819783	12.797444
10.1421691	9.634173323	9.525471414	9.151431899	9.550769788	9.054860525
10.18468509	9.141588162	9.073600626	9.611261414	9.363823202	9.034403333
8.954176293	10.28005063	9.445197818	9.769994879	10.11555853	9.780583495
7.825440475	8.102124455	7.722297051	7.435155859	8.033283465	7.630295798
6.265533657	6.095658889	6.015303091	6.077855707	6.34170502	6.439328828
6.67377398	6.019346071	6.389460141	5.866213242	5.922892646	5.752952475
6.855446939	6.389669667	6.647679424	6.632101727	6.791029141	6.362894646
7.302140758	7.061330929	7.360165061	7.791533949	7.518430525	7.583692414
6.474717424	7.648319737	7.459115707	6.635108111	7.990313949	6.447069212
7.751645273	8.196571586	8.12949599	7.678291495	8.162329636	7.686071172
7.027167101	6.372542859	6.488413788	6.753469455	6.953635141	6.558838808
10.61992902	10.23351878	9.993897848	10.32309273	10.35591393	10.16546821
9.003357768	9.11154599	9.387656788	8.725555596	8.76386996	9.214694788
9.653591273	9.208914778	9.234418071	9.653591273	9.623794152	9.318228697
14.99939481	10.95849458	12.94821125	13.76702458	13.83173935	14.35197184
7.475313929	7.310197869	7.116617232	7.030162636	7.137721343	7.466342606
7.113452899	7.70299303	7.189144101	7.01152302	7.103541778	7.356542485
6.853717071	6.911201424	7.355706667	7.762794545	7.410364737	8.108917222
6.670578394	6.897195879	6.906092707	7.176878273	6.967663404	6.651970061
6.602427343	7.398370889	7.016197848	7.790916071	7.691187768	7.286722061
7.956286283	7.482605354	7.632536929	7.474688838	7.545986354	7.41959598
8.590695768	7.517772121	8.447526838	8.234636465	8.143040162	8.627322343
7.928125404	7.110215707	7.664565879	7.346280818	7.036210545	7.58642403
6.524488081	6.921167192	6.490634808	7.28113	7.090758091	6.433428778
10.35591393	11.39772243	10.64623997	11.0859227	11.34892675	10.56655729
8.137860071	7.303836939	7.831503424	7.979863071	7.764931778	8.47107903
6.479009949	6.437269283	6.218902788	6.17767296	6.157664758	6.265911495
10.76857843	11.17213305	10.36349344	11.01209098	10.76471511	10.59685822
7.40806004	7.539809273	7.428935273	7.763186	7.981345788	7.525550222
9.061091758	8.77608297	8.748883111	8.257595869	8.832790172	8.493653778
9.499247869	10.3779146	10.2572153	9.963504081	9.790672212	10.32946621
12.23191382	11.97447062	11.71244686	11.47127063	11.46145043	11.67849095
10.4322159	10.78014639	10.78551178	10.31095185	10.42297323	10.48782122
13.40302459	13.5360606	13.3718573	13.39659876	13.28559105	12.9851923
7.636533838	7.153761556	7.44169298	7.467830485	7.31189602	8.10683704
13.64789782	14.38889659	13.78027417	14.29173122	14.15092087	13.97489728
8.597538556	8.097566687	8.895503556	9.433258586	8.968391182	9.528804131
12.60396528	13.57574016	12.76207554	13.40990013	12.81379045	12.47441306
15.16803204	15.99508933	15.58150095	15.70163214	15.72204116	15.13975511
7.325732596	7.762794545	7.617533182	7.856753939	7.973227556	7.474397404
6.955200051	6.823157384	6.817048798	7.085629475	6.977817737	7.358688323
7.111135707	7.230593889	7.470009697	7.371279929	7.038073394	6.470823475
8.04259702	7.776293606	8.085771838	7.844877657	7.89560499	7.836828747
8.307093606	8.450171172	8.239069586	9.053706202	8.768863636	8.597538556
9.398445515	8.329784131	9.967178364	7.522209596	6.956636515	7.659186848
7.360165061	6.697417434	6.809240364	6.950382242	7.185402798	6.852150101
8.689422596	8.568768141	8.311348051	8.267698071	8.169730152	8.352537101
7.999327475	8.030181424	7.69973097	7.866656354	7.743466333	7.681826051
7.546292778	7.676956101	7.264510162	7.40806004	7.428605818	7.209485919
8.487847515	8.528915121	8.148682091	8.33796001	8.898709273	8.114611232
10.38738452	10.96311364	11.03580591	10.88546605	10.85513517	11.24001011
11.45932673	11.64244603	11.28370047	11.95261936	11.72807534	11.28731295
9.192533242	9.376046232	9.050265121	8.749346626	9.150235333	8.998420707
11.74935785	10.91356479	11.90674449	12.09804166	11.94234713	11.911688
11.50632759	12.82153585	12.53563192	12.85048632	12.71142619	12.33273925

10.11452638	8.814101707	9.308918081	9.807061475	9.50650501	10.07580535
9.020337111	6.945626949	7.567404636	7.688949081	7.689906808	8.044608626
7.789255535	8.782655051	8.242431778	8.40702104	8.425403333	8.232591606
10.13671639	11.17041501	10.25906505	10.49348589	10.62647628	10.42409385
11.565007	11.79870316	11.78265022	11.78265022	11.9091429	11.41238356
12.36759935	12.8293171	12.35798465	12.26475658	12.06098883	11.9577766
7.854325061	7.262427071	8.131862263	8.599954677	7.99823603	8.148682091
7.776955808	7.195783737	7.341641707	7.095808323	7.148774485	7.097914788
7.731201121	8.733083212	8.562227051	8.325717687	8.226165	8.581684657
8.625920364	8.945493505	8.784649081	9.301561	9.009915859	8.821324343
6.684320869	6.573293212	6.28064496	6.339459364	6.708308495	8.305359949
8.567801737	8.907915899	7.94734596	8.000144121	8.22479296	8.097222414
10.10278369	9.415217505	9.731150899	9.849671929	10.02061657	9.378583414
8.379884889	7.792587404	8.796713535	8.751946515	8.700142556	8.960218374
10.09597734	9.323195364	9.565660949	9.872596081	9.911926424	10.05144087
9.731150899	10.66945215	10.46342714	10.53737043	9.928455091	10.62895885
7.040256253	7.701991131	7.696259101	7.833986465	7.528303323	7.449265303
6.825072616	6.465574545	6.975426091	7.195783737	6.765793475	7.396159798
6.10907198	6.684542485	7.304711495	7.411561343	7.31258798	6.740749081
8.844732879	9.015412101	9.102676354	8.423588242	8.744562374	8.900779434
7.950135394	7.158590636	7.601973414	7.430781919	7.06536003	7.635267465
7.294038515	7.395300939	7.105750636	7.118512586	7.052221778	7.608934747
10.64502212	11.11560115	10.80091358	10.16073454	10.71031814	10.93023415
8.901935111	9.480384485	9.379180747	9.29794999	9.180906778	9.470840162
6.906335848	6.935043828	7.421928768	7.151031394	7.278747747	7.370661465
8.353342091	7.246141232	7.209785606	7.493565131	7.692888131	7.295094879
11.31449746	11.35657003	11.48840455	11.37847359	11.51465712	11.25523353
8.124973859	7.394370293	7.38400901	7.409710545	7.692552364	7.300950758
7.271416848	8.086566798	7.330192545	7.728361283	7.61856696	7.344596535
6.390967051	6.597742293	6.929631566	6.881752616	6.634122485	6.495011879
8.970028374	8.220225172	9.004940778	9.303342798	8.906407303	8.71485401
8.55725397	9.298556212	9.124449354	8.970028374	8.634342798	8.929972798
9.968215758	9.373397364	9.757590192	9.930141081	9.984347768	9.903372323
13.65450155	12.68903729	13.79381649	13.7211242	13.49745762	13.74093847
10.67939021	10.98749259	10.97631922	11.02700786	10.69793218	10.74558579
8.037984616	7.794719919	7.098240939	7.094450071	7.177746566	7.15072903
10.11849919	9.875361242	9.747577162	10.13862753	10.06356315	9.70798304
11.36793803	11.96636849	11.80342824	11.87912023	11.8164662	11.75662781
10.14809211	11.07493153	10.46766096	10.64128512	10.43122177	10.59809845
7.055055273	6.589168273	6.909921869	7.136875657	7.020179424	7.284105152
9.046362323	7.851640596	8.170129495	7.920494253	8.226840717	7.743466333
8.462637525	8.106019798	8.205892515	8.222643394	8.170954818	8.010523545
8.771856424	8.48093102	8.981479273	8.843670869	8.736738475	9.038261515
12.35168631	11.39185194	11.63629658	11.90152869	11.63401423	11.14211434
9.36570098	9.851320949	9.708693071	10.01168912	9.724814657	10.16937171
9.009407333	8.985325394	9.412634061	9.492465394	9.222631263	9.460382818
6.048882657	6.298407384	6.56193898	6.551390626	6.365723081	6.542314697
10.63014735	10.55966675	10.3025572	10.8935809	10.75865445	10.38430498
7.501443394	7.440186081	7.767362636	8.074096707	7.909031556	7.863195747
6.282381596	7.068665737	6.979123747	7.102982434	6.96124304	6.96545202
8.295839707	7.927113394	7.786298434	8.131862263	8.053413657	7.802836333
9.727055768	8.648914657	9.825199323	9.898158111	9.771430818	10.36903954
8.494130061	9.273640758	9.032817303	9.103811545	8.885078273	9.042346232
7.923423949	7.377557566	8.544134919	8.104500051	8.278157657	9.273640758
10.1985269	9.045168101	9.288999586	9.586238192	9.579413505	9.535633242
13.32669092	13.92172837	13.2504616	13.74093847	13.2504616	13.57574016
7.462172081	8.127895253	8.031407364	8.206288727	7.659186848	8.012698303
9.19736601	9.448606737	8.342773172	10.03205964	10.36349344	9.622300838

8.595283071	8.233006697	8.424058939	8.708030737	8.541524636	8.625920364
9.766921414	9.368875727	9.872596081	9.484209455	10.0406788	10.27236099
7.713270545	6.829819869	6.830856061	7.483852838	7.42989803	7.541090081
9.651465606	9.149195859	9.190767364	9.590351455	9.186711646	9.070117818
8.675348646	7.535866141	7.724673141	7.909412687	7.949400121	7.596851556
9.687048303	9.161459798	9.384078242	9.490338525	9.462343535	9.07526898
7.970233808	8.453639616	8.40702104	8.737817051	8.640789455	8.730448515
10.31480319	10.10841911	10.3305428	10.75987116	10.6488688	10.21279484
10.38001468	10.36349344	9.687758929	8.979921919	9.324388263	10.84176532
9.851320949	9.818860343	9.998242414	9.533596364	9.881176566	9.856174394
10.45628541	12.32964397	11.76804793	11.69891546	11.69891546	11.542568
10.49592664	10.43998577	10.6362571	10.09339803	10.06173934	10.19236777
9.545191596	9.265583747	9.618256808	9.554043101	9.659537192	9.428845576
8.40702104	8.607126586	8.451947071	7.85268101	8.479185606	8.198599495
8.709941838	7.766738848	8.446184828	8.380307172	8.452374444	8.825911939
7.008962394	7.58864003	7.228468091	6.58977503	7.096055535	7.424346313
6.963370131	7.672313364	7.705454838	8.037287293	7.611975737	7.440763838
8.207099091	8.607626293	8.756396081	9.180906778	8.692466525	8.551311374
6.771840333	5.949920465	6.322925	5.921340081	5.907643202	6.27602701
8.62460002	7.302435273	7.980934222	7.527697455	7.369510384	8.796713535
7.322217273	6.816006121	6.862895808	7.08136002	6.98921798	6.735859162
8.490053091	7.757234778	7.573484121	7.557888444	7.795765162	7.54731098
7.710917071	7.55847901	7.320933798	7.657256192	7.74961303	7.816627253
5.819048768	7.438935576	5.757057035	5.79820198	5.822840646	5.75938702
9.598265626	7.97284099	8.690381253	8.875206121	8.668931657	9.29496
10.47584162	9.987077505	11.29399858	9.912769303	9.424985495	11.47682743
7.932066141	7.756175162	7.803193929	7.952774121	7.956615091	7.81270001
8.598980253	8.509065354	8.429130081	8.31214998	8.330618758	8.435296202
9.969958172	10.81280665	10.16262583	10.28899471	10.36903954	9.979092404
6.773917293	7.432070859	7.231529313	7.053922394	7.584276586	7.017237545
7.811684818	8.653441707	8.248566778	8.428176394	8.206690121	8.374810828
8.571676263	8.382609505	8.568242293	8.50618604	8.633351606	8.218887434
9.037616626	9.315671101	9.068490586	8.671783687	8.869521111	8.876650909
7.353244222	7.33350399	7.205228253	7.145208253	7.151031394	7.001104828
6.139284859	6.073069071	6.575452697	6.56193898	6.232346939	6.788076343
7.195224778	7.039183869	7.156444778	6.708797384	7.149343495	6.872867768
8.805485081	10.05688349	9.330464051	9.59387599	9.164000111	8.995099212
12.27101	10.80091358	11.39983314	11.75462574	11.6445291	11.87912023
6.409265687	6.128153374	6.110155364	6.158655535	6.052879131	7.193299919
12.13867659	12.67700968	12.37416009	12.93430142	11.68948846	12.22629088
7.445746576	7.310482798	7.375790182	7.537240869	7.646953505	7.220240556
8.076118121	7.870669919	8.091323071	8.427236737	8.095330333	8.439112919
6.864987879	6.825870455	6.996297444	7.150169929	6.84678303	7.071486061
9.066117364	9.368224626	8.690918616	8.896578162	9.050265121	8.750392838
8.057063626	7.673376626	7.247290424	7.74578199	7.842359758	7.987188889
8.712915394	9.416442616	9.127872697	9.514496606	9.198436465	9.033394545
15.08134613	12.00644476	12.32364634	12.33907422	11.97196754	13.09045797
9.143279535	9.689129545	9.535633242	9.047491869	9.214111545	9.254253192
7.866656354	7.073400869	7.006478434	7.58642403	7.850535525	6.996042242
6.896472707	7.154318051	7.275508121	7.560760293	7.359577545	7.017519192
6.791799101	7.490096869	7.378186273	7.200432616	6.804242869	6.782643404
8.297630758	7.969793182	8.159434576	8.236986626	8.385303111	7.939609869
7.98690202	7.705814323	7.188870657	7.580904889	7.650231051	7.325732596
6.908859909	6.79370899	6.818245747	7.119927869	6.491055869	6.238895919
6.274687444	10.21769501	9.333361616	9.738398616	6.729687141	9.280305798
7.136586475	8.043387838	8.009440313	8.145962667	7.967524909	7.768772758
7.799854778	7.723687384	7.917403434	8.509065354	8.679110091	7.539809273
8.005371424	7.908308323	7.291982576	7.411868313	7.314300525	7.737987747

9.153232364	6.329109535	6.504049202	11.20498985	10.43564423	11.26815786
7.438305869	9.279768394	9.740584727	9.123297364	8.799348343	8.735287485
8.105629576	6.865318939	7.659186848	7.153495505	7.137147091	8.485631939
6.475742909	6.432225313	6.392337434	7.254644071	6.978866758	7.258107788
11.24816417	10.70052069	11.17041501	11.43938222	10.99345288	12.38794592
15.52328152	10.76361324	13.01016522	13.47832004	13.05987966	14.7528858
7.212383152	6.934796758	6.771393131	7.02934998	6.903822293	6.899006192
7.282635596	7.993077394	7.744777657	7.837507091	7.47214603	7.993875677
10.75865445	12.43279956	10.9889639	10.82153564	12.14789109	11.6137143
5.928490485	7.148452788	6.928307859	6.702513515	6.76944904	6.539984525
9.597513697	9.913634949	9.609756293	9.961780586	9.45706402	8.895503556
12.42943771	12.38063147	12.38794592	12.89513437	12.26162026	12.75784116
7.255805293	8.248566778	7.660150727	7.674109172	7.792215626	7.643273222
10.76983035	10.1421691	10.57159251	9.921211657	10.44392641	10.30695028
9.39974104	9.397864101	9.021393263	8.540202646	9.206631485	9.11393401
7.049507061	7.51247098	7.171336586	7.279669313	6.965960869	6.871158202
11.32559397	10.24511785	10.40925212	10.68338253	10.89604789	10.15379546
8.687053253	7.725042051	9.062220818	8.818210192	8.549517636	9.37540303
8.200163707	7.788172758	8.019434586	7.749299101	7.922697606	8.633351606
8.181196919	8.263464364	8.450646323	8.317411434	8.280707687	8.286610323
13.62106704	12.73731843	12.71520416	12.87421343	13.22730043	12.83405786
8.084674061	7.166060222	7.459405444	7.859670455	7.649926505	7.882189879
9.119222505	9.128533354	8.587234515	8.412956141	8.769824364	8.47014499
8.784105778	8.288815495	8.463063475	8.749910828	8.793339475	8.209054152
8.104133364	7.36203996	7.364797848	7.11316801	7.280239242	7.261824374
8.52575703	8.703733141	8.657806242	9.048100778	8.873520545	8.782655051
12.4571909	12.11216258	13.33661988	12.78645268	12.91656586	13.32669092
9.157435455	9.378583414	9.050797061	9.749660394	8.727989172	8.854603364
8.332313525	8.419866586	8.671783687	8.765765323	8.665165081	8.334981101
9.523397737	9.745446081	10.04682541	9.58708204	9.732604394	9.811091061
8.962353222	7.985458788	8.120795899	8.261781515	8.265565879	7.901917293
8.211411717	9.062220818	9.281446566	9.421700798	9.261851101	9.669847545
7.84419497	7.807594768	8.665165081	7.303019677	7.170797768	8.445297313
11.14211434	10.84844593	11.48061491	11.16423752	11.71443529	11.04156144
8.643157737	9.658772677	9.713858919	9.76546198	9.891739697	9.108564253
6.417239485	7.294583061	6.27919301	6.094493333	6.051028485	5.838675343
14.27397716	8.804952828	9.648698737	10.7982674	10.88681216	10.73014715
7.593038414	6.986482212	6.836284939	7.490096869	7.176396859	7.154007354
13.42888134	13.05987966	12.86955734	13.41514643	13.37822002	12.66167346
8.561321323	6.933000202	7.134964384	7.544367232	7.341641707	8.070136677
13.48990673	15.32551587	14.48025691	14.89443037	14.34357305	14.33555643
7.33182303	7.269598859	7.53302901	7.799196616	7.698638758	7.470612081
7.850535525	7.487078798	7.346280818	7.228759818	7.317590788	7.252299788
14.34357305	14.77222727	14.28325373	14.9591327	14.65397593	14.34357305
7.048729333	7.658441616	7.589888475	7.414724596	7.616235111	7.584620515
8.389653889	7.664957788	8.068931465	7.996770556	8.482406475	7.690204818
10.55213977	9.917043434	9.848227263	9.967178364	10.00361533	9.831834717
11.88649013	6.583167424	6.974671657	7.855731	8.067132556	7.543396525
9.533596364	9.220211768	9.17861001	8.990220404	9.151431899	8.740682
8.574674323	8.159434576	8.637513768	8.784105778	8.562227051	9.049171303
6.501249222	7.644277273	7.728361283	7.424346313	7.12986801	7.347734202
7.570765313	8.06745399	7.718083212	7.808339242	7.552833101	7.438305869
7.787223354	6.918043889	6.72626003	6.851123798	7.053635222	6.839464636
9.158023	10.09339803	10.03026493	9.817379515	9.875361242	10.01257663
7.614146707	9.152615889	9.16271003	8.687541707	9.072998758	8.986432162
6.638813101	8.211411717	7.969427899	8.279462	7.405524869	7.103815788
7.975784071	8.743617505	9.35796903	6.927779576	6.964897566	7.856391758
14.67552064	15.18392271	15.36066179	15.29549959	15.15443825	15.67809237

8.461315273	8.227270172	8.377562293	8.371229848	8.410908071	8.162707626
7.713612677	8.472435657	7.825767899	7.953486636	7.865908465	8.079553929
11.34202326	11.74205097	11.66708917	11.73070023	11.53050203	11.43773109
10.55474917	11.78023213	11.58814301	11.45176908	11.28731295	11.35278885
10.0143126	10.95683616	10.68572341	10.41530743	10.31289083	10.39641485
6.610458242	6.838435556	6.812925505	6.984849141	6.899979889	6.002122737
9.898158111	8.686679798	9.35188598	9.37540303	9.184000242	9.807452253
7.026391172	6.858687869	6.804033586	7.052522778	7.252886707	7.076589626
9.11211799	8.674900657	8.62460002	8.496365737	8.821324343	8.446641939
9.886762101	10.72516317	10.34626898	10.31013098	9.955574404	9.945028121
10.06255975	10.12126634	9.853774707	9.708693071	9.698896848	9.630502939
8.710842768	8.948168879	8.508189222	8.147857556	8.359650919	8.328154263
10.80782516	10.23948175	10.89094316	10.97476091	10.86173253	11.26446827
7.597232061	8.294235768	8.023099172	7.64030596	7.729067364	7.814093232
6.145849162	6.267641192	6.55251301	6.486835303	6.593141616	6.371798101
9.193130899	9.165786899	8.837022465	8.554027364	8.671783687	8.804952828
10.97054498	11.14211434	11.03163187	10.62764398	10.84176532	10.92337564
10.61001127	11.04316622	10.79686873	10.4283734	10.59541023	10.72127155
9.810255869	9.903372323	10.04160563	9.93099102	9.933588354	9.966143818
7.283800475	7.610574101	7.443192556	7.186028475	7.31937996	7.31374401
11.56725755	12.01956714	11.70557621	11.48840455	11.45932673	11.5612151
9.574750414	9.760900556	9.916104475	9.641007131	9.660200838	9.722667242
9.945028121	10.59809845	10.32946621	10.33252085	10.18291229	10.44712342
10.22164118	10.20164867	10.44602022	9.831834717	9.847335909	10.15783547
7.574489596	7.900011667	7.10076196	6.882764848	7.260583141	7.222163485
7.518690889	9.757590192	9.36570098	10.47123164	9.263233333	8.53537201
6.822666394	6.791799101	7.430781919	7.345267404	7.183932212	6.906335848
7.431765758	7.538207606	7.267645253	7.313208869	7.298118263	7.649291899
7.187213162	6.414036263	8.460863192	7.523479626	7.122697263	7.111963455
7.658441616	7.990313949	7.623386535	7.340147596	7.208594677	7.339214566
9.029402949	9.786685869	9.549314313	9.337187111	9.528176495	9.271759586
9.473962505	10.60743282	10.16355358	10.60161194	9.52752797	10.58469265
7.772843323	7.60736902	7.539167263	7.831854586	7.439837192	7.610260323
8.119679141	7.91465098	8.874191697	8.313601758	8.083135202	8.631321253
9.005975162	9.671170444	9.465071586	9.573320202	9.604846424	9.533033172
7.988882495	6.784856394	7.149343495	7.039421667	7.335252788	7.141989162
6.889990505	6.826879909	7.462491444	7.571747222	7.182015707	7.200432616
10.57885616	8.891390919	9.016491202	9.374121768	9.106823152	9.275532697
10.59292728	11.565007	10.87092264	10.33890802	10.17613521	11.02700786
9.829822242	10.12314196	10.23160947	10.29432836	10.10187506	9.737663949
10.50057506	11.38025327	10.59924244	10.31597323	10.88809182	10.4687107
10.10454015	10.60383217	9.881912677	9.856174394	10.95093974	9.550085313
9.067241768	8.856343687	9.053043889	9.372025394	9.669114061	9.164000111
9.449254061	10.01340346	9.417594202	9.518503263	10.16262583	9.31393701
9.689129545	10.48185706	9.877792838	9.820433545	10.52364746	9.606298465
10.31013098	10.79564384	10.01974654	10.16937171	11.32376318	10.06822268
9.643287657	10.32208546	9.507772687	9.546645747	10.49140257	9.435122859
11.96947069	12.93862501	12.16942168	12.03068847	13.45466248	11.96947069
10.05880999	8.580722586	8.808345061	9.021393263	9.058356636	8.992473788
11.04886125	11.48459908	11.16423752	11.86922315	11.20345844	11.20498985
8.358783152	8.638952889	8.300888657	7.670275758	8.007258162	8.246913576
7.124596343	6.81164602	7.587635919	6.867884374	6.595504071	7.287262414
7.268778051	8.346198293	8.053971495	7.873080636	7.778011646	8.363019899
11.17910752	11.75662781	11.76588718	11.88148631	11.86922315	11.5737214
7.762794545	7.025021455	6.861163889	6.89207597	6.789286121	6.652192667
7.983913121	7.82717702	7.475313929	8.01423197	8.154705535	7.688949081
9.712341424	9.346872071	9.296718121	9.684886576	9.235747242	9.413942061
7.595597162	8.110881556	7.840676768	7.700026646	7.759936788	7.743818828

8.055171202	6.924473131	6.980785162	7.531770848	7.181699919	7.892604899
8.212685152	7.614831859	6.823157384	6.773706576	7.292561596	7.500795283
7.010236465	6.898538505	6.764835354	7.094132182	7.881102323	7.132177788
9.663106646	8.81572204	9.531623293	9.417037424	9.205404404	9.687758929
7.497307495	7.228169333	7.544047919	7.828584192	7.603719626	7.589252838
7.519695475	7.11316801	6.982135444	7.190368869	7.23119303	6.904630343
11.18291631	13.00101781	11.40145091	12.51066587	11.9577766	11.72126504
10.30358984	10.42297323	9.962571606	10.55474917	10.49592664	10.25220538
11.61117883	12.44611991	12.52130815	12.91656586	13.10484893	12.49170848
8.262181081	8.216823323	8.024633293	8.280295667	8.33024502	8.07563398
11.56291631	10.71992703	12.12939166	11.93770961	11.77305263	12.62807191
6.590932293	6.539984525	6.551008899	6.751435283	7.19681397	6.845568374
8.267698071	8.667049253	9.157435455	8.656360697	8.513135323	8.03179299
7.056197182	6.640921556	6.939947626	7.070381242	6.874731313	7.353900202
9.315088131	8.540635232	9.024707707	9.251353071	9.35128199	8.952561111
9.424985495	7.18283101	8.864430475	9.901640636	9.121511687	8.195405505
9.041236566	6.500408909	6.636272576	7.89464897	7.849463182	8.066374202
8.466446051	8.521230828	8.633848111	9.073600626	8.801904485	8.546056747
10.0053333	9.146732444	9.660861778	10.26116661	9.799317404	10.761049
9.800083455	8.228510596	8.385303111	8.859715222	8.761177727	8.867411152
10.82664672	10.3494333	10.39296715	10.73264046	10.60863639	10.25811169
9.50650501	8.852576737	9.150235333	9.32739897	9.211846273	10.01071956
7.522559465	7.396823121	6.625506687	7.432376434	7.461239687	6.60386102
8.170954818	9.582761798	9.69725304	9.87177298	9.515069697	9.520598525
8.344860929	8.537956091	8.761644081	8.69335002	9.019287687	8.038354192
8.482852263	7.493254434	7.870978364	8.030983081	8.576430182	7.920494253
11.96636849	12.36759935	12.62127559	12.71142619	12.50706636	11.93486375
10.05607537	9.963504081	9.84311402	10.0178996	10.24828231	9.660861778
8.775519707	8.264694495	8.625920364	8.041000495	8.364710758	8.420387707
9.530933293	7.565085768	7.853698798	8.509936919	8.358783152	8.461795505
6.919663687	7.885002121	7.204042263	7.557551899	7.251769333	7.423140192
10.10736711	7.983184263	9.653591273	10.20748734	9.551451798	10.93494087
7.024510747	6.833840465	6.577737222	9.107945202	7.924885333	7.368846081
9.156208313	8.125818909	8.321919424	8.425847798	8.412956141	8.246470545
8.125818909	8.928828384	8.430534091	8.140128606	8.50473503	8.454049747
9.326238727	9.672623929	9.461673394	9.733230051	9.526870273	9.784374313
13.27940818	13.37822002	13.18858184	12.75784116	13.07454735	12.92081703
8.765233303	7.981345788	7.977207111	8.607626293	8.798818646	9.448606737
9.135618929	10.00886723	9.895856061	9.66831603	9.32739897	9.667708556
10.29432836	10.15379546	9.954681949	10.34193651	10.02269473	9.857104919
9.493894545	9.051957646	8.911610283	8.808345061	9.024212859	8.810446485
11.38775416	11.36610866	11.2518391	11.62494396	11.01376888	11.23641801
8.284876758	8.670884566	8.904793061	9.078331919	8.918756414	8.640789455
7.858612495	8.426780919	7.98690202	8.446184828	8.754441596	7.863532101
12.84627851	14.21449649	13.64083669	14.06476988	14.13521971	13.47327752
7.819446808	7.539487212	7.920144808	8.227704253	8.006915303	7.915342566
10.64379219	10.34311059	10.80900786	10.6820228	10.36032821	9.995698596
9.06333597	8.579336081	8.755916071	8.892401576	8.380828141	8.515864333
10.53141254	10.27905981	10.50794857	10.78705857	10.19528987	10.54190997
9.496413788	8.870602515	8.776574232	8.745901071	8.971160737	8.785177889
11.6445291	11.75215821	12.39973853	12.41033676	11.93486375	12.25844332
9.553430788	10.21279484	10.23660558	10.39072525	9.379180747	10.63870636
7.183404394	6.601482788	6.510064202	6.675450515	6.748092424	6.176869667
7.276013455	8.08972896	7.877487515	8.018723424	7.497307495	7.163863636
9.832711535	9.436357141	9.662314131	9.563605545	9.848227263	9.465071586
8.982611727	8.253254586	8.634342798	8.534432515	8.64507902	8.438325768
9.803356646	11.00902763	11.21668249	10.9088749	11.04156144	11.26262606
8.829061111	9.899010949	10.02821235	9.942152202	9.636929838	10.0728998

13.0147344	13.52334642	13.55640443	13.39037726	13.1944969	13.35359128
8.732044667	10.06822268	10.22761479	10.18198959	10.32531651	10.76361324
6.318299141	6.366139859	6.477093667	6.103147919	7.145493505	6.700579303
8.097958939	8.412956141	8.329784131	8.216297535	8.262596626	8.447526838
13.00581854	12.91656586	12.60396528	13.23341018	13.14093405	12.44611991
14.60195877	15.41210544	15.08134613	15.22815255	14.91973608	14.66395575
10.43757507	10.39641485	10.27714677	11.09089144	10.73384189	10.4283734
7.314025869	7.101319909	7.778752535	7.66930299	7.610260323	7.944406939
9.693515768	11.23280633	11.20840189	10.71992703	10.65751844	10.86566339
10.72786832	11.3733012	11.4481816	11.46539851	11.62494396	11.47496104
8.092218303	7.955335374	7.907924051	8.174069929	7.873080636	7.758961677
6.288951848	6.440388828	6.813963929	6.039380545	6.079051081	6.771393131
10.1300726	9.36570098	9.207791152	9.723272263	8.606099333	9.310205828
9.207219232	10.02821235	9.808672222	9.837575172	9.785983535	10.04963797
9.178084667	9.342353505	9.21067496	8.941359636	8.729972586	9.195833419
7.042911333	7.114036293	7.049798838	6.838696091	6.831129859	7.054238101
7.617189485	7.160551111	7.534556657	7.26025397	7.316719848	7.017803152
7.345589182	6.865560778	7.593038414	7.534885556	7.471589222	8.188779434
10.40810668	9.772250768	10.6313464	10.41224321	10.41626133	10.37124354
11.07493153	10.58469265	11.09089144	10.97185531	10.91641235	10.83642757
12.46797904	11.76101506	11.88375452	12.00644476	11.98440811	11.78265022
8.443175586	8.102862354	8.112161727	7.702311758	8.261781515	7.849821828
9.74383503	10.1597097	10.32635032	10.23660558	10.37467131	9.679645121
8.457043091	8.316570081	8.367840636	8.620790505	8.510413414	8.240781051
13.31405593	10.8193093	12.01673563	12.38063147	12.32024961	12.94821125
7.152088687	7.417927455	7.555661677	7.140009717	7.262118616	7.21346404
10.06547375	9.388912091	9.265583747	9.604846424	9.509876273	9.360502949
10.60507136	10.34100163	10.52247938	10.2829717	10.39296715	10.82290783
10.64128512	11.2786408	11.22294817	10.90466263	11.10761793	11.11388317
11.94481318	12.60396528	12.47761486	12.23505088	12.04734694	12.06098883
7.084738172	7.500167182	7.398020586	7.226432323	7.207500556	7.304428303
10.36349344	10.45877561	9.984347768	10.06547375	10.24227579	9.93099102
10.74045328	9.925226333	10.14713571	10.14809211	10.10841911	10.33146686
10.66832974	11.97196754	12.03912361	11.64044206	11.55322016	11.68700199
8.826846768	9.453743545	9.441234879	8.956407626	9.013830768	9.080683394
11.53239215	10.83642757	11.05191968	10.9991534	11.565007	11.42562271
9.316319051	9.681893121	9.709426838	9.548028869	9.698181576	9.588958727
11.25898056	10.44934262	10.54565144	10.13193078	10.52247938	9.952967535
5.767025162	5.760828616	5.767906202	5.794104303	5.794704838	5.770335253
7.589252838	7.497675424	7.655304091	7.913871889	7.74753198	7.851640596
8.69919198	9.53690398	9.244399414	8.986932636	8.932594273	9.380344141
7.270153101	8.242431778	7.952029848	7.939228495	7.977207111	7.80435998
7.798826303	7.505759091	7.761378475	7.786927414	7.328126596	7.550541152
9.372025394	9.303342798	9.731806091	9.984347768	9.66831603	9.230770667
8.419470808	8.429582192	7.42612497	7.142895121	7.938531869	8.428176394
7.514821949	7.228759818	7.419275485	7.23452504	7.378842657	7.405823253
7.924172566	7.712908172	7.81061403	7.798177384	7.596851556	7.897743687
12.78185519	8.223899788	9.01707996	10.45150222	10.41426339	10.67815746
12.68484185	9.348735909	10.40387449	10.88681216	10.83223099	10.44832821
7.598885727	9.084401768	9.036569232	9.459003	9.021393263	8.570262
10.39968902	10.51291939	9.886762101	10.10736711	10.05426836	9.889177586
7.684965778	7.575132364	7.949071818	8.363862202	8.108523939	8.028563859
6.588475747	6.746121293	7.453623293	7.398370889	6.958200131	7.387965737
10.99623862	11.81018832	11.56725755	11.88909817	11.55928848	11.33848921
7.100167172	7.693516556	8.271405859	8.245649515	8.072532475	8.671783687
13.10484893	14.19920898	13.21058954	13.42888134	13.60269863	13.22730043
7.788532485	8.666113404	8.572701232	8.44129198	8.391460737	8.354182626
9.425521616	9.515069697	9.203189636	9.400967465	9.414554808	9.149195859

6.513004101	6.068020455	6.487487818	6.672823111	6.99955204	5.960394838
9.631182697	10.30358984	9.995698596	9.76471501	9.840616414	9.978126798
11.59427705	12.42616911	12.28369472	12.33273925	12.59668127	12.32659908
8.72650097	9.536330707	9.37540303	9.232683343	9.383480283	9.268661657
7.521885657	7.73355204	8.052077899	7.787525596	7.812003626	8.08075902
5.982320636	6.109691141	6.194253556	6.70156496	6.251035687	6.080051636
6.984268657	7.37546403	7.36694602	7.423140192	7.499556727	6.896238273
12.74093701	12.72219022	12.16279806	12.66167346	12.47130519	12.34577038
9.818860343	9.475206131	10.67815746	9.588958727	10.25220538	9.797856576
7.581254909	8.692466525	8.404396899	8.025051697	8.066736566	8.239069586
8.098747556	8.453185455	8.204737434	7.780823677	7.942071303	8.004655889
9.310775172	8.503920141	8.660608859	8.662363172	8.790654051	8.600356081
10.19332507	7.553068374	7.770819434	8.881310737	9.098746404	8.93587504
12.66627862	11.8358519	12.32659908	11.15966862	11.57178154	11.55106122
8.25570701	8.645541747	8.666113404	8.76626204	8.521696414	8.284073869
15.88528111	15.34617167	15.74256723	15.90458538	15.61926687	16.49551516
7.765298909	6.541370778	6.924473131	7.235967596	7.016433495	7.875808798
8.22479296	7.807154444	8.134366646	8.556855828	8.197004869	8.589388657
11.01376888	10.26632574	10.69931975	10.78856661	10.74558579	10.74172297
7.91307104	8.757866131	8.845785121	7.614146707	7.934739636	7.604463141
6.852390212	6.644450293	6.453428828	6.451227182	6.659312505	6.825870455
9.606954152	10.0188097	9.35858599	9.725631121	9.768390455	9.741357455
8.862225737	8.215875455	8.080354404	8.155393222	8.572701232	7.874063444
9.880317061	9.886023253	9.730279475	10.02741959	10.28005063	9.637663091
10.22070473	10.68572341	10.85100254	10.58347296	11.13713447	10.60277882
10.72384747	10.53247367	10.54692936	10.68687892	10.41224321	10.27804811
8.977005354	9.019287687	9.224923687	8.64752602	8.817191758	8.838495424
9.143918798	9.601568657	9.564343333	9.21905796	9.326739394	9.528176495
12.19086998	12.41634374	12.51424883	12.08680505	12.3613163	12.29548604
11.69188536	12.35798465	11.61814895	11.94979793	11.89361272	11.50425191
10.21279484	9.487680697	9.821195081	9.318228697	9.524156586	9.370832535
9.004398101	9.066605263	9.118059475	9.260036485	8.601896768	8.522156747
8.532980343	8.241600556	8.142197384	8.142197384	7.999327475	8.012346152
8.879330566	9.608350808	9.166446616	10.00361533	9.207219232	9.581418808
7.19832496	7.626793707	7.391952354	7.112898869	7.340448859	7.549206232
7.124332475	7.111681374	7.944058707	7.623386535	7.666980273	7.454874889
12.17513939	13.0147344	12.92081703	13.23864174	12.95787308	12.80949032
9.750460616	10.16642595	9.928455091	9.675185677	9.739033131	9.491039162
10.8405808	10.24414187	10.14631732	10.10454015	10.41957053	10.17440526
12.07810634	12.71520416	12.14507673	12.50328361	12.13538265	12.01191659
7.996393263	9.324388263	9.684886576	9.70798304	9.494490616	9.650129091
8.023866626	8.66101001	8.522156747	8.147147636	8.420871687	8.097566687
11.66934136	9.096395636	10.62764398	10.9102587	10.27804811	12.01673563
12.55983323	12.29275278	13.02006671	12.51766186	12.57107442	12.87819387
11.08264222	9.507772687	10.15585749	10.45996909	10.59062542	10.4302696
9.567720788	8.191204879	8.588971687	8.769824364	8.677707162	8.939630657
7.180020091	6.713757434	7.28582396	7.102414636	6.871904646	7.561342071
8.939630657	8.894491141	9.045168101	9.491039162	9.122138505	9.433258586
12.63888703	13.46712151	12.93430142	12.89113428	13.00101781	12.83827393
7.181699919	7.244122677	7.508388697	7.650231051	7.569572879	7.747112485
8.65497299	9.603529667	9.211846273	8.73262002	9.136296333	8.63794701
7.030688768	7.253764222	7.561342071	8.014589606	7.720197707	7.638981707
7.381324374	7.878518051	7.681488071	7.544047919	7.325732596	7.372842667
11.72126504	10.75062885	11.24993851	11.12315281	11.18941159	11.36065784
11.40145091	11.96947069	12.18142186	11.71895032	11.84291489	12.38063147
8.938439909	7.663284545	8.304980606	8.418118394	8.305359949	8.226840717
6.182576	5.749417192	6.817526586	6.537080394	5.948125434	5.74913202
6.308703535	6.598211152	7.225001677	7.298462212	6.858687869	6.470121364

7.842683778	8.99408802	8.830660505	8.865407737	8.466026899	8.762202232
8.91530399	9.391460545	8.594861495	9.385290182	9.687048303	9.698896848
9.328020364	8.047933768	8.352537101	8.764299566	8.602379626	8.867918475
8.294640202	6.966976535	7.595301182	7.547922717	6.711872081	7.733228788
8.232591606	8.886620687	8.445297313	8.633848111	8.503061444	8.261781515
9.40167604	8.617840212	8.989142232	8.939630657	8.972363838	8.715845505
12.35798465	11.81887725	11.91416468	12.35798465	12.27773509	10.9889639
10.60635433	9.449254061	10.573928	10.48897537	10.14999886	11.42371373
8.497259596	6.859701798	7.269324172	8.03364202	7.85268101	8.292509485
9.861430101	10.82416773	10.61261659	10.70534521	10.85361094	10.30810853
10.37346225	10.6768734	10.24316974	9.928455091	10.37124354	10.0079343
8.77608297	8.463578343	8.333749495	8.012346152	8.681067818	9.067856838
10.53505365	10.13193078	10.12224713	10.07956055	10.0841781	10.00002479
10.88302044	10.31013098	10.35178281	10.3779146	10.56802411	10.35699559
7.31374401	7.267388222	7.008416879	6.989960667	6.779669273	7.195783737
6.318093747	6.622442465	7.02750098	6.560645212	7.146637616	6.75400198
6.403079273	6.708053838	6.691558677	7.18482696	6.773706576	7.055909434
8.363862202	9.889177586	9.069558111	9.184000242	8.917069505	9.212980727
10.31193963	10.45375986	10.2692332	10.44602022	10.78269205	9.972781707
9.451851384	9.290152727	9.701165495	10.32635032	9.960976253	9.507772687
8.477856384	7.743818828	8.179581869	8.238629646	8.029836697	8.45110203
6.618269616	6.315590828	7.039421667	7.56574702	7.072297535	6.200564747
9.93663897	10.71503879	10.17440526	10.33989796	10.28190294	10.07670007
6.419103354	6.850608899	6.558602434	6.268241131	6.322079051	6.550562869
9.847335909	9.973623707	9.883591758	9.794631646	9.913634949	9.900778232
7.997498828	7.477539293	7.270153101	7.258107788	7.295703091	7.219017232
8.522156747	7.859670455	8.175288444	8.122747707	8.354947869	8.039790061
9.353057535	9.35371997	9.35128199	8.985325394	9.188424626	9.156208313
9.658049545	10.19236777	10.41123643	10.04776272	9.857104919	10.64379219
8.243245818	7.580594545	7.760294414	8.132293172	8.179581869	7.891143768
9.922126404	8.379884889	8.456191465	8.842109	8.826846768	8.799348343
6.519217414	6.514326051	6.306878242	6.204530263	6.324763646	6.337791596
7.759566061	7.338325414	7.90403798	8.127056495	7.83046296	8.052417212
7.430781919	8.221403505	7.793711495	7.612294131	7.505175253	7.788923101
7.184502222	7.137721343	6.791503909	6.945089283	6.713303939	7.073657889
8.463578343	7.355380455	7.629989283	7.767702525	7.799524616	7.344882899
10.31597323	10.70425349	10.26541026	10.84176532	10.43440935	10.48185706
9.487680697	7.710578727	8.921533879	9.356741384	8.762202232	10.45628541
9.973623707	9.988725727	10.18468509	9.859664869	9.965319232	9.617464455
8.788135667	8.280295667	8.531247657	8.092218303	8.288815495	8.116928465
9.53690398	9.237064687	9.851320949	9.582761798	9.593075545	9.337862091
8.187968455	7.714004283	7.968277051	7.654890798	8.243245818	7.75479003
8.823394192	8.452737606	8.43006898	7.983913121	8.43615796	7.983548525
11.42787377	12.0838996	11.72355631	11.56291631	11.41778198	11.70138789
8.191204879	8.498167798	8.345353354	8.166172404	8.301264212	8.465000141
7.151822525	7.366646525	8.324880909	8.158602879	7.836828747	8.750916455
8.168196313	7.603389051	8.290353162	7.572084505	7.20129803	8.967408879
6.32832399	6.310688818	6.386847838	6.487487818	6.419103354	6.364705596
7.955993172	6.876372364	7.276635293	7.998937818	7.623693616	8.256499515
14.86987024	13.98081649	15.06622261	14.82007331	14.47046648	15.83165575
7.572856394	8.294640202	7.950449798	8.322806515	8.184399818	7.956286283
6.629471747	6.79323798	6.394292394	6.42621898	6.470357717	6.459603455
7.272331859	7.833640626	6.974926293	7.568622485	7.457632404	7.242080394
10.08231743	10.02549055	8.866888202	9.884409465	9.599465606	9.476588061
7.702676091	8.562227051	7.880750535	7.986161141	7.598885727	8.185943182
10.67572435	11.19447199	10.64738653	11.16579624	10.9889639	11.1276132
8.897177899	9.520598525	9.201355202	9.600185283	9.644739414	9.009407333
6.708053838	6.581253303	6.605442354	6.923167202	6.764355354	6.663185626

7.75371804	8.699656879	8.696280707	8.426330949	8.742149222	8.380828141
10.61133106	10.49140257	10.27905981	10.13580598	10.36239309	10.37215733
11.10460602	11.27306561	11.26446827	10.56421743	11.11560115	11.07797425
11.98999321	11.99535117	12.61090773	11.80554878	12.15440913	12.30789527
7.606699253	6.782643404	7.186679828	7.452739495	7.173915838	7.554702939
6.176327717	6.623160525	6.737335172	6.939947626	6.838224475	6.878445485
7.409335747	7.07468796	7.036743576	7.075769859	7.358075697	6.857241172
15.90458538	13.30795266	14.17411554	14.67552064	14.68633385	14.94534759
10.19418334	10.92608881	10.63266161	10.80782516	10.61743608	10.89604789
9.466304111	9.680349586	9.782868192	9.795448596	9.634846707	9.960976253
7.358375414	7.201015091	8.412956141	6.727165222	6.207516667	6.195084242
8.836542848	8.026271202	8.274284293	8.537544354	8.345353354	8.747892899
8.08537204	9.026636737	8.039068889	8.757405333	9.035040848	8.282900293
12.61761735	9.939327313	10.62647628	11.82112126	11.51260306	12.24649354
6.244906081	6.186736495	6.145849162	6.275835929	6.659807838	6.19610797
6.762568667	6.780968242	7.223856	7.340746384	7.099574394	7.358963616
9.018299374	10.19957169	10.5397012	10.35069541	9.957374697	10.20532801
14.15092087	13.95955505	14.32804886	13.90036211	13.5160991	14.64328928
8.718769576	9.746868596	10.03299879	10.2861211	9.702015293	9.03221704
7.163563677	7.891531071	8.096128192	8.455403657	8.309668384	8.121211444
6.414426394	6.881514152	7.290204495	7.325732596	7.066488111	6.816280253
12.42616911	10.23660558	11.09721747	13.69473258	12.80602179	13.79381649
8.024267525	6.850100909	7.092334828	7.787863889	7.758192424	8.962353222
7.175214949	7.316095192	6.733232384	8.068931465	7.508388697	7.842359758
6.49370698	7.372238061	7.88183703	7.97284099	7.898595626	7.225864141
6.960463354	6.839681778	7.940608939	6.865560778	6.352052949	7.525015414
8.720627081	7.469634172	7.992662242	8.212243444	8.067838768	8.352134333
8.597027768	7.400314747	9.060017677	9.07581704	9.283145788	9.634173323
6.588688525	6.220864051	6.627087081	7.019697232	6.479663677	7.30556202
8.466852202	8.660608859	9.067856838	9.316319051	9.099925818	9.324388263
6.330710616	6.819416596	6.948873394	7.092334828	6.800553495	6.14867199
6.012730253	6.191799808	5.892740343	6.685804808	6.137509616	6.309659747
6.729176758	7.215458101	7.418614091	7.824443343	7.45998899	7.574140061
7.401155333	7.989587677	7.309054838	7.850535525	7.533663232	7.957321232
6.328925929	6.053560162	6.32497801	6.50824402	6.74585002	7.139668182
6.849574919	7.341966626	7.626793707	7.600615697	7.435155859	7.223856
6.821271707	6.16228202	6.496103798	6.442114232	6.014049576	7.257203303
7.743818828	6.912541192	7.213710414	7.74007802	7.327234414	8.336257222
6.505445727	6.69350096	7.252038162	7.178323677	6.83507799	6.858444758
7.973992111	6.273265505	7.234805667	7.183662697	6.902482606	8.633848111
6.197355596	6.430913434	6.739283293	6.739790818	6.561721071	7.102685081
7.841440677	8.090157434	8.655437626	8.960218374	8.398231626	9.025691242
6.181117949	6.766521566	7.152420131	7.20129803	6.916922333	7.102414636
9.250125475	9.387059283	9.041820404	9.510536	9.735412273	9.091299889
8.940763364	8.991410131	8.493155899	9.049629657	8.499836475	7.85604701
7.85299496	7.775998535	7.228759818	7.797507939	7.408681949	7.100441616
11.2608846	10.26832521	10.90606985	11.36979611	11.15183093	11.79870316
9.587719636	9.715215101	9.46096901	9.808672222	9.806332162	9.285000091
7.890755525	7.317897131	7.973992111	8.015705778	7.932453717	7.970233808
8.881310737	9.726443586	9.221294354	9.533033172	9.245932475	9.479751879
11.48061491	11.40306525	11.02551486	11.41238356	11.00620373	10.78987961
8.223899788	8.679578434	8.423588242	9.124449354	8.853585596	8.50618604
9.249612495	8.973478152	8.700606657	8.824851909	8.907915899	8.922632727
8.134366646	8.549517636	8.48742196	8.882379495	8.222994222	8.396963808
9.684886576	9.347406172	10.11941657	9.295516616	9.642500081	9.982665242
8.00874802	6.974196465	7.41345197	7.627997354	7.216692263	7.797507939
6.941467394	7.453291162	7.821271606	8.69723302	8.273483707	8.232163879
7.131863879	6.751435283	6.990487535	7.225301495	6.42385696	7.121605929

8.27018399	7.613528424	7.686775222	8.055566596	7.833986465	7.936282586
6.509844303	6.424673818	6.724256232	6.86313897	6.731476051	6.622911394
6.291872394	7.596246919	6.745632475	7.288128061	6.395573626	6.384073253
7.266262606	7.335553323	7.181436869	6.71776502	7.164674212	7.350388939
9.819688798	8.383846919	8.710842768	9.692793657	9.493200828	11.21668249
8.053619525	8.147147636	7.693516556	7.310482798	7.764199131	7.719466677
8.854603364	6.923947081	7.366317111	8.074904687	7.923423949	8.603745778
7.582311101	8.336257222	8.501579646	8.272231939	8.440878707	8.424058939
8.150448232	7.59656001	7.429246232	7.350114798	7.304428303	7.619933919
10.02161965	9.958247111	10.60635433	10.22267877	10.65634987	11.9091429
8.621272737	7.997183333	8.635209293	8.933180404	8.493653778	9.099925818
7.084441778	7.574795778	7.427701768	7.610574101	7.549557061	7.474059606
8.579336081	8.844262616	8.971727333	9.420326576	9.132080535	8.991410131
8.27861104	9.790672212	8.016429949	8.532119949	9.146732444	7.838908253
8.141770949	8.206288727	8.295009141	8.513601889	8.195405505	8.624974828
8.491898242	8.602379626	8.333227545	8.36556403	8.898231131	8.30460302
9.919491525	10.05970796	10.38531322	9.728754818	9.902579253	10.13768673
11.238428	11.26990549	11.46539851	11.17910752	11.30301738	11.24323027
5.779008051	6.103934949	5.851773455	5.772280253	6.301115768	5.810702081
13.02006671	14.26676228	14.39736901	13.99495772	14.21449649	13.96779242
9.756054152	9.204855576	9.263851586	10.54441943	11.0387546	10.80782516
7.877487515	7.431765758	8.072532475	8.952561111	8.508189222	7.728706424
7.761767192	8.136235949	8.490053091	8.649944576	8.48093102	8.493155899
7.638981707	8.48873997	8.599954677	8.761177727	8.50618604	8.500240172
7.425828556	7.348317141	6.963370131	7.472789818	7.40744601	7.013931768
7.324608717	7.354199768	7.334394303	7.658148485	6.988716071	7.495760485
8.62412801	8.189194404	8.294235768	8.526186556	8.784649081	8.32939098
9.376046232	8.917682242	9.057828929	9.078885242	9.596773313	9.882779162
7.606990414	7.020179424	7.025866354	7.195224778	7.639990515	8.03364202
9.404333879	8.765233303	9.056062667	9.128533354	9.644080808	10.24828231
12.06964699	12.89113428	12.89886333	12.80166365	12.77841715	12.3843369
8.838495424	9.360502949	8.753927596	9.280885222	9.288419414	8.90531298
8.882882525	8.725555596	9.641787333	8.950355889	9.026117657	8.615330515
7.861189172	6.709075525	6.847264	7.190644495	7.107120283	7.590558081
7.138827606	7.738963899	7.998937818	7.396513838	7.187484444	7.807594768
7.615524929	6.995294596	7.118205404	7.731871869	7.384903253	7.591410606
12.64708972	11.93975968	12.05489479	12.01191659	12.80166365	12.68903729
7.500167182	8.094605657	7.866656354	7.341339747	8.01013803	7.567690545
9.274850788	9.734703111	9.660200838	9.661659101	9.783680727	9.504584061
7.142295949	6.32832399	6.51696904	6.159167768	6.11047004	7.079103131
8.023506091	9.277455131	9.174793152	9.300983919	9.479084687	9.206093556
8.93638097	9.788274313	9.484209455	9.212349869	9.463745455	8.889380424
6.110289768	6.01801997	6.135241576	5.994768081	7.020439899	6.198948818
11.03296099	9.572453101	10.25422487	10.31480319	10.36134852	10.28506122
10.23160947	10.35284213	10.11246778	9.926784556	9.777520899	9.793942798
12.09497108	10.75865445	11.27153548	11.61814895	11.6295292	11.45738434
8.85049999	8.550414556	7.752031929	8.095746162	8.260520646	7.916712838
7.949400121	7.519695475	7.513736343	7.318216242	7.394027687	7.312858101
8.760676242	7.814874545	7.829325051	7.632536929	8.00165403	7.530907465
9.538964939	11.10761793	9.556759333	10.15186663	10.29324401	9.625623061
7.238429162	7.093254121	7.237206848	7.383097848	7.034867141	6.633705889
9.441234879	8.544134919	8.551751717	8.891888576	8.934248131	8.717364515
7.717096556	8.493653778	8.060661899	7.093254121	7.97753898	7.941721242
6.583409828	7.095808323	7.018881949	7.248166182	7.258429848	7.372532788
9.792275424	10.1250404	9.526200354	8.823889374	9.400327152	9.798623747
8.904793061	8.519692939	8.812013889	8.636577313	8.62460002	8.314030071
9.811091061	9.366909111	9.276789111	8.712915394	9.058895657	9.247274333
7.208855192	6.545978343	6.648873556	6.771393131	6.594827192	7.301216929

5.982672192	6.30015496	6.158655535	6.17582696	6.583409828	5.851394535
7.027744404	6.609470354	6.752477677	7.130177747	7.398370889	7.212087616
6.675987374	6.168165919	6.338230899	6.122708465	6.302911687	5.982672192
7.65951298	6.927779576	7.396159798	7.70790696	7.518690889	8.079179394
6.526325283	7.210929778	7.067867747	7.234805667	7.025866354	7.047335818
7.144299071	6.197694323	7.055367566	7.708922879	7.284105152	8.532119949
7.002659525	6.128608687	6.495899485	6.734478838	6.538425424	6.910146768
8.660608859	8.130993828	8.919352101	8.263464364	8.002011657	9.124970051
6.183137202	7.075225141	7.421318101	7.300031424	7.00817199	6.700124848
7.678591667	7.392563535	7.712908172	7.906425364	8.524867051	7.732919727
8.330618758	9.66831603	8.881310737	9.292435667	9.846508495	9.051375475
10.79420974	10.33890802	10.5336638	10.30358984	10.56084718	10.13671639
8.945002717	9.07526898	8.708995576	8.973478152	8.989652131	8.408718253
8.978723121	8.758871	9.189045596	9.445197818	9.097596465	8.964642636
11.5386618	8.08112003	8.281588828	9.826793596	9.978126798	9.572453101
8.228946717	8.848410616	8.826393101	8.832790172	8.737323475	8.69919198
7.529949465	7.370383778	7.464704768	7.468171747	7.555661677	7.421037465
10.99469779	10.06173934	10.68072859	10.43998577	10.7442872	10.7982674
9.662314131	9.467500556	9.763952101	8.78214798	9.054860525	9.079484556
8.157153081	8.448008778	8.526649808	8.035675313	8.157153081	8.25927404
10.71613341	10.92880859	10.4700758	10.85923156	11.26262606	10.68072859
6.982654747	8.123471404	7.899682051	7.701352778	7.49864798	7.927113394
7.823737283	8.366003535	8.771856424	8.384333949	7.866656354	8.040196323
6.94072599	6.872396859	6.955441212	7.065642788	6.399678273	6.915399929
10.27714677	10.32309273	10.1885747	9.781348374	9.84311402	10.06904603
8.118879606	8.967895455	8.668931657	8.383429707	8.304169929	8.631899566
8.940148162	10.00985275	9.570470929	9.477759242	9.516398909	9.629101929
7.545335788	7.334908263	7.500795283	7.647631172	7.616841162	7.048974677
8.743124222	7.102116364	7.391342576	7.882189879	7.869033162	8.007258162
9.054860525	8.802385697	9.508442768	9.293710889	8.980943495	9.348735909
12.04990566	12.47441306	13.70717091	12.9851923	13.12562627	12.73352188
11.54470354	11.26446827	11.15183093	10.4322159	10.87092264	10.94640834
10.74819911	11.11714857	11.41605585	11.30131952	11.02551486	10.65246846
9.34289298	9.544495798	9.128533354	9.422377343	9.97079498	9.372025394
9.495686737	10.86691694	10.81796524	10.82664672	10.39414857	10.97896732
9.137373131	8.29291099	8.22344601	8.210602626	8.641737071	8.053619525
9.564343333	9.960013465	10.04511943	9.836777707	9.556759333	9.949411172
7.540114586	6.820457899	6.62508102	6.130736131	6.589360121	6.655001313
8.988577162	9.574050242	9.07581704	8.940148162	9.204855576	9.167114
9.312041414	6.385400465	8.331458747	8.84935699	8.924201657	8.140538697
13.42153085	14.48025691	14.66395575	14.83117585	14.32804886	13.54221797
10.60277882	8.943861384	8.749346626	9.278597747	9.485668061	10.71145754
7.063477202	6.959934293	6.447271273	6.461008283	6.422635263	6.595700424
6.340852071	7.265083586	7.001104828	7.226681909	7.276277889	6.701095535
6.715208525	7.037248667	7.007335798	7.242080394	7.112620848	6.932737172
10.07107569	9.617464455	9.557589465	9.953782444	10.14809211	9.35128199
9.267923343	7.351625758	7.257203303	8.360501364	8.451947071	8.337550606
7.278115626	7.655968232	7.132177788	7.564402343	7.456765576	7.676615465
10.21769501	9.638947465	9.482360616	9.210065	9.046362323	9.119222505
11.36245739	10.80389925	11.15808934	11.13713447	10.81664077	11.09565519
7.783847071	6.579612929	6.519217414	7.277222202	6.813695162	6.857999616
9.415860727	9.751080646	9.323195364	9.655770101	9.571117879	9.657223606
10.63752253	10.4283734	10.47123164	10.67815746	10.60277882	10.31095185
11.73972102	11.83072395	12.55625498	12.20960187	11.79405389	12.71142619
8.989142232	9.514496606	9.395177889	9.469429253	9.442729677	9.393335687
8.742635939	8.680072242	8.691458141	8.525321657	8.009072212	7.963792818
8.729485838	9.435722162	9.368875727	9.219562515	9.436898869	9.159196141
7.610574101	7.880363606	7.824443343	7.460591212	8.207099091	8.648914657

7.130177747	8.344441616	8.273483707	8.150892606	8.120448121	8.143040162
9.558925838	10.31193963	10.24038579	9.851320949	9.851320949	9.91449603
14.5108222	12.94367069	14.25895571	14.23242051	13.65450155	15.56435861
9.083741606	9.776839404	9.10558002	9.473222667	9.647889364	9.390110384
9.419654929	8.761644081	9.137887434	9.129114848	9.388912091	9.09824599
10.47370876	9.942152202	9.630502939	9.763952101	9.916104475	9.836777707
9.243679384	9.995698596	9.196689051	9.154469939	9.444057586	9.158666485
7.170007293	7.036743576	7.226157253	6.941709929	6.991264788	7.208321485
8.271405859	8.139691131	7.796747616	7.723009	8.117134071	7.684267263
8.612685364	7.981709394	8.233838889	8.547449101	8.553132192	8.830117929
16.42659037	14.24162707	15.16803204	15.65613201	15.52328152	15.63778652
8.326162172	8.134790222	9.411394242	9.730279475	9.773700283	9.368875727
9.870931818	9.274850788	9.616057222	9.574400328	9.470840162	9.288999586
8.399553939	7.613815293	7.943433747	8.177906263	8.138156535	7.975414556
7.856391758	9.145078374	8.813595202	8.853585596	8.513601889	8.743124222
7.968691808	8.538380869	8.559456798	8.094605657	8.301657889	8.36556403
8.014979636	8.395296687	8.156085586	7.447717303	7.728044798	8.023099172
9.022000869	8.886096818	9.748306121	8.460488909	10.09426568	10.22070473
8.391869283	8.25570701	8.220625586	8.454954939	8.339546939	8.423588242
7.441979727	8.406604404	8.321152475	8.36093298	8.321919424	8.239938859
9.759214424	10.24828231	10.24612484	10.0188097	10.12126634	10.0406788
6.510509061	7.684965778	7.241538444	7.585519525	7.266262606	7.356848556
8.98371896	8.961854929	8.63794701	8.674900657	8.559456798	8.51269104
9.238865747	9.124970051	9.164545343	9.442729677	9.293710889	8.989142232
8.151247717	7.877119465	7.850890354	8.186741323	8.018337859	7.724049626
10.72633412	10.3025572	10.3494333	10.60507136	10.52476206	10.1985269
11.60479972	11.56291631	11.565007	11.49636188	11.46320141	11.39983314
14.06476988	14.010587	14.4427031	13.82538175	13.80033164	14.62217632
10.24316974	10.9660467	10.36589334	10.4524716	10.34626898	10.71613341
12.67700968	13.03496614	12.797444	13.32669092	13.16824615	12.92542832
8.727989172	7.074397545	7.124025636	7.693516556	7.485191121	7.819838162
11.45526933	10.6313464	11.47871073	11.12151875	11.31852116	11.61814895
11.69440164	11.51867768	12.32024961	11.96947069	12.05489479	12.84197228
6.549138121	6.519665929	6.796104869	6.615401818	6.966460535	6.978374222
10.4429644	7.894315778	7.989587677	8.810986707	8.788135667	8.942756889
7.282365313	7.624419899	7.983548525	8.194244232	7.700334879	7.453021566
8.78214798	8.717867636	8.251922909	8.964085616	8.585303495	8.304980606
7.88753598	7.980256929	7.940978939	8.005371424	8.105207222	7.577325354
6.030939667	6.491493939	6.733451596	7.364797848	6.951451	7.308145939
8.635209293	8.957404525	8.762202232	8.277405556	8.381309202	8.557697889
8.876650909	8.890936242	8.900236364	8.912452162	8.924680667	8.466852202
8.001277303	8.000521869	8.192266657	7.757833798	7.382821899	7.664275131
9.019287687	8.559011545	8.684654737	8.785177889	8.878201818	8.670447949
7.640998838	7.26819499	7.670902747	7.816627253	7.708254091	7.865213
8.360024717	7.612909768	7.550541152	7.842052606	7.738963899	7.535530424
6.722042727	6.236419566	6.343264394	6.601976232	6.633705889	6.904054566
6.736085162	6.239584586	6.338010192	6.208377172	6.676440081	6.631383222
6.177840061	6.962034242	7.021742939	7.046480485	6.43322899	6.468467859
8.949784505	8.748883111	8.700142556	8.909404788	9.451212687	9.384078242
10.9991534	11.42787377	11.17378617	10.88166557	11.1276132	10.90324033
6.189761525	6.686787465	6.931948737	6.863923949	6.744878414	7.197080263
8.733447455	8.067132556	8.301264212	7.606990414	7.337098929	8.659649677
6.958952465	7.442908828	7.820934747	7.731201121	7.435437465	7.889381343
8.489178071	7.45998899	7.935468505	7.917403434	7.931306778	8.463063475
8.366003535	8.591239465	8.734416606	8.321919424	8.684081152	8.304169929
8.788663253	7.929187444	8.248986687	8.584413131	8.502042192	8.788135667
10.25422487	11.05356995	10.71860391	10.72384747	10.49241362	10.6313464
8.864955909	9.826793596	8.962901121	9.35128199	9.280885222	8.982611727

8.978204141	8.63568099	8.841575667	8.322342051	8.858692343	8.953653768
11.2518391	10.00002479	11.9982543	11.58005536	11.02700786	12.60396528
6.661016606	6.890996051	6.875487455	6.652395162	6.560645212	6.67332503
7.08589403	8.08193004	8.104133364	8.039451586	8.114611232	8.238629646
10.35917202	9.211184727	10.98492733	9.886762101	9.736139061	10.62764398
9.440510152	8.911610283	9.433849505	9.015966535	9.056533626	9.043522909
6.375715869	7.88753598	7.616841162	7.679923515	7.557551899	7.325162263
8.205892515	7.997875909	8.337550606	8.406604404	8.157542717	8.69335002
9.94402302	9.848227263	9.976273051	10.1597097	10.07580535	9.471411141
7.053922394	6.964897566	6.982654747	6.751979343	6.811363364	6.824838515
8.116577384	7.455517596	7.626470505	8.126253929	7.885378	7.563439404
11.41605585	11.308834	11.17910752	11.44491584	11.36423723	11.13551505
11.51260306	11.15503783	11.26623333	11.10170724	10.99046505	11.57765358
9.291393586	8.001277303	8.154705535	9.264483889	8.446641939	8.62991701
9.166446616	8.830660505	9.093001495	9.424273283	9.281923404	9.033915556
9.519820636	10.59418919	9.835894606	10.49009312	10.08799683	9.786685869
14.42673578	14.32804886	15.10875812	14.76275492	14.5108222	15.12351424
6.422232	6.485516717	6.419503354	6.881026485	6.501664081	6.624843121
6.110013152	6.289741071	6.32750801	6.809486374	6.447069212	6.344403182
7.546592828	7.149049263	7.023397576	6.311444323	6.789025475	6.909401626
7.091328697	6.197355596	6.521900899	6.427492121	6.530842141	6.96434804
10.03205964	9.806332162	9.804037697	10.39641485	9.883591758	10.16165443
7.592388485	8.215082485	8.079553929	8.696739152	8.843148071	8.29927897
6.75222999	7.534885556	7.568310444	7.817596162	7.462172081	7.481001515
7.874403566	6.951745222	6.933584646	7.494510091	7.451183323	6.974196465
8.255317818	7.369773828	7.324608717	7.399358222	7.194952889	6.984268657
6.690815606	6.131204707	6.838696091	6.501046121	7.049242879	6.827661505
10.00262223	10.38208186	10.70425349	10.25906505	10.63516273	10.25906505
8.706127333	8.258860253	7.775630727	8.152794889	8.05474504	7.780116303
8.990775909	8.687969051	8.831800202	9.182204323	8.963499596	8.82789604
7.457632404	8.468418253	8.824851909	9.032817303	8.656835556	8.886620687
7.822730929	7.582311101	8.101068051	8.284073869	7.840025778	7.758641747
8.594861495	7.529949465	8.181574081	8.284505182	8.322342051	8.572701232
6.497644434	6.647920455	6.636058242	6.853461566	6.689415172	6.446834051
7.606043535	6.969919475	7.066488111	7.223246162	7.257203303	7.080525788
7.213710414	6.868868606	7.13936198	7.221345788	6.858444758	7.600615697
9.063885091	8.901935111	8.977558859	8.658802051	9.004398101	8.752912545
7.477877475	7.379145515	7.430156263	7.60255999	7.805068949	7.122942091
8.486543404	7.667962596	8.464558212	8.548679899	8.389653889	8.526649808
10.91766747	9.168828475	9.978126798	10.57885616	10.20964764	10.6488688
6.507609283	6.619525869	6.877410515	6.918279333	6.787317758	6.772767788
7.723687384	8.36556403	8.266842545	8.072110051	8.117134071	8.142577798
6.983707354	6.86140099	6.979690485	7.807908545	7.320252869	7.446413172
7.273224596	7.144887111	7.142895121	7.214315212	7.062959768	7.144299071
9.887613414	9.566253768	9.373397364	9.026117657	9.177504333	9.222631263
8.700606657	8.879330566	9.189590182	8.85518403	8.903025495	9.648698737
6.893883192	7.474397404	7.545986354	7.528986283	7.590787525	7.640998838
6.507425384	6.824838515	6.800323596	6.83457999	7.059956313	6.884060404
9.226128374	9.857104919	8.904239657	8.190781646	8.678658293	8.094605657
8.539776737	7.655304091	8.097958939	8.771294657	8.531655515	9.290785283
7.349181162	7.477877475	7.479706475	7.115131646	7.233950838	7.428605818
8.730448515	8.827377505	8.738735232	9.296718121	9.227886646	8.906407303
11.71895032	10.77890896	12.04160066	11.52238659	11.11388317	12.62449502
10.25519474	10.42062148	10.45628541	10.76857843	10.31480319	10.29949835
10.03492192	9.943097475	10.33890802	10.35284213	9.721175788	9.560980909
12.56726296	8.661956242	9.579413505	11.1612024	11.20840189	10.61001127
7.275765808	6.488188354	6.57397498	6.889521525	6.831619364	6.864750879
9.269847242	8.260123323	9.245445293	9.118629636	8.829514889	9.673359929

8.80133401	9.438799404	8.82024202	8.657372182	9.007167758	9.119831202
8.366948404	8.358783152	8.673363869	8.721054424	8.616914869	8.503455465
6.758137737	7.686444293	7.982067081	7.458509535	7.574489596	7.365381232
7.72538904	7.594662899	7.56165098	6.991849242	7.539167263	7.498029465
6.545504717	7.444499747	7.019151556	6.73803103	6.581763828	6.948873394
12.37087917	14.53168901	13.31405593	13.76108056	12.88708268	12.37416009
8.38301802	8.395713444	8.077230061	8.212685152	9.037153444	8.347358778
8.033283465	8.061773232	9.04056096	9.34289298	8.784105778	8.601896768
10.05780386	9.843921515	10.62536143	10.05053825	9.962571606	10.08700287
7.391952354	8.022720303	7.595878485	7.426803586	7.377269152	7.537817576
7.000584848	7.259306869	7.854005263	7.224144889	7.202436384	7.138827606
8.81572204	8.355387535	8.323598384	8.229339455	8.353342091	8.130283051
8.746311293	7.798826303	7.919201576	8.368780919	8.315715768	8.043818737
6.795847303	7.390207394	7.695912303	7.500501152	7.55945201	7.813414727
10.9889639	10.74690378	10.91514001	11.07493153	10.80091358	10.75453103
8.572163455	8.848914293	9.293710889	9.137887434	8.905887879	8.929384727
9.036569232	7.647631172	7.560760293	8.060252263	8.10683704	8.079973212
7.850180343	8.780092131	8.412578899	8.669446475	8.509502414	8.128290707
10.74558579	10.03299879	10.3896003	10.56655729	10.39072525	10.4429644
7.238133434	7.089371313	7.669946162	7.916025434	7.287262414	6.754460545
8.855784909	8.53537201	8.805485081	9.113305192	8.696739152	8.637513768
9.173615515	9.31393701	9.275532697	9.833438364	9.441908576	9.266794162
8.233838889	9.636296737	10.08978265	10.36239309	10.05880999	9.932690333
8.511758747	10.03123363	9.858738737	9.62845904	9.419654929	9.665316566
9.18155302	9.812775374	9.679062616	9.467500556	9.343457394	9.770750222
8.717867636	8.115821848	8.463578343	8.688838939	8.362537455	9.072998758
8.998420707	9.162016475	8.920488949	8.352944343	8.682574626	8.402589283
8.425847798	7.348914263	7.800216455	7.878949444	7.897352323	7.410944101
11.52238659	11.72126504	11.55524693	11.67371856	11.85033795	11.50261564
8.947623838	9.329922273	9.724814657	10.1985269	9.93099102	9.248396293
10.05320582	9.55462702	10.12126634	9.775265525	9.629839465	9.933588354
10.51771241	10.42927797	10.13768673	9.86640601	9.942152202	9.846508495
8.76328402	9.62024901	9.445852485	9.696433626	9.247274333	9.584100455
7.651926253	7.815915242	7.603719626	8.048299333	7.755825222	7.629673333
6.880211293	7.70365599	7.910975505	8.013081051	7.657544313	7.788172758
10.01974654	11.18291631	11.0859227	11.15808934	10.88546605	10.23752804
9.856174394	9.848945263	9.876880455	9.943097475	10.14131493	9.713188212
6.413017273	6.369870263	7.201570444	6.060926152	6.138681313	6.563407434
9.939327313	9.717365758	9.968215758	9.641787333	9.755162434	9.727935707
9.015412101	8.050484323	8.410410394	8.730448515	8.716387192	8.64224801
10.58347296	10.78269205	10.68687892	10.17789322	10.39641485	10.53141254
12.13244857	11.911688	12.06397065	12.17829278	12.03912361	11.89130603
6.671832111	6.406383182	6.437442354	6.683063909	6.62116196	6.586881556
6.508927202	6.382405131	6.615162343	6.791503909	6.562503909	6.526565121
9.097596465	6.729463253	6.845837626	7.783465061	7.937868929	7.805068949
7.608647091	6.739283293	7.237499505	7.249695535	6.898276768	7.953126222
6.87893297	7.863195747	7.532150707	7.676956101	8.123844788	7.787863889
5.874506374	5.757790323	5.76123004	5.925842949	6.234440616	5.758070626
11.0387546	10.47479655	10.36687712	10.17336172	10.4655823	10.23452156
8.647073404	8.090488788	9.314493212	8.967408879	8.943348051	9.811898121
11.61814895	11.94979793	12.12692782	12.28369472	12.07810634	12.58287167
14.7172184	14.33555643	15.21350605	14.9727616	15.22815255	16.09492117
9.348136535	8.76626204	8.93638097	8.934248131	9.171801828	8.675348646
11.25700815	11.24993851	11.17571904	11.46724679	11.29230964	10.96460614
12.53563192	14.36070414	14.010587	13.97489728	13.58996905	13.44126502
10.1597097	9.235179424	9.621550919	9.465659495	9.09824599	8.885588879
8.825911939	7.945472162	8.424934535	8.693780273	8.640338566	8.860747333
8.177146606	7.001104828	8.161911788	7.575420636	7.330546788	8.319524414

9.07554301	9.599465606	9.634173323	9.687048303	9.477181455	9.185497838
9.077100232	9.335891293	9.337862091	9.536330707	9.434425707	8.298396535
10.76609029	10.65634987	10.51650912	11.11714857	10.93789932	10.6768734
7.555333263	7.446413172	7.086390323	7.204042263	7.132449434	7.425828556
10.76361324	10.18762619	9.975431768	10.87092264	10.90466263	10.47123164
8.35796399	8.465492313	8.302900242	8.273061586	8.221010253	8.511758747
7.902575141	8.292068253	7.508036465	7.285574899	8.074904687	7.424927172
8.091323071	7.121055596	7.921614596	7.492628778	6.922161646	7.715785354
6.088964717	6.495899485	6.654772152	6.69036997	6.730688788	6.504049202
7.840676768	7.978709162	7.924172566	8.226500707	7.735250384	8.838020616
8.960773354	9.975431768	9.604175636	9.542425758	9.591816061	9.302735434
7.623386535	7.515816273	7.704008545	7.803554182	7.702676091	7.820934747
12.75396286	12.84627851	13.39659876	14.1830221	13.27940818	12.78645268
10.42062148	8.713417869	9.530205505	9.147296667	8.242431778	10.15483313
11.85889751	10.10454015	10.29000276	9.368224626	11.25523353	10.66465811
8.289996192	7.364257101	7.719108384	7.532150707	7.791885697	7.245319404
10.9088749	10.36589334	10.22361636	10.17613521	10.50057506	10.01974654
10.55070525	9.385837242	9.580097263	9.671170444	9.886762101	9.449254061
7.93027604	9.160413333	9.135618929	9.461673394	9.296718121	9.345614434
10.51650912	7.909763434	8.434806414	9.29496	9.277455131	9.513795939
14.91973608	13.5964752	14.47046648	14.48025691	14.20688311	14.89443037
10.82416773	9.54312803	10.0060683	10.35699559	10.43998577	10.09795649
6.185668061	6.663676879	6.875911141	7.237499505	6.832629131	6.937364475
7.045230232	8.446184828	6.861931182	7.59656001	7.702311758	7.280239242
7.478449101	7.423727141	7.776293606	8.303356141	7.844877657	8.761644081
7.396513838	6.713421434	7.257525758	7.248451727	7.070666535	7.41439101
7.307022919	7.332078838	7.55178802	6.958200131	6.950902859	6.975978909
6.748324394	6.323943747	7.253230737	7.020439899	6.981335535	7.406158394
7.233650202	7.207500556	7.556302505	7.750957677	7.732206152	7.280525364
11.64848719	11.37847359	9.875361242	8.287663293	9.286110869	11.37490296
6.511476192	6.930222384	6.829096384	7.159453505	7.039966242	6.732989909
7.249375465	6.695720192	6.802568323	7.310724131	7.076844636	7.785940475
6.528858343	6.025860101	6.186015354	6.105835768	6.201441303	6.850882899
7.094132182	7.506099556	6.678316576	6.544097556	6.880211293	6.442753616
11.01045685	11.74670945	11.98196729	12.0838996	11.53677901	11.80342824
10.14631732	8.929972798	9.172421828	9.545881535	9.524763869	9.094691273
8.104500051	8.658285303	8.486053202	8.627322343	8.383846919	8.820759798
8.012346152	8.301264212	8.018723424	8.355847778	8.497259596	8.359263212
7.587331949	6.525823616	6.825325	7.517772121	7.086390323	8.312729141
5.989763313	6.604297121	6.898792364	7.470886646	7.117115202	6.988716071
9.954681949	11.32027501	10.61001127	10.31289083	10.45877561	10.4994288
10.16073454	10.70643744	10.30695028	10.70643744	10.65127749	10.64005918
9.201355202	9.798623747	9.159785394	9.152615889	9.09013302	8.988577162
10.70902317	11.66189432	11.16579624	11.03163187	10.94208941	11.19293398
12.15130062	12.88265743	12.36759935	12.62807191	12.71830686	11.94979793
9.135096212	8.332313525	8.482406475	8.494130061	8.600831152	8.292068253
7.938205586	8.917069505	8.62412801	8.419470808	8.279462	8.477856384
8.537956091	8.473802646	8.879851283	8.897751606	8.712915394	8.679578434
9.493200828	10.36687712	10.03492192	9.891739697	9.869466131	10.04776272
13.34730616	14.52085241	13.50997822	13.57574016	13.83772389	13.60269863
10.0719806	8.986432162	10.36134852	9.716648152	9.778131475	10.56908161
9.132080535	8.919862162	8.983202444	8.540635232	8.564366949	8.674900657
7.706567859	8.540202646	8.222994222	8.115437899	8.245649515	8.436545051
9.010430586	7.04319302	7.386471808	8.195405505	8.191909586	8.181963182
9.304577515	7.620680879	7.712213273	7.711891616	9.100524758	8.174871818
7.088518818	6.819189677	6.837226	6.984268657	6.979690485	6.919083737
12.10135206	12.20960187	11.93178106	12.14191765	12.04432	11.70348036
11.34381562	10.83930015	10.8443678	11.15666056	11.44643795	10.82153564

8.480158333	8.261007747	8.44129198	8.11773804	8.225261616	8.400903717
8.491490828	8.741655828	9.108564253	9.132651727	9.022478232	9.160413333
6.611373919	7.03146702	6.935569505	7.135537212	6.949335212	5.986157242
11.70557621	11.41778198	11.60888892	12.19727311	11.78023213	12.7452022
9.052474394	9.267383556	8.542859455	9.315671101	9.134591697	8.755421576
9.967178364	10.5104744	10.47820756	10.6240143	10.5104744	10.3896003
8.335861768	8.073652495	8.346198293	8.666113404	8.572163455	8.517288667
11.39382595	10.32849396	10.66465811	10.95374716	10.54565144	9.909202788
8.115821848	8.642670768	8.360024717	8.08075902	8.313601758	8.391869283
8.694292242	8.33796001	8.284876758	8.886620687	8.77962597	8.635209293
9.049629657	9.218466939	8.858692343	9.127872697	8.82024202	8.349393212
7.46750399	7.447389475	7.275223848	7.56409603	7.797125465	7.158043384
8.541524636	6.876878333	6.916181	7.817254242	7.591105343	8.019434586
8.838020616	7.945111576	7.779426687	8.324880909	8.028563859	7.877844556
11.93178106	12.27421104	12.49170848	12.37087917	12.48124823	12.23191382
9.449948384	9.989520798	10.31480319	10.50794857	10.95234717	10.43609926
14.223913	11.41071085	12.81379045	13.35919327	12.97977545	14.67552064
8.271029889	7.876807081	7.994919717	8.071703475	7.993077394	7.865549818
8.594455404	9.616057222	9.151976485	9.496413788	9.341818545	9.026636737
10.10841911	11.03163187	10.84176532	11.15503783	10.98749259	10.33890802
10.93935201	9.588410576	9.683334444	10.40387449	10.43655428	9.713858919
11.87445957	12.65819872	12.49965499	12.7494647	12.8652792	12.41337695
10.23247836	10.18198959	10.0719806	9.740584727	9.878686616	9.624391323
9.675904202	10.28506122	10.82664672	11.01683173	11.00754401	10.53623255
7.033114263	6.691780525	6.900245465	7.203245626	6.665428859	6.421771838
9.555296242	9.759214424	10.20060626	9.985290333	10.11051676	10.13580598
8.091725323	8.135177152	7.457078101	7.698928879	8.038354192	7.95565902
9.077686808	9.915234909	9.686378667	9.406122899	9.530933293	9.94767803
7.883157929	7.441392919	6.224601374	7.215458101	6.818722313	6.293717162
10.75062885	10.68072859	10.35591393	10.11246778	9.912769303	10.22164118
6.705415545	6.866055313	6.283373303	6.895441525	6.289561192	6.777950687
11.4481816	7.896287434	8.371674091	9.11393401	8.650965909	9.962571606
9.137887434	8.467667768	8.555429434	8.996148737	8.71185199	8.33273403
6.057581384	6.342888505	6.630903202	6.69943696	6.762066263	6.521008828
8.954798808	9.297353606	9.420326576	9.598265626	9.415217505	9.257979848
8.640338566	8.451947071	8.254919778	8.922082808	8.952561111	8.808928141
11.45738434	11.98440811	11.3403052	11.94234713	11.73710477	11.36793803
8.837022465	7.146069808	7.842359758	8.266388465	8.226500707	8.474228909
7.526779747	6.748092424	7.012316737	6.774434798	7.040256253	6.627335
7.370383778	7.378508566	7.434501333	6.804242869	7.222960121	7.769805091
10.28795108	11.78498903	11.32185019	11.59021288	11.29744903	11.31267384
9.772980061	9.910074283	9.997192202	9.751080646	9.459664354	10.34311059
8.581684657	8.954798808	8.98371896	8.679578434	8.796283929	8.813595202
8.563085788	7.889381343	8.217667525	8.193820172	8.31008898	7.883536596
8.964642636	9.135618929	9.138451657	9.170513566	9.202552434	9.719830172
6.910693596	7.995673758	8.294640202	8.144908747	7.675167556	6.900488939
7.286993737	7.116617232	6.836530859	6.242810566	6.944866586	6.713757434
8.406205495	8.168587596	7.421647414	7.532708636	7.877844556	7.371279929
8.384837869	9.132651727	8.990220404	9.080683394	8.977558859	8.877161475
9.237064687	8.357077121	8.687541707	8.83948901	8.643636162	8.833337414
11.77305263	12.41931404	11.80104424	12.24933389	12.30465105	11.76804793
6.465818222	6.894135505	6.874493707	7.928481586	7.276013455	8.26142704
8.584413131	9.339764051	8.703161646	8.716864899	8.85049999	8.457043091
7.065642788	8.053207788	7.543124535	7.541090081	7.752360667	7.609574333
11.27499716	11.91912022	11.51465712	11.90674449	11.53473316	11.5386618
9.007843273	9.129654758	9.042944374	8.583542788	8.618302869	8.676791091
9.940265465	10.46766096	9.746868596	9.796940758	9.620873889	9.565032515
7.284105152	7.272016424	7.197446212	7.202115061	7.819085697	7.173570899

9.051375475	9.310775172	9.203760495	9.519820636	9.161011869	9.08614898
8.279889293	9.008956374	8.877161475	8.846850111	8.638952889	8.963499596
7.935081758	8.466026899	8.180785838	8.439112919	8.275579182	8.155735586
7.393766606	8.449337667	8.151663141	8.294235768	7.905781333	8.32856404
8.835019636	8.063461202	8.320817869	8.314398727	8.144908747	7.868335495
8.896578162	8.039068889	9.308265131	9.250770152	8.646531515	9.526870273
6.59116396	6.121456293	6.232903485	6.376098505	6.312403384	6.873088737
7.143440162	9.092457495	8.862785404	9.202552434	8.90531298	8.944409727
8.594016788	8.505287909	8.419470808	8.783158111	8.990220404	8.269818727
7.665645141	8.221794202	7.941320778	8.382609505	7.731871869	7.049242879
9.679062616	10.19649023	9.61181703	9.702758343	9.702758343	9.512482222
10.2692332	9.466909636	10.38738452	9.879566364	10.01524205	10.37346225
10.58469265	12.61761735	12.60022648	12.36759935	12.24933389	12.10135206
7.304975899	8.213079374	7.606370131	7.583998525	7.514821949	7.730154939
8.096525737	7.567065616	8.469339576	7.611330343	7.750957677	8.200961636
7.319114313	8.174468182	7.847720859	7.506099556	7.827906879	7.64030596
6.887288828	6.652654	7.030688768	7.13105099	6.913511828	7.336476646
11.52052132	10.89755671	11.49239347	11.64244603	11.09721747	12.12151457
8.049354232	7.517077263	8.41552702	9.001802606	7.991473828	9.005433152
12.16279806	12.02514953	11.49839615	10.90192126	11.35858946	11.238428
8.498949848	8.957944414	8.460488909	8.565878434	8.068200283	8.477505576
7.30673204	7.633233848	7.863889121	8.432612263	8.006495333	8.116212253
13.49745762	14.4185289	13.16151113	13.10484893	12.79024082	12.93862501
10.94786936	10.31289083	10.55333109	10.23752804	10.37215733	10.2026692
13.18858184	14.11997744	14.24162707	14.11151028	14.010587	13.89331098
8.563501616	9.008373061	9.600185283	9.576136707	9.332175414	9.517013525
10.85782556	9.280305798	10.60863639	10.67190595	10.25811169	11.19638858
9.379720636	9.099925818	9.479751879	9.509876273	9.607593848	9.145664475
10.60863639	10.94496034	10.84844593	10.42622906	10.58935103	10.67939021
10.11150219	9.690534253	9.106823152	9.670530667	9.86640601	9.152615889
7.605411525	7.533296202	7.605113071	7.583321859	7.528640475	7.532439838
7.423727141	8.170129495	8.083485404	7.618887111	8.020096283	7.775998535
10.17224075	10.9991534	11.02403513	10.38208186	10.67190595	10.78269205
8.251492909	8.585836293	8.546545061	8.292068253	8.432612263	8.458697343
7.320933798	7.987573434	8.269818727	8.230181798	8.107705111	8.075284384
10.62895885	9.857940545	11.30131952	10.85782556	11.08756575	11.28565422
9.514496606	9.236429667	9.210065	9.149684939	9.377330121	9.258612929
9.878686616	10.86963124	10.01703805	10.18468509	9.614560172	10.49713487
8.376150616	8.267698071	8.431326525	8.819236758	8.253254586	9.126663535
8.769824364	9.496413788	9.348136535	8.988033424	8.949784505	9.250770152
9.84311402	10.22761479	9.542425758	9.718307192	9.981718727	9.567720788
6.510064202	6.909691071	6.873999848	5.785833313	6.35069898	6.075849657
7.117635758	7.595301182	7.516462556	7.174161374	7.427372788	7.56320098
9.905938879	8.678658293	9.184000242	9.07526898	9.538964939	11.29076429
7.501110505	7.045715273	7.004269242	7.253486596	7.096340343	7.128773535
10.3305428	11.17910752	10.81034167	10.59541023	10.71503879	11.01951417
9.445197818	9.474605101	8.79576798	9.344897333	9.408808788	9.026117657
8.270601414	9.395854121	10.20964764	10.0143126	9.632768747	10.10946026
8.448008778	7.732206152	7.941721242	7.980256929	7.936282586	7.969427899
8.323157747	8.100639	8.298396535	8.558108202	8.215875455	8.687969051
7.399358222	7.85710597	7.556609313	7.604779273	7.732550404	7.570765313
8.079553929	7.434204455	7.378842657	7.561342071	7.640998838	7.343750657
12.22341713	13.29117716	12.27421104	12.95787308	12.3774088	12.56315114
11.12315281	11.69188536	11.59021288	11.26623333	11.32027501	11.66189432
8.934777717	9.143918798	8.910531141	9.176146596	9.070117818	8.736738475
8.156772778	7.884314808	8.265965545	7.694191859	7.772503354	8.387083515
6.60520404	5.878464747	6.188035909	6.524717879	6.330318636	6.941957697
7.901561475	7.774283859	8.226840717	7.333224889	7.010749455	7.665645141

5.835033172	5.760968091	5.804161788	5.758791525	6.751979343	5.760741364
9.430143091	9.750460616	10.42297323	9.632768747	9.76546198	10.46664928
9.918671212	9.571117879	9.494490616	9.439917707	9.706432808	9.57181996
9.316888828	8.592622071	8.780092131	9.164545343	8.530333535	8.859179455
11.5571076	10.96748398	11.12613667	11.565007	11.18291631	10.78014639
6.480989929	6.433428778	6.287217818	6.742219182	6.383199758	6.139423374
12.14191765	12.89886333	12.5521346	12.85589012	12.48458958	12.39973853
6.908606212	6.23392001	5.969986778	5.975569111	6.464011586	5.791090768
8.336651323	7.775269606	7.364257101	7.120482	8.048636061	6.968641576
6.359365273	6.260257263	6.465574545	6.493950384	6.076756677	6.644450293
14.53168901	14.9847254	15.26374094	14.90726461	15.24813401	15.54332231
9.291911616	8.677215515	8.71485401	9.249034202	9.359783616	8.544134919
12.9851923	13.55640443	13.44126502	13.45466248	13.91489381	12.59323492
8.955359253	8.660166475	8.708030737	9.168828475	8.940763364	8.700606657
15.31193978	16.39933956	15.99508933	15.88528111	15.99508933	15.48449671
7.464704768	7.212654808	7.36076402	6.777485293	7.25688896	7.205804788
8.239474646	7.792215626	7.939228495	8.05474504	8.098747556	7.920144808
12.00086001	12.54268587	12.13538265	12.5521346	11.74205097	11.67371856
15.09418019	10.46442497	11.65261158	13.18858184	13.3718573	13.37822002
6.406575848	6.158655535	6.570961455	6.245816848	6.558125737	6.783136778
5.759615182	6.460273586	6.239068939	6.668303455	5.812465879	6.885309253
6.694222899	7.226157253	7.640655485	7.839647131	8.133179586	7.250899535
11.29947244	11.05796986	11.08435313	11.42178201	11.33477589	11.03737422
9.471411141	9.402814081	9.388912091	8.915814212	9.155642525	9.012760899
10.5336638	9.503271071	9.894213939	9.733950273	10.06547375	9.62845904
8.004282899	7.585519525	7.67441401	7.97830402	7.832891081	8.226165
11.50814936	10.89912578	11.12935449	11.51260306	11.56291631	11.671419
12.56315114	13.38447499	12.80602179	13.03025597	12.7939548	12.71520416
8.18280501	7.960941889	8.019769071	8.286610323	8.326162172	7.832891081
8.682101313	8.040575909	8.047553323	7.244714616	8.516793455	8.309668384
10.01524205	10.28710845	10.91176188	10.48074239	10.60743282	11.45526933
7.993428586	7.377037515	7.468792788	7.500795283	7.559118222	7.182015707
8.863375313	9.888393747	9.742895778	9.609027141	9.604175636	9.576740677
7.693213707	7.825440475	7.765675424	8.355387535	7.374297343	7.239398515
6.912025515	6.768428949	7.079910848	7.413750677	6.368932879	6.433607111
7.523206263	8.821324343	8.387507626	8.298896434	8.147147636	8.225777414
10.3025572	10.18000397	10.13276962	9.717365758	9.956562586	9.833438364
13.35919327	12.18492446	13.22730043	13.04381444	12.93430142	13.41514643
11.41415047	11.5571076	11.86401838	11.45738434	11.80104424	11.52052132
9.132651727	10.17613521	9.692043475	9.359207242	9.566253768	9.688441414
8.817758687	10.24139873	9.544495798	9.957374697	9.128533354	9.575401111
8.339115859	7.56574702	8.03601997	8.129067798	8.380307172	8.177146606
6.201940414	7.835124576	6.573540778	6.444612172	6.488188354	6.436392303
8.856895172	10.49009312	10.14534091	10.02439619	10.09254304	9.785983535
12.33273925	13.40302459	13.20018245	13.42153085	13.13007894	13.21058954
7.601643838	7.709511535	7.965660091	7.591751808	7.632536929	8.214296636
9.793942798	9.722667242	9.867106131	10.01071956	9.677492576	9.917863333
9.273022949	10.54565144	9.734703111	9.976273051	9.802496737	10.09079019
8.428176394	9.548028869	8.385691051	8.489580414	8.674900657	8.415109929
14.52085241	15.78787594	15.38120792	15.39581235	15.06622261	15.06622261
6.323128667	6.418240687	6.157329697	6.238895919	6.364484303	6.153019566
8.065602475	7.988882495	8.055171202	8.261007747	8.011643808	8.086153313
7.005077596	6.04174803	10.65127749	5.791350485	6.22946	7.018669091
9.174120707	8.246470545	9.230770667	8.989142232	8.900779434	9.092457495
7.116869414	6.806225091	6.989715687	7.264510162	7.015108687	7.012571859
7.272599475	8.499404646	8.205457293	8.102862354	7.983184263	8.219832939
7.589888475	6.975188859	7.025021455	7.170490939	7.104098253	7.848096879
7.058479485	6.771840333	6.771840333	7.009209616	6.871668566	7.18283101

7.001612657	6.691055667	6.769655182	6.533739475	6.778966495	6.710946737
9.246644323	8.781156657	8.85518403	9.233272283	9.035586242	8.60808904
8.013081051	7.090185162	7.235967596	7.549206232	7.579959303	7.169717707
7.133007667	7.833986465	7.472789818	7.515531818	7.507667889	7.453956636
9.573320202	8.902484929	9.280885222	9.436357141	9.238221677	9.571117879
7.431389424	7.358375414	7.407756293	7.723384495	7.107390485	7.415639222
11.28370047	10.77103399	10.85654613	11.06877356	10.87518762	10.65987528
8.979921919	8.229339455	7.922697606	8.546545061	8.333227545	9.136296333
8.148308414	8.560860505	8.511305758	8.648450303	8.920488949	8.428657566
8.209054152	8.514037303	7.896600606	8.71485401	8.473392556	8.094218354
7.51310204	7.681167667	8.012010707	8.354947869	8.041000495	8.39867397
8.715845505	7.816254515	7.867312374	8.057063626	8.519178475	8.250622253
7.855731	7.885731566	9.186711646	8.143776848	7.797507939	9.189045596
[truncated: 544,202 more chars]
